# Brønsted acid–catalyzed asymmetric dearomatization for synthesis of chiral fused polycyclic enone and indoline scaffolds

**DOI:** 10.1126/sciadv.adg4648

**Published:** 2023-03-15

**Authors:** Tong-De Tan, Gan-Lu Qian, Hao-Ze Su, Lu-Jing Zhu, Long-Wu Ye, Bo Zhou, Xin Hong, Peng-Cheng Qian

**Affiliations:** ^1^ College of Chemistry and Materials Engineering, Wenzhou University, Wenzhou 325035, China.; ^2^ State Key Laboratory of Physical Chemistry of Solid Surfaces, Key Laboratory of Chemical Biology of Fujian Province, and College of Chemistry and Chemical Engineering, Xiamen University, Xiamen 361005, China.; ^3^ Center of Chemistry for Frontier Technologies, Department of Chemistry, State Key Laboratory of Clean Energy Utilization, Zhejiang University, Hangzhou 310027, China.; ^4^ State Key Laboratory of Organometallic Chemistry, Shanghai Institute of Organic Chemistry, Chinese Academy of Sciences, Shanghai 200032, China.; ^5^ Beijing National Laboratory for Molecular Sciences, Zhongguancun North First Street No. 2, Beijing 100190, China.; ^6^ Key Laboratory of Precise Synthesis of Functional Molecules of Zhejiang Province, School of Science, Westlake University, 18 Shilongshan Road, Hangzhou 310024, China.; ^7^ Wenzhou Key Laboratory of Technology and Application of Environmental Functional Materials, Institute of New Materials and Industry Technology, Wenzhou University, Wenzhou 325000, China.

## Abstract

In the past two decades, substantial advances have been made on the asymmetric alkyne functionalization by the activation of inert alkynes. However, these asymmetric transformations have so far been mostly limited to transition metal catalysis, and chiral Brønsted acid–catalyzed examples are rarely explored. Here, we report a chiral Brønsted acid–catalyzed dearomatization reaction of phenol- and indole-tethered homopropargyl amines, allowing the practical and atom-economical synthesis of a diverse array of valuable fused polycyclic enones and indolines bearing a chiral quaternary carbon stereocenter and two contiguous stereogenic centers in moderate to good yields with excellent diastereoselectivities and generally excellent enantioselectivities (up to >99% enantiomeric excess). This protocol demonstrates Brønsted acid–catalyzed asymmetric dearomatizations via vinylidene–quinone methides.

## Teaser

Organocatalysis enables asymmetric functionalization of inert alkynes.

## INTRODUCTION

**Fig. 1.**
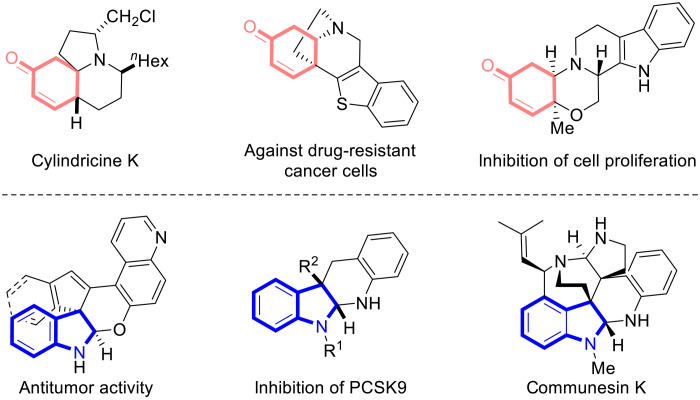
Selected bioactive molecules and natural products containing fused polycyclic enone and indoline motifs. Some of representative molecules are listed.

The N-containing polycyclic motifs are one of the most important scaffold segments in the framework of various biologically active species (*1*, *2*). Among these, fused polycyclic enone and indoline derivatives bearing contiguous stereogenic centers with one quaternary carbon stereocenter are two kinds of important N-heterocycles found in a variety of bioactive molecules and natural products (Fig. 1) (*3*–*15*). However, the synthesis of these fused polycyclic scaffolds has been faced with tremendous challenges, probably owing to the fact that the unique segments of quaternary carbon stereocenter make the molecules structurally less flexible (*4*, *9*–*11*). Driven by their potential pharmaceutical value and synthetic challenge, it is highly desirable to develop efficient methods to construct these fused polycyclic skeletons, especially those with high flexibility, efficiency, and stereoselectivity.

**Fig. 2.**
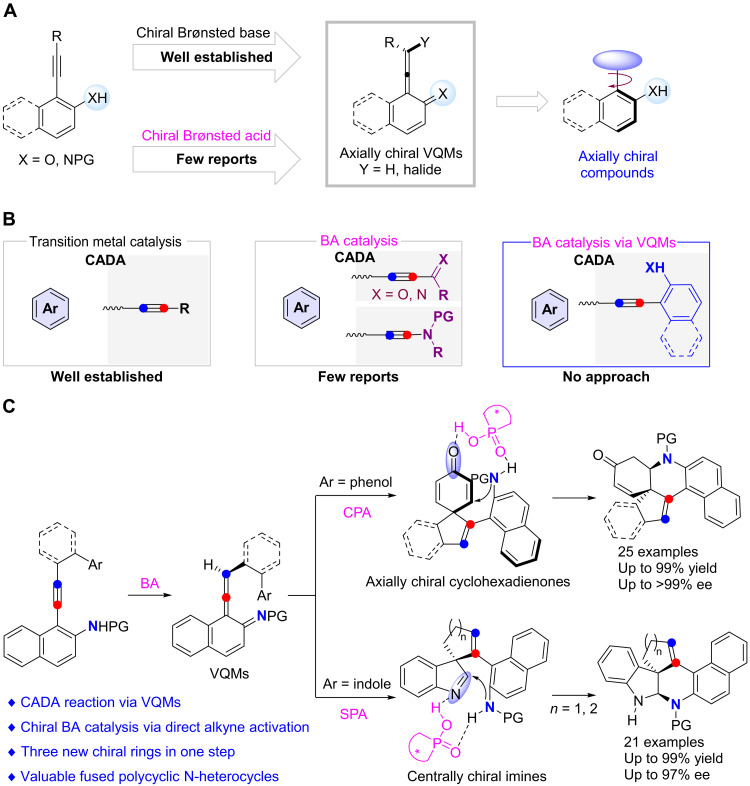
Chiral BA-catalyzed reactions of alkynes. (**A**) Organocatalysis via VQMs. (**B**) CADA reactions based on alkynes. (**C**) CADA by BA via VQMs (this work). PG, protecting group.

In the past two decades, catalytic asymmetric alkyne functionalization by the activation of inert alkynes has received extensive attention. Compared with the well-established transition metal–catalyzed transformations, asymmetric organocatalysis based on the activation of inert alkynes has been relatively less exploited (*16*, *17*). Recently, vinylidene–quinone methides (VQMs) derived from 2-alkynylnaphthols under organocatalysis, a well-known variant of *ortho*-QMs (*18*), have been widely used in such organocatalytic enantioselective alkyne transformations (*19*–*33*). The VQMs were first used to asymmetric synthesis by Irie and colleagues in 2013 (*19*) and later were extensively exploited by the same group (*20*) and Yan and colleagues (*21*–*28*). However, these protocols always rely on the use of the tertiary amine–derived organocatalyst as chiral catalyst and are typically applied into the assembly of axially chiral scaffolds (Fig. 2A) (*28*, *29*). In this regard, state-of-the-art advances from Tan, Houk, and colleagues take advantage of *ortho*-alkynyl-naphthols or *ortho*-alkynyl-naphthylamines as precursors of VQMs to accomplish a chiral Brønsted acid (BA)–catalyzed asymmetric hydroarylation of alkynes (*30*), thus providing an alternative avenue for organocatalytic asymmetric alkyne functionalization via the activation of inert alkynes (*31*–*33*). Despite these remarkable achievements, these BA-catalyzed reactions of alkynes were invariably used to construct axially chiral compounds (Fig. 2A). Therefore, the development of chiral BA–catalyzed alkyne transformations by the activation of inert alkynes, especially for the corresponding cascade cyclization to build previously inaccessible molecular complexity such as those bearing contiguous stereogenic centers, is highly desirable.

Catalytic asymmetric dearomatization (CADA; coined by You and colleagues) reactions have emerged as powerful tools to assemble polycyclic backbones bearing quaternary carbon stereocenters (*34*–*39*). Among these, CADA reactions of alkynes have attracted particular attention over the past decade. However, these reactions generally depend on the transition metals (Fig. 2B, left), except those based on electron-deficient alkynes involving Michael-type addition (*40*–*42*), in which chiral BAs did not directly activate the carbon-carbon triple bonds (Fig. 2B, middle). Very recently, our group disclosed the CADA reactions of naphthol-, phenol-, and pyrrole-ynamides (*43*–*53*) via direct activation of the electron-rich alkynes by BAs, leading to various valuable spirocyclic enones and 2*H*-pyrroles bearing a chiral quaternary carbon stereocenter with excellent chemo-, regio-, and enantioselectivities (Fig. 2B, middle) (*54*). Inspired by the above results and by our recent work on developing homopropargyl amides for N-heterocycle synthesis (*55*–*58*), we envisioned that the reaction of homopropargyl amines with chiral BAs would deliver chiral VQM intermediates, which could be further attacked by intramolecular nucleophiles such as phenols and indoles to generate the axially chiral cyclohexadienone intermediates and centrally chiral imine intermediates, followed by BA-catalyzed intramolecular nucleophilic addition to eventually afford the centrally chiral fused polycyclic N-heterocycles (Fig. 2B, right). However, realizing this cascade cyclization in such an orderly manner is highly challenging: (i) how to prevent the competing cyclization of the alkyne moiety by the highly nucleophilic phenol or indole moiety; (ii) how to achieve the desired cascade cyclization but not stop at the dearomatization step; and (iii) how to control the enantioselectivity and diastereoselectivity. Here, we report such a CADA reaction of phenol- and indole-tethered homopropargyl amines by using chiral phosphoric acid (CPA) or spinol phosphoric acid (SPA) as catalyst (*59*–*65*), allowing the practical and atom-economical synthesis of a diverse array of valuable fused polycyclic enones and indolines bearing a chiral quaternary carbon stereocenter in good yields with high enantioselectivities and diastereoselectivities (Fig. 2C).

## RESULTS

At the outset, the phenol-tethered homopropargyl amine **1a** was used as the model substrate to optimize the reaction conditions, and selected results are listed in Table 1. To our delight, the reaction proceeded smoothly at 60°C in the presence of racemic diphenyl phosphate, providing the desired polycyclic enone **2a** in 99% yield (Table 1, entry 1). Encouraged by this result, we then investigated a variety of CPAs **A1** to **A6** as catalysts (Table 1, entries 2 to 7) and were pleased to find that **2a** could be obtained in 92% yield with 96% enantiomeric excess (ee) by using **A4** as catalyst via a remote control of enantioselectivity (Table 1, entry 5). The use of other more acidic catalysts such as chiral phosphoramide **A7** and SPAs **A8** to **A9** failed to improve the reaction (Table 1, entries 8 to 10). Further screening of solvents such as 1,2-dichloroethane (DCE), benzene, and *tert*-butyl methyl ether led to a slightly decreased yield and enantioselectivity (Table 1, entries 11 to 13). In addition, the reaction proved to be less efficient when performed at room temperature (rt) (Table 1, entry 14).

**Table 1.**
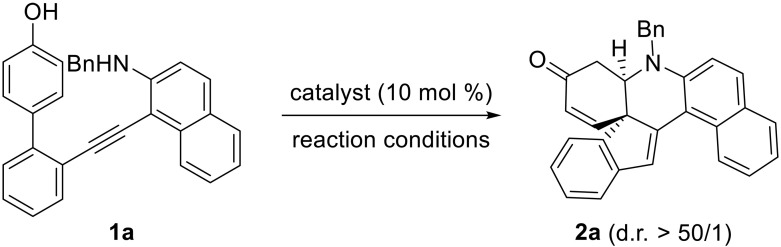
Optimization of reaction conditions. Reaction conditions: **1a** (0.05 mmol), catalyst (0.005 mmol), solvent (1 ml), room temperature (rt) to 60°C, 2 to 96 hours, in vials.

Entry	Catalyst	Reaction conditions	Yield (%)*	ee (%)†
1	(PhO)_2_PO_2_H	DCM, 60°C, 2 hours	99	–
2	**A1**	DCM, 60°C, 6 hours	95	50 (+)
3	**A2**	DCM, 60°C, 6 hours	92	74 (+)
4	**A3**	DCM, 60°C, 6 hours	86	88 (+)
**5**	**A4**	**DCM, 60°C, 6 hours**	**92**	**96 (+)**
6	**A5**	DCM, 60°C, 6 hours	83	72 (+)
7	**A6**	DCM, 60°C, 6 hours	94	80 (+)
8	**A7**	DCM, 60°C, 12 hours	86	24 (+)
9	**A8**	DCM, 60°C, 12 hours	92	74 (−)
10	**A9**	DCM, 60°C, 12 hours	41	60 (−)
11	**A4**	DCE, 60°C, 6 hours	90	92 (+)
12	**A4**	PhH, 60°C, 5 hours	92	93 (+)
13	**A4**	*^t^*BuOMe, 60°C, 10 hours	86	95 (+)
14	**A4**	DCM, rt, 96 hours	51	96 (+)

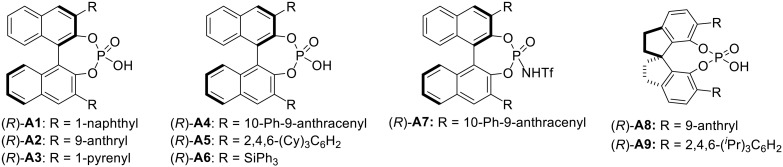

*Measured by ^1^H nuclear magnetic resonance (NMR) using 2,6-dimethoxytoluene as the internal standard.

†Determined by high-performance liquid chromatography analysis on a chiral stationary phase.

**Fig. 3.**
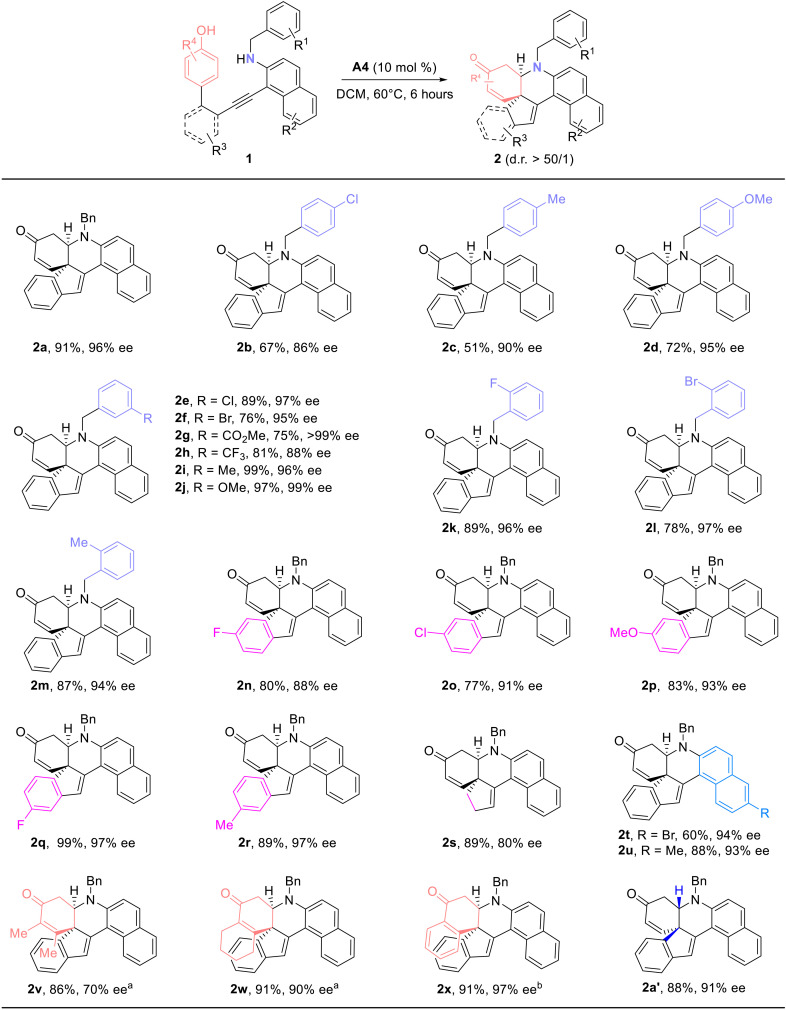
Synthesis of chiral fused polycyclic enones 2. Reaction conditions: **1** (0.1 mmol), **A4** (0.01 mmol), dichloromethane (DCM; 2 ml), 60°C, 6 hours, in vials; yields are those for the isolated products; ees are determined by high-performance liquid chromatography (HPLC) analysis on a chiral stationary phase. a, **A8** (0.005 mmol), DCM (2 ml), 60°C, 5 hours, in vials; b, **A6** (0.01 mmol), benzene (2 ml), rt, 12 hours, in vials. mol %, mole percent.

With the optimized reaction conditions in hand (Table 1, entry 5), the scope of this CPA-catalyzed cascade cyclization was explored. As depicted in Fig. 3, in addition to the benzyl-protected model substrate **1a**, different benzyl-substituted homopropargyl amines were suitable substrates to furnish the corresponding chiral fused polycyclic enones **2b** to **2m** in moderate to good yields with 86 to >99% ees. Subsequently, we turned our attention to study the substrates bearing different biaryl moieties. The positions and the electronic properties of the R^3^ groups had a negligible impact on this reaction, affording the expected products **2n** to **2r** in high yields (77 to 99%) and enantioselectivities (88 to 97% ees). Moreover, the reaction was also extended to the linearly connected substrate **1s**, and the desired product **2s** was obtained in 89% yield with 80% ee. In addition, substrates with different R^2^ groups on the naphthalene ring also underwent smooth cyclization, furnishing the expected polycyclic enones **2t** (60%, 94% ee) and **2u** (88%, 93% ee), respectively. Substituted phenol substrates **1v** to **1x** were all tolerated by using **A8** or **A6** as catalyst. Last, the reaction occurred smoothly by the use of **A4** with the opposite configuration as chiral catalyst, delivering the desired **2a′** in 88% yield and 91% ee. The absolute configuration of product **2g** was confirmed by x-ray diffraction. Notably, excellent diastereoselectivities [d.r. > 50/1; determined by ^1^H nuclear magnetic resonance (NMR) analysis of the reaction mixtures] were achieved in all of these cases. Thus, this protocol constitutes the first CADA reaction of *para*-phenols tethered alkynes catalyzed by BA via the activation of carbon-carbon triple bonds. This cascade cyclization provides a highly efficient and practical route for the construction of valuable chiral fused polycyclic enones bearing two contiguous stereogenic centers. Despite the fact that impressive CADA reactions of phenols have been developed, these protocols generally focus on the transition metal catalysis or oxidation strategy, such as using chiral hypervalent iodine reagent and *m*-CPBA (3-chloroperoxybenzoic acid) as the external oxidant (*59*–*63*).

**Fig. 4.**
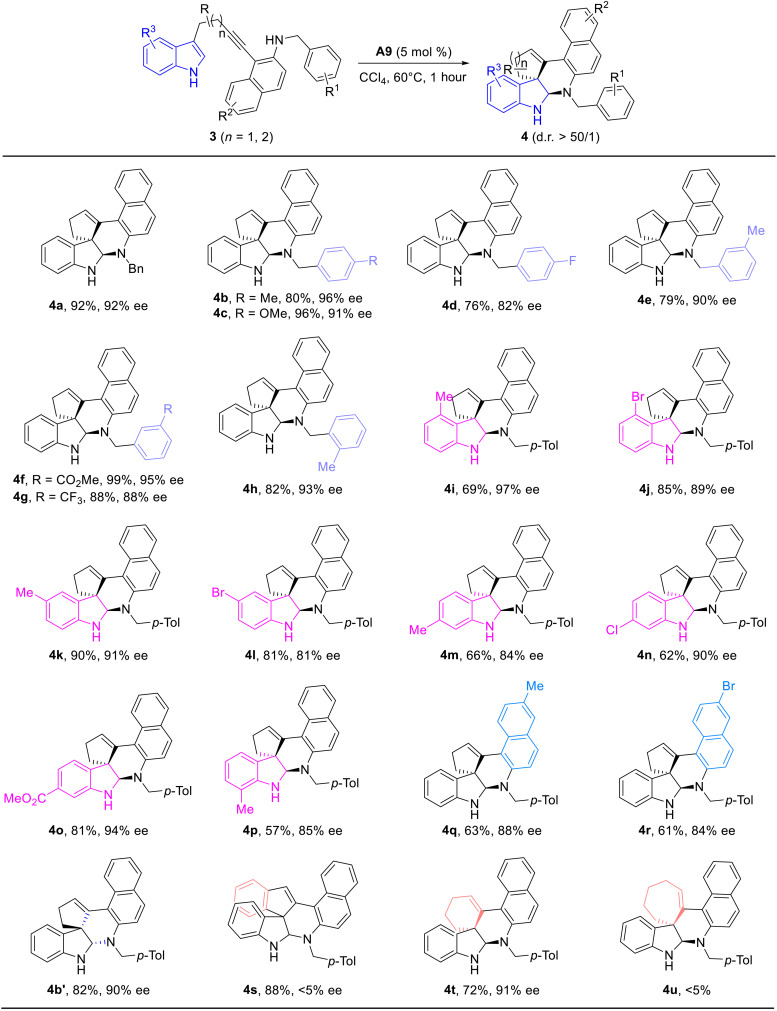
Synthesis of chiral fused polycyclic indolines 4. Reaction conditions: **3** (0.1 mmol), **A9** (0.005 mmol), CCl_4_ (2 ml), 60°C, 1 hour, in vials; yields are those for the isolated products; ees are determined by HPLC analysis on a chiral stationary phase.

Then, we considered the possibility of extending the above CADA to indole-tethered homopropargyl amines. Notably, achieving this cascade cyclization is more challenging, owing to the more nucleophilic indole moiety, which might trap the alkyne moiety directly. Under the optimized reaction conditions by the use of 5 mole percent of chiral SPA **A9** as catalyst (see the table S1), we were pleased to find that this CADA occurred smoothly for a variety of indolyl homopropargyl amines **3**, and the corresponding chiral polycyclic indolines **4** were formed in moderate to excellent yields with generally high enantioselectivities and excellent diastereoselectivities (d.r. > 50/1). As summarized in Fig. 4, a wide array of aryl-substituted homopropargyl amines bearing both electron-withdrawing and electron-donating groups at para, meta, and even ortho positions were well tolerated in this cascade cyclization to produce the expected indolines **4a** to **4h** in good to excellent yields (76 to 99%) with high enantioselectivities (82 to 96% ees). In addition, the reaction proceeded efficiently with various indole-tethered homopropargyl amines **3** bearing both electron-donating and electron-withdrawing groups on the aromatic indole ring, affording the desired polycyclic indolines **4i** to **4p** in 57 to 90% yields with 81 to 97% ees. As expected, different substituents on the naphthalene ring were also tolerated to deliver the desired products **4q** (63%, 88% ee) and **4r** (61%, 84% ee), respectively. Again, the use of **A9** with the opposite configuration as catalyst led to the corresponding **4b′** in 82% yield and 90% ee. In the case of the phenyl-linked 3-indole-homopropargyl amine **3s**, the desired indoline **4s** was formed in 88% yield with almost no enantioselectivity (see table S2). Furthermore, this cascade cyclization could also be extended to the preparation of the fused polycyclic skeleton **4t** (*n* = 2) in 72% yield with 91% ee. Our attempts to extend the reaction to the indolyl homopropargyl amine **3u** (*n* = 3) only led to the formation of complicated mixtures, and attempts to synthesize the pyrrole-tethered homopropargyl amines failed. The absolute configuration of product **4j** (recrystallization to 99% ee) was confirmed by x-ray diffraction. Chiral BA–catalyzed dearomatization reaction of indole-tethered alkynes via the electrophilic activation of alkynes, however, has not been reported to the best of our knowledge (*40*, *66*).

**Fig. 5.**
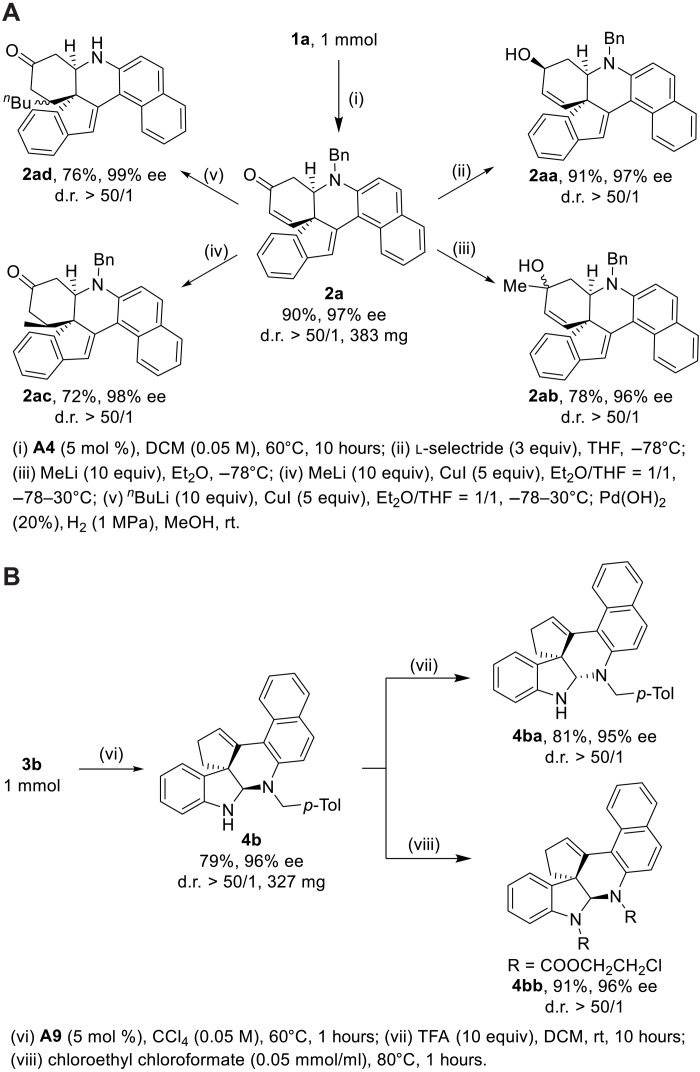
Synthetic applications. (**A**) Preparative-scale reaction of **1a** and synthetic applications. THF, tetrahydrofuran. (**B**) Preparative-scale reaction of **3b** and synthetic applications. TFA, trifluoroacetic acid.

To further demonstrate the synthetic potential of this chemistry, the preparative-scale reaction of homopropargyl amines **1a** and **3b** was first conducted under the optimal reaction conditions, and the desired products **2a** and **4b** were obtained in 90 and 79% yields, respectively (Fig. 5). Then, several synthetic transformations were explored. It was found that the reduction of the carbonyl group of **2a** by l-selectride led to the corresponding alcohol **2aa** in 91% yield. In addition, **2a** could undergo selective addition by MeLi and Me_2_CuLi to deliver the valuable spirocyclic compounds **2ab** and **2ac** (the relative configuration was assigned by nuclear Overhauser effect spectroscopy experiments; for more details, see the Supplementary Materials), respectively. Moreover, the debenzylation product **2ad** bearing three contiguous stereogenic centers was obtained through selective Michael addition followed by hydrogenation. On the other hand, indoline **4b** could be readily converted into the thermodynamically stable product **4ba** and amide product **4bb** by the treatment with trifluoroacetic acid and chloroethyl chloroformate (*67*), respectively. Excellent enantio- and diastereoselectivities (d.r. > 50/1) were achieved in all of these transformations. The molecular structures of **2aa** and **4bb** were further confirmed by x-ray diffraction. Last, it is notable that preliminary biological tests revealed a potential application of these heterocyclic compounds in medicinal chemistry (see table S3).

**Fig. 6.**
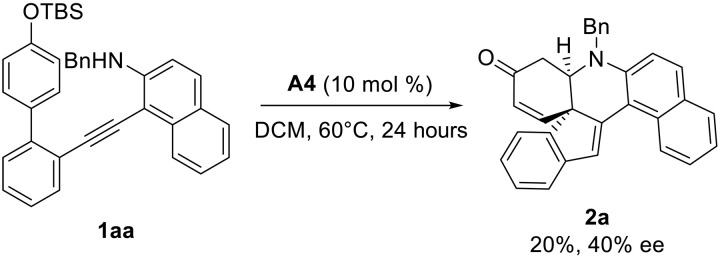
Mechanistic investigations. Substrate scope of t-butyldimethylsilyl (TBS)-protected substrate **1aa**.

To understand the reaction mechanism, a control experiment was first conducted. With the tert-Butyldimethylsilyl chloride (TBS)-protected substrate **1aa** under the standard reaction conditions, the corresponding product **2a** was formed in 20% yield with 40% ee (Fig. 6). This result indicated that the O─H of the phenol may help reduce the barrier to accelerate the Michael addition step and is essential for this asymmetric control (*66*).

**Fig. 7.**
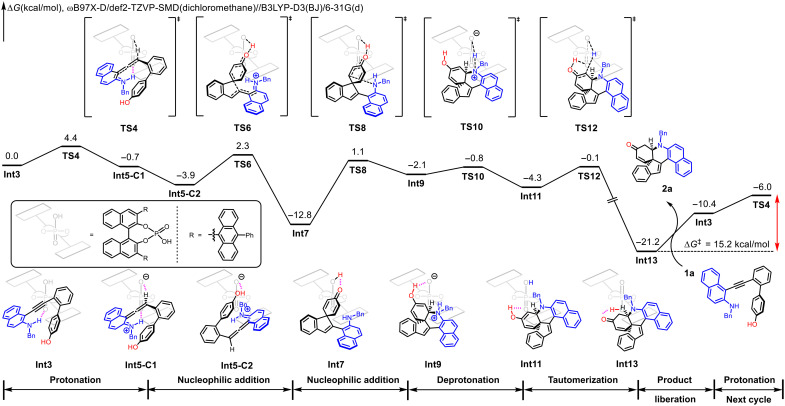
Mechanistic hypothesis. Density functional theory (DFT)–computed free energy diagram of CPA-catalyzed asymmetric dearomatization reaction of the homopropargyl amine **1a**.

To further understand the mechanistic details, computational calculations were performed using substrate **1a** and **CPA-A4** as models (*68*–*74*). The density functional theory (DFT)–computed free energy diagram of the operative mechanism is shown in Fig. 7. From the substrate-CPA complex **Int3**, the CPA catalyst protonates the alkyne substrate via **TS4**, generating the cationic axially chiral allene intermediate **Int5-C1**. **Int5-C1** undergoes a conformational change to form a more stable conformer **Int5-C2**, and subsequent intramolecular nucleophilic addition through **TS6** produces the enone intermediate **Int7**. The direct intramolecular nucleophilic addition of **Int5-C1** is possible but requires a higher barrier as compared to **TS6** (see fig. S1). From **Int7**, the nucleophilic addition of the amine group via **TS8** results in the C─N bond formation and generation of **Int9**. This step transfers the axial chirality of **Int7** to the adjacent quaternary and tertiary stereogenic centers of **Int9**. The ammonium group of **Int9** is deprotonated by CPA anion via **TS10**, leading to the enol intermediate **Int11**. Subsequent tautomerization via **TS12** produces the ketone product–CPA complex **Int13**. **Int13** then liberates the product **2a** and regenerates the active intermediate **Int3** for the next catalytic cycle. Because of the endergonic product liberation, **Int13** is the on-cycle resting state of the catalytic cycle; the protonation via **TS4** is the rate- and enantioselectivity-determining step, which requires a 15.2 kcal/mol barrier as compared to **Int13**. We also verified the stability of axial chirality of **Int7** to ensure that the in-cycle racemization is unfeasible (see fig. S2). In the cases where indole-tethered homopropargyl amines **3** are used, the described sequence presumably involves the cyclization onto C3 position of indole moiety followed by intramolecular cyclization of in situ–generated imine species.

**Fig. 8.**
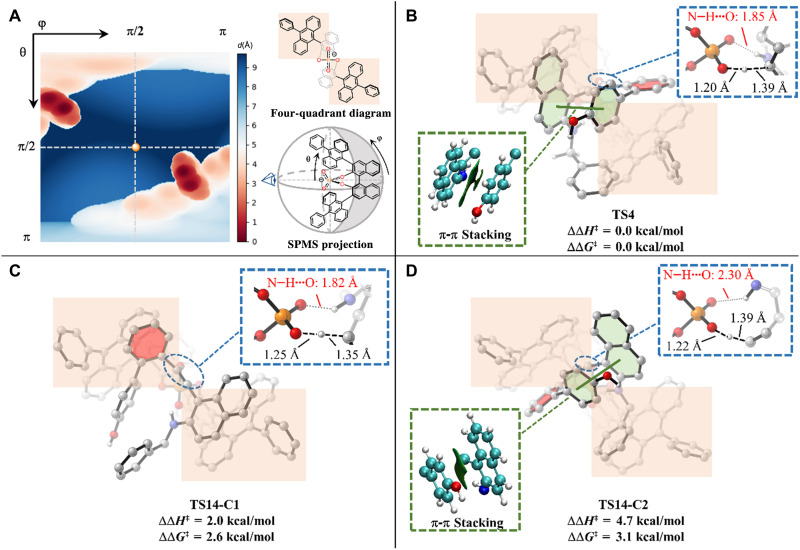
DFT-computed free energy differences of enantioselec-tive protonation transition states and rationalization of chirality control. (**A**) Chiral steric environment of the phosphate anion of **CPA-A4**. (**B**) Optimized structure and relative energies of the major protonation transition state **TS4**. (**C**) Optimized structure and relative energies of the minor protonation transition state **TS14-C1**. (**D**) Optimized structure and relative energies of the minor protonation transition state **TS14-C2**.

We next explored the origins of enantioselectivity by calculating the enantioisomeric protonation transition states (Fig. 8). The chiral steric environment of the phosphate anion of **CPA-A4** is elaborated in Fig. 8A using the spherical projection descriptor of molecular stereostructure (SPMS) method (*75*) developed by us. The SPMS method projects the van der Waals surface of the stereostructure from a designated center (phosphine of **CPA-A4** in this case), which gives a quantified steric representation with atomic resolution. For the phosphate anion of **CPA-A4**, the bulky 10-Ph-9-anthracenyl substituents are positioned in the second and fourth quadrants (represented by the red regions), which corroborated the empirical understanding based on the four-quadrant diagram. In the favored transition state **TS4**, the phenyl group (highlighted in red) of the biphenyl moiety of the substrate is placed in the first quadrant, which avoids the steric repulsions with the CPA anion. This allows the favorable hydrogen bonding between CPA and amine (N─H···O being 1.85 Å) and intramolecular π-π stacking of the substrate in the favorable protonation transition state **TS4**. In the enantioisomeric transition state **TS14-C2**, the highlighted phenyl group of the substrate is now placed in the second quadrant, which dissociates the substrate from the CPA anion and removes the favorable hydrogen bonding (N─H···O being 2.30 Å). **TS14-C2** is 3.1 kcal/mol less favorable as compared to **TS4** in terms of free energy. We can also locate a conformation of the minor protonation transition state **TS14-C1**, which retains the N─H···O hydrogen bonding (N─H···O being 1.82 Å), but the π-π stacking is no longer present. Therefore, only the major protonation transition state **TS4** allows the favorable N─H···O hydrogen bonding and π-π stacking in the confined environment of protonation process, which results in the observed stereoselectivity.

## DISCUSSION

In summary, we have developed a chiral BA–catalyzed dearomatization reaction of phenol- and indole-tethered homopropargyl amines, where chiral BA catalyzes both the asymmetric dearomatization and asymmetric intramolecular nucleophilic addition process in an orderly manner by activation of C─C triple bonds and C─X double bonds, respectively. This method allows the practical and atom-economical synthesis of a diverse array of valuable fused polycyclic enones and indolines bearing a chiral quaternary carbon stereocenter in moderate to excellent yields (up to 99% yield) with excellent diastereoselectivities (d.r. > 50/1) and generally excellent enantioselectivities (up to >99% ee). Notably, this protocol represents the first example of CADA reactions catalyzed by BA via VQMs. The utility of this methodology was illustrated through further transformations into a series of chiral fused polycyclic N-heterocycles. Moreover, this VQM-involved cascade cyclization and the origin of enantioselectivity are strongly supported by theoretical calculations. Further exploration of chiral BA–catalyzed alkyne transformation by the activation of inert alkynes is now underway in our laboratory.

## MATERIALS AND METHODS

Unless otherwise noted, materials were obtained commercially and used without further purification. All the solvents were treated according to general methods. Flash column chromatography was performed over silica gel (300 to 400 mesh). See Supplementary Materials and Methods for experimental details.

^1^H NMR spectra were recorded on a Bruker AV-400 spectrometer and a Bruker AV-500 spectrometer in chloroform-d_3_. Chemical shifts are reported in parts per million (ppm) with the internal tetramethylsilane (TMS) signal at 0.0 ppm as a standard. The data are reported as follows: s = singlet, d = doublet, t = triplet, m = multiplet or unresolved, brs = broad singlet, coupling constant(s) in hertz, integration. ^13^C NMR spectra were recorded on a Bruker AV-400 spectrometer and a Bruker AV-500 spectrometer in chloroform-d_3_. Chemical shifts are reported in ppm with the internal chloroform signal at 77.0 ppm as a standard. Mass spectra were recorded with a Micromass quadrupole/time-of-flight tandem mass spectrometer using electron spray ionization.

### General procedure for the synthesis of chiral fused polycyclic enones 2

**A4** (8.5 mg, 0.01 mmol) was added to a solution of homopropargyl amine **1** (0.10 mmol) in dichloromethane (DCM; 2.0 ml) at rt. The reaction mixture was then stirred at 60°C, and the reaction progress was monitored by thin-layer chromatography (TLC). The reaction typically took 6 hours. Upon completion, the mixture was concentrated under reduced pressure, and the residue was purified by column chromatography on silica gel (hexanes/ethyl acetate) to afford the desired product **2**.

### General procedure for the synthesis of chiral fused polycyclic indolines 4

**A9** (3.6 mg, 0.005 mmol) was added to a solution of homopropargyl amine **3** (0.10 mmol) in CCl_4_ (2.0 ml) at rt. The reaction mixture was then stirred at 60°C, and the reaction progress was monitored by TLC. The reaction typically took 1 hour. Upon completion, the mixture was concentrated under reduced pressure, and the residue was purified by column chromatography on silica gel (hexanes/ethyl acetate) to afford the desired product **4**.
