# Supplementary material for: Brønsted acid–catalyzed asymmetric dearomatization for synthesis of chiral fused polycyclic enone and indoline scaffolds
Source: Sci Adv. 2023 Mar 15;9(11):eadg4648. doi: 10.1126/sciadv.adg4648 (PMC10017053; doi:10.1126/sciadv.adg4648)

Science Advances
AAAS

Supplementary Materials for

Brønsted acid–catalyzed asymmetric dearomatization for synthesis of chiral fused polycyclic enone and indoline scaffolds

Tong-De Tan *et al.*

Corresponding author: Long-Wu Ye, longwuye@xmu.edu.cn; Xin Hong, hxchem@zju.edu.cn; Peng-Cheng Qian, qpc@wzu.edu.cn

Sci. Adv. **9**, eadg4648 (2023)
DOI: 10.1126/sciadv.adg4648

This PDF file includes:

Supplementary Materials and Methods
Supplementary Text
Figs. S1 and S2
Tables S1 to S14

1. General Information

Ethyl acetate (ACS grade), hexanes (ACS grade) and anhydrous 1,2-dichloroethane (ACS grade) were obtained commercially and used without further purification. Methylene chloride, tetrahydrofuran and diethyl ether were purified according to standard methods unless otherwise noted. Commercially available reagents were used without further purification. Reactions were monitored by thin layer chromatography (TLC) using silicycle pre-coated silica gel plates. Flash column chromatography was performed over silica gel (300-400 mesh). Infrared spectra were recorded on a Nicolet AVATER FTIR330 spectrometer as thin film and are reported in reciprocal centimeter (cm^{-1}). Mass spectra were recorded with Micromass QTOF2 Quadrupole/Time-of-Flight Tandem mass spectrometer using electron spray ionization.

^{1}H NMR spectra were recorded on a Bruker AV-400 spectrometer and a Bruker AV-500 spectrometer in chloroform-d_3. Chemical shifts are reported in ppm with the internal TMS signal at 0.0 ppm as a standard. The data is being reported as (s = singlet, d = doublet, t = triplet, m = multiplet or unresolved, brs = broad singlet, coupling constant(s) in Hz, integration).

^{13}C NMR spectra were recorded on a Bruker AV-400 spectrometer and a Bruker AV-500 spectrometer in chloroform-d_3. Chemical shifts are reported in ppm with the internal chloroform signal at 77.0 ppm as a standard.

2. More Reaction Condition Studies

Table S1. For reaction condition studies on the formation of fused polycyclic indoline **4a**[a]

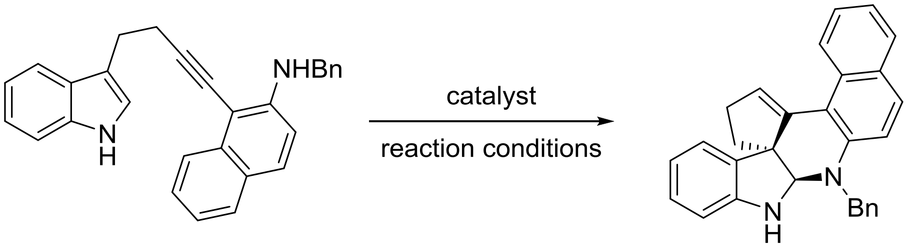

3a **4a**

entry	catalyst	reaction conditions	yield (%)[b] 4a	ee (%)[c] 4a
1	$(PhO)_2POOH$	DCM, rt, 6 h	99	-
2	**A1** (10 mol %)	DCM, rt, 10 h	90	30 (+)
3	**A2** (10 mol %)	DCM, rt, 10 h	91	10 (+)
4	**A3** (10 mol %)	DCM, rt, 10 h	92	28 (+)
5	**A4** (10 mol %)	DCM, rt, 10 h	89	2 (+)
6	**A5** (10 mol %)	DCM, rt, 10 h	91	<1
7	**A6** (10 mol %)	DCM, rt,10 h	90	6 (+)
8	**A7** (5 mol %)	DCM, rt, 10 h	90	50 (+)
9	**A8** (5 mol %)	DCM, rt, 10 h	92	70 (+)
10	**A9** (5 mol %)	DCM, rt, 6 h	92	86 (-)
11	**A9** (5 mol %)	CCl_4, rt, 15 h	93	88 (-)
**12**	**A9 (5 mol %)**	**CCl_4, 60 °C, 1 h**	**94**	**92 (-)**

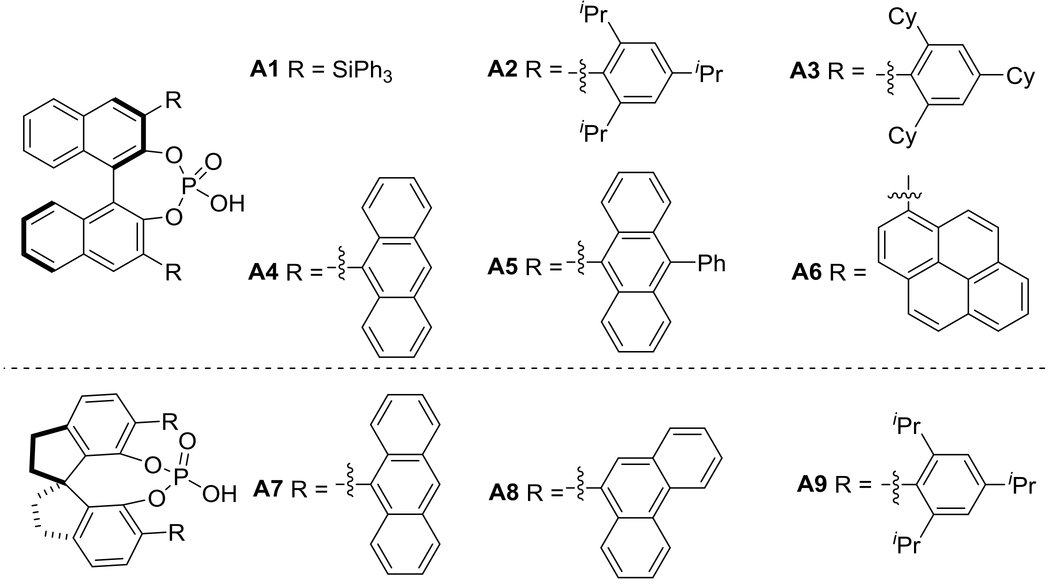

[a]Reaction conditions: **3a** (0.05 mmol), catalyst (0.0025-0.005 mmol), solvent (1 mL), rt-60 °C, 1-15 h, in vials. [b]Measured by ^1H NMR using 2,6-dimethoxytoluene as the internal standard. [c]Determined by HPLC analysis on a chiral stationary phase.

Table S2. For reaction condition studies on the formation of fused polycyclic indoline **4s**[a]

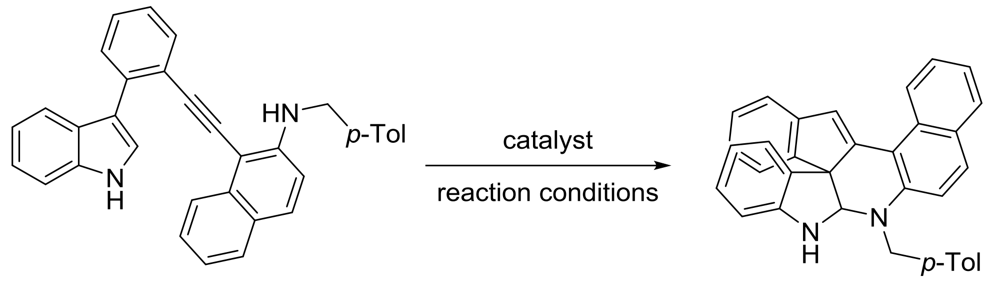

3s **4s**

entry	catalyst	reaction conditions	yield (%)[b] **4a**	ee (%)[c] **4a**
1	$(PhO)_2POOH$	DCM, rt, 6 h	84	-
2	**A1** (10 mol %)	DCM, rt, 15 h	80	<1
3	**A2** (10 mol %)	DCM, rt, 15 h	82	1
4	**A3** (10 mol %)	DCM, rt, 15 h	84	<1
5	**A4** (10 mol %)	DCM, rt, 15 h	83	2
6	**A5** (10 mol %)	DCM, rt, 15 h	81	3
7	**A6** (5 mol %)	DCM, rt,15 h	88	6
8	**A7** (5 mol %)	DCM, rt, 15 h	89	<1

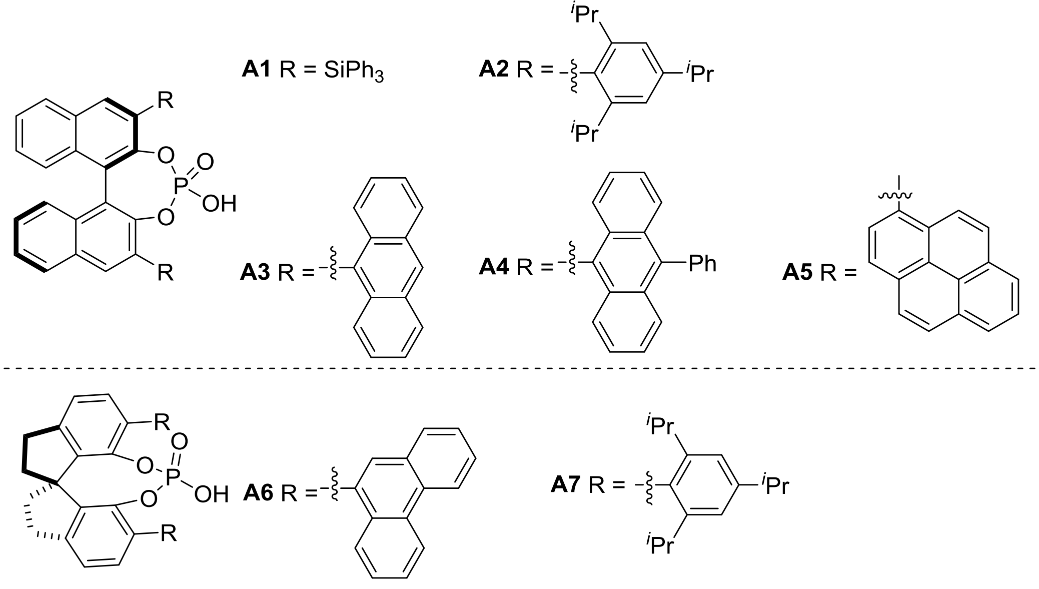

[a]Reaction conditions: **3s** (0.05 mmol), catalyst (0.0025-0.005 mmol), solvent (1 mL), rt, 1-15 h, in vials. [b]Measured by ^1H NMR using 2,6-dimethoxytoluene as the internal standard. [c]Determined by HPLC analysis on a chiral stationary phase.

3. Biological Tests

Preliminary Cell Viability Assay

We also tested the newly synthesized fused polycyclic enones **2** and fused polycyclic indolines **4** for their bioactivity as antitumor agents. The cytotoxic effects of these compounds were evaluated against a panel of cancer cells, including melanoma cells A375, breast cancer cells MDA-MB-231 and MCF-7, osteosarcoma cancer cells U2OS, leukemia cells HL-60, lymphoma cells JeKo-1 and HepG2 cells by the use of cell viability assay, using a commercially available proliferation assay kit (Promega, US). Briefly, the cells were plated in 96-well culture plates at an appropriate density in culture medium and allowed to attach overnight. After treatment of vehicle (0.1% DMSO as control) or test compounds for indicated times and concentrations, 20 μL of MTS reaction solution (3-(4,5-dimethylthiazol-2-yl)-5-(3-carboxymethoxyphenyl)-2-(4-sulfophenyl)-2H-tetrazolium, inner salt; MTS (a) and 100 μg/mL phenazine methosulfate; PES) was added to each well. The absorbance values were read at 490 nm wavelength with a spectrophotometer (Varioskan Flash, Thermo, US) after 1 to 4 hours incubation. The cell viability was calculated as: cell survival = (ODcompd. - ODblank)/(ODcontrol - ODblank)*100%.

Table S3. The cytotoxic effects of the newly synthesized fused polycyclic enones **2** and fused polycyclic indolines **4** against cancer cells

Cmpd ID	Cell viability at 20 uM (%)									
	BGC823	SKGT4	A375	MDA-MB-231	MCF-7	U2OS	Jeko-1	K562	HepG2	kyse450
**2a**	96.4	86.6	106.0	118.0	115.0	93.8	106.3	99.3	100.4	97.8
**2b**	94.9	119.2	108.0	109.0	120.3	90.9	105.3	96.2	102.1	105.1
**2c**	96.3	87.2	104.0	90.0	120.0	92.6	95.6	82.7	100.4	82.5
**2d**	96.9	104.2	96.0	89.0	118.9	90.1	95.3	87.8	102.0	105.1
**2e**	105.4	101.3	108.0	90.0	114.1	88.0	93.5	88.9	100.6	107.6
**2f**	84.5	118.1	105.0	80.0	108.6	86.5	92.1	93.9	98.5	102.2
**2g**	94.9	98.2	105.0	80.0	108.5	88.7	92.8	97.2	96.1	84.5
**2h**	85.2	103.5	96.0	88.0	111.3	85.8	90.9	94.6	101.0	92.0
**2i**	97.8	99.0	91.0	121.0	118.6	91.2	100.9	95.5	98.6	99.4
**2j**	81.8	86.3	89.0	115.0	121.0	90.4	98.1	92.6	96.9	80.3
**2k**	99.0	126.1	83.0	103.0	111.9	92.7	103.7	90.2	94.8	62.7
**2l**	86.2	103.1	91.0	105.0	110.3	92.3	98.0	88.7	98.3	65.8
**2m**	101.6	103.5	93.0	106.0	112.5	88.9	94.6	88.8	99.2	86.4
**2n**	108.0	117.2	85.0	92.0	112.9	86.2	87.0	86.1	101.0	104.0
**2o**	113.6	114.5	89.0	89.0	111.9	85.2	87.3	87.7	98.0	78.2
**2p**	126.2	109.8	84.0	84.0	110.6	87.5	89.5	96.7	91.3	88.6
**2q**	95.3	90.0	93.0	113.0	110.5	89.2	105.1	91.2	100.7	104.0
**2r**	95.1	88.9	95.0	118.0	110.8	86.5	103.2	94.0	99.0	102.5
**2s**	101.9	108.6	76.0	90.0	118.5	87.5	98.6	90.7	85.4	83.1
**2t**	88.2	94.9	95.0	114.0	119.6	89.0	100.5	83.1	97.0	101.5
**2u**	103.8	107.1	97.0	113.0	111.5	87.8	101.7	90.3	93.0	104.9
**2v**	122.7	122.1	94.0	103.0	103.7	89.0	89.3	89.6	93.2	108.5
**2w**	121.0	97.1	88.0	100.0	108.0	85.3	85.0	89.6	95.0	107.1
**2x**	88.9	104.0	103.0	106.0	101.8	100.0	96.6	91.4	97.9	101.4
**4a**	85.2	97.9	94.0	95.0	106.7	95.8	77.8	83.9	96.5	100.2
**4b**	85.9	88.5	95.0	88.0	104.3	98.5	78.1	90.3	99.7	99.0
**4c**	90.2	101.0	84.0	91.0	101.6	91.7	57.7	71.7	98.3	103.2

**4d**	101.9	87.9	93.0	92.0	110.9	92.6	88.6	77.9	100.2	102.9
**4e**	105.0	91.4	96.0	86.0	107.6	95.5	93.1	87.6	97.3	107.4
**4f**	99.7	93.0	92.0	80.0	100.9	89.9	82.3	78.7	96.0	103.2
**4g**	116.4	100.6	85.0	86.0	98.6	89.0	86.0	83.3	95.8	106.4
**4h**	80.7	104.4	100.0	136.0	108.2	89.5	86.0	79.8	100.8	107.2
**4i**	90.0	91.3	103.0	147.0	110.3	97.9	43.8	66.8	104.8	109.6
**4j**	91.2	97.4	101.0	139.0	100.9	99.6	99.8	87.9	101.5	110.8
**4k**	96.7	108.8	101.0	119.0	108.4	99.0	99.6	87.2	99.6	110.3
**4l**	101.7	100.8	98.0	130.0	108.5	94.8	92.1	82.4	99.8	108.8
**4m**	108.7	108.2	92.0	124.0	104.9	97.8	99.0	77.9	104.0	109.5
**4n**	123.2	95.2	95.0	128.0	102.6	95.1	76.6	68.3	101.5	100.7
**4o**	122.1	114.5	95.0	111.0	102.1	96.7	103.6	82.0	104.4	104.3
**4p**	94.0	99.0	102.0	143.0	109.6	95.6	97.3	93.6	104.3	105.8
**4q**	95.9	82.5	99.0	142.0	106.4	95.2	78.6	89.8	101.2	99.7
**4r**	76.3	108.9	99.0	143.0	103.9	93.8	72.6	80.8	99.0	106.9
**4s**	98.1	116.5	104.0	139.0	106.7	98.9	99.7	86.7	106.2	104.4
**4t**	101.8	108.1	98.0	134.0	103.7	95.0	91.5	84.1	104.4	102.8

Results are average of two experiments.

4. Preparation of Starting Materials

Compounds **1a-1r**, **1t-1x** were prepared according to the following known procedures (*31*).

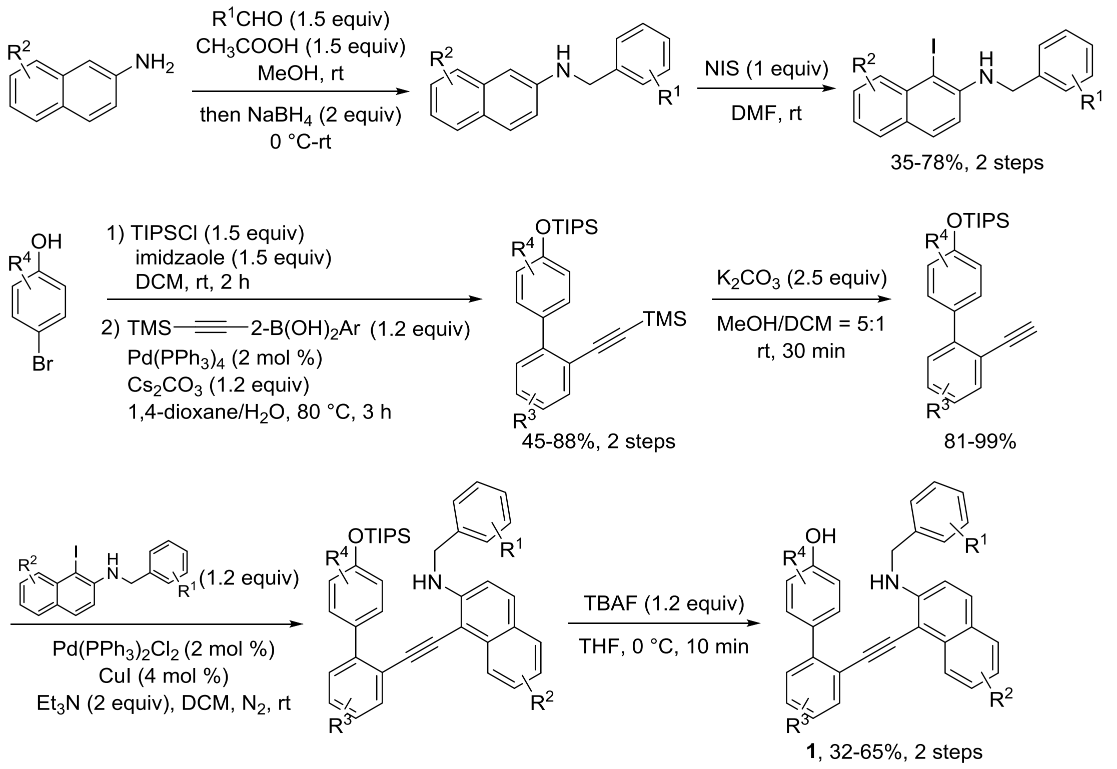

2'-((2-(benzylamino)naphthalen-1-yl)ethynyl)-[1,1'-biphenyl]-4-ol (1a)

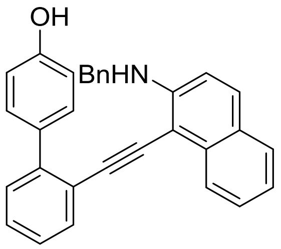

1a

The product was isolated through silica gel column chromatography (PE:EA = 5:1) as a pale yellow oil. ^{1}H NMR (500 MHz, $CDCl_3$) δ 7.98 (d, *J* = 8.0 Hz, 1H), 7.72 – 7.69 (m, 1H), 7.57 (d, *J* = 8.0 Hz, 1H), 7.50 – 7.45 (m, 3H), 7.42 – 7.38 (m, 1H), 7.34 – 7.30 (m, 3H), 7.29 – 7.14 (m, 6H), 6.76 – 6.71 (m, 3H), 5.13 (s, 1H), 4.60 (s, 1H), 4.35 (d, *J* = 4.5 Hz, 2H); ^{13}C NMR (125 MHz, $CDCl_3$) δ 155.1, 147.9, 142.8, 139.4, 134.3, 133.8, 132.3, 130.4, 130.1, 129.4, 128.6, 128.1, 127.9, 127.1, 127.0, 126.9, 126.5, 124.2, 122.5, 122.3, 117.2, 115.2, 112.7, 100.8, 99.4, 87.8, 47.0; IR

(neat): 3396(br), 2928, 2188, 1620, 1595, 1518, 1331, 1263, 1124, 1072, 836, 749, 702; HRESIMS Calcd for $[C_{31}H_{23}NNaO]^+$ (M + Na^+) 448.1672, found 448.1675.

***N*-benzyl-1-((4'-((*tert*-butyldimethylsilyl)oxy)-[1,1'-biphenyl]-2-yl)ethynyl)naphthalen-2-amine (1aa)**

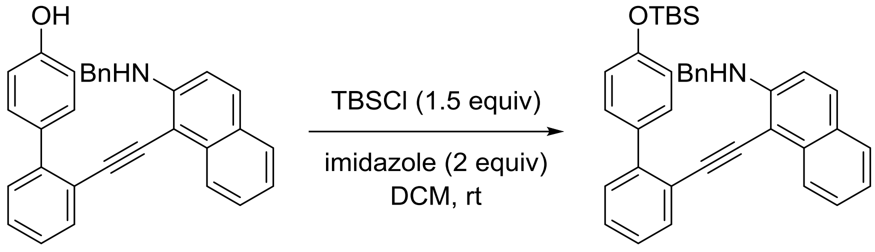

1a **1aa**, 94%

The product was isolated through silica gel column chromatography (PE:EA = 20:1) as a pale yellow oil. ^{1}H NMR (400 MHz, $CDCl_3$) δ 8.01 (d, *J* = 8.4 Hz, 1H), 7.73 – 7.69 (m, 1H), 7.59 – 7.49 (m, 4H), 7.41 (t, *J* = 8.0 Hz, 1H), 7.37 – 7.15 (m, 9H), 6.85 (d, *J* = 8.4 Hz, 2H), 6.77 (d, *J* = 8.8 Hz, 1H), 5.22 (s, 1H), 4.41 (s, 2H), 0.96 (s, 9H), 0.15 (s, 6H); ^{13}C NMR (100 MHz, $CDCl_3$) δ 155.3, 148.1, 142.9, 139.4, 134.4, 134.3, 132.4, 130.3, 130.0, 129.5, 128.6, 128.1, 127.9, 127.1, 126.8(9), 126.8(6), 126.5(2), 126.4(7), 124.2, 122.6, 122.2, 119.7, 112.8, 100.7, 99.4, 88.0, 47.0, 25.6, 18.1, -4.5; IR (neat): 3128, 2920, 1631, 1595, 1522, 1337, 1259, 1124, 1079, 838, 786; HRESIMS Calcd for $[C_{37}H_{37}NNaOSi]^+$ (M + Na^+) 562.2537, found 562.2531.

2'-((2-((4-chlorobenzyl)amino)naphthalen-1-yl)ethynyl)-[1,1'-biphenyl]-4-ol (1b)

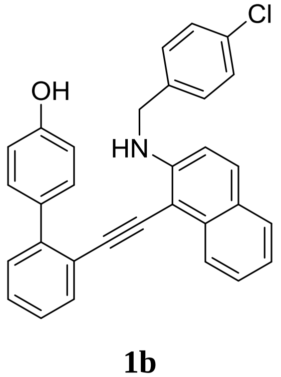

1b

The product was isolated through silica gel column chromatography (PE:EA = 7:1) as a pale yellow oil. ^{1}H NMR (400 MHz, $CDCl_3$) δ 7.99 (d, *J* = 8.4 Hz, 1H), 7.74 – 7.72 (m, 1H), 7.59 (d, *J* = 8.0 Hz, 1H), 7.53 – 7.47 (m, 3H), 7.45 – 7.40 (m, 1H), 7.39 – 7.28 (m, 3H), 7.26 – 7.19 (m, 3H),

7.16 – 7.13 (m, 2H), 6.78 (d, *J* = 8.0 Hz, 2H), 6.70 (d, *J* = 9.2 Hz, 1H), 5.25 (s, 1H), 5.09 (s, 1H), 4.33 (s, 2H); ^{13}C NMR (100 MHz, $CDCl_3$) δ 155.1, 147.6, 142.8, 138.0, 134.2, 133.9, 132.6, 132.3, 130.5, 130.1, 129.5, 128.7, 128.1, 128.0, 127.9, 127.2, 127.0, 126.6, 124.3, 122.5, 115.1, 112.5, 100.8, 99.7, 87.7, 46.4; IR (neat): 3396(br), 2926, 2188, 1618, 1582, 1345, 1180, 1078, 907, 810, 702; HRESIMS Calcd for $[C_{31}H_{22}ClNNaO]^+$ (M + Na^+) 482.1282, found 482.1288.

2'-((2-((4-methylbenzyl)amino)naphthalen-1-yl)ethynyl)-[1,1'-biphenyl]-4-ol (1c)

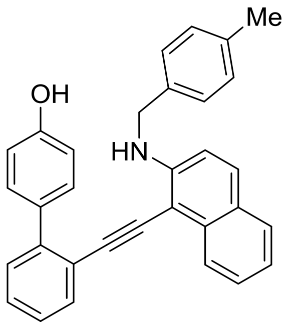

1c

The product was isolated through silica gel column chromatography (PE:EA = 5:1) as a pale yellow oil. ^{1}H NMR (500 MHz, $CDCl_3$) δ 7.97 (d, *J* = 8.5 Hz, 1H), 7.73 – 7.70 (m, 1H), 7.58 (d, *J* = 8.0 Hz, 1H), 7.52 – 7.47 (m, 3H), 7.43 – 7.38 (m, 1H), 7.33 – 7.30 (m, 3H), 7.19 – 7.13 (m, 3H), 7.11 – 7.08 (m, 2H), 6.79 – 6.73 (m, 3H), 5.15 (s, 1H), 4.97 (s, 1H), 4.34 (s, 2H), 2.30 (s, 3H); ^{13}C NMR (125 MHz, $CDCl_3$) δ 155.1, 148.0, 142.8, 136.6, 136.4, 134.3, 133.8, 132.3, 130.4, 130.1, 129.4, 129.3, 128.1, 127.9, 127.1, 126.9, 126.5, 124.2, 122.6, 122.2, 115.2, 112.7, 100.7, 99.3, 87.9, 46.8, 21.0; IR (neat): 3398(br), 3061, 2952, 2190, 1615, 1595, 1515, 1428, 1260, 1172, 1073, 810, 750; HRESIMS Calcd for $[C_{32}H_{25}NNaO]^+$ (M + Na^+) 462.1828, found 462.1832.

2'-((2-((4-methoxybenzyl)amino)naphthalen-1-yl)ethynyl)-[1,1'-biphenyl]-4-ol (1d)

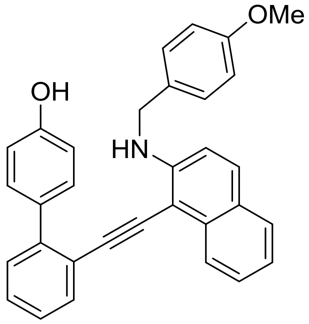

1d

The product was isolated through silica gel column chromatography (PE:EA = 5:1) as a pale yellow oil. ^{1}H NMR (500 MHz, $CDCl_3$) δ 7.96 (d, *J* = 8.5 Hz, 1H), 7.72 – 7.69 (m, 1H), 7.57 (d, *J* = 8.0 Hz, 1H), 7.51 (d, *J* = 9.0 Hz, 1H), 7.49 – 7.46 (m, 2H), 7.42 – 7.38 (m, 1H), 7.33 – 7.29 (m, 3H), 7.17 (d, *J* = 6.0 Hz, 1H), 7.14 (d, *J* = 8.0 Hz, 2H) 6.84 – 6.80 (m, 2H), 6.78 (d, *J* = 9.0 Hz, 1H), 6.76 – 6.73 (m, 2H), 5.19 (s, 1H), 5.10 (s, 1H), 4.31 (s, 2H), 3.72 (s, 3H); ^{13}C NMR (125 MHz, $CDCl_3$) δ 158.5, 155.1, 148.0, 142.7, 134.3, 133.8, 132.3, 131.4, 130.4, 130.1, 129.4, 128.1, 127.9, 127.8, 127.1, 126.9, 126.5, 124.2, 122.5, 122.3, 115.1, 114.0, 112.8, 100.7, 99.4, 87.9, 55.3, 46.5; IR (neat): 3386(br), 3041, 2923, 1614, 1595, 1510, 1275, 1260, 1173, 1037, 811, 749; HRESIMS Calcd for $[C_{32}H_{25}NNaO_2]^+$ (M + Na^+) 478.1778, found 478.1771.

2'-((2-((3-chlorobenzyl)amino)naphthalen-1-yl)ethynyl)-[1,1'-biphenyl]-4-ol (1e)

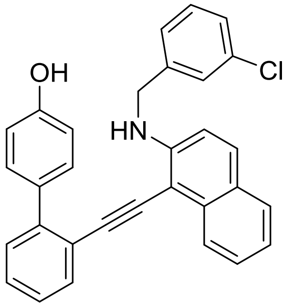

1e

The product was isolated through silica gel column chromatography (PE:EA = 7:1) as a pale yellow oil. ^{1}H NMR (500 MHz, $CDCl_3$) δ 7.99 (d, *J* = 8.0 Hz, 1H), 7.76 – 7.73 (m, 1H), 7.59 (d, *J* = 8.0 Hz, 1H), 7.53 (d, *J* = 9.0 Hz, 1H), 7.50 – 7.48 (m, 2H), 7.44 – 7.40 (m, 1H), 7.38 – 7.33 (m, 4H), 7.23 – 7.10 (m, 4H), 6.79 – 6.76 (m, 2H), 6.72 – 6.69 (m, 1H), 5.14 (s, 1H), 5.07 (s, 1H), 4.46 (s, 2H); ^{13}C NMR (125 MHz, $CDCl_3$) δ 155.2, 147.6, 142.9, 136.6, 133.9, 132.8, 132.4, 132.3, 130.5, 130.2, 129.5, 129.4, 128.2, 128.1, 128.0, 127.2, 127.0, 126.9, 126.7, 124.3, 122.6, 122.4, 117.2, 115.2, 112.5, 100.8, 99.6, 87.7, 44.9; IR (neat): 3385(br), 2932, 2191, 1615, 1560, 1515, 1128, 1072, 907, 811; HRESIMS Calcd for $[C_{31}H_{22}ClNNaO]^+$ (M + Na^+) 482.1282, found 482.1278.

2'-((2-((3-bromobenzyl)amino)naphthalen-1-yl)ethynyl)-[1,1'-biphenyl]-4-ol (1f)

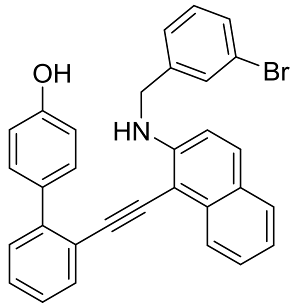

1f

The product was isolated through silica gel column chromatography (PE:EA = 7:1) as a yellow oil. ^{1}H NMR (400 MHz, $CDCl_3$) δ 8.00 (d, *J* = 8.4 Hz, 1H), 7.76 – 7.72 (m, 1H), 7.60 (d, *J* = 8.0 Hz, 1H), 7.54 – 7.48 (m, 3H), 7.45 – 7.39 (m, 2H), 7.38 – 7.31 (m, 4H), 7.22 – 7.17 (m, 1H), 7.16 – 7.11 (m, 2H), 6.80 (d, *J* = 8.4 Hz, 2H), 6.70 (d, *J* = 8.8 Hz, 1H), 5.10 (t, *J* = 6.0 Hz, 1H), 4.34 (d, *J* = 6.0 Hz, 2H), 4.16 (s, 1H); ^{13}C NMR (125 MHz, $CDCl_3$) δ 155.2, 147.5, 142.8, 142.0, 134.2, 133.7, 132.3, 130.5, 130.2, 130.1(3), 130.0(7), 129.5, 129.4, 128.1, 128.0, 127.2, 127.0, 126.6, 125.0, 124.2, 122.7, 122.5, 122.4, 115.1, 112.5, 100.9, 99.7, 87.7, 46.5; IR (neat): 3398(br), 3054, 2925, 2190, 1615, 1595, 1515, 1478, 1427, 1275, 1261, 836, 750; HRESIMS Calcd for $[C_{31}H_{22}BrNNaO]^+$ (M + Na^+) 526.0777, found 526.0782.

methyl 3-(((1-((4'-hydroxy-[1,1'-biphenyl]-2-yl)ethynyl)naphthalen-2-yl)amino)methyl)benzoate (1g)

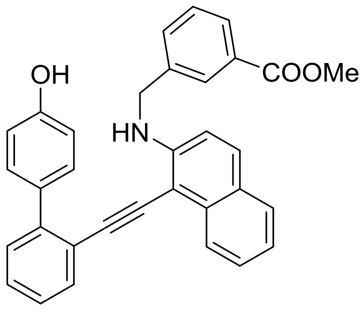

1g

The product was isolated through silica gel column chromatography (PE:EA = 5:1) as a pale yellow oil. ^{1}H NMR (400 MHz, $CDCl_3$) δ 8.07 (d, *J* = 8.4 Hz, 1H), 8.00 (s, 1H), 7.89 (d, *J* = 8.0 Hz, 1H), 7.77 – 7.74 (m, 1H), 7.61 (d, *J* = 8.0 Hz, 1H), 7.55 – 7.50 (m, 3H), 7.49 – 7.43 (m, 2H), 7.40 – 7.32 (m, 4H), 7.23 – 7.18 (m, 1H), 6.90 (d, *J* = 8.8 Hz, 2H), 6.75 (d, *J* = 8.8 Hz, 1H), 6.16 (s, 1H), 5.12 (s, 1H), 4.36 (s, 2H), 3.93 (s, 3H); ^{13}C NMR (125 MHz, $CDCl_3$) δ 168.1, 155.7, 147.7,

143.0, 140.5, 134.3, 133.5, 132.4, 131.6, 130.6, 130.4, 130.1, 129.4, 128.7, 128.3(1), 128.2(9), 128.1(0), 128.0(8), 127.2, 126.9, 126.7, 124.3, 122.4, 115.3, 112.5, 100.8, 99.8, 87.6, 52.5, 47.3; IR (neat): 3566(br), 2928, 2091, 1716, 1688, 1594, 1540, 1507, 1456, 1275, 1260, 814, 749; HRESIMS Calcd for $[C_{33}H_{25}NNaO_3]^+$ (M + Na$^+$) 506.1727, found 506.1735.

2'-((2-((3-(trifluoromethyl)benzyl)amino)naphthalen-1-yl)ethynyl)-[1,1'-biphenyl]-4-ol (1h)

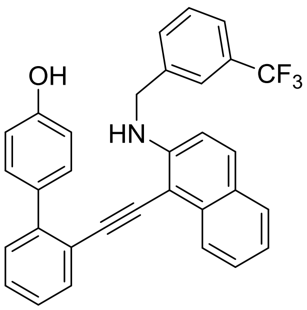

1h

The product was isolated through silica gel column chromatography (PE:EA = 5:1) as a pale yellow oil. ^{1}H NMR (400 MHz, CDCl$_3$) δ 8.00 (d, *J* = 8.0 Hz, 1H), 7.76 – 7.70 (m, 1H), 7.61 (d, *J* = 7.6 Hz, 1H), 7.56 – 7.49 (m, 5H), 7.45 – 7.34 (m, 6H), 7.24 – 7.19 (m, 1H), 6.80 (d, *J* = 8.0 Hz, 2H), 6.72 (d, *J* = 9.2 Hz, 1H), 5.12 (s, 1H), 4.98 (s, 1H), 4.43 (s, 2H); ^{13}C NMR (125 MHz, CDCl$_3$) δ 155.2, 147.5, 142.9, 140.7, 134.2, 133.9, 132.3, 130.8 (d, *J* = 31.3 Hz), 130.5, 130.2, 129.8, 129.5, 129.1, 128.1 (d, *J* = 7.5 Hz), 127.3, 127.0, 126.7, 125.9 (d, *J* = 175.0 Hz), 124.3, 123.9 (q, *J* = 3.8 Hz), 123.3 (q, *J* = 3.8 Hz), 122.5(4), 122.4(6), 115.1, 112.4, 100.9, 99.9, 87.6, 46.8; IR (neat): 3381(br), 2932, 2091, 1618, 1595, 1341, 1128, 1076, 908, 812, 750; HRESIMS Calcd for $[C_{32}H_{22}F_3NNaO]^+$ (M + Na$^+$) 516.1546, found 516.1541.

2'-((2-((3-methylbenzyl)amino)naphthalen-1-yl)ethynyl)-[1,1'-biphenyl]-4-ol (1i)

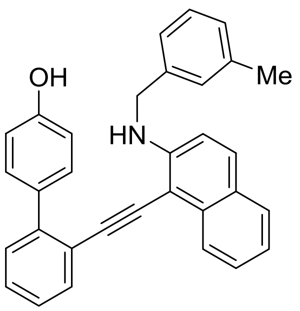

1i

The product was isolated through silica gel column chromatography (PE:EA = 5:1) as a pale yellow oil. ^{1}H NMR (500 MHz, $CDCl_3$) δ 7.97 (d, *J* = 8.5 Hz, 1H), 7.74 – 7.71 (m, 1H), 7.60 (d, *J* = 8.0 Hz, 1H), 7.54 (d, *J* = 9.0 Hz, 1H), 7.52 – 7.49 (m, 2H), 7.44 – 7.39 (m, 1H), 7.36 – 7.31 (m, 3H), 7.22 – 7.16 (m, 2H), 7.09 – 7.04 (m, 3H), 6.81 – 6.75 (m, 3H), 5.14 (s, 1H), 4.93 (s, 1H), 4.37 (s, 2H), 2.31 (s, 3H); ^{13}C NMR (100 MHz, $CDCl_3$) δ 155.2, 148.1, 142.8, 139.5, 138.3, 134.3, 133.8, 132.3, 130.4, 130.1, 129.4, 128.5, 128.1, 127.9, 127.7, 127.2, 127.1, 126.9, 126.5, 124.2, 123.6, 122.5, 122.2, 115.2, 112.7, 100.7, 99.3, 87.9, 47.0, 21.4; IR (neat): 3366(br), 2926, 2291, 1618, 1695, 1515, 1423, 1275, 1260, 1076, 907, 835, 749; HRESIMS Calcd for $[C_{32}H_{25}NNaO]^+$ (M + Na^+) 462.1828, found 462.1831.

2'-((2-((3-methoxybenzyl)amino)naphthalen-1-yl)ethynyl)-[1,1'-biphenyl]-4-ol (1j)

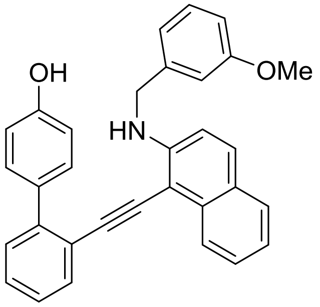

1j

The product was isolated through silica gel column chromatography (PE:EA = 5:1) as a pale yellow oil. ^{1}H NMR (400 MHz, $CDCl_3$) δ 8.00 (d, *J* = 8.4 Hz, 1H), 7.74 – 7.71 (m, 1H), 7.58 (d, *J* = 8.0 Hz, 1H), 7.53 – 7.47 (m, 3H), 7.42 (t, *J* = 7.6 Hz, 1H), 7.38 – 7.29 (m, 3H), 7.23 – 7.15 (m, 2H), 6.87 – 6.82 (m, 2H), 6.79 – 6.70 (m, 4H), 5.34 (s, 1H), 5.12 (s, 1H), 4.34 (s, 2H), 3.72 (s, 3H); ^{13}C NMR (100 MHz, $CDCl_3$) δ 159.7, 155.2, 147.9, 142.8, 141.4, 134.3, 133.8, 132.3, 130.5, 130.1, 129.6, 129.4, 128.1, 128.0, 127.1, 126.9, 126.6, 124.2, 122.5, 122.3, 119.0, 115.2, 112.7, 112.4, 112.2, 100.7, 99.4, 87.8, 55.2, 47.0; IR (neat): 3396(br), 3058, 2190, 1595, 1507, 1456, 1275, 1260, 1047, 814, 749; HRESIMS Calcd for $[C_{32}H_{25}NNaO_2]^+$ (M + Na^+) 478.1778, found 478.1784.

2'-((2-((2-fluorobenzyl)amino)naphthalen-1-yl)ethynyl)-[1,1'-biphenyl]-4-ol (1k)

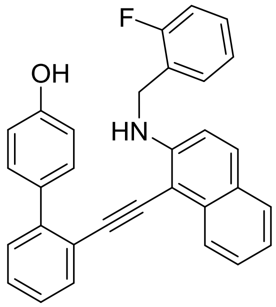

1k

The product was isolated through silica gel column chromatography (PE:EA = 7:1) as a pale yellow oil. ^{1}H NMR (500 MHz, $CDCl_3$) δ 7.97 (d, *J* = 8.5 Hz, 1H), 7.75 – 7.72 (m, 1H), 7.60 (d, *J* = 8.0 Hz, 1H), 7.55 (d, *J* = 8.5 Hz, 1H), 7.52 – 7.48 (m, 2H), 7.44 – 7.39 (m, 1H), 7.35 – 7.33 (m, 3H), 7.21 – 7.15 (m, 3H), 7.07 – 6.99 (m, 2H), 6.81 – 6.75 (m, 3H), 5.07 (s, 1H), 5.00 (s, 1H), 4.46 (s, 2H); ^{13}C NMR (125 MHz, $CDCl_3$) δ 160.6 (d, *J* = 243.8 Hz), 155.1, 147.6, 142.9, 134.2, 133.8, 132.3, 130.5, 130.1, 129.4, 128.5 (d, *J* = 7.5 Hz), 128.3 (d, *J* = 5.0 Hz), 128.0 (d, *J* = 12.5 Hz), 127.2, 126.9, 126.7, 126.3 (d, *J* = 15.0 Hz), 124.3, 124.2 (d, *J* = 3.8 Hz), 122.5, 122.4, 115.2, 115.1, 115.0, 112.5, 100.8, 99.8, 87.7, 40.8 (d, *J* = 5.0 Hz); IR (neat): 3399(br), 3058, 2925, 2190, 1614, 1596, 1515, 1487, 1457, 1428, 1275, 1260, 836, 750; HRESIMS Calcd for $[C_{31}H_{22}FNNaO]^+$ (M + Na^+) 466.1578, found 466.1581.

2'-((2-((2-bromobenzyl)amino)naphthalen-1-yl)ethynyl)-[1,1'-biphenyl]-4-ol (1l)

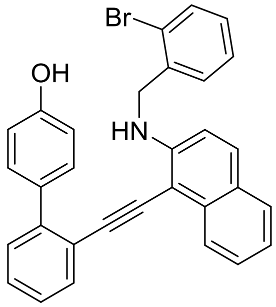

1l

The product was isolated through silica gel column chromatography (PE:EA = 7:1) as a pale yellow oil. ^{1}H NMR (500 MHz, $CDCl_3$) δ 7.99 (d, *J* = 8.5 Hz, 1H), 7.76 – 7.73 (m, 1H), 7.59 (d, *J* = 8.0 Hz, 1H), 7.56 – 7.51 (m, 2H), 7.48 (d, *J* = 8.5 Hz, 2H), 7.42 (t, *J* = 8.0 Hz, 1H), 7.36 – 7.31 (m, 3H), 7.21 – 7.15 (m, 3H), 7.11 – 7.06 (m, 1H), 6.77 (d, *J* = 8.5 Hz, 2H), 6.68 (d, *J* = 9.0 Hz, 1H), 5.16 (s, 1H), 4.41 (d, *J* = 6.0 Hz, 2H); ^{13}C NMR (125 MHz, $CDCl_3$) δ 155.2, 147.6, 142.9,

138.0, 134.2, 133.8, 132.6, 132.3, 130.5, 130.2, 129.5, 128.5, 128.2, 128.1, 128.0, 127.5, 127.2, 127.0, 126.6, 124.3, 122.7, 122.5, 122.4, 115.2, 112.4, 100.9, 99.6, 87.7, 47.4; IR (neat): 3388(br), 2926, 1615, 1585, 1518, 1328, 1168, 1125, 1078, 915, 812, 750; HRESIMS Calcd for $[C_{31}H_{22}BrNNaO]^+$ (M + Na^+) 526.0777, found 526.0781.

2'-((2-((2-methylbenzyl)amino)naphthalen-1-yl)ethynyl)-[1,1'-biphenyl]-4-ol (1m)

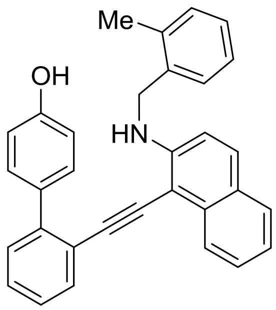

1m

The product was isolated through silica gel column chromatography (PE:EA = 5:1) as a pale yellow oil. ^{1}H NMR (500 MHz, $CDCl_3$) δ 7.98 (d, *J* = 8.5 Hz, 1H), 7.70 – 7.67 (m, 1H), 7.58 (d, *J* = 8.0 Hz, 1H), 7.51 (d, *J* = 9.0 Hz, 1H), 7.48 – 7.44 (m, 2H), 7.43 – 7.39 (m, 1H), 7.32 – 7.29 (m, 3H), 7.20 – 7.15 (m, 4H), 7.13 – 7.08 (m, 1H), 6.71 (d, *J* = 9.0 Hz, 1H), 6.70 – 6.67 (m, 2H), 5.00 (s, 1H), 4.88 (s, 1H), 4.28 (s, 2H), 2.31 (s, 3H); ^{13}C NMR (125 MHz, $CDCl_3$) δ 155.0, 148.1, 142.8, 137.0, 135.6, 134.3, 133.8, 132.2, 130.4, 130.2, 129.4, 128.1, 127.9, 127.1, 126.9, 126.5, 126.1, 124.2, 122.6, 122.2, 115.2, 112.5, 100.7, 99.2, 87.9, 45.1, 18.9; IR (neat): 3396(br), 2926, 2190, 1615, 1595, 1518, 1275, 1260, 1074, 835, 749; HRESIMS Calcd for $[C_{32}H_{25}NNaO]^+$ (M + Na^+) 462.1828, found 462.1829.

2'-((2-(benzylamino)naphthalen-1-yl)ethynyl)-5'-fluoro-[1,1'-biphenyl]-4-ol (1n)

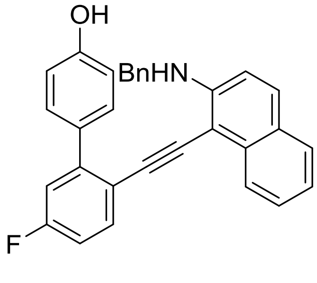

1n

The product was isolated through silica gel column chromatography (PE:EA = 7:1) as a pale yellow oil. ^{1}H NMR (500 MHz, $CDCl_3$) δ 7.94 (d, *J* = 8.5 Hz, 1H), 7.69 – 7.65 (m, 1H), 7.59 (d, *J* = 8.0 Hz, 1H), 7.52 (d, *J* = 9.0 Hz, 1H), 7.47 (d, *J* = 8.5 Hz, 2H), 7.41 (t, *J* = 7.0 Hz, 1H), 7.32 – 7.28 (m, 2H), 7.26 – 7.22 (m, 3H), 7.18 (t, *J* = 7.0 Hz, 1H), 7.07 – 6.99 (m, 2H), 6.79 – 6.74 (m, 3H), 4.99 (s, 1H), 4.39 (s, 2H); ^{13}C NMR (125 MHz, $CDCl_3$) δ 162.1 (d, *J* = 248.8 Hz), 155.5, 147.9, 145.0 (d, *J* = 8.8 Hz), 139.4, 134.1 (d, *J* = 31.3 Hz), 134.0, 132.7, 130.3, 130.1, 128.6, 128.1, 127.2, 127.1, 126.6, 124.2, 122.3, 118.7, 116.4 (d, *J* = 21.3 Hz), 115.3, 114.2 (d, *J* = 21.3 Hz), 112.7, 99.7, 99.3, 87.4, 47.1; IR (neat): 3396(br), 2926, 2188, 1618, 1605, 1327, 1186, 1104, 1078, 912, 810; HRESIMS Calcd for $[C_{31}H_{22}FNNaO]^+$ (M + Na^+) 466.1578, found 466.1581.

2'-((2-(benzylamino)naphthalen-1-yl)ethynyl)-5'-chloro-[1,1'-biphenyl]-4-ol (1o)

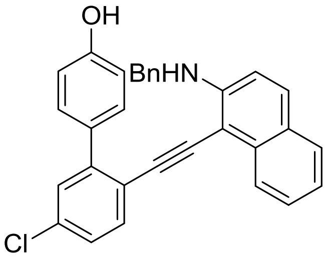

1o

The product was isolated through silica gel column chromatography (PE:EA = 7:1) as a pale yellow oil. ^{1}H NMR (500 MHz, $CDCl_3$) δ 7.93 (d, *J* = 8.5 Hz, 1H), 7.61 (d, *J* = 8.5 Hz, 1H), 7.58 (d, *J* = 8.0 Hz, 1H), 7.52 (d, *J* = 9.0 Hz, 1H), 7.46 (d, *J* = 8.5 Hz, 2H), 7.41 – 7.39 (m, 1H), 7.33 – 7.27 (m, 4H), 7.25 – 7.21 (m, 3H), 7.18 (t, *J* = 7.0 Hz, 1H), 6.78 – 6.73 (m, 3H), 5.05 (s, 1H), 4.38 (s, 2H); ^{13}C NMR (125 MHz, $CDCl_3$) δ 155.5, 148.1, 144.2, 139.4, 134.3, 133.6, 133.3, 132.6, 130.4, 129.5, 128.6, 128.2, 127.2, 127.1, 127.0(6), 126.6, 124.1, 122.4, 121.2, 115.3, 112.7, 99.8, 99.0, 88.8, 47.0; IR (neat): 3388(br), 3056, 2926, 2220, 1615, 1595, 1515, 1339, 1275, 1260, 814, 749; HRESIMS Calcd for $[C_{31}H_{22}ClNNaO]^+$ (M + Na^+) 482.1282, found 482.1279.

2'-((2-(benzylamino)naphthalen-1-yl)ethynyl)-5'-methoxy-[1,1'-biphenyl]-4-ol (1p)

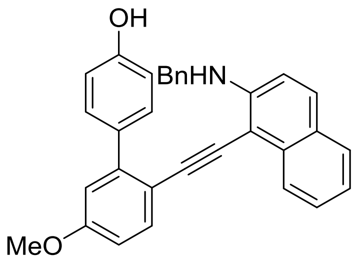

1p

The product was isolated through silica gel column chromatography (PE:EA = 5:1) as a pale yellow oil. ^{1}H NMR (500 MHz, $CDCl_3$) δ 7.97 (d, *J* = 8.5 Hz, 1H), 7.66 – 7.63 (m, 1H), 7.59 (d, *J* = 8.0 Hz, 1H), 7.53 – 7.49 (m, 3H), 7.43 – 7.38 (m, 1H), 7.32 – 7.29 (m, 2H), 7.27 – 7.21 (m, 3H), 7.20 – 7.16 (m, 1H), 6.91 – 6.88 (m, 2H), 6.80 – 6.75 (m, 3H), 5.12 (s, 1H), 4.99 (s, 1H), 4.40 (s, 2H), 3.84 (s, 3H); ^{13}C NMR (125 MHz, $CDCl_3$) δ 159.3, 155.2, 147.5, 144.5, 139.6, 134.2, 133.8, 133.7, 130.4, 129.6, 128.6, 128.1, 127.0, 126.6, 124.3, 122.2, 115.2, 115.0, 114.8, 113.0, 112.7, 100.6, 99.9, 86.2, 55.4, 47.1; IR (neat): 3386(br), 3005, 2191, 1594, 1516, 1339, 1275, 1260, 1061, 814, 749; HRESIMS Calcd for $[C_{32}H_{25}NNaO_2]^+$ (M + Na^+) 478.1778, found 478.1783.

2'-((2-(benzylamino)naphthalen-1-yl)ethynyl)-4'-fluoro-[1,1'-biphenyl]-4-ol (1q)

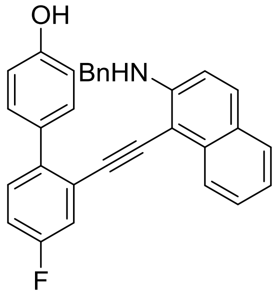

1q

The product was isolated through silica gel column chromatography (PE:EA = 7:1) as a pale yellow oil. ^{1}H NMR (400 MHz, $CDCl_3$) δ 7.93 (d, *J* = 8.4 Hz, 1H), 7.60 (d, *J* = 8.0 Hz, 1H), 7.54 (d, *J* = 9.2 Hz, 1H), 7.47 – 7.38 (m, 4H), 7.34 – 7.30 (m, 2H), 7.28 – 7.23 (m, 4H), 7.22 – 7.17 (m, 1H), 7.08 – 7.02 (m, 1H), 6.79 – 6.74 (m, 3H), 5.08 (t, *J* = 5.6 Hz, 1H), 4.40 (d, *J* = 5.6 Hz, 2H); ^{13}C NMR (100 MHz, $CDCl_3$) δ 161.5 (d, *J* = 245.0 Hz), 155.2, 148.3, 139.4, 139.0, 134.3, 133.0, 131.0 (d, *J* = 8.0 Hz), 130.6, 130.5, 128.6, 128.1, 127.3, 127.1, 126.5, 124.1, 122.4, 118.5, 118.3 (d, *J* = 22.0 Hz), 115.2, 112.6, 99.7, 98.7, 88.8, 46.9; IR (neat): 3396(br), 3006, 2191, 1646, 1540,

1456, 1275, 1260, 1073, 812, 749; HRESIMS Calcd for $[C_{31}H_{22}FNNaO]^+$ (M + Na^+) 466.1578, found 466.1583.

2'-((2-(benzylamino)naphthalen-1-yl)ethynyl)-4'-methyl-[1,1'-biphenyl]-4-ol (1r)

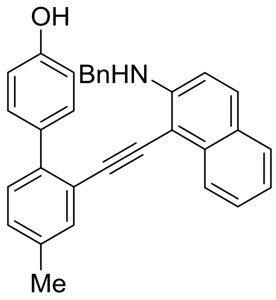

1r

The product was isolated through silica gel column chromatography (PE:EA = 5:1) as a pale yellow oil. ^{1}H NMR (500 MHz, $CDCl_3$) δ 7.99 (d, *J* = 8.0 Hz, 1H), 7.61 (d, *J* = 8.0 Hz, 1H), 7.57 (d, *J* = 8.0 Hz, 1H), 7.50 – 7.45 (m, 3H), 7.42 – 7.38 (m, 1H), 7.29 – 7.26 (m, 2H), 7.24 – 7.20 (m, 3H), 7.18 – 7.16 (m, 1H), 7.15 – 7.11 (m, 2H), 6.74 (d, *J* = 9.0 Hz, 1H), 6.73 – 6.70 (m, 2H), 5.11 (s, 1H), 5.08 (s, 1H), 4.35 (s, 2H), 2.37 (s, 3H); ^{13}C NMR (125 MHz, $CDCl_3$) δ 155.1, 147.8, 142.7, 139.5, 138.1, 134.2, 133.9, 132.2, 130.4, 130.2, 129.8, 128.6, 128.1, 127.8, 127.1, 126.9, 126.6, 124.3, 122.2, 119.5, 115.1, 112.7, 100.9, 99.7, 87.0, 47.0, 21.4; IR (neat): 3388(br), 3006, 2220, 1595, 1515, 1429, 1275, 1260, 1074, 813, 749; HRESIMS Calcd for $[C_{32}H_{25}NNaO]^+$ (M + Na^+) 462.1828, found 462.1821.

4-(4-(2-(benzylamino)naphthalen-1-yl)but-3-yn-1-yl)phenol (1s)

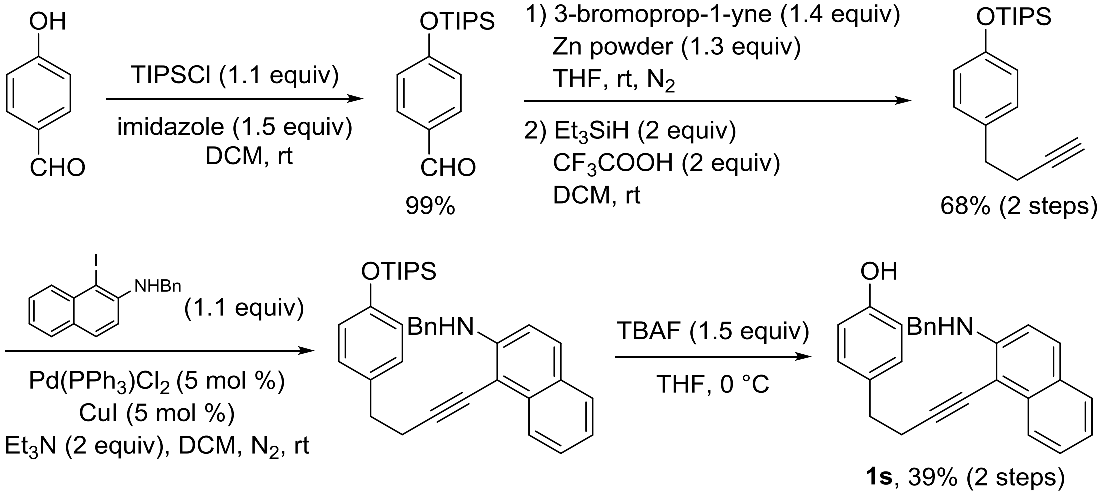

Compound **1s** was prepared according to the above procedures (*31*). The product was isolated through silica gel column chromatography (PE:EA = 5:1) as a pale yellow oil. ^{1}H NMR (500 MHz, $CDCl_3$) δ 7.97 (d, *J* = 8.5 Hz, 1H), 7.57 (d, *J* = 8.0 Hz, 1H), 7.50 (d, *J* = 9.0 Hz, 1H), 7.40 – 7.36 (m, 1H), 7.30 – 7.26 (m, 4H), 7.21 – 7.18 (m, 1H), 7.17 – 7.12 (m, 1H), 7.09 (d, *J* = 8.5 Hz, 2H), 6.81 (d, *J* = 9.0 Hz, 1H), 6.64 (d, *J* = 8.5 Hz, 2H), 5.20 (s, 2H), 4.41 (s, 2H), 2.86 (s, 4H); ^{13}C NMR (125 MHz, $CDCl_3$) δ 153.9, 147.7, 139.5, 134.5, 132.6, 129.7, 129.2, 128.5, 128.0, 127.0, 126.9, 126.7, 126.6, 124.3, 122.1, 115.2, 112.7, 100.6, 100.1, 76.4, 47.3, 34.2, 22.1; IR (neat): 3396(br), 2926, 2223, 1618, 1595, 1575, 1275, 1168, 1078, 912, 749; HRESIMS Calcd for $[C_{27}H_{23}NNaO]^+$ (M + Na^+) 400.1672, found 400.1678.

2'-((2-(benzylamino)-6-bromonaphthalen-1-yl)ethynyl)-[1,1'-biphenyl]-4-ol (1t)

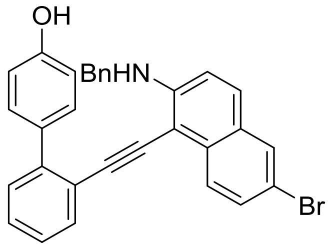

1t

The product was isolated through silica gel column chromatography (PE:EA = 7:1) as a pale yellow oil. ^{1}H NMR (400 MHz, $CDCl_3$) δ 7.80 (d, *J* = 8.8 Hz, 1H), 7.73 – 7.69 (m, 2H), 7.50 – 7.47 (m, 4H), 7.36 – 7.30 (m, 5H), 7.28 – 7.24 (m, 3H), 6.80 – 6.77 (m, 3H), 5.16 (s, 1H), 4.40 (d, *J* = 5.2 Hz, 2H); ^{13}C NMR (125 MHz, $CDCl_3$) δ 155.3, 148.1, 143.0, 139.2, 133.7, 132.8, 132.3, 130.4, 130.1, 129.9, 129.5, 129.0, 128.7, 128.2, 127.6, 127.1, 127.0, 126.5, 126.1, 122.3, 115.5, 115.2, 113.6, 101.1, 99.5, 87.2, 47.0; IR (neat): 3396(br), 3005, 2191, 1615, 1595, 1506, 1397, 1275, 1260, 1073, 815, 749; HRESIMS Calcd for $[C_{31}H_{22}BrNNaO]^+$ (M + Na^+) 526.0777, found 526.0771.

2'-((2-(benzylamino)-6-methylnaphthalen-1-yl)ethynyl)-[1,1'-biphenyl]-4-ol (1u)

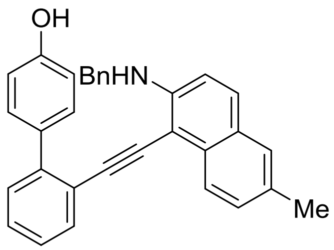

1u

The product was isolated through silica gel column chromatography (PE:EA = 5:1) as a pale yellow oil. ^{1}H NMR (400 MHz, $CDCl_3$) δ 7.89 (d, *J* = 8.4 Hz, 1H), 7.70 – 7.68 (m, 1H), 7.46 (d, *J* = 8.0 Hz, 2H), 7.41 (d, *J* = 8.8 Hz, 1H), 7.33 (s, 1H), 7.30 – 7.29 (m, 3H), 7.27 – 7.18 (m, 6H), 6.77 – 6.69 (m, 3H), 5.22 (s, 1H), 5.05 (s, 1H), 4.33 (s, 2H), 2.38 (s, 3H); ^{13}C NMR (100 MHz, $CDCl_3$) δ 155.0, 147.4, 142.7, 139.6, 133.8, 132.4, 132.3, 131.6, 130.4, 129.5, 129.4, 129.3, 128.5, 127.9, 127.1, 126.9, 126.8, 126.6, 124.1, 122.5, 115.1, 112.8, 100.6, 99.4, 88.0, 47.1, 21.2; IR (neat): 3389(br), 3005, 2926, 2189, 1596, 1516, 1373, 1275, 1260, 1073, 818, 749; HRESIMS Calcd for $[C_{32}H_{25}NNaO]^+$ (M + Na^+) 462.1828, found 462.1836.

2'-((2-(benzylamino)naphthalen-1-yl)ethynyl)-2,3-dimethyl-[1,1'-biphenyl]-4-ol (1v)

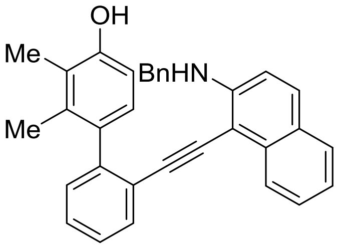

1v

The product was isolated through silica gel column chromatography (PE:EA = 5:1) as a pale yellow oil. ^{1}H NMR (500 MHz, $CDCl_3$) δ 7.82 (d, *J* = 8.0 Hz, 1H), 7.73 – 7.70 (m, 1H), 7.56 (d, *J* = 8.0 Hz, 1H), 7.48 (d, *J* = 9.0 Hz, 1H), 7.41 – 7.37 (m, 1H), 7.36 – 7.32 (m, 2H), 7.30 – 7.26 (m, 2H), 7.23 – 7.14 (m, 5H), 7.03 (d, *J* = 8.0 Hz, 1H), 6.72 (d, *J* = 9.0 Hz, 1H), 6.57 (d, *J* = 8.0 Hz, 1H), 4.84 (s, 1H), 4.67 (s, 1H), 4.41 – 4.31 (m, 1H), 2.09 (s, 3H), 2.08 (s, 3H); ^{13}C NMR (125 MHz, $CDCl_3$) δ 152.9, 147.9, 144.0, 139.6, 136.5, 134.3, 134.2, 131.3, 130.0, 128.5, 128.0, 127.5, 127.4, 127.1, 126.9, 126.8, 126.5, 126.3, 124.1, 122.9, 122.2, 112.6, 112.4, 100.5, 99.2, 87.7, 46.7, 17.1, 11.9; IR (neat): 3388(br), 3048, 2220, 1629, 1588, 1575, 1275, 1166, 1124, 1072, 810, 749; HRESIMS Calcd for $[C_{33}H_{27}NNaO]^+$ (M + Na^+) 476.1985, found 476.1991.

4-(2-((2-(benzylamino)naphthalen-1-yl)ethynyl)phenyl)-5,6,7,8-tetrahydronaphthalen-1-ol (1w)

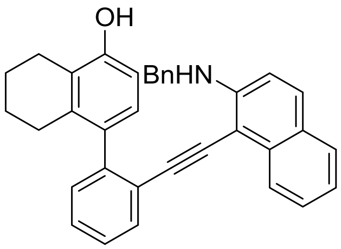

1w

The product was isolated through silica gel column chromatography (PE:EA = 5:1) as a pale yellow oil. ^{1}H NMR (500 MHz, $CDCl_3$) δ 7.84 (d, *J* = 8.5 Hz, 1H), 7.73 – 7.70 (m, 1H), 7.56 (d, *J* = 8.0 Hz, 1H), 7.48 (d, *J* = 9.0 Hz, 1H), 7.42 – 7.37 (m, 1H), 7.36 – 7.32 (m, 2H), 7.30 – 7.26 (m, 2H), 7.23 – 7.19 (m, 4H), 7.18 – 7.16 (m, 1H), 7.01 (d, *J* = 8.0 Hz, 1H), 6.71 (d, *J* = 9.0 Hz, 1H), 6.61 (d, *J* = 8.0 Hz, 1H), 4.79 (s, 1H), 4.42 – 4.34 (m, 2H), 2.74 – 2.53 (m, 3H), 2.44 – 2.36 (m, 2H), 1.77 – 1.73 (m, 1H), 1.70 – 1.58 (m, 2H); ^{13}C NMR (125 MHz, $CDCl_3$) δ 152.8, 147.9, 143.4, 139.6, 136.9, 134.2, 134.0, 131.3, 130.0, 129.9, 128.5, 128.0, 127.6, 127.1, 127.0, 126.8, 126.4, 126.3, 124.1, 124.0, 123.6, 122.2, 113.4, 112.6, 111.8, 100.5, 99.1, 87.6, 46.7, 27.9, 23.1, 22.6, 22.2; IR (neat): 3376(br), 3040, 2191, 1620, 1595, 1329, 1270, 1124, 1078, 911, 812, 755; HRESIMS Calcd for $[C_{35}H_{29}NNaO]^+$ (M + Na^+) 502.2141, found 502.2145.

4-(2-((2-(benzylamino)naphthalen-1-yl)ethynyl)phenyl)naphthalen-1-ol (1x)

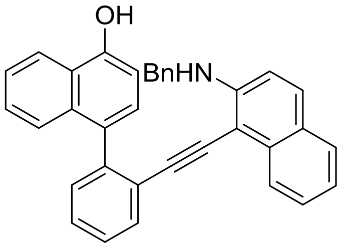

1x

The product was isolated through silica gel column chromatography (PE:EA = 5:1) as a pale yellow oil. ^{1}H NMR (500 MHz, $CDCl_3$) δ 8.17 – 8.13 (m, 1H), 7.82 – 7.80 (m, 1H), 7.67 – 7.62 (m, 2H), 7.51 (d, *J* = 8.0 Hz, 1H), 7.48 – 7.35 (m, 7H), 7.33 – 7.29 (m, 1H), 7.28 – 7.20 (m, 2H), 7.14 – 7.10 (m, 1H), 7.07 (d, *J* = 7.0 Hz, 2H), 6.73 (d, *J* = 8.0 Hz, 1H), 6.60 (d, *J* = 9.0 Hz, 1H),

5.18 (s, 1H), 4.42 (s, 1H), 4.20 – 4.06 (m, 2H); ^{13}C NMR (125 MHz, CDCl3) δ 151.1, 147.7, 141.9, 139.5, 134.1, 132.8, 132.0, 131.7, 130.9, 129.9, 128.4, 127.9, 127.6, 127.4, 127.0(2), 127.0(0), 126.7(9), 126.7(5), 126.3, 126.1, 125.3, 124.7, 124.2, 124.0, 122.1, 121.8, 112.5, 108.4, 100.4, 99.0, 88.0, 46.7; IR (neat): 3389(br), 3056, 2926, 2098, 1642, 1601, 1528, 1188, 1073, 905, 815, 750; HRESIMS Calcd for $[C_{35}H_{25}NNaO]^+$ (M + Na$^+$) 498.1828, found 498.1831.

Compounds **3a**-**3r** were prepared according to the following procedures.

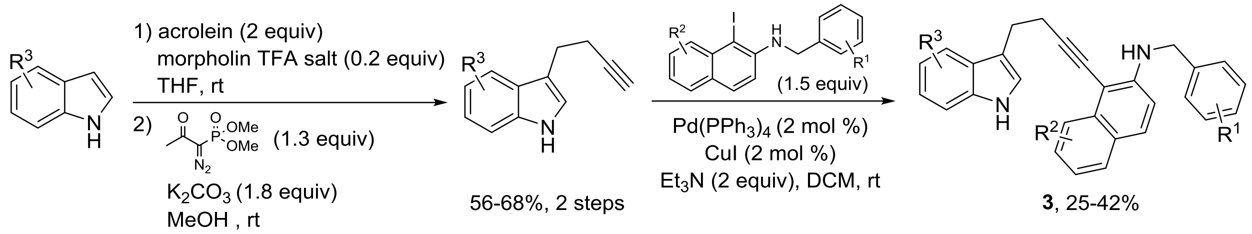

1-(4-(1*H*-indol-3-yl)but-1-yn-1-yl)-*N*-benzylnaphthalen-2-amine (3a)

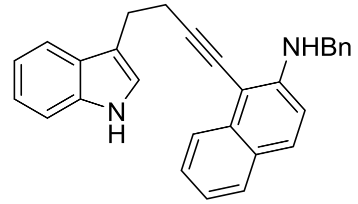

3a

The product was isolated through silica gel column chromatography (PE:EA = 7:1) as a pale yellow oil. ^{1}H NMR (500 MHz, CDCl$_3$) δ 8.02 (d, *J* = 8.5 Hz, 1H), 7.59 – 7.54 (m, 2H), 7.47 (d, *J* = 9.0 Hz, 1H), 7.38 – 7.32 (m, 2H), 7.24 – 7.20 (m, 2H), 7.19 – 7.17 (m, 2H), 7.15 – 7.09 (m, 3H), 7.08 – 7.04 (m, 1H), 7.01 (s, 1H), 6.82 (d, *J* = 2.0 Hz, 1H), 6.78 (d, *J* = 9.0 Hz, 1H), 5.11 (s, 1H), 4.23 (s, 2H), 3.05 (t, *J* = 7.0 Hz, 2H), 2.95 (t, *J* = 7.0 Hz, 2H); ^{13}C NMR (125 MHz, CDCl$_3$) δ 147.7, 139.5, 136.0, 134.4, 129.1, 128.5, 128.0, 127.1, 127.0, 126.9, 126.8, 126.5, 124.2, 122.0, 121.8, 119.2, 118.6, 114.6, 112.6, 111.1, 101.4, 100.1, 76.2, 47.3, 25.0, 21.0; IR (neat): 3349(br), 2359, 1384, 1275, 1124, 1096, 749, 656, 633, 600; HRESIMS Calcd for $[C_{29}H_{24}N_2Na]^+$ (M + Na$^+$) 423.1832, found 423.1835.

1-(4-(1*H*-indol-3-yl)but-1-yn-1-yl)-*N*-(4-methylbenzyl)naphthalen-2-amine (3b)

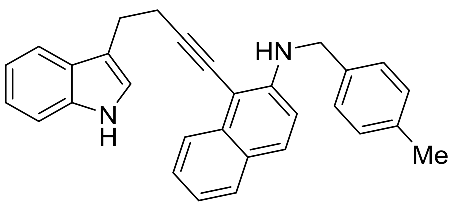

3b

The product was isolated through silica gel column chromatography (PE:EA = 7:1) as a pale yellow oil. ^{1}H NMR (400 MHz, $CDCl_3$) δ 8.00 (d, *J* = 8.4 Hz, 1H), 7.66 – 7.60 (m, 3H), 7.56 (d, *J* = 9.2 Hz, 1H), 7.41 – 7.36 (m, 2H), 7.30 – 7.26 (m, 1H), 7.22 – 7.09 (m, 6H), 7.06 (s, 1H), 6.89 (d, *J* = 8.8 Hz, 1H), 5.14 (s, 1H), 4.34 (s, 2H), 3.15 (t, *J* = 6.8 Hz, 2H), 3.04 (t, *J* = 6.8 Hz, 2H), 2.35 (s, 3H); ^{13}C NMR (125 MHz, $CDCl_3$) δ 147.8, 136.7, 136.5, 136.2, 134.5, 129.3, 129.1, 128.0, 127.2, 127.0, 126.9, 126.5, 124.3, 122.0(1), 121.9(6), 121.9, 119.3, 118.7, 114.9, 112.6, 111.1, 101.3, 100.0, 76.2, 47.2, 25.1, 21.1(0), 21.0(6); IR (neat): 3351(br), 2340, 1373, 1270, 1096, 1083, 760, 658, 602, 574; HRESIMS Calcd for $[C_{30}H_{26}N_2Na]^+$ (M + Na^+) 437.1988, found 437.1991.

1-(4-(1*H*-indol-3-yl)but-1-yn-1-yl)-*N*-(4-methoxybenzyl)naphthalen-2-amine (3c)

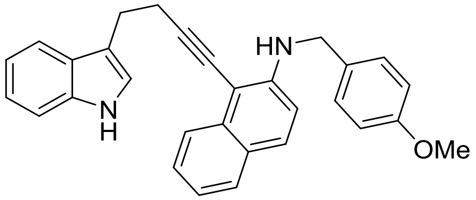

3c

The product was isolated through silica gel column chromatography (PE:EA = 7:1) as a pale yellow oil. ^{1}H NMR (500 MHz, $CDCl_3$) δ 8.00 (d, *J* = 8.5 Hz, 1H), 7.69 (s, 1H), 7.66 – 7.60 (m, 2H), 7.56 (d, *J* = 9.0 Hz, 1H), 7.40 – 7.34 (m, 1H), 7.29 – 7.27 (m, 2H), 7.22 – 7.15 (m, 3H), 7.13 – 7.09 (m, 1H), 7.05 (d, *J* = 2.0 Hz, 1H), 6.89 (d, *J* = 9.0 Hz, 1H), 6.87 – 6.82 (m, 2H), 5.12 (t, *J* = 6.0 Hz, 1H), 4.31 (d, *J* = 5.5 Hz, 2H), 3.78 (s, 3H), 3.14 (t, *J* = 7.0 Hz, 2H), 3.03 (t, *J* = 7.0 Hz, 2H); ^{13}C NMR (125 MHz, $CDCl_3$) δ 158.7, 147.8, 136.2, 134.5, 131.5, 129.1, 128.2, 128.0, 127.2, 126.9, 126.6, 124.3, 122.0(4), 122.0(1), 121.9, 119.3, 118.7, 115.0, 114.0, 112.7, 111.1, 101.3, 100.1, 76.2, 55.3, 47.0, 25.1, 21.1; IR (neat): 3376(br), 2384, 1330, 1252, 1180, 1112, 750, 673, 621, 597; HRESIMS Calcd for $[C_{30}H_{26}N_2NaO]^+$ (M + Na^+) 453.1937, found 453.1937.

1-(4-(1*H*-indol-3-yl)but-1-yn-1-yl)-*N*-(4-fluorobenzyl)naphthalen-2-amine (3d)

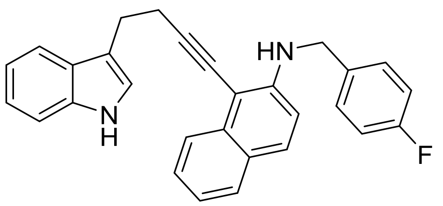

3d

The product was isolated through silica gel column chromatography (PE:EA = 7:1) as a pale yellow oil. ^{1}H NMR (500 MHz, $CDCl_3$) δ 8.00 (d, *J* = 8.5 Hz, 1H), 7.78 (s, 1H), 7.69 – 7.63 (m, 1H), 7.62 – 7.59 (m, 1H), 7.56 – 7.53 (m, 1H), 7.41 – 7.35 (m, 1H), 7.29 – 7.26 (m, 1H), 7.21 – 7.16 (m, 4H), 7.14 – 7.11 (m, 1H), 7.07 (d, *J* = 2.5 Hz, 1H), 6.99 – 6.95 (m, 2H), 6.81 (d, *J* = 9.0 Hz, 1H), 5.13 (s, 1H), 4.29 (s, 2H), 3.16 (t, *J* = 7.0 Hz, 2H), 3.05 (t, *J* = 7.0 Hz, 2H); ^{13}C NMR (125 MHz, $CDCl_3$) δ 161.0 (d, *J* = 242.5 Hz), 147.5, 136.2, 135.2 (d, *J* = 3.8 Hz), 134.5, 129.2, 128.3 (d, *J* = 8.8 Hz), 128.0, 127.3, 127.0, 126.6, 124.3, 122.2, 122.1, 121.8, 119.4, 118.7, 115.4 (d, *J* = 21.3 Hz), 115.0, 112.6, 111.1, 101.4, 100.4, 76.1, 46.8, 25.1, 21.1; IR (neat): 3303(br), 2209, 1360, 1248, 1156, 1080, 734, 667, 620, 601; HRESIMS Calcd for $[C_{29}H_{23}FN_2Na]^+$ (M + Na^+) 441.1737, found 441.1732.

1-(4-(1*H*-indol-3-yl)but-1-yn-1-yl)-*N*-(3-methylbenzyl)naphthalen-2-amine (3e)

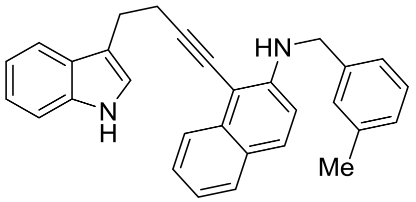

3e

The product was isolated through silica gel column chromatography (PE:EA = 7:1) as a pale yellow oil. ^{1}H NMR (500 MHz, $CDCl_3$) δ 8.00 (d, *J* = 8.5 Hz, 1H), 7.65 – 7.60 (m, 3H), 7.55 (d, *J* = 9.0 Hz, 1H), 7.40 – 7.32 (m, 2H), 7.27 – 7.21 (m, 2H), 7.19 – 7.15 (m, 2H), 7.13 – 7.08 (m, 3H), 7.03 (d, *J* = 2.0 Hz, 1H), 6.88 (d, *J* = 9.0 Hz, 1H), 5.16 (s, 1H), 4.33 (s, 2H), 3.14 (t, *J* = 7.0 Hz, 2H), 3.03 (t, *J* = 7.0 Hz, 2H), 2.33 (s, 3H); ^{13}C NMR (125 MHz, $CDCl_3$) δ 147.9, 139.6, 138.3, 136.2, 134.5, 129.2, 128.5, 128.0, 127.9, 127.7, 127.2, 126.9, 126.6, 124.3, 124.1, 122.0(4), 121.9(9), 121.9, 119.3, 118.7, 114.9, 112.6, 111.1, 101.3, 100.1, 76.2, 47.5, 25.1, 21.4, 21.2; IR (neat): 3373(br), 2369, 1373, 1225, 1143, 1082, 743, 673, 620, 610; HRESIMS Calcd for $[C_{30}H_{26}N_2Na]^+$ (M + Na^+) 437.1988, found 437.1991.

methyl 3-(((1-(4-(1*H*-indol-3-yl)but-1-yn-1-yl)naphthalen-2-yl)amino)methyl)benzoate (3f)

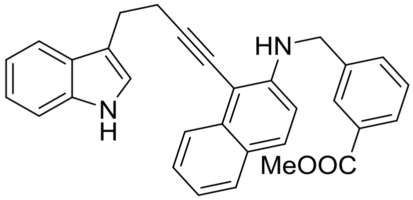

3f

The product was isolated through silica gel column chromatography (PE:EA = 7:1) as a pale yellow oil. ^{1}H NMR (500 MHz, $CDCl_3$) δ 8.18 (s, 1H), 8.01 (d, *J* = 8.5 Hz, 1H), 7.97 (s, 1H), 7.92 (d, *J* = 8.0 Hz, 1H), 7.67 (d, *J* = 8.0 Hz, 1H), 7.60 (d, *J* = 8.0 Hz, 1H), 7.52 (d, *J* = 9.0 Hz, 1H), 7.44 (d, *J* = 8.0 Hz, 1H), 7.41 – 7.32 (m, 2H), 7.20 – 7.15 (m, 3H), 7.14 – 7.09 (m, 2H), 6.76 (d, *J* = 9.0 Hz, 1H), 5.15 (t, *J* = 6.0 Hz, 1H), 4.28 (d, *J* = 6.0 Hz, 2H), 3.91 (s, 3H), 3.18 (t, *J* = 7.0 Hz, 2H), 3.08 (t, *J* = 7.0 Hz, 2H); ^{13}C NMR (125 MHz, $CDCl_3$) δ 167.3, 147.4, 140.3, 136.2, 134.4, 131.4, 130.4, 129.1, 128.7, 128.3, 128.0, 127.3, 126.9, 126.6, 124.3, 122.2, 122.1, 121.9, 119.3, 118.7, 114.8, 112.4, 111.2, 101.4, 100.4, 76.2, 52.2, 47.1, 25.0, 21.0; IR (neat): 3418(br), 2410, 1714, 1652, 1276, 1198, 1093, 763, 749, 418; HRESIMS Calcd for $[C_{31}H_{26}N_2NaO_2]^+$ (M + Na^+) 481.1886, found 481.1891.

1-(4-(1*H*-indol-3-yl)but-1-yn-1-yl)-*N*-(3-(trifluoromethyl)benzyl)naphthalen-2-amine (3g)

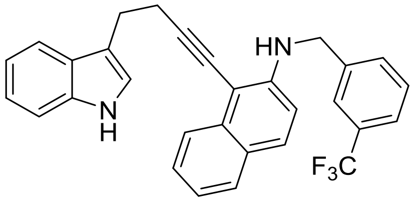

3g

The product was isolated through silica gel column chromatography (PE:EA = 7:1) as a yellow oil. ^{1}H NMR (500 MHz, $CDCl_3$) δ 8.01 (d, *J* = 8.5 Hz, 1H), 7.74 (s, 1H), 7.65 (d, *J* = 8.0 Hz, 1H), 7.60 (d, *J* = 8.0 Hz, 1H), 7.55 – 7.50 (m, 2H), 7.49 – 7.47 (m, 1H), 7.41 – 7.32 (m, 3H), 7.23 – 7.18 (m, 2H), 7.17 – 7.14 (m, 1H), 7.11 – 7.07 (m, 1H), 7.06 – 7.05 (m, 1H), 6.73 (d, *J* = 9.0 Hz, 1H), 5.13 (s, 1H), 4.27 (s, 2H), 3.16 (t, *J* = 7.0 Hz, 2H), 3.05 (t, *J* = 7.0 Hz, 2H); ^{13}C NMR (125 MHz, $CDCl_3$) δ 147.3, 140.9, 136.2, 134.4, 130.7, 129.9, 129.2, 129.0, 128.0, 127.3, 127.0, 126.7, 124.4, 123.8 (q, *J* = 3.8 Hz), 123.4 (q, *J* = 3.8 Hz), 122.3, 122.1, 121.9, 119.4, 118.7, 114.9 (q, *J* = 1.3 Hz),

112.4, 111.1, 101.5, 100.6, 76.1, 47.0, 25.0, 21.1; IR (neat): 3450(br), 2398, 1734, 1602, 1237, 1201, 1075, 726, 701, 447; HRESIMS Calcd for $[C_{30}H_{23}F_3N_2Na]^+$ (M + Na^+) 491.1706, found 491.1701.

1-(4-(1*H*-indol-3-yl)but-1-yn-1-yl)-*N*-(2-methylbenzyl)naphthalen-2-amine (3h)

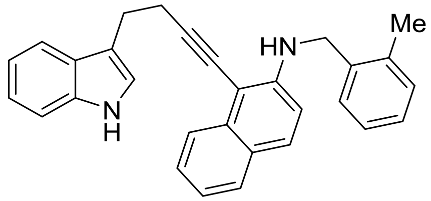

3h

The product was isolated through silica gel column chromatography (PE:EA = 7:1) as a pale yellow oil. ^{1}H NMR (500 MHz, $CDCl_3$) δ 8.01 (d, J = 8.0 Hz, 1H), 7.62 (d, J = 8.0 Hz, 2H), 7.58 – 7.55 (m, 2H), 7.41 – 7.35 (m, 2H), 7.27 – 7.21 (m, 3H), 7.20 – 7.14 (m, 3H), 7.11 – 7.08 (m, 1H), 6.99 (d, J = 2.0 Hz, 1H), 6.85 (d, J = 9.0 Hz, 1H), 4.99 (s, 1H), 4.27 (s, 2H), 3.12 (t, J = 7.0 Hz, 2H), 3.02 (t, J = 7.0 Hz, 2H), 2.32 (s, 3H); ^{13}C NMR (125 MHz, $CDCl_3$) δ 147.8, 137.2, 136.2, 136.0, 134.5, 130.3, 129.2, 128.0, 127.3, 127.2, 126.9, 126.6, 126.1, 124.3, 122.1, 122.0, 121.9, 119.3, 118.7, 114.8, 112.5, 111.1, 101.3, 100.0, 76.2, 45.6, 25.0, 21.1, 18.9; IR (neat): 3423(br), 2397, 1773, 1681, 1243, 1162, 1039, 785, 740, 399; HRESIMS Calcd for $[C_{30}H_{26}N_2Na]^+$ (M + Na^+) 437.1988, found 437.1995.

1-(4-(4-methyl-1*H*-indol-3-yl)but-1-yn-1-yl)-*N*-(4-methylbenzyl)naphthalen-2-amine (3i)

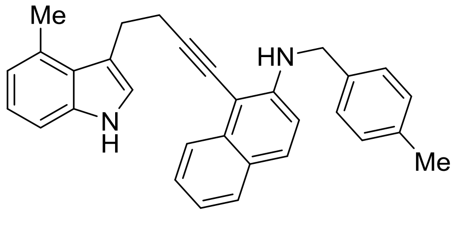

3i

The product was isolated through silica gel column chromatography (PE:EA = 7:1) as a pale yellow oil. ^{1}H NMR (400 MHz, $CDCl_3$) δ 8.04 (d, J = 8.0 Hz, 1H), 7.58 (d, J = 8.0 Hz, 1H), 7.51 (d, J = 8.8 Hz, 1H), 7.40 – 7.35 (m, 2H), 7.23 – 7.13 (m, 2H), 7.09 – 7.04 (m, 3H), 6.98 (s, 2H), 6.85 (s, 2H), 6.79 (d, J = 5.2 Hz, 1H), 5.16 (s, 1H), 4.26 (s, 2H), 3.23 (t, J = 12.8 Hz, 2H), 2.93 (t, J = 12.8 Hz, 2H), 2.66 (s, 3H), 2.29 (s, 3H); ^{13}C NMR (100 MHz, $CDCl_3$) δ 147.8, 136.7, 136.5,

136.4, 134.5, 130.5, 129.2, 129.1, 128.0, 127.0, 126.9, 126.5, 125.6, 124.2, 122.0, 121.8, 120.9, 115.4, 112.6, 109.0, 101.2, 100.0, 76.3, 47.2, 26.9, 22.1, 21.0, 20.3; IR (neat): 3349(br), 2301, 1647, 1513, 1275, 1260, 1122, 1093, 766, 754; HRESIMS Calcd for $[C_{31}H_{28}N_2Na]^+$ (M + Na^+) 451.2145, found 451.2146.

1-(4-(4-bromo-1*H*-indol-3-yl)but-1-yn-1-yl)-*N*-(4-methylbenzyl)naphthalen-2-amine (3j)

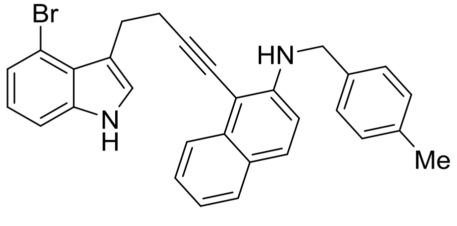

3j

The product was isolated through silica gel column chromatography (PE:EA = 7:1) as a pale yellow oil. ^{1}H NMR (500 MHz, $CDCl_3$) δ 8.01 (d, *J* = 8.0 Hz, 1H), 7.61 (d, *J* = 8.0 Hz, 1H), 7.56 (d, *J* = 9.0 Hz, 2H), 7.39 (t, *J* = 15.0 Hz, 1H), 7.26 – 7.22 (m, 3H), 7.21 – 7.13 (m, 4H), 7.02 (d, *J* = 2.0 Hz, 1H), 6.98 – 6.90 (m, 2H), 5.16 (s, 1H), 4.37 (s, 2H), 3.34 (t, *J* = 14.0 Hz, 2H), 3.05 (t, *J* = 13.5 Hz, 2H), 2.35 (s, 3H); ^{13}C NMR (125 MHz, $CDCl_3$) δ 147.8, 137.5, 136.9, 136.5, 134.6, 129.4, 129.1, 128.0, 127.1, 126.9, 126.6, 125.3, 124.3, 124.0, 123.9, 122.7, 122.1, 115.6, 114.2, 112.6, 110.4, 101.4, 100.3, 76.3, 47.4, 25.9, 22.5, 21.1; IR (neat): 3351(br), 2346, 1695, 1573, 1280, 1237, 1150, 1082, 767, 735; HRESIMS Calcd for $[C_{30}H_{25}BrN_2Na]^+$ (M + Na^+) 515.1093, found 515.1088.

1-(4-(5-methyl-1*H*-indol-3-yl)but-1-yn-1-yl)-*N*-(4-methylbenzyl)naphthalen-2-amine (3k)

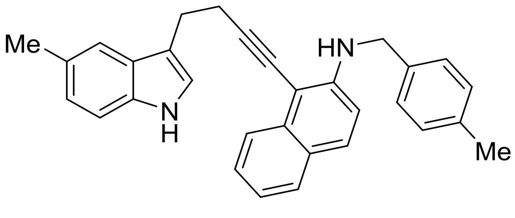

3k

The product was isolated through silica gel column chromatography (PE:EA = 7:1) as a pale yellow oil. ^{1}H NMR (400 MHz, $CDCl_3$) δ 8.02 (d, *J* = 8.4 Hz, 1H), 7.60 – 7.50 (m, 3H), 7.46 – 7.32 (m, 4H), 7.22 – 7.08 (m, 4H), 6.97 (d, *J* = 8.0 Hz, 1H), 6.92 (s, 1H), 6.85 (d, *J* = 8.8 Hz, 1H), 5.13 (s, 1H), 4.28 (s, 2H), 3.07 (t, *J* = 6.0 Hz, 2H), 2.98 (t, *J* = 6.0 Hz, 2H), 2.42 (s, 3H), 2.31 (s,

3H); ^{13}C NMR (100 MHz, $CDCl_3$) δ 147.8, 136.6, 136.5, 134.6, 134.1, 129.3, 129.1, 128.4, 128.3, 128.0, 127.4, 126.9, 126.8, 126.6, 124.3, 123.6, 122.0, 118.3, 114.3, 112.7, 110.8, 101.4, 100.2, 76.2, 47.2, 25.1, 21.5, 21.1, 21.0; IR (neat): 3343(br), 2295, 1642, 1494, 1280, 1254, 1110, 1072, 784, 737; HRESIMS Calcd for $[C_{31}H_{28}N_2Na]^+$ (M + Na^+) 451.2145, found 451.2138.

1-(4-(5-bromo-1*H*-indol-3-yl)but-1-yn-1-yl)-*N*-(4-methylbenzyl)naphthalen-2-amine (3l)

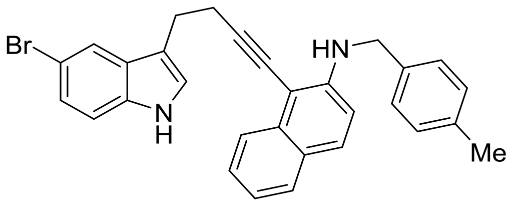

3l

The product was isolated through silica gel column chromatography (PE:EA = 7:1) as a pale yellow oil. ^{1}H NMR (400 MHz, $CDCl_3$) δ 8.02 (d, *J* = 8.4 Hz, 1H), 7.73 (s, 1H), 7.60 (d, *J* = 6.4 Hz, 1H), 7.56 – 7.53 (m, 2H), 7.39 – 7.35 (m, 1H), 7.23 – 7.20 (m, 1H), 7.19 – 7.15 (m, 3H), 7.13 – 7.10 (m, 2H), 7.04 (d, *J* = 6.8 Hz, 1H), 6.96 (s, 1H), 6.87 (d, *J* = 7.2 Hz, 1H), 5.11 (s, 1H), 4.32 (s, 2H), 3.03 (t, *J* = 5.6 Hz, 2H), 2.96 (t, *J* = 5.6 Hz, 2H), 2.33 (s, 3H); ^{13}C NMR (125 MHz, $CDCl_3$) δ 147.8, 136.8, 136.4, 134.7, 134.5, 129.3, 129.2, 128.9, 128.0, 127.0, 126.9, 126.6, 124.8, 124.2, 123.2, 122.1, 121.3, 114.5, 112.6(3), 112.5(7), 112.5, 101.0, 99.9, 76.4, 47.2, 24.8, 21.1, 21.0; IR (neat): 3370(br), 2367, 1643, 1560, 1255, 1228, 1119, 1063, 771, 756; HRESIMS Calcd for $[C_{30}H_{25}BrN_2Na]^+$ (M + Na^+) 515.1093, found 515.1100.

1-(4-(6-methyl-1*H*-indol-3-yl)but-1-yn-1-yl)-*N*-(4-methylbenzyl)naphthalen-2-amine (3m)

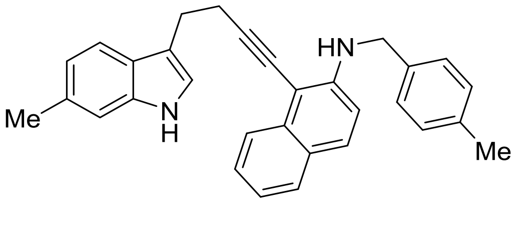

3m

The product was isolated through silica gel column chromatography (PE:EA = 7:1) as a pale yellow oil. ^{1}H NMR (400 MHz, $CDCl_3$) δ 8.02 (d, *J* = 8.4 Hz, 1H), 7.59 (d, *J* = 8.0 Hz, 1H), 7.54 – 7.48 (m, 2H), 7.41 (s, 1H), 7.37 (t, *J* = 7.5 Hz, 1H), 7.18 – 7.15 (m, 3H), 7.12 – 7.09 (m, 2H), 6.99 (s, 1H), 6.94 – 6.89 (m, 2H), 6.85 (d, *J* = 9.0 Hz, 1H), 5.13 (s, 1H), 4.29 (s, 2H), 3.08 (t, *J* =

7.0 Hz, 2H), 2.99 (t, *J* = 7.0 Hz, 2H), 2.42 (s, 3H), 2.32 (s, 3H); ^{13}C NMR (125 MHz, $CDCl_3$) δ 147.8, 136.6, 136.5, 134.5, 131.7, 129.3, 129.1, 128.0, 126.9, 126.8, 126.5, 125.1, 124.3, 122.0, 121.2, 121.0, 118.3, 114.7, 112.6, 111.1, 101.3, 100.1, 76.2, 47.2, 25.1, 21.6, 21.1(2), 21.0(5); IR (neat): 3331(br), 2307, 1667, 1585, 1273, 1226, 1171, 1068, 746, 728; HRESIMS Calcd for $[C_{31}H_{28}N_2Na]^+$ (M + Na^+) 451.2145, found 451.2151.

1-(4-(6-chloro-1*H*-indol-3-yl)but-1-yn-1-yl)-*N*-(4-methylbenzyl)naphthalen-2-amine (3n)

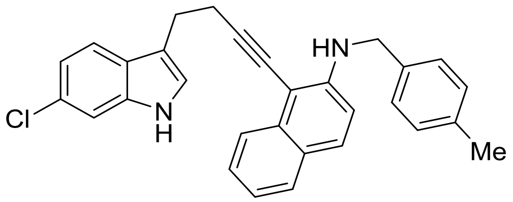

3n

The product was isolated through silica gel column chromatography (PE:EA = 7:1) as a pale yellow oil. ^{1}H NMR (500 MHz, $CDCl_3$) δ 7.96 (d, *J* = 8.5 Hz, 1H), 7.67 – 7.60 (m, 2H), 7.56 (d, *J* = 9.0 Hz, 1H), 7.51 (d, *J* = 8.5 Hz, 1H), 7.39 – 7.35 (m, 1H), 7.22 (d, *J* = 2 Hz, 1H), 7.20 – 7.15 (m, 3H), 7.13 (d, *J* = 8.0 Hz, 2H), 7.07 – 7.05 (m, 1H), 7.01 (d, *J* = 2.5 Hz, 1H), 6.89 (d, *J* = 9.0 Hz, 1H), 5.09 (s, 1H), 4.34 (s, 2H), 3.09 (t, *J* = 7.0 Hz, 2H), 3.00 (t, *J* = 7.0 Hz, 2H), 2.35 (s, 3H); ^{13}C NMR (125 MHz, $CDCl_3$) δ 147.9, 136.8, 136.5(1), 136.4(6), 134.5, 129.3, 129.2, 128.0, 127.0, 126.9, 126.6, 125.9, 124.2, 122.6, 122.1, 120.0, 119.6, 115.1, 112.6, 111.0, 101.0, 100.0, 76.4, 47.2, 24.9, 21.0(8), 21.0(5); IR (neat): 3329(br), 2267, 1680, 1506, 1243, 1199, 1149, 1070, 738, 686; HRESIMS Calcd for $[C_{30}H_{25}ClN_2Na]^+$ (M + Na^+) 471.1598, found 471.1593.

methyl 3-(4-(2-((4-methylbenzyl)amino)naphthalen-1-yl)but-3-yn-1-yl)-1*H*-indole-6-carboxylate (3o)

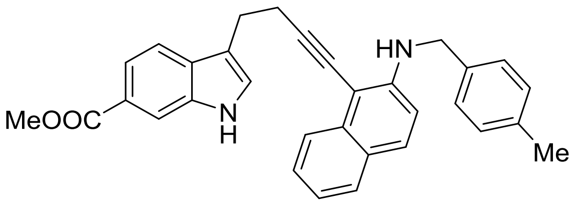

3o

The product was isolated through silica gel column chromatography (PE:EA = 7:1) as a pale yellow oil. ^{1}H NMR (500 MHz, $CDCl_3$) δ 8.10 (s, 1H), 8.02 (s, 1H), 7.96 (d, *J* = 8.5 Hz, 1H), 7.80

– 7.77 (m, 1H), 7.63 – 7.58 (m, 2H), 7.53 (d, *J* = 9.0 Hz, 1H), 7.36 (t, *J* = 8.0 Hz, 1H), 7.19 – 7.13 (m, 4H), 7.09 (d, *J* = 8.0 Hz, 2H), 6.86 (d, *J* = 9.0 Hz, 1H), 5.11 (s, 1H), 4.30 (s, 2H), 3.90 (s, 3H), 3.10 (t, *J* = 6.5 Hz, 2H), 2.99 (t, *J* = 6.5 Hz, 2H), 2.31 (s, 3H); ^{13}C NMR (125 MHz, $CDCl_3$) δ 168.2, 147.8, 136.7, 136.3, 135.4, 134.5, 130.7, 129.3, 129.2, 128.0, 126.9(1), 126.8(7), 126.5, 125.5, 124.1, 123.5, 122.1, 120.3, 118.2, 115.2, 113.6, 112.6, 101.0, 100.0, 76.4, 51.9, 47.2, 24.8, 21.0(2), 21.0(0); IR (neat): 3411(br), 2328, 1276, 1260, 1093, 1037, 764, 753, 747, 471; HRESIMS Calcd for $[C_{32}H_{28}N_2NaO_2]^+$ (M + Na^+) 495.2043, found 495.2040.

1-(4-(7-methyl-1*H*-indol-3-yl)but-1-yn-1-yl)-*N*-(4-methylbenzyl)naphthalen-2-amine (3p)

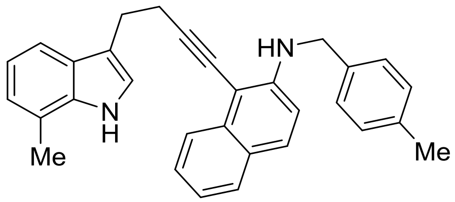

3p

The product was isolated through silica gel column chromatography (PE:EA = 7:1) as a pale yellow oil. ^{1}H NMR (500 MHz, $CDCl_3$) δ 8.01 (d, *J* = 8.5 Hz, 1H), 7.59 (d, *J* = 8.0 Hz, 1H), 7.54 – 7.46 (m, 3H), 7.37 (t, *J* = 7.5 Hz, 1H), 7.18 – 7.14 (m, 3H), 7.11 (d, *J* = 7.5 Hz, 2H), 7.03 (t, *J* = 7.0 Hz, 1H), 6.99 (s, 1H), 6.95 (d, *J* = 7.0 Hz, 1H), 6.85 (d, *J* = 9.0 Hz, 1H), 5.15 (s, 1H), 4.29 (s, 2H), 3.11 (t, *J* = 7.0 Hz, 2H), 3.01 (t, *J* = 7.0 Hz, 2H), 2.33 (s, 3H), 2.32 (s, 3H); ^{13}C NMR (125 MHz, $CDCl_3$) δ 147.8, 136.7, 136.5, 135.7, 134.5, 129.3, 129.1, 128.0, 126.8(9), 126.8(5), 126.7, 126.5, 124.3, 122.5, 122.0, 121.6, 120.3, 119.6, 116.4, 115.3, 112.6, 101.3, 100.1, 76.2, 47.1, 25.2, 21.1(0), 21.0(5), 16.4; IR (neat): 3347(br), 2350, 1527, 1228, 1140, 1032, 780, 777, 720, 454; HRESIMS Calcd for $[C_{31}H_{28}N_2Na]^+$ (M + Na^+) 451.2145, found 451.2144.

1-(4-(1*H*-indol-3-yl)but-1-yn-1-yl)-6-methyl-*N*-(4-methylbenzyl)naphthalen-2-amine (3q)

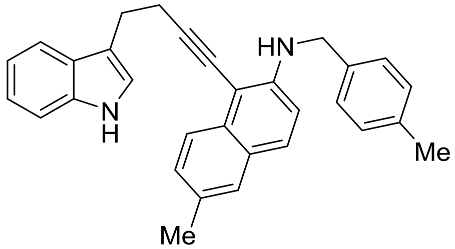

3q

The product was isolated through silica gel column chromatography (PE:EA = 7:1) as a pale yellow oil. ^{1}H NMR (500 MHz, $CDCl_3$) δ 7.91 (d, *J* = 8.5 Hz, 1H), 7.62 (d, *J* = 8.0 Hz, 2H), 7.52 (s, 1H), 7.47 (d, *J* = 9.0 Hz, 1H), 7.38 (s, 1H), 7.36 – 7.34 (m, 1H), 7.28 – 7.21 (m, 2H), 7.18 – 7.14 (m, 2H), 7.13 – 7.09 (m, 2H), 7.00 (d, *J* = 1.5 Hz, 1H), 6.85 (d, *J* = 9.0 Hz, 1H), 4.99 (s, 1H), 4.31 (s, 2H), 3.12 (t, *J* = 7.0 Hz, 2H), 3.01 (t, *J* = 7.0 Hz, 2H), 2.41 (s, 3H), 2.33 (s, 3H); ^{13}C NMR (125 MHz, $CDCl_3$) δ 147.3, 136.7, 136.6, 136.2, 134.3, 132.7, 131.3, 129.3, 129.1, 128.5, 127.2, 127.0, 126.8, 124.2, 122.0, 121.9, 119.3, 118.7, 114.9, 112.7, 111.1, 101.1, 100.2, 76.3, 47.4, 25.1, 21.2, 21.1(1), 21.0(6); IR (neat): 3399 (br), 2320, 1864, 1620, 1243, 1201, 1047, 789, 727, 436; HRESIMS Calcd for $[C_{31}H_{28}N_2Na]^+$ (M + Na^+) 451.2145, found 451.2141.

1-(4-(1*H*-indol-3-yl)but-1-yn-1-yl)-6-bromo-*N*-(4-methylbenzyl)naphthalen-2-amine (3r)

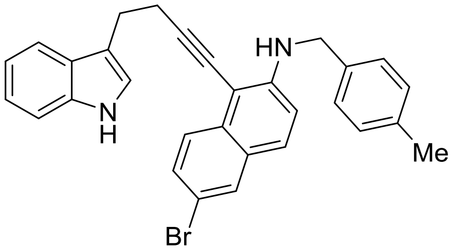

3r

The product was isolated through silica gel column chromatography (PE:EA = 7:1) as a pale yellow oil. ^{1}H NMR (500 MHz, $CDCl_3$) δ 7.81 (d, *J* = 9.0 Hz, 1H), 7.72 (s, 1H), 7.63 (d, *J* = 7.5 Hz, 2H), 7.43 – 7.38 (m, 2H), 7.36 – 7.32 (m, 1H), 7.26 (d, *J* = 8.0 Hz, 1H), 7.20 – 7.15 (m, 2H), 7.14 – 7.09 (m, 3H), 7.01 (s, 1H), 6.85 (d, *J* = 9.0 Hz, 1H), 5.13 (s, 1H), 4.29 (s, 2H), 3.12 (t, *J* = 13.5 Hz, 2H), 3.02 (t, *J* = 13.5 Hz, 2H), 2.34 (s, 3H); ^{13}C NMR (125 MHz, $CDCl_3$) δ 148.0, 136.8, 136.2, 133.0, 129.9, 129.8, 129.3, 128.1, 127.6, 127.2, 126.9, 126.2, 122.1, 121.9, 119.4, 118.7, 115.3, 114.8, 113.5, 111.1, 101.7, 100.2, 75.8, 47.1, 25.0, 21.1; IR (neat): 3375(br), 2207, 1684, 1602, 1254, 1193, 1086, 743, 705; HRESIMS Calcd for $[C_{30}H_{25}BrN_2Na]^+$ (M + Na^+) 515.1093, found 515.1096.

1-((2-(1*H*-indol-3-yl)phenyl)ethynyl)-*N*-(4-methylbenzyl)naphthalen-2-amine (3s)

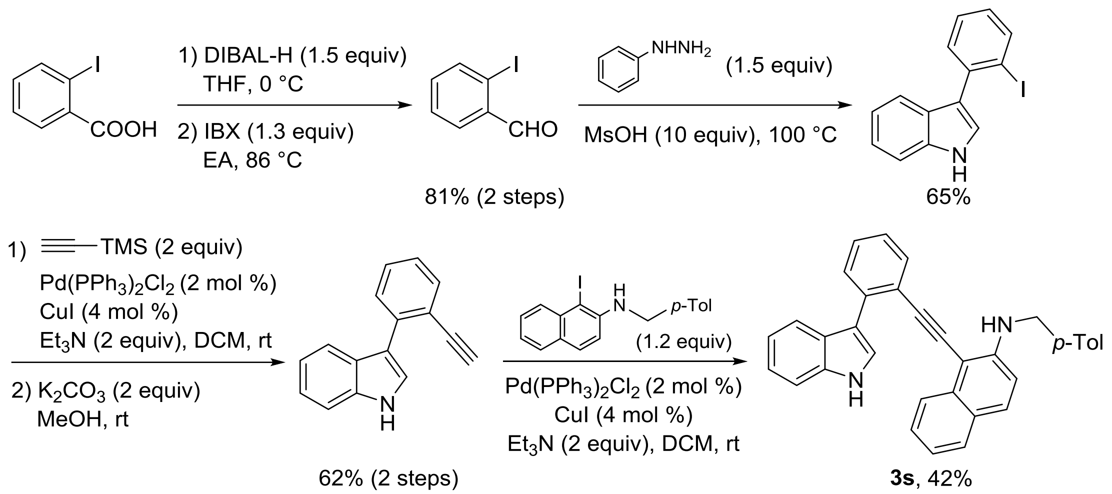

Compound **3s** was prepared according to the above procedures. The product was isolated through silica gel column chromatography (PE:EA = 5:1) as a pale yellow oil. ^{1}H NMR (400 MHz, $CDCl_3$) δ 7.96 (d, *J* = 8.4 Hz, 1H), 7.80 – 7.76 (m, 1H), 7.74 (d, *J* = 7.2 Hz, 1H), 7.57 – 7.53 (m, 3H), 7.49 (d, *J* = 9.2 Hz, 1H), 7.41 (d, *J* = 2.4 Hz, 1H), 7.37 – 7.29 (m, 4H), 7.18 – 7.11 (m, 2H), 7.09 – 7.03 (m, 5H), 6.75 (d, *J* = 9.2 Hz, 1H), 4.91 (s, 1H), 4.07 (d, *J* = 5.6 Hz, 2H), 2.31 (s, 3H); ^{13}C NMR (100 MHz, $CDCl_3$) δ 147.9, 136.7, 136.5, 136.1, 135.8, 134.4, 132.8, 130.1, 130.0, 129.3, 128.1, 127.9, 127.1, 126.9, 126.5, 126.4, 126.3, 124.2, 123.6, 123.1, 122.3, 122.2, 120.2, 120.0, 116.6, 112.6, 111.3, 101.3, 99.4, 87.7, 46.9, 21.0; IR (neat): 3428(br), 2921, 2088, 1590, 1547, 1458, 1278, 816, 755; HRESIMS Calcd for $[C_{34}H_{26}N_2Na]^+$ (M + Na$^+$) 485.1988, found 485.1991.

1-(5-(1*H*-indol-3-yl)pent-1-yn-1-yl)-*N*-(4-methylbenzyl)naphthalen-2-amine (3t)

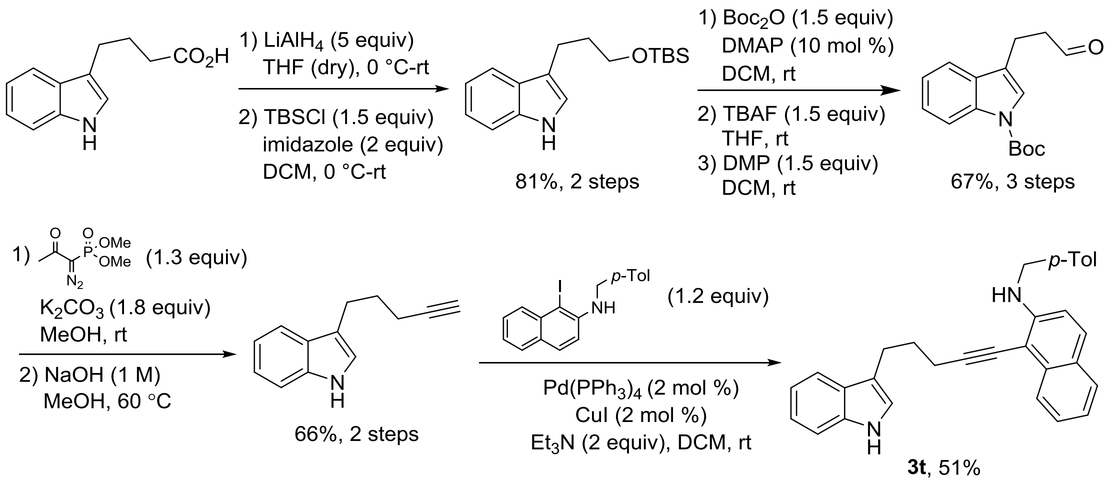

Compound **3t** was prepared according to the above procedures. The product was isolated through silica gel column chromatography (PE:EA = 10:1) as a pale yellow oil. ^{1}H NMR (400 MHz, $CDCl_3$)

δ 8.20 (d, *J* = 8.4 Hz, 1H), 7.91 (s, 1H), 7.71 (d, *J* = 8.0 Hz, 2H), 7.67 (d, *J* = 8.8 Hz, 1H), 7.51 (t, *J* = 8.4 Hz, 1H), 7.39 (d, *J* = 8.0 Hz, 1H), 7.33 (d, *J* = 8.0 Hz, 2H), 7.27 – 7.23 (m, 2H), 7.19 – 7.14 (m, 3H), 7.05 – 7.01 (m, 2H), 5.46 (s, 1H), 4.58 (s, 2H), 3.06 (t, *J* = 7.2 Hz, 2H), 2.73 (t, *J* = 7.2 Hz, 2H), 2.37 (s, 3H), 2.20 – 2.12 (m, 2H); ^{13}C NMR (100 MHz, $CDCl_3$) δ 147.8, 136.8, 136.3, 134.6, 129.3, 129.2, 128.1, 127.5, 126.9(9), 126.9(5), 126.7, 124.3, 122.1, 121.9, 121.6, 119.2, 118.9, 115.6, 112.8, 111.1, 101.4, 100.4, 75.9, 47.5, 29.6, 24.3, 21.0, 19.7; IR (neat): 3407(br), 2352, 1275, 1261, 1093, 1037, 765, 757, 750, 436; HRESIMS Calcd for $[C_{31}H_{28}N_2Na]^+$ (M + Na^+) 451.2145, found 451.2152.

1-(6-(1*H*-indol-3-yl)hex-1-yn-1-yl)-*N*-(4-methylbenzyl)naphthalen-2-amine (3u)

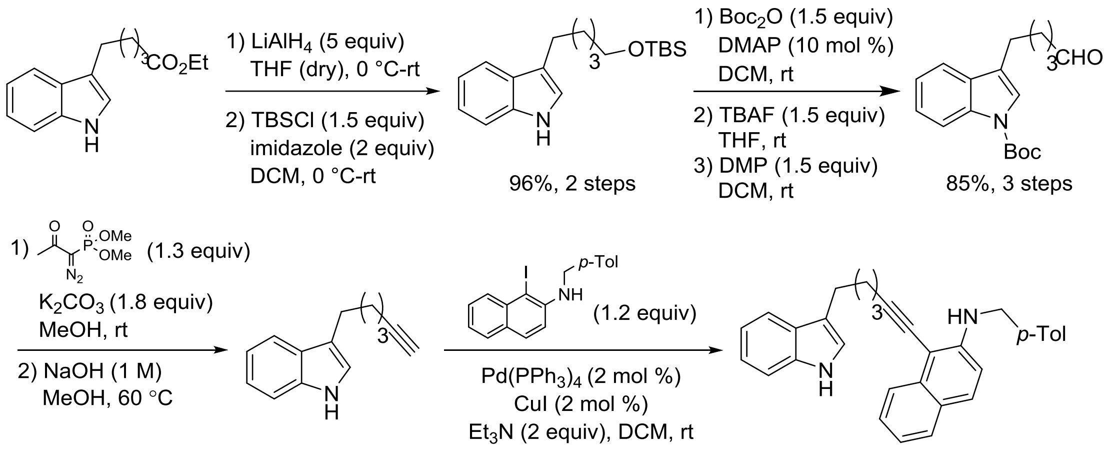

3u, 48%

Compound **3u** was prepared according to the above procedures. The product was isolated through silica gel column chromatography (PE:EA = 10:1) as a pale yellow oil. ^{1}H NMR (400 MHz, $CDCl_3$) δ 8.09 (d, *J* = 8.4 Hz, 1H), 7.78 – 7.66 (m, 2H), 7.63 – 7.52 (m, 3H), 7.42 – 7.37 (m, 1H), 7.35 – 7.31 (m, 1H), 7.27 – 7.17 (m, 3H), 7.16 – 7.12 (m, 1H), 7.10 – 7.04 (m, 2H), 6.92 (d, *J* = 8.8 Hz, 1H), 6.85 (d, *J* = 2.0 Hz, 1H), 5.35 (s, 1H), 4.44 (s, 2H), 2.78 (t, *J* = 7.2 Hz, 2H), 2.63 (t, *J* = 6.8 Hz, 2H), 2.29 (s, 3H), 1.96 – 1.88 (m, 2H), 1.80 – 1.72 (m, 2H); ^{13}C NMR (100 MHz, $CDCl_3$) δ 147.8, 136.7, 136.2(9), 136.2(5), 135.2, 134.6, 129.3, 129.1, 128.0, 127.5, 126.9, 126.6, 124.3, 122.1, 121.8, 121.1, 119.0, 118.8, 116.3, 112.8, 111.0, 101.5, 100.3, 75.7, 47.4, 29.3, 28.9, 24.6, 21.0, 19.9; IR (neat): 3298(br), 2222, 1585, 1269, 1093, 1042, 861, 767, 752; HRESIMS Calcd for $[C_{32}H_{30}N_2Na]^+$ (M + Na^+) 465.2301, found 465.2308

5. General Procedure: CPA Catalysis

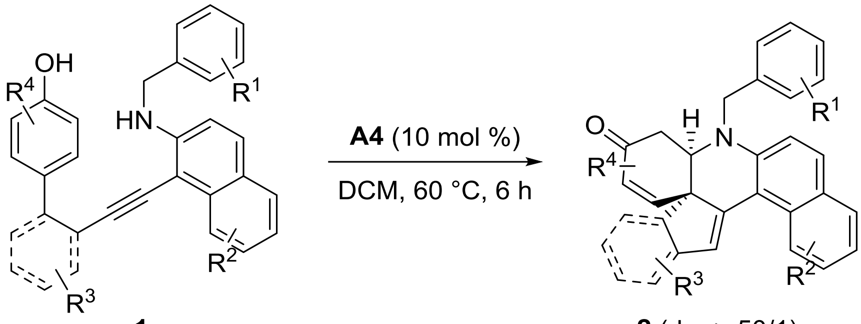

1 **2** (d.r. > 50/1)

A4 (8.5 mg, 0.01 mmol) were added to a solution of homopropargyl amines **1** (0.10 mmol) in DCM (2.0 mL) at room temperature (rt). The reaction mixture was then stirred at 60 ^{o}C, and the reaction progress was monitored by TLC. The reaction typically took 6 h. Upon completion, the mixture was concentrated under reduced pressure and the residue was purified by column chromatography on silica gel (hexanes/ethyl acetate) to afford the desired product **2**.

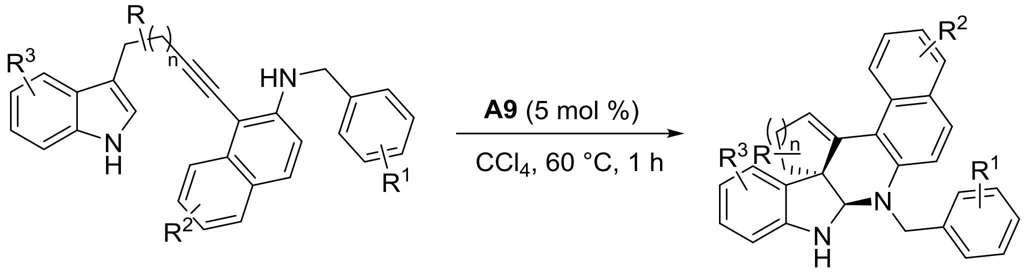

3 (n = 1, 2) **4** (d. r. > 50/1)

A9 (3.6 mg, 0.005 mmol) were added to a solution of homopropargyl amine **3** (0.10 mmol) in CCl_4 (2.0 mL) at room temperature (rt). The reaction mixture was then stirred at 60 ^{o}C, and the reaction progress was monitored by TLC. The reaction typically took 1 h. Upon completion, the mixture was concentrated under reduced pressure and the residue was purified by column chromatography on silica gel (hexanes/ethyl acetate) to afford the desired product **4**.

6. Analytical Data of the Products

(7a*R*,11a*S*)-7-benzyl-7a,8-dihydrobenzo[*a*]indeno[1,2-*l*]acridin-9(7*H*)-one (2a)

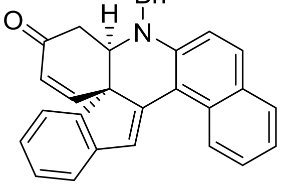

2a

Compound **2a** was prepared in 91% yield (38.7 mg) according to the general procedure (Fig. 3, entry 1). The product was isolated through silica gel column chromatography (PE:EA = 5:1) as a pale yellow solid (mp 166-167 °C). $[\alpha]_D^{20}$ = -4.6 °(c = 1.0, $CHCl_3$). 96% ee (determined by HPLC: Chiralpak ADH Column, 20/80 *i*-PrOH/hexane, 1.0 mL/min, 254 nm; TR = 10.78 min (major), 14.64 min (minor)). ^{1}H NMR (500 MHz, $CDCl_3$) δ 8.44 (d, *J* = 8.5 Hz, 1H), 7.70 (d, *J* = 8.0 Hz, 1H), 7.57 (d, *J* = 9.5 Hz, 1H), 7.53 – 7.48 (m, 2H), 7.45 (d, *J* = 7.5 Hz, 1H), 7.42 – 7.38 (m, 1H), 7.32 – 7.24 (m, 5H), 7.22 – 7.18 (m, 1H), 7.13 (d, *J* = 7.0 Hz, 2H), 7.02 (d, *J* = 9.5 Hz, 1H), 6.26 (dd, *J* = 10.0, 1.5 Hz, 1H), 6.14 (d, *J* = 10.0 Hz, 1H), 4.64 (s, 2H), 3.74 – 3.71 (m, 1H), 3.24 (dd, *J* = 17.5, 3.0 Hz, 1H), 3.04 (dd, *J* = 18.0, 2.5 Hz, 1H); ^{13}C NMR (125 MHz, $CDCl_3$) δ 196.8, 148.1, 145.6, 145.1, 144.8, 142.8, 138.5, 131.0, 130.7, 129.9, 128.9, 128.7, 128.4, 127.8, 127.3, 126.2, 125.8, 125.1, 124.3, 123.2, 123.0, 121.7, 116.8, 112.0, 62.6, 55.7, 55.1, 40.1; IR (neat): 3006, 1681, 1507, 1456, 1385, 1275, 1260, 1063, 814, 749; HRESIMS Calcd for $[C_{31}H_{23}NNaO]^+$ (M + Na^+) 448.1672, found 448.1676.

(7a*R*,11a*S*)-7-(4-chlorobenzyl)-7a,8-dihydrobenzo[*a*]indeno[1,2-*l*]acridin-9(7*H*)-one (2b)

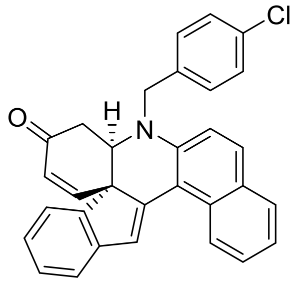

2b

Compound **2b** was prepared in 67% yield (30.8 mg) according to the general procedure (Fig. 3, entry 2). The product was isolated through silica gel column chromatography (PE:EA = 7:1) as a pale yellow oil. $[\alpha]_D^{20}$ = -1.3 °(c = 1.0, $CHCl_3$). 86% ee (determined by HPLC: Chiralpak ODH Column, 30/70 *i*-PrOH/hexane, 1.0 mL/min, 254 nm; TR = 9.57 min (major), 14.79 min (minor)). ^{1}H NMR (400 MHz, $CDCl_3$) δ 8.44 (d, *J* = 8.4 Hz, 1H), 7.72 (d, *J* = 8.0 Hz, 1H), 7.59 (d, *J* = 9.2 Hz, 1H), 7.55 – 7.50 (m, 2H), 7.48 – 7.39 (m, 2H), 7.35 – 7.30 (m, 1H), 7.26 – 7.20 (m, 4H), 7.06 (d, *J* = 8.4 Hz, 2H), 6.95 (d, *J* = 9.2 Hz, 1H), 6.27 (dd, *J* = 10.0, 1.6 Hz, 1H), 6.15 (d, *J* = 10.0 Hz, 1H), 4.60 (s, 2H), 3.75 – 3.67 (m, 1H), 3.27 (dd, *J* = 17.6, 3.2 Hz, 1H), 3.00 (dd, *J* = 17.6, 2.0 Hz, 1H); ^{13}C NMR (100 MHz, $CDCl_3$) δ 196.8, 148.2, 145.5, 144.9, 144.5, 142.7, 137.1, 133.1, 131.0, 130.7, 130.0, 129.0, 128.9, 128.4, 127.9, 127.6, 127.4, 126.0, 125.2, 124.3, 123.2(2), 123.1(7),

121.8, 116.6, 112.3, 62.8, 55.4, 55.1, 40.1; IR (neat): 3007, 1574, 1540, 1507, 1488, 1456, 1397, 1275, 1260, 1069, 750; HRESIMS Calcd for $[C_{31}H_{22}ClNNaO]^+$ (M + Na^+) 482.1282, found 482.1286.

(7a*R*,11a*S*)-7-(4-methylbenzyl)-7a,8-dihydrobenzo[*a*]indeno[1,2-*l*]acridin-9(7*H*)-one (2c)

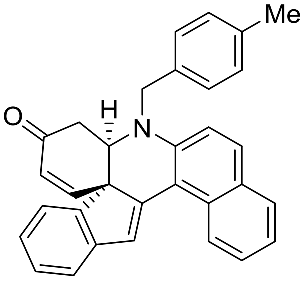

2c

Compound **2c** was prepared in 51% yield (22.4 mg) according to the general procedure (Fig. 3, entry 3). The product was isolated through silica gel column chromatography (PE:EA = 5:1) as a pale yellow oil. $[\alpha]_D^{20}$ = -1.0 °(c = 1.0, $CHCl_3$). 90% ee (determined by HPLC: Chiralpak ODH Column, 20/80 *i*-PrOH/hexane, 1.0 mL/min, 254 nm; TR = 8.77 min (major), 13.57 min (minor)). ^{1}H NMR (500 MHz, $CDCl_3$) δ 8.44 (d, *J* = 8.5 Hz, 1H), 7.71 (d, *J* = 7.5 Hz, 1H), 7.59 (d, *J* = 9.0 Hz, 1H), 7.54 – 7.49 (m, 2H), 7.46 (d, *J* = 7.5 Hz, 1H), 7.41 (t, *J* = 7.5 Hz, 1H), 7.31 (t, *J* = 7.0 Hz, 1H), 7.25 – 7.19 (m, 2H), 7.09 (d, *J* = 8.0 Hz, 2H), 7.06 – 7.01 (m, 3H), 6.27 (dd, *J* = 10.0, 1.5 Hz, 1H), 6.16 (d, *J* = 10.0 Hz, 1H), 4.68 – 4.59 (m, 2H), 3.74 (s, 1H), 3.25 (d, *J* = 17.5 Hz, 1H), 3.07 (d, *J* = 17.5 Hz, 1H), 2.31 (s, 3H); ^{13}C NMR (125 MHz, $CDCl_3$) δ 196.9, 148.1, 145.7, 145.2, 144.9, 142.8, 136.9, 135.4, 130.7, 129.9, 129.4, 128.9, 128.4, 127.8, 127.3, 126.2, 125.7, 125.1, 124.3, 123.2, 123.0, 121.6, 116.9, 115.0, 111.9, 62.5, 55.5, 55.1, 40.1, 21.1; IR (neat): 3005, 1681, 1646, 1507, 1397, 1275, 1260, 1066, 749; HRESIMS Calcd for $[C_{32}H_{25}NNaO]^+$ (M + Na^+) 462.1828, found 462.1832.

(7a*R*,11a*S*)-7-(4-methoxybenzyl)-7a,8-dihydrobenzo[*a*]indeno[1,2-*l*]acridin-9(7*H*)-one (2d)

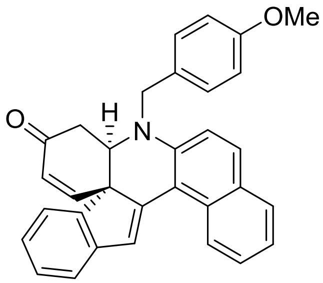

2d

Compound **2d** was prepared in 72% yield (32.8 mg) according to the general procedure (Fig. 3, entry 4). The product was isolated through silica gel column chromatography (PE:EA = 5:1) as a pale yellow solid (mp 193-194 ºC). $[\alpha]_D^{20}$ = -2.2 º(c = 1.0, $CHCl_3$). 95% ee (determined by HPLC: Chiralpak ODH Column, 20/80 *i*-PrOH/hexane, 1.0 mL/min, 254 nm; TR = 13.82 min (major), 20.07 min (minor)). ^1H NMR (500 MHz, $CDCl_3$) δ 8.43 (d, *J* = 8.5 Hz, 1H), 7.71 (d, *J* = 8.0 Hz, 1H), 7.59 (d, *J* = 9.0 Hz, 1H), 7.53 – 7.49 (m, 2H), 7.46 (d, *J* = 7.5 Hz, 1H), 7.40 (t, *J* = 7.5 Hz, 1H), 7.31 (t, *J* = 7.5 Hz, 1H), 7.24 – 7.19 (m, 2H), 7.06 – 7.02 (m, 3H), 6.80 (d, *J* = 9.0 Hz, 2H), 6.26 (dd, *J* = 10.0, 1.5 Hz, 1H), 6.14 (d, *J* = 9.5 Hz, 1H), 4.66 – 4.54 (m, 2H), 3.75 (s, 3H), 3.74 – 3.66 (m, 1H), 3.25 (dd, *J* = 17.5, 3.0 Hz, 1H), 3.08 (dd, *J* = 17.5, 2.0 Hz, 1H); ^{13}C NMR (125 MHz, $CDCl_3$) δ 196.9, 158.8, 148.1, 145.6, 145.2, 144.8, 142.8, 131.0, 130.7, 130.2, 129.9, 128.9, 128.4, 127.8, 127.4, 127.3, 125.7, 125.0, 124.3, 123.2, 123.0, 121.6, 117.0, 114.1, 112.0, 62.4, 55.2, 55.0, 40.0; IR (neat): 3006, 1681, 1540, 1507, 1456, 1396, 1275, 1260, 1066, 749; HRESIMS Calcd for $[C_{32}H_{25}NNaO_2]^+$ (M + Na^+) 478.1778, found 478.1771.

(7a*R*,11a*S*)-7-(3-chlorobenzyl)-7a,8-dihydrobenzo[*a*]indeno[1,2-*l*]acridin-9(7*H*)-one (2e)

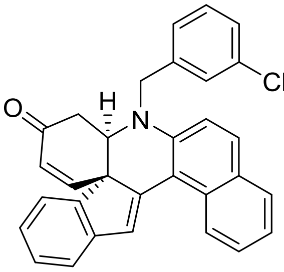

2e

Compound **2e** was prepared in 89% yield (40.9 mg) according to the general procedure (Fig. 3, entry 5). The product was isolated through silica gel column chromatography (PE:EA = 7:1) as a pale yellow oil. $[\alpha]_D^{20}$ = -2.3 º(c = 1.0, $CHCl_3$). 97% ee (determined by HPLC: Chiralpak ADH Column, 20/80 *i*-PrOH/hexane, 1.0 mL/min, 254 nm; TR = 12.13 min (minor), 15.13 min (major)). ^1H NMR (500 MHz, $CDCl_3$) δ 8.45 (d, *J* = 8.5 Hz, 1H), 7.71 (d, *J* = 8.0 Hz, 1H), 7.57 (d, *J* = 9.0 Hz, 1H), 7.55 – 7.51 (m, 2H), 7.47 – 7.37 (m, 3H), 7.34 – 7.29 (m, 1H), 7.28 (s, 1H), 7.24 – 7.20 (m, 2H), 7.19 – 7.17 (m, 1H), 7.16 – 7.13 (m, 1H), 6.85 (d, *J* = 9.0 Hz, 1H), 6.32 (dd, *J* = 10.0, 2.0 Hz, 1H), 6.18 (d, *J* = 10.0 Hz, 1H), 4.69 – 4.58 (m, 2H), 3.82 – 3.79 (m, 1H), 3.26 (dd, *J* = 18.0, 3.5 Hz, 1H), 2.92 (dd, *J* = 18.0, 2.0 Hz, 1H); ^{13}C NMR (125 MHz, $CDCl_3$) δ 196.7, 148.4, 145.5, 144.9, 144.6, 142.7, 135.9, 131.8, 130.9, 130.7, 130.0, 129.4, 129.0, 128.7, 128.4(4), 128.4(1), 127.9, 127.4, 127.0, 126.1, 125.2, 124.3, 123.3, 123.1, 121.7, 116.3, 112.0, 63.4, 55.0, 40.1, 29.7;

IR (neat): 3006, 1681, 1540, 1507, 1456, 1396, 1275, 1260, 1047, 750; HRESIMS Calcd for $[C_{31}H_{22}ClNNaO]^+$ (M + Na^+) 482.1282, found 482.1286.

(7a*R*,11a*S*)-7-(3-bromobenzyl)-7a,8-dihydrobenzo[*a*]indeno[1,2-*l*]acridin-9(7*H*)-one (2f)

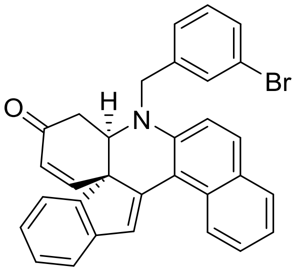

2f

Compound **2f** was prepared in 76% yield (38.3 mg) according to the general procedure (Fig. 3, entry 6). The product was isolated through silica gel column chromatography (PE:EA = 7:1) as a pale yellow oil. $[\alpha]_D^{20}$ = -3.5 °(c = 1.0, $CHCl_3$). 95% ee (determined by HPLC: Chiralpak ODH Column, 20/80 *i*-PrOH/hexane, 1.0 mL/min, 254 nm; TR = 10.92 min (major), 19.08 min (minor)). ^1H NMR (400 MHz, $CDCl_3$) δ 8.44 (d, *J* = 8.4 Hz, 1H), 7.72 (d, *J* = 8.0 Hz, 1H), 7.60 (d, *J* = 9.2 Hz, 1H), 7.55 – 7.50 (m, 2H), 7.47 – 7.30 (m, 5H), 7.27 (s, 1H), 7.24 – 7.20 (m, 1H), 7.15 (t, *J* = 7.6 Hz, 1H), 7.06 (d, *J* = 7.6 Hz, 1H), 6.93 (d, *J* = 9.2 Hz, 1H), 6.28 (dd, *J* = 10.0, 1.6 Hz, 1H), 6.17 (d, *J* = 10.0 Hz, 1H), 4.66 – 4.52 (m, 2H), 3.74 – 3.71 (m, 1H), 3.26 (dd, *J* = 18.0, 3.2 Hz, 1H), 2.96 (dd, *J* = 18.0, 2.4 Hz, 1H); ^{13}C NMR (100 MHz, $CDCl_3$) δ 196.5, 148.1, 145.5, 144.9, 144.4, 142.7, 141.2, 130.9, 130.8, 130.5, 130.3, 130.0, 129.3, 129.0, 128.5, 127.9, 127.4, 126.1, 125.2, 124.8, 124.3, 123.2(2), 123.1(8), 122.9, 121.7, 116.4, 112.2, 63.0, 55.6, 55.1, 40.2; IR (neat): 3006, 1681, 1540, 1456, 1423, 1275, 1260, 1066, 750; HRESIMS Calcd for $[C_{31}H_{22}BrNNaO]^+$ (M + Na^+) 526.0777, found 526.0781.

methyl 3-(((7a*R*,11a*S*)-9-oxo-8,9-dihydrobenzo[*a*]indeno[1,2-*l*]acridin-7(7a*H*)-yl)methyl)benzoate (2g)

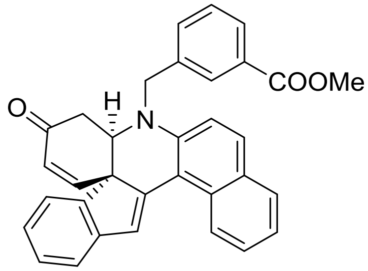

2g

Compound **2g** was prepared in 75% yield (36.3 mg) according to the general procedure (Fig. 3, entry 7). The product was isolated through silica gel column chromatography (PE:EA = 5:1) as a pale yellow solid (mp 197-198 ºC). $[\alpha]_D^{20}$ = -0.8 º(c = 1.0, $CHCl_3$). >99% ee(determined by HPLC: Chiralpak ODH Column, 20/80 *i*-PrOH/hexane, 1.0 mL/min, 254 nm; TR = 24.15 min (major), 28.30 min (minor)). ^1H NMR (500 MHz, $CDCl_3$) δ 8.45 (d, *J* = 8.5 Hz, 1H), 7.93 (d, *J* = 7.5 Hz, 1H), 7.86 (s, 1H), 7.71 (d, *J* = 8.0 Hz, 1H), 7.58 (d, *J* = 9.0 Hz, 1H), 7.55 – 7.50 (m, 2H), 7.46 (d, *J* = 7.5 Hz, 1H), 7.41 (t, *J* = 8.0 Hz, 1H), 7.37 – 7.30 (m, 3H), 7.27 (s, 1H), 7.24 – 7.20 (m, 1H), 6.94 (d, *J* = 9.5 Hz, 1H), 6.29 (dd, *J* = 10.0, 2.0 Hz, 1H), 6.17 (d, *J* = 10.0 Hz, 1H), 4.72 – 4.62 (m, 2H), 3.88 (s, 3H), 3.78 – 3.72 (m, 1H), 3.26 (dd, *J* = 18.0, 3.0 Hz, 1H), 2.98 (dd, *J* = 18.0, 2.0 Hz, 1H); ^{13}C NMR (125 MHz, $CDCl_3$) δ 196.4, 166.7, 148.0, 145.5, 144.9, 144.5, 142.7, 139.2, 130.9, 130.7, 130.5, 130.0, 128.9(2), 128.8(5), 128.6, 128.4, 127.9, 127.4, 126.0, 125.1, 124.3, 123.2, 123.1, 121.7, 116.5, 112.2, 62.9, 55.7, 55.1, 52.1, 40.1; IR (neat): 3005, 1540, 1507, 1456, 1397, 1275, 1260, 1065, 749; HRESIMS Calcd for $[C_{33}H_{25}NNaO_3]^+$ (M + Na^+) 506.1727, found 506.1732.

(7a*R*,11a*S*)-7-(3-(trifluoromethyl)benzyl)-7a,8-dihydrobenzo[*a*]indeno[1,2-*l*]acridin-9(7*H*)-one (2h)

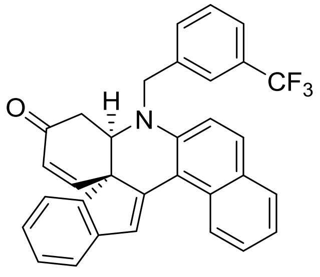

2h

Compound **2h** was prepared in 81% yield (40.0 mg) according to the general procedure (Fig. 3, entry 8). The product was isolated through silica gel column chromatography (PE:EA = 5:1) as a pale yellow oil. $[\alpha]_D^{20}$ = -2.5 º(c = 1.0, $CHCl_3$). 88% ee (determined by HPLC: Chiralpak ADH Column, 10/90 *i*-PrOH/hexane, 1.0 mL/min, 254 nm; TR = 16.27 min (major), 18.95 min (minor)). ^1H NMR (500 MHz, $CDCl_3$) δ 8.45 (d, *J* = 8.5 Hz, 1H), 7.73 (d, *J* = 7.5 Hz, 1H), 7.60 (d, *J* = 9.0 Hz, 1H), 7.56 – 7.51 (m, 3H), 7.46 (d, *J* = 8.0 Hz, 1H), 7.44 – 7.40 (m, 3H), 7.36 – 7.32 (m, 2H), 7.28 (s, 1H), 7.25 – 7.22 (m, 1H), 6.91 (d, *J* = 9.0 Hz, 1H), 6.30 (dd, *J* = 10.0, 1.5 Hz, 1H), 6.18 (d, *J* = 10.0 Hz, 1H), 4.74 – 4.61 (m, 2H), 3.79 – 3.72 (m, 1H), 3.27 (dd, *J* = 18.0, 3.5 Hz, 1H), 2.94 (dd, *J* = 18.0, 2.5 Hz, 1H); ^{13}C NMR (125 MHz, $CDCl_3$) δ 196.4, 148.1, 145.5, 144.9, 144.4,

142.7, 139.9, 131.2, 130.9(4), 130.9(1), 130.8, 130.1, 129.6, 129.3, 129.0, 128.5, 128.0, 127.5, 126.2, 125.2, 124.3(3), 124.3(0) (q, *J* = 3.8 Hz), 123.3, 123.0 (q, *J* = 3.8 Hz), 121.8, 116.3, 112.4, 63.2, 56.0, 55.1, 40.2; IR (neat): 3006, 1681, 1568, 1540, 1507, 1456, 1275, 1260, 1073, 749; HRESIMS Calcd for $[C_{32}H_{22}F_3NNaO]^+$ (M + Na^+) 516.1546, found 516.1551.

(7a*R*,11a*S*)-7-(3-methylbenzyl)-7a,8-dihydrobenzo[*a*]indeno[1,2-*l*]acridin-9(7*H*)-one (2i)

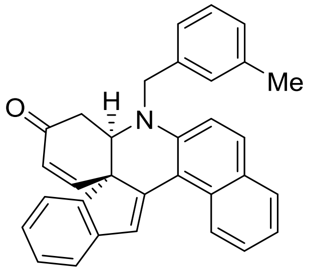

2i

Compound **2i** was prepared in 99% yield (43.5 mg) according to the general procedure (Fig. 3, entry 9). The product was isolated through silica gel column chromatography (PE:EA = 5:1) as a pale yellow solid (mp 229-230 °C). $[\alpha]_D^{20}$ = -0.8 °(c = 1.0, $CHCl_3$). 96% ee (determined by HPLC: Chiralpak ODH Column, 20/80 *i*-PrOH/hexane, 1.0 mL/min, 254 nm; TR = 7.60 min (major), 11.68 min (minor)). ^{1}H NMR (500 MHz, $CDCl_3$) δ 8.44 (d, *J* = 8.5 Hz, 1H), 7.71 (d, *J* = 8.0 Hz, 1H), 7.58 (d, *J* = 9.0 Hz, 1H), 7.54 – 7.49 (m, 2H), 7.46 (d, *J* = 7.5 Hz, 1H), 7.43 – 7.38 (m, 1H), 7.31 (t, *J* = 6.0 Hz, 1H), 7.25 (s, 1H), 7.23 – 7.15 (m, 2H), 7.05 (d, *J* = 7.5 Hz, 1H), 7.02 (d, *J* = 9.0 Hz, 1H), 6.96 (s, 1H), 6.93 (d, *J* = 7.5 Hz, 1H), 6.28 (dd, *J* = 10.0, 2.0 Hz, 1H), 6.16 (d, *J* = 9.5 Hz, 1H), 4.66 – 4.56 (m, 2H), 3.75 – 3.73 (m, 1H), 3.23 (dd, *J* = 18.0, 3.5 Hz, 1H), 3.03 (dd, *J* = 18.0, 2.0 Hz, 1H), 2.29 (s, 3H); ^{13}C NMR (100 MHz, $CDCl_3$) δ 196.8, 148.1, 145.6, 145.2, 144.9, 142.8, 138.5, 138.4, 131.0, 130.7, 129.9, 128.9, 128.6, 128.4, 128.0, 127.7, 127.3, 126.9, 125.7, 125.1, 124.3, 123.2, 122.9, 121.6, 116.8, 111.8, 62.7, 55.9, 55.1, 40.1, 21.4; IR (neat): 3005, 1540, 1507, 1456, 1397, 1338, 1275, 1260, 1219, 1073, 750; HRESIMS Calcd for $[C_{32}H_{25}NNaO]^+$ (M + Na^+) 462.1828, found 462.1832.

(7a*R*,11a*S*)-7-(3-methoxybenzyl)-7a,8-dihydrobenzo[*a*]indeno[1,2-*l*]acridin-9(7*H*)-one (2j)

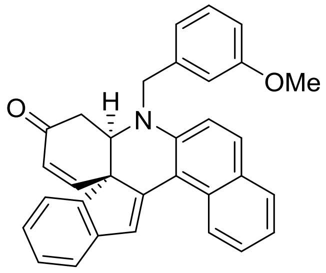

2j

Compound **2j** was prepared in 97% yield (44.2 mg) according to the general procedure (Fig. 3, entry 10). The product was isolated through silica gel column chromatography (PE:EA = 5:1) as a pale yellow oil. $[\alpha]_D^{20}$ = -2.9 °(c = 1.0, $CHCl_3$). 99% ee (determined by HPLC: Chiralpak ODH Column, 20/80 *i*-PrOH/hexane, 1.0 mL/min, 254 nm; TR = 11.49 min (major), 16.99 min (minor)). ^{1}H NMR (500 MHz, $CDCl_3$) δ 8.44 (d, *J* = 8.5 Hz, 1H), 7.71 (d, *J* = 8.0 Hz, 1H), 7.58 (d, *J* = 9.0 Hz, 1H), 7.54 – 7.49 (m, 2H), 7.46 (d, *J* = 7.5 Hz, 1H), 7.41 (t, *J* = 7.5 Hz, 1H), 7.31 (t, *J* = 7.5 Hz, 1H), 7.25 (s, 1H), 7.24 – 7.18 (m, 2H), 7.02 (d, *J* = 9.0 Hz, 1H), 6.79 – 6.76 (m, 1H), 6.73 (d, *J* = 7.5 Hz, 1H), 6.69 (s, 1H), 6.28 (dd, *J* = 10.0, 2.0 Hz, 1H), 6.16 (d, *J* = 10.0 Hz, 1H), 4.66 – 4.57 (m, 2H), 3.77 – 3.73 (m, 1H), 3.70 (s, 3H), 3.25 (dd, *J* = 18.0, 3.0 Hz, 1H), 3.05 (dd, *J* = 18.0, 2.5 Hz, 1H); ^{13}C NMR (125 MHz, $CDCl_3$) δ 196.8, 160.0, 148.2, 145.6, 145.1, 144.9, 142.8, 140.4, 131.0, 130.7, 129.9, 129.7, 128.9, 128.4, 127.8, 127.3, 125.8, 125.1, 124.3, 123.2, 123.0, 121.7, 118.4, 116.8, 113.1, 112.0, 111.6, 62.8, 56.0, 55.1(4), 55.0(7), 40.1; IR (neat): 3005, 2989, 1540, 1456, 1275, 1260, 1219, 1073, 764, 670; HRESIMS Calcd for $[C_{32}H_{25}NNaO_2]^+$ (M + Na^+) 478.1778, found 478.1783.

(7a*R*,11a*S*)-7-(2-fluorobenzyl)-7a,8-dihydrobenzo[*a*]indeno[1,2-*l*]acridin-9(7*H*)-one (2k)

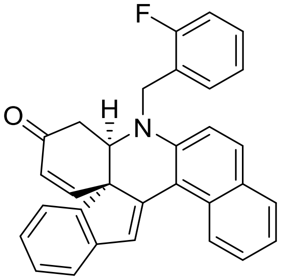

2k

Compound **2k** was prepared in 89% yield (39.5 mg) according to the general procedure (Fig. 3, entry 11). The product was isolated through silica gel column chromatography (PE:EA = 7:1) as a pale yellow oil. $[\alpha]_D^{20}$ = -1.3 °(c = 1.0, $CHCl_3$). 96% ee (determined by HPLC: Chiralpak ODH Column, 20/80 *i*-PrOH/hexane, 1.0 mL/min, 254 nm; TR = 10.88 min (major), 12.51 min (minor)).

^{1}H NMR (500 MHz, $CDCl_3$) δ 8.44 (d, *J* = 8.5 Hz, 1H), 7.71 (d, *J* = 9.0 Hz, 1H), 7.59 (d, *J* = 9.0 Hz, 1H), 7.54 – 7.48 (m, 2H), 7.46 (d, *J* = 7.5 Hz, 1H), 7.41 (t, *J* = 7.5 Hz, 1H), 7.31 (t, *J* = 7.5 Hz, 1H), 7.26 (s, 1H), 7.24 – 7.20 (m, 2H), 7.10 – 7.00 (m, 3H), 6.97 (d, *J* = 9.5 Hz, 1H), 6.28 (dd, *J* = 9.5, 1.5 Hz, 1H), 6.16 (d, *J* = 10.0 Hz, 1H), 4.67 (s, 2H), 3.78 – 3.71 (m, 1H), 3.27 (dd, *J* = 17.5, 3.0 Hz, 1H), 3.02 (dd, *J* = 18.0, 2.0 Hz, 1H); ^{13}C NMR (125 MHz, $CDCl_3$) δ 196.7, 159.8 (d, *J* = 242.5 Hz), 148.2, 145.6, 144.9, 144.5, 142.7, 130.9, 130.7, 129.9, 129.0, 128.9, 128.6 (d, *J* = 3.8 Hz), 128.4, 127.9, 127.3, 126.0, 125.6 (d, *J* = 13.8 Hz), 125.2, 124.2(9), 124.2(7), 123.2, 123.1, 121.7, 116.5, 115.1 (d, *J* = 21.3 Hz), 112.3, 63.0, 55.1, 50.0, 40.1; IR (neat): 3005, 2989, 1540, 1456, 1275, 1260, 1219, 1073, 750, 667; HRESIMS Calcd for $[C_{31}H_{22}FNNaO]^+$ (M + Na^+) 466.1578, found 466.1571.

(7a*R*,11a*S*)-7-(2-bromobenzyl)-7a,8-dihydrobenzo[*a*]indeno[1,2-*l*]acridin-9(7*H*)-one (2l)

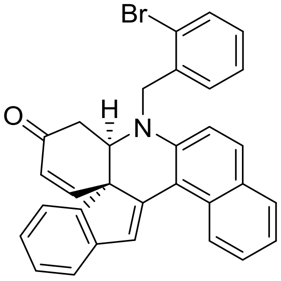

2l

Compound **2l** was prepared in 78% yield (39.3 mg) according to the general procedure (Fig. 3, entry 12). The product was isolated through silica gel column chromatography (PE:EA = 7:1) as a pale yellow solid (mp 151-152 ºC). $[\alpha]_D^{20}$ = -2.1 º(c = 1.0, $CHCl_3$). 97% ee (determined by HPLC: Chiralpak ODH Column, 20/80 *i*-PrOH/hexane, 1.0 mL/min, 254 nm; TR = 6.95 min (major), 9.80 min (minor)). ^{1}H NMR (500 MHz, $CDCl_3$) δ 8.46 (d, *J* = 8.5 Hz, 1H), 7.71 (d, *J* = 8.0 Hz, 1H), 7.59 – 7.56 (m, 2H), 7.55 – 7.51 (m, 2H), 7.46 (d, *J* = 7.5 Hz, 1H), 7.44 – 7.41 (m, 1H), 7.34 – 7.30 (m, 1H), 7.28 (s, 1H), 7.25 – 7.20 (m, 2H), 7.18 – 7.12 (m, 2H), 6.83 (d, *J* = 9.5 Hz, 1H), 6.32 (dd, *J* = 10.0, 1.5 Hz, 1H), 6.19 (d, *J* = 10.0 Hz, 1H), 4.64 – 4.53 (m, 2H), 3.83 – 3.81 (m, 1H), 3.26 (dd, *J* = 18.0, 3.5 Hz, 1H), 2.91 (dd, *J* = 18.0, 2.0 Hz, 1H); ^{13}C NMR (125 MHz, $CDCl_3$) δ 196.7, 148.4, 145.5, 144.9, 144.5, 142.7, 137.3, 132.7, 130.9, 130.8, 130.0, 129.0(1), 128.9(7), 128.7, 128.4, 127.9, 127.7, 127.4, 126.1, 125.2, 124.3, 123.3, 123.1, 121.8, 121.7, 116.3, 112.0, 63.5, 57.8, 55.0, 40.1; IR (neat): 3005, 2989, 1540, 1456, 1275, 1260, 1065, 751, 667; HRESIMS Calcd for $[C_{31}H_{22}BrNNaO]^+$ (M + Na^+) 526.0777, found 526.0771.

(7a*R*,11a*S*)-7-(2-methylbenzyl)-7a,8-dihydrobenzo[*a*]indeno[1,2-*l*]acridin-9(7*H*)-one (2m)

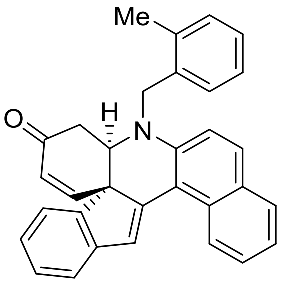

2m

Compound **2m** was prepared in 87% yield (38.2 mg) according to the general procedure (Fig. 3, entry 13). The product was isolated through silica gel column chromatography (PE:EA = 5:1) as a pale yellow oil. $[\alpha]_D^{20}$ = -1.1 °(c = 1.0, $CHCl_3$). 94% ee (determined by HPLC: Chiralpak ODH Column, 20/80 *i*-PrOH/hexane, 1.0 mL/min, 254 nm; TR = 7.40 min (major), 11.09 min (minor)). ^1H NMR (400 MHz, $CDCl_3$) δ 8.45 (d, *J* = 8.8 Hz, 1H), 7.70 (d, *J* = 8.0 Hz, 1H), 7.57 – 7.49 (m, 3H), 7.46 – 7.38 (m, 2H), 7.33 – 7.27 (m, 2H), 7.23 – 7.15 (m, 3H), 7.14 – 7.07 (m, 2H), 6.90 (d, *J* = 9.2 Hz, 1H), 6.31 (dd, *J* = 10.0, 1.6 Hz, 1H), 6.19 (d, *J* = 10.0 Hz, 1H), 4.59 – 4.45 (m, 1H), 3.82 – 3.74 (m, 1H), 3.23 (dd, *J* = 18.0, 3.6 Hz, 1H), 2.94 (dd, *J* = 18.0, 2.0 Hz, 1H), 2.25 (s, 3H); ^{13}C NMR (100 MHz, $CDCl_3$) δ 196.8, 148.3, 145.6, 145.1, 144.9, 142.8, 136.1, 134.2, 130.9, 130.8, 130.2, 129.9, 128.9, 128.4, 127.7, 127.3, 127.2, 126.6, 126.2, 125.8, 125.1, 124.3, 123.2, 122.9, 121.6, 116.6, 111.7, 63.0, 55.1, 54.7, 40.0, 19.0; IR (neat): 3005, 2989, 1540, 1456, 1397, 1275, 1260, 1073, 669; HRESIMS Calcd for $[C_{32}H_{25}NNaO]^+$ ($M + Na^+$) 462.1828, found 462.1831.

(7a*R*,11a*S*)-7-benzyl-13-fluoro-7a,8-dihydrobenzo[*a*]indeno[1,2-*l*]acridin-9(7*H*)-one (2n)

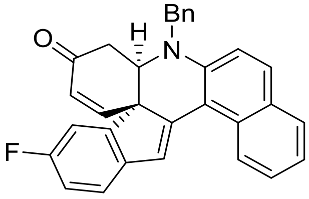

2n

Compound **2n** was prepared in 80% yield (35.5 mg) according to the general procedure (Fig. 3, entry 14). The product was isolated through silica gel column chromatography (PE:EA = 7:1) as a pale yellow oil. $[\alpha]_D^{20}$ = -2.7 °(c = 1.0, $CHCl_3$). 88% ee (determined by HPLC: Chiralpak ODH Column, 20/80 *i*-PrOH/hexane, 1.0 mL/min, 254 nm; TR = 9.48 min (major), 13.91 min (minor)). ^1H NMR (500 MHz, $CDCl_3$) δ 8.41 (d, *J* = 8.5 Hz, 1H), 7.72 (d, *J* = 8.0 Hz, 1H), 7.59 (d, *J* = 9.1 Hz, 1H), 7.52 (t, *J* = 7.0 Hz, 1H), 7.44 – 7.41 (m, 1H), 7.34 – 7.25 (m, 4H), 7.20 (s, 1H), 7.19 –

7.15 (m, 1H), 7.14 – 7.10 (m, 3H), 7.03 (d, *J* = 9.0 Hz, 1H), 6.26 (dd, *J* = 10.0, 2.0 Hz, 1H), 6.17 (d, *J* = 10.0 Hz, 1H), 4.71 – 4.62 (m, 2H), 3.79 – 3.72 (m, 1H), 3.17 (dd, *J* = 17.5, 3.0 Hz, 1H), 3.07 (dd, *J* = 18.0, 2.5 Hz, 1H); ^{13}C NMR (125 MHz, $CDCl_3$) δ 196.3, 160.2, 147.3, 145.1 (d, *J* = 5.0 Hz), 144.8 (d, *J* = 7.5 Hz), 144.7, 141.5 (d, *J* = 2.5 Hz), 138.4, 131.0, 130.9, 130.0, 128.7, 128.5, 127.8, 127.4, 127.3, 126.3, 124.9, 123.1 (d, *J* = 3.8 Hz), 122.2 (d, *J* = 7.5 Hz), 116.8, 115.9 (d, *J* = 22.5 Hz), 112.4, 112.2, 111.9, 62.3, 55.7, 55.2, 39.8; IR (neat): 3005, 2989, 1681, 1540, 1471, 1396, 1275, 1260, 1074, 747, 668; HRESIMS Calcd for $[C_{31}H_{22}FNNaO]^+$ (M + Na^+) 466.1578, found 466.1581.

(7a*R*,11a*S*)-7-benzyl-13-chloro-7a,8-dihydrobenzo[*a*]indeno[1,2-*l*]acridin-9(7*H*)-one (2o)

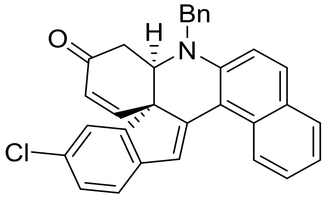

2o

Compound **2o** was prepared in 77% yield (35.4 mg) according to the general procedure (Fig. 3, entry 15). The product was isolated through silica gel column chromatography (PE:EA = 7:1) as a pale yellow solid (mp 218-219 ºC). $[\alpha]_D^{20}$ = -1.3 º(c = 1.0, $CHCl_3$). 91% ee (determined by HPLC: Chiralpak ODH Column, 20/80 *i*-PrOH/hexane, 1.0 mL/min, 254 nm; TR = 9.02 min (major), 15.00 min (minor)). ^{1}H NMR (500 MHz, $CDCl_3$) δ 8.40 (d, *J* = 8.5 Hz, 1H), 7.71 (d, *J* = 7.5 Hz, 1H), 7.59 (d, *J* = 9.0 Hz, 1H), 7.54 – 7.50 (m, 1H), 7.42 – 7.36 (m, 3H), 7.34 – 7.22 (m, 4H), 7.20 (s, 1H), 7.12 (d, *J* = 7.0 Hz, 2H), 7.03 (d, *J* = 9.0 Hz, 1H), 6.23 (dd, *J* = 10.0, 1.5 Hz, 1H), 6.16 (d, *J* = 10.0 Hz, 1H), 4.70 – 4.61 (m, 2H), 3.77 – 3.71 (m, 1H), 3.18 (dd, *J* = 17.5, 3.0 Hz, 1H), 3.07 (dd, *J* = 18.0, 2.5 Hz, 1H); ^{13}C NMR (125 MHz, $CDCl_3$) δ 196.3, 147.0, 145.7, 144.8, 144.5, 144.0, 138.3, 131.1, 131.0, 130.9, 130.2, 129.1, 128.7, 128.5, 127.8, 127.4(2), 127.3(8), 126.3, 124.8(0), 124.7(7), 123.1(1), 123.0(5), 122.3, 116.8, 111.7, 62.2, 55.7, 55.1, 40.0; IR (neat): 3005, 2989, 1540, 1456, 1397, 1275, 1260, 1074, 765, 668; HRESIMS Calcd for $[C_{31}H_{22}ClNNaO]^+$ (M + Na^+) 482.1282, found 482.1292.

(7a*R*,11a*S*)-7-benzyl-13-methoxy-7a,8-dihydrobenzo[*a*]indeno[1,2-*l*]acridin-9(7*H*)-one (2p)

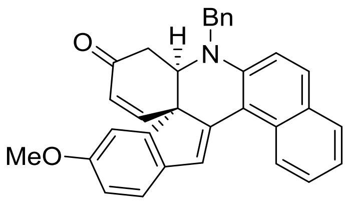

2p

Compound **2p** was prepared in 83% yield (37.8 mg) according to the general procedure (Fig. 3, entry 16). The product was isolated through silica gel column chromatography (PE:EA = 5:1) as a pale yellow oil. $[\alpha]_D^{20}$ = -2.9 °(c = 1.0, $CHCl_3$). 93% ee (determined by HPLC: Chiralpak ADH Column, 20/80 *i*-PrOH/hexane, 1.0 mL/min, 254 nm; TR = 14.51 min (major), 18.11 min (minor)). ^{1}H NMR (500 MHz, $CDCl_3$) δ 8.43 (d, *J* = 8.5 Hz, 1H), 7.71 (d, *J* = 8.0 Hz, 1H), 7.57 (d, *J* = 9.0 Hz, 1H), 7.53 – 7.48 (m, 1H), 7.41 (d, *J* = 8.0 Hz, 1H), 7.33 – 7.24 (m, 2H), 7.23 – 7.21 (m, 1H), 7.18 (s, 1H), 7.13 (d, *J* = 7.0 Hz, 2H), 7.04 – 7.01 (m, 2H), 6.97 – 6.94 (m, 1H), 6.88 – 6.85 (m, 1H), 6.29 (dd, *J* = 10.0, 1.5 Hz, 1H), 6.15 (d, *J* = 10.0 Hz, 1H), 4.70 – 4.61 (m, 2H), 3.82 (s, 3H), 3.78 – 3.75 (m, 1H), 3.25 (dd, *J* = 17.5, 3.0 Hz, 1H), 3.03 (dd, *J* = 18.0, 2.5 Hz, 1H); ^{13}C NMR (125 MHz, $CDCl_3$) δ 197.0, 158.1, 148.6, 144.7, 144.5, 143.0, 138.6, 130.9, 130.6, 129.5, 128.7, 128.4, 127.8, 127.3, 127.2, 126.3, 125.5, 123.2, 122.9, 122.0, 116.8, 115.7, 113.8, 112.4, 111.7, 62.6, 55.8, 55.2, 39.7; IR (neat): 3005, 2989, 1472, 1275, 1260, 1219, 1074, 759, 668; HRESIMS Calcd for $[C_{32}H_{25}NNaO_2]^+$ (M + Na^+) 478.1778, found 478.1771.

(7a*R*,11a*S*)-7-benzyl-14-fluoro-7a,8-dihydrobenzo[*a*]indeno[1,2-*l*]acridin-9(7*H*)-one (2q)

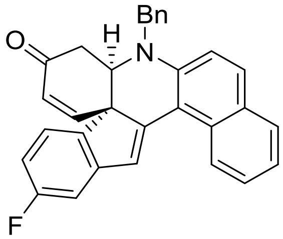

2q

Compound **2q** was prepared in 99% yield (43.9 mg) according to the general procedure (Fig. 3, entry 17). The product was isolated through silica gel column chromatography (PE:EA = 7:1) as a pale yellow oil. $[\alpha]_D^{20}$ = -2.0 °(c = 1.0, $CHCl_3$). 97% ee (determined by HPLC: Chiralpak ODH Column, 20/80 *i*-PrOH/hexane, 1.0 mL/min, 254 nm; TR = 11.39 min (major), 17.33 min (minor)). ^{1}H NMR (400 MHz, $CDCl_3$) δ 8.40 (d, *J* = 8.0 Hz, 1H), 7.72 (d, *J* = 8.0 Hz, 1H), 7.60 (d, *J* = 9.2 Hz, 1H), 7.55 – 7.50 (m, 1H), 7.39 – 7.25 (m, 5H), 7.20 – 7.11 (m, 4H), 7.03 (d, *J* = 9.2 Hz, 1H), 6.92 – 6.86 (m, 1H), 6.24 (dd, *J* = 10.0, 1.6 Hz, 1H), 6.14 (d, *J* = 10.0 Hz, 1H), 4.67 (s, 2H), 3.75

– 3.72 (m, 1H), 3.19 (dd, *J* = 17.6, 3.2 Hz, 1H), 3.06 (dd, *J* = 18.0, 2.4 Hz, 1H); ^{13}C NMR (100 MHz, $CDCl_3$) δ 196.5, 163.6 (d, *J* = 240.0 Hz), 147.7 (d, *J* = 10.0 Hz), 147.4 (d, *J* = 16.3 Hz), 144.9, 138.3, 138.2, 130.9, 130.7, 130.3, 128.7, 128.5, 127.8, 127.5, 127.4, 126.2, 125.2 (d, *J* = 9.0 Hz), 124.9 (d, *J* = 3.0 Hz), 123.1 (d, *J* = 6.0 Hz), 116.8, 111.8, 111.6 (d, *J* = 4.0 Hz), 109.9, 108.8, 108.5, 62.6, 55.7, 53.4, 40.0; IR (neat): 3005, 2989, 1540, 1471, 1275, 1260, 1219, 1074, 755, 668; HRESIMS Calcd for $[C_{31}H_{22}FNNaO]^+$ (M + Na^+) 466.1578, found 466.1581.

(7a*R*,11a*S*)-7-benzyl-14-methyl-7a,8-dihydrobenzo[*a*]indeno[1,2-*l*]acridin-9(7*H*)-one (2r)

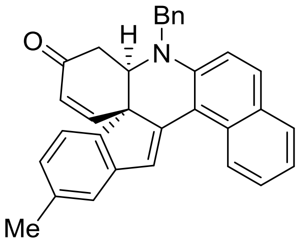

2r

Compound **2r** was prepared in 89% yield (39.1 mg) according to the general procedure (Fig. 3, entry 18). The product was isolated through silica gel column chromatography (PE:EA = 5:1) as a pale yellow solid (mp 205-206 ºC). $[\alpha]_D^{20}$ = -2.8 º(c = 1.0, $CHCl_3$). 97% ee (determined by HPLC: Chiralpak ODH Column, 20/80 *i*-PrOH/hexane, 1.0 mL/min, 254 nm; TR = 7.74 min (major), 9.21 min (minor)). ^{1}H NMR (500 MHz, $CDCl_3$) δ 8.43 (d, *J* = 8.5 Hz, 1H), 7.70 (d, *J* = 8.0 Hz, 1H), 7.56 (d, *J* = 9.0 Hz, 1H), 7.52 – 7.48 (m, 1H), 7.39 (d, *J* = 7.5 Hz, 1H), 7.32 – 7.24 (m, 4H), 7.23 – 7.19 (m, 3H), 7.13 (d, *J* = 7.0 Hz, 2H), 7.01 (d, *J* = 9.0 Hz, 1H), 6.27 (dd, *J* = 10.0, 2.0 Hz, 1H), 6.15 (d, *J* = 10.0 Hz, 1H), 4.64 (s, 2H), 3.74 – 3.72 (m, 1H), 3.26 (dd, *J* = 17.5, 3.0 Hz, 1H), 3.03 (dd, *J* = 18.0, 2.5 Hz, 1H), 2.39 (s, 3H); ^{13}C NMR (125 MHz, $CDCl_3$) δ 197.0, 148.4, 144.6, 144.1, 143.1, 143.0, 138.6, 135.0, 131.0, 130.6, 129.7, 129.6, 128.7, 128.4, 127.8, 127.3, 127.2, 126.2, 125.7, 125.2, 123.2, 122.9, 121.3, 116.8, 112.2, 62.7, 55.7, 55.0, 40.1, 21.5; IR (neat): 3005, 2989, 1472, 1456, 1275, 1260, 1218, 1074, 745, 668; HRESIMS Calcd for $[C_{32}H_{25}NNaO]^+$ (M + Na^+) 462.1828, found 462.1832.

(7a*R*,11a*R*)-7-benzyl-7a,8,12,13-tetrahydrobenzo[*a*]cyclopenta[*l*]acridin-9(7*H*)-one (2s)

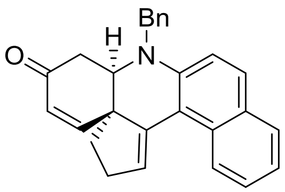

2s

Compound **2s** was prepared in 89% yield (33.6 mg) according to the general procedure (Fig. 3, entry 19). The product was isolated through silica gel column chromatography (PE:EA = 5:1) as a pale yellow oil. $[\alpha]_D^{20}$ = -8.2 °(c = 1.0, $CHCl_3$). 80% ee (determined by HPLC: Chiralpak ADH Column, 20/80 *i*-PrOH/hexane, 1.0 mL/min, 254 nm; TR = 7.30 min (major), 8.33 min (minor)). ^{1}H NMR (500 MHz, $CDCl_3$) δ 8.33 (d, *J* = 9.0 Hz, 1H), 7.65 (d, *J* = 8.0 Hz, 1H), 7.50 (d, *J* = 9.0 Hz, 1H), 7.46 – 7.42 (m, 1H), 7.30 – 7.22 (m, 4H), 7.13 (d, *J* = 7.0 Hz, 2H), 6.96 (d, *J* = 9.5 Hz, 1H), 6.72 – 6.68 (m, 1H), 6.26 (t, *J* = 2.5 Hz, 1H), 5.85 (d, *J* = 10.0 Hz, 1H), 4.77 (d, *J* = 18.0 Hz, 1H), 4.62 (d, *J* = 18.0 Hz, 1H), 3.85 (d, *J* = 2.0 Hz, 1H), 2.98 – 2.90 (m, 1H), 2.79 (d, *J* = 3.0 Hz, 2H), 2.71 – 2.63 (m, 1H), 2.23 – 2.19 (m, 2H); ^{13}C NMR (125 MHz, $CDCl_3$) δ 197.0, 154.2, 144.8, 138.6, 138.0, 131.5, 129.1, 128.6, 128.2, 127.8, 127.7, 127.1, 126.8, 126.5, 126.4, 123.3, 122.5, 116.6, 112.2, 64.9, 54.0, 52.5, 38.8, 34.5, 32.2; IR (neat): 3005, 2989, 1540, 1456, 1275, 1260, 1219, 1073, 746, 668; HRESIMS Calcd for $[C_{27}H_{23}NNaO]^+$ (M + Na^+) 400.1672, found 400.1678.

(7a*R*,11a*S*)-7-benzyl-3-bromo-7a,8-dihydrobenzo[*a*]indeno[1,2-*l*]acridin-9(7*H*)-one (2t)

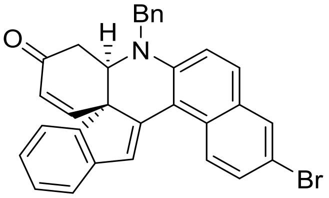

2t

Compound **2t** was prepared in 60% yield (30.3 mg) according to the general procedure (Fig. 3, entry 20). The product was isolated through silica gel column chromatography (PE:EA = 7:1) as a pale yellow oil. $[\alpha]_D^{20}$ = -0.4 °(c = 1.0, $CHCl_3$). 94% ee (determined by HPLC: Chiralpak ADH Column, 30/70 *i*-PrOH/hexane, 1.0 mL/min, 254 nm; TR = 13.67 min (major), 17.79 min (minor)). ^{1}H NMR (500 MHz, $CDCl_3$) δ 8.29 (d, *J* = 9.0 Hz, 1H), 7.85 (d, *J* = 2.0 Hz, 1H), 7.58 – 7.54 (m, 1H), 7.53 – 7.45 (m, 3H), 7.42 (t, *J* = 7.5 Hz, 1H), 7.31 – 7.20 (m, 5H), 7.13 (d, *J* = 7.0 Hz, 2H), 7.04 (d, *J* = 9.0 Hz, 1H), 6.26 (dd, *J* = 10.0, 1.5 Hz, 1H), 6.17 (d, *J* = 10.0 Hz, 1H), 4.66 (s, 2H), 3.79 – 3.72 (m, 1H), 3.26 (dd, *J* = 18.0, 3.0 Hz, 1H), 3.05 (dd, *J* = 18.0, 2.0 Hz, 1H); ^{13}C NMR (125 MHz, $CDCl_3$) δ 196.6, 147.8, 145.4, 144.9, 144.6, 142.8, 138.2, 130.8, 130.4, 130.3, 129.6,

129.0, 128.8(2), 128.7(6), 127.4, 126.2, 125.3, 125.1, 124.3, 121.8, 117.8, 116.4, 112.1, 62.7, 55.6, 55.0, 40.0; IR (neat): 3005, 2989, 1540, 1456, 1275, 1260, 1219, 1073, 766, 668; HRESIMS Calcd for $[C_{31}H_{22}BrNNaO]^+$ (M + Na^+) 526.0777, found 526.0771.

(7a*R*,11a*S*)-7-benzyl-3-methyl-7a,8-dihydrobenzo[*a*]indeno[1,2-*l*]acridin-9(7*H*)-one (2u)

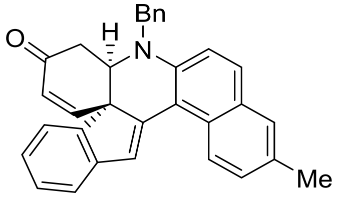

2u

Compound **2u** was prepared in 88% yield (38.7 mg) according to the general procedure (Fig. 3, entry 21). The product was isolated through silica gel column chromatography (PE:EA = 5:1) as a pale yellow solid (mp 201-202 ºC). $[\alpha]_D^{20}$ = -2.1 º(c = 1.0, $CHCl_3$). 93% ee (determined by HPLC: Chiralpak ADH Column, 20/80 *i*-PrOH/hexane, 1.0 mL/min, 254 nm; TR = 14.18 min (minor), 18.31 min (major)). ^{1}H NMR (500 MHz, $CDCl_3$) δ 8.33 (d, *J* = 8.5 Hz, 1H), 7.51 – 7.47 (m, 3H), 7.44 (d, *J* = 8.0 Hz, 1H), 7.41 – 7.37 (m, 1H), 7.36 – 7.34 (m, 1H), 7.29 – 7.18 (m, 5H), 7.12 (d, *J* = 7.0 Hz, 2H), 6.98 (d, *J* = 9.5 Hz, 1H), 6.25 (dd, *J* = 10.0, 2.0 Hz, 1H), 6.14 (d, *J* = 10.0 Hz, 1H), 4.62 (s, 2H), 3.74 – 3.68 (m, 1H), 3.23 (dd, *J* = 17.5, 3.0 Hz, 1H), 3.03 (dd, *J* = 17.5, 2.5 Hz, 1H), 2.47 (s, 3H); ^{13}C NMR (125 MHz, $CDCl_3$) δ 196.9, 148.0, 145.6, 145.3, 144.2, 142.8, 138.7, 132.5, 130.7, 129.4, 129.3, 129.0, 128.9, 128.6, 128.0, 127.5, 127.2, 126.3, 125.6, 125.0, 124.3, 123.1, 121.6, 116.8, 112.0, 62.6, 55.8, 55.1, 40.1, 21.2; IR (neat): 3005, 2989, 1540, 1456, 1397, 1275, 1260, 1219, 1073, 762, 668; HRESIMS Calcd for $[C_{32}H_{25}NNaO]^+$ (M + Na^+) 462.1828, found 462.1831.

(7a*R*,11a*S*)-7-benzyl-10,11-dimethyl-7a,8-dihydrobenzo[*a*]indeno[1,2-*l*]acridin-9(7*H*)-one (2v)

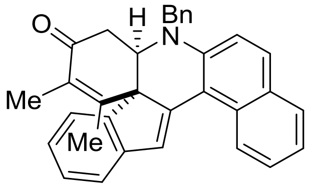

2v

Compound **2v** was prepared in 86% yield (39.0 mg) according to the general procedure (Fig. 3, entry 22). The product was isolated through silica gel column chromatography (PE:EA = 5:1) as a

pale yellow oil. $[\alpha]_D^{20}$ = +2.4 °(c = 1.0, $CHCl_3$). 70% ee (determined by HPLC: Chiralpak ADH Column, 20/80 *i*-PrOH/hexane, 1.0 mL/min, 254 nm; TR = 7.14 min (minor), 10.77 min (major)). ^{1}H NMR (500 MHz, $CDCl_3$) δ 8.42 (d, *J* = 8.5 Hz, 1H), 7.74 (d, *J* = 8.0 Hz, 1H), 7.60 (d, *J* = 9.0 Hz, 1H), 7.56 – 7.48 (m, 2H), 7.43 – 7.38 (m, 2H), 7.35 – 7.32 (m, 1H), 7.29 – 7.24 (m, 4H), 7.22 – 7.20 (m, 1H), 7.12 (d, *J* = 7.0 Hz, 2H), 7.01 (d, *J* = 9.5 Hz, 1H), 4.64 – 4.59 (m, 2H), 3.69 – 3.63 (m, 1H), 3.23 (dd, *J* = 18.0, 3.5 Hz, 1H), 3.06 (dd, *J* = 18.0, 2.0 Hz, 1H), 1.77 (s, 3H), 1.36 (s, 3H); ^{13}C NMR (125 MHz, $CDCl_3$) δ 196.0, 151.3, 146.5, 145.7, 144.8, 144.3, 138.6, 133.5, 130.8, 129.7, 128.6(1), 128.5(8), 128.4, 127.7, 127.3, 127.2, 126.7, 126.2, 124.8, 123.9, 122.8, 121.6, 116.6, 112.2, 62.2, 58.9, 55.1, 39.7, 15.7, 11.2; IR (neat): 3005, 2991, 1478, 1356, 1278, 1260, 1209, 1078, 782, 669; HRESIMS Calcd for $[C_{33}H_{27}NNaO]^+$ (M + Na^+) 476.1985, found 476.1988.

(4b*S*,10a*R*)-11-benzyl-5,6,7,8,10a,11-hexahydrodibenzo[*a*,*j*]indeno[1,2-*l*]acridin-9(10*H*)-one (2w)

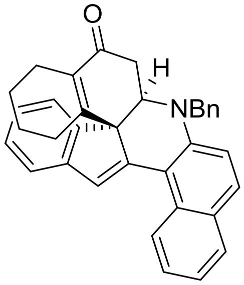

2w

Compound **2w** was prepared in 91% yield (43.6 mg) according to the general procedure (Fig. 3, entry 23). The product was isolated through silica gel column chromatography (PE:EA = 5:1) as a pale yellow solid (mp 245-246 °C). $[\alpha]_D^{20}$ = +0.3 °(c = 1.0, $CHCl_3$). 90% ee (determined by HPLC: Chiralpak ADH Column, 20/80 *i*-PrOH/hexane, 1.0 mL/min, 254 nm; TR = 7.96 min (minor), 12.09 min (major)). ^{1}H NMR (500 MHz, $CDCl_3$) δ 8.37 (d, *J* = 8.5 Hz, 1H), 7.71 (d, *J* = 8.0 Hz, 1H), 7.56 (d, *J* = 9.0 Hz, 1H), 7.51 (t, *J* = 7.0 Hz, 1H), 7.45 (t, *J* = 7.0 Hz, 1H), 7.39 – 7.35 (m, 2H), 7.32 – 7.28 (m, 1H), 7.27 – 7.15 (m, 5H), 7.09 (d, *J* = 6.5 Hz, 2H), 6.97 (d, *J* = 9.0 Hz, 1H), 4.57 – 4.50 (m, 2H), 3.60 (s, 1H), 3.19 (d, *J* = 18.0 Hz, 1H), 2.99 (d, *J* = 17.5 Hz, 1H), 2.33 – 2.28 (m, 1H), 2.24 – 2.11 (m, 1H), 1.78 – 1.73 (m, 1H), 1.58 – 1.55 (m, 1H), 1.46 – 1.30 (m, 2H), 1.21 – 1.15 (m, 1H), 1.10 – 1.01 (m, 1H); ^{13}C NMR (125 MHz, $CDCl_3$) δ 196.1, 153.9, 145.9, 145.6, 144.7, 144.3, 138.6, 134.3, 130.7, 129.6, 128.6, 128.5, 128.4, 127.7, 127.2(2), 127.1(8), 126.9, 126.1, 124.7, 123.8, 122.8, 122.7, 121.5, 116.5, 112.2, 62.6, 58.2, 55.3, 39.8, 25.4, 22.5, 21.9, 21.4;

IR (neat): 3005, 2989, 1540, 1456, 1275, 1260, 1219, 1073, 768, 668; HRESIMS Calcd for $[C_{35}H_{29}NNaO]^+$ (M + Na^+) 502.2141, found 502.2146.

(4b*R*,10a*R*)-11-benzyl-10a,11-dihydrodibenzo[*a,j*]indeno[1,2-*l*]acridin-9(10*H*)-one (2x)

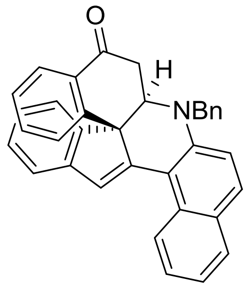

2x

Compound **2x** was prepared in 91% yield (43.3 mg) according to the general procedure (Fig. 3, entry 24). The product was isolated through silica gel column chromatography (PE:EA = 5:1) as a pale yellow oil. $[\alpha]_D^{20}$ = +3.3 °(c = 1.0, $CHCl_3$). 97% (determined by HPLC: Chiralpak ADH Column, 20/80 *i*-PrOH/hexane, 1.0 mL/min, 254 nm; TR = 12.01 min (major), 16.53 min (minor)). ^{1}H NMR (400 MHz, $CDCl_3$) δ 8.57 (d, *J* = 8.8 Hz, 1H), 8.02 (d, *J* = 7.2 Hz, 1H), 7.66 (d, *J* = 8.0 Hz, 1H), 7.57 (t, *J* = 7.6 Hz, 1H), 7.51 – 7.45 (m, 2H), 7.36 – 7.30 (m, 3H), 7.29 – 7.19 (m, 3H), 7.18 – 7.02 (m, 6H), 6.88 – 6.84 (m, 2H), 4.70 – 4.58 (m, 2H), 3.83 (s, 1H), 3.48 (dd, *J* = 18.4, 2.4 Hz, 1H), 3.23 (d, *J* = 18.4 Hz, 1H); ^{13}C NMR (125 MHz, $CDCl_3$) δ 196.0, 146.9, 146.4, 144.4, 144.3, 141.6, 138.4, 134.8, 132.4, 131.2, 129.6, 128.6, 128.4, 128.2, 127.8, 127.5, 127.3, 126.9, 126.6, 126.4, 126.2, 124.9, 124.5, 123.0, 122.8, 121.9, 116.4, 112.5, 63.2, 56.3, 55.1, 40.5; IR (neat): 3005, 2989, 1576, 1436, 1328, 1262, 1222, 1072, 796, 669; HRESIMS Calcd for $[C_{35}H_{25}NNaO]^+$ (M + Na^+) 498.1828, found 498.1832.

(7a*R*,11a*S*)-7-benzyl-7a,8-dihydrobenzo[*a*]indeno[1,2-*l*]acridin-9(7*H*)-one (2a′)

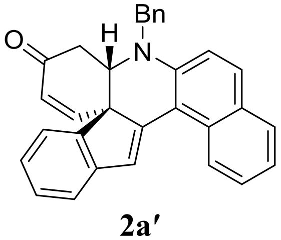

2a′

Compound **2a′** was prepared in 88% yield (37.4 mg) according to the general procedure (Fig. 3, entry 25). $[\alpha]_D^{20}$ = +5.1 °(c = 1.0, $CHCl_3$). 91% ee (determined by HPLC: Chiralpak ADH Column, 20/80 i-PrOH/hexane, 1.0 mL/min, 254 nm; TR = 10.89 min (minor), 14.76 min (major)).

(7a*R*,12b*R*)-7-benzyl-7a,8,13,14-tetrahydro-7*H*-benzo[*f*]cyclopenta[*c*]indolo[2,3-*b*]quinoline (4a)

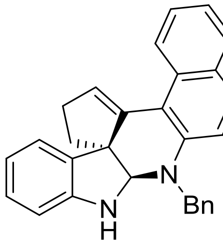

4a

Compound **4a** was prepared in 92% yield (36.8 mg) according to the general procedure (Fig. 4, entry 1). The product was isolated through silica gel column chromatography (PE:EA = 7:1) as a pale yellow oil. $[\alpha]_D^{20}$ = -2.7 ° (c = 1.0, $CHCl_3$). 92% ee (determined by HPLC: Chiralpak IG Column, 10/90 *i*-PrOH/hexane, 1.0 mL/min, 254 nm; TR = 7.03 min (minor), 9.21 min (major)). ^{1}H NMR (500 MHz, $CDCl_3$) δ 8.28 (d, *J* = 8.5 Hz, 1H), 7.58 (d, *J* = 8.0 Hz, 1H), 7.44 (d, *J* = 9.0 Hz, 1H), 7.37 (t, *J* = 8.0 Hz, 1H), 7.31 – 7.28 (m, 4H), 7.27 – 7.25 (m, 1H), 7.18 (t, *J* = 7.5 Hz, 1H), 7.06 (d, *J* = 7.0 Hz, 1H), 6.90 – 6.85 (m, 2H), 6.58 (t, *J* = 7.0 Hz, 1H), 6.38 – 6.35 (m, 2H), 5.03 (s, 1H), 4.73 (d, *J* = 16.0 Hz, 1H), 4.57 (d, *J* = 16.0 Hz, 1H), 3.07 – 2.99 (m, 1H), 2.73 – 2.66 (m, 1H), 2.40 – 2.36 (m, 2H); ^{13}C NMR (125 MHz, $CDCl_3$) δ 148.3, 143.0, 138.5, 137.9, 135.0, 131.5, 128.7, 128.2, 128.1, 128.0, 127.7, 127.2(4), 127.1(8), 126.9, 126.3, 123.8, 122.6, 122.4, 119.3, 115.9, 109.2, 86.1, 65.0, 53.7, 41.2, 32.0; IR (neat): 3392(br), 2921, 1605, 1512, 1347, 1228, 1019, 758, 746, 687; HRESIMS Calcd for $[C_{29}H_{24}N_2Na]^+$ (M + Na^+) 423.1832, found 423.1833.

(7a*R*,12b*R*)-7-(4-methylbenzyl)-7a,8,13,14-tetrahydro-7*H*-benzo[*f*]cyclopenta[*c*]indolo[2,3-*b*]quinoline (4b)

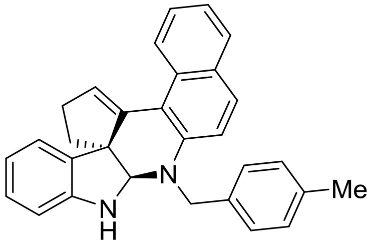

4b

Compound **4b** was prepared in 80% yield (33.2 mg) according to the general procedure (Fig. 4, entry 2). The product was isolated through silica gel column chromatography (PE:EA = 7:1) as a pale yellow solid (mp 124-125 °C). $[\alpha]_D^{20}$ = -5.3 °(c = 1.0, $CHCl_3$). 96% ee (determined by HPLC:

Chiralpak IG Column, 20/80 *i*-PrOH/hexane, 1.0 mL/min, 254 nm; TR = 5.58 min (minor), 6.71 min (major)). ^{1}H NMR (500 MHz, $CDCl_3$) δ 8.28 (d, *J* = 8.5 Hz, 1H), 7.58 (d, *J* = 8.0 Hz, 1H), 7.44 (d, *J* = 9.0 Hz, 1H), 7.38 – 7.34 (m, 1H), 7.20 – 7.15 (m, 3H), 7.11 (d, *J* = 8.0 Hz, 2H), 7.05 (d, *J* = 6.5 Hz, 1H), 6.91 – 6.85 (m, 2H), 6.60 – 6.56 (m, 1H), 6.38 – 6.35 (m, 2H), 5.01 (s, 1H), 4.70 (d, *J* = 16.0 Hz, 1H), 4.55 (d, *J* = 16.0 Hz, 1H), 4.28 (s, 1H), 3.07 – 2.99 (m, 1H), 2.75 – 2.68 (m, 1H), 2.39 – 2.35 (m, 2H), 2.33 (s, 3H); ^{13}C NMR (125 MHz, $CDCl_3$) δ 148.4, 143.2, 138.0, 136.9, 135.5, 135.0, 131.5, 129.4, 128.2, 128.0, 127.7, 127.1, 126.8, 126.3, 123.8, 122.6, 122.3, 119.3, 115.9, 115.6, 109.1, 85.9, 65.0, 53.4, 41.2, 32.0, 21.1; IR (neat): 3390(br), 2243, 1634, 1508, 1482, 1267, 1095, 805, 760, 645; HRESIMS Calcd for $[C_{30}H_{26}N_2Na]^+$ (M + Na^+) 437.1988, found 437.1979.

(7a*R*,12b*R*)-7-(4-methoxybenzyl)-7a,8,13,14-tetrahydro-7*H*-benzo[*f*]cyclopenta[*c*]indolo[2,3-*b*]quinoline (4c)

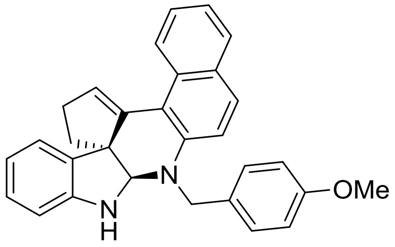

4c

Compound **4c** was prepared in 96% yield (41.3 mg) according to the general procedure (Fig. 4, entry 3). The product was isolated through silica gel column chromatography (PE:EA = 7:1) as a pale yellow oil. $[\alpha]_D^{20}$ = -20.3 °(c = 1.0, $CHCl_3$). 91% ee (determined by HPLC: Chiralpak IG Column, 20/80 *i*-PrOH/hexane, 1.0 mL/min, 254 nm; TR = 7.02 min (minor), 8.65 min (major)). ^{1}H NMR (500 MHz, $CDCl_3$) δ 8.27 (d, *J* = 8.5 Hz, 1H), 7.58 (d, *J* = 8.0 Hz, 1H), 7.45 (d, *J* = 8.5 Hz, 1H), 7.39 – 7.35 (m, 1H), 7.23 – 7.16 (m, 3H), 7.05 (d, *J* = 7.0 Hz, 1H), 6.92 (d, *J* = 9.0 Hz, 1H), 6.89 – 6.82 (m, 3H), 6.60 – 6.56 (m, 1H), 6.38 – 6.35 (m, 2H), 5.03 (s, 1H), 4.69 (d, *J* = 16.0 Hz, 1H), 4.51 (d, *J* = 16.0 Hz, 1H), 3.77 (s, 3H), 3.07 – 2.99 (m, 1H), 2.73 – 2.68 (m, 1H), 2.41 – 2.32 (m, 2H); ^{13}C NMR (125 MHz, $CDCl_3$) δ 158.9, 148.3, 138.1, 134.9, 134.3, 134.2, 132.0, 131.5, 128.4, 128.2, 128.0, 127.7, 126.8, 126.3, 123.8, 122.7, 122.5, 119.3, 116.1, 114.1, 109.2, 85.8, 65.1, 55.3, 53.1, 41.3, 32.1; IR (neat): 3278(br), 2561, 1780, 1563, 1340, 1217, 1052, 763, 724, 673; HRESIMS Calcd for $[C_{30}H_{26}N_2NaO]^+$ (M + Na^+) 453.1937, found 453.1941.

(7a*R*,12b*R*)-7-(4-fluorobenzyl)-7a,8,13,14-tetrahydro-7*H*-benzo[*f*]cyclopenta[*c*]indolo[2,3-*b*]quinoline (4d)

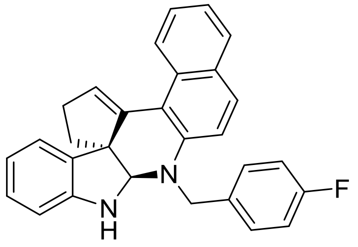

4d

Compound **4d** was prepared in 76% yield (31.8 mg) according to the general procedure (Fig. 4, entry 4). The product was isolated through silica gel column chromatography (PE:EA = 10:1) as a pale yellow solid (mp 154-155 ºC). $[\alpha]_D^{20}$ = -10.9 º(c = 1.0, $CHCl_3$). 82% ee (determined by HPLC: Chiralpak ADH Column, 10/90 *i*-PrOH/hexane, 1.0 mL/min, 254 nm; TR = 9.42 min (minor), 15.90 min (major)). ^{1}H NMR (500 MHz, $CDCl_3$) δ 8.27 (d, *J* = 9.0 Hz, 1H), 7.59 (d, *J* = 8.0 Hz, 1H), 7.44 (d, *J* = 9.0 Hz, 1H), 7.40 – 7.35 (m, 1H), 7.28 – 7.24 (m, 2H), 7.21 – 7.18 (m, 1H), 7.08 – 7.05 (m, 1H), 7.02 – 6.98 (m, 2H), 6.90 – 6.84 (m, 2H), 6.61 – 6.57 (m, 1H), 6.39 – 6.35 (m, 2H), 5.03 (s, 1H), 4.71 (d, *J* = 16.0 Hz, 1H), 4.52 (d, *J* = 16.0 Hz, 1H), 4.11 (s, 1H), 3.09 – 2.99 (m, 1H), 2.74 – 2.71 (m, 1H), 2.44 – 2.33 (m, 2H); ^{13}C NMR (125 MHz, $CDCl_3$) δ 162.0 (d, *J* = 243.8 Hz), 148.3, 142.7, 138.1, 134.9, 134.1, 131.5, 128.7 (d, *J* = 7.5 Hz), 128.1, 128.0, 127.7, 126.9, 126.4, 123.8, 122.7, 122.6, 119.4, 115.9, 115.5 (d, *J* = 21.3 Hz), 109.2, 86.0, 65.3, 52.9, 41.3, 32.0; IR (neat): 3358(br), 1603, 1507, 1218, 1097, 1018, 811, 763, 759, 480; HRESIMS Calcd for $[C_{29}H_{23}FN_2Na]^+$ (M + Na^+) 441.1737, found 441.1730.

(7a*R*,12b*R*)-7-(3-methylbenzyl)-7a,8,13,14-tetrahydro-7*H*-benzo[*f*]cyclopenta[*c*]indolo[2,3-*b*]quinoline (4e)

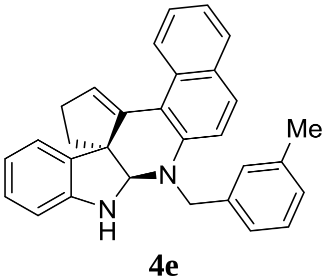

4e

Compound **4e** was prepared in 79% yield (32.7 mg) according to the general procedure (Fig. 4, entry 5). The product was isolated through silica gel column chromatography (PE:EA = 7:1) as a pale yellow oil. $[\alpha]_D^{20}$ = -5.4 º (c = 1.0, $CHCl_3$). 90% ee (determined by HPLC: Chiralpak IG

Column, 20/80 *i*-PrOH/hexane, 1.0 mL/min, 254 nm; TR = 5.20 min (minor), 6.60 min (major)). ^{1}H NMR (500 MHz, $CDCl_3$) δ 8.30 (d, *J* = 8.5 Hz, 1H), 7.59 (d, *J* = 8.0 Hz, 1H), 7.44 (d, *J* = 9.0 Hz, 1H), 7.40 – 7.35 (m, 1H), 7.22 – 7.16 (m, 2H), 7.10 – 7.04 (m, 4H), 6.90 – 6.86 (m, 2H), 6.61 – 6.57 (m, 1H), 6.40 – 6.35 (m, 2H), 5.02 (s, 1H), 4.68 (d, *J* = 16.0 Hz, 1H), 4.58 (d, *J* = 16.0 Hz, 1H), 4.29 (s, 1H), 3.07 – 2.99 (m, 1H), 2.76 – 2.71 (m, 1H), 2.43 – 2.36 (m, 2H), 2.29 (s, 3H); ^{13}C NMR (125 MHz, $CDCl_3$) δ 148.3, 138.4, 137.7, 135.1, 131.6, 128.6, 128.2, 128.0(3), 128.0(1), 127.8, 127.7, 127.1, 126.4, 124.1(6), 124.1(2), 123.8, 122.6, 122.3, 119.3, 115.8, 109.3, 86.1, 64.8, 53.8, 41.0, 32.0, 21.4; IR (neat): 3363(br), 1606, 1511, 1260, 1094, 763, 684, 597, 484, 444; HRESIMS Calcd for $[C_{30}H_{26}N_2Na]^+$ (M + Na^+) 437.1988, found 437.1981.

methyl 3-(((7a*R*,12b*R*)-7a,8,13,14-tetrahydro-7*H*-benzo[*f*]cyclopenta[*c*]indolo[2,3-*b*]quinolin-7-yl)methyl)benzoate (4f)

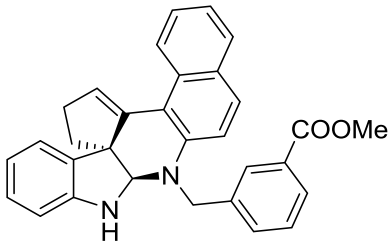

4f

Compound **4f** was prepared in 99% yield (45.4 mg) according to the general procedure (Fig. 4, entry 6). The product was isolated through silica gel column chromatography (PE:EA = 7:1) as a pale yellow oil. $[\alpha]_D^{20}$ = -0.2 °(c = 1.0, $CHCl_3$). 95% ee (determined by HPLC: Chiralpak ADH Column, 20/80 *i*-PrOH/hexane, 1.0 mL/min, 254 nm; TR = 9.48 min (minor), 19.45 min (major)). ^{1}H NMR (500 MHz, $CDCl_3$) δ 8.28 (d, *J* = 8.5 Hz, 1H), 8.03 (s, 1H), 7.94 (d, *J* = 7.5 Hz, 1H), 7.59 (d, *J* = 8.0 Hz, 1H), 7.50 – 7.44 (m, 2H), 7.40 – 7.34 (m, 2H), 7.20 (t, *J* = 8.0 Hz, 1H), 7.07 (d, *J* = 7.5 Hz, 1H), 6.90 – 6.85 (m, 2H), 6.62 – 6.58 (m, 1H), 6.40 – 6.36 (m, 2H), 5.04 (s, 1H), 4.82 (d, *J* = 16.0 Hz, 1H), 4.60 (d, *J* = 16.0 Hz, 1H), 4.36 (s, 1H), 3.90 (s, 3H), 3.09 – 3.01 (m, 1H), 2.78 – 2.71 (m, 1H), 2.44 – 2.37 (m, 2H); ^{13}C NMR (125 MHz, $CDCl_3$) δ 166.9, 148.2, 142.6, 139.1, 138.1, 134.9, 131.8, 131.5, 130.6, 128.8, 128.6, 128.3, 128.2, 128.0, 127.7, 127.0, 126.4, 123.9, 122.7, 122.6, 119.5, 115.9, 109.2, 86.1, 65.4, 53.3, 52.1, 41.3, 32.1; IR (neat): 3332(br), 1274, 1261, 1092, 803, 765, 742, 654, 605, 492; HRESIMS Calcd for $[C_{31}H_{26}N_2NaO_2]^+$ (M + Na^+) 481.1886, found 481.1891.

(7a*R*,12b*R*)-7-(3-(trifluoromethyl)benzyl)-7a,8,13,14-tetrahydro-7*H*-benzo[*f*]cyclopenta[*c*]indolo[2,3-*b*]quinoline (4g)

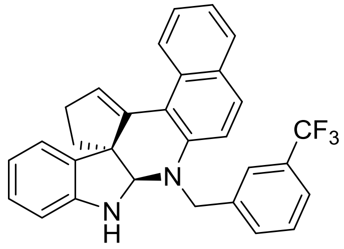

4g

Compound **4g** was prepared in 88% yield (41.2 mg) according to the general procedure (Fig. 4, entry 7). The product was isolated through silica gel column chromatography (PE:EA = 7:1) as a pale yellow oil. $[\alpha]_D^{20}$ = -4.2 ° (c = 1.0, $CHCl_3$). 88% ee (determined by HPLC: Chiralpak IG Column, 20/80 *i*-PrOH/hexane, 1.0 mL/min, 254 nm; TR = 4.40 min (minor), 4.97 min (major)). ^{1}H NMR (500 MHz, $CDCl_3$) δ 8.28 (d, *J* = 8.5 Hz, 1H), 7.60 – 7.58 (m, 2H), 7.51 (d, *J* = 8.0 Hz, 1H), 7.46 – 7.43 (m, 2H), 7.40 – 7.36 (m, 2H), 7.20 (t, *J* = 8.0 Hz, 1H), 7.08 – 7.06 (m, 1H), 6.90 – 6.85 (m, 1H), 6.82 (d, *J* = 9.0 Hz, 1H), 6.62 – 6.58 (m, 1H), 6.42 – 6.36 (m, 2H), 5.02 (s, 1H), 4.79 (d, *J* = 16.5 Hz, 1H), 4.57 (d, *J* = 16.5 Hz, 1H), 3.94 (s, 1H), 3.08 – 3.00 (m, 1H), 2.76 – 2.70 (m, 1H), 2.43 – 2.33 (m, 2H); ^{13}C NMR (125 MHz, $CDCl_3$) δ 148.1, 142.5, 139.9, 138.0, 134.9, 131.5, 131.1, 130.9, 130.5, 129.1, 128.2(6), 128.2(3), 128.0, 127.8, 127.1, 126.5, 125.1, 124.1 (q, *J* = 3.8 Hz), 123.9, 123.8 (d, *J* = 3.8 Hz), 122.7, 119.6, 115.8, 109.3, 86.1, 65.4, 53.2, 41.3, 32.1; IR (neat): 3398(br), 1512, 1328, 1275, 1262, 1164, 1093, 801, 759, 592; HRESIMS Calcd for $[C_{30}H_{23}F_3N_2Na]^+$ (M + Na^+) 491.1706, found 491.1711.

(7a*R*,12b*R*)-7-(2-methylbenzyl)-7a,8,13,14-tetrahydro-7*H*-benzo[*f*]cyclopenta[*c*]indolo[2,3-*b*]quinoline (4h)

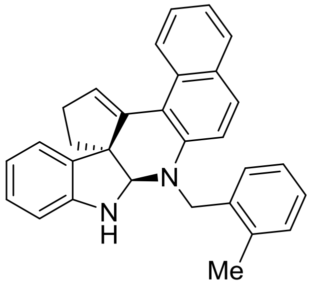

4h

Compound **4h** was prepared in 82% yield (33.9 mg) according to the general procedure (Fig. 4, entry 8). The product was isolated through silica gel column chromatography (PE:EA = 7:1) as a

pale yellow solid (mp 150-151 ºC). $[\alpha]_D^{20}$ = -23.9 º(c = 1.0, $CHCl_3$). 93% ee(determined by HPLC: Chiralpak IG Column, 20/80 *i*-PrOH/hexane, 1.0 mL/min, 254 nm; TR = 5.03 min (minor), 5.72 min (major)). ^{1}H NMR (500 MHz, $CDCl_3$) δ 8.30 (d, *J* = 8.5 Hz, 1H), 7.58 (d, *J* = 8.0 Hz, 1H), 7.44 (d, *J* = 9.0 Hz, 1H), 7.39 – 7.35 (m, 1H), 7.19 – 7.17 (m, 3H), 7.13 – 7.08 (m, 1H), 7.07 – 7.04 (m, 2H), 6.91 – 6.86 (m, 1H), 6.81 (d, *J* = 9.0 Hz, 1H), 6.61 – 6.57 (m, 1H), 6.41 – 6.36 (m, 2H), 4.96 (s, 1H), 4.68 (d, *J* = 16.0 Hz, 1H), 4.48 (d, *J* = 16.0 Hz, 1H), 4.27 (s, 1H), 3.06 – 2.98 (m, 1H), 2.74 – 2.69 (m, 1H), 2.35 (s, 3H), 2.34 – 2.31 (m, 2H); ^{13}C NMR (125 MHz, $CDCl_3$) δ 148.4, 143.1, 137.7, 135.9, 135.1, 131.6, 130.5, 128.2, 128.0(3), 127.9(7), 127.7, 127.3, 127.0, 126.3, 126.1, 123.8, 122.6, 122.3, 119.4, 115.7, 115.4, 114.9, 109.3, 85.8, 64.9, 52.1, 41.3, 32.0, 19.2; IR (neat): 3396(br), 1605, 1510, 1275, 1260, 1093, 805, 768, 743, 509; HRESIMS Calcd for $[C_{30}H_{26}N_2Na]^+$ (M + Na^+) 437.1988, found 437.1995.

(7a*R*,12b*R*)-12-methyl-7-(4-methylbenzyl)-7a,8,13,14-tetrahydro-7*H*-benzo[*f*]cyclopenta[*c*]indolo[2,3-*b*]quinoline (4i)

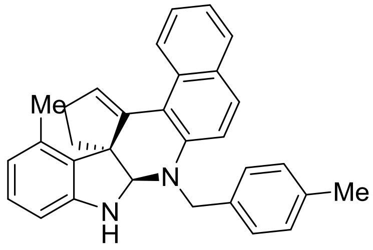

4i

Compound **4i** was prepared in 69% yield (29.6 mg) according to the general procedure (Fig. 4, entry 9). The product was isolated through silica gel column chromatography (PE:EA = 7:1) as a pale yellow oil. $[\alpha]_D^{20}$ = -8.7 º(c = 1.0, $CHCl_3$). 97% ee (determined by HPLC: Chiralpak IG Column, 10/90 *i*-PrOH/hexane, 1.0 mL/min, 254 nm; TR = 5.77 min (minor), 6.73 min (major)). ^{1}H NMR (500 MHz, $CDCl_3$) δ 8.30 (d, *J* = 8.5 Hz, 1H), 7.59 (d, *J* = 8.0 Hz, 1H), 7.41 – 7.37 (m, 2H), 7.19 – 7.15 (m, 1H), 7.11 – 7.05 (m, 4H), 6.82 (t, *J* = 7.5 Hz, 1H), 6.73 (d, *J* = 9.0 Hz, 1H), 6.38 – 6.34 (m, 2H), 6.22 (d, *J* = 8.0 Hz, 1H), 4.90 (s, 1H), 4.72 (d, *J* = 17.5 Hz, 1H), 4.60 (d, *J* = 17.5 Hz, 1H), 4.21 (s, 1H), 3.08 – 3.01 (m, 1H), 2.89 – 2.81 (m, 1H), 2.56 – 2.48 (m, 1H), 2.34 (s, 3H), 2.32 – 2.28 (m, 1H), 2.27 (s, 3H); ^{13}C NMR (125 MHz, $CDCl_3$) δ 148.6, 144.0, 136.9, 136.8, 134.7, 133.1, 131.1, 129.4, 129.3, 128.3, 128.1, 127.6, 126.4(3), 126.3(9), 123.3, 122.3, 122.1, 121.9, 121.1, 115.0, 107.9, 87.3, 64.2, 53.8, 37.2, 33.2, 21.0, 17.5; IR (neat): 3383(br), 2853, 1667,

1596, 1461, 1349, 1180, 1086, 807, 760; HRESIMS Calcd for $[C_{31}H_{28}N_2Na]^+$ (M + Na^+) 451.2145, found 451.2140.

(7a*R*,12b*R*)-12-bromo-7-(4-methylbenzyl)-7a,8,13,14-tetrahydro-7*H*-benzo[*f*]cyclopenta[*c*]indolo[2,3-*b*]quinoline (4j)

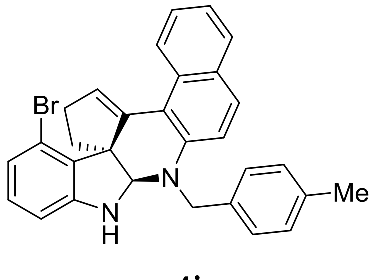

4j

Compound **4j** was prepared in 85% yield (41.9 mg) according to the general procedure (Fig. 4, entry 10). The product was isolated through silica gel column chromatography (PE:EA = 10:1) as a pale yellow solid (mp 172-173 °C). $[\alpha]_D^{20}$ = -0.6 °(c = 1.0, $CHCl_3$). 89% ee (determined by HPLC: Chiralpak IG Column, 20/80 *i*-PrOH/hexane, 1.0 mL/min, 254 nm; TR = 4.84 min (minor), 5.45 min (major)). ^1H NMR (500 MHz, $CDCl_3$) δ 8.45 (d, *J* = 8.5 Hz, 1H), 7.57 (d, *J* = 8.0 Hz, 1H), 7.41 – 7.36 (m, 2H), 7.18 – 7.14 (m, 1H), 7.11 – 7.06 (m, 4H), 6.77 (d, *J* = 9.0 Hz, 1H), 6.73 – 6.66 (m, 2H), 6.34 (s, 1H), 6.24 (d, *J* = 7.0 Hz, 1H), 4.92 (s, 1H), 4.60 (q, *J* = 17.0 Hz, 2H), 4.35 (s, 1H), 3.21 – 3.13 (m, 1H), 2.85 – 2.78 (m, 1H), 2.47 – 2.40 (m, 2H), 2.33 (s, 3H); ^{13}C NMR (125 MHz, $CDCl_3$) δ 150.6, 143.2, 136.9, 135.7, 135.2, 133.3, 131.3, 131.2, 129.4, 129.1, 127.9, 127.8(4), 127.7(8), 126.6, 126.1, 124.5, 123.9, 122.2, 119.4, 115.0, 108.4, 87.2, 66.2, 53.6, 37.4, 33.8, 21.0; IR (neat): 3392(br), 2922, 1599, 1511, 1270, 1094, 799, 752, 595, 454; HRESIMS Calcd for $[C_{30}H_{25}BrN_2Na]^+$ (M + Na^+) 515.1093, found 515.1095.

(7a*R*,12b*R*)-11-methyl-7-(4-methylbenzyl)-7a,8,13,14-tetrahydro-7*H*-benzo[*f*]cyclopenta[*c*]indolo[2,3-*b*]quinoline (4k)

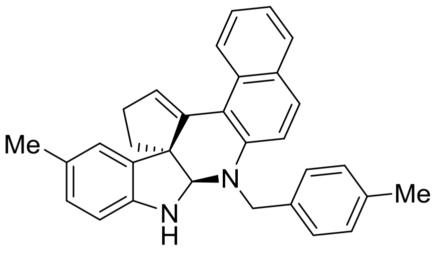

4k

Compound **4k** was prepared in 90% yield (38.6 mg) according to the general procedure (Fig. 4, entry 11). The product was isolated through silica gel column chromatography (PE:EA = 7:1) as a pale yellow oil. $[\alpha]_D^{20}$ = -22.5 °(c = 1.0, $CHCl_3$). 91% ee (determined by HPLC: Chiralpak IG Column, 10/90 *i*-PrOH/hexane, 1.0 mL/min, 254 nm; TR = 6.46 min (minor), 7.03 min (major)). ^{1}H NMR (500 MHz, $CDCl_3$) δ 8.31 (d, *J* = 8.5 Hz, 1H), 7.58 (d, *J* = 8.0 Hz, 1H), 7.44 (d, *J* = 9.0 Hz, 1H), 7.40 – 7.36 (m, 1H), 7.20 – 7.16 (m, 3H), 7.10 (d, *J* = 8.0 Hz, 2H), 6.89 (d, *J* = 9.0 Hz, 1H), 6.85 (d, *J* = 1.5 Hz, 1H), 6.70 – 6.67 (m, 1H), 6.38 (t, *J* = 2.5 Hz, 1H), 6.31 (d, *J* = 8.0 Hz, 1H), 5.02 (s, 1H), 4.70 (d, *J* = 16.0 Hz, 1H), 4.56 (d, *J* = 16.0 Hz, 1H), 3.47 (s, 1H), 3.07 – 2.99 (m, 1H), 2.73 – 2.67 (m, 1H), 2.39 – 2.34 (m, 2H), 2.33 (s, 3H), 2.13 (s, 3H); ^{13}C NMR (125 MHz, $CDCl_3$) δ 145.8, 142.4, 140.2, 137.9, 136.9, 135.4, 132.1, 131.6, 129.3, 128.8, 128.2, 128.0, 127.1, 127.0, 126.3, 123.9, 123.3, 122.3, 115.9, 109.4, 86.3, 64.8, 53.5, 41.2, 32.0, 21.1, 20.9; IR (neat): 3385(br), 2923, 2851, 1618, 1512, 1436, 1593, 1094, 753, 705; HRESIMS Calcd for $[C_{31}H_{28}N_2Na]^+$ (M + Na^+) 451.2145, found 451.2142.

(7a*R*,12b*R*)-11-bromo-7-(4-methylbenzyl)-7a,8,13,14-tetrahydro-7*H*-benzo[*f*]cyclopenta[*c*]indolo[2,3-*b*]quinoline (4l)

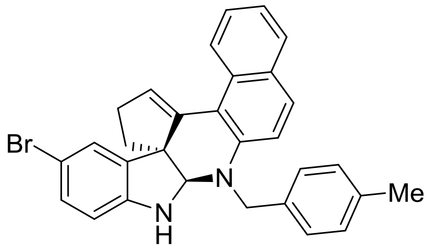

4l

Compound **4l** was prepared in 81% yield (39.9 mg) according to the general procedure (Fig. 4, entry 12). The product was isolated through silica gel column chromatography (PE:EA = 10:1) as a pale yellow solid (mp 100-101 ºC). $[\alpha]_D^{20}$ = -21.2 °(c = 1.0, $CHCl_3$). 81% ee (determined by HPLC: Chiralpak ADH Column, 5/95 *i*-PrOH/hexane, 1.0 mL/min, 254 nm; TR = 20.21 min (major), 22.59 min (minor)). ^{1}H NMR (500 MHz, $CDCl_3$) δ 8.27 (d, *J* = 8.5 Hz, 1H), 7.60 (d, *J* = 8.0 Hz, 1H), 7.47 (d, *J* = 9.0 Hz, 1H), 7.42 – 7.36 (m, 1H), 7.24 –7.19 (m, 1H), 7.17 (d, *J* = 7.5 Hz, 2H), 7.12 – 7.09 (m, 3H), 6.96 – 6.89 (m, 2H), 6.36 (s, 1H), 6.20 (d, *J* = 8.0 Hz, 1H), 5.01 (s, 1H), 4.67 (d, *J* = 15.5 Hz, 1H), 4.51 (d, *J* = 15.5 Hz, 1H), 4.32 (s, 1H), 3.04 – 2.95 (m, 1H), 2.74 – 2.71

(m, 1H), 2.40 – 2.34 (m, 2H), 2.33 (s, 3H); ^{13}C NMR (125 MHz, $CDCl_3$) δ 147.4, 137.5, 137.3, 137.1, 135.2, 132.7, 131.4, 130.5, 129.4, 128.4, 128.1, 127.4, 127.2, 126.6, 125.6, 123.8, 122.7, 116.0, 114.9, 110.8, 110.4, 85.9, 65.1, 53.5, 41.1, 32.0, 21.1; IR (neat): 3385(br), 2921, 1593, 1512, 1476, 1217, 1094, 804, 742, 497; HRESIMS Calcd for $[C_{30}H_{25}BrN_2Na]^+$ (M + Na^+) 515.1093, found 515.1089.

(7a*R*,12b*R*)-10-methyl-7-(4-methylbenzyl)-7a,8,13,14-tetrahydro-7*H*-benzo[*f*]cyclopenta[*c*]indolo[2,3-*b*]quinoline (4m)

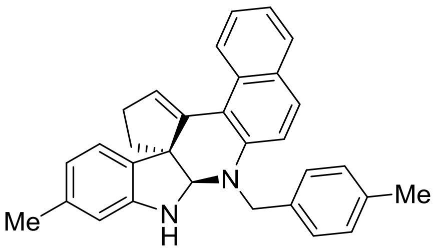

4m

Compound **4m** was prepared in 66% yield (28.3 mg) according to the general procedure (Fig. 4, entry 13). The product was isolated through silica gel column chromatography (PE:EA = 7:1) as a pale yellow oil. $[\alpha]_D^{20}$ = -28.3 °(c = 1.0, $CHCl_3$). 84% ee (determined by HPLC: Chiralpak IG Column, 10/90 *i*-PrOH/hexane, 1.0 mL/min, 254 nm; TR = 7.30 min (minor), 9.50 min (major)). ^{1}H NMR (500 MHz, $CDCl_3$) δ 8.27 (d, *J* = 8.5 Hz, 1H), 7.58 (d, *J* = 8.0 Hz, 1H), 7.45 (d, *J* = 9.0 Hz, 1H), 7.38 – 7.34 (m, 1H), 7.21 – 7.18 (m, 2H), 7.18 – 7.15 (m, 1H), 7.12 (d, *J* = 8.0 Hz, 2H), 6.95 – 6.90 (m, 2H), 6.39 (d, *J* = 7.5 Hz, 1H), 6.34 – 6.32 (m, 1H), 6.20 (s, 1H), 5.02 (s, 1H), 4.71 (d, *J* = 15.5 Hz, 1H), 4.54 (d, *J* = 15.5 Hz, 1H), 4.23 (s, 1H), 3.06 – 2.98 (m, 1H), 2.74 – 2.68 (m, 1H), 2.37 – 2.34 (m, 2H), 2.33 (s, 3H), 2.12 (s, 3H); ^{13}C NMR (125 MHz, $CDCl_3$) δ 150.5, 148.5, 148.4, 138.2, 137.6, 136.9, 132.1, 131.5, 129.9, 129.3, 128.1, 128.0, 127.3, 126.6, 126.3, 123.9, 122.3, 120.1, 116.1, 110.0, 86.2, 64.9, 53.5, 41.4, 32.0, 21.4, 21.1; IR (neat): 3389(br), 2923, 1621, 1513, 1091, 1018, 798, 760, 685, 454; HRESIMS Calcd for $[C_{31}H_{28}N_2Na]^+$ (M + Na^+) 451.2145, found 451.2146.

(7a*R*,12b*R*)-10-chloro-7-(4-methylbenzyl)-7a,8,13,14-tetrahydro-7*H*-benzo[*f*]cyclopenta[*c*]indolo[2,3-*b*]quinoline (4n)

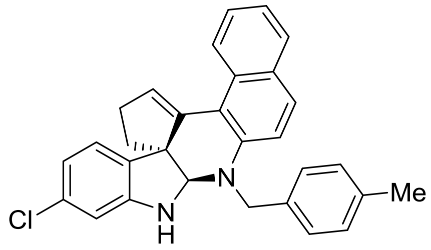

4n

Compound **4n** was prepared in 62% yield (27.8 mg) according to the general procedure (Fig. 4, entry 14). The product was isolated through silica gel column chromatography (PE:EA = 10:1) as a pale yellow solid (mp 120-121 °C). $[\alpha]_D^{20}$ = -2.8 °(c = 1.0, $CHCl_3$). 90% ee (determined by HPLC: Chiralpak IG Column, 10/90 *i*-PrOH/hexane, 1.0 mL/min, 254 nm; TR = 6.42 min (minor), 8.41 min (major)). ^1H NMR (500 MHz, $CDCl_3$) δ 8.23 (d, *J* = 8.5 Hz, 1H), 7.60 (d, *J* = 8.0 Hz, 1H), 7.48 (d, *J* = 9.0 Hz, 1H), 7.40 – 7.35 (m, 1H), 7.22 – 7.18 (m, 3H), 7.13 (d, *J* = 8.0 Hz, 2H), 6.95 – 6.91 (m, 2H), 6.53 – 6.50 (m, 1H), 6.34 (t, *J* = 2.5 Hz, 1H), 6.31 (d, *J* = 1.5 Hz, 1H), 5.03 (s, 1H), 4.69 (d, *J* = 15.5 Hz, 1H), 4.52 (d, *J* = 15.5 Hz, 1H), 4.32 (s, 1H), 3.04 – 2.96 (m, 1H), 2.76 – 2.70 (m, 1H), 2.38 – 2.35 (m, 2H), 2.34 (s, 3H); ^{13}C NMR (125 MHz, $CDCl_3$) δ 149.6, 143.1, 137.8, 137.1, 135.2, 133.5, 133.1, 131.4, 129.4, 128.4, 128.1, 127.2, 126.9, 126.5, 123.7, 123.4, 122.6, 118.9, 116.0, 109.0, 86.0, 64.9, 53.5, 41.1, 32.0, 21.1; IR (neat): 3405(br), 2923, 1603, 1512, 1482, 1092, 758, 754, 746, 587; HRESIMS Calcd for $[C_{30}H_{25}ClN_2Na]^+$ (M + Na^+) 471.1598, found 471.1594.

methyl (7a*R*,12b*R*)-7-(4-methylbenzyl)-7a,8,13,14-tetrahydro-7*H*-benzo[*f*]cyclopenta[*c*]indolo[2,3-*b*]quinoline-10-carboxylate (4o)

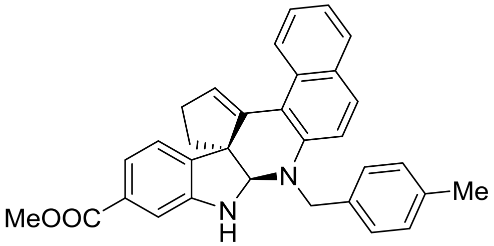

4o

Compound **4o** was prepared in 81% yield (38.3 mg) according to the general procedure (Fig. 4, entry 15). The product was isolated through silica gel column chromatography (PE:EA = 7:1) as a pale yellow oil. $[\alpha]_D^{20}$ = -2.0 ° (c = 1.0, $CHCl_3$). 94% ee (determined by HPLC: Chiralpak IG

Column, 20/80 *i*-PrOH/hexane, 1.0 mL/min, 254 nm; TR = 10.87 min (minor), 13.02 min (major)). ^{1}H NMR (500 MHz, $CDCl_3$) δ 8.24 (d, *J* = 8.5 Hz, 1H), 7.58 (d, *J* = 8.0 Hz, 1H), 7.46 (d, *J* = 9.0 Hz, 1H), 7.37 (t, *J* = 7.5 Hz, 1H), 7.28 (d, *J* = 7.5 Hz, 1H), 7.20 – 7.16 (m, 3H), 7.12 (d, *J* = 8.0 Hz, 2H), 7.08 (d, *J* = 8.0 Hz, 1H), 6.99 (s, 1H), 6.92 (d, *J* = 9.0 Hz, 1H), 6.36 (s, 1H), 5.04 (s, 1H), 4.69 (d, *J* = 16.0 Hz, 1H), 4.52 (d, *J* = 16.0 Hz, 1H), 4.41 (s, 1H), 3.76 (s, 3H), 3.07 – 2.97 (m, 1H), 2.76 – 2.71 (m, 1H), 2.42 – 2.36 (m, 2H), 2.33 (s, 3H); ^{13}C NMR (125 MHz, $CDCl_3$) δ 167.3, 148.6, 143.1, 140.4, 137.7, 137.0, 135.2, 131.4, 129.8, 129.4, 128.4, 128.1(1), 128.0(6), 127.2, 126.5, 123.6, 122.5, 122.3, 121.2, 116.0, 115.9, 109.4, 85.8, 65.4, 53.5, 51.8, 41.0, 32.1, 21.1; IR (neat): 3378(br), 1715, 1513, 1456, 1286, 1227, 1093, 767, 749, 453; HRESIMS Calcd for $[C_{32}H_{28}N_2NaO_2]^+$ (M + Na^+) 495.2043, found 495.2045.

(7a*R*,12b*R*)-9-methyl-7-(4-methylbenzyl)-7a,8,13,14-tetrahydro-7*H*-benzo[*f*]cyclopenta[*c*]indolo[2,3-*b*]quinoline (4p)

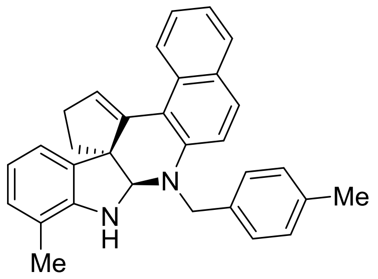

4p

Compound **4p** was prepared in 57% yield (24.4 mg) according to the general procedure (Fig. 4, entry 16). The product was isolated through silica gel column chromatography (PE:EA = 7:1) as a pale yellow oil. $[\alpha]_D^{20}$ = -0.4 °(c = 1.0, $CHCl_3$). 85% ee (determined by HPLC: Chiralpak ADH Column, 10/90 *i*-PrOH/hexane, 1.0 mL/min, 254 nm; TR = 5.46 min (minor), 6.07 min (major)). ^{1}H NMR (500 MHz, $CDCl_3$) δ 8.31 (d, *J* = 8.5 Hz, 1H), 7.58 (d, *J* = 8.0 Hz, 1H), 7.42 (d, *J* = 9.0 Hz, 1H), 7.37 (t, *J* = 7.5 Hz, 1H), 7.21 – 7.15 (m, 3H), 7.10 (d, *J* = 8.0 Hz, 2H), 6.91 (d, *J* = 7.5 Hz, 1H), 6.87 (d, *J* = 9.0 Hz, 1H), 6.73 (d, *J* = 7.5 Hz, 1H), 6.54 (t, *J* = 7.5 Hz, 1H), 6.39 (s, 1H), 5.06 (s, 1H), 4.72 – 4.63 (m, 2H), 3.83 (s, 1H), 3.06 – 2.98 (m, 1H), 2.75 – 2.72 (m, 1H), 2.44 – 2.34 (m, 2H), 2.33 (s, 3H), 1.86 (s, 3H); ^{13}C NMR (125 MHz, $CDCl_3$) δ 146.7, 143.2, 137.6, 136.9, 135.8, 134.4, 131.7, 129.4, 128.7, 128.2, 128.0, 127.1, 126.9, 126.4, 123.9, 122.3, 120.0, 119.5, 118.6, 115.8, 115.7, 115.0, 86.3, 64.8, 53.8, 41.1, 32.0, 21.1, 16.4; IR (neat): 3365(br), 2922, 1598,

1512, 1467, 1264, 1095, 770, 752, 556; HRESIMS Calcd for $[C_{31}H_{28}N_2Na]^+$ (M + Na^+) 451.2145, found 451.2140.

(7a*R*,12b*R*)-3-methyl-7-(4-methylbenzyl)-7a,8,13,14-tetrahydro-7*H*-benzo[*f*]cyclopenta[*c*]indolo[2,3-*b*]quinoline (4q)

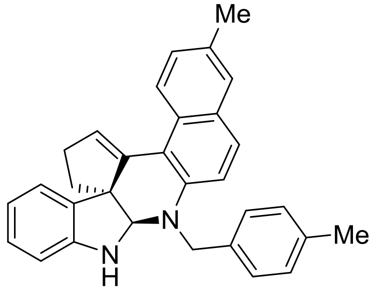

4q

Compound **4q** was prepared in 63% yield (27.0 mg) according to the general procedure (Fig. 4, entry 17). The product was isolated through silica gel column chromatography (PE:EA = 7:1) as a pale yellow solid (mp 124-125 ºC). $[\alpha]_D^{20}$ = -1.2 º(c = 1.0, $CHCl_3$). 88% ee (determined by HPLC: Chiralpak ADH Column, 10/90 *i*-PrOH/hexane, 1.0 mL/min, 254 nm; TR = 8.63 min (minor), 12.11 min (major)). ^{1}H NMR (500 MHz, $CDCl_3$) δ 8.18 (d, *J* = 9.0 Hz, 1H), 7.37 (d, *J* = 9.0 Hz, 2H), 7.23 – 7.18 (m, 3H), 7.11 (d, *J* = 8.0 Hz, 2H), 7.04 (d, *J* = 7.5 Hz, 1H), 6.90 – 6.84 (m, 2H), 6.57 (t, *J* = 7.5 Hz, 1H), 6.38 – 6.33 (m, 2H), 5.02 (s, 1H), 4.70 (d, *J* = 15.5 Hz, 1H), 4.53 (d, *J* = 15.5 Hz, 1H), 4.15 (s, 1H), 3.07 – 2.99 (m , 1H), 2.74 – 2.67 (m, 1H), 2.40 (s, 3H), 2.39 – 2.35 (m, 2H), 2.33 (s, 3H); ^{13}C NMR (125 MHz, $CDCl_3$) ^{13}C NMR (125 MHz, $CDCl_3$) δ 148.5, 142.6, 138.3, 136.8, 135.5, 135.0, 131.7, 129.7, 129.3, 128.6, 128.3, 127.6, 127.5, 127.2, 126.9, 126.5, 123.8, 122.6, 119.2, 116.0, 109.1, 85.7, 65.1, 53.5, 41.2, 32.0, 21.2, 21.1; IR (neat): 3383(br), 2920, 1603, 1484, 1261, 1093, 818, 763, 756, 436; HRESIMS Calcd for $[C_{31}H_{28}N_2Na]^+$ (M + Na^+) 451.2145, found 451.2143.

(7a*R*,12b*R*)-3-bromo-7-(4-methylbenzyl)-7a,8,13,14-tetrahydro-7*H*-benzo[*f*]cyclopenta[*c*]indolo[2,3-*b*]quinoline (4r)

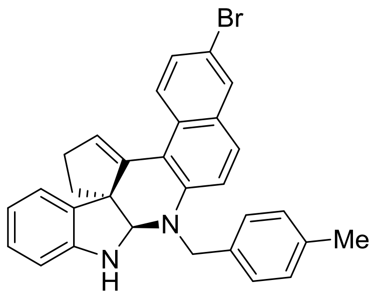

4r

Compound **4r** was prepared in 61% yield (30.1 mg) according to the general procedure (Fig. 4, entry 18). The product was isolated through silica gel column chromatography (PE:EA = 10:1) as a pale yellow oil. $[\alpha]_D^{20}$ = -18.6 °(c = 1.0, $CHCl_3$). 84% ee (determined by HPLC: Chiralpak IG Column, 10/90 *i*-PrOH/hexane, 1.0 mL/min, 254 nm; TR = 8.41 min (minor), 9.95 min (major)). ^1H NMR (500 MHz, $CDCl_3$) δ 8.14 (d, *J* = 9.0 Hz, 1H), 7.71 (s, 1H), 7.40 (d, *J* = 9.0 Hz, 1H), 7.32 (d, *J* = 9.0 Hz, 1H), 7.17 (d, *J* = 8.0 Hz, 2H), 7.12 (d, *J* = 8.0 Hz, 2H), 7.02 (d, *J* = 7.5 Hz, 1H), 6.90 – 6.87 (m, 2H), 6.59 (t, *J* = 7.5 Hz, 1H), 6.38 (d, *J* = 8.0 Hz, 1H), 6.32 (s, 1H), 5.01 (s, 1H), 4.68 (d, *J* = 16.0 Hz, 1H), 4.54 (d, *J* = 16.0 Hz, 1H), 4.30 (s, 1H), 3.06 – 2.98 (m, 1H), 2.76 – 2.68 (m, 1H), 2.39 – 2.35 (m, 2H), 2.33 (s, 3H); ^{13}C NMR (125 MHz, $CDCl_3$) δ 148.3, 143.5, 137.7, 137.0, 135.2, 134.8, 130.0, 129.8, 129.5, 129.4, 129.1, 127.8, 127.3, 127.2, 127.0, 125.7, 122.5, 119.4, 116.8, 115.8, 115.7, 109.3, 85.8, 65.0, 53.4, 41.1, 32.1, 21.1; IR (neat): 3390(br), 2923, 2851, 1606, 1584, 1499, 1346, 1229, 801; HRESIMS Calcd for $[C_{30}H_{25}BrN_2Na]^+$ (M + Na^+) 515.1093, found 515.1091.

(7a*S*,12b*S*)-7-(4-methylbenzyl)-7a,8,13,14-tetrahydro-7*H*-benzo[*f*]cyclopenta[*c*]indolo[2,3-*b*]quinoline (4b′)

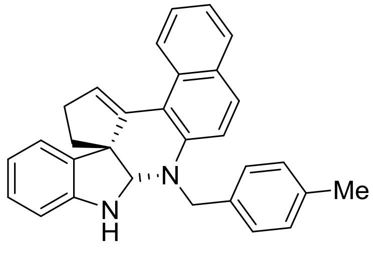

4b′

Compound **4b′** was prepared in 82% yield (34.0 mg) according to the general procedure (Fig. 4, entry 19). $[\alpha]_D^{20}$ = +6.3 °(c = 1.0, $CHCl_3$). 90% ee (determined by HPLC: Chiralpak IG Column, 20/80 *i*-PrOH/hexane, 1.0 mL/min, 254 nm; TR = 5.56 min (major), 6.66 min (minor)).

10-(4-methylbenzyl)-9a,10-dihydro-9*H*-benzo[*f*]indeno[1,2-*c*]indolo[2,3-*b*]quinoline (4s)

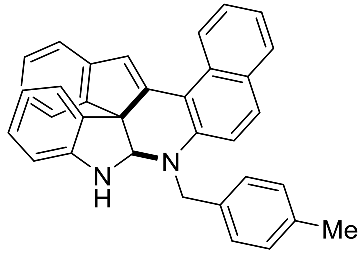

4s

Compound **4s** was prepared in 88% yield (40.7 mg) according to the general procedure (Fig. 4, entry 20). The product was isolated through silica gel column chromatography (PE:EA = 7:1) as a pale yellow solid (mp 205-206 °C). ^{1}H NMR (500 MHz, $CDCl_3$) δ 8.49 (d, *J* = 8.5 Hz, 1H), 7.61 (d, *J* = 8.0 Hz, 1H), 7.51 – 7.42 (m, 3H), 7.38 – 7.35 (m, 2H), 7.30 (t, *J* = 7.5 Hz, 1H), 7.23 (t, *J* = 7.5 Hz, 1H), 7.10 – 7.07 (m, 2H), 7.06 – 7.02 (m, 3H), 6.93 (t, *J* = 7.5 Hz, 1H), 6.79 (d, *J* = 9.0 Hz, 1H), 6.56 – 6.51 (m, 2H), 6.44 (t, *J* = 7.5 Hz, 1H), 5.11 (s, 1H), 4.72 (d, *J* = 17.7 Hz, 1H), 4.63 (s, 1H), 4.47 (d, *J* = 17.7 Hz, 1H), 2.33 (s, 3H); ^{13}C NMR (125 MHz, $CDCl_3$) δ 149.3, 148.9, 143.4, 143.1, 141.7, 136.9, 135.7, 131.3(4), 131.2(7), 129.4, 129.0, 128.2, 128.1, 127.8, 127.5, 127.4, 126.9, 126.3, 125.1, 123.7, 122.3, 122.1, 122.0, 120.9, 119.7, 115.2, 111.6, 110.4, 82.9, 65.0, 54.6, 21.0; IR (neat): 3416(br), 2932, 2078, 1694, 1541, 1456, 1285, 1018, 817, 749; HRESIMS Calcd for $[C_{34}H_{26}N_2Na]^+$ (M + Na^+) 485.1988, found 485.1991.

(7a*R*,12b*R*)-7-(4-methylbenzyl)-7,7a,8,13,14,15-hexahydrobenzo[*a*]indolo[2,3-*g*]phenanthridine (4t)

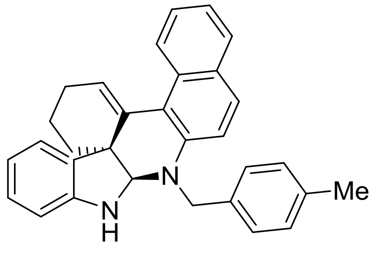

4t

Compound **4t** was prepared in 72% yield (30.8 mg) according to the general procedure (Fig. 4, entry 21). The product was isolated through silica gel column chromatography (PE:EA = 7:1) as a pale yellow oil. $[\alpha]_D^{20}$ = -3.7 °(c = 1.0, $CHCl_3$). 91% ee (determined by HPLC: Chiralpak ADH

Column, 20/80 *i*-PrOH/hexane, 1.0 mL/min, 254 nm; TR = 5.48 min (minor), 8.26 min (major)). ^{1}H NMR (500 MHz, $CDCl_3$) δ 8.19 (d, *J* = 8.5 Hz, 1H), 7.55 (d, *J* = 8.0 Hz, 1H), 7.40 (d, *J* = 9.0 Hz, 1H), 7.31 (t, *J* = 7.5 Hz, 1H), 7.21 (d, *J* = 8.0 Hz, 2H), 7.14 (t, *J* = 8.0 Hz, 3H), 7.08 (d, *J* = 7.0 Hz, 1H), 6.90 (d, *J* = 9.0 Hz, 1H), 6.81 (t, *J* = 7.5 Hz, 1H), 6.50 (t, *J* = 7.5 Hz, 1H), 6.37 – 6.31 (m, 2H), 4.87 (s, 1H), 4.69 (d, *J* = 16.0 Hz, 1H), 4.53 (d, *J* = 16.0 Hz, 1H), 4.30 (s, 1H), 2.62 – 2.56 (m, 1H), 2.47 – 2.38 (m, 1H), 2.34 (s, 3H), 2.09 – 1.99 (m, 3H), 1.83 – 1.76 (m, 1H); ^{13}C NMR (125 MHz, $CDCl_3$) δ 149.1, 142.6, 136.9, 135.6, 131.5, 131.3, 129.3, 128.6, 128.3, 127.9, 127.3, 125.7, 124.1, 123.8, 122.1, 121.4, 118.7, 115.7, 108.9, 85.1, 54.6, 53.0, 37.2, 25.7, 21.1, 18.7; IR (neat): 3391(br), 1646, 1511, 1276, 1261, 1137, 1093, 763, 740, 595; HRESIMS Calcd for $[C_{31}H_{28}N_2Na]^+$ (M + Na^+) 451.2145, found 451.2148.

(7a*R*,9*S*,11a*S*)-7-benzyl-7,7a,8,9-tetrahydrobenzo[*a*]indeno[1,2-*l*]acridin-9-ol (2aa)

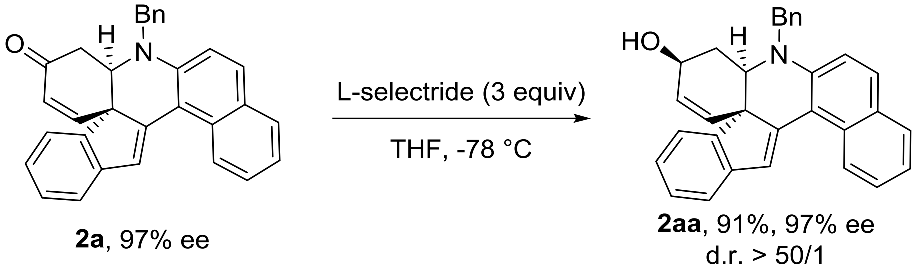

2a, 97% ee

2aa, 91%, 97% ee
d.r. > 50/1

Compound **2aa** was prepared in 91% yield (38.9 mg) according to the above procedure (0.1 mmol scale). The product was isolated through silica gel column chromatography (PE:EA = 3:1) as a pale yellow solid (mp 195-196 ºC). $[\alpha]_D^{20}$ = -5.0 º(c = 1.0, $CHCl_3$). 97% ee (determined by HPLC: Chiralpak ADH Column, 20/80 *i*-PrOH/hexane, 1.0 mL/min, 254 nm; TR = 9.41 min (major), 11.03 min (minor)). ^{1}H NMR (600 MHz, $CDCl_3$) δ 8.40 (d, *J* = 7.8 Hz, 1H), 7.72 (d, *J* = 7.8 Hz, 1H), 7.63 (d, *J* = 9.0 Hz, 1H), 7.49 (t, *J* = 7.2 Hz, 1H), 7.44 (d, *J* = 7.2 Hz, 1H), 7.37 (d, *J* = 7.2 Hz, 1H), 7.34 – 7.28 (m, 3H), 7.17 – 7.13 (m, 2H), 7.09 – 7.03 (m, 3H), 6.96 – 6.94 (m, 2H), 6.07 (dd, *J* = 9.6, 4.8 Hz, 1H), 5.18 – 5.12 (m, 1H), 5.14 (d, *J* = 9.6 Hz, 1H), 4.68 (d, *J* = 16.2 Hz, 1H), 4.36 (s, 1H), 3.39 (s, 1H), 2.92 (d, *J* = 15.6 Hz, 1H), 2.78 (s, 1H), 2.55 – 2.50 (m, 1H); ^{13}C NMR (150 MHz, $CDCl_3$) δ 146.6, 145.5, 145.0, 142.9, 137.2, 131.3, 130.5, 129.5, 128.5(3), 128.5(0), 128.3, 128.0, 127.3, 127.0(9), 127.0(5), 125.4, 124.6, 124.2, 123.5, 123.2, 121.4, 117.8, 114.7, 63.0, 56.2, 55.3, 52.3, 31.1; IR (neat): 3298(br), 1539, 1508, 1266, 1260, 1117, 1081, 778, 724, 695; HRESIMS Calcd for $[C_{31}H_{25}NNaO]^+$ (M + Na^+) 450.1828, found 450.1831.

(7a*R*,11a*S*)-7-benzyl-9-methyl-7,7a,8,9-tetrahydrobenzo[*a*]indeno[1,2-*l*]acridin-9-ol (2ab)

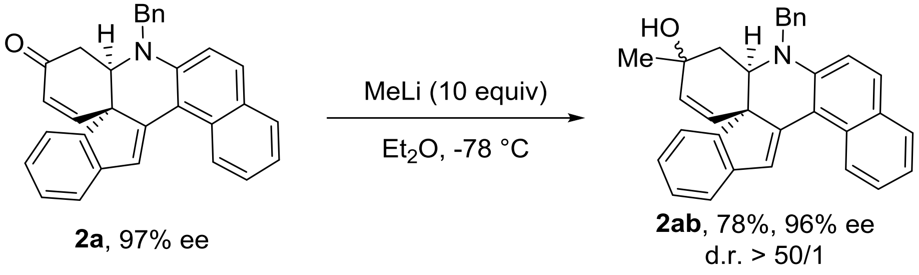

Compound **2ab** was prepared in 78% yield (34.4 mg) according to the above procedure (0.1 mmol scale). The product was isolated through silica gel column chromatography (PE:EA = 5:1) as a pale yellow oil. $[\alpha]_D^{20}$ = -7.2 º (c = 1.0, $CHCl_3$). 96% ee (determined by HPLC: Chiralpak IC Column, 20/80 *i*-PrOH/hexane, 1.0 mL/min, 254 nm; TR = 5.10 min (major), 6.51 min (minor)). ^{1}H NMR (500 MHz, $CDCl_3$) δ 8.39 (d, *J* = 8.5 Hz, 1H), 7.73 (d, *J* = 7.5 Hz, 1H), 7.65 (d, *J* = 8.5 Hz, 1H), 7.55 – 7.48 (m, 1H), 7.45 – 7.43 (m, 1H), 7.33 – 7.27 (m, 3H), 7.23 – 7.20 (m, 1H), 7.17 – 7.11 (m, 2H), 7.06 – 6.99 (m, 3H), 6.94 – 6.87 (m, 2H), 5.87 (d, *J* = 9.5 Hz, 1H), 5.18 (d, *J* = 16.5 Hz, 1H), 5.02 (d, *J* = 9.5 Hz, 1H), 4.64 (d, *J* = 16.5 Hz, 1H), 3.59 (s, 1H), 3.40 (s, 1H), 2.81 (d, *J* = 15.0 Hz, 1H), 2.43 (d, *J* = 15.0 Hz, 1H), 1.48 (s, 3H); ^{13}C NMR (125 MHz, $CDCl_3$) δ 146.8, 145.7, 144.9, 142.9, 137.0, 135.7, 131.2, 129.5, 128.5, 128.4, 128.2, 128.0, 127.4, 127.1, 126.4, 125.6, 124.6, 124.1, 123.5, 123.3, 121.4, 118.1, 115.3, 66.4, 57.4, 55.5, 52.5, 36.6, 29.3; IR (neat): 3328(br), 1656, 1508, 1176, 1161, 1037, 1013, 778, 741, 695; HRESIMS Calcd for $[C_{32}H_{27}NNaO]^+$ (M + Na^+) 464.1985, found 464.1991.

(7a*R*,11*S*,11a*S*)-7-benzyl-11-methyl-7a,8,10,11-tetrahydrobenzo[*a*]indeno[1,2-*l*]acridin-9(7*H*)-one (2ac)

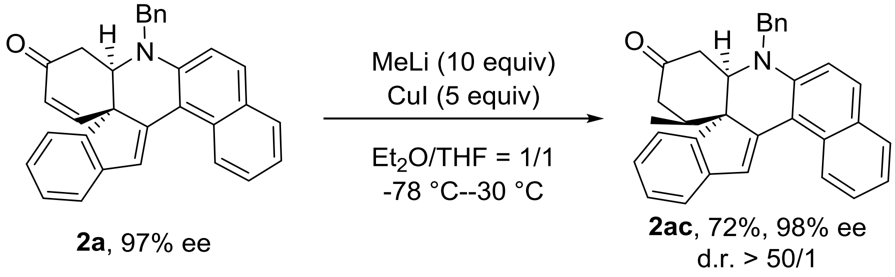

Compound **2ac** was prepared in 72% yield (31.8 mg) according to the above procedure (0.1 mmol scale). The product was isolated through silica gel column chromatography (PE:EA = 5:1) as a pale yellow oil. $[\alpha]_D^{20}$ = -0.4 º (c = 1.0, $CHCl_3$). 98% ee (determined by HPLC: Chiralpak IG

Column, 20/80 *i*-PrOH/hexane, 1.0 mL/min, 254 nm; TR = 10.63 min (major), 11.89 min (minor)). ^{1}H NMR (600 MHz, $CDCl_3$) δ 8.40 (d, *J* = 8.4 Hz, 1H), 7.73 (d, *J* = 7.8 Hz, 1H), 7.60 (d, *J* = 9.0 Hz, 1H), 7.52 (t, *J* = 7.2 Hz, 1H), 7.50 – 7.46 (m, 2H), 7.36 (t, *J* = 7.8 Hz, 1H), 7.34 – 7.30 (m, 1H), 7.22 (t, *J* = 7.2 Hz, 1H), 7.18 – 7.11 (m, 4H), 7.08 (d, *J* = 9.0 Hz, 1H), 6.98 (d, *J* = 7.2 Hz, 2H), 4.85 (d, *J* = 17.4 Hz, 1H), 4.49 (d, *J* = 17.4 Hz, 1H), 3.65 (s, 1H), 3.21 (dd, *J* = 16.8, 1.8 Hz, 1H), 3.12 (dd, *J* = 16.8, 3.6 Hz, 1H), 2.77 – 2.69 (m, 1H), 2.25 (dd, *J* = 18.0, 14.4 Hz, 1H), 2.10 (dd, *J* = 18.0, 3.6 Hz, 1H), 0.52 (d, *J* = 6.6 Hz, 3H); ^{13}C NMR (125 MHz, $CDCl_3$) δ 211.0, 151.1, 144.3, 143.5, 143.3, 137.9, 130.2, 129.4, 128.6, 128.5, 127.9, 127.1(2), 127.0(5), 126.6, 126.5, 124.9, 123.5, 122.9, 121.3, 121.0, 116.4, 114.2, 60.3, 56.7, 53.4, 43.6, 42.2, 35.3, 14.7; IR (neat): 3042, 1676, 1508, 1376, 1268, 1139, 1087, 766, 742, 662; HRESIMS Calcd for $[C_{32}H_{27}NNaO]^+$ (M + Na^+) 464.1985, found 464.1986.

(7a*R*,11a*S*)-11-butyl-7a,8,10,11-tetrahydrobenzo[*a*]indeno[1,2-*l*]acridin-9(7*H*)-one (2ad)

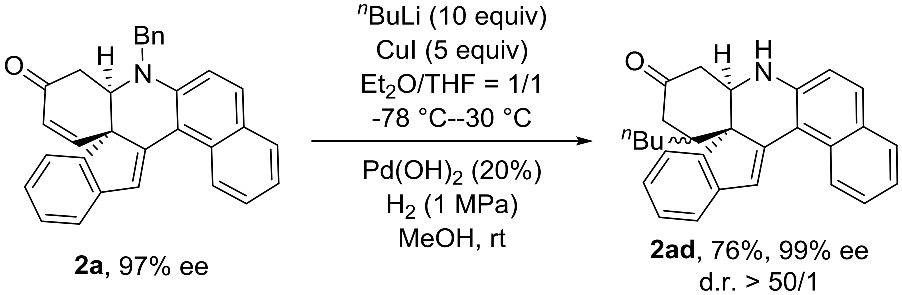

2a, 97% ee

2ad, 76%, 99% ee
d.r. > 50/1

Compound **2ad** was prepared in 76% overall yield (29.9 mg) according to the above procedure (0.1 mmol scale). The product was isolated through silica gel column chromatography (PE:EA = 4:1) as a pale yellow oil. $[\alpha]_D^{20}$ = -2.6 º(c = 1.0, $CHCl_3$). 99% ee (determined by HPLC: Chiralpak IG Column, 10/90 *i*-PrOH/hexane, 1.0 mL/min, 254 nm; TR = 7.63 min (major), 8.23 min (minor)). ^{1}H NMR (500 MHz, $CDCl_3$) δ 8.38 (d, *J* = 8.5 Hz, 1H), 7.72 (d, *J* = 8.0 Hz, 1H), 7.63 (d, *J* = 7.5 Hz, 1H), 7.58 (d, *J* = 9.0 Hz, 1H), 7.52 – 7.48 (m, 2H), 7.41 (t, *J* = 7.5 Hz, 1H), 7.31 – 7.27 (m, 1H), 7.24 – 7.22 (m, 2H), 6.82 (d, *J* = 9.0 Hz, 1H), 4.04 (s, 1H), 3.71 (s, 1H), 3.35 (dd, *J* = 15.5, 3.5 Hz, 1H), 2.73 – 2.62 (m, 2H), 2.56 (dd, *J* = 17.0, 4.0 Hz, 1H), 2.41 – 2.34 (m, 1H), 1.02 – 0.93 (m, 2H), 0.91 – 0.73 (m, 3H), 0.66 – 0.58 (m, 1H), 0.54 (t, *J* = 7.0 Hz, 3H); ^{13}C NMR (125 MHz, $CDCl_3$) δ 210.1, 146.7, 146.4, 144.3, 140.4, 131.6, 129.6, 128.7, 128.5, 128.0, 127.1, 126.1, 124.3, 124.2, 123.3, 122.4, 121.7, 116.7, 108.9, 59.0, 53.0, 45.6, 44.1, 37.2, 29.3, 29.0, 22.3, 13.7; IR

(neat): 3041(br), 1662, 1578, 1476, 1271, 1237, 1078, 863, 741, 695; HRESIMS Calcd for $[C_{28}H_{27}NNaO]^+$ (M + Na$^+$) 416.1985, found 416.1981.

(7a*S*,12b*R*)-7-(4-methylbenzyl)-7a,8,13,14-tetrahydro-7H-benzo[*f*]cyclopenta[*c*]indolo[2,3-*b*]quinoline (4ba)

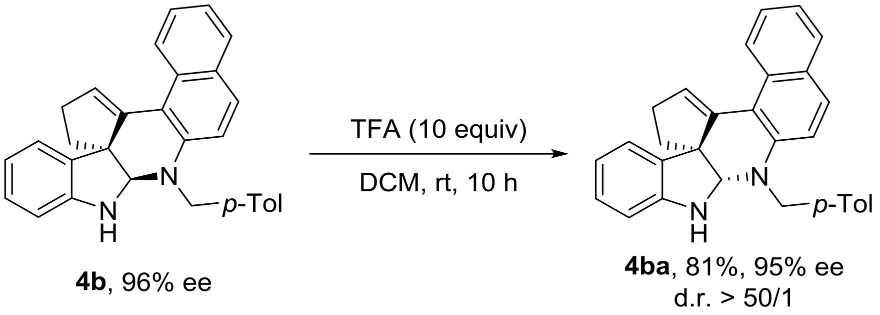

Compound **4ba** was prepared in 81% yield (33.6 mg) according to the above procedure (0.1 mmol scale). The product was isolated through silica gel column chromatography (PE:EA = 7:1) as a pale yellow oil. $[\alpha]_D^{20}$ = -0.5 °(c = 1.0, $CHCl_3$). 95% ee (determined by HPLC: Chiralpak ADH Column, 20/80 *i*-PrOH/hexane, 1.0 mL/min, 254 nm; TR = 10.10 min (minor), 12.87 min (major)). ^{1}H NMR (400 MHz, $CDCl_3$) δ 7.51 – 7.46 (m, 2H), 7.33 (d, *J* = 7.2 Hz, 1H), 7.27 – 7.24 (m, 3H), 7.19 (d, *J* = 7.2 Hz, 1H), 7.17 – 7.13 (m, 2H), 7.01 (d, *J* = 9.2 Hz, 1H), 6.96 – 6.91 (m, 1H), 6.88 (d, *J* = 7.2 Hz, 1H), 6.64 – 6.59 (m, 2H), 6.56 (d, *J* = 7.6 Hz, 1H), 5.36 (s, 1H), 4.95 (s, 1H), 4.51 (m, 2H), 2.91 – 2.81 (m, 1H), 2.65 – 2.56 (m, 1H), 2.42 – 2.29 (m, 5H); ^{13}C NMR (100 MHz, $CDCl_3$) δ 148.5, 143.8, 138.7, 136.8, 136.4, 136.1, 135.3, 130.5, 129.4, 128.4, 127.8, 127.3, 127.1, 126.9, 126.7, 124.5, 121.9(4), 121.8(5), 119.0, 114.3, 110.2, 109.5, 70.0, 59.6, 48.1, 41.2, 31.0, 21.1; IR (neat): 3291(br), 1546, 1510, 1376, 1268, 1135, 1078, 764, 739, 695; HRESIMS Calcd for $[C_{30}H_{26}N_2Na]^+$ (M + Na$^+$) 437.1988, found 437.1981.

(7a*R*,12b*R*)-bis(2-chloroethyl) 13,14-dihydro-7*H*-benzo[*f*]cyclopenta[*c*]indolo[2,3-*b*]quinoline-7,8(7a*H*)-dicarboxylate (4bb)

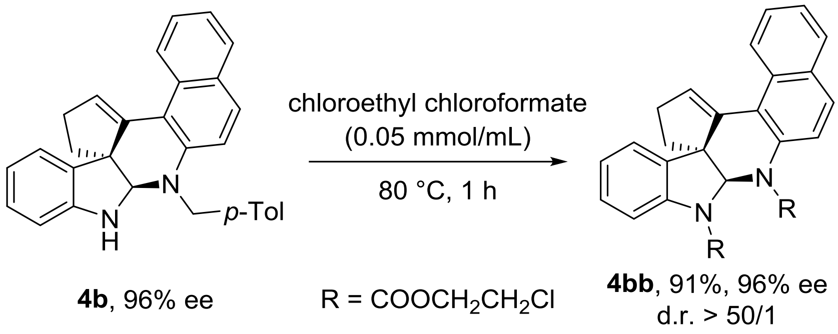

Compound **4bb** was prepared in 91% yield (47.6 mg) according to the above known procedure (0.1 mmol scale) (*67*). The product was isolated through silica gel column chromatography (PE:EA = 3:1) as a pale yellow solid (mp 200-201 ºC). $[\alpha]_D^{20}$ = -0.4 º(c = 1.0, $CHCl_3$). 96% ee (determined by HPLC: Chiralpak IG Column, 20/80 *i*-PrOH/hexane, 1.0 mL/min, 254 nm; TR = 13.99 min (major), 18.25 min (minor)). ^{1}H NMR (500 MHz, $CDCl_3$) δ 8.12 (d, *J* = 8.0 Hz, 1H), 7.78 – 7.75 (m, 1H), 7.70 (d, *J* = 9.0 Hz, 1H), 7.59 – 7.57 (m, 1H), 7.47 – 7.40 (m, 2H), 7.22 (d, *J* = 7.5 Hz, 1H), 7.04 – 6.99 (m, 1H), 6.91 – 6.86 (m, 2H), 6.31 (s, 1H), 4.72 – 4.69 (m, 1H), 4.66 – 4.59 (m, 1H), 4.45 – 4.38 (m, 1H), 4.36 – 4.30 (m, 1H), 3.95 – 3.87 (m, 2H), 3.79 – 3.61 (m, 2H), 3.19 – 3.11 (m, 1H), 2.87 – 2.80 (m, 1H), 2.73 – 2.68 (m, 1H), 2.66 – 2.58 (m, 1H); ^{13}C NMR (125 MHz, $CDCl_3$) δ 153.9, 152.4, 138.6, 134.8, 133.2, 132.0, 130.1, 128.3, 128.2, 128.1, 127.7, 126.6, 125.7, 124.4, 123.7, 122.7, 114.1, 80.6, 65.5, 42.1, 39.7, 32.7; IR (neat): 3081(br), 1671(s), 1530, 1287, 1278, 1109, 1081, 786, 752, 691; HRESIMS Calcd for $[C_{28}H_{24}Cl_2N_2NaO_4]^+$ (M + Na$^+$) 545.1005, found 545.1012.

7. Crystal Data

Table S4. Crystal data and structure refinement for **2g**. CCDC Number = 2207497

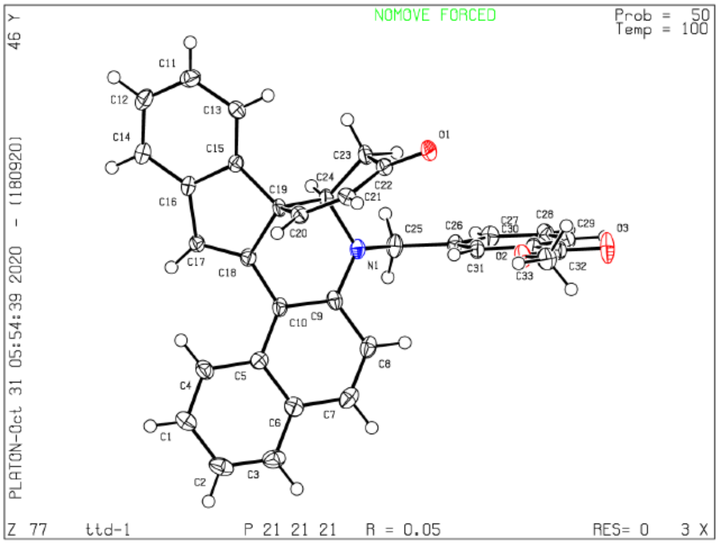

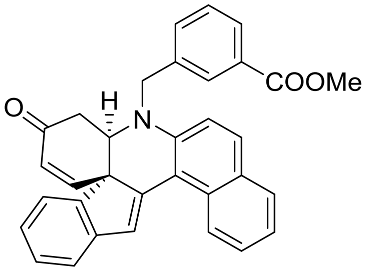

Bond precision: C-C = 0.0054 A Wavelength=1.54184

Cell:	a=5.9809(2)	b=13.2316(4)	c=30.7627(11)
	alpha=90	beta=90	gamma=90
Temperature:	100 K		

	Calculated	Reported
Volume	2434.46(14)	2434.46(14)
Space group	P 21 21 21	P 21 21 21
Hall group	P 2ac 2ab	P 2ac 2ab
Moiety formula	C33 H25 N O3	C33 H25 N O3
Sum formula	C33 H25 N O3	C33 H25 N O3
Mr	483.54	483.54
Dx,g cm-3	1.319	1.319
Z	4	4
Mu (mm-1)	0.668	0.668
F000	1016.0	1016.0
F000’	1018.97	
h,k,lmax	7,15,36	6,15,36
Nref	4123[2409]	4080
Tmin,Tmax	0.993,0.993	0.715,1.000
Tmin’	0.993	

Correction method= # Reported T Limits: Tmin=0.715 Tmax=1.000
AbsCorr = MULTI-SCAN

Data completeness= 1.69/0.99 Theta(max)= 65.063

R(reflections)= 0.0472(3498) wR2(reflections)= 0.1179(4080)

S = 1.062 Npar= 335

Table S5. Crystal data and structure refinement for **4j**. CCDC Number = 2207498

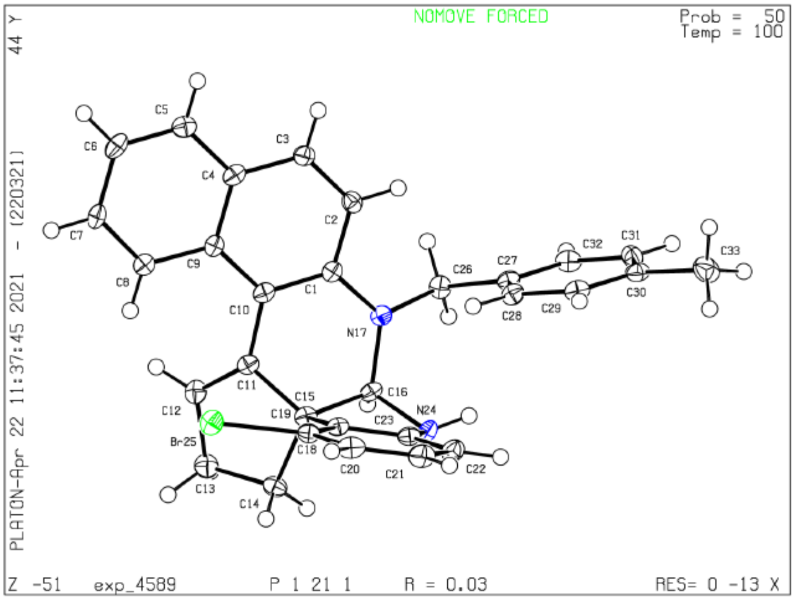

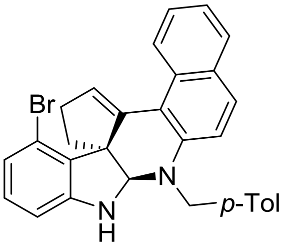

Bond precision: C-C = 0.0054 A Wavelength=1.54184

Cell:	a=7.2161(1)	b=11.7832(2)	c=13.4945(2)
	alpha=90	beta=103.304(2)	gamma=90
Temperature:	100 K		

	Calculated	Reported
Volume	1116.63(3)	1116.63(3)
Space group	P 21	P 1 21 1
Hall group	P 2yb	P 2yb
Moiety formula	C30 H25 Br N2	C30 H25 Br N2
Sum formula	C30 H25 Br N2	C30 H25 Br N2
Mr	493.42	493.43
Dx,g cm-3	1.467	1.468
Z	2	2
Mu (mm-1)	2.661	2.661
F000	508.0	508.0
F000'	507.78	
h,k,lmax	8,13,15	8,13,15
Nref	3845[2025]	2736
Tmin,Tmax	0.441,0.450	0.693,1.000
Tmin'	0.230	

Correction method= # Reported T Limits: Tmin=0.693 Tmax=1.000
AbsCorr = MULTI-SCAN

Data completeness= 1.35/0.71 Theta(max)= 65.557

R(reflections)= 0.0300(2731) wR2(reflections)= 0.0791(2736)

S = 1.050 Npar= 299

Table S6. Crystal data and structure refinement for (rac)-**2aa**. CCDC Number = 2207502

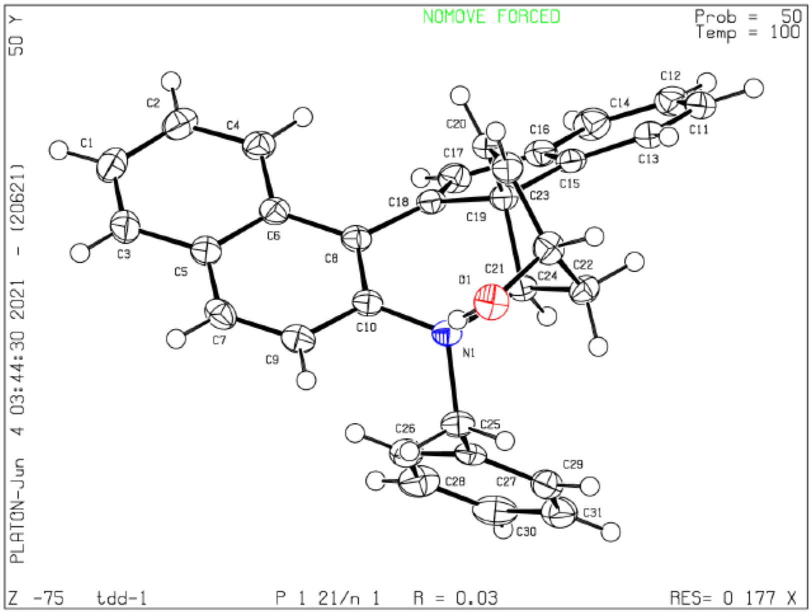

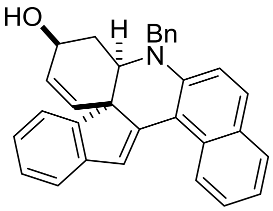

Bond precision: C-C = 0.0018 A Wavelength=1.54184

Cell:	a=14.9337(2)	b=10.6238(1)	c=15.1594(2)
	alpha=90	beta=114.533(2)	gamma=90
Temperature:	100 K		

	Calculated	Reported
Volume	2187.95(6)	2187.95(6)
Space group	P 21/n	P 1 21/n 1
Hall group	-P 2yn	-P 2yn
Moiety formula	C31 H25 N O	C31 H25 N O
Sum formula	C31 H25 N O	C31 H25 N O
Mr	427.52	427.52
Dx,g cm-3	1.298	1.298
Z	4	4
Mu (mm-1)	0.600	0.600
F000	904.0	904.0
F000’	906.44	
h,k,lmax	17,12,17	17,12,17
Nref	3716	3672
Tmin,Tmax	0.993,0.994	0.711,1.000
Tmin’	0.988	

Correction method= # Reported T Limits: Tmin=0.711 Tmax=1.000
AbsCorr = MULTI-SCAN

Data completeness= 0.988 Theta(max)= 64.820

R(reflections)= 0.0328(3445) wR2(reflections)= 0.0836(3672)

S = 1.046 Npar= 299

Table S7. Crystal data and structure refinement for (rac)-**4bb**. CCDC Number = 2207507

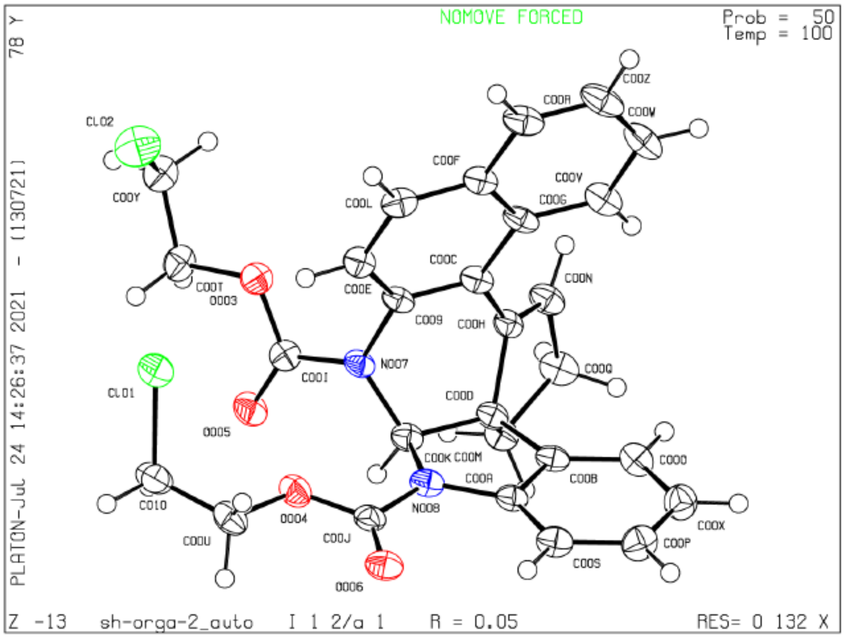

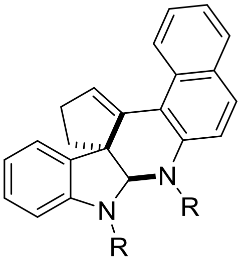

R = $COOCH_2CH_2Cl$

Bond precision: C-C = 0.0048 A Wavelength=1.54184

Cell:	a=12.0006(6)	b=13.5267(8)	c=32.8793(19)
	alpha=90	beta=100.277(5)	gamma=90
Temperature:	100 K		

	Calculated	Reported
Volume	5251.6(5)	5251.6(5)
Space group	I 2/a	I 1 2/a 1
Hall group	-I 2ya	-I 2ya
Moiety formula	C28 H24 Cl2 N2 O4 [+ solvent]	C28 H24 Cl2 N2 O4
Sum formula	C28 H24 Cl2 N2 O4 [+ solvent]	C28 H24 Cl2 N2 O4
Mr	523.39	523.39
Dx,g cm-3	1.324	1.324
Z	8	8
Mu (mm-1)	2.525	2.525
F000	2176.0	2176.0
F000'	2187.71	
h,k,lmax	14,15,38	14,15,38
Nref	4456	4384
Tmin,Tmax	0.463,0.604	0.725,1.000
Tmin'	0.246	

Correction method= # Reported T Limits: Tmin=0.725 Tmax=1.000
AbsCorr = MULTI-SCAN

Data completeness= 0.984 Theta(max)= 64.952

R(reflections)= 0.0481(3105) wR2(reflections)= 0.1378(4384)

S = 1.101 Npar= 325

8. Computational Studies

8.1. Computational Details

All density functional theory (DFT) calculations were performed using Gaussian 16 program. Geometry optimizations were conducted with B3LYP functional (*68, 69*) employing the D3 version of Grimme's dispersion corrections (*70*) with Becke-Johnson damping (*71*), and 6-31G* basis set was used for all atoms. Frequency analysis was also performed at the same level of theory as geometry optimization to confirm whether optimized stationary points were either local minimum or transition state, as well as to evaluate zero-point vibrational energies and thermal corrections for enthalpies and free energies at 298.15 K. Single-point energies and solvent effects were evaluated with ωB97X-D (*72*) functional and def2-TZVP (*73*, *74*) basis set for all atoms. The solvation energies were calculated with a self-consistent reaction field (SCRF) using the SMD implicit solvent model (solvent = Dichloromethane). The 3D diagrams of molecules were generated using CYLView.

To correct the Gibbs free energies under 1 atm to the standard state in solution (1 mol/L), a correction of $RT\ln(cs/cg)$ is added to energies of all species. *cs* stands for the standard molar concentration in solution (1 mol/L), *cg* stands for the standard molar concentration in gas phase (0.040876 mol/L), and *R* is the gas constant. For calculated intermediates at the standard state of 1 mol/L at 298.15 K, the correction value equaling to 1.89 kcal/mol was used.

We used the Conformer-Rotamer Ensemble Sampling Tool CREST (version 2.10.2) in combination with the xTB package (version 6.1) to sample low-energy conformations. The CREST conformational sampling was performed with GFN2-xTB in gas phase. Atoms in the forming/cleaving bonds were constrained by applying a force constant of 1.0 Hartree/Bohr^2. An energy window of 6.0 kcal/mol and a RMSD threshold of 0.25 Å was used. Fully relaxed TS optimizations were performed for the low-lying TSs as well as for those TSs with remarkably different structural features. Based on the conformers obtained by the CREST/xTB sampling, we optimized the geometries at the B3LYP-D3(BJ)/6-31G(d) level of theory, followed by single point energy calculations at the ωB97X-D /def2-TZVP level of theory with SMD (solvent = Dichloromethane) solvation model. Representative types of conformers with low energy were shown in **Table S2-S7** with relative enthalpies and Gibbs free energy stability with respect to the most stable geometry.

Table S8. Located low-energy conformers of **TS4** with relative enthalpies and Gibbs free energies (in kcal/mol) as compared to the most stable conformer

Structure	ΔΔ*H*	ΔΔ*G*
**TS4**	0.0	0.0
**TS4-S1**	1.4	1.4
**TS4-S2**	3.9	3.0
**TS4-S3**	4.2	3.3
**TS4-S4**	5.0	3.8
**TS4-S5**	2.6	4.0
**TS4-S6**	4.6	4.2
**TS4-S7**	5.0	5.4
**TS4-S8**	7.3	5.9
**TS4-S9**	5.6	6.1

Table S9. Located low-energy conformers of **TS6** with relative enthalpies and Gibbs free energies (in kcal/mol) as compared to the most stable conformer

Structure	ΔΔ*H*	ΔΔ*G*
**TS6**	0.0	0.0
**TS6-S1**	2.4	0.8
**TS6-S2**	3.1	1.1
**TS6-S3**	3.0	2.5
**TS6-S4**	5.0	3.2
**TS6-S5**	5.4	5.5

Table S10. Located low-energy conformers of **TS8** with relative enthalpies and Gibbs free energies (in kcal/mol) as compared to the most stable conformer

Structure	ΔΔ*H*	ΔΔ*G*
**TS8**	0.0	0.0
**TS8-S1**	1.6	1.7
**TS8-S2**	1.0	1.8
**TS8-S3**	2.6	3.5
**TS8-S4**	5.5	5.3
**TS8-S5**	8.8	6.0

Table S11. Located low-energy conformers of **TS10** with relative enthalpies and Gibbs free energies (in kcal/mol) as compared to the most stable conformer

Structure	ΔΔ*H*	ΔΔ*G*
**TS10**	0.0	0.0
**TS10-S1**	1.8	1.3
**TS10-S2**	2.9	1.7
**TS10-S3**	3.5	2.7
**TS10-S4**	4.1	3.4

Table S12. Located low-energy conformers of **TS12** with relative enthalpies and Gibbs free energies (in kcal/mol) as compared to the most stable conformer

Structure	$\Delta\Delta H$	$\Delta\Delta G$
**TS12**	0.0	0.0
**TS12-S1**	0.2	0.7
**TS12-S2**	2.0	1.4
**TS12-S3**	2.8	2.7
**TS12-S4**	2.9	3.3

Table S13. Located low-energy conformers of **TS14** with relative enthalpies and Gibbs free energies (in kcal/mol) as compared to the most stable conformer

Structure	$\Delta\Delta H$	$\Delta\Delta G$
**TS14-C1**	0.0	0.0
**TS14-C2**	2.6	0.6
**TS14-S1**	2.8	1.4
**TS14-S2**	3.5	1.6
**TS14-S3**	4.4	2.9
**TS14-S4**	7.4	5.0

8.2. DFT calculations of alternative transition state of nucleophilic addition of allene intermediate Int5.

After the protonation via **TS4**, the generated allene intermediate **Int5-C1** can undergo the direct nucleophilic addition instead of the conformational change to **Int5-C2**. We explored this possibility, and the competing pathways are shown in Supplementary Figure 1. The direct nucleophilic addition from **Int5-C1** without the conformational change proceeds via **TS6-S6**, which is 11.0 kcal/mol less favorable than the nucleophilic addition via **TS6** from **Int5-C2**. Therefore, the conformational change from **Int5-C1** to **Int5-C2** is necessary to allow the more favorable nucleophilic addition via **TS6**.

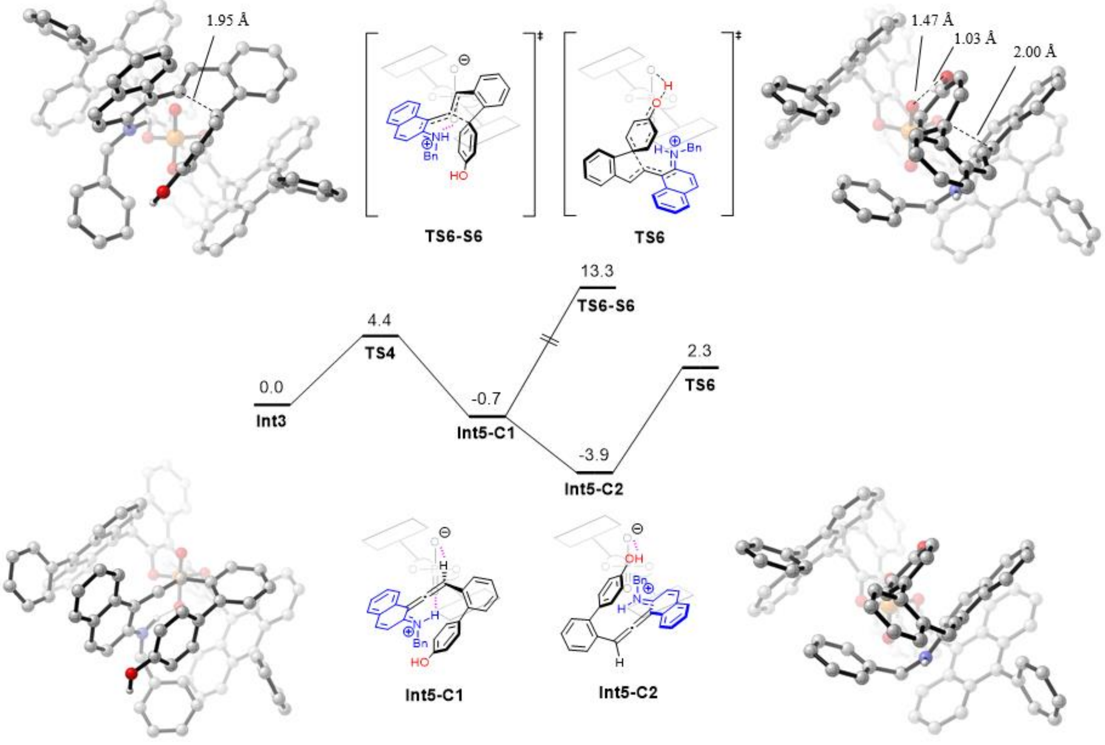

Fig. S1. Alternative transition state of nucleophilic addition of allene intermediate Int5.

8.3. DFT calculations of racemization barrier of Int7

We calculated the racemization barrier of **Int7** to verify that the in-cycle racemization is unlikely. The energies and optimized structures of the corresponding racemization transition state **TS15** and **TS16** are shown in Supplementary Figure 2. Both racemizations are over 10 kcal/mol less favorable than the productive nucleophilic addition via **TS8**.

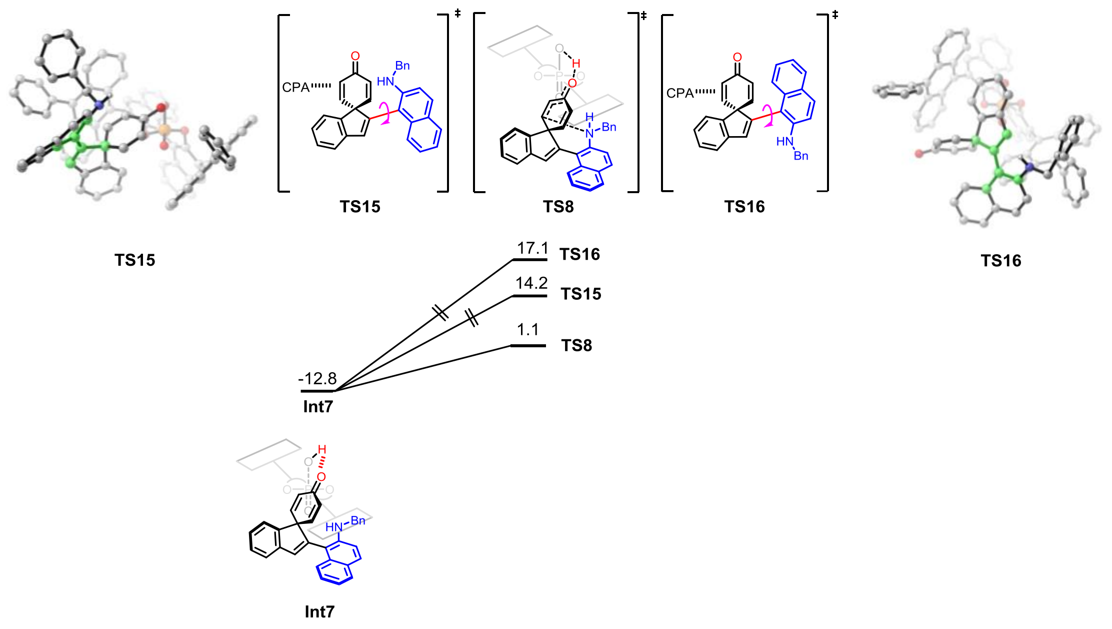

Fig. S2. Optimized structures and DFT-computed free energies of the racemization transition states from Int7, the free energies are compared with Int3.

8.4. Table of Energies

Table S14. Energies in Fig. 7, Fig. 8, Table S8-S13, Fig. S1. Zero-point correction (*ZPE*), thermal correction to enthalpy (*TCH*), thermal correction to Gibbs free energy (*TCG*), energies (*E*), enthalpies (*H*), and Gibbs free energies (*G*) (in Hartree) of the structures calculated at ωB97X-D/def2-TZVP-SMD(Dichloromethane)//B3LYP-D3(BJ)/6-31G(d) level of theory.

Strucutre	*ZPE*	*TCH*	*TCG*	*E*	*H*	*G*	Imaginary Frequency
**1a**	0.451488	0.47895	0.391901	-1325.155899	-1324.676949	-1324.763998	
**2a**	0.455469	0.480576	0.401347	-1325.181889	-1324.701313	-1324.780542	
**anion-CPA-A4**	0.782361	0.831891	0.694654	-2950.753827	-2949.921936	-2950.059173	
**Int3**	1.249944	1.3268	1.135134	-4276.397615	-4275.070815	-4275.262481	
**TS4**	1.245774	1.321785	1.132915	-4276.388443	-4275.066658	-4275.255528	1316.3*i*
**TS4-S1**	1.245662	1.321721	1.132831	-4276.386078	-4275.064357	-4275.253247	1314.1*i*
**TS4-S2**	1.245022	1.321265	1.130993	-4276.381787	-4275.060522	-4275.250794	1357.6*i*
**TS4-S3**	1.244956	1.321388	1.130972	-4276.381302	-4275.059914	-4275.250330	1316.7*i*
**TS4-S4**	1.245465	1.321689	1.130865	-4276.380339	-4275.058650	-4275.249474	1284.5*i*
**TS4-S5**	1.245884	1.321593	1.134984	-4276.38408	-4275.062487	-4275.249096	1380*i*
**TS4-S6**	1.245292	1.32159	1.132003	-4276.380884	-4275.059294	-4275.248881	1336.2*i*
**TS4-S7**	1.24615	1.32212	1.133801	-4276.380754	-4275.058634	-4275.246953	1362.5*i*
**TS4-S8**	1.245578	1.321901	1.130811	-4276.377002	-4275.055101	-4275.246191	1333.6*i*
**TS4-S9**	1.245802	1.321744	1.133648	-4276.379431	-4275.057687	-4275.245783	1266.4*i*
**Int5-C1**	1.251131	1.327240	1.137646	-4276.401311	-4275.074071	-4275.263665	
**Int5-C2**	1.252115	1.327972	1.140814	-4276.409484	-4275.081512	-4275.268670	

**TS6**	1.250684	1.325804	1.140088	-4276.398927	-4275.073123	-4275.258839	389.7*i*
**TS6-S1**	1.249351	1.324677	1.136374	-4276.393972	-4275.069295	-4275.257598	381.8*i*
**TS6-S2**	1.249548	1.324942	1.136122	-4276.393144	-4275.068202	-4275.257022	376.2*i*
**TS6-S3**	1.250539	1.325621	1.139009	-4276.39391	-4275.068289	-4275.254901	371.3*i*
**TS6-S4**	1.250128	1.325436	1.136877	-4276.390578	-4275.065142	-4275.253701	388.8*i*
**TS6-S5**	1.250522	1.325607	1.139936	-4276.390048	-4275.064441	-4275.250112	380.4*i*
**TS6-S6**	1.251781	1.326905	1.141856	-4276.383194	-4275.056289	-4275.241338	336.7i
**Int7**	1.251636	1.327338	1.139571	-4276.422488	-4275.095150	-4275.282917	
**TS8**	1.253971	1.328032	1.144719	-4276.405435	-4275.077403	-4275.260716	223.8*i*
**TS8-S1**	1.2542	1.328215	1.145164	-4276.403124	-4275.074909	-4275.257960	212.2*i*
**TS8-S2**	1.253922	1.327876	1.145717	-4276.403641	-4275.075765	-4275.257924	179.1*i*
**TS8-S3**	1.254772	1.328634	1.146802	-4276.4019	-4275.073266	-4275.255098	231.7*i*
**TS8-S4**	1.254272	1.328338	1.144819	-4276.397018	-4275.068680	-4275.252199	281.5*i*
**TS8-S5**	1.253242	1.327743	1.140011	-4276.391151	-4275.063408	-4275.251140	227.3*i*
**Int9**	1.255445	1.329935	1.144603	-4276.410415	-4275.080480	-4275.265812	
**TS10**	1.250729	1.324789	1.141724	-4276.405437	-4275.080648	-4275.263713	993.1*i*
**TS10-S1**	1.250766	1.325034	1.141182	-4276.40281	-4275.077776	-4275.261628	935.9*i*
**TS10-S2**	1.250153	1.324539	1.139532	-4276.400529	-4275.075990	-4275.260997	1070.9*i*
**TS10-S3**	1.250253	1.324633	1.140253	-4276.399711	-4275.075078	-4275.259458	1044.2*i*
**TS10-S4**	1.250475	1.32471	1.140504	-4276.398837	-4275.074127	-4275.258333	1032.5*i*
**Int11**	1.253988	1.328579	1.14193	-4276.411239	-4275.082660	-4275.269309	
**TS12**	1.249657	1.323557	1.139069	-4276.401675	-4275.078118	-4275.262606	1602.5*i*
**TS12-S1**	1.249672	1.323535	1.139858	-4276.401322	-4275.077787	-4275.261464	1593*i*
**TS12-S2**	1.249517	1.323565	1.138088	-4276.398487	-4275.074922	-4275.260399	1565.1*i*
**TS12-S3**	1.248705	1.322716	1.137983	-4276.396332	-4275.073616	-4275.258349	1480.4*i*
**TS12-S4**	1.249292	1.323121	1.139256	-4276.39656	-4275.073439	-4275.257304	1524.7*i*
**Int13**	1.254559	1.329194	1.142022	-4276.438221	-4275.109027	-4275.296199	
**TS14-C1**	1.245694	1.321643	1.133581	-4276.385042	-4275.063399	-4275.251461	1137.8*i*
**TS14-C2**	1.245355	1.321827	1.13051	-4276.381054	-4275.059227	-4275.250544	1480.9*i*
**TS14-S1**	1.245583	1.321882	1.131641	-4276.380879	-4275.058997	-4275.249238	1487.9*i*
**TS14-S2**	1.24551	1.321908	1.13082	-4276.379734	-4275.057826	-4275.248914	1488.1*i*
**TS14-S3**	1.245308	1.321651	1.131342	-4276.378116	-4275.056465	-4275.246774	1433.9*i*
**TS14-S4**	1.244798	1.321451	1.129478	-4276.373032	-4275.051581	-4275.243554	1217.3*i*
**TS15**	1.251749	1.326507	1.139422	-4276.379275	-4275.052768	-4275.239853	44.3*i*
**TS16**	1.251983	1.326979	1.14147	-4276.376621	-4275.049642	-4275.235151	16.3*i*

Cartesian Coordinates for Calculated Species

1a

C	-1.27537100	-1.83945000	0.07031500
C	-0.08743700	-1.70010300	-0.16444900
C	1.28127400	-1.47239500	-0.44168800
C	1.68184200	-0.20057400	-0.90278700
N	0.74335100	0.77644100	-1.13399000
H	-0.19539500	0.56421000	-0.82005000
C	1.06506700	2.17813200	-1.28607800
C	1.67099000	2.85430400	-0.06316900
C	1.61929100	2.26673500	1.20369400
C	2.14616800	2.92894700	2.31398900
C	2.73147600	4.18651000	2.17008600
C	2.79039400	4.77799300	0.90647200
C	2.26598400	4.11305300	-0.20037100
H	2.31567300	4.57691400	-1.18350400
H	3.24805300	5.75579400	0.78308200
H	3.14298000	4.70087500	3.03388500
H	2.10224700	2.45678500	3.29159400
H	1.17400700	1.28406100	1.31897800
H	1.73469700	2.31284200	-2.14565700
H	0.13369200	2.68926700	-1.54812400
C	3.06477100	0.04366000	-1.13617700
C	3.99659600	-0.94359100	-0.94463400
C	3.62965800	-2.24277700	-0.50506900
C	4.58787900	-3.26756000	-0.30722300
C	4.20809200	-4.51744200	0.12897800
C	2.84334400	-4.78361500	0.38525000
C	1.88866000	-3.80659800	0.20278800
C	2.25021000	-2.51187800	-0.24761500
H	0.84193000	-4.01422700	0.40078900
H	2.54430700	-5.76983500	0.72992300
H	4.95004300	-5.29665600	0.27684900
H	5.63351000	-3.04575900	-0.50744800
H	5.04741700	-0.73221100	-1.12615600
H	3.38392000	1.02866100	-1.45475900
C	-2.65782100	-2.06456100	0.31232800
C	-3.10191200	-3.37205000	0.59411900
C	-4.45259600	-3.64564200	0.77309300
C	-5.39061400	-2.61655200	0.66958500
C	-4.96338300	-1.31768500	0.39999400
C	-3.60837400	-1.01332300	0.22727700
C	-3.19699300	0.38371300	-0.05087100
C	-3.78465900	1.10404700	-1.09837400
C	-3.40725000	2.41715900	-1.37378400
C	-2.43392300	3.03834400	-0.58793800

C	-1.85254800	2.34647800	0.48017500
C	-2.22869300	1.03242100	0.73445700
H	-1.76917400	0.49811800	1.55959200
H	-1.10268400	2.84431000	1.08482000
O	-1.99030900	4.31086500	-0.82677800
H	-2.48272900	4.67933500	-1.57674600
H	-3.85959600	2.95204300	-2.20669800
H	-4.52916000	0.61976100	-1.72335600
H	-5.68731700	-0.50978500	0.34191900
H	-6.44817300	-2.82182600	0.80742300
H	-4.77343300	-4.66081500	0.98812400
H	-2.36652800	-4.16807000	0.65344300

2a

O	-2.65803700	-2.48191300	1.85080000
N	-0.91201600	0.10461900	-0.73859700
C	3.90585000	3.71433000	0.63389100
H	4.95891700	3.71389500	0.90183500
C	3.20709600	4.93915500	0.54869400
H	3.72506700	5.87541500	0.73438700
C	1.86323700	4.93175000	0.25041500
H	1.30140800	5.86165100	0.20515000
C	3.26534000	2.51740200	0.39452200
H	3.81204900	1.58850600	0.49856100
C	1.88811700	2.47577300	0.04940200
C	1.17651300	3.71681100	0.00623000
C	-0.21718400	3.69167000	-0.25580200
H	-0.76432900	4.63085600	-0.26742400
C	-0.88630700	2.51503500	-0.46587400
H	-1.95947500	2.53093300	-0.61203400
C	-0.19472200	1.26779600	-0.46801100
C	1.19056100	1.25275400	-0.22473200
C	2.75607200	-4.65680400	-0.09659500
H	2.63464700	-5.71577800	0.11087000
C	3.94229600	-4.19172900	-0.67150400
H	4.73320400	-4.89639600	-0.91286200
C	1.72430200	-3.76481100	0.22586900
H	0.82258100	-4.12917900	0.70865500
C	4.13199000	-2.83011000	-0.92140700
H	5.06553900	-2.47079700	-1.34553000
C	1.89287100	-2.41729100	-0.05554700
C	3.10529400	-1.93922600	-0.60254100
C	3.02822300	-0.48800600	-0.71807700
H	3.80682800	0.12762400	-1.15226500
C	1.82659100	-0.04862900	-0.26481300
C	0.97101200	-1.22702400	0.19009900

C	0.63678300	-1.12310800	1.65979100
H	1.42947600	-0.73979900	2.29813100
C	-0.53678100	-1.50668800	2.18410300
H	-0.73816400	-1.43381100	3.24912100
C	-1.60951600	-2.09226200	1.36011300
C	-1.32447200	-2.25108400	-0.12867400
H	-2.27945600	-2.20759200	-0.65584900
H	-0.92696400	-3.26022100	-0.28999000
C	-0.30839400	-1.23821900	-0.67676700
H	-0.01537700	-1.54540000	-1.69289000
C	-2.08768600	0.20071500	-1.60064500
H	-2.07469100	-0.65281900	-2.29027900
H	-1.98285700	1.08806700	-2.23659700
C	-3.42478300	0.24255800	-0.88101300
C	-4.60228900	0.05312200	-1.61456500
H	-4.54384500	-0.14127200	-2.68383000
C	-5.84470600	0.10527400	-0.98703600
H	-6.75033300	-0.04823700	-1.56739800
C	-5.92451700	0.34430200	0.38714800
H	-6.89212000	0.37668900	0.87965000
C	-4.75593600	0.53101500	1.12301900
C	-3.51238300	0.48377400	0.49119400
H	-2.60176800	0.61677800	1.06510800
H	-4.80677300	0.70439000	2.19393700

anion-CPA-A4

C	-2.38861400	3.61158300	1.79742000
C	-1.07992800	3.66673900	1.21533100
C	-0.66275200	2.61767700	0.33505300
C	-1.49803500	1.52079500	0.14111600
C	-2.80161400	1.46465500	0.71481800
C	-3.22632400	2.50494600	1.51099000
C	-2.81187200	4.65543500	2.66091500
C	-1.97844500	5.70611800	2.96825100
C	-0.67216800	5.74457100	2.42591300
C	-0.23549600	4.75286800	1.57663400
C	1.07976300	3.66670200	-1.21532800
C	2.38839700	3.61154400	-1.79754100
C	3.22618800	2.50496900	-1.51109000
C	2.80163500	1.46475700	-0.71473500
C	1.49812900	1.52091700	-0.14085100
C	0.66275100	2.61770700	-0.33488000
C	0.23524700	4.75273900	-1.57668900
C	0.67179700	5.74436200	-2.42613300
C	1.97801800	5.70591500	-2.96858800
C	2.81152800	4.65530500	-2.66120100

C	5.11534400	-2.68104700	-2.21459300
C	4.43333100	-2.61527100	-3.39945700
C	3.46653500	-1.59623800	-3.62140700
C	3.21946400	-0.65722400	-2.65864600
C	3.93355900	-0.66436200	-1.41966400
C	3.70368600	0.31205500	-0.43227900
C	4.36585000	0.23676600	0.80937500
C	4.13800500	1.20111500	1.84013400
C	4.79183900	1.12934900	3.04039200
C	5.72903700	0.08782200	3.28342900
C	5.98002700	-0.85547100	2.32324600
C	5.30907300	-0.82650000	1.05922000
C	5.56733600	-1.78248000	0.05563300
C	4.89294500	-1.71709600	-1.18001700
C	6.54772500	-2.87735300	0.31015800
C	6.19591200	-3.97764100	1.10438600
C	7.10981500	-5.00334700	1.34596400
C	8.39102400	-4.94593500	0.79470800
C	8.75188600	-3.85620700	0.00064800
C	7.83674300	-2.83078500	-0.23799900
C	-5.11526800	-2.68126400	2.21446900
C	-4.43331100	-2.61548400	3.39936600
C	-3.46659800	-1.59639700	3.62140900
C	-3.21951500	-0.65735700	2.65867800
C	-3.93355000	-0.66450200	1.41966100
C	-3.70362500	0.31192300	0.43230600
C	-4.36573400	0.23667300	-0.80938300
C	-4.13782500	1.20105000	-1.84010200
C	-4.79165000	1.12937400	-3.04037000
C	-5.72892700	0.08793000	-3.28343600
C	-5.97996600	-0.85539600	-2.32329600
C	-5.30897900	-0.82656300	-1.05928500
C	-5.56728000	-1.78258800	-0.05573200
C	-4.89291900	-1.71724500	1.17994400
C	-6.54768500	-2.87742200	-0.31031200
C	-6.19628500	-3.97713300	-1.10552800
C	-7.11021500	-5.00280000	-1.34716200
C	-8.39104700	-4.94594000	-0.79497400
C	-8.75149900	-3.85679800	0.00007600
C	-7.83633300	-2.83141200	0.23877400
O	-1.09572100	0.49685700	-0.65028700
O	1.09598800	0.49709300	0.65077000
P	0.00012100	-0.65286400	0.00034500
O	-0.61697100	-1.30698100	1.18513700
O	0.61728500	-1.30724500	-1.18423900
H	-4.22201200	2.47110300	1.94633800

H	-3.81209400	4.59958000	3.08504700
H	-2.31370600	6.49670100	3.63463400
H	-0.00385900	6.56093100	2.68796200
H	0.77094800	4.78624100	1.17655800
H	4.22181100	2.47111500	-1.94658700
H	-0.77116400	4.78613400	-1.17653900
H	0.00341800	6.56065500	-2.68821100
H	2.31317500	6.49643200	-3.63510000
H	3.81171100	4.59944600	-3.08542300
H	5.82986500	-3.47733000	-2.03841400
H	4.61322600	-3.36192800	-4.16921100
H	2.89806300	-1.58524900	-4.54690700
H	2.44277000	0.08211500	-2.79956000
H	3.42256400	1.99381800	1.65567800
H	4.59342900	1.86789200	3.81239400
H	6.24837200	0.04254500	4.23748900
H	6.69895400	-1.64544300	2.51030600
H	5.19654400	-4.01987200	1.52732900
H	6.81895400	-5.85001200	1.96242800
H	9.10302900	-5.74572200	0.98163100
H	9.74766000	-3.80318200	-0.43247100
H	8.11352100	-1.98117400	-0.85582000
H	-5.82968800	-3.47762200	2.03823400
H	-4.61317800	-3.36219900	4.16907000
H	-2.89818000	-1.58540000	4.54694100
H	-2.44283800	0.08199100	2.79962500
H	-3.42234400	1.99370700	-1.65560600
H	-4.59318800	1.86793300	-3.81234300
H	-6.24830600	0.04275000	-4.23747700
H	-6.69898400	-1.64527400	-2.51039100
H	-5.19720400	-4.01895300	-1.52919100
H	-6.81966200	-5.84901200	-1.96439300
H	-9.10307200	-5.74570000	-0.98193800
H	-9.74697900	-3.80419800	0.43392300
H	-8.11280000	-1.98225800	0.85736100

Int3

C	-1.69391900	0.38565200	-1.49023100
H	-0.02839400	-0.62524200	-1.23308000
O	0.96209800	-0.70809700	-1.26500500
C	3.42456600	-2.05697500	-0.20680700
C	3.01648500	-3.31438600	-0.72561900
C	3.89091900	-3.96988900	-1.56358900
C	5.98259300	-4.04664800	-2.87484300
C	7.14420400	-3.45210700	-3.30819100
C	7.48876800	-2.16106300	-2.84503000

C	6.68622400	-1.50212100	-1.94135500
C	6.16693400	0.09779200	0.85389500
C	6.42010000	1.40684300	1.38142300
C	5.44627800	2.42833000	1.23079000
C	4.24801800	2.19002200	0.59702000
C	4.04900300	0.90572200	0.02678300
C	4.96026700	-0.13195200	0.10928300
C	7.10858900	-0.92993100	1.12789900
C	8.25866300	-0.66737900	1.83739700
C	8.52923900	0.63548700	2.31592400
C	7.62314100	1.64620600	2.09648100
C	0.18107700	4.05552000	2.72898800
C	0.51075900	3.41660300	3.89362100
C	1.71636900	2.67044800	3.98553200
C	2.55913400	2.58585900	2.91085700
C	2.26862300	3.25925700	1.68285200
C	3.11479700	3.15793400	0.56033700
C	2.82049200	3.86026100	-0.62219800
C	3.69234600	3.82065200	-1.75438800
C	3.42475300	4.54866200	-2.88302300
C	2.25842100	5.35965600	-2.95087000
C	1.37609100	5.39171300	-1.90414800
C	1.61027600	4.64045700	-0.70952900
C	0.70487300	4.65916000	0.37478500
C	1.04146900	4.01755100	1.58716500
C	-0.63265700	5.29178600	0.19572700
C	-1.53332900	4.72362500	-0.71881000
C	-2.79672400	5.27873500	-0.91486300
C	-3.18127700	6.41232600	-0.19480500
C	-2.29363500	6.98500200	0.71798500
C	-1.02733500	6.43079100	0.90962700
C	-1.78316700	-3.96480200	-1.86373200
C	-1.54999600	-3.44797200	-3.10897700
C	-0.22946400	-3.10364300	-3.50780200
C	0.81587500	-3.25643400	-2.63891900
C	0.61907700	-3.77391300	-1.31883600
C	1.66820900	-3.85080100	-0.37937800
C	1.43388100	-4.36378000	0.91153300
C	2.48145600	-4.46151000	1.87990500
C	2.25607900	-4.99064500	3.12154500
C	0.96472700	-5.47150000	3.47079300
C	-0.06775800	-5.39303600	2.57527400
C	0.11426200	-4.82118900	1.27587000
C	-0.94868700	-4.70725400	0.35294600
C	-0.71523000	-4.17187300	-0.93221000
C	-2.31666500	-5.15838700	0.72981500

C	-3.03411100	-4.50525700	1.73995800
C	-4.31313900	-4.93336600	2.09080100
C	-4.89835400	-6.01475500	1.43293000
C	-4.19356000	-6.67136300	0.42268900
C	-2.91176400	-6.24785000	0.07648800
O	2.52959500	-1.40946400	0.65046700
O	2.85278500	0.70858800	-0.66386000
P	1.68403200	-0.16978300	0.03617700
C	4.63208000	-1.44864000	-0.50451300
O	0.84383900	0.48695400	1.05879100
H	3.61392000	-4.94004700	-1.96636000
H	5.69281100	-5.02828200	-3.24047300
H	7.78998300	-3.96338100	-4.01601800
H	8.39234900	-1.68174800	-3.21042900
H	6.95661600	-0.50967100	-1.60221800
H	5.63193200	3.40406300	1.67083200
H	6.90964500	-1.93443900	0.77532200
H	8.96190500	-1.47074000	2.03682100
H	9.44356500	0.83015900	2.86869800
H	7.80541900	2.64649700	2.48053200
H	-0.75573000	4.59120400	2.66060600
H	-0.16890800	3.45851500	4.73902200
H	1.96235000	2.15367700	4.90911900
H	3.46537800	1.99616300	2.97907300
H	4.59171000	3.21702200	-1.69312100
H	4.11011900	4.51802000	-3.72548600
H	2.06489300	5.95065200	-3.84176600
H	0.48331400	6.00245400	-1.96469200
H	-1.23499400	3.84257300	-1.27379400
H	-3.47909000	4.81461700	-1.62053000
H	-4.16648000	6.84572400	-0.34316500
H	-2.58426100	7.86890000	1.27912800
H	-0.33529600	6.88261900	1.61441000
H	-2.79425300	-4.20158200	-1.55762600
H	-2.37912100	-3.28856100	-3.79246200
H	-0.05509900	-2.69302100	-4.49817200
H	1.81205200	-2.94885900	-2.93134500
H	3.47256200	-4.11497000	1.60926700
H	3.06800800	-5.05528400	3.84039200
H	0.80070500	-5.90979400	4.45117900
H	-1.04727200	-5.77112500	2.84188200
H	-2.59007100	-3.64957700	2.23692400
H	-4.85583100	-4.40854000	2.87052500
H	-5.89839000	-6.34252300	1.70265500
H	-4.64088700	-7.51548200	-0.09494800
H	-2.36015600	-6.75870100	-0.70737400

C	5.49562700	-2.10017200	-1.44867900
C	5.12413900	-3.38840400	-1.95514400
C	-2.59533100	2.13234600	-2.96585400
C	0.01663700	2.83970600	-3.75893100
H	0.68557600	1.30651500	-2.42091500
C	-2.37251100	3.13968900	-3.91139000
C	-1.08407700	3.49809700	-4.30555400
H	1.02469800	3.12042000	-4.04060700
H	-3.23192500	3.66445700	-4.31912900
H	-0.94156500	4.29786500	-5.02664900
C	-3.97923300	1.78353400	-2.56083700
C	-4.36445600	1.76904000	-1.21210800
C	-4.93368700	1.42341000	-3.52366400
C	-5.63204800	1.34699600	-0.83069800
H	-3.66022800	2.07498900	-0.44819500
C	-6.20611400	0.99839800	-3.15425700
H	-4.65546200	1.43061000	-4.57401500
C	-6.54545300	0.92329900	-1.80110800
H	-5.89774800	1.31507500	0.22293400
H	-6.93002800	0.67528300	-3.89516100
C	-3.06089300	-1.01571900	0.26452700
C	-4.27927600	-1.62407100	-0.19452300
C	-2.82586800	-0.83431600	1.64483300
C	-4.53392400	-1.86479800	-1.56631700
C	-5.30262100	-1.93465400	0.75041000
C	-3.87422900	-1.14621800	2.56252400
C	-5.73788700	-2.39262900	-1.97949600
H	-3.76978200	-1.60634200	-2.28958200
C	-6.53085200	-2.47601300	0.29696900
C	-5.06537200	-1.65821000	2.12421500
H	-3.72392100	-0.95144500	3.61750200
C	-6.75081300	-2.70338000	-1.04473100
H	-5.91593100	-2.55759100	-3.03802200
H	-7.29515900	-2.71783500	1.03242800
H	-5.85555000	-1.87008400	2.84082900
H	-7.69566100	-3.11543400	-1.38560800
O	-7.78058800	0.43097500	-1.47924900
H	-7.74233600	0.08424700	-0.57368400
N	-1.63343500	-0.35204300	2.09550300
H	-0.91991000	-0.13033200	1.40695300
C	-1.49501000	0.41101500	3.32434600
H	-1.68255200	-0.22259700	4.20071000
H	-0.44262900	0.70863000	3.36767500
C	-2.38011200	1.64389900	3.37528500
C	-2.60228900	2.40052900	2.21912200
C	-2.95332700	2.06336800	4.57762400

C	-3.38211800	3.55394400	2.26445000
H	-2.14005400	2.09319400	1.28586600
C	-3.72661000	3.22545400	4.63150900
H	-2.79220100	1.47726200	5.48039600
C	-3.94410500	3.97186400	3.47332400
H	-3.53342000	4.13652300	1.36246200
H	-4.16443500	3.54035000	5.57509600
H	-4.54718600	4.87488000	3.50905300
C	-1.46689200	1.44908800	-2.42512700
C	-2.24564800	-0.34492700	-0.67609300
C	-0.17329900	1.82144800	-2.83087700

TS4

C	-1.37531700	0.22170800	-1.83217700
H	-0.15391400	-0.42512900	-1.66833800
O	0.98183300	-0.76303900	-1.49563200
C	3.19844500	-2.21023600	-0.06221600
C	2.75327200	-3.48527600	-0.49954600
C	3.63865500	-4.26878100	-1.20618400
C	5.81374200	-4.59393100	-2.33306900
C	7.04952500	-4.11922100	-2.70508500
C	7.45410700	-2.82366200	-2.30706900
C	6.63305800	-2.04070700	-1.52745200
C	6.01111900	-0.24358900	1.12760200
C	6.32866400	1.06310000	1.62429500
C	5.45960100	2.14877000	1.34568400
C	4.30849200	1.97636400	0.61091300
C	4.04994100	0.69183400	0.06359300
C	4.86324500	-0.40770300	0.27964400
C	6.83187800	-1.33041100	1.53314100
C	7.93182000	-1.12917400	2.33617000
C	8.27093800	0.16868700	2.78379800
C	7.47842300	1.23804800	2.43880600
C	0.48429100	4.49844300	2.50627700
C	0.68460100	3.87677500	3.70890200
C	1.74232100	2.94048800	3.86284500
C	2.58013300	2.66971200	2.81516000
C	2.42421400	3.31610100	1.54791600
C	3.27149100	3.03303400	0.45790800
C	3.06909900	3.65743600	-0.78632400
C	3.93322000	3.40777400	-1.89704100
C	3.73640100	4.02390200	-3.10324900
C	2.65847300	4.93588000	-3.27194700
C	1.79632400	5.18878100	-2.23880400
C	1.95848600	4.55952800	-0.96409500
C	1.08000900	4.81246600	0.11196000

C	1.32603700	4.23928400	1.37897300
C	-0.15641100	5.60831000	-0.12612700
C	-1.15031900	5.08292000	-0.96688500
C	-2.33101200	5.78672300	-1.19933400
C	-2.53699800	7.02903500	-0.59520000
C	-1.55213900	7.56345200	0.23784700
C	-0.36966900	6.85903800	0.46845000
C	-1.91000400	-4.45451700	-1.95594300
C	-1.57728200	-4.09163500	-3.23296900
C	-0.25709300	-3.66157600	-3.53795300
C	0.69576400	-3.61166900	-2.55791600
C	0.39967200	-3.99746800	-1.21203900
C	1.36703300	-3.93493900	-0.18935400
C	1.02973100	-4.28165600	1.13467300
C	1.99174200	-4.23263500	2.19181200
C	1.66277900	-4.58481700	3.47240100
C	0.34672700	-5.02737600	3.77684600
C	-0.60404400	-5.09535300	2.79435100
C	-0.31116000	-4.71515000	1.44548500
C	-1.28237400	-4.78289000	0.42270800
C	-0.94396000	-4.42843100	-0.90038500
C	-2.66456000	-5.23530500	0.74507400
C	-3.50391200	-4.44827500	1.54378000
C	-4.79043300	-4.87963700	1.86165100
C	-5.26205800	-6.10036600	1.37971700
C	-4.43645000	-6.89044200	0.57807800
C	-3.14706000	-6.46197200	0.26630800
O	2.28556400	-1.42878300	0.63641700
O	2.93182400	0.57029200	-0.74432600
P	1.59335100	-0.19624500	-0.19406700
C	4.47649200	-1.72612700	-0.28974600
O	0.73607900	0.60469400	0.72148800
H	3.32636900	-5.25367000	-1.54205200
H	5.48020100	-5.57880600	-2.64981600
H	7.70978100	-4.72908400	-3.31484800
H	8.41947600	-2.44010200	-2.62471500
H	6.94966500	-1.04612600	-1.23735700
H	5.68226100	3.12498200	1.76750600
H	6.57973200	-2.33155400	1.20644100
H	8.54146200	-1.97750200	2.63389000
H	9.14538500	0.31425500	3.41134600
H	7.71017300	2.23785200	2.79701900
H	-0.34150700	5.18762100	2.39290800
H	0.01703800	4.08341100	4.54039400
H	1.88119400	2.43323000	4.81348500
H	3.37448800	1.94337600	2.93320500

H	4.76239200	2.72193700	-1.76216100
H	4.40918200	3.82463800	-3.93257700
H	2.52087100	5.43554100	-4.22699000
H	0.97659600	5.88399000	-2.37446000
H	-0.98783800	4.11964700	-1.43916000
H	-3.08988500	5.35583400	-1.84489400
H	-3.45764000	7.57752800	-0.77383800
H	-1.70162900	8.53235500	0.70635400
H	0.39835100	7.27788200	1.11224100
H	-2.92558700	-4.74769200	-1.72255500
H	-2.33086000	-4.11474200	-4.01579400
H	-0.01065700	-3.35289900	-4.54980500
H	1.68900300	-3.24632700	-2.78411300
H	3.00110400	-3.91210200	1.96146600
H	2.41103300	-4.53609200	4.25845700
H	0.09937900	-5.32255000	4.79281200
H	-1.60090300	-5.44916400	3.02831800
H	-3.14392500	-3.49122300	1.90756500
H	-5.42790300	-4.25324300	2.47859900
H	-6.26686500	-6.43344100	1.62431800
H	-4.79467000	-7.84356800	0.19870400
H	-2.50025800	-7.07783100	-0.35182300
C	5.36220800	-2.51127100	-1.10115200
C	4.93620400	-3.80608600	-1.54233200
C	-2.46400700	2.18761700	-2.92198700
C	-0.03358900	2.83193700	-4.18432200
H	0.78408300	1.14165000	-3.12745400
C	-2.38027100	3.26302900	-3.80946400
C	-1.17656500	3.58810800	-4.43893600
H	0.91303900	3.09422000	-4.64365500
H	-3.26825800	3.86298800	-3.98810600
H	-1.13159900	4.43957400	-5.11206300
C	-3.71386200	1.92304000	-2.15794100
C	-3.83217500	2.43297500	-0.85861500
C	-4.75911800	1.13668300	-2.66102800
C	-4.93629400	2.14225100	-0.06666900
H	-3.03605700	3.04280400	-0.45316000
C	-5.87409600	0.84167100	-1.88167400
H	-4.68139400	0.72285500	-3.66208800
C	-5.95354200	1.33028200	-0.57507700
H	-4.99068500	2.52851500	0.94826300
H	-6.66322100	0.19538900	-2.24999400
C	-3.09418700	-0.85155400	-0.15376500
C	-4.22201000	-1.65355900	-0.57938600
C	-2.98790300	-0.41259100	1.20754500
C	-4.31423700	-2.18043100	-1.88375200

C	-5.29644900	-1.87186200	0.32269900
C	-4.09599900	-0.65605900	2.08204700
C	-5.42811800	-2.89867700	-2.27296800
H	-3.49221300	-2.01700600	-2.57040600
C	-6.43313400	-2.59344800	-0.10358600
C	-5.19698200	-1.33663300	1.64042700
H	-4.06264700	-0.28129500	3.09635100
C	-6.50199500	-3.10559200	-1.38234600
H	-5.47784800	-3.30672600	-3.27891600
H	-7.25108400	-2.74130500	0.59647400
H	-6.03081600	-1.49609500	2.32049500
H	-7.37399300	-3.66756000	-1.70242600
O	-7.03985500	0.97189700	0.17583400
H	-6.91602300	1.32348300	1.07121600
N	-1.88081900	0.21389200	1.63210900
H	-1.06801300	0.28878600	1.01312900
C	-1.71676500	0.87587700	2.91981600
H	-1.95458600	0.18661600	3.73927800
H	-0.65111700	1.11228900	2.98864200
C	-2.54434400	2.14126900	3.03330200
C	-2.35576300	3.16931200	2.10314800
C	-3.50299700	2.30269000	4.03540800
C	-3.11462000	4.33525500	2.16753700
H	-1.60229600	3.05039600	1.33165900
C	-4.27070400	3.46874200	4.10508300
H	-3.65007300	1.51317200	4.76978500
C	-4.07897300	4.48487900	3.16903500
H	-2.95366900	5.12307000	1.43874100
H	-5.01448900	3.58069600	4.88938200
H	-4.67373000	5.39272100	3.21967400
C	-1.30784500	1.39330000	-2.69925800
C	-2.23906100	-0.30609800	-1.08120300
C	-0.10093400	1.73652800	-3.32667300

TS4-S1

O	0.45963100	-0.55993300	-2.02746700
H	-0.27112300	0.36999500	-2.15410600
C	-0.89353000	1.63470000	-2.10267500
C	-3.17495500	1.08761800	0.43047800
C	-3.24360300	1.26910800	-0.99036500
C	0.04105100	2.73493300	-2.31037500
C	2.85219200	-6.06908200	-3.50332200
C	4.17650500	-6.14885800	-3.86631400
C	5.11542200	-5.25036400	-3.30813300
C	4.72065800	-4.30756100	-2.38608000
C	4.86192200	-2.97186500	0.61224800

C	5.70760700	-2.10644500	1.38081200
C	5.46918000	-0.70893600	1.37474400
C	4.44448800	-0.15680900	0.64026900
C	3.67341700	-1.01802800	-0.18211700
C	3.84910300	-2.39116900	-0.22681800
C	5.04185200	-4.37494900	0.75275200
C	6.03417900	-4.88770000	1.55738100
C	6.89985400	-4.02921400	2.27361700
C	6.73014500	-2.66753500	2.19043700
C	1.88580100	3.35791300	3.02487800
C	1.38789300	2.39685300	3.86468100
C	1.75095000	1.03172100	3.69550700
C	2.61736000	0.67187400	2.70099600
C	3.17140500	1.64162000	1.80636200
C	4.06440700	1.27893600	0.77904600
C	4.57499800	2.24939000	-0.10047500
C	5.45751000	1.89875600	-1.16940800
C	5.96111000	2.84960600	-2.01598200
C	5.60777200	4.21747400	-1.84682600
C	4.75395800	4.59319300	-0.84440900
C	4.20524500	3.63481300	0.06563800
C	3.30893400	4.00339400	1.09117900
C	2.78347600	3.02319200	1.96045400
C	2.89753800	5.42725500	1.24663300
C	1.59197500	5.81866200	0.92229000
C	1.18817100	7.14536100	1.05955300
C	2.08865200	8.10484100	1.52597700
C	3.39177700	7.72677000	1.85428800
C	3.79282600	6.39742900	1.71668800
C	-2.91999800	-4.92619700	2.01540600
C	-2.04884900	-5.74870200	2.67847800
C	-0.72063600	-5.91989800	2.20205100
C	-0.31556600	-5.29232800	1.05385300
C	-1.20841600	-4.46094600	0.30559600
C	-0.82564200	-3.87752200	-0.91786300
C	-1.75205100	-3.13556400	-1.67274300
C	-1.42165200	-2.63270200	-2.96912200
C	-2.32302100	-1.90761900	-3.69799100
C	-3.59773900	-1.60047000	-3.15273200
C	-3.94291900	-2.03311100	-1.90076700
C	-3.06214300	-2.85346700	-1.12752900
C	-3.42401000	-3.37416200	0.13647300
C	-2.53718500	-4.23661700	0.82146000
C	-4.70923800	-2.98838200	0.78144000
C	-5.95553300	-3.25706300	0.19714300
C	-7.13641700	-2.85741100	0.82276400

C	-7.09231000	-2.19680700	2.05235300
C	-5.85764900	-1.93924500	2.65059700
C	-4.67818600	-2.32530400	2.01811200
O	1.07082800	-2.08234800	-0.01411100
O	2.71462400	-0.42459700	-0.98731600
P	1.12408000	-0.56015800	-0.63046100
C	2.91702300	-3.23638500	-1.02167800
O	0.62803300	0.39421500	0.39517700
H	0.32778900	-5.70299800	-2.62286200
H	2.11771500	-6.74281200	-3.93708800
H	4.50207500	-6.89172600	-4.58865400
H	6.15642100	-5.30114800	-3.61406100
H	5.44496300	-3.61749700	-1.96925600
H	6.07628900	-0.06699300	2.00695200
H	4.38233300	-5.04858600	0.22097400
H	6.14822100	-5.96419200	1.64770100
H	7.68272200	-4.44745300	2.89954100
H	7.36782600	-1.99369800	2.75682900
H	1.61281400	4.39710100	3.16572800
H	0.71382100	2.67699100	4.67004500
H	1.32576600	0.27414800	4.34671600
H	2.88065000	-0.37023700	2.56616300
H	5.72453400	0.85501000	-1.29541100
H	6.63162000	2.56198300	-2.82093100
H	6.01075800	4.96449000	-2.52515900
H	4.47265700	5.63338400	-0.73149100
H	0.90285300	5.07430700	0.54391200
H	0.17092800	7.42600500	0.79949400
H	1.77750900	9.14017400	1.63384300
H	4.09743300	8.46707200	2.22127100
H	4.80573100	6.10199800	1.97521000
H	-3.92873800	-4.79562400	2.38766000
H	-2.37177800	-6.27149800	3.57441500
H	-0.03051000	-6.56036600	2.74413200
H	0.69104500	-5.44015000	0.67949300
H	-0.43173200	-2.82912600	-3.36086400
H	-2.05538700	-1.54122400	-4.68484800
H	-4.29398800	-0.99182600	-3.71883600
H	-4.90046600	-1.74984500	-1.48348400
H	-5.99525800	-3.78538000	-0.75068900
H	-8.09267100	-3.07278400	0.35385800
H	-8.01390700	-1.89479700	2.54283200
H	-5.80448400	-1.43158100	3.61014000
H	-3.72120000	-2.10938600	2.47606800
C	3.36487900	-4.21118000	-1.97451100
C	2.40885500	-5.09888300	-2.56684900

C	-0.33911200	4.05877900	-1.96741500
C	2.23520200	3.53597000	-2.97168800
H	1.62483000	1.47314100	-3.03322200
C	0.57185300	5.09633800	-2.17380400
C	1.85266300	4.84094300	-2.66767300
H	3.24174900	3.32727200	-3.31765500
H	0.28360100	6.10974900	-1.91139300
H	2.55727700	5.65768900	-2.78918100
C	-1.66942100	4.30539900	-1.34680500
C	-2.80712000	4.55899000	-2.12932800
C	-1.82960600	4.19505800	0.04065000
C	-4.07047000	4.65129200	-1.55802700
H	-2.69834500	4.64316400	-3.20664100
C	-3.08908500	4.29432100	0.62522700
H	-0.96862300	3.97698200	0.66445900
C	-4.21659800	4.48779100	-0.17718700
H	-4.95639300	4.79726600	-2.16615400
H	-3.20126300	4.18145400	1.70166800
C	0.59332500	-4.00635100	-1.36026800
C	-4.52417500	1.32714400	-1.65655500
C	1.55282000	-3.10945800	-0.81847900
C	-4.63759200	1.56917300	-3.04200300
C	-5.71374600	1.16261800	-0.89652200
C	-4.39771100	0.93164800	1.15669400
C	-5.87781600	1.62897700	-3.64875300
H	-3.73032100	1.70650900	-3.62123500
C	-6.97066500	1.21521700	-1.54044600
C	-5.60067300	0.95229400	0.51078000
H	-4.35621400	0.72433100	2.21801000
C	-7.05783900	1.44478700	-2.89840800
H	-5.94380900	1.81766400	-4.71681200
H	-7.86840300	1.07762700	-0.94313000
H	-6.51014000	0.77780500	1.07928100
H	-8.02617000	1.48891200	-3.38745300
O	-5.48444000	4.51744800	0.33241800
H	-5.45022200	4.23860200	1.26038800
N	-1.99101200	1.09087700	1.05923000
H	-1.13047200	1.03288500	0.51149200
C	-1.78977500	1.15888400	2.49714600
H	-0.79848500	1.60109500	2.63278600
H	-2.51726600	1.85335600	2.93373000
C	-1.84542500	-0.17471700	3.21371600
C	-1.06489200	-1.24494700	2.75748600
C	-2.64001400	-0.34232300	4.35033600
C	-1.08527900	-2.46261700	3.43153800
H	-0.44597700	-1.11679000	1.87729100

C	-2.66261000	-1.56548200	5.02664000
H	-3.24600800	0.48749000	4.70972300
C	-1.88569100	-2.62742700	4.56549700
H	-0.48754600	-3.28810900	3.06098500
H	-3.29082800	-1.68729700	5.90497300
H	-1.91080900	-3.58593700	5.07509600
C	1.03928400	-4.99450600	-2.20783400
C	-2.06862400	1.52245700	-1.65947000
C	1.33046600	2.49137900	-2.80418900

TS4-S2

O	0.00373600	0.27105900	-1.84784500
H	0.51790500	-0.80609800	-1.70592300
C	0.78740100	-2.10509100	-1.28038100
C	2.97717200	-1.67365300	1.46646700
C	3.03021700	-2.11837100	0.10138500
C	-0.38796600	-2.95884500	-1.42293900
C	-0.49320600	6.77351400	-2.99330500
C	-1.71140600	7.40996800	-3.04973000
C	-2.81043200	6.89174800	-2.32635800
C	-2.66983700	5.76368500	-1.54968100
C	-2.88643800	4.12161400	1.28030200
C	-3.93940300	3.54017600	2.05758000
C	-4.39233400	2.23162200	1.75270200
C	-3.79190100	1.46893400	0.77636100
C	-2.71143900	2.04370000	0.05716400
C	-2.30365600	3.35762400	0.21618200
C	-2.40982200	5.40832800	1.64772100
C	-2.95606900	6.08905200	2.71280500
C	-4.01643600	5.52563800	3.46086500
C	-4.49578600	4.27784200	3.13490100
C	-3.98316900	-3.48505900	1.37895300
C	-3.01430300	-3.39841000	2.34216300
C	-2.48473400	-2.13516600	2.71900400
C	-2.91784500	-0.99228100	2.10543800
C	-3.91279600	-1.03902600	1.08079300
C	-4.32812200	0.12400800	0.40721200
C	-5.29689100	0.05128000	-0.61397300
C	-5.71084500	1.21402200	-1.33877900
C	-6.68824500	1.14923100	-2.29409300
C	-7.33456400	-0.08596600	-2.57028000
C	-6.96419000	-1.22431100	-1.90592400
C	-5.91671600	-1.21504400	-0.92935900
C	-5.50721300	-2.38599600	-0.25196800
C	-4.48947700	-2.31679200	0.72659800
C	-6.16807400	-3.68834800	-0.54667700

C	-6.11062700	-4.26765000	-1.82341100
C	-6.72990500	-5.48906100	-2.08618400
C	-7.42417000	-6.15501100	-1.07507400
C	-7.49771800	-5.58671500	0.19743300
C	-6.87702000	-4.36566400	0.45710500
C	4.36274000	0.26664100	-2.84529300
C	3.57753500	-0.02078900	-3.92947300
C	2.41347400	0.74828800	-4.20298100
C	2.04358000	1.75977600	-3.36026700
C	2.81894400	2.08077700	-2.20315800
C	2.40913200	3.07014700	-1.29144800
C	3.18358300	3.35377700	-0.14895000
C	2.76792700	4.32775100	0.81404600
C	3.52583600	4.60850600	1.91865200
C	4.76983900	3.94990400	2.11543000
C	5.20886200	3.01641900	1.21469200
C	4.42876500	2.65614100	0.06908300
C	4.84360900	1.65967600	-0.84366600
C	4.03559500	1.34094100	-1.95733700
C	6.14404200	0.96198300	-0.64791300
C	6.37596700	0.16673200	0.48088300
C	7.60141600	-0.47141500	0.66348300
C	8.61556400	-0.33001500	-0.28300200
C	8.39600600	0.45785800	-1.41451000
C	7.17210600	1.09967500	-1.59343100
O	0.17512300	2.10094000	-0.02241200
O	-2.06470000	1.24170100	-0.87224300
P	-0.56315400	0.71320500	-0.47870900
C	-1.23543000	3.91405200	-0.65824800
O	-0.49376000	-0.22953200	0.66696500
H	1.78039600	5.35348300	-2.70652500
H	0.35598800	7.15342700	-3.55558600
H	-1.83487800	8.30291700	-3.65554300
H	-3.77616400	7.38517000	-2.38847900
H	-3.52032700	5.36979200	-1.00580800
H	-5.22693900	1.81740100	2.31135800
H	-1.59203500	5.84585200	1.08773800
H	-2.56622600	7.06596700	2.98393000
H	-4.44225500	6.07552100	4.29504600
H	-5.30004100	3.82619900	3.70998400
H	-4.36733500	-4.45342000	1.08459000
H	-2.63902900	-4.30306300	2.81318100
H	-1.71187700	-2.07726300	3.47901000
H	-2.47809800	-0.03895500	2.36550100
H	-5.23246300	2.16091800	-1.11730900
H	-6.98112100	2.04484600	-2.83464700

H	-8.13151800	-0.12405100	-3.30769600
H	-7.47495100	-2.15685900	-2.11214500
H	-5.57485700	-3.75180800	-2.61346500
H	-6.66766900	-5.92065100	-3.08142100
H	-7.90642300	-7.10697600	-1.27818700
H	-8.04237700	-6.09277900	0.98979300
H	-6.93678500	-3.92372500	1.44716000
H	5.23650700	-0.33706500	-2.63358500
H	3.84100300	-0.84843600	-4.58248900
H	1.79987300	0.50986600	-5.06667400
H	1.13126400	2.31537900	-3.53973800
H	1.83138600	4.84870400	0.65230800
H	3.18392700	5.34452100	2.64058400
H	5.37902800	4.19738200	2.98016400
H	6.16672500	2.53317500	1.36314000
H	5.58326700	0.03946100	1.20937400
H	7.75814200	-1.08847000	1.54362600
H	9.56949700	-0.83055400	-0.14227900
H	9.18036600	0.57646000	-2.15700500
H	7.00203100	1.71669300	-2.47074700
C	-1.42162800	5.09313200	-1.45402300
C	-0.31537500	5.60279300	-2.21094400
C	-0.44734900	-4.20428000	-0.74910600
C	-2.62256600	-3.33063000	-2.29332000
H	-1.44811300	-1.56610900	-2.67014700
C	-1.59400600	-4.98917800	-0.87542900
C	-2.67708100	-4.56296200	-1.64515000
H	-3.46749900	-2.97566600	-2.87488000
H	-1.63749100	-5.94037200	-0.35267300
H	-3.56610900	-5.18068000	-1.71862600
C	0.69158800	-4.64355200	0.09850700
C	1.80086100	-5.28086600	-0.46976900
C	0.70984800	-4.36208400	1.47257700
C	2.91333500	-5.60455700	0.29974700
H	1.80467100	-5.49320300	-1.53457800
C	1.81665900	-4.68176400	2.25229000
H	-0.13855700	-3.84832300	1.91402400
C	2.92905700	-5.28808700	1.66020400
H	3.78499500	-6.06229200	-0.16254900
H	1.85041700	-4.44386700	3.31062300
C	1.10127600	3.77145500	-1.44796200
C	4.22982300	-2.74681300	-0.41235500
C	-0.01716100	3.26589200	-0.73442000
C	4.35443900	-3.11349600	-1.76979800
C	5.29889300	-3.05209600	0.47245900
C	4.08309300	-2.00654700	2.31943500

C	5.48657000	-3.76311100	-2.22375100
H	3.54491100	-2.87094300	-2.44950700
C	6.44313200	-3.72519200	-0.01219800
C	5.16950500	-2.67968900	1.84203700
H	4.03517400	-1.76440300	3.37262800
C	6.54089000	-4.08098600	-1.34223500
H	5.56561700	-4.03093200	-3.27378700
H	7.25336500	-3.94293900	0.67987800
H	5.97125200	-2.94401300	2.52692300
H	7.42589200	-4.58957900	-1.71228800
O	4.01830600	-5.53512400	2.45095900
H	4.75005400	-5.81403900	1.87827700
N	1.92339700	-0.99424400	1.94545500
H	1.10724500	-0.81302800	1.34514100
C	1.80160600	-0.54707900	3.33201600
H	1.52534200	-1.39069300	3.98095900
H	2.78119700	-0.18909600	3.66752600
C	0.80100400	0.57458100	3.48485000
C	-0.34705600	0.40515000	4.26111700
C	1.03262400	1.80850800	2.86922200
C	-1.26192400	1.44868200	4.41292400
H	-0.52755800	-0.54892300	4.75011900
C	0.12019900	2.84927900	3.01777400
H	1.91410200	1.94887400	2.25163900
C	-1.02966700	2.67474500	3.78981400
H	-2.15678300	1.30067300	5.01095900
H	0.30189300	3.79566100	2.51862800
H	-1.73937200	3.48758500	3.90102400
C	0.93707500	4.93419200	-2.16475900
C	1.87472200	-2.14930500	-0.64654000
C	-1.48596000	-2.53471800	-2.18440700

TS4-S3

C	-1.41954400	-1.48787700	0.89922900
H	-0.66340800	-0.36066800	0.88475900
O	-0.00090400	0.65430000	1.03884700
C	0.36884400	3.60836600	0.04871600
C	-0.76877100	4.10540500	0.73787500
C	-0.60630700	5.22171700	1.52905200
C	0.82254200	6.94775100	2.56880900
C	2.06248300	7.50129900	2.78454500
C	3.19840000	6.94946200	2.14735800
C	3.07324100	5.87804900	1.29230100
C	3.57085500	4.34155600	-1.52593200
C	4.69561200	3.70752100	-2.14760900
C	4.97156200	2.34428500	-1.87152000

C	4.15666400	1.59401700	-1.05209800
C	3.04058500	2.23719900	-0.45566200
C	2.76165200	3.58533100	-0.61522500
C	3.28174400	5.68874800	-1.87238900
C	4.08107300	6.38086400	-2.75367000
C	5.21230500	5.76457200	-3.33817600
C	5.50660900	4.45430300	-3.04235900
C	4.28174900	-3.16859300	-2.51626800
C	3.69497000	-2.80883300	-3.70031800
C	3.31182500	-1.45883000	-3.93259100
C	3.55410300	-0.50009100	-2.98737700
C	4.19171500	-0.82266100	-1.74766300
C	4.44510600	0.15776100	-0.76836800
C	4.98615200	-0.21035900	0.48118100
C	5.21889400	0.75213900	1.51291400
C	5.71844400	0.38136400	2.73237400
C	6.03650100	-0.97974100	2.99397700
C	5.83994600	-1.93222900	2.03043900
C	5.30340100	-1.59286300	0.74887400
C	5.08069600	-2.57113900	-0.24203700
C	4.54066600	-2.20145000	-1.49086100
C	5.36907400	-4.00615100	0.04680100
C	4.51834800	-4.74084900	0.88572800
C	4.77379600	-6.08696000	1.14495700
C	5.88039200	-6.71710500	0.57316800
C	6.73398400	-5.99209100	-0.26008000
C	6.47926500	-4.64550000	-0.52102700
C	-4.31347500	1.19605600	2.68092400
C	-3.52269600	0.98641900	3.77712400
C	-2.23856500	1.59063600	3.86193100
C	-1.77609300	2.37868800	2.84537500
C	-2.56425800	2.62209900	1.67613600
C	-2.09520100	3.42988100	0.62120100
C	-2.88599500	3.62911900	-0.52760500
C	-2.42425200	4.41646100	-1.62811200
C	-3.19887500	4.61247300	-2.73923000
C	-4.49745900	4.03939100	-2.81582400
C	-4.98280200	3.28560400	-1.78085200
C	-4.20358000	3.04318900	-0.60448300
C	-4.68357400	2.24792800	0.45892700
C	-3.87130200	2.01094000	1.58851400
C	-6.05894200	1.67328900	0.39879300
C	-6.25443300	0.29598400	0.23124400
C	-7.53810200	-0.24593400	0.18982600
C	-8.65204600	0.58586000	0.31195000
C	-8.47124600	1.96036500	0.47603500

C	-7.18516100	2.49889300	0.52138200
O	0.18989900	2.50564700	-0.76699900
O	2.22091900	1.46660500	0.34903300
P	0.74432500	1.04625300	-0.25264100
C	1.62589400	4.18519900	0.13770700
O	0.77455300	0.11845900	-1.40842100
H	-1.46421700	5.62625900	2.05899800
H	-0.05686200	7.34639400	3.06819100
H	2.17509600	8.35052300	3.45226000
H	4.18079300	7.37157300	2.33918500
H	3.95306700	5.45998000	0.81890700
H	5.83702700	1.87714700	-2.33328400
H	2.41244800	6.16761000	-1.43777000
H	3.83740100	7.40861000	-3.00737800
H	5.83752300	6.32412100	-4.02789000
H	6.36204300	3.96218000	-3.49808100
H	4.55239000	-4.20182800	-2.33180100
H	3.50691600	-3.55992800	-4.46303900
H	2.81226000	-1.19195800	-4.85909300
H	3.23494900	0.52095900	-3.15335300
H	4.98537900	1.79201100	1.31608900
H	5.87808100	1.12978100	3.50337100
H	6.44141300	-1.26147900	3.96190300
H	6.08581900	-2.96853500	2.22941600
H	3.66034500	-4.25292500	1.33243000
H	4.10285300	-6.64130900	1.79558900
H	6.07846900	-7.76591700	0.77673700
H	7.60023500	-6.47357100	-0.70571000
H	7.14177000	-4.07868900	-1.16926300
H	-5.29701100	0.74476200	2.62796600
H	-3.86892200	0.34764200	4.58412100
H	-1.61720300	1.40763000	4.73314400
H	-0.78584900	2.81244700	2.89968400
H	-1.43568300	4.85725700	-1.56993500
H	-2.82254700	5.20697200	-3.56664700
H	-5.10630800	4.20212200	-3.70075400
H	-5.97816100	2.85989800	-1.83966500
H	-5.38840800	-0.34339400	0.11776500
H	-7.66614400	-1.31806600	0.06630700
H	-9.65381100	0.16671100	0.28118100
H	-9.33294700	2.61485500	0.57435300
H	-7.04405200	3.56743700	0.65587900
C	1.80300600	5.30013900	1.02313700
C	0.66020200	5.83390900	1.70322000
C	-2.57935300	-3.01350000	2.50522400
C	-1.16548500	-1.83560800	4.62628900

H	-0.30878600	-0.54584700	3.12688600
C	-2.73191600	-3.48241100	3.81106300
C	-2.03077900	-2.90036400	4.86969800
H	-0.61349000	-1.38010100	5.44360300
H	-3.41688200	-4.30531900	3.99508500
H	-2.16489900	-3.27865900	5.87945300
C	-3.33607000	-3.61129400	1.37680400
C	-2.98718100	-4.85130600	0.82679800
C	-4.37652800	-2.88712100	0.78167500
C	-3.62063700	-5.33148700	-0.31656100
H	-2.18243400	-5.42295100	1.27898800
C	-5.02041700	-3.35611400	-0.35681000
H	-4.65737100	-1.93136700	1.21051400
C	-4.62203400	-4.56988100	-0.92504600
H	-3.33053100	-6.27374900	-0.76957300
H	-5.79416500	-2.76089800	-0.83610200
C	-1.84653000	-2.32502300	-1.56471700
C	-2.98545400	-1.84764600	-2.32842200
C	-1.10019200	-3.44104300	-2.04604700
C	-3.64281200	-0.64310600	-2.00764600
C	-3.46717300	-2.61844800	-3.42222200
C	-1.64054100	-4.20731200	-3.12596400
C	-4.75558200	-0.23740700	-2.72173600
H	-3.26049500	-0.03094400	-1.19686400
C	-4.62002900	-2.19609600	-4.12295500
C	-2.77774400	-3.81919500	-3.76980200
H	-1.10836700	-5.10068800	-3.44289500
C	-5.26294500	-1.02340600	-3.77502400
H	-5.23607900	0.69971900	-2.46819900
H	-4.97786700	-2.80009100	-4.95400200
H	-3.16472500	-4.42105800	-4.58747400
H	-6.14155900	-0.69532200	-4.32233500
O	-5.17176700	-5.04537000	-2.08391000
H	-5.67267600	-4.32442700	-2.49855200
N	0.07917100	-3.83876600	-1.51227700
H	0.45603600	-4.68470400	-1.91820200
C	1.07261200	-2.95140500	-0.90337700
H	2.02188700	-3.16347500	-1.40567700
H	0.82466800	-1.91746400	-1.15579700
C	1.27029100	-3.09364300	0.58792200
C	0.79317900	-4.18364400	1.31675600
C	2.01172500	-2.10558400	1.24543900
C	1.05861600	-4.29082900	2.68326800
H	0.19890800	-4.94000700	0.81268200
C	2.28621100	-2.21401400	2.60651400
H	2.38355300	-1.25422700	0.68747900

C	1.81159700	-3.31046900	3.33018700
H	0.66760800	-5.13514300	3.24435700
H	2.87256100	-1.44132600	3.09404900
H	2.01474000	-3.39326000	4.39397100
C	-1.68718600	-1.93938700	2.26036600
C	-1.64706400	-1.88029200	-0.27495900
C	-0.99437600	-1.35875300	3.32909900

TS4-S4

O	-0.23964200	-1.08213700	1.40723800
H	0.69912000	-0.25301100	1.49948900
C	1.44765900	0.84650300	1.62638600
C	2.67115500	1.35961800	-1.59734900
C	3.20967100	0.99417100	-0.31255700
C	0.85149800	1.74017800	2.62799600
C	-2.34161600	-6.65765400	2.47208400
C	-3.54722200	-6.84582800	3.10660900
C	-4.58692900	-5.90207900	2.93692500
C	-4.41324400	-4.80687200	2.12121000
C	-5.25336700	-2.92271800	-0.43838700
C	-6.23167400	-1.93787700	-0.79569700
C	-5.93850200	-0.56332900	-0.60991600
C	-4.73715800	-0.14964800	-0.08084700
C	-3.82337000	-1.14030900	0.36614600
C	-4.03961000	-2.50024900	0.20474100
C	-5.51789400	-4.27746200	-0.77840000
C	-6.70215300	-4.64227200	-1.37801600
C	-7.68765800	-3.67408100	-1.67979000
C	-7.44852100	-2.34931400	-1.40062200
C	-2.70878400	3.63793300	-2.54946100
C	-2.65756200	2.85351500	-3.67022300
C	-3.13510400	1.51566600	-3.63316700
C	-3.66142100	1.00347600	-2.47856400
C	-3.76933700	1.79827300	-1.29389900
C	-4.31467100	1.28021900	-0.10156400
C	-4.42663400	2.08907600	1.04151600
C	-5.00096000	1.59379900	2.25347100
C	-5.16362100	2.40291400	3.34504000
C	-4.76739400	3.76805900	3.28737300
C	-4.18200900	4.27352300	2.15642300
C	-3.96416700	3.45583200	1.00107200
C	-3.34687700	3.95684900	-0.16637400
C	-3.27418400	3.15508700	-1.32717900
C	-2.73887400	5.31736200	-0.14774500
C	-1.62911400	5.56939100	0.67414700
C	-1.04772800	6.83607100	0.71656200

C	-1.55804300	7.86967300	-0.07190400
C	-2.65681100	7.62785800	-0.89796100
C	-3.24543100	6.36363400	-0.93062900
C	4.12232300	-2.70168800	0.68771100
C	3.97826100	-2.63932700	2.04615600
C	2.74106500	-2.98462300	2.65455500
C	1.68033500	-3.38026000	1.88654800
C	1.79185200	-3.48640200	0.46338700
C	0.70527600	-3.88241900	-0.34215200
C	0.84864200	-3.96364300	-1.74286500
C	-0.22999000	-4.38185600	-2.58381100
C	-0.08214200	-4.47689000	-3.94078500
C	1.16718800	-4.17335800	-4.54646900
C	2.22753400	-3.77213300	-3.77916600
C	2.11151200	-3.63481200	-2.35877900
C	3.19637300	-3.21667000	-1.55672600
C	3.05265200	-3.14209900	-0.15476300
C	4.49307100	-2.85505200	-2.19450300
C	4.58441900	-1.73900500	-3.03630800
C	5.79238200	-1.40237500	-3.64434700
C	6.93349700	-2.17012400	-3.41288000
C	6.85636500	-3.28230600	-2.57282400
C	5.64538100	-3.62361700	-1.97289300
O	-1.41767000	-2.11355900	-0.64809600
O	-2.67945400	-0.69220300	1.00350700
P	-1.21777200	-0.76375300	0.26333700
C	-2.96985100	-3.46592100	0.57533600
O	-0.94251900	0.40523800	-0.61946900
H	-0.06594900	-6.02643200	1.17778500
H	-1.52710300	-7.36414900	2.61008900
H	-3.69930200	-7.70895500	3.74816100
H	-5.52905200	-6.03945500	3.45985200
H	-5.21239500	-4.08443100	2.00461200
H	-6.65343800	0.17973000	-0.95238100
H	-4.77074800	-5.03105800	-0.56457300
H	-6.87767000	-5.68475100	-1.62796600
H	-8.62041000	-3.97684000	-2.14667500
H	-8.18230900	-1.58822300	-1.65347000
H	-2.30638400	4.64145500	-2.58095700
H	-2.22122600	3.24971400	-4.58180100
H	-3.06889500	0.89340500	-4.52135200
H	-4.00155900	-0.02428500	-2.44847800
H	-5.31845100	0.55714800	2.28538900
H	-5.60593600	2.00844500	4.25521200
H	-4.92873600	4.41134600	4.14793900
H	-3.87931900	5.31355200	2.11981400

H	-1.22489600	4.76519500	1.28082400
H	-0.19201200	7.01221300	1.36282600
H	-1.10415900	8.85634400	-0.04155500
H	-3.06296300	8.42676400	-1.51224800
H	-4.10752400	6.17987600	-1.56511600
H	5.05606700	-2.39884400	0.23189800
H	4.80632200	-2.29815500	2.65832100
H	2.63197800	-2.91551400	3.73347700
H	0.72513700	-3.59565400	2.34719300
H	-1.17910300	-4.63318500	-2.12494100
H	-0.91764700	-4.79617800	-4.55729600
H	1.28238700	-4.27168900	-5.62236900
H	3.18248000	-3.55977100	-4.24451200
H	3.70209600	-1.12974000	-3.20253100
H	5.84180500	-0.53234400	-4.29284000
H	7.87625600	-1.90521300	-3.88347800
H	7.73904300	-3.88836700	-2.38809700
H	5.58284600	-4.49227600	-1.32407100
C	-3.19032500	-4.59633900	1.43044400
C	-2.12360500	-5.53065200	1.63661300
C	1.58966600	2.17496100	3.74889500
C	-1.06140200	3.03165600	3.37136500
H	-1.03487000	1.82472000	1.58955000
C	0.98756500	3.06966500	4.64325700
C	-0.32502800	3.49891600	4.46063800
H	-2.09087900	3.33922000	3.23186600
H	1.55894700	3.40444500	5.50467800
H	-0.77855800	4.17950900	5.17576800
C	2.96475400	1.68092900	4.01675100
C	4.05215500	2.55921500	4.07356900
C	3.19846800	0.31852700	4.26208900
C	5.33873400	2.09701000	4.34855100
H	3.89262600	3.61569100	3.87870700
C	4.47257800	-0.15147300	4.55907700
H	2.36770300	-0.37829900	4.21672100
C	5.54991000	0.73894900	4.59092600
H	6.17743400	2.79028500	4.36114900
H	4.65210400	-1.20145900	4.76393200
C	-0.62023900	-4.17753600	0.27206200
C	4.64341700	0.84174500	-0.13904900
C	-1.69273800	-3.26719000	0.07826900
C	5.20673900	0.43432900	1.08462800
C	5.50784900	1.14381100	-1.22418300
C	3.58638500	1.73490400	-2.63419100
C	6.57636400	0.28608400	1.21373100
H	4.55275500	0.22514400	1.92064200

C	6.90233300	0.98200100	-1.07271600
C	4.93350400	1.62396100	-2.44163000
H	3.19737500	2.11194200	-3.57152100
C	7.43337100	0.54955900	0.12691200
H	6.98723000	-0.03670600	2.16617900
H	7.54905000	1.19920100	-1.91881800
H	5.60788600	1.90367400	-3.24746900
H	8.50599900	0.41871200	0.23479700
O	6.79364500	0.21599600	4.83800300
H	7.43854000	0.94037000	4.84601400
N	1.35225500	1.33709300	-1.81004600
H	0.71504100	0.98030300	-1.08983400
C	0.68415300	1.76293100	-3.02747800
H	1.11878800	1.25678700	-3.89880000
H	-0.34600400	1.40806300	-2.92824700
C	0.68580700	3.26433300	-3.23568600
C	0.54632800	4.13476500	-2.14991300
C	0.74405600	3.79280800	-4.52783200
C	0.45140000	5.50973800	-2.35537200
H	0.48559500	3.72983200	-1.14415300
C	0.64381200	5.16929000	-4.73811000
H	0.85543300	3.12099900	-5.37666200
C	0.49434700	6.03040700	-3.65056300
H	0.31886400	6.17142500	-1.50632200
H	0.68612300	5.56652400	-5.74870700
H	0.41174200	7.10200100	-3.80902300
C	-0.86391000	-5.30538300	1.02349700
C	2.34887000	0.91741100	0.74923900
C	-0.47569500	2.15974600	2.45670300

TS4-S5

C	-0.39459300	-2.23967900	2.04081300
H	-0.05076600	-0.95321300	1.81041900
O	0.33245100	0.18040500	1.40843500
C	1.04409500	2.75130000	0.18205200
C	0.04499200	3.49750800	0.86250800
C	0.44847000	4.47986600	1.73763600
C	2.23140100	5.63264000	3.00490000
C	3.56014100	5.78415100	3.32849700
C	4.53915800	5.00578900	2.66465700
C	4.17550200	4.12041800	1.67167300
C	4.26318400	3.04736400	-1.44948600
C	5.11820500	2.31617600	-2.33917000
C	4.98575600	0.90564400	-2.44034500
C	4.06799500	0.22049700	-1.68161100
C	3.30091100	0.94728000	-0.72952600

C	3.35875700	2.32221300	-0.59729900
C	4.32713600	4.46645600	-1.48161400
C	5.22156000	5.12108900	-2.29904900
C	6.09722100	4.39535800	-3.13800400
C	6.03581000	3.02148300	-3.16036800
C	1.07991300	-3.10773500	-3.77455800
C	0.62867800	-2.14274200	-4.63559000
C	1.16335200	-0.82640400	-4.58345400
C	2.16617900	-0.52671900	-3.70346800
C	2.68581100	-1.51008800	-2.80309800
C	3.69656900	-1.20820000	-1.87169400
C	4.17684300	-2.19107100	-0.98968600
C	5.22873100	-1.90694500	-0.06560800
C	5.67075200	-2.85440800	0.81912200
C	5.06385500	-4.13880300	0.84742600
C	4.03890700	-4.44355500	-0.00775800
C	3.56652300	-3.49804400	-0.96974000
C	2.49971800	-3.78131200	-1.85180500
C	2.09609400	-2.82589700	-2.80671600
C	1.71073400	-5.03500400	-1.70146800
C	2.23813200	-6.29990800	-1.98965900
C	1.46613900	-7.44890900	-1.81060100
C	0.15312900	-7.34764500	-1.34806000
C	-0.38514800	-6.08997700	-1.06689500
C	0.38865600	-4.94427500	-1.23880400
C	-4.26286300	1.32958400	2.09465100
C	-3.72228700	0.95928600	3.29545200
C	-2.37600400	1.28580800	3.61127200
C	-1.61377900	2.00032300	2.72942000
C	-2.15697200	2.45763500	1.48713800
C	-1.39048100	3.21408200	0.58147200
C	-1.96446400	3.70264100	-0.60892400
C	-1.23303900	4.53960700	-1.50953600
C	-1.81952600	5.06295900	-2.63148300
C	-3.18039200	4.77639700	-2.92649900
C	-3.90433100	3.94945300	-2.10980600
C	-3.33052100	3.37275500	-0.93290400
C	-4.07242300	2.52803800	-0.07791400
C	-3.51988600	2.10741400	1.15227300
C	-5.40645400	2.03159300	-0.51663600
C	-5.47949000	1.20598500	-1.64878500
C	-6.70148400	0.69395200	-2.07982500
C	-7.87321600	0.99225900	-1.38104000
C	-7.81336500	1.81933800	-0.25731400
C	-6.59044200	2.34001000	0.16736400
O	0.61110600	1.71544500	-0.63811200

O	2.48612600	0.20940900	0.10270300
P	0.85298600	0.19855900	-0.03280400
C	2.40021900	3.01323900	0.30402100
O	0.35034900	-0.82387900	-0.99344000
H	-0.29958800	5.06787600	2.26136900
H	1.46739100	6.19861000	3.53055400
H	3.85917400	6.48204700	4.10458600
H	5.58518000	5.10690400	2.93922900
H	4.93006900	3.52906200	1.16561700
H	5.58382300	0.37002300	-3.17249300
H	3.65611300	5.04015600	-0.85500200
H	5.24828800	6.20705400	-2.30348900
H	6.80182200	4.92347900	-3.77378000
H	6.68207700	2.44922700	-3.82107700
H	0.66949100	-4.11029200	-3.81738800
H	-0.14711100	-2.37769000	-5.35919300
H	0.77040500	-0.06046100	-5.24499500
H	2.57538700	0.47606600	-3.67035800
H	5.68025500	-0.92049300	-0.08836100
H	6.47988000	-2.62436000	1.50680500
H	5.40395000	-4.87679800	1.56767500
H	3.55923600	-5.41243400	0.04875900
H	3.25738200	-6.37834700	-2.35709500
H	1.88948600	-8.42337300	-2.03835600
H	-0.44778300	-8.24250200	-1.21185500
H	-1.40713300	-5.99986600	-0.70764500
H	-0.01696800	-3.96570300	-1.00268300
H	-5.27418100	1.03361100	1.84997500
H	-4.31736900	0.38520500	3.99613400
H	-1.94416300	0.94867300	4.54915400
H	-0.57765600	2.21411700	2.95582600
H	-0.20069800	4.77522200	-1.27649400
H	-1.24798500	5.70817000	-3.29279700
H	-3.64485200	5.21399000	-3.80581700
H	-4.93910600	3.72806200	-2.34210000
H	-4.56890000	0.96284100	-2.18218100
H	-6.73148400	0.06082300	-2.96264200
H	-8.82745500	0.59328100	-1.71448100
H	-8.72154200	2.06756800	0.28528500
H	-6.54794400	2.99096100	1.03571000
C	2.81268500	3.95462900	1.30297100
C	1.82104800	4.70536600	2.01200500
C	1.82330700	-2.74078200	3.09770700
C	1.49150800	-5.46243700	2.43322300
H	-0.39081500	-4.85754000	1.59348600
C	2.80805600	-3.68869500	3.40671800

C	2.64884300	-5.03358800	3.08562000
H	1.36099500	-6.50577600	2.16135700
H	3.70499300	-3.35244400	3.91773700
H	3.42875900	-5.74593300	3.33995700
C	2.01999600	-1.32091600	3.47636900
C	3.12015700	-0.60388000	2.99163900
C	1.09905300	-0.65548300	4.29976700
C	3.28134200	0.74634200	3.28860300
H	3.82581500	-1.09604400	2.33009100
C	1.26004000	0.68648700	4.61777500
H	0.24370300	-1.19951800	4.68976900
C	2.34635500	1.39836900	4.09757300
H	4.11669700	1.30296900	2.87110600
H	0.55286800	1.20523800	5.25724900
C	-2.88633500	-2.14077500	1.20964600
C	-3.98549800	-2.14149900	2.15982900
C	-3.14907400	-2.03671500	-0.19960200
C	-3.78218600	-2.32537800	3.54200600
C	-5.31339300	-1.97223400	1.68462200
C	-4.50740100	-1.90084800	-0.62888400
C	-4.85100500	-2.31202200	4.42024200
H	-2.77087600	-2.46577900	3.90938000
C	-6.38729000	-1.93766800	2.60109800
C	-5.52486400	-1.83789800	0.27802400
H	-4.71444500	-1.75730800	-1.68065400
C	-6.16420500	-2.10364300	3.95459400
H	-4.67273600	-2.45395900	5.48253900
H	-7.39375700	-1.78533500	2.21966900
H	-6.53608500	-1.65671400	-0.07649300
H	-6.99381600	-2.08261300	4.65462700
O	2.44238400	2.71981600	4.42398900
H	3.16336000	3.12828900	3.91666100
N	-2.14302300	-2.05460700	-1.08779700
H	-1.19646300	-1.81577500	-0.77539700
C	-2.30405000	-2.13475400	-2.53441400
H	-1.34436900	-2.49667100	-2.91259700
H	-3.05286500	-2.90000800	-2.76659600
C	-2.65190600	-0.83506200	-3.23340200
C	-1.90940200	0.32536100	-2.98066500
C	-3.67964800	-0.79736500	-4.18030900
C	-2.19813500	1.50313500	-3.66466400
H	-1.10466200	0.29653700	-2.25597400
C	-3.96822900	0.38424800	-4.86849100
H	-4.25727700	-1.69750700	-4.38293400
C	-3.22783100	1.53697700	-4.60863200
H	-1.62445600	2.39794400	-3.44938400

H	-4.77390300	0.40323000	-5.59751100
H	-3.45789100	2.46366700	-5.12568800
C	0.65443400	-3.17752800	2.43309600
C	-1.59467800	-2.28036400	1.65018400
C	0.50482900	-4.53867600	2.11434000

TS4-S6

C	-0.78749500	-0.74463100	1.44190300
H	-0.90906900	-0.10839800	0.25028100
O	-1.06594200	0.31700300	-0.89381800
C	-1.34044000	3.44946600	-0.32248400
C	-2.68846700	3.34224900	0.13031400
C	-3.05065000	4.08508200	1.23646300
C	-2.48707100	5.48488900	3.18927800
C	-1.54837800	6.09652800	3.98612900
C	-0.18367400	6.06253600	3.61434500
C	0.21297300	5.44626300	2.44803400
C	1.83011800	5.27719200	-0.31092100
C	3.22950700	5.13327000	-0.57617100
C	3.79954000	3.83752700	-0.60903300
C	3.03170400	2.70008200	-0.46136200
C	1.63875500	2.86102600	-0.25012100
C	1.04271700	4.10319600	-0.08156900
C	1.27162700	6.58335000	-0.32121700
C	2.06018800	7.68743200	-0.55242100
C	3.44912900	7.54595500	-0.78375200
C	4.01727300	6.29387600	-0.79788300
C	5.93113500	-0.59863800	-2.75032400
C	5.74444500	0.07333900	-3.92769700
C	4.82700600	1.15632000	-4.00298400
C	4.15958300	1.57146000	-2.88359000
C	4.34331900	0.91320600	-1.62660900
C	3.68367100	1.36153800	-0.46482500
C	3.73237500	0.59208100	0.71811200
C	2.99479500	0.95243300	1.89037900
C	2.98983600	0.16325800	3.00868500
C	3.75883800	-1.03176000	3.03674500
C	4.52326800	-1.38930400	1.95669900
C	4.55121900	-0.59745000	0.76190700
C	5.33223800	-0.96478800	-0.36023300
C	5.20981600	-0.24133800	-1.56703200
C	6.28159500	-2.10834600	-0.25191200
C	6.14510100	-3.25653400	-1.04647800
C	7.03753600	-4.32078900	-0.92044800
C	8.08576300	-4.25581700	-0.00195300
C	8.23709900	-3.11564300	0.78929400

C	7.34347500	-2.05326900	0.66480200
C	-5.62330100	-0.62171900	0.69878800
C	-5.28392300	-0.68074600	2.02421800
C	-4.29558500	0.19961200	2.54052900
C	-3.74258400	1.16228900	1.73882800
C	-4.12165200	1.31252000	0.36606400
C	-3.63067100	2.35345000	-0.45952000
C	-4.01255500	2.41915600	-1.81443600
C	-3.60513600	3.49928300	-2.65703100
C	-3.92865600	3.52688400	-3.98571000
C	-4.67926600	2.46609500	-4.56311500
C	-5.11609000	1.42668200	-3.78768200
C	-4.83193800	1.37658500	-2.38594000
C	-5.32610600	0.34354800	-1.56406400
C	-5.02889000	0.33450200	-0.18535300
C	-6.12856500	-0.77202200	-2.13947000
C	-5.57547600	-2.05997900	-2.19697200
C	-6.31275900	-3.12823100	-2.70473300
C	-7.61398100	-2.92331700	-3.16761000
C	-8.17242500	-1.64482000	-3.11882300
C	-7.43494600	-0.57660600	-2.60712000
O	-0.98130500	2.82317800	-1.49675800
O	0.86775900	1.71584900	-0.16813600
P	-0.14078300	1.40495200	-1.45901700
C	-0.37288600	4.16635100	0.36581300
O	0.57968500	1.22890000	-2.73820000
H	-4.07708100	4.03824900	1.58823300
H	-3.53591600	5.47646300	3.47438300
H	-1.84747900	6.59096700	4.90570900
H	0.56095800	6.52297600	4.25744500
H	1.26248700	5.42106600	2.18417000
H	4.86920200	3.73634000	-0.76837200
H	0.20688600	6.69996500	-0.15529000
H	1.61132000	8.67653500	-0.56411500
H	4.06005400	8.42642100	-0.96045100
H	5.07991100	6.16928500	-0.98974100
H	6.62770800	-1.42629300	-2.70689300
H	6.29259200	-0.22998800	-4.81523700
H	4.65318100	1.65186400	-4.95366500
H	3.43989400	2.37850500	-2.94208100
H	2.40555300	1.85958700	1.88184800
H	2.39088800	0.44666200	3.86802500
H	3.75212300	-1.65693200	3.92578900
H	5.11593800	-2.29599500	1.99175800
H	5.33553500	-3.30905200	-1.76539600
H	6.91184100	-5.20239700	-1.54345600

H	8.78137600	-5.08466300	0.09494600
H	9.05426600	-3.05126400	1.50251000
H	7.46241100	-1.16604600	1.27977600
H	-6.36193800	-1.30308700	0.29372800
H	-5.75586700	-1.41056500	2.67688200
H	-3.97806400	0.11775100	3.57637600
H	-2.99647500	1.82387300	2.15396500
H	-3.02508700	4.30509300	-2.22339800
H	-3.60139000	4.35550000	-4.60689600
H	-4.90683400	2.48456400	-5.62519000
H	-5.69072400	0.62084000	-4.22886000
H	-4.56524700	-2.21713600	-1.83357900
H	-5.86740800	-4.11871900	-2.73416600
H	-8.19022500	-3.75455000	-3.56469800
H	-9.18547700	-1.47877100	-3.47506400
H	-7.87071200	0.41736700	-2.56106700
C	-0.73498800	4.82933500	1.58628400
C	-2.10842300	4.82455600	1.99020100
C	-0.48345300	-0.80229900	3.90229900
C	-0.24686300	2.00170700	3.96130100
H	-0.51065900	1.90700500	1.82798100
C	-0.26935600	-0.12979900	5.10746600
C	-0.15181700	1.26252000	5.14155400
H	-0.15891800	3.08350700	3.97694800
H	-0.19687400	-0.70661600	6.02505900
H	0.01138600	1.76694100	6.08983900
C	-0.61452700	-2.28298000	3.84018600
C	-1.88201400	-2.88287400	3.83395700
C	0.51189400	-3.10254700	3.66648600
C	-2.02738600	-4.24904200	3.61924700
H	-2.76401500	-2.26057400	3.95107600
C	0.37752800	-4.46851900	3.44475600
H	1.49922100	-2.65134000	3.65289500
C	-0.89747000	-5.03934800	3.38743500
H	-3.01962500	-4.69137800	3.57331600
H	1.24347900	-5.09704700	3.26640500
C	-0.95639100	-3.20063100	0.52324800
C	-2.20812400	-3.90199800	0.33136900
C	0.27638500	-3.80015800	0.11624200
C	-3.44290200	-3.34126800	0.71276700
C	-2.19645700	-5.19366100	-0.26168300
C	0.23669800	-5.07195000	-0.53224100
C	-4.61896300	-4.04415300	0.53052300
H	-3.46245900	-2.34729000	1.14193600
C	-3.41181500	-5.89612600	-0.43330000
C	-0.94688800	-5.73483800	-0.68955600

H	1.15549200	-5.51697800	-0.89014700
C	-4.61021800	-5.33381000	-0.03926200
H	-5.56005800	-3.58703200	0.81806100
H	-3.38585400	-6.88253400	-0.88989600
H	-0.94717500	-6.71233300	-1.16569200
H	-5.54235500	-5.87334200	-0.17722200
O	-0.98319300	-6.36948500	3.08831500
H	-1.91658100	-6.59807800	2.95647200
N	1.45114000	-3.18718400	0.36543100
H	1.38706000	-2.27197800	0.79754000
C	2.69451300	-3.48313100	-0.33844600
H	3.47794500	-2.94549400	0.19635200
H	2.92891300	-4.54793500	-0.24202500
C	2.66215400	-3.06758800	-1.79537100
C	2.22063800	-1.78851600	-2.15111100
C	3.09848900	-3.94114900	-2.79517100
C	2.22350300	-1.38169200	-3.48279200
H	1.87251200	-1.09552100	-1.39120200
C	3.11836000	-3.53338300	-4.13064900
H	3.43256500	-4.94184200	-2.52620000
C	2.68298700	-2.25333100	-4.47275700
H	1.86104000	-0.38870300	-3.72194300
H	3.46510700	-4.21855400	-4.89969300
H	2.69446500	-1.93522900	-5.51149900
C	-0.57709900	-0.04873800	2.70423900
C	-0.91105000	-1.96174600	1.11531900
C	-0.45502100	1.34552100	2.75030100

TS4-S7

C	-0.83203100	2.24091100	0.40795000
H	-0.47352800	1.29864500	-0.49790400
O	0.14143800	0.48329400	-1.21596200
C	0.24649900	-2.77604400	-1.06676900
C	-0.98284900	-3.00404500	-1.73456100
C	-1.00865300	-3.98509800	-2.70555200
C	0.11719700	-5.66654500	-4.12154100
C	1.26188500	-6.30912400	-4.52966900
C	2.49836600	-6.00118900	-3.91468300
C	2.56497900	-5.08224100	-2.89213900
C	3.41789100	-4.11666200	0.10132200
C	4.62119100	-3.71593300	0.76724800
C	5.03163600	-2.35940300	0.71613900
C	4.27445200	-1.39861500	0.08303300
C	3.07944900	-1.81447400	-0.56064100
C	2.65711000	-3.13238200	-0.61313000
C	3.00073500	-5.47005000	0.22206500

C	3.74471500	-6.38009900	0.93874700
C	4.94818400	-5.98886900	1.57011100
C	5.37245000	-4.68360300	1.48490900
C	4.70871300	3.04168500	2.29810900
C	4.22146700	2.50315000	3.45812500
C	3.89160000	1.12148400	3.52522600
C	4.04056200	0.32269900	2.42518800
C	4.54677200	0.84186800	1.19116300
C	4.67563700	0.03575700	0.04390500
C	5.16442500	0.57927200	-1.16121900
C	5.31824100	-0.22019300	-2.33693500
C	5.81494800	0.30770400	-3.49748400
C	6.20625000	1.67283100	-3.55206000
C	6.07656100	2.47378500	-2.44952400
C	5.53921600	1.97111700	-1.22172700
C	5.39390100	2.78915700	-0.07954700
C	4.90268500	2.24138600	1.12572300
C	5.74856100	4.23461900	-0.15695000
C	5.00110600	5.10685600	-0.96297400
C	5.31940800	6.46259700	-1.03050700
C	6.39451700	6.96829800	-0.29760400
C	7.15030000	6.10872800	0.50097600
C	6.82976600	4.75296400	0.56953600
C	-4.54995200	-0.08723300	-3.50319400
C	-3.80054000	0.11974300	-4.63076800
C	-2.46567900	-0.36310500	-4.70443600
C	-1.94677600	-1.10006200	-3.67540400
C	-2.70708900	-1.37044500	-2.49247600
C	-2.20277800	-2.19303900	-1.46264700
C	-2.89226400	-2.29461500	-0.23813400
C	-2.37655100	-3.05692200	0.85609100
C	-3.02103400	-3.10003900	2.06188700
C	-4.23934600	-2.39117500	2.24158400
C	-4.78977000	-1.67889200	1.21043900
C	-4.15911500	-1.61949500	-0.07275400
C	-4.74507400	-0.93077700	-1.16117900
C	-4.01968500	-0.78037500	-2.36632600
C	-6.13891000	-0.41873000	-1.05619200
C	-6.44555200	0.93022900	-1.27872200
C	-7.76139700	1.38538900	-1.22196200
C	-8.79814100	0.50185000	-0.92346300
C	-8.50778100	-0.84413900	-0.69046700
C	-7.19284000	-1.29962600	-0.76264400
O	0.25736000	-1.83379900	-0.05541300
O	2.29651700	-0.82739600	-1.15563600
P	0.99272200	-0.38296800	-0.27138300

C	1.41366600	-3.46563500	-1.35747200
O	1.33379400	0.13359700	1.08720800
H	-1.93954100	-4.18425800	-3.22846100
H	-0.83623700	-5.87419800	-4.60034000
H	1.22371800	-7.04031100	-5.33185400
H	3.40585700	-6.48990200	-4.25762000
H	3.52021200	-4.84876700	-2.43778300
H	5.95416500	-2.06841200	1.21067200
H	2.07892000	-5.78067700	-0.25506400
H	3.40213100	-7.40743800	1.02241700
H	5.52821200	-6.71781500	2.12834800
H	6.28760700	-4.36579400	1.97773900
H	4.95521700	4.09582800	2.25387300
H	4.08073800	3.13394500	4.33126500
H	3.50931700	0.70423200	4.45292500
H	3.75486500	-0.72068100	2.46769400
H	5.03746900	-1.26619500	-2.29533000
H	5.92011900	-0.31922400	-4.37829400
H	6.61727000	2.07875500	-4.47207200
H	6.39017900	3.50973700	-2.49338900
H	4.15875600	4.71524600	-1.52373400
H	4.72535200	7.12333400	-1.65601800
H	6.64297000	8.02459500	-0.34989300
H	7.99261500	6.49271500	1.06984600
H	7.41860800	4.08311400	1.18953100
H	-5.56665700	0.28216100	-3.46152000
H	-4.22747700	0.65826600	-5.47233800
H	-1.86189300	-0.15430400	-5.58278700
H	-0.93138400	-1.47203000	-3.73288900
H	-1.44280800	-3.58935600	0.73210800
H	-2.58860700	-3.66084600	2.88428300
H	-4.72988900	-2.40424200	3.20993000
H	-5.70666900	-1.12368100	1.36432700
H	-5.64561200	1.62866200	-1.48613500
H	-7.96334500	2.43484200	-1.41364400
H	-9.82457300	0.85556100	-0.87692200
H	-9.30776100	-1.54347200	-0.46282100
H	-6.97117600	-2.34953600	-0.59649400
C	1.39618600	-4.42299700	-2.42412800
C	0.15299200	-4.70344100	-3.07815400
C	-0.62828600	4.55347900	-0.50117700
C	2.00226300	4.69910300	0.48222700
H	1.70037800	2.66245800	1.13680100
C	0.13174000	5.72104000	-0.65165800
C	1.43515700	5.79890600	-0.16357300
H	3.01468200	4.75125800	0.86409800

H	-0.30956300	6.56661300	-1.17234800
H	2.01316600	6.70950100	-0.29483000
C	-2.02189400	4.47798000	-1.00691200
C	-2.97208300	5.42949500	-0.61753000
C	-2.43476000	3.44194300	-1.86291800
C	-4.29617400	5.34542300	-1.04629100
H	-2.67731400	6.22968200	0.05502900
C	-3.74912700	3.35260900	-2.30747400
H	-1.71765300	2.69482700	-2.18896700
C	-4.68628500	4.30243100	-1.88789900
H	-5.02586500	6.08175700	-0.71423100
H	-4.05399700	2.55490900	-2.97613300
C	-2.39352900	1.18087500	2.23134600
C	-3.81611200	1.46997000	2.26755400
C	-1.78586100	0.37293800	3.25562700
C	-4.43848900	2.27374000	1.29299100
C	-4.59830800	0.96731800	3.34174200
C	-2.61804000	-0.10361000	4.31806600
C	-5.79079300	2.54896100	1.37347200
H	-3.84875200	2.67124500	0.47522200
C	-5.98248800	1.24516300	3.39022700
C	-3.95121400	0.18857900	4.34776700
H	-2.18725700	-0.72949400	5.08740900
C	-6.57727900	2.02294100	2.41604600
H	-6.24976200	3.16658600	0.60930600
H	-6.56987000	0.83847800	4.20996100
H	-4.55842000	-0.19689500	5.16386700
H	-7.64275600	2.22766300	2.44951100
O	-5.98178900	4.15369200	-2.32008700
H	-6.50201500	4.90832500	-2.00351100
N	-0.47346400	0.10821800	3.21828800
H	0.05746800	0.33056300	2.36853200
C	0.28057400	-0.64978400	4.21075000
H	1.31894500	-0.33011900	4.08611900
H	-0.03289700	-0.35089000	5.21635200
C	0.18046000	-2.15466500	4.04543600
C	-0.46442900	-2.94820000	4.99718500
C	0.74117700	-2.76255100	2.91654400
C	-0.56592600	-4.32997900	4.81940200
H	-0.88400300	-2.48786900	5.88939500
C	0.64122400	-4.13961600	2.73539700
H	1.23662200	-2.14954200	2.17360500
C	-0.01677200	-4.92677600	3.68415700
H	-1.07032200	-4.93648200	5.56672600
H	1.08708900	-4.59824400	1.85923500
H	-0.09369800	-6.00116800	3.54194000

C	-0.04912300	3.45986300	0.17434500
C	-1.59662300	1.77419200	1.28805400
C	1.26951200	3.52616500	0.64120500

TS4-S8

C	1.27624000	-2.34176900	-0.64131300
H	1.26453600	-1.00885800	-0.87550900
O	1.15681200	0.22298500	-1.14161900
C	2.39659700	2.83567400	-0.09088500
C	3.68670300	2.48748300	-0.56901000
C	4.36552500	3.41899900	-1.32652200
C	4.47093400	5.58166000	-2.51771200
C	3.87276700	6.75181100	-2.92181400
C	2.54880200	7.04183800	-2.51563800
C	1.85955200	6.17544200	-1.69786000
C	0.13312300	5.43979400	0.98287100
C	-1.20221700	5.62447400	1.46546000
C	-2.18740800	4.65318900	1.16880400
C	-1.89751600	3.51702700	0.44032400
C	-0.57828500	3.36429500	-0.05754300
C	0.42361400	4.29915800	0.16544300
C	1.12485600	6.37829600	1.37871100
C	0.80181700	7.46083600	2.16451000
C	-0.52812500	7.66458200	2.60314000
C	-1.50530100	6.75943100	2.26395000
C	-5.13515500	0.29634100	2.37142200
C	-4.41700400	0.29124800	3.53766800
C	-3.18976600	1.00278800	3.63158800
C	-2.73241200	1.72972800	2.56692800
C	-3.45436600	1.77249500	1.33216200
C	-2.97630800	2.50869100	0.22806000
C	-3.60324700	2.37851400	-1.02644500
C	-3.08596400	3.02615000	-2.19313900
C	-3.69352700	2.89564800	-3.41284500
C	-4.89577300	2.14403900	-3.53356800
C	-5.44239900	1.52980300	-2.43473900
C	-4.80862900	1.59159300	-1.15044800
C	-5.35456300	0.94570000	-0.01998900
C	-4.67508400	1.01013100	1.21687100
C	-6.63293100	0.18463800	-0.12221700
C	-6.71425900	-0.99924100	-0.86921500
C	-7.90666200	-1.72150200	-0.92487600
C	-9.03517900	-1.27115300	-0.24024600
C	-8.96774500	-0.08822700	0.49912200
C	-7.77627900	0.63269900	0.55719300
C	5.14652300	-1.91524800	-2.33562000

C	4.64603400	-1.57460500	-3.56197300
C	3.98272800	-0.33203700	-3.74431900
C	3.86280700	0.54539600	-2.70162500
C	4.38988700	0.24062300	-1.40629300
C	4.26518800	1.13640600	-0.32347900
C	4.70891200	0.76624900	0.96084700
C	4.58705700	1.64814700	2.07985500
C	5.04073200	1.29291800	3.32056300
C	5.67007400	0.03342200	3.51752200
C	5.80862000	-0.84269200	2.47433600
C	5.32342200	-0.52469600	1.16507700
C	5.47598600	-1.41212500	0.07760500
C	5.02583300	-1.04107000	-1.20890200
C	6.10147600	-2.74877900	0.28940200
C	5.44469000	-3.73546700	1.04066700
C	6.01482400	-4.99584900	1.21855400
C	7.25612800	-5.28948300	0.65249700
C	7.92420500	-4.31318800	-0.08844800
C	7.35148700	-3.05431500	-0.26725400
O	1.73041700	1.92252400	0.70625500
O	-0.28914900	2.22541400	-0.79337700
P	0.52943200	1.03279400	0.01011300
C	1.78262400	4.04403600	-0.37982000
O	-0.26769300	0.36928100	1.06603200
H	5.35754600	3.17749000	-1.69784900
H	5.47908800	5.33434600	-2.84032400
H	4.40683100	7.44734200	-3.56268600
H	2.06938800	7.95478600	-2.85735200
H	0.84329400	6.40464800	-1.40045300
H	-3.19410300	4.79164000	1.55292800
H	2.14877100	6.22870400	1.05925100
H	1.57750400	8.16292500	2.45666400
H	-0.76910600	8.52686500	3.21801500
H	-2.52688700	6.88920900	2.61196100
H	-6.06109000	-0.26171900	2.30191300
H	-4.77921600	-0.27316400	4.39274500
H	-2.60602100	0.95434300	4.54563400
H	-1.78208600	2.24342200	2.62584000
H	-2.18240700	3.61617600	-2.09944700
H	-3.26955900	3.37898700	-4.28779100
H	-5.39362000	2.07355600	-4.49658000
H	-6.37372700	0.98291700	-2.52661000
H	-5.84449600	-1.35652400	-1.40972500
H	-7.94403100	-2.64391800	-1.49694200
H	-9.96247900	-1.83564700	-0.28288700
H	-9.84342100	0.27396400	1.03070200

H	-7.72103300	1.55098000	1.13490000
H	5.61987000	-2.87855800	-2.19677600
H	4.72318300	-2.27312200	-4.38975400
H	3.55601600	-0.08543800	-4.71223800
H	3.33089900	1.47787500	-2.83680800
H	4.12357100	2.61583000	1.92819600
H	4.93057800	1.97712600	4.15676400
H	6.05211500	-0.22870500	4.50047400
H	6.30520700	-1.79393500	2.62650400
H	4.48024600	-3.51124400	1.48615000
H	5.48656600	-5.74773200	1.79816600
H	7.70100600	-6.27097200	0.78976400
H	8.89405000	-4.53090400	-0.52729100
H	7.87000200	-2.29493800	-0.84524600
C	2.45855100	4.97186700	-1.23716700
C	3.78306400	4.65968300	-1.68478100
C	0.83411000	-3.45661000	-2.84105500
C	3.26743900	-4.85612900	-2.60356300
H	3.50370900	-3.79686400	-0.74766900
C	1.24859800	-4.36580100	-3.82449900
C	2.45486600	-5.05616200	-3.71871000
H	4.20438600	-5.39545200	-2.49554600
H	0.60432500	-4.52521300	-4.68452300
H	2.75235900	-5.75023100	-4.49971900
C	-0.51005300	-2.82958400	-2.94420600
C	-0.70169800	-1.44055100	-2.90444600
C	-1.64355100	-3.64757700	-3.08577800
C	-1.97885900	-0.88473800	-2.98258600
H	0.14726600	-0.77628900	-2.80873800
C	-2.92109500	-3.10721000	-3.17961900
H	-1.51773100	-4.72657000	-3.10442300
C	-3.09183700	-1.71991000	-3.13361900
H	-2.10340900	0.19506000	-2.93639700
H	-3.79614200	-3.74187400	-3.27049400
C	0.20774900	-2.44049800	1.74198600
C	0.91312200	-2.42362500	3.00629200
C	-1.22863800	-2.40330700	1.71022500
C	2.31193400	-2.28226000	3.08343600
C	0.16610200	-2.52749200	4.21016100
C	-1.93962400	-2.53388200	2.94277600
C	2.95080500	-2.28024600	4.31057600
H	2.87964600	-2.13421900	2.17043000
C	0.84054200	-2.53406300	5.45101900
C	-1.25949800	-2.60034600	4.12554000
H	-3.02107100	-2.57050900	2.93227400
C	2.21627900	-2.41883500	5.50422400

H	4.02662000	-2.14718100	4.34567400
H	0.25655500	-2.62037500	6.36412500
H	-1.82089100	-2.69257900	5.05248600
H	2.72930100	-2.41797800	6.46126000
O	-4.36854200	-1.24644500	-3.22297000
H	-4.37119600	-0.27123700	-3.18768100
N	-1.87464300	-2.25132700	0.54631100
H	-1.30862900	-2.04209300	-0.26787900
C	-3.31309700	-2.09568900	0.41192600
H	-3.67777900	-1.40083300	1.17061600
H	-3.47852300	-1.61146100	-0.55307100
C	-4.08486300	-3.39844700	0.47771800
C	-3.54811100	-4.58388300	-0.03469900
C	-5.37385700	-3.41320100	1.01839800
C	-4.29490800	-5.76090600	-0.01822200
H	-2.54363300	-4.57699500	-0.44505200
C	-6.12589000	-4.58818900	1.02883700
H	-5.79450400	-2.49760200	1.42160200
C	-5.58811300	-5.76596900	0.50946900
H	-3.86708600	-6.67610800	-0.41855800
H	-7.12901500	-4.58103000	1.44613400
H	-6.16964700	-6.68365400	0.52017800
C	1.67531000	-3.24761100	-1.72590000
C	0.85972000	-2.46490900	0.54417600
C	2.87756600	-3.95539100	-1.61693200

TS4-S9

C	-1.38374300	-0.98654000	1.57807000
H	-0.83745500	0.28619100	1.41975300
O	-0.16671600	1.26458000	1.32788500
C	0.83898800	3.81736400	0.18112500
C	-0.32821000	4.51851500	0.58367900
C	-0.16524300	5.61773600	1.39852700
C	1.25798400	7.09915200	2.77326700
C	2.49070500	7.43774800	3.28006900
C	3.63253600	6.68639000	2.91575400
C	3.52706400	5.63173000	2.03743200
C	4.32347500	4.05085400	-0.72941800
C	5.38803500	3.24660100	-1.25131400
C	5.33558600	1.84000700	-1.10193200
C	4.28605300	1.21383900	-0.46184200
C	3.28073000	2.03055600	0.11276100
C	3.27111000	3.41387900	0.00920700
C	4.33949100	5.44453900	-1.00972100
C	5.37535100	6.01438800	-1.71431500
C	6.45051800	5.22449800	-2.18516600

C	6.44857500	3.86813500	-1.96300900
C	3.87325600	-2.93790200	-3.10868700
C	3.45558500	-2.19411600	-4.17975700
C	3.22131500	-0.79872500	-4.03916200
C	3.46165200	-0.17978800	-2.84287000
C	3.91345900	-0.91581700	-1.70149400
C	4.19569500	-0.27451800	-0.47707600
C	4.45374100	-1.03906400	0.67532800
C	4.63613400	-0.42864900	1.95553900
C	4.82692300	-1.18300200	3.08142900
C	4.85602300	-2.60246300	2.99493000
C	4.72886000	-3.22478500	1.78180100
C	4.54279400	-2.47725400	0.57486200
C	4.42851000	-3.10955200	-0.68475000
C	4.07106400	-2.34652600	-1.81948200
C	4.70862500	-4.56628200	-0.80883000
C	3.73692600	-5.46627200	-1.26741500
C	4.02012700	-6.82673500	-1.37472100
C	5.28211100	-7.31266200	-1.03142600
C	6.26104500	-6.42608300	-0.57868000
C	5.97565200	-5.06631900	-0.46809300
C	-4.71419100	2.39023800	1.59835500
C	-4.34847900	2.27890300	2.91259800
C	-3.06881600	2.72686000	3.34112800
C	-2.20303300	3.29598400	2.44841100
C	-2.55287500	3.46086900	1.07007800
C	-1.66806700	4.04338400	0.13981900
C	-2.02966500	4.14817100	-1.21882800
C	-1.16831000	4.76349300	-2.18014100
C	-1.52424400	4.85994300	-3.49774500
C	-2.77737000	4.35500100	-3.93946300
C	-3.63595500	3.76468600	-3.05174500
C	-3.29941400	3.63049200	-1.66687200
C	-4.18347900	3.03587900	-0.74026600
C	-3.83710100	2.96934400	0.62581300
C	-5.46631200	2.44882600	-1.22011500
C	-5.45303200	1.29495900	-2.01741000
C	-6.64227800	0.74010200	-2.48414100
C	-7.86592900	1.32192800	-2.15033400
C	-7.89208300	2.46651700	-1.35312900
C	-6.69975000	3.02850600	-0.89564700
O	0.66537900	2.70165100	-0.63247800
O	2.26566400	1.39398100	0.81173300
P	0.78853500	1.25136200	0.11408400
C	2.12069900	4.18122200	0.55680700
O	0.69803100	0.15260200	-0.88464900

H	-1.03973400	6.18083500	1.71235700
H	0.36915500	7.65479000	3.06098200
H	2.58904200	8.27136500	3.96931900
H	4.60078900	6.94056900	3.33738400
H	4.40633700	5.05730600	1.77094000
H	6.11973000	1.23398700	-1.54669700
H	3.51899100	6.06187400	-0.66639500
H	5.36321100	7.08152500	-1.91689100
H	7.26414300	5.68923600	-2.73438400
H	7.25284400	3.24261100	-2.34155900
H	4.05437200	-3.99832300	-3.22837200
H	3.30180600	-2.67205700	-5.14337300
H	2.85693600	-0.22300400	-4.88502200
H	3.28200200	0.88266800	-2.73581400
H	4.59993300	0.65243300	2.02360000
H	4.94838000	-0.69859700	4.04637900
H	4.97976300	-3.19430900	3.89755500
H	4.74770200	-4.30635000	1.72171900
H	2.75409300	-5.09691200	-1.52877100
H	3.24584100	-7.50643500	-1.71971800
H	5.50233100	-8.37332600	-1.11539900
H	7.24895600	-6.79259500	-0.31337000
H	6.73757200	-4.37661100	-0.11685800
H	-5.67977500	2.02721300	1.26928600
H	-5.03187400	1.83364200	3.63086300
H	-2.77444500	2.60423900	4.37939300
H	-1.21843900	3.60736100	2.77210100
H	-0.21788700	5.16318600	-1.84588800
H	-0.85145500	5.33057100	-4.20901100
H	-3.05713200	4.44830200	-4.98507700
H	-4.59726100	3.39628000	-3.38928500
H	-4.50117900	0.83452900	-2.26291100
H	-6.61202400	-0.15688500	-3.09406500
H	-8.79376200	0.88455400	-2.50851600
H	-8.84052100	2.92848700	-1.09251300
H	-6.71748100	3.92576700	-0.28354800
C	2.27347300	5.27231200	1.47450800
C	1.11157500	6.00770200	1.87684800
C	-1.16074900	-3.04940800	2.97214800
C	1.26834700	-2.07926200	4.00866600
H	0.90195200	-0.32954600	2.81371400
C	-0.41257200	-3.80703200	3.87645500
C	0.79486700	-3.33328700	4.39280500
H	2.21559900	-1.70729400	4.38515700
H	-0.78158700	-4.78790000	4.16208000
H	1.36490400	-3.94602500	5.08583100

C	-2.40515100	-3.60556000	2.37489800
C	-3.67392500	-3.22068200	2.82328400
C	-2.32039000	-4.50624500	1.30514500
C	-4.82573300	-3.71391500	2.21590800
H	-3.75944900	-2.51843300	3.64754800
C	-3.46379400	-4.98731900	0.67753500
H	-1.34628800	-4.80273300	0.94103900
C	-4.72230900	-4.57927800	1.12368500
H	-5.80555400	-3.39489800	2.56083900
H	-3.39536800	-5.65248500	-0.17686400
C	-3.03736800	-1.47718300	-0.40774700
C	-4.47914700	-1.50750900	-0.29928300
C	-2.38575700	-2.03936500	-1.55602800
C	-5.16515000	-0.83564000	0.73194500
C	-5.22190900	-2.27543300	-1.23502700
C	-3.16531200	-2.84629700	-2.44297000
C	-6.53895400	-0.92567300	0.83527100
H	-4.59347200	-0.24534800	1.43811300
C	-6.62560300	-2.36959500	-1.09764400
C	-4.51715500	-2.95643000	-2.27497400
H	-2.67878300	-3.34370000	-3.27404400
C	-7.27983800	-1.70644000	-0.07682900
H	-7.05475500	-0.38807300	1.62598500
H	-7.18173600	-2.96870500	-1.81462600
H	-5.09212400	-3.56747100	-2.96643600
H	-8.36090900	-1.76339800	0.00945100
O	-5.82138600	-5.05024100	0.45478900
H	-6.56587200	-4.45622200	0.64197300
N	-1.08240200	-1.78871500	-1.77217500
H	-0.63880900	-1.06526800	-1.19866900
C	-0.10398500	-2.70271700	-2.36161200
H	-0.52406100	-3.20036300	-3.23961200
H	0.73315400	-2.08449100	-2.69656300
C	0.35482900	-3.71898900	-1.33139200
C	1.02904400	-3.27831400	-0.18363800
C	0.05524300	-5.07808200	-1.46665700
C	1.38549600	-4.18149300	0.81457800
H	1.26590200	-2.22433000	-0.07371500
C	0.42527000	-5.98833400	-0.47201100
H	-0.47251000	-5.42636300	-2.35162500
C	1.08653800	-5.53879600	0.67154500
H	1.89712500	-3.82318000	1.70064000
H	0.18869000	-7.04246300	-0.58798500
H	1.36940500	-6.24200300	1.44917500
C	-0.67992800	-1.77269700	2.58850100
C	-2.24536400	-1.19564600	0.68402800

C	0.53026700	-1.30048000	3.12061800

Int5-C1

C	-1.51384500	0.26179400	-2.12063700
H	-0.66705700	-0.44448500	-2.22455100
O	1.09184800	-0.83415500	-1.91548000
C	2.86222600	-2.46773500	-0.19085600
C	2.27945500	-3.69746400	-0.59903200
C	3.07174800	-4.60942700	-1.25804200
C	5.21765400	-5.23595000	-2.31415900
C	6.51754100	-4.93668500	-2.64968200
C	7.07715300	-3.69858900	-2.25694600
C	6.34110200	-2.79858000	-1.51975900
C	5.86501500	-0.92196900	1.11808000
C	6.34264600	0.32241000	1.64612400
C	5.63998600	1.51892500	1.35518500
C	4.50746600	1.51305200	0.57310700
C	4.09986600	0.28700100	-0.02363200
C	4.74677100	-0.91659800	0.21574100
C	6.50788400	-2.11549600	1.54597000
C	7.59077300	-2.07687000	2.39541300
C	8.09104200	-0.84324300	2.87235800
C	7.47042900	0.32865800	2.50855900
C	1.14877000	4.56228300	2.56940500
C	1.29629700	3.92170200	3.76925600
C	2.18838700	2.82283800	3.88654900
C	2.93481800	2.42470500	2.81018100
C	2.84351700	3.09264900	1.54675300
C	3.61434100	2.69465600	0.43443600
C	3.45401300	3.33564100	-0.80817300
C	4.24740100	2.97504500	-1.94060200
C	4.07500400	3.58455100	-3.15332100
C	3.09561500	4.60409300	-3.30684100
C	2.31157900	4.97770200	-2.24842300
C	2.45431400	4.36228900	-0.96418400
C	1.66417400	4.74681800	0.14057200
C	1.88720600	4.16672400	1.40942900
C	0.52417500	5.68321500	-0.06256500
C	-0.56070900	5.27092000	-0.85343400
C	-1.66906400	6.09812300	-1.02886600
C	-1.71165200	7.35354000	-0.41847000
C	-0.63222500	7.77966300	0.35751300
C	0.47821700	6.95216300	0.53020100
C	-2.51399700	-4.06733700	-1.92294200
C	-2.17674900	-3.71434000	-3.20211800
C	-0.82347800	-3.44737100	-3.54552000

C	0.15726400	-3.53657100	-2.59626800
C	-0.14621300	-3.91094900	-1.24890600
C	0.85471600	-3.98033600	-0.26139600
C	0.52200500	-4.31827500	1.06657500
C	1.51787600	-4.38563100	2.09132500
C	1.20052400	-4.74462300	3.37295000
C	-0.13866500	-5.08225200	3.70758400
C	-1.12377700	-5.02986700	2.75810800
C	-0.84520600	-4.62545500	1.41278000
C	-1.85347000	-4.55499700	0.42413400
C	-1.52062200	-4.19289800	-0.89991700
C	-3.26332300	-4.87387100	0.78245500
C	-3.95629500	-4.09565900	1.71955300
C	-5.26913400	-4.40164500	2.07232100
C	-5.91897400	-5.48791900	1.48676700
C	-5.24176100	-6.27003800	0.54967300
C	-3.92528300	-5.96803100	0.20493500
O	2.03256200	-1.54465000	0.41266400
O	3.05130300	0.35158700	-0.90303400
P	1.56044100	-0.30031400	-0.59423400
C	4.20454500	-2.17074300	-0.37232000
O	0.71272900	0.61822400	0.24413500
H	2.64858700	-5.56116400	-1.56797800
H	4.76756900	-6.17514900	-2.62610600
H	7.11081800	-5.64096100	-3.22586000
H	8.09540000	-3.45264200	-2.54492300
H	6.77588000	-1.84852300	-1.23280200
H	5.97164200	2.44870400	1.80959000
H	6.13227000	-3.06953600	1.19811700
H	8.06137900	-3.00517800	2.70703500
H	8.95052500	-0.82621100	3.53638100
H	7.82383700	1.28399700	2.88872500
H	0.43773300	5.37243400	2.48300700
H	0.70559100	4.23564600	4.62508500
H	2.27669500	2.29575700	4.83252600
H	3.60548600	1.58012300	2.90262400
H	4.99723900	2.20165200	-1.81883300
H	4.68969800	3.29474000	-4.00072700
H	2.97625200	5.09502100	-4.26906600
H	1.57407800	5.76204900	-2.37041700
H	-0.52565500	4.29599100	-1.32959400
H	-2.50182100	5.75371700	-1.63385800
H	-2.57712900	7.99679700	-0.55081100
H	-0.65163700	8.75950900	0.82680100
H	1.31899800	7.28640500	1.13103800
H	-3.55105400	-4.24114900	-1.66622600

H	-2.95169500	-3.62562100	-3.95944100
H	-0.57186400	-3.14842100	-4.55886800
H	1.17880900	-3.28017000	-2.84061900
H	2.54295600	-4.14754700	1.83354900
H	1.97462100	-4.78527900	4.13398400
H	-0.37860200	-5.39471400	4.72030500
H	-2.13744700	-5.30898800	3.01840900
H	-3.45693500	-3.24253300	2.16678400
H	-5.78773900	-3.78426000	2.80101600
H	-6.94389800	-5.72470800	1.75846900
H	-5.73669300	-7.12183300	0.09128700
H	-3.39533100	-6.58285800	-0.51640800
C	5.00451900	-3.08655300	-1.13378500
C	4.42636700	-4.32346100	-1.56836600
C	-2.37459800	2.51105600	-2.87079700
C	0.02822000	2.91338800	-4.27412100
H	0.61839200	0.98869000	-3.48045400
C	-2.17769200	3.66490200	-3.63263500
C	-0.98555600	3.86951500	-4.33124400
H	0.97221200	3.08355200	-4.78062000
H	-2.96019600	4.41793400	-3.65609000
H	-0.84441700	4.78414800	-4.89997300
C	-3.56706600	2.35910900	-1.99499200
C	-3.49198300	2.84000700	-0.68058000
C	-4.74384400	1.71420700	-2.40519000
C	-4.52771000	2.63457900	0.22105500
H	-2.59609600	3.35008000	-0.35356300
C	-5.79417500	1.51180200	-1.51657600
H	-4.82009400	1.33814700	-3.42101200
C	-5.67465800	1.95131600	-0.19449400
H	-4.43376100	2.98818700	1.24483600
H	-6.68842300	0.97476200	-1.81314000
C	-3.24536800	-0.56124500	-0.31865000
C	-4.51403400	-1.23063300	-0.61066500
C	-2.91644700	-0.20286400	1.05934100
C	-4.82084000	-1.70097400	-1.89618800
C	-5.47588800	-1.36486700	0.41657000
C	-3.94777800	-0.33856500	2.05063700
C	-6.04942900	-2.28962100	-2.15300400
H	-4.07915800	-1.60216000	-2.67987000
C	-6.72379800	-1.95367000	0.13537800
C	-5.15575000	-0.88031700	1.72679200
H	-3.75520400	-0.00601100	3.06090700
C	-7.01125600	-2.41602500	-1.13596200
H	-6.26883700	-2.65707000	-3.15146600
H	-7.45424500	-2.04373700	0.93434200

H	-5.91566600	-0.97544300	2.49871000
H	-7.97073500	-2.87760100	-1.34694500
O	-6.69822000	1.66667100	0.66520300
H	-6.46156800	2.00813400	1.54192800
N	-1.70695500	0.23530500	1.36452300
H	-0.94423200	0.27592200	0.65777700
C	-1.28726300	0.76415500	2.66574700
H	-1.53348100	0.04968900	3.45855800
H	-0.19906700	0.83587200	2.59896500
C	-1.89999400	2.12087200	2.94512300
C	-1.62676600	3.18621300	2.08107700
C	-2.75518600	2.32627300	4.03043300
C	-2.22005700	4.43057900	2.28372100
H	-0.93687300	3.03346700	1.25675900
C	-3.34725900	3.57372300	4.24281500
H	-2.95495100	1.50858200	4.72021800
C	-3.08602000	4.62499700	3.36317300
H	-2.00298000	5.24698600	1.60305500
H	-4.00818000	3.72180000	5.09251500
H	-3.54698000	5.59600100	3.52137700
C	-1.36340200	1.52342200	-2.85680300
C	-2.44061400	-0.12299900	-1.29752200
C	-0.16319900	1.73886900	-3.55068700

Int5-C2

C	4.73628200	-0.88547600	1.05856600
C	4.63085600	1.74375900	0.47664100
C	-4.69810600	-1.00534000	0.41176400
C	-3.62972100	-1.84615700	0.14231600
C	-3.42630700	-3.09078200	0.80175200
C	-4.38247200	-3.50426700	1.70297500
C	-6.43923100	-3.10171600	3.01086100
C	-7.46979200	-2.27592300	3.39464200
C	-7.58835800	-0.98861900	2.82097400
C	-6.69764900	-0.56294700	1.86136000
C	-5.92394000	0.70953900	-1.04706600
C	-5.93959900	2.00631200	-1.65687700
C	-4.80114700	2.84332500	-1.54608600
C	-3.66296100	2.42887500	-0.88989200
C	-3.66931900	1.14942900	-0.26989100
C	-4.77293500	0.30523100	-0.29326000
C	-7.04329100	-0.14140600	-1.25529300
C	-8.13207500	0.28245300	-1.98278700
C	-8.16269900	1.57947900	-2.54682700
C	-7.08417700	2.41770200	-2.38963600
C	0.37724200	4.34594500	-3.08680200

C	0.07268700	3.64636300	-4.22374300
C	-1.07439800	2.80706000	-4.26862900
C	-1.88842900	2.69503300	-3.17562000
C	-1.62468000	3.42467600	-1.97319800
C	-2.44439400	3.28956200	-0.83480600
C	-2.11677400	3.96841900	0.35618100
C	-2.89903200	3.81038200	1.54299000
C	-2.56712800	4.45233000	2.70622200
C	-1.44194800	5.32073900	2.74696900
C	-0.67195800	5.50898300	1.63020400
C	-0.95933300	4.83112700	0.40304700
C	-0.14362000	4.97781400	-0.73979300
C	-0.45689800	4.27653400	-1.92416300
C	1.08082400	5.82560100	-0.67989900
C	2.15863200	5.45441000	0.13679900
C	3.31775500	6.22606700	0.18719700
C	3.41933500	7.38756300	-0.58010400
C	2.35093600	7.77178900	-1.39263800
C	1.19154700	6.99764300	-1.44132400
C	1.09992800	-4.40097700	2.38184800
C	0.85473700	-3.77026000	3.57035200
C	-0.39288300	-3.12837300	3.79596900
C	-1.36682300	-3.15652300	2.83708000
C	-1.16382300	-3.81817000	1.58455800
C	-2.15245900	-3.83400500	0.57850100
C	-1.89477300	-4.45475500	-0.65879900
C	-2.87933600	-4.48733900	-1.69463500
C	-2.61618400	-5.05105400	-2.91320300
C	-1.34031600	-5.62056800	-3.17484200
C	-0.37206900	-5.62454000	-2.20619700
C	-0.60702100	-5.05638800	-0.91243700
C	0.38816900	-5.03353200	0.08947800
C	0.11998000	-4.43246600	1.33780300
C	1.73975300	-5.59822400	-0.18905400
C	2.84896000	-4.74521200	-0.27799500
C	4.11632200	-5.24757300	-0.56742600
C	4.29804700	-6.61730500	-0.76624900
C	3.20336100	-7.47877300	-0.66932500
C	1.93496500	-6.97298800	-0.38311600
O	-2.71238200	-1.46523400	-0.81223600
O	-2.52435500	0.75826500	0.38944100
P	-1.55466300	-0.37417000	-0.36750800
O	-0.70074800	-0.94037700	0.72853100
O	-0.98121100	0.17626500	-1.63337700
H	-4.25737000	-4.45478100	2.21455100
H	-6.32006100	-4.08411000	3.46100300

H	-8.18279600	-2.60209200	4.14640500
H	-8.38678000	-0.32670200	3.14455400
H	-6.79482400	0.42866100	1.43629400
H	-4.81872600	3.82211700	-2.01786700
H	-7.02725500	-1.14077800	-0.83776100
H	-8.97357200	-0.38845200	-2.13086900
H	-9.03074800	1.90332300	-3.11381000
H	-7.08323300	3.40901800	-2.83598600
H	1.26616400	4.96415400	-3.05196400
H	0.71936900	3.72002800	-5.09428900
H	-1.28857100	2.23352400	-5.16544800
H	-2.73401100	2.02025900	-3.19334100
H	-3.76521100	3.16011700	1.50815500
H	-3.17153400	4.30909500	3.59738200
H	-1.19623800	5.84192000	3.66797800
H	0.17900200	6.17813400	1.66714500
H	2.07769400	4.56224700	0.74338400
H	4.13761700	5.91711600	0.83045100
H	4.32106700	7.99243300	-0.54216600
H	2.41792900	8.67862500	-1.98741600
H	0.36039100	7.29769900	-2.07290800
H	2.05410600	-4.88488900	2.21222500
H	1.61990300	-3.74796700	4.34114300
H	-0.56449000	-2.59852800	4.72806200
H	-2.30551600	-2.64483400	3.00597600
H	-3.84981500	-4.04644000	-1.49636400
H	-3.37885800	-5.05758100	-3.68658000
H	-1.13364700	-6.05402800	-4.14946300
H	0.59752300	-6.06269500	-2.41291600
H	2.70864000	-3.68307500	-0.12223600
H	4.95636800	-4.56233700	-0.64900600
H	5.28452300	-7.01245000	-0.99331400
H	3.33664300	-8.54719300	-0.81599100
H	1.08348800	-7.64347200	-0.30957200
H	3.70400500	1.48649800	-1.60167600
C	-5.64115100	-1.40283700	1.41680000
C	5.85460100	1.57465900	0.02852400
H	6.34884700	2.45936700	-0.38302300
C	3.37198100	2.07482800	0.77939000
C	2.97892700	2.54064100	2.11568700
C	2.32251000	1.82707500	-0.22330100
C	3.92807500	2.93260400	3.07046900
C	1.60303900	2.59334700	2.44361900
C	0.95962000	1.89939600	0.17218000
C	3.52409100	3.34712100	4.33199800
H	4.98262700	2.89553600	2.81495500

C	1.21231400	3.01069300	3.72894100
C	0.62873300	2.22236200	1.45478400
H	0.18819900	1.66121300	-0.54573700
C	2.16124700	3.38284600	4.66720400
H	4.26964800	3.64608500	5.06314200
H	0.15323200	3.04238500	3.96363500
H	-0.41919700	2.23289200	1.73415500
H	1.85295500	3.70590400	5.65677500
C	6.63814100	0.33038500	-0.01628400
C	6.09256900	-0.88358400	0.45933900
C	7.91978300	0.34293100	-0.58395000
C	6.84286600	-2.05468300	0.33651100
C	-5.49529600	-2.68891900	2.03379900
C	8.65709400	-0.83341300	-0.69371400
H	8.33309000	1.27982800	-0.94876000
C	8.11612700	-2.03556000	-0.23678300
H	6.41843100	-2.98570500	0.70075700
C	4.54561400	-0.52830100	2.40939900
C	3.61581400	-1.23640800	0.28783200
H	9.64760600	-0.81197800	-1.13825000
H	8.68415700	-2.95721500	-0.32353200
C	3.27379700	-0.42939400	2.94219700
H	5.41089900	-0.29055200	3.02187500
C	2.33472300	-1.12592200	0.80461300
H	3.75349200	-1.54988500	-0.74118600
C	2.14824100	-0.68007700	2.13207400
H	3.11155000	-0.10958400	3.96579800
H	1.46291100	-1.36048500	0.20647500
O	0.94400800	-0.44702500	2.65279400
H	0.23200500	-0.63902600	1.96771500
N	2.69964200	1.55241800	-1.46743600
C	1.90168600	1.01722700	-2.58322700
H	0.84567100	1.01218100	-2.31906600
H	2.03971800	1.69487300	-3.43224100
C	2.37481400	-0.38681600	-2.90089800
C	3.68548400	-0.61032100	-3.34202000
C	1.52623800	-1.47872600	-2.68938800
C	4.15638100	-1.90913300	-3.53415300
H	4.34969500	0.23261200	-3.53030700
C	1.99337500	-2.77740400	-2.89548600
H	0.50827800	-1.29585600	-2.36002900
C	3.31001700	-2.99644300	-3.30396400
H	5.17956800	-2.07153900	-3.86172300
H	1.32774000	-3.61654800	-2.72291100
H	3.67538300	-4.01055900	-3.43462000

TS6

C	4.71651100	-0.40232400	1.00237700
C	4.49474100	1.55672900	0.64763900
C	-4.60402100	-1.24561300	0.45152000
C	-3.49471700	-2.02323600	0.16017400
C	-3.20103400	-3.24948900	0.82044600
C	-4.11133000	-3.71462900	1.74417200
C	-6.15867400	-3.42873400	3.09675700
C	-7.22706900	-2.66313000	3.50117900
C	-7.43432000	-1.38760300	2.92626400
C	-6.59234500	-0.91455300	1.94515200
C	-5.96446500	0.38060300	-0.98708600
C	-6.07489900	1.67000100	-1.60216000
C	-4.98936800	2.57754000	-1.51944500
C	-3.81246100	2.24020200	-0.88795300
C	-3.72431600	0.96523300	-0.26430100
C	-4.77348700	0.05386800	-0.25799000
C	-7.03258600	-0.54003600	-1.16600800
C	-8.16162600	-0.18952100	-1.87084500
C	-8.28593400	1.09961100	-2.44001800
C	-7.25887300	2.00460800	-2.31094100
C	0.10628200	4.35179000	-3.12994200
C	-0.15752800	3.61574700	-4.25389800
C	-1.25860000	2.71640400	-4.28473000
C	-2.06800800	2.58039300	-3.19100100
C	-1.84449600	3.34255300	-2.00103900
C	-2.65358700	3.18146100	-0.85849500
C	-2.37269600	3.90853100	0.31543900
C	-3.14883700	3.73529300	1.50417600
C	-2.85599300	4.42173800	2.65248200
C	-1.77787900	5.34882400	2.67649300
C	-1.01466000	5.55135500	1.55756400
C	-1.26421700	4.83366100	0.34529700
C	-0.44945400	4.99339300	-0.79530600
C	-0.72475900	4.25744700	-1.96742600
C	0.74062600	5.89021800	-0.74084600
C	1.84507100	5.55104100	0.05481400
C	2.96883100	6.37407400	0.10086400
C	3.00783800	7.55144100	-0.64796500
C	1.91300700	7.90104100	-1.44032100
C	0.78842200	7.07679700	-1.48544500
C	1.41488100	-4.31988800	2.32928100
C	1.14687200	-3.73112200	3.53485100
C	-0.13452200	-3.17096000	3.78764900
C	-1.11532200	-3.23304600	2.83661100
C	-0.89113500	-3.85964200	1.56972300

C	-1.89027000	-3.91787400	0.57456200
C	-1.61441800	-4.51591800	-0.67030000
C	-2.60913500	-4.59964900	-1.69362600
C	-2.33233300	-5.14951100	-2.91543200
C	-1.03284900	-5.65484600	-3.19345700
C	-0.05273800	-5.60704800	-2.23821400
C	-0.29987900	-5.04693400	-0.94346300
C	0.70692100	-4.97086500	0.04263200
C	0.42277400	-4.39544300	1.29892000
C	2.08347500	-5.46145800	-0.25628400
C	3.13401900	-4.54682100	-0.41328700
C	4.42332200	-4.98232100	-0.71470800
C	4.68521100	-6.34596700	-0.85797600
C	3.64890900	-7.26772600	-0.69628500
C	2.35848300	-6.82860900	-0.39825700
O	-2.62523100	-1.59147200	-0.81798300
O	-2.54053900	0.64670700	0.37034200
P	-1.52865100	-0.43088500	-0.40036200
O	-0.63729300	-0.97119200	0.68685600
O	-0.98915100	0.13369600	-1.67025800
H	-3.91915600	-4.65376600	2.25597300
H	-5.97185100	-4.40039900	3.54719200
H	-7.90251500	-3.02721100	4.26999900
H	-8.26287500	-0.77248700	3.26591600
H	-6.75729300	0.06744100	1.51874300
H	-5.07935600	3.55144200	-1.99298700
H	-6.94491100	-1.53411400	-0.74471600
H	-8.96245500	-0.91265700	-1.99695400
H	-9.18486500	1.36506800	-2.98891000
H	-7.32986100	2.99132600	-2.76172100
H	0.95995800	5.01834200	-3.10429400
H	0.48626400	3.70726200	-5.12476600
H	-1.44058100	2.11700700	-5.17172800
H	-2.87806900	1.86292000	-3.19895800
H	-3.97813800	3.03774900	1.48304700
H	-3.45470200	4.26688800	3.54561500
H	-1.56243800	5.90158000	3.58659100
H	-0.19647000	6.26094300	1.58096500
H	1.81527800	4.64198200	0.64179600
H	3.81186700	6.09205100	0.72617100
H	3.88278300	8.19470100	-0.61206400
H	1.93201900	8.81874000	-2.02185900
H	-0.06426000	7.34869700	-2.10108300
H	2.39376100	-4.74260800	2.13670800
H	1.91762800	-3.68259800	4.29917800
H	-0.32804700	-2.67430400	4.73369900

H	-2.07952100	-2.77948400	3.02659300
H	-3.59895500	-4.21095000	-1.48287100
H	-3.10350000	-5.19597900	-3.67884900
H	-0.81842300	-6.08090800	-4.16959300
H	0.93503500	-5.99775800	-2.45445600
H	2.92903300	-3.48960600	-0.30448000
H	5.21693200	-4.25174100	-0.85053400
H	5.68857500	-6.68951100	-1.09444000
H	3.84495400	-8.33124000	-0.80203200
H	1.55204300	-7.54573100	-0.27431900
H	3.64069400	1.51771800	-1.49291300
C	-5.49849500	-1.69288700	1.47964800
C	5.71157100	1.82707600	0.17628500
H	5.97530500	2.83433800	-0.14282400
C	3.21104000	2.06181700	0.88501000
C	2.81131900	2.55418000	2.18477300
C	2.22030400	1.89226900	-0.14390200
C	3.75047800	2.88703600	3.18540900
C	1.42478600	2.69852400	2.47325000
C	0.84214900	2.02947700	0.19113200
C	3.33078200	3.33378000	4.42499800
H	4.80916400	2.78817000	2.96658500
C	1.02181500	3.15286900	3.74703600
C	0.47180900	2.37038000	1.46048100
H	0.09000800	1.82847500	-0.55740900
C	1.95797300	3.46582300	4.71379800
H	4.06765900	3.58999300	5.18137700
H	-0.04067400	3.26008200	3.94331300
H	-0.58356900	2.42189300	1.70356900
H	1.64011400	3.81861800	5.69029900
C	6.66484800	0.72404700	0.07084000
C	6.11735700	-0.50024200	0.50009000
C	7.96938200	0.77961600	-0.42206000
C	6.87006100	-1.66725300	0.42772600
C	-5.26294800	-2.96527900	2.09720100
C	8.72172500	-0.39485400	-0.48983900
H	8.39066200	1.72416800	-0.75514000
C	8.17649600	-1.61093600	-0.07125100
H	6.44219200	-2.61093200	0.75453500
C	4.49832300	-0.32427800	2.43268000
C	3.64599600	-0.95809400	0.21135900
H	9.73669100	-0.36211800	-0.87486000
H	8.76873000	-2.51911200	-0.13282800
C	3.23764000	-0.40019000	2.94222600
H	5.34636400	-0.09749500	3.07095500
C	2.37660300	-1.01846800	0.71209900

H	3.84488900	-1.22950300	-0.81906900
C	2.13535200	-0.62547600	2.06451700
H	3.02511200	-0.22715300	3.99086900
H	1.53336200	-1.32819000	0.10745800
O	0.93719500	-0.47398000	2.55612100
H	0.21819800	-0.63691800	1.83274600
N	2.63636100	1.59953000	-1.39040800
C	1.86276300	1.10942600	-2.53762400
H	0.80734800	1.05053700	-2.27849100
H	1.97199300	1.83250900	-3.35429500
C	2.37801500	-0.25823700	-2.93995900
C	3.69127000	-0.40927600	-3.40491600
C	1.57774900	-1.39437400	-2.77561400
C	4.21312400	-1.67762100	-3.66165800
H	4.31718300	0.46893400	-3.55929800
C	2.09527400	-2.66236800	-3.04494900
H	0.55505600	-1.26938200	-2.43398200
C	3.41540600	-2.80895100	-3.47399900
H	5.23744600	-1.78195300	-4.00930600
H	1.46534900	-3.53513600	-2.90748200
H	3.82028200	-3.79986100	-3.65700000

TS6-S1

C	-2.01476500	4.92868500	0.13361800
C	-3.10186000	4.10386000	1.63575600
C	1.98485100	-1.79702500	2.24343900
C	2.65042400	-1.24722200	1.15789200
C	3.70829700	-0.30059100	1.29910500
C	4.00221300	0.15126300	2.56817300
C	3.54574900	0.24181300	4.99707600
C	2.83475000	-0.17961500	6.09771600
C	1.80538700	-1.13803800	5.94402000
C	1.51841000	-1.66587300	4.70424000
C	1.20149000	-4.17820000	2.68632900
C	0.22167100	-5.20401600	2.49291300
C	-0.86675600	-4.96596700	1.61794200
C	-0.98611300	-3.79009600	0.91092200
C	-0.00670300	-2.77617800	1.11051700
C	1.03205200	-2.91724800	2.02101700
C	2.32497100	-4.47426600	3.50566900
C	2.44712600	-5.69531500	4.12938000
C	1.45517500	-6.69084200	3.96950300
C	0.36965500	-6.44759600	3.16139500
C	-2.98501900	-4.04475300	-3.64639900
C	-1.80872500	-4.56394800	-4.11688800
C	-0.71045700	-4.77503100	-3.24077700

C	-0.81549600	-4.46059100	-1.91450600
C	-2.02250200	-3.91755000	-1.37789700
C	-2.13866200	-3.58306700	-0.01501300
C	-3.35745400	-3.09390800	0.49633200
C	-3.50696800	-2.74129000	1.87595800
C	-4.69471800	-2.26902900	2.36849800
C	-5.81738900	-2.12140800	1.50816100
C	-5.71347800	-2.42867600	0.17735300
C	-4.49063800	-2.91866500	-0.38442800
C	-4.35542100	-3.19260200	-1.76207400
C	-3.14165800	-3.70456700	-2.26628900
C	-5.47888400	-2.89373300	-2.69497400
C	-5.37271200	-1.81607700	-3.58699300
C	-6.42173900	-1.50876300	-4.45203200
C	-7.58937700	-2.27406700	-4.44230700
C	-7.70056300	-3.35305400	-3.56392600
C	-6.65175700	-3.66049400	-2.69667600
C	5.25819000	3.25614200	-1.84546100
C	4.54894100	4.18972300	-1.13974400
C	3.78593100	3.79822400	-0.00662000
C	3.76721600	2.49145100	0.39992300
C	4.50063100	1.48179300	-0.29831500
C	4.49609200	0.13038100	0.11015400
C	5.25405700	-0.82746500	-0.59957500
C	5.33662900	-2.19247300	-0.18027500
C	6.05748500	-3.11551100	-0.88743900
C	6.74629700	-2.73532700	-2.07117300
C	6.71327100	-1.43612200	-2.49902800
C	5.98899800	-0.43417500	-1.77776000
C	5.97528600	0.91109200	-2.19797000
C	5.25609400	1.87589800	-1.46575200
C	6.72231800	1.31551800	-3.42408100
C	6.03913000	1.55373100	-4.62434800
C	6.73317400	1.93236600	-5.77330400
C	8.12088500	2.07866600	-5.73780500
C	8.81056100	1.84380400	-4.54735300
C	8.11534200	1.46478200	-3.39902000
O	2.34100500	-1.68767600	-0.10534200
O	-0.14965100	-1.59193200	0.42375200
P	0.92315400	-1.19035200	-0.81461800
O	0.85888500	0.32351000	-0.84940100
O	0.71254400	-2.00592400	-2.03138500
H	4.81274400	0.86159200	2.70382900
H	4.32955900	0.98821200	5.09754600
H	3.05351800	0.22743200	7.08064700
H	1.22935800	-1.45322500	6.80922300

H	0.72175100	-2.39242400	4.59669900
H	-1.61516800	-5.74224300	1.48383900
H	3.09988100	-3.72765300	3.62826900
H	3.31888500	-5.89868800	4.74485600
H	1.56174200	-7.64786300	4.47196500
H	-0.39029800	-7.20982700	3.00908200
H	-3.82278700	-3.89489900	-4.31676600
H	-1.71273300	-4.81884900	-5.16874500
H	0.22321100	-5.16834800	-3.63038500
H	0.03080300	-4.59706800	-1.25436000
H	-2.65168100	-2.85524500	2.53245200
H	-4.78050300	-2.00496000	3.41863000
H	-6.75673500	-1.74977400	1.90804900
H	-6.56451700	-2.29092500	-0.47914800
H	-4.46342700	-1.22626500	-3.59932000
H	-6.32374600	-0.66796100	-5.13340000
H	-8.40621200	-2.03430000	-5.11753700
H	-8.60262800	-3.95886800	-3.55547700
H	-6.73516300	-4.50159700	-2.01408100
H	5.83986600	3.55205400	-2.71076900
H	4.56549000	5.23194000	-1.44680200
H	3.20859800	4.54112300	0.53696500
H	3.16498800	2.20442200	1.24929800
H	4.81678300	-2.49561900	0.72039900
H	6.09944400	-4.14609200	-0.54666700
H	7.30142600	-3.48004000	-2.63467600
H	7.24098700	-1.14449100	-3.39957500
H	4.95958200	1.43685000	-4.64772200
H	6.18976000	2.11062300	-6.69723200
H	8.66181900	2.37325000	-6.63284300
H	9.89075200	1.95664500	-4.51167700
H	8.64891300	1.28074000	-2.47089000
H	-4.29032500	2.52547900	0.39882000
C	2.24909900	-1.26956600	3.55086300
C	-4.12250600	4.95823800	1.64347800
H	-4.94694300	4.83611300	2.34466300
C	-2.56033200	2.93163100	2.16990200
C	-1.59595400	2.96239500	3.24835500
C	-2.81827100	1.69704900	1.48490100
C	-1.38300100	4.11537500	4.03515100
C	-0.81254500	1.80375700	3.50996300
C	-2.00675000	0.56181900	1.76963400
C	-0.40918100	4.12830100	5.01714400
H	-1.99049000	4.99637600	3.85365400
C	0.18447800	1.84540200	4.50808300
C	-1.02756200	0.63434800	2.72017000

H	-2.11479900	-0.34333100	1.19224700
C	0.39011900	2.99156100	5.25045300
H	-0.25923700	5.02518500	5.61212400
H	0.78552300	0.96160800	4.68174300
H	-0.38494900	-0.22449400	2.88136200
H	1.16174900	3.01144800	6.01402600
C	-4.11316400	6.05218100	0.67438700
C	-2.98027300	6.02721200	-0.16306700
C	-5.08937600	7.03686600	0.51561800
C	-2.83228700	6.98496200	-1.16071400
C	3.27289100	-0.27957100	3.70484500
C	-4.93280500	7.99610300	-0.48663500
H	-5.96211300	7.05272600	1.16266100
C	-3.81340400	7.97001700	-1.32131400
H	-1.96010100	6.96464000	-1.80760200
C	-0.86228300	5.22696400	0.95677400
C	-1.89096800	3.81520200	-0.77667700
H	-5.68806100	8.76519000	-0.61886800
H	-3.70159500	8.71834200	-2.10024600
C	0.10762200	4.28986400	1.14583500
H	-0.84814500	6.16420000	1.50390800
C	-0.92124900	2.86981300	-0.59419600
H	-2.63885400	3.68366600	-1.55136400
C	0.02907300	3.03757900	0.46277400
H	0.91587100	4.43000500	1.85425200
H	-0.86635000	1.97579100	-1.20497700
O	0.84689500	2.09947100	0.83153800
H	0.83719600	1.26047800	0.17216300
N	-3.82441000	1.64741900	0.58607400
C	-4.17296400	0.51795100	-0.27389700
H	-3.95020100	-0.40078800	0.26142900
H	-5.25922600	0.54394500	-0.40302900
C	-3.48407100	0.53645400	-1.62009200
C	-2.41136600	-0.31758000	-1.88427900
C	-3.92312400	1.41185000	-2.62184700
C	-1.77602600	-0.30220700	-3.12833800
H	-2.06814200	-1.01029100	-1.12518200
C	-3.28976200	1.43836900	-3.86352900
H	-4.77604000	2.06225800	-2.43404000
C	-2.21484400	0.57967600	-4.11616900
H	-0.93834900	-0.97193600	-3.29311600
H	-3.64001300	2.11768100	-4.63626000
H	-1.72340800	0.59715100	-5.08484600

TS6-S2

C	4.52874800	1.09039100	-0.41464000

C	4.16094700	2.49278200	1.02023900
C	-3.45458300	-3.23153200	0.13968400
C	-2.10469600	-3.25483800	-0.17561800
C	-1.26112900	-4.37349500	0.06993700
C	-1.82372500	-5.48552600	0.65846900
C	-3.72684500	-6.62692700	1.74534500
C	-5.02453100	-6.61800300	2.20050600
C	-5.82881000	-5.47036100	2.00834000
C	-5.33366400	-4.36956200	1.34636800
C	-5.44045200	-2.15129600	-1.05203700
C	-6.17656900	-0.96487100	-1.37409900
C	-5.67928200	0.29593400	-0.96624000
C	-4.49499100	0.42176900	-0.26878200
C	-3.81830000	-0.77014000	0.10914800
C	-4.26137200	-2.03897200	-0.24182200
C	-5.89766900	-3.38572300	-1.58895900
C	-7.04321300	-3.44928100	-2.34945800
C	-7.79560500	-2.28271700	-2.62238200
C	-7.36281500	-1.06704400	-2.14876600
C	-2.99365400	4.79185700	-2.05295200
C	-3.04111300	4.27398500	-3.31775500
C	-3.30676700	2.89272700	-3.51599900
C	-3.57375000	2.08173700	-2.44728400
C	-3.57477100	2.58727100	-1.10753300
C	-3.92203400	1.77287000	-0.00543100
C	-3.75817200	2.26176300	1.30689100
C	-4.05599000	1.45953400	2.45292600
C	-3.81096800	1.90878400	3.72182900
C	-3.25793900	3.20195800	3.92774300
C	-3.01508800	4.02677100	2.86224200
C	-3.28185200	3.60907600	1.51839200
C	-3.08337200	4.47298300	0.41555600
C	-3.20696000	3.97019800	-0.89920900
C	-2.78370300	5.91311100	0.65651900
C	-1.60502600	6.51570000	0.19312900
C	-1.34631000	7.86444300	0.43428000
C	-2.26421100	8.64027700	1.14250200
C	-3.44527900	8.05626800	1.60390900
C	-3.70115300	6.70776800	1.36279300
C	3.52898100	-4.27602800	1.44625800
C	3.14699700	-4.36816100	2.75622200
C	1.76820400	-4.40325300	3.09876200
C	0.81115700	-4.37450300	2.12211200
C	1.16163400	-4.32104800	0.73496600
C	0.18514900	-4.30383200	-0.28398000
C	0.57472500	-4.19189600	-1.63623000

C	-0.38097400	-4.21256800	-2.69930900
C	0.00240900	-4.08770500	-4.00685600
C	1.37665900	-3.94291000	-4.34006000
C	2.32786600	-3.93375300	-3.35584700
C	1.97061600	-4.05748700	-1.97557800
C	2.94696400	-4.07861500	-0.95746100
C	2.56335800	-4.23796500	0.38947700
C	4.38731400	-3.90485400	-1.30502200
C	4.87787000	-2.64340800	-1.67125900
C	6.22892500	-2.46524200	-1.96638100
C	7.10917600	-3.54687800	-1.90974100
C	6.62932700	-4.80912900	-1.55683300
C	5.27857100	-4.98523600	-1.25622700
O	-1.53632900	-2.15025200	-0.77372100
O	-2.68870000	-0.63860300	0.88461400
P	-1.19956100	-0.86454100	0.21062500
O	-0.35025700	-1.29356500	1.38342300
O	-0.79089800	0.25531000	-0.68909500
H	-1.20649200	-6.36008600	0.84483500
H	-3.08999100	-7.49344500	1.90494500
H	-5.42945000	-7.48256200	2.71864200
H	-6.84519500	-5.45521100	2.39162700
H	-5.95578100	-3.49269600	1.21203900
H	-6.22125500	1.19178400	-1.25568500
H	-5.32788700	-4.28646800	-1.39846300
H	-7.36896300	-4.40522300	-2.74979500
H	-8.70139300	-2.34879200	-3.21821200
H	-7.91334600	-0.15666800	-2.37224000
H	-2.78024000	5.84353500	-1.91093700
H	-2.85327200	4.91595600	-4.17321400
H	-3.29410000	2.47934400	-4.52040800
H	-3.76858400	1.02821600	-2.60202700
H	-4.46435000	0.46873900	2.30073900
H	-4.02845300	1.27193700	4.57441200
H	-3.03344000	3.53844600	4.93614300
H	-2.60095700	5.01450500	3.02587800
H	-0.89562900	5.92442700	-0.37293300
H	-0.42522000	8.30880900	0.06633400
H	-2.06374800	9.69137100	1.33019800
H	-4.17123200	8.65249900	2.14989100
H	-4.62102400	6.25544600	1.72089300
H	4.57997100	-4.21878900	1.18822100
H	3.89791300	-4.39167100	3.54137500
H	1.47388200	-4.43572700	4.14396400
H	-0.23735600	-4.36626800	2.39072000
H	-1.42927100	-4.33251000	-2.45300400

H	-0.74483900	-4.10326900	-4.79512300
H	1.67150300	-3.85077900	-5.38177800
H	3.37693900	-3.84252600	-3.61251400
H	4.19129900	-1.80398100	-1.71055500
H	6.59878900	-1.47779300	-2.22894900
H	8.16206400	-3.40739400	-2.13889600
H	7.30662100	-5.65776300	-1.51476800
H	4.90344700	-5.96552800	-0.97647700
H	2.28310400	3.43152600	-0.01662700
C	-4.00698900	-4.35555800	0.83839500
C	4.98178200	3.44395100	0.58159800
H	5.06202400	4.39538700	1.10576200
C	3.18483200	2.13709100	1.95510300
C	3.51143000	1.42816600	3.17285900
C	1.80703900	2.31989000	1.59156200
C	4.83959700	1.29860700	3.63422000
C	2.46565500	0.81852000	3.92188300
C	0.78548500	1.70747500	2.37707000
C	5.12286700	0.57348200	4.77633800
H	5.63869200	1.77288800	3.07353100
C	2.78263800	0.07565700	5.07982000
C	1.11719800	0.96105800	3.47078700
H	-0.25064900	1.80433700	2.07861100
C	4.09079100	-0.05166900	5.50384900
H	6.15140200	0.48445600	5.11503500
H	1.97425400	-0.39873200	5.62967800
H	0.32907300	0.45543800	4.02045400
H	4.32456800	-0.62575400	6.39527900
C	5.74271300	3.19423100	-0.64134900
C	5.47152400	1.93184600	-1.20559200
C	6.62651000	4.07381900	-1.26830800
C	6.07053800	1.56408400	-2.40591000
C	-3.17987800	-5.50233300	1.07266900
C	7.23276200	3.69281900	-2.46684200
H	6.83225500	5.04726700	-0.83165300
C	6.95331500	2.44853300	-3.03588100
H	5.84454100	0.60038300	-2.85263200
C	5.07983900	0.11235800	0.50081200
C	3.19580900	0.84286300	-0.90255400
H	7.91982200	4.37215700	-2.96267100
H	7.42101600	2.16453800	-3.97386200
C	4.26222000	-0.76018400	1.15045500
H	6.14088800	0.15671200	0.72397200
C	2.36333600	-0.01860400	-0.24107100
H	2.82185800	1.42907400	-1.73645900
C	2.85832100	-0.74360200	0.88638400

H	4.62189200	-1.43922600	1.91359500
H	1.32050900	-0.10140700	-0.52962800
O	2.10082900	-1.42122700	1.69500400
H	1.07718100	-1.35569300	1.48834300
N	1.50510300	3.04854600	0.50463400
C	0.18321000	3.16830300	-0.12005400
H	-0.33745600	2.21056400	-0.06019100
H	-0.40495300	3.91903300	0.42054800
C	0.36174500	3.54975000	-1.56943200
C	0.90268400	4.79506300	-1.91829400
C	0.02465500	2.64166000	-2.57833300
C	1.08947700	5.13778200	-3.25670300
H	1.17587100	5.50226700	-1.13712200
C	0.21238900	2.98701200	-3.91732000
H	-0.39331000	1.67988400	-2.30043200
C	0.74073000	4.23184600	-4.26042800
H	1.50240100	6.10889400	-3.51576400
H	-0.06237600	2.27954900	-4.69466300
H	0.88203300	4.49689300	-5.30472800

TS6-S3

C	4.49334700	-1.83174900	0.97339100
C	4.68480500	0.20213500	0.90215400
C	-4.73463800	-1.17720400	0.29624900
C	-3.66767300	-1.91754400	-0.19034200
C	-3.46180400	-3.29018100	0.13140200
C	-4.41824700	-3.92456300	0.89355500
C	-6.46102200	-3.87356300	2.28146500
C	-7.47792000	-3.17161700	2.88492500
C	-7.58843200	-1.77726400	2.67514000
C	-6.70595900	-1.11942700	1.84822100
C	-6.01112100	0.82713600	-0.65672300
C	-6.05354200	2.23392200	-0.92560100
C	-4.90555500	3.02561500	-0.67572800
C	-3.73771600	2.47301300	-0.19704700
C	-3.72742100	1.08791900	0.12090400
C	-4.83191500	0.26418500	-0.06384100
C	-7.13540200	0.04203800	-1.03198400
C	-8.25325300	0.62097600	-1.58843600
C	-8.31056400	2.01756300	-1.80646300
C	-7.22778300	2.80214600	-1.48687600
C	0.03499400	4.81176000	-2.42049400
C	-0.44436400	4.39170300	-3.63081200
C	-1.59669900	3.56385200	-3.70002800
C	-2.25257400	3.20047800	-2.55691200
C	-1.81204100	3.64966800	-1.27151500

C	-2.49045400	3.28422500	-0.09125300
C	-1.99673900	3.68910200	1.16361700
C	-2.64757700	3.30835200	2.37868200
C	-2.16854100	3.70414000	3.59905500
C	-1.01439200	4.53165100	3.67801000
C	-0.35867700	4.91638400	2.53859600
C	-0.80309000	4.49866100	1.24352300
C	-0.11841000	4.86397200	0.06351200
C	-0.61878100	4.46258200	-1.19612800
C	1.15763000	5.62951500	0.15109000
C	2.28309000	5.05848000	0.76642800
C	3.49259600	5.74899000	0.82608500
C	3.60023500	7.02604100	0.27137200
C	2.48523000	7.60911300	-0.33339200
C	1.27500800	6.91788600	-0.38881200
C	1.05001200	-4.93023300	1.39737100
C	0.74929000	-4.71155300	2.71389800
C	-0.53515000	-4.23176000	3.08352600
C	-1.47971000	-3.98806200	2.12540100
C	-1.22499900	-4.23484200	0.73868200
C	-2.18292200	-3.94916300	-0.25888800
C	-1.88877900	-4.19513400	-1.61344500
C	-2.85739700	-3.96406000	-2.63882000
C	-2.57626800	-4.21312000	-3.95409100
C	-1.30235900	-4.72191300	-4.32775000
C	-0.34327800	-4.95350500	-3.37863600
C	-0.58739700	-4.68966800	-1.99290400
C	0.39549200	-4.91419100	-1.00371500
C	0.08286700	-4.72098700	0.36120200
C	1.78157900	-5.28698000	-1.40349100
C	2.55501300	-4.39009100	-2.15742700
C	3.87656300	-4.68382900	-2.48936200
C	4.45045600	-5.88834100	-2.07762000
C	3.68632500	-6.79995000	-1.34716900
C	2.36337400	-6.50287500	-1.01745300
O	-2.75996400	-1.30599900	-1.02802300
O	-2.56646200	0.57093900	0.65105500
P	-1.59345800	-0.36578500	-0.32566100
O	-0.81288500	-1.21851700	0.64253200
O	-0.94059000	0.42725100	-1.40414200
H	-4.29353700	-4.97599500	1.13828400
H	-6.34584700	-4.94070400	2.45455000
H	-8.18514100	-3.67990600	3.53408500
H	-8.37391500	-1.21829300	3.17594300
H	-6.79696400	-0.04996500	1.70320500
H	-4.93348200	4.08474100	-0.91698200

H	-7.10144100	-1.02993400	-0.88148300
H	-9.09737500	-0.00248100	-1.86950900
H	-9.20149500	2.46238700	-2.24036600
H	-7.24502200	3.87313600	-1.67286300
H	0.93804600	5.40387800	-2.38239700
H	0.08122300	4.66699000	-4.53907900
H	-1.94567900	3.20269400	-4.66320400
H	-3.10951300	2.54188300	-2.60860400
H	-3.53638200	2.69118600	2.31560300
H	-2.67721200	3.39773900	4.50866600
H	-0.65522300	4.86506400	4.64777500
H	0.51575400	5.55191900	2.60798000
H	2.20249000	4.06494300	1.19291400
H	4.35088100	5.28644500	1.30620500
H	4.54213000	7.56573200	0.31735800
H	2.55542000	8.60639600	-0.75901300
H	0.40825600	7.37425400	-0.85760200
H	2.04703200	-5.24943300	1.12178300
H	1.50628400	-4.87331300	3.47626500
H	-0.75441200	-4.02405500	4.12656900
H	-2.43823800	-3.57463100	2.40975400
H	-3.83305000	-3.58987100	-2.35060800
H	-3.32871400	-4.03043200	-4.71580800
H	-1.09409100	-4.93640900	-5.37234800
H	0.61882500	-5.35909200	-3.66915200
H	2.10552100	-3.45333300	-2.47431900
H	4.46209500	-3.97024100	-3.06278200
H	5.48153400	-6.11716300	-2.33245300
H	4.11853800	-7.74699000	-1.03567100
H	1.77150000	-7.21332700	-0.44804200
H	3.79150000	0.59672900	-1.20896800
C	-5.66529200	-1.81932100	1.18000400
C	5.93473500	0.26455400	0.44953000
H	6.41215200	1.22632400	0.26566100
C	3.52860600	0.90386000	1.25143300
C	3.25425600	1.28033000	2.62249200
C	2.51425200	1.08654700	0.25025500
C	4.24533700	1.23535600	3.62676000
C	1.94129100	1.69850200	2.97677300
C	1.21637500	1.52452400	0.64366800
C	3.94142400	1.57463500	4.93244200
H	5.25177700	0.92870000	3.35996400
C	1.65374500	2.03308300	4.31772800
C	0.93986000	1.76280700	1.96033700
H	0.43020000	1.59911300	-0.09520700
C	2.63661800	1.97132700	5.28647700

H	4.71797600	1.53699100	5.69155000
H	0.64448700	2.34792300	4.56428400
H	-0.07160000	2.02903200	2.24526200
H	2.40903100	2.23302600	6.31544900
C	6.63542700	-0.98768400	0.16584200
C	5.84561900	-2.12621400	0.42082500
C	7.92395900	-1.12449300	-0.35327600
C	6.33539500	-3.39603200	0.13244300
C	-5.52440700	-3.22243400	1.43580200
C	8.41523300	-2.40228000	-0.62820700
H	8.53165900	-0.24579800	-0.55115500
C	7.62506600	-3.53008400	-0.39389400
H	5.71363200	-4.27007500	0.30017900
C	4.28166700	-1.90394600	2.40204900
C	3.33745400	-2.01170500	0.13601300
H	9.41531500	-2.51788300	-1.03564200
H	8.01153500	-4.51882700	-0.62320000
C	3.02694600	-1.77696100	2.91952700
H	5.15049500	-1.95562400	3.05053800
C	2.07826900	-1.87593800	0.64483300
H	3.48925100	-2.17523500	-0.92314000
C	1.91042800	-1.63889900	2.04174100
H	2.84588000	-1.71101500	3.98616500
H	1.19413200	-1.90722800	0.02158700
O	0.76221900	-1.31586700	2.57292600
H	0.03918800	-1.22247300	1.84041300
N	2.81559900	0.80441500	-1.03635500
C	2.02966400	1.11449100	-2.22310000
H	2.10242200	0.25938300	-2.90529600
H	0.97113000	1.17967800	-1.95806300
C	2.52637200	2.36919100	-2.91724300
C	2.38494100	2.50277300	-4.30180800
C	3.10441200	3.41602300	-2.19384500
C	2.79851500	3.66699000	-4.94816200
H	1.93717000	1.69387200	-4.87426300
C	3.51675800	4.58384500	-2.83507900
H	3.21929800	3.33382400	-1.11965100
C	3.36373500	4.71362800	-4.21586300
H	2.68160900	3.75636700	-6.02495000
H	3.93902500	5.39342200	-2.24689900
H	3.68407100	5.62151800	-4.71941400

TS6-S4

C	-4.54388800	-0.40802300	-2.46406100
C	-4.64941400	1.14794700	-1.19025800
C	4.28555000	-1.23755100	-1.03697100

C	3.31836700	-2.09991500	-0.55758800
C	2.97711500	-3.33565100	-1.17977500
C	3.68442600	-3.70194200	-2.30263400
C	5.33831300	-3.20638200	-4.06996200
C	6.23503900	-2.34911300	-4.66413700
C	6.48546800	-1.08341400	-4.08579700
C	5.85997200	-0.70992800	-2.91734500
C	5.88569000	0.34649500	0.15790900
C	6.12118500	1.60657100	0.79568000
C	5.03399900	2.48974100	1.00593200
C	3.74570300	2.14743400	0.66038400
C	3.51978900	0.88716800	0.03423600
C	4.56047600	0.02090400	-0.28833300
C	6.98118500	-0.55071400	0.02939300
C	8.23946900	-0.20345500	0.46599200
C	8.47594800	1.05945200	1.05762500
C	7.43338100	1.94048800	1.22244700
C	-0.13297900	3.70164500	3.38261500
C	0.14574200	2.73382200	4.31032300
C	1.27006000	1.88148300	4.14503400
C	2.07191400	1.99893600	3.04372800
C	1.81318800	2.98503000	2.04343700
C	2.61936600	3.10073300	0.89363800
C	2.38221400	4.13272400	-0.03858900
C	3.17901700	4.28060300	-1.21866800
C	2.95108900	5.29472300	-2.11066400
C	1.89922800	6.22535900	-1.88535900
C	1.09165100	6.10189800	-0.78652100
C	1.29395600	5.06043100	0.17434900
C	0.43831800	4.89382800	1.28328600
C	0.69072900	3.87632700	2.22670000
C	-0.75623500	5.77349400	1.43821800
C	-2.03574500	5.27498000	1.15445700
C	-3.15755500	6.09349700	1.27410200
C	-3.01857800	7.42075300	1.68349800
C	-1.75001200	7.92410700	1.97531400
C	-0.62701500	7.10531400	1.85331800
C	-1.81045700	-4.67886500	-1.35874300
C	-1.88059400	-4.27054500	-2.66287700
C	-0.72863700	-3.74891700	-3.31195500
C	0.46482800	-3.67910800	-2.64640200
C	0.60471700	-4.15717800	-1.30480600
C	1.83161300	-4.09816900	-0.61016900
C	1.92662400	-4.60793300	0.70069600
C	3.17447200	-4.65652800	1.39529100
C	3.25784000	-5.12405300	2.67832200

C	2.09038000	-5.57428300	3.35332400
C	0.87417200	-5.54321200	2.72612700
C	0.74289700	-5.06333300	1.38426200
C	-0.50467300	-5.02680100	0.72710600
C	-0.57708800	-4.64765800	-0.63071400
C	-1.74932200	-5.28616900	1.50475300
C	-2.14246000	-4.35354300	2.47851900
C	-3.31776400	-4.53233300	3.20659800
C	-4.11659600	-5.65468300	2.97611700
C	-3.72843400	-6.59613000	2.02158800
C	-2.55252400	-6.41374300	1.29167600
O	2.64096700	-1.75087900	0.59106800
O	2.22160200	0.58086600	-0.31399300
P	1.40184600	-0.68193900	0.41630000
O	0.44433600	-1.16064500	-0.65455400
O	0.91175200	-0.35443800	1.78205000
H	3.46516100	-4.64777700	-2.79071200
H	5.11881100	-4.17334700	-4.51583500
H	6.74008400	-2.63484700	-5.58237500
H	7.17413400	-0.39696200	-4.57023200
H	6.05415600	0.26515500	-2.48719400
H	5.21962700	3.45459300	1.47004900
H	6.81343400	-1.52608300	-0.41036400
H	9.05794500	-0.91006600	0.36124500
H	9.47510900	1.32271600	1.39254600
H	7.59290800	2.90651300	1.69487000
H	-0.98299400	4.35987200	3.51808800
H	-0.49772300	2.61088900	5.17637300
H	1.47935200	1.11441700	4.88448100
H	2.90882000	1.32651400	2.90502000
H	3.97798900	3.56947900	-1.39390000
H	3.57281300	5.38795500	-2.99662900
H	1.72517400	7.02506300	-2.59990600
H	0.26926200	6.79085100	-0.63590700
H	-2.14196300	4.24337700	0.83891800
H	-4.14090000	5.69327100	1.04236500
H	-3.89309900	8.05887400	1.77603300
H	-1.63353200	8.95461400	2.29969500
H	0.36170600	7.49483900	2.07897100
H	-2.70006500	-5.03888600	-0.85561800
H	-2.82708800	-4.31024000	-3.19483700
H	-0.80288000	-3.38363200	-4.33227800
H	1.32912800	-3.24635900	-3.13408300
H	4.06644100	-4.31920200	0.87916000
H	4.21849800	-5.15519500	3.18439000
H	2.16777400	-5.95088200	4.36940900

H	-0.01204500	-5.89933100	3.23839900
H	-1.51375000	-3.48830900	2.66609000
H	-3.60304500	-3.79222700	3.94888800
H	-5.03281800	-5.79879400	3.54220800
H	-4.33945800	-7.47747900	1.84597600
H	-2.24946400	-7.14845700	0.55105000
H	-4.20655100	-0.02994600	0.80187900
C	4.95140600	-1.58213400	-2.26127400
C	-5.96900900	1.19334200	-1.01989100
H	-6.42052700	1.93533700	-0.36328300
C	-3.41584800	1.70528100	-0.83763200
C	-2.80137100	2.71867000	-1.67192200
C	-2.64829500	1.08007100	0.19793300
C	-3.52739200	3.43579300	-2.64577600
C	-1.40472900	2.95791500	-1.54624100
C	-1.28220600	1.43885700	0.36918100
C	-2.88473100	4.32629700	-3.48844000
H	-4.59712300	3.27385500	-2.73308900
C	-0.76717500	3.84963000	-2.43364300
C	-0.67996500	2.28944200	-0.51414800
H	-0.70085200	0.99522600	1.16701800
C	-1.49441000	4.52614400	-3.39481400
H	-3.45975300	4.87213000	-4.23157200
H	0.30272700	3.99668000	-2.34483700
H	0.38250700	2.47515900	-0.42149200
H	-0.99586700	5.21794400	-4.06690200
C	-6.78699100	0.21697700	-1.73512000
C	-6.01403500	-0.66321400	-2.51704900
C	-8.17635800	0.09246600	-1.69358400
C	-6.63335900	-1.66863400	-3.25154500
C	4.66436100	-2.84689200	-2.87334100
C	-8.79163900	-0.91758800	-2.43558900
H	-8.77011500	0.77287100	-1.08946100
C	-8.02661700	-1.79371400	-3.20875700
H	-6.03657100	-2.34890100	-3.85230100
C	-3.91668100	0.28995000	-3.56567800
C	-3.70131600	-1.30195400	-1.70548300
H	-9.87219400	-1.02294000	-2.40929100
H	-8.51425600	-2.57744500	-3.78075700
C	-2.55854700	0.37948900	-3.64651600
H	-4.55139700	0.82671600	-4.26308200
C	-2.34098400	-1.20129400	-1.76701500
H	-4.16922500	-2.00500000	-1.02310500
C	-1.74389000	-0.27851800	-2.67671900
H	-2.06554600	0.99220900	-4.39271400
H	-1.69257300	-1.79145200	-1.13366400

O	-0.46262300	-0.02947900	-2.68903700
H	0.01164100	-0.48122100	-1.87906600
N	-3.22480800	0.13592400	0.97762900
C	-2.50955300	-0.91822800	1.70641200
H	-2.96597000	-1.87186000	1.41373900
H	-1.47418900	-0.94552900	1.36591000
C	-2.56676100	-0.76397400	3.21107000
C	-1.39737300	-0.53320000	3.94177000
C	-3.78966900	-0.87179200	3.88607700
C	-1.45848900	-0.40943600	5.33221400
H	-0.45002900	-0.45374500	3.41711800
C	-3.84921000	-0.74216900	5.27211600
H	-4.70154600	-1.06286500	3.32288100
C	-2.67830500	-0.51043700	5.99961600
H	-0.54448500	-0.23521400	5.89352600
H	-4.80326500	-0.82685700	5.78556500
H	-2.72055500	-0.41444100	7.08117300

TS6-S5

C	-4.84887600	0.03822700	-1.41083200
C	-4.68638900	1.76159500	-0.35011800
C	3.47643100	-3.01892700	-0.70984500
C	2.20227500	-2.84331200	-0.17693500
C	1.19721800	-3.84960900	-0.23165500
C	1.51156300	-5.04740000	-0.84188600
C	3.02962000	-6.45865700	-2.18407900
C	4.20925800	-6.62409500	-2.87017600
C	5.16373900	-5.57961200	-2.88729600
C	4.93799400	-4.41028300	-2.19747000
C	5.80015000	-2.24902500	0.04975400
C	6.74487300	-1.17960700	0.18675400
C	6.33243100	0.15316400	-0.06710400
C	5.04567900	0.45166300	-0.46025900
C	4.18154300	-0.64096100	-0.77285100
C	4.50626400	-1.96249500	-0.50763200
C	6.18787800	-3.53925000	0.50122900
C	7.45413600	-3.77299600	0.98985500
C	8.40427500	-2.72863700	1.06235000
C	8.04822600	-1.45745400	0.67677600
C	3.64663000	4.27771800	2.37767400
C	4.29520200	3.72590900	3.44812700
C	4.93038000	2.46298000	3.31469700
C	4.95466500	1.82343800	2.10385800
C	4.37467200	2.40852600	0.93231100
C	4.46772200	1.81969000	-0.35257900
C	3.88679300	2.46163800	-1.46355000

C	4.04624400	1.94266800	-2.78528900
C	3.42109900	2.51392600	-3.85950700
C	2.58609300	3.64949900	-3.67532500
C	2.42415300	4.19470900	-2.42958200
C	3.07892000	3.64350800	-1.28063800
C	2.92992800	4.19405400	0.01028200
C	3.63810300	3.63864000	1.09737400
C	1.95447400	5.28879800	0.26561700
C	0.85307100	5.02280900	1.09601500
C	-0.11396000	5.99773900	1.32719500
C	0.00686200	7.25909000	0.74109800
C	1.10729100	7.54107900	-0.07021000
C	2.07501600	6.56338500	-0.30484600
C	-3.70720700	-3.82192100	-0.99879300
C	-3.48206600	-3.94362500	-2.34281600
C	-2.15755100	-3.90101300	-2.85365000
C	-1.09347700	-3.79375700	-1.99955400
C	-1.27573800	-3.71920300	-0.58059100
C	-0.17638600	-3.62787800	0.30468200
C	-0.40212600	-3.37558800	1.67592200
C	0.67753900	-3.24926000	2.60566000
C	0.45727000	-2.94333900	3.92095100
C	-0.86913500	-2.78825100	4.40712800
C	-1.93642800	-2.95335800	3.56558400
C	-1.75221600	-3.23992300	2.17437600
C	-2.84955500	-3.42320600	1.30411200
C	-2.62625800	-3.67683600	-0.06818800
C	-4.24808800	-3.29215800	1.80696400
C	-4.73735900	-2.05291400	2.24898700
C	-6.06646400	-1.89843300	2.63686200
C	-6.93367300	-2.99007100	2.60186000
C	-6.45645800	-4.23578500	2.19227200
C	-5.12645800	-4.38499400	1.79993100
O	1.87882200	-1.66500700	0.45681000
O	2.98076600	-0.36242700	-1.37952700
P	1.58630700	-0.33070600	-0.49637800
O	0.49660400	-0.68196000	-1.48652300
O	1.50281500	0.83797600	0.41976800
H	0.76567200	-5.83631500	-0.87971300
H	2.27412600	-7.24041500	-2.18293100
H	4.40406800	-7.54493800	-3.41233700
H	6.08150200	-5.69908800	-3.45608500
H	5.67208900	-3.61438500	-2.22656500
H	7.02580400	0.96270100	0.14361100
H	5.47087400	-4.34942100	0.46180200
H	7.72255300	-4.76966400	1.32878600

H	9.40248900	-2.92804800	1.44141000
H	8.75404900	-0.63478000	0.76062300
H	3.13446500	5.22645800	2.48334600
H	4.30473800	4.24021200	4.40523600
H	5.39873700	1.99924800	4.17884500
H	5.43124200	0.85567600	2.02600900
H	4.67623700	1.07071900	-2.92083800
H	3.55331900	2.09620300	-4.85324100
H	2.07874500	4.08875300	-4.53003000
H	1.79776800	5.06958100	-2.30038600
H	0.75808200	4.04299900	1.55048400
H	-0.96295000	5.76319200	1.96297600
H	-0.74891700	8.01959400	0.91760100
H	1.21520000	8.52459900	-0.51967300
H	2.93398200	6.78563300	-0.93167100
H	-4.72259700	-3.80114600	-0.62260500
H	-4.32109000	-4.03991700	-3.02689900
H	-1.98985700	-3.93806400	-3.92595200
H	-0.08794400	-3.72525900	-2.39303100
H	1.68944900	-3.38724300	2.24867600
H	1.29713200	-2.83062800	4.59989700
H	-1.03502800	-2.55963100	5.45600000
H	-2.94610900	-2.87621700	3.95114200
H	-4.06205000	-1.20318500	2.27705000
H	-6.42842200	-0.92390900	2.94933700
H	-7.97318700	-2.86984000	2.89158800
H	-7.12167300	-5.09464900	2.17449900
H	-4.75928100	-5.35282800	1.47147000
H	-3.49214000	0.90335200	1.52440700
C	3.74432100	-4.21823300	-1.45019000
C	-5.86899400	1.73725000	0.26010700
H	-6.15509200	2.53208700	0.94761500
C	-3.46723200	2.42443100	-0.50793500
C	-3.26344700	3.37233900	-1.58349400
C	-2.34426900	1.97854400	0.26015000
C	-4.33602800	3.90855700	-2.32741500
C	-1.93632400	3.76022700	-1.92157000
C	-1.03226100	2.37978600	-0.11352100
C	-4.10171700	4.78307300	-3.37354500
H	-5.35145000	3.62572800	-2.06812800
C	-1.72316000	4.65010100	-2.99623800
C	-0.84794500	3.21196200	-1.17992800
H	-0.16729400	1.97763700	0.39942400
C	-2.78769500	5.15612200	-3.71747700
H	-4.94058100	5.18719000	-3.93363500
H	-0.70159200	4.92437000	-3.24433600

H	0.16394900	3.46654700	-1.47384300
H	-2.61540600	5.84090200	-4.54235900
C	-6.76123900	0.61085800	-0.00485100
C	-6.20844400	-0.31643000	-0.90885800
C	-8.01491100	0.38210900	0.56474600
C	-6.90048800	-1.48117600	-1.22199800
C	2.75784400	-5.25570400	-1.47908000
C	-8.70746300	-0.78532200	0.23923000
H	-8.44082400	1.10068200	1.25983100
C	-8.15236300	-1.71456900	-0.64322500
H	-6.46138800	-2.20368000	-1.90341500
C	-4.67577300	0.60902900	-2.72681300
C	-3.72667000	-0.67706300	-0.86432400
H	-9.68111900	-0.97491500	0.68153100
H	-8.69262300	-2.62628500	-0.87934900
C	-3.42521500	0.76050000	-3.25275600
H	-5.54535000	1.00858100	-3.23866300
C	-2.47436600	-0.53698100	-1.38648100
H	-3.88734600	-1.27250200	0.02099400
C	-2.28671200	0.26495000	-2.54577600
H	-3.25493800	1.28587600	-4.18571100
H	-1.60857700	-1.00499600	-0.93775000
O	-1.09952700	0.54913400	-3.01778300
H	-0.36672500	0.12876700	-2.42445700
N	-2.53705700	1.15709900	1.31776200
C	-1.46831100	0.50947300	2.06903200
H	-1.93461100	-0.27547100	2.67349800
H	-0.78409000	0.00677400	1.37803900
C	-0.67530100	1.45429500	2.95215400
C	0.63994900	1.11854200	3.28800400
C	-1.22294000	2.64701100	3.43105100
C	1.39611400	1.96836700	4.09082600
H	1.08295300	0.21814100	2.87749200
C	-0.46312500	3.50072400	4.23301000
H	-2.23831100	2.91809600	3.15605500
C	0.84984100	3.16356700	4.56179200
H	2.42687300	1.71554400	4.31259600
H	-0.89378000	4.43256000	4.59010900
H	1.45224500	3.83643200	5.16521300

TS6-S6

C	-1.87366500	2.66635200	-1.14667300
C	-2.62253100	0.86952500	-1.18956800
C	3.67750300	-2.74130600	-0.25911300
C	2.31431000	-2.91153600	-0.04835900
C	1.55214100	-3.92289000	-0.69466600

C	2.22129800	-4.83916900	-1.47743600
C	4.28022800	-5.65864300	-2.56625500
C	5.60928700	-5.49990500	-2.88023600
C	6.32464400	-4.39502200	-2.36196700
C	5.71255300	-3.49243200	-1.52208100
C	5.52397800	-1.62831100	1.15381800
C	6.12096600	-0.41473400	1.63036400
C	5.51286500	0.83266500	1.33280900
C	4.34151800	0.90767700	0.61258100
C	3.78718200	-0.30333200	0.11328500
C	4.35074900	-1.55138200	0.33413600
C	6.10048800	-2.86311300	1.55536300
C	7.22968700	-2.89730300	2.34206500
C	7.84282200	-1.69827700	2.77456000
C	7.29305400	-0.48596900	2.42846300
C	1.52117100	4.65251900	2.30095400
C	1.58831200	4.12179100	3.56103100
C	2.30418700	2.91476300	3.79445200
C	2.94873600	2.28632600	2.76502200
C	2.93257400	2.81949600	1.43741500
C	3.61057700	2.18670400	0.37551200
C	3.53645900	2.71858200	-0.92773900
C	4.21838600	2.10219300	-2.02288800
C	4.11713500	2.59435100	-3.29515800
C	3.31828500	3.74016800	-3.55409500
C	2.65949700	4.36992300	-2.53329300
C	2.74928900	3.90137600	-1.18388000
C	2.07432200	4.54150500	-0.11950000
C	2.16745800	4.02033900	1.18960800
C	1.25926500	5.76131500	-0.38507200
C	-0.13238600	5.74579400	-0.20746800
C	-0.90315800	6.87447000	-0.48286300
C	-0.29394900	8.04540500	-0.93699100
C	1.09140500	8.07878300	-1.10533300
C	1.86008100	6.94762900	-0.83068200
C	-2.77797900	-3.17779400	-2.99939900
C	-2.09183500	-2.77402700	-4.11444200
C	-0.67448600	-2.66101300	-4.08148300
C	0.01745900	-3.00346000	-2.95206800
C	-0.65246000	-3.48412800	-1.78216600
C	0.06010900	-3.90390400	-0.63858000
C	-0.63739800	-4.29182800	0.52063000
C	0.05567000	-4.69264600	1.70533700
C	-0.61931700	-5.04208000	2.84357400
C	-2.04098700	-5.01500500	2.86712900
C	-2.74601300	-4.64523800	1.75191000

C	-2.08247200	-4.28159000	0.53540000
C	-2.79814700	-3.90468300	-0.62267500
C	-2.09630700	-3.51680100	-1.78536700
C	-4.28866300	-3.91489300	-0.62081600
C	-5.01094500	-2.72256800	-0.77340000
C	-6.40497200	-2.72402700	-0.76996700
C	-7.10176600	-3.92313700	-0.61432200
C	-6.39556200	-5.11863800	-0.46698200
C	-5.00109600	-5.11428600	-0.47165600
O	1.65438100	-2.06435900	0.80895100
O	2.61541500	-0.21355400	-0.60758900
P	1.24144400	-0.54002700	0.26444600
O	0.11203700	-0.61911900	-0.72036100
O	1.14334800	0.31473800	1.48543700
H	1.65987700	-5.62489000	-1.97576900
H	3.70834200	-6.48594000	-2.97917600
H	6.10645000	-6.20805700	-3.53690900
H	7.36661100	-4.25304100	-2.63481600
H	6.27100100	-2.64594800	-1.14240000
H	5.96481700	1.74297700	1.71757100
H	5.63265100	-3.78716600	1.23676900
H	7.65027000	-3.85412900	2.63840900
H	8.73698600	-1.73859400	3.38998300
H	7.74086900	0.44299500	2.77269000
H	0.97829900	5.57438900	2.13051900
H	1.09598100	4.62702800	4.38811100
H	2.33559000	2.48782400	4.79289900
H	3.46595200	1.35222800	2.93819000
H	4.82197300	1.22377800	-1.82666300
H	4.64060900	2.10487600	-4.11139200
H	3.22522900	4.11313400	-4.57025400
H	2.04590200	5.23726000	-2.74196700
H	-0.60593900	4.84134200	0.15231100
H	-1.97988500	6.83319000	-0.34237400
H	-0.89218700	8.92611300	-1.15309500
H	1.57630700	8.98823900	-1.44942400
H	2.93768100	6.97514600	-0.96309200
H	-3.85808900	-3.25584600	-3.02715900
H	-2.63271300	-2.53205400	-5.02550800
H	-0.13938100	-2.30401300	-4.95698400
H	1.09436300	-2.90059400	-2.92536800
H	1.13937600	-4.70459900	1.68700800
H	-0.07057000	-5.33476500	3.73394600
H	-2.56893000	-5.28345500	3.77818400
H	-3.82878400	-4.61425400	1.78044400
H	-4.47272400	-1.79014000	-0.89351400

H	-6.94031400	-1.78578300	-0.88262700
H	-8.18833700	-3.92774800	-0.61083700
H	-6.93059600	-6.05755700	-0.35310000
H	-4.45183500	-6.04497600	-0.36211500
H	-1.29133600	-0.55596600	0.46092800
C	4.35078100	-3.64732500	-1.14380600
C	-2.12132000	0.44202300	-2.34520400
H	-2.32990400	-0.56416100	-2.69874300
C	-3.56910100	0.51628400	-0.20454000
C	-4.94075800	0.92141900	-0.43860200
C	-3.23377800	-0.20603800	0.98508900
C	-5.33848200	1.58676700	-1.62263600
C	-5.94430800	0.63881000	0.53172000
C	-4.28601900	-0.48544200	1.92234900
C	-6.65426700	1.96637200	-1.82166100
H	-4.60128700	1.77805900	-2.39319500
C	-7.28008700	1.03401700	0.30682200
C	-5.56439300	-0.07415000	1.70650300
H	-4.05611500	-1.05009700	2.81001400
C	-7.63824800	1.69518800	-0.85290100
H	-6.93021900	2.47149700	-2.74319200
H	-8.02525000	0.80246500	1.06397800
H	-6.33278900	-0.31264700	2.43764100
H	-8.66822500	1.99498700	-1.02031600
C	-1.36389500	1.39719300	-3.14005800
C	-1.32347000	2.66921000	-2.53546700
C	-0.77954900	1.19104500	-4.38843200
C	-0.71630300	3.73888000	-3.17915400
C	3.61497700	-4.73841300	-1.71337400
C	-0.17345600	2.26961500	-5.03656100
H	-0.79980700	0.20520700	-4.84164500
C	-0.15137700	3.53331100	-4.44356100
H	-0.66704300	4.71182200	-2.70105700
C	-2.95731600	3.50968500	-0.72190600
C	-0.92048200	2.26948400	-0.13333600
H	0.28537600	2.12320000	-6.01010200
H	0.32232400	4.36353500	-4.95841600
C	-3.26152600	3.61239500	0.61235000
H	-3.59024000	3.97400700	-1.46925600
C	-1.18825900	2.43248000	1.19880900
H	-0.01402600	1.77702900	-0.45266100
C	-2.41526200	3.00565300	1.57928300
H	-4.14291800	4.14049600	0.95915000
H	-0.47142100	2.08530900	1.92676500
O	-2.80126700	3.06128900	2.85954800
H	-2.18521300	2.54098400	3.41610300

N	-1.98815800	-0.64527700	1.21563600
C	-1.55719000	-1.36920500	2.41785800
H	-2.16676100	-2.26877200	2.54108700
H	-0.53865300	-1.70026000	2.21725900
C	-1.59455700	-0.54401700	3.69645100
C	-2.52193600	-0.82691800	4.70411600
C	-0.69427900	0.51354500	3.89485700
C	-2.61561300	-0.02845200	5.84625700
H	-3.18066700	-1.68642000	4.59933300
C	-0.79302900	1.32260200	5.02964200
H	0.10080900	0.66000300	3.17056700
C	-1.76761200	1.06663200	6.00084400
H	-3.35301800	-0.26125200	6.60936500
H	-0.08687200	2.13711000	5.16368400
H	-1.84412700	1.69892600	6.88100900

Int7

C	-3.10118100	-1.19973900	-0.44061400
N	-3.10598300	0.55887300	2.13007000
C	-2.00312800	-0.72565800	-1.05011800
H	-3.01053200	-1.88409800	0.39589600
C	-5.21146200	-0.06487300	0.29097300
C	-5.35471700	-2.12696600	-0.92704500
C	-4.55689200	-0.07200200	-2.13380400
C	-2.11367000	0.11362600	-2.24016300
H	-1.01290500	-0.98301500	-0.69629500
C	-6.20706900	-0.82101600	0.80106500
C	-4.82522500	1.32205600	0.60959400
C	-6.33488600	-2.08903200	0.08376100
C	-5.26764700	-3.19553100	-1.80401100
C	-3.45955800	0.39780000	-2.74674300
H	-5.54598600	0.14326100	-2.52537200
O	-1.12208400	0.55347700	-2.85386600
H	-6.85684600	-0.50699400	1.61142900
C	-5.52207200	2.40685300	-0.00993400
C	-3.72217000	1.57732100	1.43261000
C	-7.23850800	-3.14229600	0.22313100
C	-6.18049800	-4.24860100	-1.66685000
H	-4.51726700	-3.20732000	-2.58823200
H	-3.51559100	0.99440400	-3.65165400
H	0.31039500	0.19826400	-2.41798900
C	-6.67399000	2.21779200	-0.82300900
C	-5.04944700	3.74696900	0.17485600
C	-4.50184500	-0.86353400	-0.86381600
C	-3.25184400	2.91486900	1.58213000
C	-7.15262000	-4.22007200	-0.66321600

H	-8.00164200	-3.12135400	0.99622600
H	-6.13088600	-5.09403600	-2.34632600
C	-7.31007900	3.28421100	-1.42110100
H	-7.06728400	1.21519100	-0.95192300
C	-5.73058000	4.82228000	-0.44655300
C	-3.89413300	3.95752100	0.97284000
H	-3.43671800	-0.36650000	1.89438800
C	-1.67941400	0.61337900	2.45687600
H	-2.36832600	3.09985000	2.17797200
H	-7.85227600	-5.04611400	-0.57153200
C	-6.83784100	4.60313800	-1.23641600
H	-8.19038500	3.10893700	-2.03396300
H	-5.35519500	5.82868000	-0.28446000
H	-3.51643100	4.96877200	1.08910300
H	-1.07328400	0.88841900	1.58648900
H	-1.52248100	1.39505300	3.21037000
C	-1.21062800	-0.70714100	3.01749600
H	-7.35157100	5.43576200	-1.70819600
C	-1.97010200	-1.39520600	3.97401600
C	0.01896200	-1.23963700	2.61122100
C	-1.50229100	-2.59056700	4.52146000
H	-2.92361100	-0.98286100	4.29383700
C	0.48837900	-2.43140500	3.16378400
H	0.60035300	-0.71392300	1.86239500
C	-0.26933700	-3.10850500	4.12037800
H	-2.09941400	-3.11591400	5.26229100
H	1.43831000	-2.83731500	2.83275200
H	0.09313900	-4.04351700	4.53565400
H	-0.78116100	3.74538500	4.31821100
C	-0.02064200	3.68158800	3.54453600
C	-0.13026100	4.44471600	2.41300800
C	1.07465900	2.78835200	3.70342100
C	0.86090100	4.39745500	1.38192000
H	-0.97824800	5.10695800	2.28432300
C	2.04781300	2.71550800	2.74495200
H	1.13077400	2.15473200	4.58373500
C	2.00306300	3.53298700	1.57080200
C	0.72968600	5.13834900	0.18946600
H	2.87182700	2.02269000	2.86429400
C	3.00670600	3.47993900	0.58283800
C	1.75451000	5.10859400	-0.78073500
C	-0.51743100	5.90514700	-0.09259500
C	4.10596600	2.48359700	0.71144400
C	2.91296900	4.27552100	-0.57489700
C	1.68981300	5.89619300	-1.97396100
C	-1.41144100	5.42943100	-1.06356200

C	-0.81952400	7.09876900	0.57599400
C	4.04851500	1.30680300	-0.08049800
C	5.13797700	2.59543600	1.61561600
C	3.93543100	4.26778500	-1.57342000
C	2.69230200	5.85917700	-2.90560000
H	0.82909600	6.53651500	-2.12659100
C	-2.57133800	6.13837600	-1.37020800
H	-1.18976200	4.49548300	-1.57103800
C	-1.98596600	7.80453300	0.27718900
H	-0.13047000	7.47435000	1.32717600
C	4.92956400	0.24916200	0.03981900
O	3.04690500	1.23759300	-1.03913800
C	6.07396700	1.54599900	1.80583200
H	5.21441300	3.48801700	2.23019600
C	3.83042100	5.03117400	-2.70421200
H	4.80810400	3.64348800	-1.41597400
H	2.62305600	6.46923100	-3.80178100
C	-2.86128000	7.32903700	-0.70080600
H	-3.25944400	5.74999000	-2.11484100
H	-2.20519800	8.73047600	0.80173700
C	5.95457000	0.33751200	1.04293500
C	4.71727800	-0.97701100	-0.77654100
P	1.72839400	0.33642800	-0.73939300
C	7.09835000	1.65266200	2.78308800
H	4.61970800	5.00979700	-3.45023500
H	-3.76505100	7.88315500	-0.93949700
C	6.83400200	-0.73580900	1.34744800
C	3.48013400	-1.60064500	-0.75638900
C	5.74919100	-1.55885400	-1.58672200
O	2.41215500	-1.00673700	-0.09128800
O	0.71301200	0.87914600	0.18610000
O	1.28967800	-0.01513900	-2.20092300
C	7.95470700	0.60531800	3.02893200
H	7.18159800	2.57962800	3.34455600
C	7.80821400	-0.60409200	2.31138200
H	6.72895200	-1.67198400	0.81373800
C	3.23914600	-2.87460000	-1.33926600
C	5.50832600	-2.81678800	-2.22877400
C	6.99436300	-0.91422000	-1.81261200
H	8.73157400	0.69781400	3.78222700
H	8.46685800	-1.44061300	2.52662600
C	4.25768700	-3.46446800	-2.05183800
C	1.94476800	-3.55435500	-1.04197600
C	6.53053000	-3.39555100	-3.02537000
C	7.95985800	-1.49611500	-2.60270300
H	7.17324800	0.05434500	-1.36082500

H	4.10322900	-4.44651500	-2.49018300
C	0.83718000	-3.42830300	-1.89975200
C	1.83080300	-4.26602300	0.16972200
C	7.73342300	-2.75405100	-3.20804800
H	6.33745400	-4.35526200	-3.49775300
H	8.90205800	-0.98149300	-2.76757800
C	0.93982900	-2.77632000	-3.16826400
C	-0.45158400	-3.93491800	-1.48492300
C	2.95352300	-4.47719900	1.03172000
C	0.55082900	-4.79590900	0.57053500
H	8.50570700	-3.20443100	-3.82471300
C	-0.15103600	-2.62635200	-3.98030100
H	1.90308400	-2.38287500	-3.46843400
C	-1.56574600	-3.74004700	-2.36065700
C	-0.58832000	-4.56333100	-0.22866600
C	2.83776300	-5.20916200	2.18391500
H	3.91303400	-4.06133900	0.74698700
C	0.47760800	-5.55728600	1.77880800
C	-1.42157800	-3.11550200	-3.56962700
H	-0.05195500	-2.11790300	-4.93444200
H	-2.53748100	-4.09333700	-2.04108200
C	-1.93895100	-4.89136200	0.30761500
C	1.58541800	-5.77101400	2.55439000
H	3.70741200	-5.36835600	2.81514500
H	-0.47978400	-5.97434400	2.06807400
H	-2.28386400	-2.97699200	-4.21661400
C	-2.42864800	-4.14086600	1.38812100
C	-2.74697800	-5.89703400	-0.23718600
H	1.50903300	-6.36637600	3.45986400
C	-3.70707100	-4.37227500	1.89266900
H	-1.80004400	-3.37376300	1.82679200
C	-4.02423200	-6.13162900	0.27274700
H	-2.37061500	-6.49138800	-1.06503200
C	-4.51035700	-5.36651300	1.33334800
H	-4.06914700	-3.77173800	2.72234200
H	-4.64403300	-6.90946800	-0.16425200
H	-5.51216200	-5.53807700	1.71388700

TS8

C	-3.02628400	-1.01983500	-0.12453800
N	-3.06168200	0.18636000	1.23650500
C	-1.90973100	-0.75050700	-0.98166400
H	-2.89462500	-1.87642500	0.53273600
C	-5.42640400	-0.13741600	0.18239600
C	-5.14531400	-2.31574200	-0.73361600
C	-4.51915700	-0.34295600	-2.08560100

C	-2.08660800	-0.16655500	-2.21216300
H	-0.91538000	-0.99534500	-0.63590100
C	-6.47863900	-0.89975600	0.55368500
C	-5.03484300	1.24889500	0.42188600
C	-6.34385900	-2.25160700	0.00681600
C	-4.77447600	-3.46761900	-1.40448100
C	-3.43165800	0.04892100	-2.75267200
H	-5.51699900	-0.21466300	-2.49438500
O	-1.12666500	0.18029800	-3.04214500
H	-7.30460800	-0.56764900	1.17420600
C	-5.80508700	2.39890400	0.07772400
C	-3.72268900	1.41160600	0.84553800
C	-7.17312000	-3.36988800	0.09631800
C	-5.60794500	-4.58857900	-1.31295400
H	-3.85671500	-3.49417000	-1.98003600
H	-3.49204800	0.50288000	-3.73649600
H	-0.19153400	0.07360900	-2.63151900
C	-7.14160900	2.31806600	-0.39283700
C	-5.18865400	3.68937800	0.18961000
C	-4.45462700	-0.95972700	-0.70585700
C	-3.09157600	2.66769600	0.89557000
C	-6.79065200	-4.53864500	-0.56912700
H	-8.10073000	-3.33285700	0.66092900
H	-5.32334500	-5.50871600	-1.81210000
C	-7.84588500	3.45902400	-0.70387700
H	-7.59886800	1.34168500	-0.51126000
C	-5.94676300	4.84528100	-0.13487800
C	-3.83049600	3.78668800	0.58614900
H	-3.69101300	-0.31275000	1.86801300
C	-1.73461000	0.33484700	1.90153900
H	-2.05128500	2.76114500	1.17685700
H	-7.42310700	-5.41977100	-0.50926000
C	-7.24778200	4.73505600	-0.56719600
H	-8.86806500	3.38149500	-1.06311800
H	-5.46749900	5.81569400	-0.04563800
H	-3.36798800	4.76571600	0.63123700
H	-1.00002200	0.64570600	1.15499700
H	-1.83400000	1.14229300	2.63494400
C	-1.30789000	-0.93825400	2.58619000
H	-7.81799300	5.62532800	-0.81604900
C	-2.13261900	-1.56956300	3.52855100
C	-0.03409600	-1.45858300	2.33343900
C	-1.69619000	-2.71251100	4.19802100
H	-3.11344100	-1.15843100	3.76820800
C	0.40799500	-2.59090800	3.01709300
H	0.60740100	-0.96072900	1.61611700

C	-0.42127400	-3.22267800	3.94343500
H	-2.34503700	-3.19695200	4.92234900
H	1.39815500	-2.98151100	2.81382000
H	-0.07755800	-4.11570200	4.45579100
H	-1.24051500	3.54279800	4.12450500
C	-0.43196300	3.56099800	3.39791100
C	-0.50990000	4.39593500	2.31416600
C	0.69317100	2.70957300	3.57066700
C	0.53633800	4.45722800	1.33809300
H	-1.38119900	5.02674000	2.18603900
C	1.71691200	2.73781000	2.66427900
H	0.73108400	2.02609200	4.41395300
C	1.69558400	3.61798700	1.53656400
C	0.44547200	5.26895500	0.18534400
H	2.56102000	2.06981500	2.77971000
C	2.74527500	3.63994300	0.59750600
C	1.51710500	5.31429600	-0.73448000
C	-0.80127100	6.02991600	-0.10991400
C	3.89296700	2.70346800	0.75923400
C	2.67921400	4.48621000	-0.52434100
C	1.50485700	6.17950400	-1.87510300
C	-1.57159500	5.68838100	-1.23363000
C	-1.23794300	7.08606100	0.70293400
C	3.93269600	1.52190200	-0.03057800
C	4.88629200	2.89880300	1.69191000
C	3.74266400	4.54074400	-1.47790400
C	2.54862200	6.20578900	-2.76071300
H	0.65289300	6.83177000	-2.02545000
C	-2.73857800	6.38650200	-1.54012300
H	-1.24702200	4.86402200	-1.86110100
C	-2.41212800	7.77876700	0.40463700
H	-0.64562200	7.36587900	1.56920800
C	4.89000100	0.53437300	0.13485700
O	2.97795400	1.37549400	-1.00974900
C	5.89240400	1.92461200	1.91678800
H	4.87967000	3.79968600	2.29965800
C	3.68078300	5.36850600	-2.56524300
H	4.60944700	3.90924500	-1.31969900
H	2.51606000	6.87491900	-3.61605000
C	-3.16403500	7.43415300	-0.72039900
H	-3.32296400	6.10271400	-2.41070500
H	-2.73258300	8.59601200	1.04493300
C	5.87981100	0.70641900	1.16045300
C	4.78222400	-0.71704700	-0.66283200
P	1.68632100	0.37699500	-0.73374100
C	6.88159500	2.11343900	2.91748400

H	4.49936600	5.39307900	-3.27874800
H	-4.07150900	7.98197800	-0.95975400
C	6.82998600	-0.29622500	1.49547900
C	3.58559500	-1.42004900	-0.64001700
C	5.86159600	-1.23451700	-1.45159600
O	2.49243400	-0.89077300	0.00983500
O	0.74829800	0.92000600	0.29037300
O	1.22267600	-0.04961500	-2.10150800
C	7.80757100	1.13473600	3.19256800
H	6.88226200	3.04819900	3.47268000
C	7.76855300	-0.08650500	2.48063000
H	6.80768700	-1.24039900	0.96587300
C	3.44393700	-2.71099700	-1.22131200
C	5.71566100	-2.51195800	-2.08380800
C	7.06519100	-0.51105900	-1.66711300
H	8.55734100	1.28977400	3.96309900
H	8.48292400	-0.87017400	2.71700800
C	4.50751300	-3.23768300	-1.91800800
C	2.19448800	-3.48435900	-0.96040000
C	6.78570200	-3.02835200	-2.86021800
C	8.07950500	-1.03470900	-2.43645100
H	7.17166700	0.47164300	-1.22264700
H	4.42211600	-4.22895000	-2.35514200
C	1.11307600	-3.45227500	-1.85955700
C	2.10281900	-4.22631500	0.23552700
C	7.94638800	-2.31055200	-3.03252800
H	6.66349700	-4.00322500	-3.32567900
H	8.98816200	-0.46010100	-2.59255200
C	1.19049100	-2.75242000	-3.10383500
C	-0.12343100	-4.11715500	-1.51739500
C	3.20140400	-4.32867600	1.14714200
C	0.87150300	-4.89885600	0.57108600
H	8.75600700	-2.71440000	-3.63363600
C	0.12698300	-2.71370700	-3.96309000
H	2.10385600	-2.22282300	-3.33992700
C	-1.20566900	-4.05443300	-2.44988700
C	-0.24359500	-4.78692900	-0.28403500
C	3.11156600	-5.07863400	2.29041600
H	4.12141700	-3.80734400	0.90965900
C	0.82290300	-5.67331700	1.77292700
C	-1.08580700	-3.37912700	-3.63411200
H	0.20028400	-2.15720500	-4.89194300
H	-2.12249900	-4.57563400	-2.20459800
C	-1.56738500	-5.29853900	0.17446600
C	1.90909300	-5.77211200	2.60094100
H	3.96307800	-5.14945000	2.96127300

H	-0.09725700	-6.19244300	2.01526300
H	-1.92204400	-3.34500400	-4.32728700
C	-2.26232200	-4.57290200	1.15415600
C	-2.13440200	-6.47978700	-0.31834300
H	1.85400300	-6.37794000	3.50128100
C	-3.49357900	-5.01698300	1.63076500
H	-1.81487400	-3.66864300	1.55049100
C	-3.36701700	-6.92741700	0.16186000
H	-1.59941600	-7.05124400	-1.07162700
C	-4.04906500	-6.19787600	1.13670800
H	-4.01665400	-4.43808300	2.38673800
H	-3.79223100	-7.85015900	-0.22409400
H	-5.01143600	-6.54319000	1.50276000

TS8-S1

C	-2.93766200	-0.67703200	-0.70922000
N	-2.43543600	-0.05080900	0.95996000
C	-1.85842500	-0.46145600	-1.61415800
H	-3.04396100	-1.69887300	-0.35788300
C	-4.89132200	0.62791900	0.28791300
C	-5.35890600	-1.04861700	-1.33686000
C	-4.23312200	1.05644600	-2.04980600
C	-1.88727400	0.57599900	-2.51191100
H	-0.97719900	-1.08213700	-1.55213200
C	-6.09712100	0.08358900	0.56335600
C	-4.08557900	1.67357300	0.91183200
C	-6.42188800	-0.95561700	-0.41535100
C	-5.37907800	-1.97118500	-2.36901100
C	-3.11408700	1.34766600	-2.72247600
H	-5.15720600	1.60078500	-2.21994600
O	-0.87756800	0.91409300	-3.28959900
H	-6.72949500	0.35705600	1.40165500
C	-4.52215900	3.02082600	1.08978800
C	-2.75315600	1.34718400	1.14241500
C	-7.52386800	-1.80214500	-0.52830100
C	-6.48062600	-2.83206600	-2.46955400
H	-4.55351100	-2.03122600	-3.07088200
H	-3.07813400	2.13350200	-3.47007900
H	-0.03282400	0.41785500	-2.99390300
C	-5.84811800	3.44725400	0.81394100
C	-3.56280900	3.99216400	1.52129400
C	-4.29169600	-0.01876800	-0.98624200
C	-1.79457700	2.30940600	1.51531600
C	-7.53931900	-2.74430200	-1.56092100
H	-8.34994800	-1.73872100	0.17462600
H	-6.51178700	-3.57384100	-3.26208100

C	-6.21164600	4.76391200	0.98451000
H	-6.57089000	2.72288600	0.45507100
C	-3.97239100	5.34020600	1.68878700
C	-2.21533200	3.60298700	1.71447000
H	-3.07605200	-0.59662100	1.53715700
C	-1.03721700	-0.49209900	1.19196300
H	-0.74988200	2.05306200	1.62728700
H	-8.38490200	-3.41909200	-1.65747600
C	-5.26853700	5.72038600	1.43149900
H	-7.22943100	5.07510300	0.76689200
H	-3.23089900	6.07054400	1.99939300
H	-1.49132300	4.35252400	2.00479900
H	-0.98924200	-1.52216100	0.83527300
H	-0.39925700	0.11540500	0.55110900
C	-0.57354100	-0.42061200	2.62561200
H	-5.56937400	6.75647600	1.55583900
C	0.71993700	0.04942400	2.88291000
C	-1.35986600	-0.87543500	3.68758000
C	1.21842700	0.04865000	4.18434700
H	1.32335100	0.41450200	2.05903400
C	-0.86508000	-0.86592600	4.99044900
H	-2.36254700	-1.25896500	3.50851300
C	0.42985200	-0.40914200	5.24082900
H	2.22464900	0.41278500	4.36933400
H	-1.48608000	-1.22396900	5.80692700
H	0.81860600	-0.40629100	6.25549500
H	0.69019300	4.69702300	3.84712400
C	1.29690700	4.34390600	3.01768400
C	1.04564800	4.77772800	1.74401100
C	2.35048400	3.42060600	3.25408600
C	1.82780000	4.33468200	0.63237900
H	0.24642900	5.48427000	1.55319100
C	3.14677200	2.99937200	2.22254800
H	2.52454200	3.04958200	4.25989200
C	2.95015900	3.46281000	0.88199700
C	1.48076100	4.67395800	-0.68955700
H	3.94395500	2.29384300	2.41925400
C	3.75077800	3.03780600	-0.20239600
C	2.32761200	4.31940200	-1.75536700
C	0.12856900	5.24077100	-0.96503600
C	4.68134200	1.89880100	0.01544600
C	3.49778300	3.51851700	-1.50114800
C	2.04608700	4.70955200	-3.10199300
C	-0.89116400	4.34189700	-1.31692300
C	-0.17510200	6.60068000	-0.83828500
C	4.28795900	0.62736000	-0.49844000

C	5.78088400	1.94652900	0.84217000
C	4.34080500	3.17667300	-2.60186400
C	2.88066500	4.35883700	-4.12877900
H	1.15771300	5.30148300	-3.29261200
C	-2.19159300	4.79780000	-1.52244300
H	-0.64818700	3.28680800	-1.40200500
C	-1.47970200	7.05572600	-1.04558100
H	0.61282100	7.29902500	-0.56922100
C	4.85653300	-0.56430200	-0.08459100
O	3.30574100	0.59481300	-1.45921800
C	6.43049300	0.76419700	1.28291300
H	6.11476900	2.90446200	1.23184700
C	4.04770600	3.58591900	-3.87425200
H	5.22466500	2.57839000	-2.40694200
H	2.65443200	4.67098700	-5.14448200
C	-2.49053200	6.15471500	-1.38302300
H	-2.97684600	4.08740800	-1.76315900
H	-1.70485500	8.11388800	-0.94102700
C	5.92570700	-0.51461000	0.87535400
C	4.26367200	-1.84710300	-0.54848900
P	1.74885800	0.20991100	-1.07744200
C	7.51614800	0.82647300	2.19543900
H	4.70277900	3.31482100	-4.69731200
H	-3.50867300	6.50510600	-1.52633400
C	6.49056400	-1.67363100	1.47223600
C	2.89469300	-2.05370500	-0.41134300
C	5.05442100	-2.89452800	-1.12780800
O	2.06827600	-1.03369700	0.00694500
O	1.04413700	1.26438100	-0.28726900
O	1.16261800	-0.35581500	-2.35000000
C	8.06592300	-0.31857000	2.72320900
H	7.89306100	1.80499800	2.48264400
C	7.53274400	-1.57852500	2.36770900
H	6.08975100	-2.64660700	1.21765200
C	2.29095500	-3.31946700	-0.64182600
C	4.44788200	-4.16230200	-1.40210700
C	6.41781200	-2.71254600	-1.48518400
H	8.89351100	-0.25737900	3.42412000
H	7.94501300	-2.48169700	2.80885900
C	3.07374000	-4.35054800	-1.11160600
C	0.85067100	-3.54509300	-0.32299800
C	5.23232200	-5.20317900	-1.96493200
C	7.14755800	-3.73665300	-2.04460500
H	6.87732500	-1.74506700	-1.32172900
H	2.62602500	-5.32594800	-1.28177500
C	-0.10305600	-3.61226000	-1.35652200

C	0.45971700	-3.73236000	1.01932100
C	6.55611400	-5.00064200	-2.27705100
H	4.75838000	-6.16279200	-2.15576100
H	8.18604400	-3.57053200	-2.31666300
C	0.25691400	-3.34156100	-2.71522300
C	-1.48523700	-3.90292700	-1.04576600
C	1.39150500	-3.59663400	2.09588600
C	-0.91337900	-4.05596600	1.33240100
H	7.14518900	-5.80210300	-2.71349500
C	-0.67891500	-3.35612800	-3.71239400
H	1.28226100	-3.07325300	-2.93201500
C	-2.42812700	-3.90012900	-2.12384700
C	-1.87309300	-4.12939900	0.29627800
C	1.01044800	-3.78634500	3.39603600
H	2.41443000	-3.32601800	1.86344700
C	-1.25190600	-4.29892100	2.70173400
C	-2.03781500	-3.64118700	-3.41045600
H	-0.38867800	-3.12414500	-4.73242700
H	-3.47214700	-4.08506700	-1.90563600
C	-3.30676100	-4.38039600	0.61497100
C	-0.32475300	-4.16285000	3.69960600
H	1.72831100	-3.65100500	4.19881800
H	-2.26578600	-4.59571700	2.94237600
H	-2.77657200	-3.63863900	-4.20785500
C	-4.03213100	-3.47423500	1.40424500
C	-3.98397800	-5.49630500	0.10254400
H	-0.61128400	-4.33794900	4.73241600
C	-5.39168700	-3.65261900	1.64727900
H	-3.51186500	-2.62637900	1.84007100
C	-5.34418200	-5.68158600	0.34652900
H	-3.43476500	-6.21441900	-0.49875900
C	-6.05499700	-4.75614600	1.11127700
H	-5.93307400	-2.92472800	2.24417300
H	-5.85028600	-6.55053900	-0.06458800
H	-7.11746500	-4.89321800	1.28821300

TS8-S2

C	-2.97003400	-0.96057000	-0.04280700
N	-3.06747200	0.08217500	1.41195500
C	-1.85354700	-0.57038300	-0.85961700
H	-2.81377700	-1.88449200	0.50955100
C	-5.42650600	-0.34729000	0.35252200
C	-4.94936100	-2.29157800	-0.93039500
C	-4.47014600	-0.05450000	-1.89755500
C	-2.04112800	0.18598500	-1.98764900
H	-0.85566400	-0.85429900	-0.55813100

C	-6.39307600	-1.26234300	0.58541800
C	-5.17389100	1.02373800	0.78623400
C	-6.13438400	-2.47843000	-0.18966200
C	-4.47760400	-3.26393100	-1.79436000
C	-3.39191300	0.47555400	-2.48046100
H	-5.47006200	0.11474700	-2.28597000
O	-1.08371300	0.66029300	-2.76017400
H	-7.24087300	-1.12616100	1.24889300
C	-6.06383700	2.12218000	0.59443400
C	-3.86861000	1.27102600	1.19088300
C	-6.84906200	-3.67145400	-0.30139400
C	-5.19418100	-4.46162100	-1.90127700
H	-3.57247000	-3.09423400	-2.36604700
H	-3.46245200	1.08581600	-3.37541800
H	-0.15019100	0.42523700	-2.39362300
C	-7.40055300	1.96046300	0.14411700
C	-5.56984100	3.44816800	0.82691500
C	-4.38644800	-0.90557000	-0.64981300
C	-3.36403800	2.56937100	1.36996800
C	-6.36454800	-4.66039400	-1.16247400
H	-7.76641600	-3.82719300	0.25967300
H	-4.83141800	-5.24698400	-2.55651600
C	-8.21806600	3.05280100	-0.03512500
H	-7.76693600	0.96193700	-0.06713100
C	-6.44231500	4.55161300	0.63400100
C	-4.21491900	3.63577200	1.19521200
H	-3.61594400	-0.53908400	2.00952200
C	-1.73113300	0.28177200	2.05473300
H	-2.32638500	2.73710800	1.62381100
H	-6.90576100	-5.59712300	-1.25958500
C	-7.73896700	4.36111600	0.21816900
H	-9.23814300	2.91289000	-0.38103700
H	-6.05490400	5.55116600	0.80785100
H	-3.84015500	4.64771900	1.30575400
H	-1.02731300	0.66032900	1.30835400
H	-1.85835400	1.05020900	2.82385300
C	-1.24027600	-0.99819800	2.68397200
H	-8.39657300	5.21278300	0.07100800
C	-2.03539000	-1.69736900	3.60419000
C	0.05047900	-1.45834100	2.40555600
C	-1.55428900	-2.85067100	4.22276200
H	-3.02690300	-1.33031000	3.87091700
C	0.53735700	-2.60085800	3.04032400
H	0.67091200	-0.90348100	1.71234400
C	-0.26323600	-3.30199700	3.94146400
H	-2.18058600	-3.38767700	4.92955000

H	1.54086800	-2.94444500	2.81870100
H	0.11643600	-4.20186700	4.41454600
H	-1.22802800	3.99066900	4.12925400
C	-0.44855300	3.93554200	3.37342800
C	-0.65982700	4.47274500	2.13118400
C	0.78745000	3.30082400	3.67514400
C	0.34669800	4.43051200	1.11551600
H	-1.60098400	4.95859900	1.90172800
C	1.78671600	3.26279100	2.74129600
H	0.93622200	2.84389600	4.64930000
C	1.62830500	3.85316600	1.44796900
C	0.09780600	4.87899400	-0.19705400
H	2.72131500	2.76670100	2.97114800
C	2.64965800	3.81963700	0.47734700
C	1.14385700	4.91377500	-1.14174400
C	-1.29234000	5.19121900	-0.64011000
C	3.86641500	2.99620800	0.71739700
C	2.44301800	4.39981300	-0.78972000
C	0.95447200	5.44177700	-2.45797700
C	-1.97060300	4.24649900	-1.42799500
C	-1.94387900	6.38559000	-0.30784200
C	3.95521800	1.75525100	0.02401500
C	4.85817900	3.30634200	1.61946200
C	3.48320400	4.44898700	-1.76770000
C	1.98022800	5.47215400	-3.36399200
H	-0.02092100	5.83245000	-2.72454400
C	-3.26792100	4.49414900	-1.87176000
H	-1.47056900	3.31806800	-1.68992500
C	-3.24373300	6.63433900	-0.75604800
H	-1.42220900	7.12434800	0.29430000
C	4.95585300	0.82620300	0.24787500
O	2.99849100	1.48860000	-0.92585900
C	5.90289600	2.39036700	1.91253700
H	4.82305400	4.25257600	2.15285400
C	3.26350900	4.96956600	-3.01389500
H	4.46113400	4.06395000	-1.50027000
H	1.81679300	5.88484800	-4.35559700
C	-3.90783200	5.69004100	-1.54067800
H	-3.78469000	3.74381500	-2.46340400
H	-3.73239100	7.57051100	-0.49848000
C	5.93885000	1.11702700	1.25305600
C	4.91778700	-0.46075000	-0.49971600
P	1.74744700	0.47945900	-0.54059900
C	6.88743600	2.69460700	2.88887900
H	4.06973000	4.99862700	-3.74128200
H	-4.92123200	5.87879200	-1.88282900

C	6.93023800	0.18283800	1.65643600
C	3.75391300	-1.21948600	-0.48717200
C	6.03225500	-0.93583700	-1.26872500
O	2.63248500	-0.75444700	0.16430100
O	0.85603300	1.01524000	0.52906100
O	1.20352800	0.02633700	-1.87134400
C	7.85328500	1.77763400	3.23276600
H	6.85263300	3.66851900	3.37095300
C	7.86256800	0.50476800	2.61723100
H	6.94351900	-0.79877500	1.19879100
C	3.67348600	-2.50928500	-1.08487000
C	5.95553000	-2.22364300	-1.89204700
C	7.20475200	-0.16022600	-1.47742900
H	8.59823900	2.02189100	3.98464700
H	8.60909700	-0.22873700	2.90872900
C	4.77239900	-2.99420600	-1.75611500
C	2.43131900	-3.31483200	-0.89645100
C	7.06125200	-2.70051000	-2.64353300
C	8.25485400	-0.64598700	-2.22323400
H	7.26021200	0.83251300	-1.04728400
H	4.73153200	-3.98357100	-2.20390000
C	1.38083100	-3.25429700	-1.83111700
C	2.30628300	-4.10808400	0.26207400
C	8.19174000	-1.93393500	-2.80425800
H	6.98968600	-3.68440400	-3.10051200
H	9.13785800	-0.03097800	-2.37255000
C	1.49951200	-2.52019400	-3.05180100
C	0.13254500	-3.92412700	-1.54868800
C	3.37694800	-4.24644200	1.20143000
C	1.06796200	-4.79850600	0.53017700
H	9.02942400	-2.30764200	-3.38610100
C	0.46262100	-2.44728600	-3.94068900
H	2.42549100	-1.99600000	-3.24886200
C	-0.92593700	-3.81510800	-2.50352600
C	-0.02311300	-4.64542800	-0.34820900
C	3.25708800	-5.04962000	2.30517800
H	4.30042000	-3.71041800	1.01494800
C	0.98748700	-5.62810400	1.69253300
C	-0.76701000	-3.10400700	-3.66230600
H	0.57034600	-1.86939500	-4.85327800
H	-1.85603500	-4.32810600	-2.29548600
C	-1.36449500	-5.16474200	0.04578400
C	2.04961700	-5.76203600	2.54649400
H	4.08835500	-5.14846700	2.99754800
H	0.06228500	-6.16020100	1.88276000
H	-1.58371900	-3.03962100	-4.37678900

C	-2.08944800	-4.47104100	1.02634100
C	-1.93054700	-6.31032100	-0.52627500
H	1.97139200	-6.40994100	3.41531300
C	-3.35469100	-4.90452900	1.41767200
H	-1.64189600	-3.59761600	1.48652200
C	-3.19500600	-6.74915400	-0.12930700
H	-1.37257000	-6.85725100	-1.28109000
C	-3.91127300	-6.04637500	0.84047100
H	-3.90152400	-4.34981500	2.17517800
H	-3.62109700	-7.64181500	-0.57951500
H	-4.90001600	-6.38189200	1.13829100

TS8-S3

C	-2.97770000	0.78009700	0.79366000
N	-1.64316900	1.30281900	1.97122500
C	-2.34501000	0.25152500	-0.37555100
H	-3.37700700	0.03234500	1.47550700
C	-3.41876200	3.17500700	1.59882400
C	-5.30978700	1.78913600	1.17252400
C	-3.91802800	2.60762800	-0.72117700
C	-2.34550400	0.94103500	-1.56179300
H	-1.84832800	-0.70663200	-0.31405200
C	-4.42447900	3.55503600	2.41505400
C	-2.03022900	3.59470900	1.44347200
C	-5.60908100	2.72739200	2.18339200
C	-6.26129500	0.88142100	0.73819600
C	-3.16969900	2.14006000	-1.72502100
H	-4.54778700	3.48374400	-0.84134500
O	-1.72966600	0.55635000	-2.66414100
H	-4.35991800	4.34346800	3.15780700
C	-1.61863600	4.88628300	1.00022300
C	-1.09527600	2.57160100	1.54485400
C	-6.86855600	2.74209100	2.78187000
C	-7.52355300	0.89202300	1.34691100
H	-6.04068200	0.18519200	-0.05910100
H	-3.16672800	2.60499200	-2.70552400
H	-1.01793600	-0.15549700	-2.49705600
C	-2.52014800	5.97006800	0.83516900
C	-0.23890700	5.07773200	0.66545700
C	-3.88741000	2.01551300	0.67158200
C	0.25661700	2.74766600	1.19901900
C	-7.82151400	1.81038600	2.35735700
H	-7.10699600	3.46270700	3.55926900
H	-8.27412300	0.18054600	1.01708400
C	-2.07263600	7.19143200	0.38440400
H	-3.57029800	5.82009000	1.06286300

C	0.18952300	6.35163600	0.20614400
C	0.66165600	3.98846000	0.76635800
H	-2.19270900	1.44593200	2.82069500
C	-0.73471600	0.14587300	2.12247900
H	0.95503700	1.92582600	1.22574500
H	-8.80667900	1.80560600	2.81509100
C	-0.70559300	7.38743300	0.07083000
H	-2.77385800	8.01211900	0.26239800
H	1.23764700	6.48465900	-0.04840100
H	1.69900800	4.13930500	0.49900600
H	-1.37605800	-0.73039500	2.26117800
H	-0.21802700	0.03486600	1.16989300
C	0.28787200	0.22219600	3.23052600
H	-0.36887200	8.35727000	-0.28402400
C	0.28918500	1.18635900	4.23940100
C	1.28890700	-0.76110400	3.22309600
C	1.27481600	1.16946200	5.23025800
H	-0.45821600	1.97550100	4.25020600
C	2.26473800	-0.78570600	4.21567800
H	1.30607000	-1.49171300	2.42060500
C	2.26131100	0.18429500	5.22294300
H	1.26912800	1.93116600	6.00516600
H	3.03312000	-1.55239500	4.19486000
H	3.02675200	0.17269400	5.99376400
H	-3.24454300	-3.11760800	-5.81858800
C	-2.91679900	-3.19610600	-4.78579800
C	-3.76681200	-2.84384100	-3.77332800
C	-1.60351000	-3.65670500	-4.49679400
C	-3.36886100	-2.93596100	-2.40038600
H	-4.76554200	-2.49567900	-4.00543300
C	-1.19454600	-3.79777200	-3.20051000
H	-0.92332200	-3.89310800	-5.30950400
C	-2.06379500	-3.47847400	-2.10955600
C	-4.21608700	-2.52793000	-1.34529900
H	-0.19076000	-4.13562000	-2.97957900
C	-1.66394600	-3.65855100	-0.77177700
C	-3.81104900	-2.70879000	-0.00188300
C	-5.49124200	-1.83229200	-1.66736800
C	-0.32560900	-4.24681000	-0.48898600
C	-2.51888800	-3.28850700	0.28348800
C	-4.61206600	-2.29142800	1.10954900
C	-5.45817700	-0.63603500	-2.40471400
C	-6.73573500	-2.32403700	-1.24748600
C	0.75339700	-3.41534900	-0.08556600
C	-0.09111100	-5.59333200	-0.66254700
C	-2.11841900	-3.44163300	1.64812300

C	-4.18661000	-2.44609300	2.40370500
H	-5.57310700	-1.83273600	0.92054600
C	-6.63278600	0.05210000	-2.70155600
H	-4.50090600	-0.25260800	-2.73919200
C	-7.91194600	-1.63225700	-1.54011900
H	-6.77680600	-3.25679100	-0.69289500
C	2.03411200	-3.90845400	0.12259400
O	0.49297400	-2.08192600	0.13458800
C	1.20297300	-6.14828000	-0.50644600
H	-0.90951400	-6.24076200	-0.96554700
C	-2.92156200	-3.03393600	2.68019200
H	-1.15105700	-3.88663300	1.85235300
H	-4.82063900	-2.11605100	3.22218000
C	-7.86427600	-0.44010900	-2.26495200
H	-6.58291700	0.98132000	-3.26176000
H	-8.86695600	-2.03053000	-1.20823400
C	2.29273400	-5.29399600	-0.14011400
C	3.10017300	-2.97345100	0.56449400
P	1.01560800	-0.94670300	-1.00406400
C	1.44683300	-7.52650600	-0.74553900
H	-2.59394600	-3.15813000	3.70849300
H	-8.77972500	0.09969800	-2.48926300
C	3.59660700	-5.85757200	-0.08692700
C	3.30520300	-1.80105900	-0.15050800
C	3.85948000	-3.20142300	1.75876000
O	2.52874700	-1.56882800	-1.26799600
O	0.21792600	-1.13255100	-2.26573600
O	1.08456700	0.35033100	-0.27236700
C	2.71712400	-8.04513200	-0.65721200
H	0.60615400	-8.16148600	-1.01366100
C	3.80196900	-7.19543600	-0.33660700
H	4.43752000	-5.21585700	0.14646600
C	4.27712900	-0.83013400	0.23025200
C	4.90350800	-2.28310400	2.09774700
C	3.58709900	-4.26847400	2.65734600
H	2.89315500	-9.10001100	-0.84699800
H	4.80861000	-7.60159900	-0.29392900
C	5.09444400	-1.12870400	1.30283800
C	4.34060600	0.51401900	-0.40311200
C	5.67040400	-2.50107800	3.27337300
C	4.33261600	-4.43966400	3.80230100
H	2.77171500	-4.94662700	2.43679200
H	5.85748000	-0.41431400	1.59742200
C	4.12772100	1.65251900	0.41067500
C	4.60636200	0.67021700	-1.77884100
C	5.39539400	-3.55727000	4.11049700

H	6.46534600	-1.79824600	3.50952000
H	4.09934500	-5.25735300	4.47829500
C	3.78536200	1.56266500	1.79859600
C	4.22547500	2.97067100	-0.16767400
C	4.90769900	-0.44534200	-2.61985400
C	4.57279600	1.98526300	-2.37508600
H	5.98038000	-3.70848900	5.01312800
C	3.68122200	2.67640800	2.58836500
H	3.58542700	0.59194700	2.22838700
C	4.16531500	4.10725000	0.70276100
C	4.37674300	3.11814000	-1.56110500
C	5.10404400	-0.29101700	-3.96489000
H	4.97545400	-1.42944600	-2.17211500
C	4.75023300	2.09720200	-3.79052400
C	3.92059000	3.96661200	2.04263000
H	3.40609000	2.56635600	3.63259200
H	4.31362700	5.09234400	0.27461400
C	4.26393600	4.47904400	-2.15884500
C	5.00687900	0.99617700	-4.56159200
H	5.32512300	-1.15593900	-4.58357900
H	4.68439800	3.07942400	-4.24438500
H	3.87854700	4.84285500	2.68379800
C	3.00344400	5.09202000	-2.23204900
C	5.38164300	5.17792200	-2.63094700
H	5.14161600	1.10318000	-5.63431500
C	2.86546800	6.37593800	-2.75722600
H	2.13251000	4.54748200	-1.87945400
C	5.24467400	6.46311600	-3.15838400
H	6.36018200	4.70951600	-2.57618700
C	3.98777300	7.06626200	-3.22046400
H	1.88072400	6.83303300	-2.80705600
H	6.12177300	6.99437000	-3.51782700
H	3.88294800	8.06703900	-3.63023400

TS8-S4

C	-2.54407800	0.88962200	0.85431800
N	-1.20179600	1.70030800	1.87300900
C	-1.92895600	0.33727300	-0.31290200
H	-2.79982400	0.16579400	1.62503100
C	-3.19923800	3.30546300	1.50079500
C	-4.94940400	1.70021400	1.32233000
C	-3.77226100	2.48440700	-0.71838700
C	-2.07441800	0.93105300	-1.54395000
H	-1.33751100	-0.56159500	-0.20472900
C	-4.18187700	3.64152700	2.36330600
C	-1.89225300	3.86802200	1.17907300

C	-5.28127100	2.67910600	2.28303000
C	-5.84063900	0.69381900	0.99534500
C	-3.03641000	2.01999700	-1.73216500
H	-4.50138700	3.27613600	-0.86091800
O	-1.48641300	0.55711300	-2.66404500
H	-4.15915500	4.49658400	3.03109700
C	-1.66724200	5.15541200	0.61109700
C	-0.84742300	2.95637800	1.25196300
C	-6.50336800	2.62423500	2.95252500
C	-7.06752700	0.63714700	1.66940600
H	-5.60248100	-0.02136900	0.22198600
H	-3.14248700	2.40298900	-2.74213000
H	-0.68052400	-0.05009800	-2.53875600
C	-2.68824800	6.13279600	0.48284900
C	-0.35413100	5.45752900	0.12116000
C	-3.59093800	2.01799000	0.70951300
C	0.42788900	3.22259700	0.71830800
C	-7.39053000	1.58829200	2.64120700
H	-6.76717300	3.37509300	3.69223300
H	-7.77382400	-0.14850800	1.41864000
C	-2.41476500	7.36221600	-0.07248200
H	-3.68979100	5.89500200	0.82578100
C	-0.10621700	6.73725500	-0.44267200
C	0.65585500	4.46466500	0.17204800
H	-1.72692400	1.90961000	2.72313000
C	-0.06915900	0.79703300	2.17578400
H	1.19441000	2.46145200	0.69010600
H	-8.34724200	1.53119300	3.15262200
C	-1.11178400	7.67143800	-0.53346900
H	-3.20515600	8.10193500	-0.16317900
H	0.89363900	6.95547600	-0.80905300
H	1.63119100	4.69236200	-0.24199600
H	0.33380600	0.45006200	1.22291400
H	0.71802300	1.41524500	2.62306500
C	-0.36805000	-0.35216500	3.10108200
H	-0.91333300	8.64680800	-0.96831100
C	0.51022600	-1.44457400	3.03943300
C	-1.36127100	-0.33933200	4.08413700
C	0.41783700	-2.48407500	3.96067500
H	1.26315800	-1.47291400	2.25956200
C	-1.46559800	-1.38884000	5.00006400
H	-2.06554600	0.48735600	4.16115200
C	-0.56936800	-2.45616100	4.94947100
H	1.11887600	-3.31213600	3.90187900
H	-2.24476500	-1.36515300	5.75681200
H	-0.64463400	-3.26499600	5.67096900

H	-3.21285500	-1.96813300	-6.10089600
C	-2.87113700	-2.21142900	-5.09860900
C	-3.73220400	-2.10202500	-4.04097100
C	-1.52984700	-2.63677000	-4.89670800
C	-3.31512700	-2.40953700	-2.70604600
H	-4.75359900	-1.78336000	-4.20870100
C	-1.09876600	-2.98209500	-3.64629200
H	-0.84588200	-2.68464100	-5.73874100
C	-1.97137000	-2.90348900	-2.51503500
C	-4.17968300	-2.27149200	-1.59741200
H	-0.07354000	-3.28790100	-3.48618000
C	-1.54184700	-3.28320900	-1.23027100
C	-3.73211100	-2.62164100	-0.30108300
C	-5.54384000	-1.70616200	-1.78774500
C	-0.20838100	-3.92239800	-1.04813900
C	-2.39418700	-3.13325300	-0.11864200
C	-4.54646300	-2.46193000	0.86552800
C	-5.70438500	-0.40529600	-2.29195600
C	-6.69342800	-2.43145200	-1.43997600
C	0.88278500	-3.21776500	-0.47469400
C	-0.02256300	-5.24004100	-1.40823100
C	-1.95774600	-3.46199200	1.20260600
C	-4.08141800	-2.76900100	2.11677700
H	-5.55509800	-2.08996600	0.75376200
C	-6.97108300	0.16056300	-2.42282600
H	-4.82312700	0.16741900	-2.55729900
C	-7.96193200	-1.86509600	-1.56691100
H	-6.58464000	-3.44466900	-1.06472100
C	2.10807900	-3.82348200	-0.22961900
O	0.69872900	-1.89587400	-0.12606000
C	1.22661000	-5.88867500	-1.25177900
H	-0.85254200	-5.79178900	-1.84095900
C	-2.76874900	-3.28114000	2.28921400
H	-0.95352400	-3.84449300	1.33797100
H	-4.72244100	-2.62170900	2.98168700
C	-8.10510000	-0.56454900	-2.05406800
H	-7.06997300	1.17439000	-2.80063000
H	-8.83947800	-2.44318000	-1.29028600
C	2.31807000	-5.17117800	-0.66597800
C	3.16577300	-3.03356600	0.44879900
P	1.39381000	-0.72523300	-1.12659600
C	1.42265000	-7.22900300	-1.67732400
H	-2.40393900	-3.51491700	3.28211900
H	-9.09266600	-0.12216200	-2.14925000
C	3.57956700	-5.82016800	-0.57870000
C	3.50624900	-1.79452500	-0.07247700

C	3.75930400	-3.45836100	1.68181000
O	2.90731900	-1.39247400	-1.24875000
O	0.73597200	-0.81109900	-2.47539100
O	1.43904300	0.53343300	-0.32846400
C	2.65040700	-7.83620100	-1.55617300
H	0.58085200	-7.76216400	-2.11192900
C	3.74013300	-7.11708000	-1.01058900
H	4.42410800	-5.27513600	-0.17392300
C	4.43338000	-0.92264600	0.56958100
C	4.77577700	-2.64301200	2.27519000
C	3.35191200	-4.63294900	2.37183300
H	2.79051500	-8.86052300	-1.88928800
H	4.71496400	-7.59110500	-0.93856600
C	5.09159000	-1.39339600	1.68799400
C	4.56314500	0.49428100	0.13701100
C	5.38728300	-3.06404300	3.48600000
C	3.94989600	-5.00210800	3.55640400
H	2.55801600	-5.23972500	1.95355700
H	5.81869600	-0.75401100	2.18083300
C	4.13034500	1.51444200	1.01644300
C	5.04069900	0.82145100	-1.14608700
C	4.98869300	-4.22012300	4.11483900
H	6.16558700	-2.43919000	3.91683100
H	3.62065500	-5.90280400	4.06690600
C	3.59791100	1.23522500	2.31585300
C	4.16792700	2.89363700	0.58718400
C	5.54733200	-0.17806100	-2.03328800
C	5.02480700	2.19287200	-1.59355700
H	5.45734800	-4.52761100	5.04516500
C	3.20659300	2.23663600	3.16525400
H	3.49416000	0.20433500	2.62763000
C	3.74870800	3.90690900	1.50831700
C	4.57001700	3.20856100	-0.72880400
C	5.99356700	0.14251600	-3.28573200
H	5.57410900	-1.20647700	-1.69286300
C	5.50206100	2.48386500	-2.91121900
C	3.29808100	3.59541100	2.76212200
H	2.81632100	1.98794400	4.14876200
H	3.78341100	4.94205800	1.19094800
C	4.44864300	4.60907200	-1.22582900
C	5.96816800	1.49277200	-3.73165100
H	6.37071300	-0.63483900	-3.94379600
H	5.50071100	3.51411400	-3.24787300
H	2.98524500	4.38483500	3.43952400
C	3.42985100	4.93170600	-2.13543700
C	5.30717400	5.62677000	-0.78901400

H	6.32974100	1.73696900	-4.72657500
C	3.26326200	6.24264200	-2.58306100
H	2.76633500	4.14445600	-2.48162100
C	5.14178100	6.93806300	-1.23581200
H	6.10350100	5.38306300	-0.09157500
C	4.11684100	7.25091800	-2.13048700
H	2.46678700	6.47443100	-3.28493400
H	5.81528600	7.71561300	-0.88603500
H	3.98862000	8.27225000	-2.47791600

TS8-S5

C	2.69202600	-1.09576200	-2.48882600
N	3.39827500	-0.19313000	-1.06611600
C	1.27306900	-1.09834200	-2.33790600
H	3.08129600	-0.39877900	-3.22803400
C	4.67977900	-2.44598900	-1.59994600
C	4.04678000	-2.74023700	-3.87460800
C	2.57835400	-3.61283100	-2.05456400
C	0.62206700	-2.19234000	-1.82319100
H	0.71087900	-0.19637900	-2.52651300
C	5.78633100	-2.73439700	-2.31817700
C	4.40335600	-2.19107800	-0.18947200
C	5.43843900	-2.92167800	-3.72881700
C	3.43210500	-2.85562900	-5.11042900
C	1.30174600	-3.48043700	-1.68171600
H	3.09430000	-4.56589700	-1.98677000
O	-0.64964700	-2.18443300	-1.49438400
H	6.79324900	-2.80441400	-1.92007000
C	4.66731100	-3.11370100	0.86819300
C	3.60739400	-1.08103800	0.06035800
C	6.22777500	-3.22140600	-4.83864400
C	4.22931800	-3.14959100	-6.22514900
H	2.35904000	-2.71968500	-5.21054000
H	0.71981300	-4.30898800	-1.29165400
H	-0.98732500	-1.20683300	-1.40775400
C	5.41480500	-4.30523000	0.67811200
C	4.11106100	-2.85015600	2.16398600
C	3.42902000	-2.44536100	-2.51574700
C	3.01484500	-0.85321300	1.31278500
C	5.60885900	-3.33138300	-6.08782800
H	7.29964200	-3.36715100	-4.73795000
H	3.77015100	-3.23775000	-7.20519500
C	5.62383500	-5.17858500	1.72114300
H	5.80940300	-4.52641400	-0.30733700
C	4.35241000	-3.76880300	3.21917100
C	3.28722100	-1.71286000	2.34963600

H	4.31106900	0.07314900	-1.44098600
C	2.60958300	1.04751200	-0.77793700
H	2.35621800	-0.01666100	1.46459500
H	6.20841900	-3.56150800	-6.96370700
C	5.09373400	-4.90766700	3.00569100
H	6.19465400	-6.08798900	1.55765100
H	3.92478900	-3.55928800	4.19601200
H	2.84272100	-1.53113200	3.32426500
H	1.60978300	0.73522800	-0.48004100
H	3.08727700	1.53042000	0.07466500
C	2.56201500	1.98578000	-1.95337000
H	5.26642600	-5.60710200	3.81818800
C	3.73605400	2.57739600	-2.43750100
C	1.33571300	2.30822200	-2.54691700
C	3.69365200	3.44379700	-3.52807000
H	4.68369300	2.38763500	-1.93664800
C	1.29455300	3.17829700	-3.63804000
H	0.41423900	1.88840400	-2.15252500
C	2.47168600	3.73634900	-4.13771400
H	4.60859900	3.90215500	-3.89167000
H	0.33750600	3.42020400	-4.09034300
H	2.43751200	4.41188300	-4.98772800
H	-5.38664300	-1.63214800	-5.35091200
C	-5.19959400	-1.60612200	-4.28078500
C	-6.13403300	-1.07305700	-3.43531300
C	-3.98083100	-2.12358400	-3.76346200
C	-5.91620400	-1.02415900	-2.02119000
H	-7.06328500	-0.67730600	-3.82850400
C	-3.73551000	-2.10947300	-2.41730400
H	-3.23589600	-2.52967300	-4.44235800
C	-4.68929800	-1.57664100	-1.49294200
C	-6.86859100	-0.46112700	-1.14901400
H	-2.80255600	-2.50274700	-2.03867100
C	-4.45985600	-1.56404200	-0.10112000
C	-6.64184600	-0.45535500	0.24237600
C	-8.11768000	0.13887700	-1.70090200
C	-3.19694200	-2.11561400	0.46476000
C	-5.42781900	-1.02538900	0.77440900
C	-7.58486200	0.11694800	1.15428400
C	-8.08814300	1.39709500	-2.31768200
C	-9.33979800	-0.54102000	-1.61294000
C	-2.27612200	-1.24659800	1.11921700
C	-2.88842100	-3.45600400	0.40181000
C	-5.25096600	-1.03101200	2.19367000
C	-7.36564900	0.10394100	2.50512800
H	-8.48469300	0.56776300	0.75211300

C	-9.25377200	1.96207800	-2.83465500
H	-7.14295100	1.92774300	-2.38578300
C	-10.50624600	0.02285800	-2.12975500
H	-9.36734500	-1.51630700	-1.13535700
C	-1.16181000	-1.70960700	1.80246400
O	-2.52984800	0.10556900	1.07897400
C	-1.66462200	-3.95946200	0.91050200
H	-3.57578000	-4.13815700	-0.09058300
C	-6.18551800	-0.48736000	3.03249600
H	-4.35445600	-1.47873800	2.60587300
H	-8.09454200	0.54594500	3.17880900
C	-10.46608300	1.27633600	-2.74226200
H	-9.21507900	2.93954600	-3.30773600
H	-11.44602100	-0.51766600	-2.05516100
C	-0.78895900	-3.08475100	1.62943300
C	-0.42205800	-0.77640000	2.69596000
P	-1.54432400	0.99222200	0.06377600
C	-1.27655200	-5.30842600	0.70089400
H	-6.02374000	-0.50252400	4.10668800
H	-11.37430200	1.71643100	-3.14477000
C	0.45119900	-3.60270900	2.08326700
C	0.02273300	0.45411700	2.22702600
C	-0.22862300	-1.09608200	4.08487100
O	-0.08316900	0.76856300	0.89817300
O	-1.31743300	0.23476800	-1.23252600
O	-1.97165800	2.40568600	0.07627800
C	-0.05959400	-5.77604300	1.14620000
H	-1.95602700	-5.96292600	0.16055700
C	0.81661200	-4.90720500	1.83757100
H	1.12718300	-2.94389900	2.60840900
C	0.73098100	1.37922400	3.06220900
C	0.51205900	-0.19375900	4.91560500
C	-0.76038900	-2.26770200	4.69044200
H	0.22994600	-6.80743300	0.96488300
H	1.78664400	-5.26417100	2.17247200
C	0.97536800	1.03006400	4.37085900
C	1.20961500	2.66375900	2.47489000
C	0.74351300	-0.52229800	6.27731600
C	-0.52764500	-2.55381000	6.01703700
H	-1.36369100	-2.94106300	4.09556700
H	1.51679200	1.71985600	5.01286600
C	0.27483700	3.66854600	2.15325800
C	2.57445100	2.84379500	2.16186800
C	0.24418100	-1.68287800	6.82019300
H	1.31524100	0.17374900	6.88622000
H	-0.94986800	-3.45501800	6.45257100

C	-1.07711600	3.60123200	2.60429700
C	0.68670800	4.81162000	1.37437300
C	3.59306700	1.93937100	2.60263700
C	2.97391200	3.95616900	1.32785800
H	0.42356900	-1.92253000	7.86432700
C	-1.96639600	4.60101000	2.32432500
H	-1.39230300	2.73750200	3.17809100
C	-0.28219100	5.82015200	1.08199400
C	2.01897300	4.90971200	0.92408200
C	4.89418200	2.06221300	2.19239300
H	3.31620500	1.13784700	3.27491000
C	4.33694900	4.02633600	0.89442400
C	-1.56556400	5.72026400	1.54867000
H	-2.99142300	4.52686300	2.67348100
H	0.02367300	6.67829900	0.49463600
C	2.41621500	6.00950300	0.00010600
C	5.26796200	3.10772100	1.30332900
H	5.64043100	1.35448000	2.54247600
H	4.62251100	4.83225900	0.22861200
H	-2.28589800	6.50153000	1.32306700
C	2.06228500	5.93683700	-1.35314100
C	3.15633500	7.11062700	0.45026900
H	6.29750800	3.18780900	0.96483200
C	2.44577500	6.94153100	-2.23987300
H	1.49385800	5.08274800	-1.70402600
C	3.53638200	8.11880700	-0.43618300
H	3.43151300	7.17058100	1.49953700
C	3.18304400	8.03579600	-1.78422100
H	2.16992500	6.86485100	-3.28795600
H	4.10729500	8.96934900	-0.07362200
H	3.48024300	8.82047000	-2.47462600

Int9

C	-3.12748700	-0.67025100	-0.10633300
N	-2.99858300	0.41493300	1.10283600
C	-2.00480600	-0.50941400	-1.03587500
H	-3.06099300	-1.59752800	0.46672100
C	-5.39008900	0.53759500	0.14332300
C	-5.45210200	-1.73249200	-0.55635900
C	-4.61399100	0.00885500	-2.10035600
C	-2.17340800	0.02262900	-2.27505400
H	-1.02335500	-0.80977200	-0.69907700
C	-6.55034400	-0.00806500	0.57185000
C	-4.74248800	1.83764000	0.29457900
C	-6.62868000	-1.41027000	0.15491700
C	-5.26358200	-2.99296800	-1.09540200

C	-3.51588300	0.28632400	-2.80408000
H	-5.60872900	0.17299400	-2.50385500
O	-1.20893200	0.28541300	-3.14800200
H	-7.30921400	0.50238700	1.15587000
C	-5.28631000	3.10554200	-0.06268100
C	-3.41055800	1.75703200	0.66931400
C	-7.62078900	-2.37191000	0.34545000
C	-6.26082400	-3.95710300	-0.90257600
H	-4.36016200	-3.22514300	-1.64744100
H	-3.56964700	0.67661900	-3.81548200
H	-0.29108300	0.14337100	-2.74175900
C	-6.63149300	3.27527500	-0.47963800
C	-4.42233300	4.24952800	-0.01615600
C	-4.57537500	-0.49852000	-0.67071300
C	-2.53585000	2.85481700	0.65574100
C	-7.42270100	-3.64903300	-0.18916700
H	-8.53087800	-2.13475200	0.88968100
H	-6.11875800	-4.95675200	-1.29829800
C	-7.11127700	4.52482100	-0.79982700
H	-7.27355400	2.40381800	-0.55064500
C	-4.95320600	5.52470800	-0.34766600
C	-3.05551900	4.08726400	0.32611600
H	-3.71764500	0.09764300	1.76094300
C	-1.68307000	0.38036200	1.83643700
H	-1.48540000	2.74624500	0.89128500
H	-8.18385200	-4.41145700	-0.04989700
C	-6.26862100	5.66065100	-0.72543300
H	-8.14311900	4.64250900	-1.11800300
H	-4.29187300	6.38515900	-0.30396100
H	-2.40659100	4.95489600	0.31176100
H	-0.88580900	0.61745800	1.12903100
H	-1.74074900	1.18850600	2.57192600
C	-1.43774300	-0.94128700	2.51041200
H	-6.66446300	6.63969100	-0.97939100
C	-2.34219400	-1.46880700	3.44303500
C	-0.23556300	-1.61246400	2.26185500
C	-2.05331700	-2.65765700	4.11170300
H	-3.26880400	-0.94224600	3.67247300
C	0.06101200	-2.79021900	2.94736900
H	0.46900000	-1.19904900	1.54912000
C	-0.84644900	-3.31701600	3.86582000
H	-2.76199300	-3.06240500	4.82873400
H	0.99857000	-3.29715700	2.75340900
H	-0.61703300	-4.24562600	4.37899900
H	-0.98326500	3.67602600	4.02924900
C	-0.14406700	3.60912000	3.34132800

C	-0.03615300	4.50709600	2.31213700
C	0.82621200	2.58337400	3.50714900
C	1.05642200	4.45855900	1.38714100
H	-0.79439300	5.27023600	2.18663200
C	1.88247200	2.49535600	2.64269700
H	0.71814800	1.85922600	4.30970200
C	2.05143100	3.42685900	1.57021300
C	1.16195500	5.35076300	0.29610000
H	2.60429800	1.69535700	2.74747900
C	3.13075200	3.32699300	0.67091700
C	2.26223600	5.26424400	-0.58596400
C	0.08946000	6.35098700	0.03361400
C	4.11769000	2.22104400	0.82631800
C	3.25353100	4.23307900	-0.39812200
C	2.44887500	6.18805400	-1.66374200
C	-0.68595800	6.25285000	-1.13317700
C	-0.18031000	7.39719200	0.92813600
C	4.00922600	1.07052800	-0.00154300
C	5.10754100	2.24737500	1.78256700
C	4.34702000	4.15628100	-1.31581200
C	3.51524300	6.08290700	-2.51588300
H	1.73204400	6.99010900	-1.79315500
C	-1.70130600	7.17189300	-1.39689700
H	-0.48873100	5.44216500	-1.82805600
C	-1.20168300	8.31167900	0.67112500
H	0.42028000	7.48956800	1.82825300
C	4.82290700	-0.04120700	0.14267300
O	3.06276300	1.08512200	-0.99881500
C	5.96644300	1.13811600	1.99016800
H	5.21280100	3.12203800	2.41888600
C	4.47366400	5.04660000	-2.34662400
H	5.08267500	3.37245600	-1.17651900
H	3.63482900	6.79899200	-3.32415800
C	-1.96553900	8.20294200	-0.49269800
H	-2.29108800	7.07602600	-2.30430000
H	-1.39512500	9.11525600	1.37632100
C	5.80633500	-0.03985800	1.18859500
C	4.56604900	-1.24195400	-0.69742100
P	1.64751700	0.25187500	-0.78226800
C	6.95063200	1.15705900	3.01316700
H	5.31074700	4.96859900	-3.03433600
H	-2.75510600	8.92095700	-0.69700300
C	6.60759900	-1.17200900	1.50004800
C	3.29057700	-1.78989700	-0.70350900
C	5.58332500	-1.87157700	-1.48645100
O	2.26245200	-1.13634200	-0.06274000

O	0.77917100	0.87862800	0.25794500
O	1.15747300	-0.06263100	-2.16729400
C	7.73159300	0.05383100	3.26583800
H	7.06478100	2.06328500	3.60287200
C	7.54496300	-1.12499500	2.50725600
H	6.47130800	-2.08525800	0.93454100
C	3.00461200	-3.05104300	-1.29723700
C	5.28987200	-3.10841000	-2.14699300
C	6.86921200	-1.29718100	-1.67295200
H	8.47912300	0.07942100	4.05353500
H	8.14363900	-2.00513000	2.72477500
C	4.00379800	-3.68878700	-1.99697000
C	1.68825300	-3.70111100	-1.02621300
C	6.29720700	-3.73490400	-2.92616400
C	7.82048300	-1.92436100	-2.44537900
H	7.08954100	-0.34512500	-1.20406600
H	3.80704400	-4.66015100	-2.44273000
C	0.59276400	-3.53784900	-1.89329400
C	1.55917400	-4.47783200	0.14442100
C	7.53860600	-3.16063200	-3.07245100
H	6.06241800	-4.67767900	-3.41403200
H	8.79465600	-1.46272500	-2.57988600
C	0.70348600	-2.79724700	-3.11060700
C	-0.68751100	-4.11323500	-1.54840500
C	2.66227800	-4.70278400	1.02889200
C	0.28376300	-5.06199600	0.48180700
H	8.29942500	-3.64723000	-3.67601600
C	-0.37130900	-2.63543900	-3.94126200
H	1.65253500	-2.33539300	-3.34814500
C	-1.78070700	-3.92134000	-2.45001300
C	-0.83429400	-4.82813700	-0.34411300
C	2.53236000	-5.48402500	2.14707500
H	3.61694700	-4.24771000	0.79258400
C	0.19487200	-5.87550700	1.65561700
C	-1.62879900	-3.20918800	-3.60873900
H	-0.27251600	-2.04650600	-4.84695000
H	-2.73322400	-4.37614300	-2.20583000
C	-2.18107700	-5.27361600	0.11623200
C	1.28359200	-6.09026800	2.45790300
H	3.38702900	-5.64619500	2.79775600
H	-0.75915100	-6.32930300	1.89802000
H	-2.47462300	-3.07211300	-4.27678200
C	-2.82007600	-4.54469200	1.13065900
C	-2.81690600	-6.40760800	-0.40289000
H	1.19567300	-6.72216700	3.33746200
C	-4.05836900	-4.94732200	1.62473700

H	-2.32461200	-3.67185200	1.53998600
C	-4.05908600	-6.81166700	0.09169900
H	-2.32671300	-6.97865800	-1.18626700
C	-4.68008800	-6.08554900	1.10923900
H	-4.53844500	-4.36731900	2.40785500
H	-4.53709600	-7.69965000	-0.31345600
H	-5.64704300	-6.39972100	1.49174900

TS10

N	0.34615000	-2.32605800	0.38865800
H	-0.34476200	-1.21642400	0.34663100
O	-0.79851700	-0.12057200	0.27242600
H	1.68785900	-0.74033200	0.50455800
C	0.04052900	-1.66199800	2.94319600
C	1.80646900	-0.08169600	2.94493100
C	2.33606900	-2.45150200	3.34608800
C	3.63429500	-3.16419500	1.42266800
H	3.18158400	-2.36352700	-0.47549300
C	-0.37792100	-0.55452400	3.59671700
C	-0.59416900	-2.90437600	2.53484700
C	0.67942400	0.45335300	3.60339800
C	2.97350800	0.64505100	2.80475900
C	3.31189500	-3.21604500	2.84606200
H	2.10355900	-2.45806900	4.40688000
O	4.77473400	-3.83052800	1.09519300
H	-1.36556000	-0.39815400	4.01207800
C	-1.37153500	-3.74633900	3.38595900
C	-0.31540300	-3.30292300	1.22919600
C	0.71413300	1.75214900	4.11035900
C	3.00529500	1.94979800	3.31340000
H	3.83675600	0.21895100	2.30786400
H	3.91367400	-3.86508200	3.47424600
H	4.91744500	-3.77036100	0.13224200
C	-1.66443800	-3.40911300	4.73383700
C	-1.87940200	-4.97879000	2.86317700
C	1.50218000	-1.50280800	2.50318600
C	-0.75975800	-4.55158000	0.74345700
C	1.89085200	2.49209600	3.95949300
H	-0.15085200	2.19014300	4.59984600
H	3.90654400	2.54439700	3.20137900
C	-2.46363400	-4.22093400	5.50697600
H	-1.24061300	-2.50230000	5.15019700
C	-2.70110700	-5.79240200	3.68679600
C	-1.53457500	-5.35927800	1.54277500
C	1.65462300	-1.72548700	0.96999100
C	0.52373300	-2.68899200	-1.04000500

H	-0.53516600	-4.85928200	-0.26822500
H	1.93070800	3.50577000	4.34183700
C	-2.99813500	-5.41950200	4.97710800
H	-2.68021100	-3.94446300	6.53489400
H	-3.08889500	-6.72134600	3.27639400
H	-1.89966000	-6.30733400	1.15781700
H	1.05542500	-1.85122300	-1.50058500
H	1.17050400	-3.57097300	-1.10719900
C	-0.75412800	-2.91418800	-1.82265700
H	-3.63057700	-6.04863800	5.59659300
C	-2.02715000	-2.54349000	-1.38034200
C	-0.62302400	-3.51243900	-3.08153700
C	-3.14355500	-2.78143500	-2.18092800
H	-2.17023000	-2.06918200	-0.41794100
C	-1.73725700	-3.73656000	-3.88735300
H	0.36626100	-3.79203400	-3.43831000
C	-3.00689900	-3.37443800	-3.43443600
H	-4.12338400	-2.49910800	-1.81646400
H	-1.61311100	-4.19698900	-4.86381700
H	-3.88454000	-3.55305300	-4.04976200
H	-4.71502000	-3.07441100	2.03043600
C	-4.53572400	-2.06687700	1.66621800
C	-5.15640300	-1.62199100	0.53047900
C	-3.63741100	-1.21924800	2.36980100
C	-4.92284600	-0.30375400	0.01862000
H	-5.83560700	-2.27410700	-0.00568600
C	-3.36332100	0.03619600	1.90280000
H	-3.15977600	-1.59065100	3.26888200
C	-3.98533200	0.54396800	0.72024000
C	-5.54169400	0.15913000	-1.16217200
H	-2.64221300	0.66458700	2.40825500
C	-3.67937600	1.82114400	0.21227600
C	-5.26717500	1.45762100	-1.64384000
C	-6.46061800	-0.73233700	-1.92496900
C	-2.63093800	2.66836100	0.85190700
C	-4.31788100	2.29538500	-0.95233400
C	-5.91902900	1.98595200	-2.80327500
C	-7.70579100	-1.11024500	-1.40432800
C	-6.08460400	-1.21647500	-3.18759400
C	-1.37907000	2.81814200	0.19618400
C	-2.81924400	3.34971800	2.03254300
C	-4.05560600	3.60043700	-1.47486900
C	-5.63875500	3.24262400	-3.26822100
H	-6.65352400	1.37163700	-3.31034200
C	-8.55040500	-1.95761400	-2.12162100
H	-8.00691900	-0.73400100	-0.43088700

C	-6.92750800	-2.06488200	-3.90438400
H	-5.12048000	-0.92948400	-3.59567000
C	-0.36850900	3.65155700	0.63877600
O	-1.19003700	2.09178500	-0.96801600
C	-1.79642300	4.16082000	2.59073800
H	-3.76738300	3.26184100	2.55597200
C	-4.68887900	4.05973700	-2.59764700
H	-3.34332300	4.23367500	-0.95874900
H	-6.14873200	3.62172800	-4.14937700
C	-8.16282700	-2.43943600	-3.37274500
H	-9.51290400	-2.23934000	-1.70346100
H	-6.61862300	-2.43270500	-4.87917200
C	-0.55569700	4.32571600	1.89182100
C	0.87549800	3.78322300	-0.17181100
P	-0.24311400	0.76489600	-0.86645500
C	-1.98124100	4.81161000	3.83899600
H	-4.47222500	5.05521100	-2.97465600
H	-8.82002900	-3.10053500	-3.93066800
C	0.45338800	5.12176500	2.49467400
C	1.58528600	2.64665400	-0.53384200
C	1.34282900	5.05613000	-0.64077900
O	1.11741100	1.40614400	-0.15492800
C	2.87399600	-2.47455600	0.55386300
O	-0.00124400	0.19384700	-2.20790000
C	-0.98740600	5.58860100	4.38814700
H	-2.92899900	4.67980100	4.35491100
C	0.24174800	5.74125200	3.70598900
H	1.40840300	5.22427400	1.99473400
C	2.81655400	2.69761600	-1.24744600
C	2.57967100	5.12356400	-1.36090900
C	0.60904600	6.25942500	-0.45682200
H	-1.14177600	6.08095400	5.34388600
H	1.03088600	6.34395000	4.14695000
C	3.29746700	3.93036800	-1.62816100
C	3.56756300	1.43598600	-1.52686900
C	3.05425000	6.38235000	-1.81436100
C	1.09078700	7.46271800	-0.92005100
H	-0.34773600	6.21949900	0.04948100
H	4.24061300	3.99132000	-2.16442600
C	3.25724900	0.65784800	-2.65865100
C	4.58166200	1.02450300	-0.63852900
C	2.33155000	7.53154200	-1.59565900
H	3.99924500	6.41581200	-2.35063300
H	0.50850000	8.36759900	-0.77124600
C	2.30294700	1.10037300	-3.62504400
C	3.93692700	-0.59975600	-2.87840900

C	5.00679200	1.85364300	0.44708000
C	5.22060200	-0.25924700	-0.81939300
H	2.70220700	8.48837000	-1.95162600
C	2.03454400	0.36736200	-4.74678700
H	1.78332500	2.03320200	-3.44750600
C	3.63187800	-1.32637700	-4.07328600
C	4.89109200	-1.05785100	-1.94025400
C	5.97164300	1.44021900	1.32517400
H	4.54172500	2.82488900	0.56440800
C	6.15254300	-0.68602600	0.18077300
C	2.71041300	-0.86016900	-4.97332900
H	1.29441600	0.71710800	-5.45925000
H	4.16190500	-2.24938600	-4.27451600
C	5.56454000	-2.37272700	-2.13626200
C	6.52397400	0.13588000	1.21146800
H	6.28806500	2.09144900	2.13501100
H	6.56377500	-1.68581200	0.12383000
H	2.50155600	-1.42969700	-5.87477300
C	4.83016300	-3.57143800	-2.19858000
C	6.96124000	-2.45115300	-2.25434500
H	7.23814300	-0.21418500	1.95110100
C	5.47175600	-4.80344600	-2.34380700
H	3.74643400	-3.53535800	-2.14807300
C	7.60285500	-3.67953900	-2.40418100
H	7.54198600	-1.53453600	-2.22660700
C	6.86238000	-4.86205300	-2.44078400
H	4.88174900	-5.71478400	-2.38143300
H	8.68509900	-3.71259200	-2.49202400
H	7.36312100	-5.81929200	-2.55074900

TS10-S1

N	0.06611100	-1.70801800	1.04418200
H	-0.46733500	-0.64378400	0.51775400
O	-0.89705000	0.30043000	-0.05659300
H	-1.71432900	-1.56116200	2.13705600
C	-1.90550900	-2.90610800	-0.41102400
C	-3.30392100	-3.53033000	1.40693600
C	-1.21470200	-4.78922700	1.05627800
C	-0.20110600	-4.31545300	3.19722800
H	-0.38221700	-2.37259600	4.01864800
C	-3.19605700	-2.78304800	-0.80200900
C	-0.61257400	-2.75575300	-1.05263700
C	-4.08633400	-3.19549300	0.28118700
C	-3.89114500	-3.96173400	2.58543900
C	-0.44600200	-5.20790500	2.06693900
H	-1.46074400	-5.44059600	0.22330700

O	0.32845800	-4.94738300	4.28422900
H	-3.54164600	-2.41306900	-1.75824700
C	-0.34146200	-3.13951000	-2.40456800
C	0.43480500	-2.34625300	-0.22179100
C	-5.47633600	-3.31278900	0.33080700
C	-5.28878800	-4.05387500	2.64187400
H	-3.28113200	-4.22709800	3.44340000
H	-0.03437900	-6.21095700	2.11055700
H	0.45879100	-4.29243200	4.98934300
C	-1.33737000	-3.62864400	-3.29104100
C	1.00025900	-3.04827600	-2.88950200
C	-1.83344000	-3.40244100	1.03247200
C	1.76333600	-2.33245100	-0.69340100
C	-6.06749700	-3.74025200	1.52274000
H	-6.08700800	-3.06540800	-0.53037900
H	-5.76935300	-4.38481600	3.55824400
C	-1.03081500	-3.94389700	-4.59568700
H	-2.34720800	-3.77089700	-2.92911300
C	1.28449700	-3.36973900	-4.24188600
C	2.02772600	-2.66675100	-2.00087800
C	-1.04363600	-2.38706000	1.89190200
C	1.23550300	-1.21442700	1.83460600
H	2.58493200	-2.03695000	-0.05808100
H	-7.14909000	-3.82794300	1.57860100
C	0.28850200	-3.79852900	-5.08600900
H	-1.81050700	-4.31427300	-5.25564000
H	2.30890900	-3.26670400	-4.58955100
H	3.04341200	-2.62679700	-2.37091500
H	1.82601000	-2.06876000	2.17472500
H	1.84107000	-0.63815300	1.13604200
C	0.93086900	-0.30010700	3.00188600
H	0.51042800	-4.03957400	-6.12162800
C	0.32429200	0.94853100	2.82148100
C	1.42104000	-0.62845300	4.27191900
C	0.20811100	1.84194800	3.88678800
H	-0.03678500	1.23110700	1.84377000
C	1.30199300	0.25878200	5.34088800
H	1.92437000	-1.58156300	4.41314500
C	0.69351700	1.50067000	5.14985500
H	-0.24516400	2.81528000	3.72085300
H	1.69300800	-0.01456600	6.31731000
H	0.60729900	2.20069200	5.97617100
H	-4.88560200	-1.17910700	3.28995000
C	-4.54712000	-0.39163600	2.62477400
C	-5.15148300	-0.22839800	1.40791100
C	-3.45949400	0.44296100	2.99702700

C	-4.72652900	0.77611600	0.48212800
H	-5.95786900	-0.88876200	1.12755600
C	-3.03201800	1.42897300	2.15027100
H	-2.95907600	0.29897200	3.95089000
C	-3.64624600	1.64829200	0.87743700
C	-5.29923100	0.90627500	-0.80397500
H	-2.19911100	2.05264700	2.43783500
C	-3.19051900	2.64644700	-0.00928600
C	-4.86676600	1.93363300	-1.67208000
C	-6.30979200	-0.07914400	-1.27876100
C	-2.03423800	3.49217700	0.38915300
C	-3.79821200	2.81441500	-1.26744900
C	-5.47763000	2.15785300	-2.94761300
C	-6.00686800	-0.91267600	-2.36756500
C	-7.56105600	-0.21716900	-0.66247500
C	-0.77453700	3.34031900	-0.25310300
C	-2.11796300	4.37764500	1.44253500
C	-3.38714000	3.84911200	-2.16422300
C	-5.05222700	3.16114000	-3.77597100
H	-6.30393400	1.52449900	-3.24735100
C	-6.92145800	-1.86302100	-2.81910600
H	-5.04385900	-0.80389300	-2.85766500
C	-8.47444900	-1.17374100	-1.10610900
H	-7.81392400	0.43131600	0.17099400
C	0.36356800	4.01521400	0.15130800
O	-0.68727900	2.48130800	-1.33405800
C	-0.97908300	5.05712000	1.94109800
H	-3.07217800	4.51406000	1.94362500
C	-3.98696800	4.01603100	-3.38202000
H	-2.58647300	4.51228400	-1.85758300
H	-5.53691100	3.31306400	-4.73632200
C	-8.15788300	-2.00167100	-2.18445900
H	-6.66598000	-2.49946700	-3.66188700
H	-9.43812200	-1.26684700	-0.61291700
C	0.29168400	4.85714800	1.31095800
C	1.65507200	3.75551200	-0.53569800
P	-0.00944100	1.00155600	-1.11018900
C	-1.06324400	5.88352400	3.09326300
H	-3.65644400	4.80811400	-4.04767100
H	-8.87029200	-2.74497400	-2.53084500
C	1.43544100	5.45905900	1.90155000
C	2.12715200	2.45416300	-0.60904500
C	2.44983700	4.80742000	-1.09570800
O	1.32458000	1.41364200	-0.18752200
C	-0.52159000	-3.00918400	3.15346400
O	0.33820100	0.38233400	-2.40311800

C	0.06153200	6.46670900	3.62828900
H	-2.03709300	6.03185700	3.55295700
C	1.32282800	6.24055100	3.02936500
H	2.40903800	5.28860500	1.45904200
C	3.44958300	2.14625300	-1.02535000
C	3.75346600	4.49977800	-1.60178000
C	1.98338000	6.14496600	-1.19879300
H	-0.01338300	7.09044300	4.51433400
H	2.21301100	6.68345100	3.46678900
C	4.23410600	3.16943300	-1.51352600
C	4.02226500	0.78803700	-0.81528500
C	4.54511800	5.53851700	-2.15792100
C	2.77203100	7.12759100	-1.75322300
H	0.98703500	6.38190500	-0.84379900
H	5.25103600	2.95319200	-1.82889500
C	4.34744800	-0.03383500	-1.90996000
C	4.29993600	0.36292000	0.50203200
C	4.06908700	6.82678400	-2.23163500
H	5.53564500	5.29153800	-2.53137400
H	2.39294400	8.14267000	-1.82974300
C	3.96270200	0.30824400	-3.24429200
C	5.05565000	-1.27325100	-1.69130400
C	3.96833600	1.16142300	1.64293300
C	4.91470600	-0.92493200	0.72416500
H	4.68171000	7.61280300	-2.66351700
C	4.31538500	-0.48075700	-4.30549900
H	3.35317700	1.19108000	-3.39469300
C	5.45123700	-2.03867200	-2.83529400
C	5.30736100	-1.71584700	-0.37694000
C	4.16848800	0.70379400	2.91638000
H	3.52640600	2.13799700	1.48862600
C	5.09245000	-1.37017800	2.07352300
C	5.09619100	-1.65304400	-4.10093500
H	3.99591000	-0.21489500	-5.30858900
H	6.02415200	-2.94545400	-2.67924200
C	5.94358200	-3.04493900	-0.14840700
C	4.72697000	-0.58525300	3.13444200
H	3.87405400	1.31435700	3.76379600
H	5.52491300	-2.34983000	2.24131700
H	5.40033400	-2.25181700	-4.95511500
C	5.20547900	-4.22190900	-0.33950700
C	7.27814900	-3.14257200	0.26680800
H	4.86391600	-0.94570900	4.15003800
C	5.79110300	-5.46841200	-0.12058500
H	4.16865400	-4.15243800	-0.65576500
C	7.86451800	-4.38945700	0.48402300

H	7.85260300	-2.23289900	0.41678100
C	7.12213500	-5.55549200	0.29089100
H	5.20617800	-6.37173100	-0.27006900
H	8.90140900	-4.44985300	0.80296700
H	7.57841400	-6.52672600	0.46039800

TS10-S2

N	-0.25812500	-2.24120800	-0.48511100
H	-0.63367600	-1.04320200	-0.41310300
O	-0.93681700	0.14205800	-0.24628500
H	1.30422700	-1.12236200	0.28969700
C	-1.00056700	-1.92510400	2.07500800
C	1.09465900	-1.09224100	2.80872100
C	0.76850300	-3.53817700	2.63142200
C	2.30578900	-4.18711300	0.87754600
H	2.63256900	-2.90023000	-0.76637900
C	-1.22542000	-0.87195700	2.89109100
C	-1.89503800	-2.70701200	1.24254000
C	0.04362200	-0.32520800	3.35627500
C	2.41867500	-0.79053200	3.08011000
C	1.59384100	-4.45781700	2.12095800
H	0.25999400	-3.69731800	3.57748200
O	3.23501600	-5.13557600	0.57360000
H	-2.19351800	-0.43934400	3.10024500
C	-3.15997300	-3.21780700	1.67306500
C	-1.46099800	-2.93841200	-0.05916100
C	0.32298000	0.77186000	4.17095900
C	2.69542900	0.30967200	3.90141000
H	3.22824600	-1.37532700	2.65541300
H	1.79634700	-5.39814800	2.62364400
H	3.70031700	-4.87074900	-0.23703600
C	-3.61256700	-3.10462800	3.01280700
C	-4.00326700	-3.87321400	0.72195800
C	0.49879600	-2.20733200	1.95865400
C	-2.28318800	-3.62263200	-0.98360100
C	1.65934700	1.08151600	4.43570800
H	-0.48104700	1.37548700	4.58273000
H	3.72627900	0.56912300	4.10854000
C	-4.85699200	-3.56598900	3.37853800
H	-2.95946900	-2.65013100	3.74825200
C	-5.29264700	-4.31020500	1.12156800
C	-3.52456600	-4.06841200	-0.59690800
C	0.96325900	-2.14351800	0.47776600
C	0.13807900	-2.38041800	-1.91028900
H	-1.94806600	-3.79275500	-1.99738100
H	1.89826100	1.93670100	5.06187100

C	-5.71750100	-4.15200600	2.42047200
H	-5.18432100	-3.47483100	4.41057100
H	-5.94103400	-4.76335300	0.37812400
H	-4.16051700	-4.57835100	-1.31337700
H	-0.70084400	-1.99441100	-2.49711400
H	0.93619600	-1.65229800	-2.07038000
C	0.56855000	-3.73393900	-2.44234200
H	-6.70737700	-4.48750300	2.71579200
C	1.05324700	-3.75470100	-3.75665700
C	0.52274300	-4.93491700	-1.72846300
C	1.50330600	-4.93905400	-4.33707800
H	1.08633600	-2.82711500	-4.32174600
C	0.98042200	-6.12117300	-2.30366200
H	0.14376900	-4.94948300	-0.71479100
C	1.47593900	-6.12878200	-3.60742000
H	1.87844000	-4.93112900	-5.35685700
H	0.94851800	-7.03979900	-1.72495200
H	1.83273900	-7.05312200	-4.05275700
H	-5.22181400	-0.60884500	3.93721800
C	-4.90438200	0.02714800	3.11715200
C	-5.28627900	-0.27204100	1.83749100
C	-4.05481700	1.13764700	3.36693400
C	-4.90662000	0.55363200	0.73178800
H	-5.88669100	-1.15332800	1.65012300
C	-3.59518400	1.90248300	2.32821300
H	-3.73612100	1.35673500	4.38287000
C	-4.01993800	1.66946600	0.98102100
C	-5.36386100	0.29361200	-0.57901500
H	-2.89822400	2.70661100	2.52433200
C	-3.59991500	2.48460200	-0.09524700
C	-5.00079300	1.15572600	-1.63775100
C	-6.24317900	-0.87709800	-0.85376100
C	-2.57763700	3.54028700	0.14479100
C	-4.10772800	2.26153100	-1.39073100
C	-5.52280600	0.99205000	-2.96135200
C	-5.80260800	-1.90850800	-1.69518100
C	-7.52925400	-0.96829100	-0.30159000
C	-1.28341700	3.41565800	-0.43682500
C	-2.79862700	4.60799400	0.98815800
C	-3.78060800	3.12977800	-2.47861900
C	-5.17698600	1.84408700	-3.97492800
H	-6.21780600	0.18297200	-3.14974200
C	-6.62602100	-2.99612800	-1.98198000
H	-4.80429000	-1.85194000	-2.11623200
C	-8.34902900	-2.06197500	-0.57669200
H	-7.88403700	-0.17039000	0.34402900

C	-0.23024000	4.26354300	-0.14209600
O	-1.11305900	2.43999600	-1.39441600
C	-1.75669900	5.49301300	1.36183700
H	-3.78493600	4.74774900	1.42163900
C	-4.29080300	2.92833200	-3.73119000
H	-3.12099200	3.96863700	-2.29096000
H	-5.59154400	1.70061500	-4.96887100
C	-7.90134100	-3.07915100	-1.42125800
H	-6.26951000	-3.78182800	-2.64301900
H	-9.34237300	-2.11405200	-0.13951800
C	-0.43894800	5.29546500	0.83543900
C	1.10094800	4.04784400	-0.77228100
P	-0.18516100	1.11643100	-1.16763100
C	-1.98547500	6.53801300	2.29568300
H	-4.02700700	3.60266700	-4.54072000
H	-8.54320600	-3.92701800	-1.64412200
C	0.60751100	6.12056500	1.32830300
C	1.71355400	2.80434900	-0.68447000
C	1.79147800	5.10063200	-1.46058700
O	1.00427800	1.72727100	-0.18027900
C	2.06304200	-3.09224200	0.13539100
O	0.30684000	0.64034000	-2.48373100
C	-0.95491600	7.33950000	2.72723800
H	-2.99444300	6.68032800	2.67461300
C	0.35611300	7.11464600	2.24714000
H	1.61826300	5.95700900	0.97602200
C	3.06880100	2.59157900	-1.05794000
C	3.13524300	4.88129600	-1.90260000
C	1.18039300	6.34750200	-1.76225200
H	-1.14065000	8.13248900	3.44586800
H	1.17488700	7.72924700	2.61044100
C	3.75385500	3.63265700	-1.64516700
C	3.78563600	1.32766700	-0.70651900
C	3.82482800	5.92132700	-2.57941500
C	1.87079400	7.33073200	-2.43383700
H	0.15131900	6.51310600	-1.46675800
H	4.79558000	3.49284100	-1.92060500
C	3.83972000	0.24058400	-1.59967100
C	4.48536000	1.27655300	0.51761100
C	3.21054600	7.12403200	-2.83832000
H	4.84831700	5.74140700	-2.89839800
H	1.37982700	8.27274400	-2.66087500
C	3.16737400	0.27357100	-2.86061800
C	4.59977800	-0.93852500	-1.25255000
C	4.48404100	2.37581800	1.43422500
C	5.26027700	0.10528900	0.85489500

H	3.74530700	7.91090900	-3.36230700
C	3.21336400	-0.79469000	-3.71553200
H	2.58133100	1.14571300	-3.11401400
C	4.61554900	-2.03209500	-2.17694500
C	5.29634200	-0.99186700	-0.02880300
C	5.22845300	2.34659100	2.58204600
H	3.88428800	3.24669600	1.19836200
C	6.01480200	0.11383200	2.07132800
C	3.94306400	-1.96487400	-3.36817900
H	2.67966100	-0.74998200	-4.66014600
H	5.18145700	-2.92050300	-1.91929400
C	6.08424300	-2.20977800	0.31759800
C	6.01193000	1.20292100	2.90137900
H	5.21773500	3.19723800	3.25752300
H	6.61020100	-0.75842600	2.31597200
H	3.95913700	-2.81156100	-4.04750400
C	7.32477500	-2.45668200	-0.28714500
C	5.58371100	-3.14216100	1.23492400
H	6.60640900	1.19417600	3.81085200
C	8.04522900	-3.61135400	0.01685800
H	7.71694300	-1.73717600	-1.00016600
C	6.29854500	-4.30270500	1.53333700
H	4.62112200	-2.96085500	1.70008800
C	7.53199800	-4.53935800	0.92552300
H	9.00605700	-3.78832600	-0.45860200
H	5.88166700	-5.02308400	2.23047500
H	8.09010600	-5.44231500	1.15659300

TS10-S3

N	-0.88151700	-2.57859300	0.06505300
H	-0.63376600	-1.28603500	0.01293800
O	-0.50945000	-0.13540000	0.29769300
H	-0.66198700	-2.65115700	-1.99964200
C	1.74532200	-2.45777800	-0.13469700
C	1.82213500	-2.90094800	-2.46285800
C	1.74588400	-4.78619600	-0.87707000
C	-0.41285700	-5.82124500	-1.17715600
H	-2.07924500	-4.64741200	-1.71207200
C	2.57011700	-1.55378200	-0.70526000
C	1.23322600	-2.70084400	1.20711600
C	2.64652700	-1.80152700	-2.14867700
C	1.70277500	-3.37061500	-3.76069400
C	1.00476700	-5.89660200	-0.84632500
H	2.81227600	-4.81251300	-0.67275500
O	-0.99732700	-7.04673200	-1.31750600
H	3.06454300	-0.73893800	-0.19306700

C	2.04751600	-2.93160400	2.35345800
C	-0.14491200	-2.88378800	1.28403800
C	3.34775400	-1.14872200	-3.15921700
C	2.41130000	-2.71152400	-4.77366000
H	1.07093700	-4.22563900	-3.98558400
H	1.42501100	-6.87343200	-0.63081500
H	-1.93054600	-6.91923500	-1.55320700
C	3.46492200	-2.91372900	2.29148300
C	1.41421600	-3.25326500	3.59749100
C	1.18291700	-3.41519700	-1.18800600
C	-0.76590300	-3.21091100	2.50676900
C	3.22216400	-1.61382600	-4.47019300
H	3.96274200	-0.28987200	-2.94218300
H	2.32586200	-3.05350900	-5.80126400
C	4.22160700	-3.17797900	3.40942300
H	3.94763700	-2.70213300	1.34559700
C	2.22465200	-3.48243700	4.74096900
C	0.00292000	-3.37189100	3.63893000
C	-0.37366800	-3.32737300	-1.19379200
C	-2.36692200	-2.65051100	0.20980000
H	-1.84071900	-3.31437700	2.57251500
H	3.76140600	-1.10592400	-5.26503900
C	3.59751200	-3.44688700	4.65131300
H	5.30553700	-3.18053400	3.33406700
H	1.73805000	-3.70944100	5.68635700
H	-0.47474900	-3.61672500	4.58400800
H	-2.64773600	-3.65798200	0.53052400
H	-2.62000800	-1.95484200	1.01046800
C	-3.12658200	-2.28741300	-1.04655200
H	4.20617500	-3.64091900	5.53004600
C	-2.96283400	-1.04755100	-1.67220400
C	-4.05758900	-3.18604100	-1.58026100
C	-3.68658700	-0.72727100	-2.81723500
H	-2.30085800	-0.31355500	-1.24243700
C	-4.80036500	-2.86209600	-2.71622100
H	-4.21008900	-4.14711700	-1.09313700
C	-4.60803400	-1.63229700	-3.34382800
H	-3.54953600	0.24750300	-3.27270400
H	-5.52982700	-3.56776000	-3.10354000
H	-5.18560100	-1.37138200	-4.22545400
H	-7.16572200	1.75886900	-3.60136600
C	-6.49636000	1.71388800	-2.74683900
C	-6.66511100	0.74304000	-1.79674900
C	-5.44243300	2.66173400	-2.62815900
C	-5.78998200	0.64423100	-0.66894100
H	-7.45855400	0.01289800	-1.90104500

C	-4.57900900	2.60295500	-1.56766200
H	-5.32008800	3.42943000	-3.38686100
C	-4.70418800	1.59032700	-0.56602900
C	-5.95920100	-0.34919700	0.32001600
H	-3.76873100	3.31786400	-1.48256800
C	-3.80083100	1.50524900	0.50900500
C	-5.00624100	-0.47996000	1.35482000
C	-7.17098900	-1.21667800	0.29501700
C	-2.85720800	2.62565700	0.79344700
C	-3.90094500	0.44738000	1.43452500
C	-5.11135800	-1.48599900	2.36876700
C	-8.43855600	-0.64737800	0.49123300
C	-7.08551000	-2.60017600	0.08839400
C	-1.62785100	2.83934200	0.11453500
C	-3.21284200	3.50878000	1.79229900
C	-2.92799200	0.27138100	2.46992800
C	-4.16821100	-1.60718900	3.35563900
H	-5.96058600	-2.15819000	2.34701800
C	-9.58692400	-1.43752100	0.47841800
H	-8.51478200	0.42350300	0.65529200
C	-8.23315200	-3.39213400	0.07504500
H	-6.11170000	-3.04507700	-0.07471500
C	-0.79285400	3.90445400	0.41792300
O	-1.28079500	1.98270600	-0.91437100
C	-2.37597100	4.57620400	2.18880600
H	-4.15507300	3.35962300	2.31196800
C	-3.05002500	-0.72860900	3.39687200
H	-2.06762500	0.92816000	2.48631400
H	-4.27314000	-2.38079100	4.11174800
C	-9.48815800	-2.81408600	0.26962500
H	-10.55895600	-0.97787300	0.63463900
H	-8.14649200	-4.46259500	-0.09161000
C	-1.12849500	4.76842900	1.51597500
C	0.49979200	4.08250400	-0.28985500
P	-0.02960700	0.90840900	-0.73380200
C	-2.73576600	5.43106500	3.26426400
H	-2.28560900	-0.85996200	4.15638100
H	-10.38236300	-3.43093600	0.25877800
C	-0.25986200	5.78323800	2.00567300
C	1.42029400	3.04764800	-0.28421000
C	0.87045300	5.33134400	-0.88605200
O	1.02987900	1.79998100	0.16678200
C	-1.04904500	-4.65234000	-1.37587900
O	0.45452100	0.48809600	-2.06501600
C	-1.88561800	6.42290200	3.69079100
H	-3.69448000	5.27286400	3.75153500

C	-0.62956300	6.58603500	3.06082600
H	0.71052800	5.91941600	1.54659500
C	2.79465500	3.25150900	-0.60339300
C	2.22914400	5.53203000	-1.28536200
C	-0.06222100	6.37865600	-1.10704400
H	-2.16601300	7.06935900	4.51732100
H	0.05537500	7.35005800	3.41748200
C	3.16920200	4.49359500	-1.07608000
C	3.80272900	2.19369300	-0.33268900
C	2.60846300	6.77516800	-1.85686000
C	0.33326900	7.56707800	-1.67825900
H	-1.09836500	6.22468700	-0.82781500
H	4.21593800	4.68247500	-1.29560100
C	4.64000500	1.71321100	-1.36327600
C	3.94693600	1.70033500	0.98490700
C	1.68285300	7.77402100	-2.04991600
H	3.64625000	6.91720000	-2.14759000
H	-0.39761100	8.35232700	-1.84885800
C	4.55290500	2.19651100	-2.70891900
C	5.60899600	0.67988800	-1.07548000
C	3.20279600	2.23884400	2.08200000
C	4.85946000	0.61397900	1.25136800
H	1.98210700	8.71886900	-2.49422400
C	5.41979300	1.76963500	-3.67949800
H	3.77335800	2.90567800	-2.95608200
C	6.49751300	0.26821800	-2.12005700
C	5.66186500	0.09872200	0.20881100
C	3.28620100	1.70135300	3.33700400
H	2.55872500	3.09190000	1.90973700
C	4.89403200	0.06839200	2.57347600
C	6.42196700	0.80809500	-3.37571400
H	5.33232400	2.15679000	-4.69053500
H	7.24623500	-0.48194400	-1.89422500
C	6.49818600	-1.11107700	0.44364600
C	4.12675300	0.58353300	3.58140900
H	2.70086000	2.12604700	4.14731000
H	5.53815700	-0.77693200	2.76868000
H	7.11474900	0.48733900	-4.14827800
C	6.16205300	-2.31486100	-0.19685500
C	7.58947200	-1.09884500	1.32272200
H	4.15876900	0.13274000	4.56885000
C	6.88388500	-3.48032700	0.05658900
H	5.32484000	-2.32987300	-0.88871900
C	8.31478000	-2.26403100	1.57307900
H	7.86207800	-0.16944400	1.81413400
C	7.95988500	-3.45972000	0.94612700

H	6.60359500	-4.40528000	-0.43971000
H	9.15796700	-2.23708400	2.25779700
H	8.52156400	-4.36801100	1.14508800

TS10-S4

N	1.17024500	0.81816600	-1.52730800
H	0.22297800	0.23263700	-0.97158300
O	-0.69259600	-0.54531900	-0.65576300
H	-0.46859300	0.56854500	-2.81461500
C	-0.16882600	3.22147700	-1.84302900
C	-1.03456200	2.80992600	-4.01004400
C	1.39355100	3.28192300	-3.80217600
C	2.16712600	1.17889800	-4.73349600
H	1.18623300	-0.62025200	-4.19752000
C	-1.41805100	3.73669900	-1.90143400
C	0.82430200	3.14496600	-0.78968100
C	-1.98154000	3.51092900	-3.23174500
C	-1.29957700	2.47198200	-5.32773400
C	2.29056800	2.62245000	-4.54446500
H	1.44823000	4.35786600	-3.66722400
O	3.04898200	0.66814400	-5.63094200
H	-1.94155700	4.20956500	-1.07858200
C	1.18190600	4.26654000	0.03165200
C	1.53710500	1.95386100	-0.68941700
C	-3.21896600	3.85668100	-3.77426400
C	-2.54526300	2.81957700	-5.86867500
H	-0.56193000	1.94256800	-5.92220600
H	3.11844200	3.12181100	-5.03675400
H	3.12049400	-0.29027000	-5.48645800
C	0.61038500	5.55400900	-0.13772300
C	2.21098200	4.11012000	1.01171300
C	0.21542200	2.57179100	-3.16680700
C	2.52892100	1.80513000	0.30629800
C	-3.49213100	3.50190700	-5.09803200
H	-3.95877200	4.38375700	-3.17886300
H	-2.77753400	2.55351900	-6.89562200
C	1.02552600	6.62032800	0.62858500
H	-0.14552800	5.70022700	-0.89881600
C	2.60291700	5.22107200	1.80025000
C	2.84230000	2.84990900	1.13717800
C	0.49291500	1.07277900	-2.92792800
C	2.12728300	-0.33385000	-1.53835900
H	3.06554400	0.87867700	0.42344600
H	-4.45387800	3.75721200	-5.53331700
C	2.02527000	6.45567300	1.61386300
H	0.58109500	7.59909000	0.47204000

H	3.38285200	5.07933100	2.54412600
H	3.61335800	2.71508000	1.88872000
H	2.27858400	-0.61387800	-0.49505900
H	1.56453500	-1.16242600	-1.97082200
C	3.45640600	-0.20133800	-2.24627300
H	2.33983400	7.30485800	2.21377500
C	4.22969100	0.96297500	-2.28374800
C	3.96528300	-1.35930300	-2.84718000
C	5.47584200	0.96214700	-2.91062200
H	3.86037600	1.88046400	-1.84486700
C	5.22078700	-1.37018600	-3.45327600
H	3.38195700	-2.27385300	-2.81854400
C	5.98268600	-0.20142800	-3.48970700
H	6.05588500	1.88038600	-2.93999100
H	5.59905700	-2.29460700	-3.88087200
H	6.95846000	-0.19643300	-3.96673100
H	7.04050500	0.25526300	-0.14714800
C	6.23560300	-0.18736900	0.43113900
C	5.77838000	-1.43759900	0.11203100
C	5.65059000	0.54009400	1.50239500
C	4.71072400	-2.05597100	0.83928100
H	6.21957700	-1.96883400	-0.72038300
C	4.58401300	0.01689500	2.18312100
H	6.03739900	1.52123200	1.76387900
C	4.05853100	-1.27586200	1.86681300
C	4.25010800	-3.36358900	0.55623300
H	4.11059900	0.57877600	2.98065500
C	2.91180100	-1.78203100	2.50871000
C	3.19564300	-3.92067400	1.31618700
C	4.83973700	-4.13920200	-0.56850900
C	2.04365000	-0.87369000	3.31197600
C	2.49927600	-3.10807200	2.28446800
C	2.78435700	-5.28223000	1.16454600
C	4.02362400	-4.55105400	-1.63487000
C	6.20412400	-4.45941800	-0.61327300
C	0.93878400	-0.26623000	2.64716500
C	2.23836600	-0.59826400	4.64476100
C	1.40235500	-3.67765600	3.00068200
C	1.74265300	-5.79493600	1.89013200
H	3.32522500	-5.91639600	0.47237000
C	4.55962200	-5.23375100	-2.72525700
H	2.96117000	-4.32809900	-1.59680600
C	6.74332600	-5.14159700	-1.70389800
H	6.84253600	-4.16857700	0.21549500
C	0.06022300	0.60071800	3.27726400
O	0.81463600	-0.52393000	1.30048100

C	1.33743200	0.22745300	5.36639800
H	3.07828800	-1.04766800	5.16769400
C	1.03487100	-4.98044800	2.81334400
H	0.86412300	-3.05407600	3.70495400
H	1.45479000	-6.83450100	1.76093400
C	5.92501400	-5.52489600	-2.76744800
H	3.91061700	-5.54034400	-3.54115600
H	7.80354400	-5.37873900	-1.71944300
C	0.21627100	0.81112000	4.69045500
C	-1.05565000	1.22176100	2.50982800
P	-0.47227900	-1.36007100	0.63740200
C	1.50735900	0.45255500	6.75744400
H	0.19726800	-5.39426600	3.36637500
H	6.34501700	-6.05689400	-3.61645900
C	-0.71982700	1.54773000	5.46559600
C	-1.91700700	0.40712400	1.79452600
C	-1.28553700	2.63859500	2.47725500
O	-1.65223500	-0.94898200	1.70815700
C	1.26964000	0.45043700	-4.04215700
O	-0.29086000	-2.82283300	0.59233500
C	0.59767000	1.19714400	7.47117300
H	2.36876700	0.00931200	7.25077800
C	-0.53337800	1.73670100	6.81649100
H	-1.59689500	1.95811600	4.98088700
C	-3.08661400	0.88072400	1.14079600
C	-2.43440600	3.14088200	1.78265500
C	-0.40973600	3.57504900	3.08706700
H	0.73577300	1.35778600	8.53645900
H	-1.26725400	2.29987900	7.38597700
C	-3.32419400	2.23578000	1.14944900
C	-4.04715200	-0.09506300	0.55111000
C	-2.67616800	4.54001100	1.75987400
C	-0.67938500	4.92367000	3.05760100
H	0.48666100	3.21445300	3.57544200
H	-4.21719700	2.62337600	0.66655600
C	-4.82985900	-0.88089100	1.42433000
C	-4.17311900	-0.23868800	-0.84362100
C	-1.82340900	5.41550800	2.39014700
H	-3.55892900	4.90309800	1.23858400
H	0.00985500	5.61761500	3.52535900
C	-4.74040500	-0.74935100	2.84579500
C	-5.75365700	-1.84952500	0.88578800
C	-3.38683200	0.52847900	-1.76007500
C	-5.11446200	-1.19537600	-1.38114100
H	-2.01783700	6.48372700	2.36677300
C	-5.48837600	-1.53196600	3.68331700

H	-4.06166700	-0.01308500	3.26003300
C	-6.50613300	-2.65545800	1.79787200
C	-5.88428100	-1.99203400	-0.51098000
C	-3.53056200	0.38223400	-3.11282300
H	-2.65647800	1.21918100	-1.35918700
C	-5.24333700	-1.30024700	-2.80315300
C	-6.37898000	-2.50413100	3.15235300
H	-5.39809800	-1.41462800	4.75956400
H	-7.18309800	-3.39900900	1.39351200
C	-6.83308300	-2.99836100	-1.06927300
C	-4.47756600	-0.53735700	-3.64102800
H	-2.92459900	0.96907400	-3.79102300
H	-5.96546300	-2.00044500	-3.20676400
H	-6.95833700	-3.12915700	3.82617200
C	-6.35667300	-4.20270600	-1.60502900
C	-8.21396600	-2.76005200	-1.06764400
H	-4.58965600	-0.63005200	-4.71793300
C	-7.24066600	-5.14656600	-2.12730400
H	-5.28713900	-4.39175500	-1.60615300
C	-9.09911400	-3.70318200	-1.58977700
H	-8.58783300	-1.82817600	-0.65307600
C	-8.61443500	-4.89924100	-2.12126500
H	-6.85608400	-6.07668400	-2.53678300
H	-10.16714300	-3.50295300	-1.58265100
H	-9.30328700	-5.63456500	-2.52777200

Int11

O	-0.92461300	-0.15289900	1.35691500
H	-0.05225800	0.28815100	1.12559400
C	1.30404200	1.04982700	-0.01430400
O	-1.63904900	0.81171800	-0.97346000
H	-0.70429100	2.25432900	-0.80693800
O	-0.09324100	2.95062100	-0.45291000
C	3.09480300	2.90964600	1.18850200
C	1.02372700	2.38379100	0.02245400
H	0.71987900	0.42924700	-0.68062900
C	5.28374500	0.69555900	2.58063200
C	5.56725000	1.28077700	0.09044100
C	4.15042500	0.64598600	3.49458600
C	1.76126200	1.11217300	3.44179900
C	1.97367500	3.33681700	0.59273900
H	3.79926200	3.60785700	1.63105700
C	2.98873000	1.04965600	2.80064900
H	6.28272000	0.36467100	2.83791200
C	6.94439900	1.65946400	-0.04028100
C	4.80046900	0.99196800	-1.05911200

C	4.07426700	0.28643900	4.84239600
C	1.68581000	0.73823000	4.78976300
H	0.87380300	1.45330500	2.91957900
H	1.72897300	4.39134100	0.51226900
C	4.88394700	1.14091300	1.35992400
C	7.74849800	2.04774700	1.06533700
C	7.55355800	1.67414200	-1.33658300
C	3.38395100	1.43507400	1.37777000
C	5.41976300	1.07652000	-2.34312800
C	2.83203400	0.33187600	5.48056900
H	4.96494100	-0.01714800	5.38631800
H	0.72857600	0.77173000	5.30150500
C	9.07831700	2.37404700	0.90453500
H	7.29726600	2.11582700	2.04716400
C	8.92375400	2.00703300	-1.46959700
C	6.74810600	1.38095600	-2.46772200
C	2.70372900	0.57791400	0.29430800
C	2.88079600	-0.22784100	-2.02572600
H	4.82686200	0.90508900	-3.23323200
H	2.75705900	0.05565800	6.52893500
C	9.68416500	2.34005100	-0.37096600
H	9.66410900	2.67197700	1.77003000
H	9.36198700	2.00272700	-2.46493400
H	7.20016600	1.42158900	-3.45552900
H	3.67739500	-0.69680700	-2.61124100
H	2.35612300	-1.05977400	-1.54660900
C	1.90247800	0.50974900	-2.92075200
H	10.73432200	2.59305500	-0.48294500
C	2.04726300	1.87280300	-3.19440700
C	0.79878800	-0.17654500	-3.43892500
C	1.10444600	2.53925100	-3.97610400
H	2.87355000	2.42029700	-2.75180300
C	-0.14585700	0.49038600	-4.21767800
H	0.65863900	-1.22789700	-3.20220700
C	0.00639000	1.84951800	-4.49191100
H	1.21757000	3.60297800	-4.16649400
H	-1.01427300	-0.04747000	-4.58702500
H	-0.73588100	2.37341200	-5.08585400
H	3.62545100	-2.42800300	4.18182500
C	2.93042300	-2.62906100	3.37468500
C	3.40023000	-2.92441900	2.12442700
C	1.53301300	-2.55510900	3.61952700
C	2.50838300	-3.16559200	1.03018500
H	4.46693400	-2.96933900	1.94562200
C	0.64247900	-2.83024600	2.61878400
H	1.17658800	-2.27092600	4.60382000

C	1.08763200	-3.16855400	1.30103200
C	2.97830400	-3.40866400	-0.28038500
H	-0.42239200	-2.76983700	2.80586800
C	0.17732800	-3.49033200	0.27361500
C	2.05282400	-3.63209800	-1.32778200
C	4.43980900	-3.39638600	-0.57175000
C	-1.26470900	-3.67955000	0.60645400
C	0.63768800	-3.69842400	-1.04246300
C	2.47050000	-3.76012900	-2.69189700
C	5.08199500	-4.53473600	-1.08243600
C	5.20286800	-2.23917000	-0.35926900
C	-2.26235200	-2.76792600	0.17892300
C	-1.67150900	-4.76629600	1.35236700
C	-0.26320500	-3.96864600	-2.12027400
C	1.56932000	-3.97916800	-3.69903300
H	3.52435300	-3.66234800	-2.92163900
C	6.44308400	-4.50750700	-1.38455500
H	4.50565700	-5.44143800	-1.24221700
C	6.56068600	-2.20447200	-0.67059900
H	4.72845700	-1.35765400	0.04777900
C	-3.61492000	-2.92798800	0.43981100
O	-1.82970000	-1.67299200	-0.57036700
C	-3.02471300	-4.95264600	1.72578900
H	-0.92824300	-5.48371100	1.68779800
C	0.18334200	-4.10177500	-3.40724800
H	-1.32179200	-4.05365800	-1.90446500
H	1.91241300	-4.05604100	-4.72661100
C	7.18496700	-3.34106600	-1.18604300
H	6.92431600	-5.39926400	-1.77684100
H	7.11962800	-1.28670600	-0.51449300
C	-4.01455400	-4.01559200	1.28579000
C	-4.59669500	-1.95960100	-0.11984800
P	-1.87845000	-0.19053800	0.09158600
C	-3.41444700	-6.04027800	2.55186900
H	-0.52274800	-4.29050100	-4.21069100
H	8.24328000	-3.31942900	-1.43043900
C	-5.34894500	-4.18146400	1.74495900
C	-4.40924400	-0.60641700	0.08335700
C	-5.70884400	-2.36626300	-0.93264500
O	-3.30777500	-0.18763000	0.83784700
N	3.48844100	0.57599200	-0.96824800
H	2.66781500	-0.45398900	0.66844800
C	-4.71998900	-6.18663000	2.95641200
H	-2.65291300	-6.74825400	2.86801900
C	-5.69122100	-5.23848000	2.55710700
H	-6.10010800	-3.45686800	1.45538900

C	-5.26028800	0.40646200	-0.42936000
C	-6.62055500	-1.36725200	-1.40835800
C	-5.92508900	-3.71460400	-1.32336500
H	-5.00624500	-7.01889800	3.59261300
H	-6.71644500	-5.34198000	2.90039900
C	-6.37134500	0.00293700	-1.13509900
C	-4.85269800	1.82961900	-0.26181300
C	-7.73514400	-1.76324800	-2.19378200
C	-7.00844200	-4.06273500	-2.09817800
H	-5.22064800	-4.47452100	-1.00834600
H	-7.04809700	0.75242600	-1.53545800
C	-4.96485300	2.44992500	0.99655500
C	-4.22500000	2.49156700	-1.33602200
C	-7.93248200	-3.08239200	-2.52798100
H	-8.42333300	-0.99569900	-2.53796600
H	-7.15125300	-5.10002900	-2.38680200
C	-5.60845300	1.80158700	2.09593700
C	-4.39930500	3.76097500	1.20322600
C	-4.07864300	1.88495900	-2.62357900
C	-3.66290400	3.80587100	-1.12958600
H	-8.78636800	-3.37198400	-3.13331100
C	-5.68322100	2.39570200	3.32657400
H	-6.04441900	0.82122000	1.93580000
C	-4.48662200	4.33668500	2.51055500
C	-3.74405000	4.41717400	0.13904300
C	-3.44581200	2.53342000	-3.64705200
H	-4.46590300	0.88482800	-2.77500500
C	-3.01927000	4.45054000	-2.23398400
C	-5.10849400	3.67902500	3.53794400
H	-6.17775200	1.88509000	4.14810400
H	-4.04226100	5.31066900	2.67781700
C	-3.10230900	5.74376400	0.36588200
C	-2.91309400	3.83684300	-3.45195200
H	-3.34179900	2.04787500	-4.61308500
H	-2.61018100	5.44266800	-2.08641600
H	-5.16076600	4.13593400	4.52213300
C	-1.70425600	5.84730300	0.41158000
C	-3.87587300	6.89907700	0.53996800
H	-2.41664300	4.34496100	-4.27369200
C	-1.09731200	7.08365300	0.63024900
H	-1.10468000	4.95352800	0.26770600
C	-3.26648500	8.13477700	0.75820200
H	-4.95887900	6.82204300	0.50152900
C	-1.87484500	8.22989600	0.80505700
H	-0.01302500	7.15174000	0.66486700
H	-3.87876700	9.02287000	0.88916100

H	-1.39953100	9.19202200	0.97517200

TS12

O	-0.77862100	-0.21866800	1.27749200
H	0.23305600	0.32062400	0.87967600
C	1.21904000	0.90552000	0.02965100
O	-1.65355800	0.79952100	-0.95792400
H	-0.76148700	2.10878100	-0.76422600
O	-0.12964200	2.82586700	-0.41991700
C	2.96973700	2.73880100	1.33906400
C	0.96167300	2.28383100	0.06624800
H	0.75940300	0.41642800	-0.82397600
C	5.17747700	0.52707900	2.65485800
C	5.49223300	1.24742000	0.20084800
C	4.02992300	0.40899200	3.54288400
C	1.62659000	0.80438700	3.46275200
C	1.87570500	3.20611600	0.71339200
H	3.63938800	3.41552800	1.86286100
C	2.86819800	0.80856000	2.84539500
H	6.17913200	0.21029500	2.91847100
C	6.86534700	1.65854600	0.12262700
C	4.75122700	1.02499400	-0.97999000
C	3.94130500	-0.01151200	4.87178600
C	1.54199500	0.37314000	4.79271900
H	0.73110500	1.11838200	2.93889700
H	1.62281400	4.26120600	0.69836900
C	4.79066500	1.01616700	1.44655900
C	7.64059700	1.99182700	1.26586000
C	7.49822800	1.76883300	-1.15714100
C	3.28274300	1.26569900	1.44768500
C	5.39432700	1.20547800	-2.24251100
C	2.68766300	-0.02633400	5.48807000
H	4.83080400	-0.31481200	5.41751200
H	0.57443300	0.35131400	5.28490500
C	8.96640100	2.35316700	1.15253800
H	7.17091600	1.99128300	2.24110100
C	8.86392700	2.13421100	-1.24205400
C	6.71921500	1.53808200	-2.32051900
C	2.64522900	0.46382200	0.30086600
C	2.86978700	-0.14053900	-2.07864100
H	4.82409600	1.08347900	-3.15485900
H	2.60237400	-0.34907700	6.52221700
C	9.59703000	2.41061600	-0.10975400
H	9.52923400	2.60742500	2.04666000
H	9.32024400	2.20164700	-2.22685700
H	7.18855000	1.65046800	-3.29454100

H	3.68668400	-0.54800100	-2.68081100
H	2.35149300	-1.01646300	-1.67843700
C	1.89592600	0.65744800	-2.92626600
H	10.64391900	2.68935800	-0.18445900
C	2.05962100	2.03220200	-3.12366700
C	0.76612300	0.02281900	-3.45495400
C	1.10769100	2.76087900	-3.83642600
H	2.90900900	2.53941800	-2.67640700
C	-0.19009300	0.75338200	-4.15897900
H	0.61059300	-1.03755800	-3.27492800
C	-0.02097100	2.12410000	-4.35387000
H	1.23626500	3.83181600	-3.96775500
H	-1.08294400	0.25610100	-4.52582400
H	-0.77596100	2.69737700	-4.88180700
H	3.70527200	-2.77325000	4.01755300
C	2.98989900	-2.90831600	3.21373000
C	3.42888300	-3.11925300	1.93537700
C	1.59911700	-2.83802200	3.49448200
C	2.50994100	-3.27688900	0.84804700
H	4.49083300	-3.16242400	1.72939000
C	0.68412300	-3.03230600	2.49721300
H	1.26679400	-2.61734200	4.50296500
C	1.09572200	-3.28853400	1.15062000
C	2.94819800	-3.42871800	-0.48704300
H	-0.37524200	-2.96661100	2.71025300
C	0.15874400	-3.53649400	0.12600900
C	1.99752800	-3.57975100	-1.52473000
C	4.40261800	-3.39360100	-0.81089600
C	-1.27788100	-3.72594500	0.48114600
C	0.58904000	-3.66119300	-1.21077700
C	2.38231100	-3.61941500	-2.90417600
C	5.03402700	-4.48943100	-1.41865300
C	5.17085900	-2.25389100	-0.53131300
C	-2.27189300	-2.78392700	0.11093700
C	-1.67999200	-4.83190700	1.20098000
C	-0.33746200	-3.85984400	-2.28245500
C	1.45691300	-3.77128900	-3.90165200
H	3.43074800	-3.50938300	-3.15269300
C	6.38765400	-4.43681300	-1.74994400
H	4.45479300	-5.38333000	-1.63109800
C	6.52101100	-2.19297400	-0.87049400
H	4.70637100	-1.40675100	-0.04688000
C	-3.61768500	-2.92970400	0.41593900
O	-1.85139600	-1.67910300	-0.61876800
C	-3.02436700	-5.00890700	1.60970000
H	-0.93947700	-5.57156500	1.49150000

C	0.07772900	-3.91039700	-3.58561300
H	-1.39057400	-3.95689800	-2.04707400
H	1.77574700	-3.78283700	-4.93989900
C	7.13371500	-3.28676800	-1.48342100
H	6.85995400	-5.29622700	-2.21796300
H	7.08355900	-1.28847500	-0.65888800
C	-4.00961600	-4.03806400	1.23730900
C	-4.59399400	-1.92060600	-0.07851200
P	-1.80863300	-0.22201900	0.12354200
C	-3.40801000	-6.11845700	2.40908400
H	-0.64782100	-4.04640200	-4.38232400
H	8.18608400	-3.24513100	-1.74993100
C	-5.33060700	-4.19558200	1.73667900
C	-4.35896300	-0.57853900	0.15740000
C	-5.73923500	-2.27474500	-0.86922200
O	-3.23669700	-0.21358800	0.89734100
N	3.43985500	0.59226300	-0.94685400
H	2.63860900	-0.59260300	0.59715600
C	-4.70189800	-6.25536100	2.85277900
H	-2.65044400	-6.85160000	2.67355200
C	-5.66677500	-5.27498700	2.52154500
H	-6.07607600	-3.44657300	1.49874300
C	-5.19106800	0.46935900	-0.31794500
C	-6.63457800	-1.23893200	-1.29448100
C	-6.00620800	-3.60574600	-1.28799500
H	-4.98309900	-7.10500000	3.46804800
H	-6.68183800	-5.37166200	2.89586400
C	-6.33354100	0.11601100	-1.00073500
C	-4.73860800	1.87908600	-0.15195000
C	-7.78293600	-1.58272000	-2.05557800
C	-7.12145700	-3.90324200	-2.03834000
H	-5.31522500	-4.39323500	-1.01369800
H	-6.99520800	0.89468500	-1.36966300
C	-4.74845800	2.48010300	1.12104400
C	-4.19131900	2.55778800	-1.26014100
C	-8.02910900	-2.88667700	-2.41588900
H	-8.45761500	-0.78695300	-2.36052100
H	-7.30244700	-4.92847200	-2.34818200
C	-5.31172800	1.81805600	2.25581500
C	-4.15935500	3.78413700	1.30563100
C	-4.13255800	1.96771700	-2.56265000
C	-3.62562900	3.87447000	-1.08018900
H	-8.90856800	-3.13633400	-3.00221500
C	-5.28142300	2.38791900	3.49946100
H	-5.76887500	0.84499800	2.11263500
C	-4.13250900	4.33320200	2.62719500

C	-3.59921200	4.46247300	0.20191200
C	-3.60084800	2.64191900	-3.62662700
H	-4.50579100	0.95986100	-2.69388600
C	-3.09568300	4.54917600	-2.22657700
C	-4.67534000	3.66023500	3.68873800
H	-5.71449100	1.86576000	4.34793500
H	-3.66575800	5.29948100	2.77743000
C	-2.94880400	5.78992300	0.39481100
C	-3.08703300	3.95696500	-3.45965700
H	-3.56544900	2.16946000	-4.60433400
H	-2.69843100	5.54913700	-2.10049200
H	-4.64141600	4.09708900	4.68286000
C	-1.55512600	5.91122700	0.29146500
C	-3.70858400	6.93213700	0.68088700
H	-2.68285800	4.49121700	-4.31507400
C	-0.93862900	7.14867000	0.47321200
H	-0.96803700	5.02695000	0.06389100
C	-3.09060100	8.16939400	0.86314800
H	-4.78852600	6.84328200	0.75786300
C	-1.70323000	8.28086500	0.76066200
H	0.14224800	7.22972000	0.39043500
H	-3.69345100	9.04653100	1.08201200
H	-1.22142300	9.24437300	0.90201600

TS12-S1

O	-0.83133100	-0.07427600	1.17543100
H	0.20205800	0.39341700	0.75279100
C	1.22892800	0.86483300	-0.12078100
O	-1.63050000	0.85310100	-1.12469900
H	-0.65807100	2.13301600	-1.02711300
O	0.01282200	2.83004000	-0.72728800
C	3.08556200	2.67882200	1.06721600
C	1.06150500	2.25557600	-0.19005800
H	0.74965400	0.34493700	-0.94513800
C	5.12408100	0.43964300	2.59194600
C	5.51097000	0.92342700	0.09104300
C	3.96129300	0.46959800	3.46766800
C	1.58974100	1.00930500	3.31441800
C	2.02928900	3.16642600	0.39410800
H	3.79822700	3.34853600	1.54109000
C	2.83675600	0.88623200	2.72083500
H	6.09928500	0.07970800	2.89634800
C	6.90976400	1.23205300	-0.00192600
C	4.76867200	0.64589300	-1.07697700
C	3.82887700	0.16285000	4.82386900
C	1.46016600	0.69004100	4.67209200

H	0.72272300	1.33418600	2.75056300
H	1.84328300	4.22991400	0.28635100
C	4.78335400	0.85048200	1.34129200
C	7.69489300	1.61302500	1.11940900
C	7.56097300	1.18344700	-1.27632400
C	3.29641000	1.20091000	1.29835400
C	5.43414500	0.66794600	-2.34040000
C	2.56892900	0.27571900	5.41689500
H	4.68972200	-0.15261300	5.40735000
H	0.48671600	0.76711400	5.14683300
C	9.04350400	1.87205700	0.99546200
H	7.21660700	1.73113400	2.08331800
C	8.94926400	1.44669400	-1.37166200
C	6.77946100	0.90172600	-2.42691100
C	2.61591500	0.35017200	0.21256300
C	2.82904400	-0.50216400	-2.08750600
H	4.86409900	0.50413600	-3.24651900
H	2.44966900	0.04123800	6.47125900
C	9.68859200	1.77345300	-0.25692100
H	9.61395900	2.16651800	1.87225600
H	9.41880900	1.39438300	-2.35115700
H	7.26502900	0.89434800	-3.39943500
H	3.62652300	-1.01751400	-2.63036100
H	2.25361200	-1.29982300	-1.60847400
C	1.91592000	0.25534200	-3.03393900
H	10.75283600	1.97300700	-0.33962700
C	2.14267700	1.59881400	-3.34839200
C	0.78263300	-0.38372600	-3.54960000
C	1.25168800	2.29167800	-4.16781900
H	2.99388600	2.11291700	-2.91249000
C	-0.11057000	0.31041000	-4.36414700
H	0.57765400	-1.41626100	-3.28100400
C	0.12323400	1.64910600	-4.67862700
H	1.42906500	3.34019200	-4.39088600
H	-1.00303200	-0.18901200	-4.72944400
H	-0.57990000	2.19415800	-5.30070700
H	3.25012900	-2.59685300	4.23181500
C	2.55366600	-2.77239300	3.41941100
C	3.02027700	-3.12319200	2.18227800
C	1.16029600	-2.60935100	3.64340600
C	2.12864500	-3.33449400	1.08138500
H	4.08439700	-3.23853900	2.01970200
C	0.26777400	-2.84796600	2.63559800
H	0.80877400	-2.28372900	4.61656600
C	0.70700600	-3.24234600	1.33186200
C	2.59964300	-3.63531600	-0.21682200

H	-0.79289100	-2.71217500	2.80433800
C	-0.20937400	-3.52486400	0.29812400
C	1.67520200	-3.82650100	-1.27167600
C	4.06324500	-3.72173900	-0.48742900
C	-1.66789800	-3.58398400	0.61029800
C	0.25508800	-3.79249300	-1.00533300
C	2.10026300	-4.01967500	-2.62604500
C	4.63848000	-4.91053200	-0.96172700
C	4.89764900	-2.61138400	-0.29166100
C	-2.56620500	-2.58315700	0.15843800
C	-2.18255100	-4.61036700	1.37378400
C	-0.64841100	-4.02525600	-2.08952500
C	1.19892800	-4.20544000	-3.63991600
H	3.16106000	-4.00036100	-2.84391300
C	6.00212500	-4.97785600	-1.24583700
H	4.00732500	-5.78232400	-1.10778300
C	6.25875400	-2.67099700	-0.58448200
H	4.47754600	-1.69178800	0.09020700
C	-3.92583600	-2.58884900	0.43646800
O	-2.02972200	-1.56914000	-0.62314400
C	-3.54802400	-4.64839200	1.74886300
H	-1.51699500	-5.39270900	1.72727600
C	-0.19612000	-4.22317000	-3.36608400
H	-1.71270500	-4.02883000	-1.88558300
H	1.54949200	-4.33497200	-4.65976900
C	6.81530500	-3.85709600	-1.06480300
H	6.42963900	-5.90803200	-1.61014800
H	6.87399400	-1.78766000	-0.44095300
C	-4.43359300	-3.61436900	1.30223100
C	-4.79501200	-1.51071600	-0.11198500
P	-1.86281600	-0.07625200	0.02246800
C	-4.04722000	-5.67690600	2.59151100
H	-0.90315600	-4.38328800	-4.17488500
H	7.87580700	-3.90933400	-1.29466200
C	-5.77316000	-3.62763500	1.77649800
C	-4.43567400	-0.19009800	0.08162500
C	-5.97017000	-1.77702800	-0.89354200
O	-3.27871000	0.09284200	0.80200200
N	3.42989700	0.30868700	-1.02753700
H	2.52942000	-0.67290700	0.60066600
C	-5.35736600	-5.67578800	3.00771600
H	-3.36430200	-6.45950000	2.91173900
C	-6.22201700	-4.63121900	2.60453300
H	-6.44309500	-2.82816000	1.48497400
C	-5.17518200	0.91933300	-0.41154100
C	-6.76730800	-0.67432000	-1.34623400

C	-6.35981800	-3.08855000	-1.27476900
H	-5.72701000	-6.46448900	3.65643500
H	-7.24918500	-4.61699800	2.95747500
C	-6.34718100	0.65630000	-1.08298500
C	-4.57508300	2.27375600	-0.24793700
C	-7.94352600	-0.93209300	-2.09831000
C	-7.49902100	-3.30272700	-2.01769300
H	-5.74341900	-3.92800200	-0.97705700
H	-6.94237800	1.48181600	-1.46336200
C	-3.89934400	2.86256200	-1.33632300
C	-4.51856000	2.86069500	1.03056800
C	-8.30922200	-2.21734500	-2.42358900
H	-8.54350600	-0.08654600	-2.42477500
H	-7.77460200	-4.31497600	-2.29920500
C	-3.93607100	2.29692200	-2.64999600
C	-3.07199000	4.02651000	-1.11488600
C	-5.22698300	2.30571700	2.14072700
C	-3.71099100	4.03541600	1.24653200
H	-9.20902400	-2.40047200	-3.00358100
C	-3.21573000	2.83705100	-3.67839300
H	-4.53267900	1.40854200	-2.81625000
C	-2.31647100	4.54201200	-2.21632200
C	-2.96746000	4.58005200	0.17810100
C	-5.16268900	2.87698300	3.38280900
H	-5.83144100	1.41991900	1.97751800
C	-3.68156900	4.60636400	2.55838300
C	-2.38896200	3.97208500	-3.45746900
H	-3.25569000	2.38329000	-4.66454400
H	-1.66489500	5.38987600	-2.04472100
C	-2.01997400	5.70082500	0.43750500
C	-4.38288400	4.04730500	3.59302800
H	-5.71348500	2.44090200	4.21140700
H	-3.09397200	5.50267400	2.71997400
H	-1.80044000	4.37790800	-4.27524400
C	-0.84098400	5.45900300	1.15790600
C	-2.27104100	6.99783600	-0.02742300
H	-4.34919500	4.50097500	4.57958300
C	0.06646600	6.48814400	1.40284000
H	-0.64171200	4.45411800	1.51508200
C	-1.36265400	8.02904100	0.21607800
H	-3.18401600	7.19286600	-0.58282600
C	-0.19098300	7.77704000	0.93054500
H	0.97414400	6.28372800	1.96488800
H	-1.57264900	9.03007300	-0.15053400
H	0.51576300	8.57992200	1.12072500

TS12-S2

O	-0.66338700	-0.17412900	1.25805300
H	0.37318800	0.33443700	0.89398000
C	1.43438200	0.86358300	0.12413600
O	-1.38458800	0.75205700	-1.07133100
H	-0.44079300	2.04376800	-0.92332000
O	0.17043100	2.76879400	-0.56385700
C	3.02906000	2.73699300	1.56621300
C	1.19189900	2.24054600	0.07307900
H	1.05717900	0.33086300	-0.74318900
C	5.06351900	0.54811400	3.18877400
C	5.64184400	1.22065600	0.76855800
C	3.82015700	0.43203600	3.94017300
C	1.44504800	0.85964500	3.59133000
C	2.02721500	3.18524400	0.79071100
H	3.63295800	3.43037100	2.14535900
C	2.74844200	0.84866900	3.11837500
H	6.03173700	0.24121900	3.56652500
C	6.98955200	1.70770800	0.81751700
C	5.04553500	0.90315600	-0.46850300
C	3.57277900	-0.01545500	5.24033800
C	1.20199000	0.40848100	4.89507000
H	0.61962600	1.19010000	2.97180300
H	1.78366700	4.23955700	0.70827600
C	4.81302300	1.01825100	1.93896500
C	7.61462500	2.12797700	2.02194300
C	7.74803300	1.80671500	-0.39244800
C	3.31720100	1.26887300	1.76499400
C	5.81404900	1.05229700	-1.66269400
C	2.25511800	-0.02618600	5.70703100
H	4.38872300	-0.34256700	5.87908800
H	0.18446700	0.39458400	5.27354300
C	8.91963200	2.57261500	2.03293400
H	7.04355000	2.12217600	2.94224200
C	9.08830200	2.26213300	-0.34920000
C	7.11776400	1.46742400	-1.61807900
C	2.80649600	0.41139100	0.59085900
C	3.38174600	-0.51979600	-1.60870200
H	5.35168500	0.84826600	-2.62151500
H	2.04727200	-0.37112000	6.71625800
C	9.67513300	2.63042800	0.84088700
H	9.36837500	2.89221400	2.96966400
H	9.64450000	2.32224300	-1.28180500
H	7.68388900	1.56497200	-2.54107200
H	4.29540200	-0.85971000	-2.10202000
H	2.96408300	-1.40916200	-1.12409900

C	2.37089400	-0.00127100	-2.61645600
H	10.70379700	2.97788700	0.86402600
C	2.41557400	1.31941200	-3.07591900
C	1.31363000	-0.82484000	-3.02101000
C	1.41147700	1.80986800	-3.90982300
H	3.20489900	1.97892400	-2.72683400
C	0.30883500	-0.33514400	-3.85538900
H	1.23915800	-1.83591000	-2.63143200
C	0.35310000	0.98560200	-4.29881300
H	1.43958000	2.84605900	-4.23564700
H	-0.52531500	-0.97736700	-4.12099200
H	-0.44658000	1.38176400	-4.91612600
H	4.21612100	-2.50698700	2.76711900
C	3.30454600	-2.77709000	2.24292500
C	3.36936300	-3.27785100	0.97002500
C	2.04664600	-2.55807000	2.86875600
C	2.18332800	-3.61475500	0.23877400
H	4.33117700	-3.40625500	0.48860800
C	0.88742300	-2.85100700	2.20316100
H	2.01026100	-2.12520500	3.86212000
C	0.90415600	-3.39419400	0.87879700
C	2.23617000	-4.11350200	-1.08329900
H	-0.07028100	-2.63986500	2.66193900
C	-0.28634300	-3.68760700	0.18434700
C	1.03823500	-4.37550000	-1.78691900
C	3.55442900	-4.35729100	-1.73590500
C	-1.61291900	-3.55247000	0.85531300
C	-0.23752700	-4.15127500	-1.14759900
C	1.04296100	-4.88780200	-3.12413900
C	3.94924800	-3.62814600	-2.86852300
C	4.43247300	-5.32466500	-1.22548800
C	-2.51279100	-2.52345300	0.47568500
C	-2.03481800	-4.45254500	1.80953600
C	-1.42819400	-4.42657500	-1.89145500
C	-0.12183300	-5.12273000	-3.80349700
H	1.99454400	-5.09400000	-3.59798300
C	5.19124500	-3.84924000	-3.46227200
H	3.27709600	-2.88464300	-3.28470000
C	5.67374900	-5.54824200	-1.81983400
H	4.13281200	-5.90131900	-0.35533400
C	-3.80912900	-2.41940500	0.95473500
O	-2.04794600	-1.59729900	-0.45155900
C	-3.31011100	-4.34493600	2.42050600
H	-1.37102200	-5.25769300	2.11168200
C	-1.37608800	-4.88217200	-3.18057300
H	-2.38840500	-4.26616600	-1.41637800

H	-0.08895300	-5.50663900	-4.81911200
C	6.05930800	-4.80812900	-2.93835200
H	5.47987400	-3.26999200	-4.33499700
H	6.33905700	-6.30271000	-1.40937400
C	-4.21336400	-3.31550500	1.99882300
C	-4.71250100	-1.38187600	0.38430500
P	-1.70033300	-0.10313900	0.11326400
C	-3.70850800	-5.23767800	3.45017300
H	-2.29557100	-5.07369600	-3.72614100
H	7.02753700	-4.97947200	-3.39993300
C	-5.46081600	-3.19733500	2.66766700
C	-4.28991100	-0.06615500	0.33670100
C	-5.97315000	-1.70568100	-0.22266800
O	-3.06972500	0.26992100	0.90813800
N	3.74714900	0.43056000	-0.55583600
H	2.73845800	-0.62069800	0.94722900
C	-4.93326800	-5.10970900	4.06224000
H	-3.01743700	-6.02037800	3.75219100
C	-5.81084200	-4.07119600	3.67194000
H	-6.13569400	-2.39891700	2.38286200
C	-5.03176300	0.98569500	-0.26146900
C	-6.77111200	-0.64701500	-0.76984400
C	-6.45171500	-3.03776600	-0.34538500
H	-5.22461000	-5.79556500	4.85234300
H	-6.76757700	-3.95851400	4.17369200
C	-6.27280600	0.68191000	-0.77344200
C	-4.39277000	2.32697900	-0.38707200
C	-8.02876500	-0.95528100	-1.35206900
C	-7.67064400	-3.30328300	-0.92778600
H	-5.84199300	-3.85338900	0.02308900
H	-6.86718900	1.46689600	-1.23258200
C	-3.79136500	2.68729000	-1.60943300
C	-4.28876700	3.16806700	0.73621100
C	-8.47664600	-2.25334500	-1.42514800
H	-8.62587500	-0.14015500	-1.75268300
H	-8.01351300	-4.33062200	-1.01102900
C	-3.84294400	1.83733300	-2.75926500
C	-3.06283900	3.93090200	-1.70720700
C	-4.88369700	2.81881000	1.98825800
C	-3.54832900	4.40329200	0.64354100
H	-9.43890500	-2.47635800	-1.87666700
C	-3.24732600	2.19593100	-3.93654300
H	-4.35340300	0.88540100	-2.67867300
C	-2.47212700	4.27323600	-2.96569500
C	-2.94428000	4.76972300	-0.57870700
C	-4.74857800	3.62174100	3.08806800

H	-5.44901000	1.89535700	2.05197200
C	-3.42012300	5.20361300	1.82329100
C	-2.55993900	3.43584500	-4.04447000
H	-3.29572200	1.52969700	-4.79334000
H	-1.94832800	5.21767300	-3.05164600
C	-2.16443400	6.03738200	-0.67161600
C	-4.00003800	4.82796700	3.00534800
H	-5.20721000	3.33584300	4.03044800
H	-2.84315900	6.11913200	1.76813300
H	-2.10350200	3.71880200	-4.98915400
C	-0.76858500	6.00486800	-0.80724300
C	-2.81027500	7.28042700	-0.62457600
H	-3.88523000	5.45094100	3.88795800
C	-0.03882200	7.19037500	-0.89121600
H	-0.26967100	5.04239800	-0.84789600
C	-2.07925900	8.46556000	-0.70738700
H	-3.89143600	7.31073300	-0.52323700
C	-0.69060700	8.42387000	-0.84009200
H	1.04235600	7.15068700	-0.99664700
H	-2.59528100	9.42116000	-0.67167600
H	-0.12068700	9.34658900	-0.90533400

TS12-S3

O	-0.62991800	-0.72757800	0.17634700
H	0.32404800	-0.07246700	-0.19610700
C	1.40914100	0.54026700	-0.79221600
O	-1.65873600	0.24327100	-2.00522100
H	-0.49941800	1.40746800	-2.15386700
O	0.09390600	2.19290600	-1.92229100
C	2.31305300	2.73551800	0.79710900
C	1.02093700	1.86504400	-1.04452600
H	1.34566600	-0.11506800	-1.66270500
C	3.91201600	0.95006400	3.22835600
C	5.13965400	1.52416900	1.03258300
C	2.51051300	0.75350000	3.58339500
C	0.32851300	0.96696500	2.51703100
C	1.55278800	2.97502300	-0.28364100
H	2.65615500	3.55607500	1.42115200
C	1.71169500	1.00452500	2.44397400
H	4.74165200	0.79762100	3.90926900
C	6.33413400	2.22084300	1.40412100
C	5.00141000	0.98638000	-0.25842400
C	1.91134100	0.38950200	4.79153600
C	-0.26739700	0.59383000	3.72774000
H	-0.29650100	1.20552200	1.66769800
H	1.25277300	3.97468300	-0.57071100

C	4.01694500	1.30121300	1.92085400
C	6.49428600	2.85514200	2.66441000
C	7.41631100	2.30162300	0.47099000
C	2.63597400	1.34651600	1.27616100
C	6.08994800	1.08891800	-1.17062500
C	0.51657900	0.30074400	4.84807100
H	2.51511900	0.18920800	5.67251700
H	-1.34978200	0.53621800	3.78655400
C	7.66794700	3.49889300	2.99366500
H	5.66831800	2.84762200	3.36605400
C	8.61107400	2.96701600	0.83985800
C	7.25444300	1.71345100	-0.81097600
C	2.59955500	0.30117100	0.13302800
C	4.03871900	-0.91899700	-1.45610700
H	5.99699400	0.65692200	-2.15975500
H	0.03597700	0.01446500	5.77967800
C	8.74469400	3.55022400	2.08027700
H	7.76228100	3.98024300	3.96333800
H	9.42282400	3.01220700	0.11765800
H	8.07667200	1.77215900	-1.51962400
H	3.05538400	-1.39010800	-1.55543600
H	4.33849100	-0.65692200	-2.47839500
C	5.00200600	-1.95004300	-0.88913900
H	9.66526300	4.05775500	2.35318000
C	5.50685500	-2.94084400	-1.73541900
C	5.34851800	-1.97886100	0.46533400
C	6.32401400	-3.95515400	-1.23924000
H	5.23905700	-2.93473600	-2.78767900
C	6.17359600	-2.98747400	0.96271800
H	4.97307600	-1.21648900	1.13714300
C	6.66097900	-3.98211600	0.11442000
H	6.69122600	-4.72449900	-1.91114900
H	6.43481800	-2.99415100	2.01750200
H	7.30062600	-4.76901200	0.50410100
H	3.39397500	-2.86051200	2.19578800
C	2.41572700	-3.08821500	1.78598700
C	2.32135000	-3.72310300	0.57566200
C	1.24502500	-2.70368000	2.49500800
C	1.04733200	-4.03941000	0.00227200
H	3.22203300	-3.98471100	0.03389100
C	0.00964100	-2.98015300	1.97881900
H	1.33336300	-2.16056500	3.43053700
C	-0.14393300	-3.66225600	0.73009000
C	0.93264000	-4.66572100	-1.25750900
H	-0.87745600	-2.64519600	2.50088700
C	-1.41214800	-3.91180900	0.16668300

C	-0.34155300	-4.95374900	-1.79606500
C	2.14735900	-5.01796400	-2.04701600
C	-2.63472300	-3.41643700	0.86023800
C	-1.52870800	-4.56097200	-1.07687000
C	-0.50427400	-5.64584700	-3.03897000
C	3.00656500	-6.04711400	-1.63926400
C	2.42182200	-4.35272400	-3.25138400
C	-3.29927500	-2.25981400	0.37196700
C	-3.12407200	-4.00012400	2.00734500
C	-2.80494800	-4.84917800	-1.65334600
C	-1.74620400	-5.90599000	-3.55301400
H	0.38036500	-5.97407200	-3.57118900
C	4.09972500	-6.41469700	-2.42291400
H	2.80309700	-6.56632300	-0.70744800
C	3.51045000	-4.72412400	-4.03991400
H	1.75810100	-3.55584600	-3.57497000
C	-4.37474100	-1.66376200	1.00874200
O	-2.81409800	-1.70012200	-0.81057700
C	-4.20535200	-3.43274800	2.72781200
H	-2.64111100	-4.89188500	2.39683900
C	-2.91414600	-5.49530700	-2.85436100
H	-3.69634600	-4.55131800	-1.11271800
H	-1.84216900	-6.43562500	-4.49668800
C	4.35026000	-5.76094800	-3.63009000
H	4.75147400	-7.21911300	-2.09361500
H	3.69982000	-4.20617700	-4.97642900
C	-4.82165000	-2.22978700	2.25126300
C	-4.95718300	-0.41041200	0.45528000
P	-1.87173300	-0.38339800	-0.66261100
C	-4.65395500	-4.00991500	3.94510400
H	-3.89449400	-5.70432200	-3.27291700
H	5.19563600	-6.05524700	-4.24592700
C	-5.81895600	-1.62280600	3.06142200
C	-4.11667700	0.65430900	0.17512400
C	-6.35519900	-0.25511600	0.17601100
O	-2.75497100	0.51292200	0.38424800
N	3.86250300	0.28205600	-0.64096100
H	2.47520800	-0.68031500	0.60520500
C	-5.64572200	-3.41382100	4.68771100
H	-4.18346000	-4.92893400	4.28491400
C	-6.22091000	-2.20025800	4.24474800
H	-6.26202000	-0.68810600	2.74144100
C	-4.56677700	1.91901400	-0.28492500
C	-6.83763100	1.02207700	-0.26084700
C	-7.28268100	-1.32580000	0.28030500
H	-5.97732900	-3.86220400	5.61969400

H	-6.98370900	-1.71493200	4.84683600
C	-5.92358400	2.08707100	-0.46320900
C	-3.57507700	2.98292500	-0.60469000
C	-8.22448800	1.18831400	-0.51762400
C	-8.61864800	-1.13440100	0.00967900
H	-6.92309300	-2.30566400	0.57103900
H	-6.30055300	3.04671900	-0.80527700
C	-2.77766500	3.54285300	0.41483500
C	-3.40115900	3.38610500	-1.94569000
C	-9.10016400	0.13699400	-0.38136000
H	-8.57728800	2.16506700	-0.83847300
H	-9.30882400	-1.96915700	0.09127500
C	-2.91853300	3.14821100	1.78288700
C	-1.76965900	4.52167600	0.08463300
C	-4.12151000	2.77600200	-3.02174200
C	-2.45076400	4.42643500	-2.26304900
H	-10.15773200	0.27530200	-0.58622500
C	-2.07925900	3.62198700	2.75387100
H	-3.70299600	2.44677300	2.04235300
C	-0.90727100	4.98152300	1.13206000
C	-1.63490900	4.96970300	-1.24795400
C	-3.97664500	3.20795200	-4.31175900
H	-4.78135800	1.94656300	-2.80092300
C	-2.36572800	4.87440400	-3.62001400
C	-1.04563200	4.53864300	2.41950700
H	-2.19173100	3.28953300	3.78134000
H	-0.12402700	5.68854700	0.88571800
C	-0.60078800	5.98501600	-1.59101100
C	-3.10560400	4.29005500	-4.61188600
H	-4.52911600	2.72228700	-5.11097000
H	-1.69660300	5.69316200	-3.85510900
H	-0.36690800	4.88890700	3.19173300
C	-0.67328700	7.28995300	-1.08268300
C	0.47534300	5.64832300	-2.42782400
H	-3.02100700	4.64690900	-5.63447300
C	0.30671800	8.23170400	-1.39460300
H	-1.50661200	7.56049100	-0.44078700
C	1.45751700	6.58910400	-2.73655700
H	0.53176600	4.64072800	-2.82748500
C	1.37698300	7.88331800	-2.22017700
H	0.23298700	9.23895600	-0.99392100
H	2.28707700	6.30944500	-3.38009900
H	2.14194000	8.61614000	-2.46079200

TS12-S4

O	-0.73191700	-0.87748100	0.44131000

H	0.36976300	-0.45485300	0.19174900
C	1.57528400	-0.03930400	-0.38099200
O	-1.32789000	0.57794600	-1.63338900
H	0.02063400	1.50133900	-1.53413500
O	0.76381500	2.07100900	-1.15227200
C	3.11808100	1.50834300	1.45521800
C	1.57474600	1.36321800	-0.40121700
H	1.28041300	-0.49062100	-1.32623600
C	3.97064400	-1.14431400	3.50262600
C	5.44378400	-0.59423200	1.46119000
C	2.57353100	-0.91014700	3.84604700
C	0.64270100	0.22560500	2.89348200
C	2.43373100	2.13287500	0.48032400
H	3.71201500	2.07703600	2.16546900
C	1.95455600	-0.20816800	2.78723500
H	4.65686000	-1.71289200	4.11864700
C	6.77621800	-0.50888000	1.98508500
C	5.22803300	-0.64687900	0.06643000
C	1.84658500	-1.23755000	4.99243700
C	-0.08780200	-0.11497000	4.03883900
H	0.16936400	0.81477700	2.11954500
H	2.44523400	3.20910100	0.35784100
C	4.25595300	-0.59888900	2.29158900
C	7.05601700	-0.35309600	3.37018600
C	7.89261900	-0.54058500	1.08931100
C	2.99769000	0.02392800	1.69081400
C	6.36284500	-0.61989500	-0.80207500
C	0.50713400	-0.84217200	5.07328500
H	2.31587300	-1.77614400	5.81138800
H	-1.12390300	0.19895600	4.11396700
C	8.35138700	-0.29131700	3.83742500
H	6.23439800	-0.25009200	4.06714800
C	9.21301000	-0.48400900	1.59891400
C	7.63797400	-0.59128600	-0.30397500
C	2.71824400	-0.70711200	0.36152000
C	3.74531700	-1.09322000	-1.86164000
H	6.22217500	-0.60427100	-1.87507800
H	-0.07271900	-1.09148800	5.95797200
C	9.44801300	-0.37419500	2.95076100
H	8.52966000	-0.16627400	4.90214700
H	10.04064400	-0.51955800	0.89425100
H	8.48101200	-0.58448400	-0.99040200
H	4.60243000	-1.67119300	-2.21839800
H	2.90397600	-1.79127300	-1.91464200
C	3.47533000	0.10177400	-2.76308900
H	10.46352800	-0.33194000	3.33326400

C	2.57553100	-0.00568700	-3.82860400
C	4.07341600	1.34111300	-2.51016500
C	2.26733000	1.10147000	-4.61905500
H	2.08649400	-0.95679700	-4.02160900
C	3.77623800	2.44681600	-3.30700500
H	4.74567000	1.44920500	-1.66634000
C	2.86742000	2.33444800	-4.35968100
H	1.54624300	1.00416800	-5.42553700
H	4.25039200	3.40121400	-3.09623400
H	2.62057500	3.20145000	-4.96542300
H	2.53064900	-3.86678900	2.30592200
C	1.57115000	-3.88895100	1.79684100
C	1.50694700	-4.22697600	0.47118200
C	0.39337200	-3.52607800	2.50377300
C	0.26096300	-4.24295500	-0.23641700
H	2.41449100	-4.47670500	-0.06532500
C	-0.81400300	-3.51207900	1.86359200
H	0.46291000	-3.22075100	3.54168600
C	-0.93602100	-3.86971900	0.48366100
C	0.18633700	-4.54684700	-1.61374800
H	-1.69980000	-3.18868000	2.39561900
C	-2.16418000	-3.78524100	-0.20409400
C	-1.06095400	-4.54319500	-2.27584500
C	1.43244400	-4.78476000	-2.39445400
C	-3.34349800	-3.17394800	0.47331200
C	-2.24905000	-4.14455500	-1.56257400
C	-1.19261500	-4.92385800	-3.64927200
C	1.81903400	-3.85462100	-3.37170000
C	2.25315900	-5.89847300	-2.17063900
C	-3.68384900	-1.82937400	0.15952600
C	-4.08109400	-3.81239700	1.44406700
C	-3.49133800	-4.11122500	-2.26866500
C	-2.40464600	-4.88741600	-4.28525500
H	-0.31004500	-5.25694700	-4.18282600
C	3.00161600	-4.01912300	-4.09129300
H	1.17516500	-3.00013000	-3.55989600
C	3.43635700	-6.06673600	-2.89043900
H	1.95876800	-6.63083100	-1.42465700
C	-4.67815800	-1.11378400	0.80579000
O	-2.95452000	-1.21862700	-0.85853800
C	-5.09526900	-3.13568300	2.16951300
H	-3.85141600	-4.84390200	1.69628800
C	-3.56997800	-4.46732500	-3.58761600
H	-4.38275400	-3.80580700	-1.73184100
H	-2.47997600	-5.18704500	-5.32675800
C	3.81741300	-5.12563700	-3.84843800

H	3.28706100	-3.28091000	-4.83600100
H	4.06135600	-6.93535500	-2.70279300
C	-5.37774700	-1.75996900	1.88193900
C	-4.92514400	0.30797200	0.43379600
P	-1.79039700	-0.16990100	-0.42182600
C	-5.80030800	-3.78662600	3.21594700
H	-4.52559000	-4.43840800	-4.10324500
H	4.74090000	-5.25645900	-4.40517700
C	-6.31061100	-1.08128200	2.71136400
C	-3.85717300	1.18933400	0.38978500
C	-6.22164100	0.80815500	0.07653000
O	-2.58802300	0.72401200	0.68854100
N	3.95464900	-0.73189900	-0.46588200
H	2.43166000	-1.73860300	0.59245700
C	-6.72269100	-3.10822800	3.97733500
H	-5.58245200	-4.83331700	3.41208000
C	-6.96606500	-1.73774700	3.72863100
H	-6.50018400	-0.02903900	2.53995300
C	-3.97521300	2.56956800	0.07778200
C	-6.37534300	2.20860400	-0.18989000
C	-7.35914900	-0.03117300	-0.06209200
H	-7.25195900	-3.61597300	4.77836900
H	-7.67267300	-1.19510000	4.35002100
C	-5.24047100	3.05875900	-0.16853200
C	-2.74648000	3.40255700	-0.05294300
C	-7.66096500	2.71512100	-0.51806800
C	-8.58839900	0.49009400	-0.39693800
H	-7.24759500	-1.09746600	0.09324000
H	-5.36852500	4.11248300	-0.39949400
C	-1.91298400	3.63184200	1.06135800
C	-2.38218000	3.89791600	-1.32421600
C	-8.74793900	1.87861400	-0.61441100
H	-7.76363700	3.78066300	-0.70663100
H	-9.44280700	-0.17243900	-0.50124600
C	-2.24455300	3.13772700	2.36237700
C	-0.67426700	4.35482100	0.89897800
C	-3.14068500	3.60338100	-2.50204900
C	-1.18979500	4.70156900	-1.46568100
H	-9.72481100	2.27740200	-0.87178800
C	-1.38368300	3.26879400	3.41766600
H	-3.19659600	2.63813200	2.49975100
C	0.20419700	4.44986300	2.02640800
C	-0.34144200	4.90674800	-0.35730000
C	-2.79868100	4.12363300	-3.72032200
H	-3.99270600	2.94075600	-2.41952300
C	-0.89715800	5.25986700	-2.75098300

C	-0.12951500	3.91437100	3.24095400
H	-1.65081200	2.87003000	4.39178900
H	1.15622800	4.95139400	1.90076600
C	0.92809900	5.66801700	-0.52258200
C	-1.67559200	4.98563600	-3.84242000
H	-3.38752100	3.87651500	-4.59904200
H	-0.03578000	5.90916600	-2.84986100
H	0.56233200	3.98333500	4.07524700
C	1.12746100	6.88139100	0.15180700
C	1.95240500	5.18277900	-1.35242000
H	-1.43026700	5.41938500	-4.80782800
C	2.31974700	7.59040100	0.00894100
H	0.33698600	7.26562300	0.78991600
C	3.14492100	5.89216000	-1.49217700
H	1.80641000	4.24478700	-1.87773400
C	3.33414000	7.09645500	-0.81247700
H	2.45497600	8.52994600	0.53761200
H	3.93006400	5.50262700	-2.13442300
H	4.26414000	7.64689300	-0.92340000

Int13

O	-1.10482300	-0.14056700	1.85716900
H	1.03902000	0.52473300	1.59157000
C	1.42927100	0.85887500	0.62592100
O	-1.80190300	0.96721200	-0.42622200
H	-1.11446300	1.67619900	-0.20649300
O	0.02875900	2.75150600	0.15465300
C	3.40216300	2.84045900	1.28993200
C	1.14428500	2.33273800	0.49177200
H	0.83672800	0.32150000	-0.11131600
C	5.85976900	0.63048700	2.23068900
C	5.62966900	1.29500400	-0.24905000
C	4.93265600	0.55189200	3.35146300
C	2.59143400	1.04816500	3.80496400
C	2.20920200	3.27114900	0.84296200
H	4.16211600	3.55471400	1.59808000
C	3.65923000	0.99001400	2.92292800
H	6.88663000	0.28824700	2.27094800
C	6.95383600	1.67865400	-0.64817700
C	4.64161900	1.04159300	-1.22286500
C	5.12484400	0.13483800	4.67011900
C	2.78668300	0.62317700	5.12616900
H	1.61847900	1.41541400	3.49496300
H	1.97267200	4.32809600	0.76426800
C	5.22548700	1.11470000	1.13073400
C	7.97087300	2.02650200	0.28085700

C	7.28037900	1.74209800	-2.04050800
C	3.75716000	1.38299300	1.45081800
C	4.98229700	1.17127400	-2.60301800
C	4.03921900	0.16962900	5.55020100
H	6.10051700	-0.20376700	5.00775400
H	1.95630900	0.65300800	5.82506400
C	9.24010900	2.36271000	-0.14015300
H	7.73428500	2.05599000	1.33691000
C	8.59442900	2.08438200	-2.44281000
C	6.25680300	1.48625300	-2.98933600
C	2.90998600	0.50137100	0.51619900
C	2.58076600	-0.16890600	-1.83020100
H	4.21862900	1.02695400	-3.35702100
H	4.17323300	-0.15051600	6.57979100
C	9.56729900	2.37882900	-1.51388500
H	9.99444100	2.62887000	0.59531500
H	8.81697400	2.11786600	-3.50666600
H	6.49410800	1.56223100	-4.04734100
H	3.26235000	-0.62511300	-2.55456800
H	2.15002100	-1.01172500	-1.28447600
C	1.46991600	0.59341600	-2.52207900
H	10.57249200	2.63916900	-1.83178100
C	1.57422600	1.96632200	-2.76513800
C	0.29731600	-0.07973200	-2.88387800
C	0.52110200	2.65709700	-3.36399300
H	2.46471000	2.49988500	-2.44768300
C	-0.75638800	0.61350800	-3.47560300
H	0.19361700	-1.13899000	-2.66648200
C	-0.64583800	1.98272900	-3.71976200
H	0.60304000	3.72759900	-3.53125300
H	-1.67942000	0.09239900	-3.71122200
H	-1.47711100	2.52584700	-4.15262000
H	3.74950400	-2.29814800	4.03363400
C	3.02557200	-2.52602100	3.25895600
C	3.45137800	-2.86964100	2.00457600
C	1.63683300	-2.43253200	3.54858200
C	2.51943600	-3.12805400	0.94759400
H	4.51133400	-2.92726600	1.78923900
C	0.70873800	-2.72737600	2.58802500
H	1.31691100	-2.11604100	4.53610600
C	1.10793100	-3.09825400	1.26470300
C	2.94009000	-3.39628900	-0.37356100
H	-0.34853300	-2.63738800	2.80228900
C	0.15872700	-3.41365800	0.27345700
C	1.97666500	-3.59816900	-1.38942800
C	4.39100600	-3.42503900	-0.71790900

C	-1.27609700	-3.61254600	0.63711500
C	0.57089000	-3.63191100	-1.05753200
C	2.34756500	-3.72816900	-2.76626000
C	4.99492300	-4.60538900	-1.17596100
C	5.17835600	-2.26946900	-0.61739900
C	-2.28293400	-2.68009700	0.27733300
C	-1.66811400	-4.76613200	1.28224300
C	-0.37169000	-3.87154500	-2.10705500
C	1.40797700	-3.91223700	-3.74526400
H	3.39679300	-3.66134600	-3.02776000
C	6.34397500	-4.62308700	-1.52929800
H	4.39736800	-5.50898800	-1.25545100
C	6.52427400	-2.28015300	-0.97829200
H	4.73374600	-1.35248900	-0.25997400
C	-3.63892700	-2.91282500	0.46341200
O	-1.86227500	-1.48732600	-0.30668800
C	-3.02409200	-5.01445100	1.60867400
H	-0.91547700	-5.49837800	1.56038300
C	0.02962400	-4.00071000	-3.40956200
H	-1.42470000	-3.92891000	-1.85745100
H	1.71518800	-3.99091200	-4.78407600
C	7.11167700	-3.46012300	-1.43629100
H	6.79566800	-5.54718100	-1.87959300
H	7.10226900	-1.36324500	-0.90609100
C	-4.02900000	-4.07847300	1.20301900
C	-4.63339400	-1.95353900	-0.09017800
P	-1.96295700	-0.15976200	0.65475400
C	-3.40498500	-6.17253300	2.33676300
H	-0.70690600	-4.16095400	-4.19154900
H	8.16041600	-3.47470100	-1.71941900
C	-5.37274500	-4.32197500	1.59528600
C	-4.50908300	-0.60680200	0.19405800
C	-5.67525700	-2.36180900	-0.99089100
O	-3.52587200	-0.20266900	1.08336300
N	3.36394100	0.62793200	-0.88156200
H	3.02662500	-0.53848900	0.84212000
C	-4.71835800	-6.39019600	2.68007000
H	-2.63142100	-6.87856500	2.62734500
C	-5.70724100	-5.44694900	2.31411500
H	-6.13668400	-3.60099100	1.33059200
C	-5.35212700	0.40148600	-0.35039800
C	-6.60269000	-1.37666600	-1.46464500
C	-5.81449400	-3.69449800	-1.46226100
H	-4.99787500	-7.27640600	3.24223300
H	-6.73998700	-5.60878600	2.60911000
C	-6.41316200	-0.01225700	-1.12628500

C	-4.96529600	1.82856600	-0.17956700
C	-7.66032100	-1.77444900	-2.32455400
C	-6.84270900	-4.04430100	-2.30866400
H	-5.09613500	-4.44245800	-1.15041500
H	-7.08961600	0.72973000	-1.54100800
C	-4.93997600	2.41373800	1.10194200
C	-4.49151700	2.54711600	-1.30052500
C	-7.78614200	-3.08099400	-2.73452300
H	-8.36138200	-1.01692200	-2.66544000
H	-6.92631500	-5.06990100	-2.65658100
C	-5.48510800	1.74521400	2.24141400
C	-4.33392900	3.70857800	1.29152300
C	-4.51330800	2.01247100	-2.62839600
C	-3.93219800	3.86584000	-1.11361300
H	-8.59656500	-3.37204000	-3.39638800
C	-5.41541200	2.29709800	3.49121500
H	-5.96248200	0.78220800	2.09672800
C	-4.25972500	4.23777900	2.61921400
C	-3.81490200	4.40667000	0.18262400
C	-4.10753100	2.75450100	-3.70446300
H	-4.86848500	1.00120900	-2.77968000
C	-3.50735300	4.60098600	-2.26591600
C	-4.78376600	3.55670900	3.68443700
H	-5.83682400	1.76995100	4.34235400
H	-3.77969800	5.19818100	2.76673000
C	-3.10984900	5.70615900	0.37859100
C	-3.61194500	4.07496100	-3.52439800
H	-4.15427200	2.32812900	-4.70294900
H	-3.11133000	5.59942300	-2.12188300
H	-4.71919200	3.97786600	4.68375500
C	-1.70889900	5.75102700	0.32237700
C	-3.82075000	6.88928700	0.61727400
H	-3.30252900	4.65854500	-4.38711900
C	-1.03804700	6.96042700	0.50274200
H	-1.15237000	4.83497800	0.14549500
C	-3.14686300	8.09779300	0.79633800
H	-4.90581800	6.85551200	0.65976100
C	-1.75295500	8.13606000	0.73924300
H	0.04791700	6.98416400	0.45914300
H	-3.71074800	9.00864700	0.97838100
H	-1.22735700	9.07680100	0.87851400

TS14-C1

C	0.25669000	-1.89702000	-1.94371100
H	0.34861300	-0.55124200	-1.87611800
O	0.54577000	0.66916100	-1.70280400

C	1.79742600	3.13206800	-0.14229900
C	3.10508300	3.06251100	-0.68908900
C	3.63090100	4.20079200	-1.25979900
C	3.41313000	6.55238300	-1.99055300
C	2.67100200	7.70475800	-2.10043400
C	1.36052400	7.75061500	-1.56973100
C	0.82662000	6.65927900	-0.92272000
C	-0.55823600	5.22326500	1.65263300
C	-1.86076100	5.19111200	2.24845300
C	-2.80630300	4.23151000	1.80924000
C	-2.49902000	3.30172900	0.83886400
C	-1.21272600	3.36523600	0.24445600
C	-0.26073500	4.30992500	0.58713000
C	0.39701700	6.13587000	2.17600400
C	0.06837400	6.99704000	3.19827900
C	-1.23152500	6.99252600	3.75631800
C	-2.17117000	6.10256600	3.29227300
C	-5.14793300	-0.76458300	1.92193900
C	-4.51660500	-0.83921400	3.13429600
C	-3.50148200	0.09797000	3.47396900
C	-3.14639500	1.08040700	2.59077800
C	-3.80919300	1.22268000	1.33049700
C	-3.48272200	2.25888500	0.43349400
C	-4.11944800	2.34115100	-0.82115600
C	-3.79776600	3.37342800	-1.75716400
C	-4.43941100	3.46850100	-2.96158400
C	-5.46607800	2.54548400	-3.29905500
C	-5.81429600	1.54865800	-2.42805100
C	-5.15062700	1.39211700	-1.16910700
C	-5.51142100	0.37809300	-0.25339400
C	-4.83907300	0.27102700	0.98291600
C	-6.64926300	-0.53208000	-0.56527000
C	-6.58240500	-1.45752700	-1.61495800
C	-7.67856100	-2.26629100	-1.91688300
C	-8.85171400	-2.17314700	-1.16827800
C	-8.92517900	-1.26309500	-0.11206200
C	-7.83411800	-0.44806600	0.18289600
C	4.97050700	-1.00067900	-2.86796000
C	4.35084600	-0.64472400	-4.03440400
C	3.56480700	0.53692800	-4.10123800
C	3.42547800	1.33365200	-2.99890700
C	4.06554300	1.00625900	-1.76179300
C	3.89143600	1.79950300	-0.61222400
C	4.46372500	1.41477200	0.61619300
C	4.26151900	2.17902500	1.80827000
C	4.80395300	1.79125500	3.00318600

C	5.61623300	0.62745500	3.07372900
C	5.84836200	-0.12274700	1.95264200
C	5.26661400	0.21841600	0.69054100
C	5.45814600	-0.57413600	-0.46330400
C	4.86165000	-0.19781600	-1.68838700
C	6.23823800	-1.83899900	-0.37307100
C	5.79655100	-2.88269500	0.45452700
C	6.49975100	-4.08449900	0.52099600
C	7.66015700	-4.26345000	-0.23456300
C	8.11675100	-3.22694700	-1.05029800
C	7.41222800	-2.02507800	-1.11683400
O	1.28315500	1.97394700	0.41947800
O	-0.91668500	2.44208800	-0.74579900
P	0.05391900	1.18433500	-0.33875600
C	1.05453000	4.30144400	-0.10520800
O	-0.55207200	0.24504200	0.64296800
H	4.63406200	4.17013500	-1.67577300
H	4.41444300	6.49577900	-2.40976200
H	3.08188800	8.57513500	-2.60376400
H	0.76718900	8.65394500	-1.67916800
H	-0.18180300	6.70340200	-0.52919600
H	-3.78917800	4.21042100	2.27167600
H	1.39865800	6.14374000	1.76407300
H	0.81691500	7.68315200	3.58417200
H	-1.47880500	7.68212400	4.55821200
H	-3.16772300	6.07242400	3.72542700
H	-5.89744600	-1.49911400	1.65355600
H	-4.78005100	-1.62700800	3.83554500
H	-2.99098100	0.01822300	4.42982100
H	-2.34306300	1.76460500	2.83248500
H	-3.02782100	4.08978600	-1.49689500
H	-4.17147200	4.25678600	-3.65921200
H	-5.98350800	2.63970400	-4.24970900
H	-6.61089200	0.86131300	-2.68474600
H	-5.66887700	-1.54127200	-2.19461500
H	-7.60757500	-2.97357900	-2.73666900
H	-9.70376200	-2.80398100	-1.40638500
H	-9.83497300	-1.18101500	0.47638700
H	-7.89264900	0.26970100	0.99593200
H	5.53258000	-1.92315700	-2.82004200
H	4.43446600	-1.28654200	-4.90538500
H	3.05553100	0.79509300	-5.02530600
H	2.79573400	2.21346800	-3.03762900
H	3.65900800	3.07824200	1.75324900
H	4.62422900	2.37940600	3.89858200
H	6.06091300	0.33785100	4.02150000

H	6.48293000	-0.99811900	2.01281700
H	4.89346900	-2.74941200	1.03732100
H	6.13490800	-4.88257700	1.16219500
H	8.20750200	-5.20071200	-0.18436200
H	9.02428700	-3.35237600	-1.63461000
H	7.76819600	-1.21916000	-1.75201000
C	1.57506600	5.46113400	-0.76988500
C	2.88720600	5.40451000	-1.34118200
C	2.43349100	-3.09766400	-1.58208100
C	2.58775100	-3.61587300	-4.34388100
H	0.79407500	-2.42890300	-4.49768100
C	3.46285700	-3.88693900	-2.11390900
C	3.53871200	-4.15583700	-3.47851300
H	2.64489200	-3.80496600	-5.41227100
H	4.22713000	-4.26405100	-1.44173300
H	4.35095800	-4.76488300	-3.86526100
C	2.39133800	-2.80937700	-0.12953000
C	2.32367100	-1.48811300	0.33942100
C	2.45826300	-3.84266300	0.81269400
C	2.29685600	-1.20091200	1.69876200
H	2.31139300	-0.67162500	-0.37101900
C	2.44231900	-3.56994300	2.17858300
H	2.50963800	-4.87324100	0.47301800
C	2.34160100	-2.24805300	2.62307900
H	2.23647400	-0.17591800	2.04586200
H	2.46433900	-4.38300700	2.89936800
C	-2.00941800	-2.69839200	-0.87432900
C	-3.08530200	-3.18827500	-1.72280400
C	-2.09916800	-2.83691200	0.55535600
C	-3.08894000	-2.99807900	-3.11881600
C	-4.14678300	-3.92733400	-1.13401300
C	-3.19589700	-3.57993000	1.10036300
C	-4.09149100	-3.54414700	-3.90222100
H	-2.28943400	-2.42071900	-3.57137300
C	-5.14976800	-4.48709300	-1.95356900
C	-4.15980000	-4.09670500	0.28579000
H	-3.25474800	-3.72380200	2.17129100
C	-5.12298100	-4.30733800	-3.32304800
H	-4.07731400	-3.38459400	-4.97660000
H	-5.95183800	-5.05096000	-1.48529600
H	-4.98411900	-4.65330000	0.72494200
H	-5.89657400	-4.74157600	-3.94920500
O	2.25533500	-1.93415500	3.95335500
H	2.09415900	-2.75553500	4.44638000
N	-1.15059600	-2.30705600	1.33508000
H	-0.59060200	-1.55370700	0.92356100

C	-1.12762500	-2.35190000	2.78462900
H	-0.48203800	-1.52874100	3.09938100
H	-2.12535300	-2.12749300	3.17931500
C	-0.61427100	-3.64809200	3.38544000
C	-0.25882900	-3.65439300	4.74132800
C	-0.47885800	-4.82662300	2.65000100
C	0.23510600	-4.81055400	5.34533400
H	-0.35924500	-2.73994000	5.32175700
C	0.00264800	-5.98958400	3.25484300
H	-0.71417300	-4.83109800	1.59242300
C	0.36766400	-5.98606400	4.60027100
H	0.51093800	-4.79632400	6.39637700
H	0.10657600	-6.89595800	2.66488800
H	0.75028200	-6.88881200	5.06761800
C	1.44742600	-2.59914700	-2.45961200
C	-0.83025500	-2.28601300	-1.43627400
C	1.55128500	-2.83624400	-3.83478000

TS14-C2

O	-0.74341000	0.56826900	1.24903700
H	-1.36137800	-0.38738800	0.80054800
C	-1.74116400	-1.55454800	0.15167500
C	-0.43892000	-3.16057300	1.74199800
C	0.96995500	-3.03655700	1.48796000
C	-2.19658400	-1.53886600	-1.23068200
C	-2.72357300	6.21413700	3.30288900
C	-1.85678400	7.14776900	3.82001400
C	-0.51350500	7.18230600	3.37708400
C	-0.06859300	6.30479700	2.41462900
C	1.34322800	5.59837200	-0.43699900
C	2.67952200	5.56967400	-0.95072400
C	3.45178200	4.39159500	-0.81365400
C	2.95056400	3.25239000	-0.21615200
C	1.63539600	3.31055000	0.31361000
C	0.84226500	4.44561800	0.25142200
C	0.55896400	6.76074500	-0.66910800
C	1.08288300	7.84695600	-1.33216900
C	2.41718200	7.83151400	-1.80321400
C	3.19381500	6.71230000	-1.61964900
C	5.50158100	-0.22599600	-2.65357100
C	4.89010500	0.15944000	-3.81512500
C	3.84958800	1.12754800	-3.79298300
C	3.48576800	1.72110300	-2.61512200
C	4.12013600	1.37239700	-1.38022500
C	3.78883600	2.01997600	-0.17059500
C	4.32550700	1.55842700	1.04826400
C	3.97288100	2.15487000	2.29914100

C	4.45278500	1.66385600	3.48250800
C	5.32648100	0.54269500	3.48973000
C	5.72247900	-0.03348100	2.31258700
C	5.26319000	0.45912900	1.04892500
C	5.69209900	-0.10714300	-0.17369600
C	5.11779600	0.32622000	-1.38968300
C	6.75528600	-1.15142300	-0.16918500
C	6.50065500	-2.45405000	-0.62153000
C	7.50256200	-3.42369000	-0.60492400
C	8.77882700	-3.10786800	-0.13767100
C	9.04654600	-1.81299200	0.31034000
C	8.04391400	-0.84462800	0.29437700
C	-5.12858700	-0.98244900	1.61583800
C	-4.51641800	-1.11109100	2.83298900
C	-3.60585300	-0.11802500	3.28855000
C	-3.34313000	0.98386600	2.52205300
C	-3.96353700	1.16546500	1.24539900
C	-3.68704100	2.28465500	0.43341100
C	-4.32525800	2.43326600	-0.81501600
C	-4.05924100	3.55096900	-1.66655500
C	-4.66109500	3.67176700	-2.89032700
C	-5.57361500	2.67885400	-3.34281200
C	-5.85885900	1.59350800	-2.55799400
C	-5.25548500	1.42785900	-1.27170000
C	-5.50762700	0.29207000	-0.47601400
C	-4.87893300	0.15041700	0.77690500
C	-6.40240500	-0.78885800	-0.98260300
C	-5.84959900	-1.96453500	-1.50965000
C	-6.67481600	-2.98050700	-1.98929700
C	-8.06294700	-2.83668400	-1.94811000
C	-8.62150700	-1.66929100	-1.42538500
C	-7.79570400	-0.65148100	-0.94616800
O	-0.96523700	2.46587500	-0.47657300
O	1.13351300	2.17761900	0.93820900
P	0.02536700	1.28586400	0.12053100
C	-0.52403300	4.41095200	0.83744300
O	0.59162300	0.47564600	-0.98893800
H	-4.18739100	4.25133000	2.18191200
H	-3.75312000	6.16226000	3.64765300
H	-2.19532500	7.85003300	4.57623600
H	0.17529100	7.90496200	3.80521300
H	0.96467300	6.33490200	2.09059400
H	4.45880700	4.37343300	-1.22044000
H	-0.46672700	6.78235000	-0.32180700
H	0.46357900	8.72328300	-1.50091100
H	2.81855700	8.69840500	-2.31996200

H	4.21346900	6.67681300	-1.99451400
H	6.28495200	-0.97307000	-2.67825600
H	5.19033300	-0.28751700	-4.75845600
H	3.34184300	1.39607000	-4.71490700
H	2.68630600	2.45107100	-2.59689900
H	3.29741300	3.00167900	2.29924800
H	4.15687200	2.12336000	4.42105900
H	5.68163200	0.14318500	4.43552900
H	6.39071300	-0.88628300	2.32476300
H	5.51478700	-2.70187200	-0.99720500
H	7.28291400	-4.42709100	-0.96011400
H	9.56015400	-3.86264100	-0.12564000
H	10.03886400	-1.55474700	0.66976600
H	8.25287400	0.16279600	0.64203700
H	-5.82330600	-1.73837400	1.26890300
H	-4.72533500	-1.97421200	3.45906500
H	-3.11106800	-0.24152800	4.24729400
H	-2.63213600	1.72555900	2.86123000
H	-3.36206400	4.30799200	-1.32574300
H	-4.43981200	4.52741100	-3.52193400
H	-6.04097500	2.78230300	-4.31808600
H	-6.54290700	0.82969200	-2.90887800
H	-4.77264500	-2.07374100	-1.54518300
H	-6.22913800	-3.88235900	-2.40044100
H	-8.70602000	-3.62859300	-2.32214800
H	-9.70096700	-1.55013000	-1.38899600
H	-8.22770900	0.25831200	-0.53872500
C	-0.94484600	5.34461800	1.84026800
C	-2.29282900	5.28594300	2.31849400
C	-2.20547400	-2.74458300	-1.98080800
C	-2.98103800	-0.32023000	-3.17626000
H	-2.53456100	0.57937900	-1.27935400
C	-2.62652700	-2.70740800	-3.31132600
C	-3.01993100	-1.50599900	-3.90718600
H	-3.28069100	0.61944100	-3.62779500
H	-2.63586100	-3.63073900	-3.88343200
H	-3.34843300	-1.49961700	-4.94272000
C	-1.71719400	-4.00351400	-1.35836700
C	-0.37818700	-4.38355900	-1.51164100
C	-2.54636300	-4.78141100	-0.53450800
C	0.12954500	-5.48519600	-0.82888800
H	0.28025000	-3.79230800	-2.13852000
C	-2.05166800	-5.89084300	0.14132100
H	-3.58461200	-4.48946700	-0.40187600
C	-0.70151300	-6.22824600	0.01371300
H	1.17844300	-5.74849700	-0.93820000

H	-2.67892700	-6.47431000	0.80596700
C	-1.40631100	3.42045500	0.43488100
C	-0.93419600	-4.10910200	2.70975700
C	-2.74516400	3.34137500	0.90726400
C	-2.30539500	-4.20257900	3.02745200
C	-0.02146000	-4.99816000	3.33793400
C	1.85187900	-3.95910600	2.13581700
C	-2.75078300	-5.14647900	3.93258500
H	-2.99918400	-3.52090600	2.54602000
C	-0.50267300	-5.96039100	4.25423900
C	1.36412400	-4.89211900	3.00824600
H	2.91611500	-3.90765700	1.94107200
C	-1.84822400	-6.03756200	4.55052300
H	-3.80963700	-5.20475100	4.17001100
H	0.20594300	-6.64038100	4.72056400
H	2.05695600	-5.57990600	3.48820500
H	-2.21294900	-6.77845200	5.25537300
O	-0.24222500	-7.29035200	0.74324600
H	0.71629600	-7.35764800	0.61441600
N	1.41662500	-2.05980300	0.68397600
H	0.71993300	-1.46546700	0.24251500
C	2.74718800	-1.89448100	0.10890900
H	2.90401100	-0.82045400	0.01010800
H	3.50490900	-2.27406400	0.79649600
C	2.83099600	-2.56223000	-1.24683300
C	3.31011000	-3.87000400	-1.38159100
C	2.35608700	-1.88523300	-2.37571000
C	3.32403000	-4.49484000	-2.62958800
H	3.67829400	-4.39976600	-0.50577900
C	2.36844200	-2.51046200	-3.62197900
H	1.96366800	-0.87938300	-2.26610300
C	2.85053000	-3.81442500	-3.75314300
H	3.70414800	-5.50846500	-2.72539400
H	2.00128900	-1.97441600	-4.49234600
H	2.85945700	-4.29882600	-4.72554100
C	-3.16446100	4.28787200	1.81759600
C	-1.27779300	-2.43118400	0.93499000
C	-2.57063900	-0.34034300	-1.84613000

TS14-S1

O	-0.79323200	0.45342700	1.19315100
H	-1.35539500	-0.54088600	0.76539700
C	-1.67023200	-1.72718000	0.10079000
C	-0.12075400	-3.25864000	1.53880900
C	1.25420600	-2.97845800	1.23255400
C	-2.23620700	-1.72756300	-1.24080800

C	-3.11876800	6.06239600	3.22330400
C	-2.30272600	7.08100300	3.65544100
C	-0.99177600	7.19979000	3.13694900
C	-0.53010300	6.31951000	2.18494000
C	0.80332900	5.60829100	-0.67968000
C	2.13301600	5.68369500	-1.20470800
C	3.02097100	4.59938100	-1.00517300
C	2.63280400	3.44458700	-0.35641200
C	1.30783500	3.38678900	0.15157800
C	0.41670100	4.44451700	0.06156500
C	-0.08881800	6.68107000	-0.94870900
C	0.32402100	7.78113900	-1.66529700
C	1.64953700	7.86963300	-2.15276000
C	2.53098900	6.83860200	-1.92920300
C	5.57676900	-0.03016800	-2.35905500
C	5.01799100	0.21766500	-3.58214000
C	3.95959700	1.15693200	-3.71099300
C	3.49922800	1.83332400	-2.61493800
C	4.07636000	1.63215400	-1.32050800
C	3.60912200	2.32987700	-0.18643900
C	4.13830900	2.04563000	1.09035800
C	3.68204500	2.72942400	2.26081100
C	4.21175500	2.46526600	3.49411600
C	5.25772300	1.51375200	3.63316100
C	5.73569800	0.84655600	2.53764800
C	5.19221000	1.06737600	1.23141400
C	5.68905800	0.39534600	0.09091800
C	5.14089900	0.66288600	-1.18399700
C	6.78532600	-0.60444300	0.23118400
C	6.60181300	-1.78242000	0.97143100
C	7.62157000	-2.72631500	1.08485500
C	8.85051400	-2.50757300	0.46084600
C	9.05188600	-1.33520100	-0.26888700
C	8.02982600	-0.39333400	-0.38023100
C	-4.94406300	-1.42062300	1.85649800
C	-4.21211300	-1.48595200	3.01108200
C	-3.37420500	-0.40232400	3.39328800
C	-3.29216300	0.71791400	2.61292800
C	-4.03259700	0.82929400	1.39393300
C	-3.93063800	1.96146200	0.55980000
C	-4.69797900	2.04788500	-0.61982100
C	-4.61233600	3.17948800	-1.49016100
C	-5.34258400	3.24426700	-2.64646200
C	-6.20931700	2.17618200	-3.00861400
C	-6.31974700	1.07286700	-2.20490300
C	-5.57822300	0.96354900	-0.98652100

C	-5.64426300	-0.19015600	-0.17886600
C	-4.88726300	-0.26968600	1.00709600
C	-6.47625600	-1.35185800	-0.60727800
C	-5.86509500	-2.47959200	-1.17366700
C	-6.63348000	-3.56733700	-1.58551900
C	-8.02138200	-3.54487900	-1.43603600
C	-8.63760200	-2.42680100	-0.87196600
C	-7.86917200	-1.33702900	-0.46076400
O	-1.27569800	2.31589200	-0.51898500
O	0.90915300	2.22818500	0.80397700
P	-0.15139900	1.24064100	0.03188700
C	-0.91860700	4.33115400	0.70662100
O	0.43555300	0.48175500	-1.10369600
H	-4.47984200	3.94842300	2.26109100
H	-4.12134600	5.94567700	3.62668300
H	-2.65533300	7.78640300	4.40224600
H	-0.34052500	7.99065000	3.49803500
H	0.47895900	6.41666100	1.80349000
H	4.03289400	4.67042000	-1.39370600
H	-1.10853400	6.62043400	-0.58797400
H	-0.37640400	8.58736000	-1.86361000
H	1.96259500	8.74638300	-2.71216800
H	3.54696600	6.88424600	-2.31287100
H	6.34338000	-0.78783000	-2.26255000
H	5.35433000	-0.33808400	-4.45182900
H	3.49563300	1.31646200	-4.68001500
H	2.66499800	2.51735300	-2.70784600
H	2.89938900	3.47103200	2.15902600
H	3.84102800	2.99213400	4.36851400
H	5.68892600	1.32638400	4.61253800
H	6.55242900	0.14399000	2.64930900
H	5.65265300	-1.95026400	1.47098900
H	7.45630500	-3.63197400	1.66225300
H	9.64631700	-3.24182400	0.54729200
H	10.00827400	-1.15055000	-0.75012400
H	8.18879500	0.51893200	-0.94735500
H	-5.58808500	-2.24318500	1.56850000
H	-4.27028600	-2.36751900	3.64360200
H	-2.78919100	-0.47053500	4.30559100
H	-2.63527200	1.53025900	2.89472300
H	-3.94949000	3.99308600	-1.21805600
H	-5.25847800	4.11250100	-3.29385800
H	-6.77987300	2.23573300	-3.93121000
H	-6.96693600	0.25187800	-2.49028200
H	-4.78833900	-2.49592100	-1.29210400
H	-6.14441900	-4.43049200	-2.02913700

H	-8.61971600	-4.39288000	-1.75770000
H	-9.71712100	-2.40224100	-0.75035800
H	-8.34601300	-0.46512100	-0.02170600
C	-1.35665600	5.27141600	1.69664900
C	-2.66838200	5.12991000	2.25188300
C	-2.17411800	-2.90803500	-2.02912700
C	-3.35532700	-0.57257400	-3.05741000
H	-2.81356200	0.33677300	-1.18876700
C	-2.74628700	-2.89870300	-3.30241800
C	-3.34413000	-1.74306100	-3.81317700
H	-3.80364000	0.33455200	-3.44806900
H	-2.70215400	-3.80271700	-3.90309400
H	-3.78492100	-1.75728300	-4.80602100
C	-1.41335900	-4.08482000	-1.53052200
C	-0.03967600	-4.15516600	-1.79664300
C	-1.99105700	-5.07830300	-0.72624400
C	0.74840300	-5.15666400	-1.24096800
H	0.42565200	-3.39394000	-2.41136000
C	-1.21559300	-6.09259100	-0.17340100
H	-3.05361900	-5.03239700	-0.50412100
C	0.16206900	-6.11585500	-0.40932700
H	1.81943700	-5.16891300	-1.43214100
H	-1.64863700	-6.84001100	0.48220100
C	-1.74082900	3.26180000	0.38952900
C	-0.47103200	-4.30544900	2.46751700
C	-3.04217100	3.09975800	0.94077400
C	-1.81000800	-4.56088300	2.83184400
C	0.55579900	-5.12797500	3.00349100
C	2.25534800	-3.84021200	1.78479300
C	-2.11486300	-5.59769600	3.69256500
H	-2.59143100	-3.92817400	2.42245800
C	0.21748200	-6.18773000	3.87466500
C	1.90627400	-4.86485900	2.62001600
H	3.29514100	-3.68301600	1.52555500
C	-1.09851000	-6.42376300	4.21717600
H	-3.15027800	-5.78083500	3.96681100
H	1.01226900	-6.81678900	4.26769400
H	2.68465200	-5.51073500	3.02024800
H	-1.35300100	-7.23948000	4.88698400
O	0.89510900	-7.09118400	0.20912000
H	1.83271000	-6.93177400	0.02032600
N	1.56201100	-1.92483300	0.46476800
H	0.79642200	-1.38708300	0.06792800
C	2.87420700	-1.52037000	-0.01935100
H	2.83630000	-0.43419400	-0.10775800
H	3.63220600	-1.76014100	0.72628200

C	3.20293800	-2.14079300	-1.35956600
C	4.20923000	-3.10006100	-1.49108100
C	2.46774600	-1.75345100	-2.48670200
C	4.47943700	-3.67609400	-2.73474100
H	4.80445900	-3.37993300	-0.62590800
C	2.73479200	-2.33119900	-3.72567400
H	1.70089800	-0.99085600	-2.38257000
C	3.73794900	-3.29598400	-3.85338600
H	5.27043000	-4.41543400	-2.82831000
H	2.16421800	-2.02115900	-4.59682200
H	3.94449500	-3.74350700	-4.82175900
C	-3.48375100	4.04786800	1.83876000
C	-1.06750000	-2.58041900	0.81201300
C	-2.80631600	-0.56956800	-1.77780500

TS14-S2

O	-0.77419900	0.59861700	1.24086400
H	-1.43453600	-0.31945800	0.76010800
C	-1.83545300	-1.47388000	0.11266400
C	-0.79788400	-3.08569700	1.88078300
C	0.63448500	-3.00360300	1.80614200
C	-2.16022400	-1.47477000	-1.30557300
C	-2.64989700	6.11508000	3.41677700
C	-1.78160400	6.98056900	4.03919900
C	-0.41389400	6.98726800	3.67686500
C	0.05485900	6.15141200	2.68900600
C	1.58043800	5.57754900	-0.13695400
C	2.93141100	5.53529300	-0.60922100
C	3.65057000	4.31859800	-0.54534100
C	3.08922200	3.15992300	-0.04669000
C	1.76960300	3.23460800	0.46893900
C	1.01809900	4.39996400	0.45624600
C	0.84513900	6.78104600	-0.31569100
C	1.42974000	7.89021100	-0.88302000
C	2.77907100	7.85883500	-1.30830100
C	3.50895500	6.70143400	-1.17871200
C	5.38058100	-0.16114800	-2.89833800
C	4.72073400	0.34283100	-3.98634500
C	3.70681500	1.32535800	-3.82118100
C	3.42634700	1.82083400	-2.57718600
C	4.11226200	1.34333600	-1.41522100
C	3.86286200	1.88785600	-0.13727100
C	4.41951400	1.28413100	1.00622400
C	4.11760800	1.75258400	2.32300100
C	4.59718900	1.11217300	3.43287000
C	5.42380400	-0.03645200	3.29386200

C	5.77451300	-0.49179900	2.05071800
C	5.30963400	0.15436500	0.86028000
C	5.67563500	-0.29897700	-0.42864300
C	5.07048200	0.27377700	-1.57015400
C	6.69934400	-1.37092800	-0.57444500
C	6.39364200	-2.59079400	-1.19538300
C	7.35835000	-3.58939100	-1.31777700
C	8.64890400	-3.38531300	-0.82786100
C	8.96845500	-2.17205000	-0.21532000
C	8.00220900	-1.17512200	-0.08973800
C	-5.32160300	-0.78702600	1.26757700
C	-4.82788500	-0.96961700	2.53061800
C	-3.88544000	-0.05335200	3.07366000
C	-3.48374400	1.03589400	2.35053700
C	-3.98695600	1.27940700	1.03279600
C	-3.57991100	2.39528000	0.27228700
C	-4.09708300	2.60041100	-1.02338800
C	-3.70028800	3.71830500	-1.82206400
C	-4.17823800	3.88966800	-3.09334900
C	-5.08953100	2.95020000	-3.65006400
C	-5.50154700	1.86924700	-2.91715400
C	-5.03347600	1.65425300	-1.58259000
C	-5.42306700	0.52546500	-0.83302700
C	-4.92056400	0.33166200	0.46919200
C	-6.32817300	-0.49446900	-1.43722400
C	-5.80396600	-1.71307900	-1.89151800
C	-6.63807400	-2.67196000	-2.46408500
C	-8.00712100	-2.42830200	-2.58887000
C	-8.53746000	-1.21828100	-2.13870000
C	-7.70249800	-0.25724700	-1.56730000
O	-0.80316700	2.51781300	-0.46754100
O	1.21835500	2.08708100	1.02027600
P	0.08853500	1.27120500	0.15164200
C	-0.37484200	4.37504100	0.97485200
O	0.65410800	0.43122500	-0.93481300
H	-4.11442400	4.28123500	2.09575600
H	-3.69933600	6.08430600	3.69829100
H	-2.13849300	7.64972000	4.81671900
H	0.27425100	7.65476200	4.18761300
H	1.10592600	6.15881800	2.42650800
H	4.66160800	4.28498900	-0.94103200
H	-0.19125900	6.81746500	-0.00382200
H	0.84685300	8.79781100	-1.01143300
H	3.22834300	8.74399300	-1.74907700
H	4.53894400	6.65320400	-1.52260000
H	6.14584700	-0.91458100	-3.03391500

H	4.96772700	-0.01474600	-4.98213300
H	3.15821900	1.68730000	-4.68582500
H	2.65325700	2.56818600	-2.45072400
H	3.47418200	2.61781400	2.43000000
H	4.33686200	1.47275400	4.42372800
H	5.77995900	-0.55312200	4.18080300
H	6.40584100	-1.36686500	1.95200300
H	5.39610600	-2.75474400	-1.57950700
H	7.09451400	-4.52876400	-1.79607900
H	9.40128700	-4.16320100	-0.92440400
H	9.97244200	-1.99955100	0.16273600
H	8.25201500	-0.23093300	0.38530200
H	-6.03724600	-1.48703600	0.85262900
H	-5.15403300	-1.81809000	3.12557100
H	-3.47918300	-0.22519800	4.06600000
H	-2.74993500	1.71868700	2.75764300
H	-3.00246800	4.43386000	-1.40234400
H	-3.85800100	4.74378000	-3.68318100
H	-5.45578600	3.09137200	-4.66309800
H	-6.18561300	1.14676400	-3.34653300
H	-4.74093100	-1.89977700	-1.79869900
H	-6.21355900	-3.60765400	-2.81798400
H	-8.65705700	-3.17586600	-3.03529100
H	-9.60214500	-1.02159700	-2.23092300
H	-8.11252700	0.68568900	-1.21646000
C	-0.82048400	5.26322700	2.00757900
C	-2.19555700	5.23029000	2.40337400
C	-2.15572400	-2.70022200	-2.02277500
C	-2.74509700	-0.28476600	-3.33614700
H	-2.40715300	0.65291400	-1.43441700
C	-2.47579400	-2.68694700	-3.38149700
C	-2.77761200	-1.48952500	-4.03597700
H	-2.97269000	0.65169700	-3.83414800
H	-2.47637400	-3.62508200	-3.92905800
H	-3.02823300	-1.50109500	-5.09300200
C	-1.75931900	-3.95468100	-1.32955000
C	-0.41491400	-4.34808900	-1.31369800
C	-2.68917000	-4.71338400	-0.60112800
C	-0.00434600	-5.44385600	-0.56010700
H	0.32153200	-3.77137200	-1.86333300
C	-2.29064500	-5.81610300	0.14595300
H	-3.73294300	-4.41128100	-0.59923200
C	-0.93852200	-6.16629900	0.18731200
H	1.04733000	-5.71695600	-0.53848200
H	-2.99939700	-6.38356700	0.73867300
C	-1.26586500	3.44352100	0.46464700

C	-1.43788800	-3.99210500	2.80290700
C	-2.63225800	3.39011400	0.85390100
C	-2.84095300	-4.05229300	2.93598900
C	-0.63703100	-4.87575500	3.57513700
C	1.40146100	-3.91431600	2.59987500
C	-3.42360600	-4.95559200	3.80361900
H	-3.44929300	-3.37811600	2.34251700
C	-1.25712000	-5.79709900	4.44932900
C	0.78181500	-4.80567200	3.43127800
H	2.48279900	-3.88877200	2.54647200
C	-2.63135100	-5.83996500	4.56577400
H	-4.50578200	-4.98775900	3.89774300
H	-0.63189900	-6.47294100	5.02752600
H	1.38932800	-5.48731000	4.02259700
H	-3.10292300	-6.54929800	5.23898700
O	-0.58084900	-7.21949700	0.98347100
H	0.38563400	-7.29530100	0.97355900
N	1.20722600	-2.07121500	1.02938500
H	0.58700200	-1.49444100	0.46885000
C	2.60970800	-1.94684800	0.64748800
H	2.79078700	-0.88280900	0.49446200
H	3.25390500	-2.26947700	1.46738700
C	2.88829700	-2.71419300	-0.62582000
C	3.33442400	-4.03926800	-0.58880600
C	2.63770800	-2.10669300	-1.86217000
C	3.53239200	-4.75319400	-1.77163000
H	3.54215000	-4.51024700	0.36858200
C	2.84534400	-2.81680500	-3.04472900
H	2.26978600	-1.08520300	-1.88984700
C	3.29019400	-4.14050400	-3.00321900
H	3.88721100	-5.77973300	-1.73336300
H	2.66405100	-2.32994700	-3.99803200
H	3.45118200	-4.69145300	-3.92556300
C	-3.07114900	4.29911800	1.79301000
C	-1.50802900	-2.35116200	0.96177100
C	-2.43666700	-0.28075300	-1.97868500

TS14-S3

O	0.07790000	-0.75934400	0.78133000
H	1.20505500	-0.49264600	0.31341700
C	2.22386600	0.20211800	-0.24358400
C	2.63669000	1.83332000	1.77466500
C	1.45333000	2.52410900	2.17217700
C	2.41129800	0.07580000	-1.67788400
C	-1.54799700	-6.53353500	3.31283800
C	-2.77677400	-6.77153800	3.88161300

C	-3.90388100	-6.03046300	3.45526100
C	-3.78979500	-5.09005900	2.45673700
C	-4.66446900	-3.74890800	-0.37135500
C	-5.75199700	-2.93857100	-0.83310900
C	-5.66144900	-1.52797900	-0.73995200
C	-4.53818700	-0.90224300	-0.24018000
C	-3.48392100	-1.72382900	0.23441800
C	-3.52702500	-3.10920400	0.22201400
C	-4.74540900	-5.15478900	-0.56131700
C	-5.85714600	-5.72895000	-1.13458400
C	-6.94783200	-4.93035400	-1.55234000
C	-6.88993500	-3.56439100	-1.40817300
C	-4.41110100	3.37833700	-2.75112200
C	-4.25309300	2.67589300	-3.91503500
C	-4.10136300	1.26240000	-3.88648000
C	-4.15956000	0.58628000	-2.69883800
C	-4.35004400	1.27656900	-1.45989700
C	-4.43765600	0.58525300	-0.23338700
C	-4.47100700	1.30112100	0.98049300
C	-4.48612300	0.63767500	2.24779900
C	-4.44801100	1.34057700	3.42156800
C	-4.40711200	2.76173400	3.40440500
C	-4.43187700	3.43887300	2.21457400
C	-4.47949300	2.74573100	0.96223800
C	-4.50724100	3.43738200	-0.26884100
C	-4.42536100	2.71926600	-1.48077200
C	-4.61143400	4.92340600	-0.27727400
C	-3.55666300	5.71465300	-0.75174400
C	-3.65214900	7.10526700	-0.74202800
C	-4.80547200	7.72790600	-0.26257300
C	-5.86477400	6.94910700	0.20726300
C	-5.76742900	5.55810800	0.20083900
C	4.37758800	-1.97972800	1.68939700
C	3.89563900	-1.56568100	2.90085800
C	2.57554600	-1.90820800	3.30083800
C	1.78294900	-2.66882900	2.48611900
C	2.24930600	-3.13418700	1.21505400
C	1.42497500	-3.88397000	0.35059700
C	1.88989400	-4.26025200	-0.92464500
C	1.06559400	-4.99153100	-1.83569000
C	1.51614200	-5.33821900	-3.08085300
C	2.83453000	-4.99249500	-3.48744000
C	3.65895100	-4.29775800	-2.64354200
C	3.22166000	-3.89104200	-1.34377900
C	4.05039100	-3.14448100	-0.47979800
C	3.58286600	-2.76748700	0.79728300

C	5.39807800	-2.69678000	-0.93440300
C	5.51238700	-1.72382600	-1.93802900
C	6.76567300	-1.26951400	-2.34631100
C	7.92478500	-1.78061600	-1.75951400
C	7.82181000	-2.75587100	-0.76616200
C	6.56764100	-3.21087800	-0.35792600
O	-0.92044500	-2.64805300	-0.66687800
O	-2.35542000	-1.09223300	0.73934400
P	-1.04406500	-1.06186000	-0.24793300
C	-2.37795800	-3.87587600	0.77643800
O	-1.18968400	-0.24025100	-1.46980000
H	0.75234300	-5.78894700	2.13392400
H	-0.66802600	-7.07781900	3.64592800
H	-2.88281300	-7.51487400	4.66637400
H	-4.86847500	-6.20089500	3.92482000
H	-4.65929300	-4.52348900	2.14691500
H	-6.48593400	-0.92242700	-1.10542300
H	-3.91269300	-5.77591800	-0.25431300
H	-5.89489800	-6.80564000	-1.27333800
H	-7.82109100	-5.39765200	-1.99808500
H	-7.71074600	-2.93610200	-1.74430800
H	-4.51619900	4.45587400	-2.77766800
H	-4.23489500	3.20047400	-4.86636700
H	-3.93498500	0.71869500	-4.81183300
H	-4.02544200	-0.48747500	-2.67740900
H	-4.51144200	-0.44533500	2.26722000
H	-4.44216500	0.81208500	4.37048600
H	-4.35887500	3.31037200	4.34096100
H	-4.40067800	4.52185800	2.20439000
H	-2.66295700	5.23440600	-1.12648500
H	-2.81923000	7.69942300	-1.10822600
H	-4.88040600	8.81187400	-0.25595700
H	-6.76898200	7.42450600	0.57760700
H	-6.59024400	4.95114000	0.56763200
H	5.37590800	-1.69669100	1.37895100
H	4.51285400	-0.94804900	3.54606700
H	2.19081700	-1.55037000	4.25136300
H	0.76815100	-2.90207300	2.78145000
H	0.06468200	-5.26583000	-1.52237500
H	0.86932000	-5.88700500	-3.75938800
H	3.18862200	-5.28710200	-4.47135400
H	4.66404800	-4.03997200	-2.95528300
H	4.61555800	-1.32591700	-2.39439300
H	6.83171700	-0.51649700	-3.12729200
H	8.90140700	-1.42575500	-2.07689000
H	8.71855600	-3.16538400	-0.30916100

H	6.48685400	-3.96835700	0.41658300
C	-2.54120200	-4.84087800	1.82509000
C	-1.39311100	-5.56056500	2.29007100
C	3.19824600	1.02807500	-2.37643900
C	2.13730300	-1.21332900	-3.71601700
H	1.29237400	-1.75647800	-1.82292500
C	3.45581100	0.81335300	-3.73364400
C	2.93354200	-0.29652300	-4.40199300
H	1.73135200	-2.08395700	-4.21982100
H	4.06275900	1.53764700	-4.26930400
H	3.14723700	-0.43995900	-5.45750700
C	3.76040800	2.21092700	-1.67835700
C	2.91827300	3.21240900	-1.17512600
C	5.14436400	2.34024900	-1.48229400
C	3.43822800	4.29470000	-0.47294500
H	1.84614300	3.13501500	-1.31830300
C	5.67375600	3.41983000	-0.78401600
H	5.80630900	1.56143100	-1.85001600
C	4.81613900	4.39113000	-0.26052000
H	2.76470100	5.04860200	-0.07322200
H	6.73906100	3.50514600	-0.59867700
C	-1.09675200	-3.60676100	0.32043200
C	3.92656000	2.20373200	2.31035500
C	0.05887600	-4.27494100	0.80500600
C	5.11053900	1.51370000	1.97498700
C	4.01639200	3.35711600	3.13995600
C	1.59026300	3.68727600	2.98829800
C	6.33036500	1.93525700	2.46554700
H	5.04662400	0.65814900	1.31398700
C	5.27974700	3.77296500	3.62125600
C	2.82138900	4.07670300	3.44159400
H	0.70513800	4.24112500	3.27774500
C	6.42208300	3.07281600	3.29420300
H	7.23119600	1.39006600	2.19814800
H	5.33462700	4.65795200	4.25053700
H	2.90031200	4.95726100	4.07545900
H	7.38913400	3.39843700	3.66493300
O	5.37840100	5.41179200	0.45551200
H	4.66520700	5.94156900	0.84348800
N	0.23925400	2.04407800	1.81327000
H	0.22346700	1.13092600	1.36140200
C	-0.94074400	2.86588500	1.52740600
H	-1.79048200	2.18240300	1.48338400
H	-1.13017900	3.55630300	2.35327500
C	-0.76870700	3.60492300	0.21572200
C	-0.26178200	4.90905600	0.18679300

C	-1.02509600	2.94397500	-0.99162500
C	-0.00483000	5.54318200	-1.03048400
H	-0.07147800	5.43356100	1.11945200
C	-0.77777200	3.58003500	-2.20829400
H	-1.39939800	1.92517400	-0.99487800
C	-0.26229600	4.87849400	-2.23156100
H	0.38691600	6.55682700	-1.04120800
H	-0.99294400	3.05352700	-3.13260800
H	-0.06454600	5.37135700	-3.17919600
C	-0.11672300	-5.25888100	1.75429800
C	2.54798000	0.96858900	0.70104600
C	1.88144200	-1.02642500	-2.36110800

TS14-S4

O	0.58063200	-1.51341100	0.18443900
H	1.21607100	-0.68055100	-0.52373000
C	1.71414700	-0.08236800	-1.59331100
C	0.16220700	2.01252700	-1.75964200
C	-1.15060500	1.60567600	-2.17398100
C	2.56869700	-0.93706100	-2.40811800
C	2.99894300	4.78147600	2.01201000
C	2.17290000	5.87493200	1.89962300
C	0.76956700	5.69856700	1.87526800
C	0.21865000	4.44514100	2.01265300
C	-1.47476400	2.46787100	3.87607100
C	-2.87031700	2.32150600	4.15233300
C	-3.65219600	1.48498000	3.32148800
C	-3.10268600	0.75967200	2.28658400
C	-1.70866500	0.89835800	2.03057500
C	-0.91208800	1.78269100	2.74403400
C	-0.69829100	3.25302800	4.77193900
C	-1.27952100	3.88893400	5.84538400
C	-2.66877800	3.77856900	6.08685800
C	-3.44388400	3.00324300	5.25780500
C	-5.49723200	-3.52094300	1.35611000
C	-4.89912600	-4.08277500	2.45211200
C	-3.98984400	-3.32929200	3.24342800
C	-3.70343200	-2.03078700	2.92213000
C	-4.30173800	-1.40369900	1.78677600
C	-4.00750400	-0.07128500	1.43834500
C	-4.64916400	0.53471700	0.33920600
C	-4.42138100	1.90449000	-0.00605700
C	-5.07150400	2.49153100	-1.05915200
C	-5.96570900	1.73398100	-1.86485900
C	-6.19769500	0.41347900	-1.58161800
C	-5.57492700	-0.22987800	-0.46455100

C	-5.83451200	-1.57783800	-0.14436900
C	-5.22441700	-2.16880500	0.97986300
C	-6.73977500	-2.38517300	-1.01109600
C	-6.19904200	-3.32644700	-1.89745200
C	-7.03381500	-4.08007600	-2.72152700
C	-8.41784000	-3.90471200	-2.67026100
C	-8.96423500	-2.97112400	-1.78805800
C	-8.12968200	-2.21591400	-0.96362100
C	5.98460700	-1.40832500	-0.78481600
C	6.05108000	-0.33114600	-1.62384500
C	5.29158400	0.83277400	-1.33832300
C	4.49568200	0.88612200	-0.22817400
C	4.43784700	-0.18911300	0.71497900
C	3.66213800	-0.13376200	1.90049900
C	3.70661600	-1.20431100	2.82011600
C	3.07738500	-1.12499900	4.10098100
C	3.10626500	-2.17545000	4.97639800
C	3.77304100	-3.38186000	4.62838300
C	4.41339100	-3.49309800	3.42439600
C	4.42052400	-2.41224800	2.48618800
C	5.13165200	-2.49438300	1.27215500
C	5.20006900	-1.37952900	0.41255500
C	5.78659100	-3.77535300	0.88162600
C	5.00140800	-4.84164400	0.41956600
C	5.59229800	-6.04262900	0.02801500
C	6.97845100	-6.19551600	0.09577100
C	7.76788700	-5.14317900	0.56140000
C	7.17540000	-3.94196200	0.95253400
O	0.87157300	-0.36941000	2.49558100
O	-1.13369300	0.14741500	1.01299000
P	-0.16650800	-1.13740700	1.47589500
C	0.51411300	1.98310200	2.38192700
O	-0.89281400	-2.18989200	2.21474000
H	4.37009100	2.46828900	2.08902600
H	4.07986300	4.89809900	1.99900200
H	2.59378800	6.87188800	1.80563300
H	0.12193100	6.55965800	1.74047500
H	-0.85752400	4.31885700	1.99648400
H	-4.71794100	1.39857800	3.51433800
H	0.36895400	3.33789100	4.61317400
H	-0.66176000	4.47618800	6.51881100
H	-3.11441300	4.29184300	6.93400700
H	-4.50892900	2.88658400	5.44116100
H	-6.19195000	-4.09589100	0.75541300
H	-5.11930700	-5.11225400	2.72100500
H	-3.50764100	-3.79153700	4.09916400

H	-2.98836500	-1.47072300	3.50848600
H	-3.72331600	2.48026400	0.59025000
H	-4.89107700	3.53766500	-1.28983500
H	-6.46330300	2.20460900	-2.70836400
H	-6.87129000	-0.16689200	-2.20119800
H	-5.12494500	-3.46379400	-1.93385500
H	-6.59918800	-4.80437000	-3.40518500
H	-9.06758600	-4.49294900	-3.31243000
H	-10.04077700	-2.83176900	-1.73925900
H	-8.55166800	-1.48919100	-0.27502300
H	6.52989800	-2.31342900	-1.02159300
H	6.65501100	-0.37829800	-2.52489900
H	5.31357700	1.67753700	-2.01847700
H	3.89126700	1.76400700	-0.05586600
H	2.57751100	-0.20499600	4.37827700
H	2.61893300	-2.08877700	5.94305300
H	3.78542400	-4.21143100	5.32962600
H	4.94503100	-4.40269600	3.17015700
H	3.92265200	-4.72037100	0.37605800
H	4.96997000	-6.85914400	-0.32787700
H	7.43998600	-7.13058600	-0.20891700
H	8.84669700	-5.25725300	0.62293700
H	7.78815800	-3.12158300	1.31510800
C	1.04246500	3.29936500	2.16995200
C	2.46069500	3.47186200	2.12068600
C	3.06802100	-0.50070700	-3.66412100
C	3.75823500	-3.03978800	-2.65355400
H	2.53014500	-2.51868900	-0.95929400
C	3.92667000	-1.34832900	-4.36994700
C	4.26822100	-2.60834900	-3.87648200
H	4.03279400	-4.01122200	-2.25443700
H	4.31308500	-1.01577400	-5.32931100
H	4.93237800	-3.24988600	-4.44896300
C	2.72240800	0.83892600	-4.20158600
C	1.39788700	1.18368000	-4.49848400
C	3.71150200	1.82096200	-4.37437600
C	1.05657000	2.48048700	-4.87288600
H	0.61833000	0.43420300	-4.40079300
C	3.38431400	3.12152900	-4.73940400
H	4.74811900	1.56239000	-4.18455800
C	2.04435500	3.46504800	-4.94792200
H	0.01616900	2.73829500	-5.05718900
H	4.14354500	3.89161800	-4.82730200
C	1.37132200	0.89723800	2.29690000
C	0.50821100	3.41298700	-1.69135300
C	2.78478400	1.04406100	2.13758100

C	1.80240200	3.84502100	-1.34056800
C	-0.45793600	4.38407000	-2.07501100
C	-2.09022700	2.61251500	-2.55184100
C	2.12568600	5.18642300	-1.36621300
H	2.54398600	3.10238000	-1.07055200
C	-0.10703200	5.75464200	-2.07874200
C	-1.74755300	3.93500200	-2.49248100
H	-3.08068300	2.31801300	-2.87139200
C	1.16791300	6.15317000	-1.73332600
H	3.12729400	5.49952800	-1.09161300
H	-0.85785900	6.48690200	-2.36662500
H	-2.48115700	4.68427800	-2.78253000
H	1.43579400	7.20512800	-1.74453300
O	1.76152000	4.77274900	-5.22316700
H	0.80725900	4.91137300	-5.11967200
N	-1.46129400	0.30260500	-2.23887700
H	-0.78601600	-0.35120800	-1.85897400
C	-2.73336700	-0.27814800	-2.66962800
H	-3.55333600	0.21066500	-2.13710600
H	-2.88162300	-0.10207900	-3.74304700
C	-2.71676900	-1.75848400	-2.37085400
C	-2.81087500	-2.71217600	-3.38740400
C	-2.55735600	-2.18229300	-1.04727900
C	-2.74906200	-4.07321000	-3.08004500
H	-2.92991400	-2.39171800	-4.41962600
C	-2.48375200	-3.53727500	-0.73602900
H	-2.48901400	-1.44763500	-0.25613200
C	-2.58195500	-4.48625700	-1.75596900
H	-2.82351100	-4.80928100	-3.87584900
H	-2.34578900	-3.83119200	0.29922600
H	-2.52704900	-5.54555700	-1.52108600
C	3.29434900	2.32841600	2.13776200
C	1.11661000	1.02867200	-1.67008400
C	2.91818700	-2.20486500	-1.92199600

TS15

C	-4.88320500	1.88376900	-0.25328400
C	-4.90436700	1.40143200	1.07334300
C	-3.70196800	0.80336500	1.68589600
C	-2.24934900	1.06000500	1.14674800
C	-3.57222400	0.17091900	2.89338800
C	-6.10928600	1.70338100	1.83720700
C	4.93792900	2.30030900	0.32553200
C	4.37463400	4.02278800	-1.34477800
C	2.44058900	5.57554700	-0.97585400
C	5.43799800	0.92590500	0.60722800

C	5.15638000	2.88322700	-0.93657800
C	4.59635800	4.56930400	-2.64778900
C	1.33639200	5.16537800	-1.73835900
C	2.60771200	6.93599900	-0.69345800
C	4.77564300	-0.15024000	-0.04798800
C	6.37078000	0.60377900	1.56595900
C	6.10907200	2.34517100	-1.85496200
C	5.52721400	4.02895100	-3.49429100
H	4.01077100	5.42907600	-2.95336200
C	0.41644000	6.10493600	-2.20327800
H	1.19572200	4.11025800	-1.95510400
C	1.68575400	7.87470300	-1.16088600
H	3.46256300	7.25445300	-0.10326100
C	4.92839300	-1.47818600	0.29252800
O	3.91059100	0.16111200	-1.09217400
C	6.59414500	-0.74166400	1.96285400
H	6.90433200	1.39869500	2.07963800
C	6.29344000	2.89983300	-3.09282500
H	6.69559500	1.48588600	-1.54611100
H	5.68362900	4.46191000	-4.47830000
C	0.58749900	7.46113300	-1.91570200
H	-0.43387800	5.77318200	-2.79344300
H	1.82683100	8.92830300	-0.93516900
C	5.83071600	-1.79864300	1.36529700
C	4.07839500	-2.50443800	-0.37090400
P	2.32391000	0.22828000	-0.76631400
C	7.51232800	-1.04994100	3.00063600
H	7.02760000	2.47859000	-3.77388100
H	-0.12982300	8.19191800	-2.27935200
C	5.97577900	-3.10961000	1.89187800
C	2.69751700	-2.34988000	-0.39748400
C	4.63658600	-3.67650100	-0.98175200
O	2.10694900	-1.17472900	0.05010000
O	1.83257500	1.36094100	0.03886400
O	1.73274000	0.05714000	-2.22254000
C	7.65447400	-2.33769900	3.46239600
H	8.09148800	-0.23910200	3.43496500
C	6.86505400	-3.37197900	2.91024100
H	5.37319300	-3.91200600	1.48522900
C	1.81242600	-3.38424300	-0.80038600
C	3.75922500	-4.70367200	-1.45566500
C	6.03421200	-3.84275800	-1.17535700
H	8.35757400	-2.55928300	4.25999200
H	6.95614000	-4.38335300	3.29589900
C	2.35924900	-4.54214300	-1.31054100
C	0.33591000	-3.28422300	-0.59436500

C	4.30956200	-5.86709100	-2.05517400
C	6.53567600	-4.97600200	-1.77416200
H	6.70611600	-3.05536600	-0.85522200
H	1.69946400	-5.34957900	-1.61528600
C	-0.52646500	-3.11009700	-1.69282900
C	-0.19307900	-3.45398500	0.70220900
C	5.66879400	-6.00573800	-2.20965300
H	3.63031400	-6.64255800	-2.39964400
H	7.60721800	-5.07777900	-1.91969000
C	-0.01445100	-2.81326700	-2.99626800
C	-1.95953000	-3.17696400	-1.50549500
C	0.65503100	-3.55131700	1.84973200
C	-1.62330400	-3.54810700	0.89103100
H	6.07889700	-6.89710700	-2.67527000
C	-0.85287700	-2.58774100	-4.05418000
H	1.05829100	-2.73068200	-3.12196100
C	-2.79381800	-2.93646200	-2.64457000
C	-2.49392000	-3.42823400	-0.21872600
C	0.13907300	-3.72190800	3.10442800
H	1.72683100	-3.47329800	1.71368000
C	-2.11122200	-3.78096900	2.21663800
C	-2.26155500	-2.65324100	-3.87441300
H	-0.44304000	-2.34301900	-5.02961300
H	-3.86922000	-2.95193400	-2.51908600
C	-3.97068400	-3.51793300	-0.03325900
C	-1.26319300	-3.85140100	3.28789700
H	0.80215900	-3.76709200	3.96301400
H	-3.17693200	-3.89980300	2.36522600
H	-2.92303700	-2.46353800	-4.71504900
C	-4.64729000	-2.60907900	0.79579000
C	-4.72490100	-4.49113700	-0.70525800
H	-1.66262500	-4.01264000	4.28483900
C	-6.03391800	-2.65255900	0.92842000
H	-4.08423900	-1.85199300	1.32906500
C	-6.11216600	-4.53891800	-0.57070500
H	-4.21517100	-5.20884500	-1.34125800
C	-6.77297300	-3.61566200	0.24067900
H	-6.53471900	-1.92304100	1.55980900
H	-6.67767900	-5.29750300	-1.10445700
H	-7.85459600	-3.64615800	0.33442700
C	2.83497600	2.86219700	3.36574200
C	2.05255300	3.97392200	2.95128800
C	2.24912100	4.52799600	1.71637300
H	1.29210300	4.37420300	3.61562000
H	1.05111900	0.78507400	-2.41230900
C	3.23852200	4.01599900	0.81910200

C	0.00753600	0.69525500	2.49130800
C	0.56233100	0.13114000	3.64681100
C	-0.24630900	-0.46945700	4.61855400
C	-1.63295200	-0.51733300	4.46522800
C	-2.19346500	0.04834500	3.31681300
C	-1.37078500	0.64841400	2.35269200
C	3.39324600	4.54193700	-0.47729100
H	2.66611600	2.42451100	4.34519600
C	4.02822100	2.88084600	1.23558300
C	-1.80933800	0.04317100	0.13498200
C	-1.07018600	0.34841100	-0.94048700
C	-0.73116400	1.73045700	-1.25455600
C	-1.23325900	2.77506100	-0.35755900
C	-1.95578900	2.46789000	0.73274400
O	-0.03179900	2.01075900	-2.25320300
C	3.78057800	2.32930900	2.53228000
H	4.35143600	1.46530100	2.85036500
C	-6.97588000	2.76314100	1.39936300
C	-6.72657100	3.40811600	0.16007400
C	-5.75265800	2.92757200	-0.66692000
H	1.64580300	5.36805300	1.39234300
C	-6.53439700	0.99438700	2.99247100
C	-7.66035600	1.35343300	3.70749200
C	-8.44255000	2.45725700	3.31952300
C	-8.10420500	3.13471700	2.17042700
N	-4.01488300	1.34461800	-1.20458200
C	-4.23298500	-0.06097000	-1.60418000
C	-5.50967000	-0.29403800	-2.37694900
C	-6.74814300	-0.34097500	-1.72211900
C	-7.92366200	-0.53091000	-2.44523000
C	-7.87875200	-0.67985900	-3.83353100
C	-6.65117700	-0.63730700	-4.49478800
C	-5.47553200	-0.44417700	-3.76765000
H	0.64189400	1.13948600	1.73223500
H	1.63944900	0.15739500	3.77620700
H	0.21075000	-0.90746000	5.50185800
H	-2.26152600	-0.98662000	5.21723000
H	-4.36772000	-0.17730800	3.52915500
H	-0.72819800	-0.42373700	-1.61896400
H	-0.96875700	3.79879800	-0.60171400
H	-2.30306700	3.24702200	1.40621300
H	-7.37335200	4.22310300	-0.15288100
H	-5.63648300	3.32113700	-1.67357100
H	-6.02480400	0.09447400	3.29253500
H	-7.95061600	0.76115800	4.57088700
H	-9.31641100	2.74390900	3.89668800

H	-8.71790300	3.95814400	1.81352900
H	-3.96469200	1.93431500	-2.02992100
H	-3.37035900	-0.36461300	-2.19777200
H	-4.22575600	-0.66670100	-0.69964000
H	-6.78599900	-0.23374800	-0.64307000
H	-8.87675200	-0.56782600	-1.92463500
H	-8.79602600	-0.83219700	-4.39558500
H	-6.60800600	-0.75713900	-5.57396400
H	-4.51654500	-0.41614700	-4.28018300
H	-2.04596300	-0.98839300	0.36509000

TS16

C	4.01481900	1.75328800	2.61633100
C	4.16740300	1.63326400	1.17749000
C	3.12025700	2.00360100	0.20193300
C	1.66779600	2.53206100	0.51538300
C	3.15671000	1.73362200	-1.14714600
C	5.35922400	0.98216800	0.74324900
H	3.83962100	-1.53719200	1.98274300
C	0.07032900	-2.38661300	2.39505700
H	0.09001900	-0.97028600	3.98603700
C	0.81155100	-3.06720500	1.37758800
C	2.98393600	-3.47402600	0.27266100
H	-0.98980800	-2.58779000	2.49214300
C	0.19496500	-3.97526900	0.49470200
C	2.36216600	-4.40659600	-0.59207800
C	4.45496900	-3.26076500	0.17497400
C	-1.27292500	-4.19378000	0.62894400
C	0.93956600	-4.63361500	-0.49636500
C	3.09302000	-5.17458600	-1.55518400
C	5.04006800	-2.78092200	-1.00651300
C	5.29712600	-3.60424000	1.24328400
C	-2.17778600	-3.35880400	-0.07409700
C	-1.79263100	-5.08911800	1.53569600
C	0.32026000	-5.54616700	-1.40604100
C	2.46380500	-6.05032000	-2.39958400
H	4.16876400	-5.06122100	-1.60639300
C	6.42571600	-2.69477900	-1.13468000
H	4.39714900	-2.50132000	-1.83583900
C	6.68216500	-3.49581200	1.12699700
H	4.85479600	-3.97395900	2.16328700
C	-3.53632800	-3.31640900	0.18398000
O	-1.64349500	-2.54990600	-1.07648900
C	-3.17667800	-5.11817400	1.84003200
H	-1.11788400	-5.74226100	2.08179500
C	1.05468600	-6.22878700	-2.33672400
H	-0.75246000	-5.69222900	-1.33701600

H	3.04582100	-6.61906500	-3.11945800
C	7.25282400	-3.05389700	-0.06790900
H	6.85732000	-2.34512300	-2.06820800
H	7.31655800	-3.77621000	1.96344100
C	-4.05891900	-4.18340300	1.20610700
C	-4.38683200	-2.29163200	-0.47584300
P	-1.44212700	-0.96345600	-0.80507700
C	-3.68718400	-6.00366700	2.82556200
H	0.56642500	-6.91766100	-3.02019100
H	8.33301200	-2.99202500	-0.16938600
C	-5.40643800	-4.13043900	1.65557200
C	-4.00391600	-0.96149500	-0.41696000
C	-5.61452600	-2.61110800	-1.14470600
O	-2.75433200	-0.62717600	0.10332100
O	-0.19713900	-0.51439100	-0.16380600
O	-1.74146500	-0.42243000	-2.27607300
C	-5.00700500	-5.95262500	3.20649300
H	-3.00399900	-6.71283500	3.28558800
C	-5.86697500	-4.99285500	2.62478200
H	-6.07827500	-3.39553600	1.23175200
C	-4.83616900	0.11331200	-0.82939000
C	-6.46224900	-1.54541900	-1.58716000
C	-6.00916800	-3.94714900	-1.41978300
H	-5.38594200	-6.63144100	3.96484500
H	-6.90114400	-4.92920900	2.95075900
C	-6.06050400	-0.20209200	-1.38071700
C	-4.39389700	1.52179000	-0.64299700
C	-7.68706400	-1.85691000	-2.23476000
C	-7.19507700	-4.21516500	-2.06516600
H	-5.35636000	-4.75908100	-1.12138600
H	-6.72520200	0.59981800	-1.68883200
C	-4.19902500	2.35669200	-1.76292900
C	-4.16230100	2.01480700	0.65985600
C	-8.05065300	-3.16256800	-2.46714400
H	-8.32635700	-1.03815800	-2.55462500
H	-7.47428800	-5.24412400	-2.27244000
C	-4.34555800	1.87733400	-3.10480600
C	-3.79931300	3.73353800	-1.57080000
C	-4.38269400	1.21299300	1.82244000
C	-3.66823300	3.35894600	0.84212000
H	-8.98673700	-3.38877500	-2.96922700
C	-4.19071500	2.71028200	-4.18084900
H	-4.56610200	0.82824400	-3.25877600
C	-3.68833900	4.57565500	-2.72378100
C	-3.50784200	4.20901100	-0.27541900
C	-4.05901600	1.66731200	3.07245400

H	-4.80504600	0.22241700	1.70243300
C	-3.31719900	3.78028500	2.16227700
C	-3.88408000	4.08580300	-3.98705400
H	-4.30252300	2.31963000	-5.18832200
H	-3.43304800	5.61842300	-2.57726800
C	-3.00062800	5.59736800	-0.08023200
C	-3.49123700	2.95781800	3.24224800
H	-4.22317000	1.03153600	3.93801200
H	-2.86943600	4.75469100	2.30203600
H	-3.79040400	4.74386500	-4.84612200
C	-1.72707200	5.96402300	-0.53948900
C	-3.77680800	6.55499600	0.58748100
H	-3.16957900	3.29127300	4.22282700
C	-1.24036300	7.25349400	-0.32787700
H	-1.11520200	5.23078400	-1.05397900
C	-3.29180500	7.84528000	0.79580400
H	-4.76307800	6.27698300	0.94741100
C	-2.02021000	8.19779500	0.34091200
H	-0.24834200	7.51799800	-0.68395700
H	-3.90630600	8.57454700	1.31619400
H	-1.63977000	9.20155300	0.50778200
C	-0.35773400	2.64077800	-1.18295200
C	-0.74245600	2.47299800	-2.52389800
C	0.18364100	2.03624000	-3.48429500
C	1.50547000	1.76229200	-3.12947100
C	1.88738300	1.94558800	-1.79682400
C	0.95729400	2.37834800	-0.84398700
C	0.67171300	-1.48085900	3.22369900
C	2.05118300	-1.17539500	3.07213500
C	2.79704500	-1.80040300	2.10808800
C	0.87974500	1.75169300	1.52890900
C	0.07385200	2.33985500	2.42807200
C	0.02229800	3.80078900	2.60163800
C	0.80399800	4.60471900	1.63736500
C	1.61154900	4.02874400	0.73351800
O	-0.60715500	4.34007200	3.51349800
H	2.50794600	-0.42976800	3.71208200
H	-1.26409600	0.42218300	-2.43824600
C	4.78484000	0.92693600	3.50309800
C	5.78912600	0.08993200	2.96329700
C	6.11087400	0.17183500	1.63743500
C	2.22195700	-2.78766200	1.24501900
C	3.16122500	2.69774000	3.23311400
C	2.97832200	2.74969200	4.59892200
C	3.65940300	1.85675600	5.45086000
C	4.56520700	0.97703400	4.90249300

N	5.80556000	1.10649900	-0.56205500
C	7.11981100	0.66870600	-1.01081800
C	7.20017800	0.76298000	-2.51774000
C	6.19706100	0.18423200	-3.30880400
C	6.26701000	0.24714200	-4.69890100
C	7.34241200	0.88920500	-5.31817500
C	8.34340000	1.46756200	-4.53853900
C	8.26922300	1.40621500	-3.14523400
H	-1.07955500	2.94742300	-0.43341500
H	-1.75665300	2.69783200	-2.82534900
H	-0.13808000	1.91390300	-4.51490200
H	2.22244300	1.41812200	-3.86968200
H	3.97591900	1.29269800	-1.68749600
H	0.91594900	0.67518100	1.42517100
H	-0.53304000	1.75030500	3.10763900
H	0.72759500	5.68301200	1.73790200
H	2.21011200	4.62505000	0.04864900
H	6.34498800	-0.57103100	3.62285000
H	6.93334200	-0.41625500	1.25331500
H	2.68017100	3.44086900	2.62377300
H	2.30276000	3.49317300	5.01239600
H	3.49948600	1.89043300	6.52447800
H	5.14831900	0.31317100	5.53619200
H	5.52908600	1.97729100	-0.99786600
H	7.93426900	1.24697900	-0.54564400
H	7.25358100	-0.37347200	-0.71409000
H	5.36231300	-0.31108600	-2.82186100
H	5.48359500	-0.20621200	-5.29995500
H	7.39678600	0.94010100	-6.40186100
H	9.17985700	1.97335300	-5.01259200
H	9.04737500	1.86456600	-2.53970000

9. HPLC Chromatograms

2a: ADH, *n*-hexane/2-propanol = 80/20, v = 1.0 mL min^{-1}, λ = 254 nm

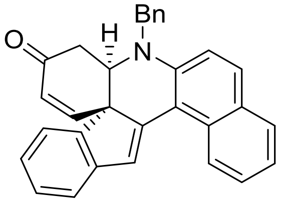

Chromatogram

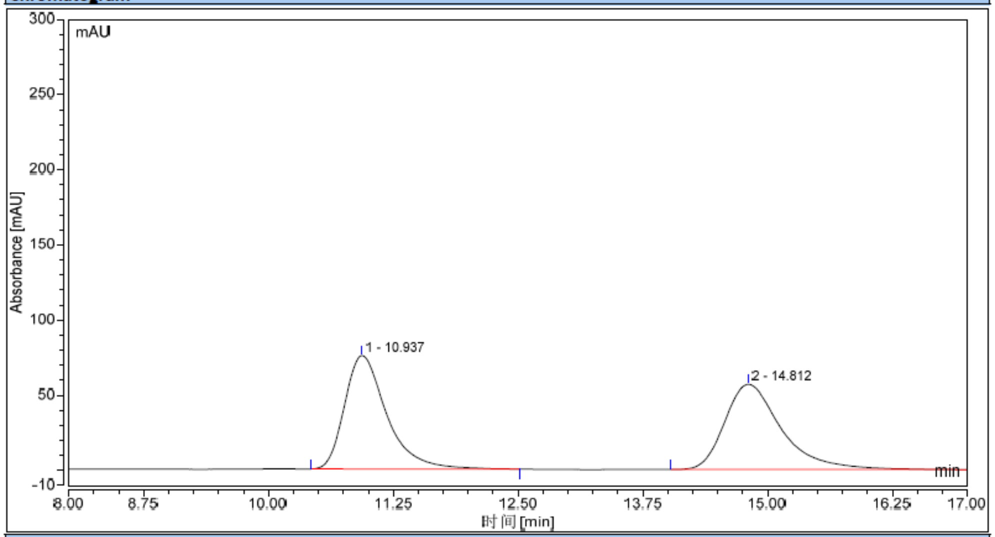

Integration Results

No.	Peak Name	Retention Time min	Area mAU*min	Height mAU	Relative Area %	Relative Height %	Amount n.a.
1		10.937	36.951	75.437	49.71	57.12	n.a.
2		14.812	37.389	56.636	50.29	42.88	n.a.
Total:			**74.339**	**132.073**	**100.00**	**100.00**	

Chromatogram

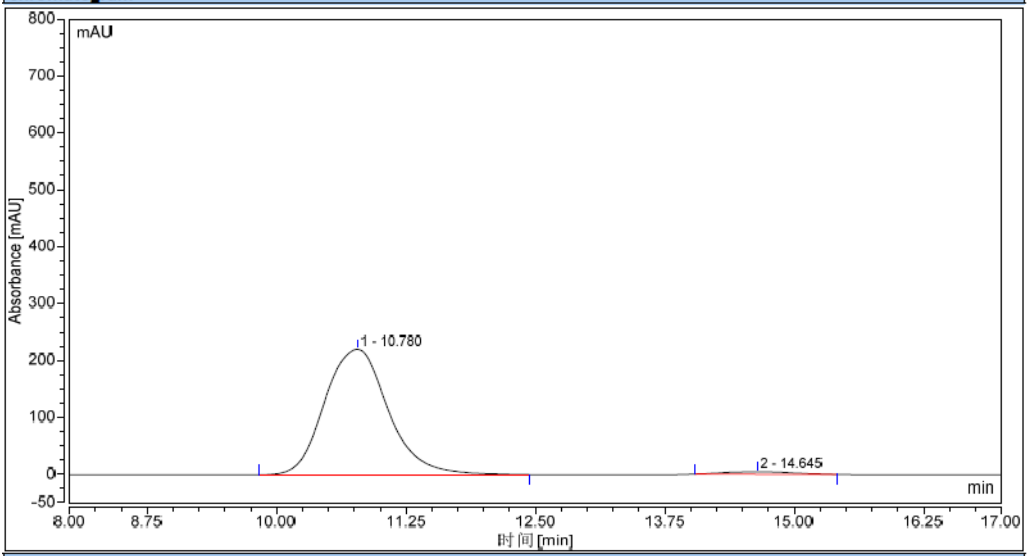

Integration Results

No.	Peak Name	Retention Time min	Area mAU*min	Height mAU	Relative Area %	Relative Height %	Amount n.a.
1		10.780	156.420	220.191	97.98	98.05	n.a.
2		14.645	3.229	4.368	2.02	1.95	n.a.
Total:			**159.649**	**224.559**	**100.00**	**100.00**	

2b: ODH, *n*-hexane/2-propanol = 70/30, v = 1.0 mL min^{-1}, λ = 254 nm

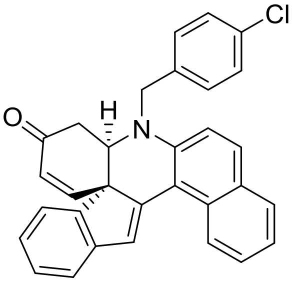

Chromatogram

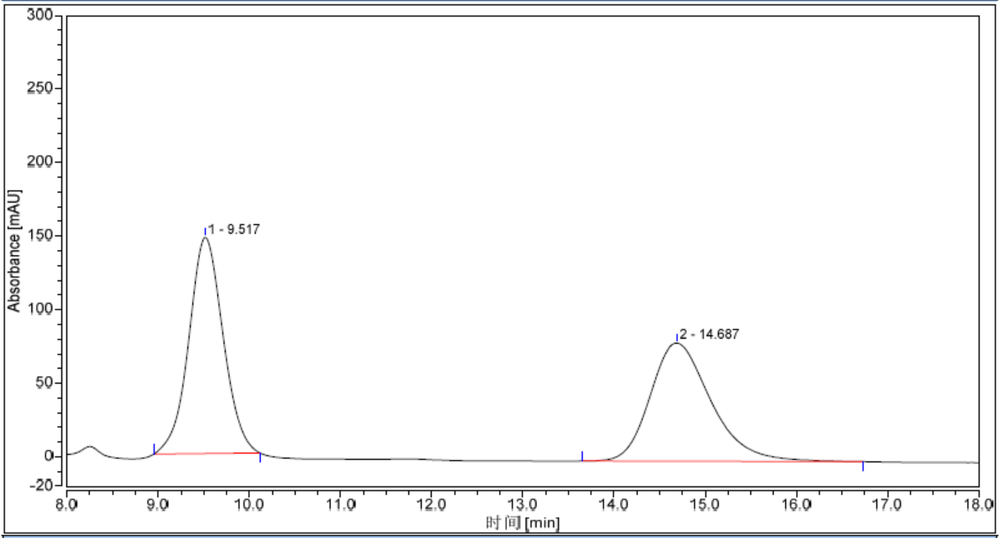

Integration Results

No.	Peak Name	Retention Time<br>min	Area<br>mAU*min	Height<br>mAU	Relative Area<br>%	Relative Height<br>%	Amount<br>n.a.
1		9.517	64.085	146.921	50.77	64.63	n.a.
2		14.687	62.147	80.403	49.23	35.37	n.a.
Total:			**126.232**	**227.324**	**100.00**	**100.00**	

Chromatogram

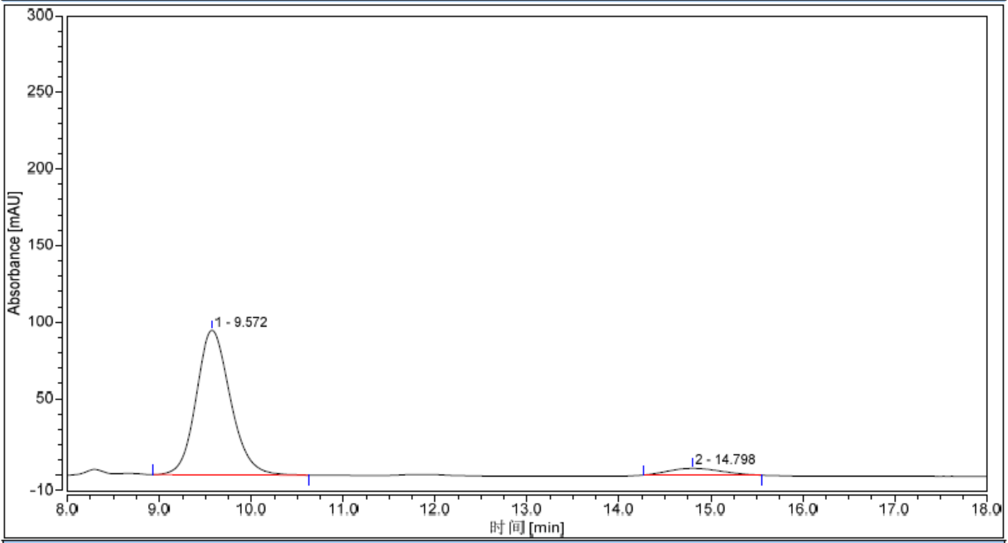

Integration Results

No.	Peak Name	Retention Time<br>min	Area<br>mAU*min	Height<br>mAU	Relative Area<br>%	Relative Height<br>%	Amount<br>n.a.
1		9.572	40.693	94.348	93.23	95.48	n.a.
2		14.798	2.953	4.466	6.77	4.52	n.a.
Total:			**43.646**	**98.815**	**100.00**	**100.00**	

2c: ODH, *n*-hexane/2-propanol = 80/20, v = 1.0 mL min^{-1}, λ = 254 nm

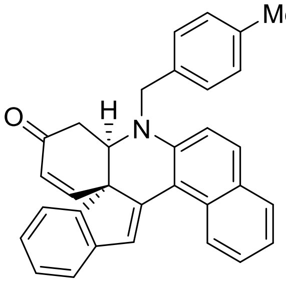

Chromatogram

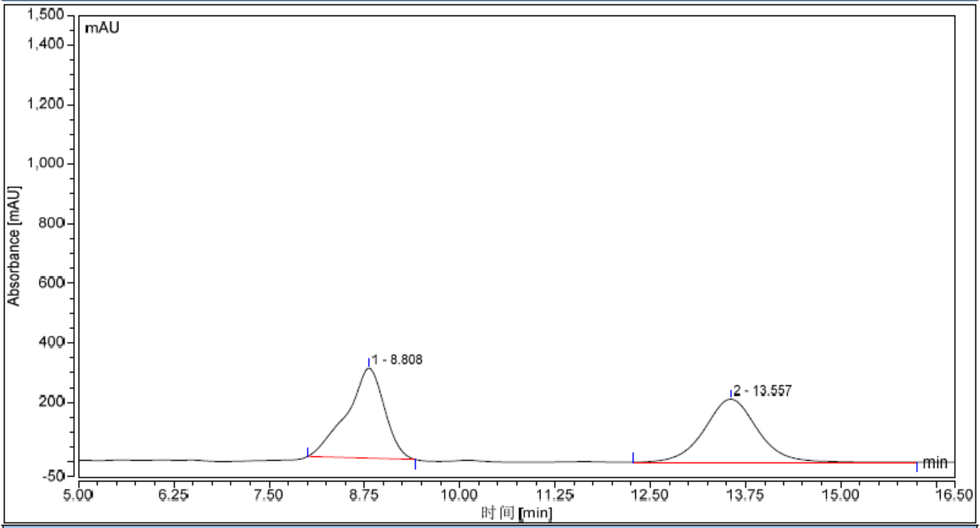

Integration Results

No.	Peak Name	Retention Time min	Area mAU*min	Height mAU	Relative Area %	Relative Height %	Amount n.a.
1		8.808	173.283	302.106	50.37	58.80	n.a.
2		13.557	170.761	211.702	49.63	41.20	n.a.
**Total:**			**344.043**	**513.808**	**100.00**	**100.00**	

Chromatogram

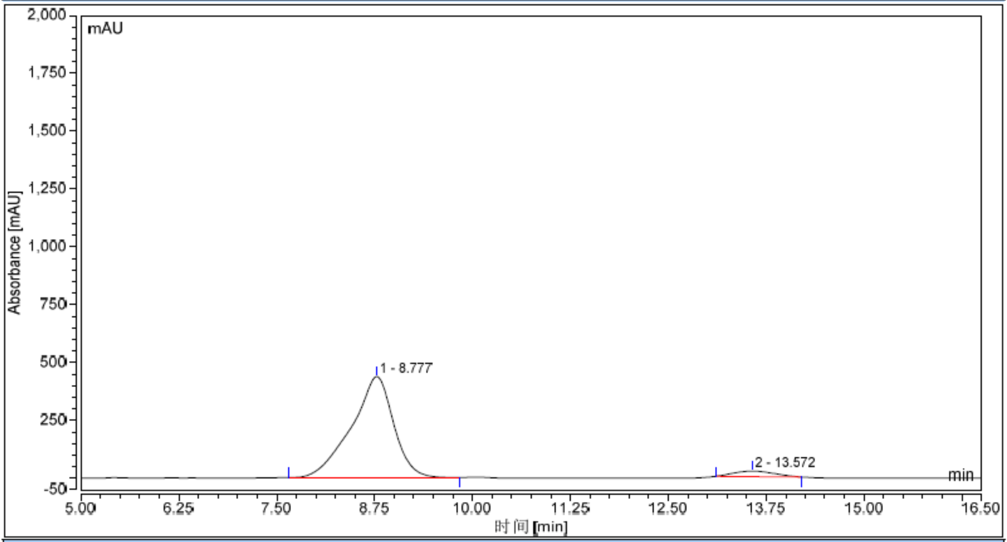

Integration Results

No.	Peak Name	Retention Time min	Area mAU*min	Height mAU	Relative Area %	Relative Height %	Amount n.a.
1		8.777	272.608	436.651	95.09	94.86	n.a.
2		13.572	14.071	23.678	4.91	5.14	n.a.
**Total:**			**286.679**	**460.329**	**100.00**	**100.00**	

2d: ODH, *n*-hexane/2-propanol = 80/20, v = 1.0 mL min^{-1}, λ = 254 nm

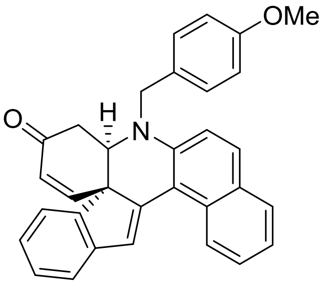

Chromatogram

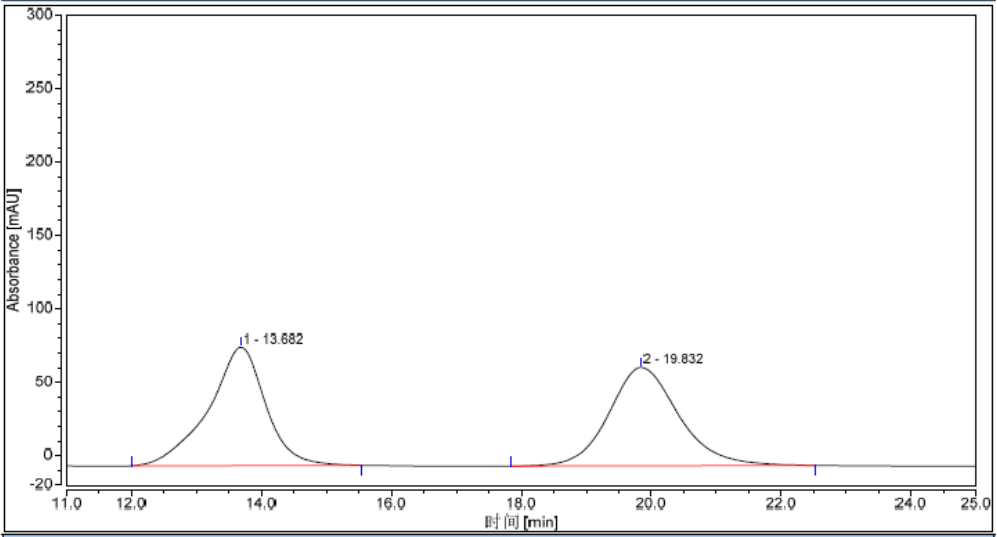

Integration Results

No.	Peak Name	Retention Time min	Area mAU*min	Height mAU	Relative Area %	Relative Height %	Amount n.a.
1		13.682	81.942	80.548	49.44	54.54	n.a.
2		19.832	83.794	67.143	50.56	45.46	n.a.
**Total:**			**165.736**	**147.690**	**100.00**	**100.00**	

Chromatogram

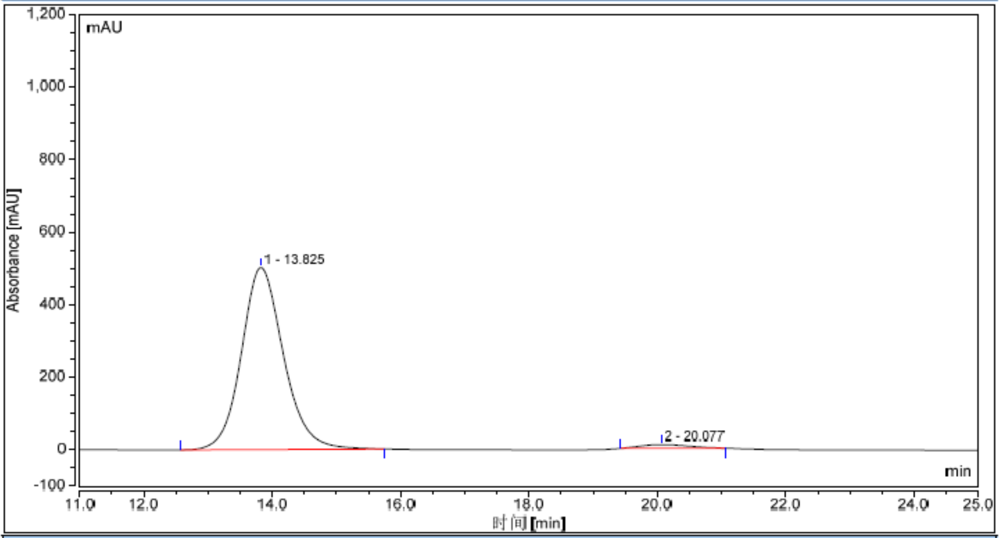

Integration Results

No.	Peak Name	Retention Time min	Area mAU*min	Height mAU	Relative Area %	Relative Height %	Amount n.a.
1		13.825	381.986	502.163	97.69	97.98	n.a.
2		20.077	9.017	10.365	2.31	2.02	n.a.
**Total:**			**391.002**	**512.527**	**100.00**	**100.00**	

2e: ADH, *n*-hexane/2-propanol = 80/20, v = 1.0 mL min^{-1}, λ = 254 nm

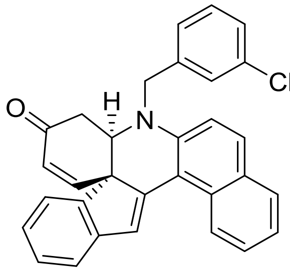

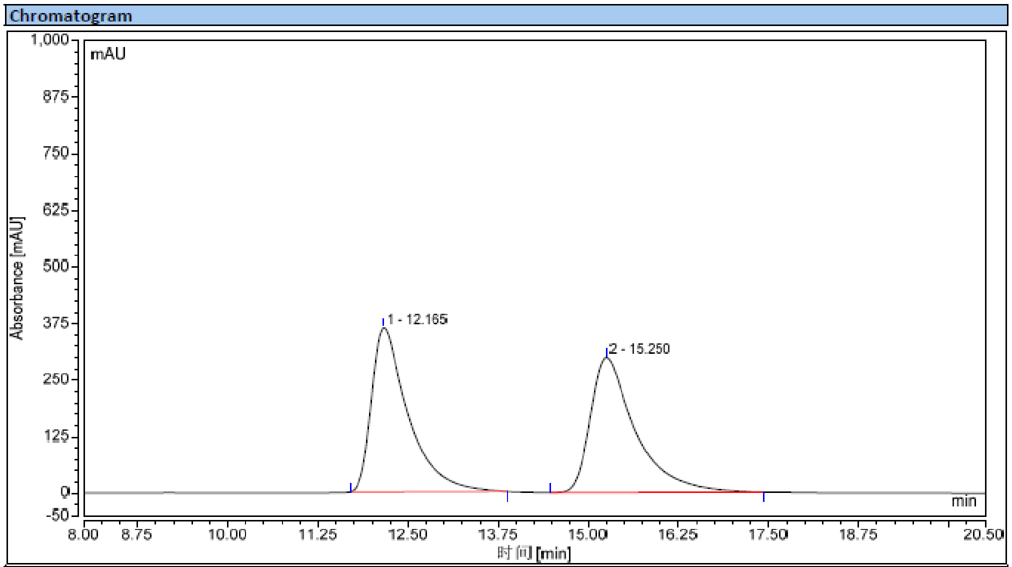

Integration Results

No.	Peak Name	Retention Time min	Area mAU*min	Height mAU	Relative Area %	Relative Height %	Amount n.a.
1		12.165	214.261	363.251	49.75	54.89	n.a.
2		15.250	216.379	298.517	50.25	45.11	n.a.
Total:			430.640	661.769	100.00	100.00	

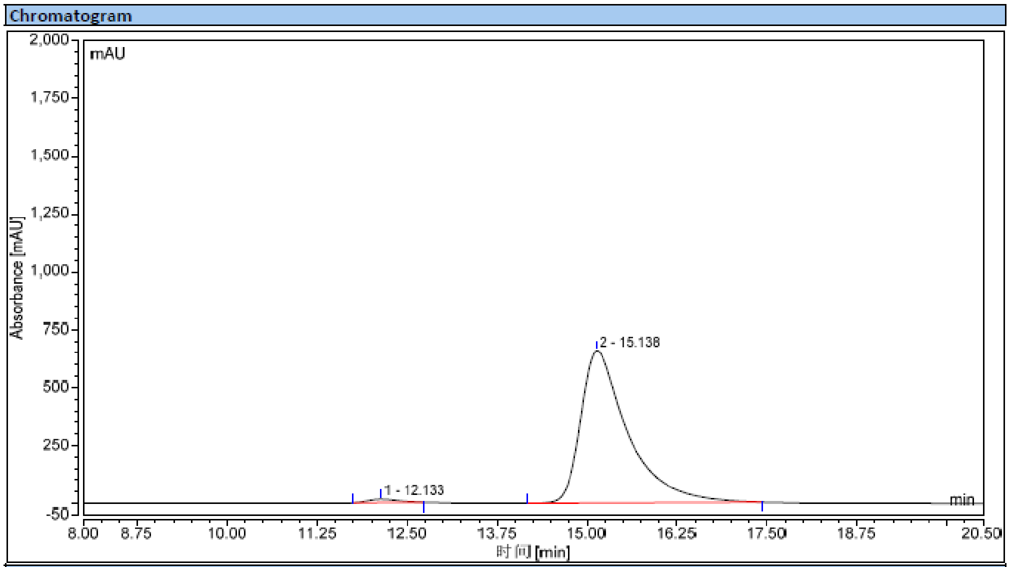

Integration Results

No.	Peak Name	Retention Time min	Area mAU*min	Height mAU	Relative Area %	Relative Height %	Amount n.a.
1		12.133	6.968	14.868	1.38	2.21	n.a.
2		15.138	499.028	658.117	98.62	97.79	n.a.
Total:			505.995	672.985	100.00	100.00	

2f: ODH, *n*-hexane/2-propanol = 80/20, v = 1.0 mL min^{-1}, λ = 254 nm

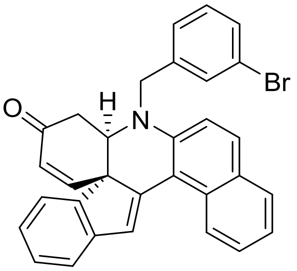

Chromatogram

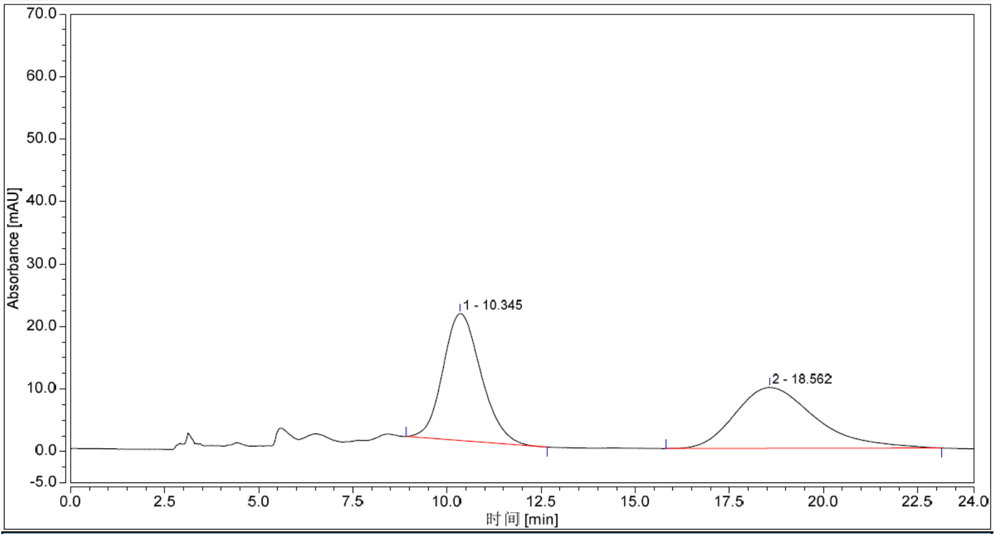

Integration Results

No.	Peak Name	Retention Time min	Area mAU*min	Height mAU	Relative Area %	Relative Height %	Amount n.a.
1		10.345	23.869	20.329	49.79	67.59	n.a.
2		18.562	24.067	9.748	50.21	32.41	n.a.
Total:			**47.936**	**30.077**	**100.00**	**100.00**	

Chromatogram

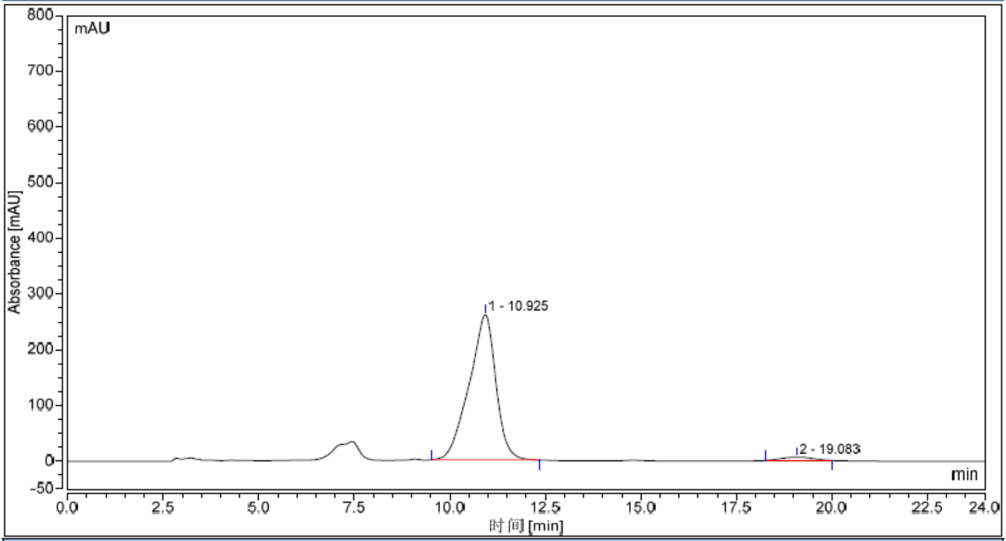

Integration Results

No.	Peak Name	Retention Time min	Area mAU*min	Height mAU	Relative Area %	Relative Height %	Amount n.a.
1		10.925	211.068	260.696	97.48	97.78	n.a.
2		19.083	5.464	5.906	2.52	2.22	n.a.
Total:			**216.532**	**266.602**	**100.00**	**100.00**	

2g: ODH, *n*-hexane/2-propanol = 80/20, v = 1.0 mL min^{-1}, λ = 254 nm

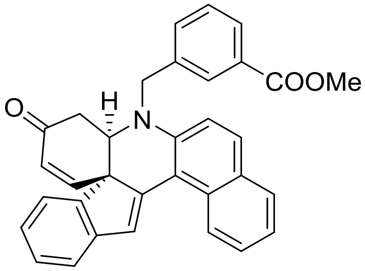

Chromatogram

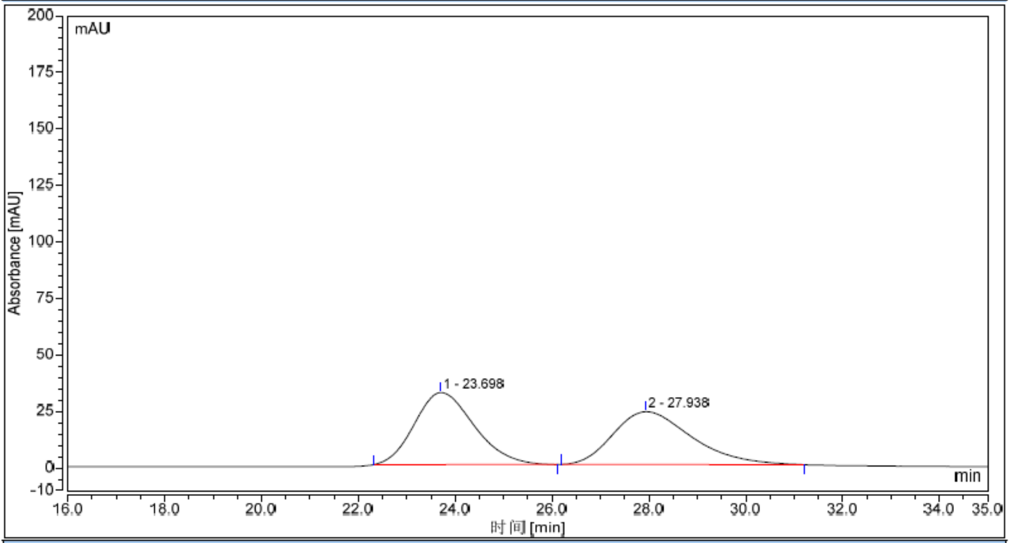

Integration Results

No.	Peak Name	Retention Time min	Area mAU*min	Height mAU	Relative Area %	Relative Height %	Amount n.a.
1		23.698	45.526	31.886	50.89	57.70	n.a.
2		27.938	43.932	23.372	49.11	42.30	n.a.
Total:			**89.458**	**55.258**	**100.00**	**100.00**	

Chromatogram

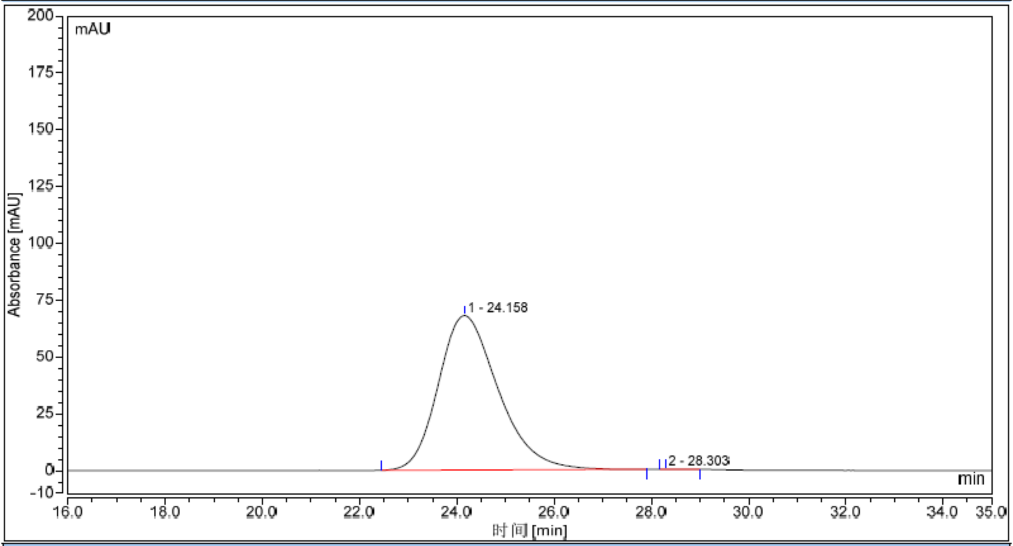

Integration Results

No.	Peak Name	Retention Time min	Area mAU*min	Height mAU	Relative Area %	Relative Height %	Amount n.a.
1		24.158	96.925	67.890	99.98	99.94	n.a.
2		28.303	0.022	0.040	0.02	0.06	n.a.
Total:			**96.947**	**67.930**	**100.00**	**100.00**	

2h: ADH, *n*-hexane/2-propanol = 90/10, v = 1.0 mL·min^{-1}, λ = 254 nm

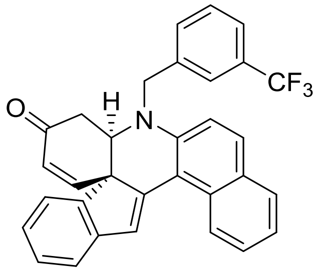

Chromatogram

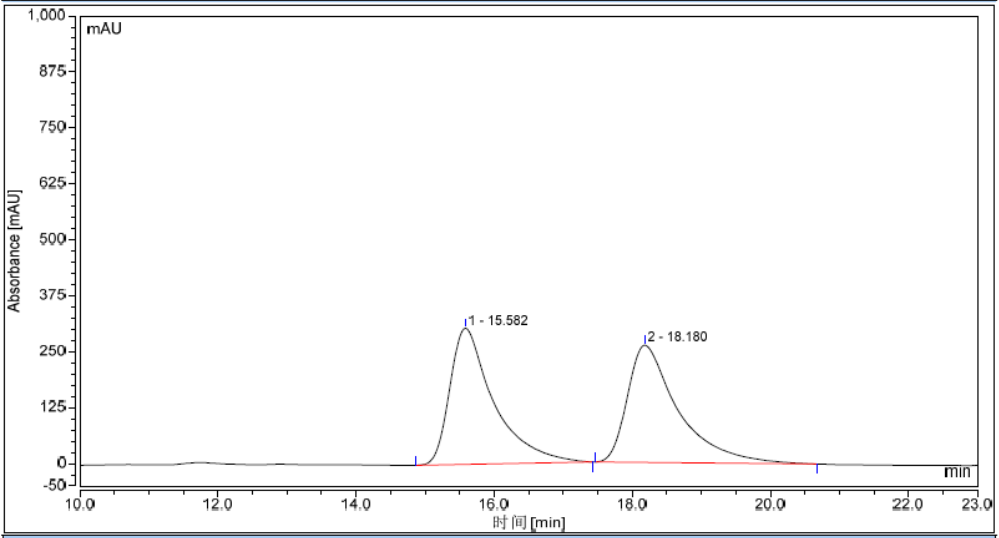

Integration Results

No.	Peak Name	Retention Time min	Area mAU*min	Height mAU	Relative Area %	Relative Height %	Amount n.a.
1		15.582	223.605	303.968	50.19	53.73	n.a.
2		18.180	221.901	261.778	49.81	46.27	n.a.
Total:			**445.507**	**565.745**	**100.00**	**100.00**	

Chromatogram

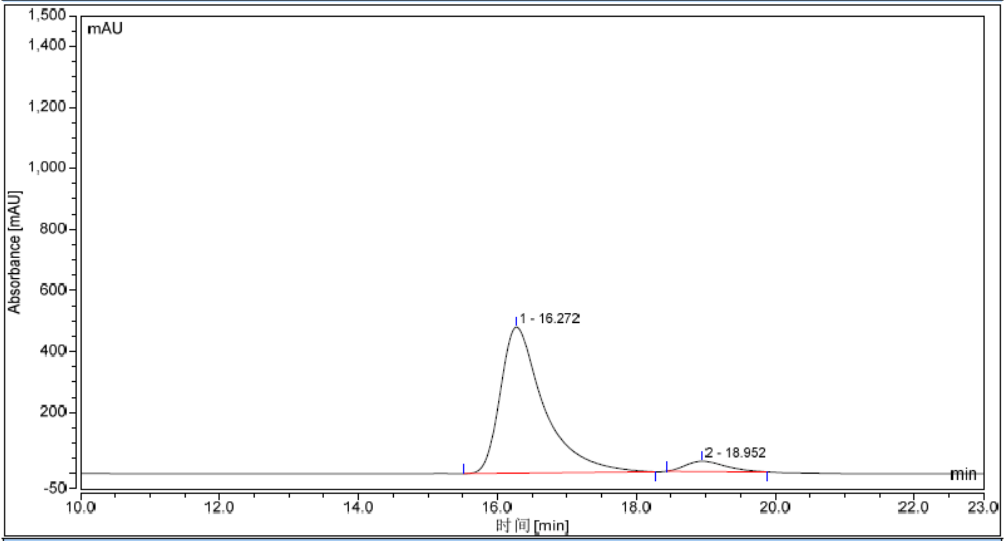

Integration Results

No.	Peak Name	Retention Time min	Area mAU*min	Height mAU	Relative Area %	Relative Height %	Amount n.a.
1		16.272	347.650	478.289	93.93	93.42	n.a.
2		18.952	22.472	33.669	6.07	6.58	n.a.
Total:			**370.122**	**511.958**	**100.00**	**100.00**	

2i: ODH, *n*-hexane/2-propanol = 80/20, v = 1.0 mL min^{-1}, λ = 254 nm

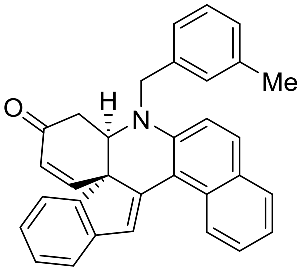

Chromatogram

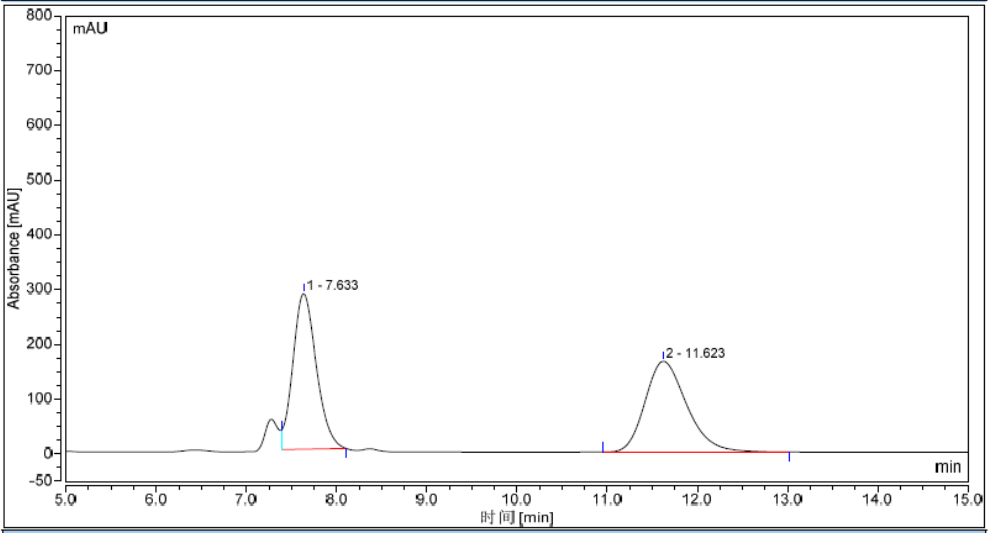

Integration Results

No.	Peak Name	Retention Time min	Area mAU*min	Height mAU	Relative Area %	Relative Height %	Amount n.a.
1		7.633	85.734	284.348	49.12	63.19	n.a.
2		11.623	88.819	165.638	50.88	36.81	n.a.
**Total:**			**174.552**	**449.986**	**100.00**	**100.00**	

Chromatogram

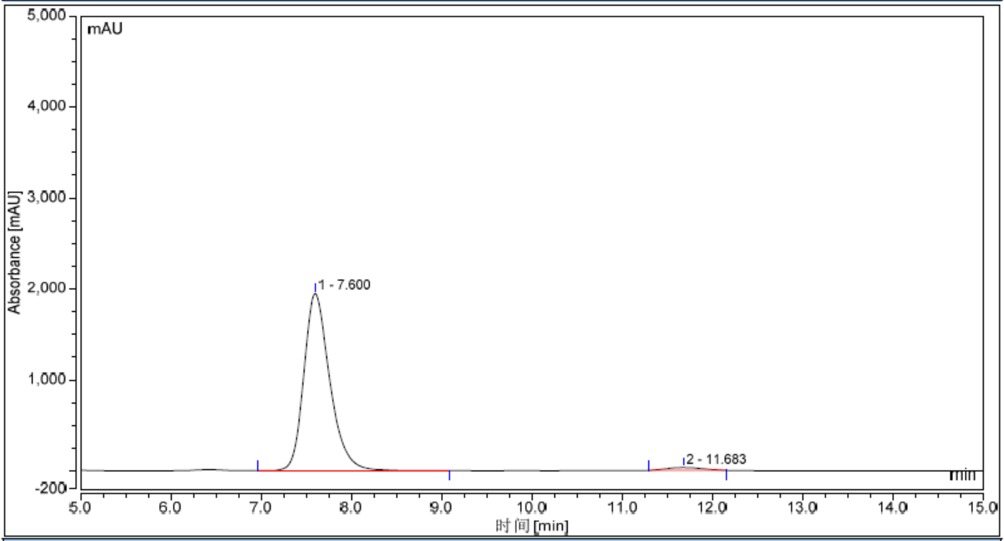

Integration Results

No.	Peak Name	Retention Time min	Area mAU*min	Height mAU	Relative Area %	Relative Height %	Amount n.a.
1		7.600	650.106	1947.593	97.92	98.47	n.a.
2		11.683	13.825	30.331	2.08	1.53	n.a.
**Total:**			**663.931**	**1977.924**	**100.00**	**100.00**	

2j: ODH, *n*-hexane/2-propanol = 80/20, v = 1.0 mL min^{-1}, λ = 254 nm

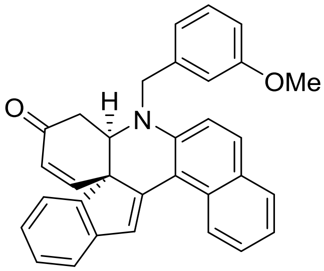

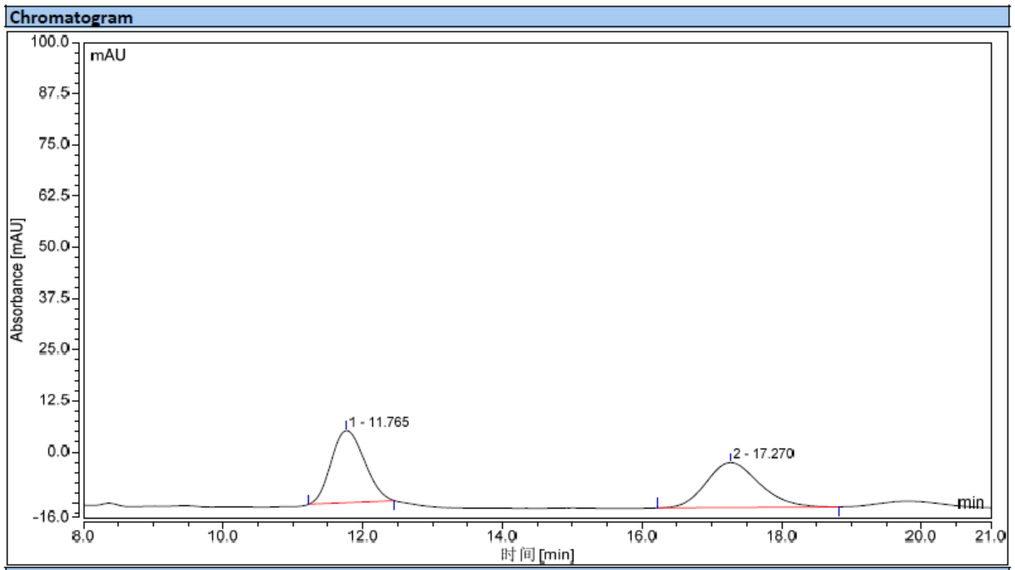

Integration Results

No.	Peak Name	Retention Time min	Area mAU*min	Height mAU	Relative Area %	Relative Height %	Amount n.a.
1		11.765	9.704	17.568	49.93	61.54	n.a.
2		17.270	9.730	10.978	50.07	38.46	n.a.
Total:			19.434	28.546	100.00	100.00	

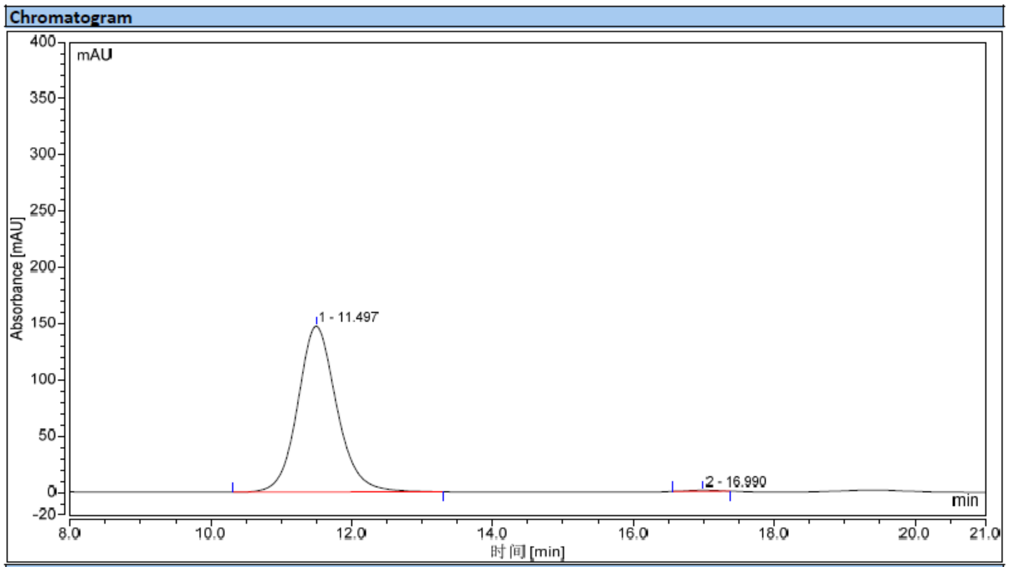

Integration Results

No.	Peak Name	Retention Time min	Area mAU*min	Height mAU	Relative Area %	Relative Height %	Amount n.a.
1		11.497	94.676	147.346	99.58	99.46	n.a.
2		16.990	0.402	0.800	0.42	0.54	n.a.
Total:			95.079	148.146	100.00	100.00	

2k: ODH, *n*-hexane/2-propanol = 80/20, v = 1.0 mL min^{-1}, λ = 254 nm

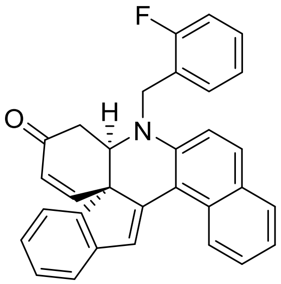

Chromatogram

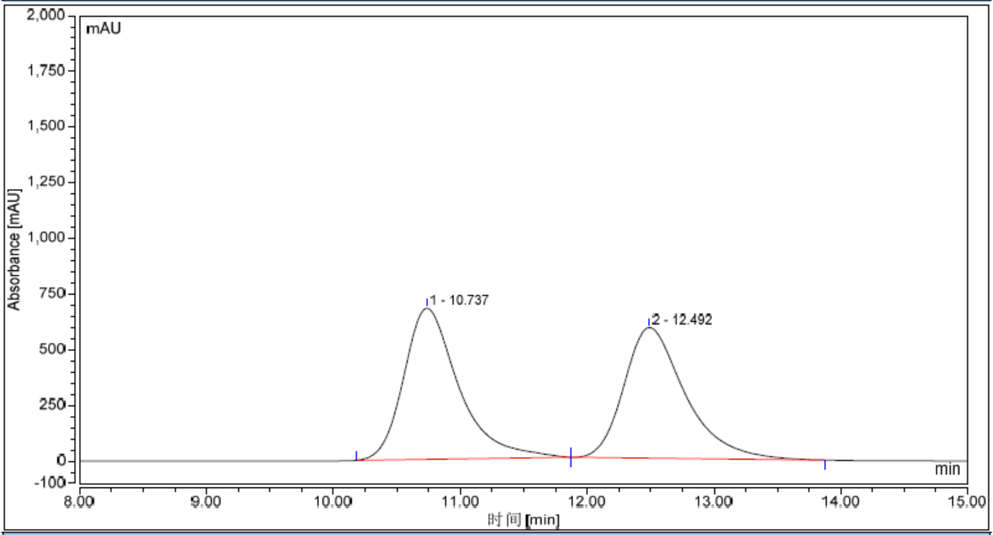

Integration Results

No.	Peak Name	Retention Time<br>min	Area<br>mAU*min	Height<br>mAU	Relative Area<br>%	Relative Height<br>%	Amount<br>n.a.
1		10.737	340.642	677.807	50.98	53.64	n.a.
2		12.492	327.590	585.891	49.02	46.36	n.a.
**Total:**			**668.232**	**1263.698**	**100.00**	**100.00**	

Chromatogram

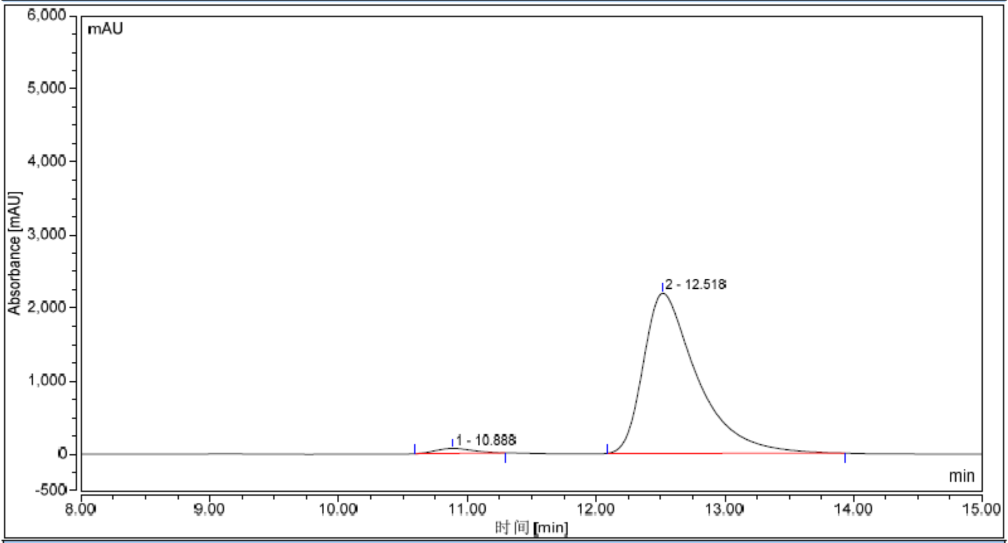

Integration Results

No.	Peak Name	Retention Time<br>min	Area<br>mAU*min	Height<br>mAU	Relative Area<br>%	Relative Height<br>%	Amount<br>n.a.
1		10.888	22.448	67.436	2.07	2.99	n.a.
2		12.518	1061.998	2190.123	97.93	97.01	n.a.
**Total:**			**1084.446**	**2257.560**	**100.00**	**100.00**	

2l: ODH, *n*-hexane/2-propanol = 80/20, v = 1.0 mL min^{-1}, λ = 254 nm

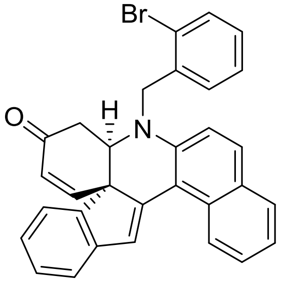

Chromatogram

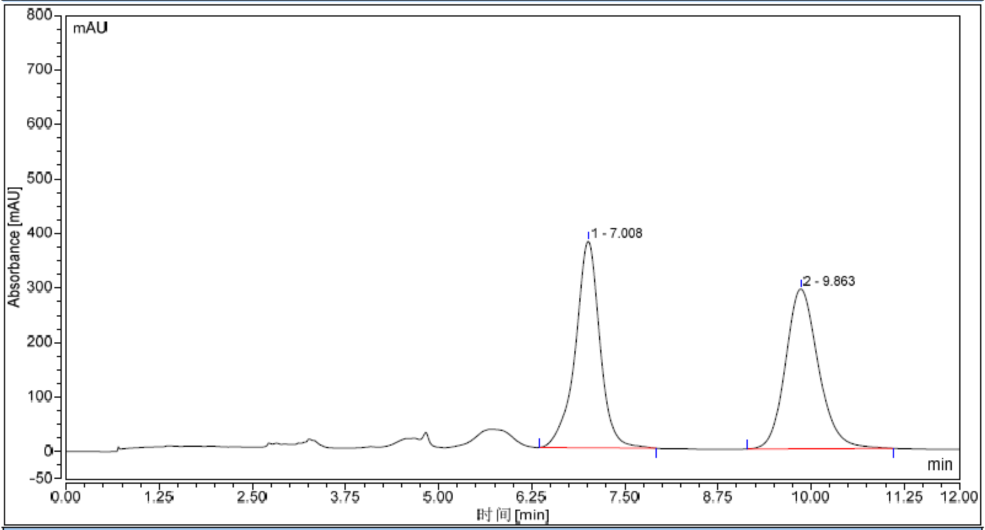

Integration Results

No.	Peak Name	Retention Time min	Area mAU*min	Height mAU	Relative Area %	Relative Height %	Amount n.a.
1		7.008	144.773	378.793	49.15	56.41	n.a.
2		9.863	149.768	292.725	50.85	43.59	n.a.
Total:			**294.541**	**671.518**	**100.00**	**100.00**	

Chromatogram

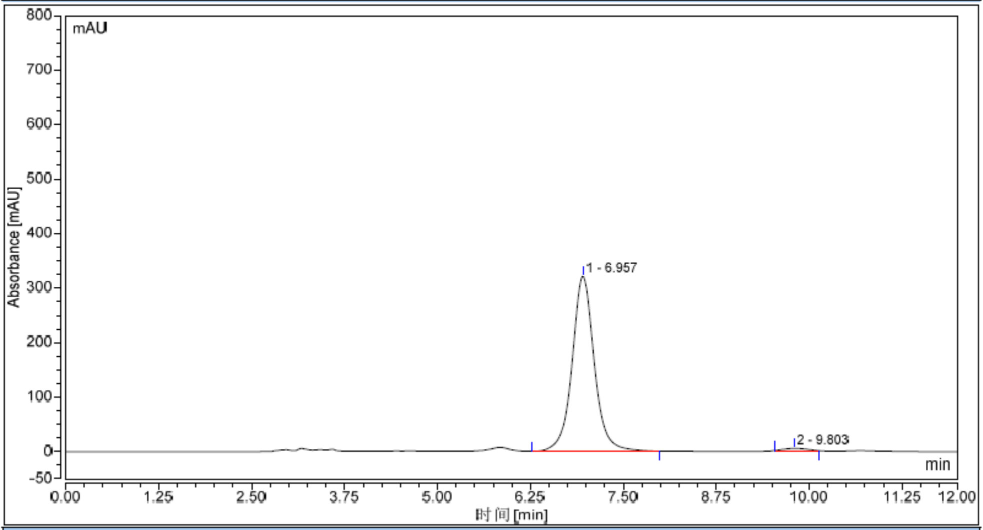

Integration Results

No.	Peak Name	Retention Time min	Area mAU*min	Height mAU	Relative Area %	Relative Height %	Amount n.a.
1		6.957	111.002	321.281	98.69	98.69	n.a.
2		9.803	1.474	4.261	1.31	1.31	n.a.
Total:			**112.476**	**325.542**	**100.00**	**100.00**	

2m: ODH, *n*-hexane/2-propanol = 80/20, v = 1.0 mL min^{-1}, λ = 254 nm

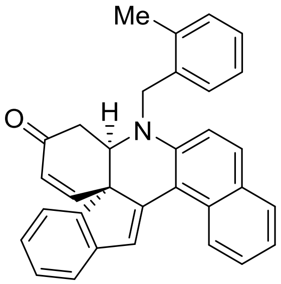

Chromatogram

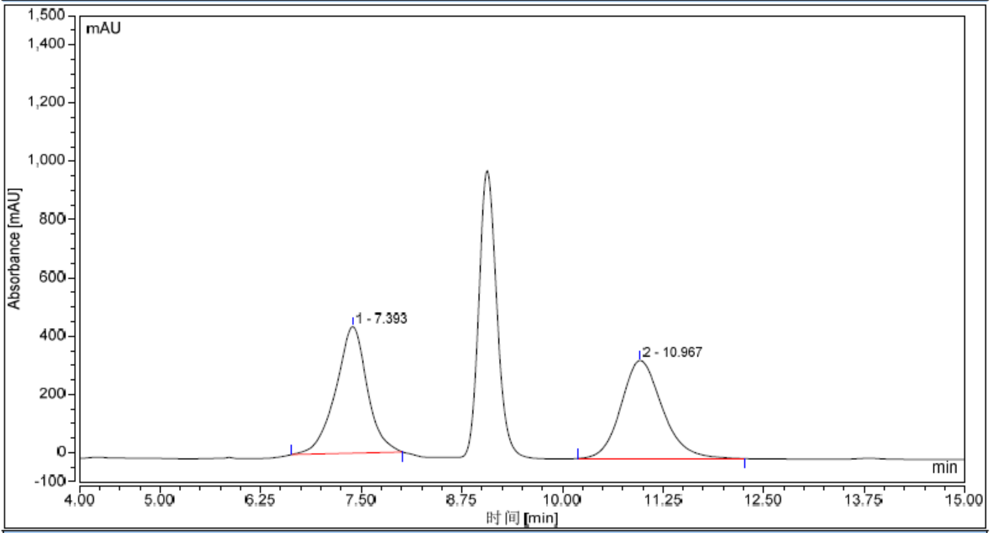

Integration Results

No.	Peak Name	Retention Time min	Area mAU*min	Height mAU	Relative Area %	Relative Height %	Amount n.a.
1		7.393	196.586	434.293	49.56	56.35	n.a.
2		10.967	200.066	336.364	50.44	43.65	n.a.
**Total:**			**396.652**	**770.657**	**100.00**	**100.00**	

Chromatogram

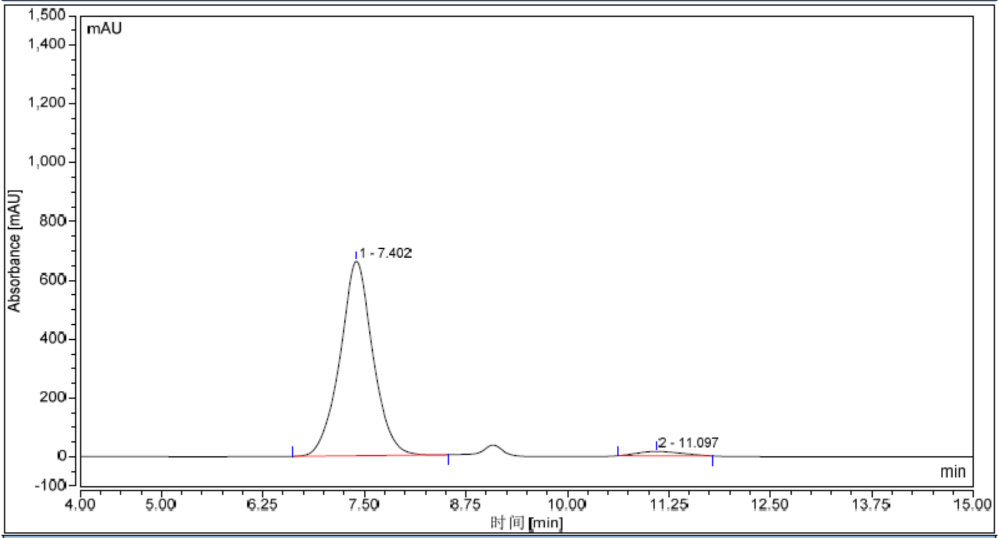

Integration Results

No.	Peak Name	Retention Time min	Area mAU*min	Height mAU	Relative Area %	Relative Height %	Amount n.a.
1		7.402	309.938	661.269	97.09	97.82	n.a.
2		11.097	9.276	14.735	2.91	2.18	n.a.
**Total:**			**319.214**	**676.004**	**100.00**	**100.00**	

2n: ODH, *n*-hexane/2-propanol = 80/20, v = 1.0 mL min^{-1}, λ = 254 nm

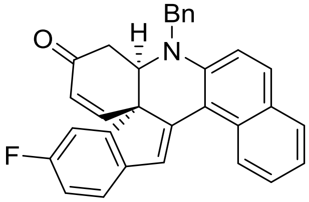

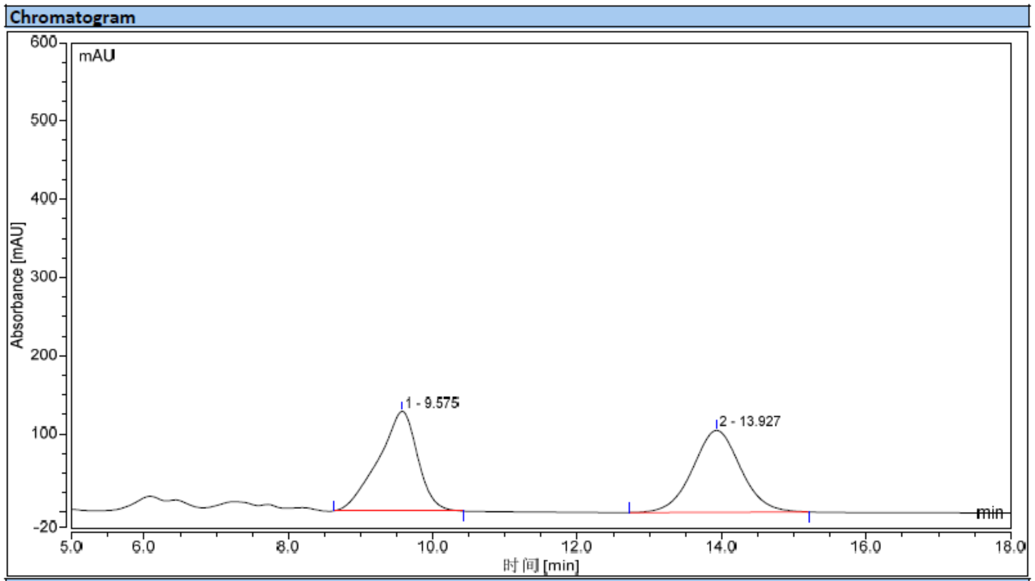

Integration Results

No.	Peak Name	Retention Time min	Area mAU*min	Height mAU	Relative Area %	Relative Height %	Amount n.a.
1		9.575	80.543	127.428	49.97	54.92	n.a.
2		13.927	80.655	104.614	50.03	45.08	n.a.
Total:			**161.198**	**232.042**	**100.00**	**100.00**	

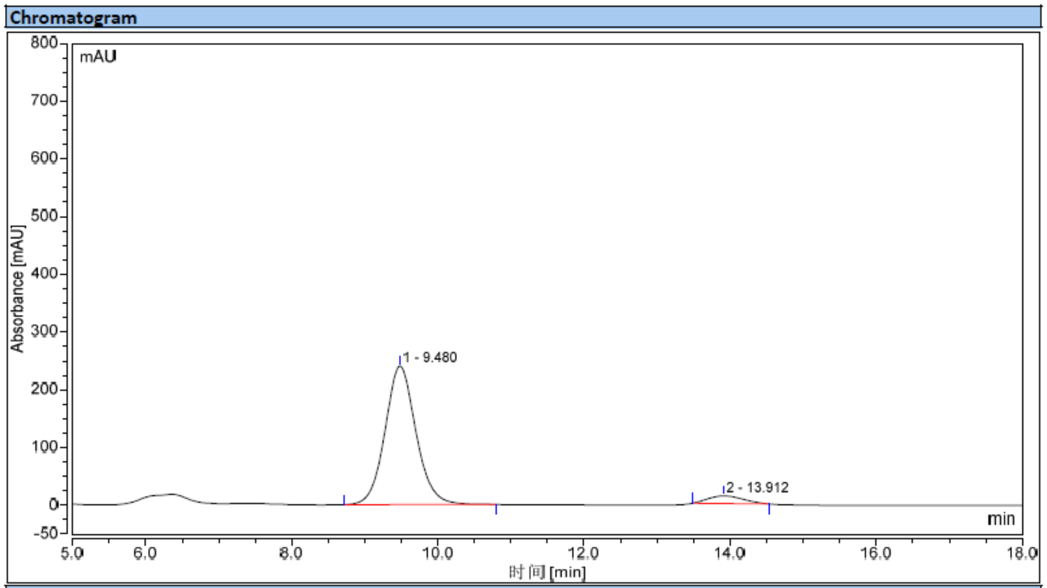

Integration Results

No.	Peak Name	Retention Time min	Area mAU*min	Height mAU	Relative Area %	Relative Height %	Amount n.a.
1		9.480	118.130	240.197	94.08	94.76	n.a.
2		13.912	7.433	13.275	5.92	5.24	n.a.
Total:			**125.563**	**253.472**	**100.00**	**100.00**	

2o: ODH, *n*-hexane/2-propanol = 80/20, v = 1.0 mL min^{-1}, λ = 254 nm

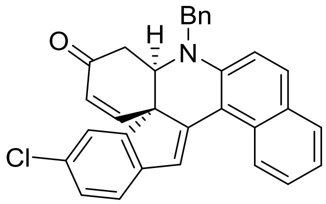

Chromatogram

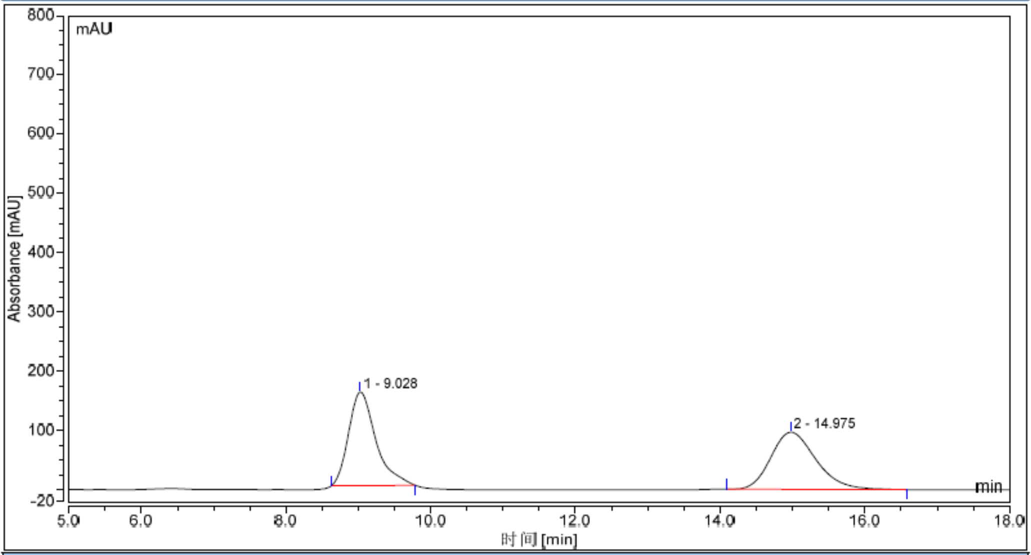

Integration Results

No.	Peak Name	Retention Time<br>min	Area<br>mAU*min	Height<br>mAU	Relative Area<br>%	Relative Height<br>%	Amount<br>n.a.
1		9.028	68.894	157.808	49.78	62.05	n.a.
2		14.975	69.499	96.510	50.22	37.95	n.a.
Total:			138.393	254.319	100.00	100.00	

Chromatogram

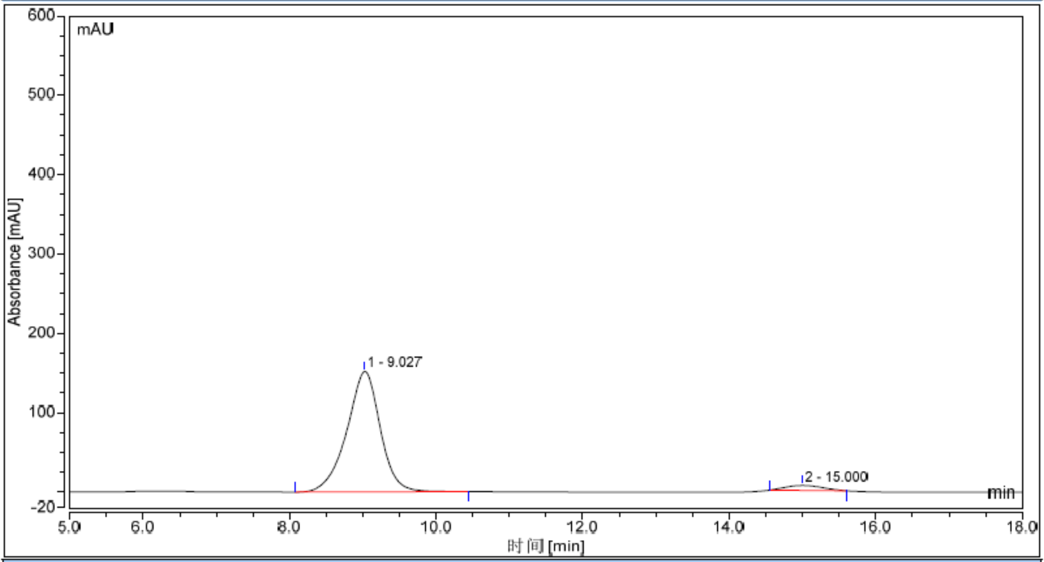

Integration Results

No.	Peak Name	Retention Time<br>min	Area<br>mAU*min	Height<br>mAU	Relative Area<br>%	Relative Height<br>%	Amount<br>n.a.
1		9.027	80.314	151.764	95.71	96.07	n.a.
2		15.000	3.601	6.208	4.29	3.93	n.a.
Total:			83.914	157.972	100.00	100.00	

2p: ADH, *n*-hexane/2-propanol = 80/20, v = 1.0 mL min^{-1}, λ = 254 nm

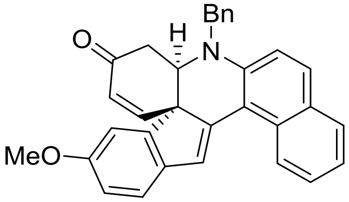

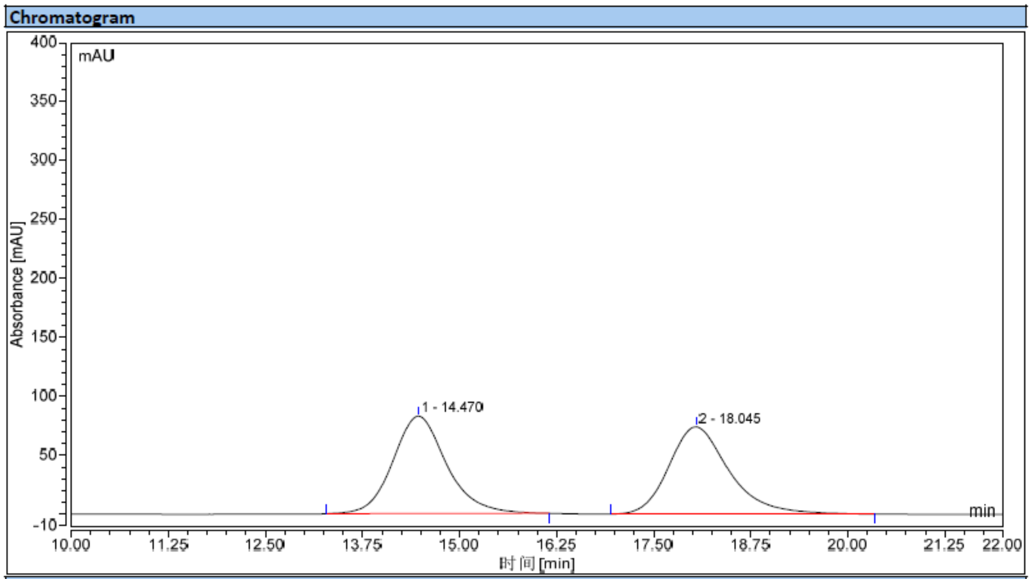

Integration Results

No.	Peak Name	Retention Time min	Area mAU*min	Height mAU	Relative Area %	Relative Height %	Amount n.a.
1		14.470	66.333	82.580	49.93	52.75	n.a.
2		18.045	66.509	73.979	50.07	47.25	n.a.
Total:			132.841	156.559	100.00	100.00	

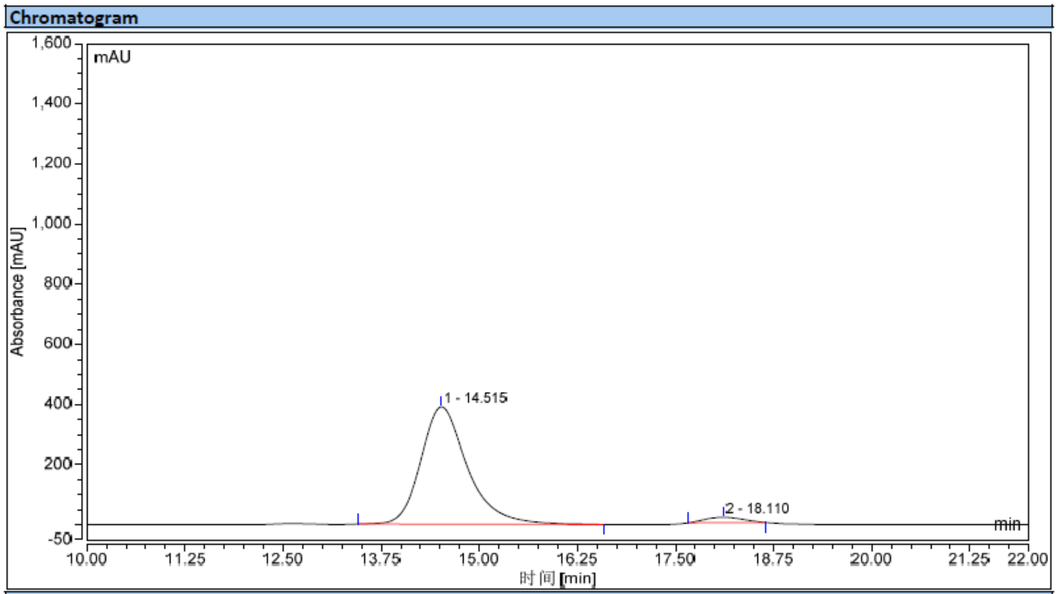

Integration Results

No.	Peak Name	Retention Time min	Area mAU*min	Height mAU	Relative Area %	Relative Height %	Amount n.a.
1		14.515	268.340	390.042	96.39	95.60	n.a.
2		18.110	10.058	17.953	3.61	4.40	n.a.
Total:			278.398	407.996	100.00	100.00	

2q: ODH, *n*-hexane/2-propanol = 80/20, v = 1.0 mL min^{-1}, λ = 254 nm

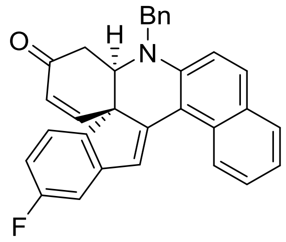

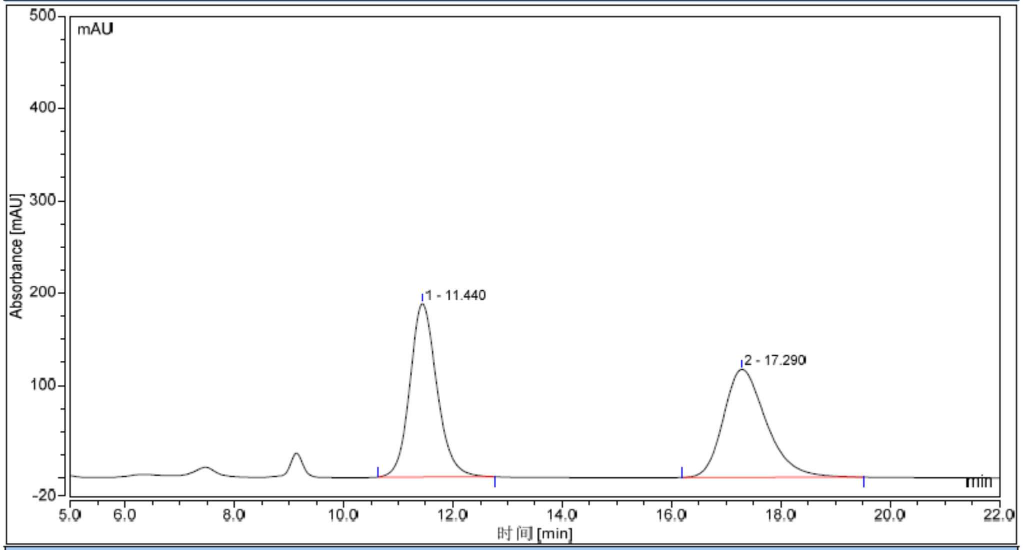

Integration Results

No.	Peak Name	Retention Time min	Area mAU*min	Height mAU	Relative Area %	Relative Height %	Amount n.a.
1		11.440	107.651	187.684	50.32	61.61	n.a.
2		17.290	106.303	116.959	49.68	38.39	n.a.
Total:			**213.954**	**304.643**	**100.00**	**100.00**	

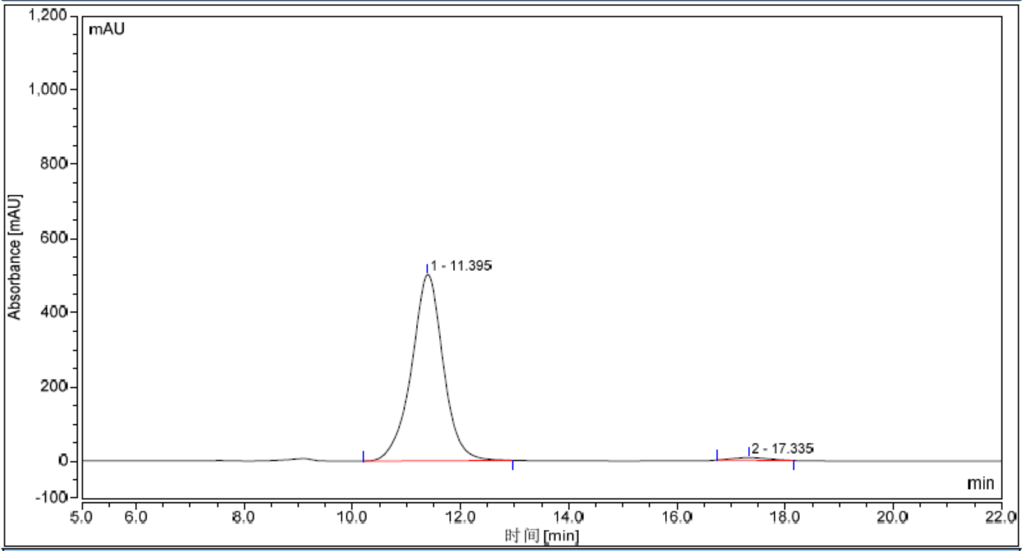

Integration Results

No.	Peak Name	Retention Time min	Area mAU*min	Height mAU	Relative Area %	Relative Height %	Amount n.a.
1		11.395	340.380	502.437	98.39	98.57	n.a.
2		17.335	5.572	7.264	1.61	1.43	n.a.
Total:			**345.952**	**509.702**	**100.00**	**100.00**	

2r: ODH, *n*-hexane/2-propanol = 80/20, v = 1.0 mL min^{-1}, λ = 254 nm

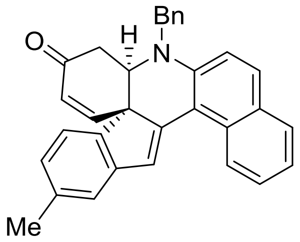

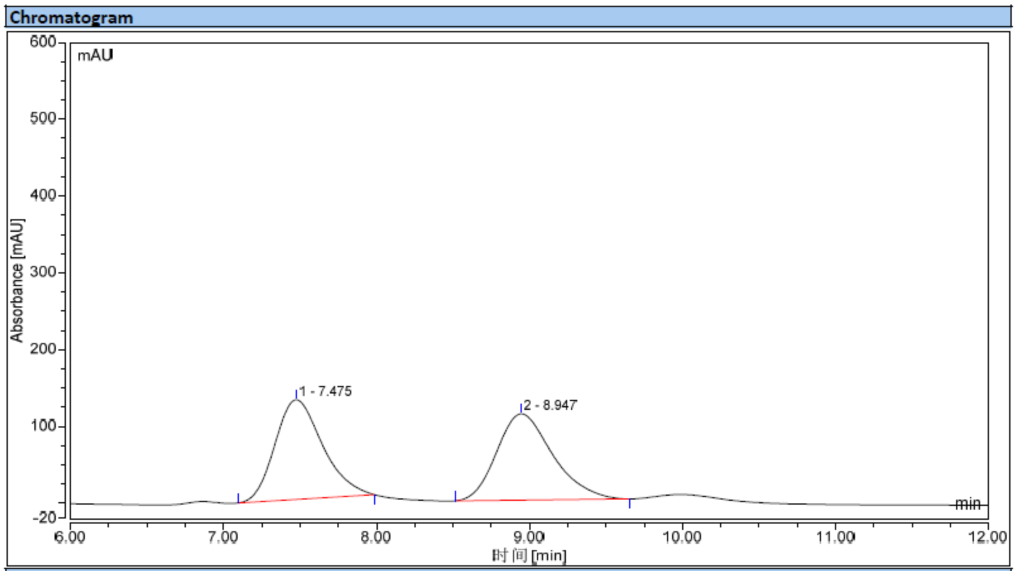

Integration Results

No.	Peak Name	Retention Time min	Area mAU*min	Height mAU	Relative Area %	Relative Height %	Amount n.a.
1		7.475	46.766	129.806	49.58	53.62	n.a.
2		8.947	47.566	112.296	50.42	46.38	n.a.
Total:			**94.332**	**242.102**	**100.00**	**100.00**	

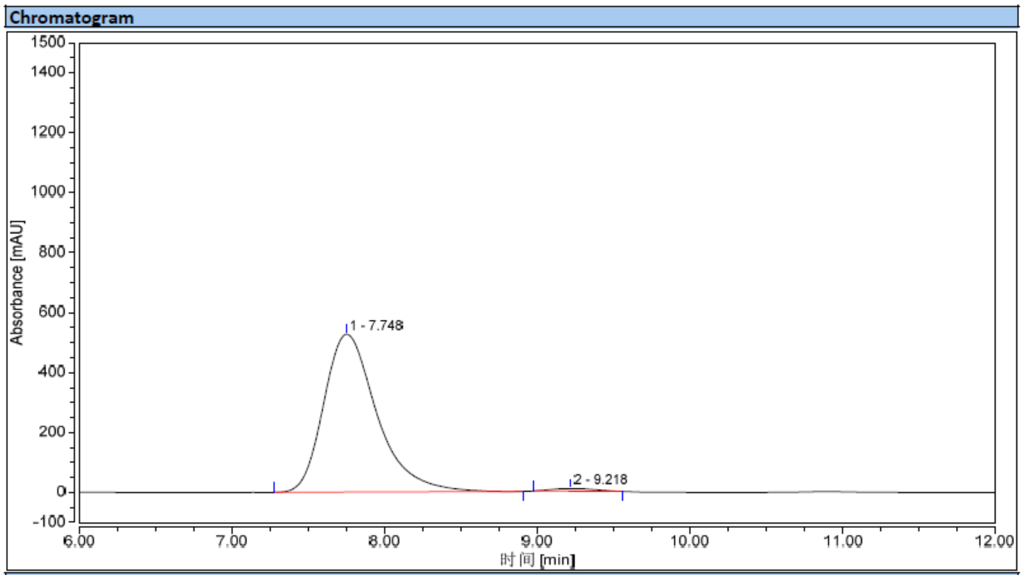

Integration Results

No.	Peak Name	Retention Time min	Area mAU*min	Height mAU	Relative Area %	Relative Height %	Amount n.a.
1		7.748	212.741	526.496	98.65	98.35	n.a.
2		9.218	2.908	8.853	1.35	1.65	n.a.
Total:			**215.649**	**535.349**	**100.00**	**100.00**	

2s: ADH, *n*-hexane/2-propanol = 80/20, v = 1.0 mL min^{-1}, λ = 254 nm

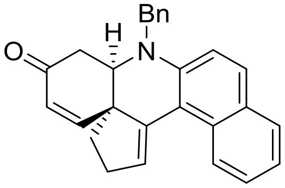

Chromatogram

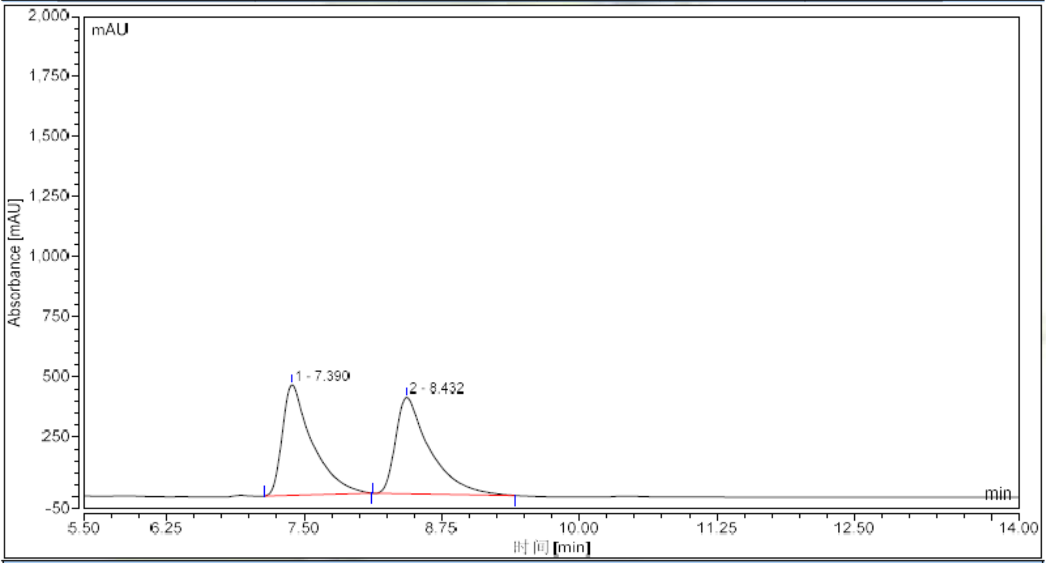

Integration Results

No.	Peak Name	Retention Time min	Area mAU*min	Height mAU	Relative Area %	Relative Height %	Amount n.a.
1		7.390	144.968	460.134	50.10	53.42	n.a.
2		8.432	144.389	401.236	49.90	46.58	n.a.
Total:			289.357	861.370	100.00	100.00	

Chromatogram

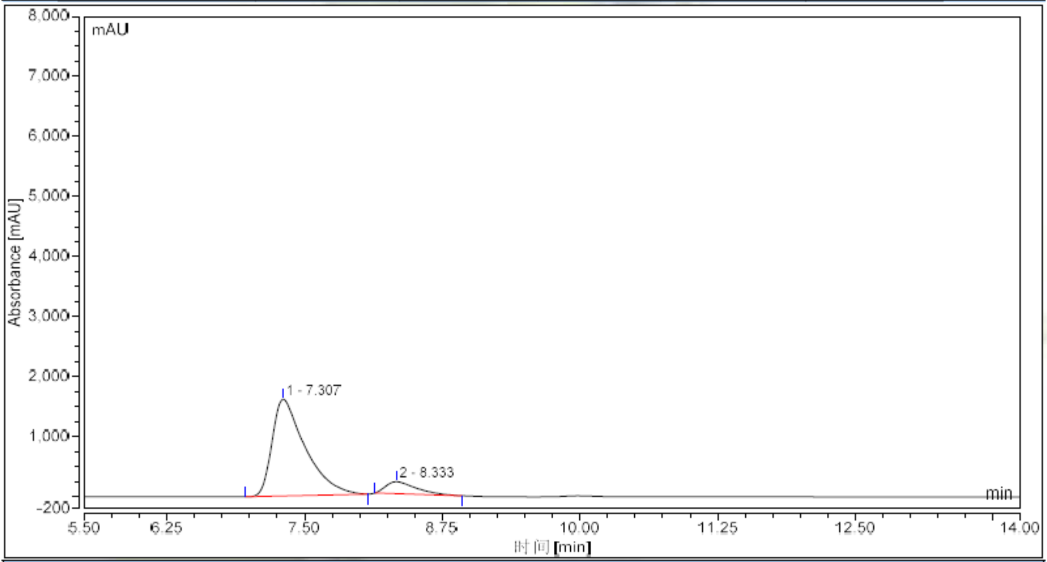

Integration Results

No.	Peak Name	Retention Time min	Area mAU*min	Height mAU	Relative Area %	Relative Height %	Amount n.a.
1		7.307	562.190	1612.117	89.80	89.04	n.a.
2		8.333	63.878	198.531	10.20	10.96	n.a.
Total:			626.067	1810.648	100.00	100.00	

2t: ADH, *n*-hexane/2-propanol = 70/30, v = 1.0 mL min^{-1}, λ = 254 nm

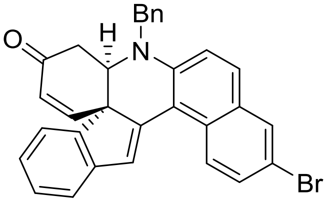

Chromatogram

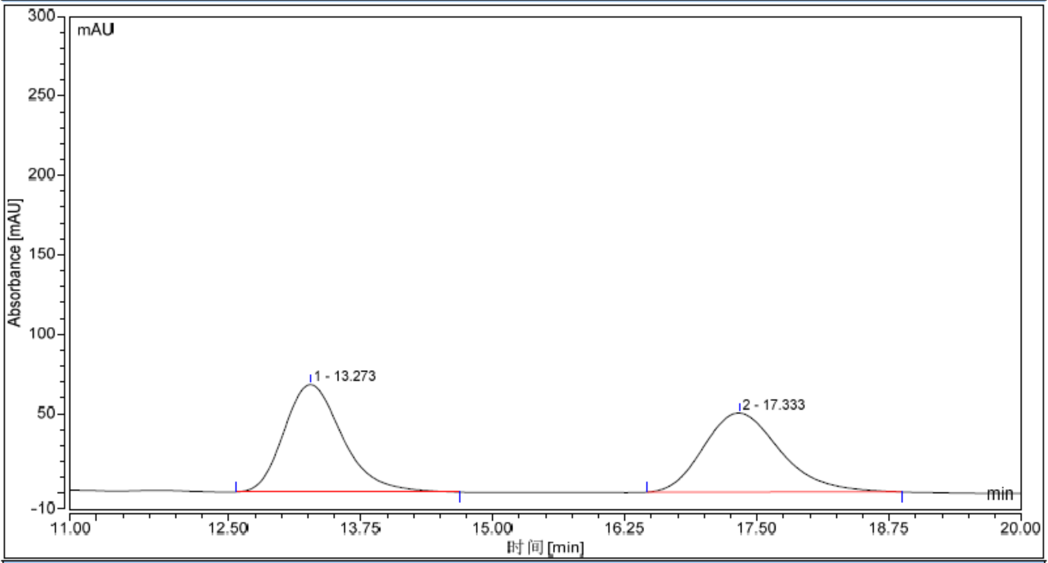

Integration Results

No.	Peak Name	Retention Time min	Area mAU*min	Height mAU	Relative Area %	Relative Height %	Amount n.a.
1		13.273	43.323	67.548	50.85	57.62	n.a.
2		17.333	41.875	49.687	49.15	42.38	n.a.
**Total:**			**85.199**	**117.236**	**100.00**	**100.00**	

Chromatogram

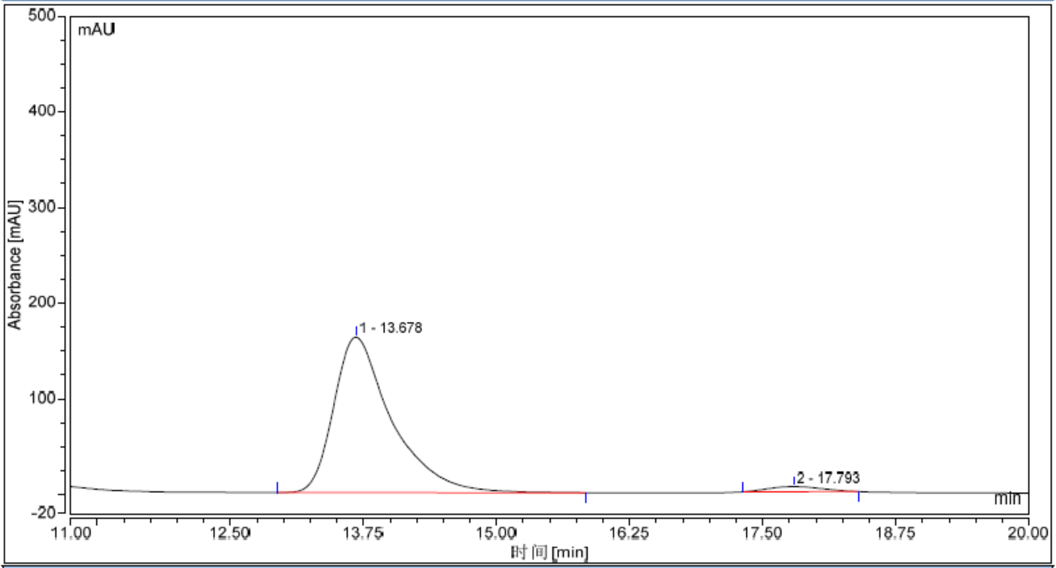

Integration Results

No.	Peak Name	Retention Time min	Area mAU*min	Height mAU	Relative Area %	Relative Height %	Amount n.a.
1		13.678	102.220	162.425	97.00	96.75	n.a.
2		17.793	3.158	5.452	3.00	3.25	n.a.
**Total:**			**105.378**	**167.878**	**100.00**	**100.00**	

2u: ADH, *n*-hexane/2-propanol = 80/20, v = 1.0 mL min^{-1}, λ = 254 nm

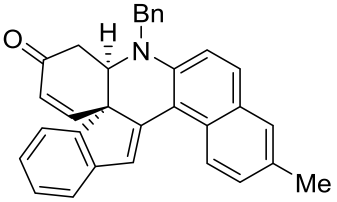

Chromatogram

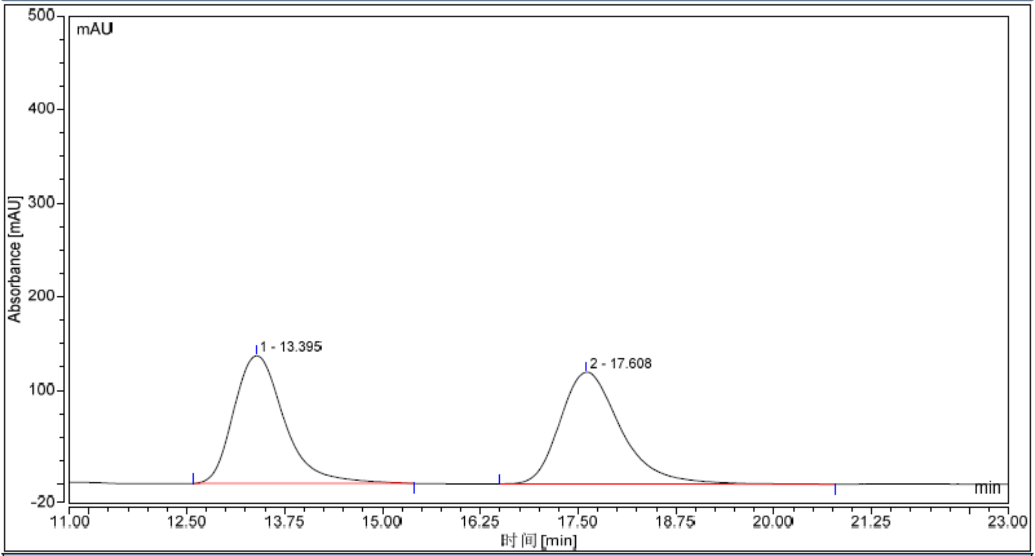

Integration Results

No.	Peak Name	Retention Time<br>min	Area<br>mAU*min	Height<br>mAU	Relative Area<br>%	Relative Height<br>%	Amount<br>n.a.
1		13.395	101.980	136.199	48.76	53.29	n.a.
2		17.608	107.189	119.395	51.24	46.71	n.a.
Total:			**209.169**	**255.594**	**100.00**	**100.00**	

Chromatogram

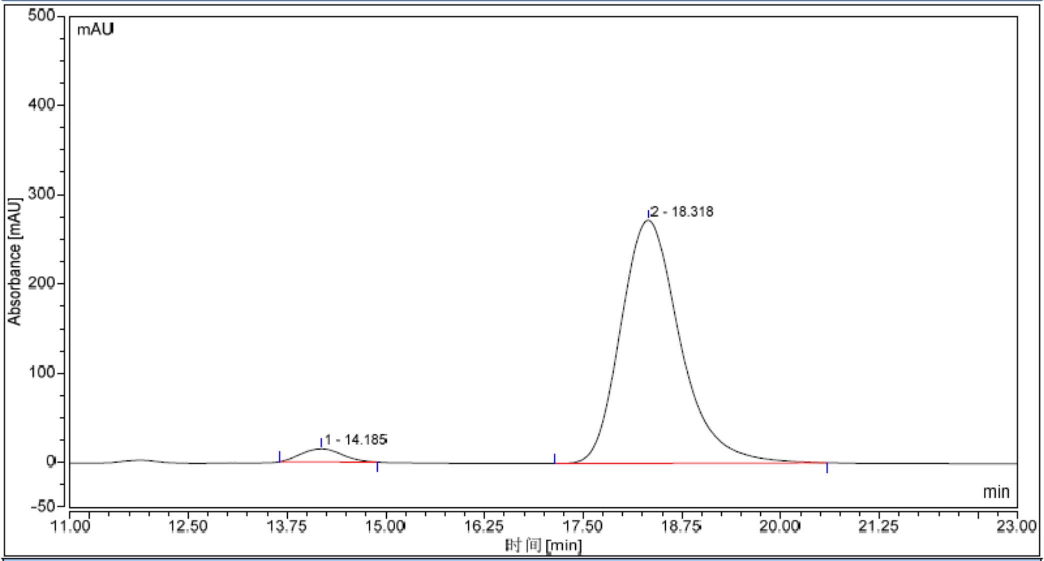

Integration Results

No.	Peak Name	Retention Time<br>min	Area<br>mAU*min	Height<br>mAU	Relative Area<br>%	Relative Height<br>%	Amount<br>n.a.
1		14.185	8.687	14.531	3.54	5.07	n.a.
2		18.318	236.759	272.139	96.46	94.93	n.a.
Total:			**245.447**	**286.670**	**100.00**	**100.00**	

2v: ADH, *n*-hexane/2-propanol = 80/20, v = 1.0 mL min^{-1}, λ = 254 nm

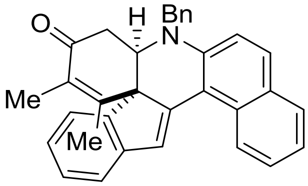

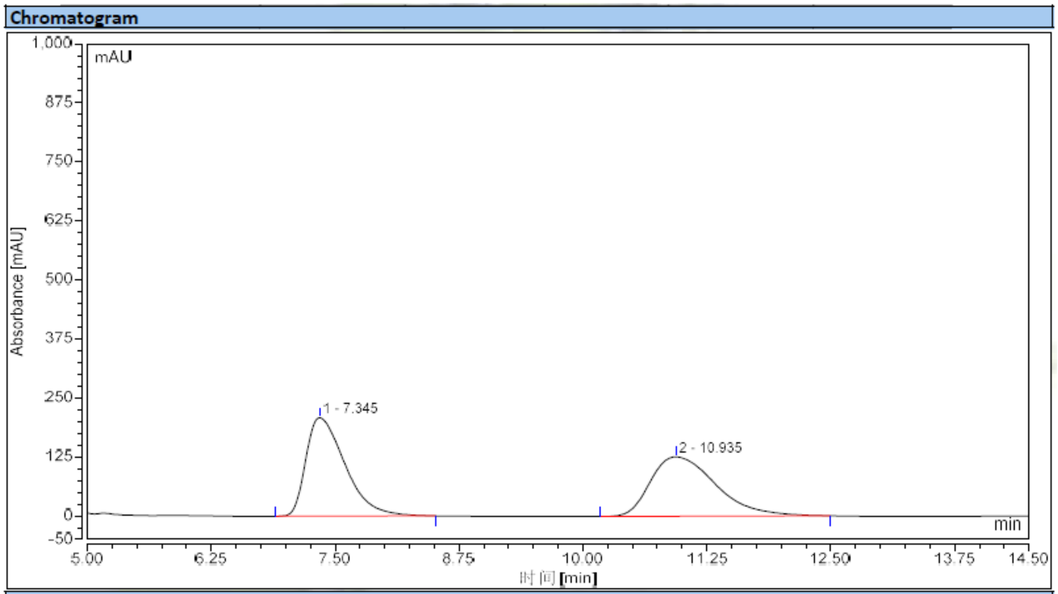

Integration Results

No.	Peak Name	Retention Time<br>min	Area<br>mAU*min	Height<br>mAU	Relative Area<br>%	Relative Height<br>%	Amount<br>n.a.
1		7.345	95.370	208.518	49.84	62.40	n.a.
2		10.935	95.980	125.649	50.16	37.60	n.a.
Total:			191.349	334.167	100.00	100.00	

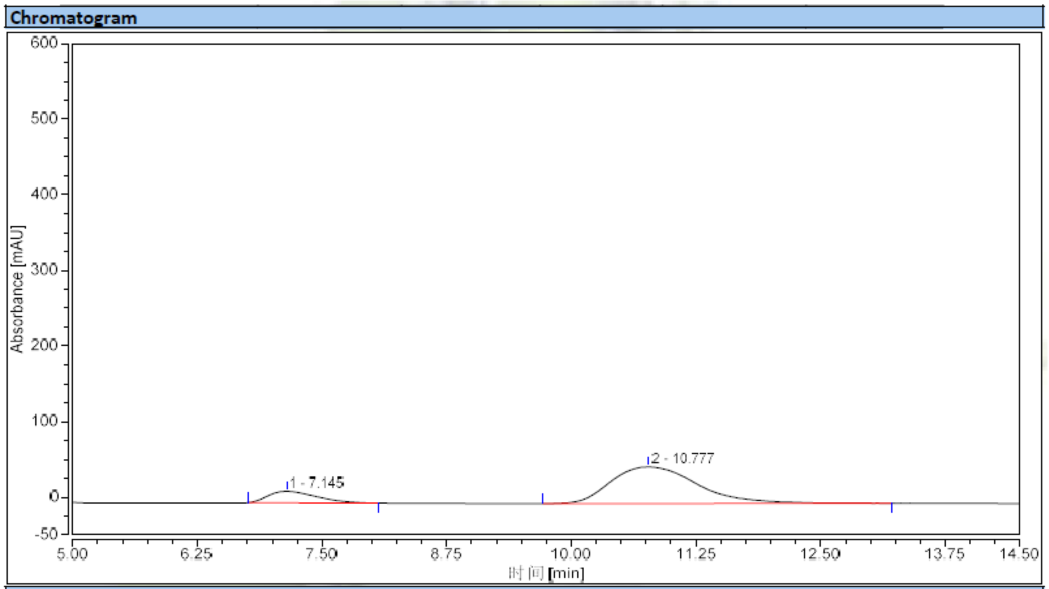

Integration Results

No.	Peak Name	Retention Time<br>min	Area<br>mAU*min	Height<br>mAU	Relative Area<br>%	Relative Height<br>%	Amount<br>n.a.
1		7.145	8.544	15.230	14.95	23.93	n.a.
2		10.777	48.597	48.409	85.05	76.07	n.a.
Total:			57.141	63.639	100.00	100.00	

2w: ADH, *n*-hexane/2-propanol = 80/20, v = 1.0 mL min^{-1}, λ = 254 nm

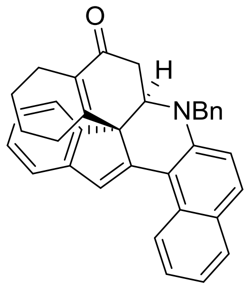

Chromatogram

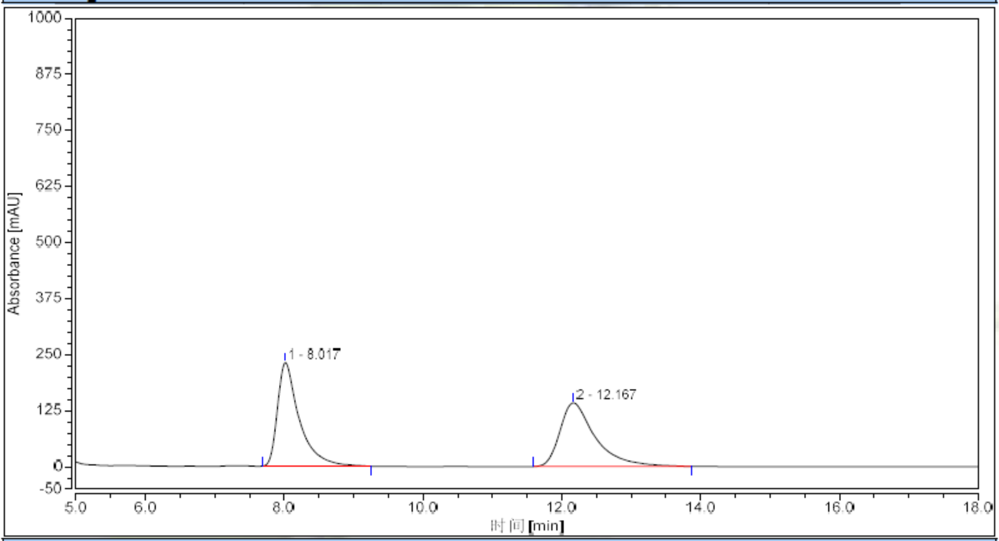

Integration Results

No.	Peak Name	Retention Time min	Area mAU*min	Height mAU	Relative Area %	Relative Height %	Amount n.a.
1		8.017	84.077	231.080	50.12	61.99	n.a.
2		12.167	83.669	141.719	49.88	38.01	n.a.
Total:			167.746	372.799	100.00	100.00	

Chromatogram

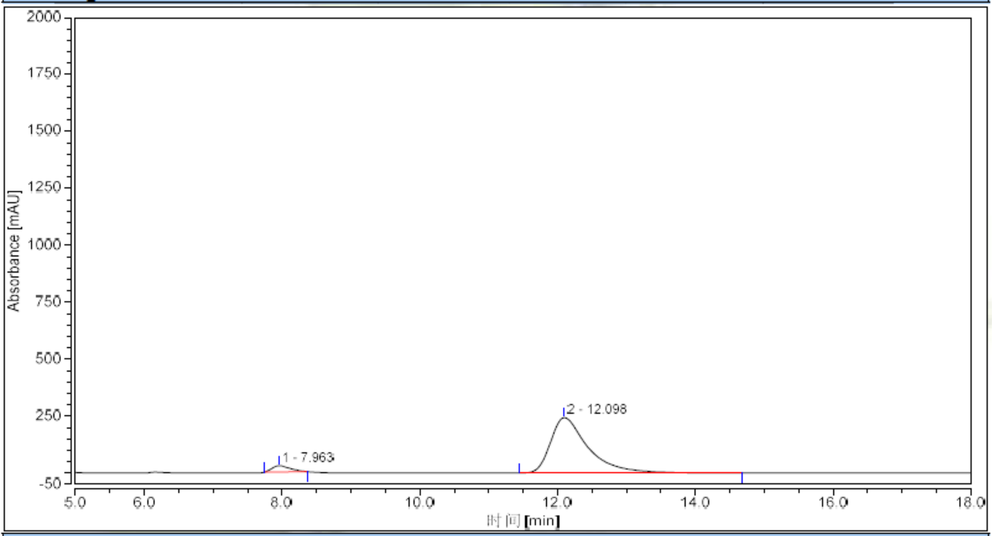

Integration Results

No.	Peak Name	Retention Time min	Area mAU*min	Height mAU	Relative Area %	Relative Height %	Amount n.a.
1		7.963	8.104	26.906	4.99	9.95	n.a.
2		12.098	154.219	243.435	95.01	90.05	n.a.
Total:			162.323	270.342	100.00	100.00	

2x: ADH, *n*-hexane/2-propanol = 80/20, v = 1.0 mL min^{-1}, λ = 254 nm

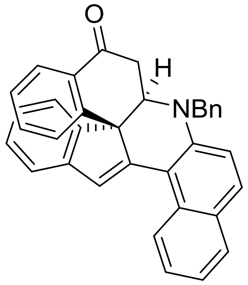

Chromatogram

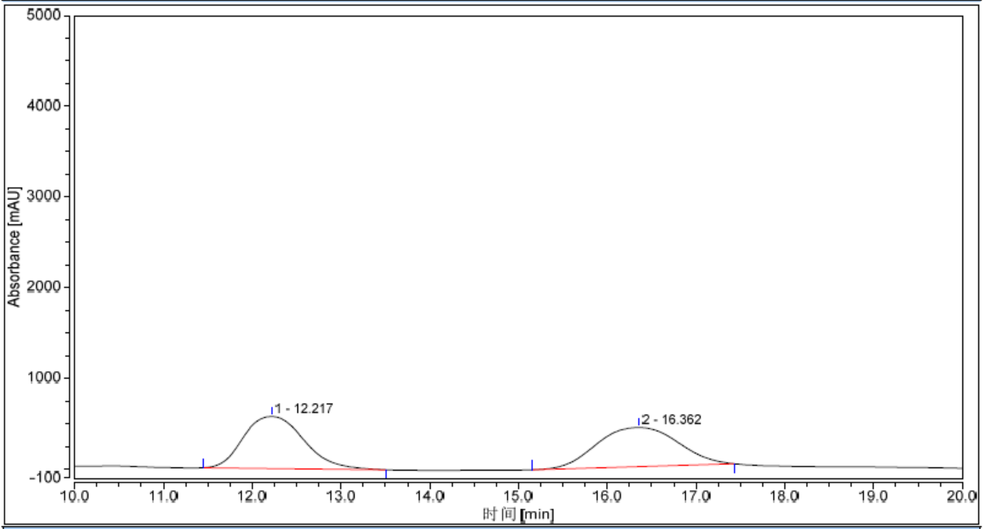

Integration Results

No.	Peak Name	Retention Time min	Area mAU*min	Height mAU	Relative Area %	Relative Height %	Amount n.a.
1		12.217	452.069	573.128	49.75	57.07	n.a.
2		16.362	456.551	431.058	50.25	42.93	n.a.
**Total:**			**908.620**	**1004.186**	**100.00**	**100.00**	

Chromatogram

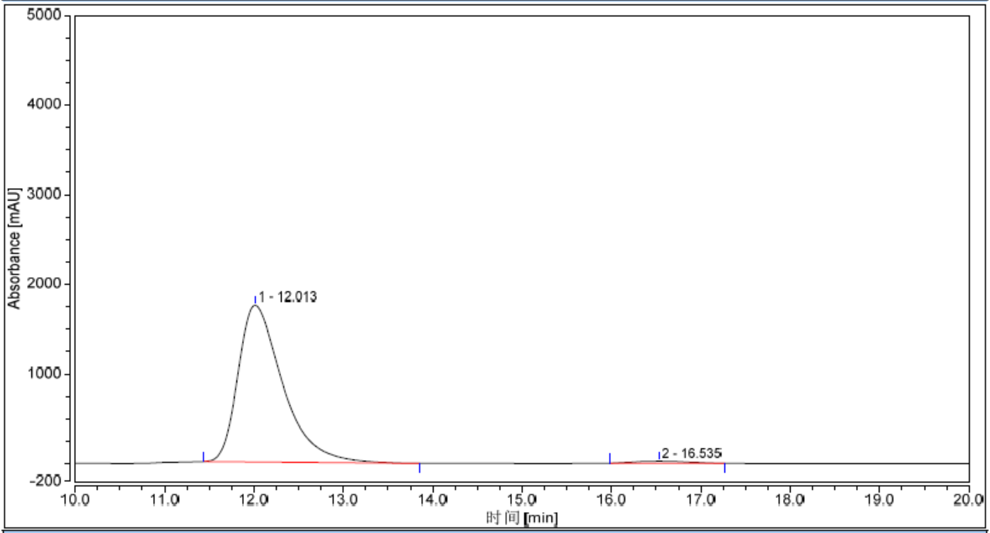

Integration Results

No.	Peak Name	Retention Time min	Area mAU*min	Height mAU	Relative Area %	Relative Height %	Amount n.a.
1		12.013	1013.003	1748.573	98.53	98.74	n.a.
2		16.535	15.112	22.357	1.47	1.26	n.a.
**Total:**			**1028.115**	**1770.930**	**100.00**	**100.00**	

2a': ADH, *n*-hexane/2-propanol = 80/20, v = 1.0 mL min^{-1}, λ = 254 nm

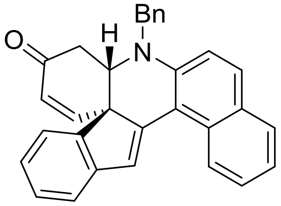

Chromatogram

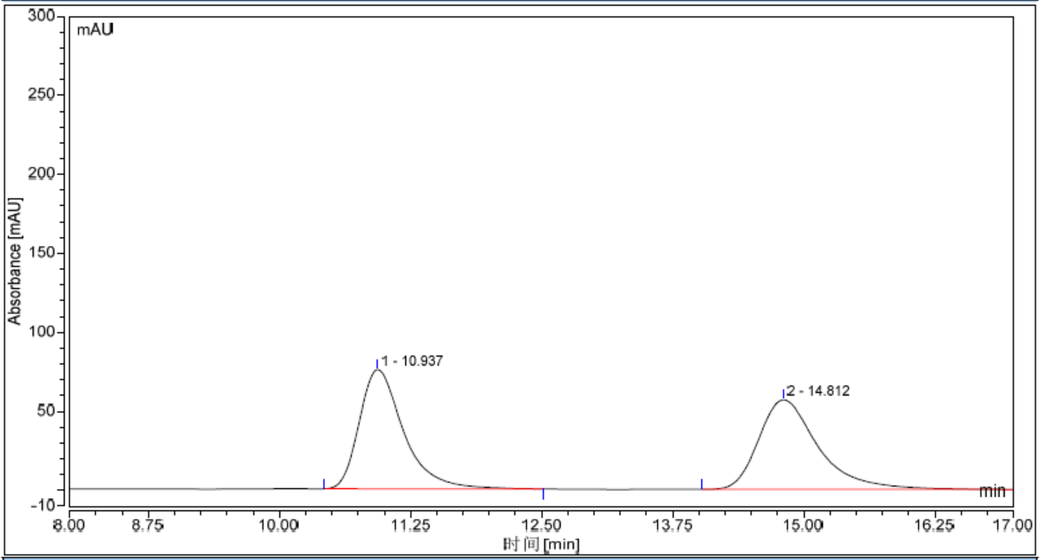

Integration Results

No.	Peak Name	Retention Time min	Area mAU*min	Height mAU	Relative Area %	Relative Height %	Amount n.a.
1		10.937	36.951	75.437	49.71	57.12	n.a.
2		14.812	37.389	56.636	50.29	42.88	n.a.
Total:			**74.339**	**132.073**	**100.00**	**100.00**	

Chromatogram

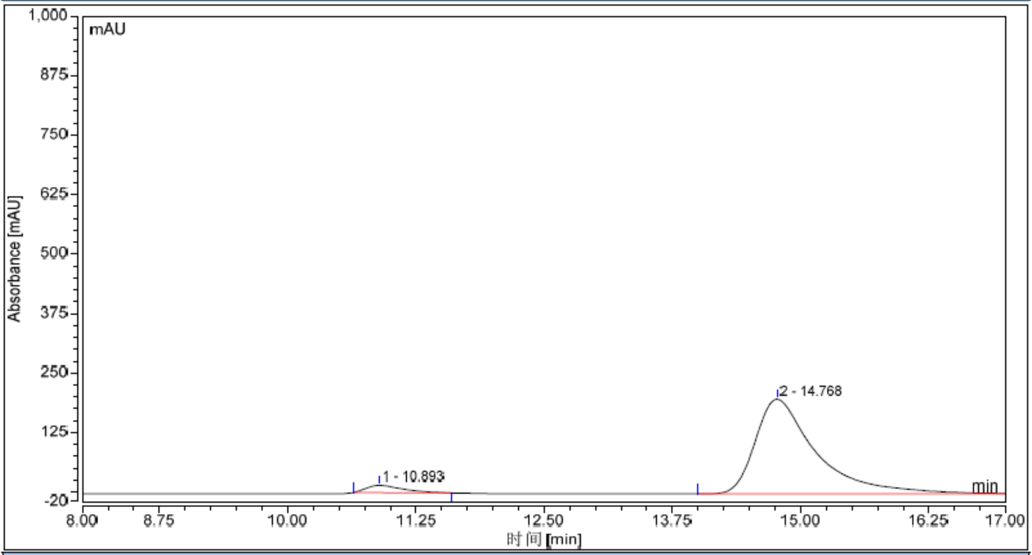

Integration Results

No.	Peak Name	Retention Time min	Area mAU*min	Height mAU	Relative Area %	Relative Height %	Amount n.a.
1		10.893	6.179	15.201	4.31	7.11	n.a.
2		14.768	137.217	198.741	95.69	92.89	n.a.
Total:			**143.396**	**213.942**	**100.00**	**100.00**	

4a: IG, *n*-hexane/2-propanol = 90/10, v = 1.0 mL min^{-1}, λ = 254 nm

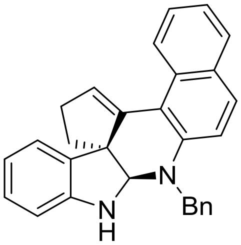

Chromatogram

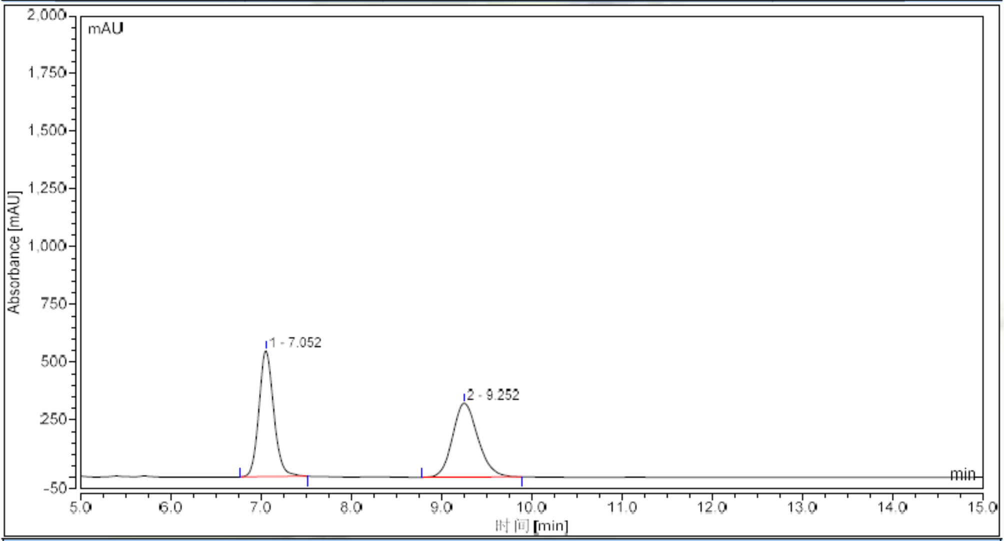

Integration Results

No.	Peak Name	Retention Time min	Area mAU*min	Height mAU	Relative Area %	Relative Height %	Amount n.a.
1		7.052	103.789	545.823	49.88	63.04	n.a.
2		9.252	104.270	320.051	50.12	36.96	n.a.
Total:			208.059	865.873	100.00	100.00	

Chromatogram

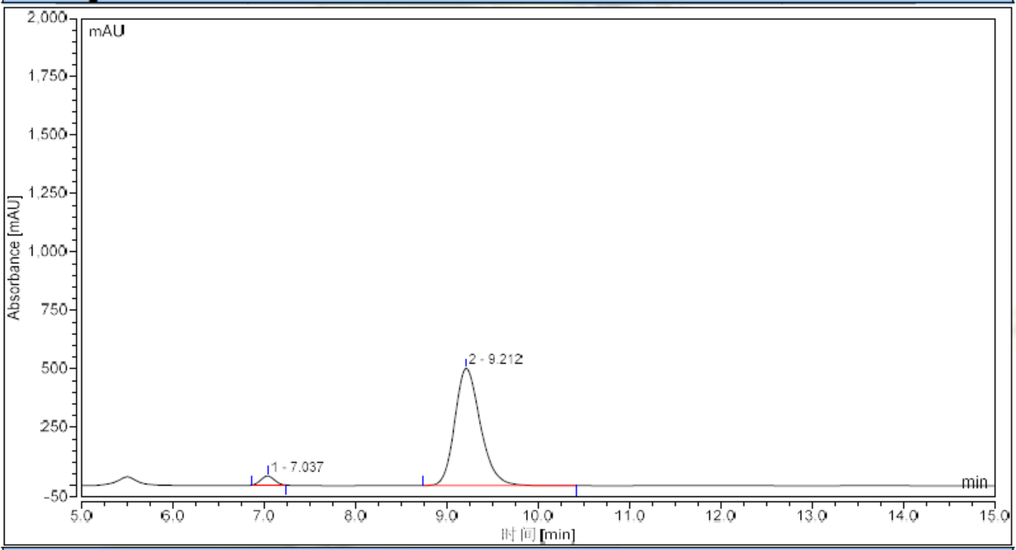

Integration Results

No.	Peak Name	Retention Time min	Area mAU*min	Height mAU	Relative Area %	Relative Height %	Amount n.a.
1		7.037	6.641	39.287	3.92	7.26	n.a.
2		9.212	162.936	501.839	96.08	92.74	n.a.
Total:			169.578	541.126	100.00	100.00	

4b: IG, *n*-hexane/2-propanol = 80/20, v = 1.0 mL min^{-1}, λ = 254 nm

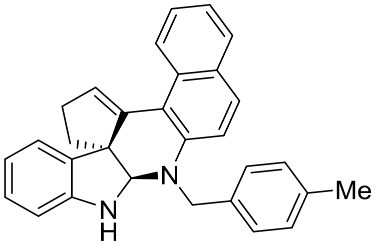

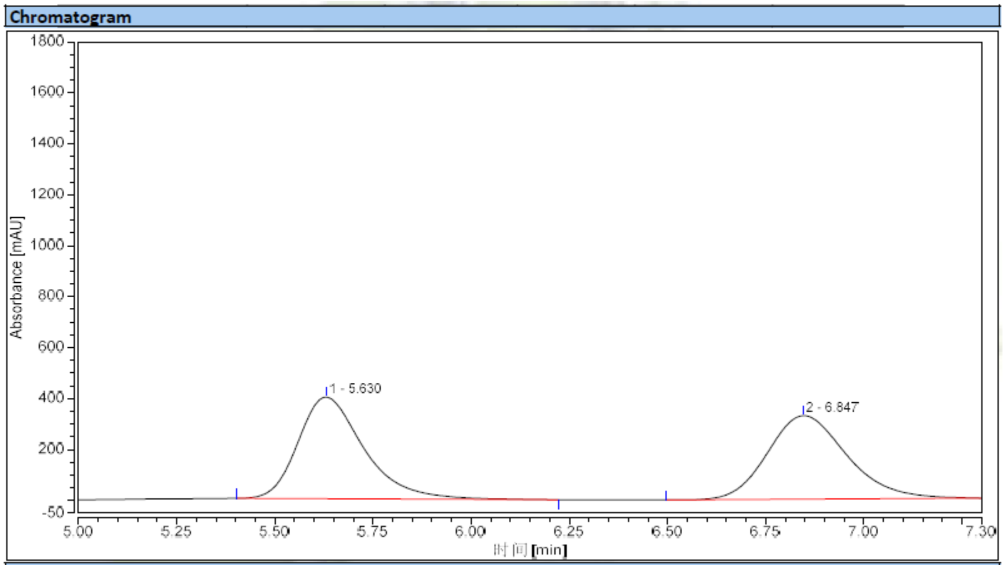

Integration Results

No.	Peak Name	Retention Time min	Area mAU*min	Height mAU	Relative Area %	Relative Height %	Amount n.a.
1		5.630	77.383	398.164	50.22	54.88	n.a.
2		6.847	76.715	327.369	49.78	45.12	n.a.
Total:			154.098	725.533	100.00	100.00	

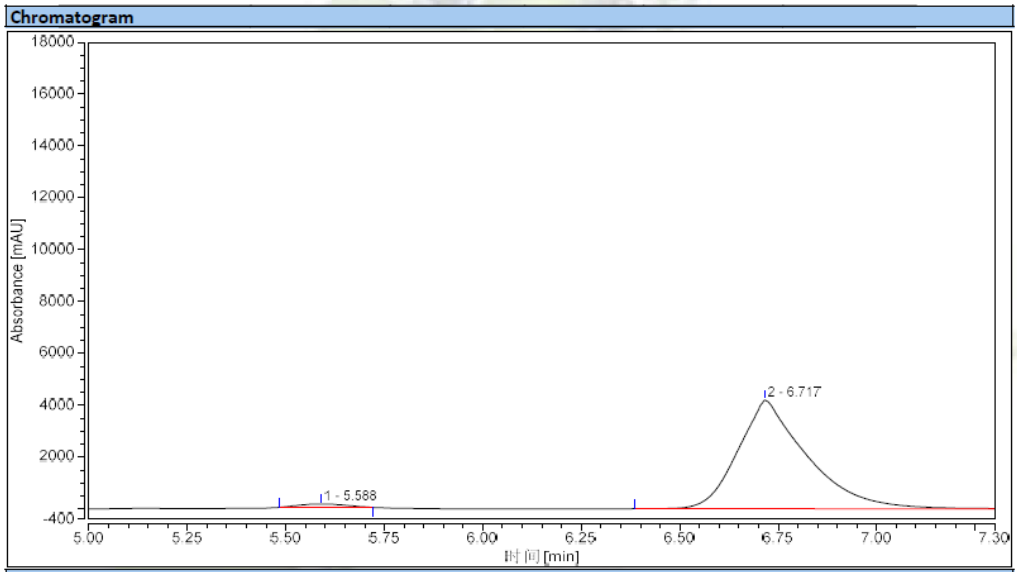

Integration Results

No.	Peak Name	Retention Time min	Area mAU*min	Height mAU	Relative Area %	Relative Height %	Amount n.a.
1		5.588	17.845	132.076	2.05	3.06	n.a.
2		6.717	852.210	4189.361	97.95	96.94	n.a.
Total:			870.055	4321.437	100.00	100.00	

4c: IG, *n*-hexane/2-propanol = 80/20, v = 1.0 mL·min^{-1}, λ = 254 nm

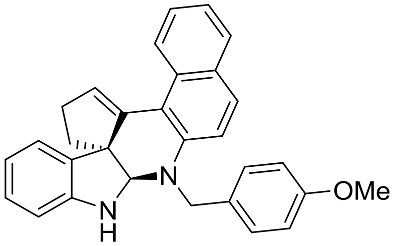

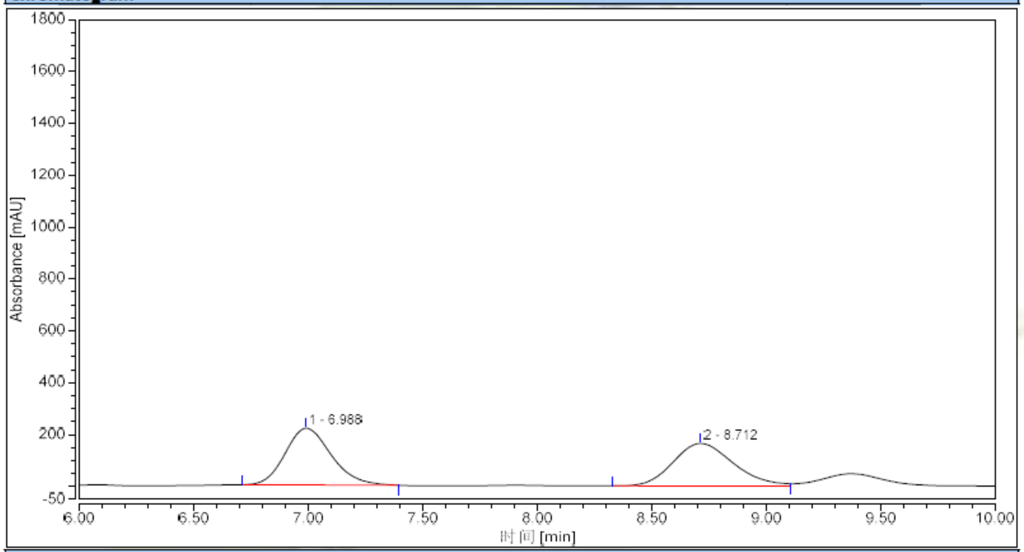

Integration Results

No.	Peak Name	Retention Time<br>min	Area<br>mAU*min	Height<br>mAU	Relative Area<br>%	Relative Height<br>%	Amount<br>n.a.
1		6.988	50.970	218.647	50.39	57.13	n.a.
2		8.712	50.189	164.054	49.61	42.87	n.a.
Total:			101.159	382.700	100.00	100.00	

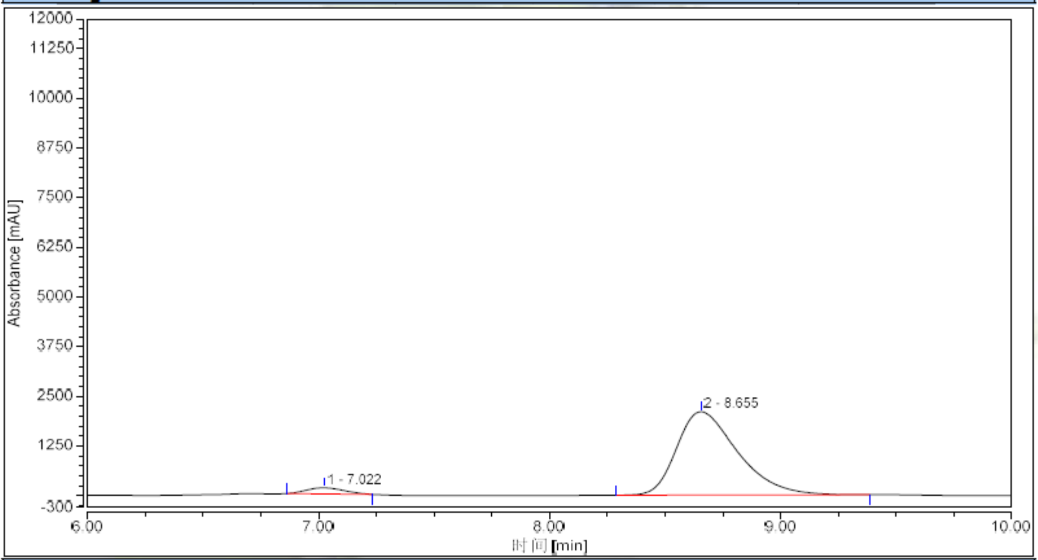

Integration Results

No.	Peak Name	Retention Time<br>min	Area<br>mAU*min	Height<br>mAU	Relative Area<br>%	Relative Height<br>%	Amount<br>n.a.
1		7.022	29.203	155.596	4.37	6.91	n.a.
2		8.655	639.687	2095.993	95.63	93.09	n.a.
Total:			668.891	2251.589	100.00	100.00	

4d: ADH, *n*-hexane/2-propanol = 90/10, v = 1.0 mL min^{-1}, λ = 254 nm

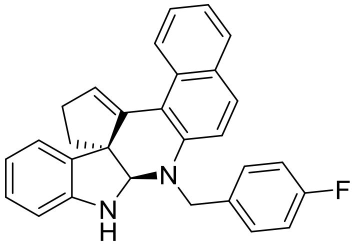

Chromatogram

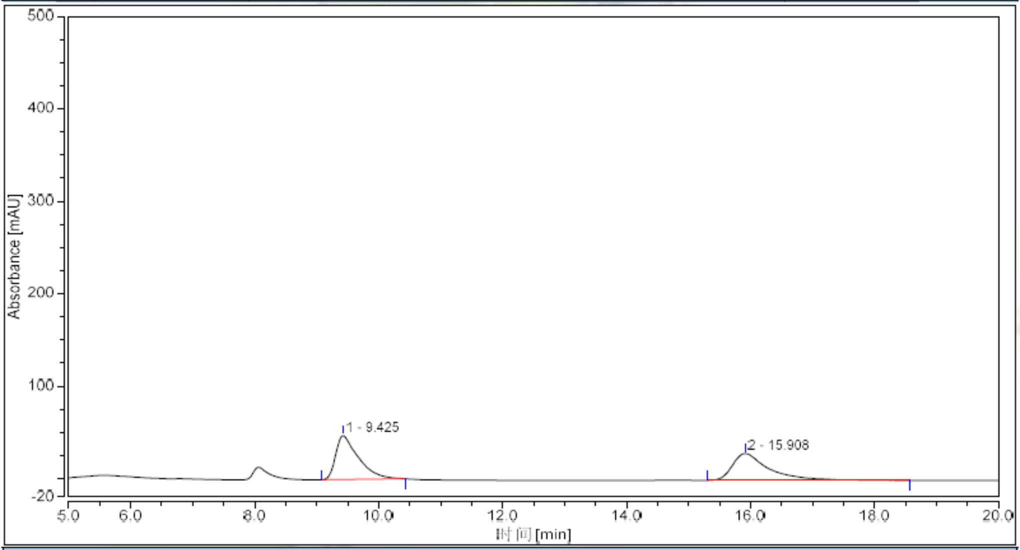

Integration Results

No.	Peak Name	Retention Time min	Area mAU*min	Height mAU	Relative Area %	Relative Height %	Amount n.a.
1		9.425	20.181	47.474	50.55	62.03	n.a.
2		15.908	19.743	29.056	49.45	37.97	n.a.
Total:			39.924	76.530	100.00	100.00	

Chromatogram

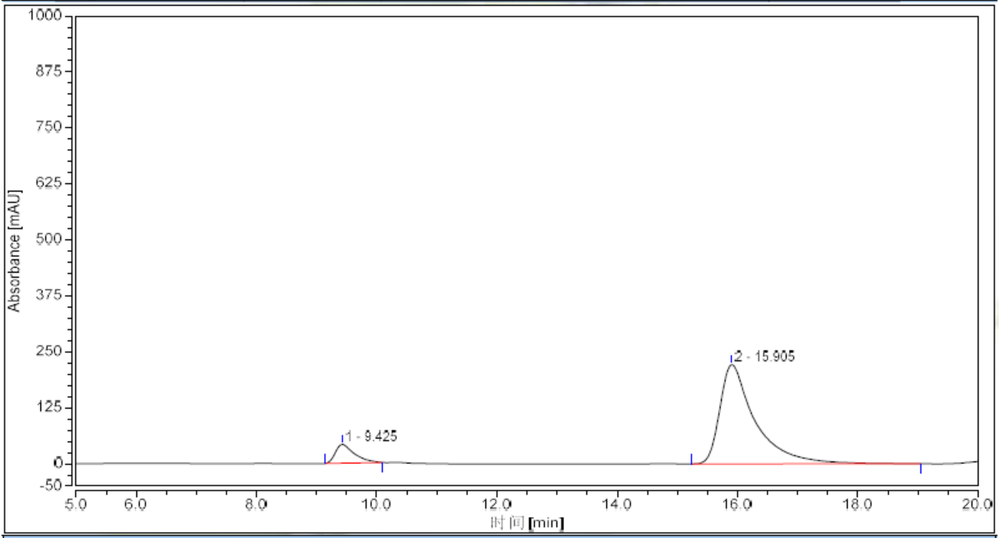

Integration Results

No.	Peak Name	Retention Time min	Area mAU*min	Height mAU	Relative Area %	Relative Height %	Amount n.a.
1		9.425	15.408	41.928	9.27	15.92	n.a.
2		15.905	150.766	221.443	90.73	84.08	n.a.
Total:			166.174	263.371	100.00	100.00	

4e: IG, *n*-hexane/2-propanol = 80/20, v = 1.0 mL min^{-1}, λ = 254 nm

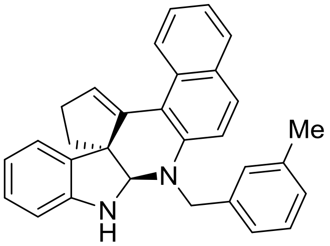

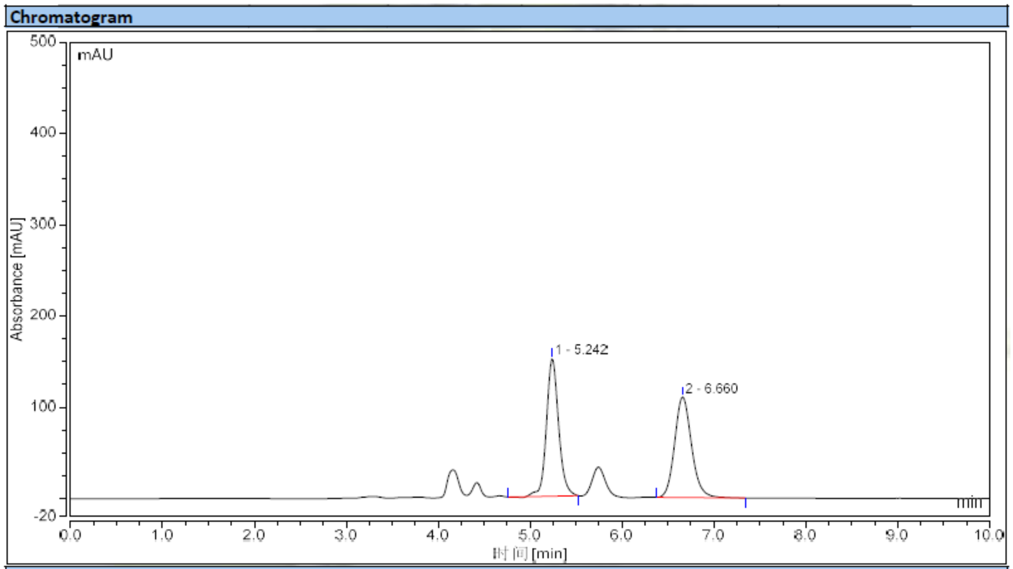

Integration Results

No.	Peak Name	Retention Time min	Area mAU*min	Height mAU	Relative Area %	Relative Height %	Amount n.a.
1		5.242	23.118	150.961	49.72	57.78	n.a.
2		6.660	23.382	110.313	50.28	42.22	n.a.
Total:			46.500	261.274	100.00	100.00	

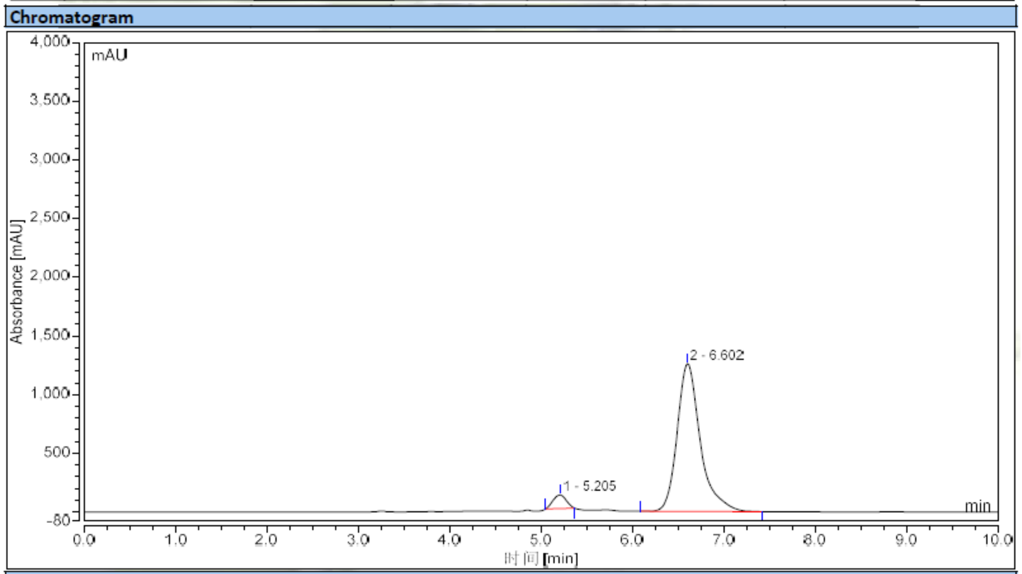

Integration Results

No.	Peak Name	Retention Time min	Area mAU*min	Height mAU	Relative Area %	Relative Height %	Amount n.a.
1		5.205	19.163	114.562	4.94	8.34	n.a.
2		6.602	368.900	1259.123	95.06	91.66	n.a.
Total:			388.064	1373.685	100.00	100.00	

4f: ADH, *n*-hexane/2-propanol = 80/20, v = 1.0 mL min^{-1}, λ = 254 nm

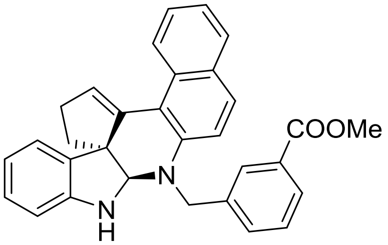

Chromatogram

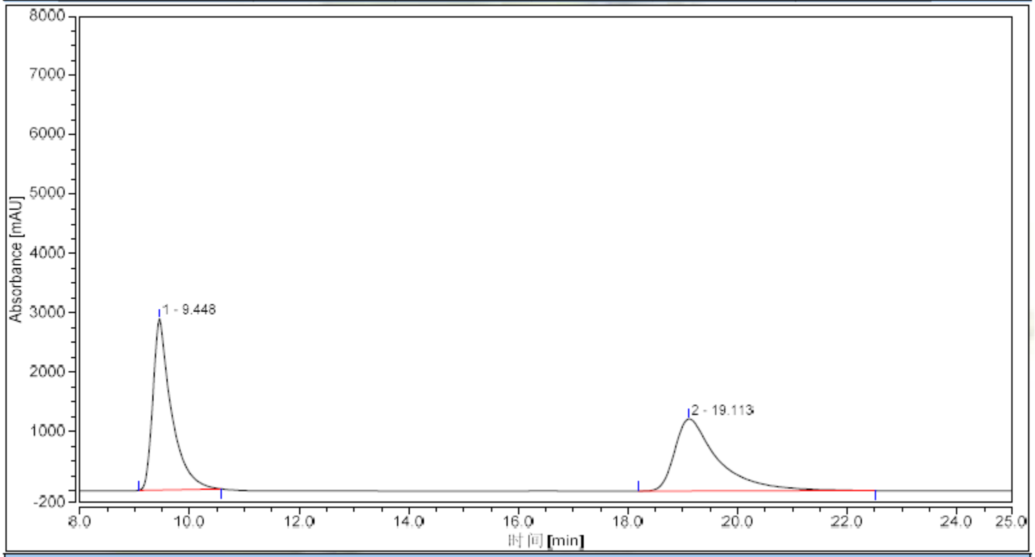

Integration Results

No.	Peak Name	Retention Time<br>min	Area<br>mAU*min	Height<br>mAU	Relative Area<br>%	Relative Height<br>%	Amount<br>n.a.
1		9.448	1129.302	2888.586	50.41	70.44	n.a.
2		19.113	1110.783	1212.097	49.59	29.56	n.a.
**Total:**			**2240.085**	**4100.683**	**100.00**	**100.00**	

Chromatogram

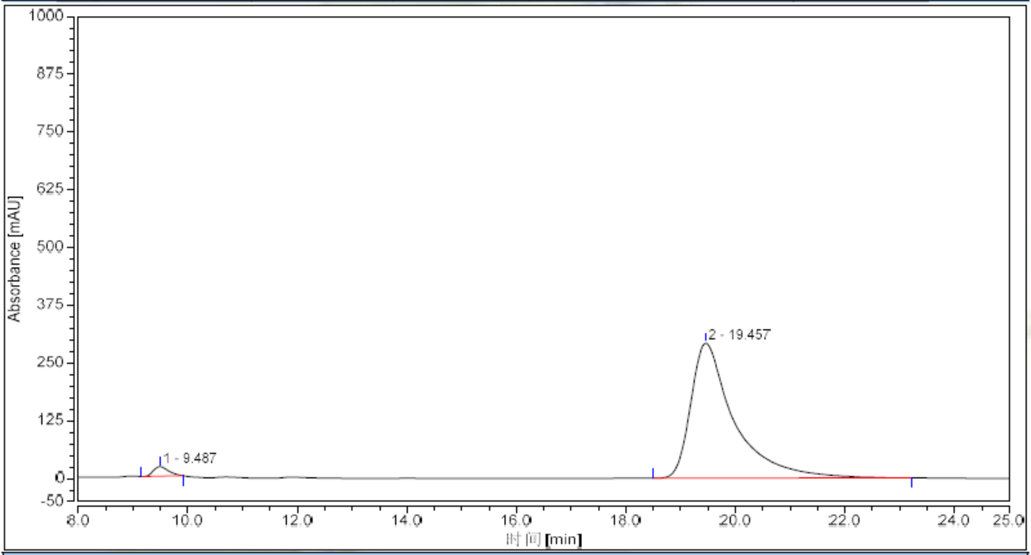

Integration Results

No.	Peak Name	Retention Time<br>min	Area<br>mAU*min	Height<br>mAU	Relative Area<br>%	Relative Height<br>%	Amount<br>n.a.
1		9.487	6.656	20.919	2.43	6.69	n.a.
2		19.457	267.550	291.981	97.57	93.31	n.a.
**Total:**			**274.206**	**312.901**	**100.00**	**100.00**	

4g: IG, *n*-hexane/2-propanol = 80/20, v = 1.0 mL min^{-1}, λ = 254 nm

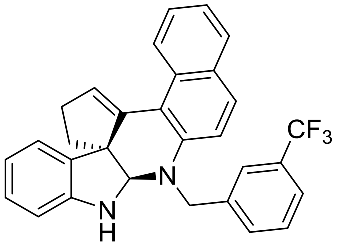

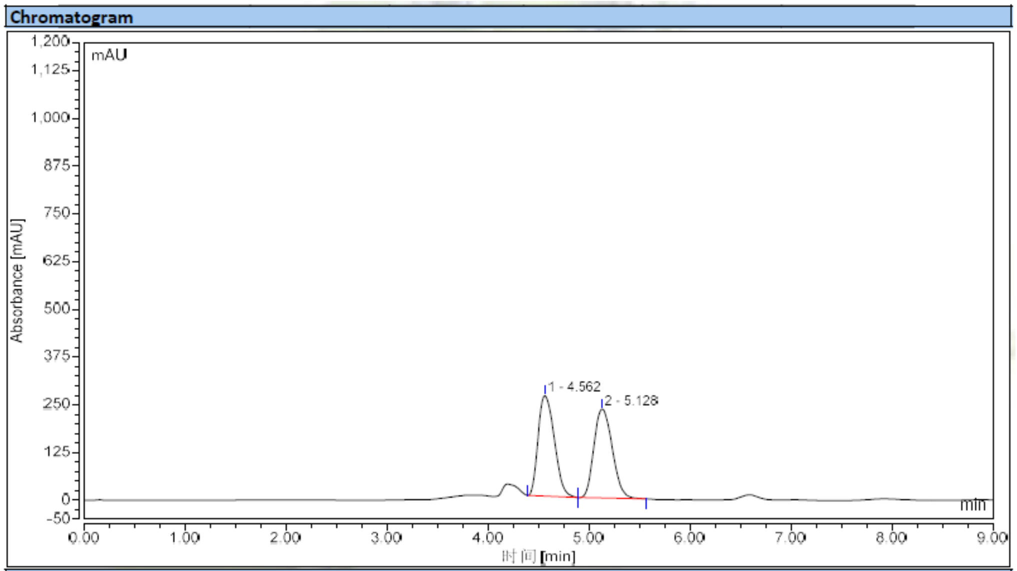

Integration Results

No.	Peak Name	Retention Time min	Area mAU*min	Height mAU	Relative Area %	Relative Height %	Amount n.a.
1		4.562	49.000	263.157	49.98	53.03	n.a.
2		5.128	49.035	233.115	50.02	46.97	n.a.
Total:			98.035	496.273	100.00	100.00	

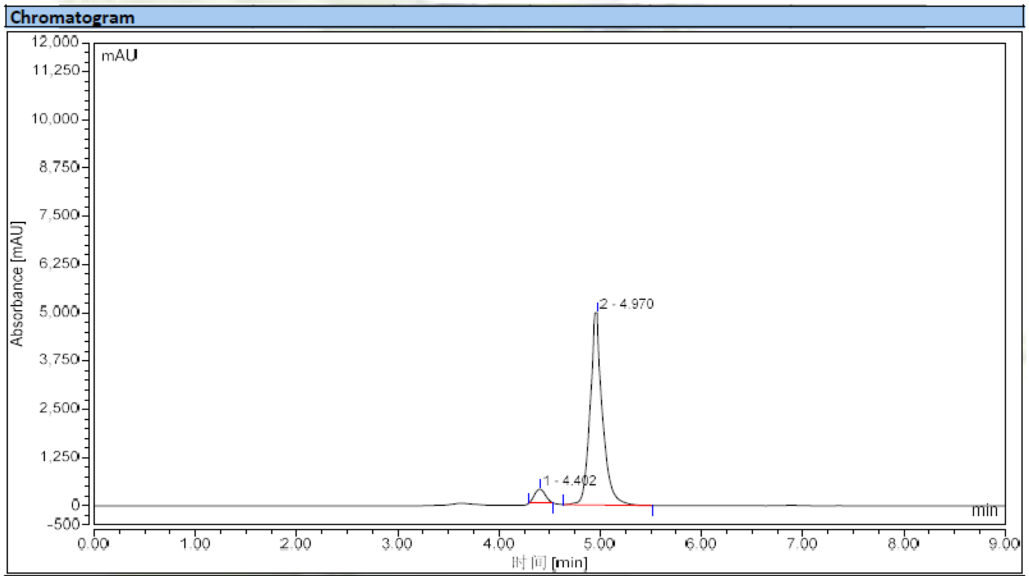

Integration Results

No.	Peak Name	Retention Time min	Area mAU*min	Height mAU	Relative Area %	Relative Height %	Amount n.a.
1		4.402	44.243	361.072	5.94	6.77	n.a.
2		4.970	701.141	4975.163	94.06	93.23	n.a.
Total:			745.384	5336.235	100.00	100.00	

4h: IG, *n*-hexane/2-propanol = 80/20, v = 1.0 mL min^{-1}, λ = 254 nm

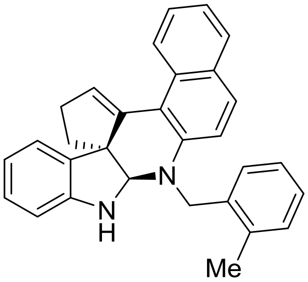

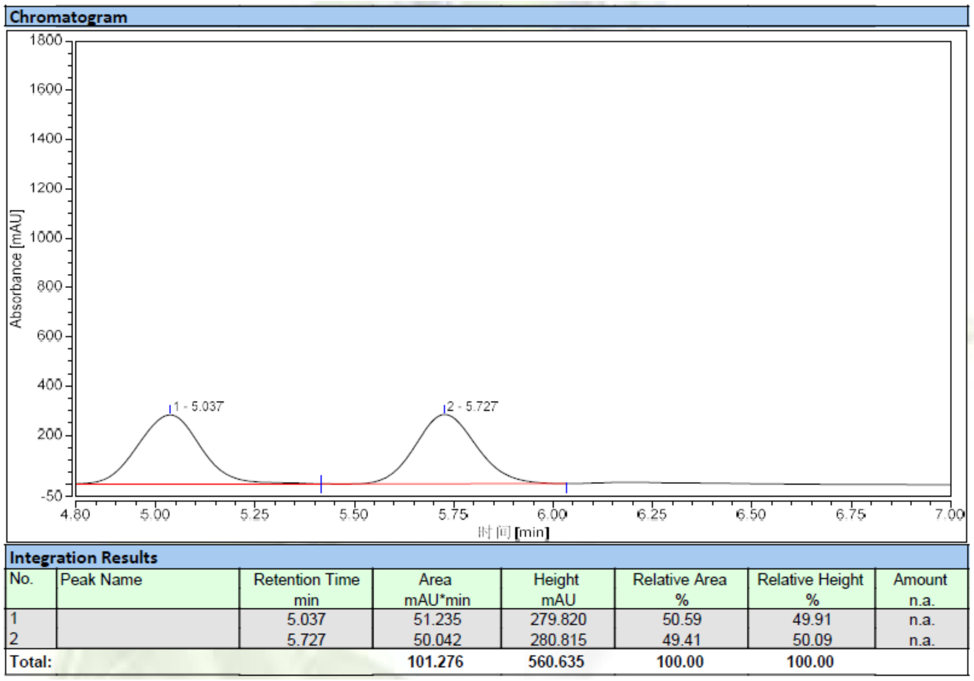

Integration Results

No.	Peak Name	Retention Time min	Area mAU*min	Height mAU	Relative Area %	Relative Height %	Amount n.a.
1		5.037	51.235	279.820	50.59	49.91	n.a.
2		5.727	50.042	280.815	49.41	50.09	n.a.
Total:			101.276	560.635	100.00	100.00	

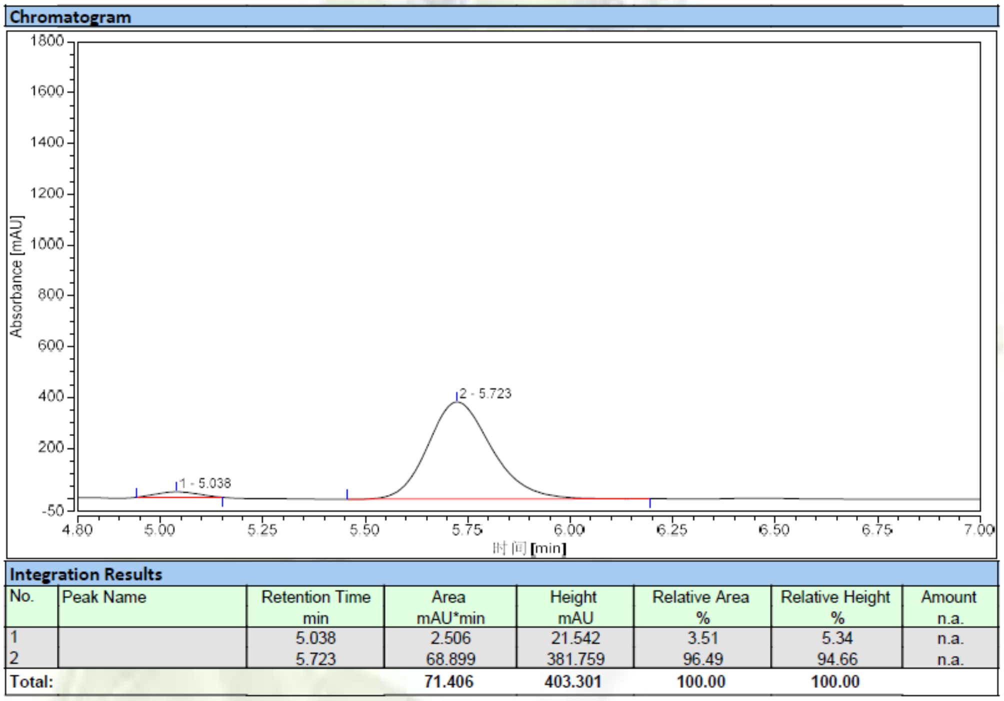

Integration Results

No.	Peak Name	Retention Time min	Area mAU*min	Height mAU	Relative Area %	Relative Height %	Amount n.a.
1		5.038	2.506	21.542	3.51	5.34	n.a.
2		5.723	68.899	381.759	96.49	94.66	n.a.
Total:			71.406	403.301	100.00	100.00	

4i: IG, *n*-hexane/2-propanol = 90/10, v = 1.0 mL min^{-1}, λ = 254 nm

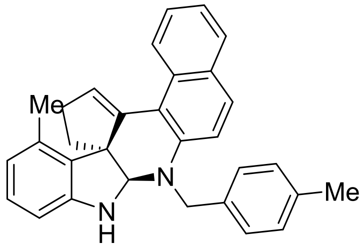

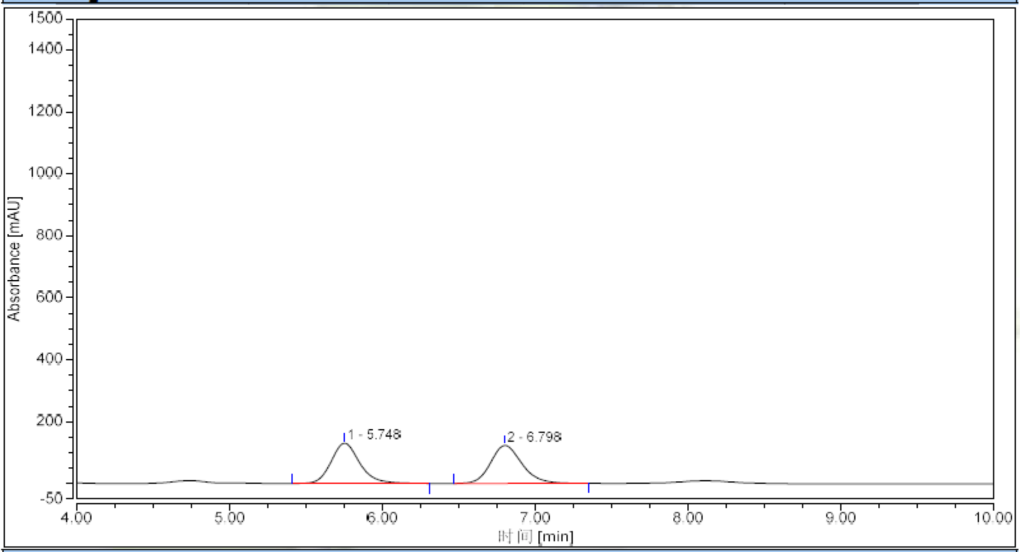

Integration Results

No.	Peak Name	Retention Time min	Area mAU*min	Height mAU	Relative Area %	Relative Height %	Amount n.a.
1		5.748	28.197	129.738	49.25	51.46	n.a.
2		6.798	29.052	122.355	50.75	48.54	n.a.
Total:			57.249	252.092	100.00	100.00	

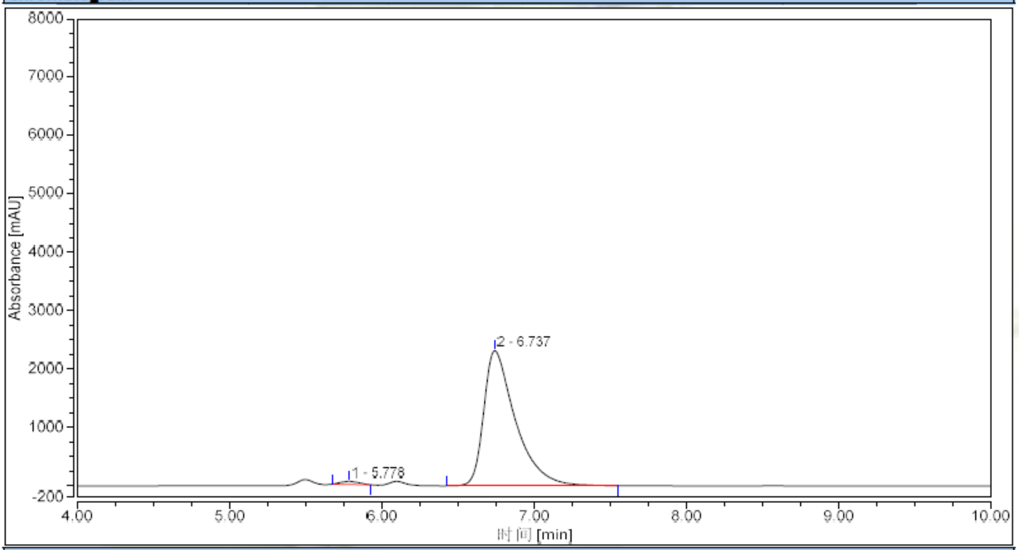

Integration Results

No.	Peak Name	Retention Time min	Area mAU*min	Height mAU	Relative Area %	Relative Height %	Amount n.a.
1		5.778	7.047	50.503	1.24	2.13	n.a.
2		6.737	559.608	2316.624	98.76	97.87	n.a.
Total:			566.656	2367.127	100.00	100.00	

4j: IG, *n*-hexane/2-propanol = 80/20, v = 1.0 mL min^{-1}, λ = 254 nm

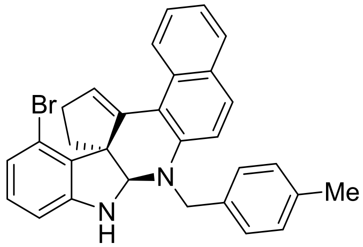

Chromatogram

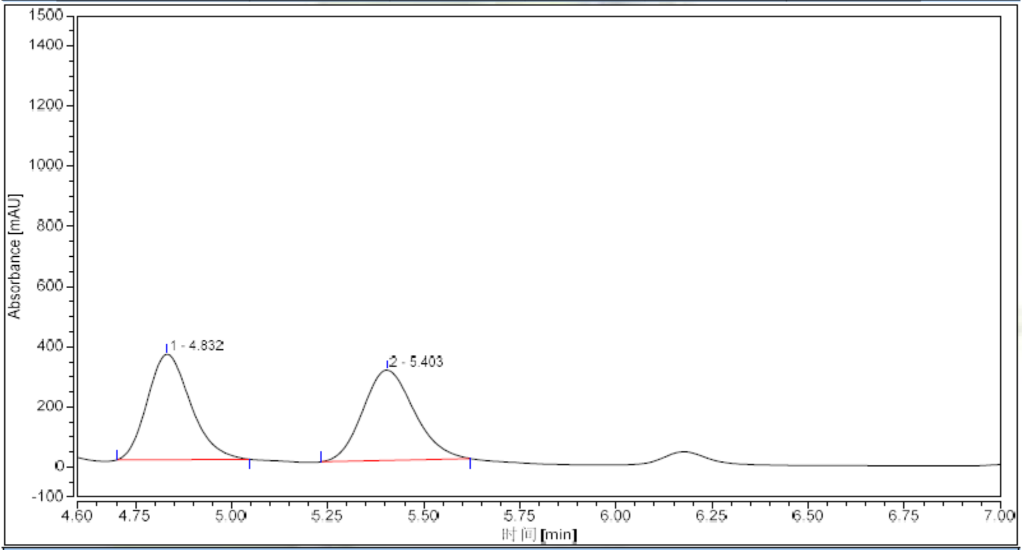

Integration Results

No.	Peak Name	Retention Time min	Area mAU*min	Height mAU	Relative Area %	Relative Height %	Amount n.a.
1		4.832	45.710	350.261	49.59	53.86	n.a.
2		5.403	46.463	300.041	50.41	46.14	n.a.
Total:			92.173	650.303	100.00	100.00	

Chromatogram

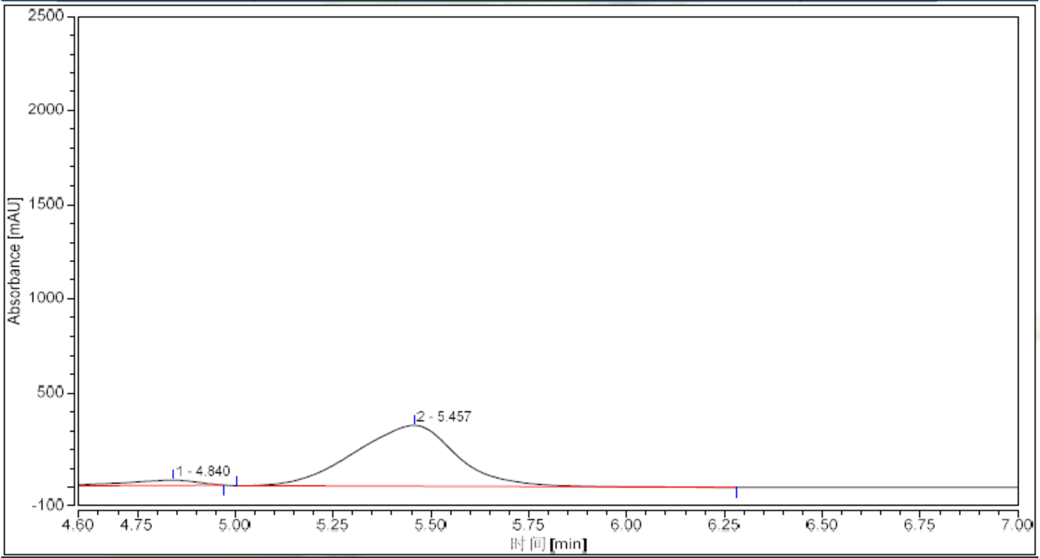

Integration Results

No.	Peak Name	Retention Time min	Area mAU*min	Height mAU	Relative Area %	Relative Height %	Amount n.a.
1		4.840	5.845	26.925	5.66	7.68	n.a.
2		5.457	97.415	323.599	94.34	92.32	n.a.
Total:			103.260	350.523	100.00	100.00	

4k: IG, *n*-hexane/2-propanol = 90/10, v = 1.0 mL min^{-1}, λ = 254 nm

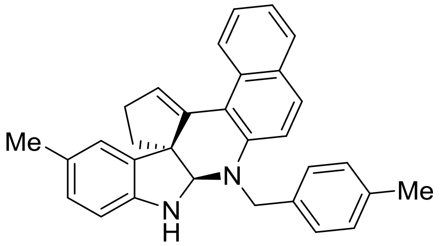

Chromatogram

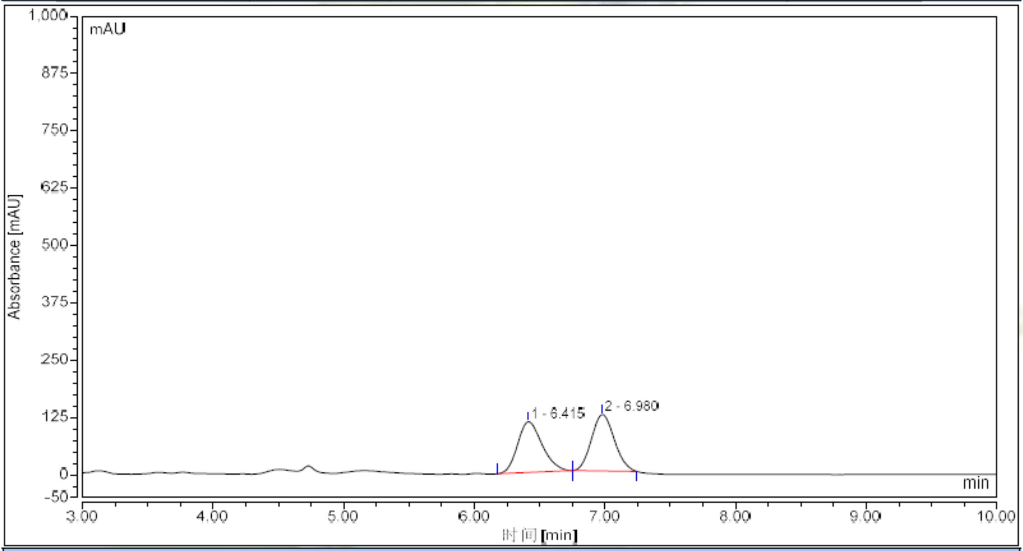

Integration Results

No.	Peak Name	Retention Time min	Area mAU*min	Height mAU	Relative Area %	Relative Height %	Amount n.a.
1		6.415	24.114	110.765	49.05	47.35	n.a.
2		6.980	25.049	123.173	50.95	52.65	n.a.
Total:			**49.163**	**233.938**	**100.00**	**100.00**	

Chromatogram

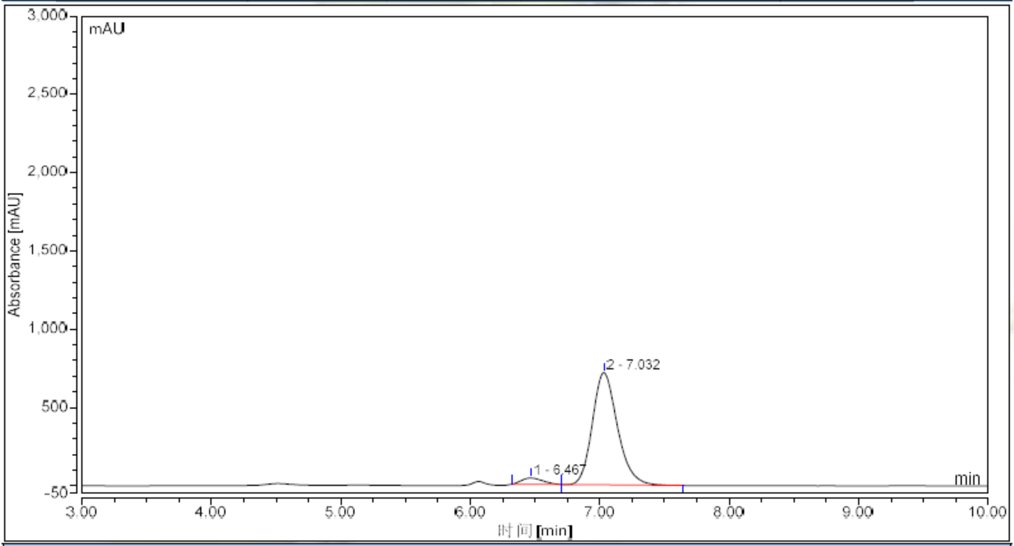

Integration Results

No.	Peak Name	Retention Time min	Area mAU*min	Height mAU	Relative Area %	Relative Height %	Amount n.a.
1		6.467	7.772	40.436	4.60	5.34	n.a.
2		7.032	161.337	716.864	95.40	94.66	n.a.
Total:			**169.108**	**757.300**	**100.00**	**100.00**	

4l: ADH, *n*-hexane/2-propanol = 95/5, v = 1.0 mL min^{-1}, λ = 254 nm

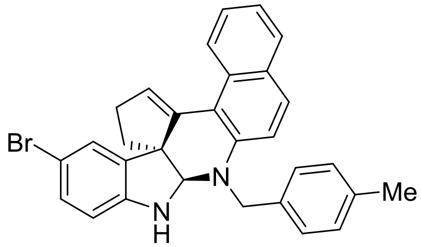

Chromatogram

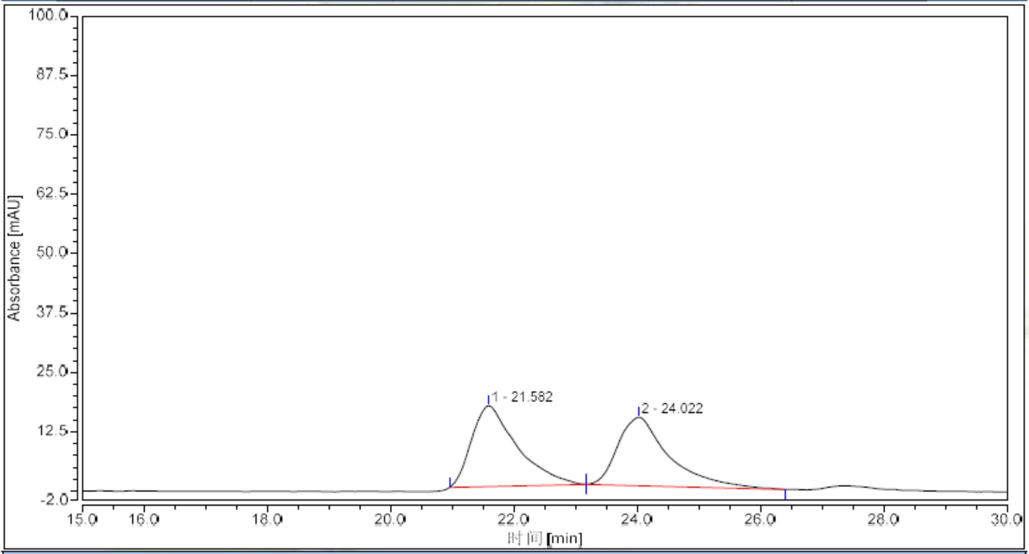

Integration Results

No.	Peak Name	Retention Time min	Area mAU*min	Height mAU	Relative Area %	Relative Height %	Amount n.a.
1		21.582	14.538	16.999	51.28	54.15	n.a.
2		24.022	13.815	14.394	48.72	45.85	n.a.
Total:			28.353	31.393	100.00	100.00	

Chromatogram

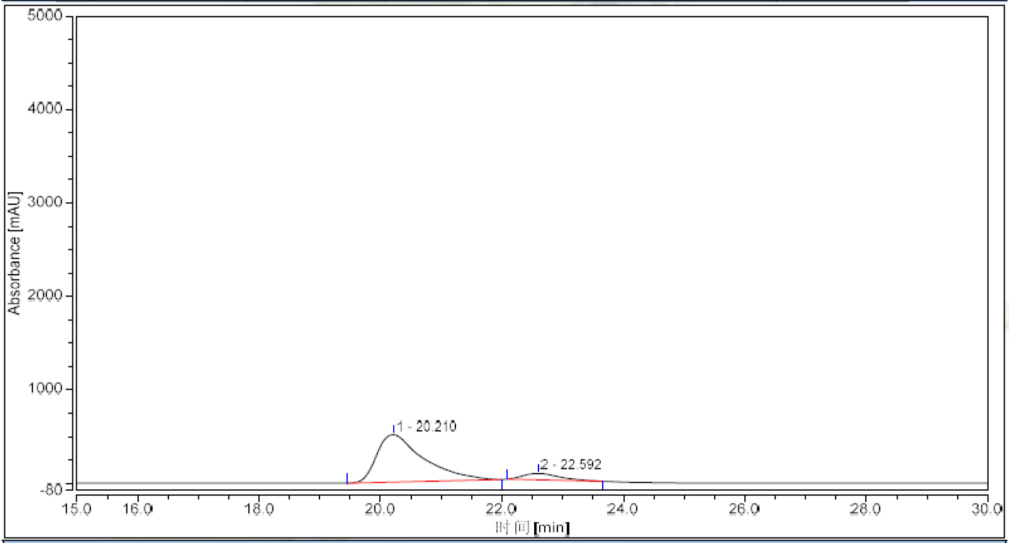

Integration Results

No.	Peak Name	Retention Time min	Area mAU*min	Height mAU	Relative Area %	Relative Height %	Amount n.a.
1		20.210	452.954	506.730	90.38	88.06	n.a.
2		22.592	48.236	68.682	9.62	11.94	n.a.
Total:			501.190	575.412	100.00	100.00	

4m: IG, *n*-hexane/2-propanol = 90/10, v = 1.0 mL min^{-1}, λ = 254 nm

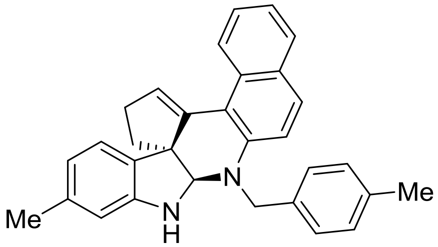

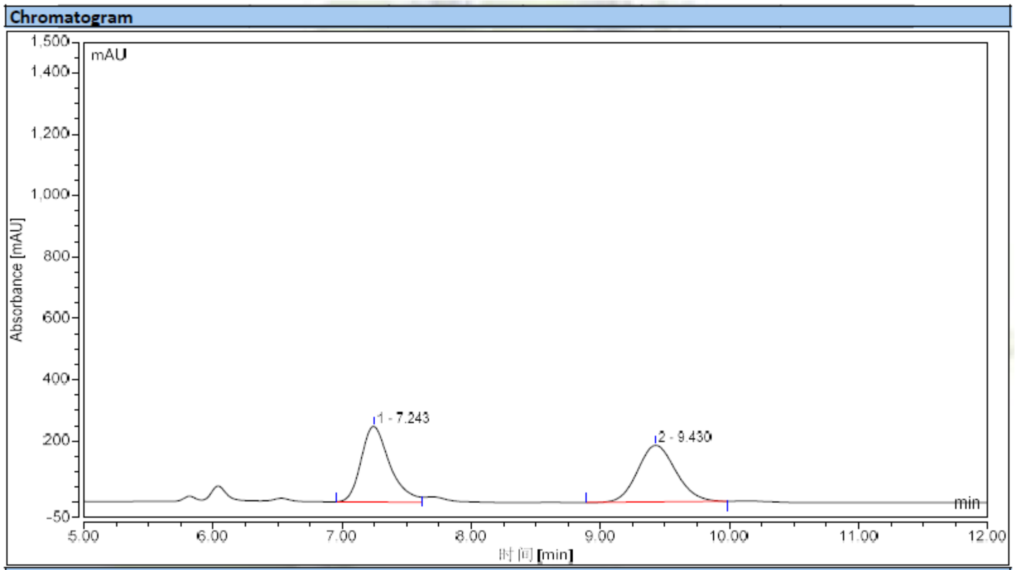

Integration Results

No.	Peak Name	Retention Time min	Area mAU*min	Height mAU	Relative Area %	Relative Height %	Amount n.a.
1		7.243	63.820	246.784	50.31	57.24	n.a.
2		9.430	63.030	184.329	49.69	42.76	n.a.
Total:			**126.849**	**431.113**	**100.00**	**100.00**	

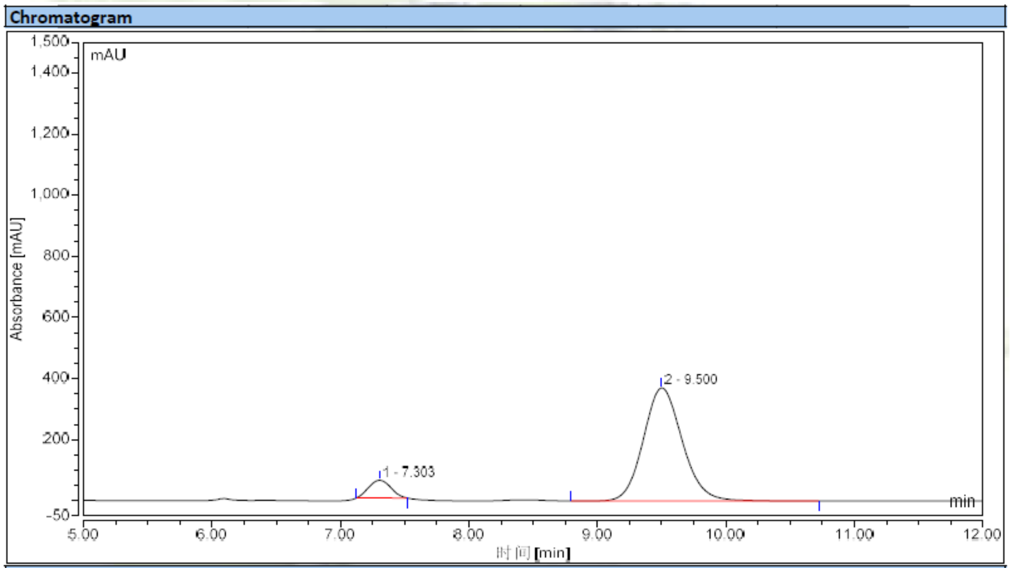

Integration Results

No.	Peak Name	Retention Time min	Area mAU*min	Height mAU	Relative Area %	Relative Height %	Amount n.a.
1		7.303	11.545	57.716	8.19	13.52	n.a.
2		9.500	129.365	369.175	91.81	86.48	n.a.
Total:			**140.911**	**426.891**	**100.00**	**100.00**	

4n: IG, *n*-hexane/2-propanol = 90/10, v = 1.0 mL min^{-1}, λ = 254 nm

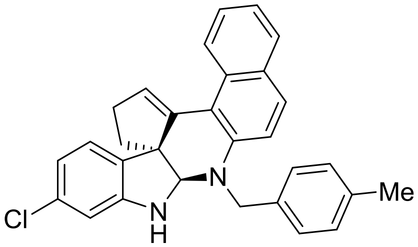

Chromatogram

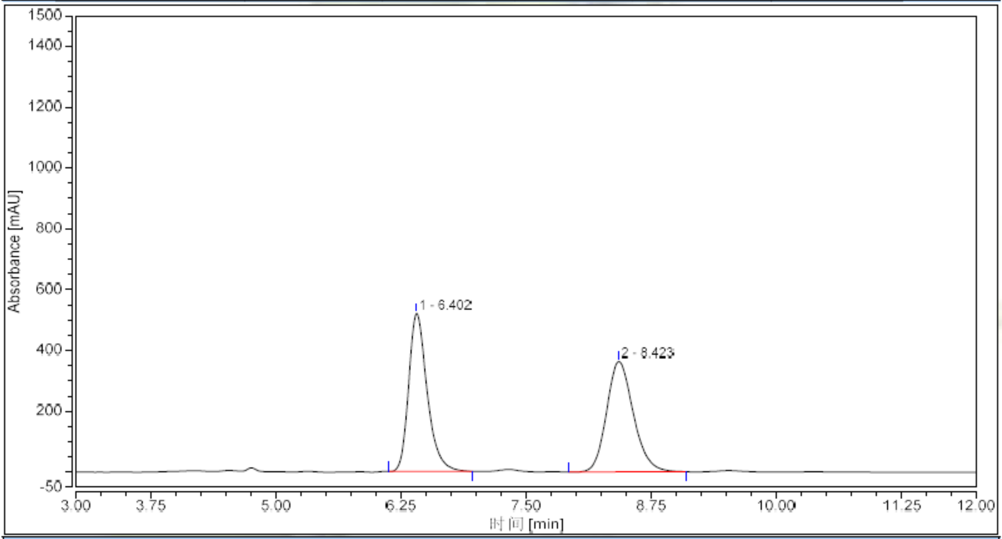

Integration Results

No.	Peak Name	Retention Time min	Area mAU*min	Height mAU	Relative Area %	Relative Height %	Amount n.a.
1		6.402	111.524	519.895	49.72	58.90	n.a.
2		8.423	112.789	362.756	50.28	41.10	n.a.
Total:			224.313	882.651	100.00	100.00	

Chromatogram

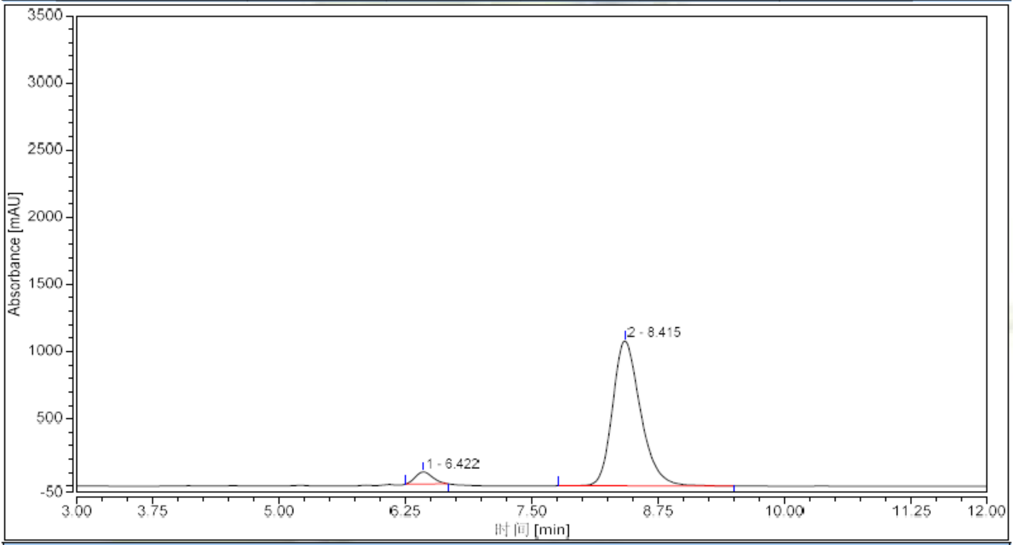

Integration Results

No.	Peak Name	Retention Time min	Area mAU*min	Height mAU	Relative Area %	Relative Height %	Amount n.a.
1		6.422	17.826	89.608	4.89	7.67	n.a.
2		8.415	346.773	1078.782	95.11	92.33	n.a.
Total:			364.598	1168.390	100.00	100.00	

4o: IG, *n*-hexane/2-propanol = 80/20, v = 1.0 mL min^{-1}, λ = 254 nm

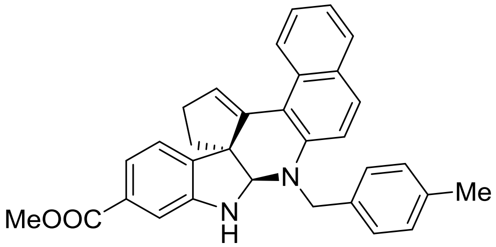

Chromatogram

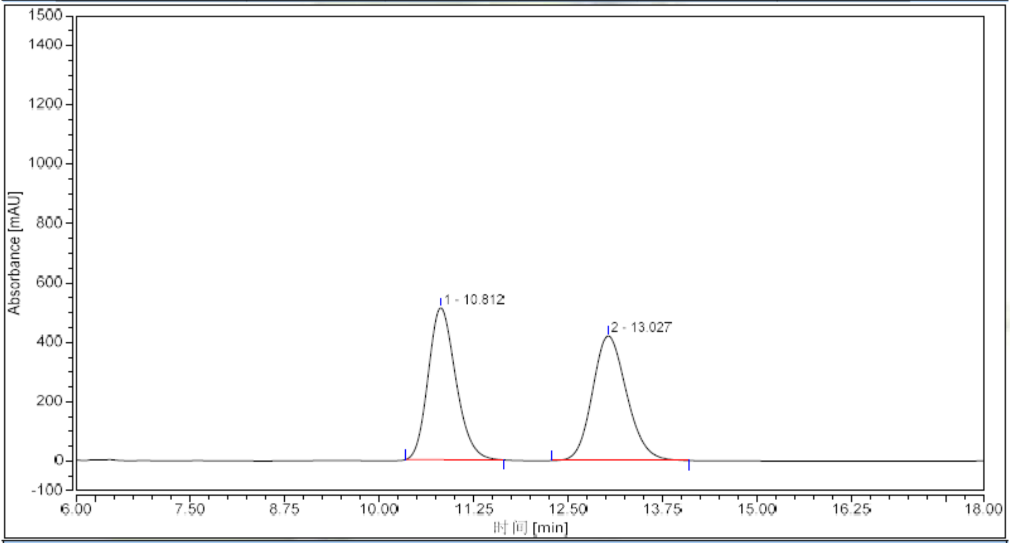

Integration Results

No.	Peak Name	Retention Time min	Area mAU*min	Height mAU	Relative Area %	Relative Height %	Amount n.a.
1		10.812	216.501	511.599	49.51	54.93	n.a.
2		13.027	220.822	419.812	50.49	45.07	n.a.
Total:			437.322	931.410	100.00	100.00	

Chromatogram

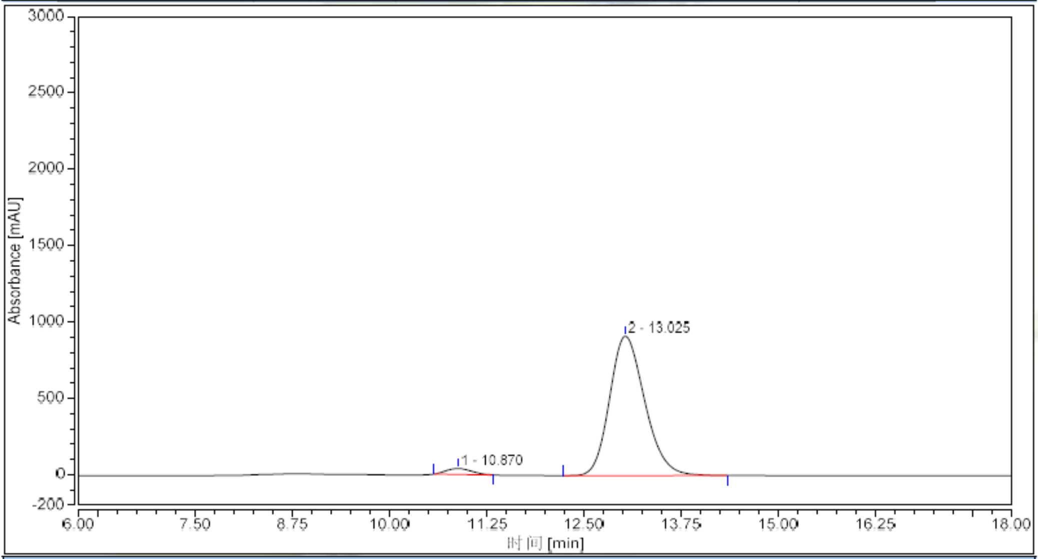

Integration Results

No.	Peak Name	Retention Time min	Area mAU*min	Height mAU	Relative Area %	Relative Height %	Amount n.a.
1		10.870	14.483	38.651	2.94	4.06	n.a.
2		13.025	478.493	912.906	97.06	95.94	n.a.
Total:			492.975	951.557	100.00	100.00	

4p: ADH, *n*-hexane/2-propanol = 90/10, v = 1.0 mL min^{-1}, λ = 254 nm

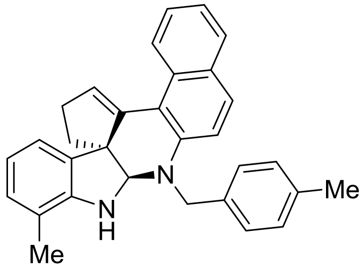

Chromatogram

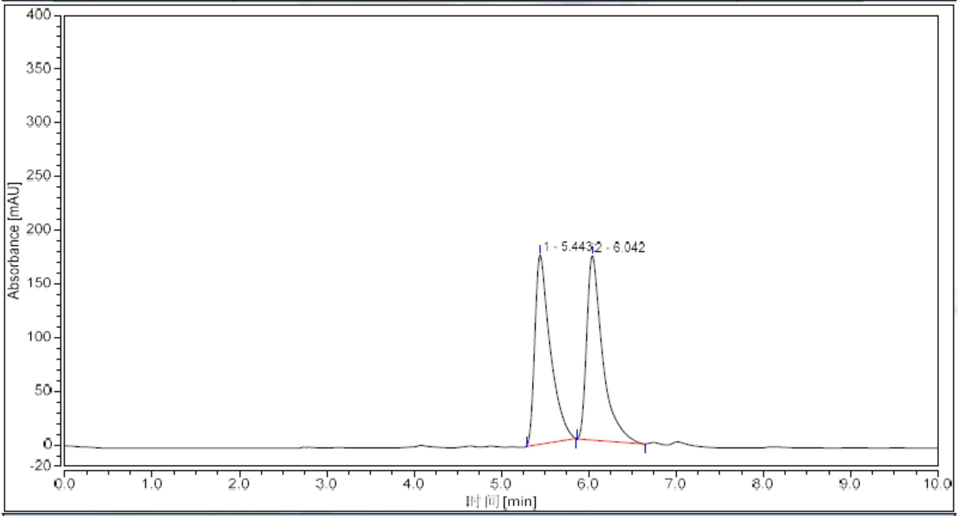

Integration Results

No.	Peak Name	Retention Time min	Area mAU*min	Height mAU	Relative Area %	Relative Height %	Amount n.a.
1		5.443	35.963	176.520	49.93	50.67	n.a.
2		6.042	36.060	171.880	50.07	49.33	n.a.
**Total:**			**72.024**	**348.400**	**100.00**	**100.00**	

Chromatogram

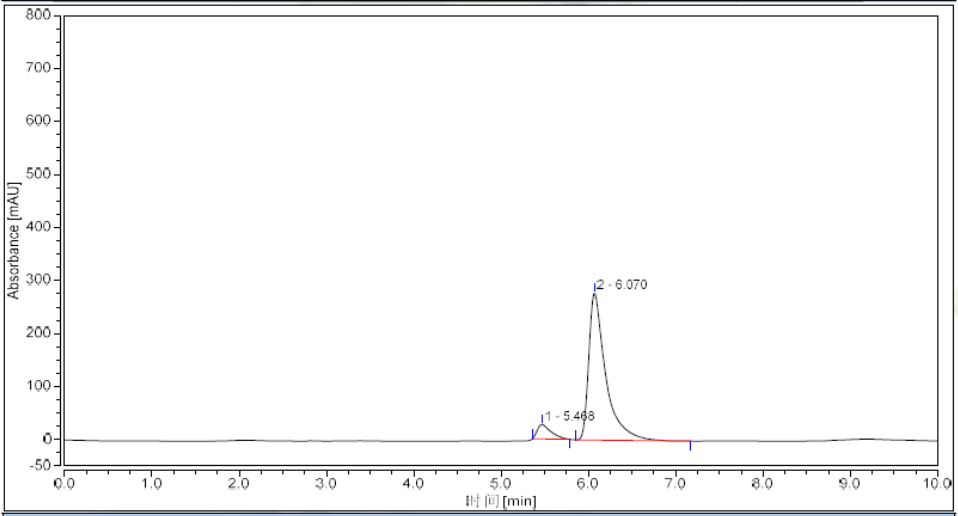

Integration Results

No.	Peak Name	Retention Time min	Area mAU*min	Height mAU	Relative Area %	Relative Height %	Amount n.a.
1		5.468	4.997	27.293	7.45	8.95	n.a.
2		6.070	62.100	277.661	92.55	91.05	n.a.
**Total:**			**67.097**	**304.954**	**100.00**	**100.00**	

4q: ADH, *n*-hexane/2-propanol = 90/10, v = 1.0 mL min^{-1}, λ = 254 nm

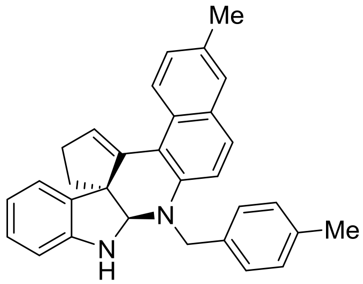

Chromatogram

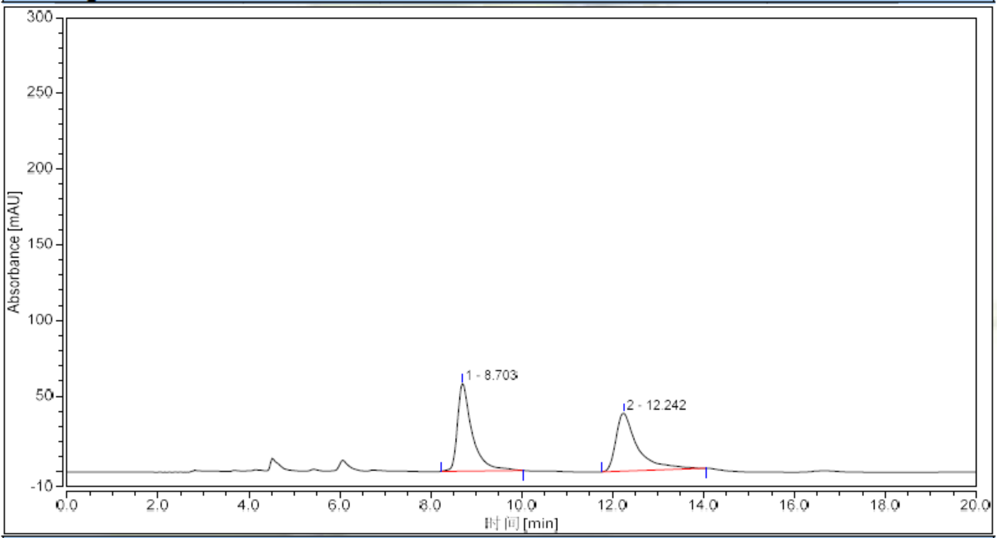

Integration Results

No.	Peak Name	Retention Time min	Area mAU*min	Height mAU	Relative Area %	Relative Height %	Amount n.a.
1		8.703	21.697	57.897	50.71	60.19	n.a.
2		12.242	21.091	38.299	49.29	39.81	n.a.
Total:			**42.788**	**96.196**	**100.00**	**100.00**	

Chromatogram

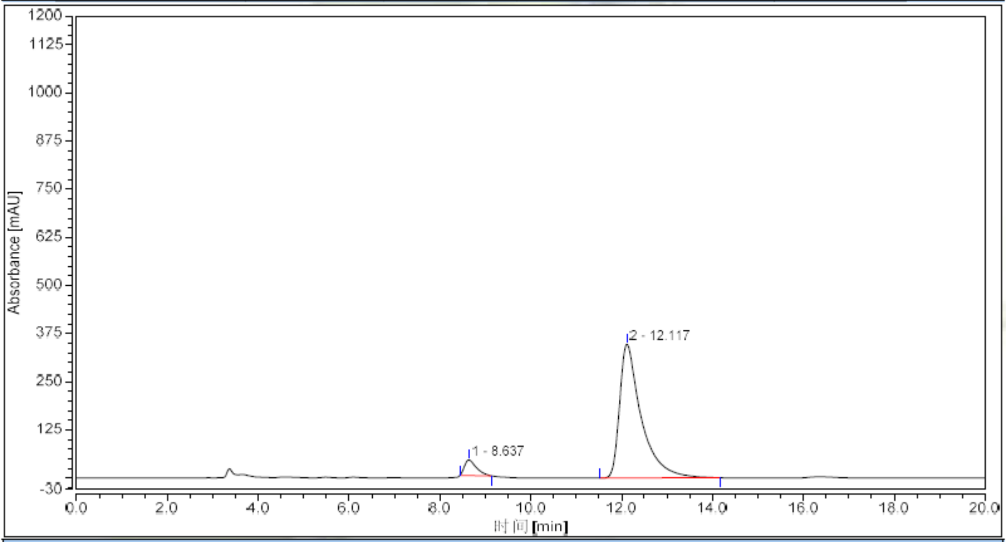

Integration Results

No.	Peak Name	Retention Time min	Area mAU*min	Height mAU	Relative Area %	Relative Height %	Amount n.a.
1		8.637	12.291	40.597	6.02	10.46	n.a.
2		12.117	191.857	347.470	93.98	89.54	n.a.
Total:			**204.147**	**388.067**	**100.00**	**100.00**	

4r: IG, *n*-hexane/2-propanol = 90/10, v = 1.0 mL min^{-1}, λ = 254 nm

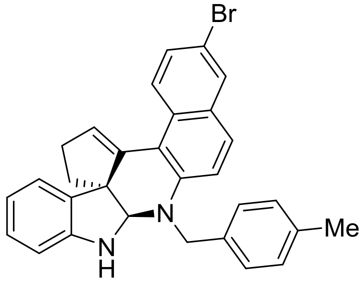

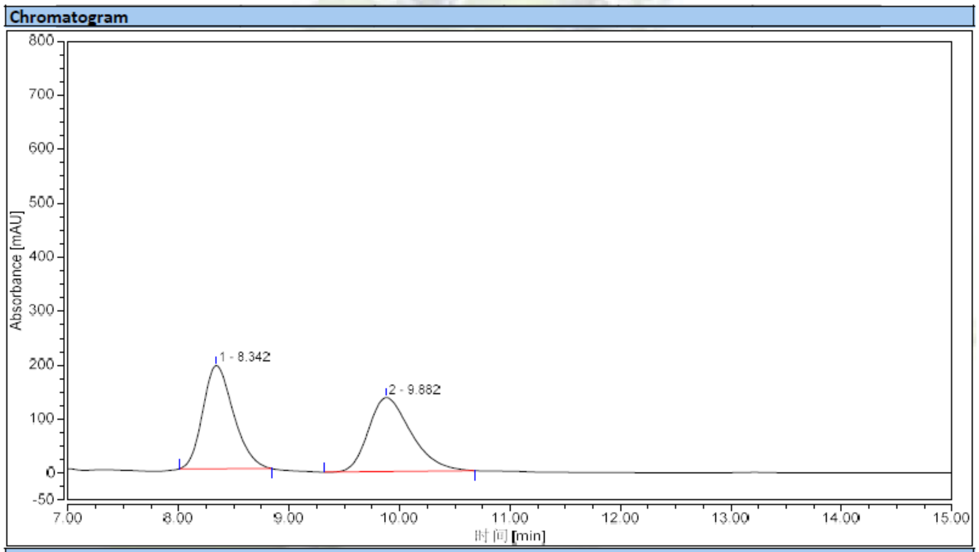

Integration Results

No.	Peak Name	Retention Time min	Area mAU*min	Height mAU	Relative Area %	Relative Height %	Amount n.a.
1		8.342	61.695	190.980	50.24	58.27	n.a.
2		9.882	61.118	136.769	49.76	41.73	n.a.
Total:			122.813	327.749	100.00	100.00	

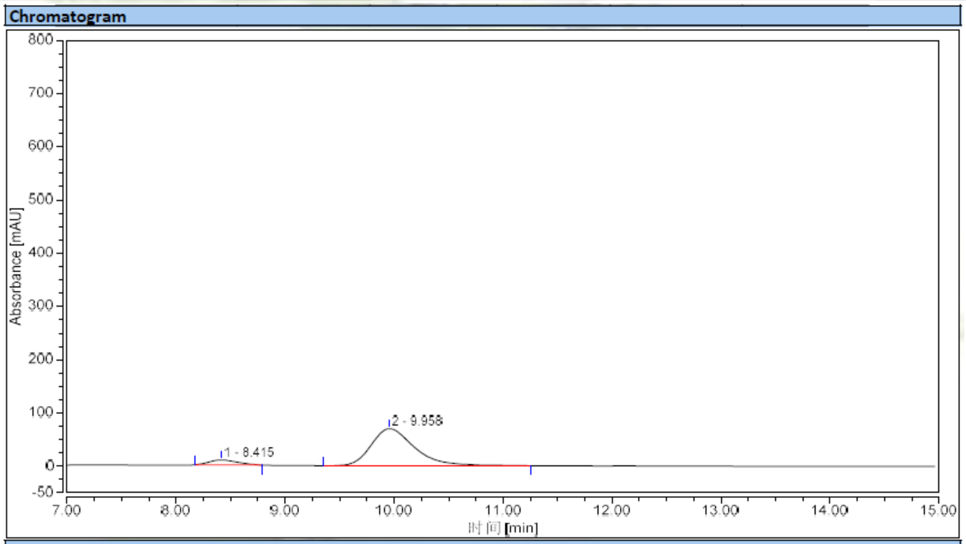

Integration Results

No.	Peak Name	Retention Time min	Area mAU*min	Height mAU	Relative Area %	Relative Height %	Amount n.a.
1		8.415	2.757	9.339	7.95	11.85	n.a.
2		9.958	31.911	69.490	92.05	88.15	n.a.
Total:			34.667	78.830	100.00	100.00	

4b': IG, *n*-hexane/2-propanol = 80/20, v = 1.0 mL min^{-1}, λ = 254 nm

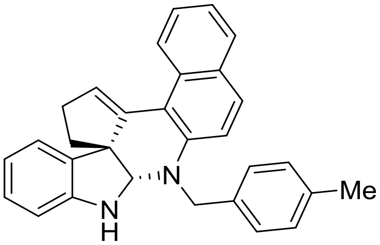

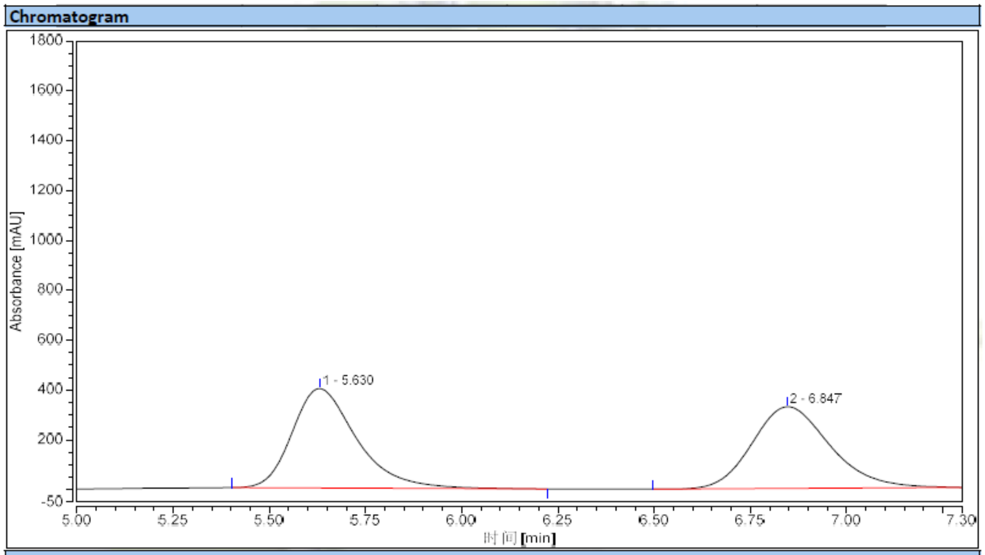

Integration Results

No.	Peak Name	Retention Time min	Area mAU*min	Height mAU	Relative Area %	Relative Height %	Amount n.a.
1		5.630	77.383	398.164	50.22	54.88	n.a.
2		6.847	76.715	327.369	49.78	45.12	n.a.
Total:			154.098	725.533	100.00	100.00	

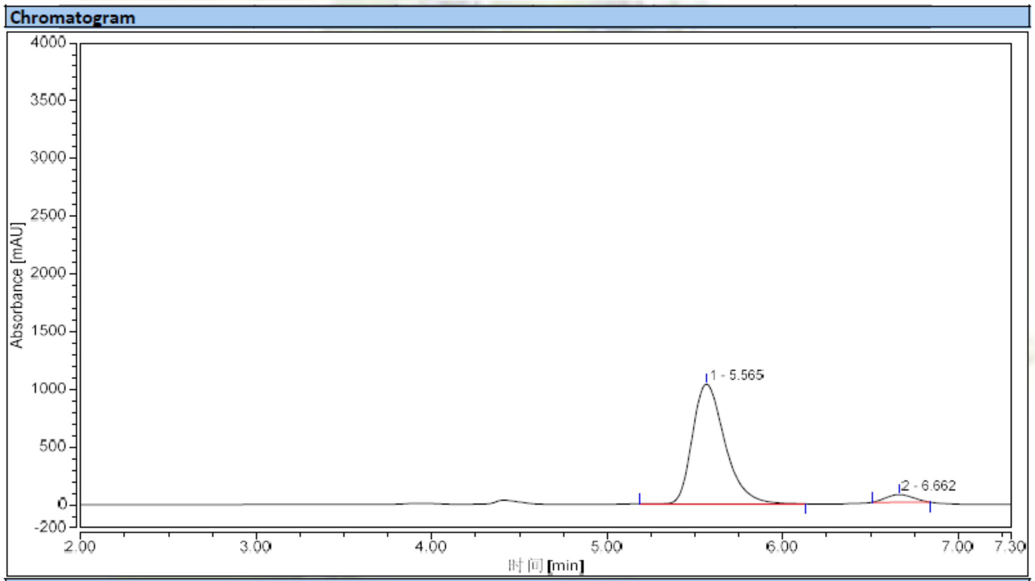

Integration Results

No.	Peak Name	Retention Time min	Area mAU*min	Height mAU	Relative Area %	Relative Height %	Amount n.a.
1		5.565	222.904	1036.783	95.01	94.01	n.a.
2		6.662	11.714	66.004	4.99	5.99	n.a.
Total:			234.618	1102.787	100.00	100.00	

4t: ADH, *n*-hexane/2-propanol = 80/20, v = 1.0 mL min^{-1}, λ = 254 nm

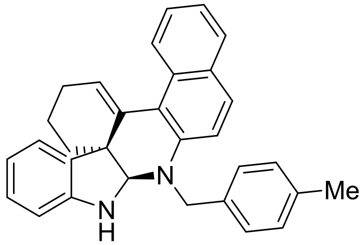

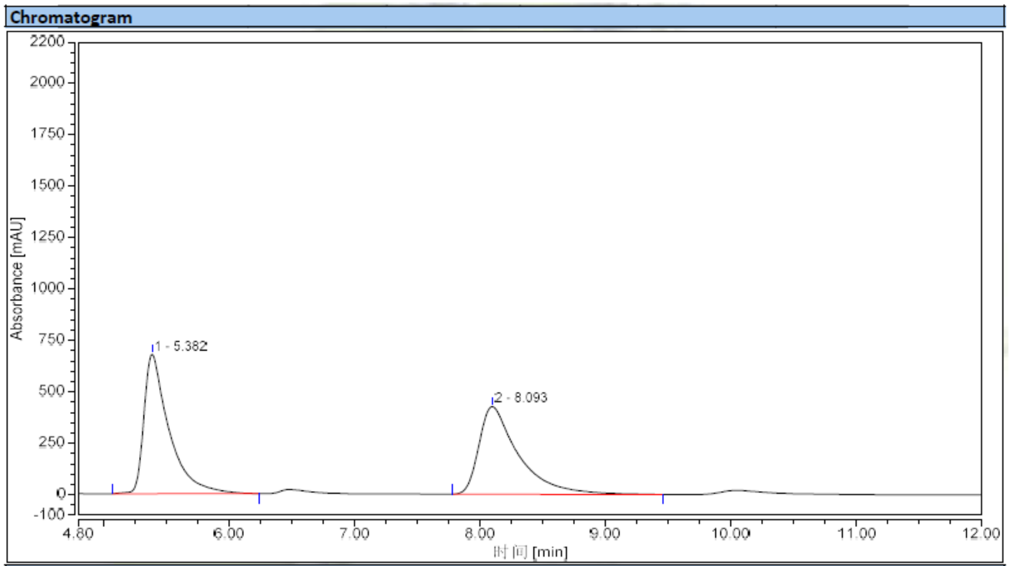

Integration Results

No.	Peak Name	Retention Time min	Area mAU*min	Height mAU	Relative Area %	Relative Height %	Amount n.a.
1		5.382	157.660	678.240	50.31	61.41	n.a.
2		8.093	155.732	426.224	49.69	38.59	n.a.
Total:			313.392	1104.464	100.00	100.00	

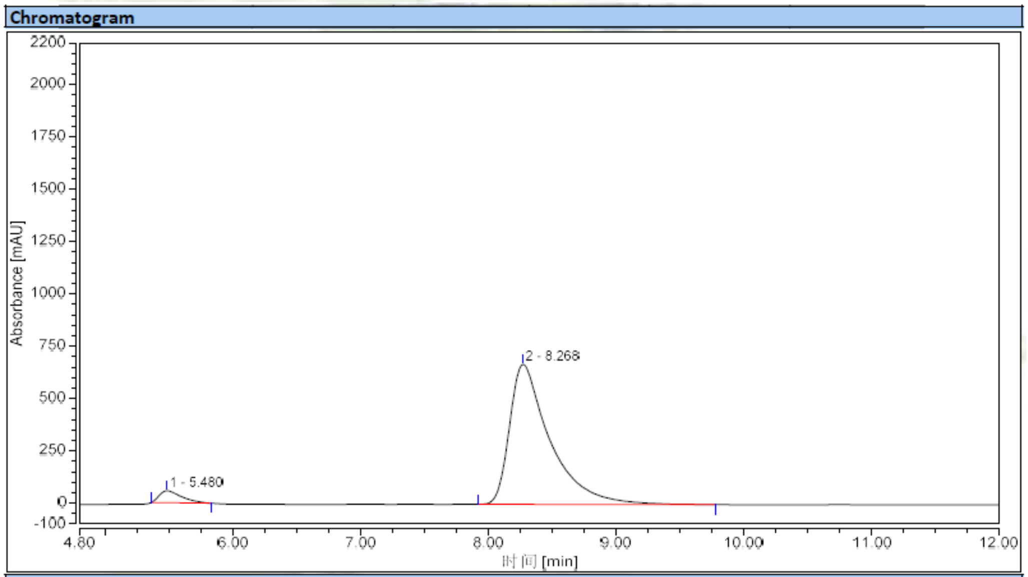

Integration Results

No.	Peak Name	Retention Time min	Area mAU*min	Height mAU	Relative Area %	Relative Height %	Amount n.a.
1		5.480	11.447	56.912	4.44	7.84	n.a.
2		8.268	246.415	669.408	95.56	92.16	n.a.
Total:			257.862	726.320	100.00	100.00	

2aa: ADH, *n*-hexane/2-propanol = 80/20, v = 1.0 mL min^{-1}, λ = 254 nm

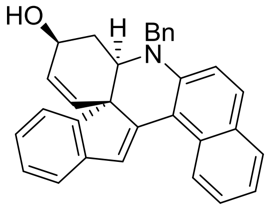

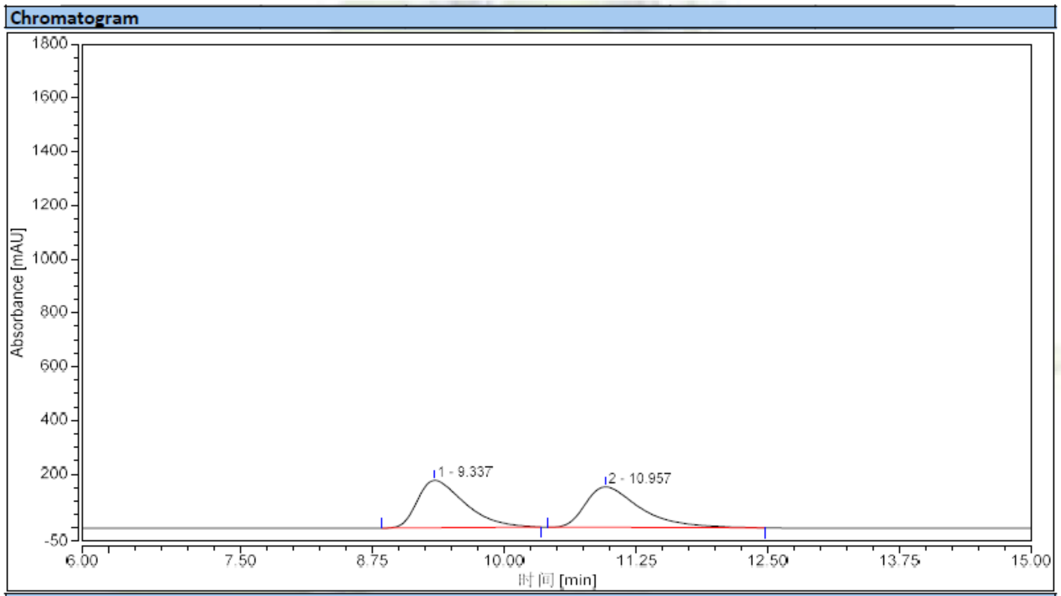

Integration Results

No.	Peak Name	Retention Time min	Area mAU*min	Height mAU	Relative Area %	Relative Height %	Amount n.a.
1		9.337	91.625	176.022	50.30	53.91	n.a.
2		10.957	90.521	150.485	49.70	46.09	n.a.
Total:			182.147	326.507	100.00	100.00	

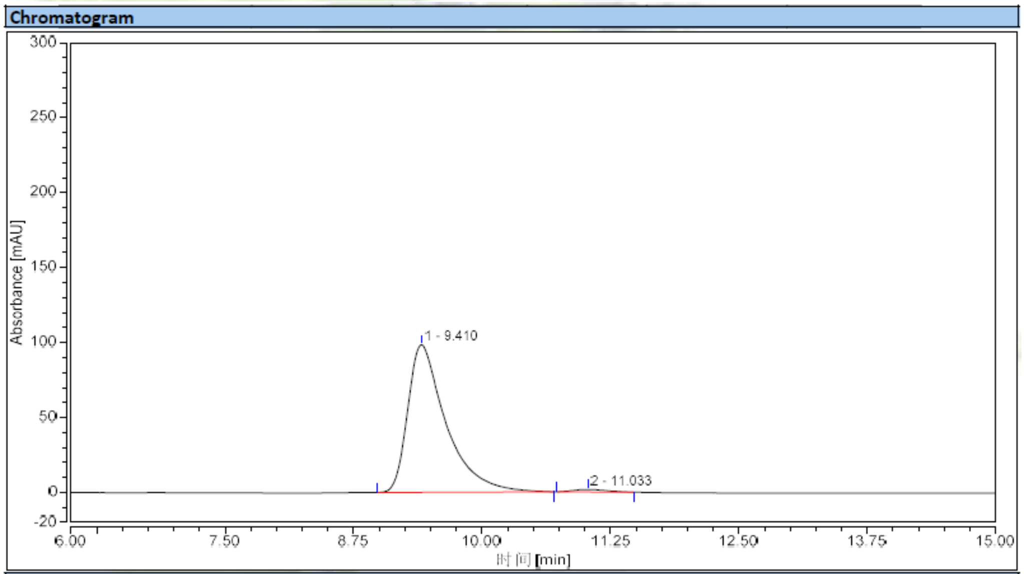

Integration Results

No.	Peak Name	Retention Time min	Area mAU*min	Height mAU	Relative Area %	Relative Height %	Amount n.a.
1		9.410	42.382	98.423	98.60	98.31	n.a.
2		11.033	0.603	1.688	1.40	1.69	n.a.
Total:			42.984	100.111	100.00	100.00	

2ab: IC, *n*-hexane/2-propanol = 80/20, v = 1.0 mL min^{-1}, λ = 254 nm

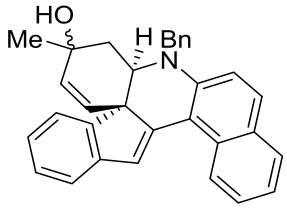

Chromatogram

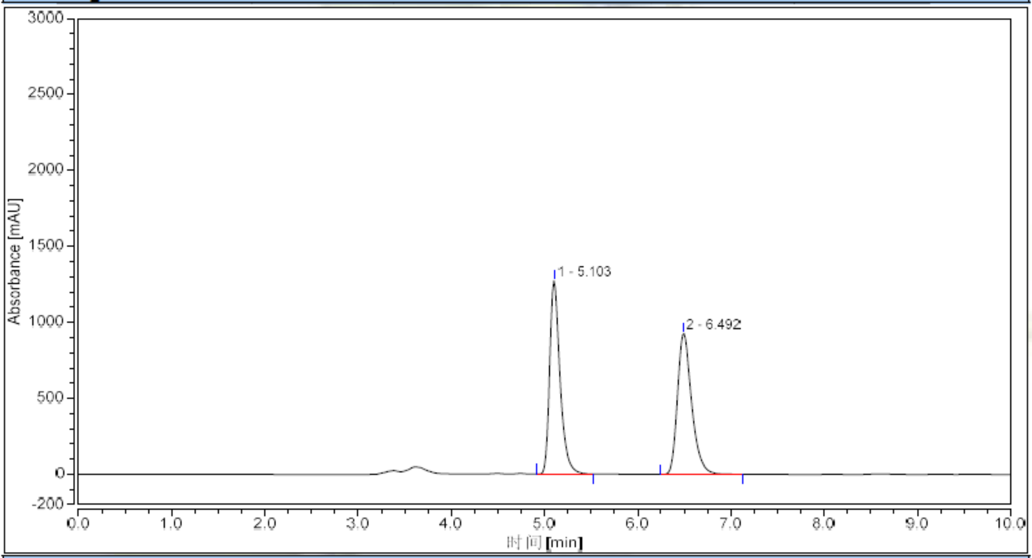

Integration Results

No.	Peak Name	Retention Time min	Area mAU*min	Height mAU	Relative Area %	Relative Height %	Amount n.a.
1		5.103	163.821	1273.300	49.91	57.80	n.a.
2		6.492	164.389	929.657	50.09	42.20	n.a.
Total:			328.211	2202.958	100.00	100.00	

Chromatogram

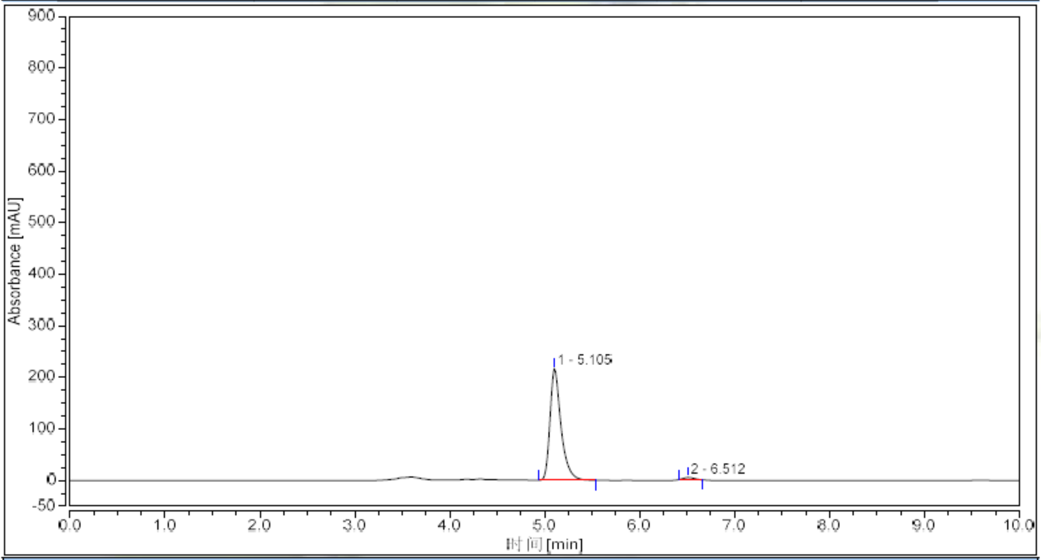

Integration Results

No.	Peak Name	Retention Time min	Area mAU*min	Height mAU	Relative Area %	Relative Height %	Amount n.a.
1		5.105	29.561	216.954	98.18	98.11	n.a.
2		6.512	0.549	4.182	1.82	1.89	n.a.
Total:			30.110	221.135	100.00	100.00	

2ac: IG, *n*-hexane/2-propanol = 80/20, v = 1.0 mL min^{-1}, λ = 254 nm

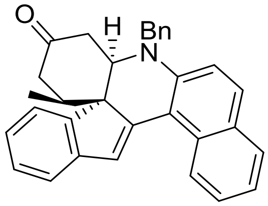

Chromatogram

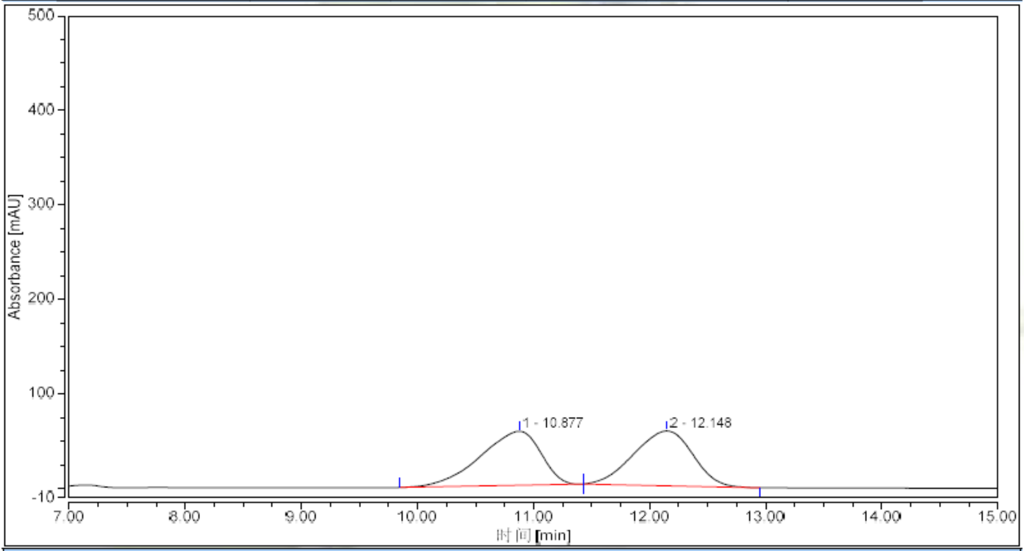

Integration Results

No.	Peak Name	Retention Time<br>min	Area<br>mAU*min	Height<br>mAU	Relative Area<br>%	Relative Height<br>%	Amount<br>n.a.
1		10.877	34.403	57.132	50.23	49.63	n.a.
2		12.148	34.084	57.981	49.77	50.37	n.a.
**Total:**			**68.487**	**115.113**	**100.00**	**100.00**	

Chromatogram

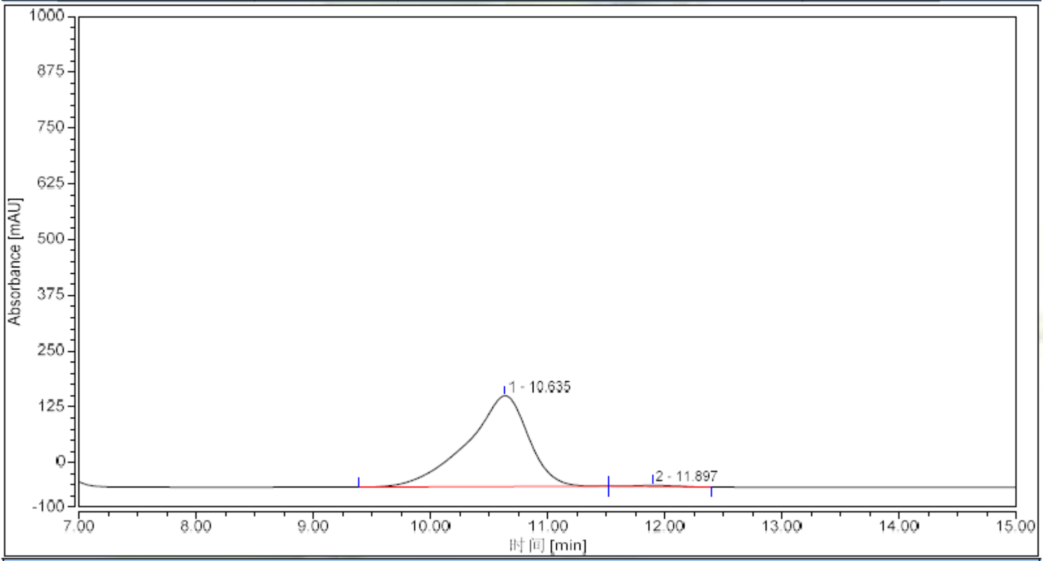

Integration Results

No.	Peak Name	Retention Time<br>min	Area<br>mAU*min	Height<br>mAU	Relative Area<br>%	Relative Height<br>%	Amount<br>n.a.
1		10.635	122.684	203.420	99.12	98.77	n.a.
2		11.897	1.086	2.536	0.88	1.23	n.a.
**Total:**			**123.770**	**205.956**	**100.00**	**100.00**	

2ad: ADH, *n*-hexane/2-propanol = 90/10, v = 1.0 mL min^{-1}, λ = 254 nm

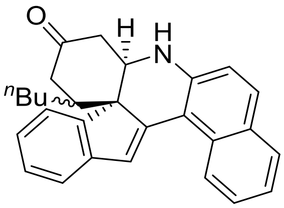

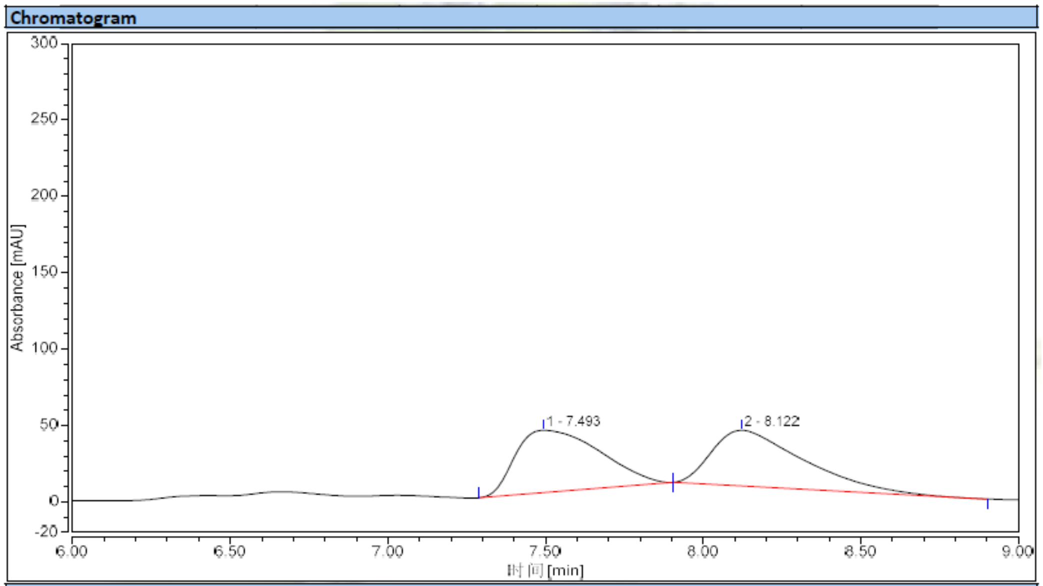

Integration Results

No.	Peak Name	Retention Time min	Area mAU*min	Height mAU	Relative Area %	Relative Height %	Amount n.a.
1		7.493	12.533	40.799	50.38	52.82	n.a.
2		8.122	12.345	36.436	49.62	47.18	n.a.
Total:			24.879	77.235	100.00	100.00	

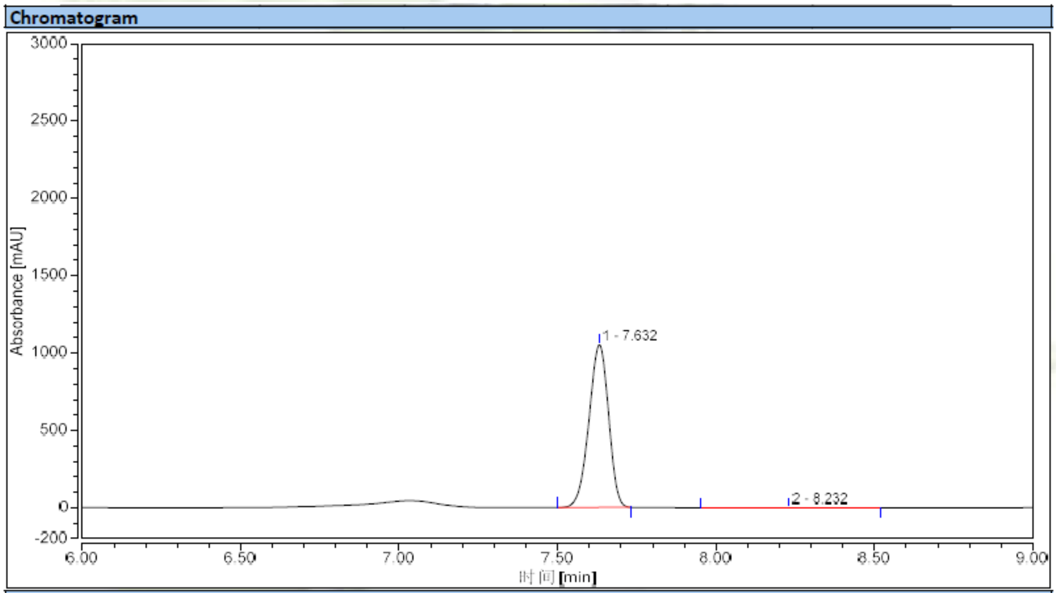

Integration Results

No.	Peak Name	Retention Time min	Area mAU*min	Height mAU	Relative Area %	Relative Height %	Amount n.a.
1		7.632	75.370	1052.994	99.95	99.98	n.a.
2		8.232	0.039	0.162	0.05	0.02	n.a.
Total:			75.409	1053.157	100.00	100.00	

4ba: ADH, *n*-hexane/2-propanol = 80/20, v = 1.0 mL min^{-1}, λ = 254 nm

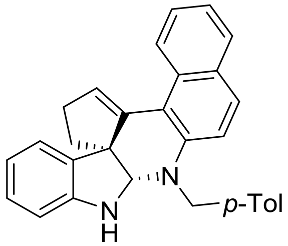

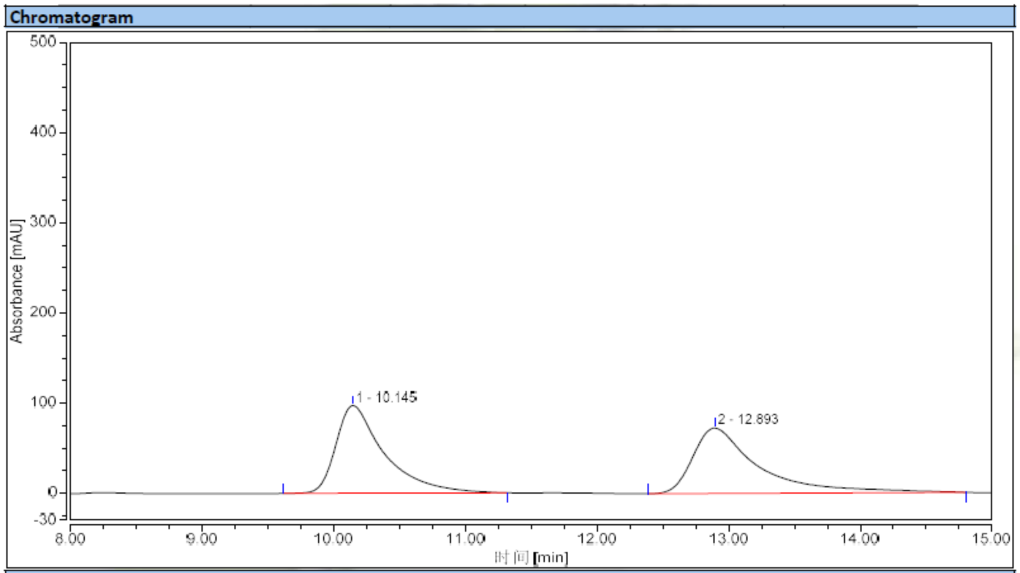

Integration Results

No.	Peak Name	Retention Time min	Area mAU*min	Height mAU	Relative Area %	Relative Height %	Amount n.a.
1		10.145	41.333	97.191	49.76	57.30	n.a.
2		12.893	41.738	72.438	50.24	42.70	n.a.
Total:			**83.070**	**169.629**	**100.00**	**100.00**	

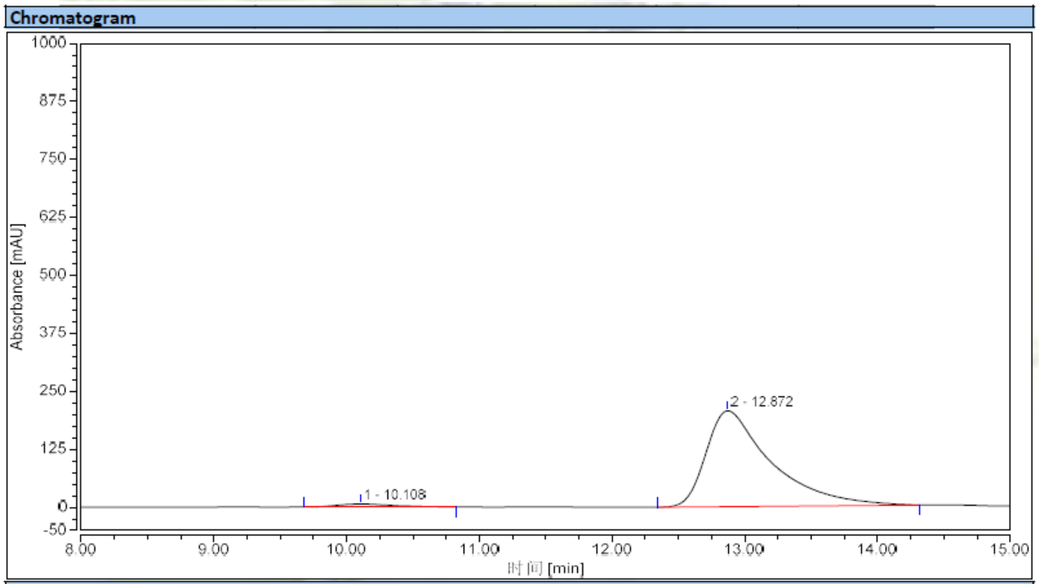

Integration Results

No.	Peak Name	Retention Time min	Area mAU*min	Height mAU	Relative Area %	Relative Height %	Amount n.a.
1		10.108	3.089	6.607	2.57	3.10	n.a.
2		12.872	117.054	206.624	97.43	96.90	n.a.
Total:			**120.143**	**213.230**	**100.00**	**100.00**	

4bb: IG, *n*-hexane/2-propanol = 80/20, v = 1.0 mL min^{-1}, λ = 254 nm

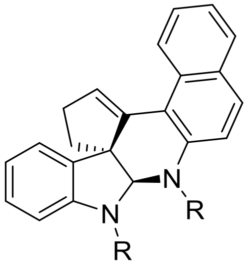

R = $COOCH_2CH_2Cl$

Chromatogram

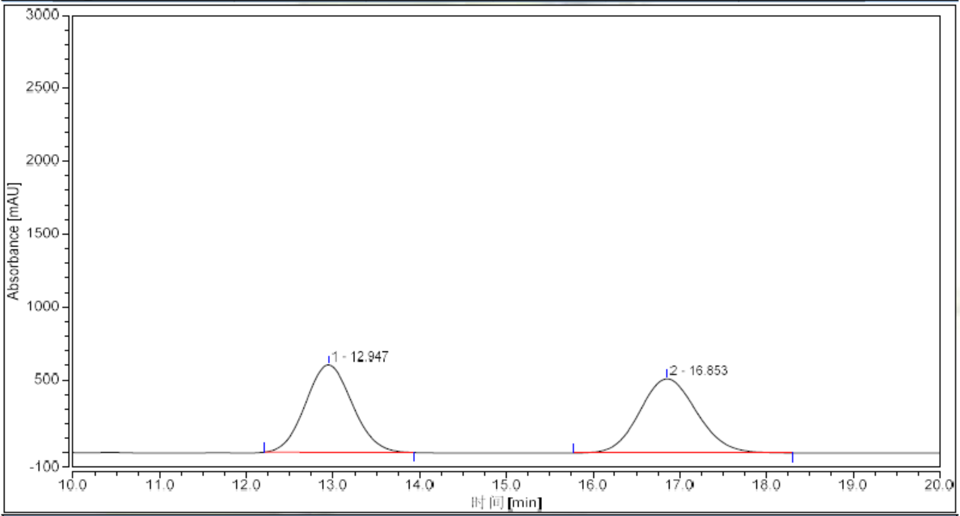

Integration Results

No.	Peak Name	Retention Time<br>min	Area<br>mAU*min	Height<br>mAU	Relative Area<br>%	Relative Height<br>%	Amount<br>n.a.
1		12.947	375.549	599.079	49.21	54.15	n.a.
2		16.853	387.556	507.183	50.79	45.85	n.a.
Total:			763.105	1106.262	100.00	100.00	

Chromatogram

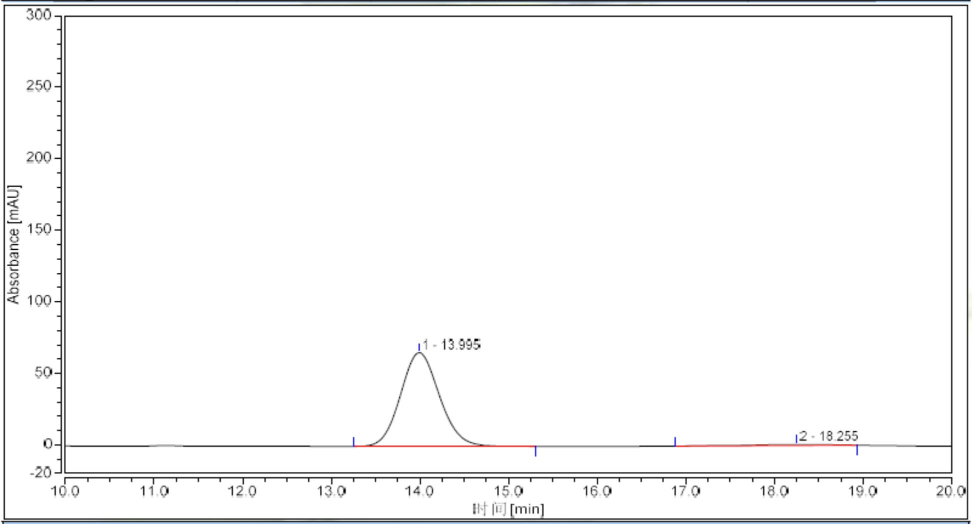

Integration Results

No.	Peak Name	Retention Time<br>min	Area<br>mAU*min	Height<br>mAU	Relative Area<br>%	Relative Height<br>%	Amount<br>n.a.
1		13.995	33.478	65.515	98.00	98.98	n.a.
2		18.255	0.682	0.675	2.00	1.02	n.a.
Total:			34.160	66.190	100.00	100.00	

10. ^{1}H and ^{13}C NMR Spectra

Parameter	Value
1 Title	ttd-24-14
2 Origin	Bruker BioSpin GmbH
3 Solvent	CDCl3
4 Temperature	298.1
5 Number of Scans	13
6 Acquisition Time	3.1719
7 Acquisition Date	2021-07-09T13:24:23
8 Spectrometer Frequency	500.17
9 Spectral Width	10330.6

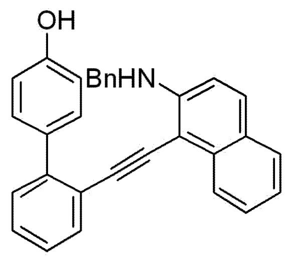

1a

^{1}H NMR of compound 1a

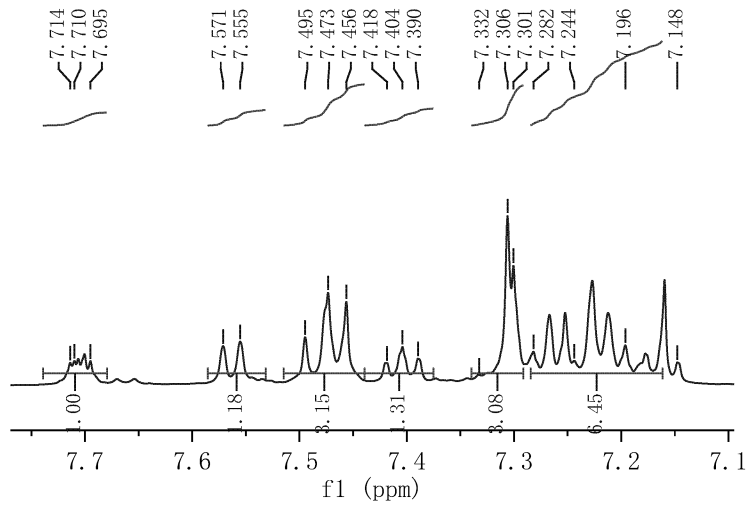

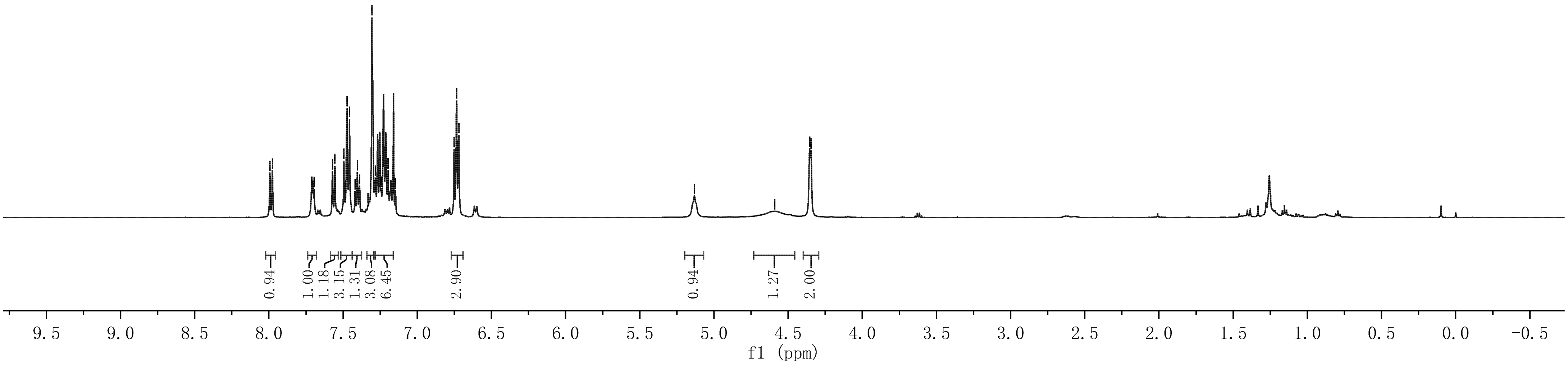

Parameter	Value
1 Title	ttd-24-14-C
2 Origin	Bruker BioSpin GmbH
3 Solvent	CDCl3
4 Temperature	298.5
5 Number of Scans	18
6 Acquisition Time	1.1010
7 Acquisition Date	2021-07-09T13:27:40
8 Spectrometer Frequency	125.77
9 Spectral Width	29761.9

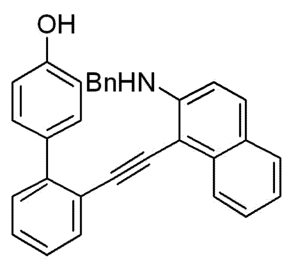

1a

^{13}C NMR of compound 1a

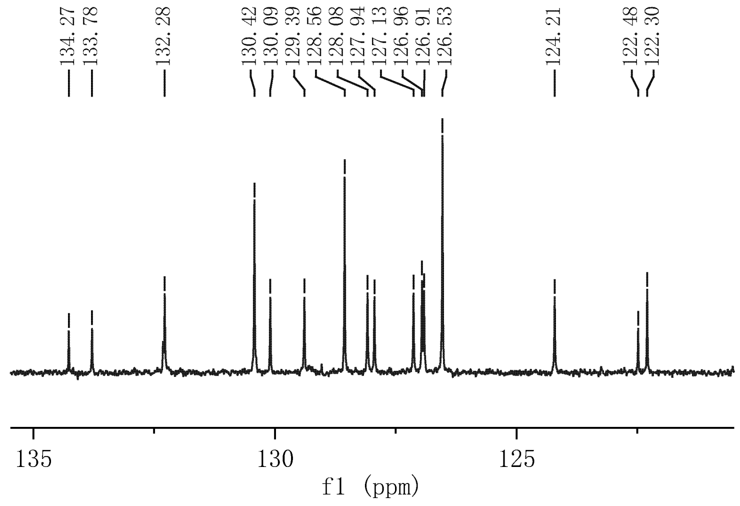

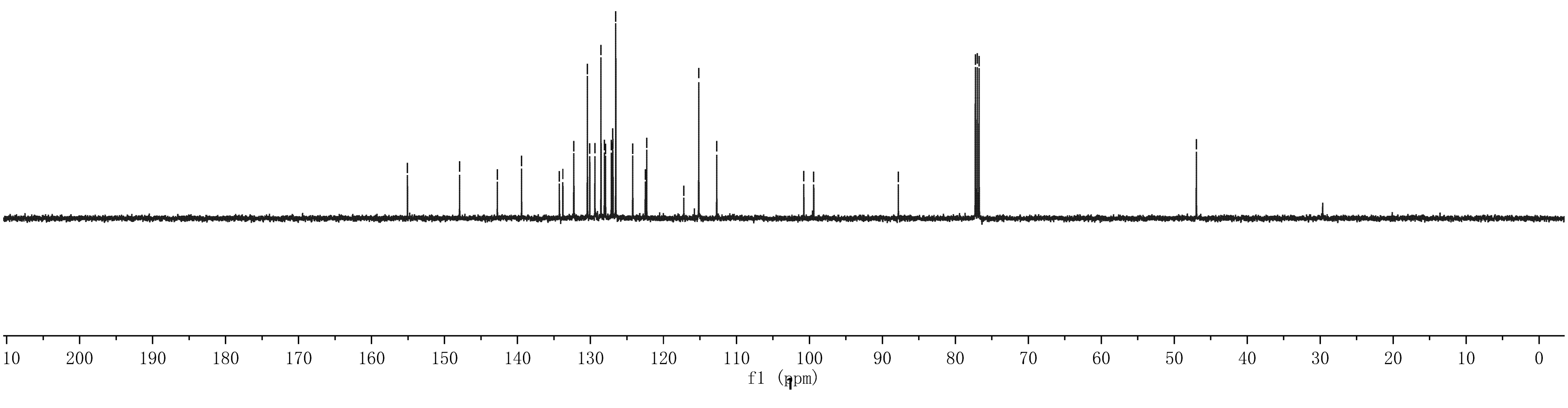

Parameter	Value
1 Title	ttd-23-191
2 Origin	Bruker BioSpin GmbH
3 Solvent	CDCl3
4 Temperature	298.0
5 Number of Scans	8
6 Acquisition Time	4.0894
7 Acquisition Date	2021-06-04T08:24:26
8 Spectrometer Frequency	400.13
9 Spectral Width	8012.8

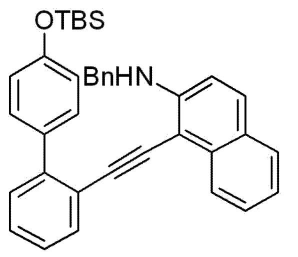

1aa

^{1}H NMR of compound 1aa

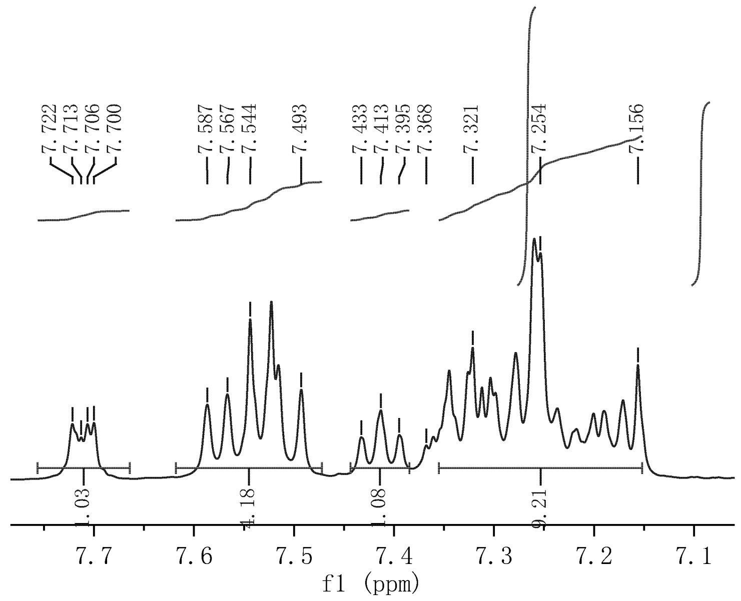

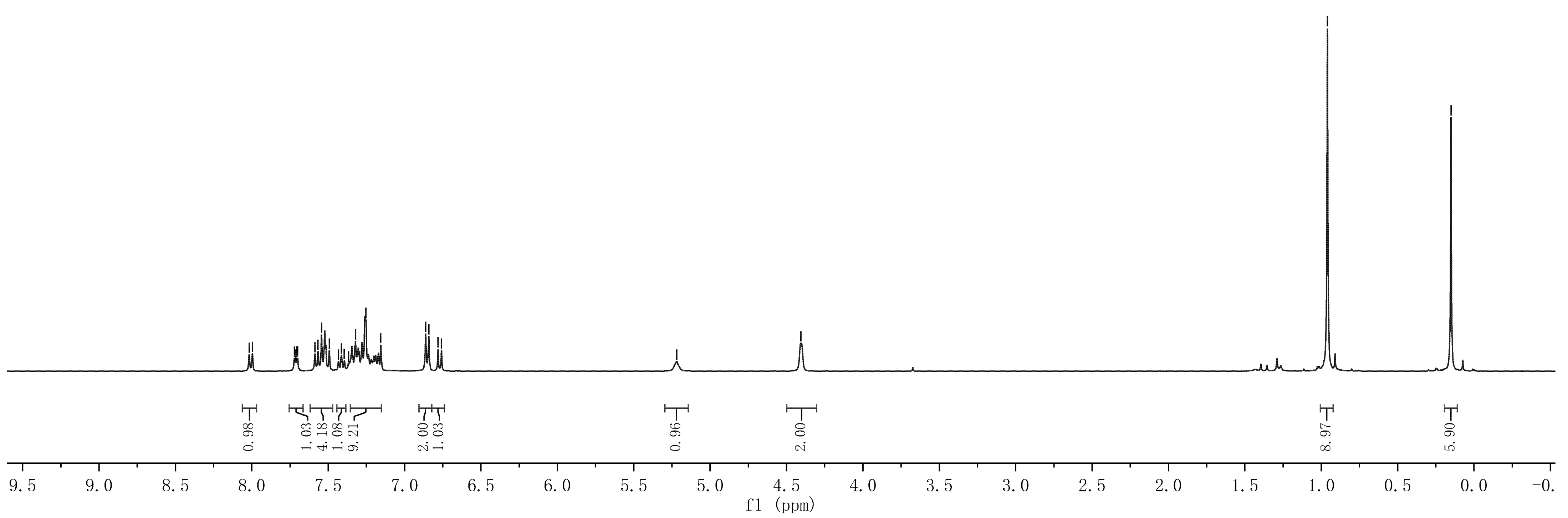

Parameter	Value
1 Title	ttd-23-191-C
2 Origin	Bruker BioSpin GmbH
3 Solvent	CDCl3
4 Temperature	300.0
5 Number of Scans	24
6 Acquisition Time	1.3631
7 Acquisition Date	2021-06-04T08:26:34
8 Spectrometer Frequency	100.61
9 Spectral Width	24038.5

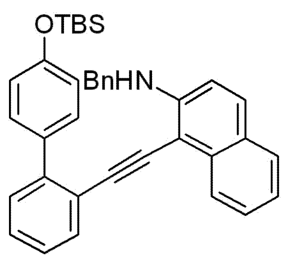

1aa

^{13}C NMR of compound 1aa

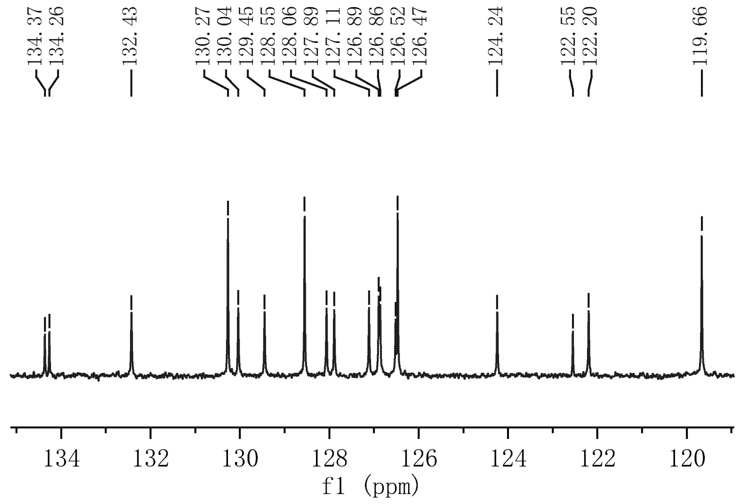

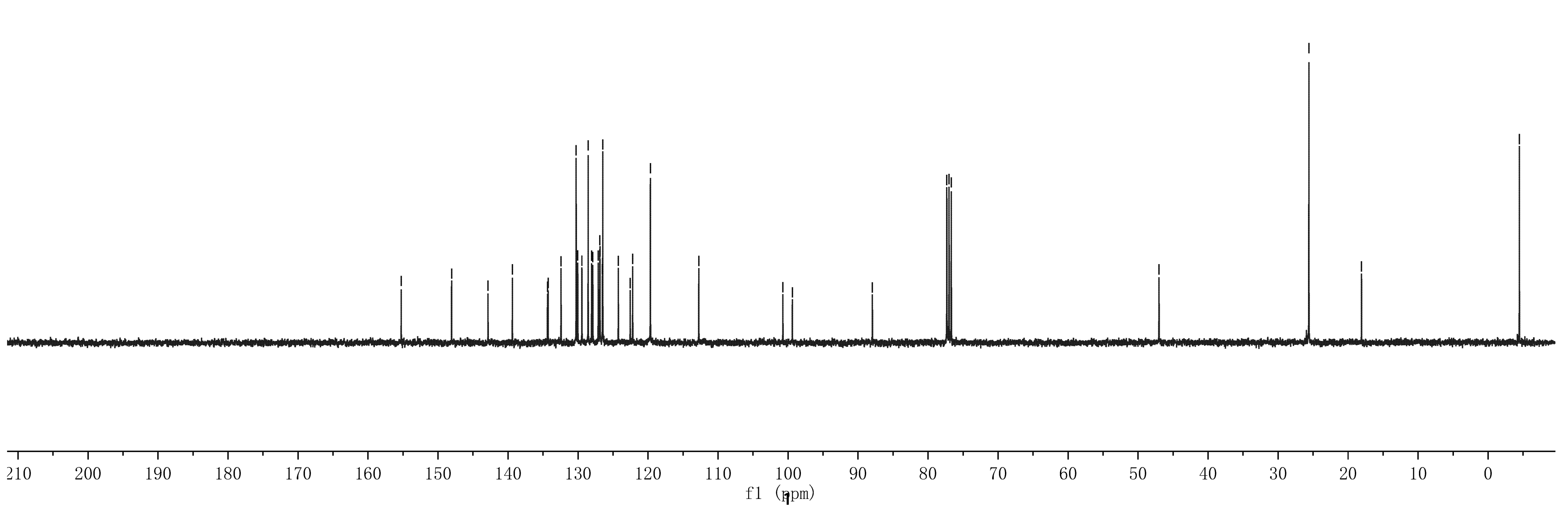

Parameter	Value
1 Title	ttd-21-86
2 Origin	Bruker BioSpin GmbH
3 Solvent	CDCl3
4 Temperature	298.0
5 Number of Scans	7
6 Acquisition Time	4.0894
7 Acquisition Date	2020-09-16T17:18:52
8 Spectrometer Frequency	400.13
9 Spectral Width	8012.8

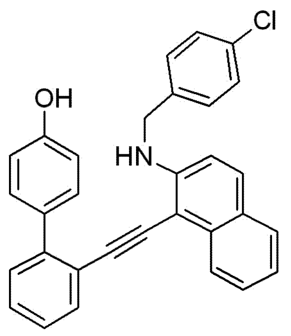

1b

^1H NMR of compound 1b

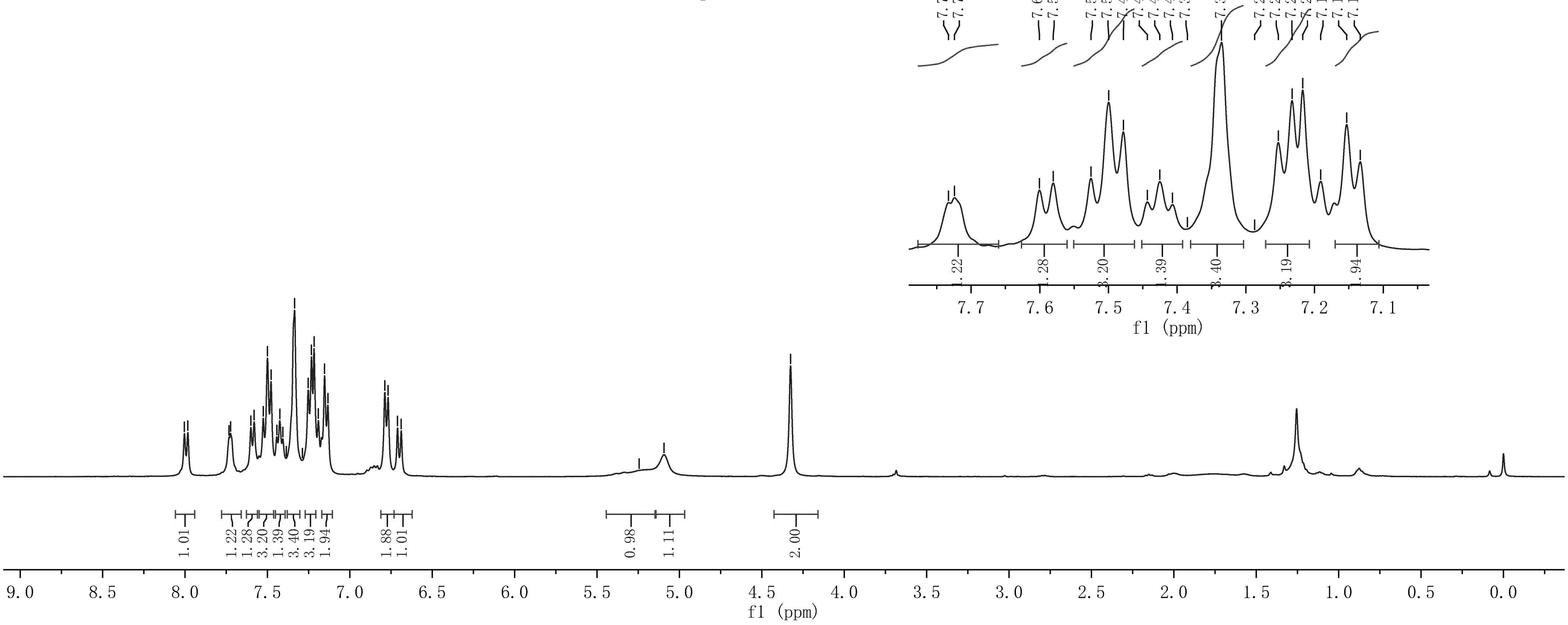

Parameter	Value
1 Title	ttd-21-86-C
2 Origin	Bruker BioSpin GmbH
3 Solvent	CDCl3
4 Temperature	300.0
5 Number of Scans	27
6 Acquisition Time	1.3631
7 Acquisition Date	2020-09-16T17:20:39
8 Spectrometer Frequency	100.61
9 Spectral Width	24038.5

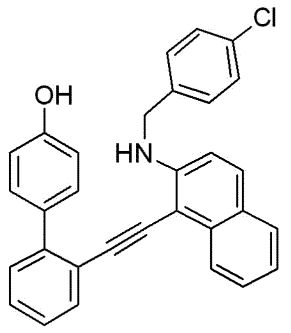

1b

^{13}C NMR of compound 1b

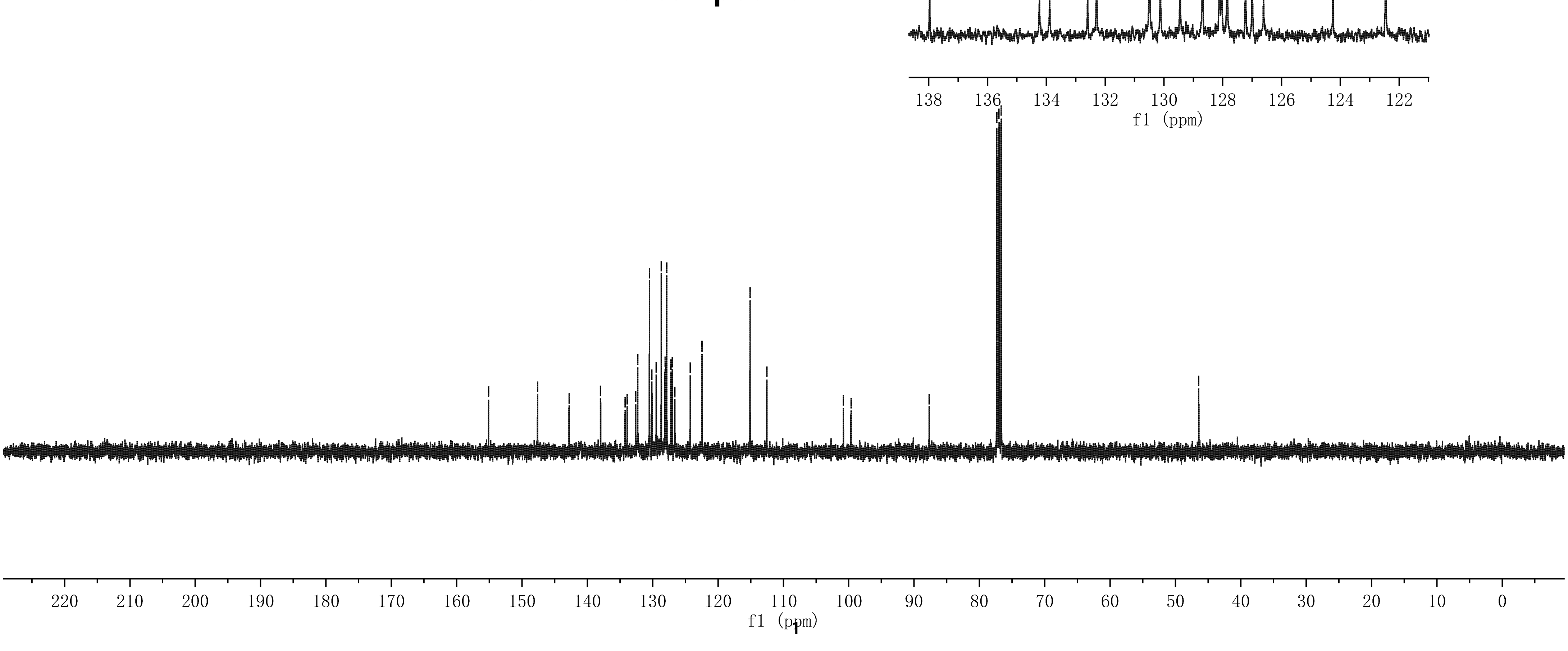

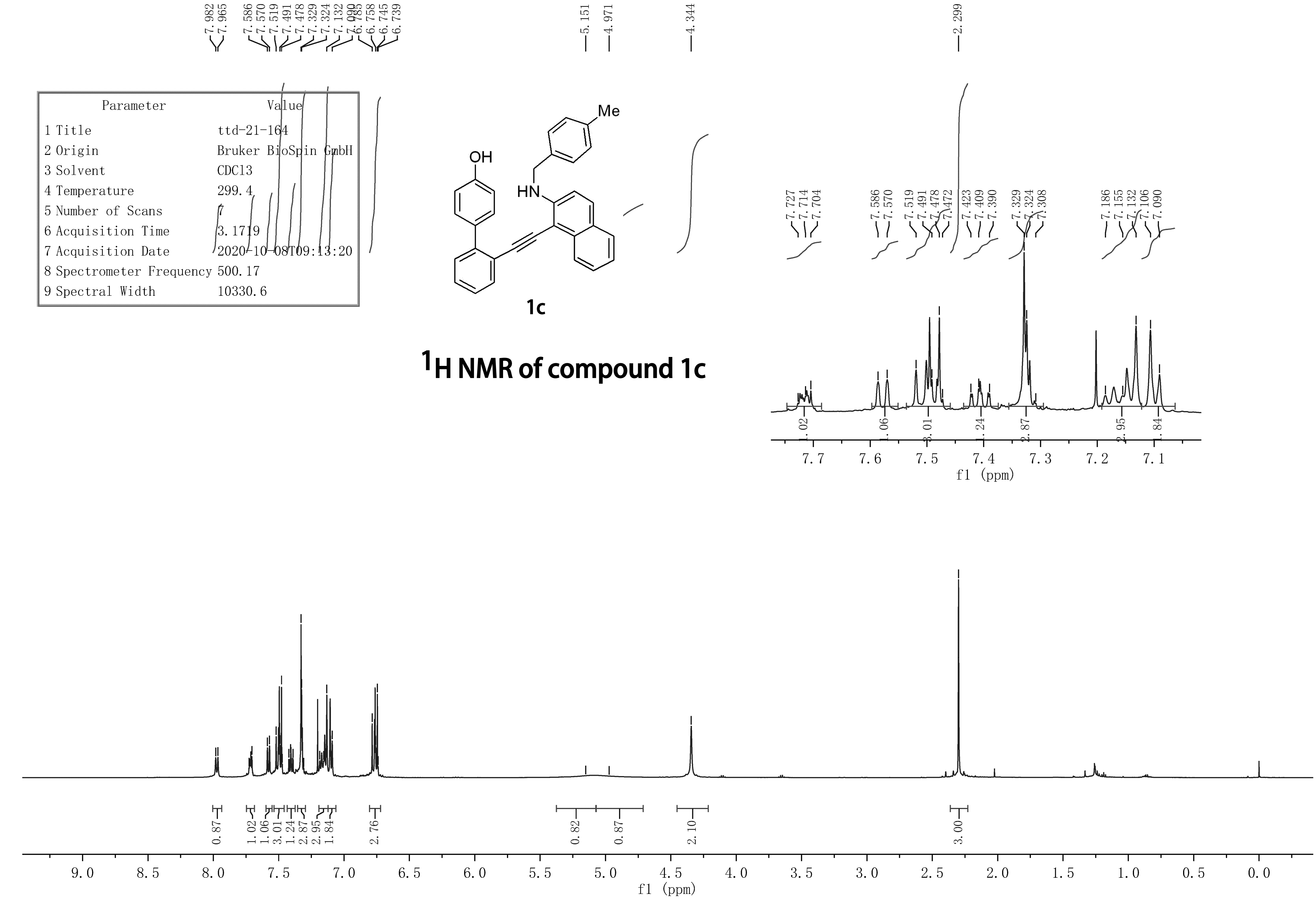

^{1}H NMR of compound 1c

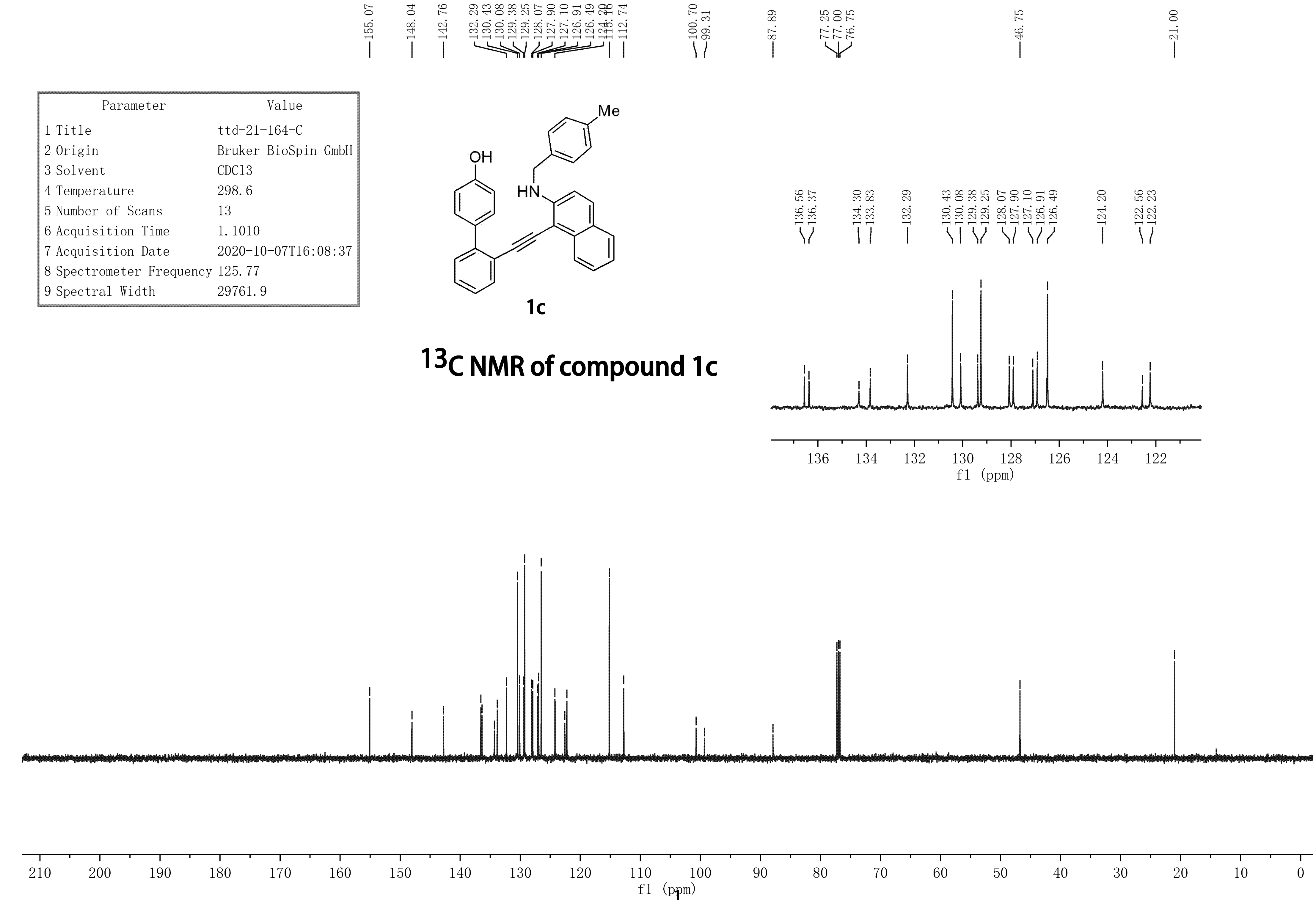

^{13}C NMR of compound 1c

Parameter	Value
1 Title	shz-1-69
2 Origin	Bruker BioSpin GmbH
3 Solvent	CDCl3
4 Temperature	298.2
5 Number of Scans	10
6 Acquisition Time	3.1719
7 Acquisition Date	2021-01-06T16:04:34
8 Spectrometer Frequency	500.17
9 Spectral Width	10330.6

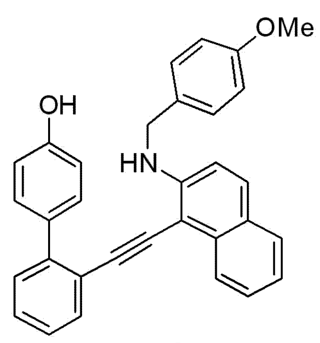

1d

1H NMR of compound 1d

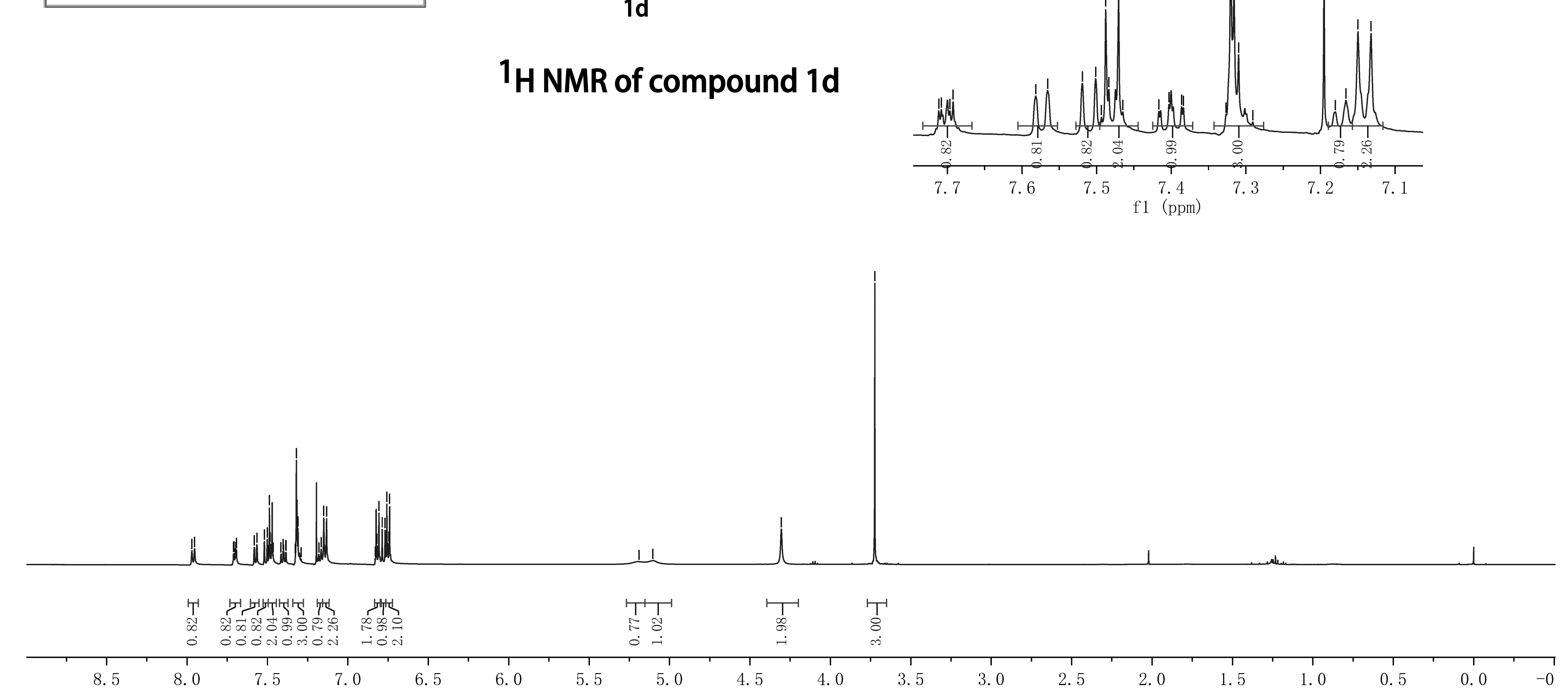

Parameter	Value
1 Title	shz-1-69-C
2 Origin	Bruker BioSpin GmbH
3 Solvent	CDCl3
4 Temperature	299.0
5 Number of Scans	38
6 Acquisition Time	1.1010
7 Acquisition Date	2021-01-06T16:08:27
8 Spectrometer Frequency	125.78
9 Spectral Width	29761.9

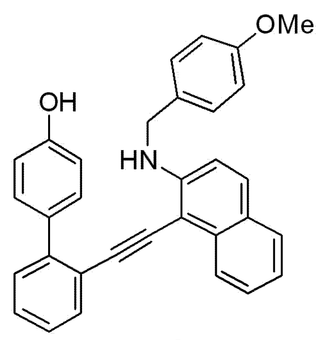

1d

13C NMR of compound 1d

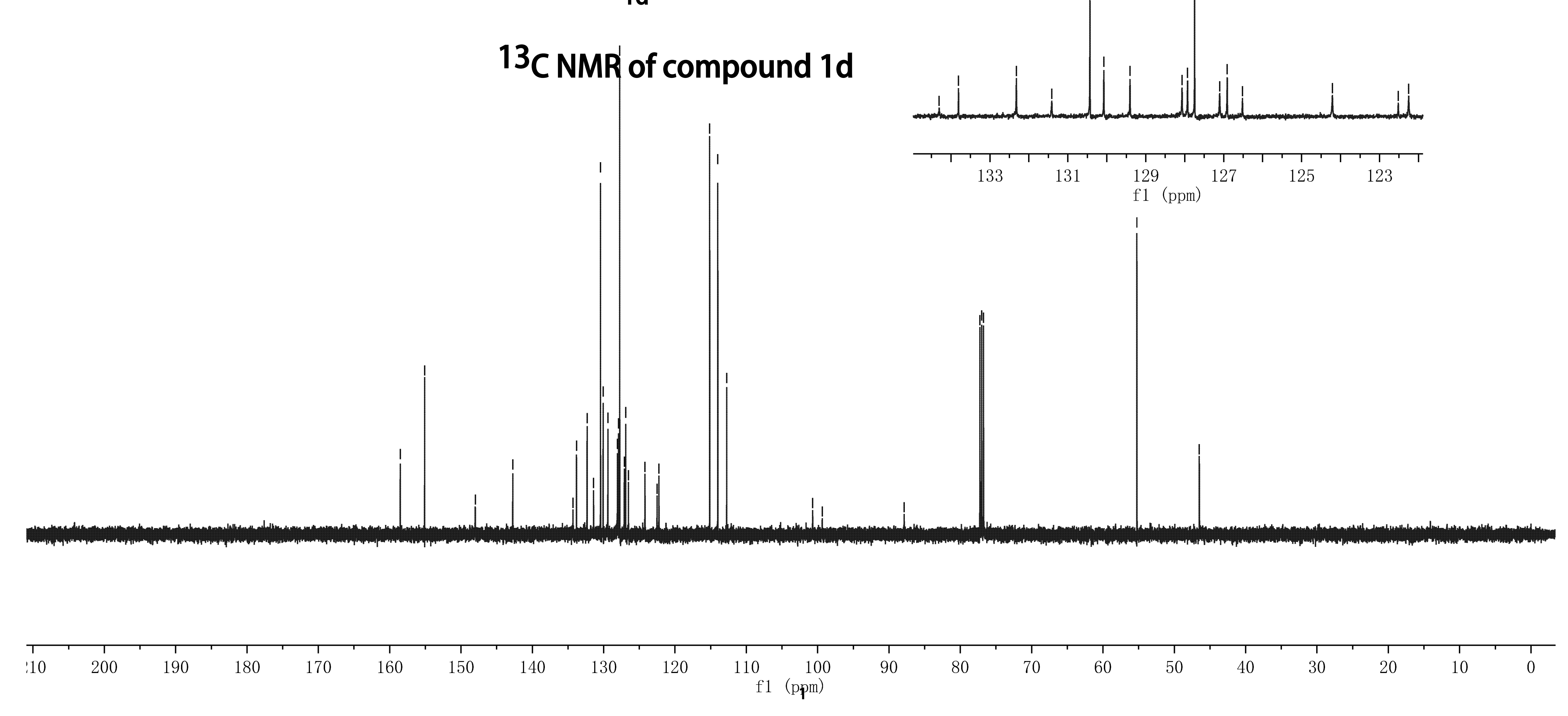

Parameter	Value
1 Title	shz-1-132-final
2 Origin	Bruker BioSpin GmbH
3 Solvent	CDCl3
4 Temperature	299.2
5 Number of Scans	5
6 Acquisition Time	3.1719
7 Acquisition Date	2020-10-26T17:11:51
8 Spectrometer Frequency	500.17
9 Spectral Width	10330.6

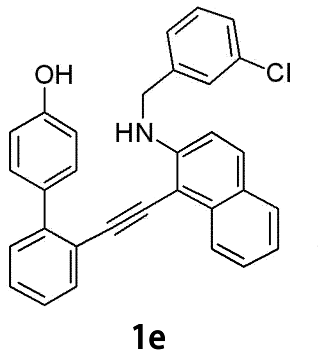

1e

^{1}H NMR of compound 1e

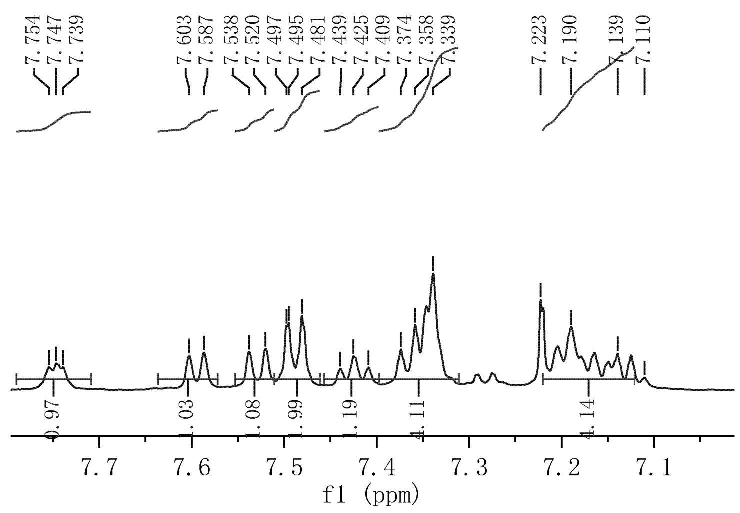

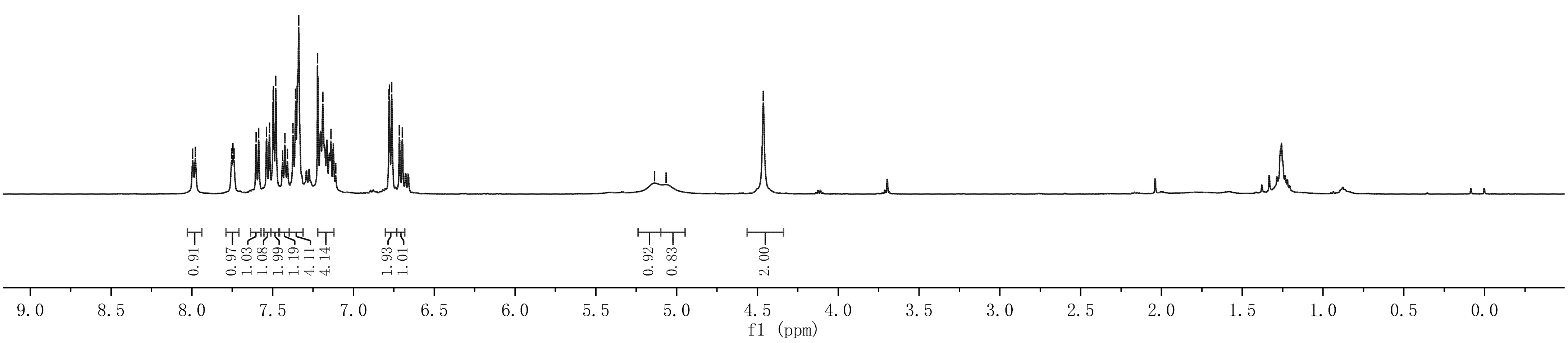

Parameter	Value
1 Title	shz-1-132-final-C
2 Origin	Bruker BioSpin GmbH
3 Solvent	CDCl3
4 Temperature	299.5
5 Number of Scans	60
6 Acquisition Time	1.1010
7 Acquisition Date	2020-10-26T17:12:56
8 Spectrometer Frequency	125.77
9 Spectral Width	29761.9

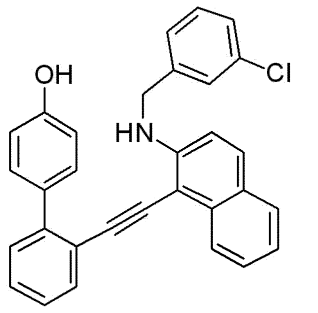

1e

^{13}C NMR of compound 1e

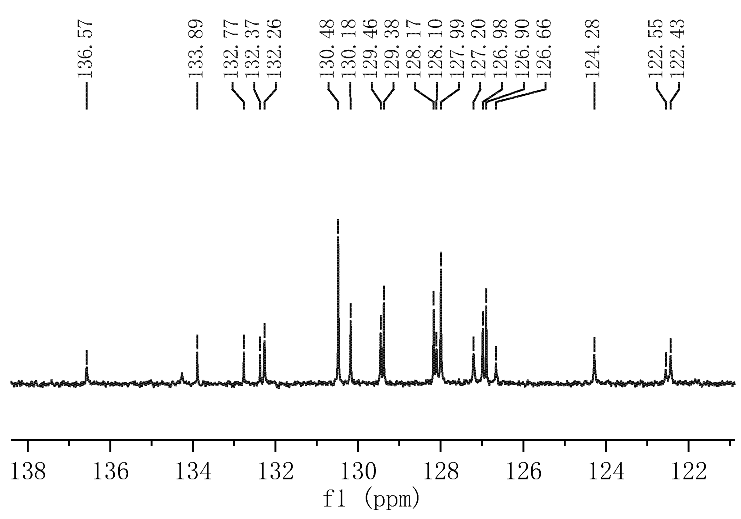

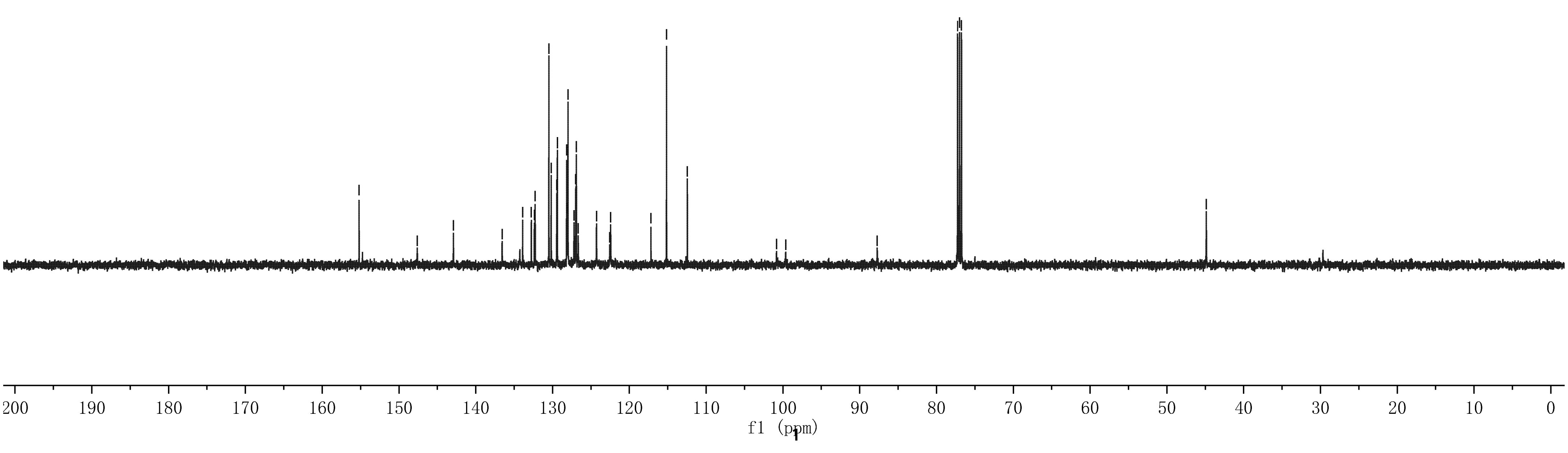

Parameter	Value
1 Title	ttd-21-123-400
2 Origin	Bruker BioSpin GmbH
3 Solvent	CDCl3
4 Temperature	298.0
5 Number of Scans	7
6 Acquisition Time	4.0894
7 Acquisition Date	2020-09-28T15:24:15
8 Spectrometer Frequency	400.13
9 Spectral Width	8012.8

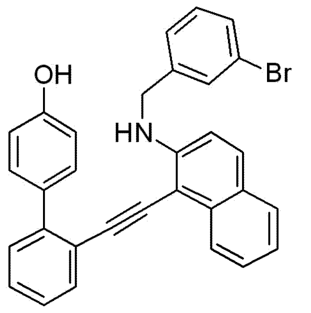

1f

^{1}H NMR of compound 1f

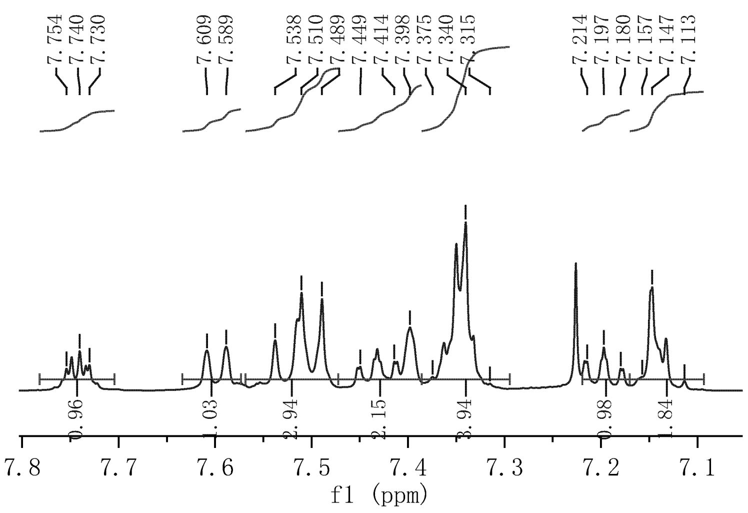

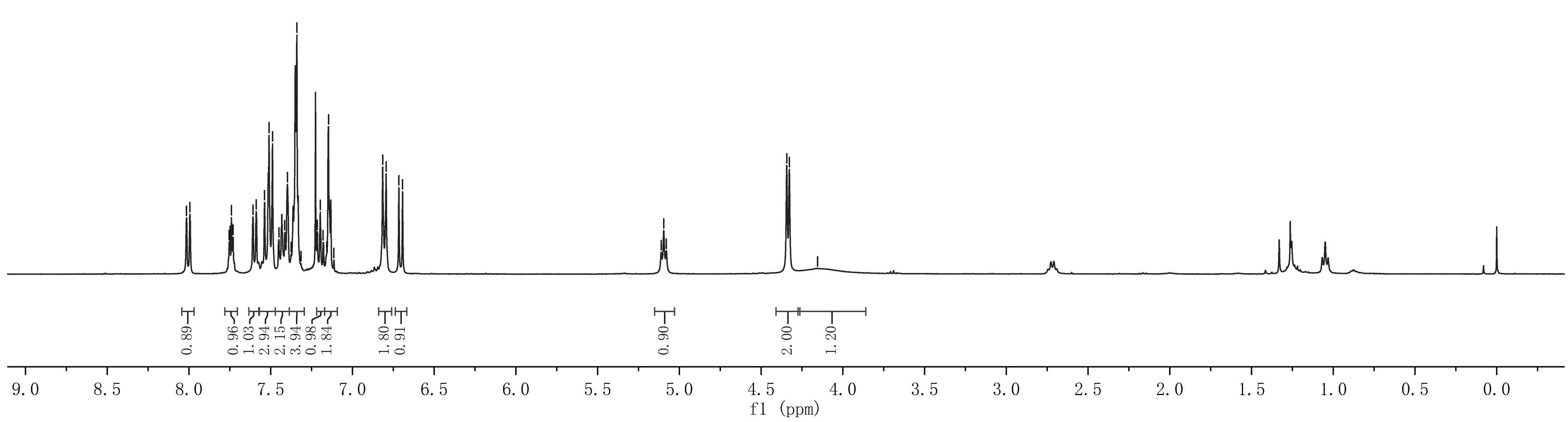

Parameter	Value
1 Title	ttd-21-123-C
2 Origin	Bruker BioSpin GmbH
3 Solvent	CDCl3
4 Temperature	298.4
5 Number of Scans	9
6 Acquisition Time	1.1010
7 Acquisition Date	2020-09-26T15:57:05
8 Spectrometer Frequency	125.77
9 Spectral Width	29761.9

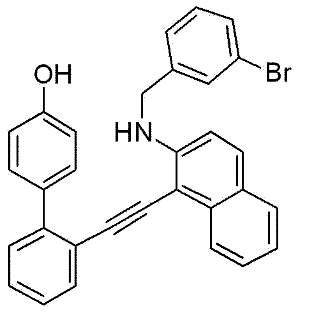

1f

^{13}C NMR of compound 1f

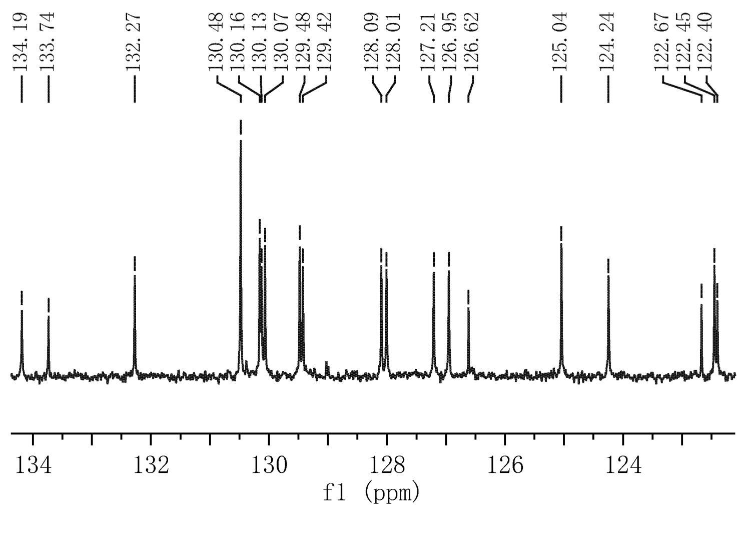

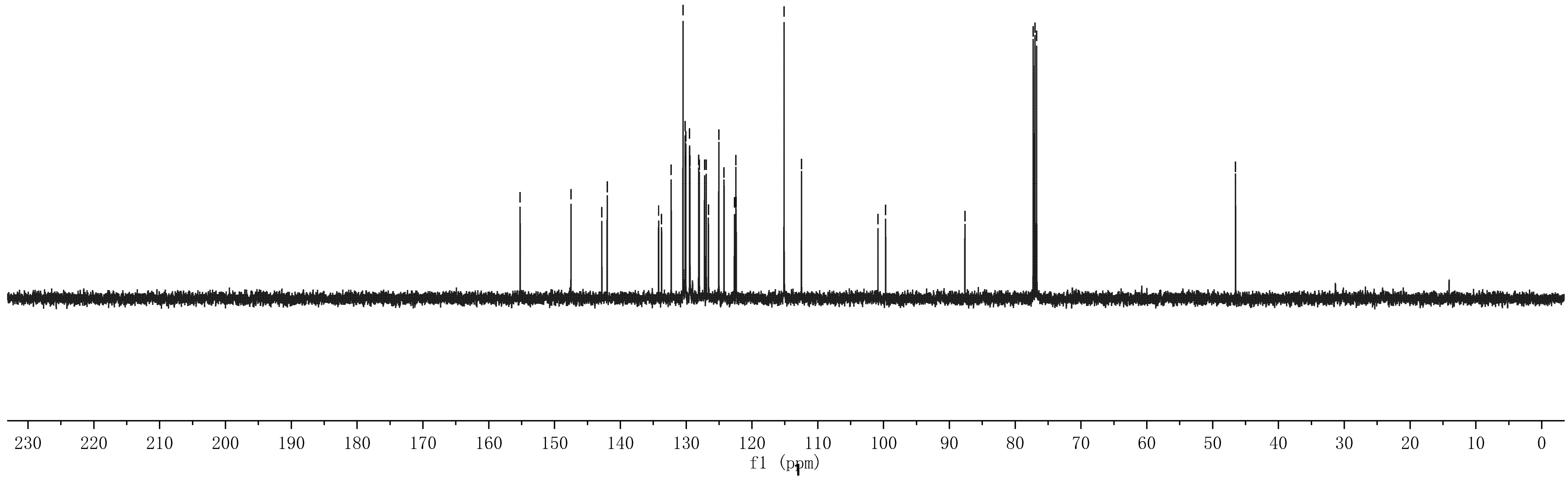

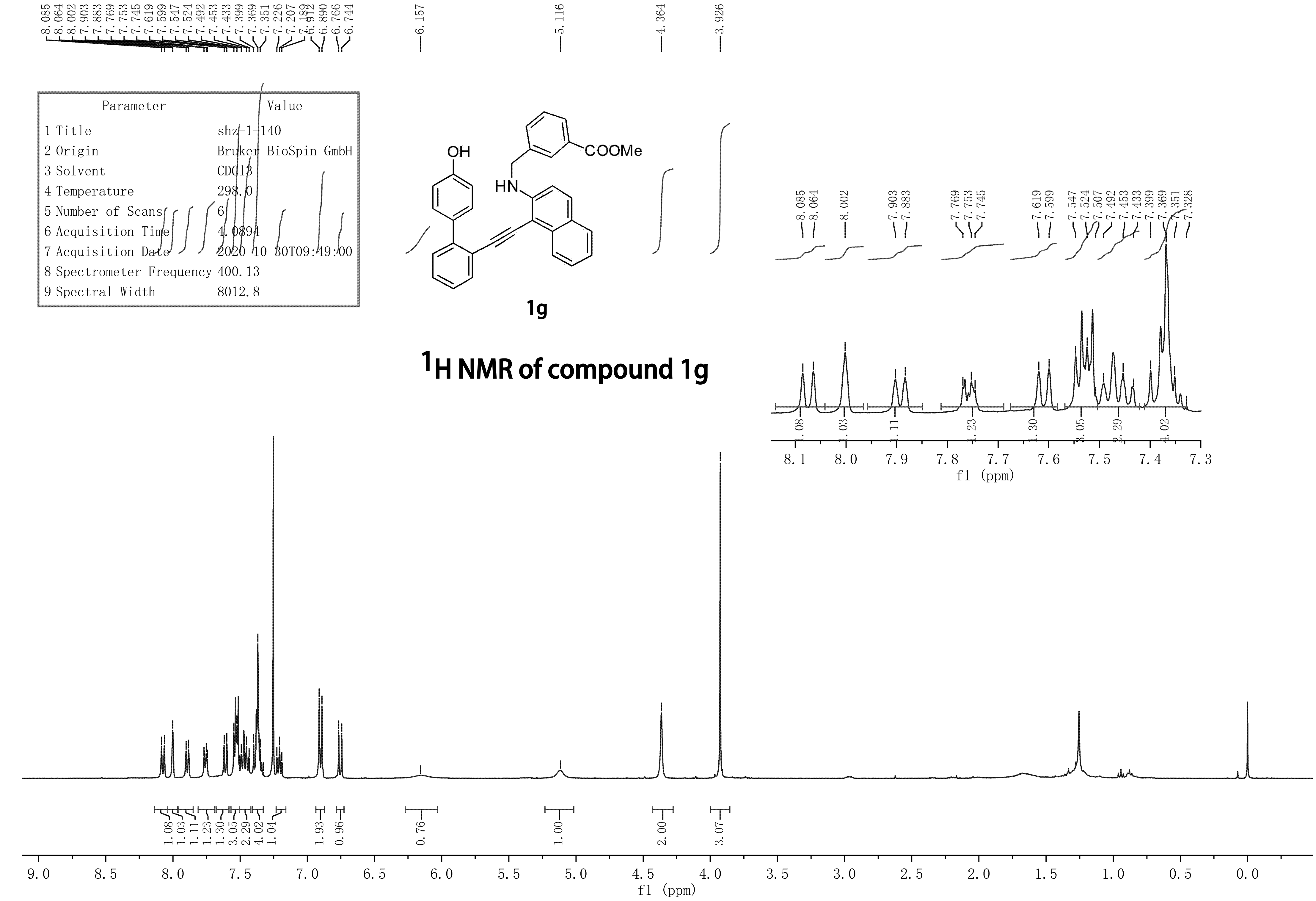

^{1}H NMR of compound 1g

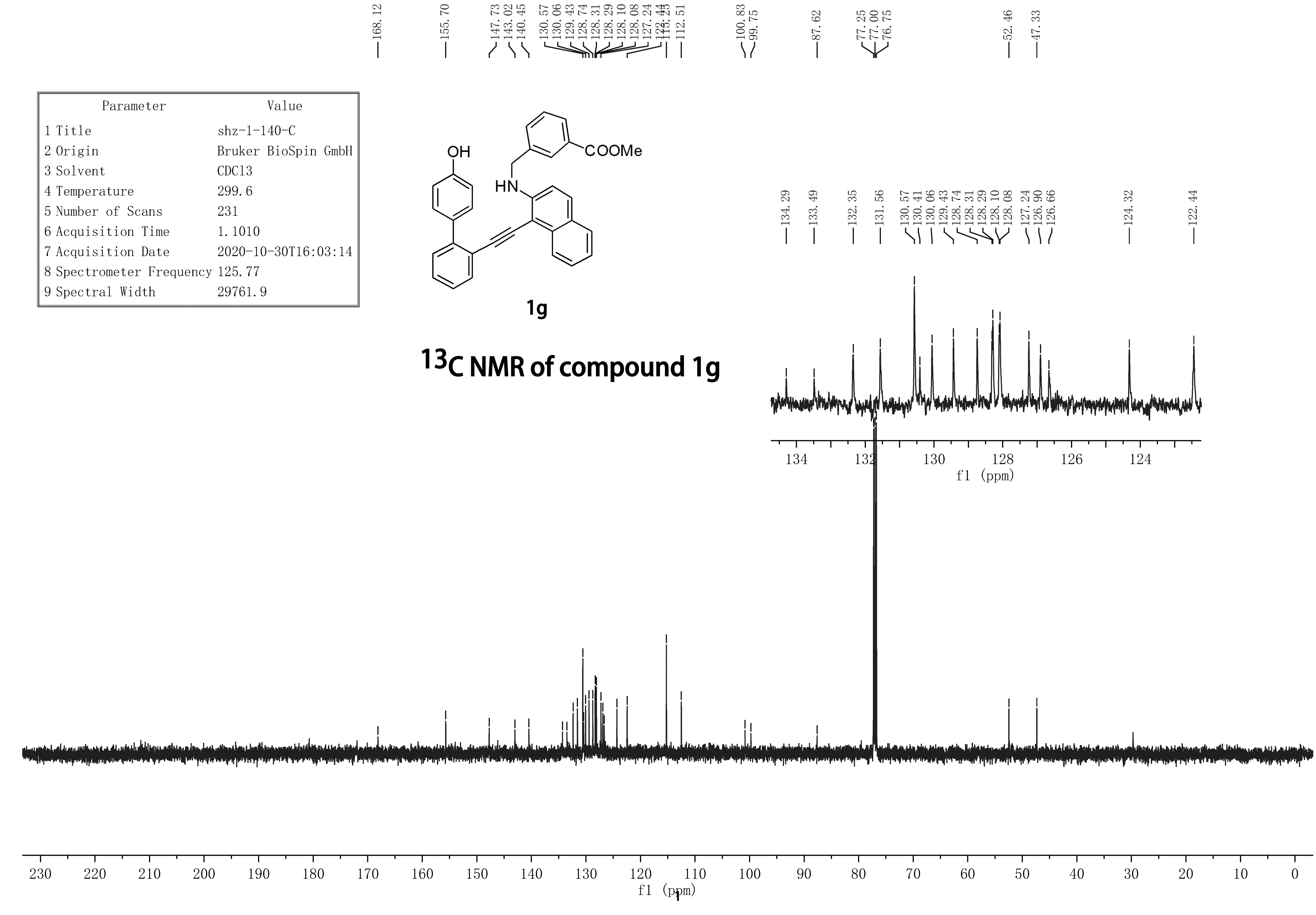

^{13}C NMR of compound 1g

Parameter	Value
1 Title	shz-1-136
2 Origin	Bruker BioSpin GmbH
3 Solvent	CDCl3
4 Temperature	298.0
5 Number of Scans	6
6 Acquisition Time	4.0894
7 Acquisition Date	2020-10-30T09:35:28
8 Spectrometer Frequency	400.13
9 Spectral Width	8012.8

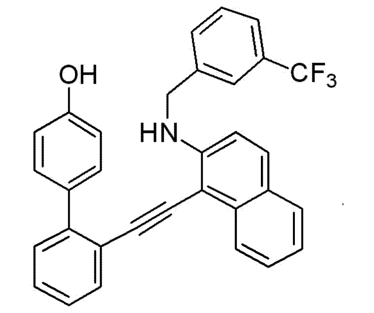

1h

^{1}H NMR of compound 1h

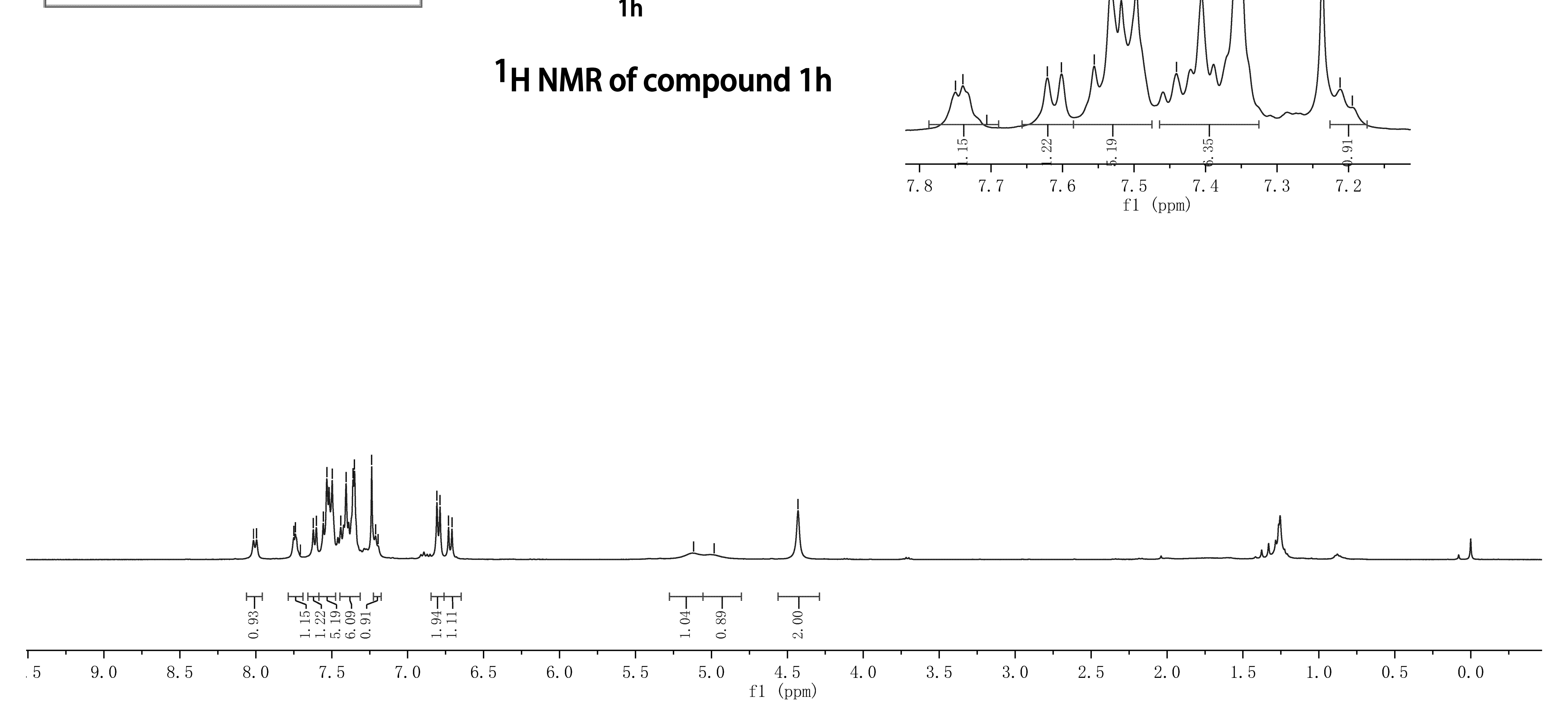

Parameter	Value
1 Title	shz-1-136-C
2 Origin	Bruker BioSpin GmbH
3 Solvent	CDCl3
4 Temperature	299.2
5 Number of Scans	22
6 Acquisition Time	1.1010
7 Acquisition Date	2020-10-29T15:52:46
8 Spectrometer Frequency	125.77
9 Spectral Width	29761.9

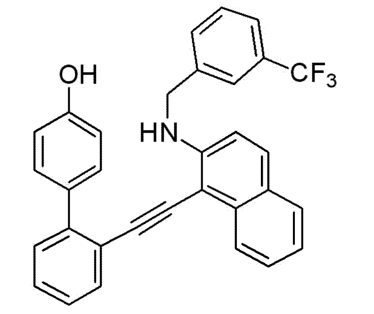

1h

^{13}C NMR of compound 1h

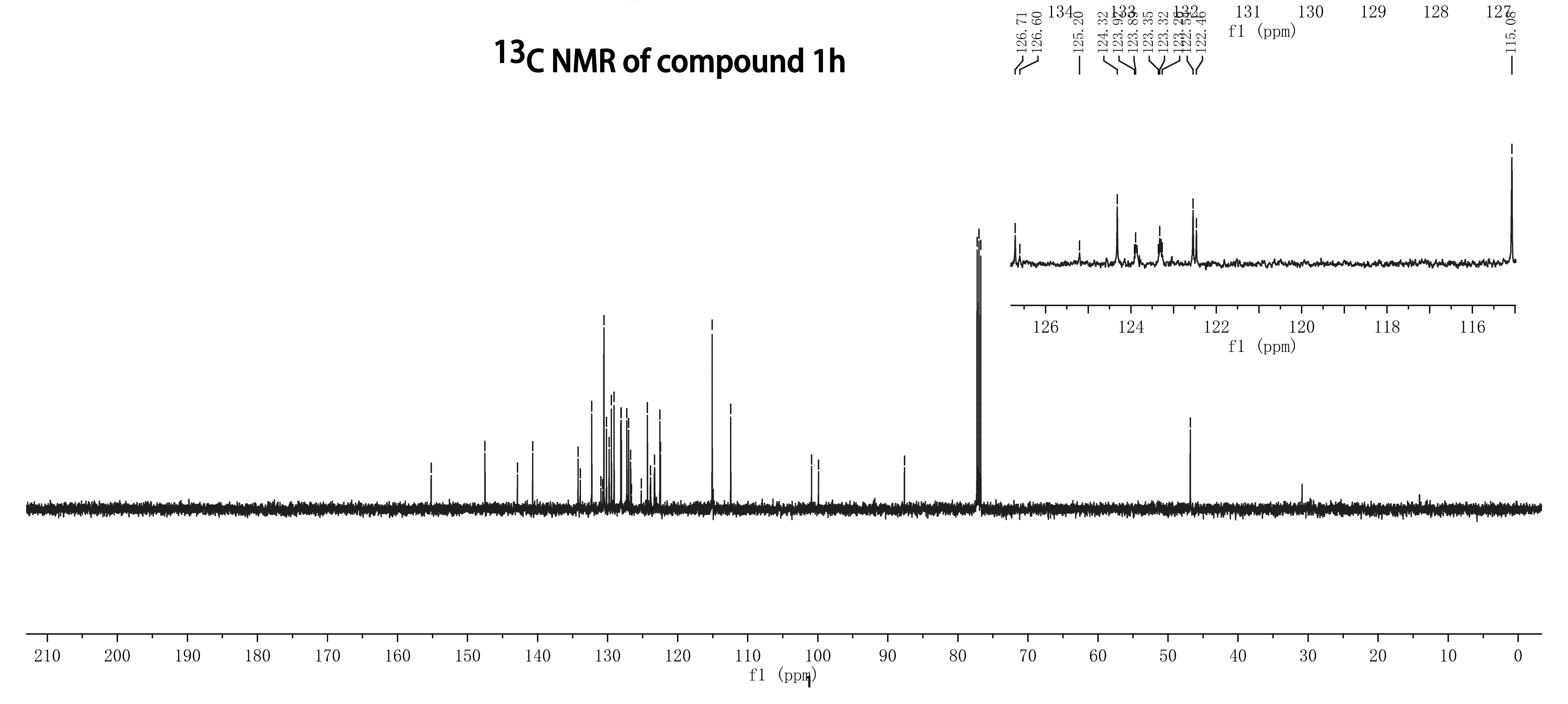

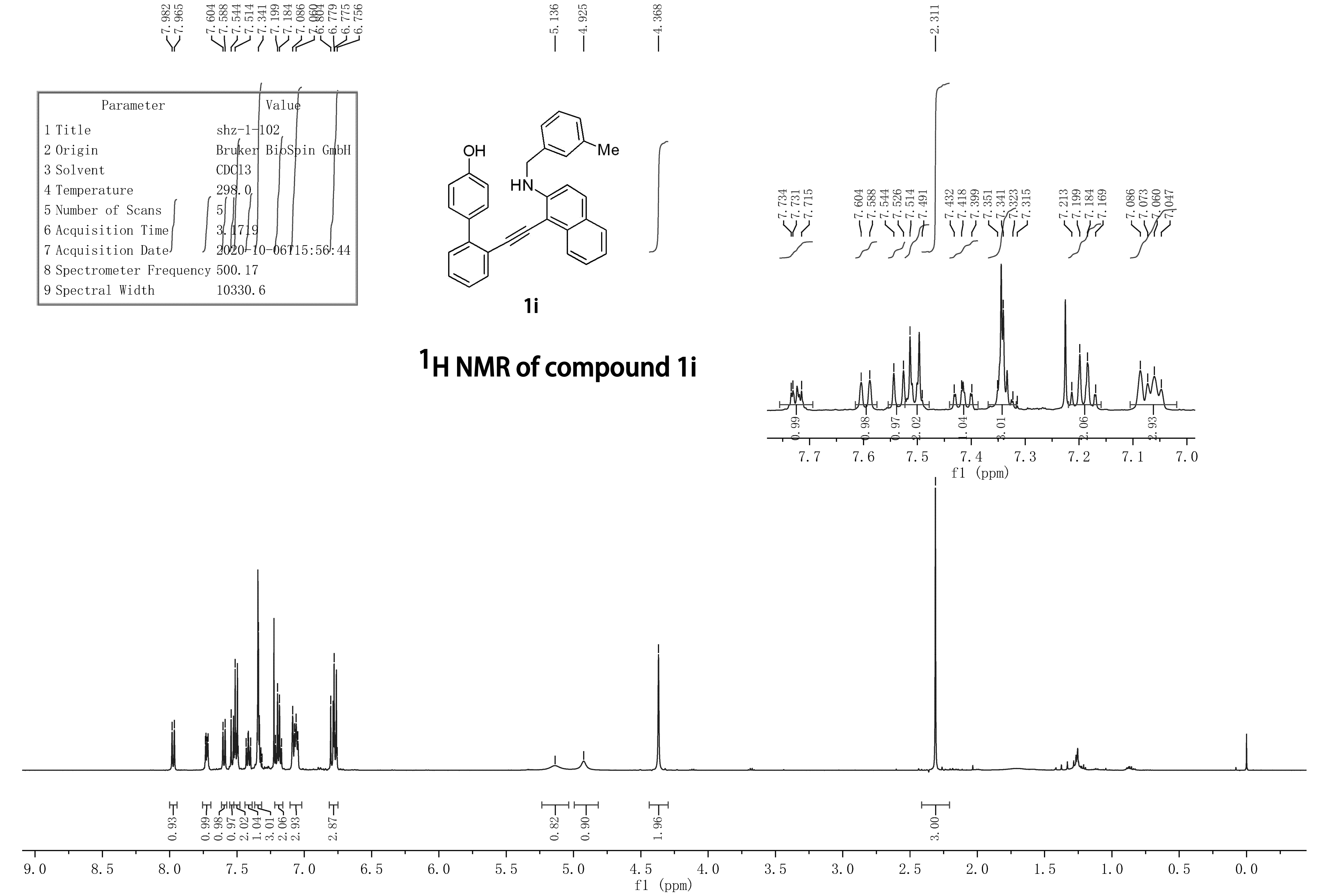

^{1}H NMR of compound 1i

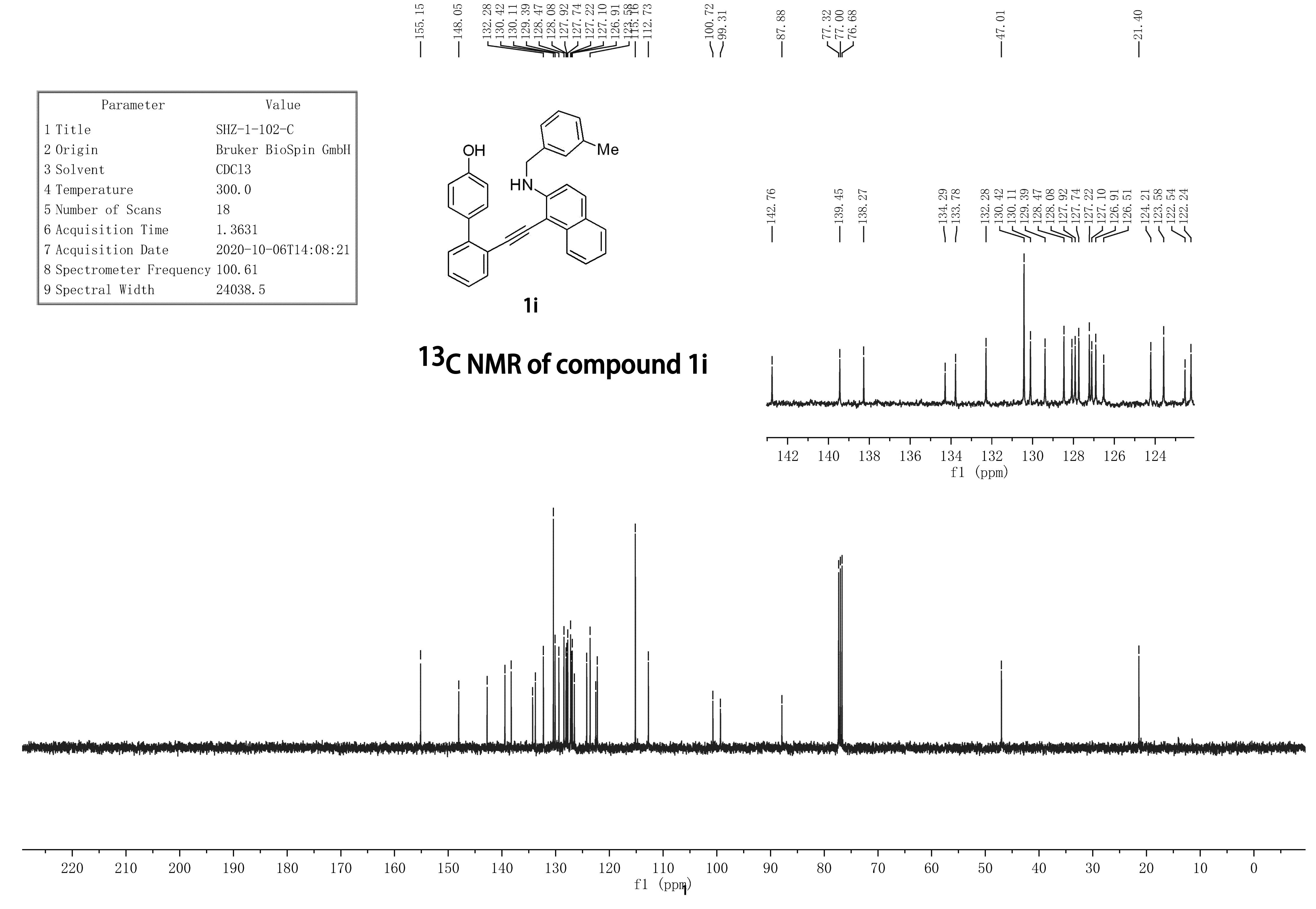

^{13}C NMR of compound 1i

Parameter	Value
1 Title	shz-1-128
2 Origin	Bruker BioSpin GmbH
3 Solvent	CDCl3
4 Temperature	298.0
5 Number of Scans	7
6 Acquisition Time	4.0894
7 Acquisition Date	2020-10-23T10:06:03
8 Spectrometer Frequency	400.13
9 Spectral Width	8012.8

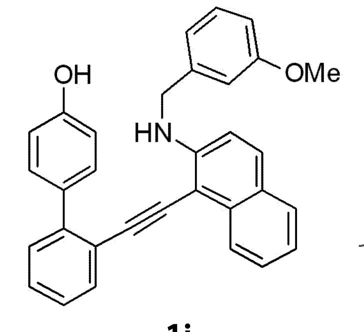

1j

^{1}H NMR of compound 1j

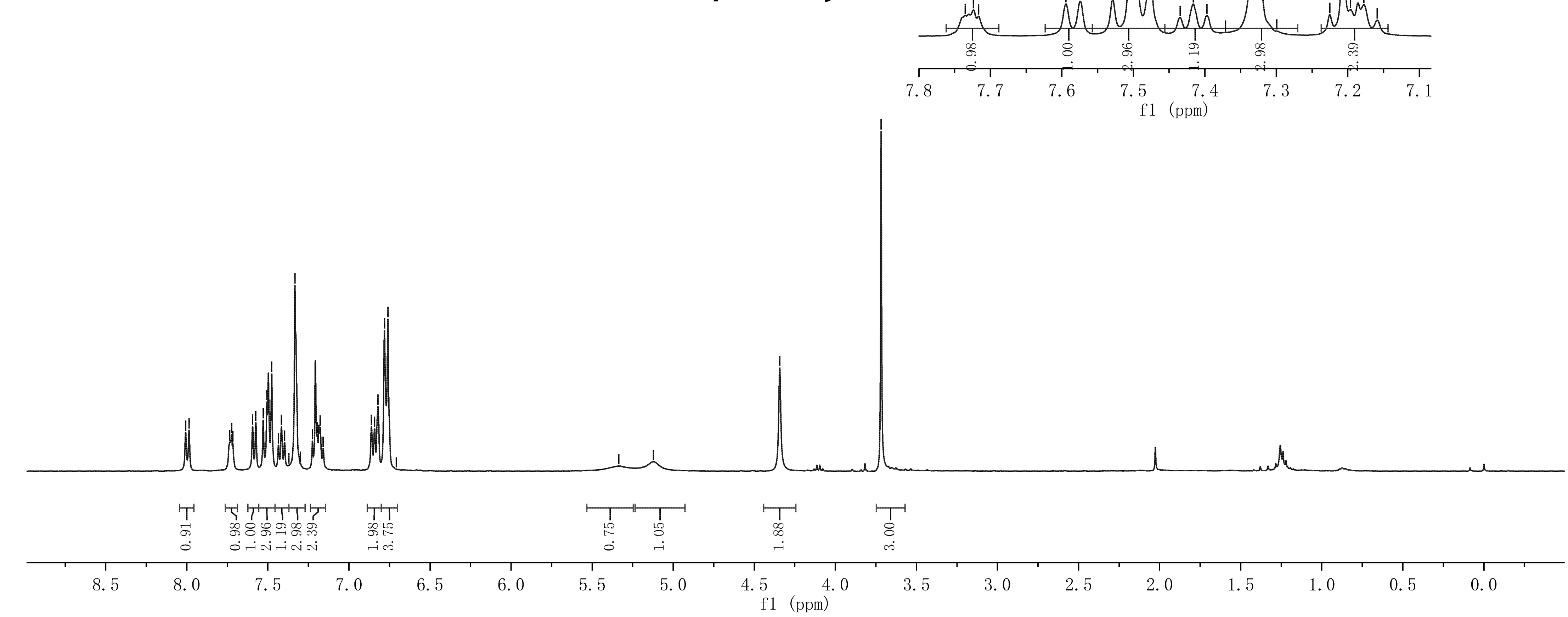

Parameter	Value
1 Title	shz-1-128-C
2 Origin	Bruker BioSpin GmbH
3 Solvent	CDCl3
4 Temperature	300.0
5 Number of Scans	38
6 Acquisition Time	1.3631
7 Acquisition Date	2020-10-23T10:07:37
8 Spectrometer Frequency	100.61
9 Spectral Width	24038.5

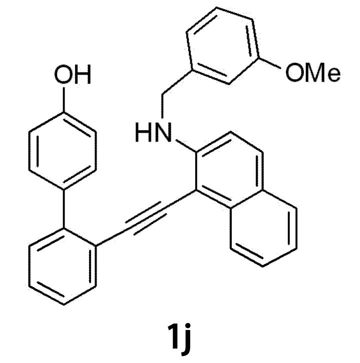

1j

^{13}C NMR of compound 1j

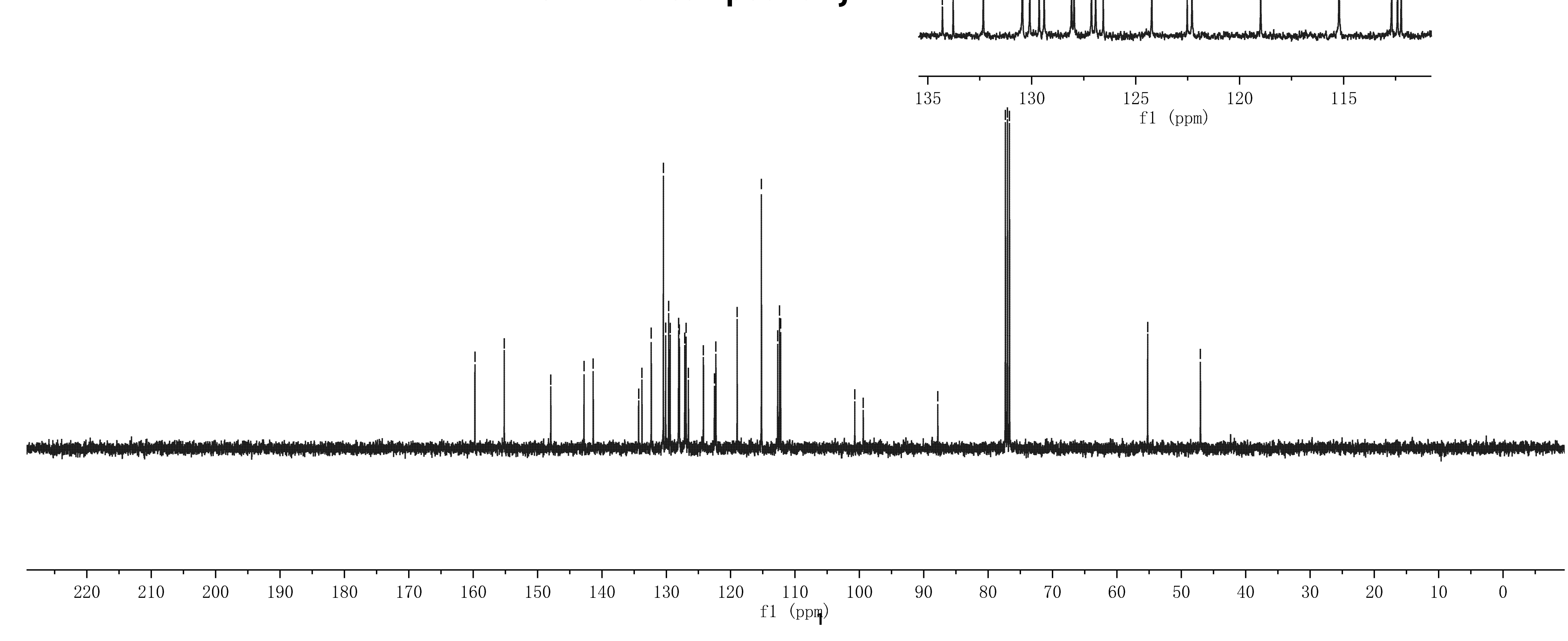

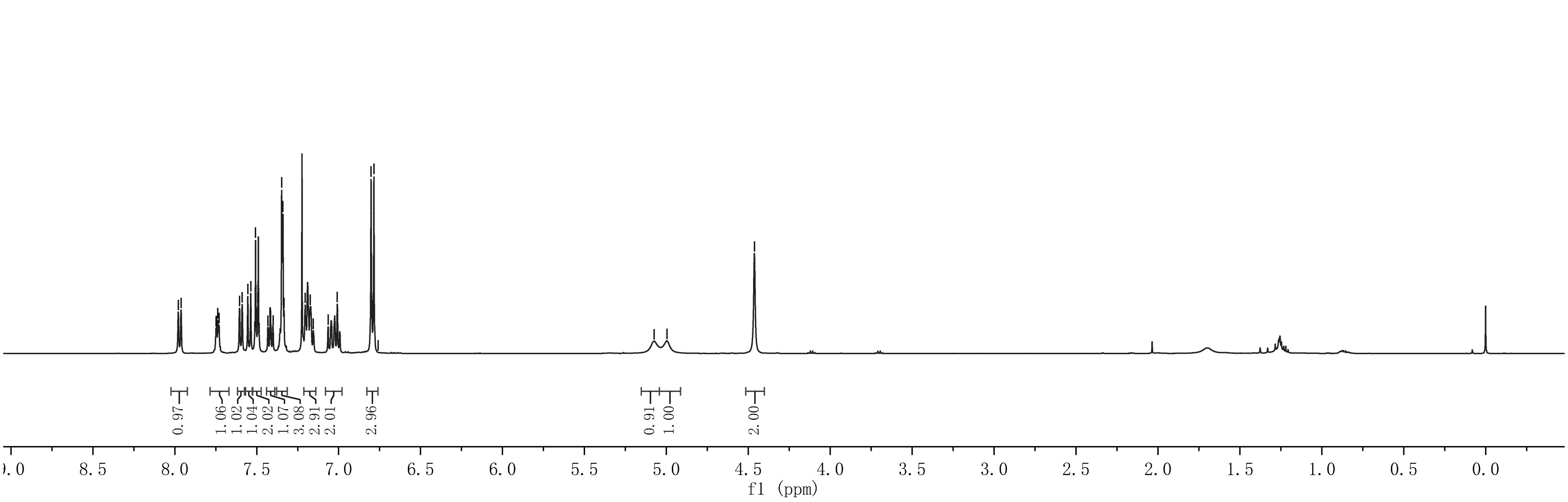

Parameter	Value
1 Title	ttd-21-131
2 Origin	Bruker BioSpin GmbH
3 Solvent	CDCl3
4 Temperature	299.2
5 Number of Scans	8
6 Acquisition Time	3.1719
7 Acquisition Date	2020-09-30T21:16:07
8 Spectrometer Frequency	500.17
9 Spectral Width	10330.6

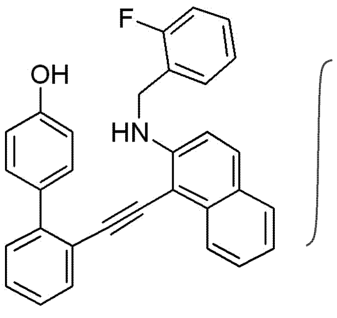

1k

^{1}H NMR of compound 1k

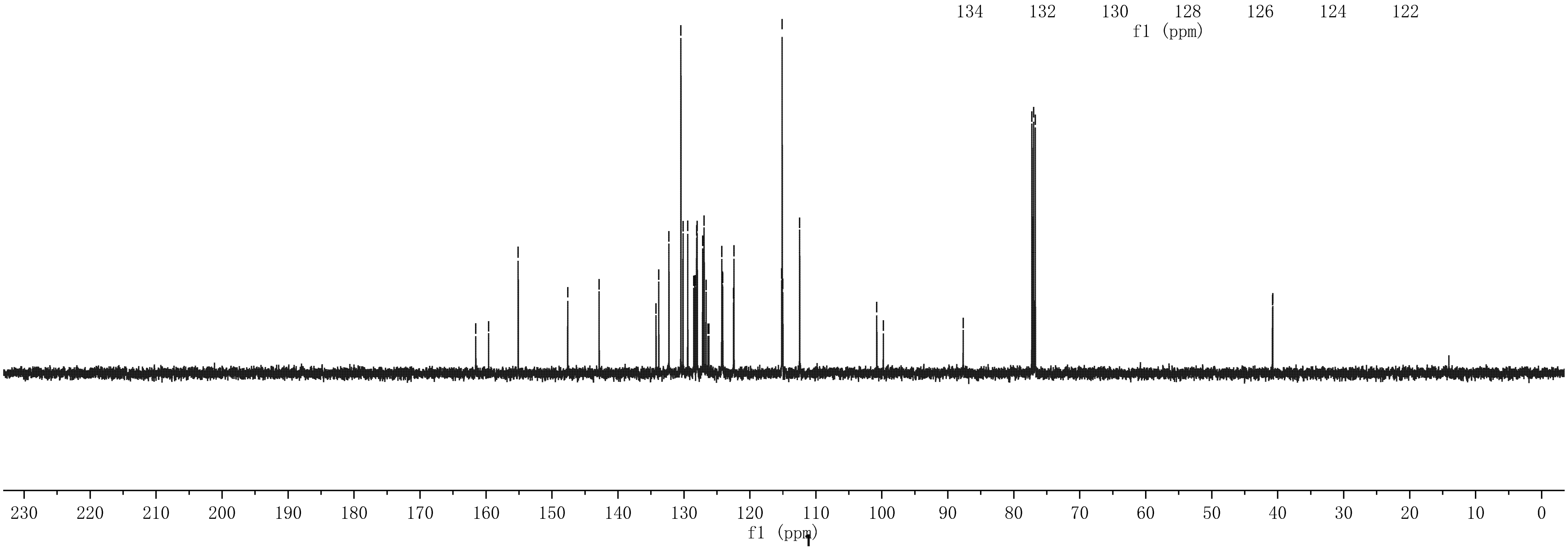

Parameter	Value
1 Title	ttd-21-131-C
2 Origin	Bruker BioSpin GmbH
3 Solvent	CDCl3
4 Temperature	298.9
5 Number of Scans	12
6 Acquisition Time	1.1010
7 Acquisition Date	2020-09-28T20:44:27
8 Spectrometer Frequency	125.77
9 Spectral Width	29761.9

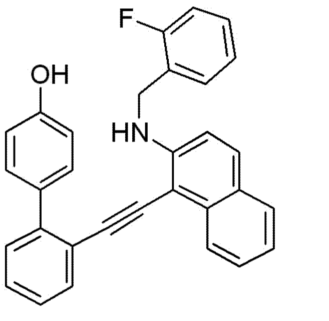

1k

^{13}C NMR of compound 1k

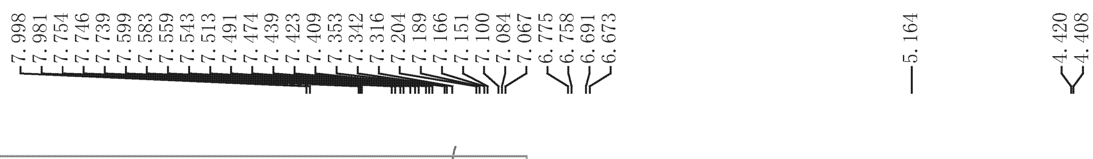

Parameter	Value
1 Title	ttd-21-105
2 Origin	Bruker BioSpin GmbH
3 Solvent	CDCl3
4 Temperature	298.2
5 Number of Scans	11
6 Acquisition Time	3.1719
7 Acquisition Date	2020-09-22T15:18:05
8 Spectrometer Frequency	500.17
9 Spectral Width	10330.6

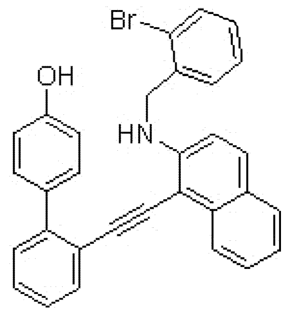

1l

^{1}H NMR of compound 1l

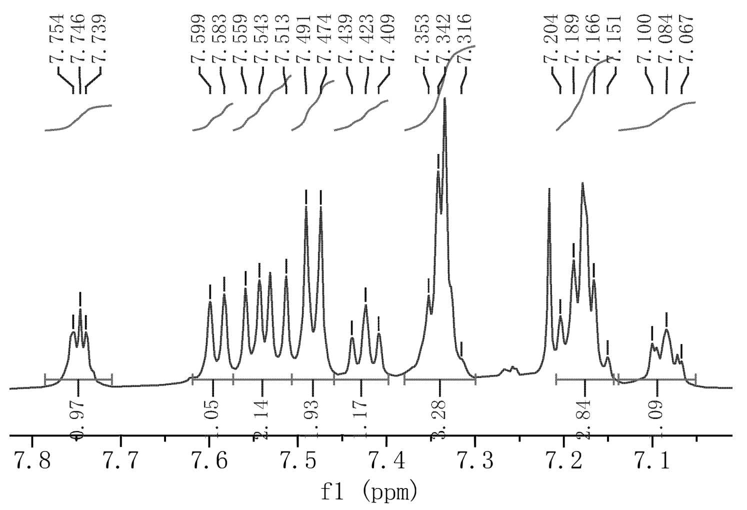

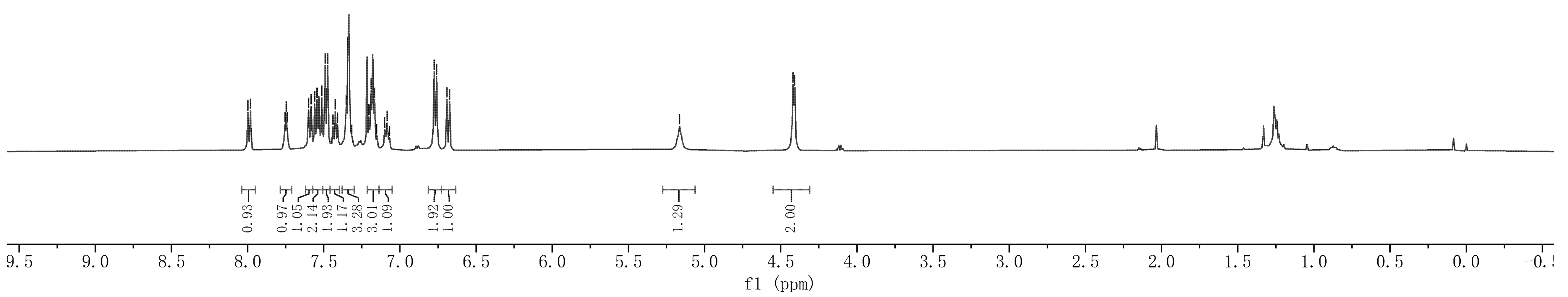

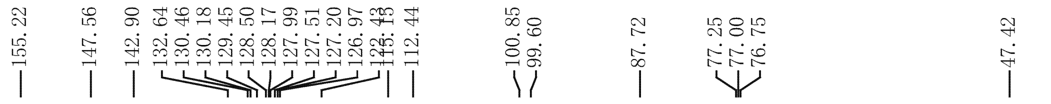

Parameter	Value
1 Title	ttd-21-105-C
2 Origin	Bruker BioSpin GmbH
3 Solvent	CDCl3
4 Temperature	298.6
5 Number of Scans	26
6 Acquisition Time	1.1010
7 Acquisition Date	2020-09-22T15:21:14
8 Spectrometer Frequency	125.77
9 Spectral Width	29761.9

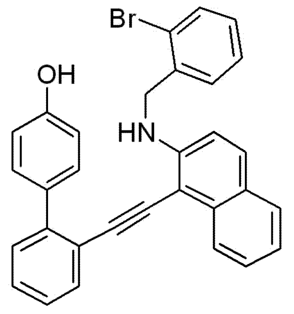

1l

^{13}C NMR of compound 1l

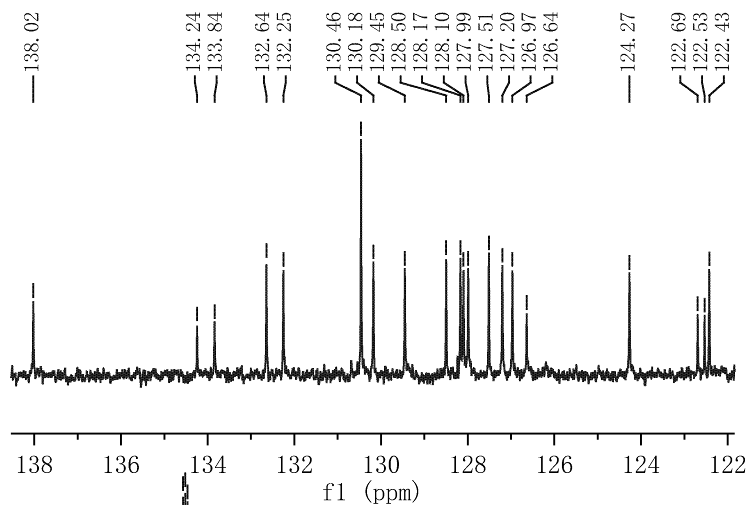

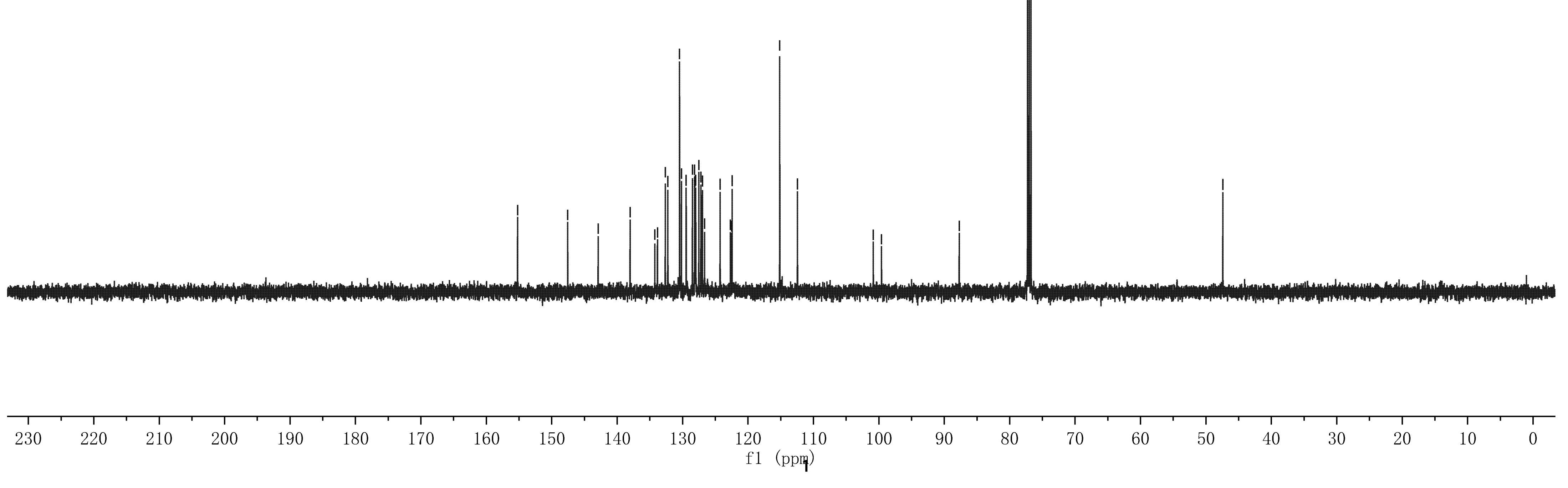

Parameter	Value
1 Title	ttd-21-124
2 Origin	Bruker BioSpin GmbH
3 Solvent	CDCl3
4 Temperature	298.1
5 Number of Scans	6
6 Acquisition Time	3.1719
7 Acquisition Date	2020-09-26T16:01:14
8 Spectrometer Frequency	500.17
9 Spectral Width	10330.6

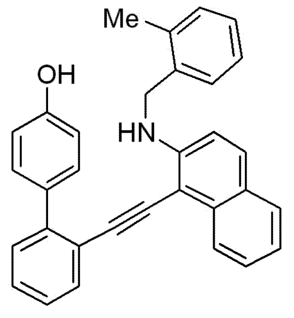

1m

^{1}H NMR of compound 1m

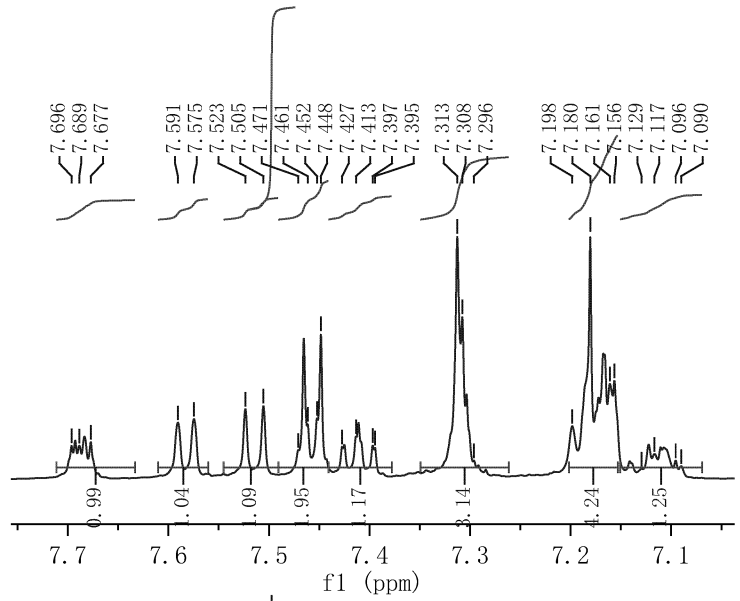

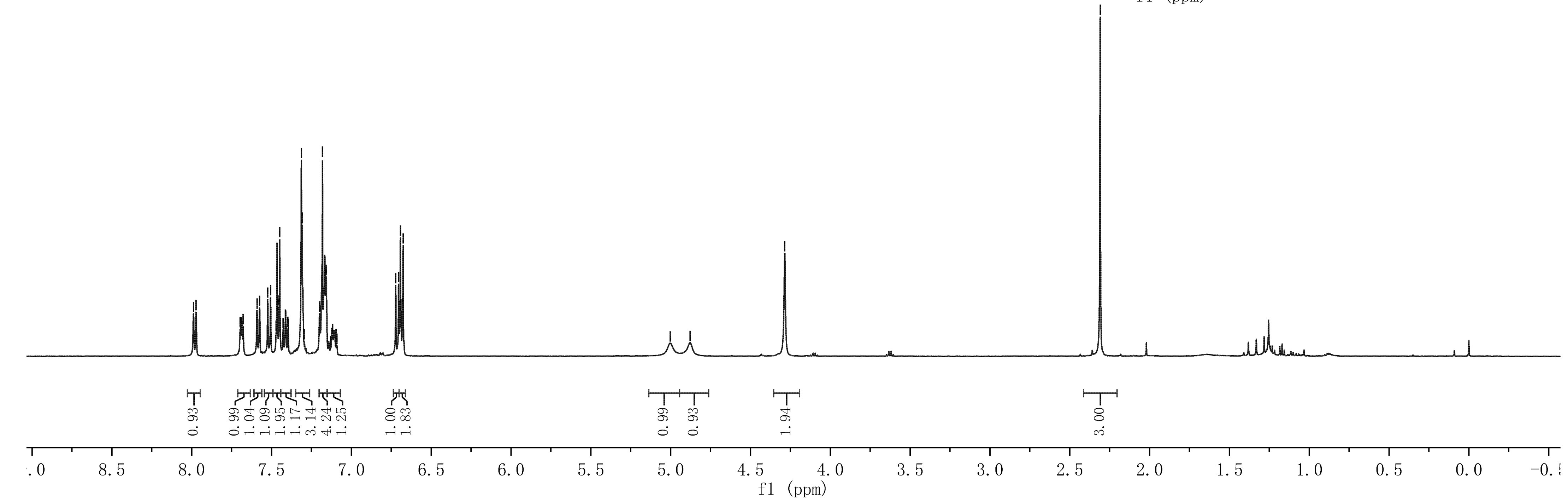

Parameter	Value
1 Title	ttd-21-124-C
2 Origin	Bruker BioSpin GmbH
3 Solvent	CDCl3
4 Temperature	298.4
5 Number of Scans	11
6 Acquisition Time	1.1010
7 Acquisition Date	2020-09-26T16:02:21
8 Spectrometer Frequency	125.77
9 Spectral Width	29761.9

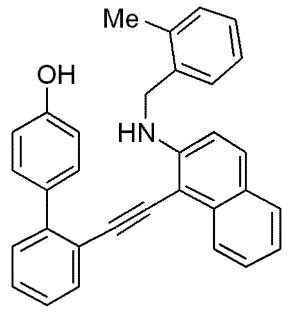

1m

^{13}C NMR of compound 1m

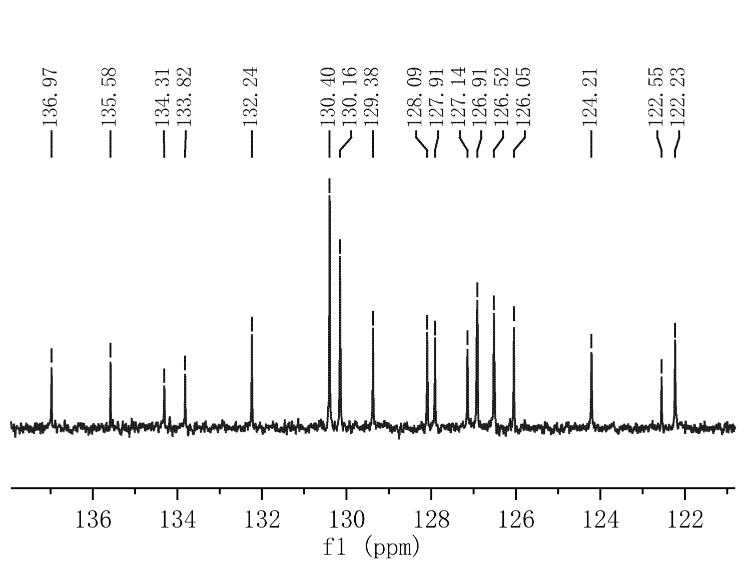

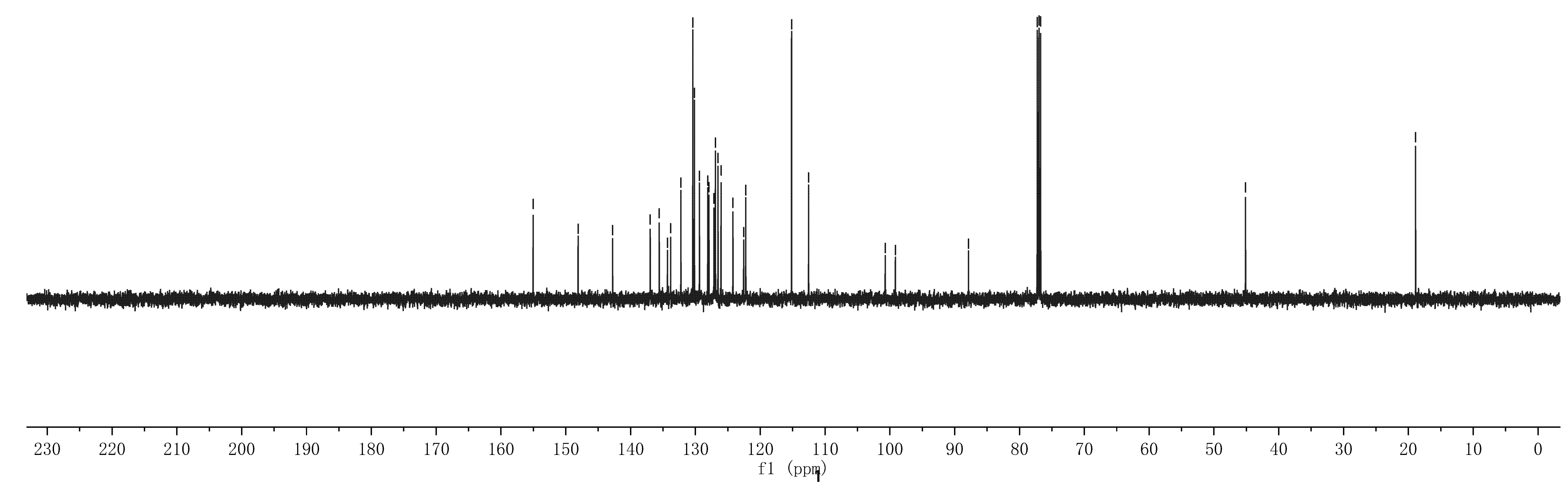

Parameter	Value
1 Title	bhz-2-64
2 Origin	Bruker BioSpin GmbH
3 Solvent	CDCl3
4 Temperature	298.5
5 Number of Scans	5
6 Acquisition Time	3.1719
7 Acquisition Date	2020-10-08T16:58:24
8 Spectrometer Frequency	500.17
9 Spectral Width	10330.6

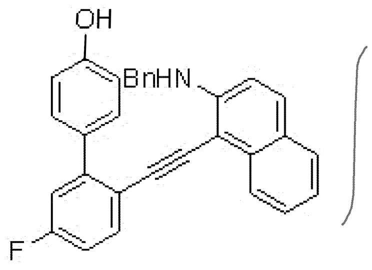

1n

^{1}H NMR of compound 1n

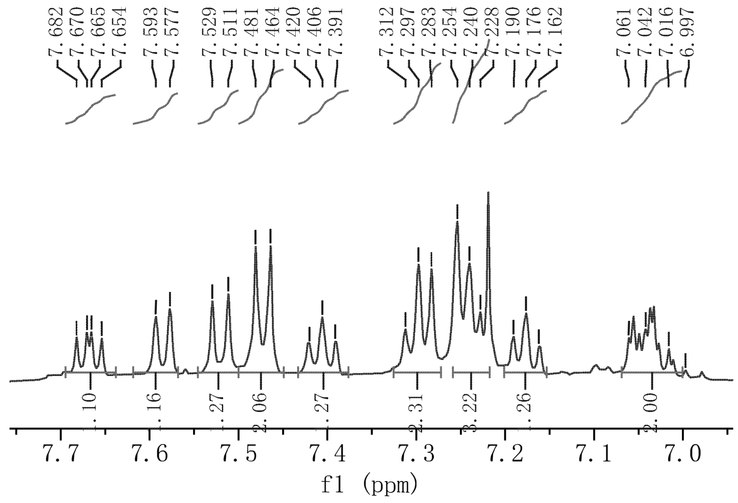

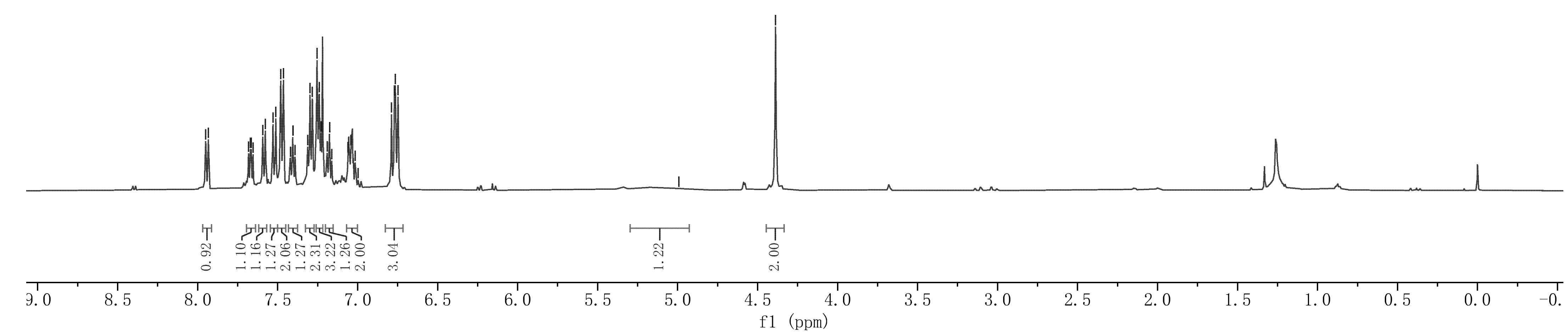

Parameter	Value
1 Title	bhz-2-64-C
2 Origin	Bruker BioSpin GmbH
3 Solvent	CDCl3
4 Temperature	299.1
5 Number of Scans	52
6 Acquisition Time	1.1010
7 Acquisition Date	2020-10-08T16:07:26
8 Spectrometer Frequency	125.77
9 Spectral Width	29761.9

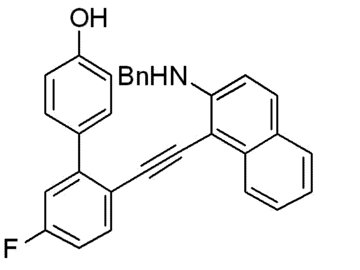

1n

^{13}C NMR of compound 1n

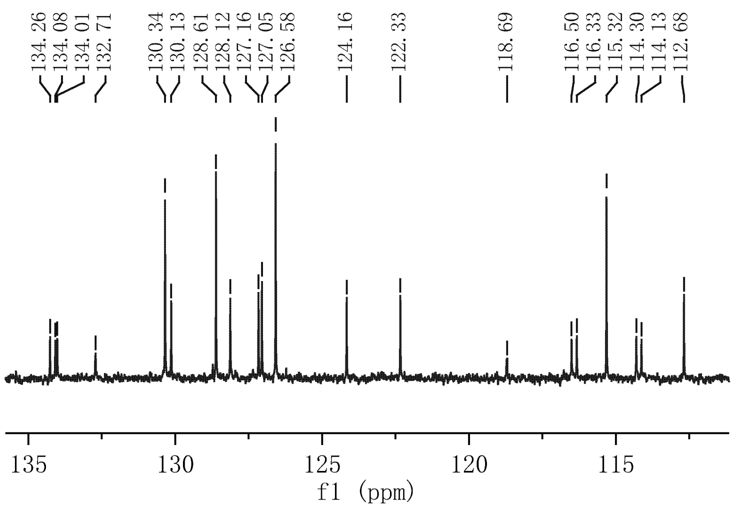

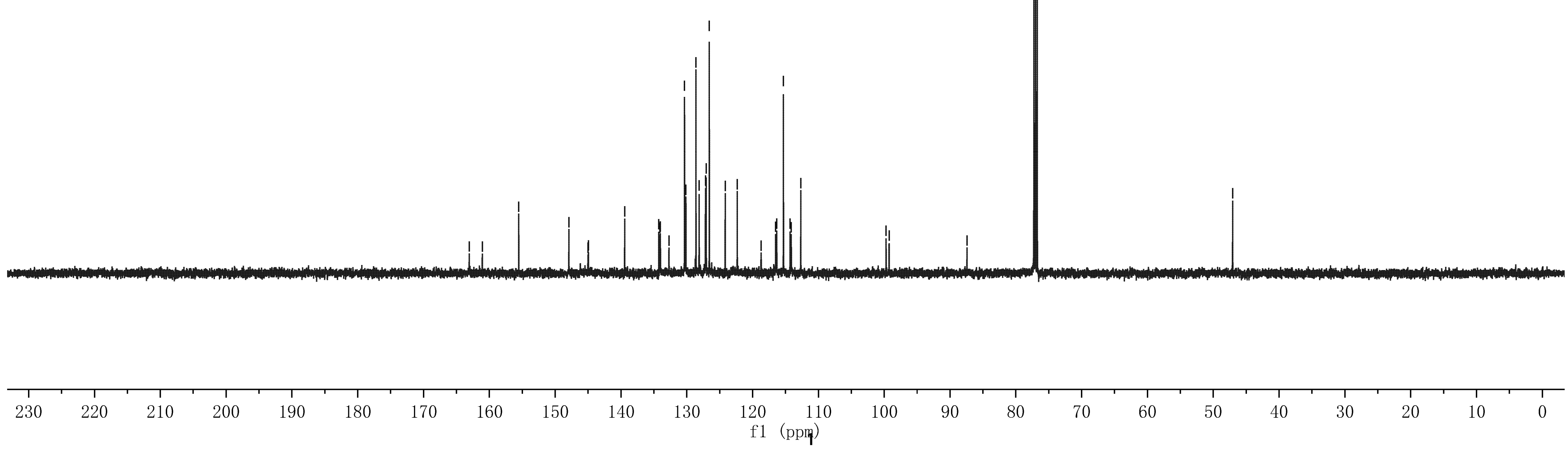

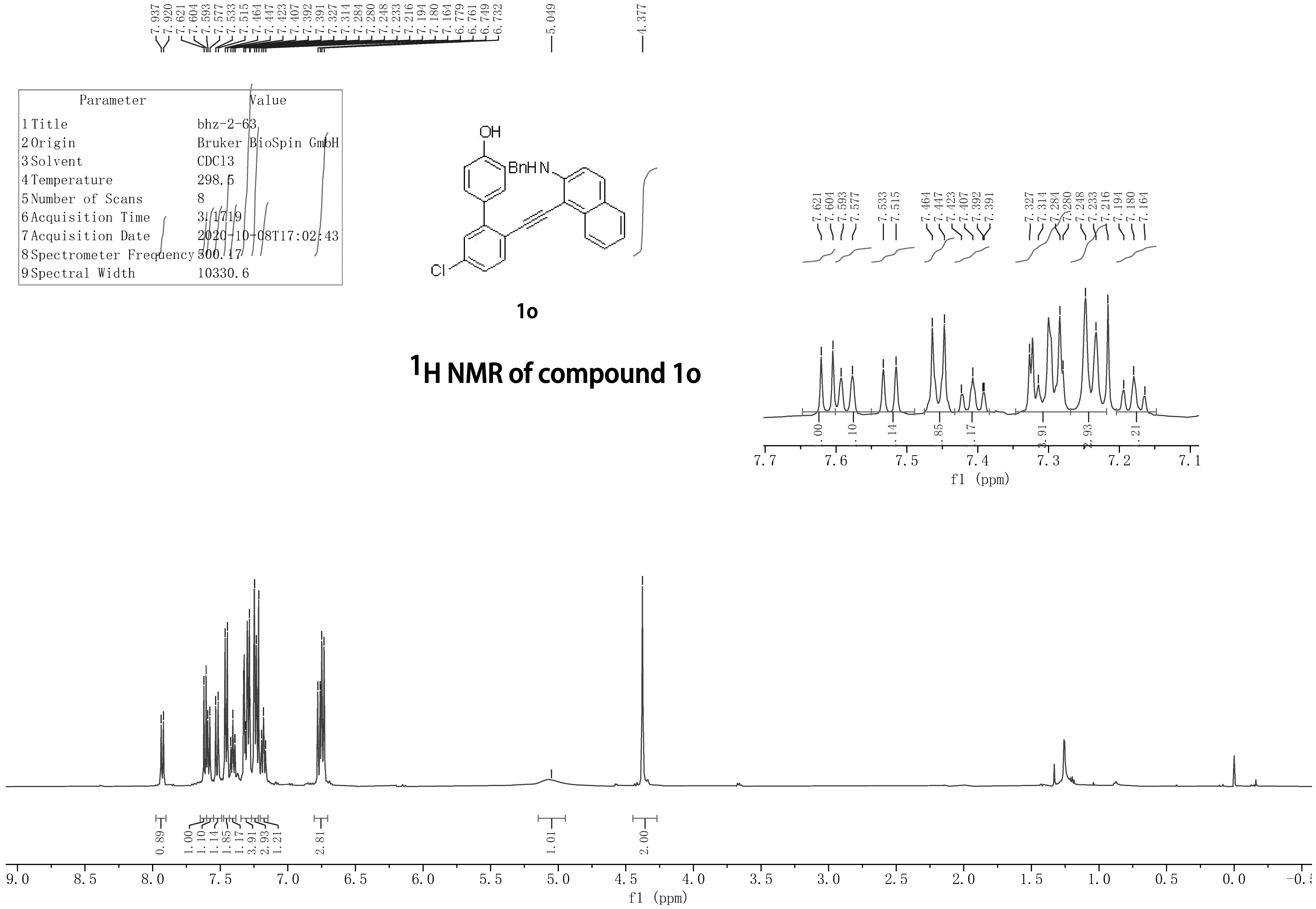

^1H NMR of compound 1o

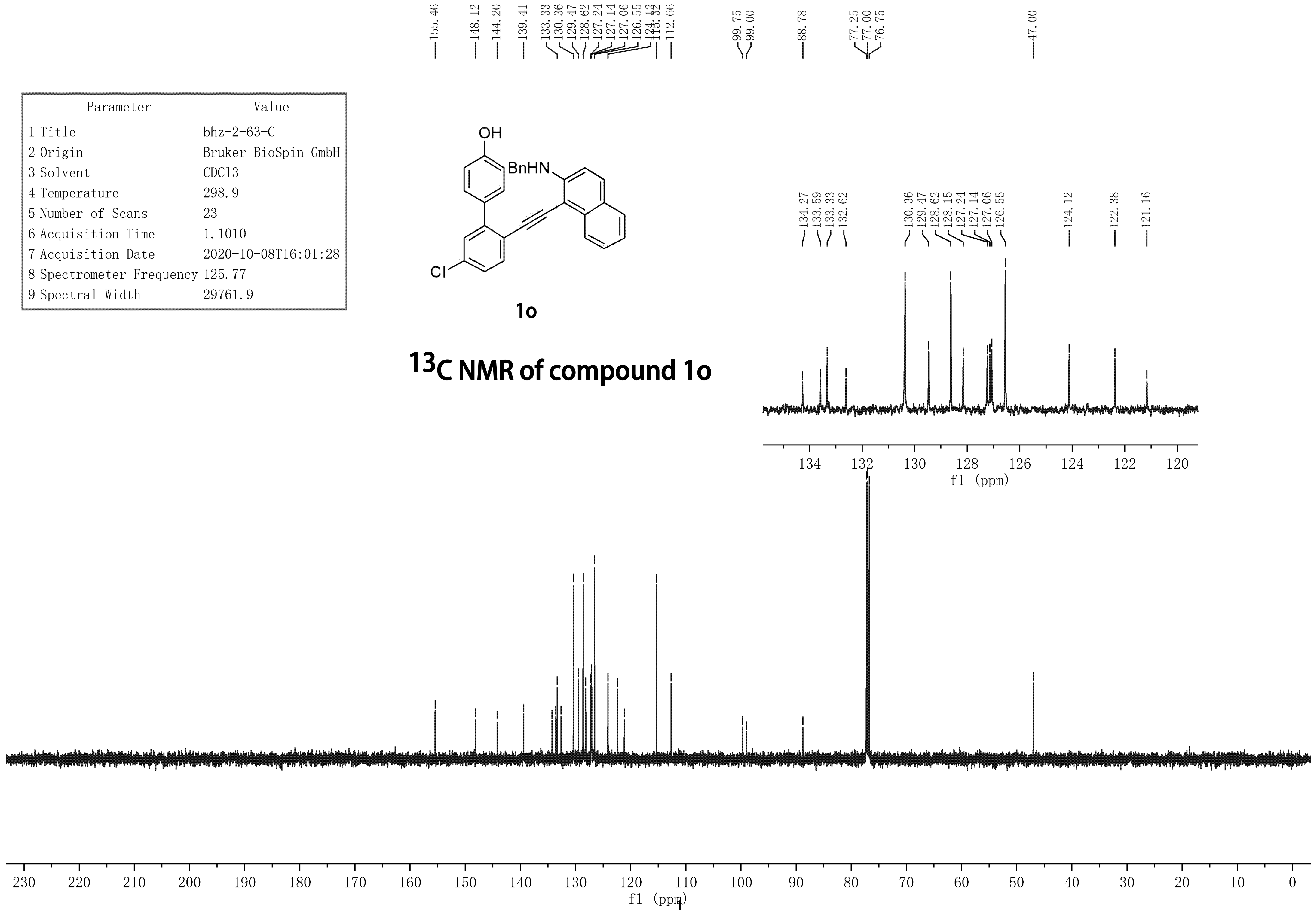

^{13}C NMR of compound 1o

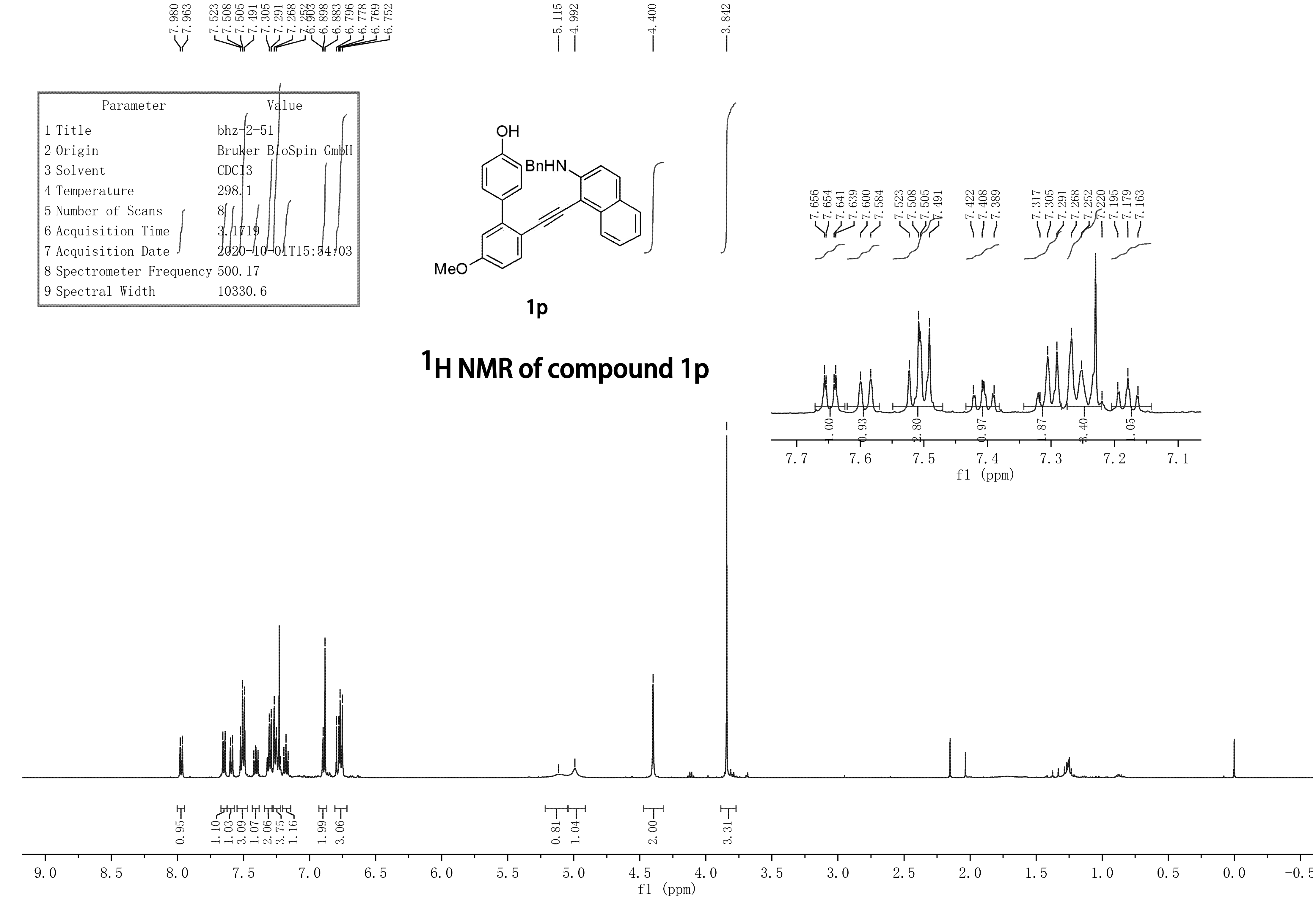

^{1}H NMR of compound 1p

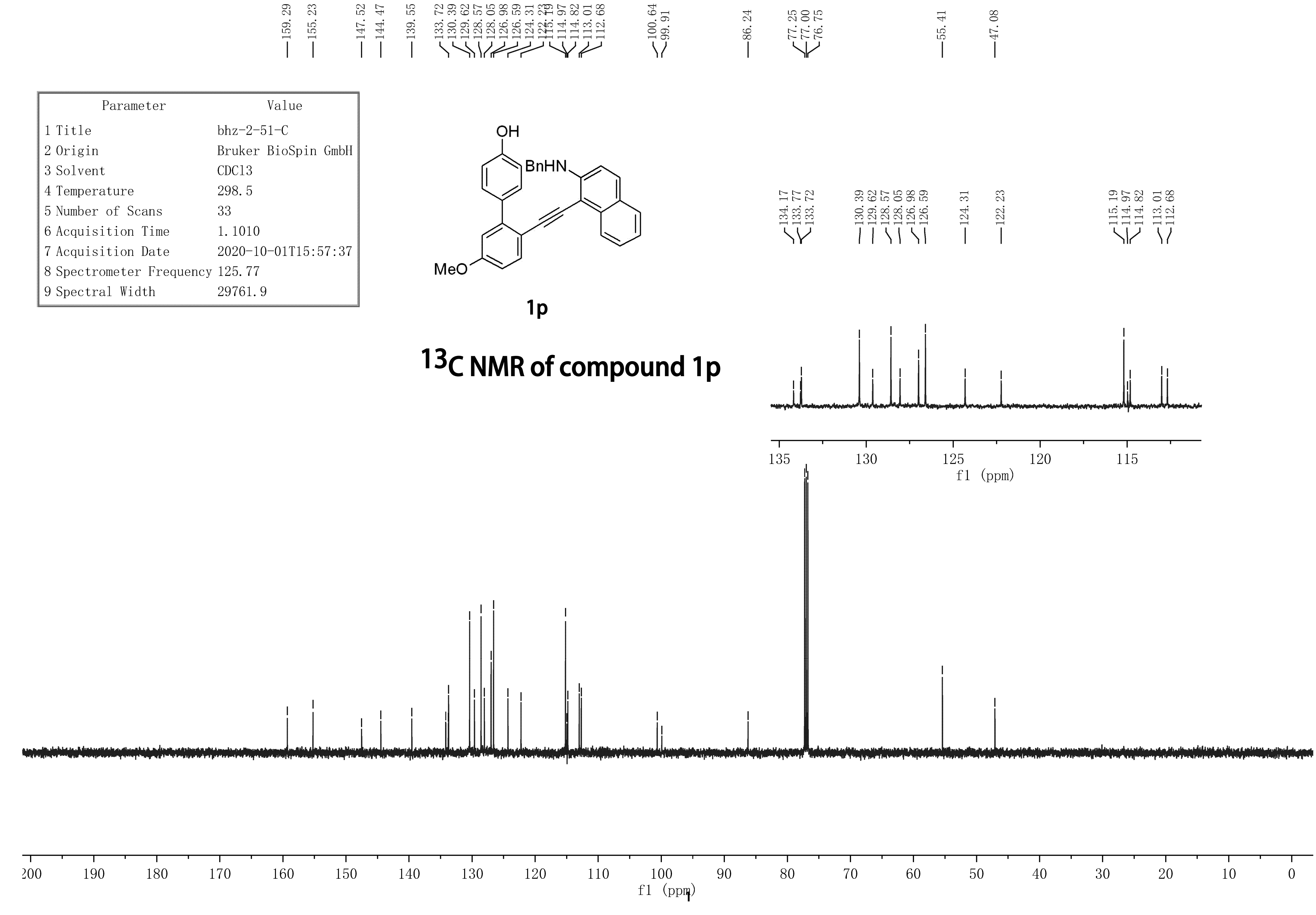

^{13}C NMR of compound 1p

Parameter	Value
1 Title	bhz-2-38-h-1
2 Origin	Bruker BioSpin GmbH
3 Solvent	CDCl3
4 Temperature	298.0
5 Number of Scans	5
6 Acquisition Time	4.0894
7 Acquisition Date	2020-09-27T16:47:55
8 Spectrometer Frequency	400.13
9 Spectral Width	8012.8

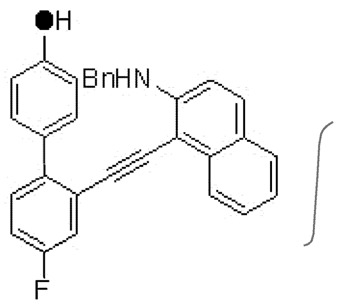

1q

^{1}H NMR of compound 1q

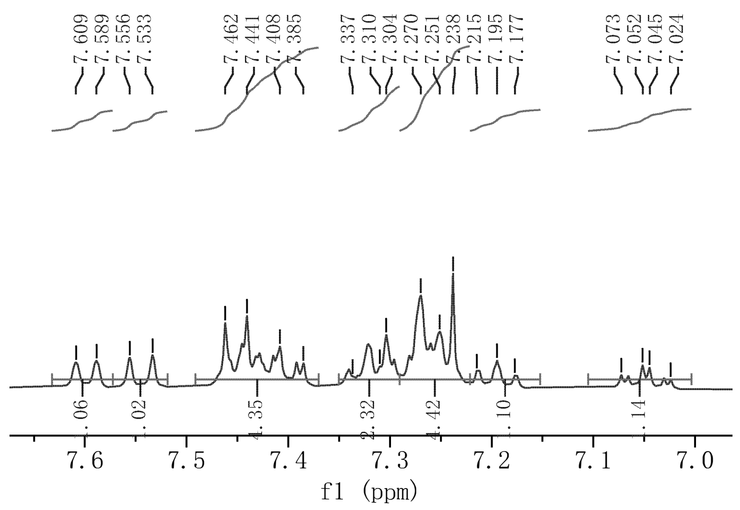

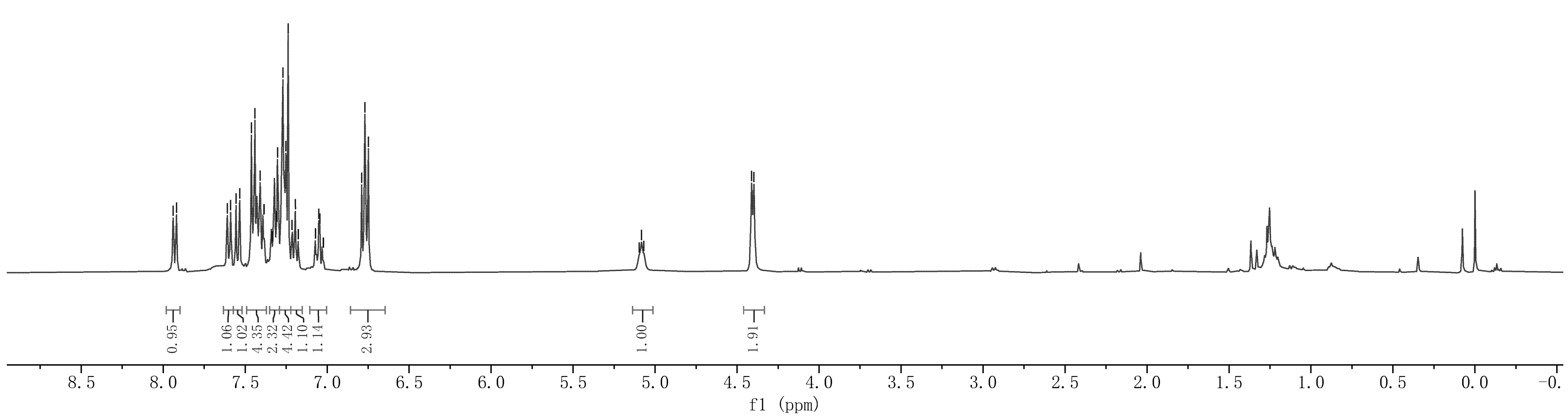

Parameter	Value
1 Title	bhz-2-38-c-1
2 Origin	Bruker BioSpin GmbH
3 Solvent	CDCl3
4 Temperature	300.0
5 Number of Scans	43
6 Acquisition Time	1.3631
7 Acquisition Date	2020-09-27T16:49:35
8 Spectrometer Frequency	100.61
9 Spectral Width	24038.5

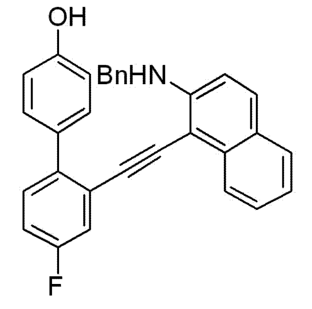

1q

^{13}C NMR of compound 1q

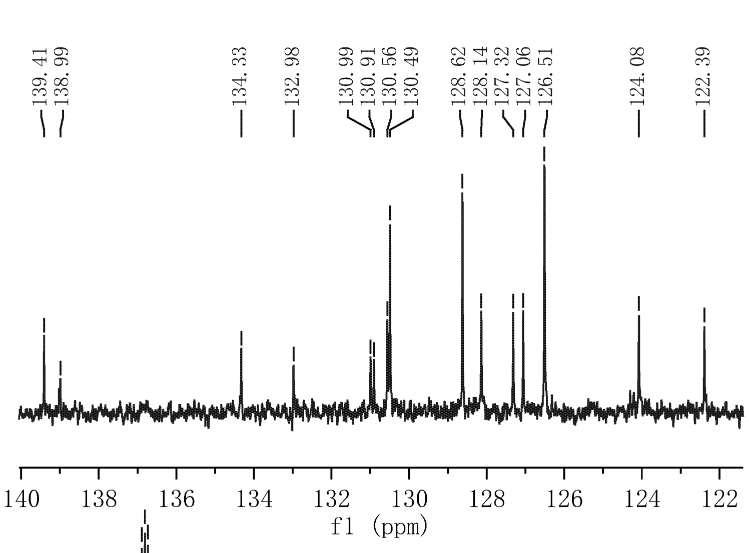

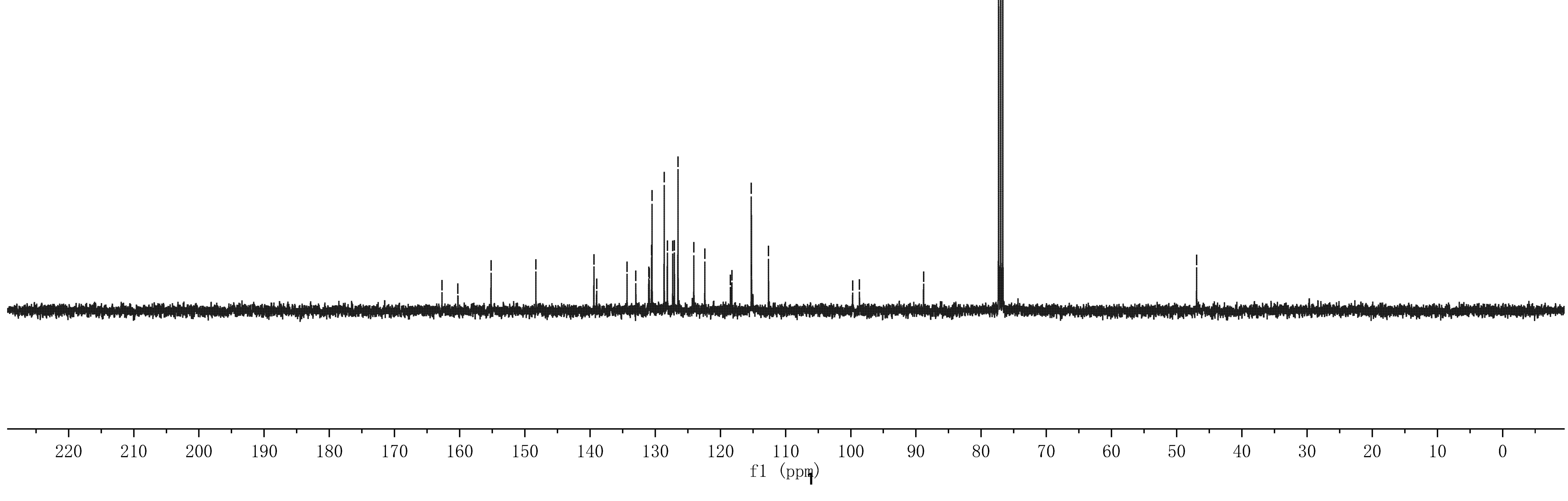

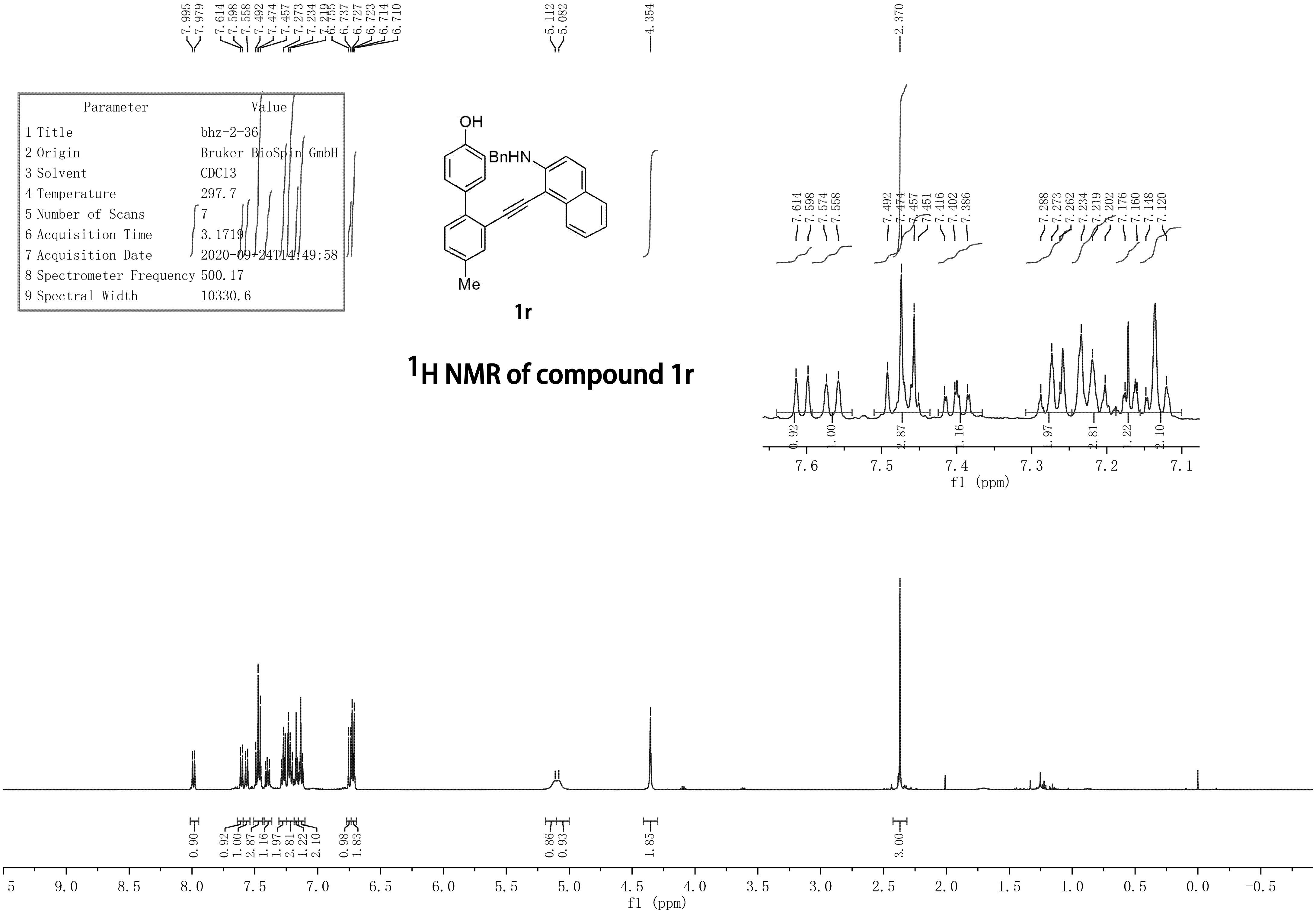

Parameter	Value
1 Title	bhz-2-36
2 Origin	Bruker BioSpin GmbH
3 Solvent	CDCl3
4 Temperature	297.7
5 Number of Scans	7
6 Acquisition Time	3.1719
7 Acquisition Date	2020-09-24T14:49:58
8 Spectrometer Frequency	500.17
9 Spectral Width	10330.6

^{1}H NMR of compound 1r

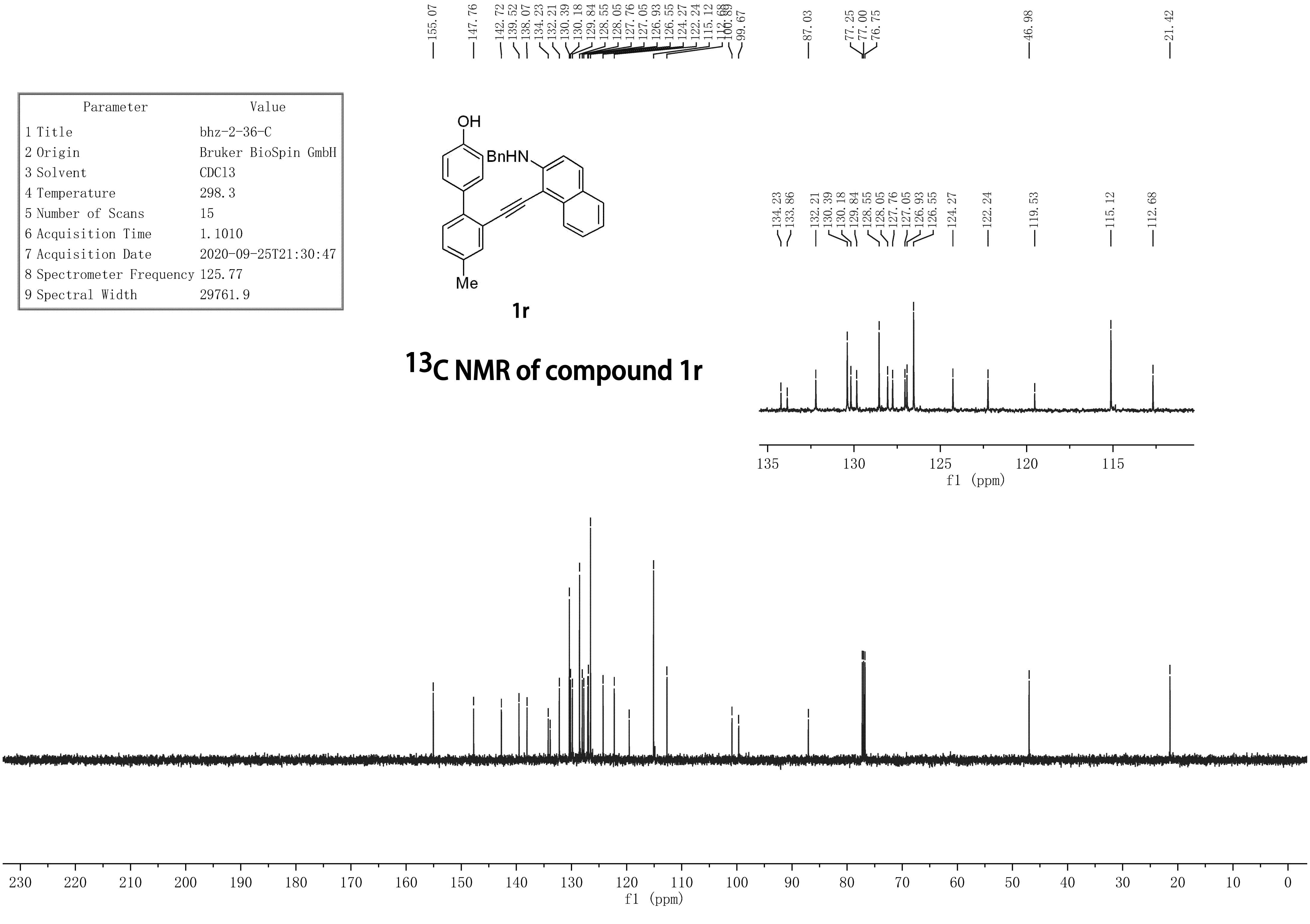

Parameter	Value
1 Title	bhz-2-36-C
2 Origin	Bruker BioSpin GmbH
3 Solvent	CDCl3
4 Temperature	298.3
5 Number of Scans	15
6 Acquisition Time	1.1010
7 Acquisition Date	2020-09-25T21:30:47
8 Spectrometer Frequency	125.77
9 Spectral Width	29761.9

^{13}C NMR of compound 1r

7.975 7.958 7.582 7.566 7.507 7.489 7.274 7.262 7.150 7.129 7.099 7.082 6.822 6.804 6.651 6.634 5.200 4.410 2.861

Parameter	Value
1 Title	ttd-21-216
2 Origin	Bruker BioSpin GmbH
3 Solvent	CDCl3
4 Temperature	298.9
5 Number of Scans	16
6 Acquisition Time	3.1719
7 Acquisition Date	2020-11-03T15:19:14
8 Spectrometer Frequency	500.17
9 Spectral Width	10330.6

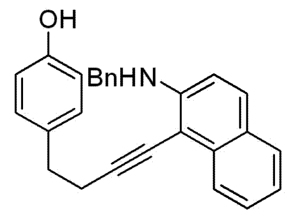

1s

^{1}H NMR of compound 1s

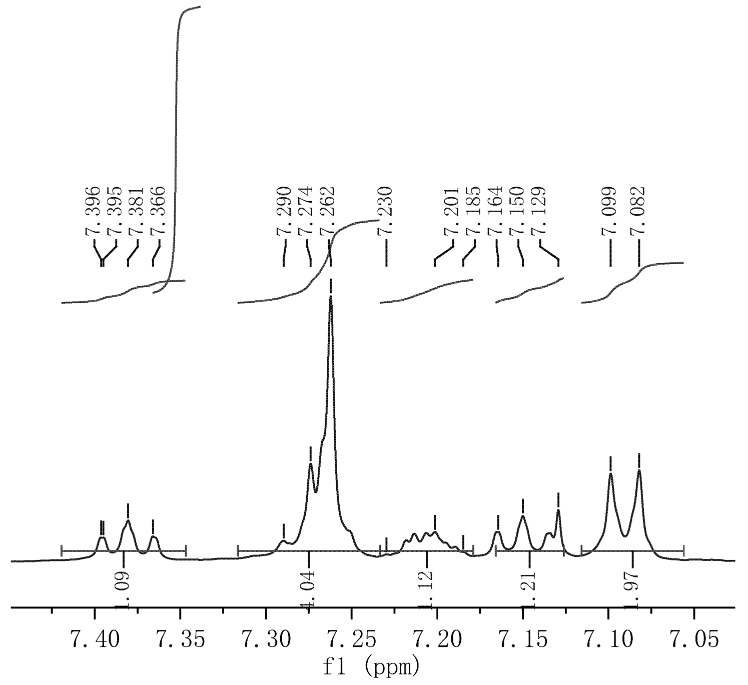

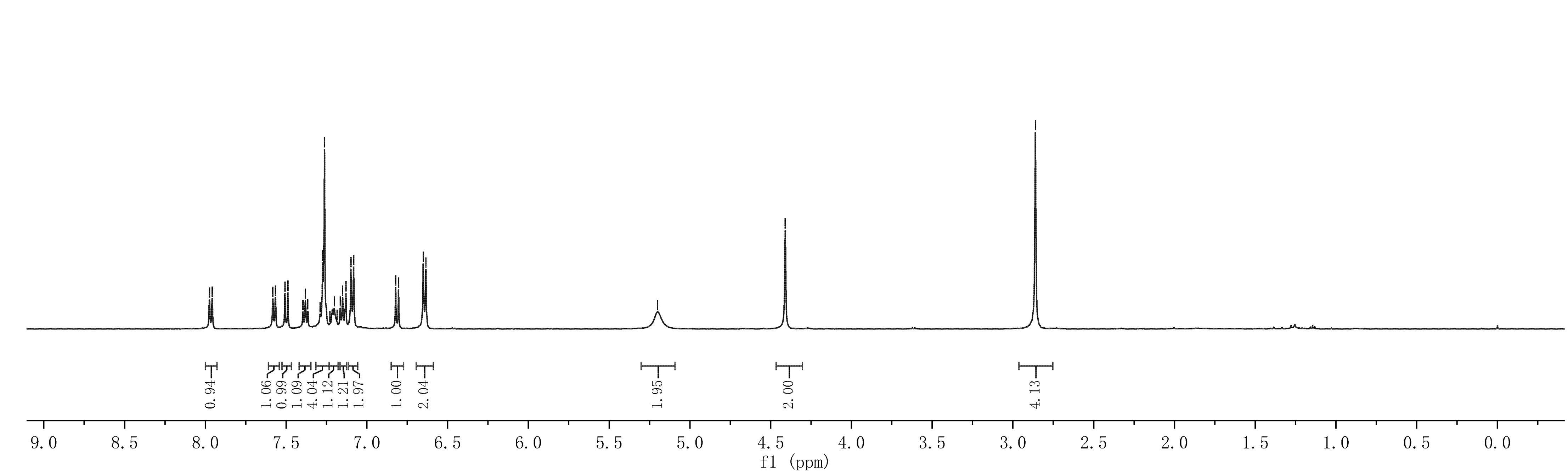

153.90 147.66 139.47 132.64 129.65 129.17 128.54 127.98 126.97 126.88 126.72 124.26 122.11 112.73 100.57 100.09 77.25 77.00 76.75 76.43 47.31 34.16 22.07

Parameter	Value
1 Title	ttd-21-216-C
2 Origin	Bruker BioSpin GmbH
3 Solvent	CDCl3
4 Temperature	299.2
5 Number of Scans	12
6 Acquisition Time	1.1010
7 Acquisition Date	2020-11-03T15:22:11
8 Spectrometer Frequency	125.77
9 Spectral Width	29761.9

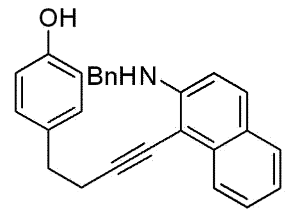

1s

^{13}C NMR of compound 1s

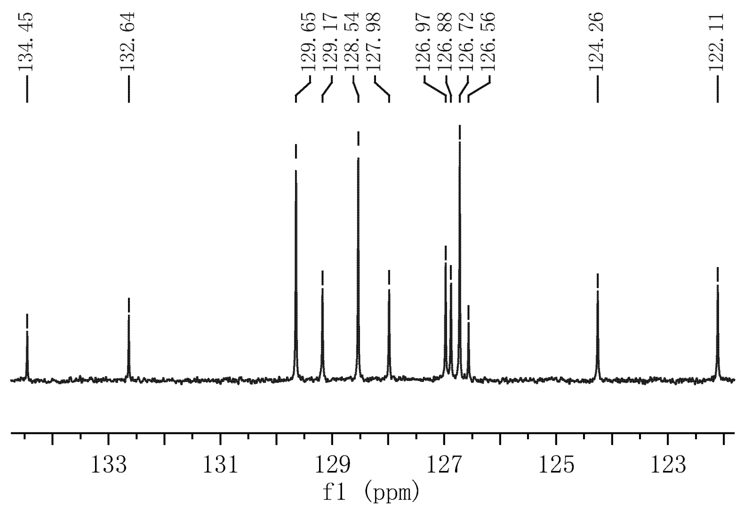

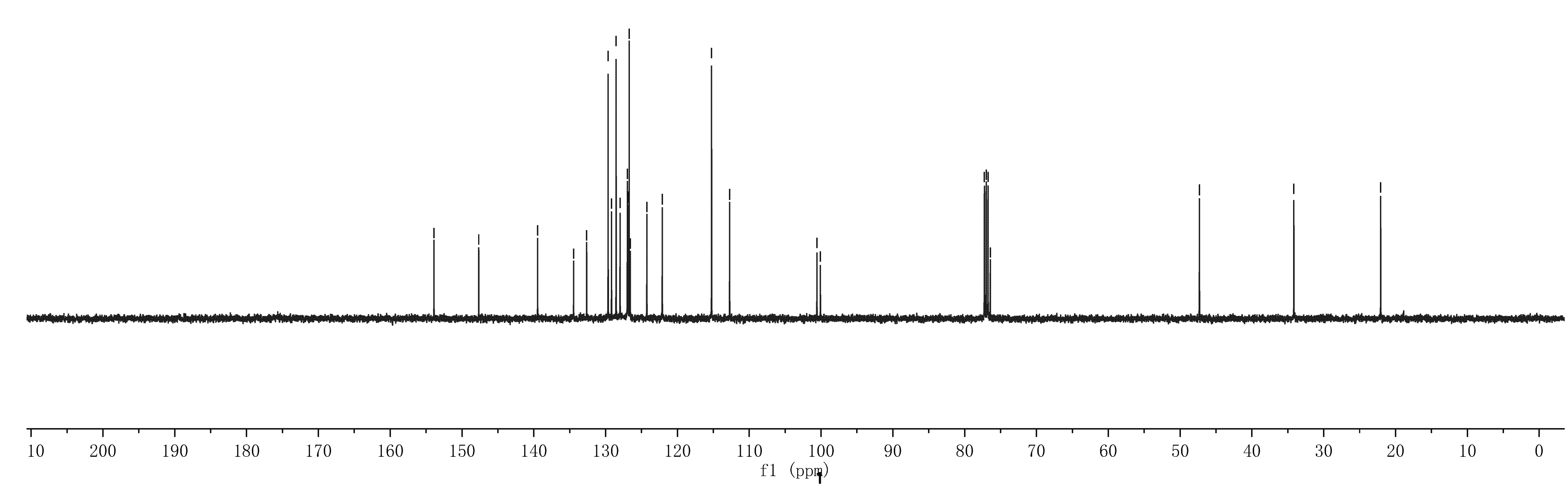

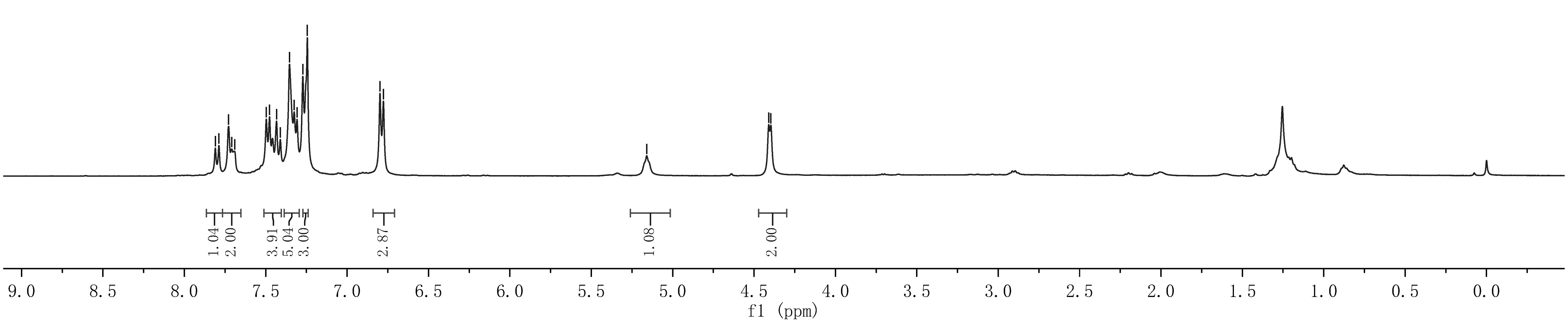

Parameter	Value
1 Title	ttd-21-71
2 Origin	Bruker BioSpin GmbH
3 Solvent	CDCl3
4 Temperature	298.0
5 Number of Scans	6
6 Acquisition Time	4.0894
7 Acquisition Date	2020-09-17T16:33:57
8 Spectrometer Frequency	400.13
9 Spectral Width	8012.8

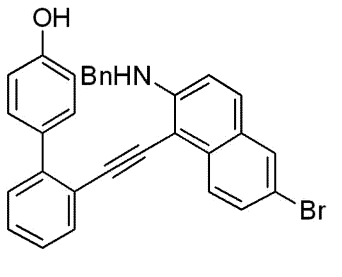

1t

^{1}H NMR of compound 1t

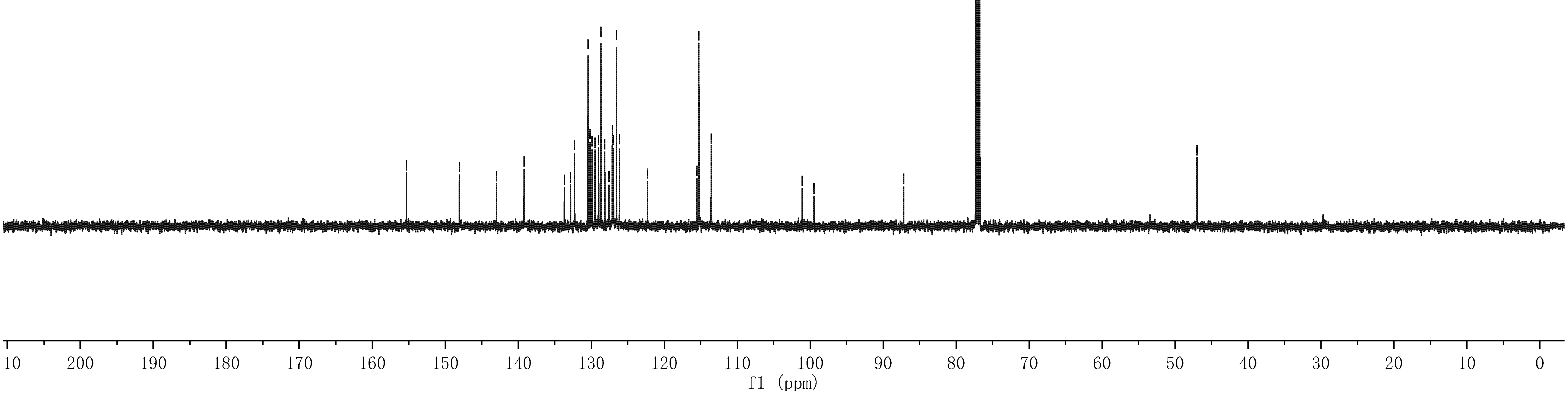

Parameter	Value
1 Title	ttd-21-71-C
2 Origin	Bruker BioSpin GmbH
3 Solvent	CDCl3
4 Temperature	298.8
5 Number of Scans	39
6 Acquisition Time	1.1010
7 Acquisition Date	2020-09-16T11:07:59
8 Spectrometer Frequency	125.77
9 Spectral Width	29761.9

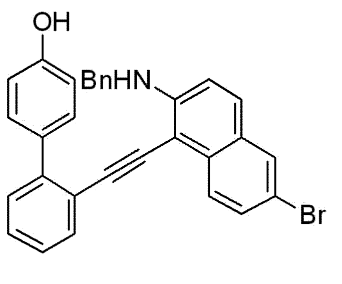

1t

^{13}C NMR of compound 1t

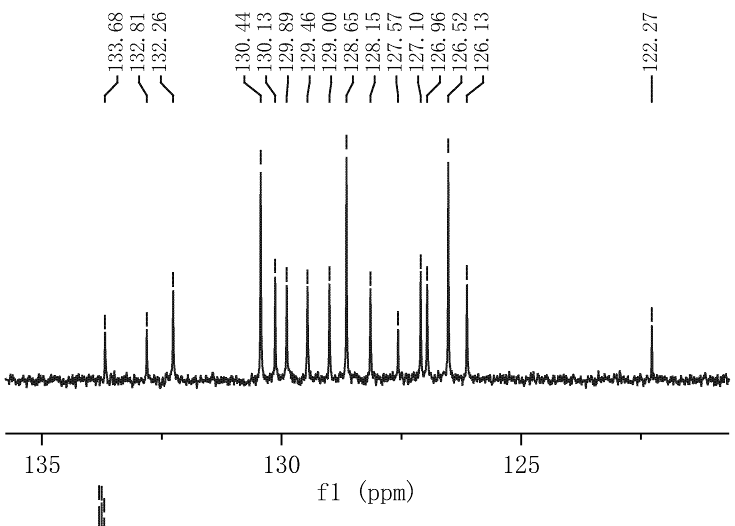

Parameter	Value
1 Title	ttd-21-90
2 Origin	Bruker BioSpin GmbH
3 Solvent	CDCl3
4 Temperature	298.0
5 Number of Scans	5
6 Acquisition Time	4.0894
7 Acquisition Date	2020-09-16T17:12:33
8 Spectrometer Frequency	400.13
9 Spectral Width	8012.8

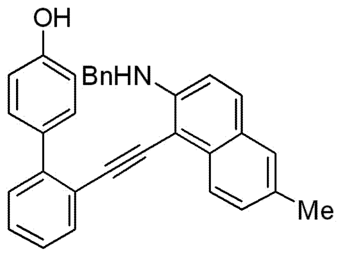

1u

1H NMR of compound 1u

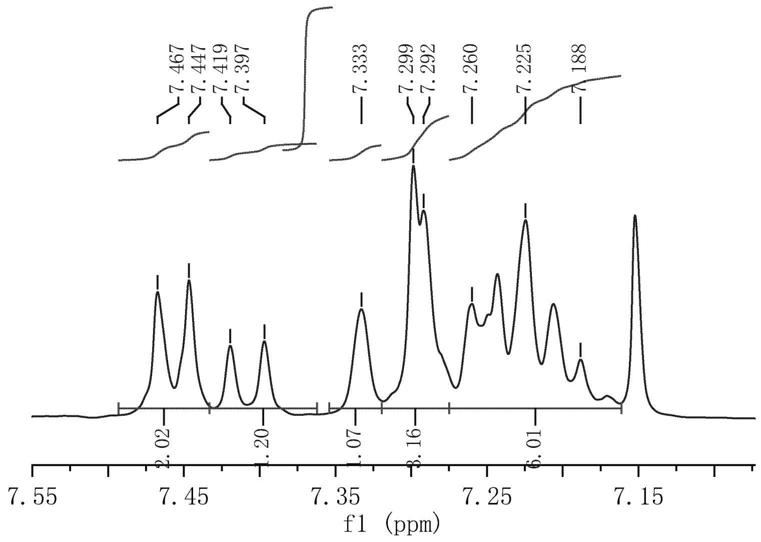

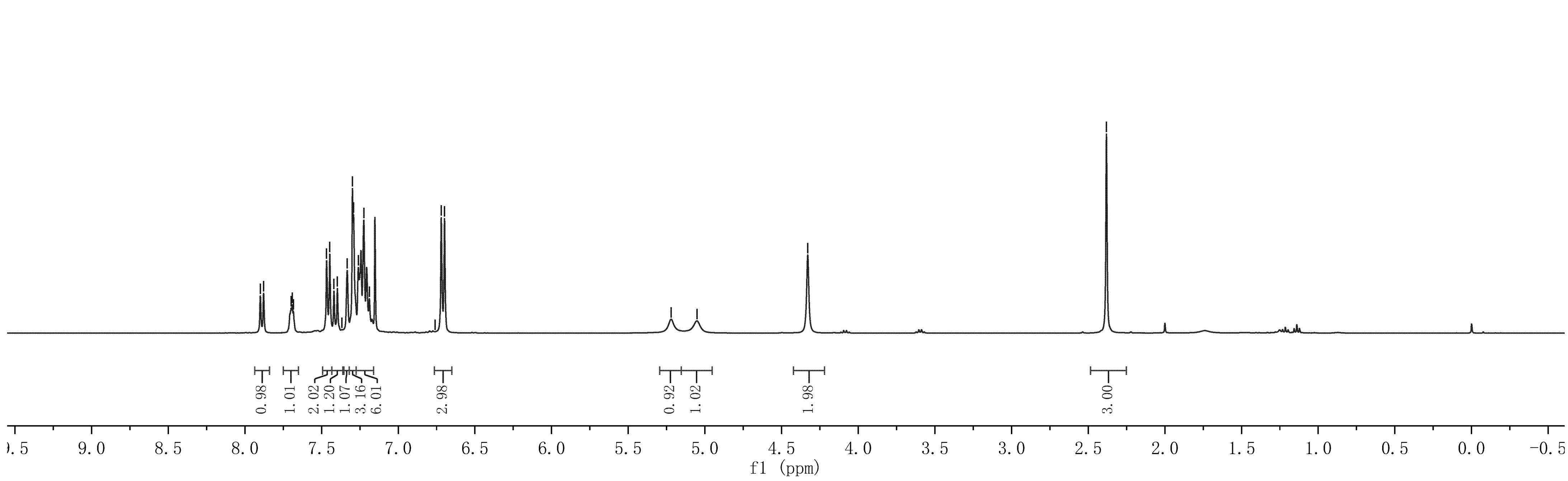

Parameter	Value
1 Title	ttd-21-90-C
2 Origin	Bruker BioSpin GmbH
3 Solvent	CDCl3
4 Temperature	300.0
5 Number of Scans	12
6 Acquisition Time	1.3631
7 Acquisition Date	2020-09-16T17:13:41
8 Spectrometer Frequency	100.61
9 Spectral Width	24038.5

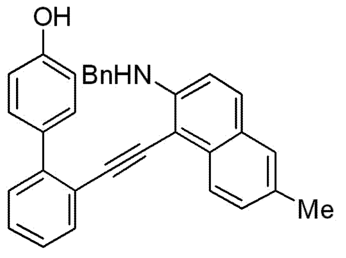

1u

13C NMR of compound 1u

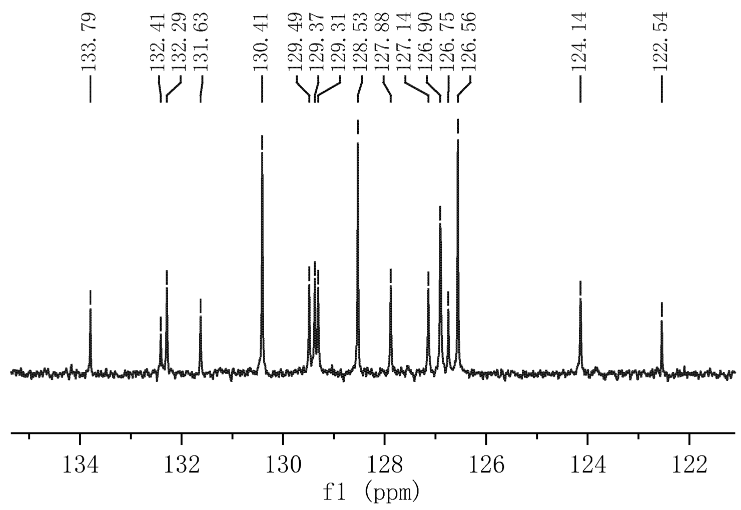

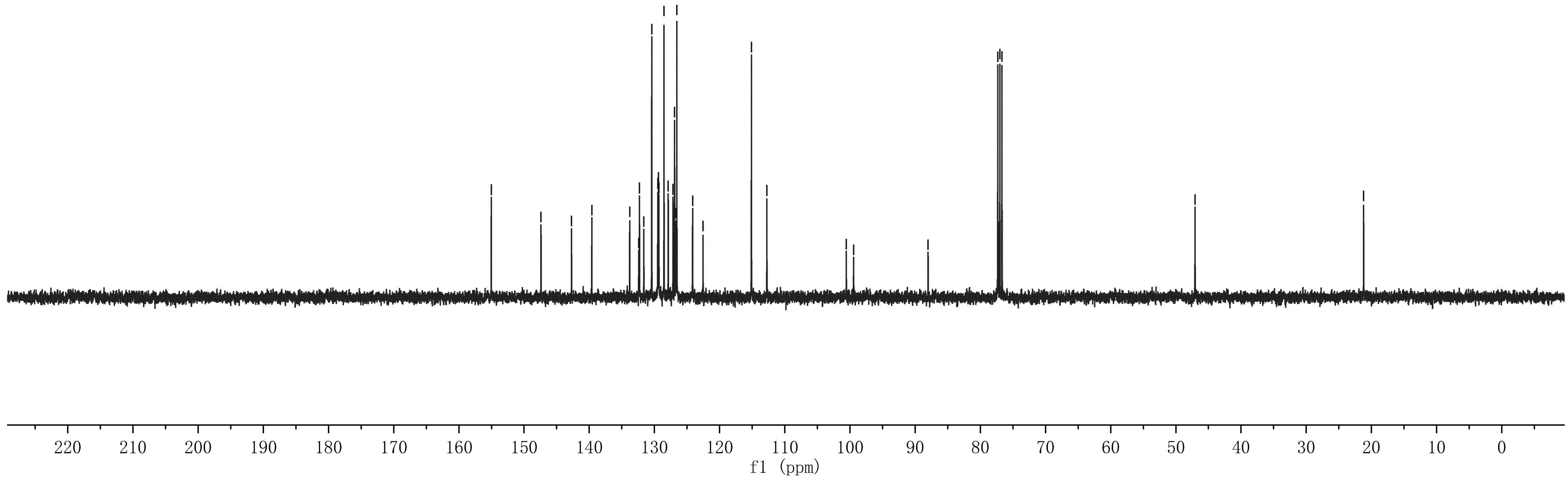

Parameter	Value
1 Title	shz-1-156-C
2 Origin	Bruker BioSpin GmbH
3 Solvent	CDCl3
4 Temperature	299.5
5 Number of Scans	32
6 Acquisition Time	1.1010
7 Acquisition Date	2020-11-07T14:55:06
8 Spectrometer Frequency	125.77
9 Spectral Width	29761.9

1v

^{1}H NMR of compound 1v

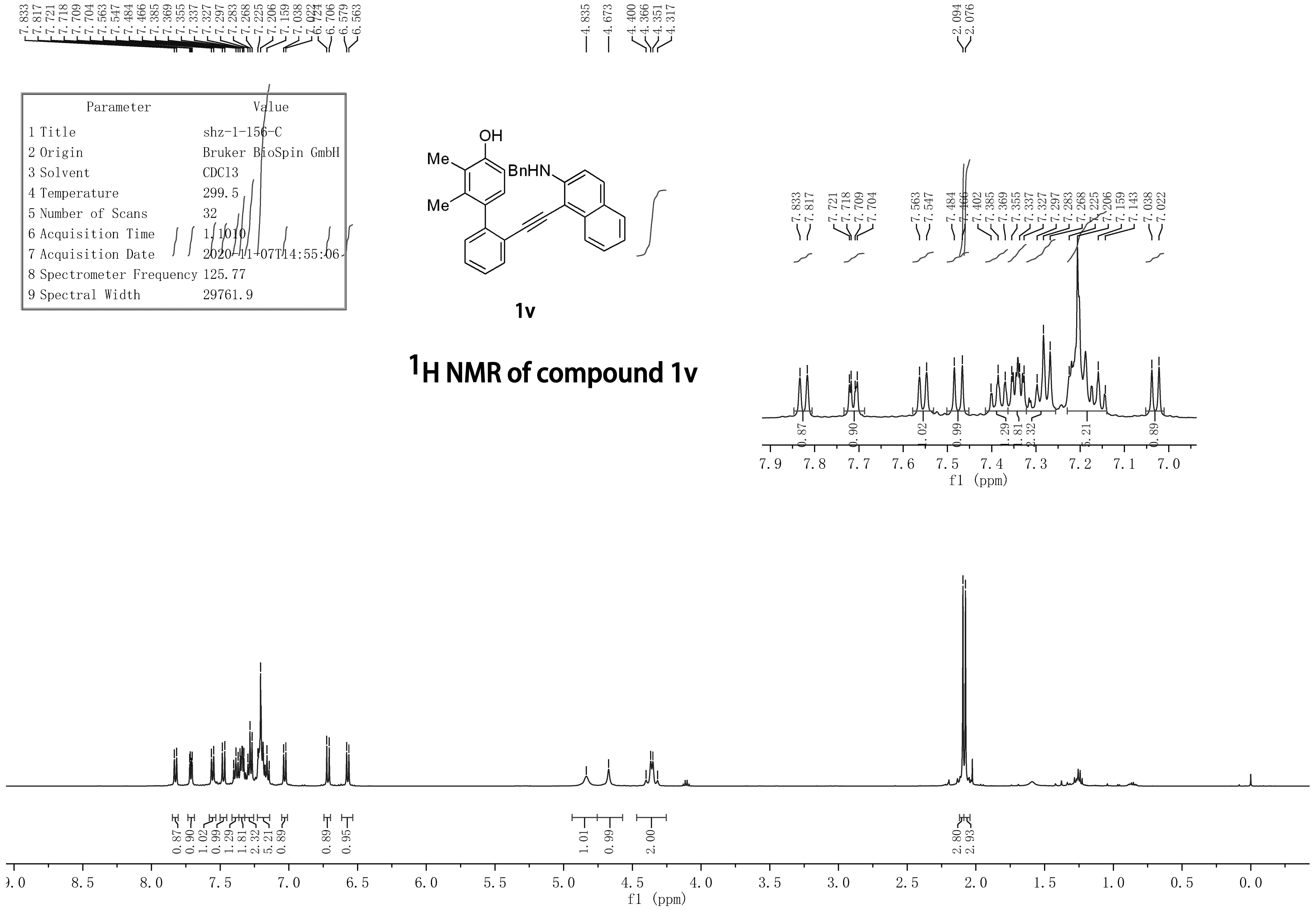

Parameter	Value
1 Title	shz-1-156
2 Origin	Bruker BioSpin GmbH
3 Solvent	CDCl3
4 Temperature	299.2
5 Number of Scans	11
6 Acquisition Time	3.1719
7 Acquisition Date	2020-11-07T14:52:24
8 Spectrometer Frequency	500.17
9 Spectral Width	10330.6

1v

^{13}C NMR of compound 1v

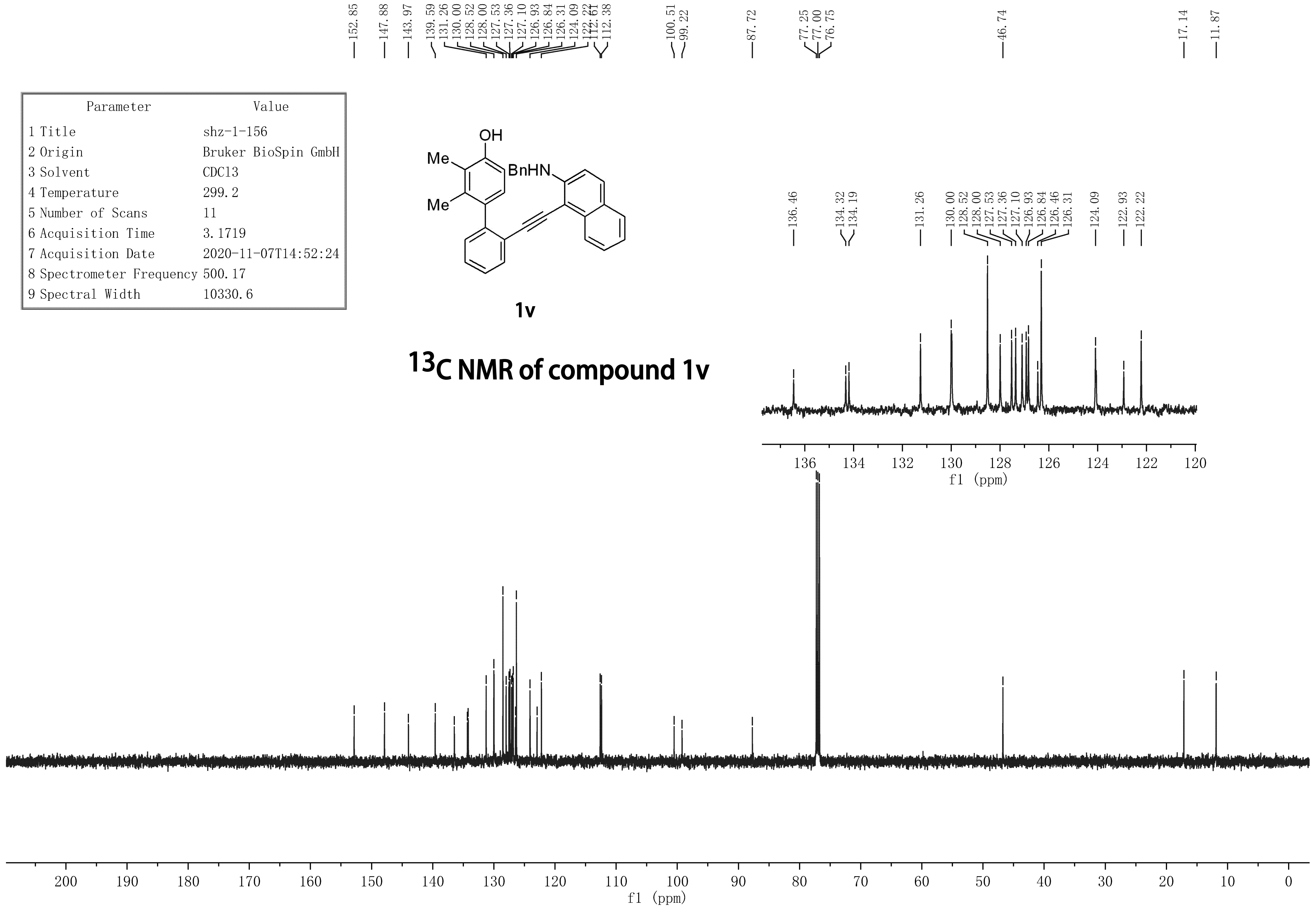

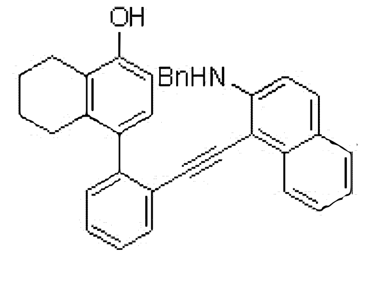

1w

^{1}H NMR of compound 1w

Parameter	Value
1 Title	shz-1-152
2 Origin	Bruker BioSpin GmbH
3 Solvent	CDCl3
4 Temperature	298.9
5 Number of Scans	12
6 Acquisition Time	3.1719
7 Acquisition Date	2020-11-07T14:59:29
8 Spectrometer Frequency	500.17
9 Spectral Width	10330.6

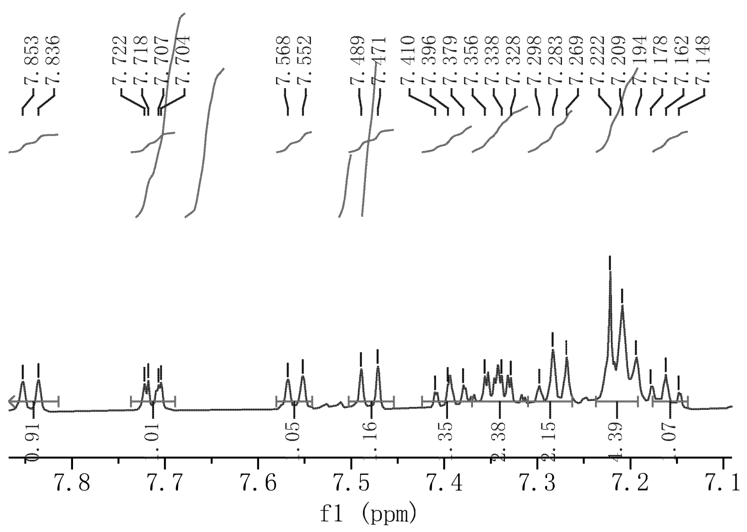

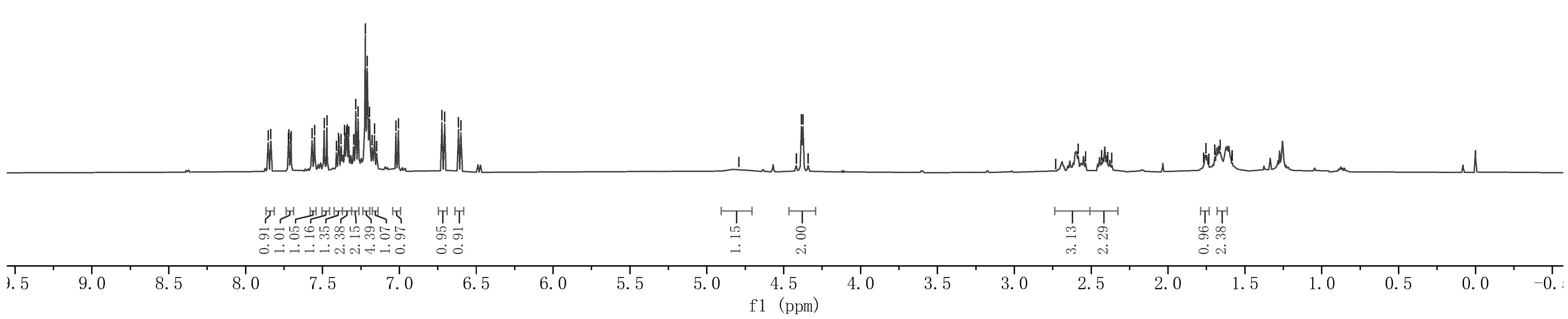

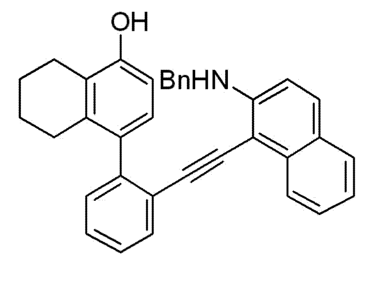

1w

^{13}C NMR of compound 1w

Parameter	Value
1 Title	shz-1-152-C
2 Origin	Bruker BioSpin GmbH
3 Solvent	CDCl3
4 Temperature	299.2
5 Number of Scans	45
6 Acquisition Time	1.1010
7 Acquisition Date	2020-11-06T15:22:15
8 Spectrometer Frequency	125.77
9 Spectral Width	29761.9

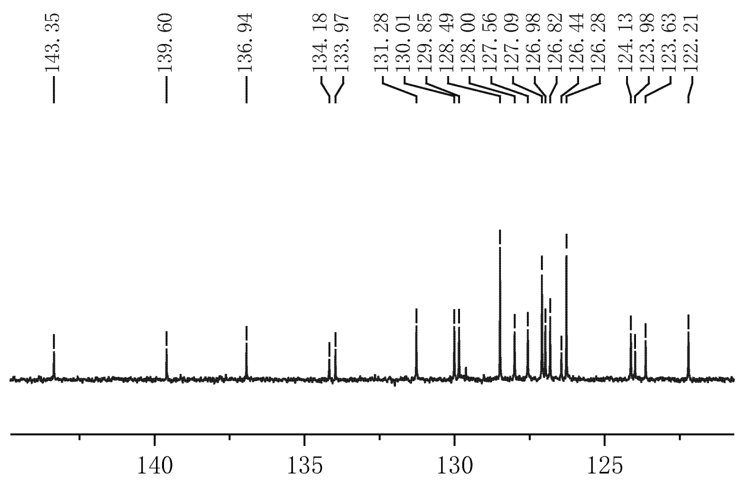

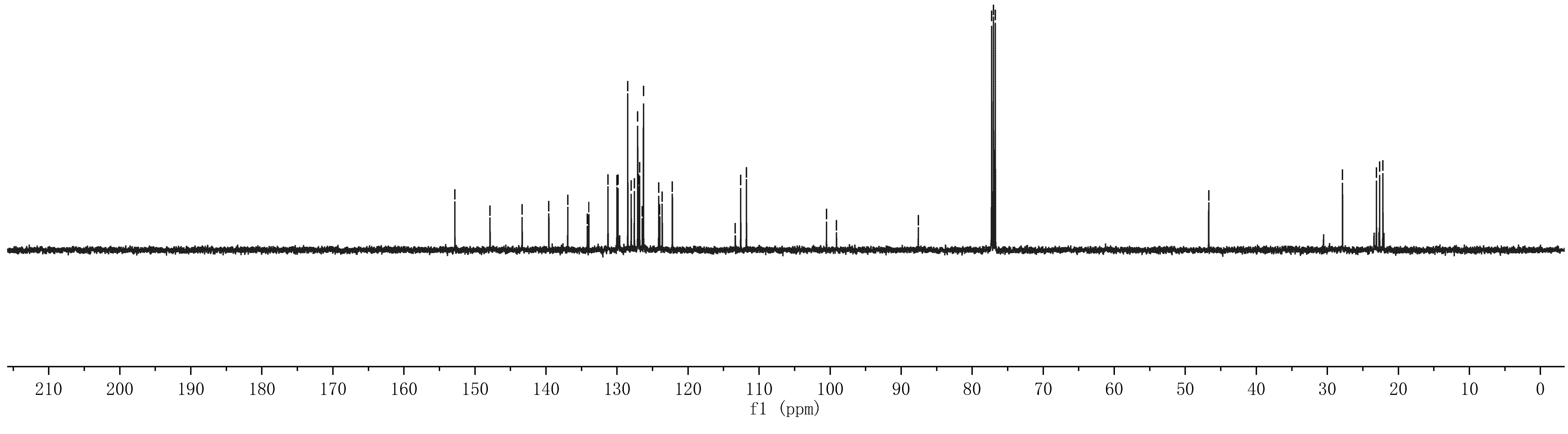

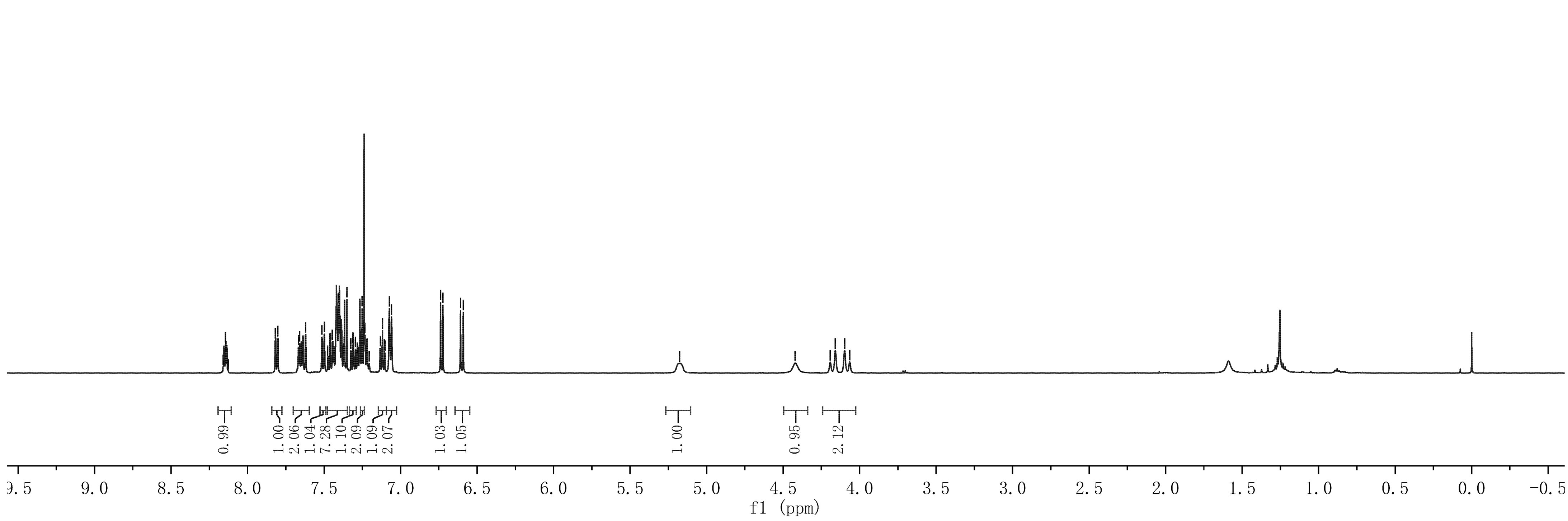

Parameter	Value
1 Title	ttd-24-39-pure
2 Origin	Bruker BioSpin GmbH
3 Solvent	CDCl3
4 Temperature	298.3
5 Number of Scans	11
6 Acquisition Time	3.1719
7 Acquisition Date	2021-07-24T08:25:01
8 Spectrometer Frequency	500.17
9 Spectral Width	10330.6

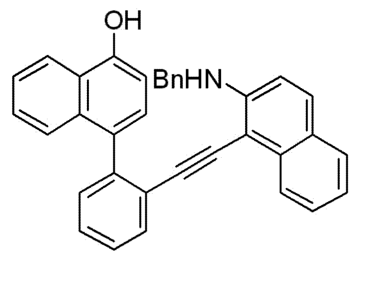

1x

^{1}H NMR of compound 1x

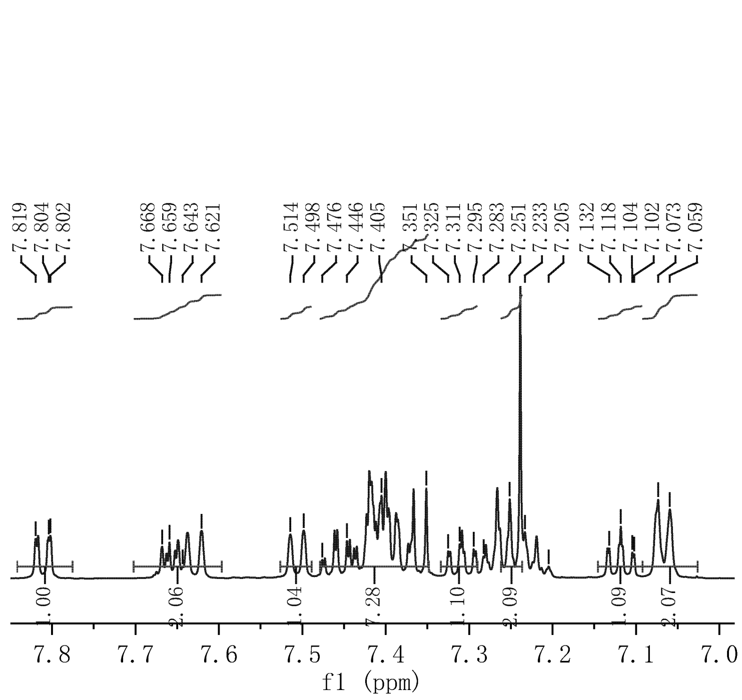

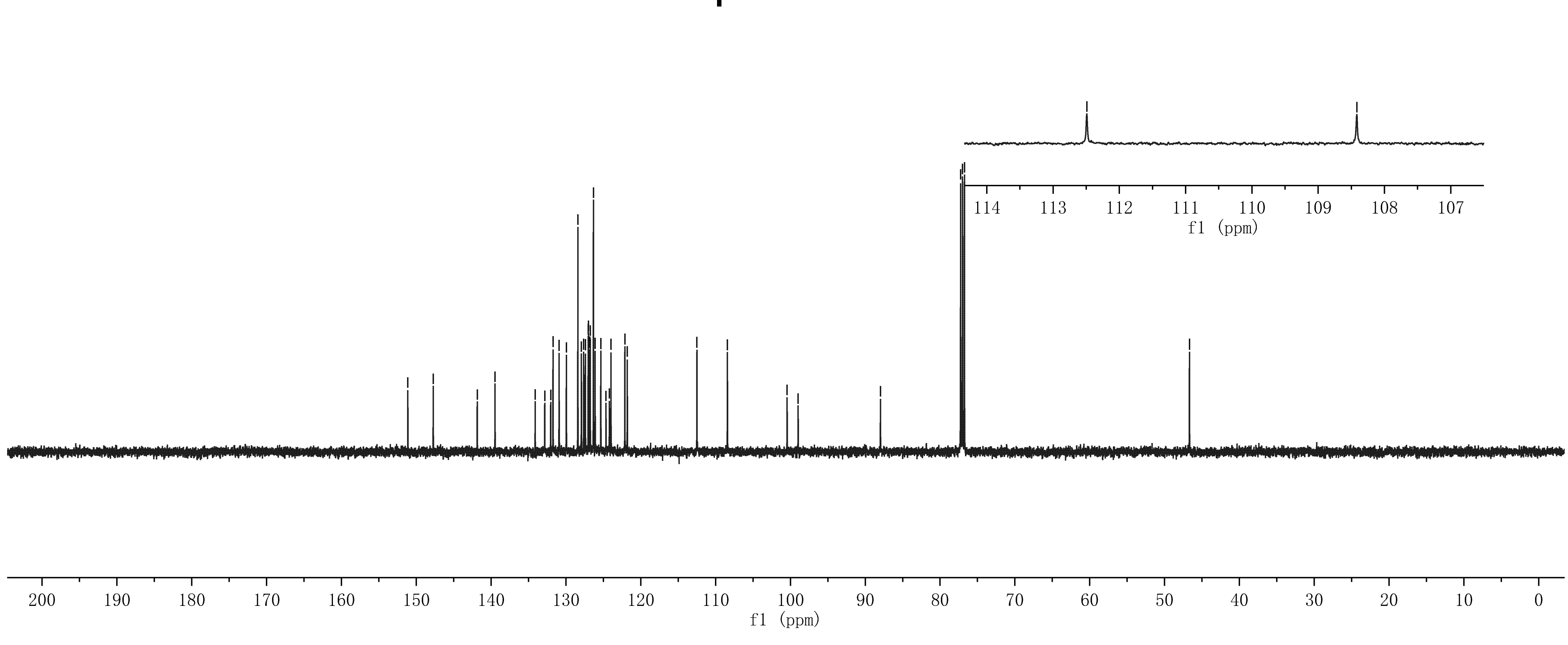

Parameter	Value
1 Title	ttd-24-39-C
2 Origin	Bruker BioSpin GmbH
3 Solvent	CDCl3
4 Temperature	297.2
5 Number of Scans	32
6 Acquisition Time	1.1010
7 Acquisition Date	2021-07-22T14:43:00
8 Spectrometer Frequency	125.77
9 Spectral Width	29761.9

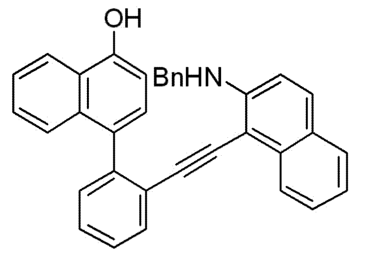

1x

^{13}C NMR of compound 1x

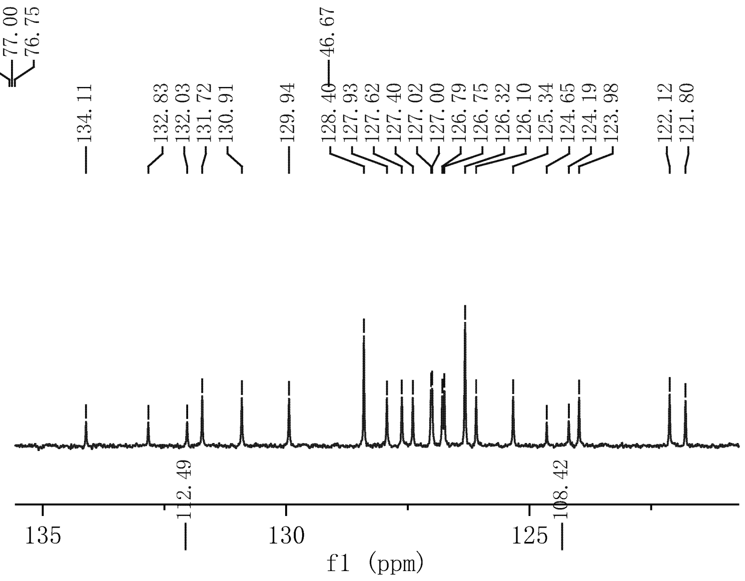

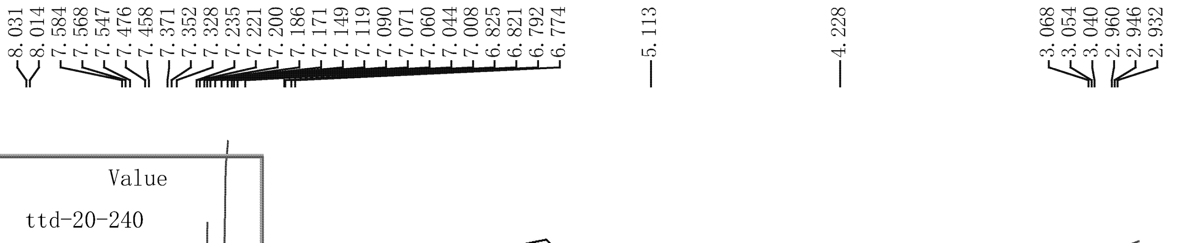

Parameter	Value
1 Title	ttd-20-240
2 Origin	Bruker BioSpin GmbH
3 Solvent	CDCl3
4 Temperature	298.7
5 Number of Scans	10
6 Acquisition Time	3.1719
7 Acquisition Date	2020-08-17T15:54:08
8 Spectrometer Frequency	500.17
9 Spectral Width	10330.6

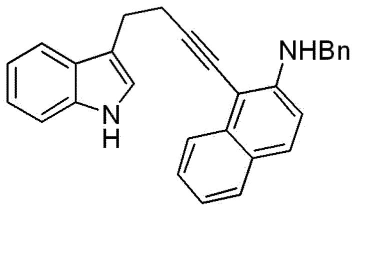

3a

^{1}H NMR of compound 3a

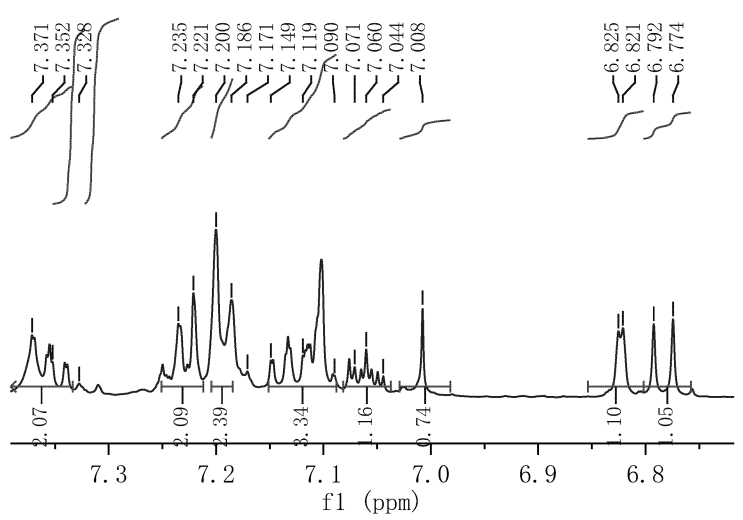

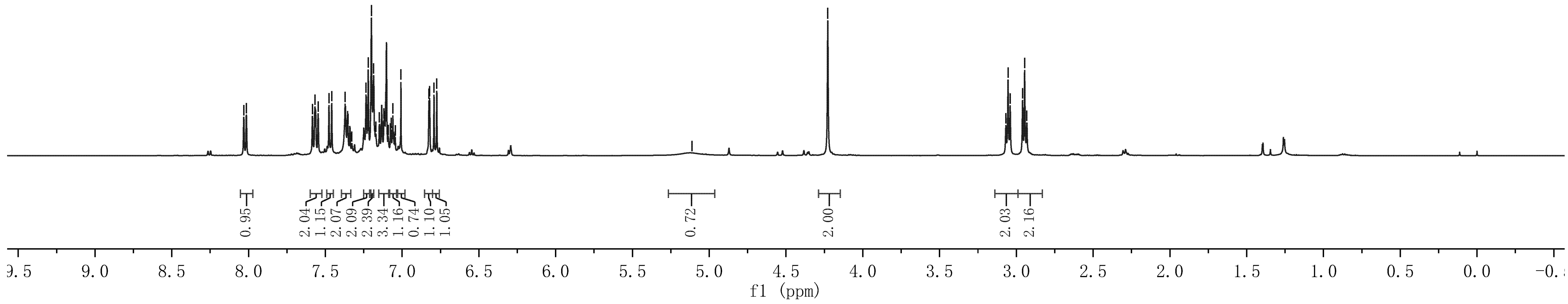

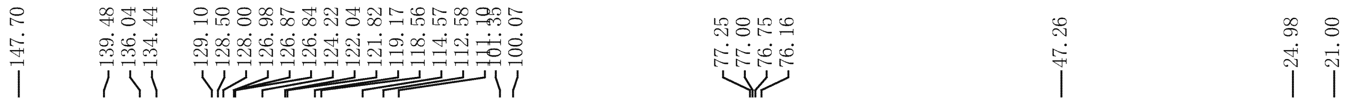

Parameter	Value
1 Title	ttd-20-240-C
2 Origin	Bruker BioSpin GmbH
3 Solvent	CDCl3
4 Temperature	299.1
5 Number of Scans	14
6 Acquisition Time	1.1010
7 Acquisition Date	2020-08-17T15:56:33
8 Spectrometer Frequency	125.77
9 Spectral Width	29761.9

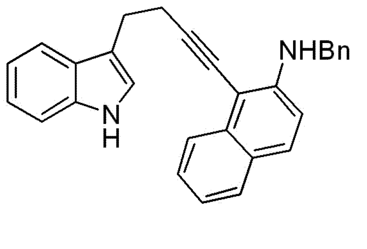

3a

^{13}C NMR of compound 3a

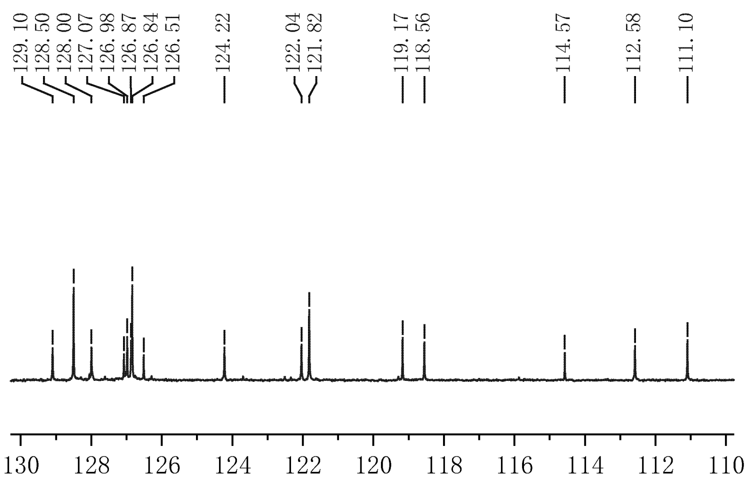

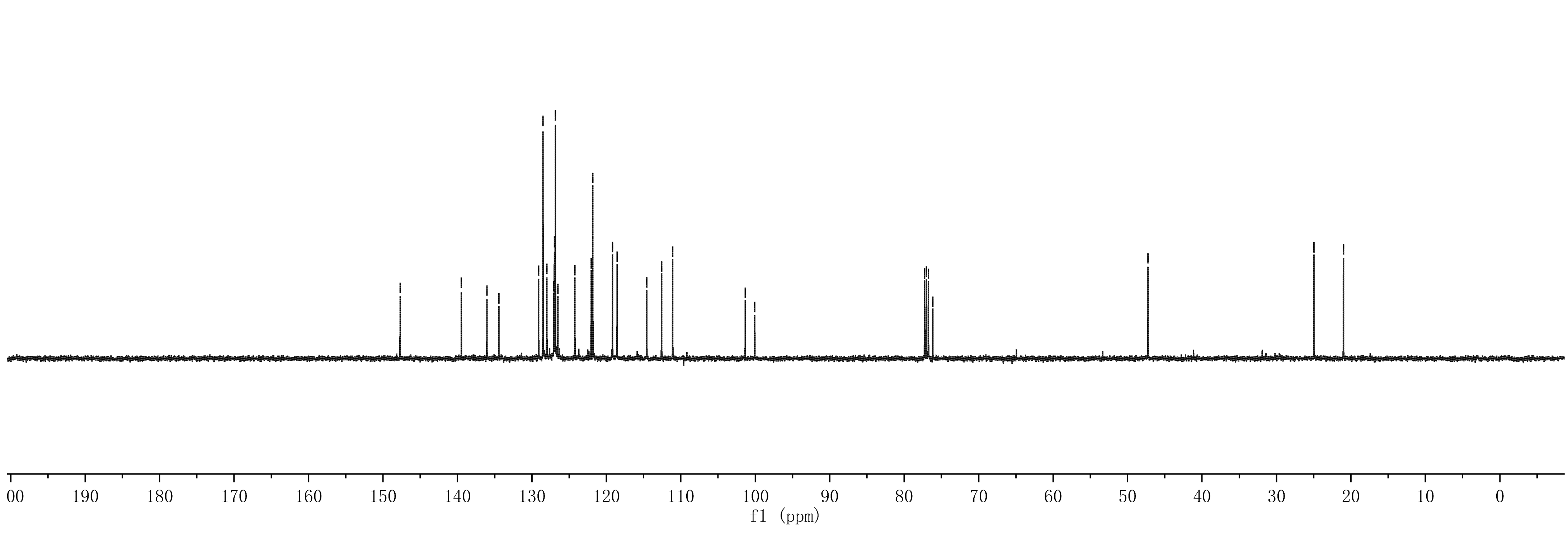

Parameter	Value
1 Title	ttd-22-173-400M
2 Origin	Bruker BioSpin GmbH
3 Solvent	CDCl3
4 Temperature	298.0
5 Number of Scans	8
6 Acquisition Time	4.0894
7 Acquisition Date	2021-01-15T08:46:41
8 Spectrometer Frequency	400.13
9 Spectral Width	8012.8

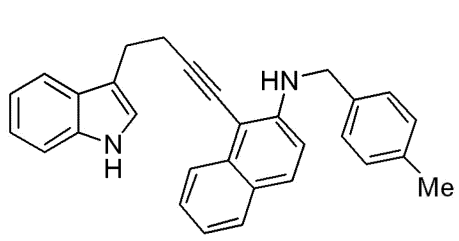

3b

^{1}H NMR of compound 3b

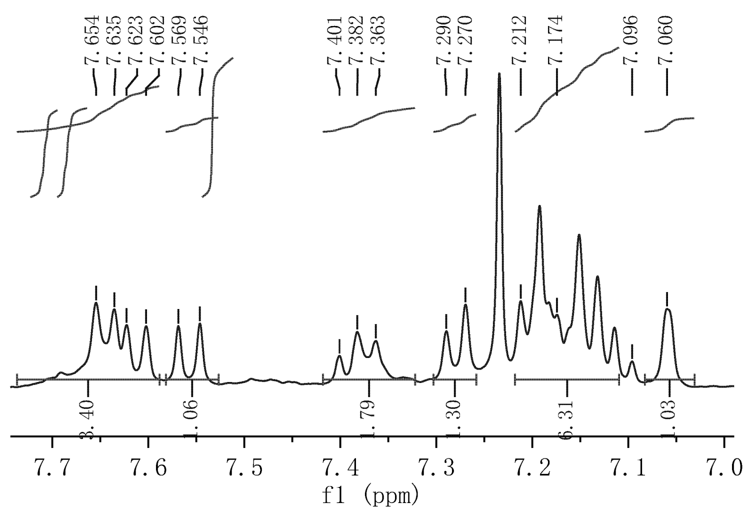

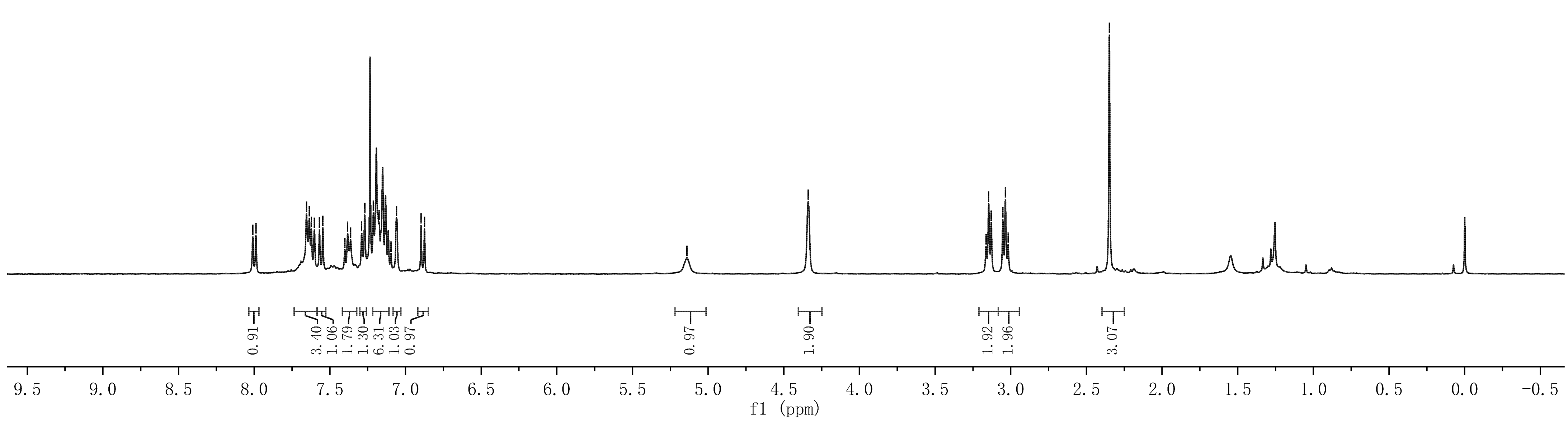

Parameter	Value
1 Title	ttd-22-173-C
2 Origin	Bruker BioSpin GmbH
3 Solvent	CDCl3
4 Temperature	297.0
5 Number of Scans	26
6 Acquisition Time	1.1010
7 Acquisition Date	2021-01-14T16:04:58
8 Spectrometer Frequency	125.77
9 Spectral Width	29761.9

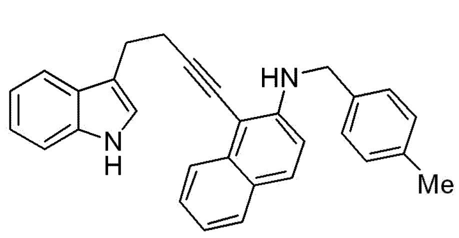

3b

^{13}C NMR of compound 3b

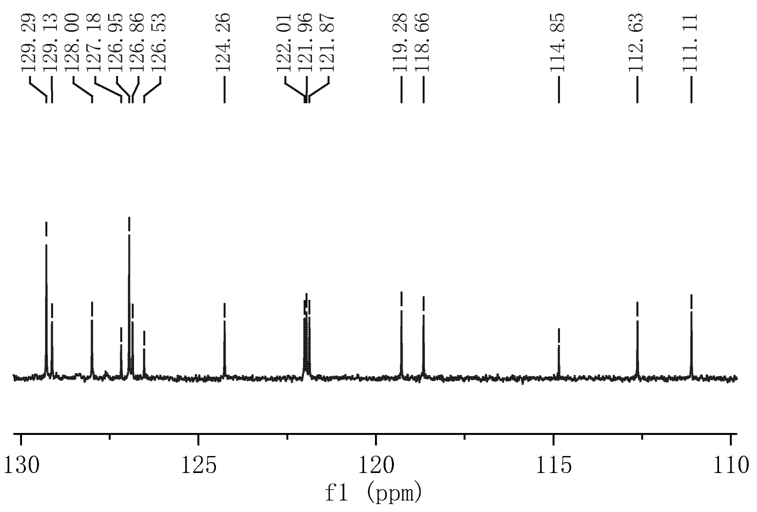

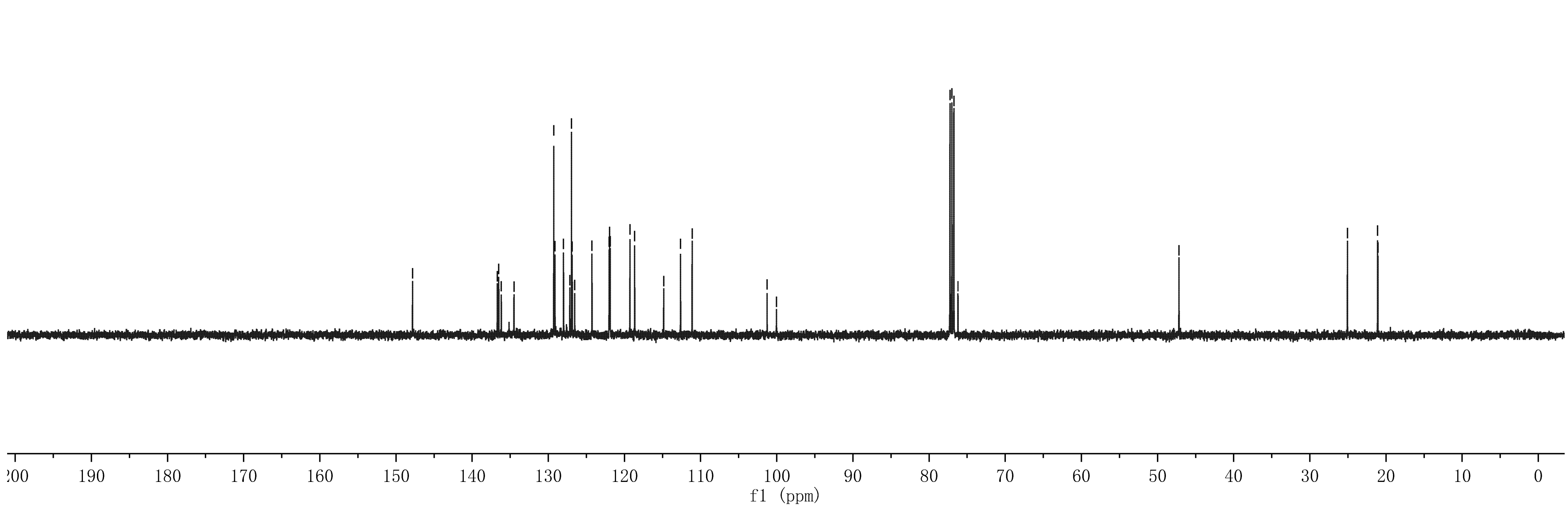

Parameter	Value
1 Title	ttd-22-191
2 Origin	Bruker BioSpin GmbH
3 Solvent	CDCl3
4 Temperature	298.2
5 Number of Scans	7
6 Acquisition Time	3.1719
7 Acquisition Date	2021-01-20T20:04:57
8 Spectrometer Frequency	500.17
9 Spectral Width	10330.6

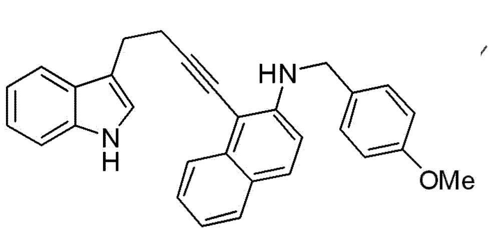

3c

^{1}H NMR of compound 3c

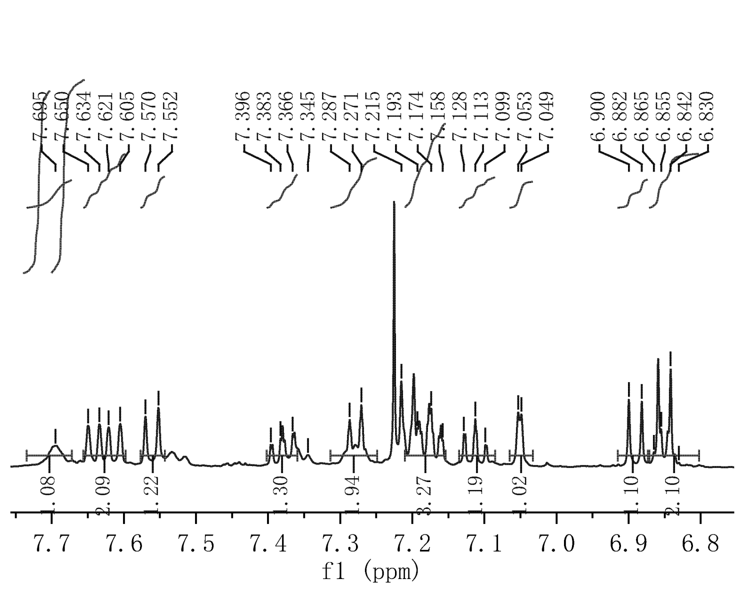

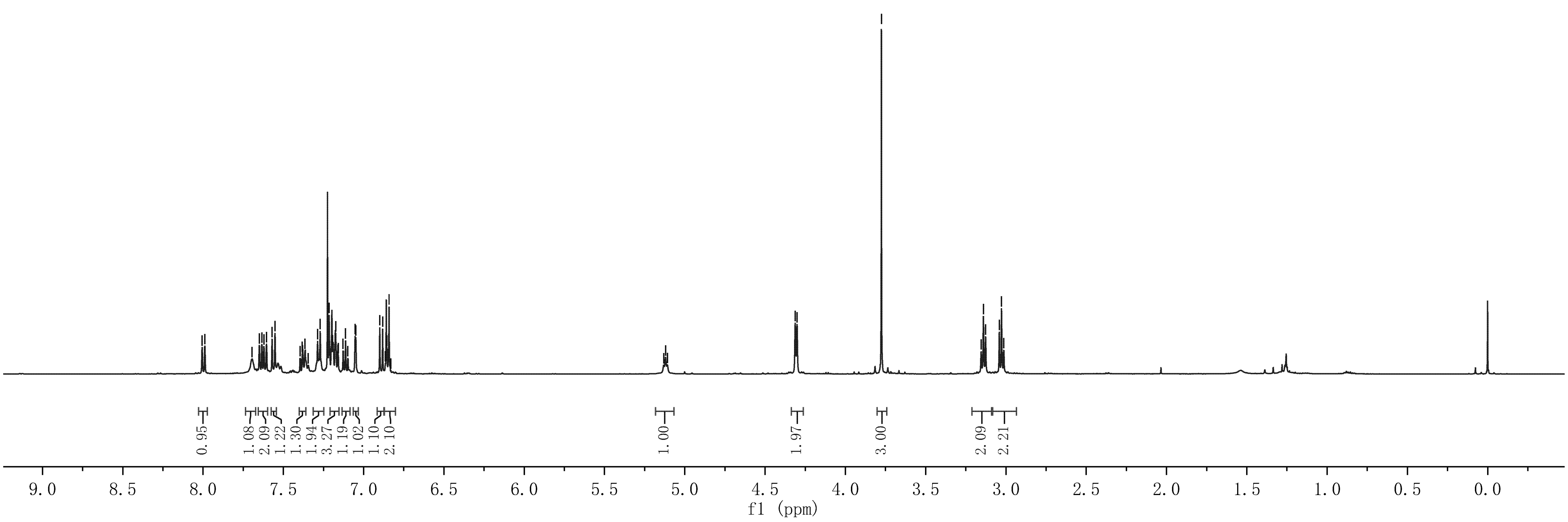

Parameter	Value
1 Title	ttd-22-191-C
2 Origin	Bruker BioSpin GmbH
3 Solvent	CDCl3
4 Temperature	298.6
5 Number of Scans	42
6 Acquisition Time	1.1010
7 Acquisition Date	2021-01-20T20:08:14
8 Spectrometer Frequency	125.77
9 Spectral Width	29761.9

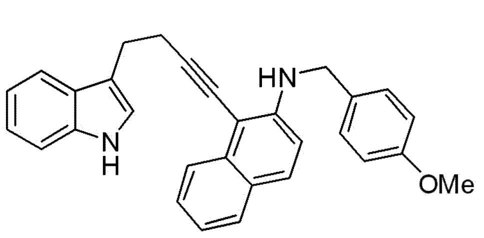

3c

^{13}C NMR of compound 3c

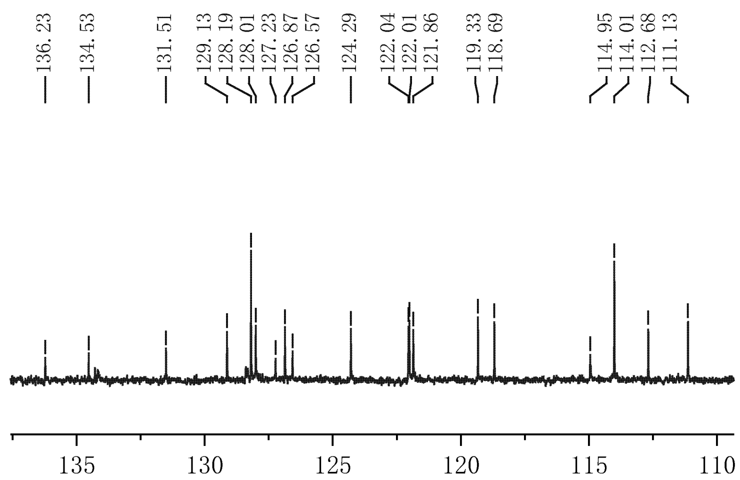

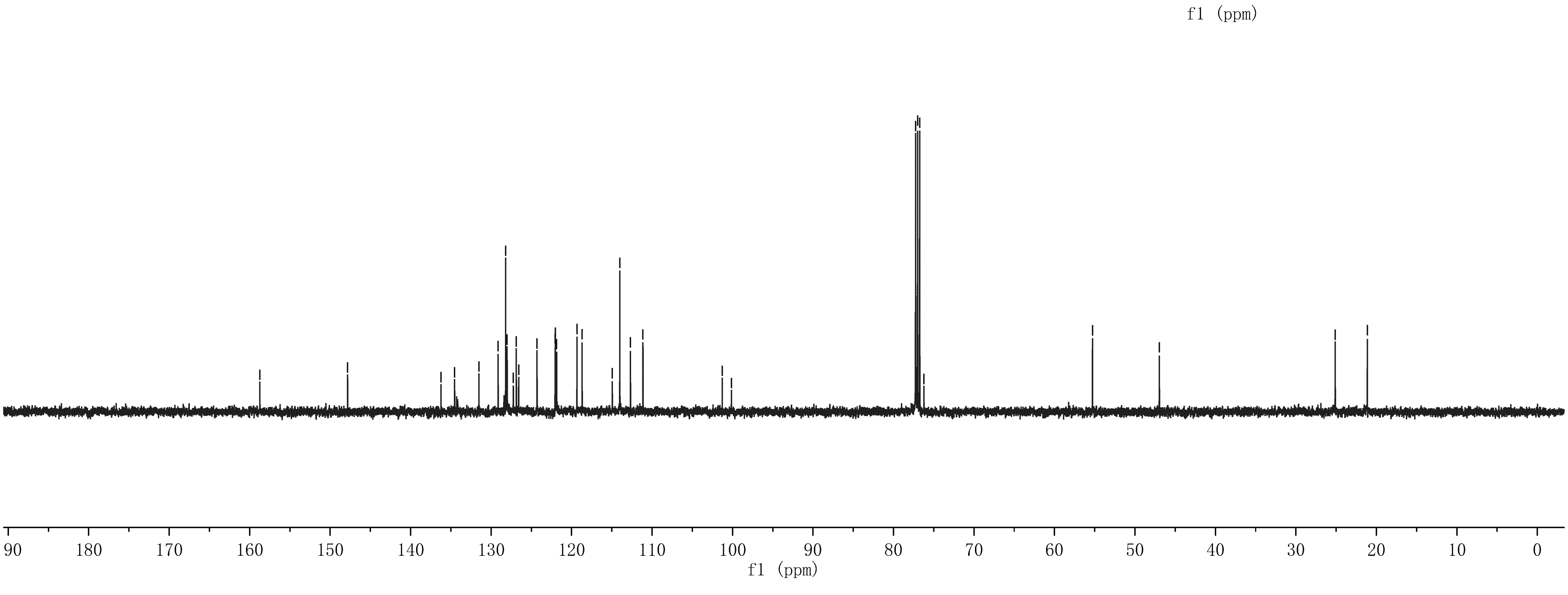

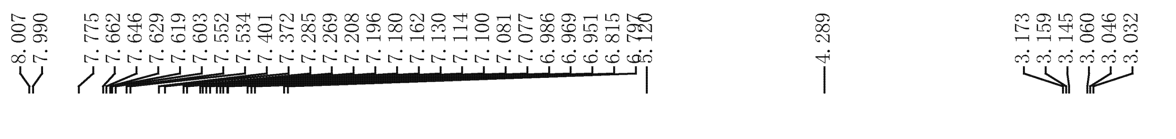

Parameter	Value
1 Title	ttd-22-236
2 Origin	Bruker BioSpin GmbH
3 Solvent	CDCl3
4 Temperature	298.2
5 Number of Scans	6
6 Acquisition Time	3.1719
7 Acquisition Date	2021-03-04T15:44:54
8 Spectrometer Frequency	500.17
9 Spectral Width	10330.6

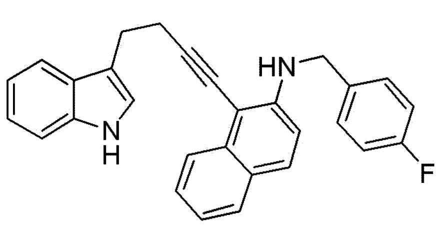

3d

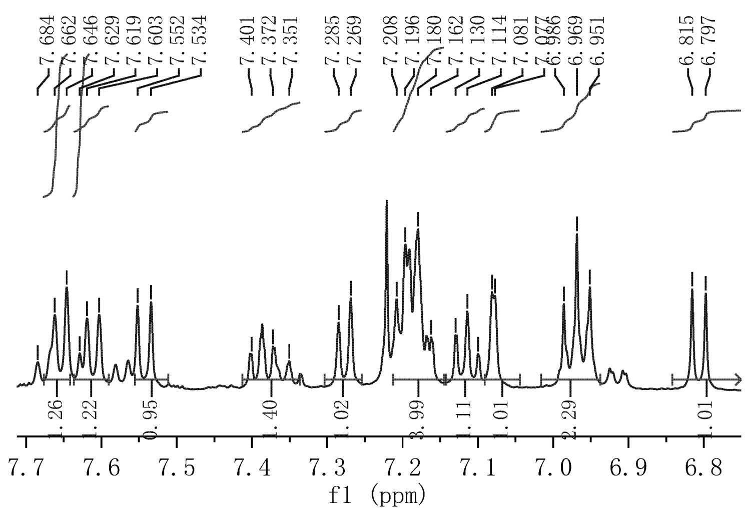

^{1}H NMR of compound 3d

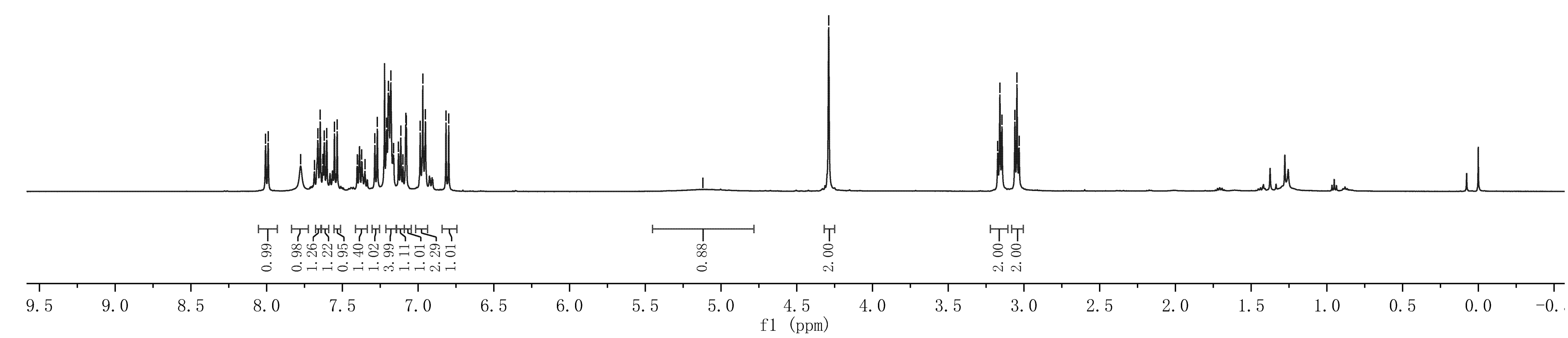

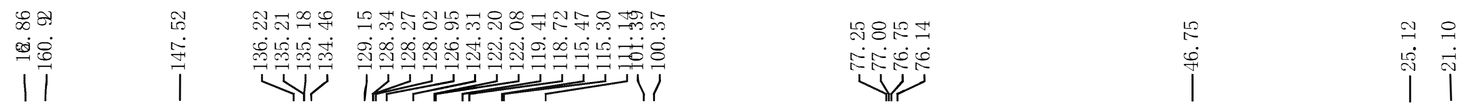

Parameter	Value
1 Title	ttd-22-236-C
2 Origin	Bruker BioSpin GmbH
3 Solvent	CDCl3
4 Temperature	298.7
5 Number of Scans	94
6 Acquisition Time	1.1010
7 Acquisition Date	2021-03-04T15:47:44
8 Spectrometer Frequency	125.77
9 Spectral Width	29761.9

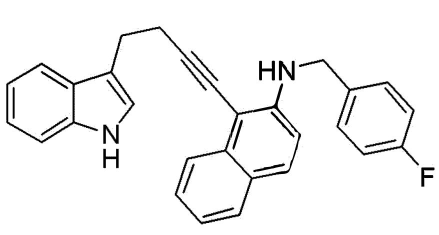

3d

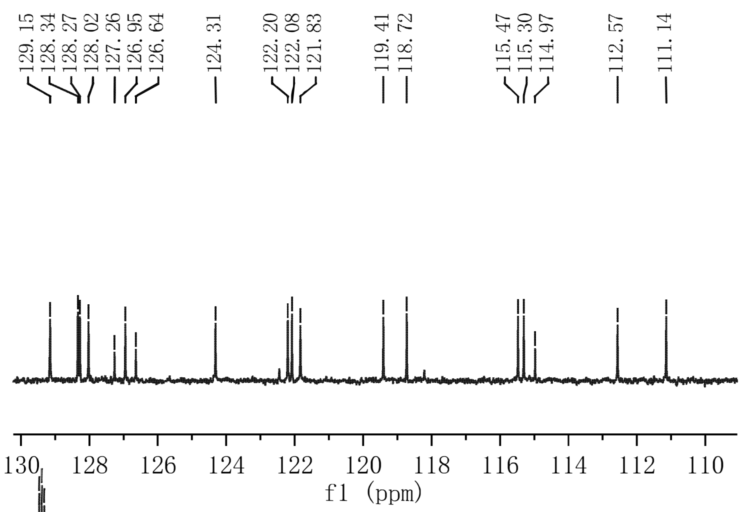

^{13}C NMR of compound 3d

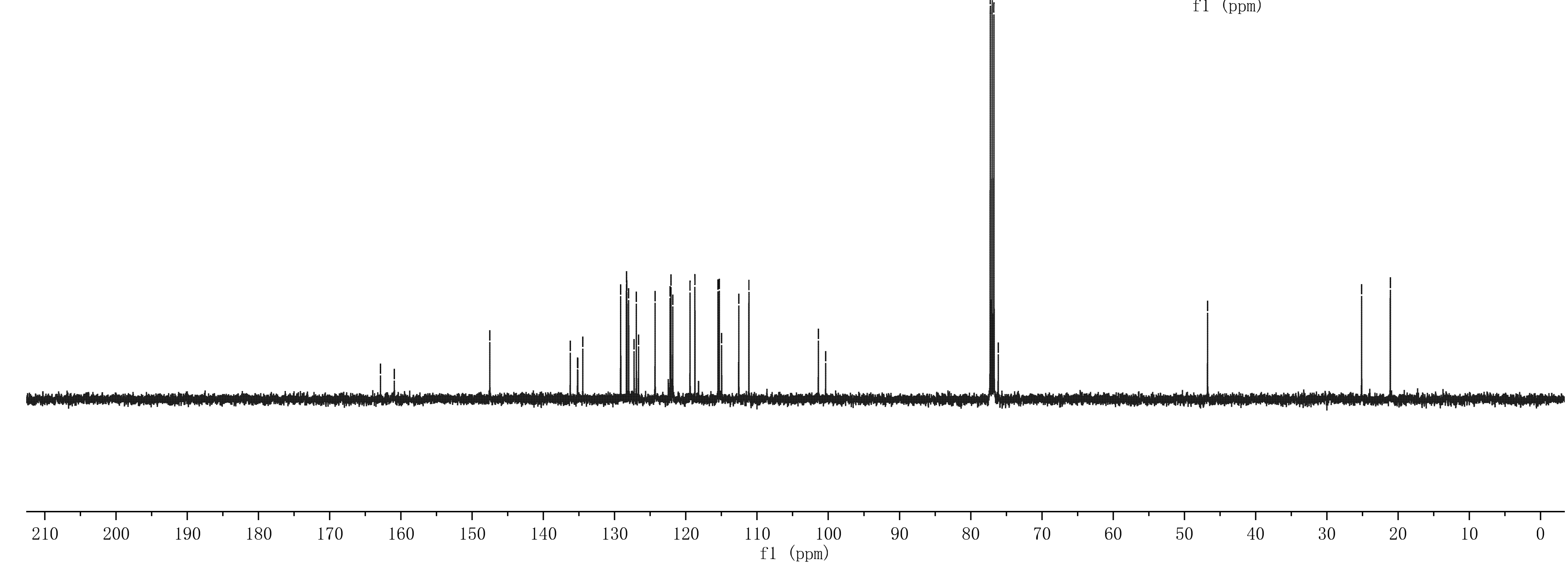

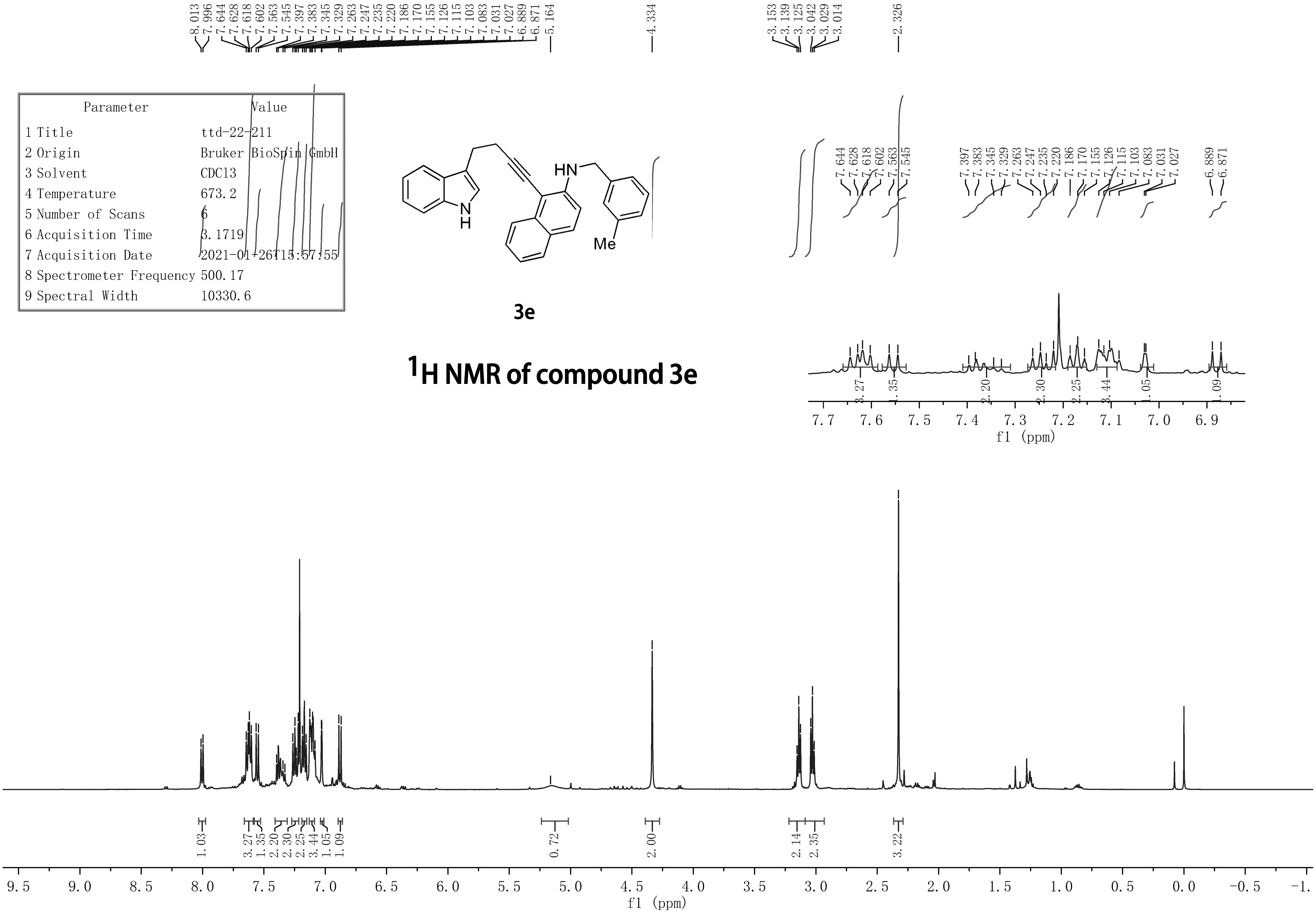
8.013
7.996
7.644
7.628
7.618
7.602
7.563
7.545
7.397
7.383
7.345
7.329
7.263
7.247
7.235
7.220
7.186
7.170
7.155
7.126
7.115
7.103
7.083
7.031
7.027
6.889
6.871
5.164
4.334
3.153
3.139
3.125
3.042
3.029
3.014
2.326
Parameter Value
1 Title ttd-22-211
2 Origin Bruker BioSpin GmbH
3 Solvent CDCl3
4 Temperature 673.2
5 Number of Scans 6
6 Acquisition Time 3.1719
7 Acquisition Date 2021-01-26T15:57:55
8 Spectrometer Frequency 500.17
9 Spectral Width 10330.6
HN
N
H
Me
3e
1H NMR of compound 3e
f1 (ppm)

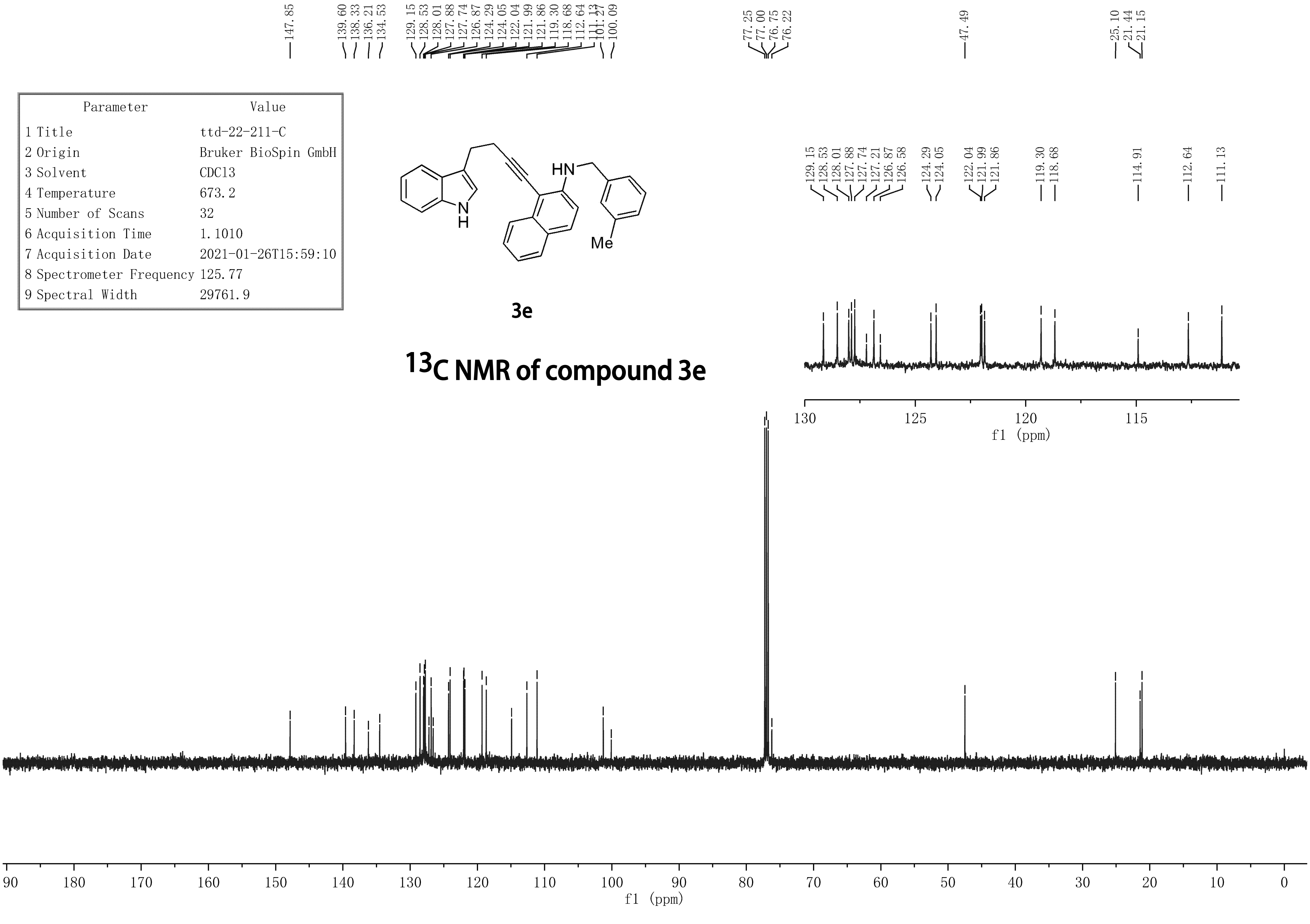
147.85
139.60
138.33
136.21
134.53
129.15
128.53
128.01
127.88
127.74
126.87
124.29
124.05
122.04
121.99
121.86
119.30
118.68
112.64
111.13
101.27
100.09
77.25
77.00
76.75
76.22
47.49
25.10
21.44
21.15
Parameter Value
1 Title ttd-22-211-C
2 Origin Bruker BioSpin GmbH
3 Solvent CDCl3
4 Temperature 673.2
5 Number of Scans 32
6 Acquisition Time 1.1010
7 Acquisition Date 2021-01-26T15:59:10
8 Spectrometer Frequency 125.77
9 Spectral Width 29761.9
HN
N
H
Me
3e
13C NMR of compound 3e
f1 (ppm)

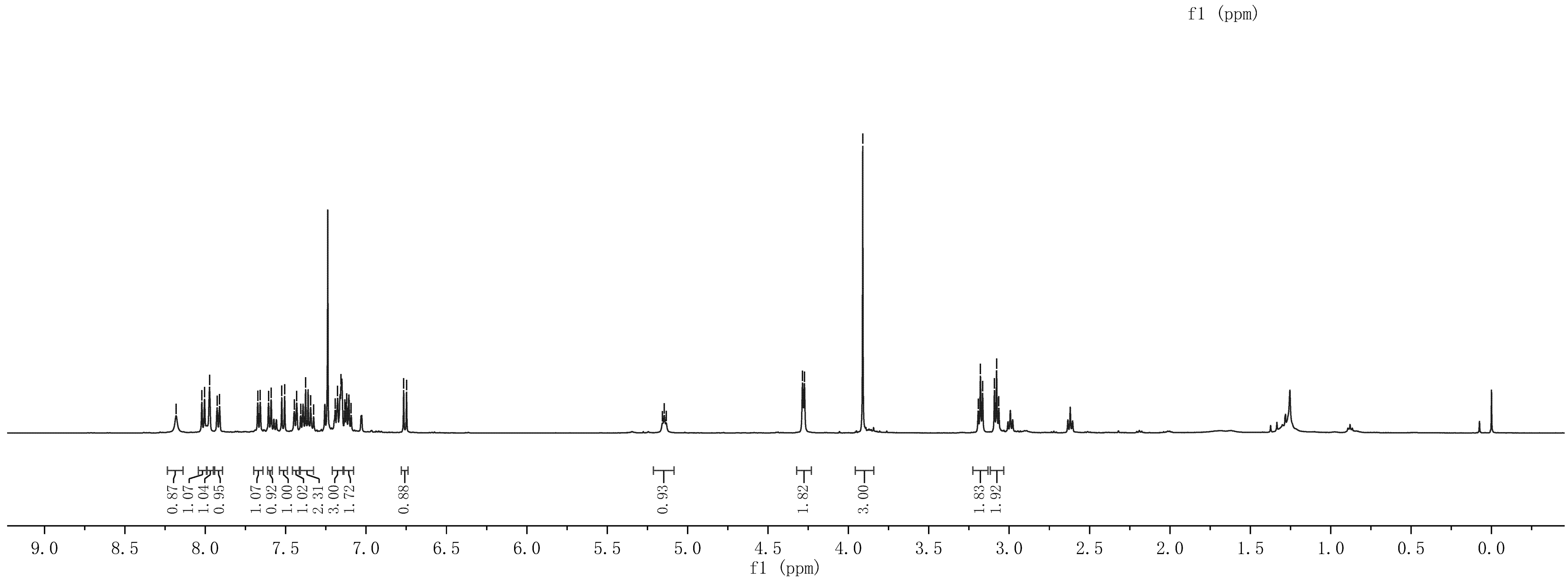

Parameter	Value
1 Title	ttd-22-164
2 Origin	Bruker BioSpin GmbH
3 Solvent	CDCl3
4 Temperature	295.6
5 Number of Scans	11
6 Acquisition Time	3.1719
7 Acquisition Date	2021-01-11T15:43:39
8 Spectrometer Frequency	500.17
9 Spectral Width	10330.6

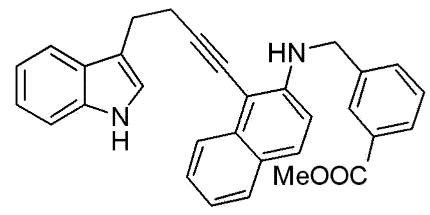

3f

^{1}H NMR of compound 3f

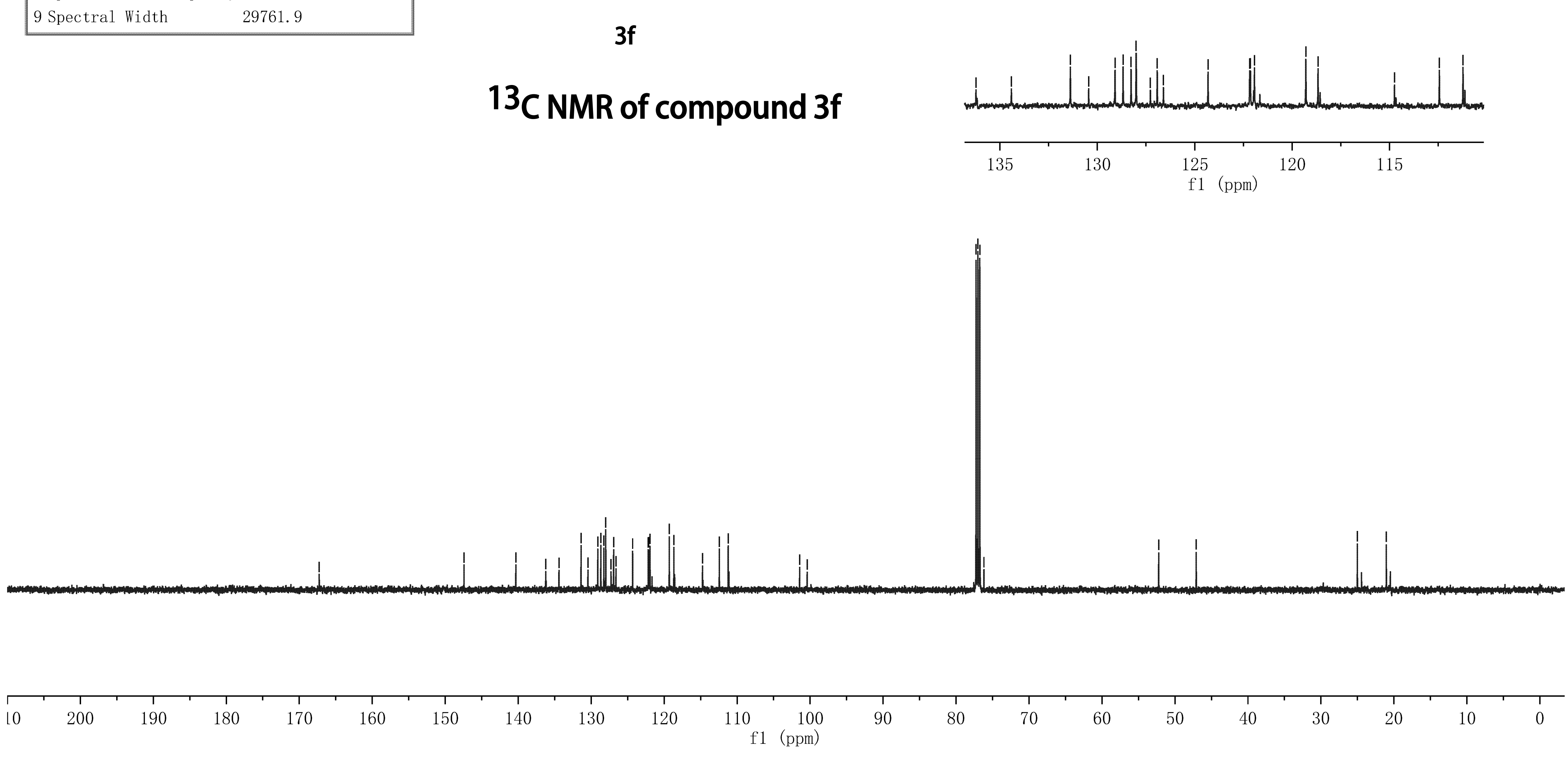

Parameter	Value
1 Title	ttd-22-164-C
2 Origin	Bruker BioSpin GmbH
3 Solvent	CDCl3
4 Temperature	296.0
5 Number of Scans	205
6 Acquisition Time	1.1010
7 Acquisition Date	2021-01-11T15:46:36
8 Spectrometer Frequency	125.77
9 Spectral Width	29761.9

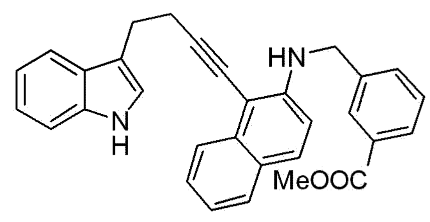

3f

^{13}C NMR of compound 3f

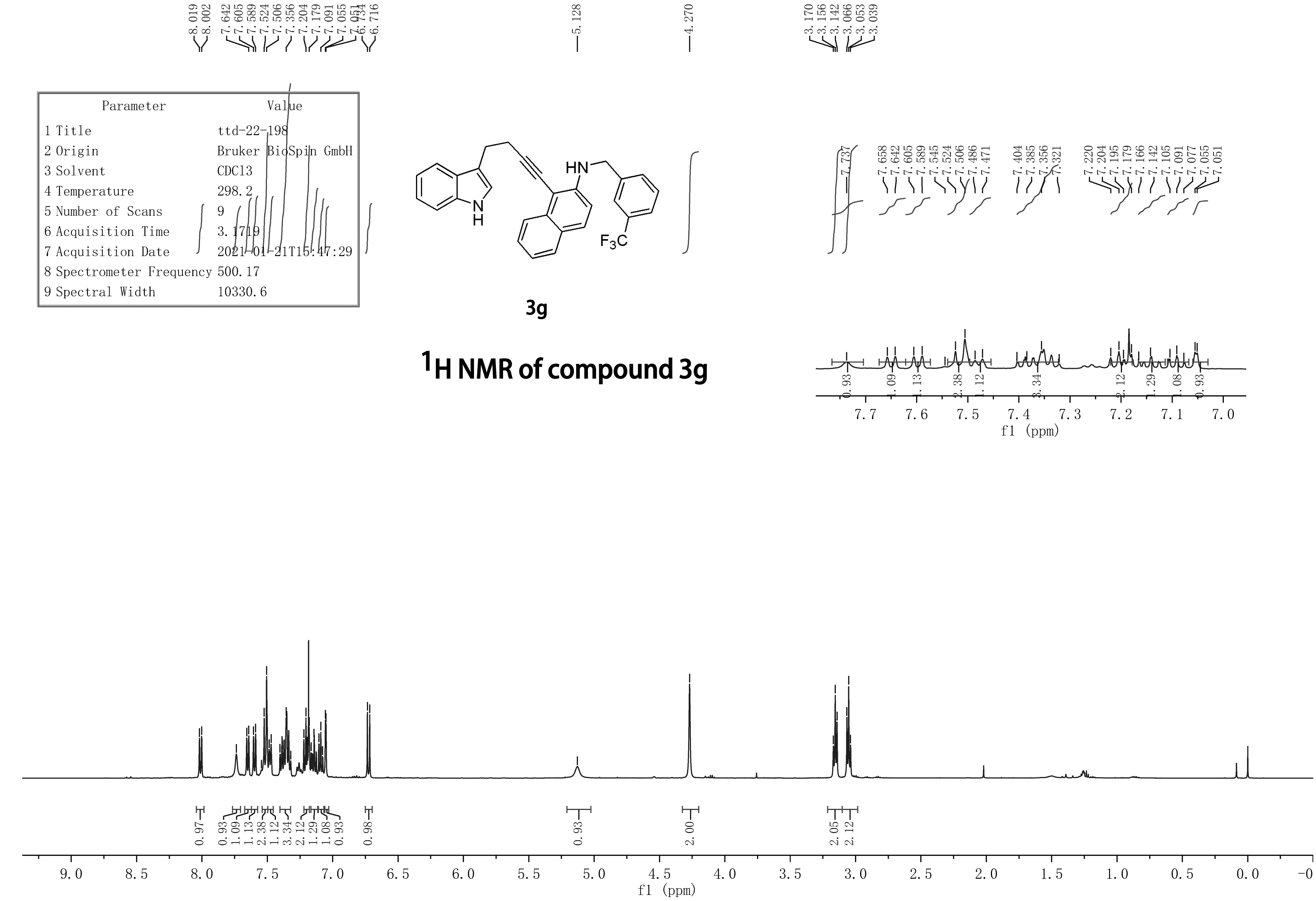

^{1}H NMR of compound 3g

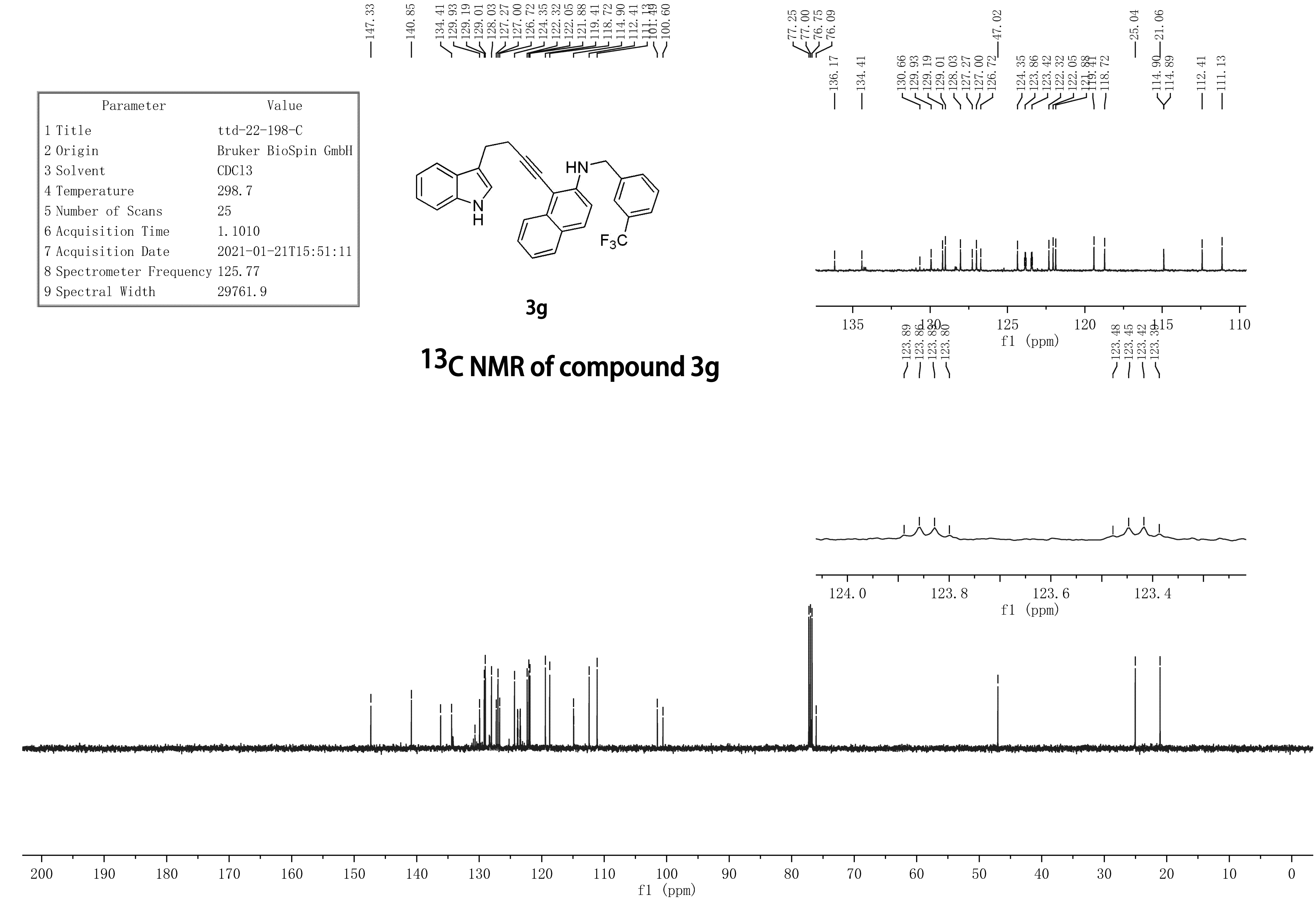

^{13}C NMR of compound 3g

Parameter	Value
1 Title	ttd-22-209
2 Origin	Bruker BioSpin GmbH
3 Solvent	CDCl3
4 Temperature	673.2
5 Number of Scans	5
6 Acquisition Time	3.1719
7 Acquisition Date	2021-01-26T15:50:26
8 Spectrometer Frequency	500.17
9 Spectral Width	10330.6

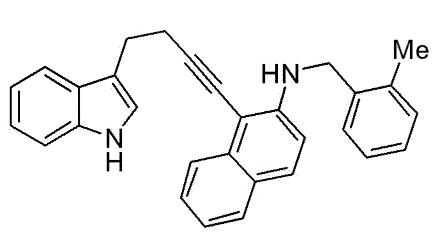

3h

^{1}H NMR of compound 3h

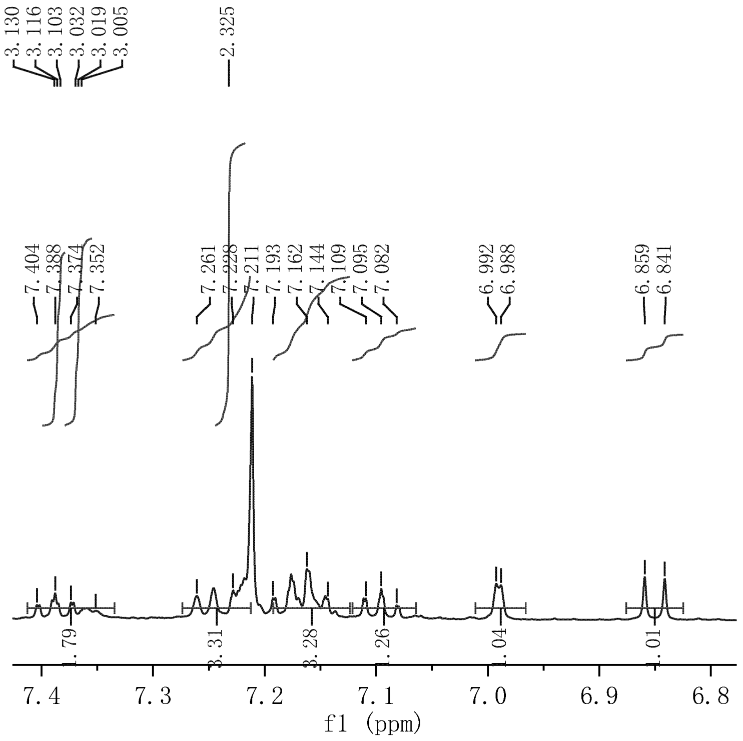

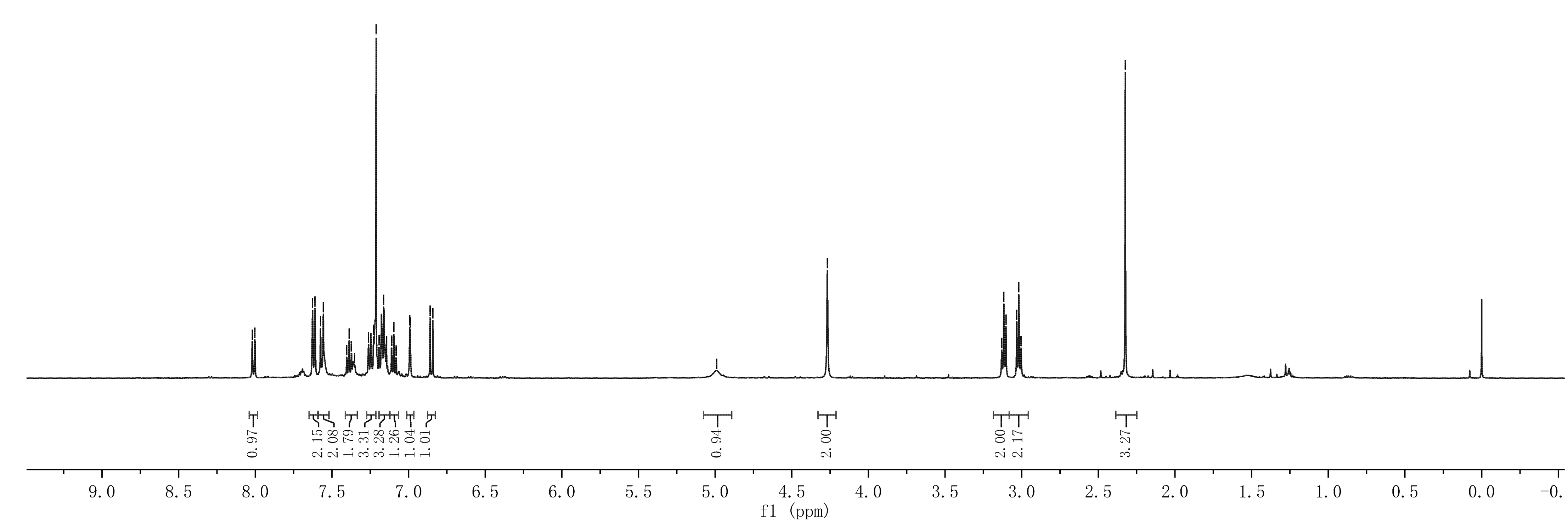

Parameter	Value
1 Title	ttd-22-209-CC
2 Origin	Bruker BioSpin GmbH
3 Solvent	CDCl3
4 Temperature	673.2
5 Number of Scans	27
6 Acquisition Time	1.1010
7 Acquisition Date	2021-01-26T15:53:55
8 Spectrometer Frequency	125.77
9 Spectral Width	29761.9

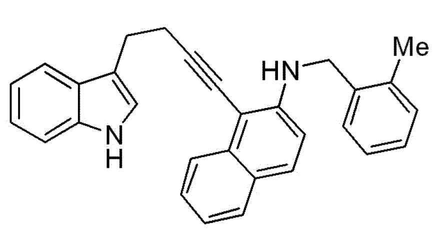

3h

^{13}C NMR of compound 3h

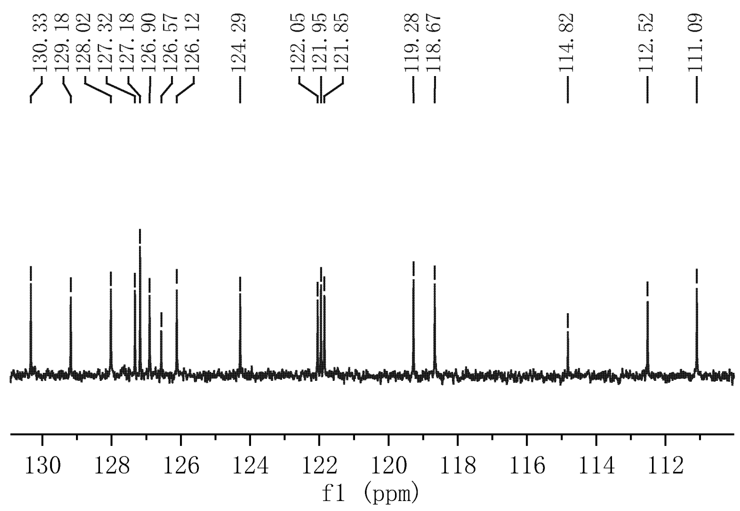

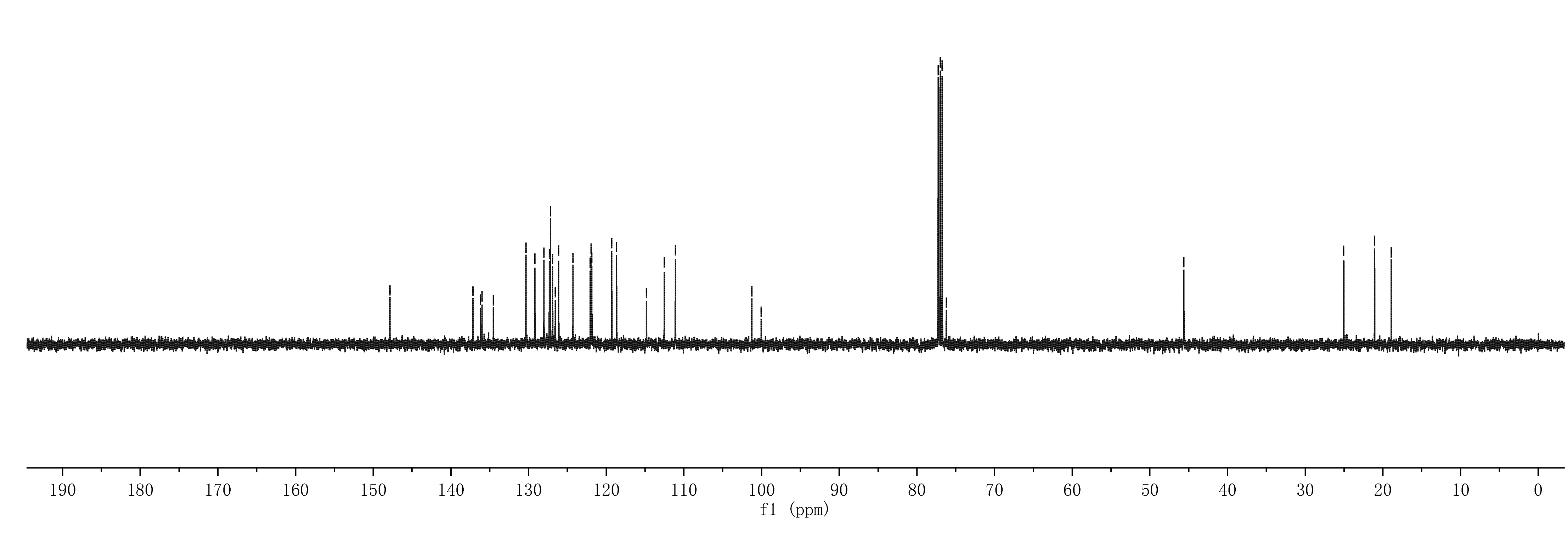

Parameter	Value
1 Title	shz-2-55
2 Origin	Bruker BioSpin GmbH
3 Solvent	CDCl3
4 Temperature	298.0
5 Number of Scans	8
6 Acquisition Time	4.0894
7 Acquisition Date	2021-03-23T09:35:47
8 Spectrometer Frequency	400.13
9 Spectral Width	8012.8

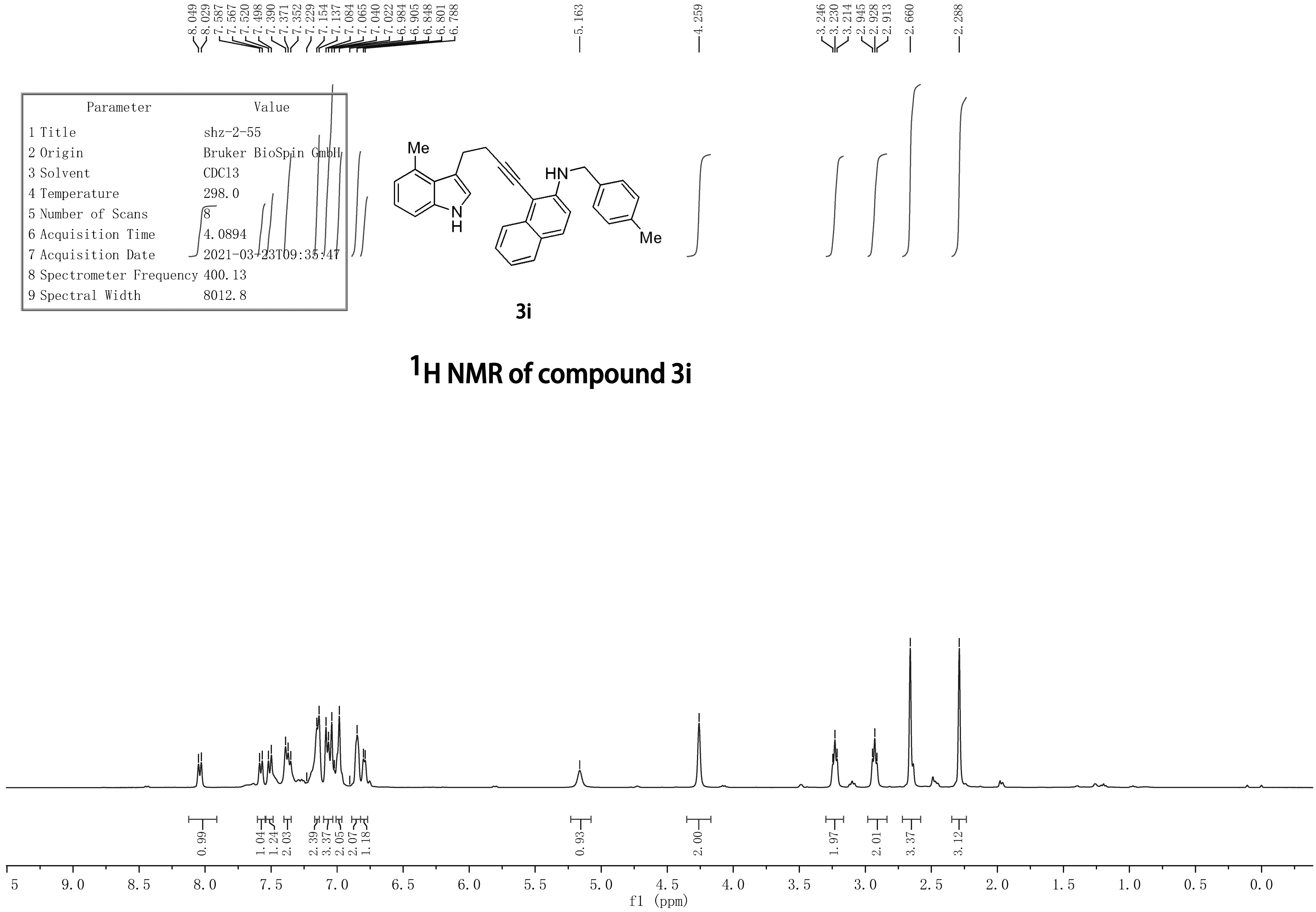

3i

^{1}H NMR of compound 3i

Parameter	Value
1 Title	shz-2-55-C
2 Origin	Bruker BioSpin GmbH
3 Solvent	CDCl3
4 Temperature	300.0
5 Number of Scans	13
6 Acquisition Time	1.3631
7 Acquisition Date	2021-03-23T09:37:18
8 Spectrometer Frequency	100.61
9 Spectral Width	24038.5

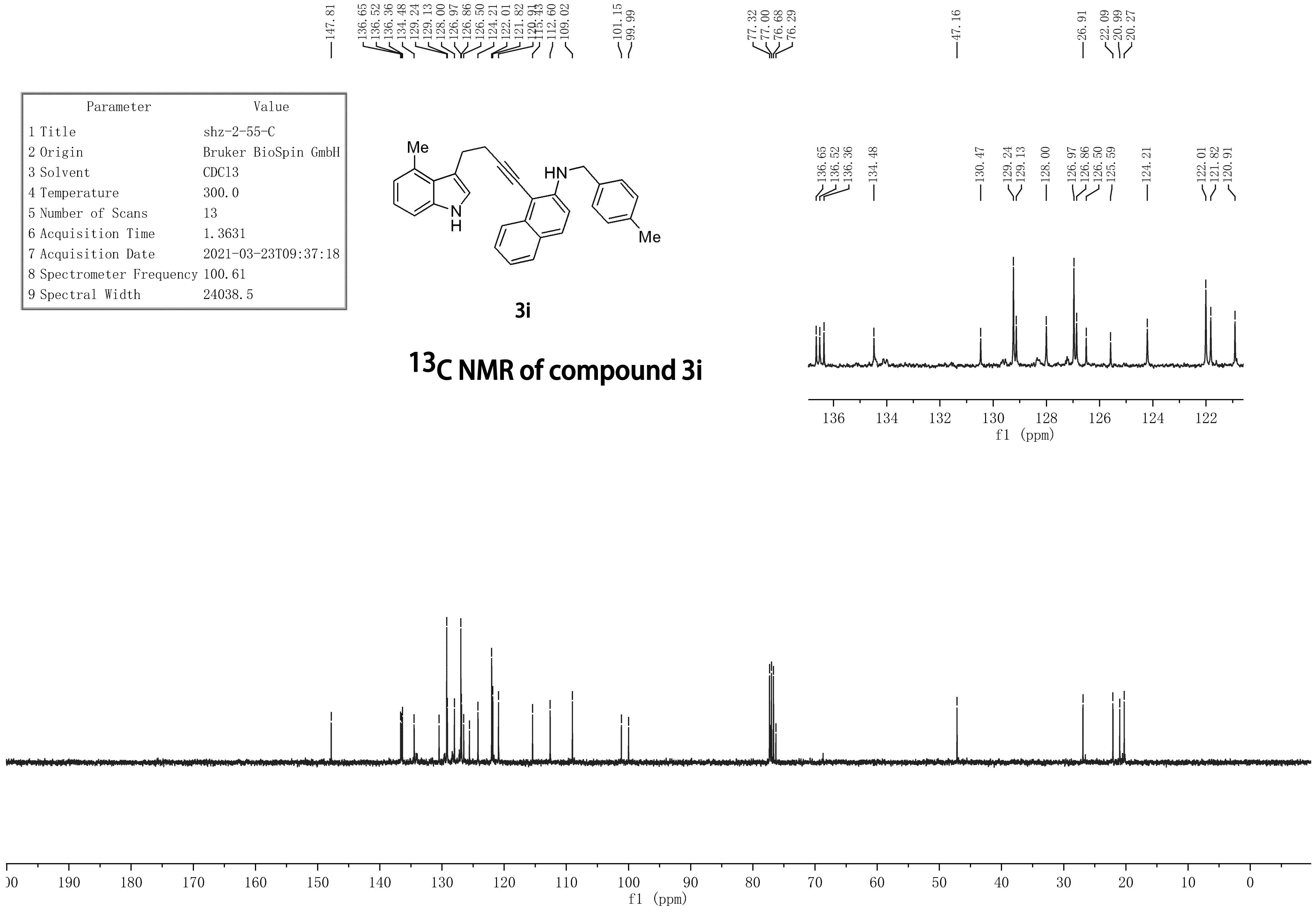

3i

^{13}C NMR of compound 3i

Parameter	Value
1 Title	shz-2-97
2 Origin	Bruker BioSpin GmbH
3 Solvent	CDCl3
4 Temperature	297.9
5 Number of Scans	8
6 Acquisition Time	3.1719
7 Acquisition Date	2021-04-13T08:11:49
8 Spectrometer Frequency	500.17
9 Spectral Width	10330.6

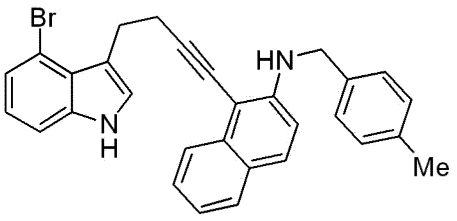

3j

^{1}H NMR of compound 3j

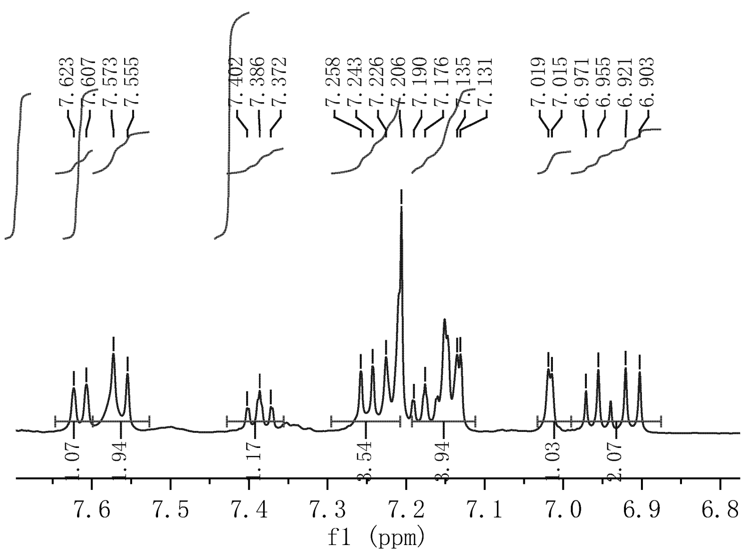

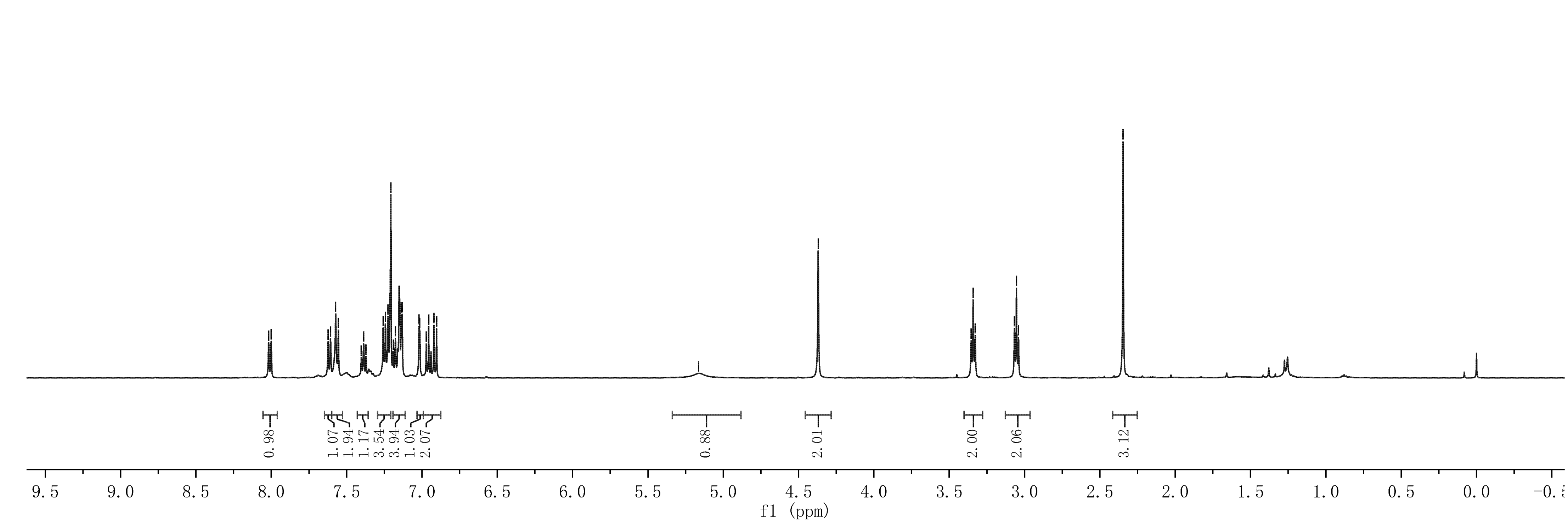

Parameter	Value
1 Title	shz-2-97-C
2 Origin	Bruker BioSpin GmbH
3 Solvent	CDCl3
4 Temperature	298.1
5 Number of Scans	27
6 Acquisition Time	1.1010
7 Acquisition Date	2021-04-13T08:14:29
8 Spectrometer Frequency	125.77
9 Spectral Width	29761.9

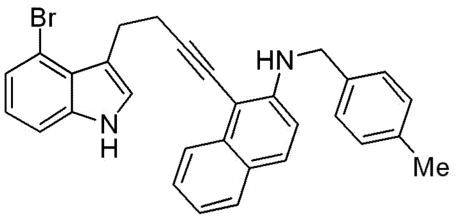

3j

^{13}C NMR of compound 3j

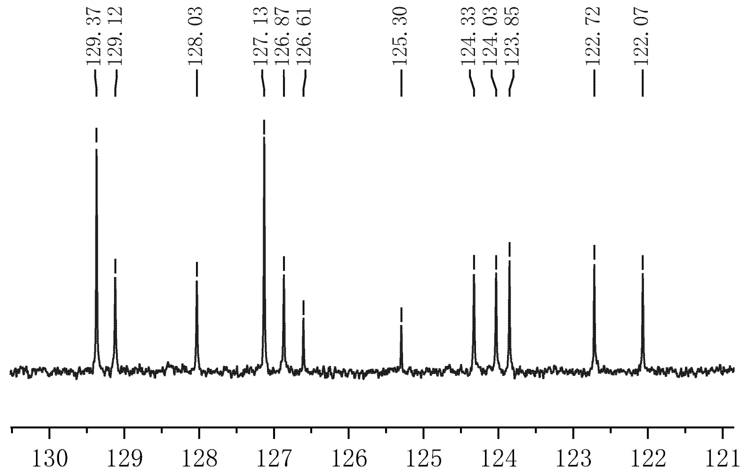

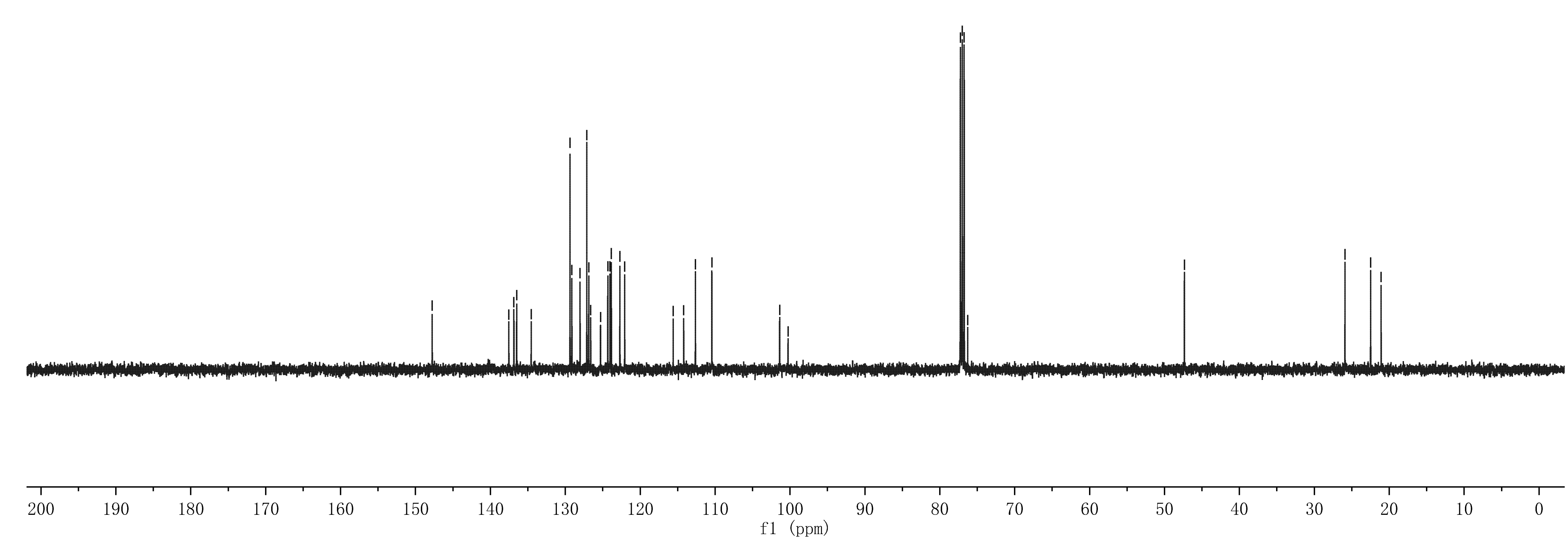

Parameter	Value
1 Title	shz-2-38
2 Origin	Bruker BioSpin GmbH
3 Solvent	CDCl3
4 Temperature	298.0
5 Number of Scans	7
6 Acquisition Time	4.0894
7 Acquisition Date	2021-03-15T21:03:04
8 Spectrometer Frequency	400.13
9 Spectral Width	8012.8

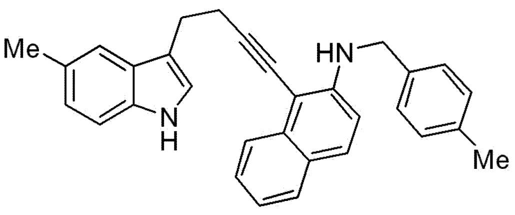

3k

^{1}H NMR of compound 3k

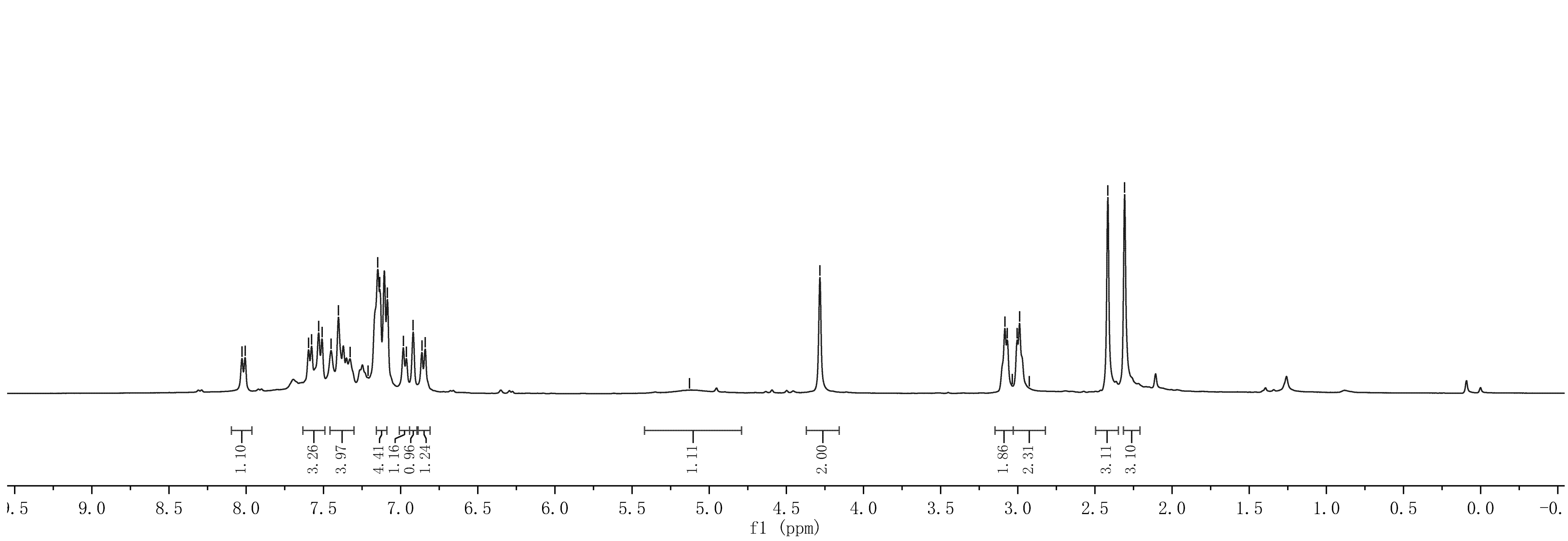

Parameter	Value
1 Title	shz-2-38-C
2 Origin	Bruker BioSpin GmbH
3 Solvent	CDCl3
4 Temperature	300.0
5 Number of Scans	45
6 Acquisition Time	1.3631
7 Acquisition Date	2021-03-15T21:04:25
8 Spectrometer Frequency	100.61
9 Spectral Width	24038.5

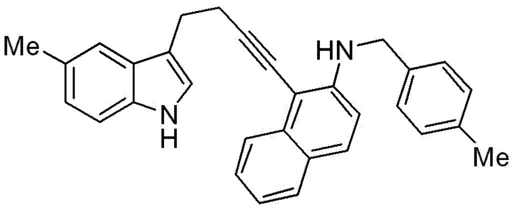

3k

^{13}C NMR of compound 3k

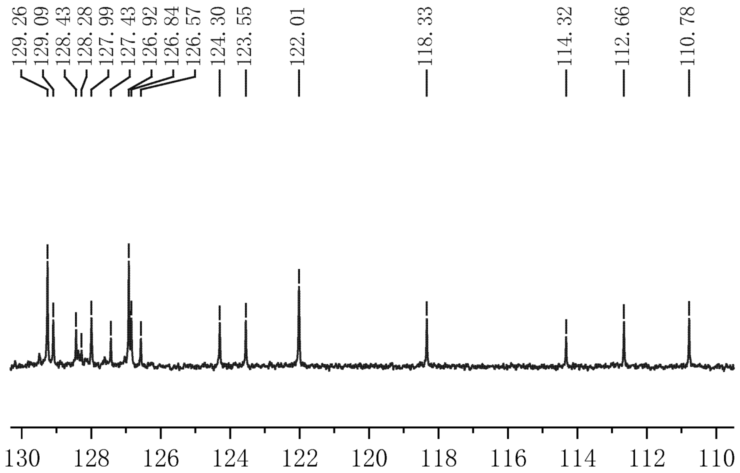

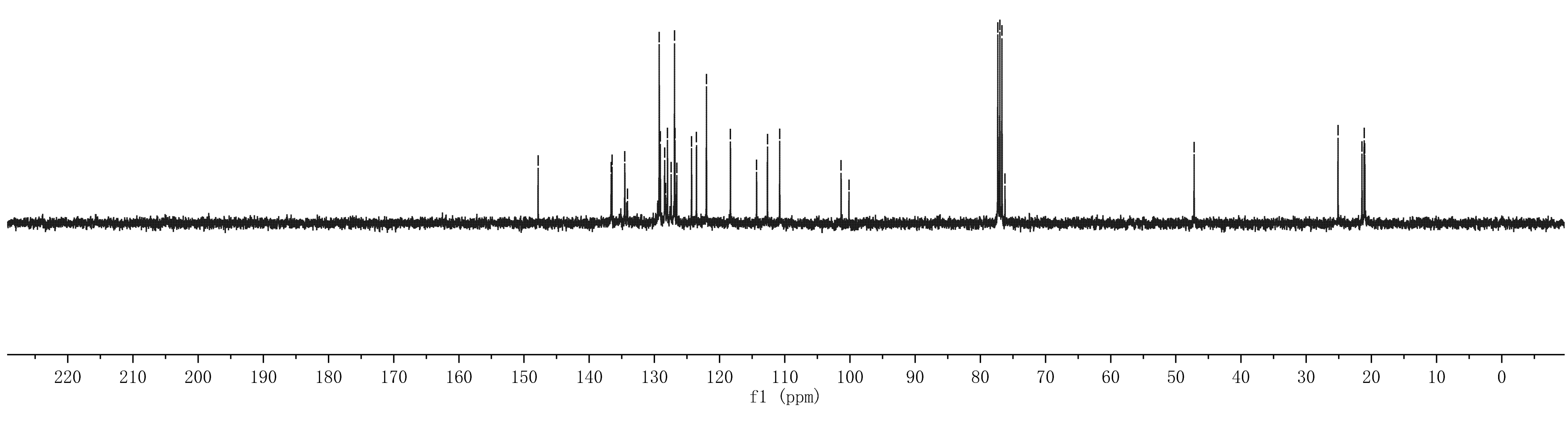

Parameter	Value
1 Title	shz-2-141
2 Origin	Bruker BioSpin GmbH
3 Solvent	CDCl3
4 Temperature	297.1
5 Number of Scans	9
6 Acquisition Time	3.1719
7 Acquisition Date	2021-05-14T13:51:22
8 Spectrometer Frequency	500.17
9 Spectral Width	10330.6

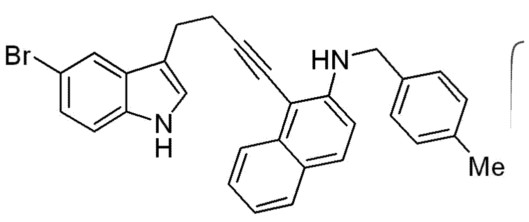

3l

^{1}H NMR of compound 3l

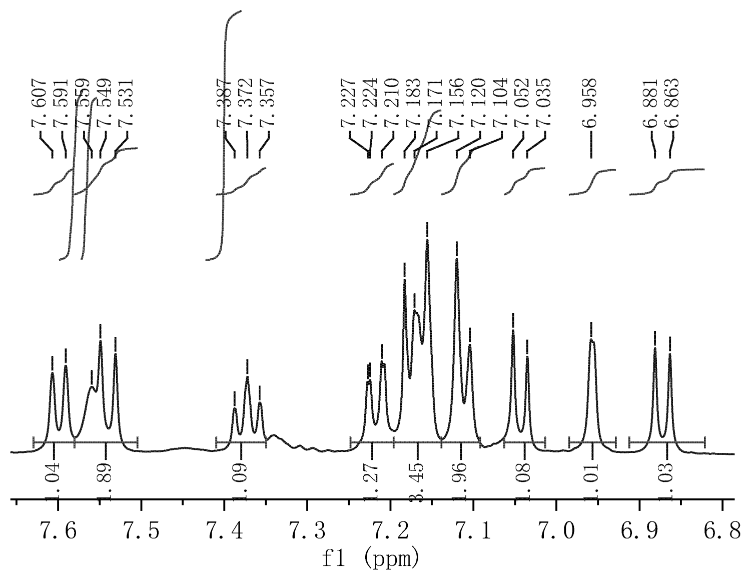

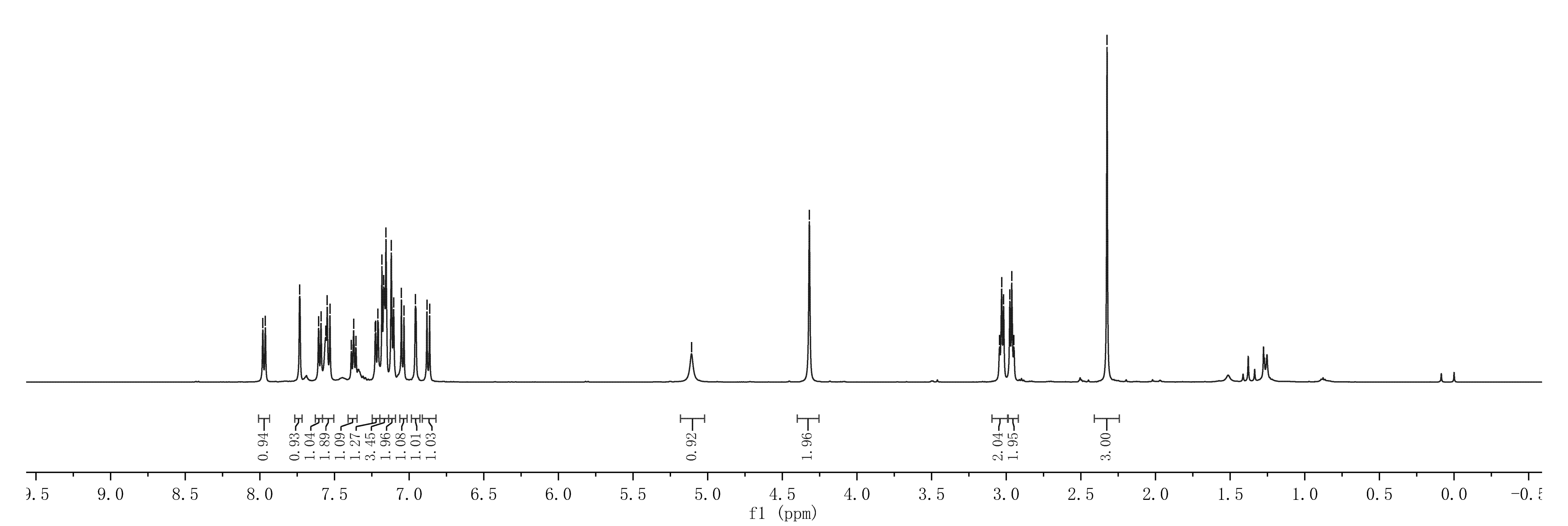

Parameter	Value
1 Title	shz-2-141-C
2 Origin	Bruker BioSpin GmbH
3 Solvent	CDCl3
4 Temperature	297.3
5 Number of Scans	18
6 Acquisition Time	1.1010
7 Acquisition Date	2021-05-14T13:52:41
8 Spectrometer Frequency	125.77
9 Spectral Width	29761.9

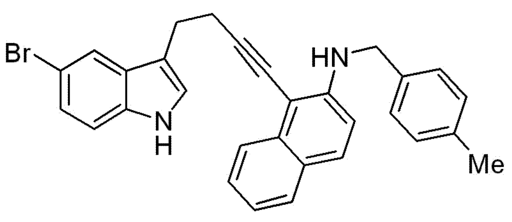

3l

^{13}C NMR of compound 3l

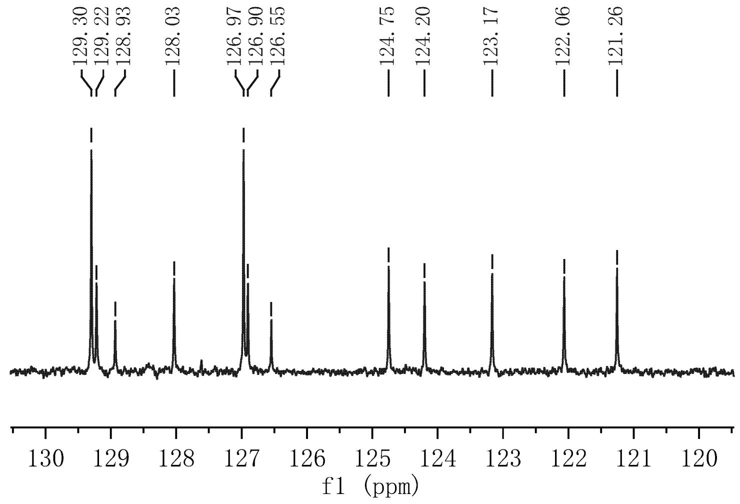

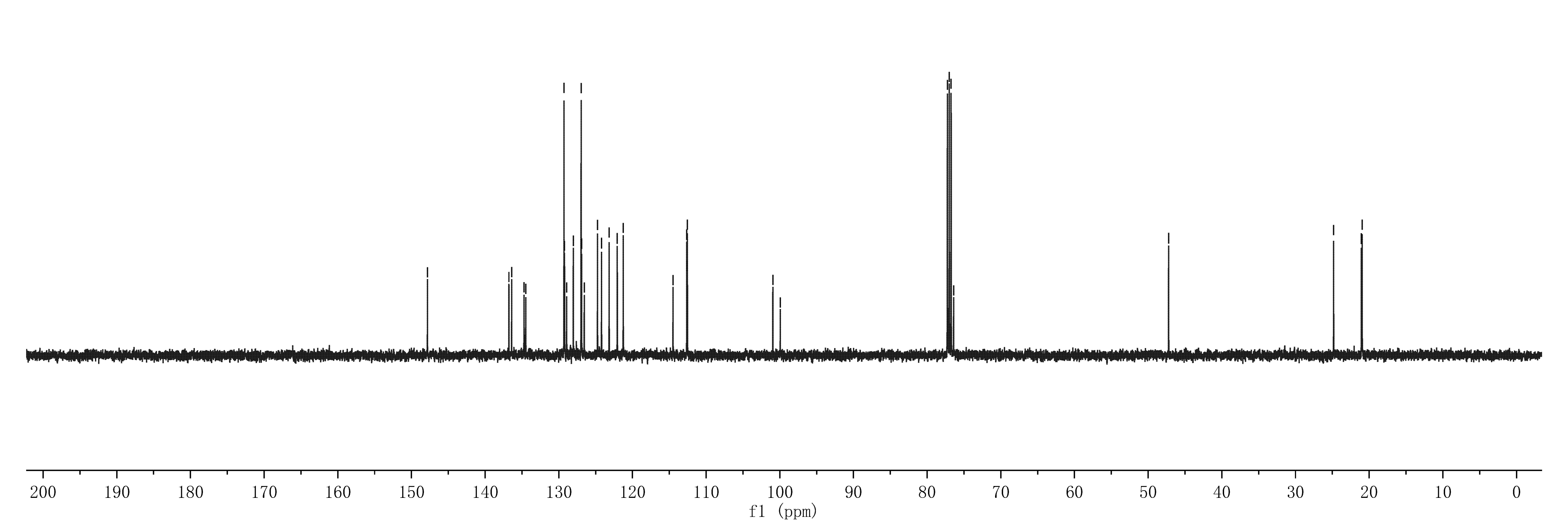

Parameter	Value
1 Title	shz-2-142
2 Origin	Bruker BioSpin GmbH
3 Solvent	CDCl3
4 Temperature	297.1
5 Number of Scans	9
6 Acquisition Time	3.1719
7 Acquisition Date	2021-05-14T13:43:41
8 Spectrometer Frequency	500.17
9 Spectral Width	10330.6

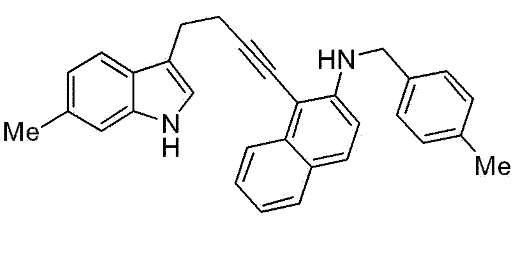

3m

^{1}H NMR of compound 3m

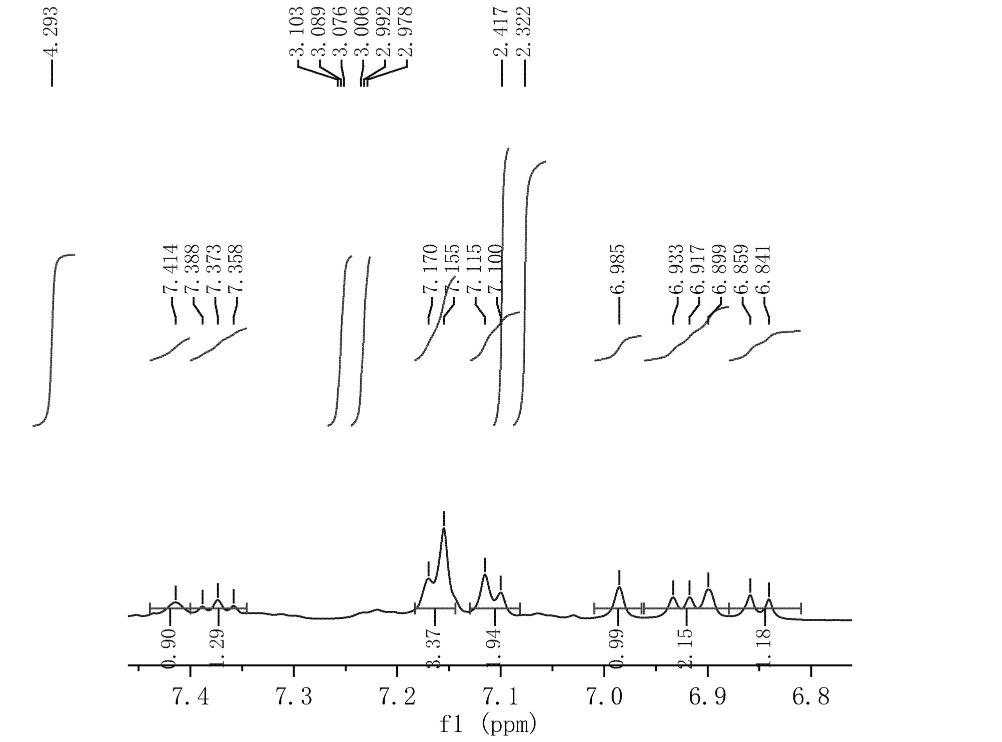

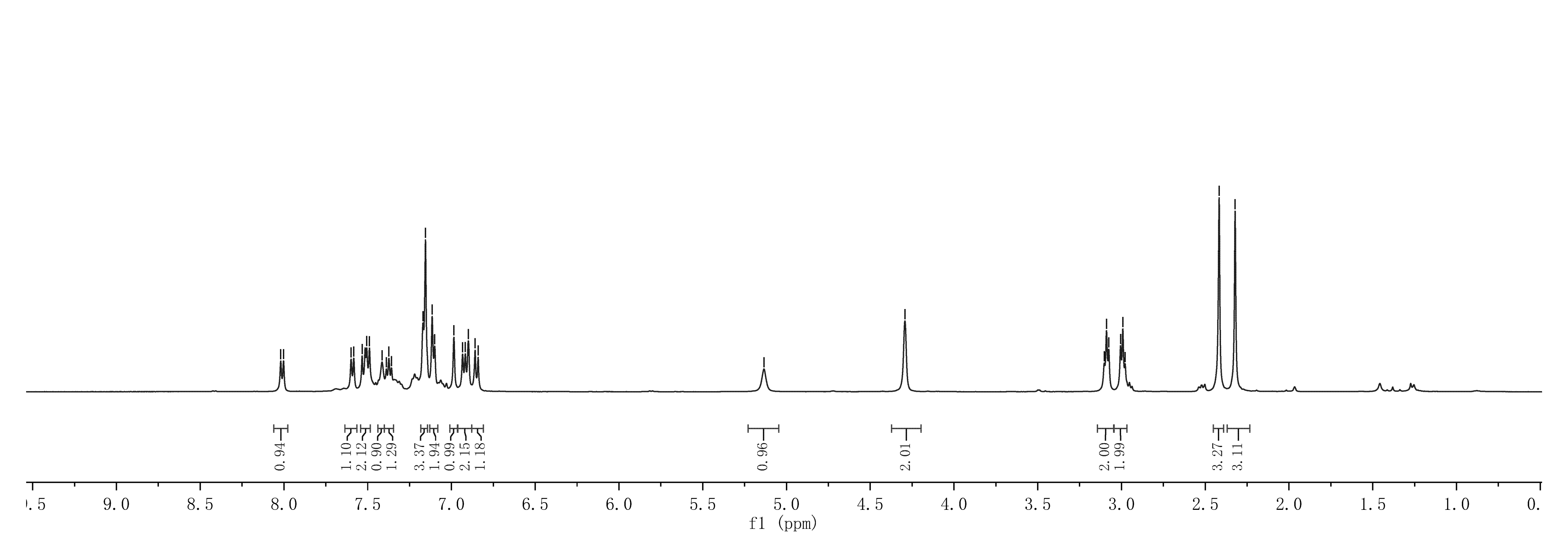

Parameter	Value
1 Title	shz-2-142-C
2 Origin	Bruker BioSpin GmbH
3 Solvent	CDCl3
4 Temperature	297.4
5 Number of Scans	18
6 Acquisition Time	1.1010
7 Acquisition Date	2021-05-14T13:47:00
8 Spectrometer Frequency	125.77
9 Spectral Width	29761.9

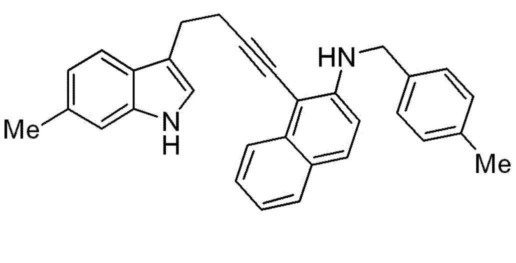

3m

^{13}C NMR of compound 3m

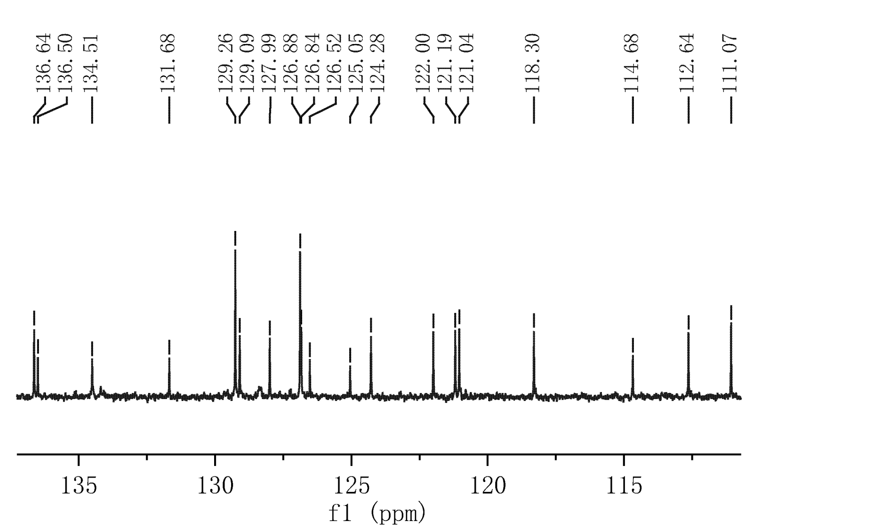

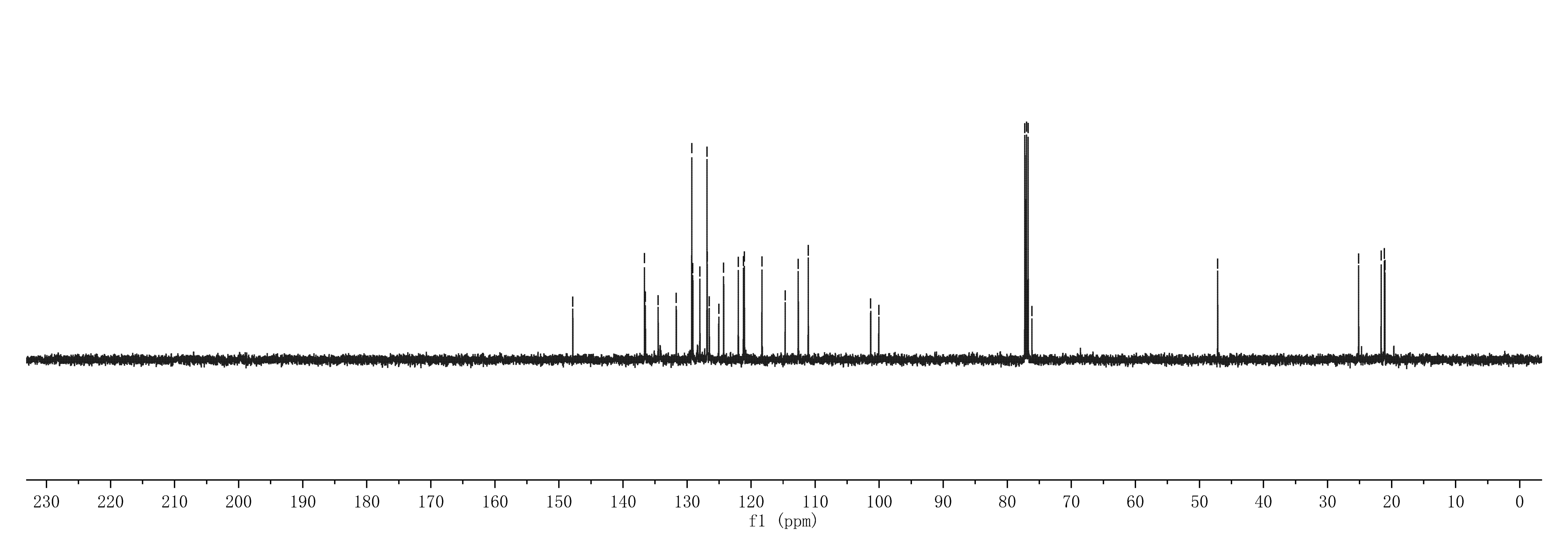

Parameter	Value
1 Title	shz-2-45
2 Origin	Bruker BioSpin GmbH
3 Solvent	CDCl3
4 Temperature	298.2
5 Number of Scans	6
6 Acquisition Time	3.1719
7 Acquisition Date	2021-03-17T17:31:40
8 Spectrometer Frequency	500.17
9 Spectral Width	10330.6

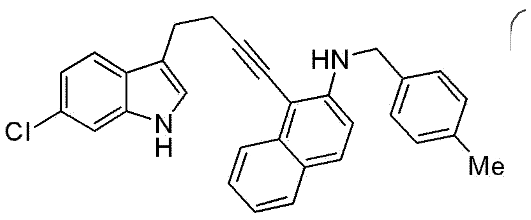

3n

^{1}H NMR of compound 3n

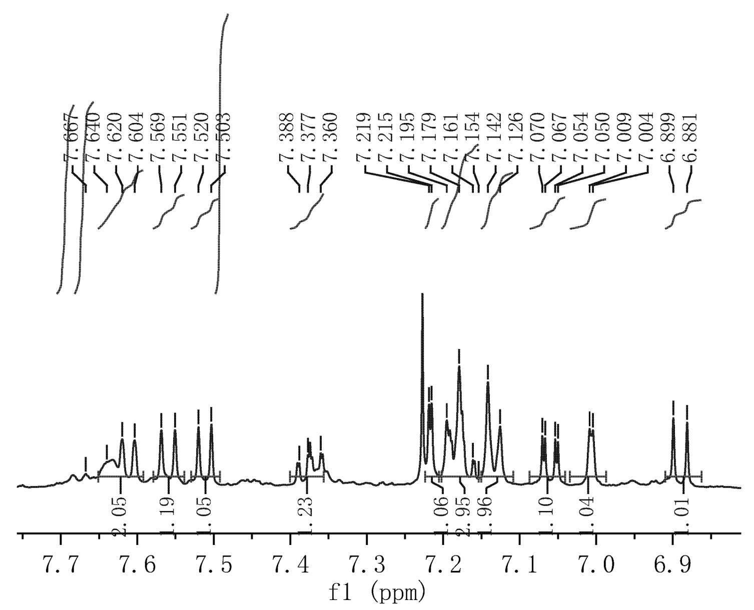

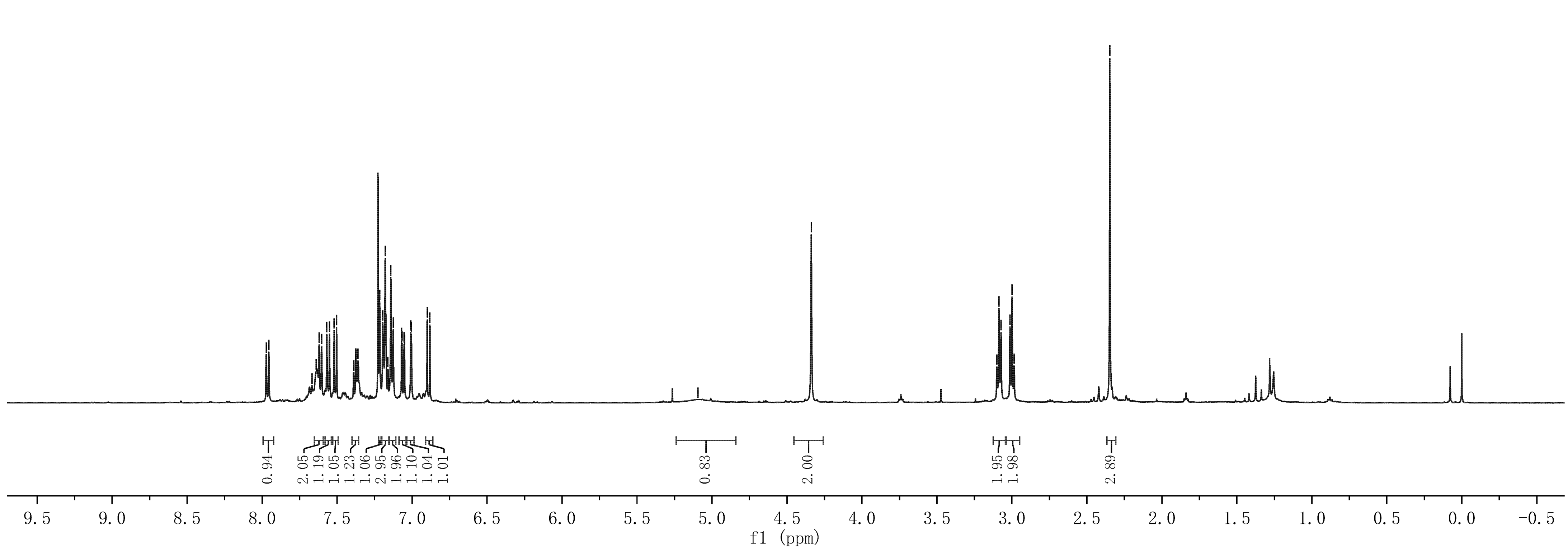

Parameter	Value
1 Title	shz-2-45-C
2 Origin	Bruker BioSpin GmbH
3 Solvent	CDCl3
4 Temperature	298.7
5 Number of Scans	25
6 Acquisition Time	1.1010
7 Acquisition Date	2021-03-17T17:35:42
8 Spectrometer Frequency	125.77
9 Spectral Width	29761.9

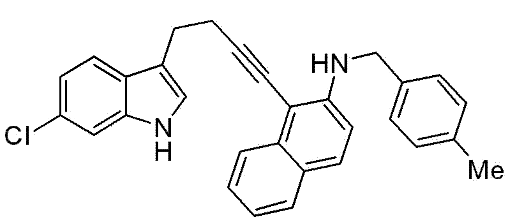

3n

^{13}C NMR of compound 3n

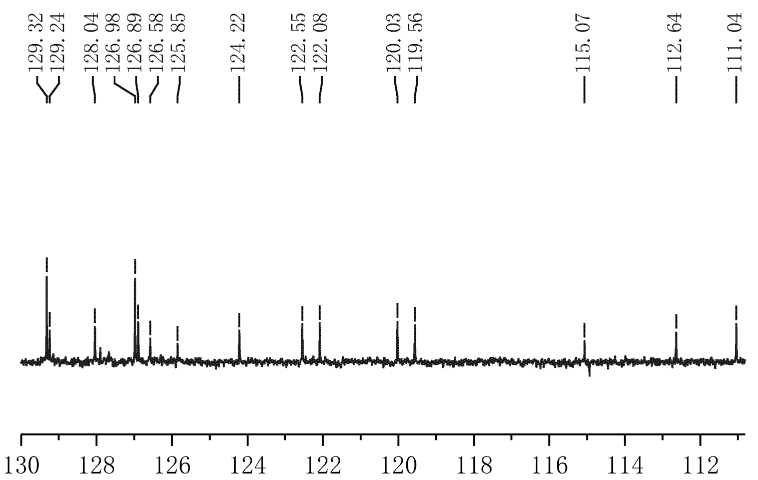

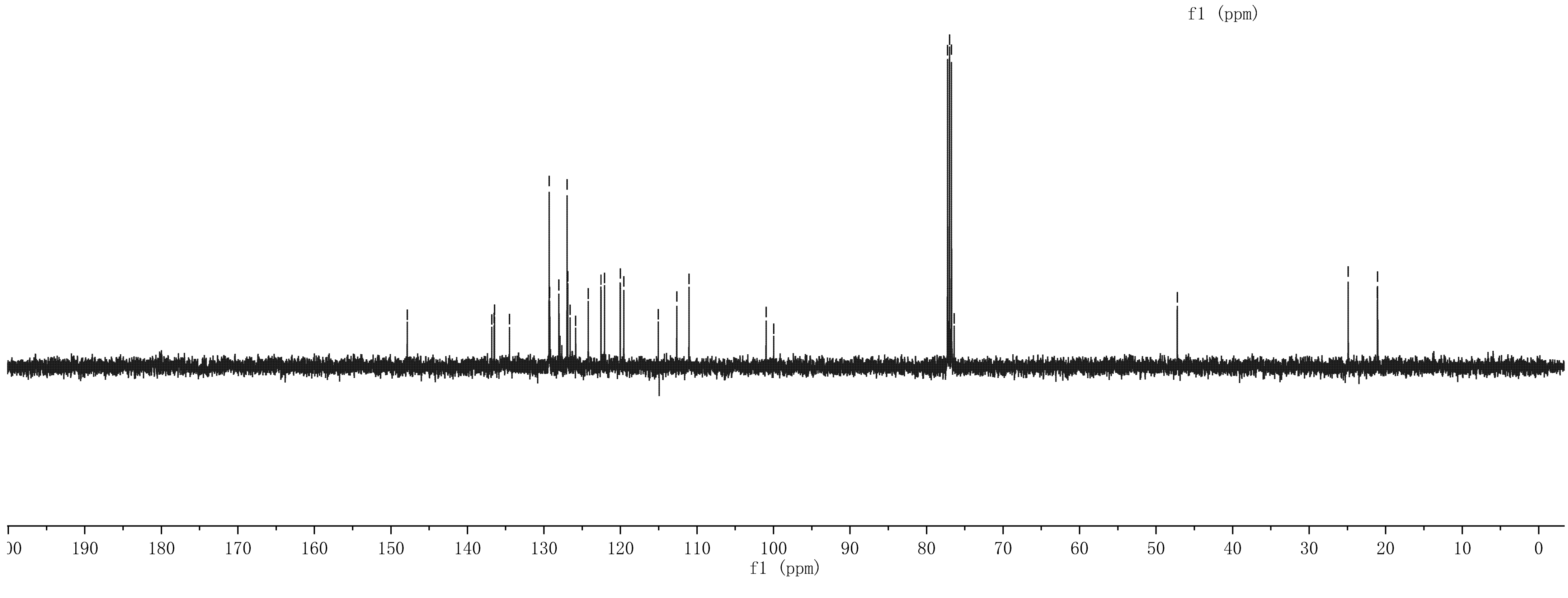

Parameter	Value
1 Title	shz-2-98
2 Origin	Bruker BioSpin GmbH
3 Solvent	CDCl3
4 Temperature	297.9
5 Number of Scans	6
6 Acquisition Time	3.1719
7 Acquisition Date	2021-04-13T08:18:42
8 Spectrometer Frequency	500.17
9 Spectral Width	10330.6

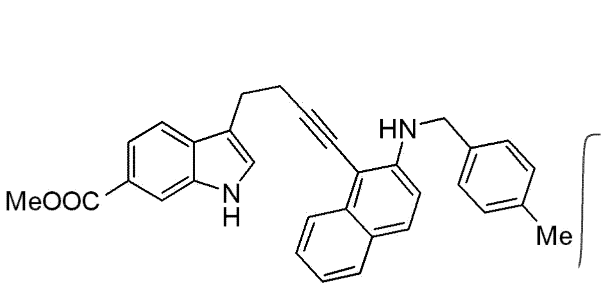

3o

^{1}H NMR of compound 3o

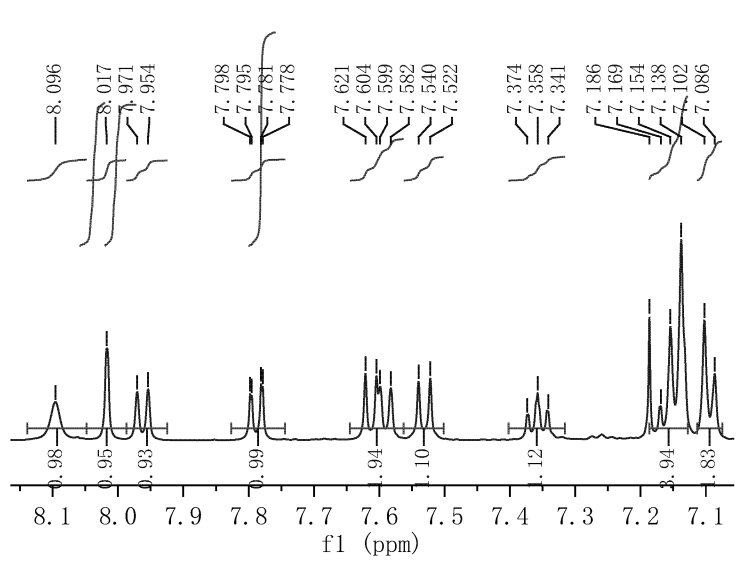

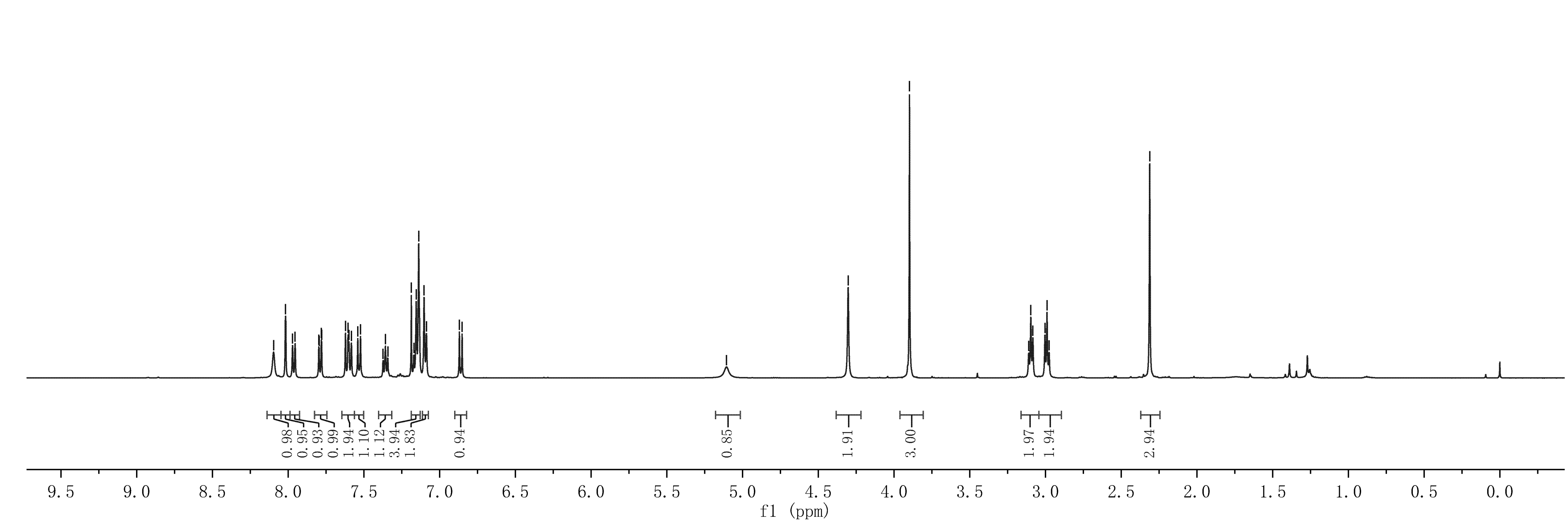

Parameter	Value
1 Title	shz-2-98-C
2 Origin	Bruker BioSpin GmbH
3 Solvent	CDCl3
4 Temperature	298.3
5 Number of Scans	16
6 Acquisition Time	1.1010
7 Acquisition Date	2021-04-13T08:20:09
8 Spectrometer Frequency	125.77
9 Spectral Width	29761.9

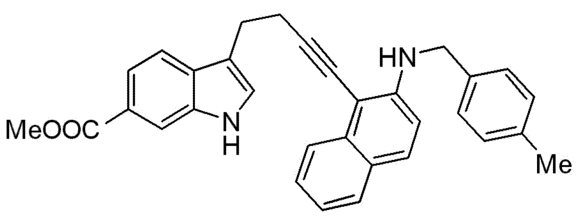

3o

^{13}C NMR of compound 3o

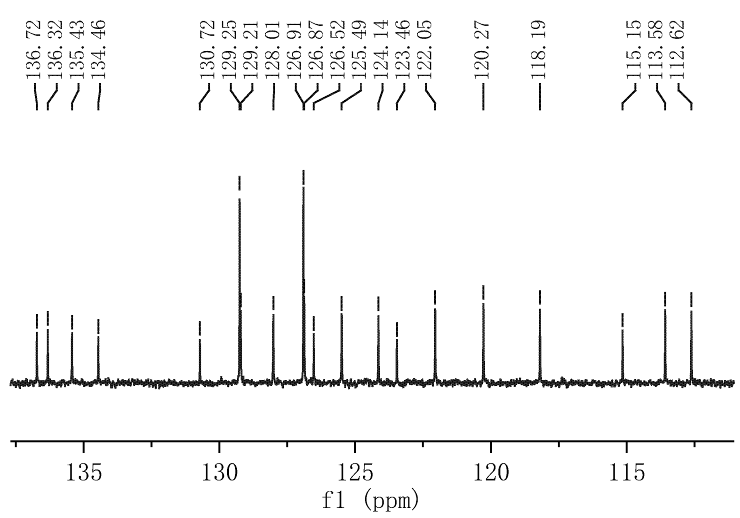

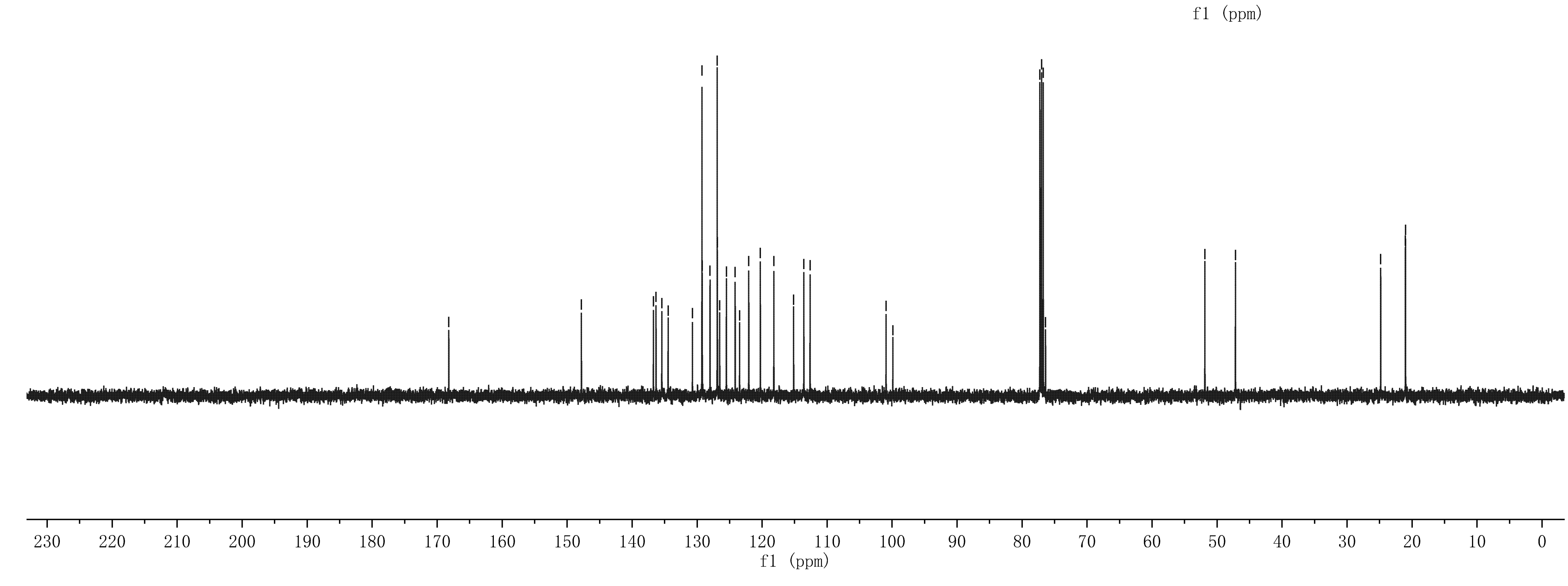

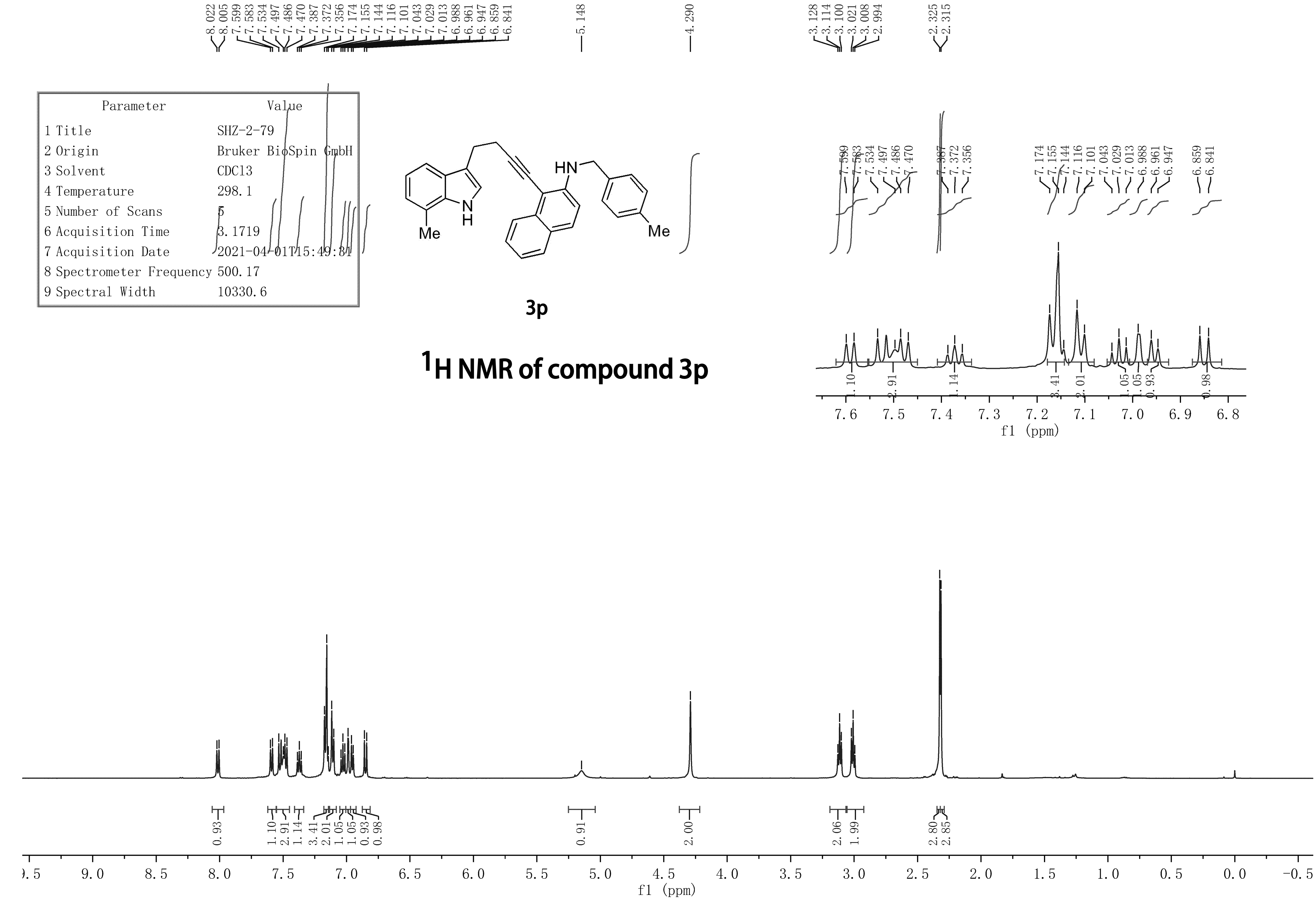

1H NMR of compound 3p

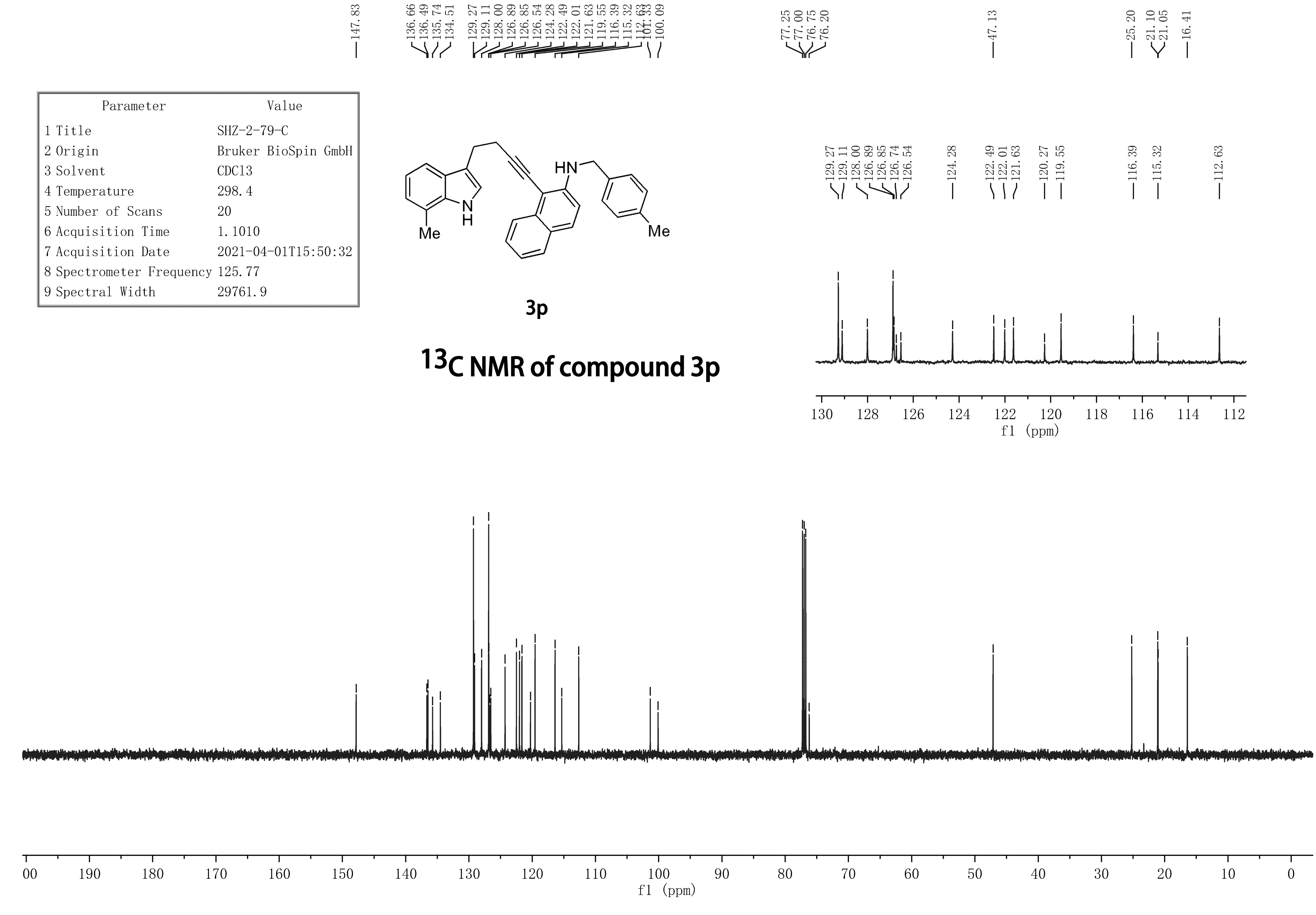

13C NMR of compound 3p

Parameter	Value
1 Title	SHZ-2-80
2 Origin	Bruker BioSpin GmbH
3 Solvent	CDCl3
4 Temperature	298.1
5 Number of Scans	9
6 Acquisition Time	3.1719
7 Acquisition Date	2021-04-01T15:55:40
8 Spectrometer Frequency	500.17
9 Spectral Width	10330.6

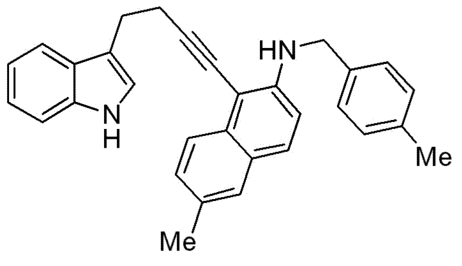

3q

^{1}H NMR of compound 3q

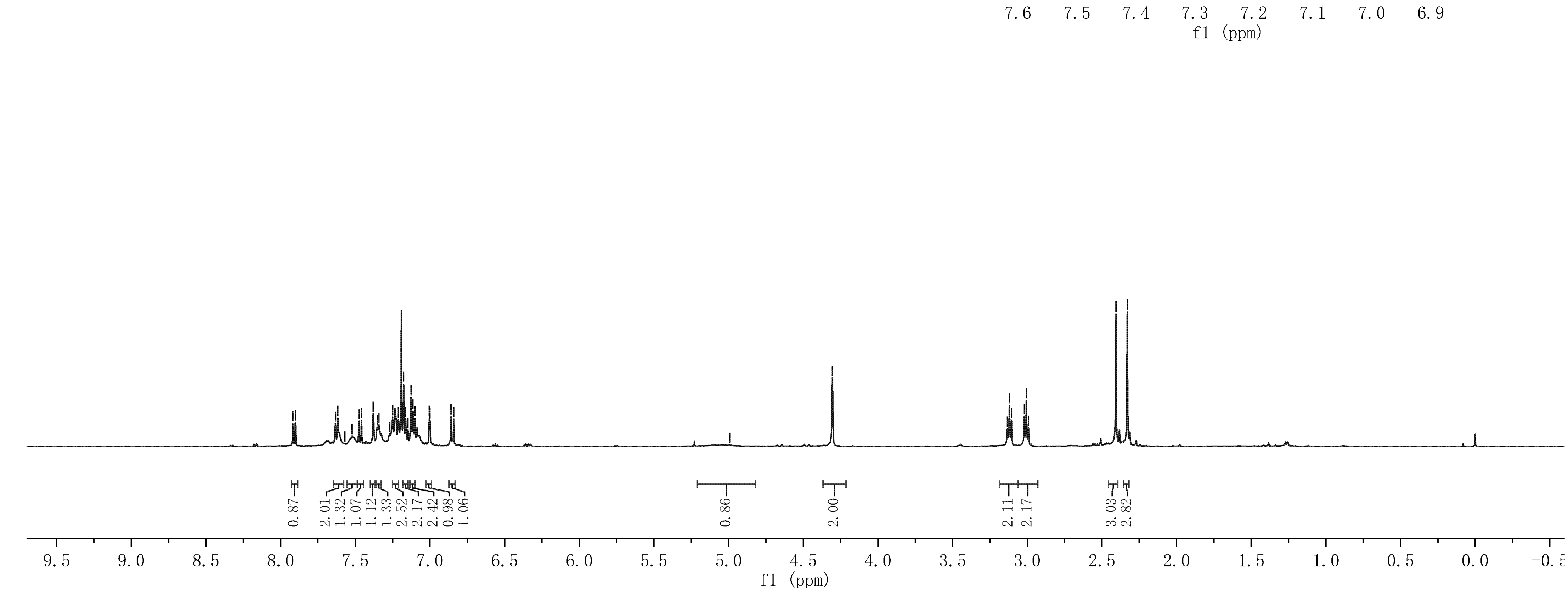

Parameter	Value
1 Title	SHZ-2-80-C
2 Origin	Bruker BioSpin GmbH
3 Solvent	CDCl3
4 Temperature	298.4
5 Number of Scans	26
6 Acquisition Time	1.1010
7 Acquisition Date	2021-04-01T15:56:54
8 Spectrometer Frequency	125.77
9 Spectral Width	29761.9

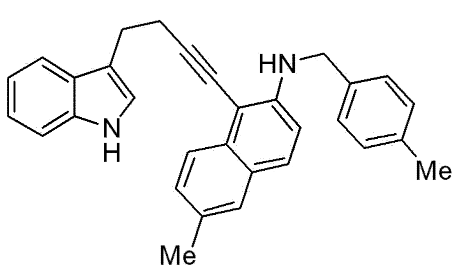

3q

^{13}C NMR of compound 3q

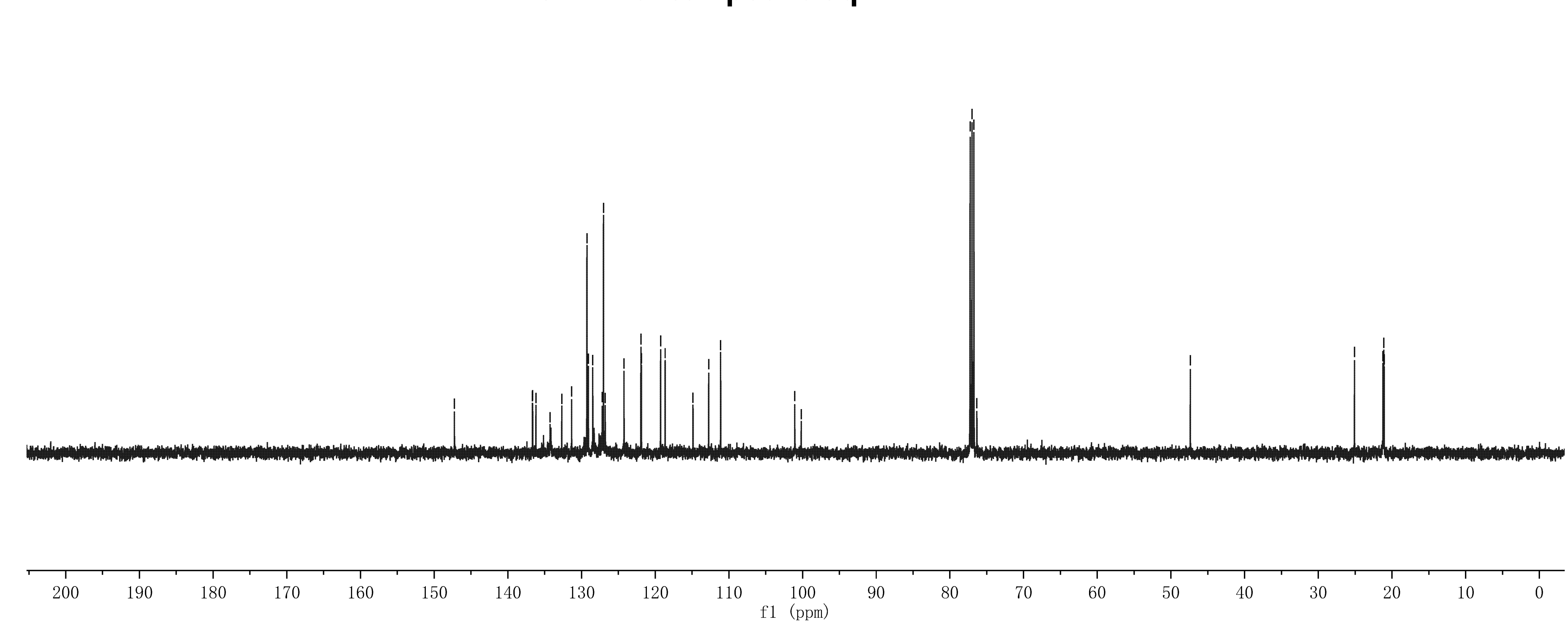

Parameter	Value
1 Title	SHZ-2-120-2
2 Origin	Bruker BioSpin GmbH
3 Solvent	CDCl3
4 Temperature	297.4
5 Number of Scans	8
6 Acquisition Time	3.1719
7 Acquisition Date	2021-04-28T11:02:55
8 Spectrometer Frequency	500.17
9 Spectral Width	10330.6

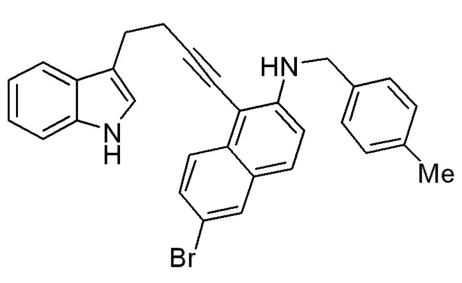

3r

^{1}H NMR of compound 3r

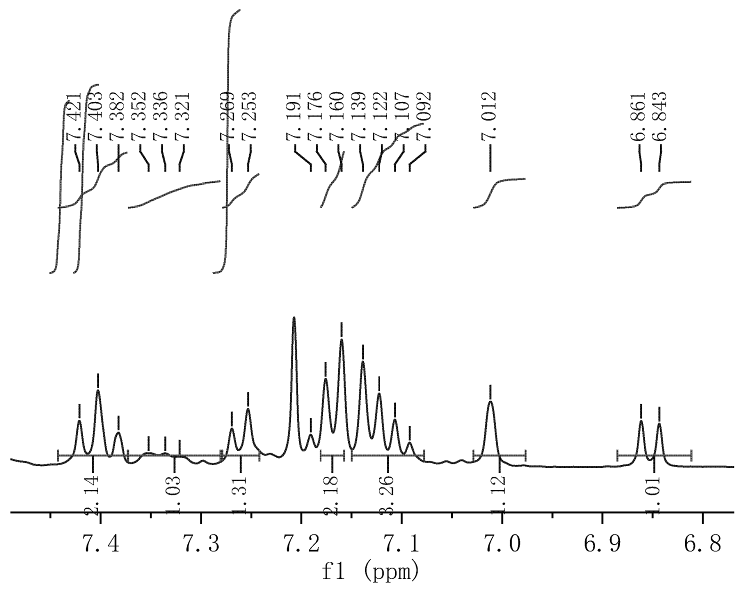

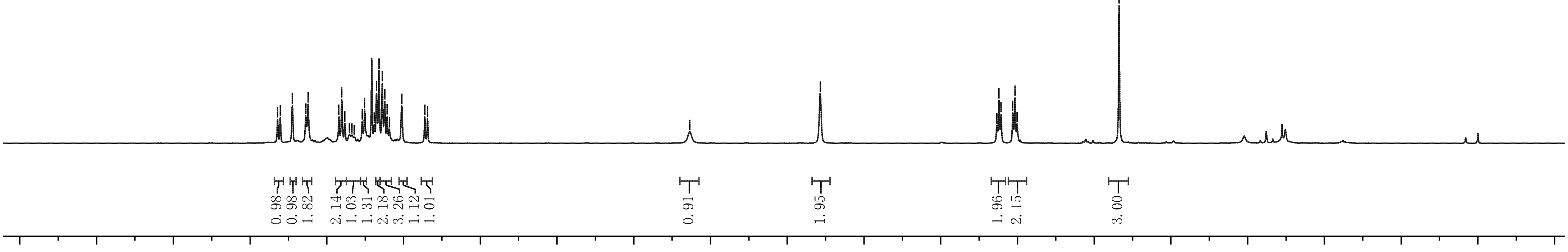

Parameter	Value
1 Title	SHZ-2-120-CC-2
2 Origin	Bruker BioSpin GmbH
3 Solvent	CDCl3
4 Temperature	297.9
5 Number of Scans	27
6 Acquisition Time	1.1010
7 Acquisition Date	2021-04-28T11:07:18
8 Spectrometer Frequency	125.77
9 Spectral Width	29761.9

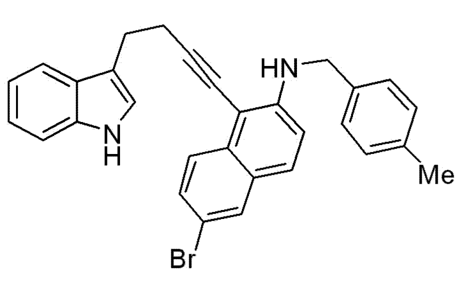

3r

^{13}C NMR of compound 3r

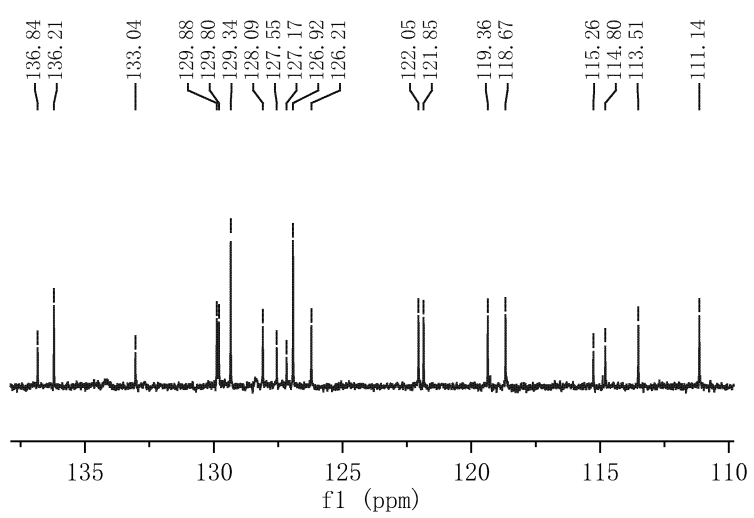

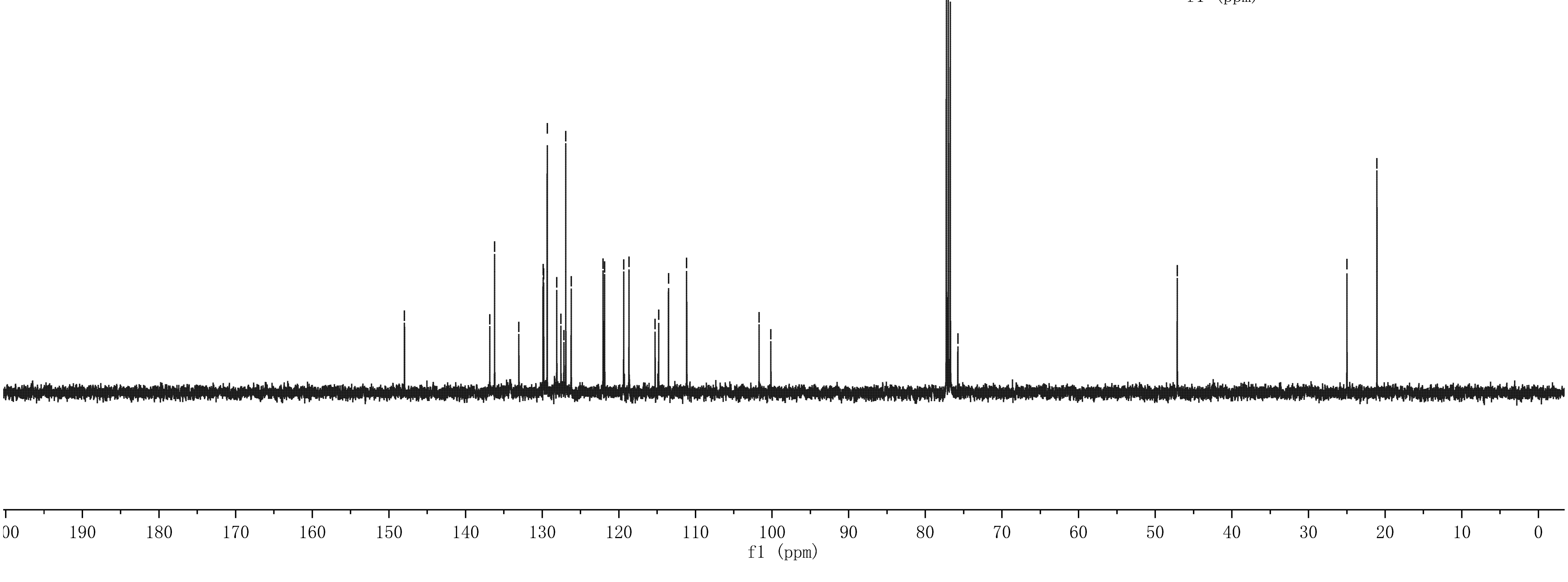

Parameter	Value
1 Title	shz-2-137
2 Origin	Bruker BioSpin GmbH
3 Solvent	CDCl3
4 Temperature	298.0
5 Number of Scans	9
6 Acquisition Time	4.0894
7 Acquisition Date	2021-05-13T08:03:45
8 Spectrometer Frequency	400.13
9 Spectral Width	8012.8

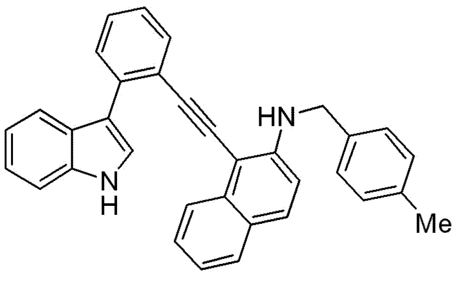

3s

^{1}H NMR of compound 3s

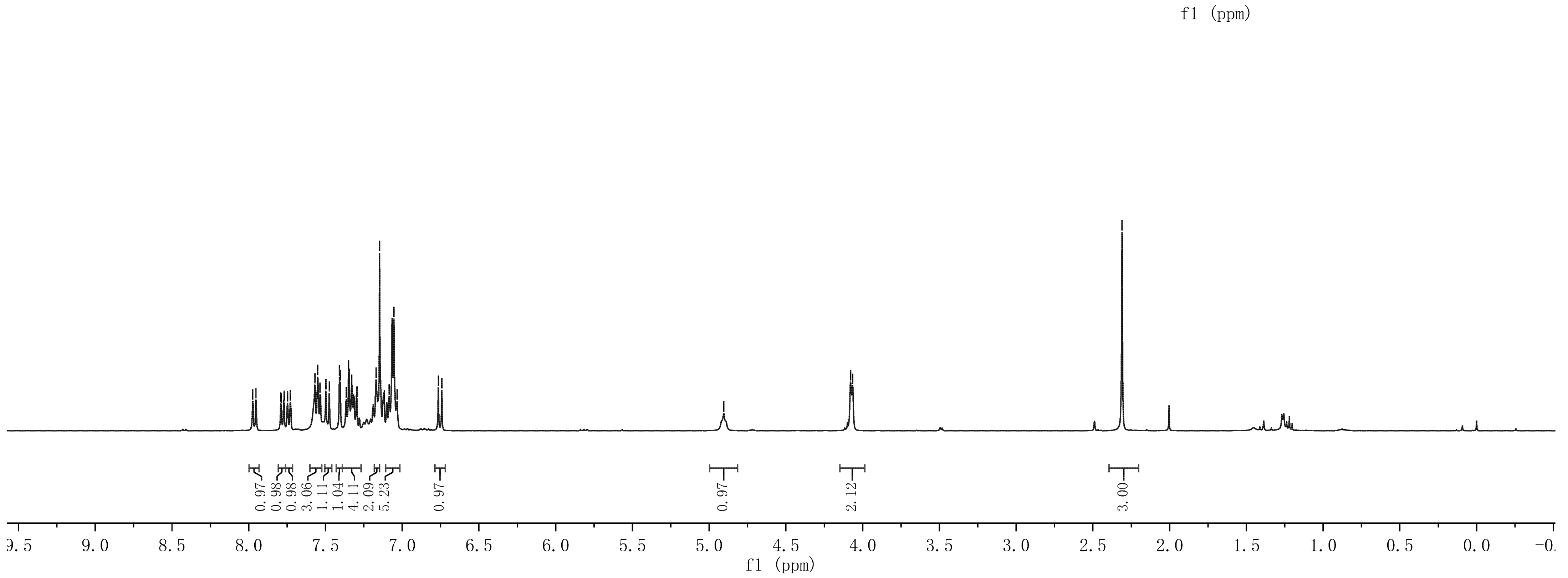

Parameter	Value
1 Title	shz-2-137-C
2 Origin	Bruker BioSpin GmbH
3 Solvent	CDCl3
4 Temperature	300.0
5 Number of Scans	21
6 Acquisition Time	1.3631
7 Acquisition Date	2021-05-13T08:05:51
8 Spectrometer Frequency	100.61
9 Spectral Width	24038.5

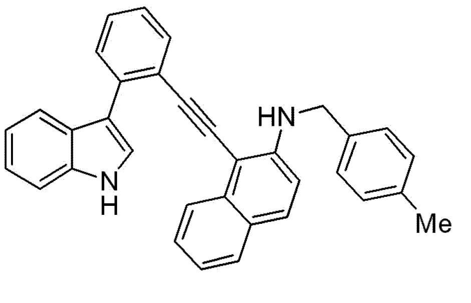

3s

^{13}C NMR of compound 3s

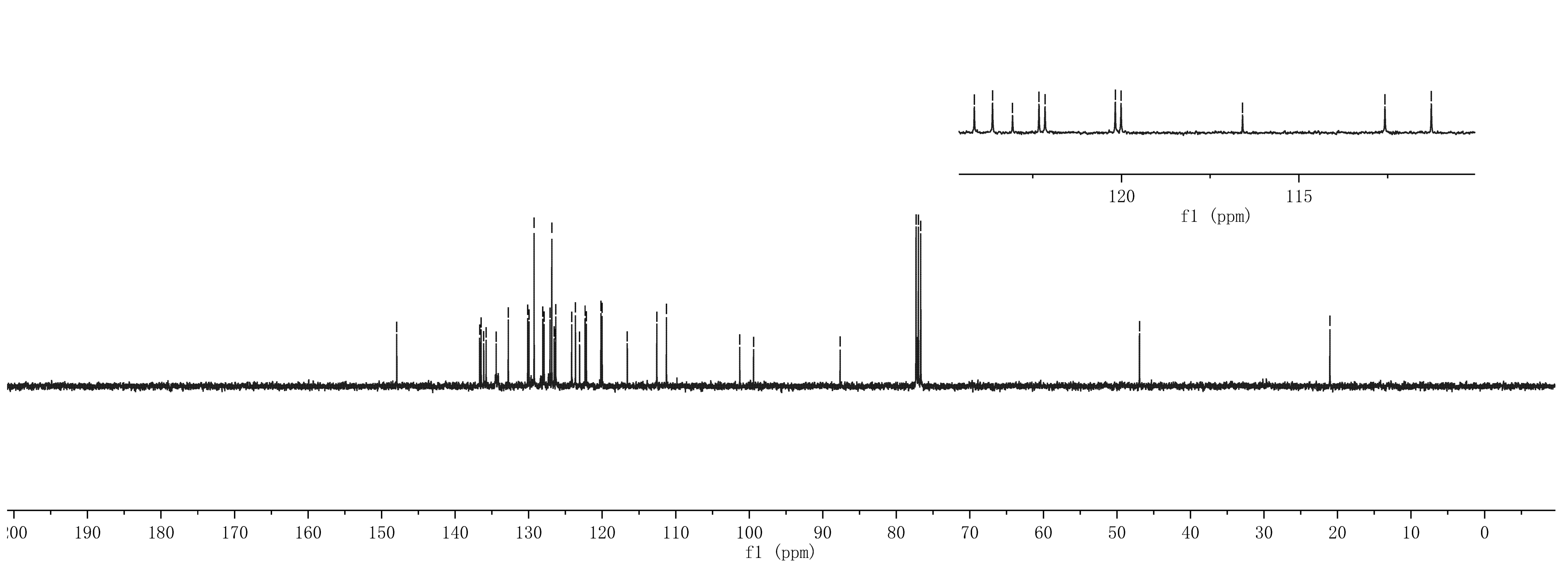

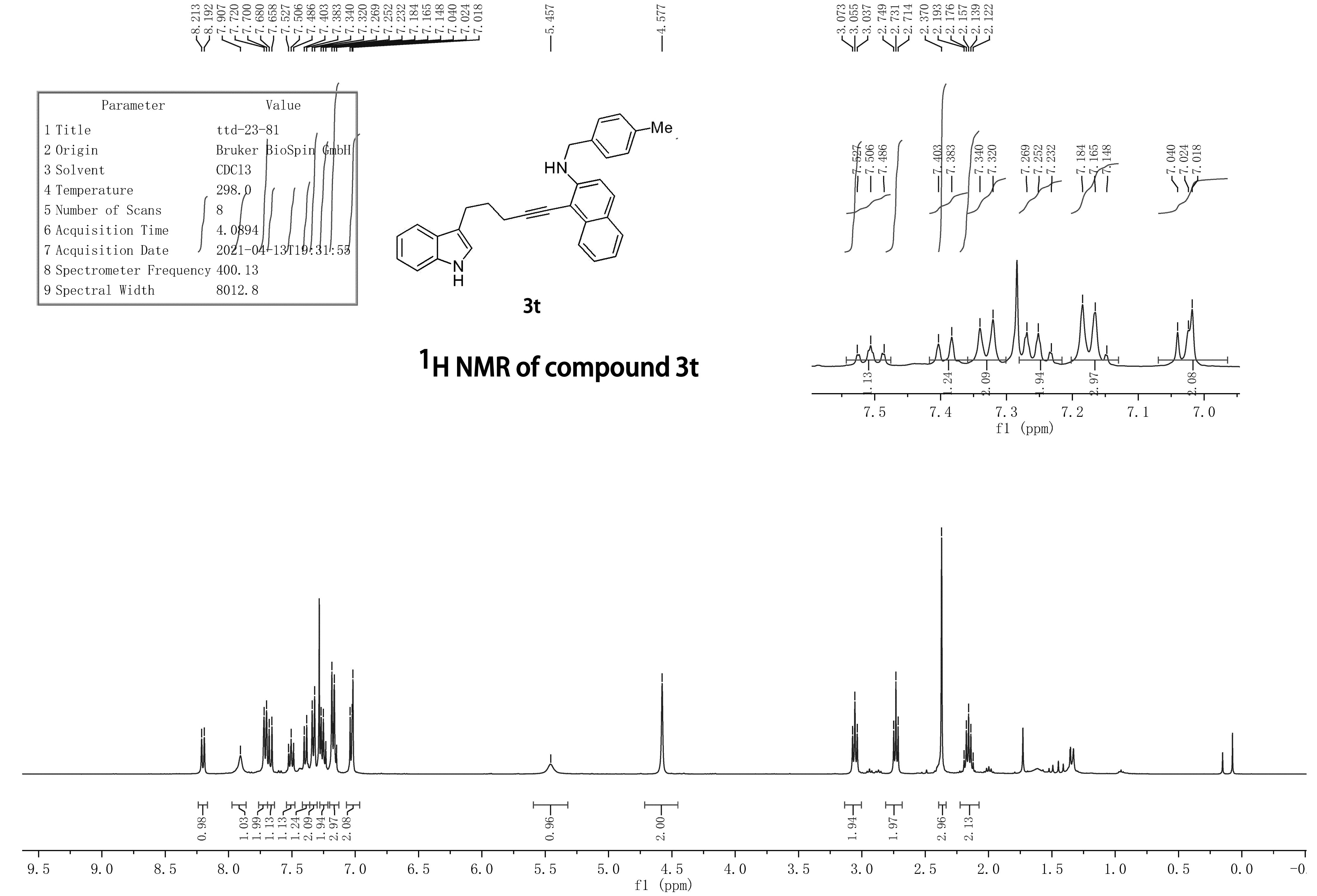

^{1}H NMR of compound 3t

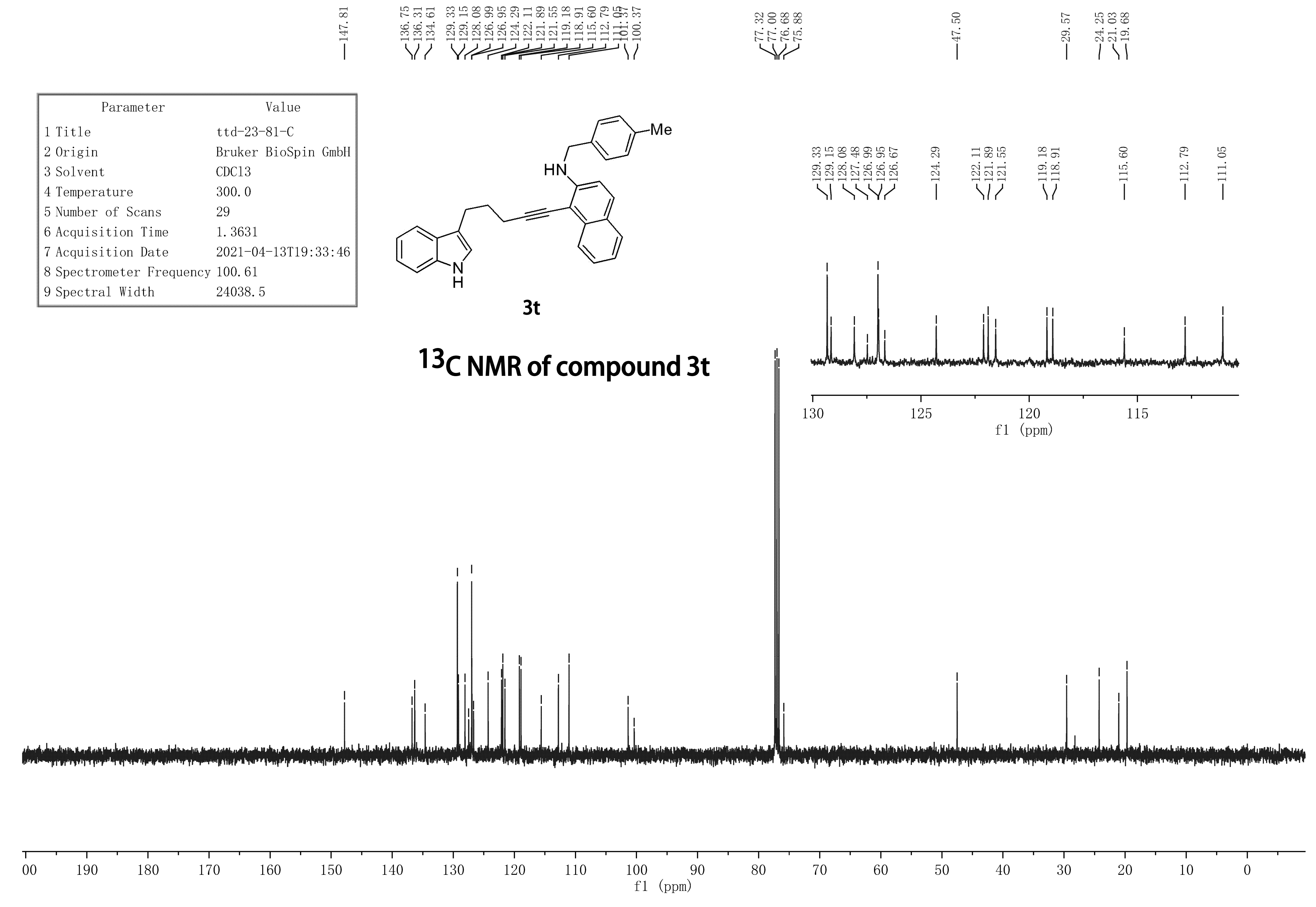

^{13}C NMR of compound 3t

Parameter	Value
1 Title	ttd-23-130
2 Origin	Bruker BioSpin GmbH
3 Solvent	CDCl3
4 Temperature	298.0
5 Number of Scans	9
6 Acquisition Time	4.0894
7 Acquisition Date	2021-05-07T09:42:05
8 Spectrometer Frequency	400.13
9 Spectral Width	8012.8

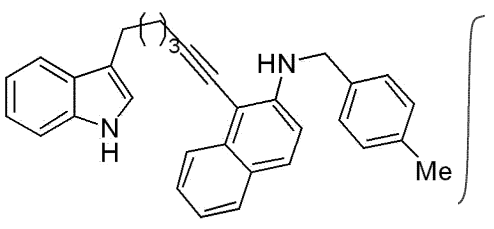

3u

^{1}H NMR of compound 3u

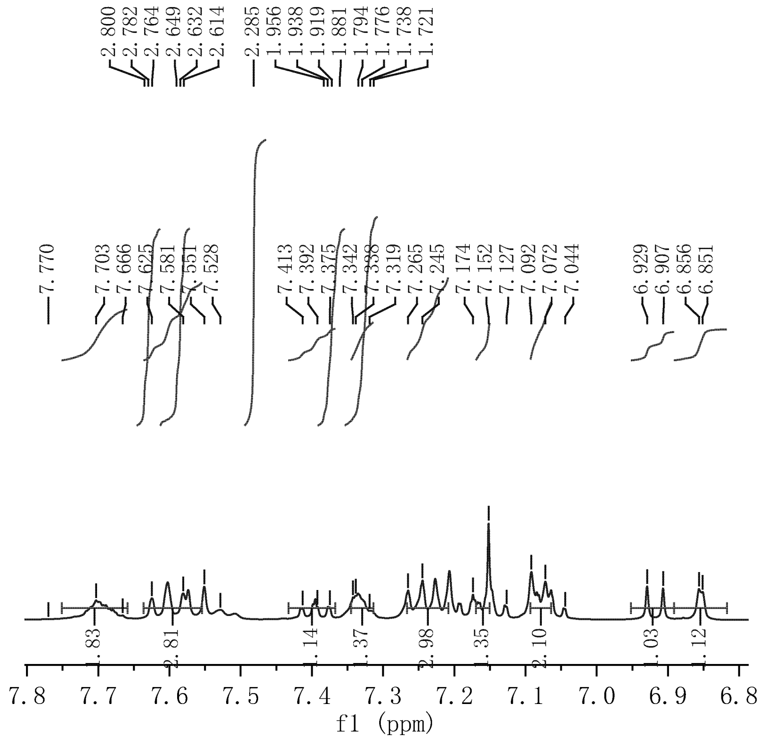

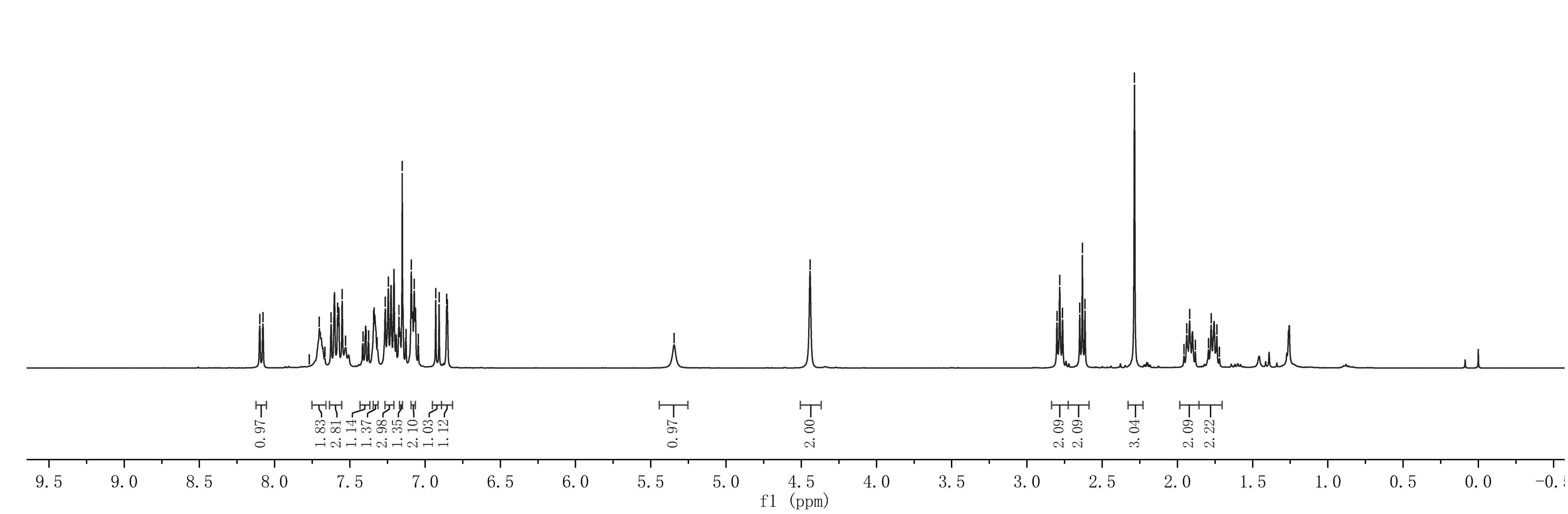

Parameter	Value
1 Title	ttd-23-130-C
2 Origin	Bruker BioSpin GmbH
3 Solvent	CDCl3
4 Temperature	300.0
5 Number of Scans	23
6 Acquisition Time	1.3631
7 Acquisition Date	2021-05-07T09:43:33
8 Spectrometer Frequency	100.61
9 Spectral Width	24038.5

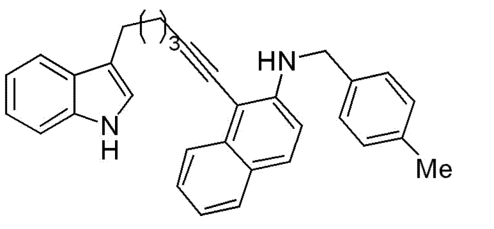

3u

^{13}C NMR of compound 3u

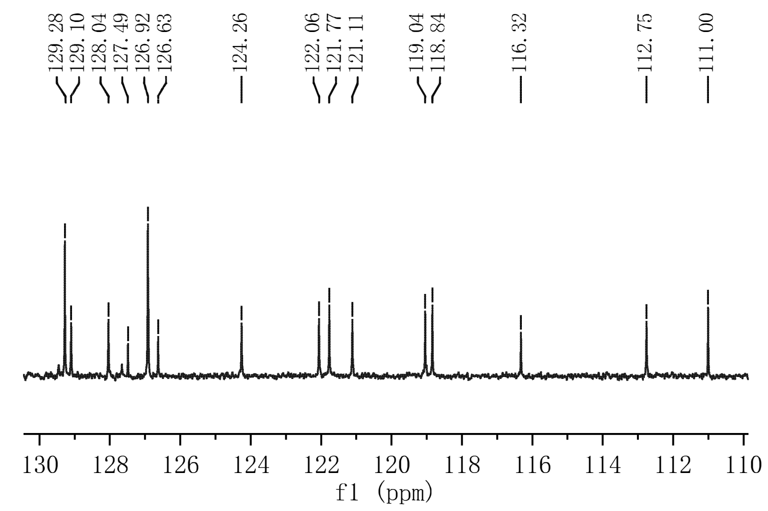

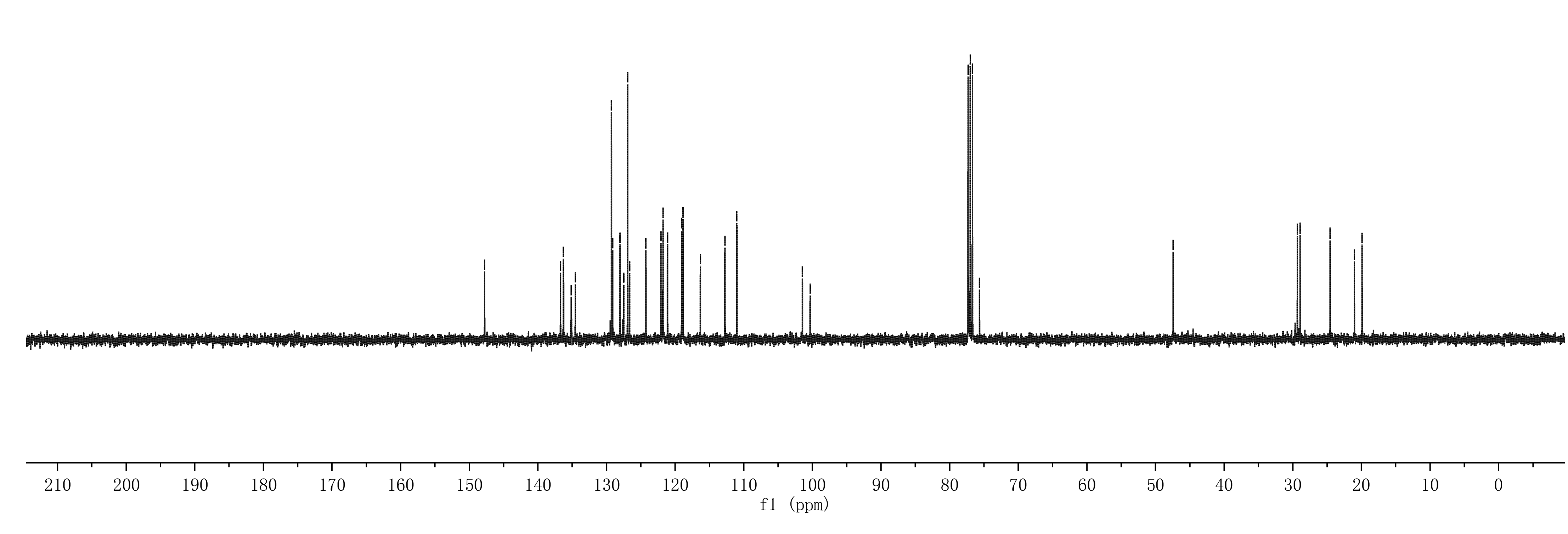

Parameter	Value
1 Title	ttd-21-87
2 Origin	Bruker BioSpin GmbH
3 Solvent	CDCl3
4 Temperature	298.5
5 Number of Scans	5
6 Acquisition Time	3.1719
7 Acquisition Date	2020-09-16T11:13:47
8 Spectrometer Frequency	500.17
9 Spectral Width	10330.6

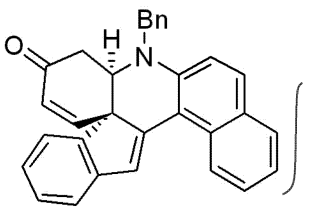

2a

^{1}H NMR of compound 2a

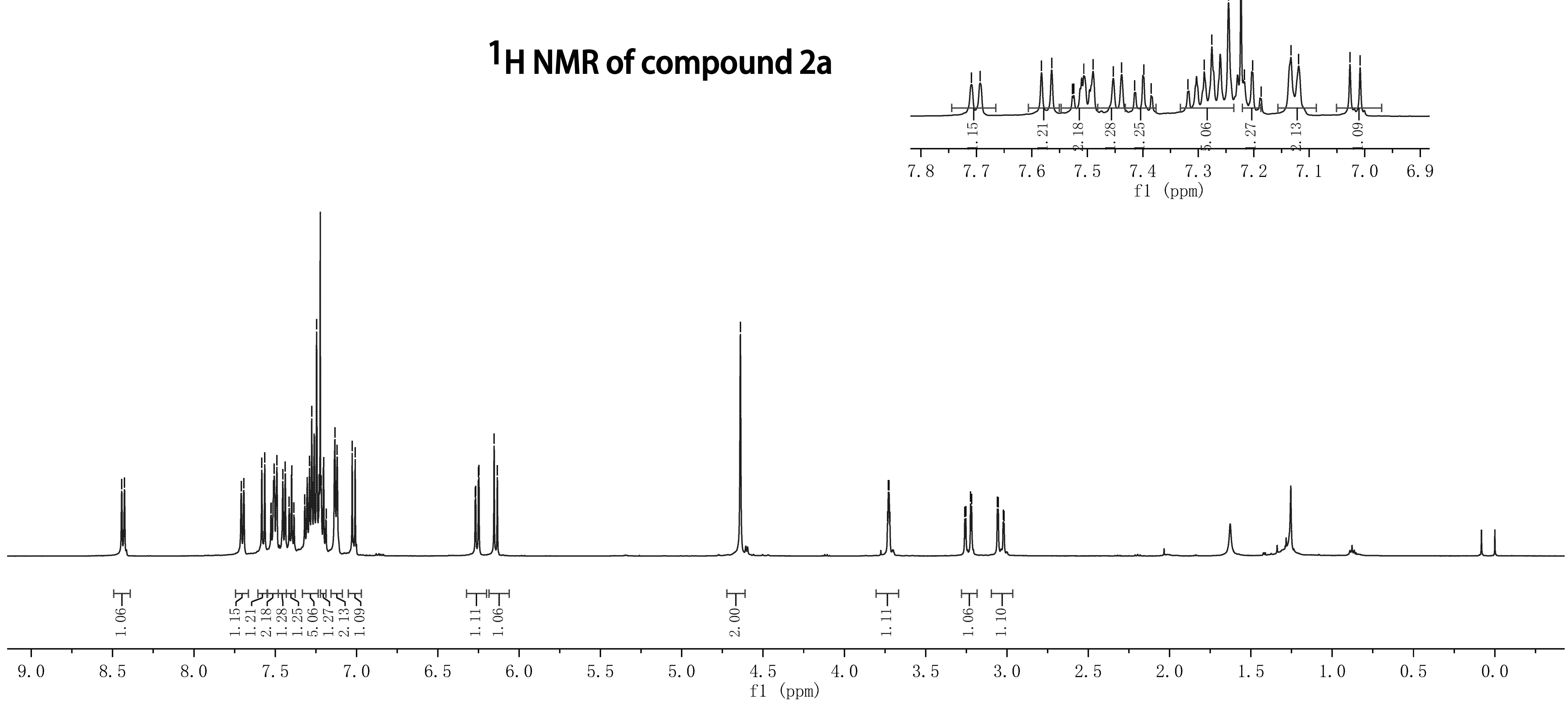

Parameter	Value
1 Title	ttd-21-87-C
2 Origin	Bruker BioSpin GmbH
3 Solvent	CDCl3
4 Temperature	298.8
5 Number of Scans	17
6 Acquisition Time	1.1010
7 Acquisition Date	2020-09-16T11:14:52
8 Spectrometer Frequency	125.77
9 Spectral Width	29761.9

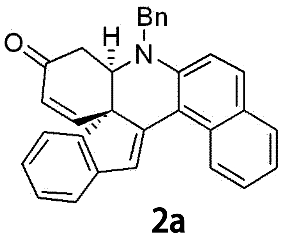

2a

^{13}C NMR of compound 2a

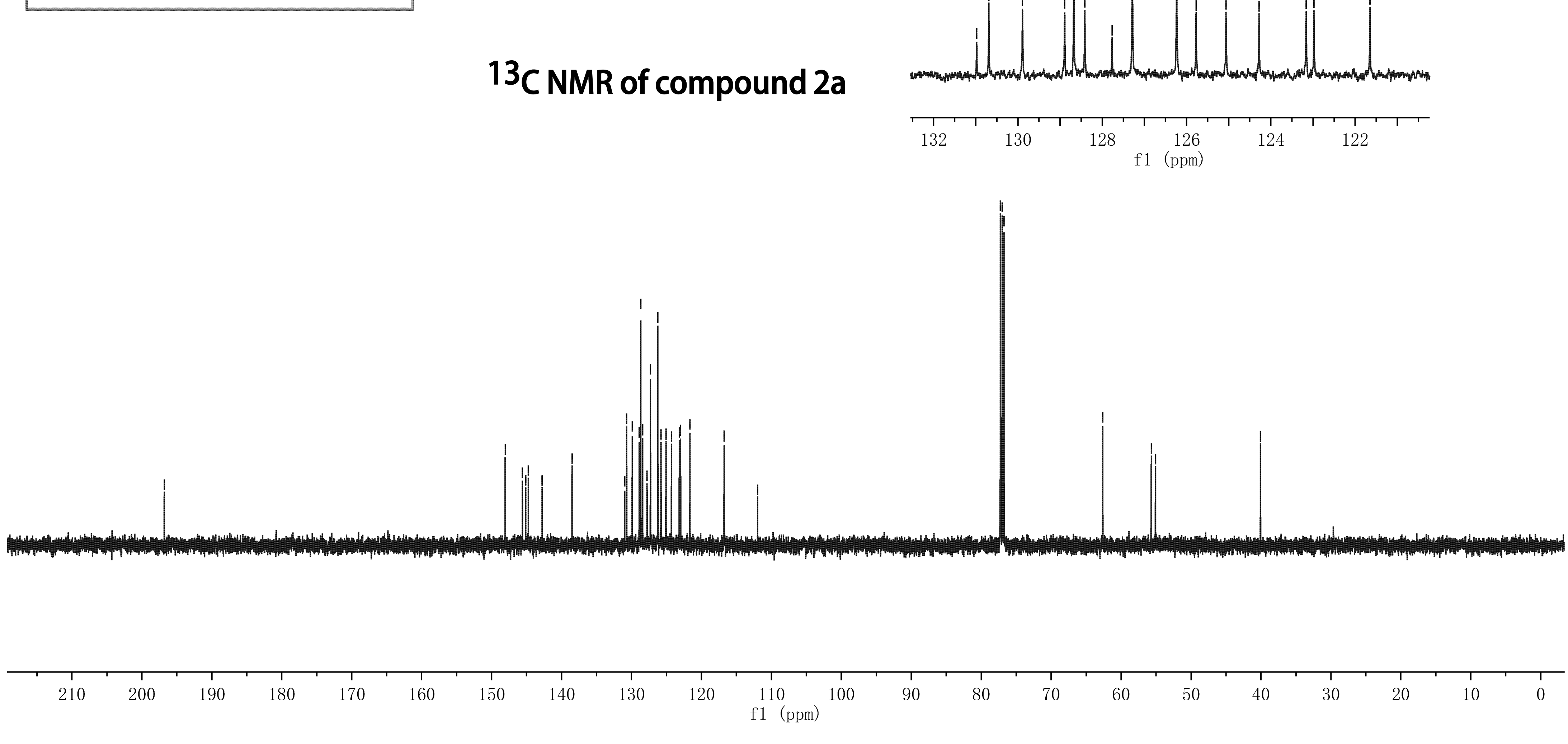

Parameter	Value
1 Title	ttd-21-87-C135
2 Origin	Bruker BioSpin GmbH
3 Solvent	CDCl3
4 Temperature	299.0
5 Number of Scans	8
6 Acquisition Time	1.1010
7 Acquisition Date	2020-09-16T11:16:15
8 Spectrometer Frequency	125.77
9 Spectral Width	29761.9

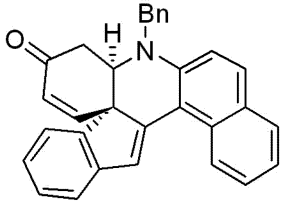

2a

^{13}C dept135 NMR of compound 2a

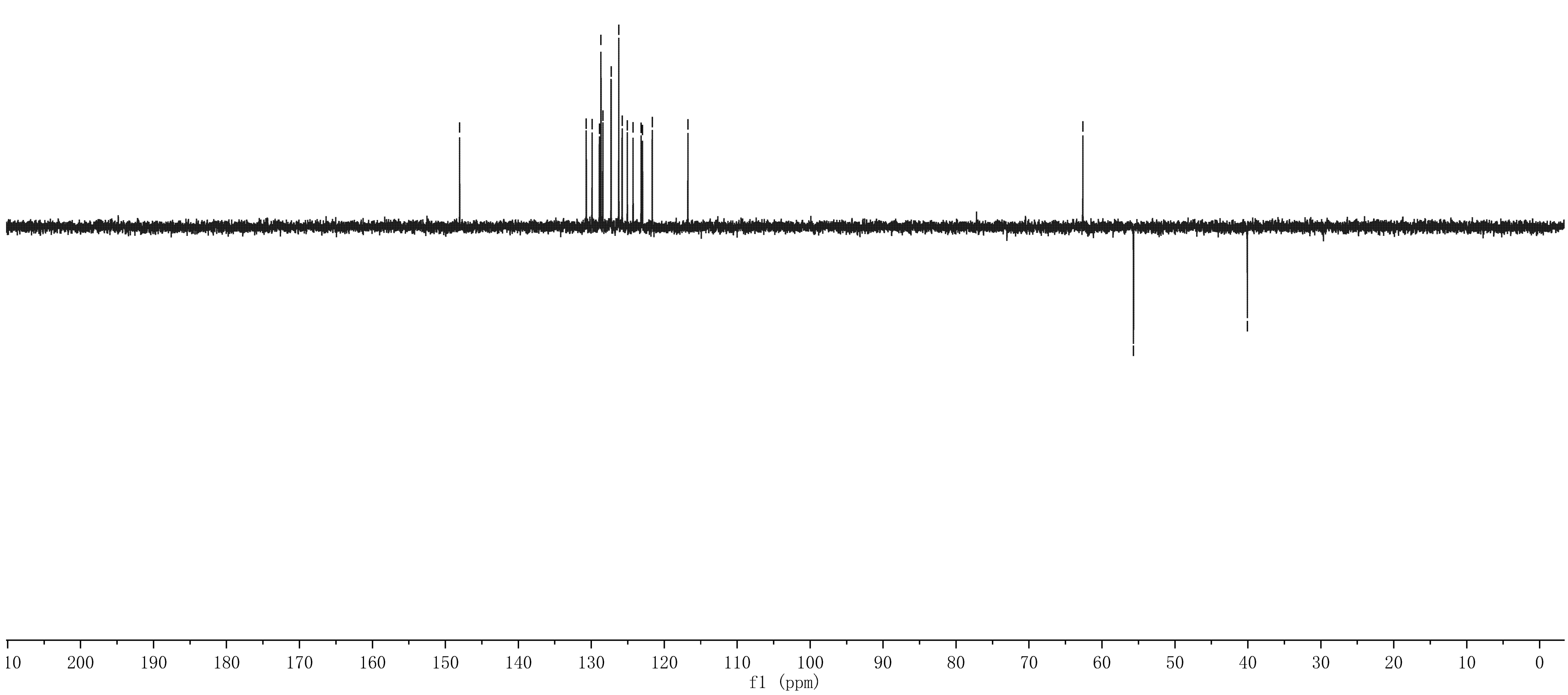

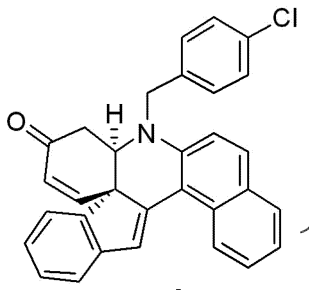

2b

1H NMR of compound 2b

Parameter	Value
1 Title	ttd-21-100
2 Origin	Bruker BioSpin GmbH
3 Solvent	CDCl3
4 Temperature	298.0
5 Number of Scans	7
6 Acquisition Time	4.0894
7 Acquisition Date	2020-09-21T16:03:25
8 Spectrometer Frequency	400.13
9 Spectral Width	8012.8

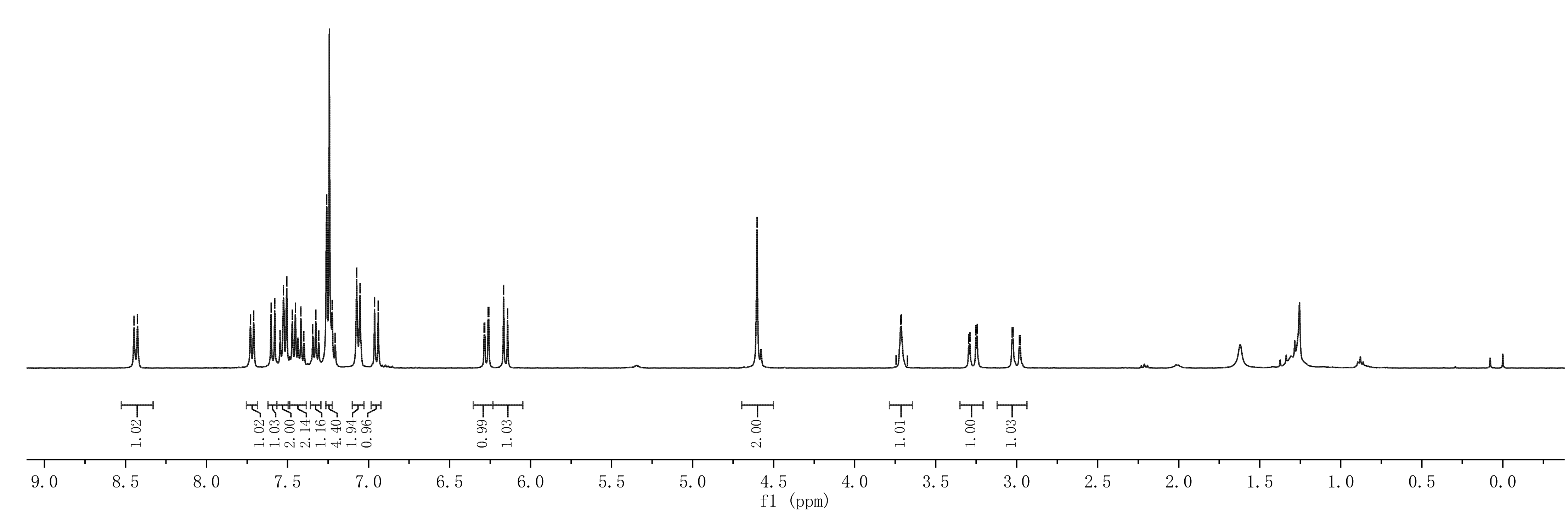

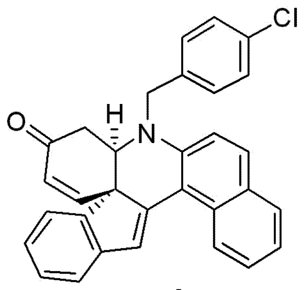

2b

13C NMR of compound 2b

Parameter	Value
1 Title	ttd-21-100-C
2 Origin	Bruker BioSpin GmbH
3 Solvent	CDCl3
4 Temperature	300.0
5 Number of Scans	49
6 Acquisition Time	1.3631
7 Acquisition Date	2020-09-21T16:06:00
8 Spectrometer Frequency	100.61
9 Spectral Width	24038.5

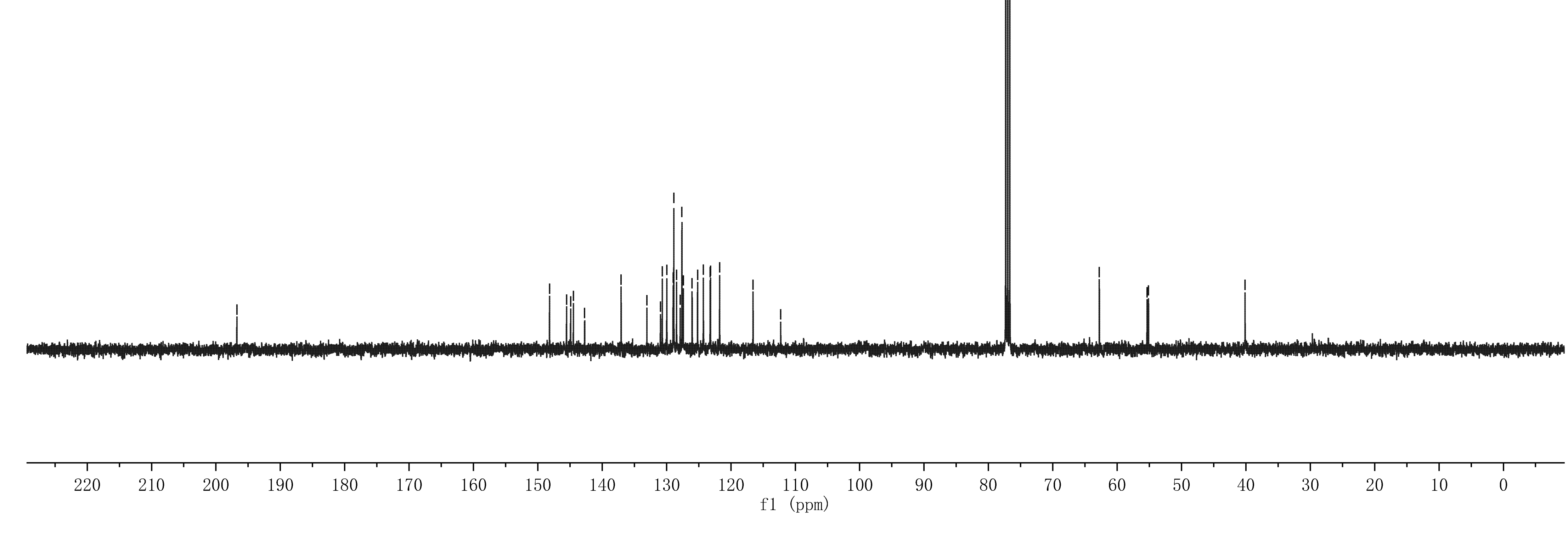

Parameter	Value
1 Title	ttd-21-165
2 Origin	Bruker BioSpin GmbH
3 Solvent	CDCl3
4 Temperature	298.6
5 Number of Scans	6
6 Acquisition Time	3.1719
7 Acquisition Date	2020-10-08T15:50:10
8 Spectrometer Frequency	500.17
9 Spectral Width	10330.6

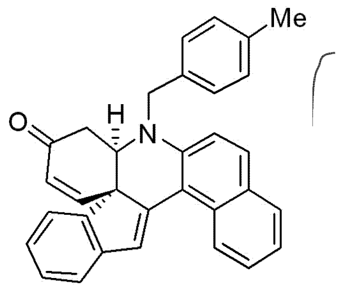

2c

^{1}H NMR of compound 2c

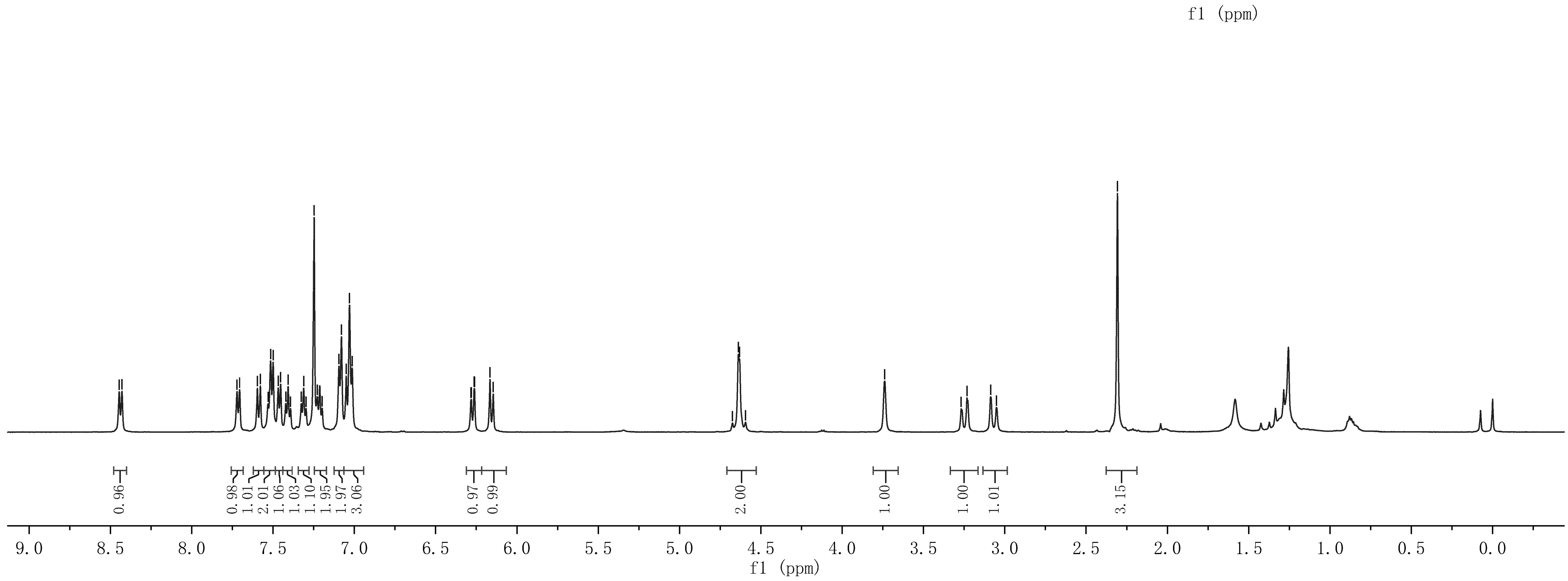

Parameter	Value
1 Title	ttd-21-165-C
2 Origin	Bruker BioSpin GmbH
3 Solvent	CDCl3
4 Temperature	298.8
5 Number of Scans	65
6 Acquisition Time	1.1010
7 Acquisition Date	2020-10-08T15:52:31
8 Spectrometer Frequency	125.77
9 Spectral Width	29761.9

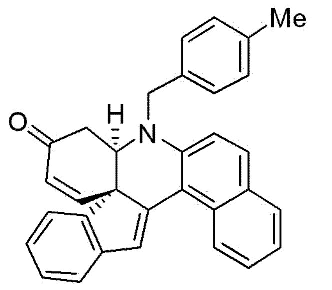

2c

^{13}C NMR of compound 2c

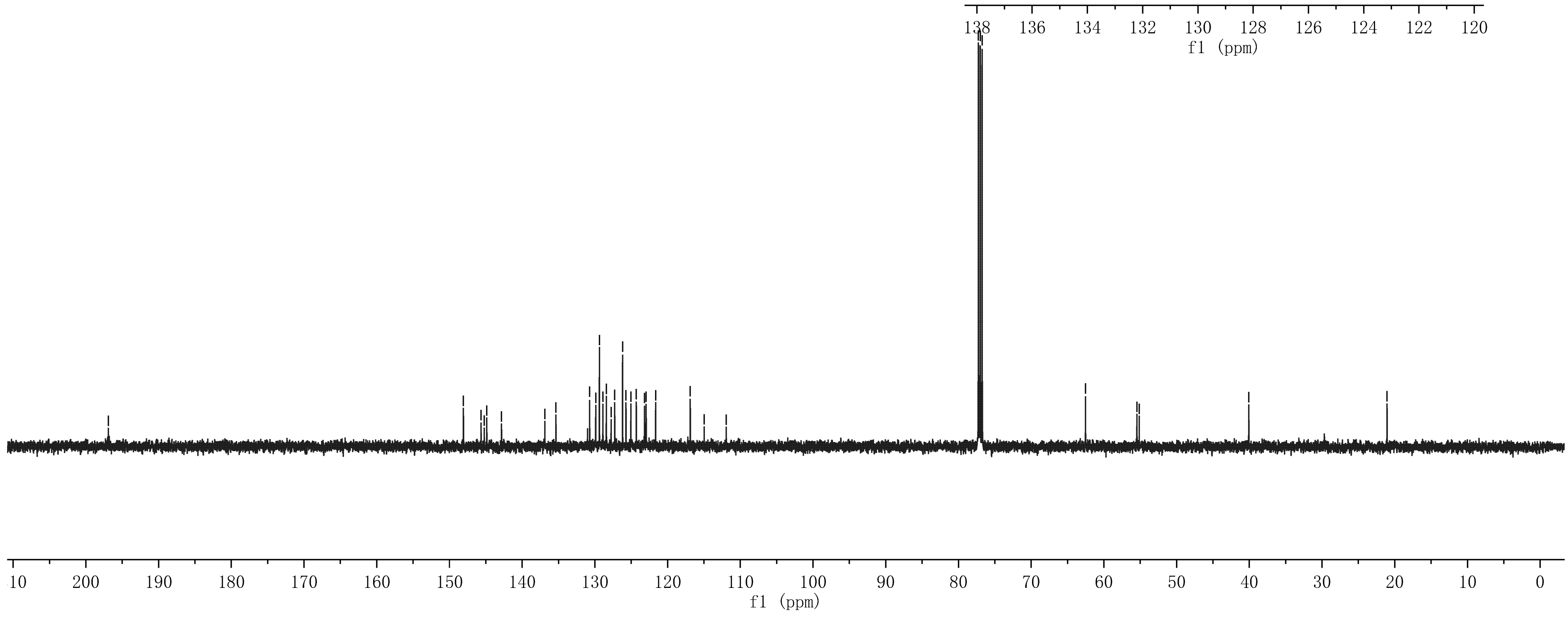

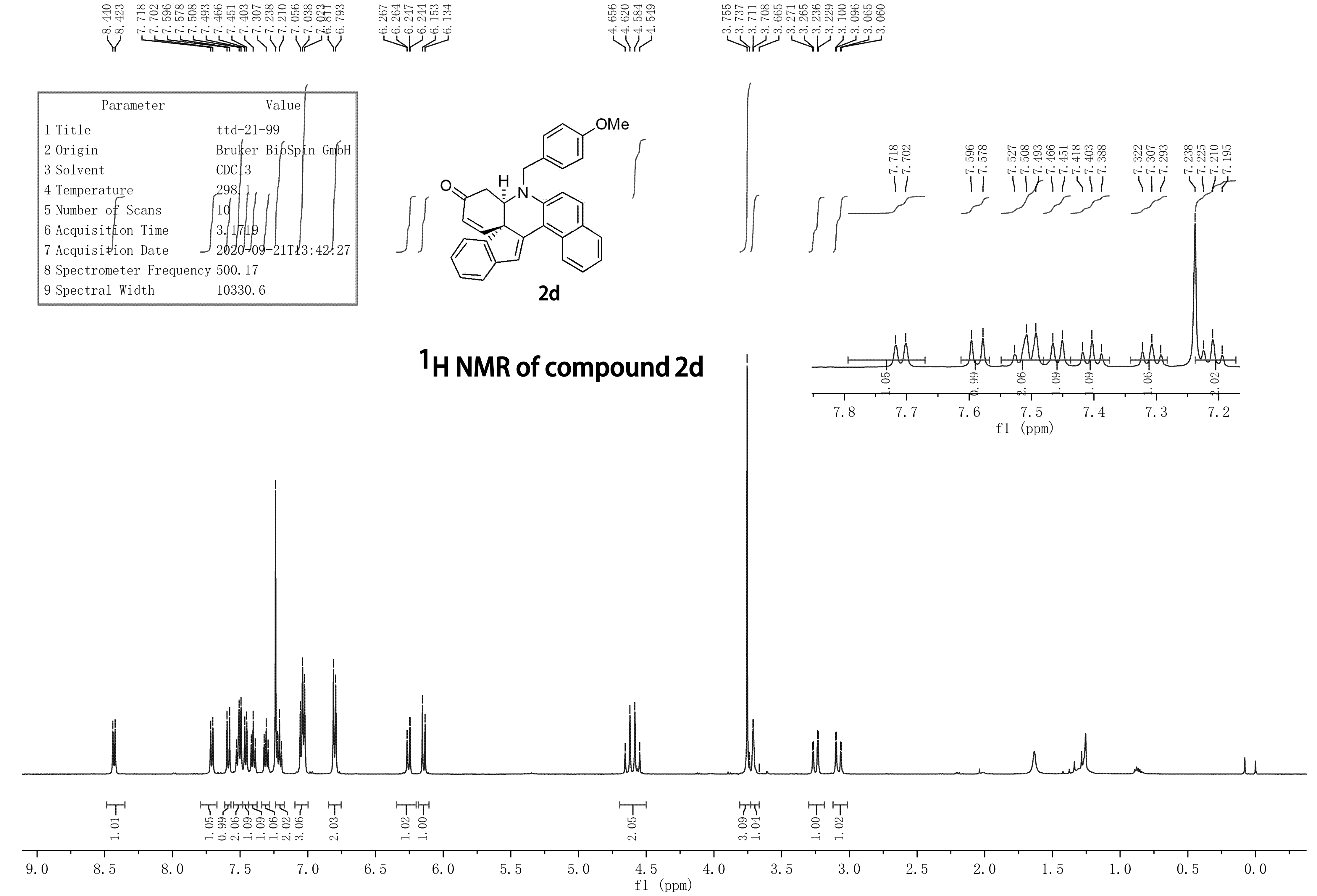
8.440 8.423 7.718 7.702 7.596 7.578 7.508 7.493 7.466 7.451 7.403 7.307 7.238 7.210 7.056 7.038 7.023 6.811 6.793
6.267 6.264 6.247 6.244 6.153 6.134
4.656 4.620 4.584 4.549
3.755 3.737 3.711 3.708 3.665 3.271 3.265 3.236 3.229 3.100 3.096 3.065 3.060
Parameter Value
1 Title ttd-21-99
2 Origin Bruker BioSpin GmbH
3 Solvent CDCl3
4 Temperature 298.1
5 Number of Scans 10
6 Acquisition Time 3.1719
7 Acquisition Date 2020-09-21T13:42:27
8 Spectrometer Frequency 500.17
9 Spectral Width 10330.6
OMe
O
H
N
2d
1H NMR of compound 2d
7.718 7.702 7.596 7.578 7.527 7.508 7.493 7.466 7.451 7.418 7.403 7.388 7.322 7.307 7.293 7.238 7.225 7.210 7.195
1.05 0.99 2.06 1.09 1.09 1.06 2.02
f1 (ppm)
1.01 1.05 0.99 2.06 1.09 1.09 1.06 2.02 3.06 2.03 1.02 1.00 2.05 3.09 1.04 1.00 1.02
f1 (ppm)

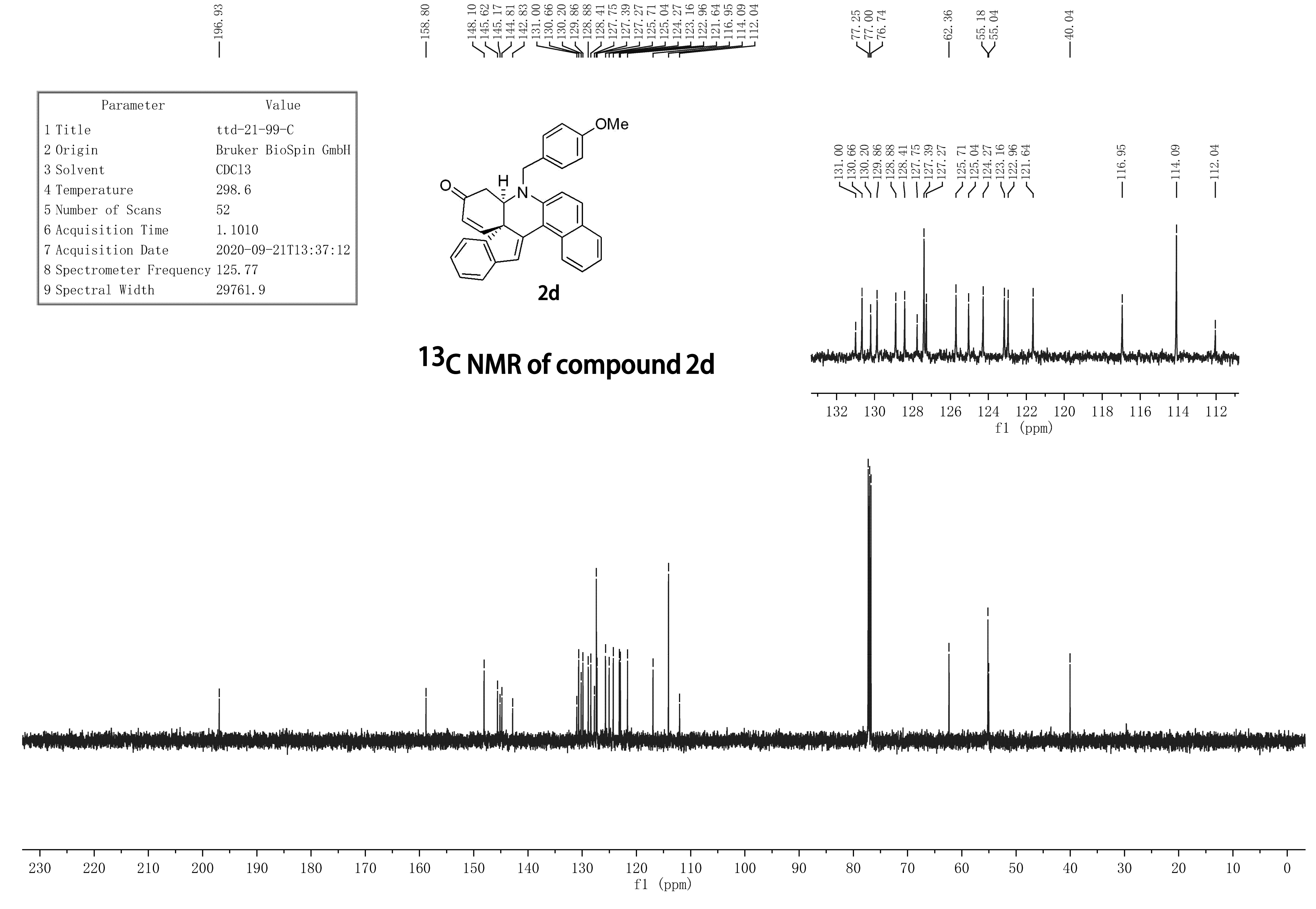
196.93
158.80
148.10 145.62 145.17 144.81 142.83 131.00 130.66 130.20 129.86 128.88 128.41 127.75 127.39 127.27 125.71 125.04 124.27 123.16 122.96 121.64 116.95 114.09 112.04
77.25 77.00 76.74
62.36
55.18 55.04
40.04
Parameter Value
1 Title ttd-21-99-C
2 Origin Bruker BioSpin GmbH
3 Solvent CDCl3
4 Temperature 298.6
5 Number of Scans 52
6 Acquisition Time 1.1010
7 Acquisition Date 2020-09-21T13:37:12
8 Spectrometer Frequency 125.77
9 Spectral Width 29761.9
OMe
O
H
N
2d
13C NMR of compound 2d
f1 (ppm)
f1 (ppm)

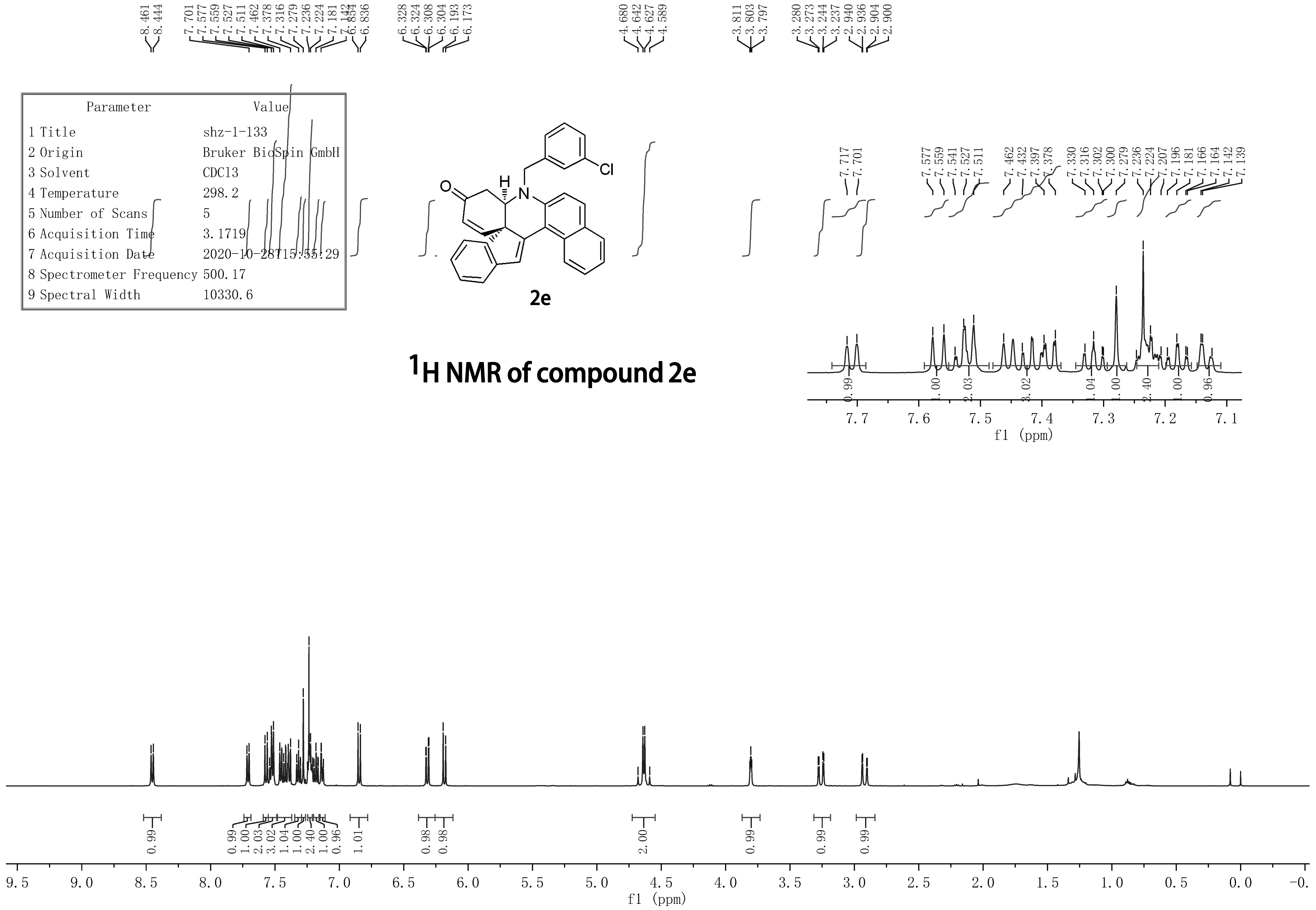
8.461
8.444
7.701
7.577
7.559
7.527
7.511
7.462
7.378
7.316
7.279
7.236
7.224
7.181
7.142
6.854
6.836
6.328
6.324
6.308
6.304
6.193
6.173
4.680
4.642
4.627
4.589
3.811
3.803
3.797
3.280
3.273
3.244
3.237
2.940
2.936
2.904
2.900
Parameter Value
1 Title shz-1-133
2 Origin Bruker BioSpin GmbH
3 Solvent CDCl3
4 Temperature 298.2
5 Number of Scans 5
6 Acquisition Time 3.1719
7 Acquisition Date 2020-10-28T15:55:29
8 Spectrometer Frequency 500.17
9 Spectral Width 10330.6
Cl
O
H
N
2e
1H NMR of compound 2e
f1 (ppm)

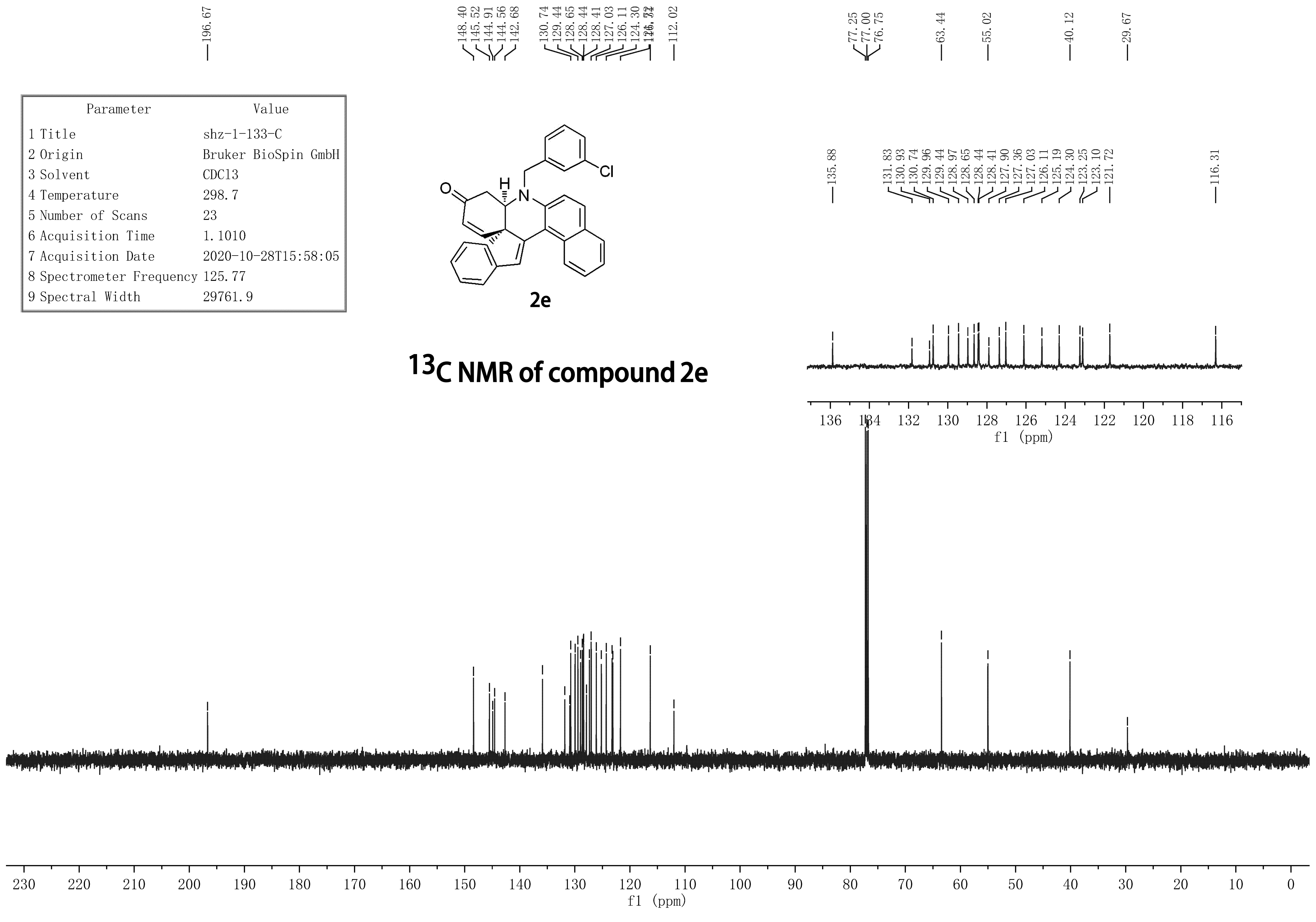
196.67
148.40
145.52
144.91
144.56
142.68
130.74
129.44
128.65
128.44
128.41
127.03
126.11
124.30
116.31
112.02
77.25
77.00
76.75
63.44
55.02
40.12
29.67
Parameter Value
1 Title shz-1-133-C
2 Origin Bruker BioSpin GmbH
3 Solvent CDCl3
4 Temperature 298.7
5 Number of Scans 23
6 Acquisition Time 1.1010
7 Acquisition Date 2020-10-28T15:58:05
8 Spectrometer Frequency 125.77
9 Spectral Width 29761.9
Cl
O
H
N
2e
13C NMR of compound 2e
135.88
131.83
130.93
130.74
129.96
129.44
128.97
128.65
128.44
128.41
127.90
127.36
127.03
126.11
125.19
124.30
123.25
123.10
121.72
116.31
f1 (ppm)

Parameter	Value
1 Title	ttd-21-125
2 Origin	Bruker BioSpin GmbH
3 Solvent	CDCl3
4 Temperature	298.0
5 Number of Scans	7
6 Acquisition Time	4.0894
7 Acquisition Date	2020-09-27T16:29:01
8 Spectrometer Frequency	400.13
9 Spectral Width	8012.8

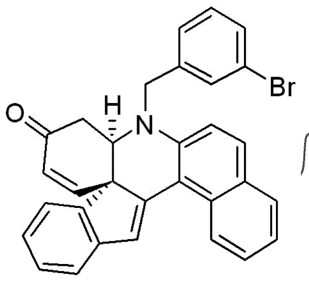

2f

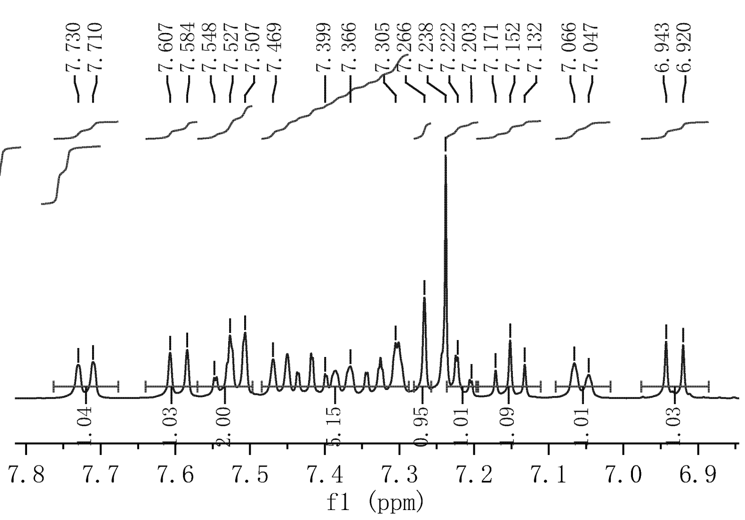

^{1}H NMR of compound 2f

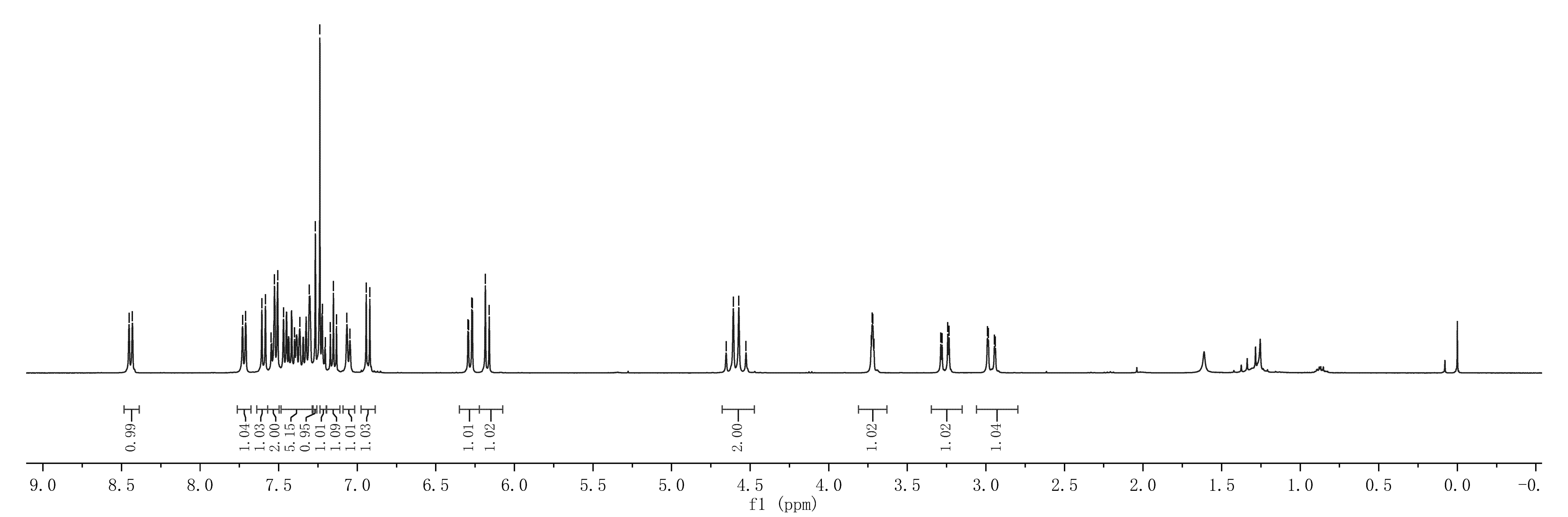

Parameter	Value
1 Title	ttd-21-125-C
2 Origin	Bruker BioSpin GmbH
3 Solvent	CDCl3
4 Temperature	300.0
5 Number of Scans	42
6 Acquisition Time	1.3631
7 Acquisition Date	2020-09-27T16:32:47
8 Spectrometer Frequency	100.61
9 Spectral Width	24038.5

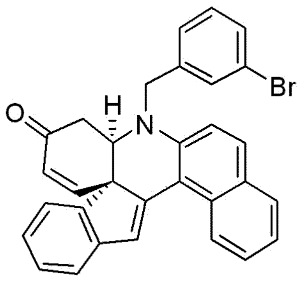

2f

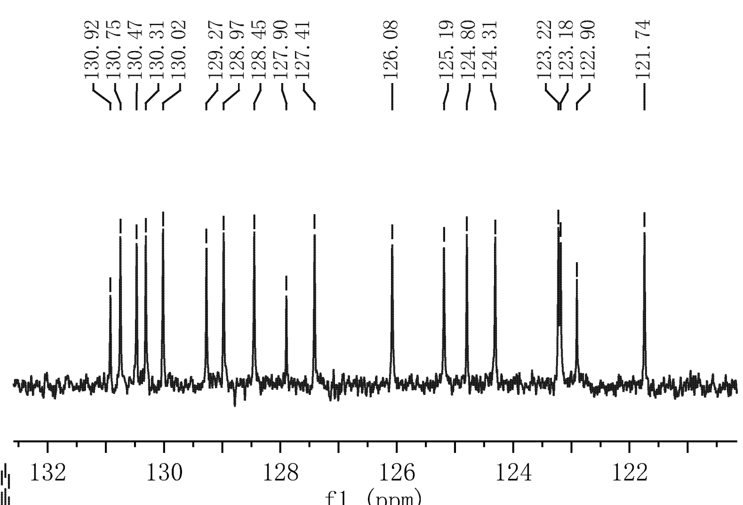

^{13}C NMR of compound 2f

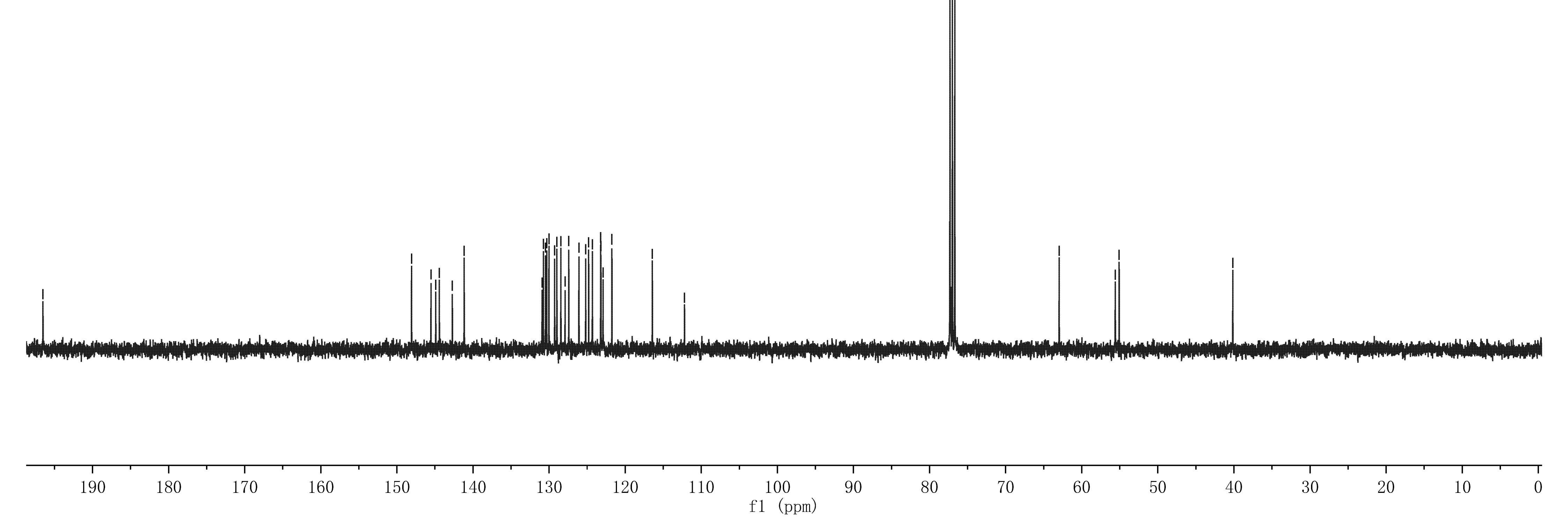

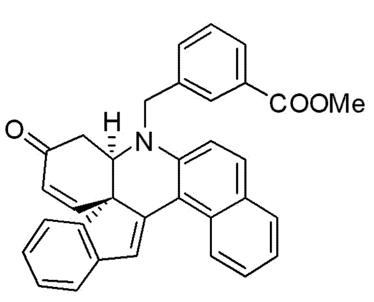

2g

^{1}H NMR of compound 2g

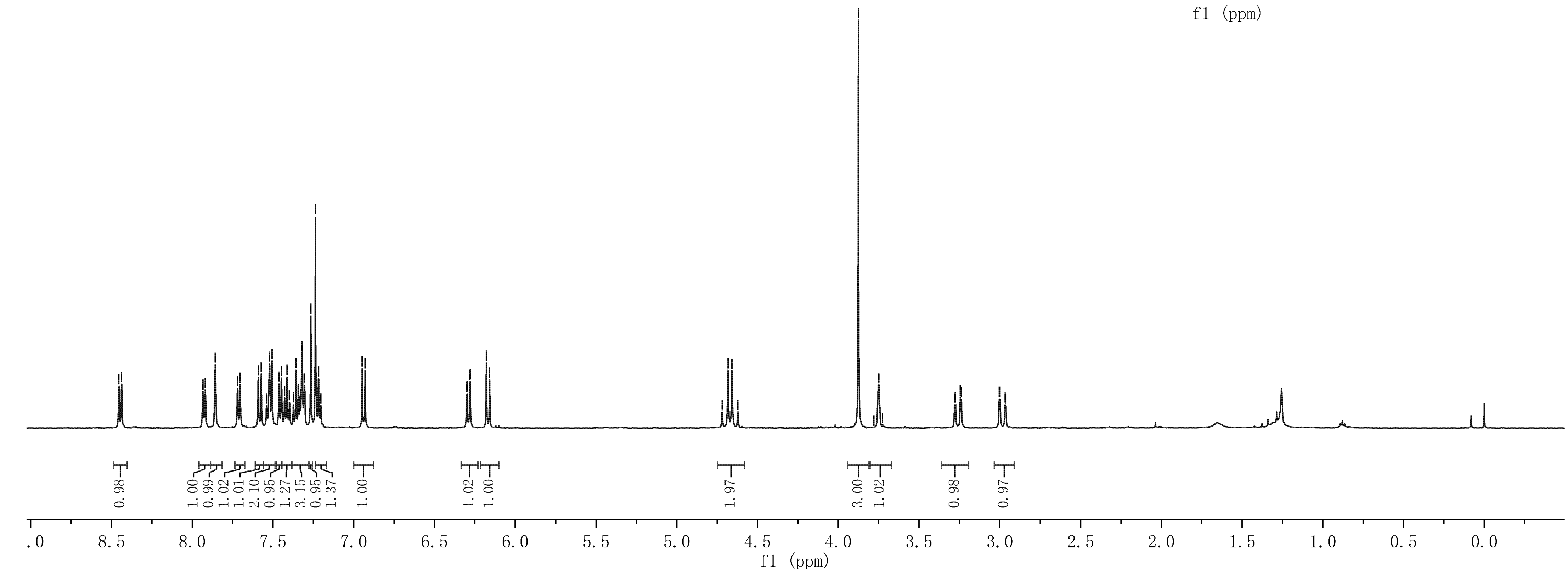

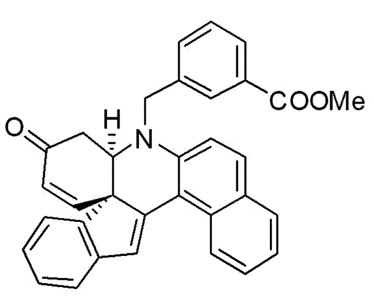

2g

^{13}C NMR of compound 2g

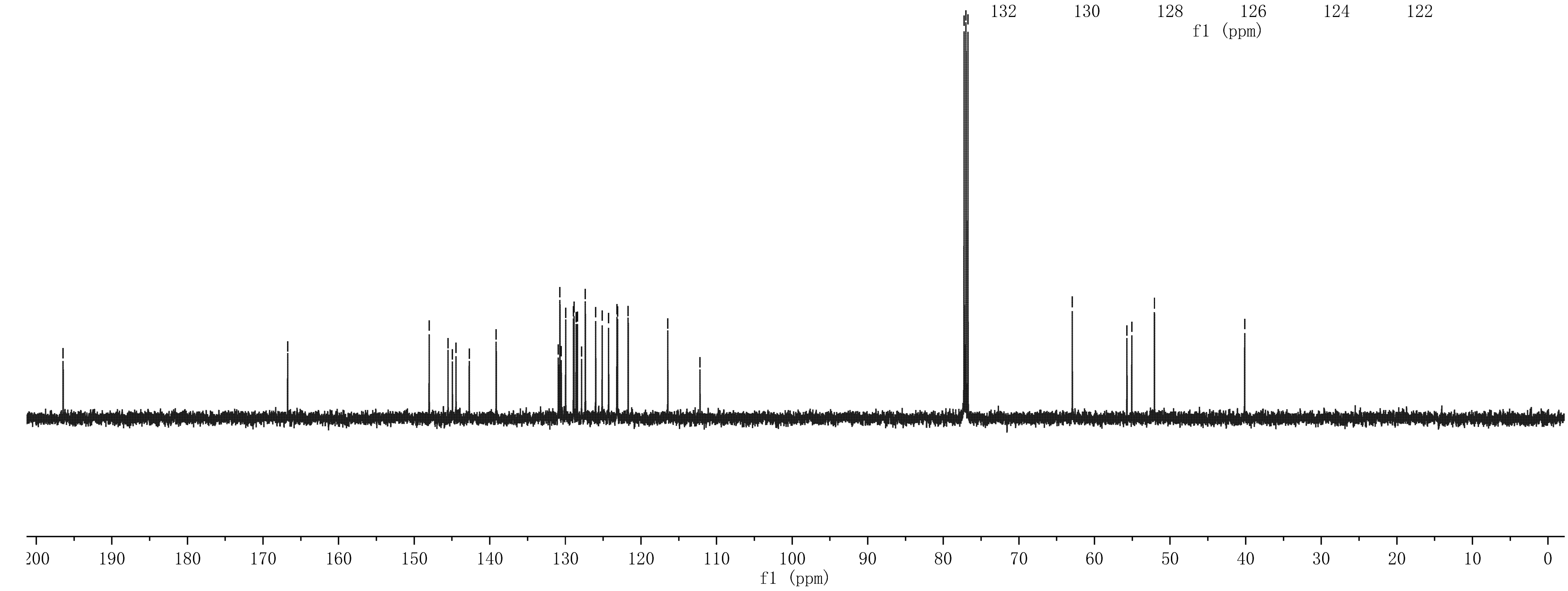

Parameter	Value
1 Title	shz-1-137-H
2 Origin	Bruker BioSpin GmbH
3 Solvent	CDCl3
4 Temperature	298.6
5 Number of Scans	9
6 Acquisition Time	3.1719
7 Acquisition Date	2020-10-31T15:56:29
8 Spectrometer Frequency	500.17
9 Spectral Width	10330.6

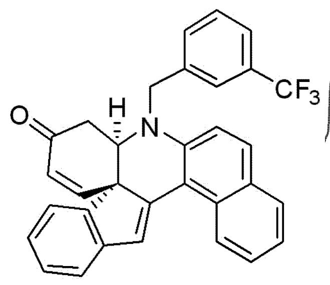

2h

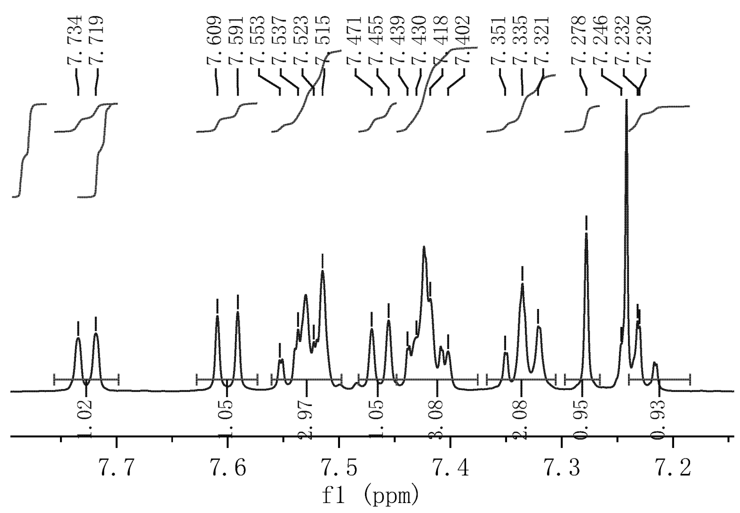

^{1}H NMR of compound 2h

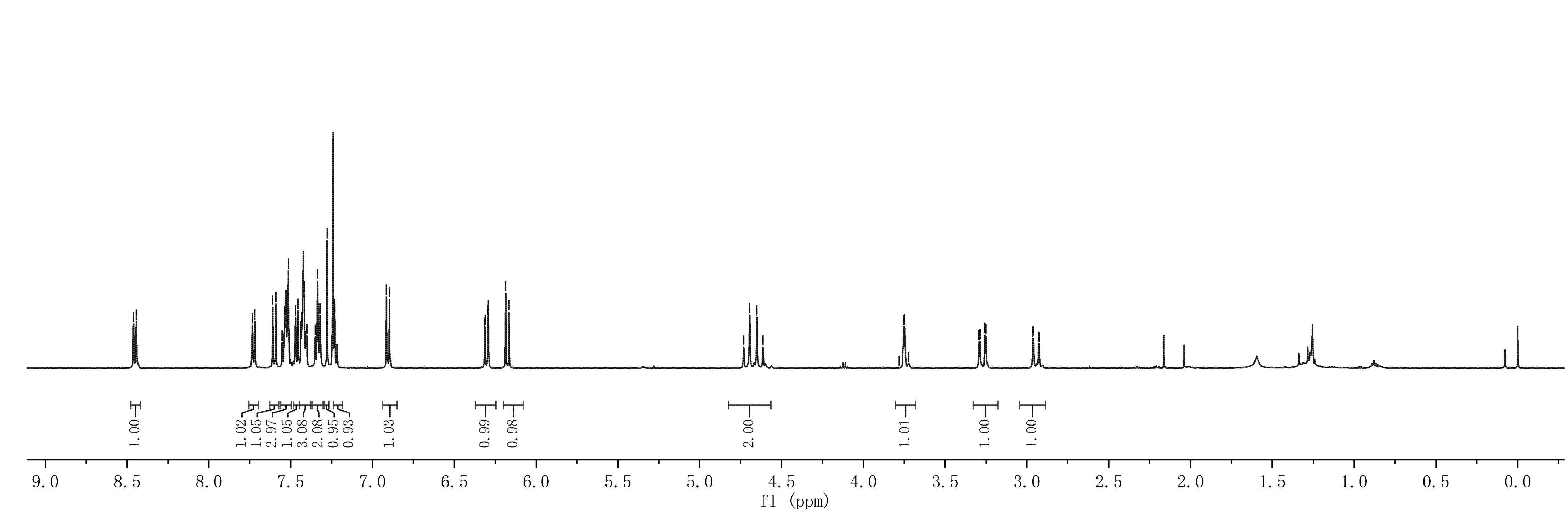

Parameter	Value
1 Title	shz-1-137-C
2 Origin	Bruker BioSpin GmbH
3 Solvent	CDCl3
4 Temperature	298.9
5 Number of Scans	53
6 Acquisition Time	1.1010
7 Acquisition Date	2020-10-31T15:58:56
8 Spectrometer Frequency	125.77
9 Spectral Width	29761.9

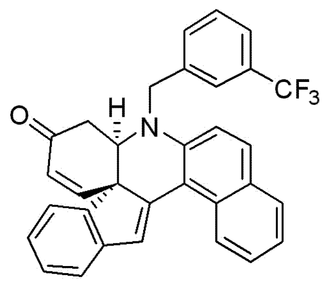

2h

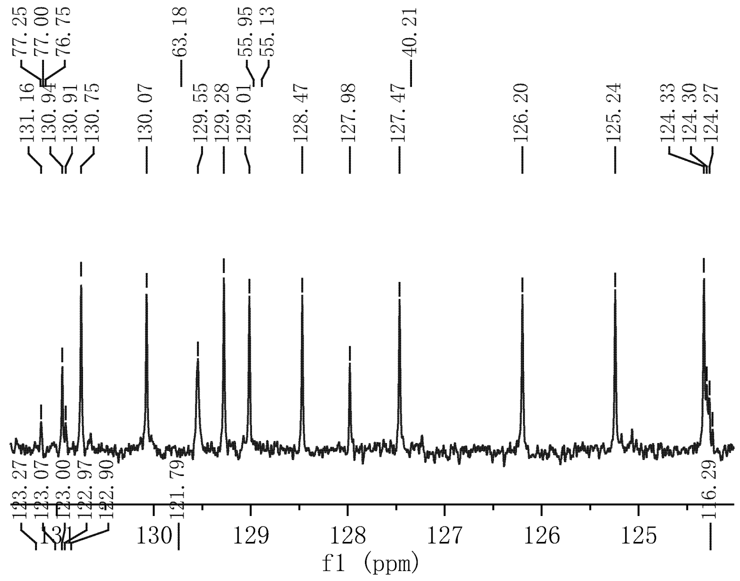

^{13}C NMR of compound 2h

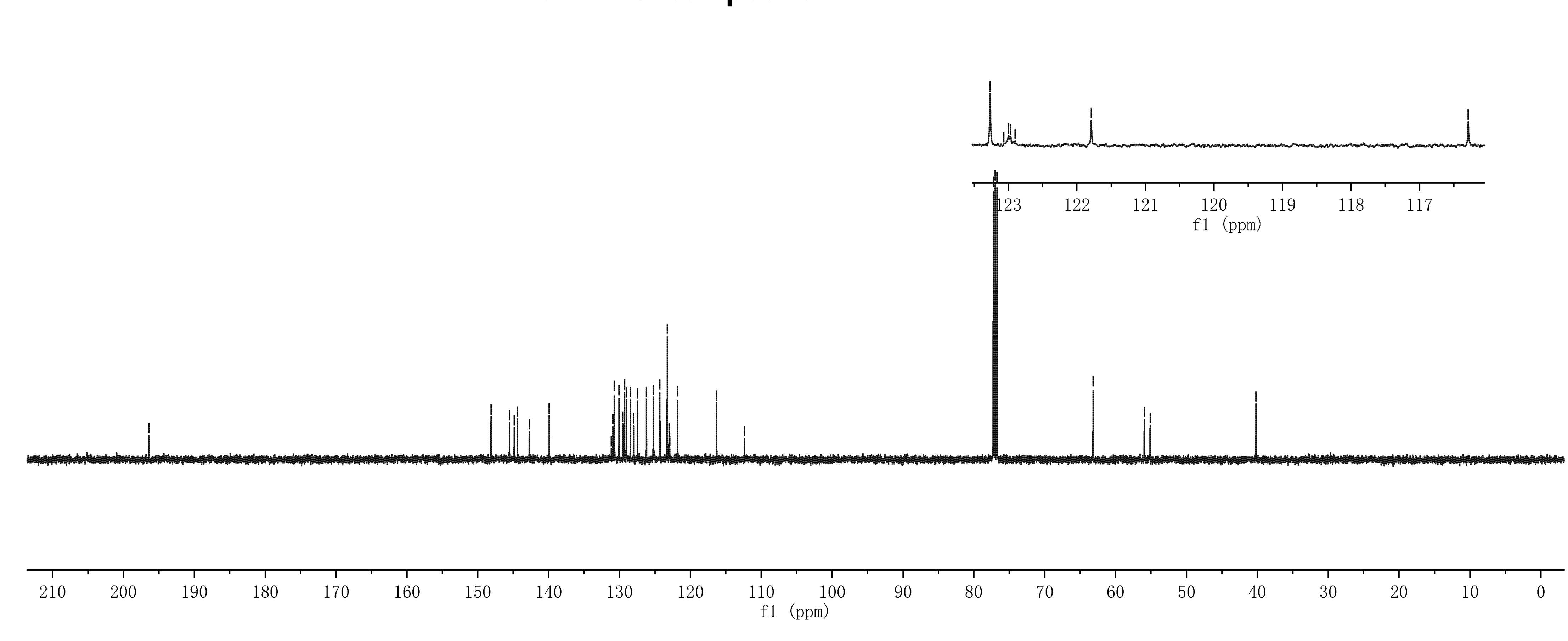

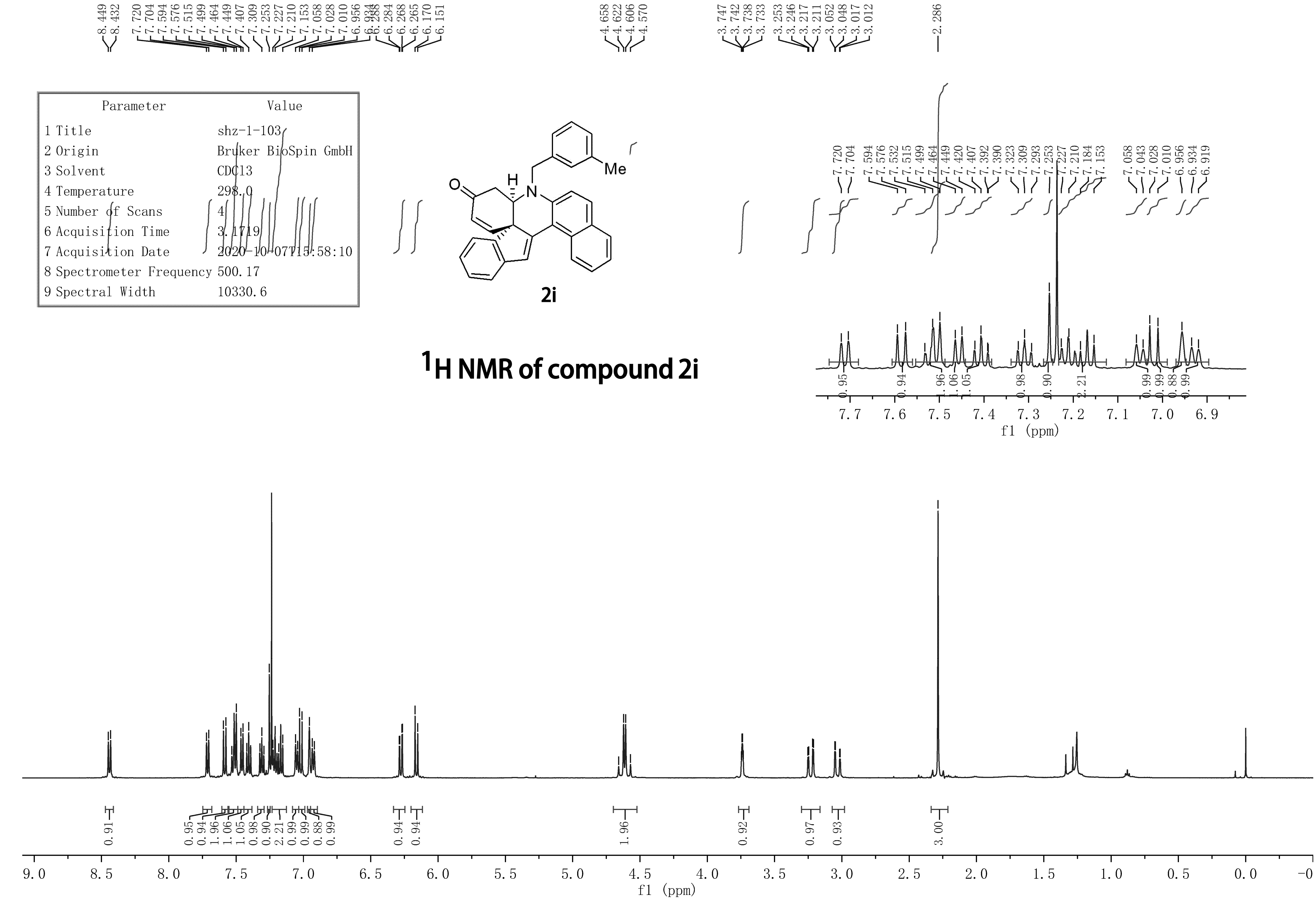

Parameter	Value
1 Title	shz-1-103
2 Origin	Bruker BioSpin GmbH
3 Solvent	CDCl3
4 Temperature	298.0
5 Number of Scans	4
6 Acquisition Time	3.1719
7 Acquisition Date	2020-10-07T15:58:10
8 Spectrometer Frequency	500.17
9 Spectral Width	10330.6

2i

^{1}H NMR of compound 2i

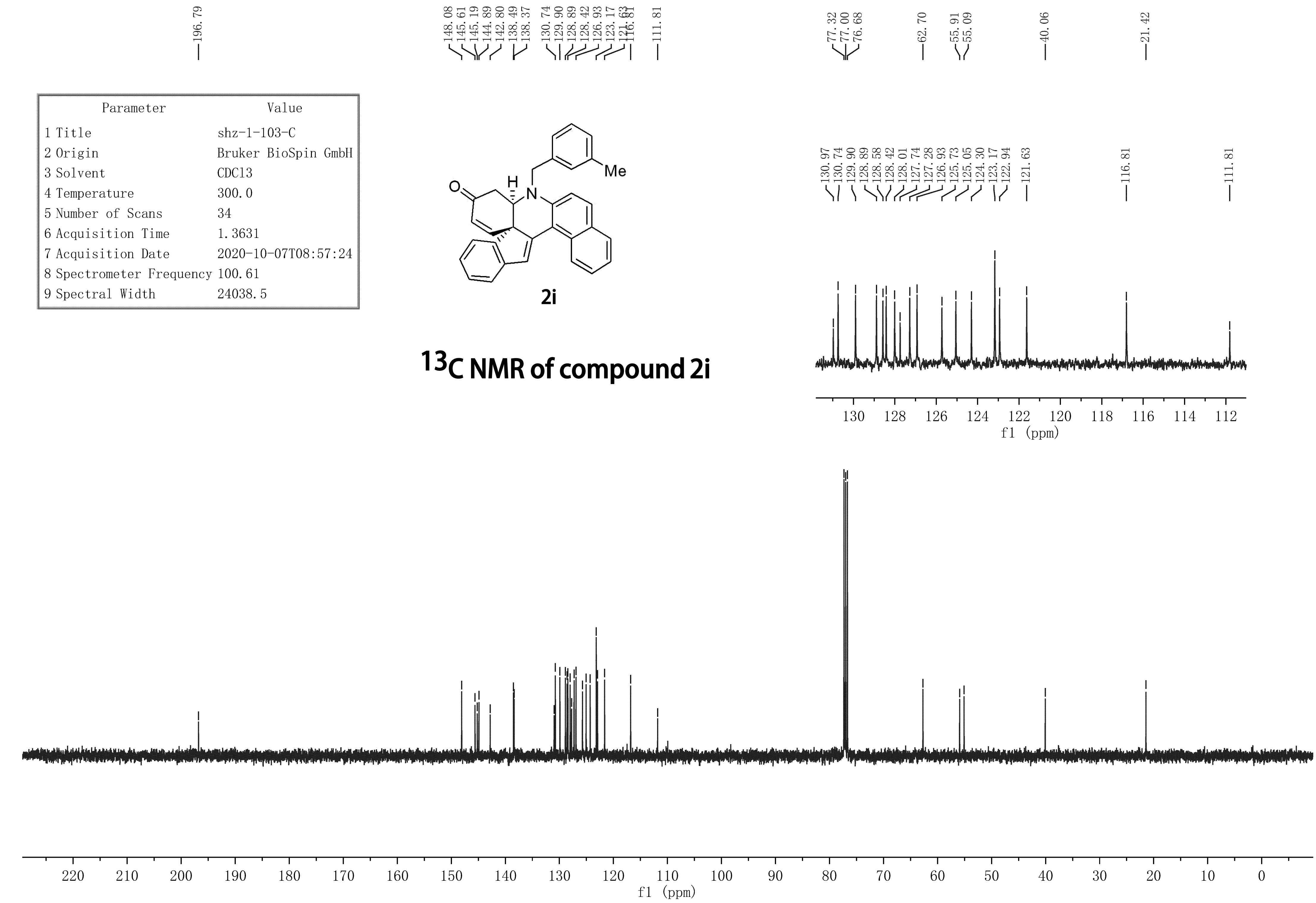

Parameter	Value
1 Title	shz-1-103-C
2 Origin	Bruker BioSpin GmbH
3 Solvent	CDCl3
4 Temperature	300.0
5 Number of Scans	34
6 Acquisition Time	1.3631
7 Acquisition Date	2020-10-07T08:57:24
8 Spectrometer Frequency	100.61
9 Spectral Width	24038.5

2i

^{13}C NMR of compound 2i

Parameter	Value
1 Title	SHZ-1-129
2 Origin	Bruker BioSpin GmbH
3 Solvent	CDCl3
4 Temperature	299.0
5 Number of Scans	8
6 Acquisition Time	3.1719
7 Acquisition Date	2020-10-26T16:18:34
8 Spectrometer Frequency	500.17
9 Spectral Width	10330.6

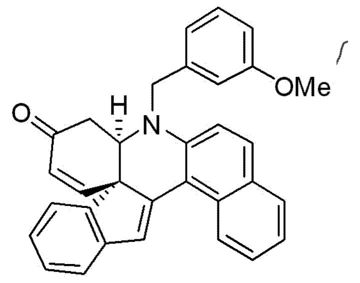

2j

^{1}H NMR of compound 2j

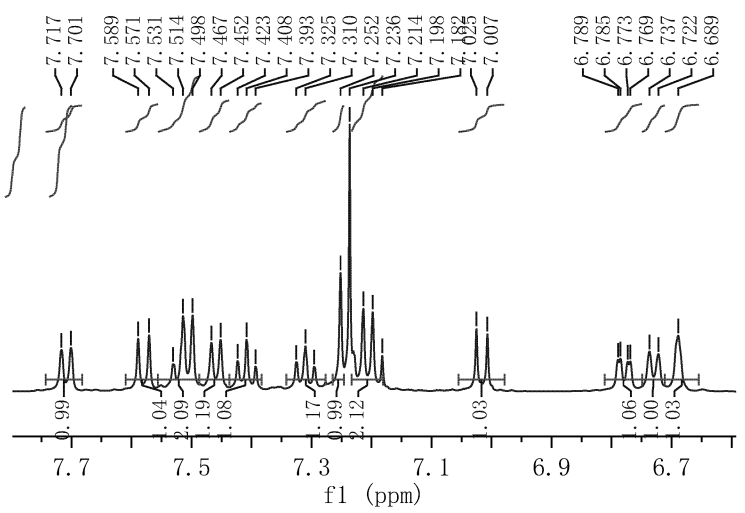

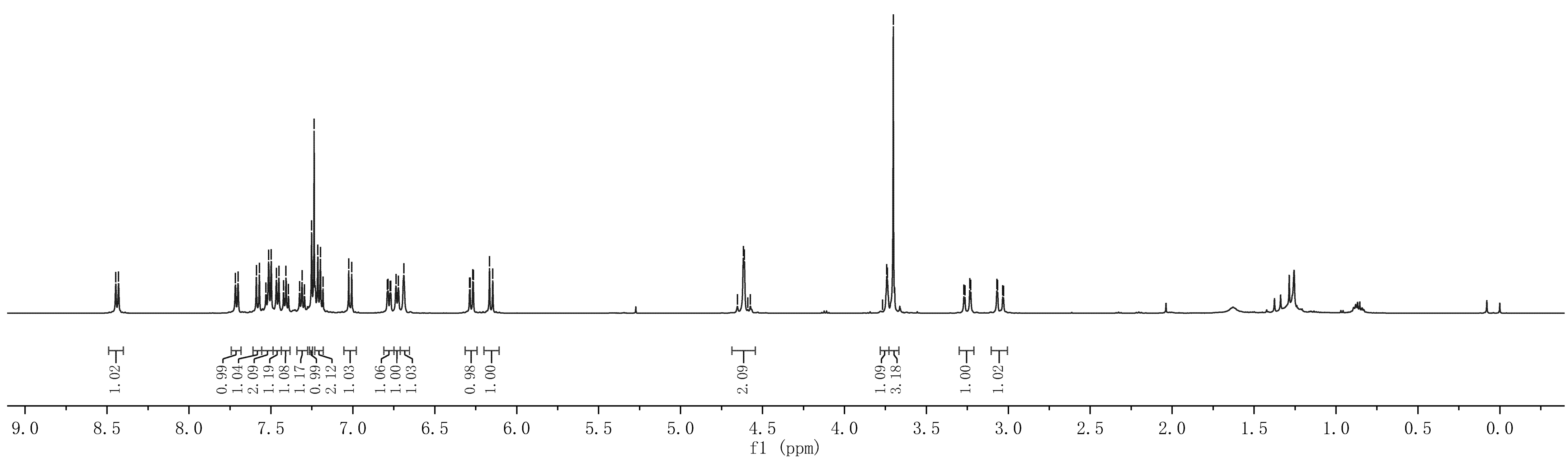

Parameter	Value
1 Title	SHZ-1-129-C
2 Origin	Bruker BioSpin GmbH
3 Solvent	CDCl3
4 Temperature	299.3
5 Number of Scans	48
6 Acquisition Time	1.1010
7 Acquisition Date	2020-10-26T16:19:34
8 Spectrometer Frequency	125.77
9 Spectral Width	29761.9

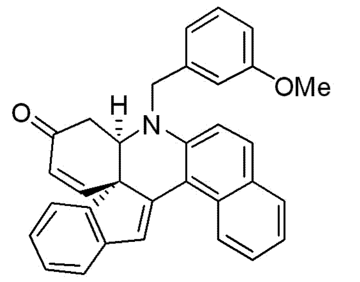

2j

^{13}C NMR of compound 2j

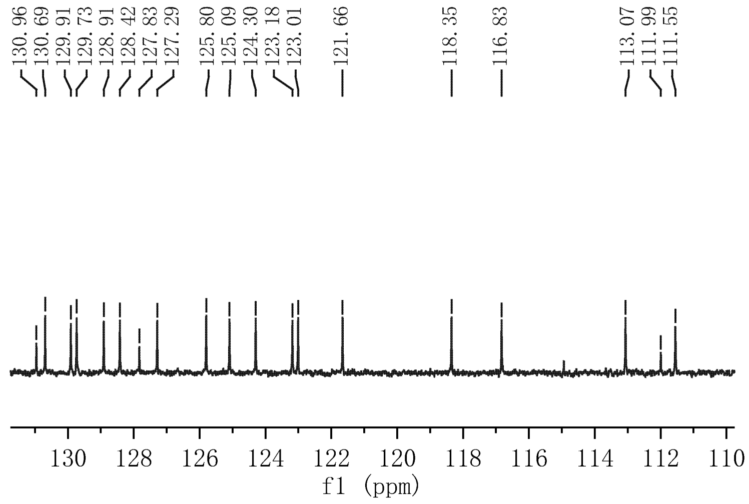

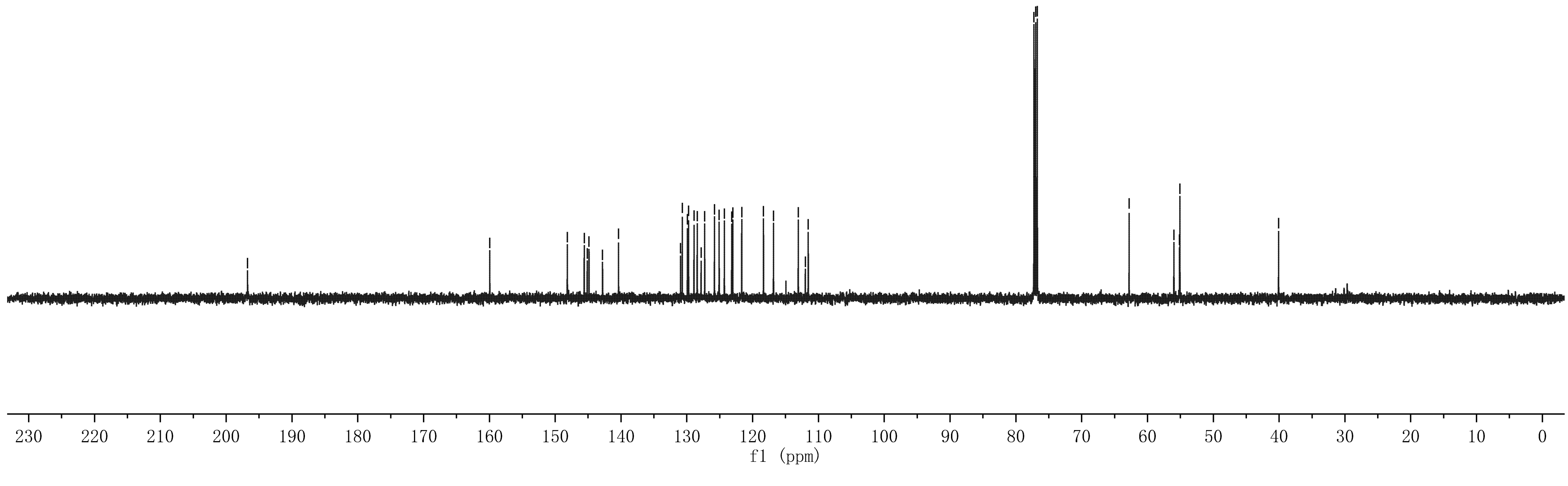

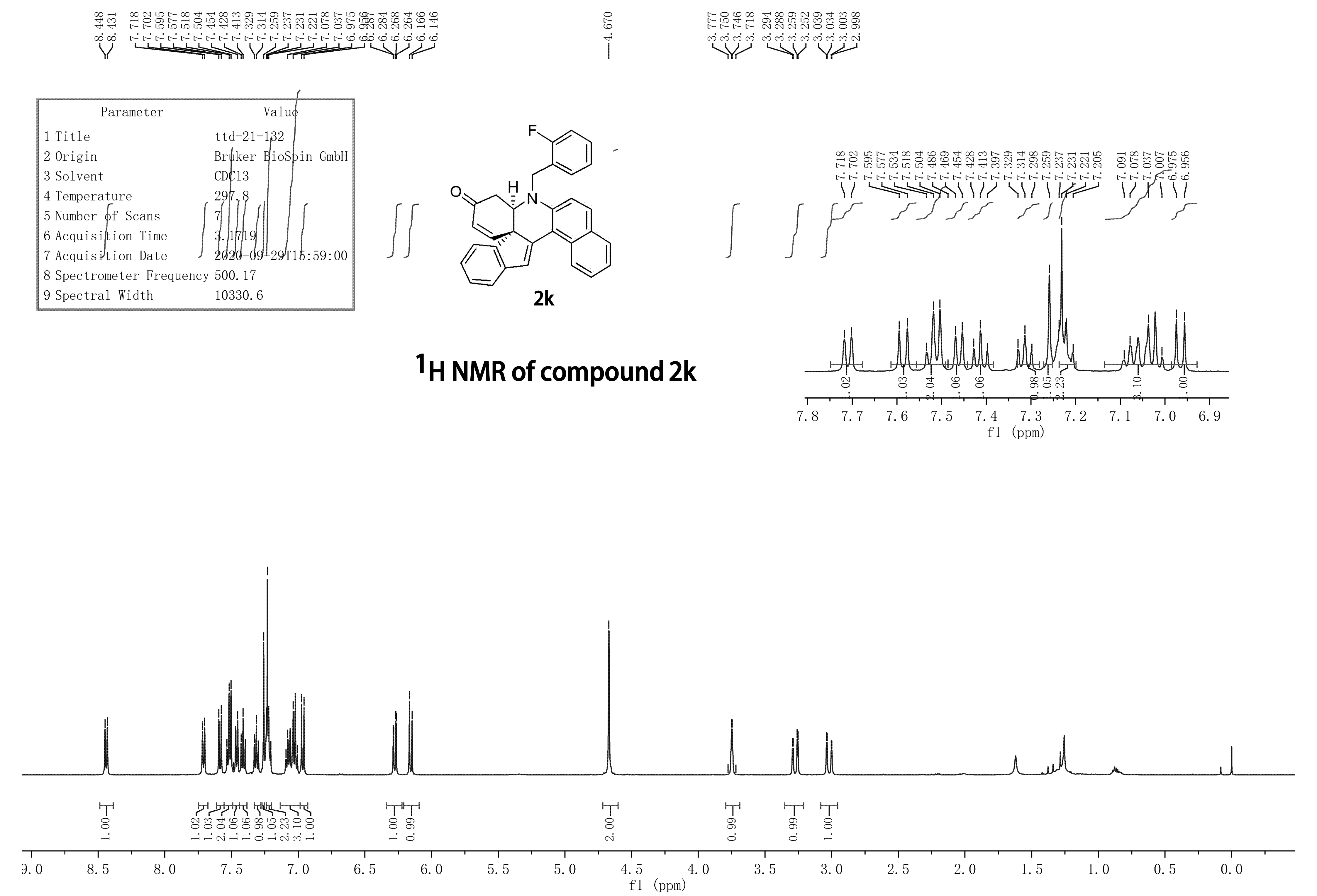

^{1}H NMR of compound 2k

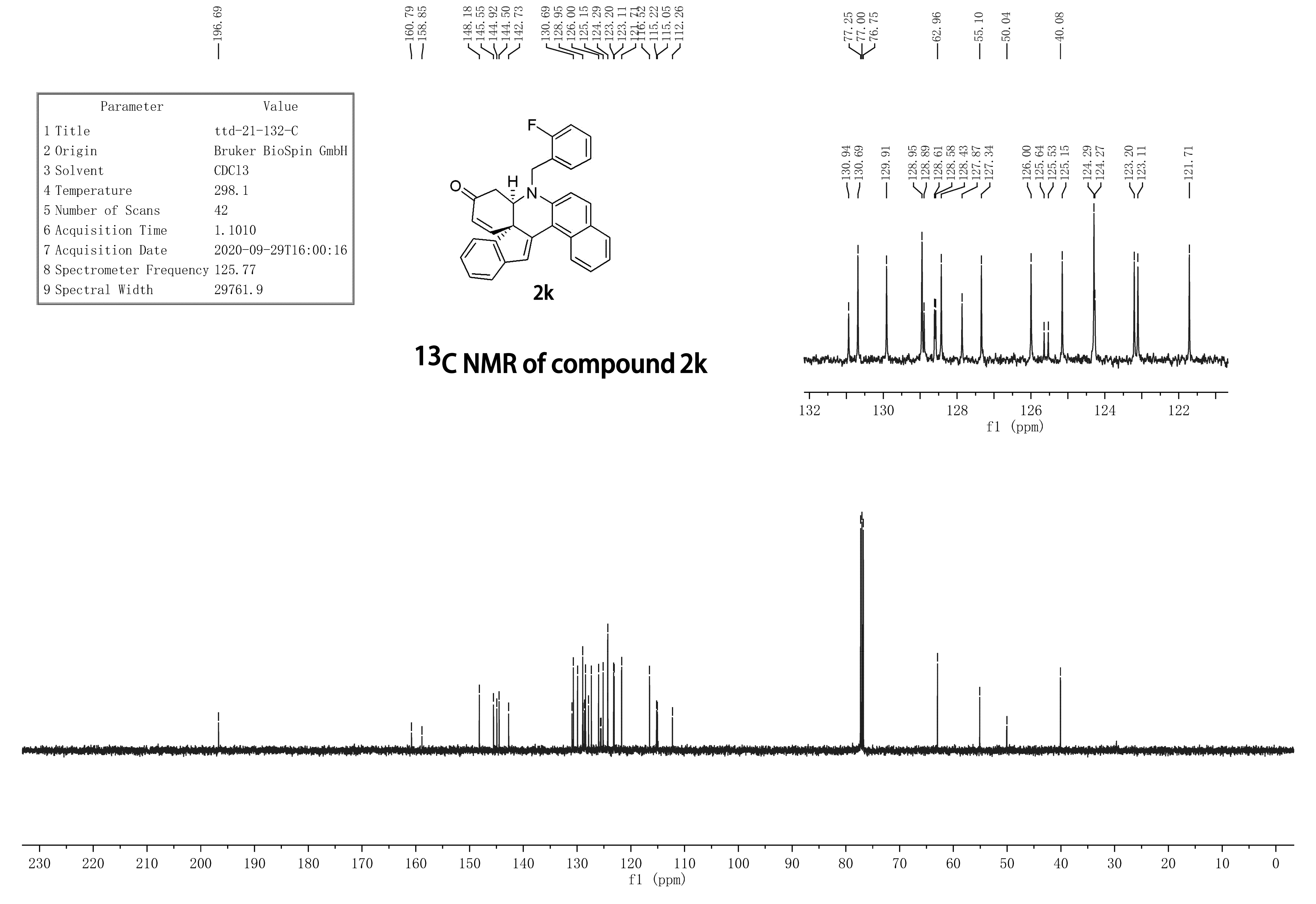

^{13}C NMR of compound 2k

Parameter	Value
1 Title	ttd-21-106
2 Origin	Bruker BioSpin GmbH
3 Solvent	CDCl3
4 Temperature	298.1
5 Number of Scans	14
6 Acquisition Time	3.1719
7 Acquisition Date	2020-09-23T15:52:34
8 Spectrometer Frequency	500.17
9 Spectral Width	10330.6

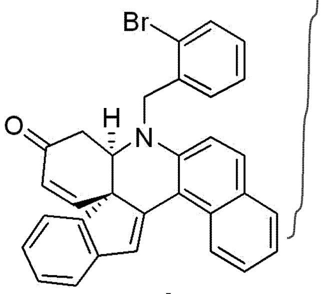

2l

^{1}H NMR of compound 2l

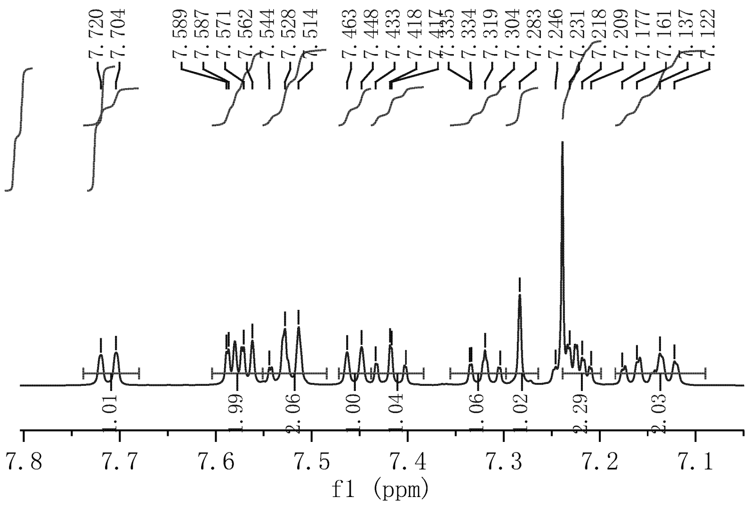

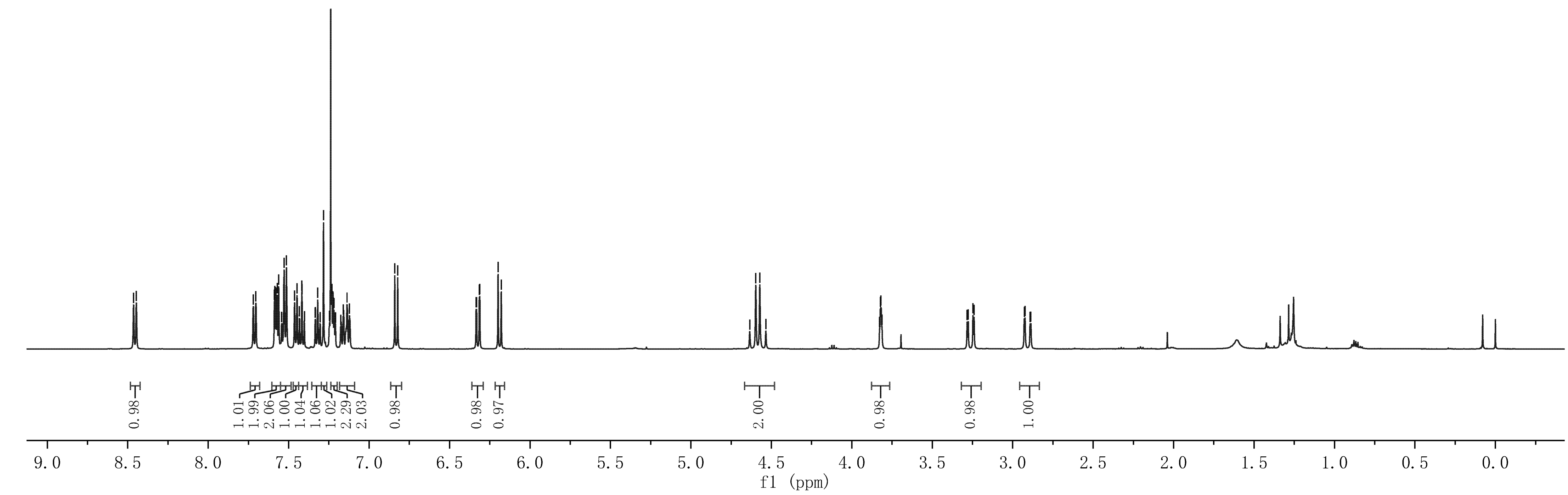

Parameter	Value
1 Title	ttd-21-106-C
2 Origin	Bruker BioSpin GmbH
3 Solvent	CDCl3
4 Temperature	298.7
5 Number of Scans	23
6 Acquisition Time	1.1010
7 Acquisition Date	2020-09-23T15:56:27
8 Spectrometer Frequency	125.77
9 Spectral Width	29761.9

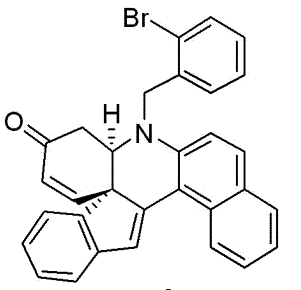

2l

^{13}C NMR of compound 2l

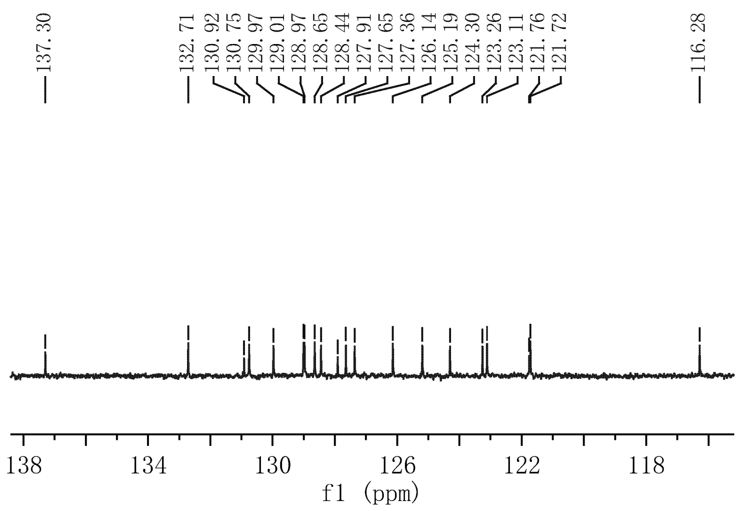

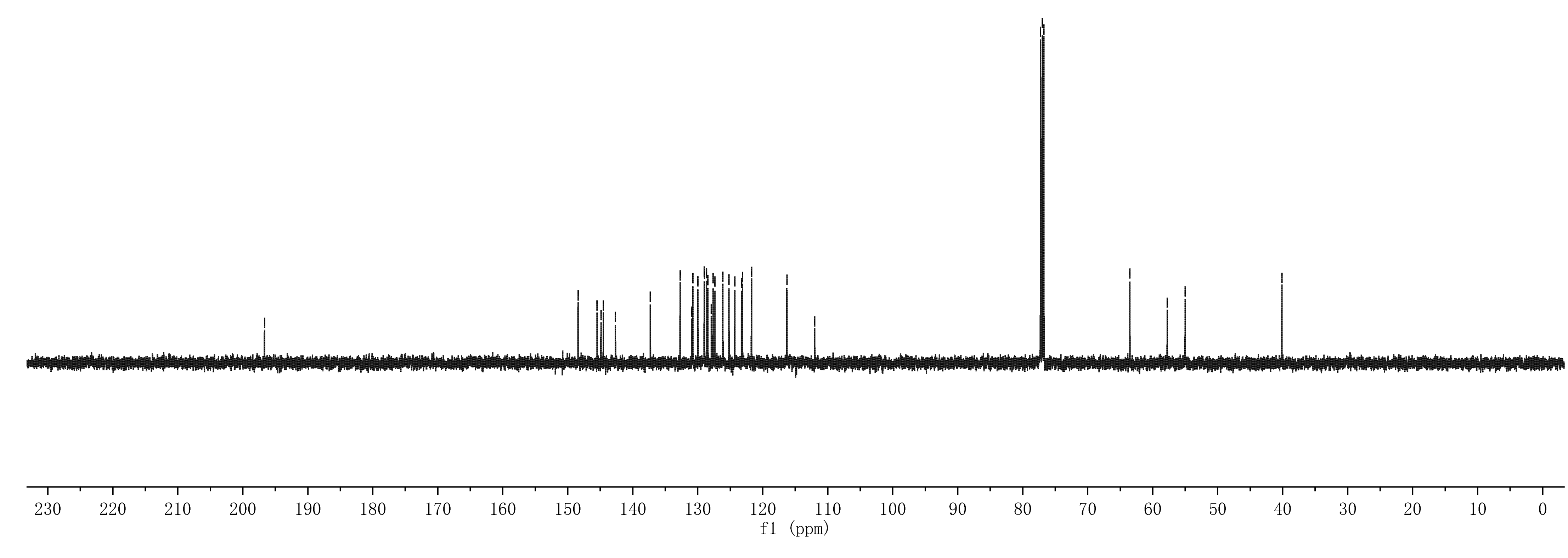

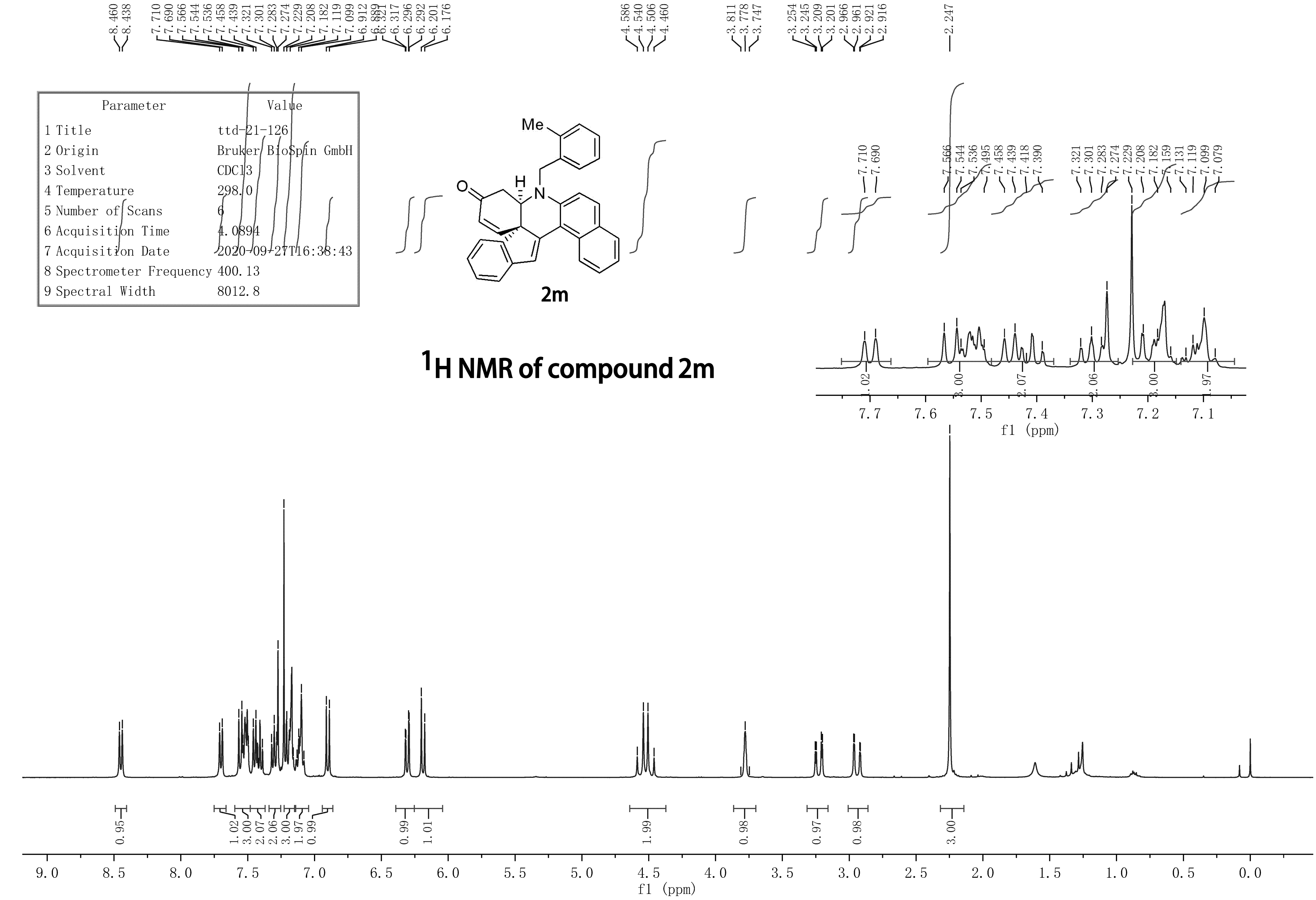
8.460
8.438
7.710
7.690
7.566
7.544
7.536
7.458
7.439
7.321
7.301
7.283
7.274
7.229
7.208
7.182
7.119
7.099
6.912
6.889
6.321
6.317
6.296
6.292
6.201
6.176
4.586
4.540
4.506
4.460
3.811
3.778
3.747
3.254
3.245
3.209
3.201
2.966
2.961
2.921
2.916
2.247
Parameter
Value
1 Title
ttd-21-126
2 Origin
Bruker BioSpin GmbH
3 Solvent
CDCl3
4 Temperature
298.0
5 Number of Scans
6
6 Acquisition Time
4.0894
7 Acquisition Date
2020-09-27T16:38:43
8 Spectrometer Frequency
400.13
9 Spectral Width
8012.8
Me
O
H
N
2m
1H NMR of compound 2m
7.710
7.690
7.566
7.544
7.536
7.495
7.458
7.439
7.418
7.390
7.321
7.301
7.283
7.274
7.229
7.208
7.182
7.159
7.131
7.119
7.099
7.079
1.02
3.00
2.07
2.06
3.00
1.97
7.7
7.6
7.5
7.4
7.3
7.2
7.1
f1 (ppm)
0.95
1.02
3.00
2.07
2.06
3.00
1.97
0.99
0.99
1.01
1.99
0.98
0.97
0.98
3.00
9.0
8.5
8.0
7.5
7.0
6.5
6.0
5.5
5.0
4.5
4.0
3.5
3.0
2.5
2.0
1.5
1.0
0.5
0.0
f1 (ppm)

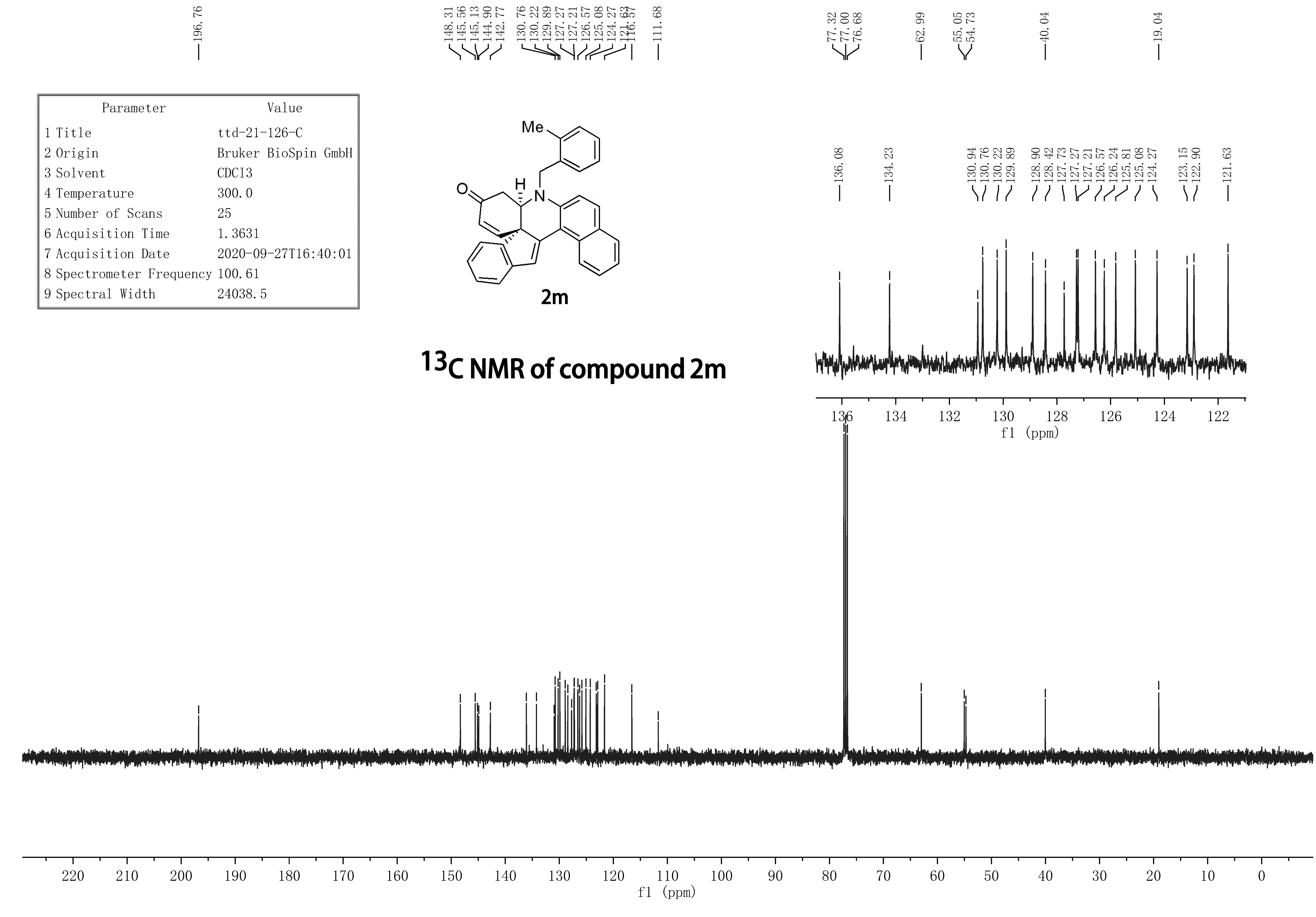
196.76
148.31
145.56
145.13
144.90
142.77
130.76
130.22
129.89
127.27
127.21
126.57
125.08
124.27
121.63
116.57
111.68
77.32
77.00
76.68
62.99
55.05
54.73
40.04
19.04
Parameter
Value
1 Title
ttd-21-126-C
2 Origin
Bruker BioSpin GmbH
3 Solvent
CDCl3
4 Temperature
300.0
5 Number of Scans
25
6 Acquisition Time
1.3631
7 Acquisition Date
2020-09-27T16:40:01
8 Spectrometer Frequency
100.61
9 Spectral Width
24038.5
Me
O
H
N
2m
13C NMR of compound 2m
136.08
134.23
130.94
130.76
130.22
129.89
128.90
128.42
127.73
127.27
127.21
126.57
126.24
125.81
125.08
124.27
123.15
122.90
121.63
136
134
132
130
128
126
124
122
f1 (ppm)
220
210
200
190
180
170
160
150
140
130
120
110
100
90
80
70
60
50
40
30
20
10
0
f1 (ppm)

Parameter	Value
1 Title	BHZ-2-65
2 Origin	Bruker BioSpin GmbH
3 Solvent	CDCl3
4 Temperature	297.9
5 Number of Scans	7
6 Acquisition Time	3.1719
7 Acquisition Date	2020-10-09T15:58:08
8 Spectrometer Frequency	500.17
9 Spectral Width	10330.6

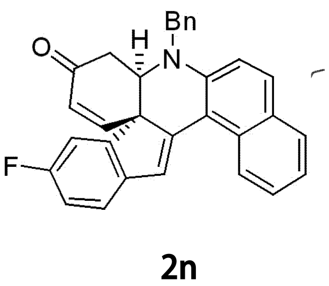

2n

^{1}H NMR of compound 2n

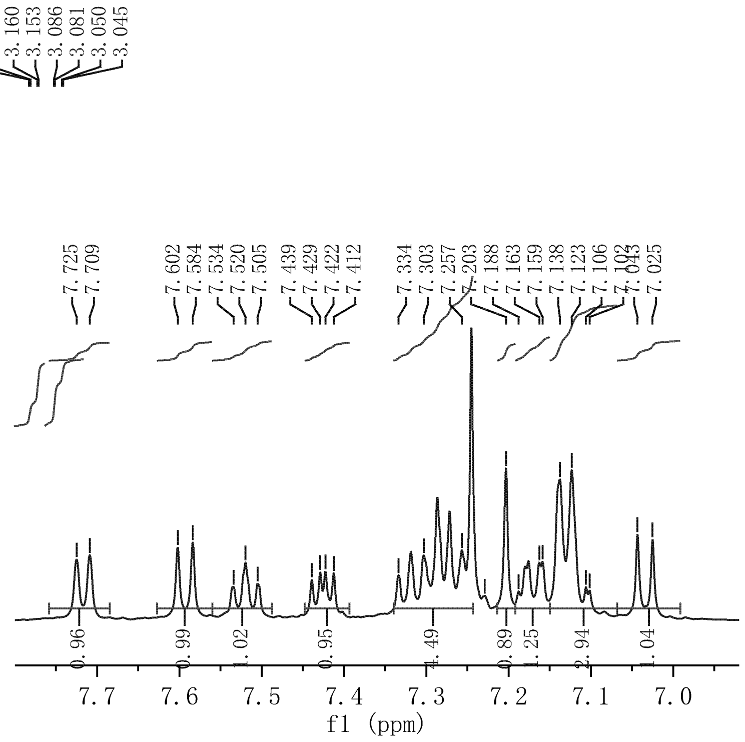

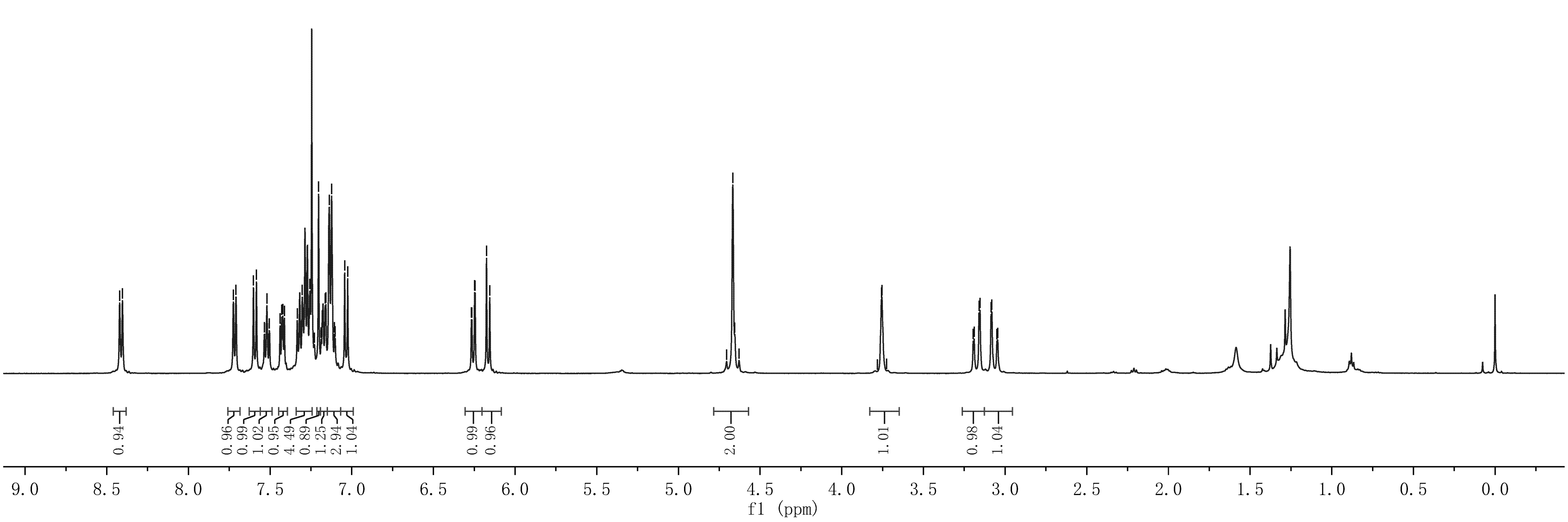

Parameter	Value
1 Title	BHZ-2-65-C
2 Origin	Bruker BioSpin GmbH
3 Solvent	CDCl3
4 Temperature	298.4
5 Number of Scans	58
6 Acquisition Time	1.1010
7 Acquisition Date	2020-10-09T15:59:25
8 Spectrometer Frequency	125.77
9 Spectral Width	29761.9

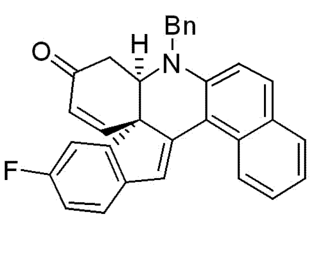

2n

^{13}C NMR of compound 2n

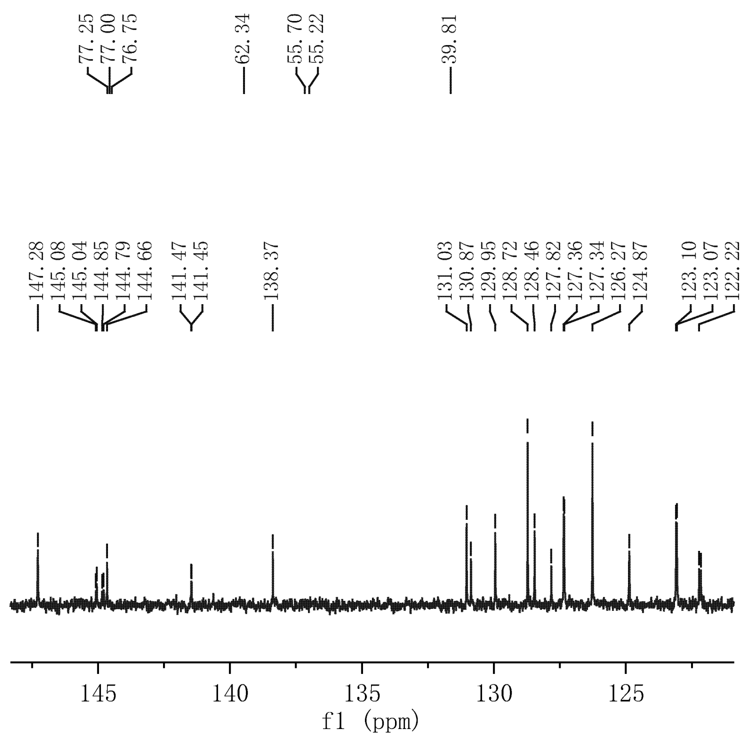

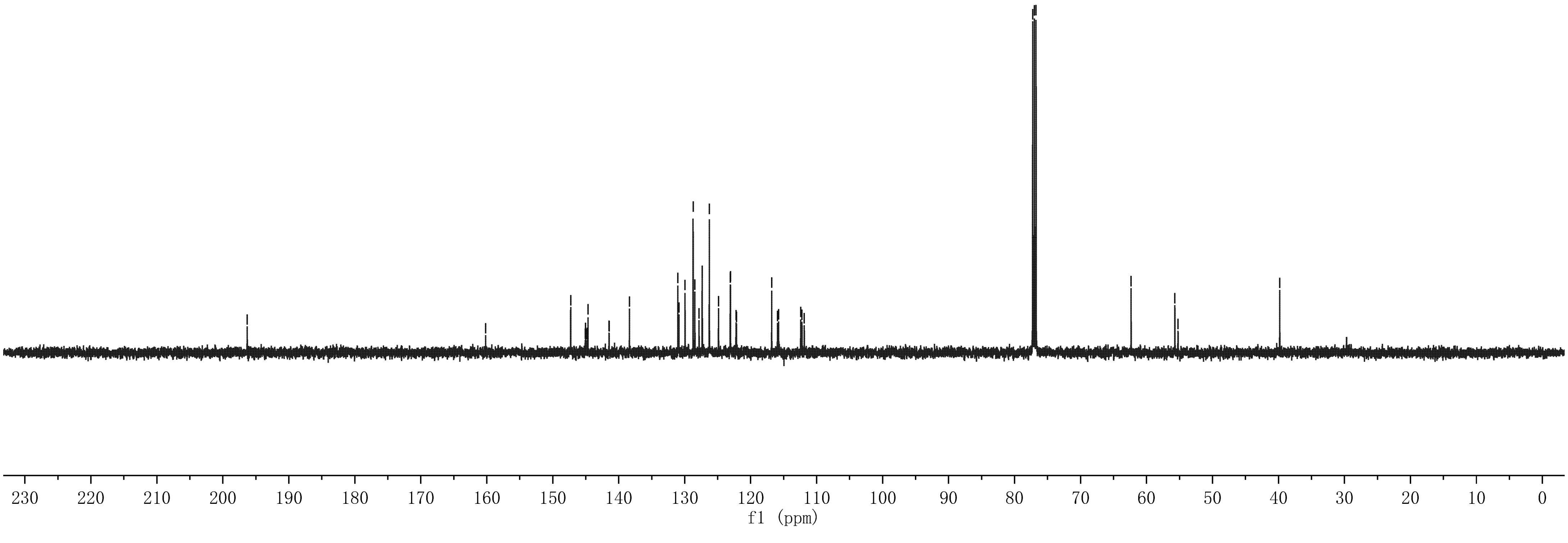

Parameter	Value
1 Title	BHZ-2-66-H
2 Origin	Bruker BioSpin GmbH
3 Solvent	CDCl3
4 Temperature	298.7
5 Number of Scans	9
6 Acquisition Time	3.1719
7 Acquisition Date	2020-10-09T17:09:58
8 Spectrometer Frequency	500.17
9 Spectral Width	10330.6

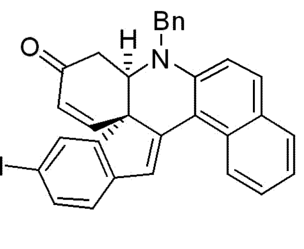

2o

^{1}H NMR of compound 2o

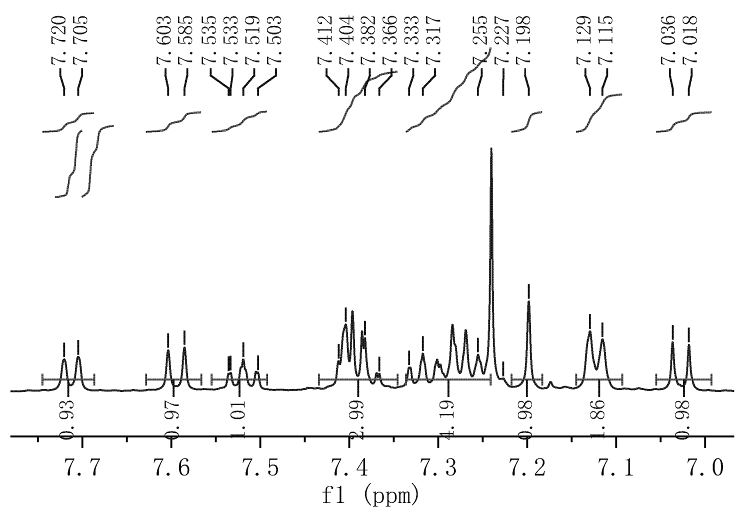

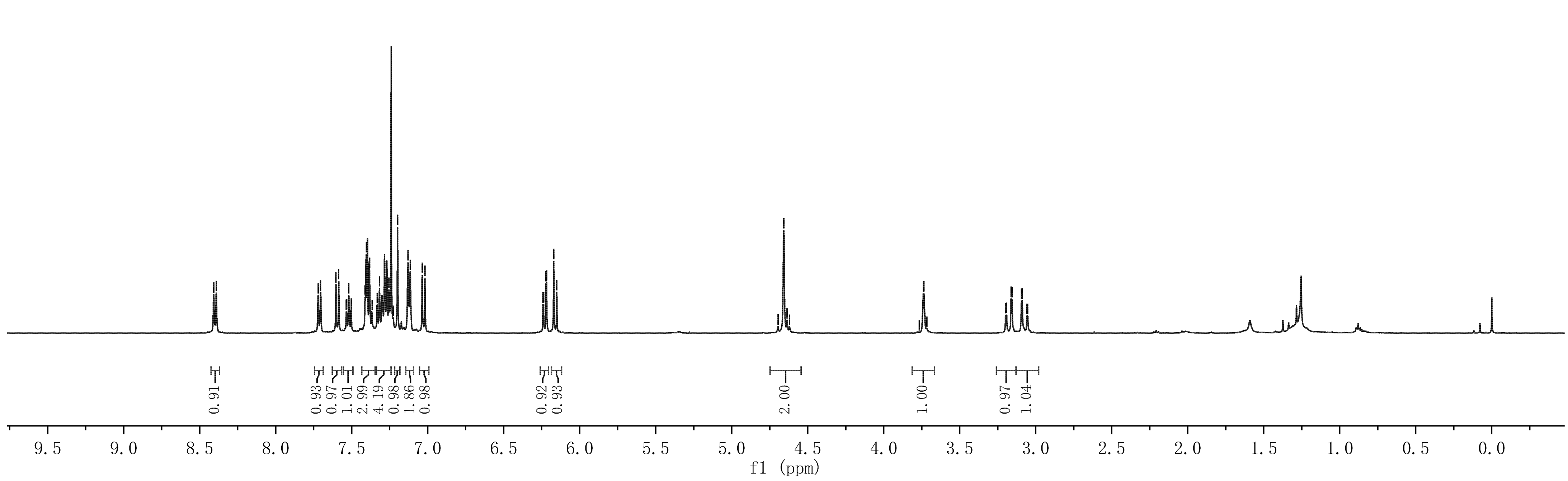

Parameter	Value
1 Title	BHZ-2-66-C
2 Origin	Bruker BioSpin GmbH
3 Solvent	CDCl3
4 Temperature	299.3
5 Number of Scans	31
6 Acquisition Time	1.1010
7 Acquisition Date	2020-10-09T17:14:40
8 Spectrometer Frequency	125.77
9 Spectral Width	29761.9

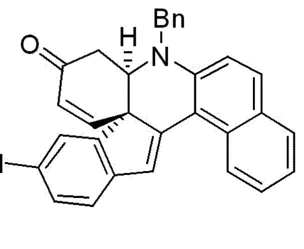

2o

^{13}C NMR of compound 2o

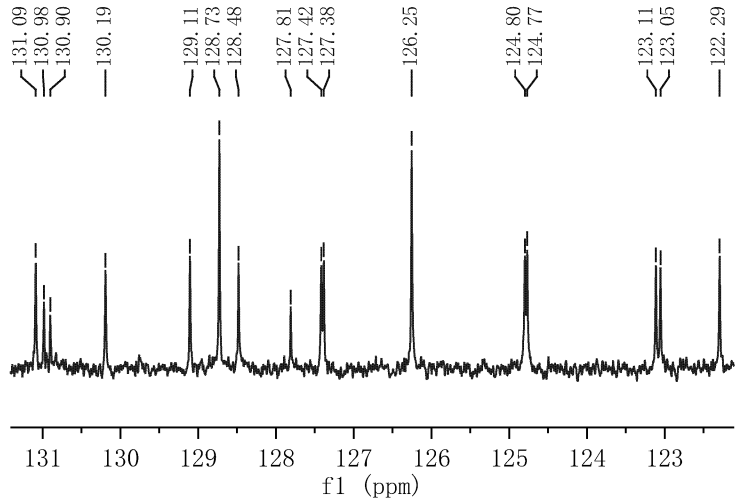

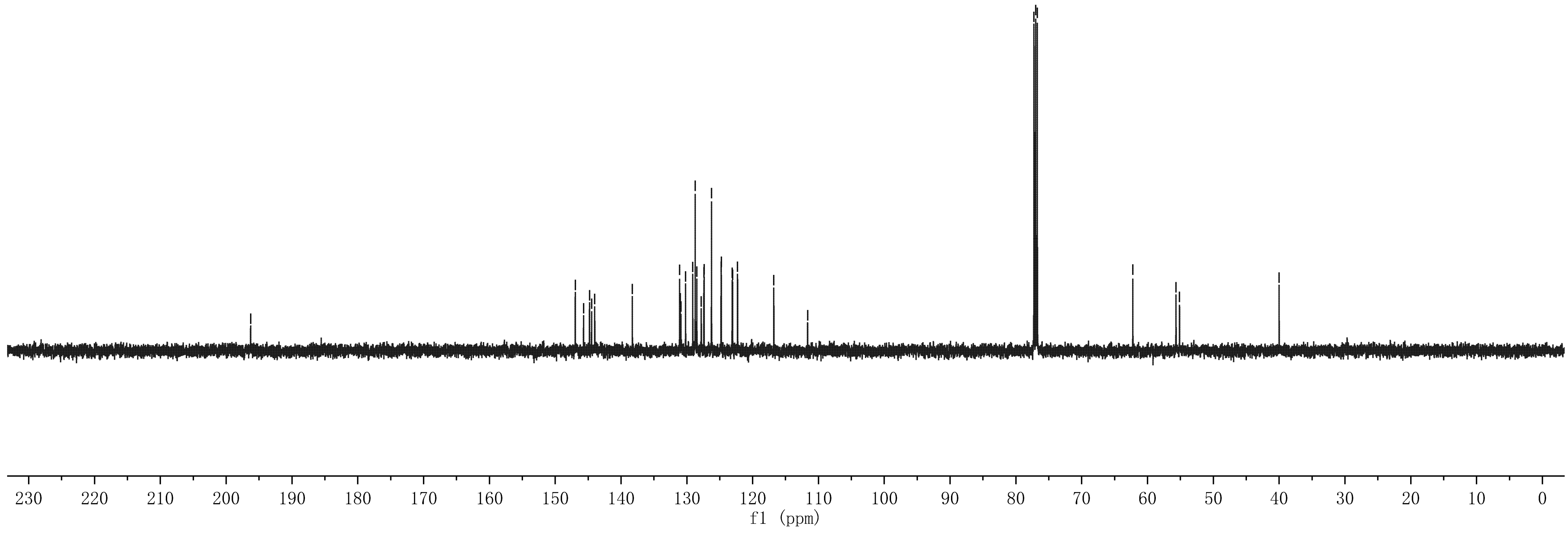

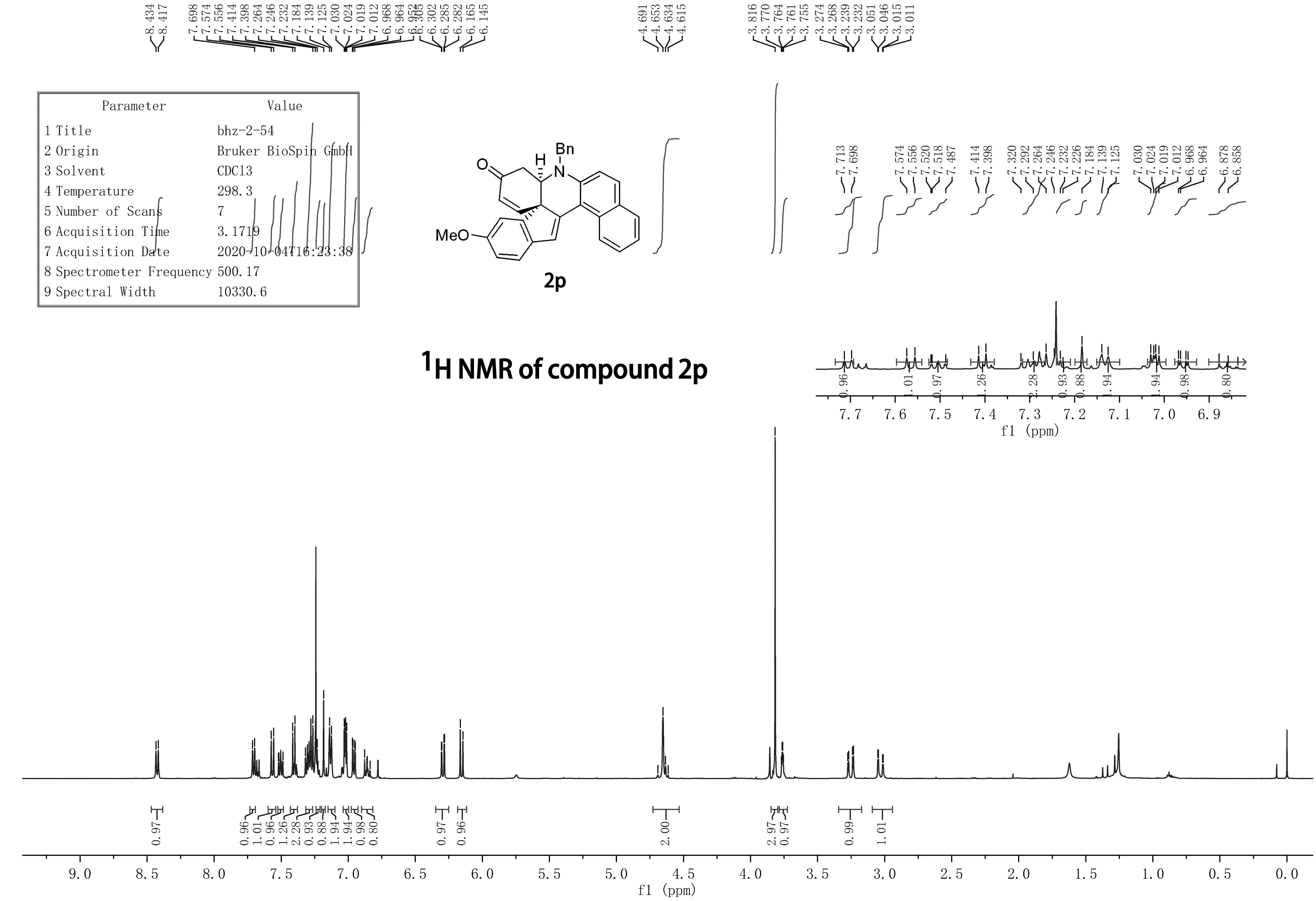

^{1}H NMR of compound 2p

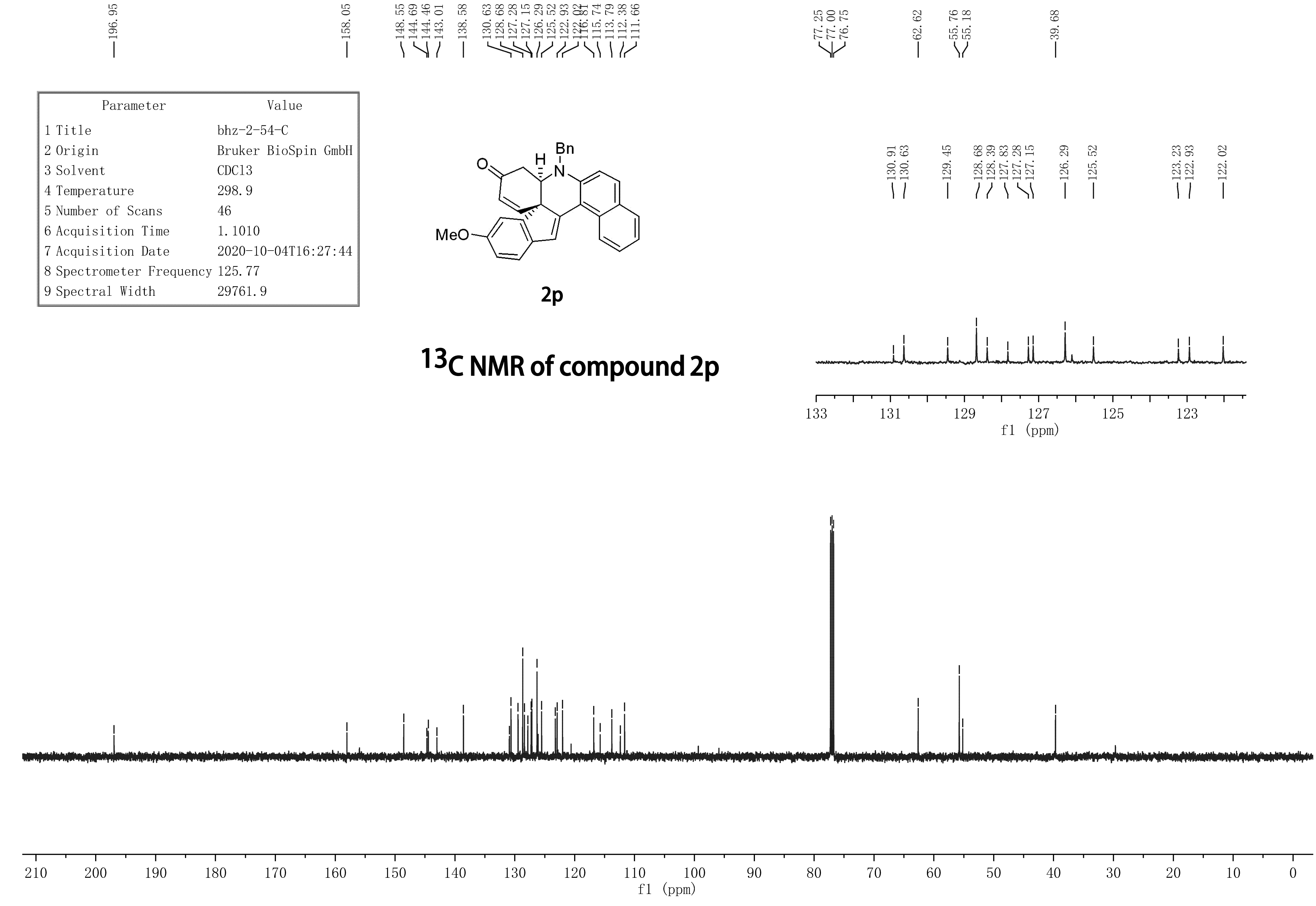

^{13}C NMR of compound 2p

Parameter	Value
1 Title	bhz-2-41-2
2 Origin	Bruker BioSpin GmbH
3 Solvent	CDCl3
4 Temperature	298.0
5 Number of Scans	8
6 Acquisition Time	4.0894
7 Acquisition Date	2021-05-18T07:59:37
8 Spectrometer Frequency	400.13
9 Spectral Width	8012.8

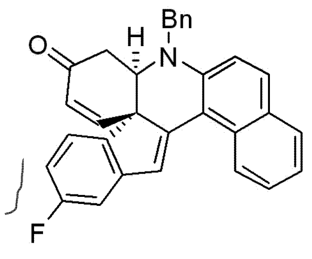

2q

1H NMR of compound 2q

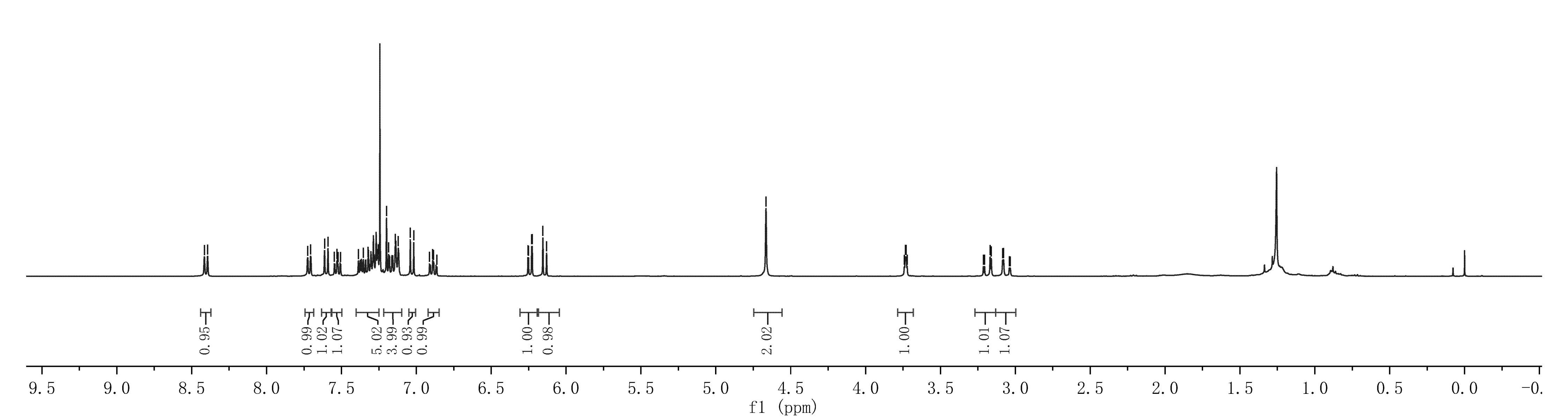

Parameter	Value
1 Title	bhz-2-41-C
2 Origin	Bruker BioSpin GmbH
3 Solvent	CDCl3
4 Temperature	300.0
5 Number of Scans	42
6 Acquisition Time	1.3631
7 Acquisition Date	2020-09-27T16:55:25
8 Spectrometer Frequency	100.61
9 Spectral Width	24038.5

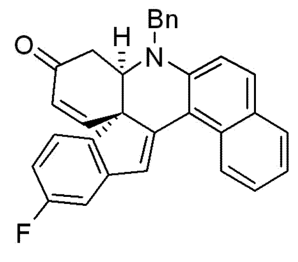

2q

13C NMR of compound 2q

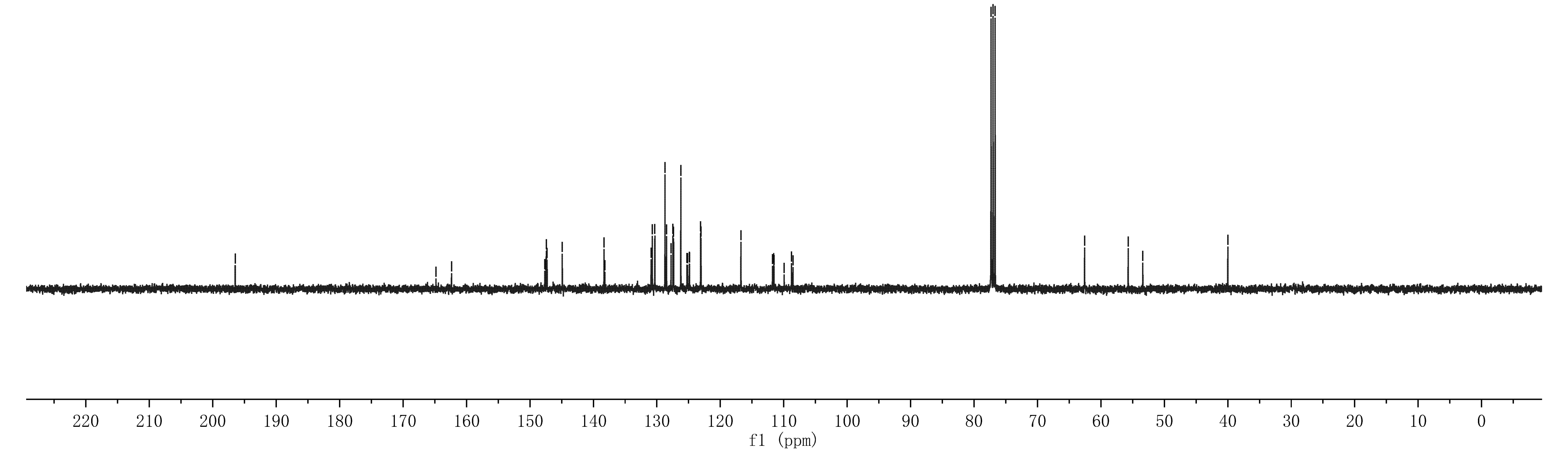

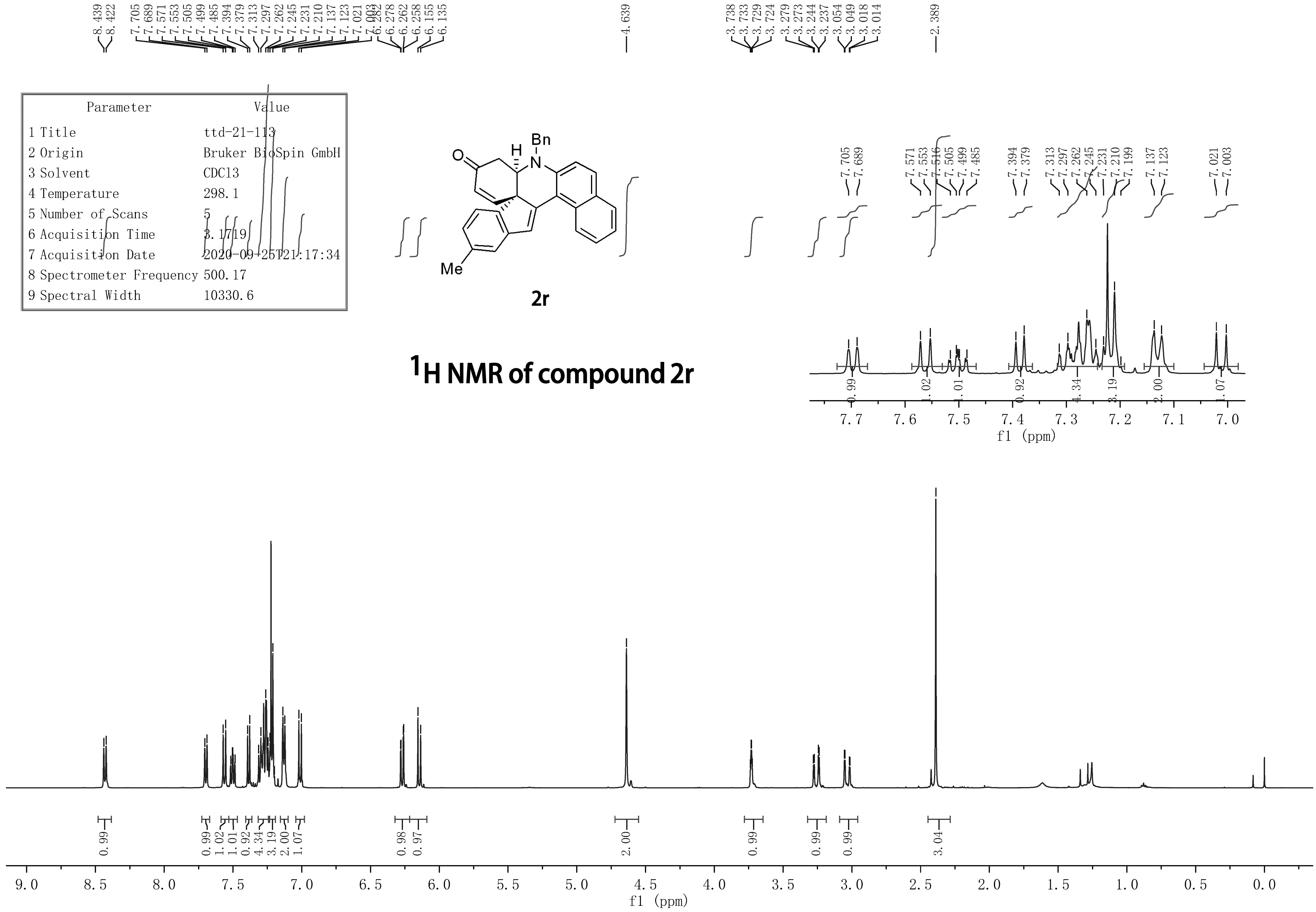

^{1}H NMR of compound 2r

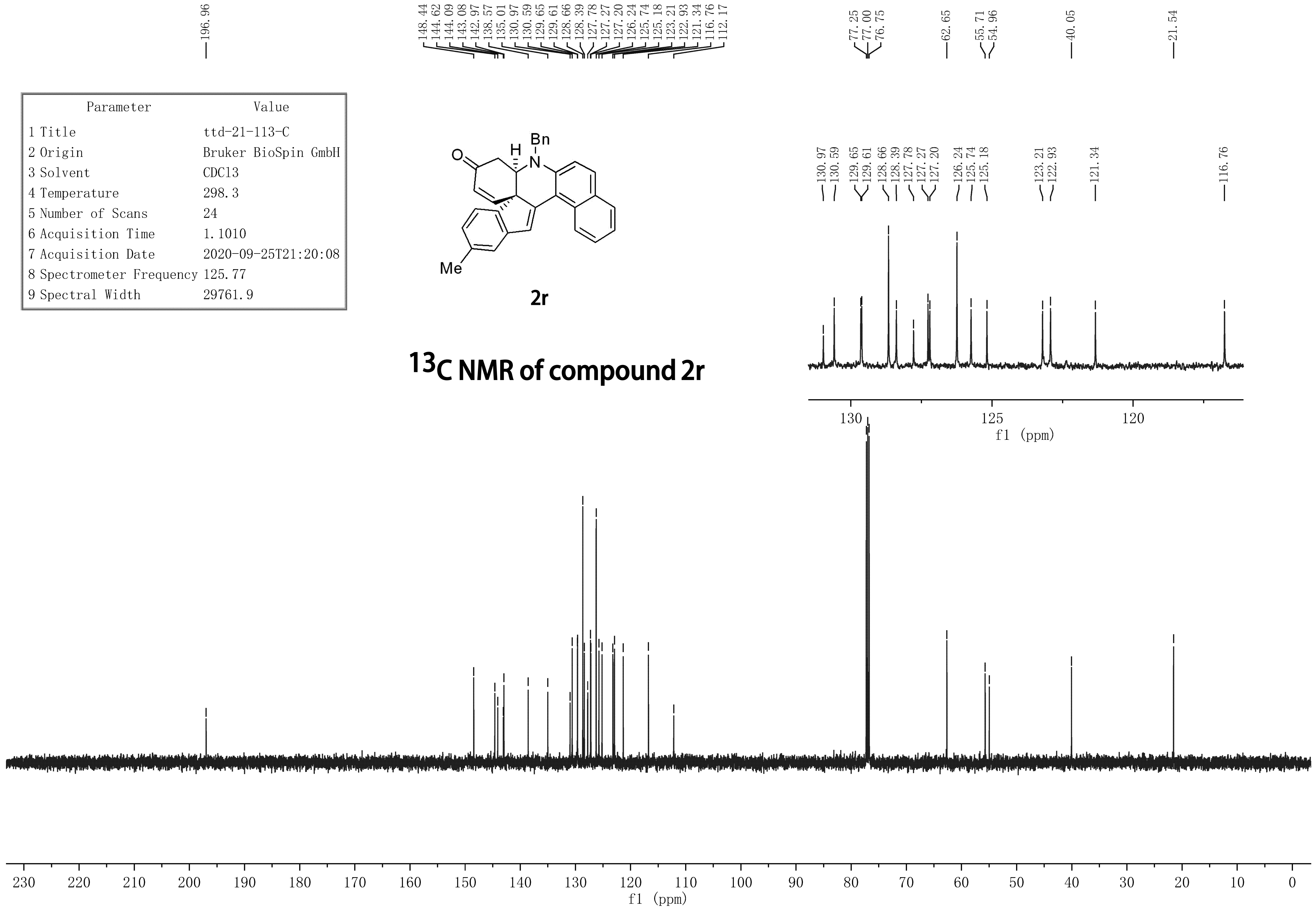

^{13}C NMR of compound 2r

Parameter	Value
1 Title	ttd-21-241
2 Origin	Bruker BioSpin GmbH
3 Solvent	CDCl3
4 Temperature	298.4
5 Number of Scans	9
6 Acquisition Time	3.1719
7 Acquisition Date	2020-11-13T19:32:51
8 Spectrometer Frequency	500.17
9 Spectral Width	10330.6

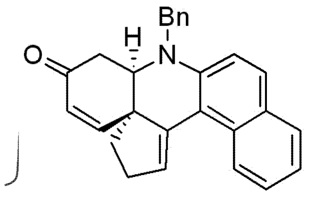

2s

1H NMR of compound 2s

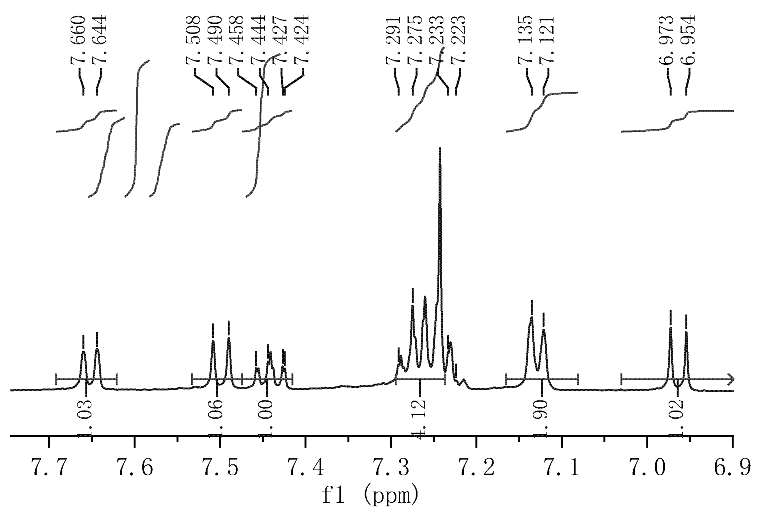

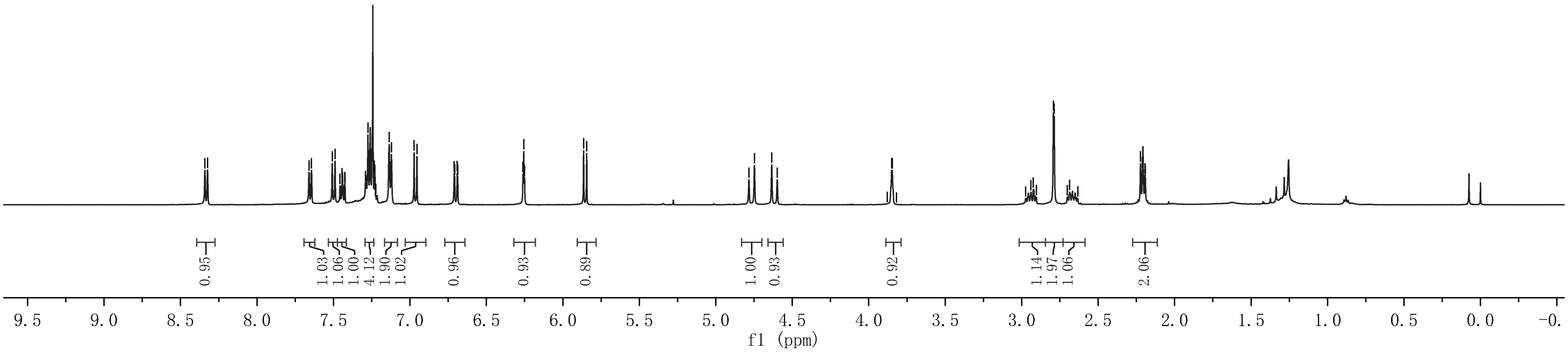

Parameter	Value
1 Title	ttd-21-241-C
2 Origin	Bruker BioSpin GmbH
3 Solvent	CDCl3
4 Temperature	299.1
5 Number of Scans	32
6 Acquisition Time	1.1010
7 Acquisition Date	2020-11-13T19:37:09
8 Spectrometer Frequency	125.77
9 Spectral Width	29761.9

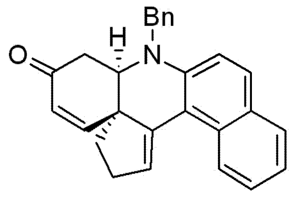

2s

13C NMR of compound 2s

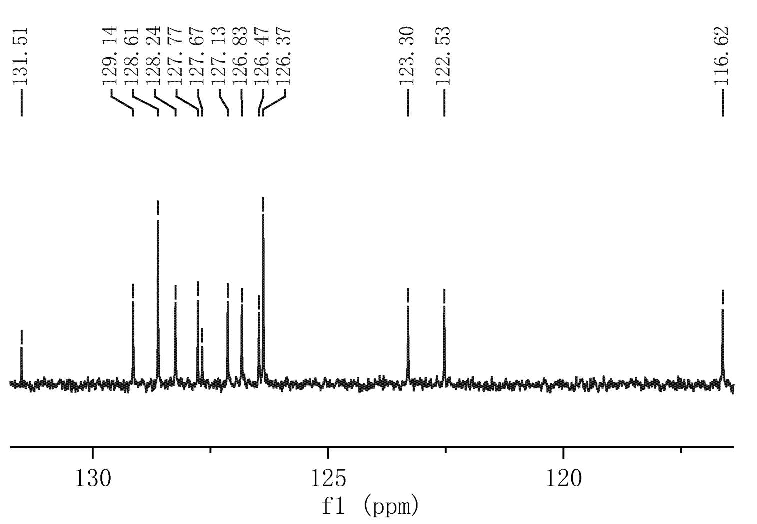

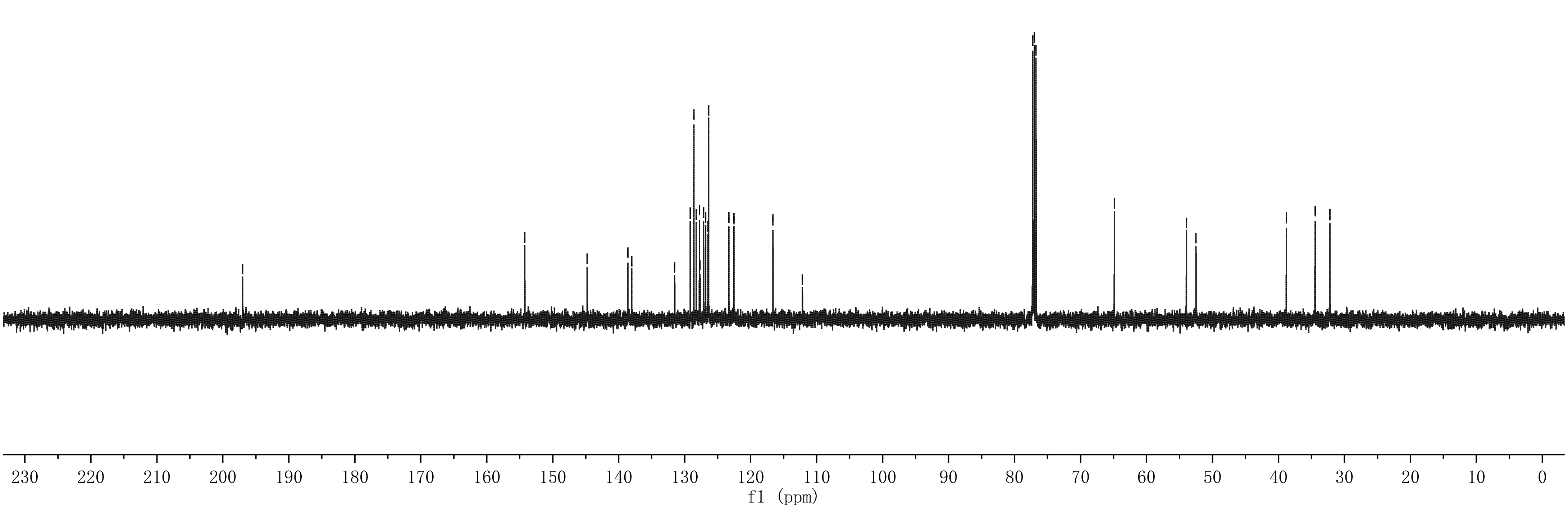

Parameter	Value
1 Title	ttd-21-241-C135
2 Origin	Bruker BioSpin GmbH
3 Solvent	CDCl3
4 Temperature	299.0
5 Number of Scans	15
6 Acquisition Time	1.1010
7 Acquisition Date	2020-11-13T19:39:26
8 Spectrometer Frequency	125.77
9 Spectral Width	29761.9

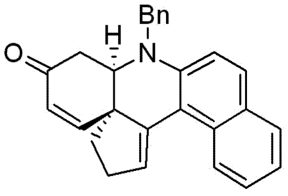

2s

^{13}C dept135 NMR of compound 2s

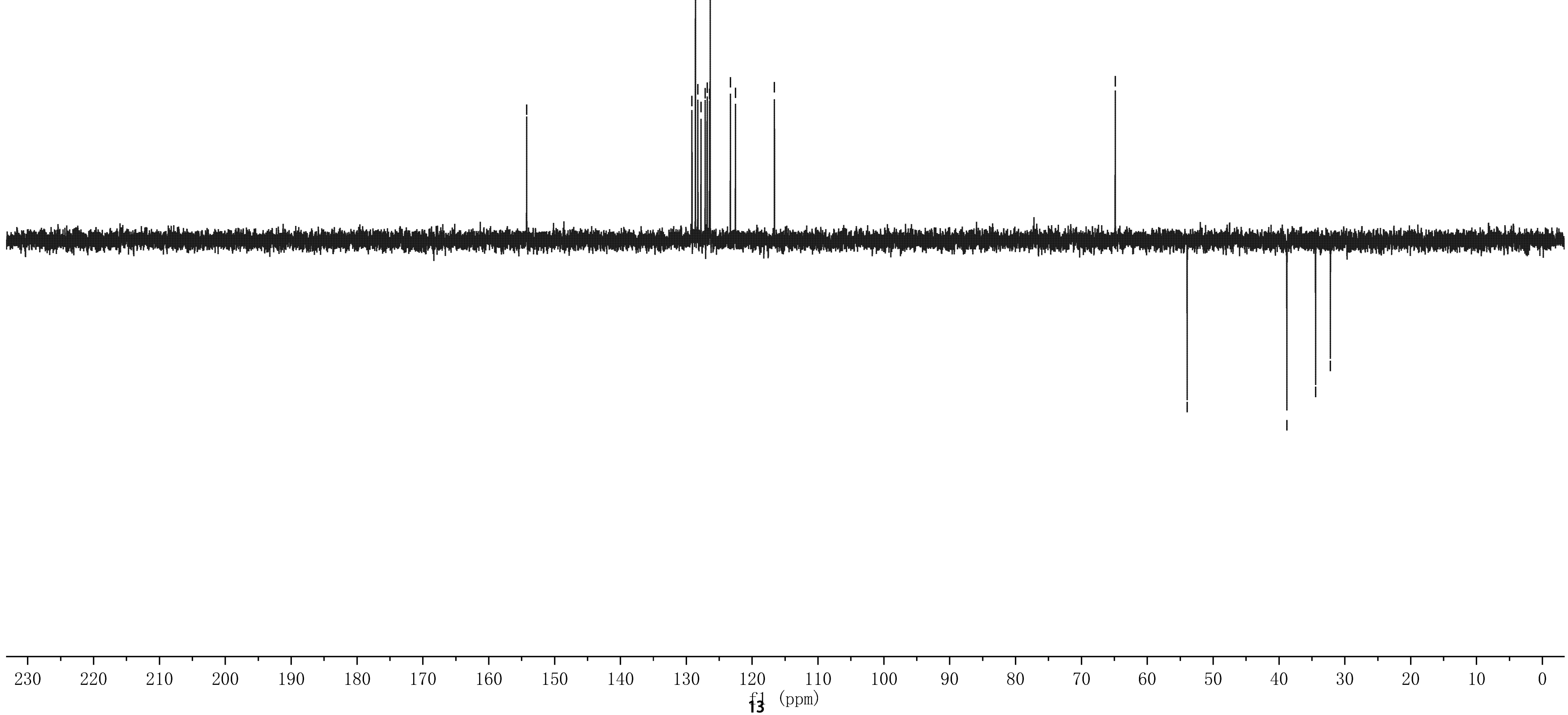

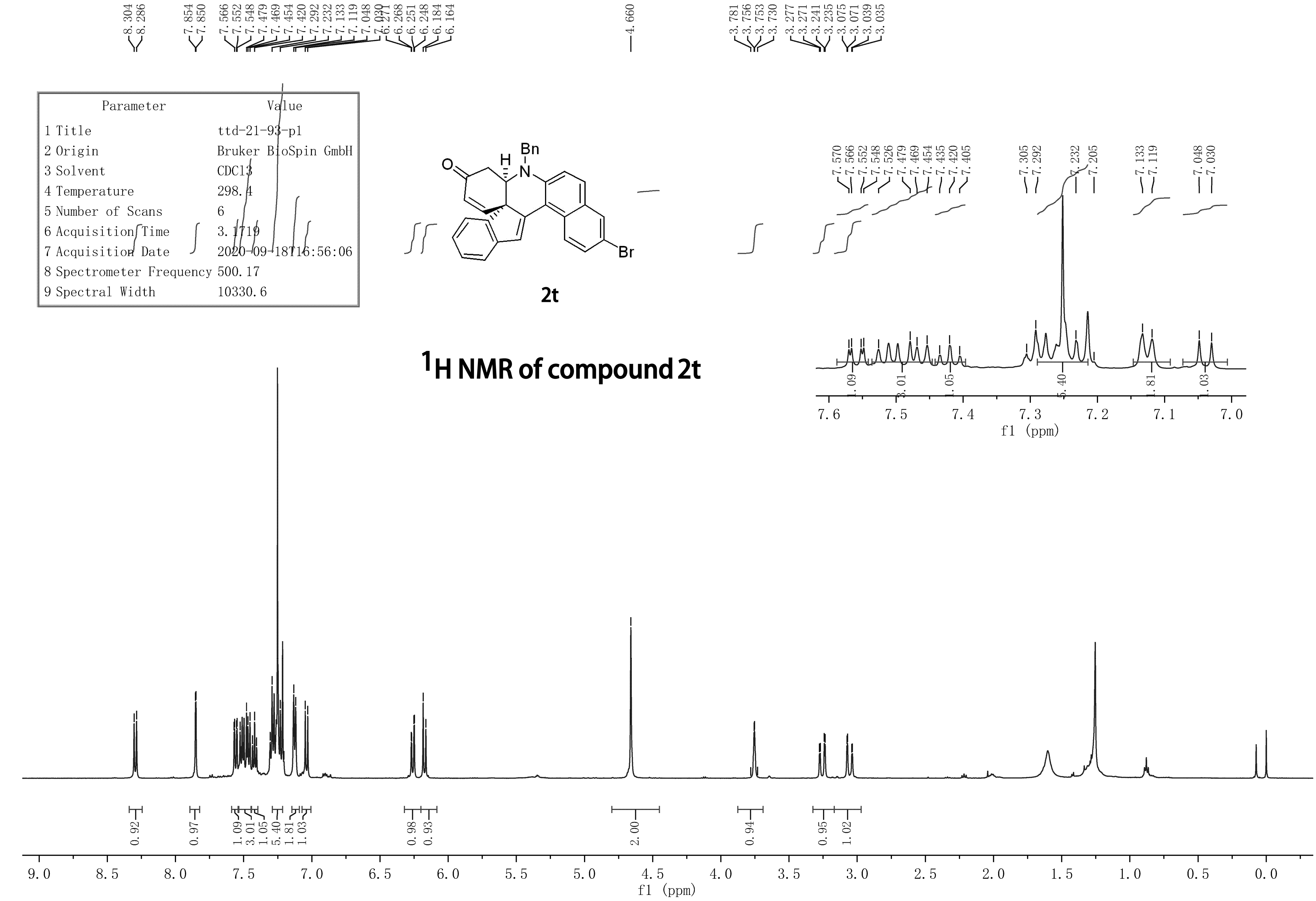
Parameter Value
1 Title ttd-21-93-p1
2 Origin Bruker BioSpin GmbH
3 Solvent CDCl3
4 Temperature 298.4
5 Number of Scans 6
6 Acquisition Time 3.1719
7 Acquisition Date 2020-09-18T16:56:06
8 Spectrometer Frequency 500.17
9 Spectral Width 10330.6
2t
1H NMR of compound 2t
f1 (ppm)

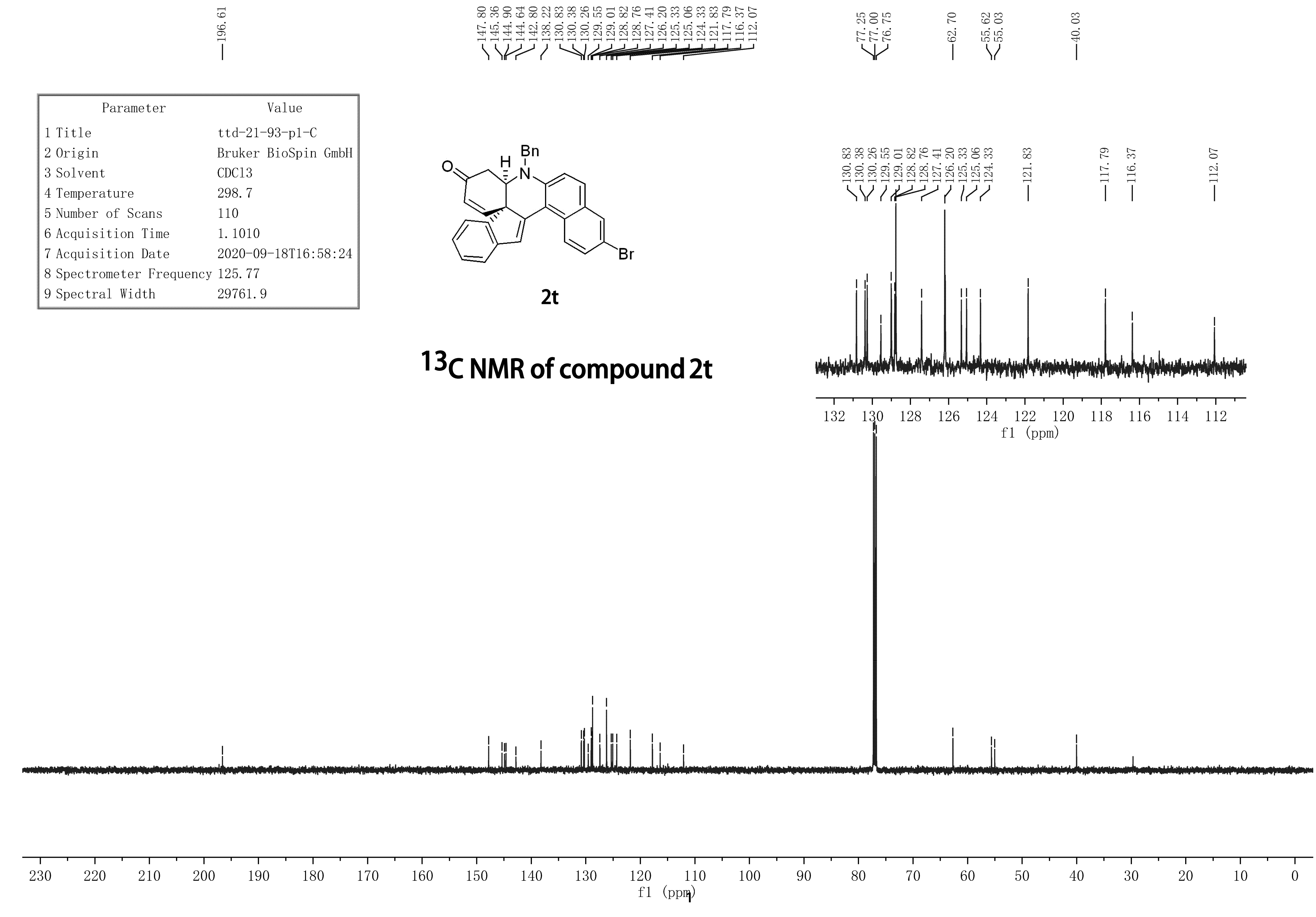
Parameter Value
1 Title ttd-21-93-p1-C
2 Origin Bruker BioSpin GmbH
3 Solvent CDCl3
4 Temperature 298.7
5 Number of Scans 110
6 Acquisition Time 1.1010
7 Acquisition Date 2020-09-18T16:58:24
8 Spectrometer Frequency 125.77
9 Spectral Width 29761.9
2t
13C NMR of compound 2t
f1 (ppm)

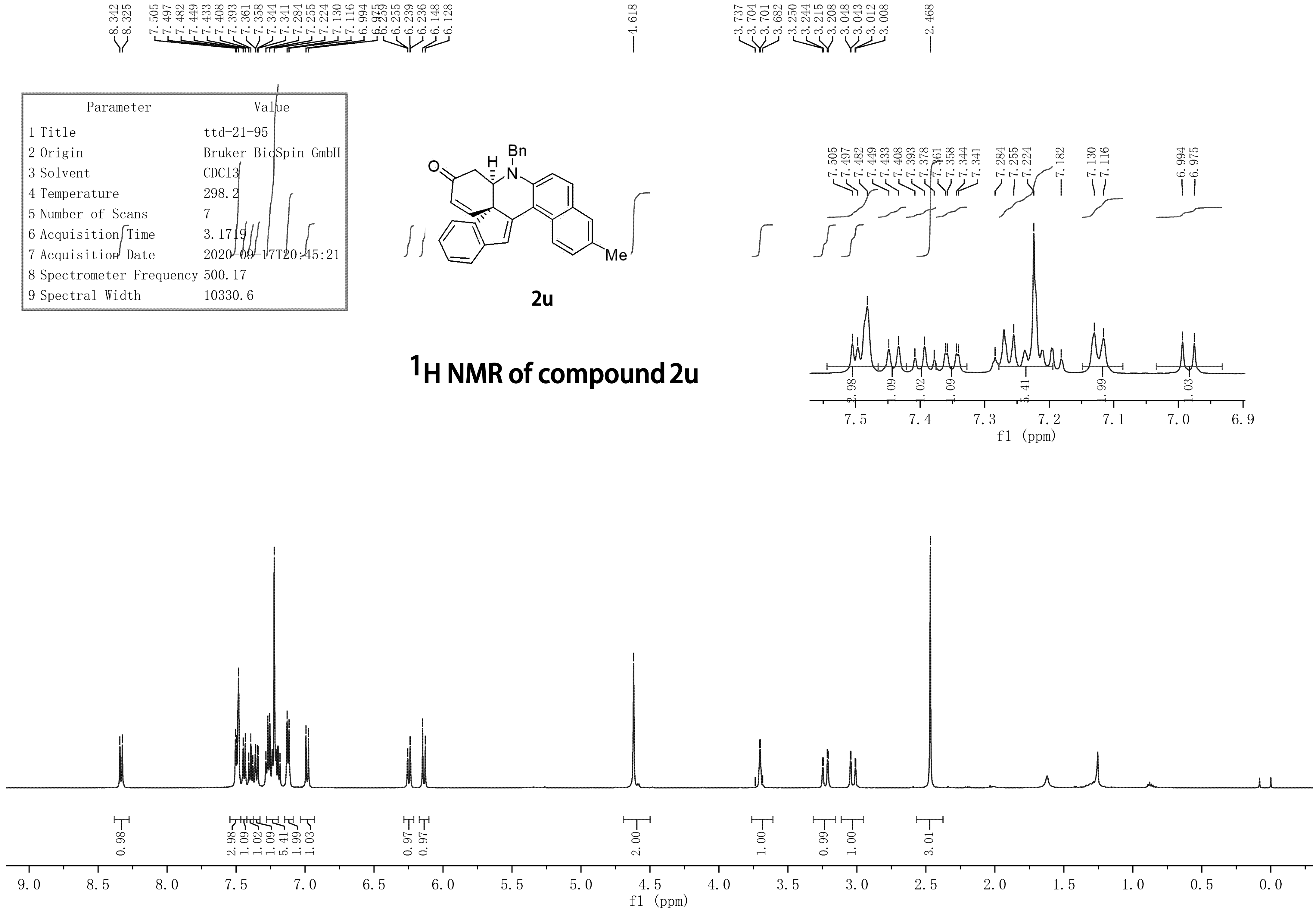

Parameter	Value
1 Title	ttd-21-95
2 Origin	Bruker BioSpin GmbH
3 Solvent	CDCl3
4 Temperature	298.2
5 Number of Scans	7
6 Acquisition Time	3.1719
7 Acquisition Date	2020-09-17T20:45:21
8 Spectrometer Frequency	500.17
9 Spectral Width	10330.6

2u

^{1}H NMR of compound 2u

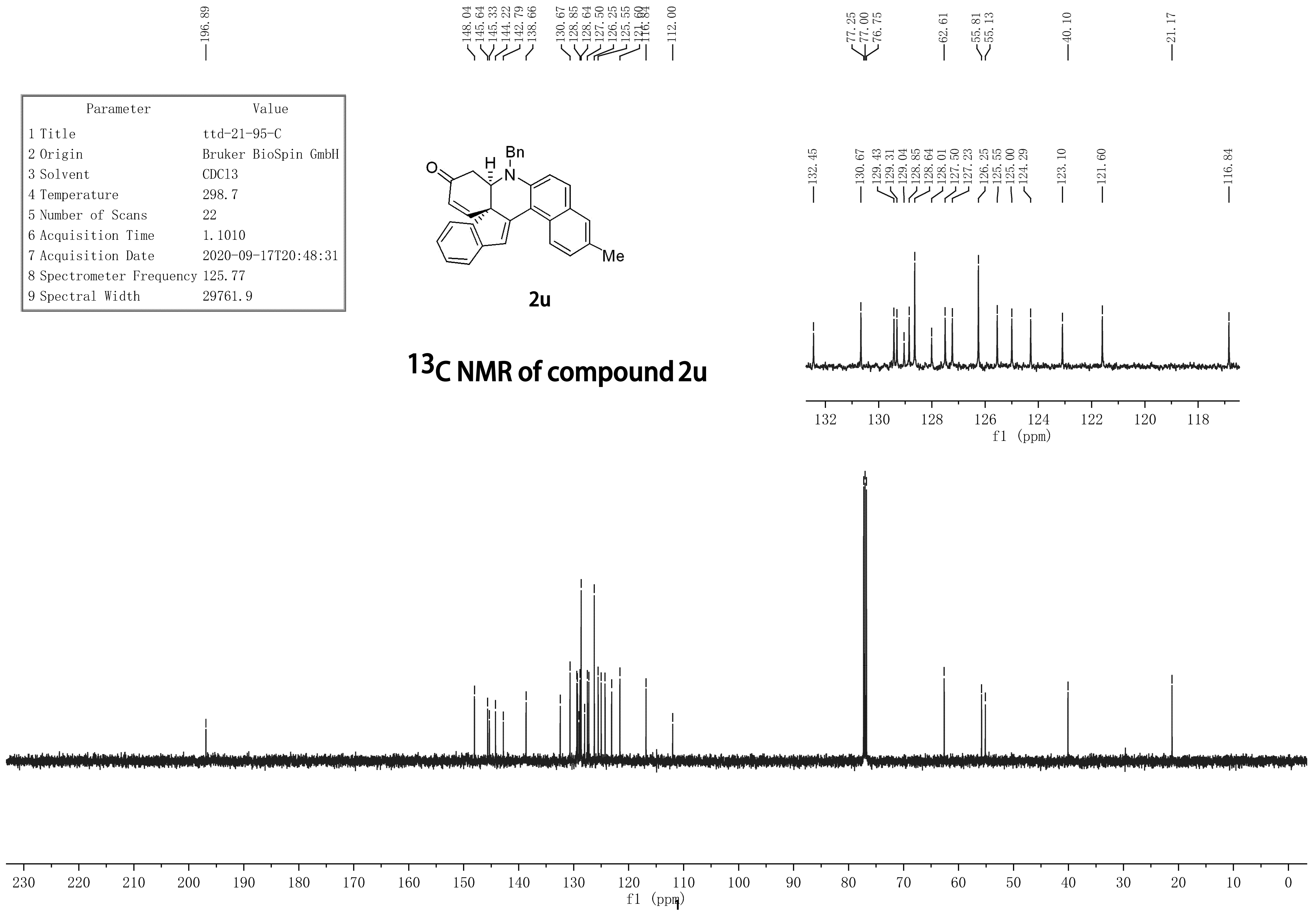

Parameter	Value
1 Title	ttd-21-95-C
2 Origin	Bruker BioSpin GmbH
3 Solvent	CDCl3
4 Temperature	298.7
5 Number of Scans	22
6 Acquisition Time	1.1010
7 Acquisition Date	2020-09-17T20:48:31
8 Spectrometer Frequency	125.77
9 Spectral Width	29761.9

2u

^{13}C NMR of compound 2u

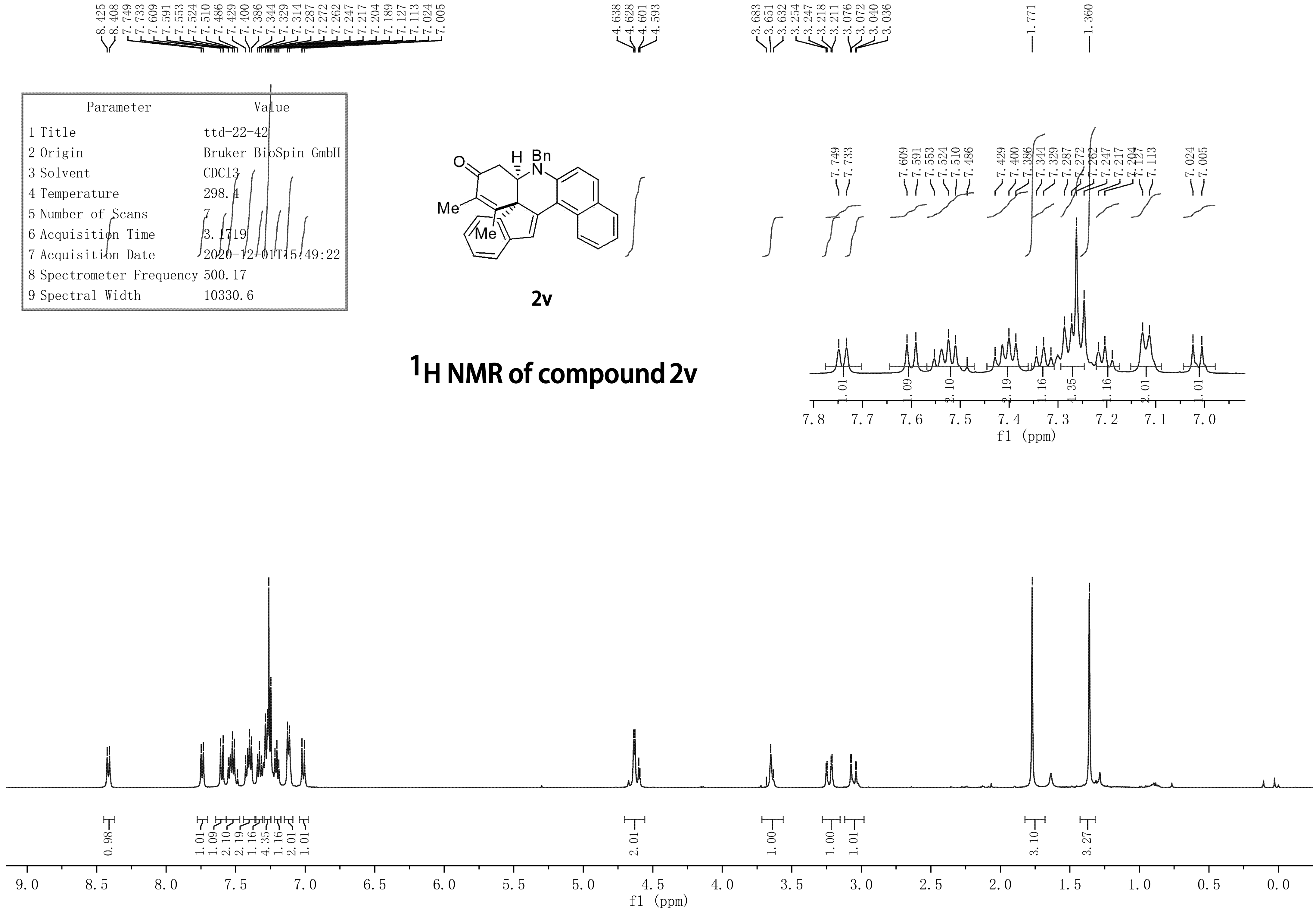

^{1}H NMR of compound 2v

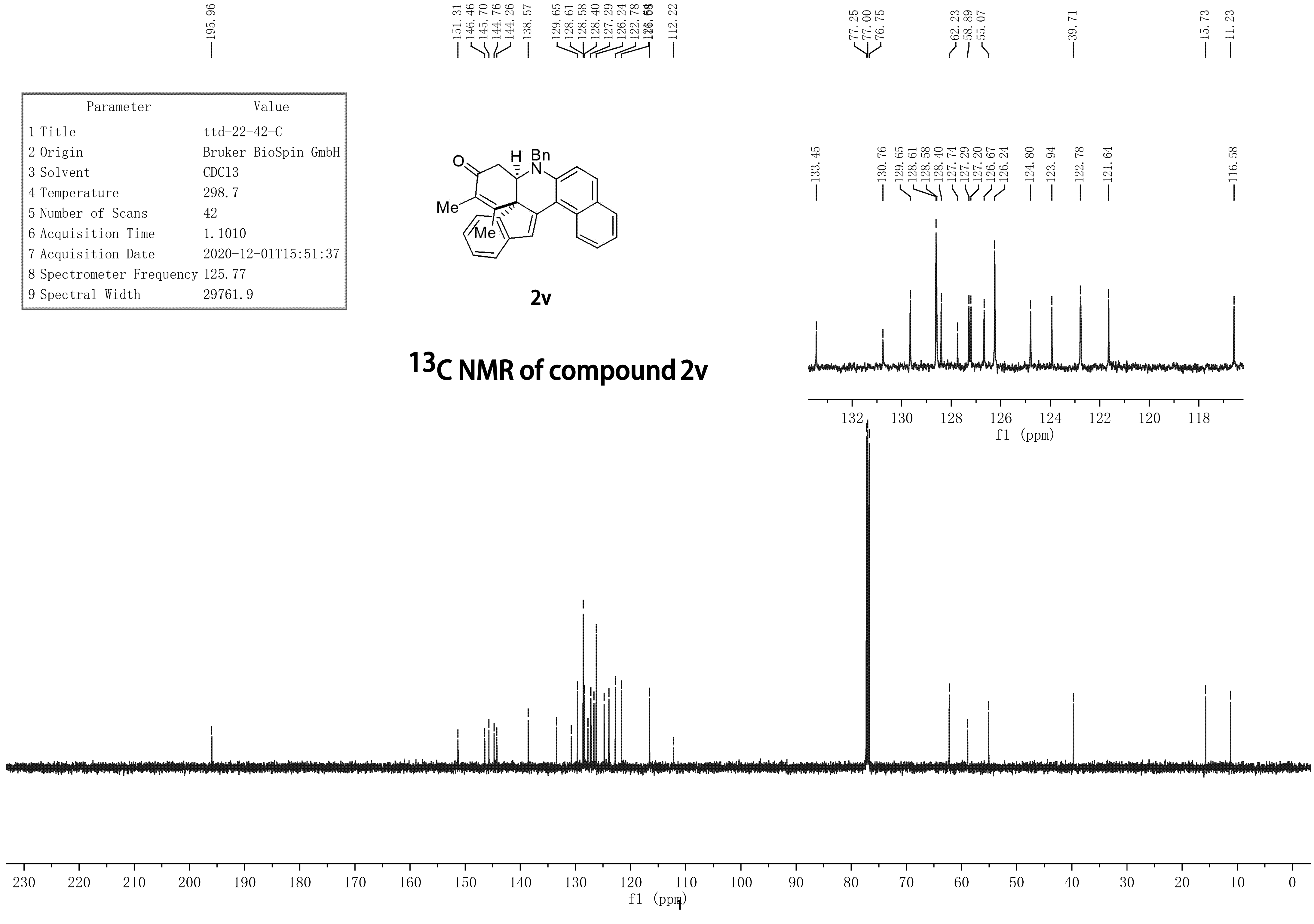

^{13}C NMR of compound 2v

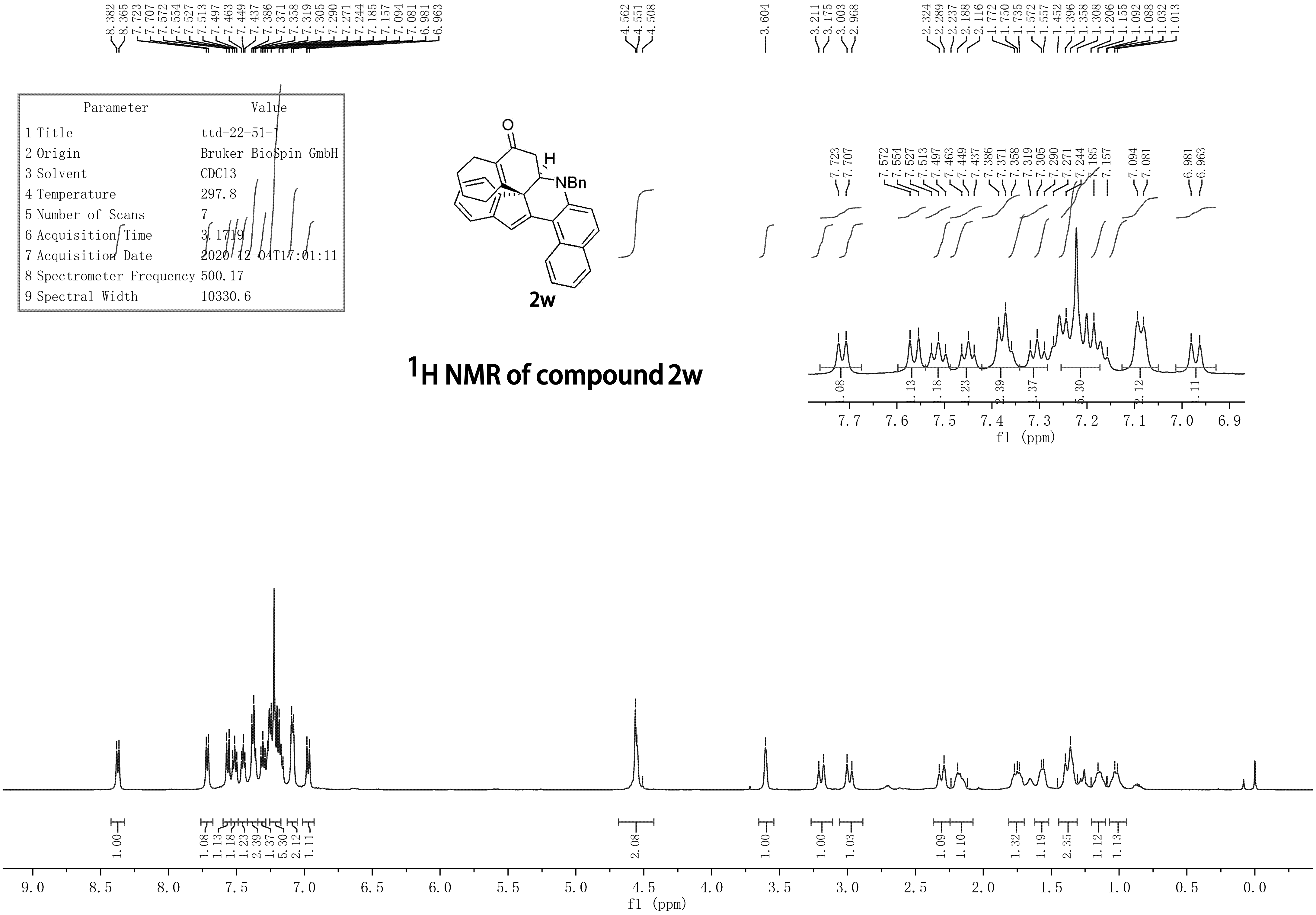
8.382 8.365 7.723 7.707 7.572 7.554 7.527 7.513 7.497 7.463 7.449 7.437 7.386 7.371 7.358 7.319 7.305 7.290 7.271 7.244 7.185 7.157 7.094 7.081 6.981 6.963
4.562 4.551 4.508
3.604
3.211 3.175 3.003 2.968
2.324 2.289 2.237 2.188 2.116 1.772 1.750 1.735 1.572 1.557 1.452 1.396 1.358 1.308 1.206 1.155 1.092 1.088 1.032 1.013
Parameter | Value
1 Title | ttd-22-51-1
2 Origin | Bruker BioSpin GmbH
3 Solvent | CDCl3
4 Temperature | 297.8
5 Number of Scans | 7
6 Acquisition Time | 3.1719
7 Acquisition Date | 2020-12-04T17:01:11
8 Spectrometer Frequency | 500.17
9 Spectral Width | 10330.6
O
H
NBn
2w
1H NMR of compound 2w
1.08 1.13 1.18 1.23 2.39 1.37 5.30 2.12 1.11
f1 (ppm)
1.00 1.08 1.13 1.18 1.23 2.39 1.37 5.30 2.12 1.11 2.08 1.00 1.00 1.03 1.09 1.10 1.32 1.19 2.35 1.12 1.13
f1 (ppm)

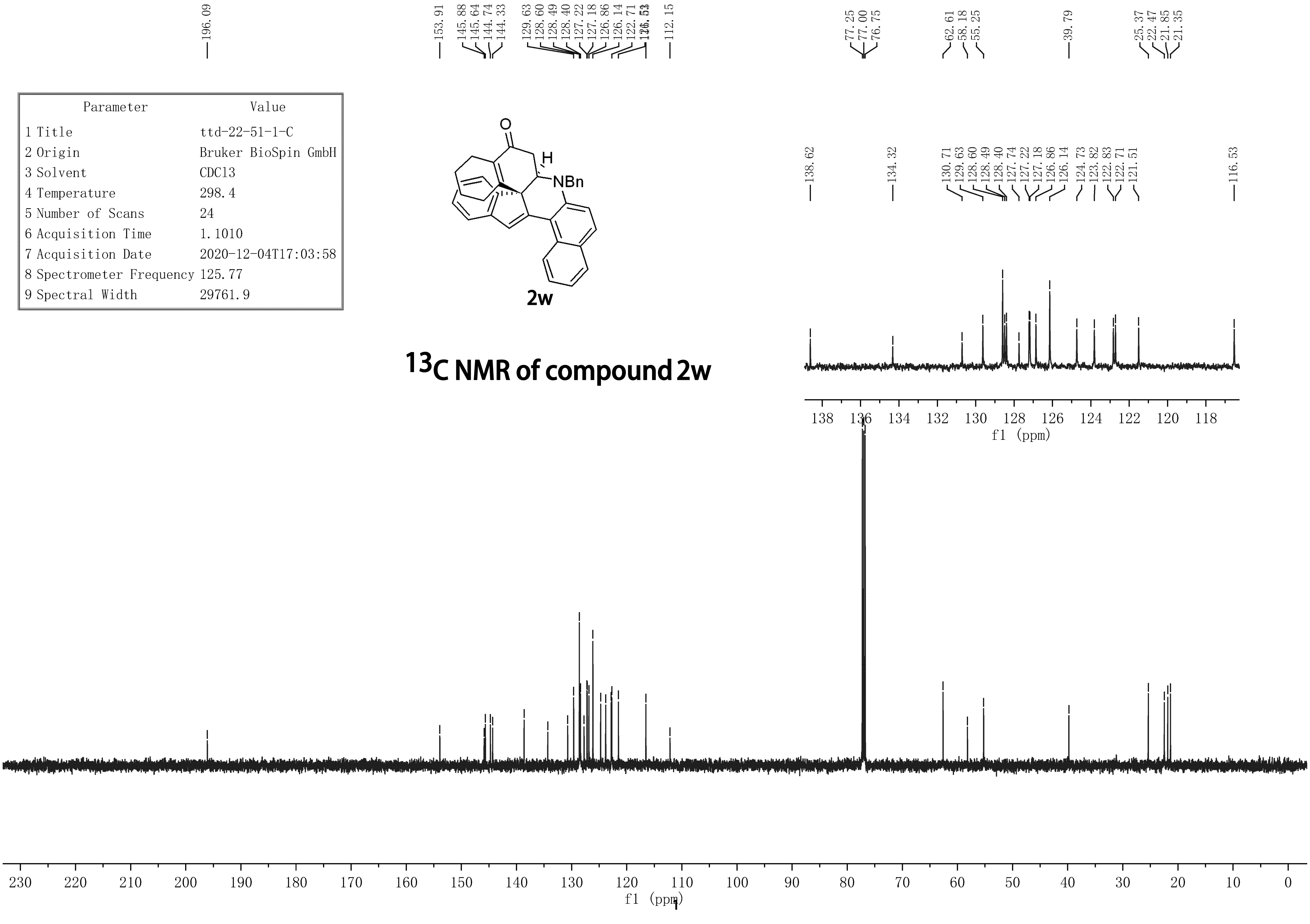
196.09
153.91 145.88 145.64 144.74 144.33 129.63 128.60 128.49 128.40 127.22 127.18 126.86 126.14 122.71 116.53 112.15
77.25 77.00 76.75
62.61 58.18 55.25
39.79
25.37 22.47 21.85 21.35
Parameter | Value
1 Title | ttd-22-51-1-C
2 Origin | Bruker BioSpin GmbH
3 Solvent | CDCl3
4 Temperature | 298.4
5 Number of Scans | 24
6 Acquisition Time | 1.1010
7 Acquisition Date | 2020-12-04T17:03:58
8 Spectrometer Frequency | 125.77
9 Spectral Width | 29761.9
O
H
NBn
2w
13C NMR of compound 2w
138.62 134.32 130.71 129.63 128.60 128.49 128.40 127.74 127.22 127.18 126.86 126.14 124.73 123.82 122.83 122.71 121.51 116.53
f1 (ppm)
f1 (ppm)

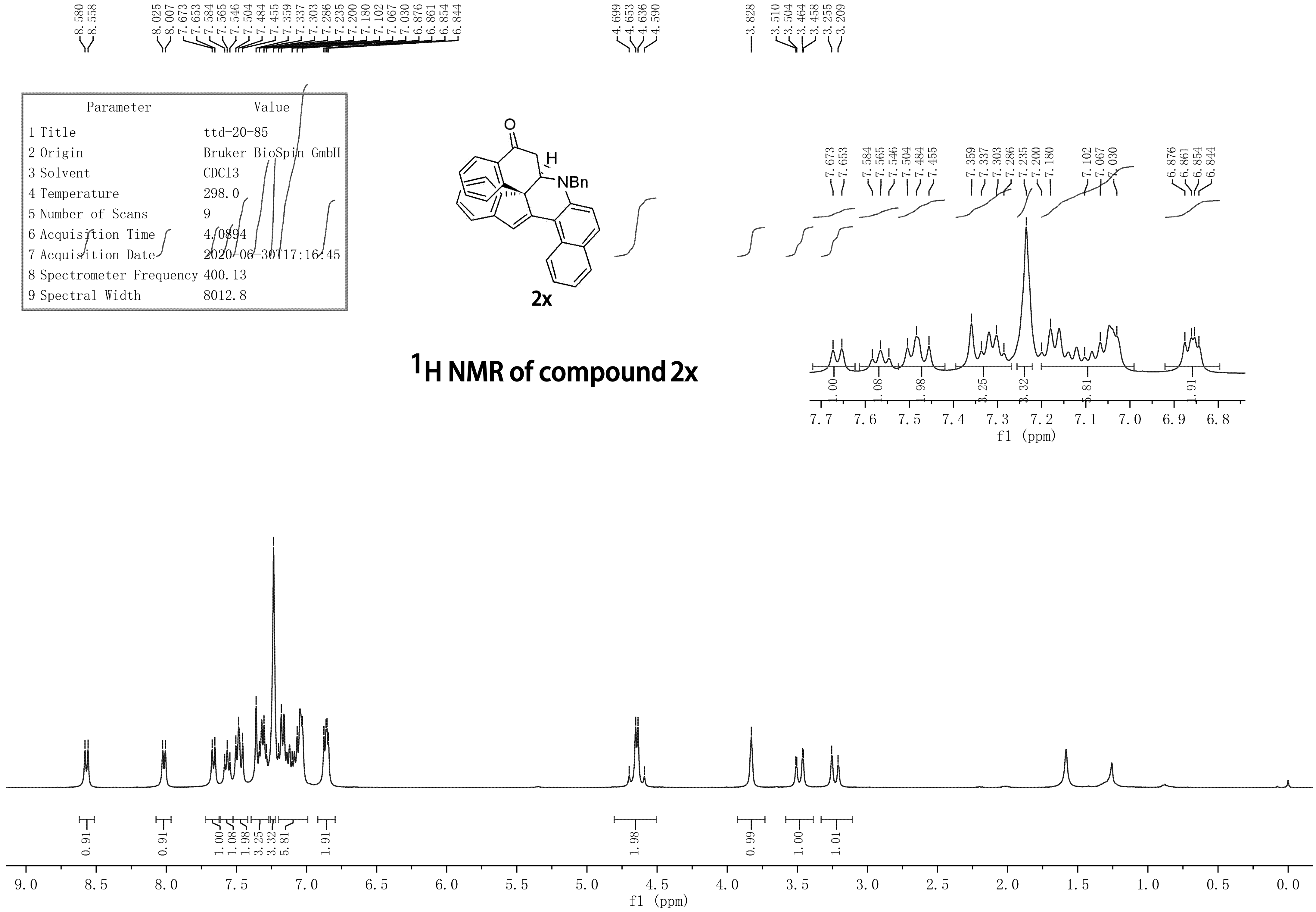

Parameter	Value
1 Title	ttd-20-85
2 Origin	Bruker BioSpin GmbH
3 Solvent	CDCl3
4 Temperature	298.0
5 Number of Scans	9
6 Acquisition Time	4.0894
7 Acquisition Date	2020-06-30T17:16:45
8 Spectrometer Frequency	400.13
9 Spectral Width	8012.8

2x

^{1}H NMR of compound 2x

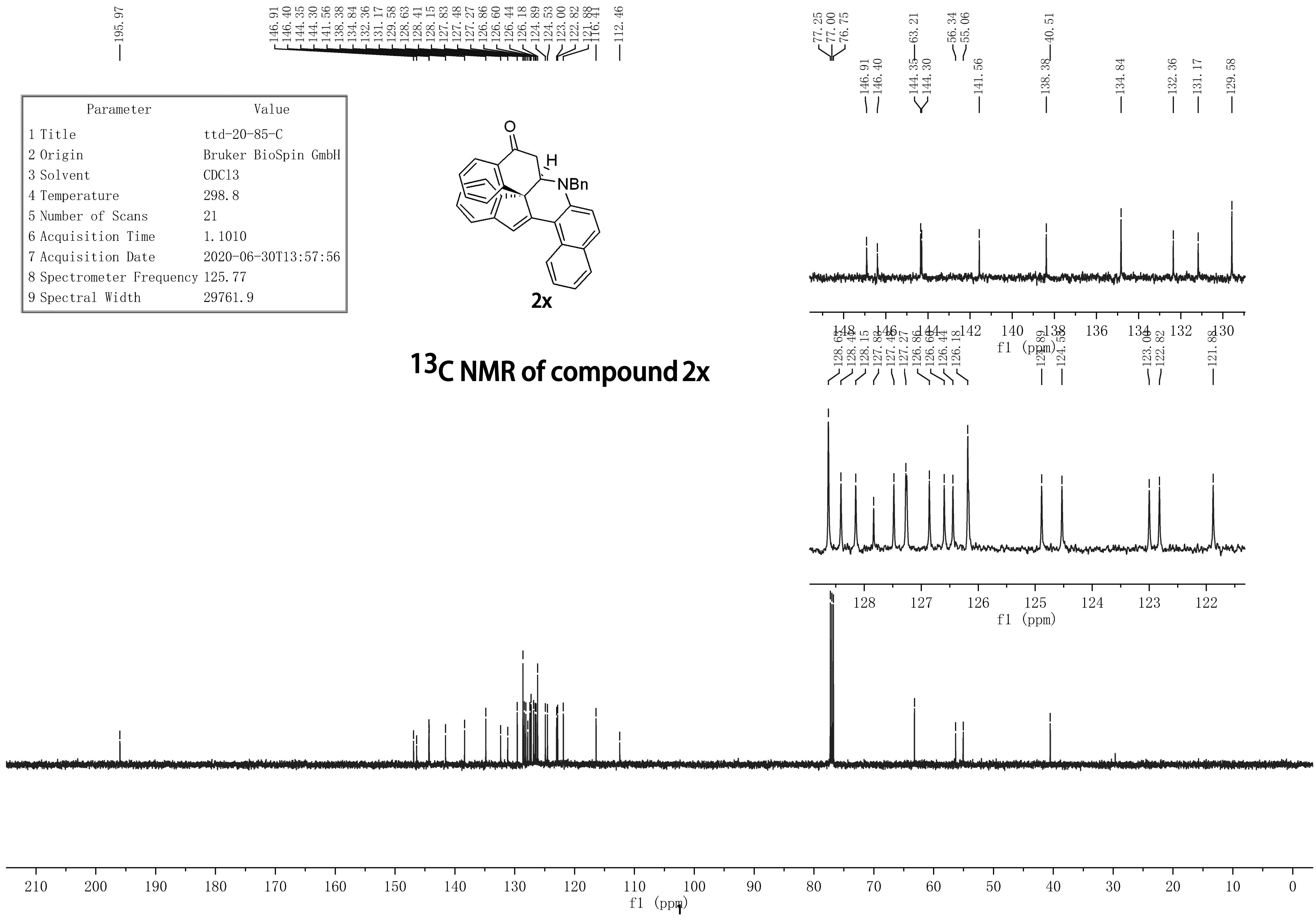

Parameter	Value
1 Title	ttd-20-85-C
2 Origin	Bruker BioSpin GmbH
3 Solvent	CDCl3
4 Temperature	298.8
5 Number of Scans	21
6 Acquisition Time	1.1010
7 Acquisition Date	2020-06-30T13:57:56
8 Spectrometer Frequency	125.77
9 Spectral Width	29761.9

2x

^{13}C NMR of compound 2x

Parameter	Value
1 Title	ttd-22-172
2 Origin	Bruker BioSpin GmbH
3 Solvent	CDCl3
4 Temperature	296.0
5 Number of Scans	7
6 Acquisition Time	3.1719
7 Acquisition Date	2021-01-14T15:49:03
8 Spectrometer Frequency	500.17
9 Spectral Width	10330.6

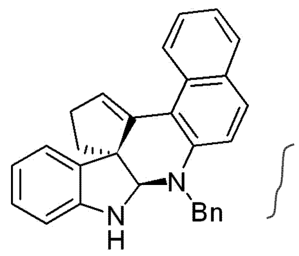

4a

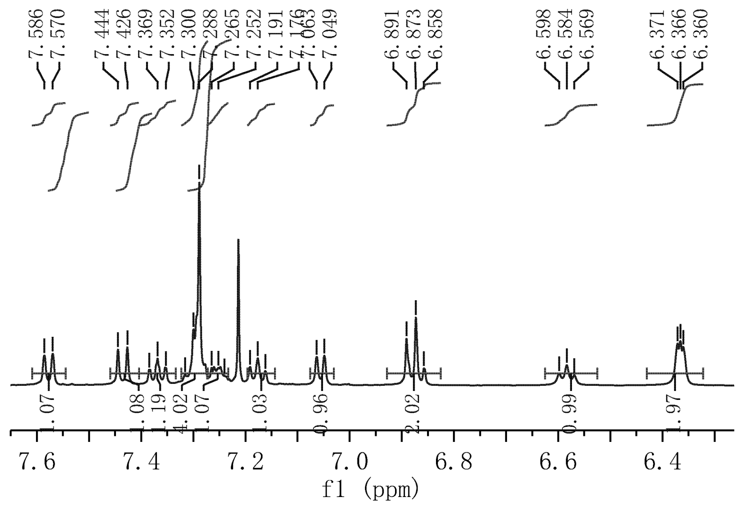

^{1}H NMR of compound 4a

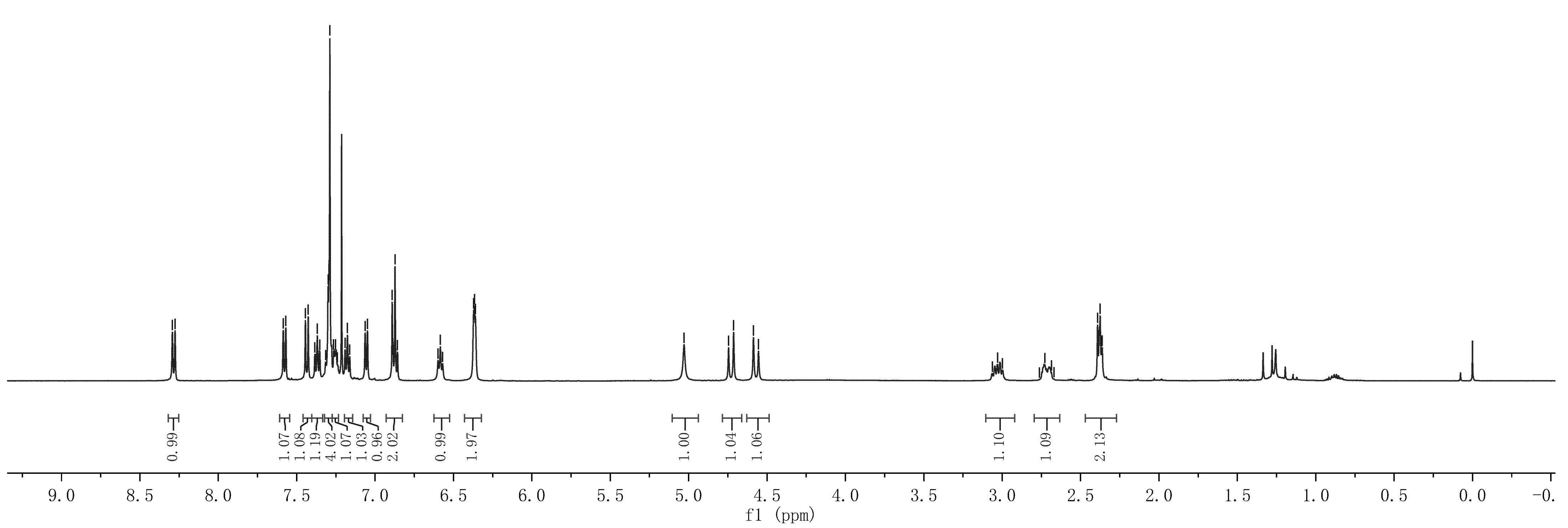

Parameter	Value
1 Title	ttd-22-172-C
2 Origin	Bruker BioSpin GmbH
3 Solvent	CDCl3
4 Temperature	296.6
5 Number of Scans	105
6 Acquisition Time	1.1010
7 Acquisition Date	2021-01-14T15:51:42
8 Spectrometer Frequency	125.77
9 Spectral Width	29761.9

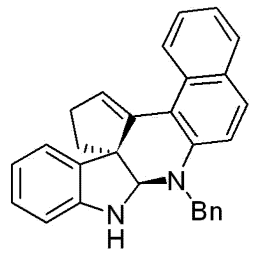

4a

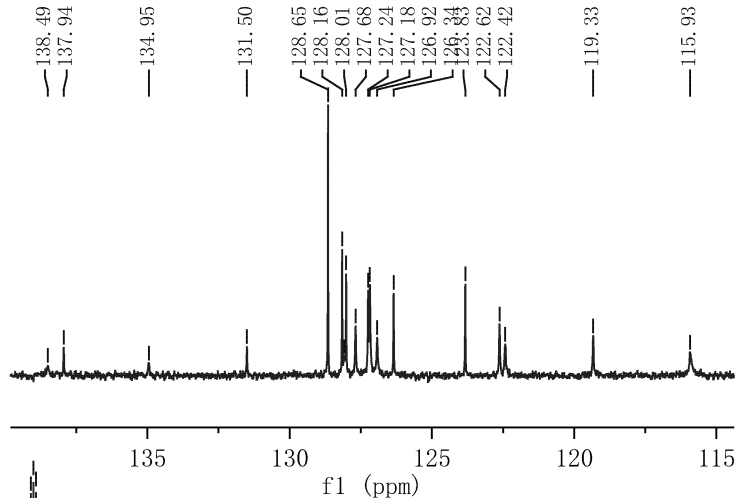

^{13}C NMR of compound 4a

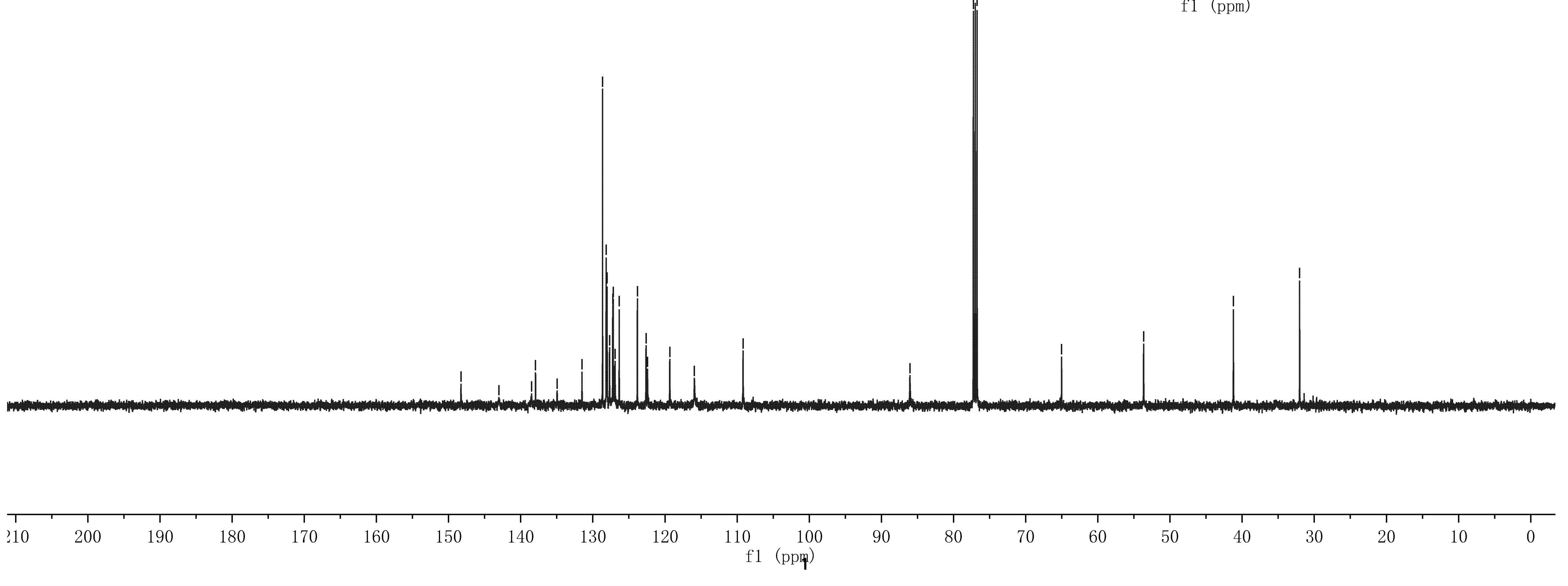

Parameter	Value
1 Title	ttd-22-172-C135
2 Origin	Bruker BioSpin GmbH
3 Solvent	CDCl3
4 Temperature	296.8
5 Number of Scans	34
6 Acquisition Time	1.1010
7 Acquisition Date	2021-01-14T15:57:45
8 Spectrometer Frequency	125.77
9 Spectral Width	29761.9

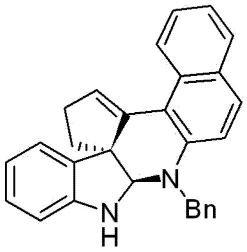

4a

^{13}C dept135 NMR of compound 4a

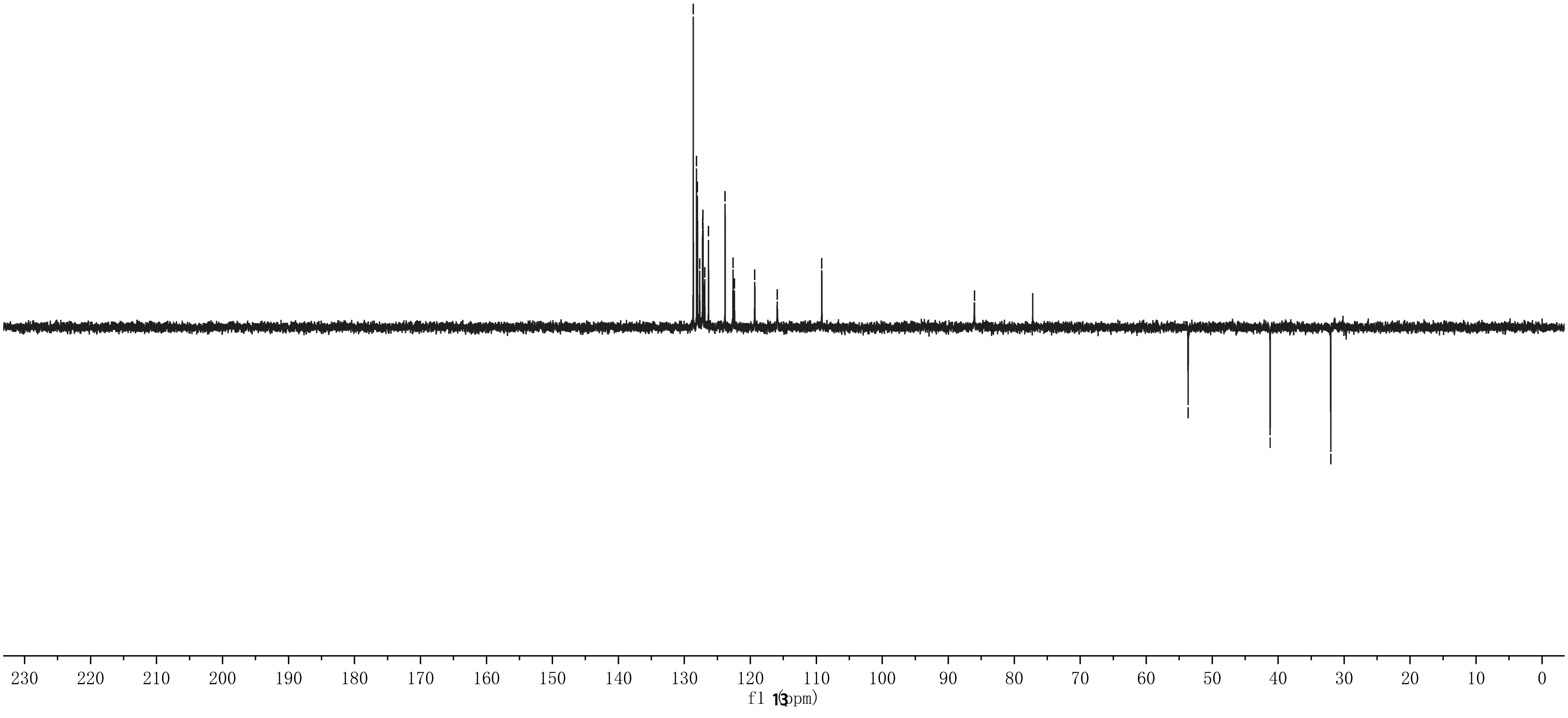

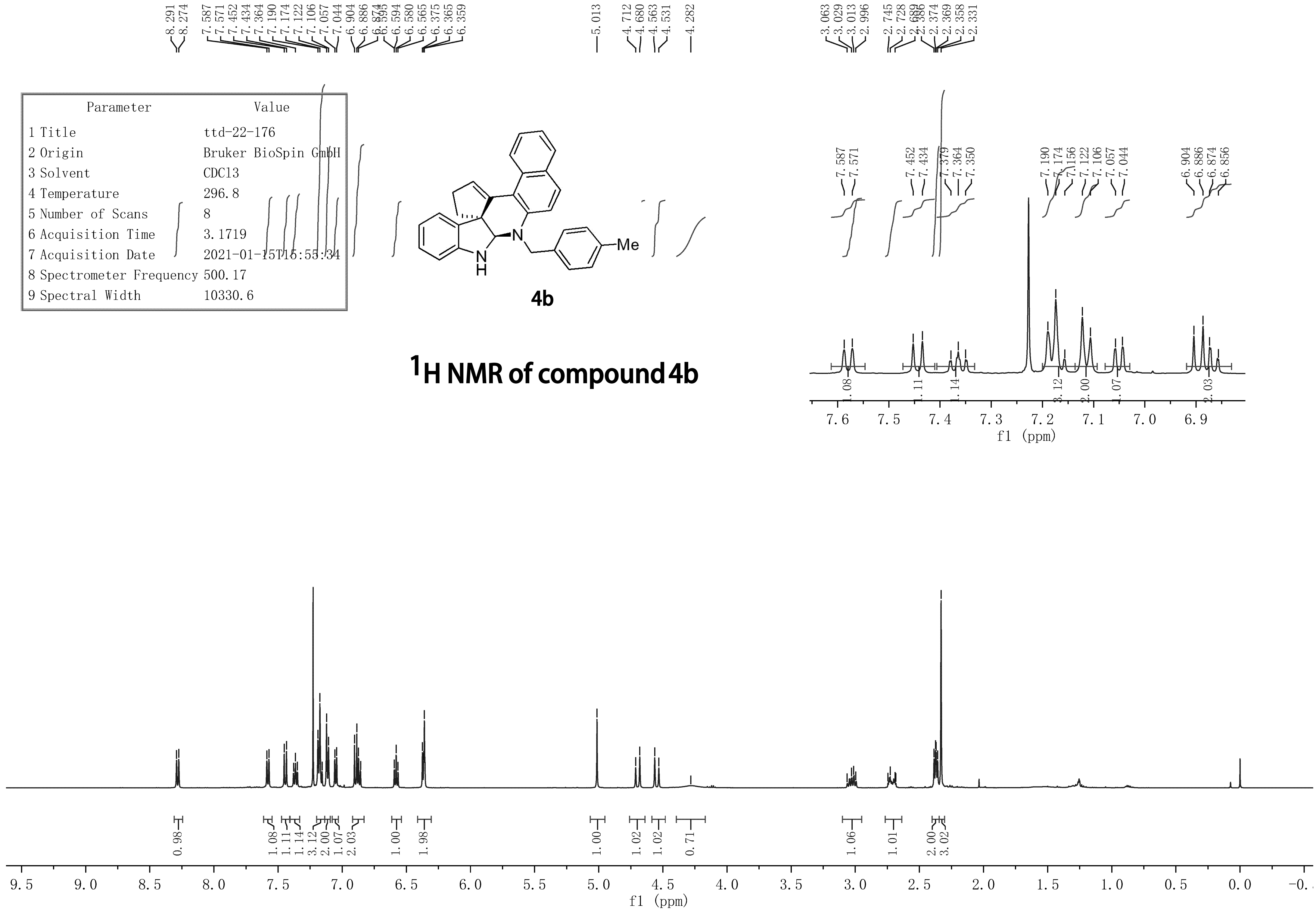

^{1}H NMR of compound 4b

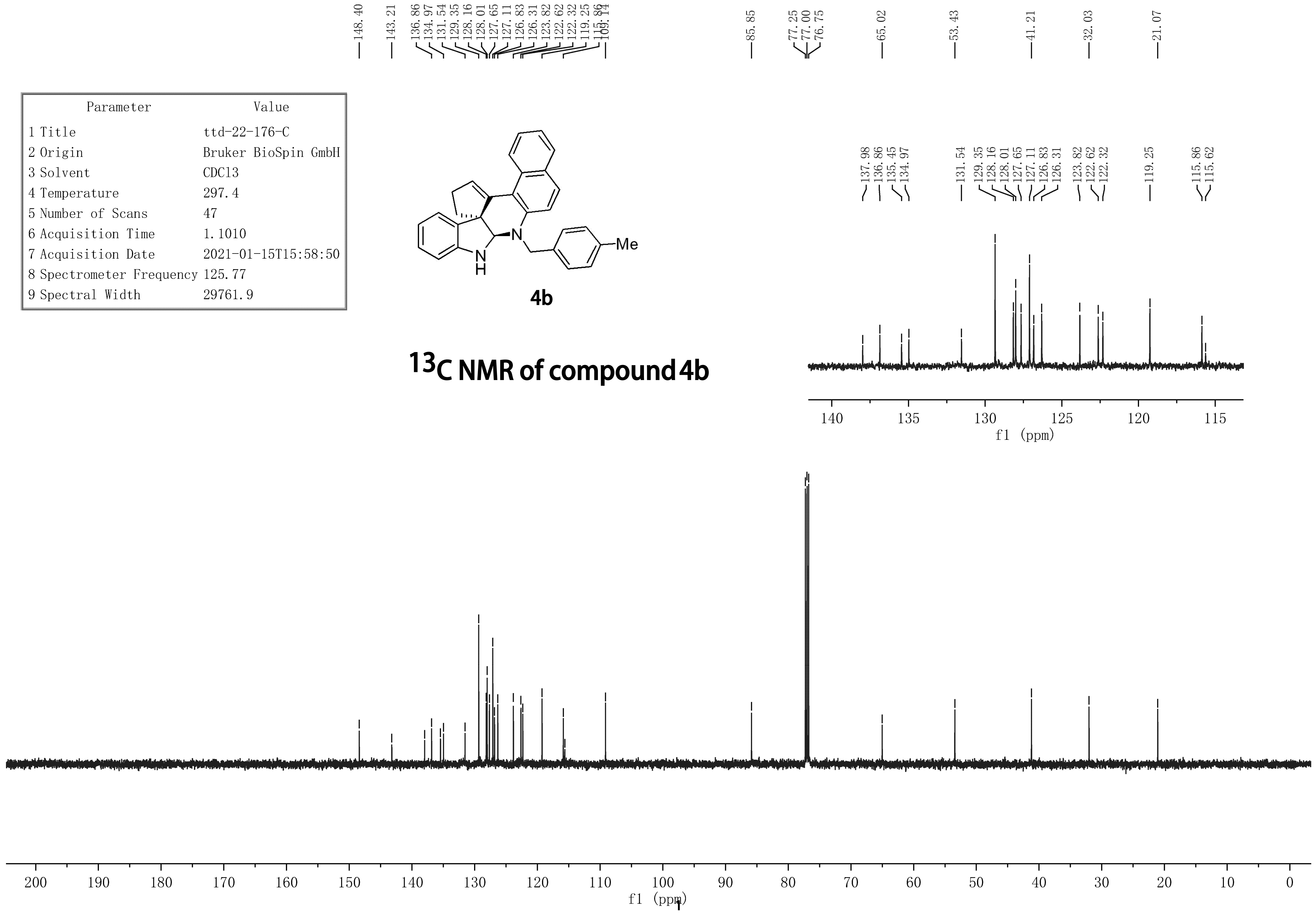

^{13}C NMR of compound 4b

Parameter	Value
1 Title	ttd-22-194
2 Origin	Bruker BioSpin GmbH
3 Solvent	CDCl3
4 Temperature	298.2
5 Number of Scans	7
6 Acquisition Time	3.1719
7 Acquisition Date	2021-01-21T16:05:21
8 Spectrometer Frequency	500.17
9 Spectral Width	10330.6

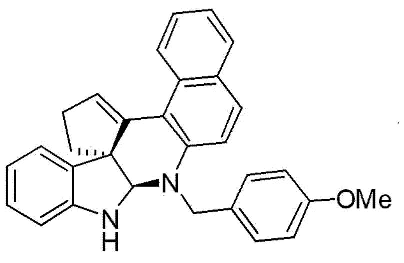

4c

^{1}H NMR of compound 4c

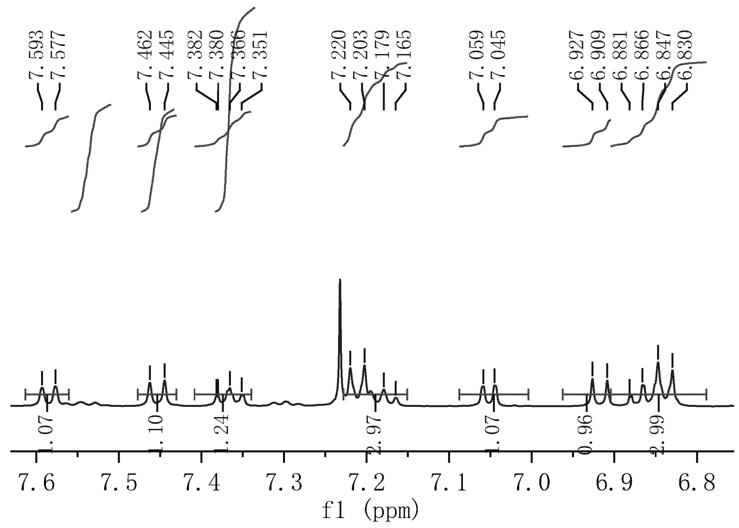

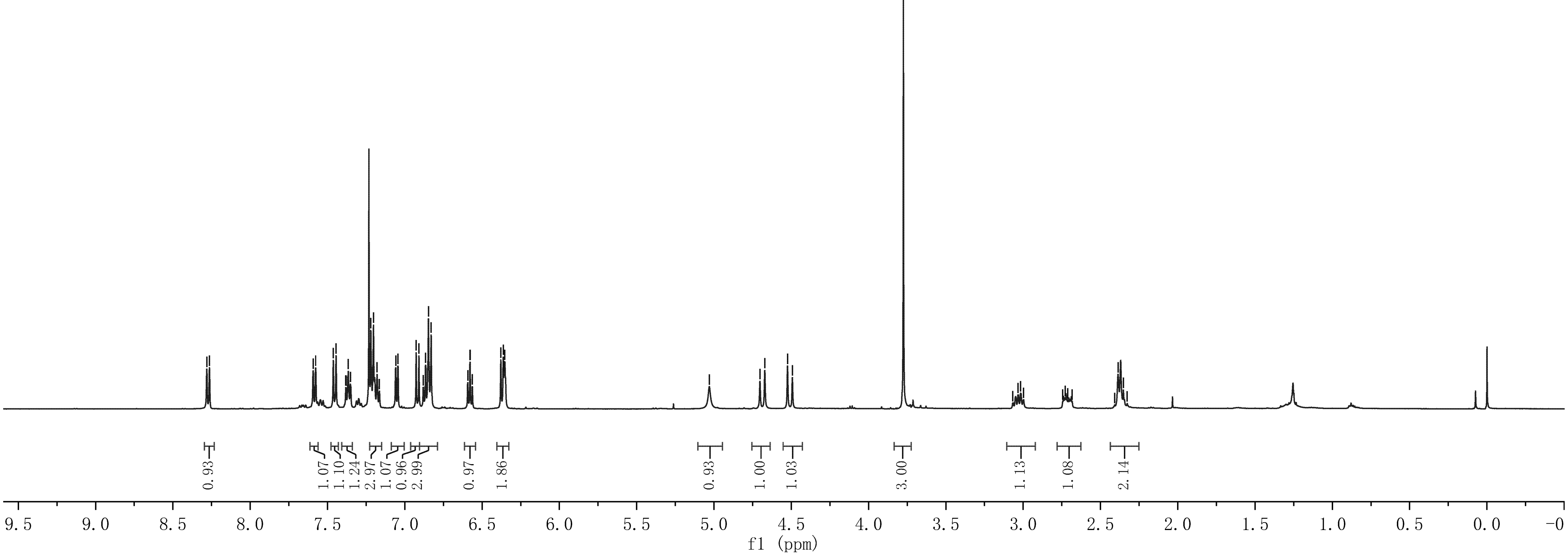

Parameter	Value
1 Title	ttd-22-194-C
2 Origin	Bruker BioSpin GmbH
3 Solvent	CDCl3
4 Temperature	298.8
5 Number of Scans	145
6 Acquisition Time	1.1010
7 Acquisition Date	2021-01-21T16:06:55
8 Spectrometer Frequency	125.77
9 Spectral Width	29761.9

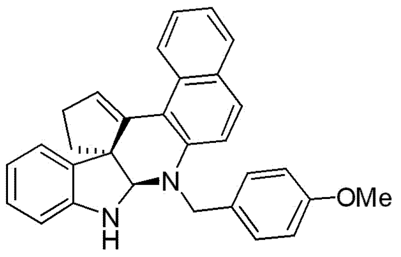

4c

^{13}C NMR of compound 4c

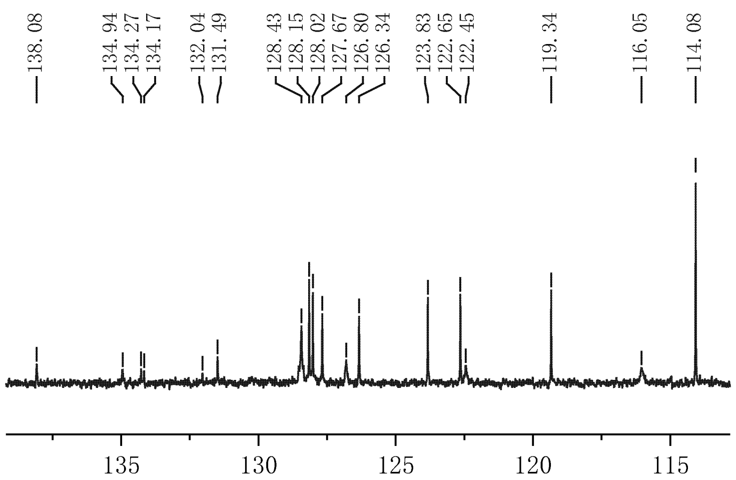

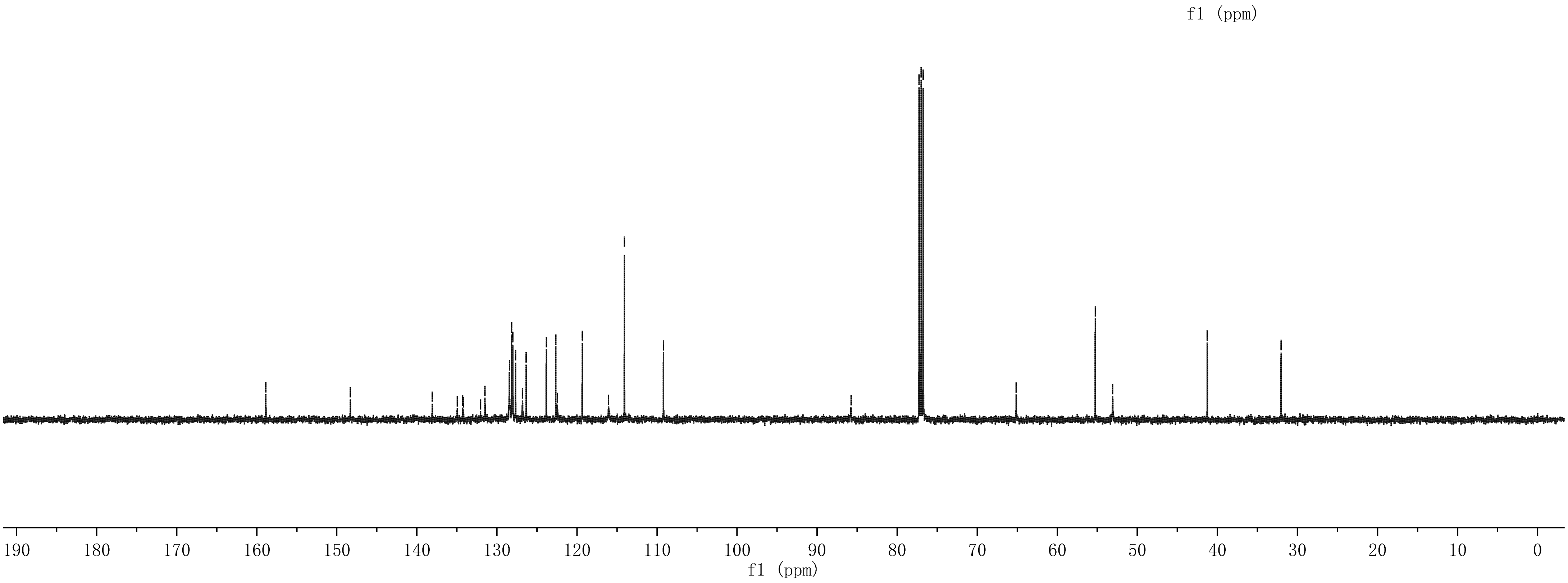

Parameter	Value
1 Title	ttd-22-238-H
2 Origin	Bruker BioSpin GmbH
3 Solvent	CDCl3
4 Temperature	298.2
5 Number of Scans	10
6 Acquisition Time	3.1719
7 Acquisition Date	2021-03-10T15:45:22
8 Spectrometer Frequency	500.17
9 Spectral Width	10330.6

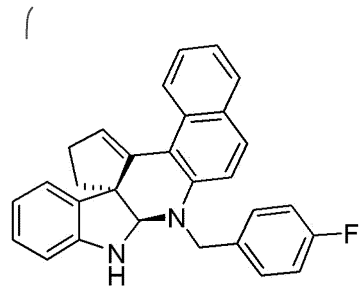

4d

^{1}H NMR of compound 4d

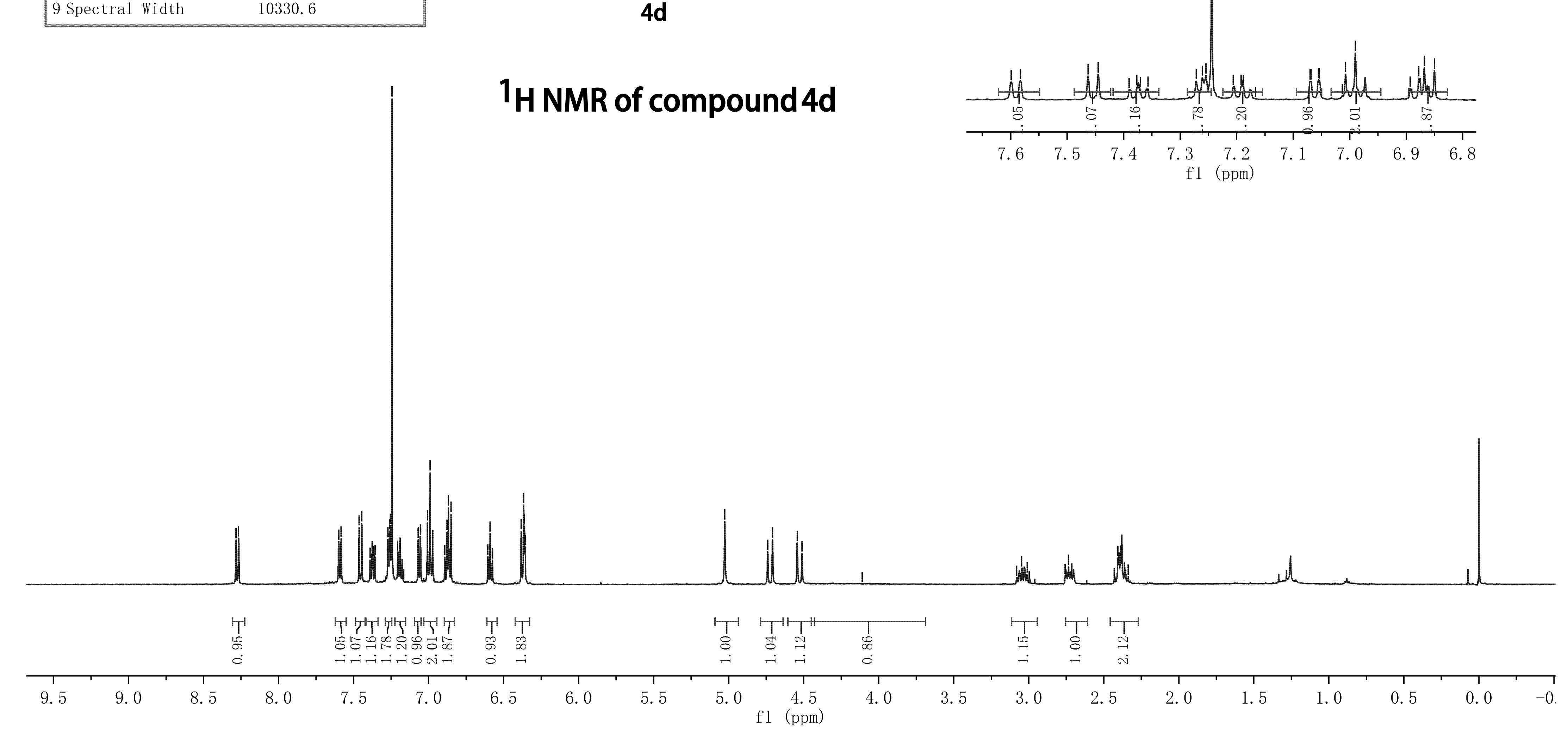

Parameter	Value
1 Title	ttd-22-238-C
2 Origin	Bruker BioSpin GmbH
3 Solvent	CDCl3
4 Temperature	298.7
5 Number of Scans	187
6 Acquisition Time	1.1010
7 Acquisition Date	2021-03-06T15:47:47
8 Spectrometer Frequency	125.77
9 Spectral Width	29761.9

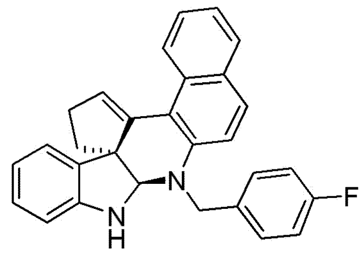

4d

^{13}C NMR of compound 4d

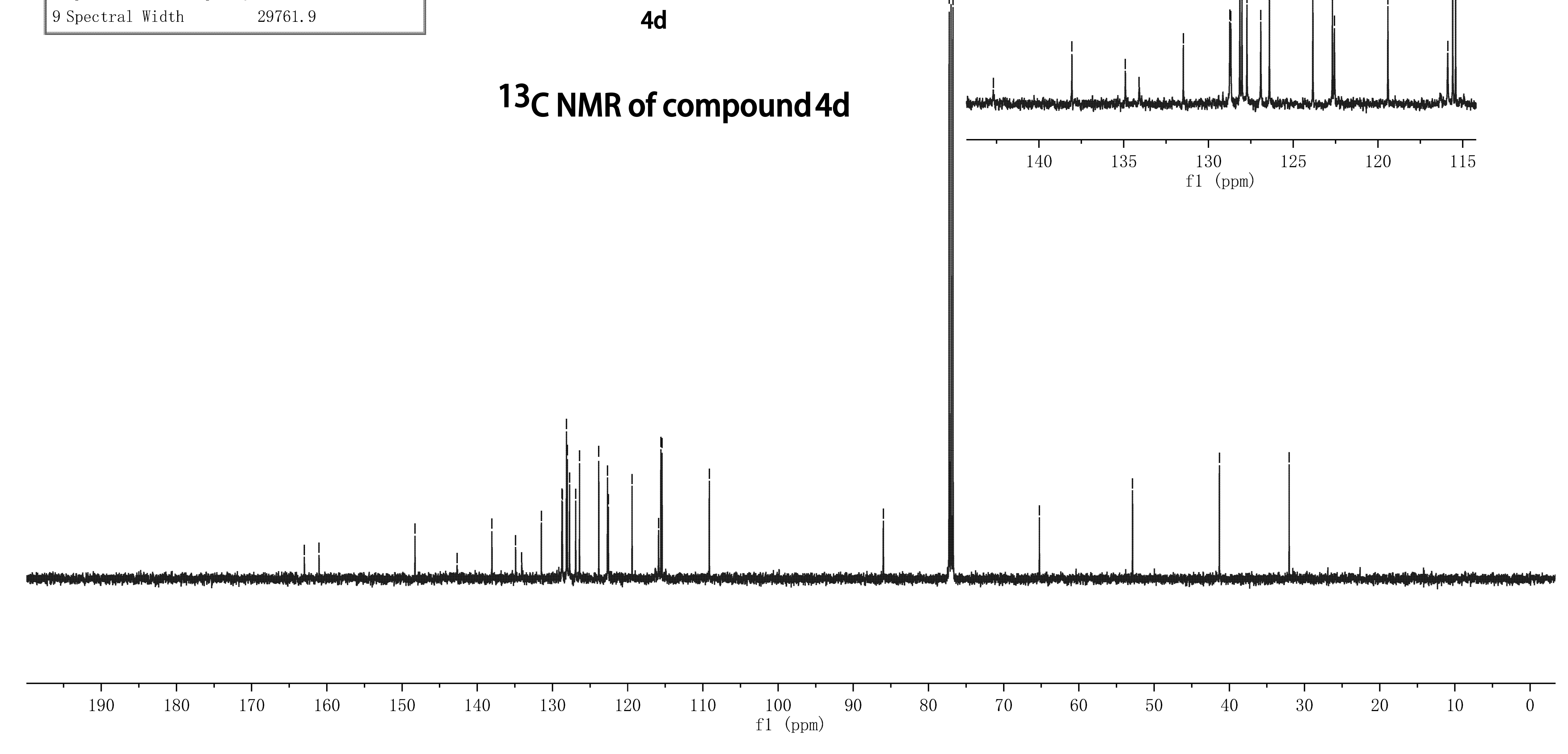

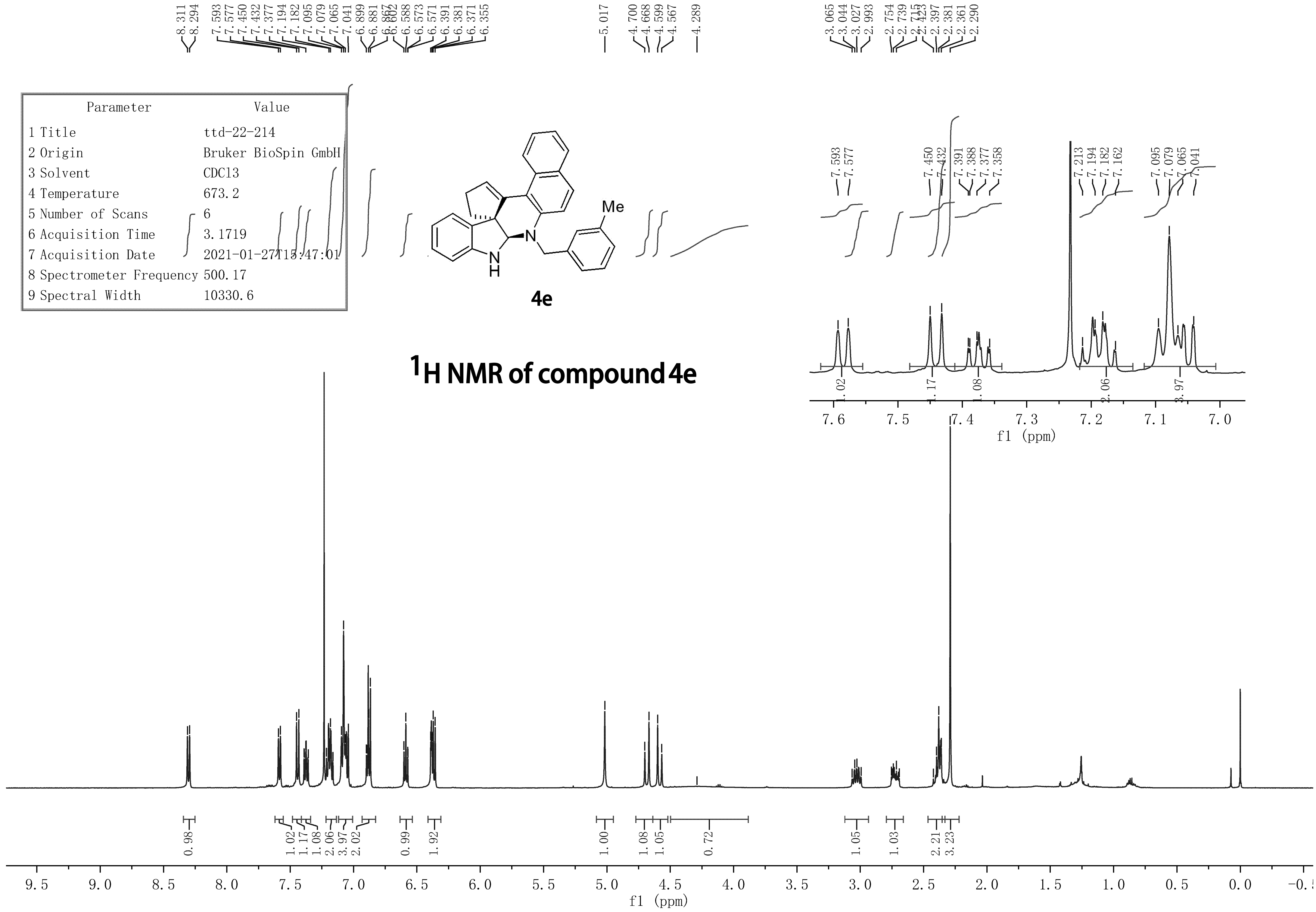
Parameter Value
1 Title ttd-22-214
2 Origin Bruker BioSpin GmbH
3 Solvent CDCl3
4 Temperature 673.2
5 Number of Scans 6
6 Acquisition Time 3.1719
7 Acquisition Date 2021-01-27T15:47:01
8 Spectrometer Frequency 500.17
9 Spectral Width 10330.6
Me
N
H
4e
1H NMR of compound 4e
f1 (ppm)

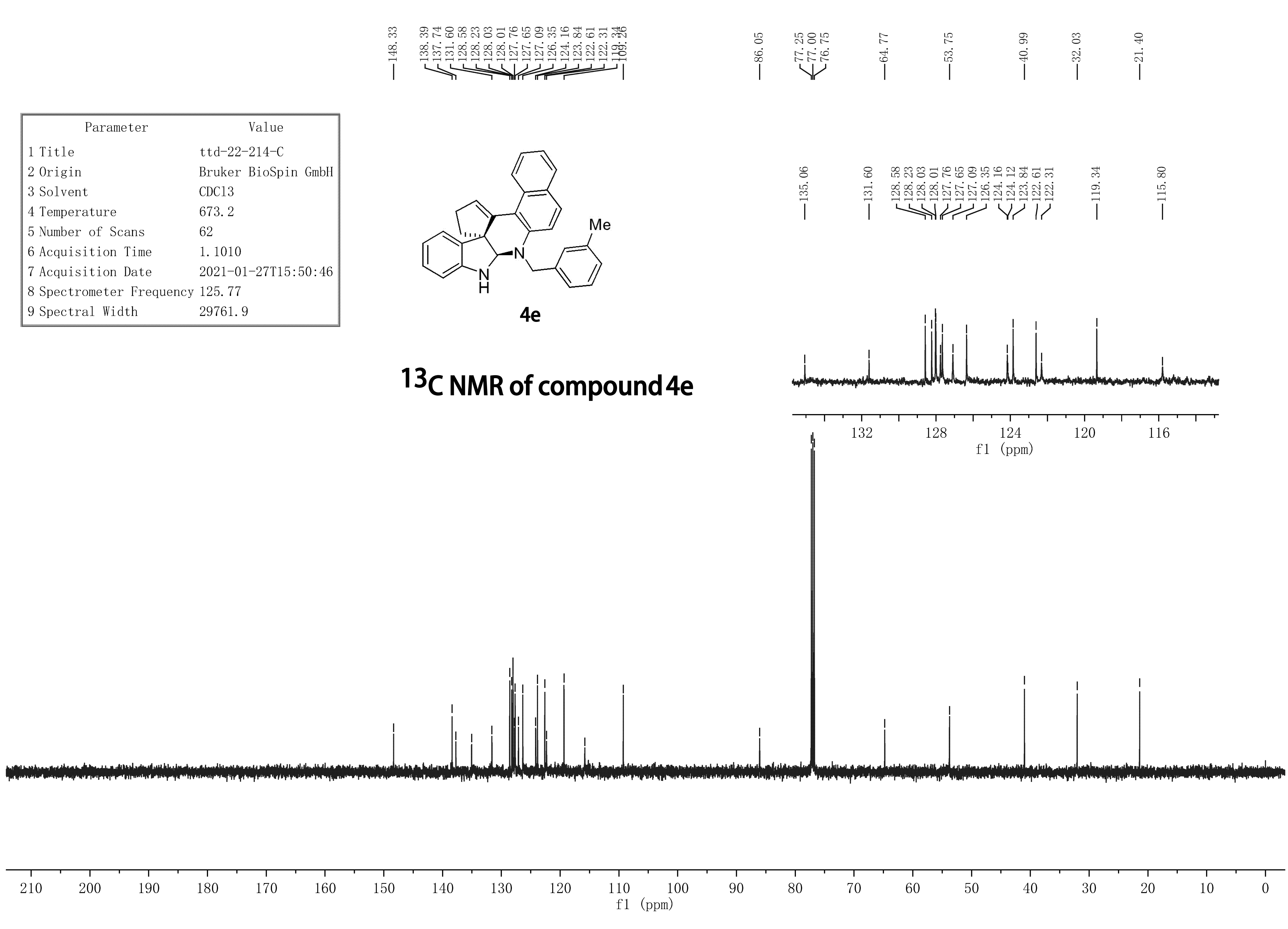
Parameter Value
1 Title ttd-22-214-C
2 Origin Bruker BioSpin GmbH
3 Solvent CDCl3
4 Temperature 673.2
5 Number of Scans 62
6 Acquisition Time 1.1010
7 Acquisition Date 2021-01-27T15:50:46
8 Spectrometer Frequency 125.77
9 Spectral Width 29761.9
Me
N
H
4e
13C NMR of compound 4e
f1 (ppm)

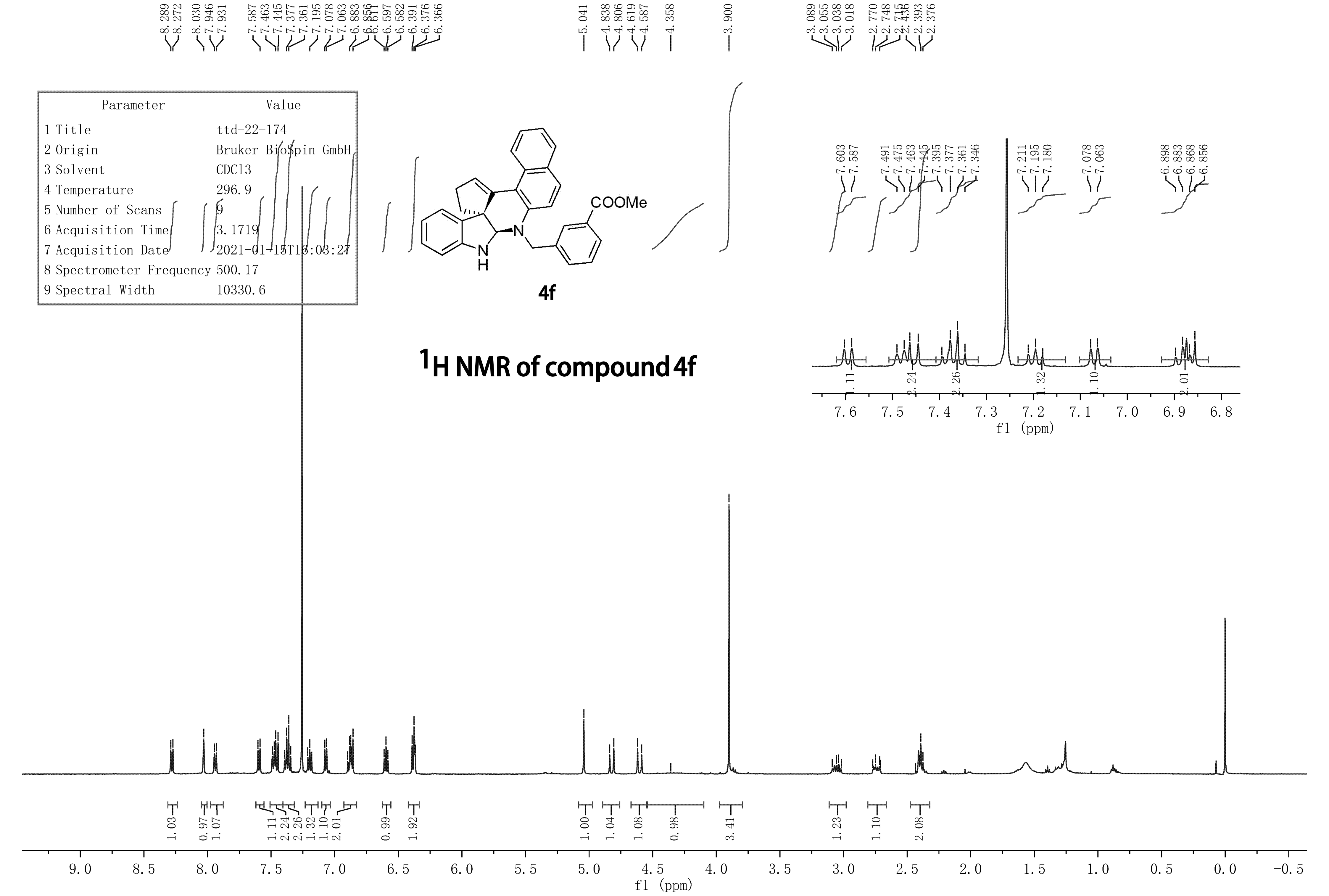

1H NMR of compound 4f

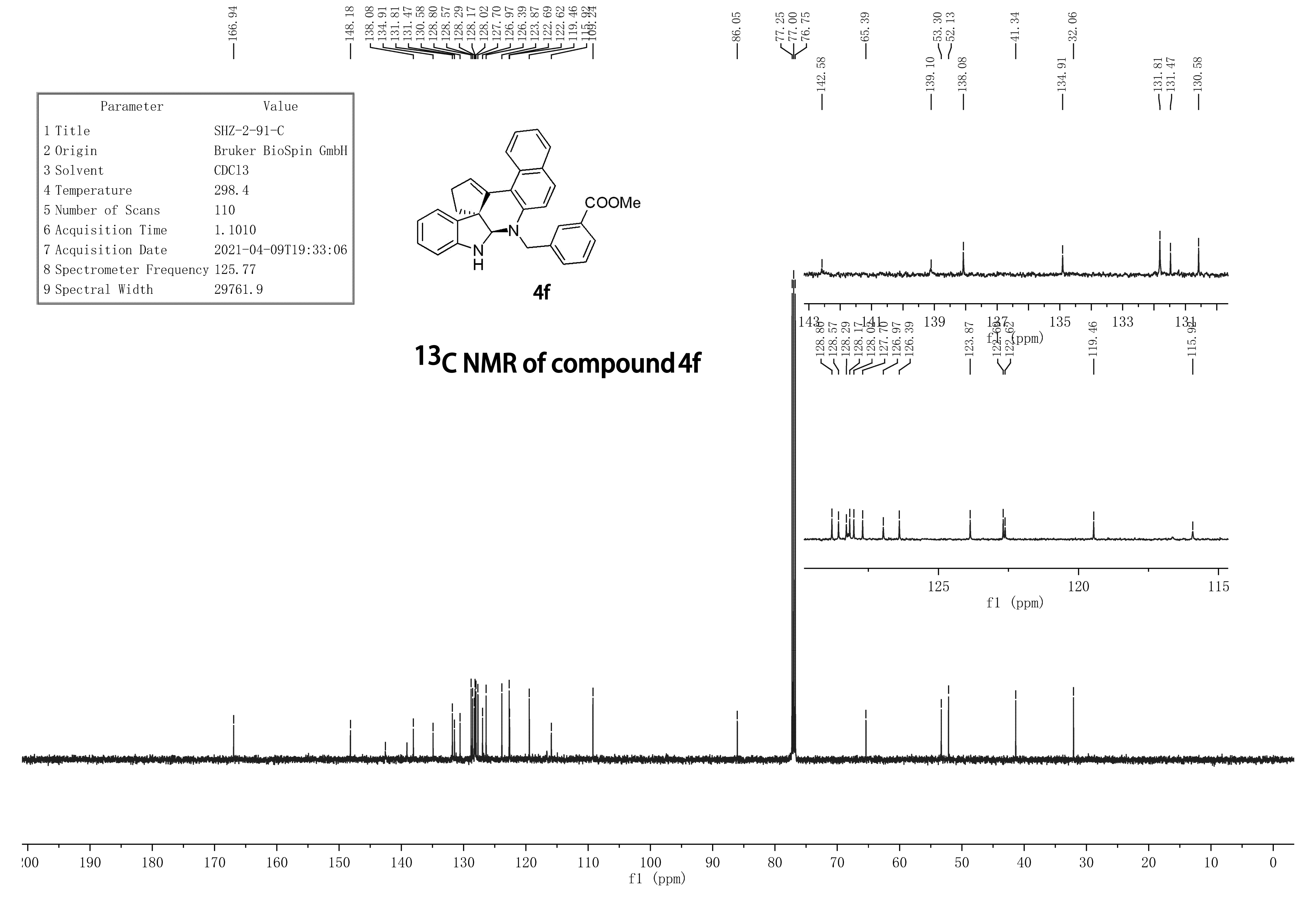

13C NMR of compound 4f

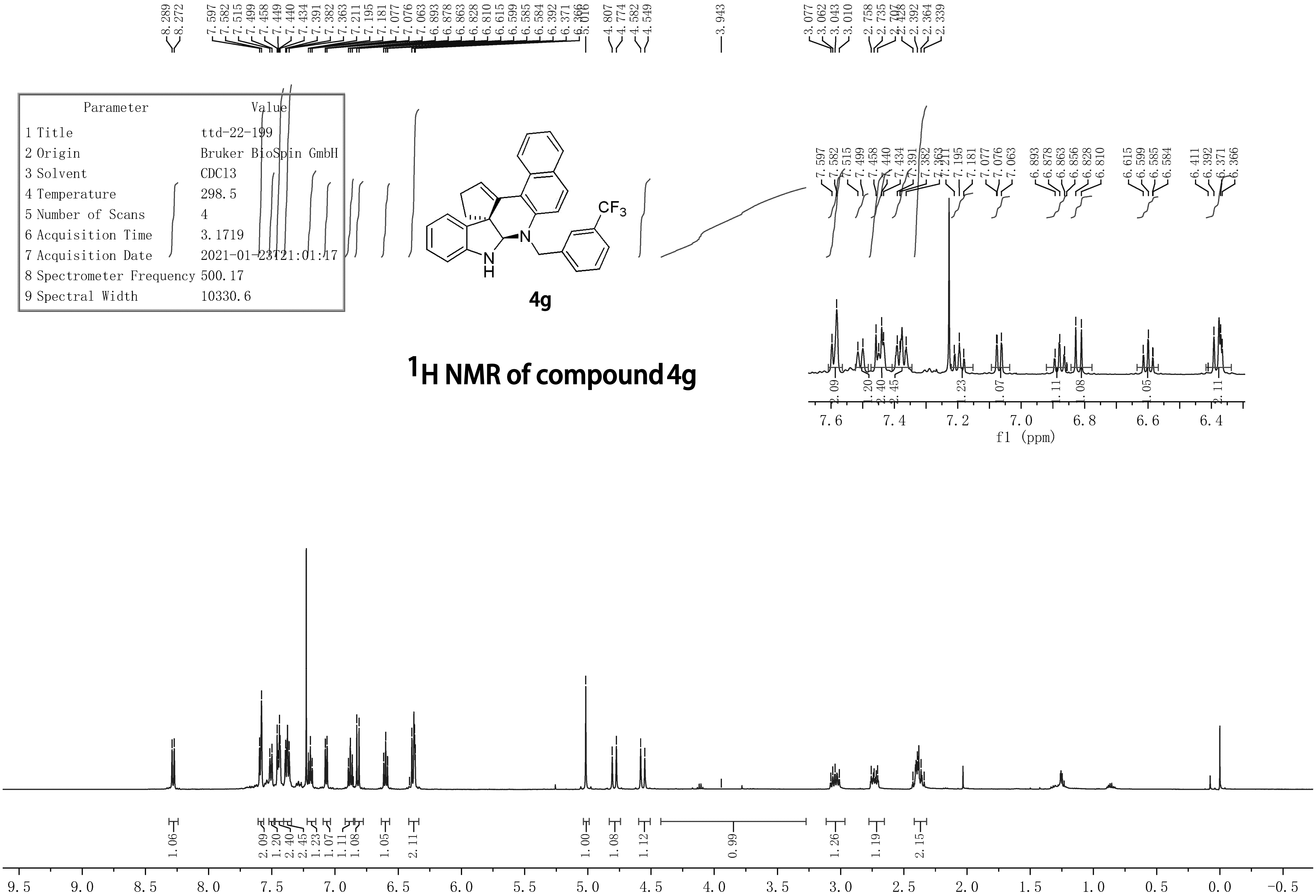

Parameter	Value
1 Title	ttd-22-199
2 Origin	Bruker BioSpin GmbH
3 Solvent	CDCl3
4 Temperature	298.5
5 Number of Scans	4
6 Acquisition Time	3.1719
7 Acquisition Date	2021-01-23T21:01:17
8 Spectrometer Frequency	500.17
9 Spectral Width	10330.6

4g

1H NMR of compound 4g

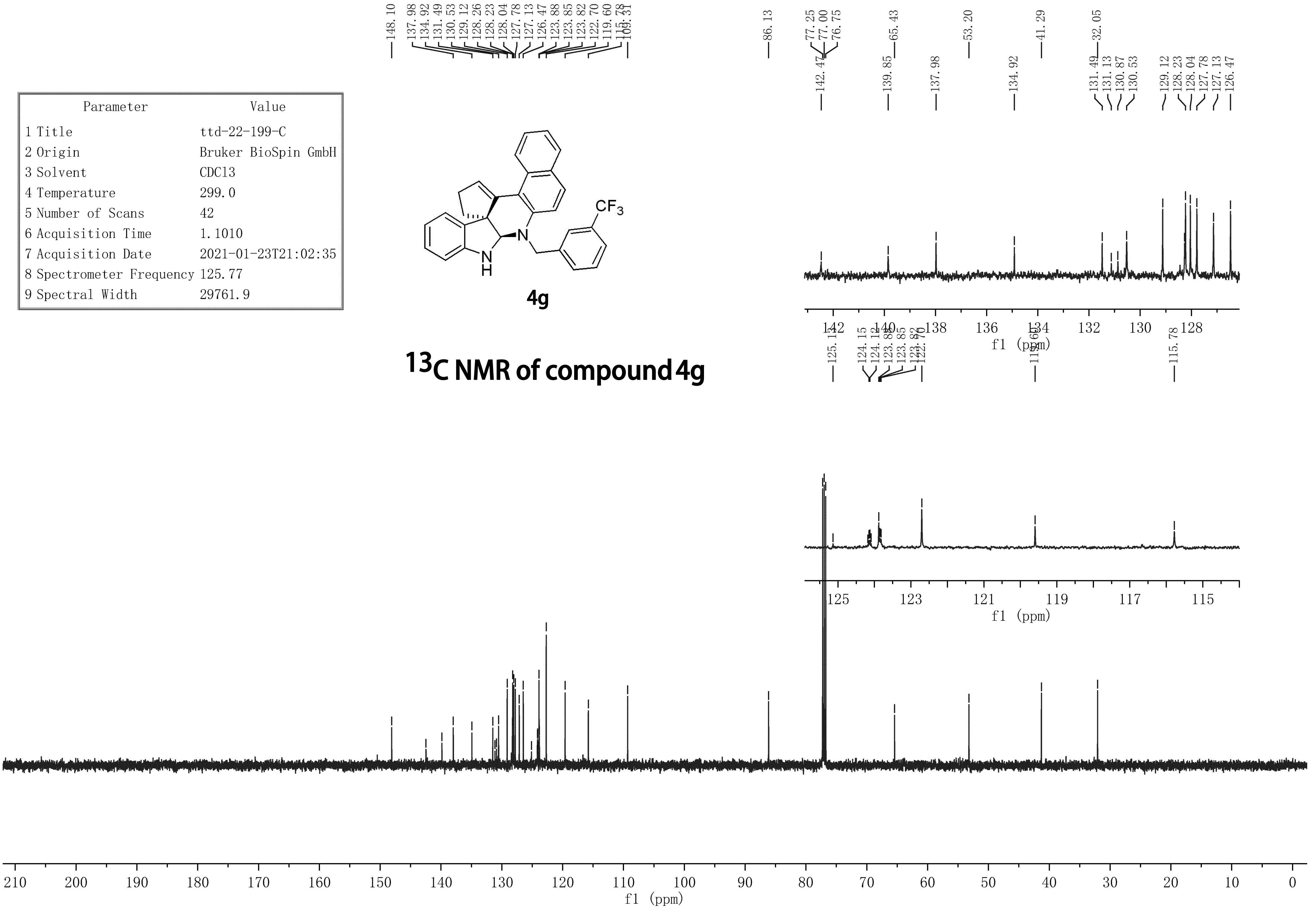

Parameter	Value
1 Title	ttd-22-199-C
2 Origin	Bruker BioSpin GmbH
3 Solvent	CDCl3
4 Temperature	299.0
5 Number of Scans	42
6 Acquisition Time	1.1010
7 Acquisition Date	2021-01-23T21:02:35
8 Spectrometer Frequency	125.77
9 Spectral Width	29761.9

4g

13C NMR of compound 4g

Parameter	Value
1 Title	ttd-22-213
2 Origin	Bruker BioSpin GmbH
3 Solvent	CDCl3
4 Temperature	673.2
5 Number of Scans	6
6 Acquisition Time	3.1719
7 Acquisition Date	2021-01-27T15:56:52
8 Spectrometer Frequency	500.17
9 Spectral Width	10330.6

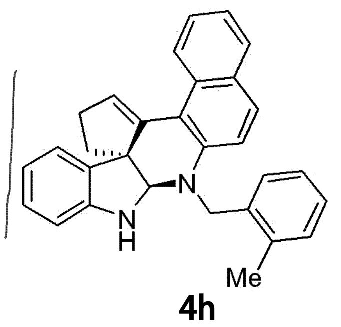

4h

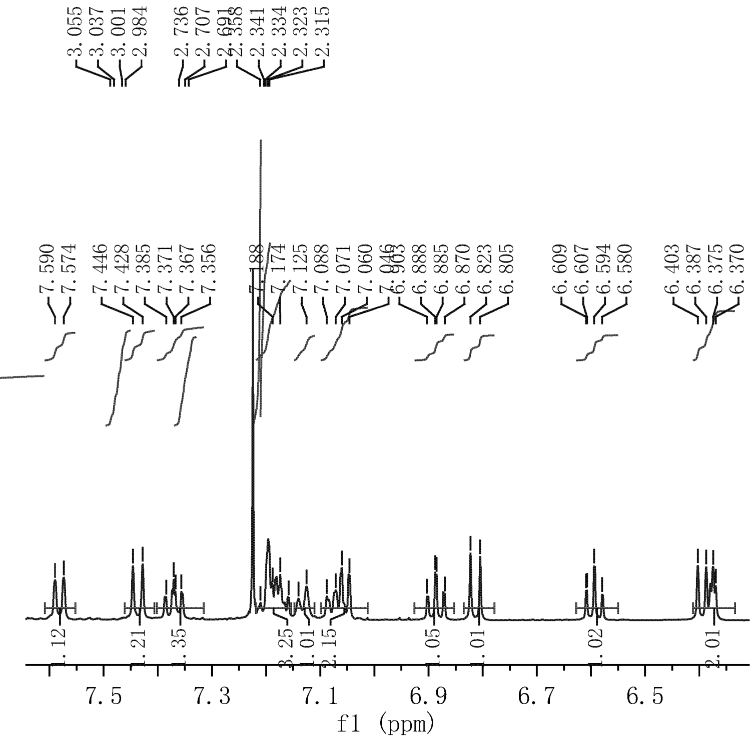

^{1}H NMR of compound 4h

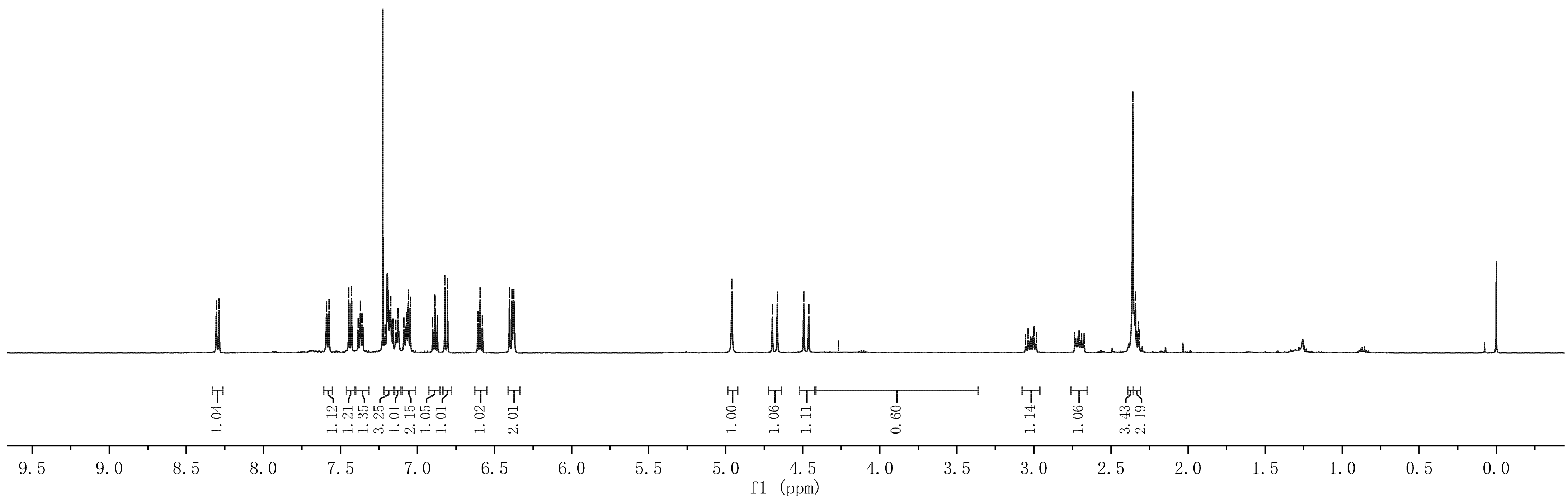

Parameter	Value
1 Title	ttd-22-213-C
2 Origin	Bruker BioSpin GmbH
3 Solvent	CDCl3
4 Temperature	673.2
5 Number of Scans	53
6 Acquisition Time	1.1010
7 Acquisition Date	2021-01-27T15:57:55
8 Spectrometer Frequency	125.77
9 Spectral Width	29761.9

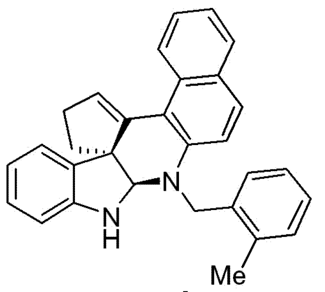

4h

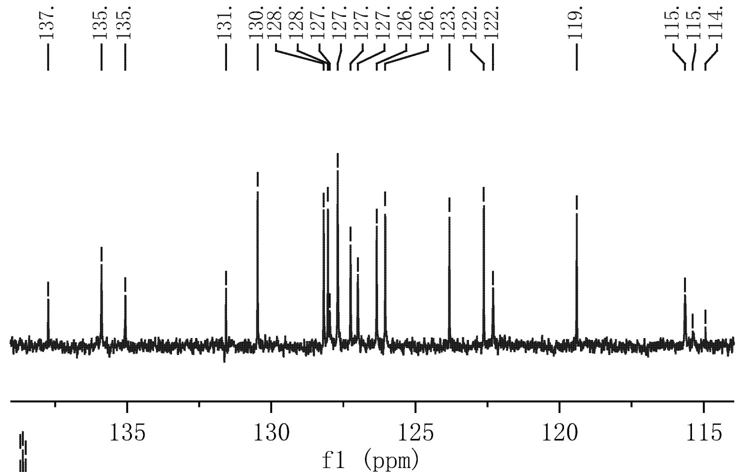

^{13}C NMR of compound 4h

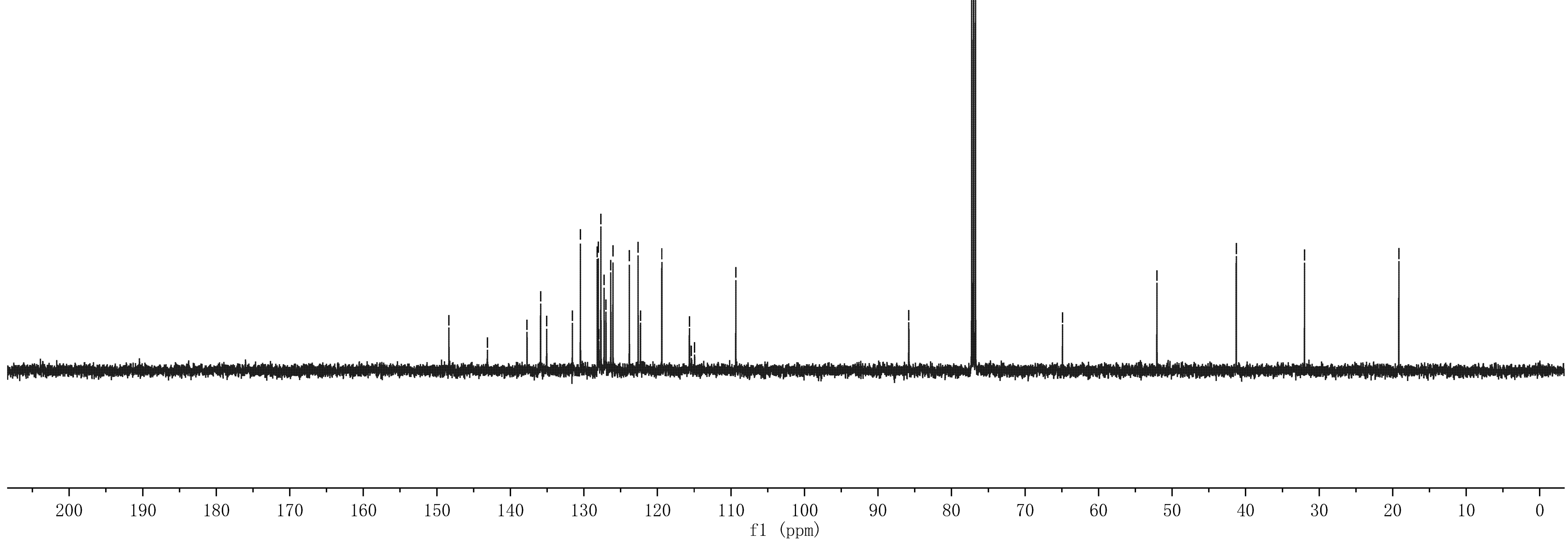

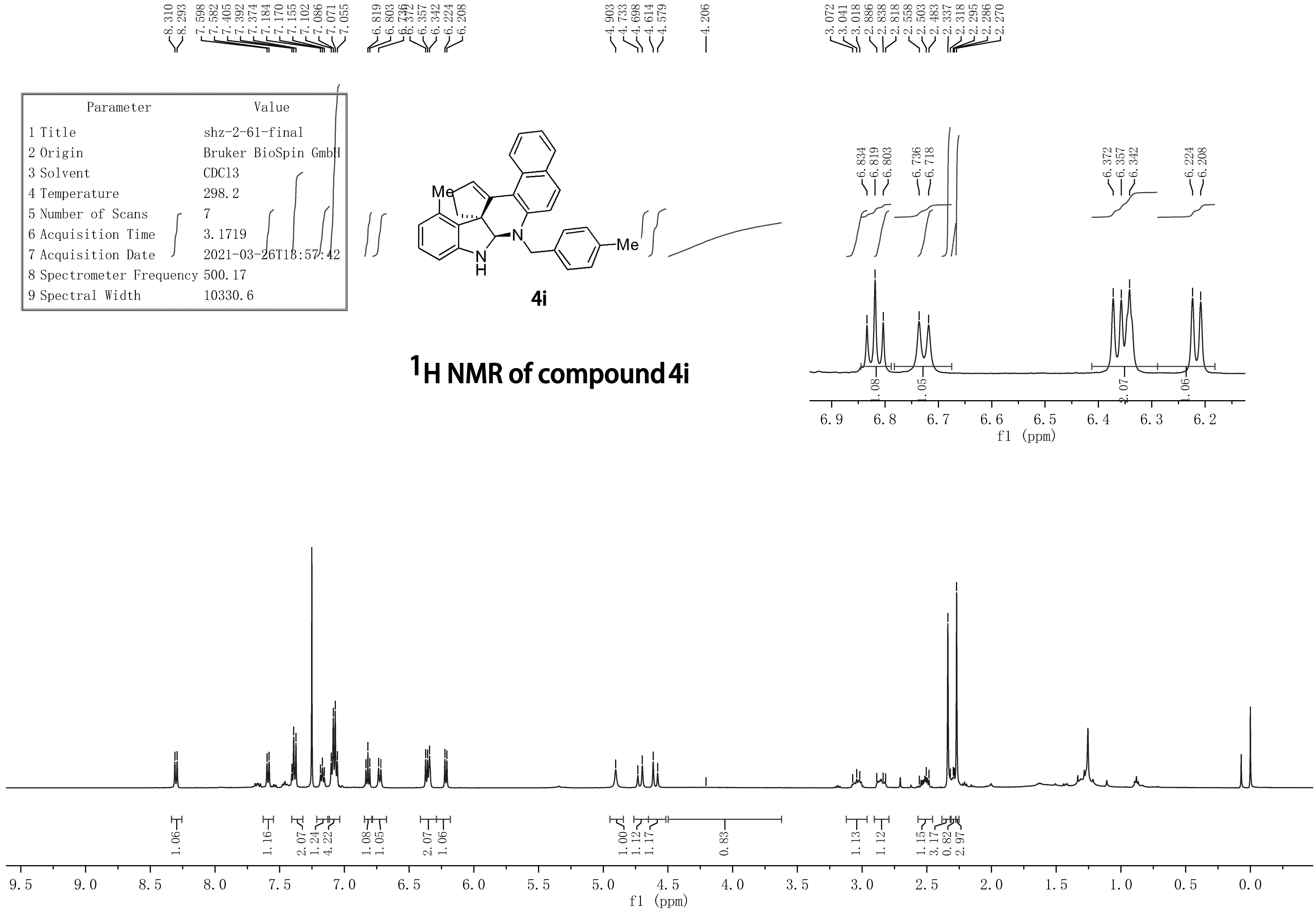

^{1}H NMR of compound 4i

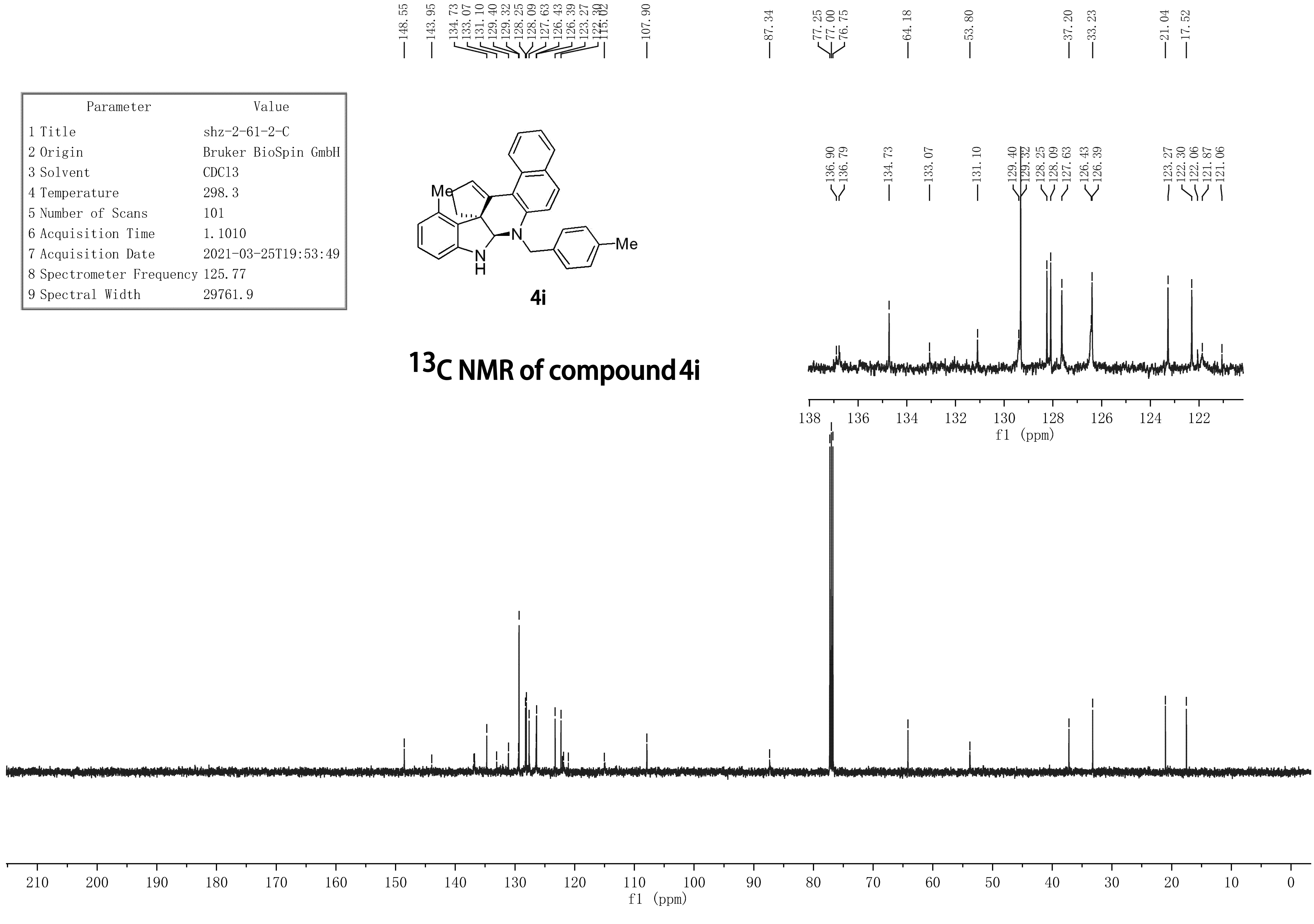

^{13}C NMR of compound 4i

Parameter	Value
1 Title	shz-2-99
2 Origin	Bruker BioSpin GmbH
3 Solvent	CDCl3
4 Temperature	298.7
5 Number of Scans	6
6 Acquisition Time	3.1719
7 Acquisition Date	2021-04-13T15:45:18
8 Spectrometer Frequency	500.17
9 Spectral Width	10330.6

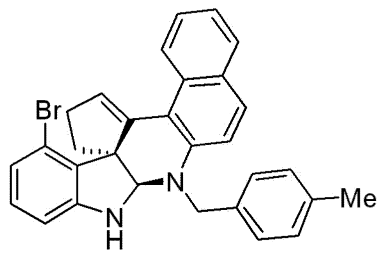

4j

^{1}H NMR of compound 4j

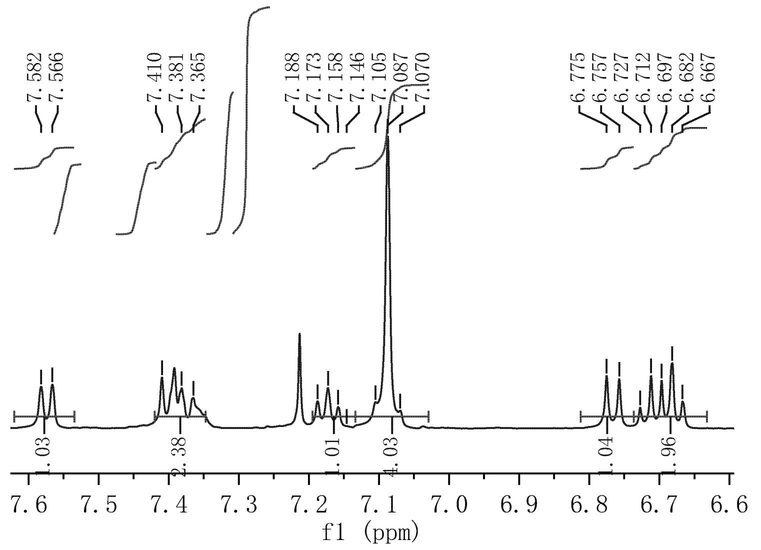

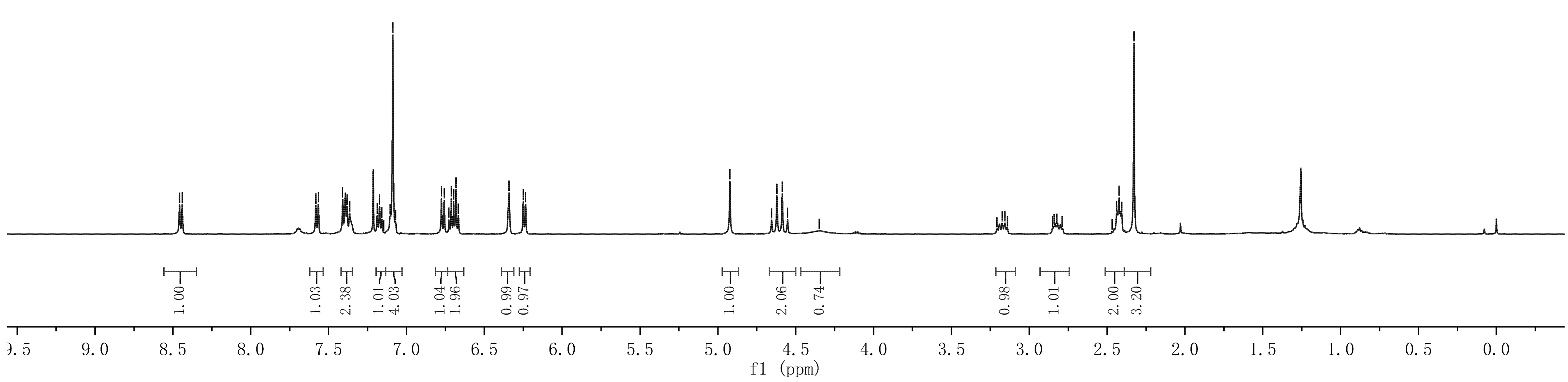

Parameter	Value
1 Title	shz-2-99-CC
2 Origin	Bruker BioSpin GmbH
3 Solvent	CDCl3
4 Temperature	299.2
5 Number of Scans	50
6 Acquisition Time	1.1010
7 Acquisition Date	2021-04-13T15:49:43
8 Spectrometer Frequency	125.77
9 Spectral Width	29761.9

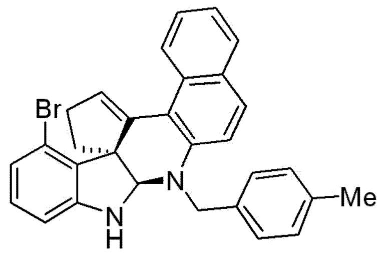

4j

^{13}C NMR of compound 4j

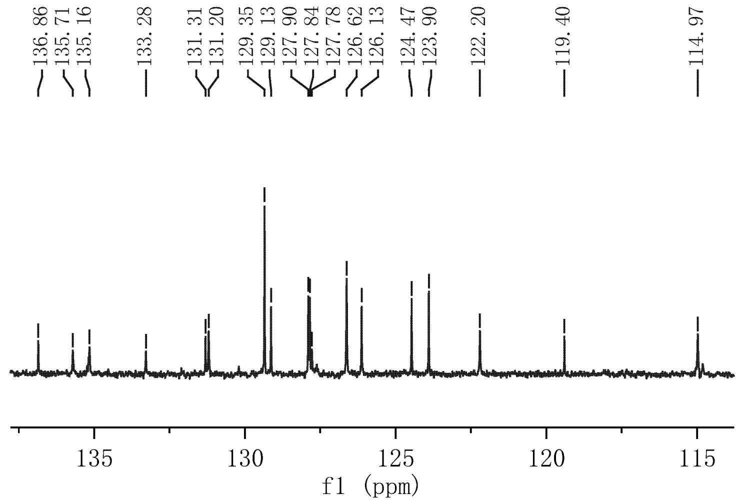

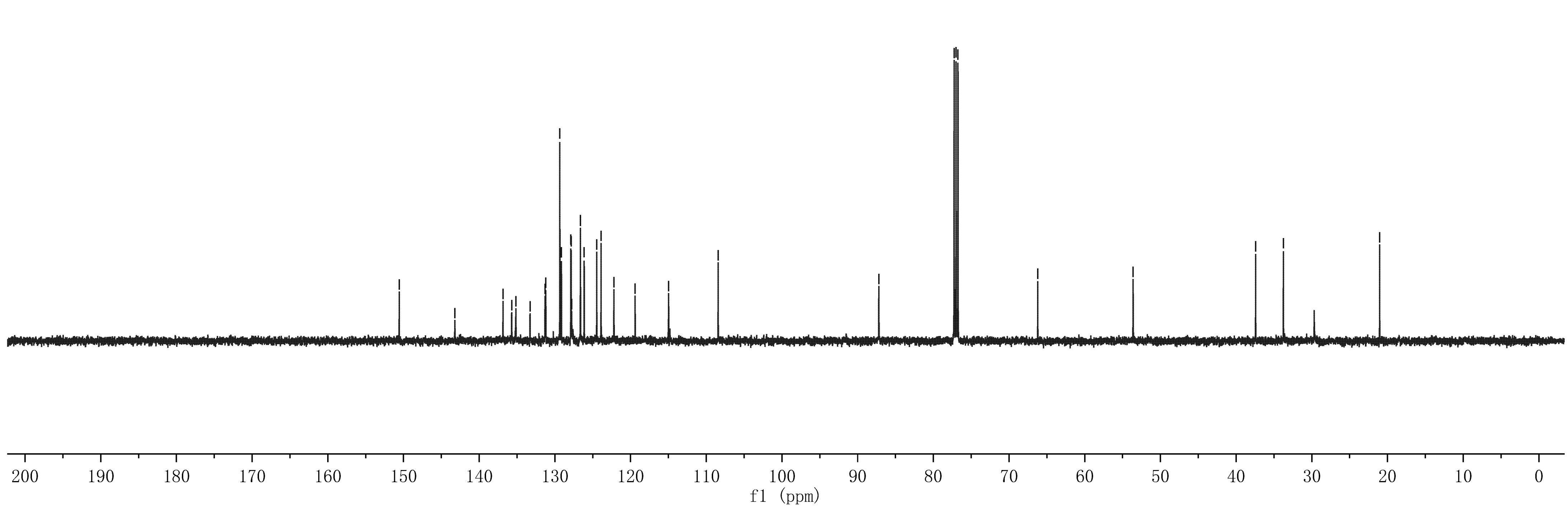

Parameter	Value
1 Title	shz-2-41
2 Origin	Bruker BioSpin GmbH
3 Solvent	CDCl3
4 Temperature	298.1
5 Number of Scans	6
6 Acquisition Time	3.1719
7 Acquisition Date	2021-03-16T16:02:30
8 Spectrometer Frequency	500.17
9 Spectral Width	10330.6

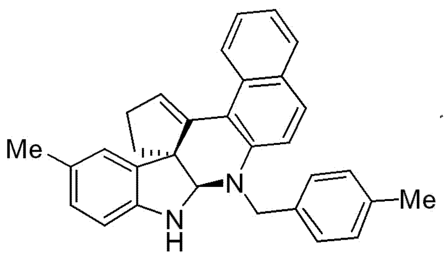

4k

^{1}H NMR of compound 4k

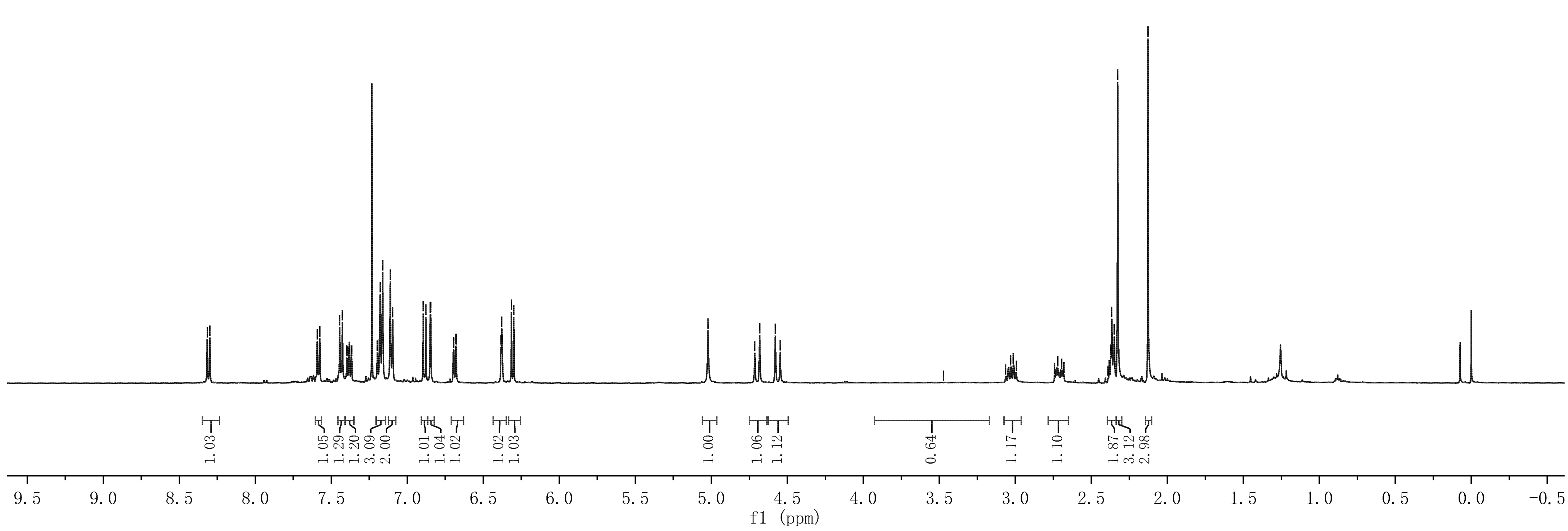

Parameter	Value
1 Title	shz-2-41-C
2 Origin	Bruker BioSpin GmbH
3 Solvent	CDCl3
4 Temperature	298.5
5 Number of Scans	105
6 Acquisition Time	1.1010
7 Acquisition Date	2021-03-16T16:05:37
8 Spectrometer Frequency	125.77
9 Spectral Width	29761.9

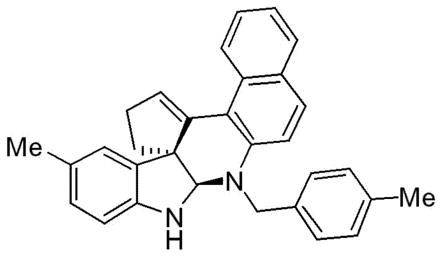

4k

^{13}C NMR of compound 4k

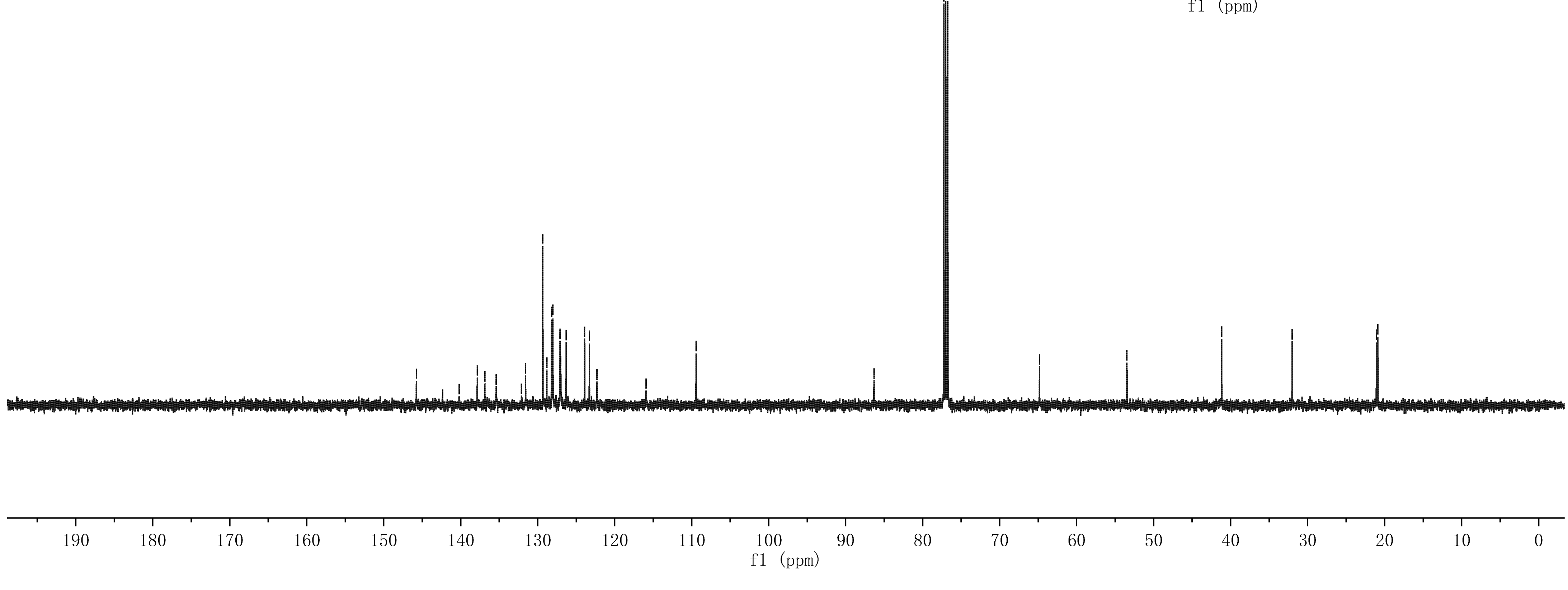

Parameter	Value
1 Title	shz-2-68
2 Origin	Bruker BioSpin GmbH
3 Solvent	CDCl3
4 Temperature	298.2
5 Number of Scans	6
6 Acquisition Time	3.1719
7 Acquisition Date	2021-03-26T14:08:20
8 Spectrometer Frequency	500.17
9 Spectral Width	10330.6

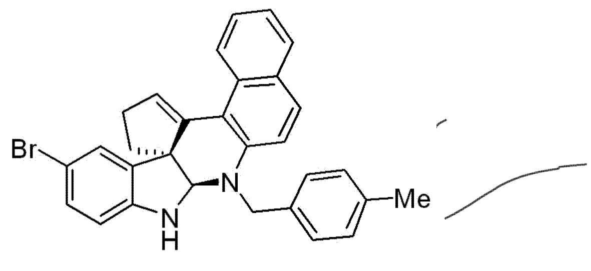

4l

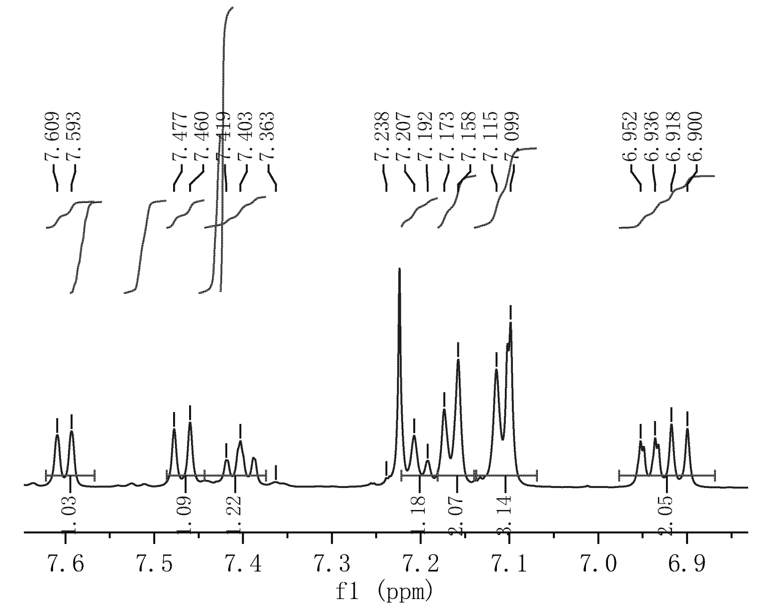

^{1}H NMR of compound 4l

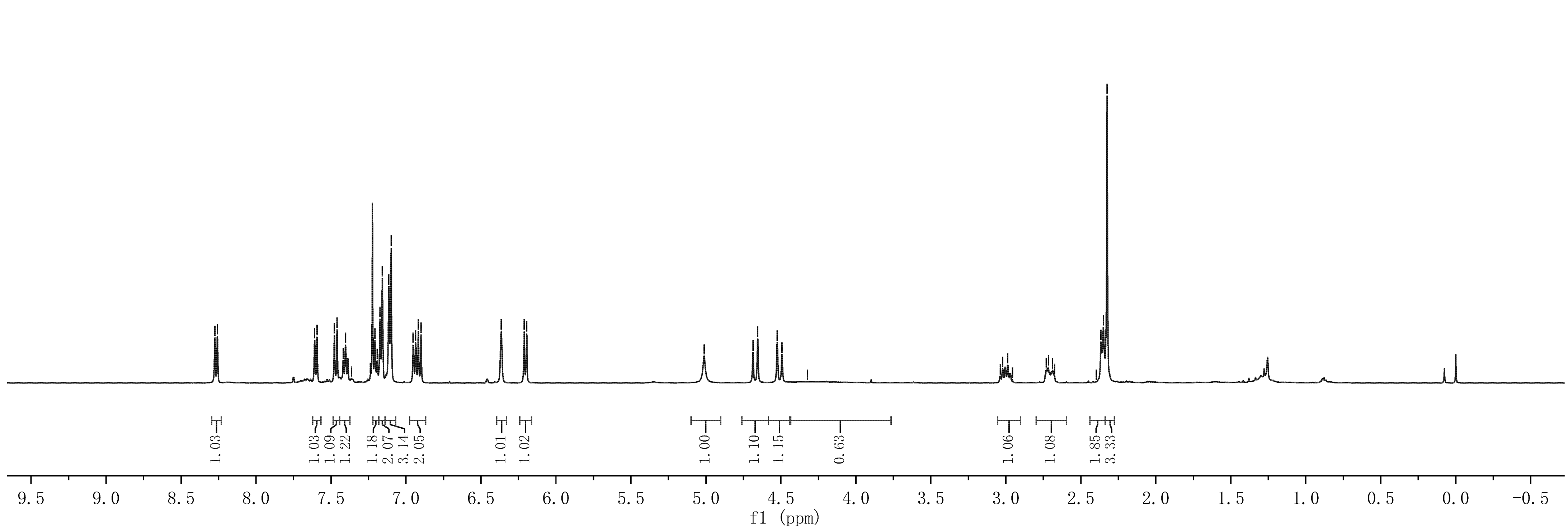

Parameter	Value
1 Title	shz-2-68-C
2 Origin	Bruker BioSpin GmbH
3 Solvent	CDCl3
4 Temperature	298.4
5 Number of Scans	78
6 Acquisition Time	1.1010
7 Acquisition Date	2021-03-26T14:09:21
8 Spectrometer Frequency	125.77
9 Spectral Width	29761.9

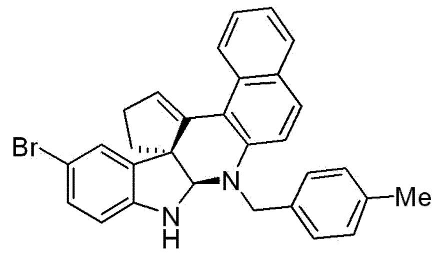

4l

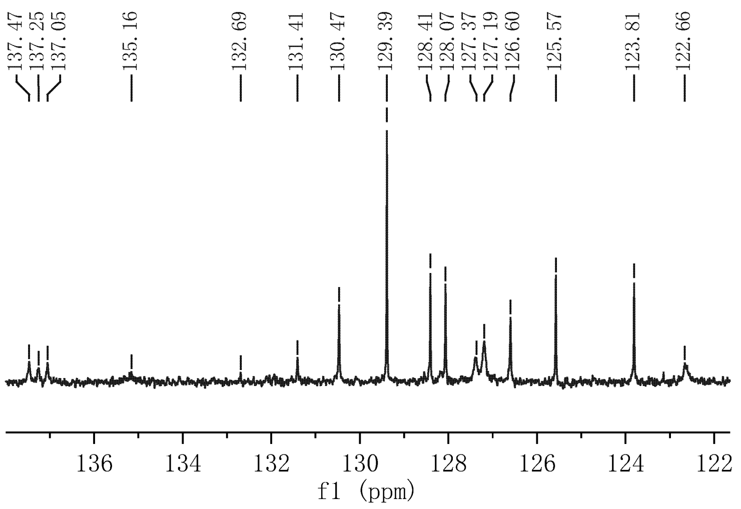

^{13}C NMR of compound 4l

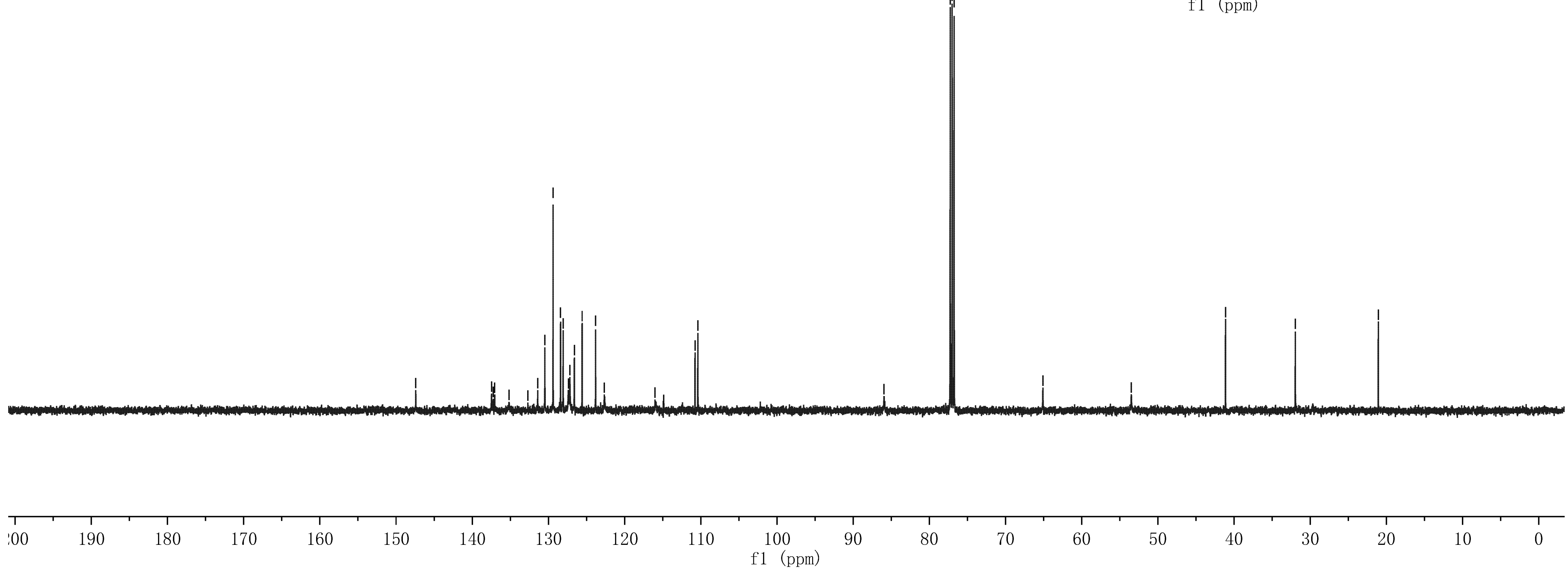

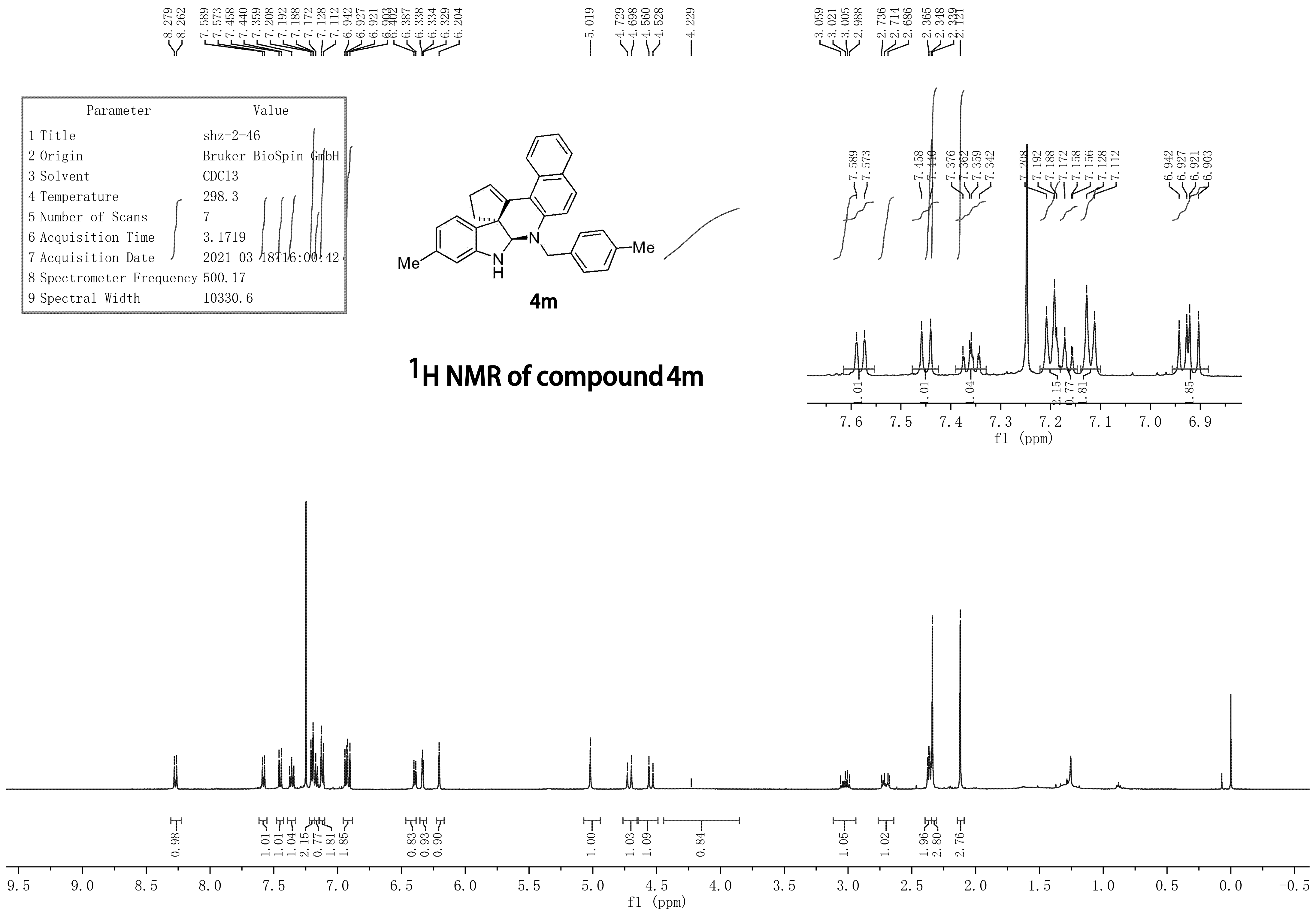
Parameter Value
1 Title shz-2-46
2 Origin Bruker BioSpin GmbH
3 Solvent CDCl3
4 Temperature 298.3
5 Number of Scans 7
6 Acquisition Time 3.1719
7 Acquisition Date 2021-03-18T16:00:42
8 Spectrometer Frequency 500.17
9 Spectral Width 10330.6
4m
1H NMR of compound 4m
f1 (ppm)

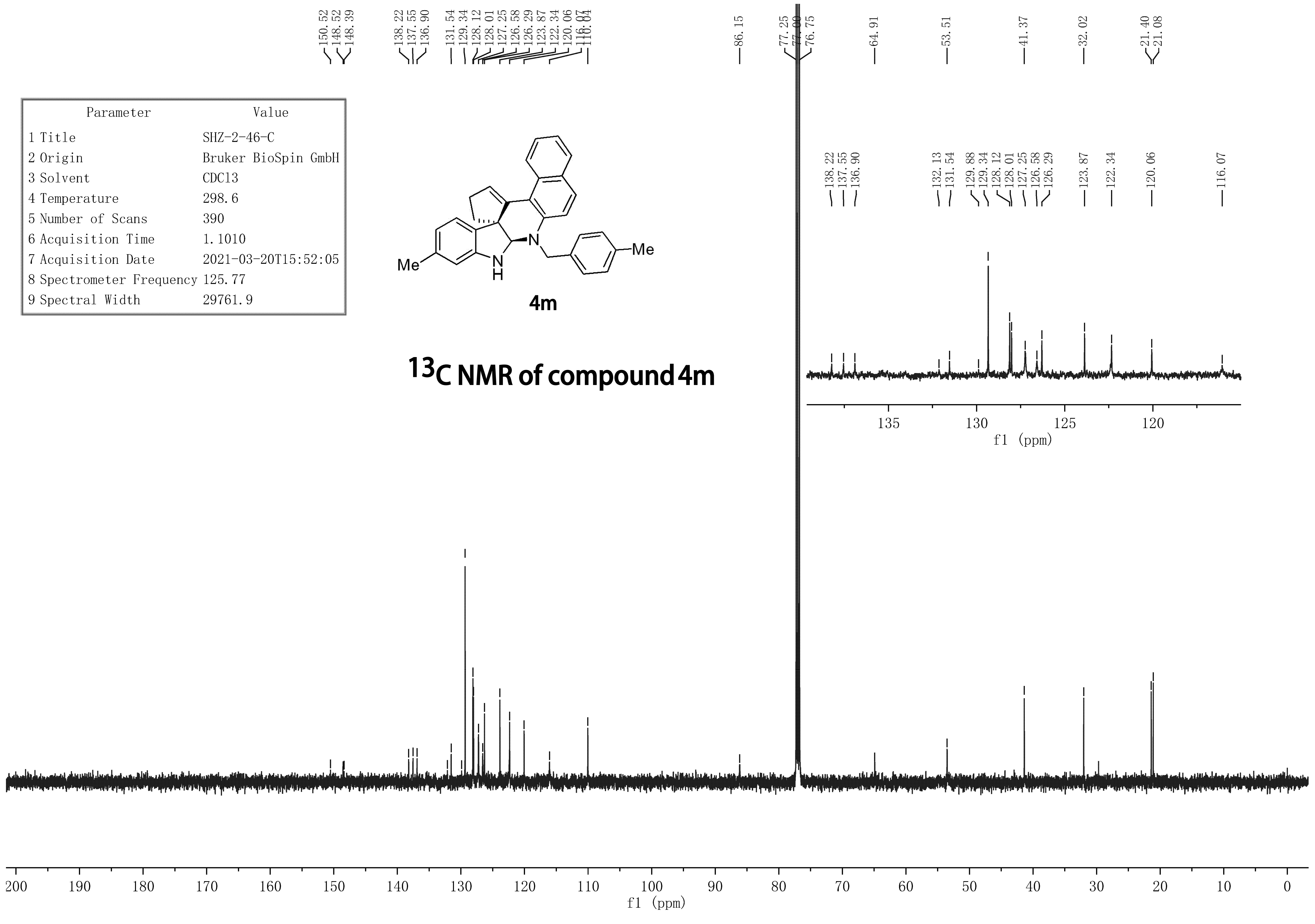
Parameter Value
1 Title SHZ-2-46-C
2 Origin Bruker BioSpin GmbH
3 Solvent CDCl3
4 Temperature 298.6
5 Number of Scans 390
6 Acquisition Time 1.1010
7 Acquisition Date 2021-03-20T15:52:05
8 Spectrometer Frequency 125.77
9 Spectral Width 29761.9
4m
13C NMR of compound 4m
f1 (ppm)

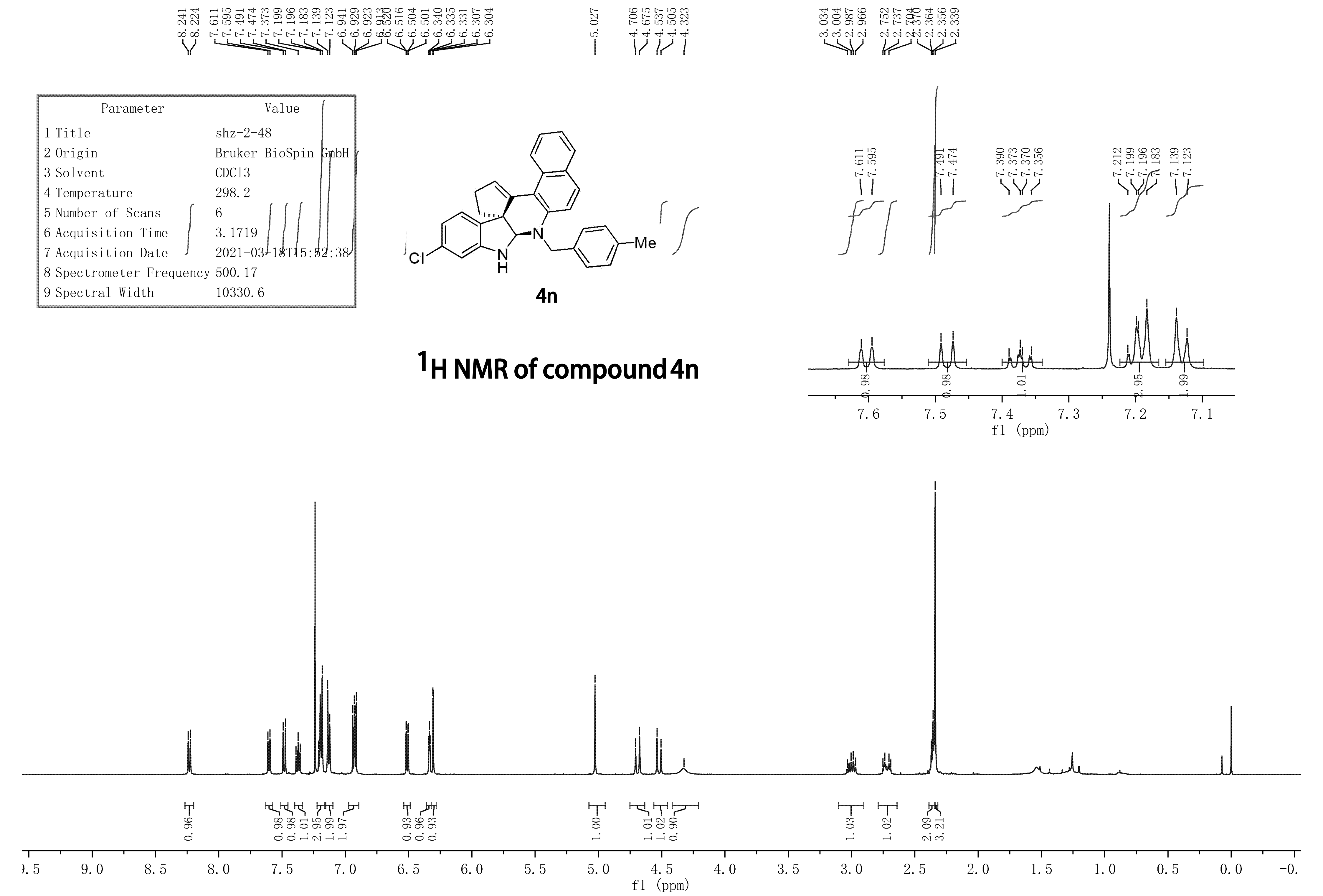

^{1}H NMR of compound 4n

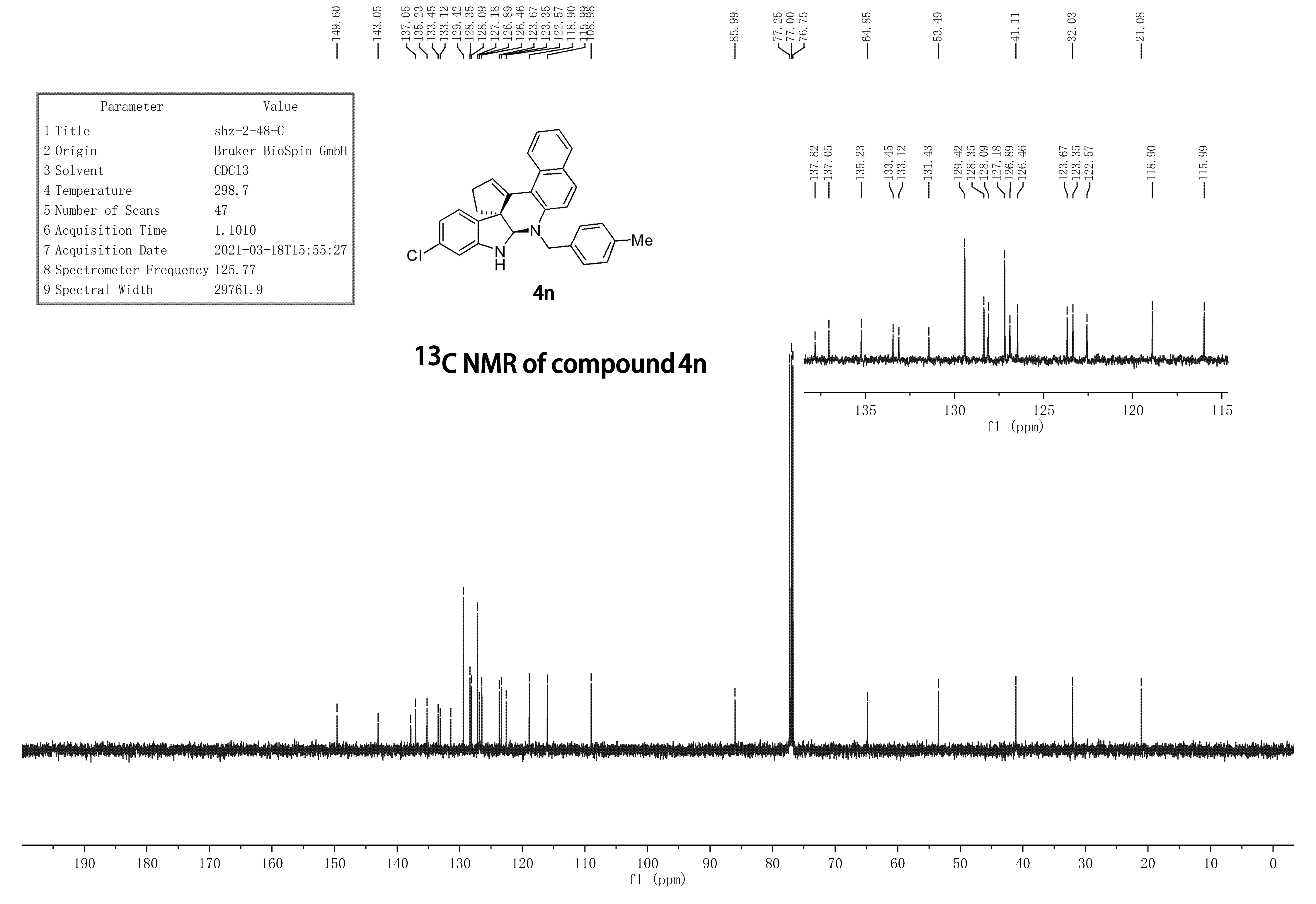

^{13}C NMR of compound 4n

Parameter	Value
1 Title	shz-2-100
2 Origin	Bruker BioSpin GmbH
3 Solvent	CDCl3
4 Temperature	299.0
5 Number of Scans	8
6 Acquisition Time	3.1719
7 Acquisition Date	2021-04-13T15:54:52
8 Spectrometer Frequency	500.17
9 Spectral Width	10330.6

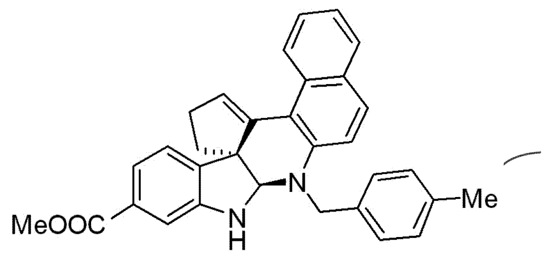

4o

^{1}H NMR of compound 4o

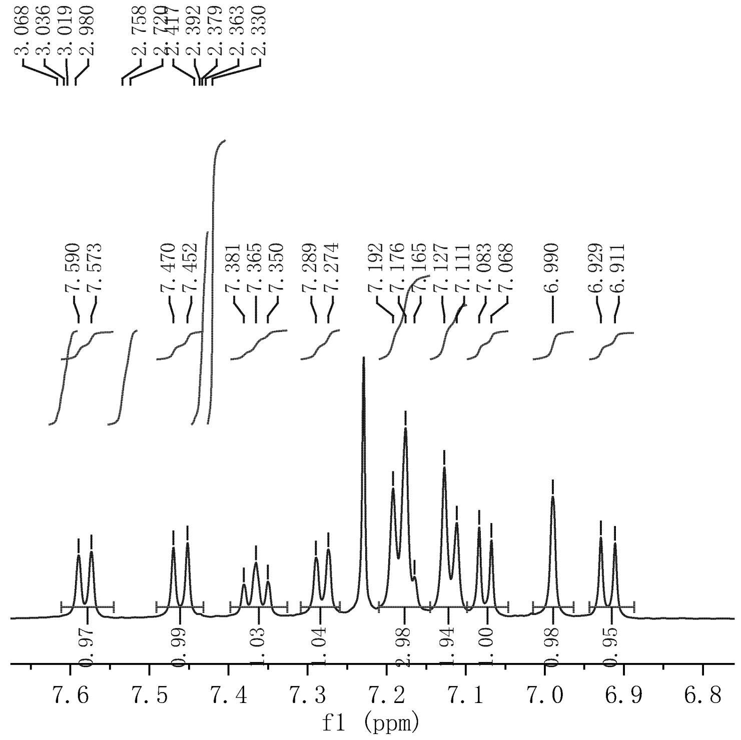

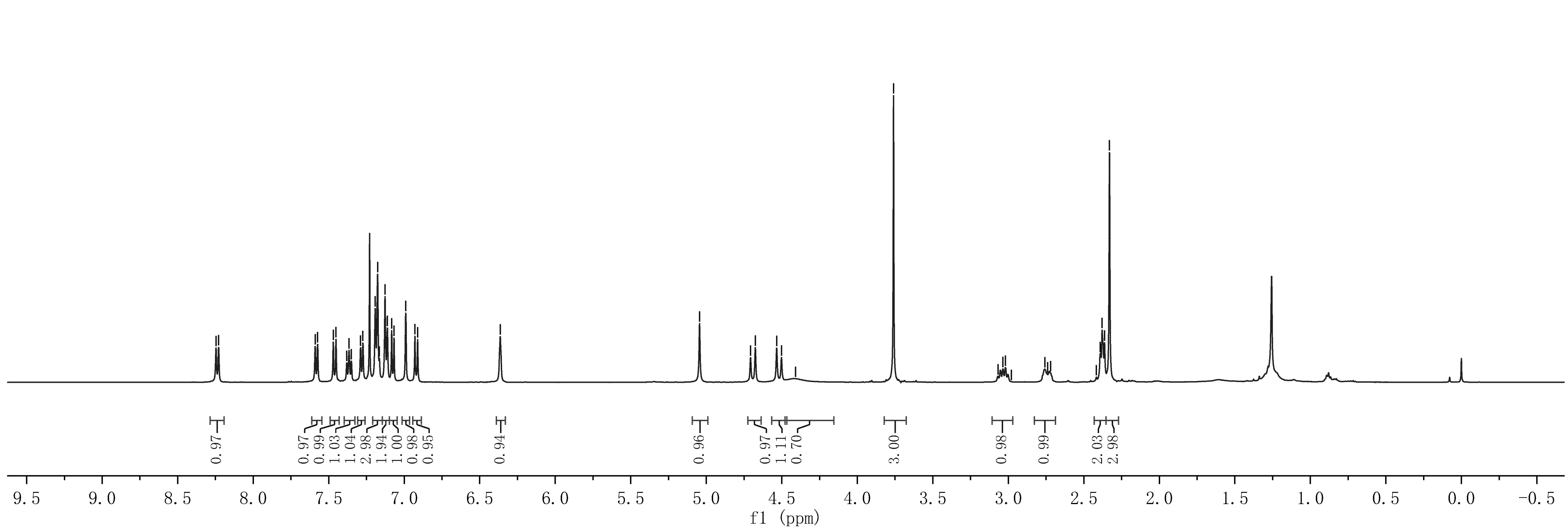

Parameter	Value
1 Title	shz-2-100-C
2 Origin	Bruker BioSpin GmbH
3 Solvent	CDCl3
4 Temperature	299.1
5 Number of Scans	33
6 Acquisition Time	1.1010
7 Acquisition Date	2021-04-13T15:56:16
8 Spectrometer Frequency	125.77
9 Spectral Width	29761.9

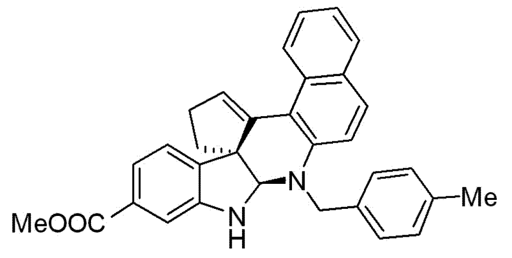

4o

^{13}C NMR of compound 4o

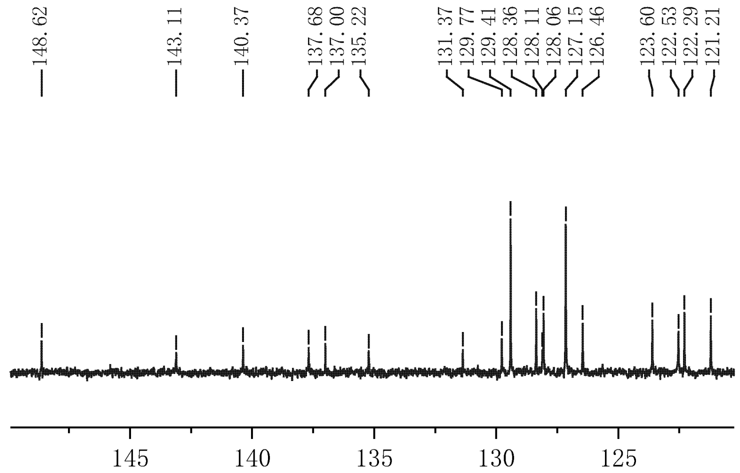

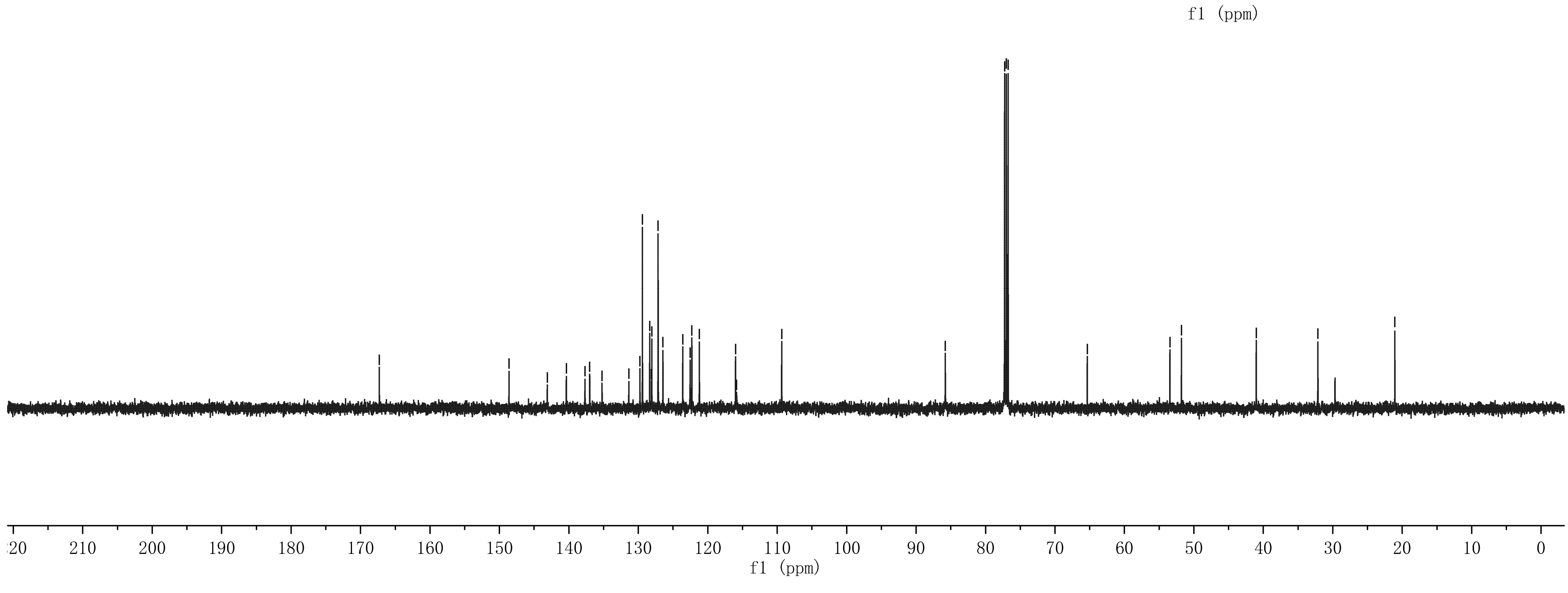

Parameter	Value
1 Title	shz-2-81
2 Origin	Bruker BioSpin GmbH
3 Solvent	CDCl3
4 Temperature	298.0
5 Number of Scans	9
6 Acquisition Time	3.1719
7 Acquisition Date	2021-04-05T15:37:55
8 Spectrometer Frequency	500.17
9 Spectral Width	10330.6

4p

^{1}H NMR of compound 4p

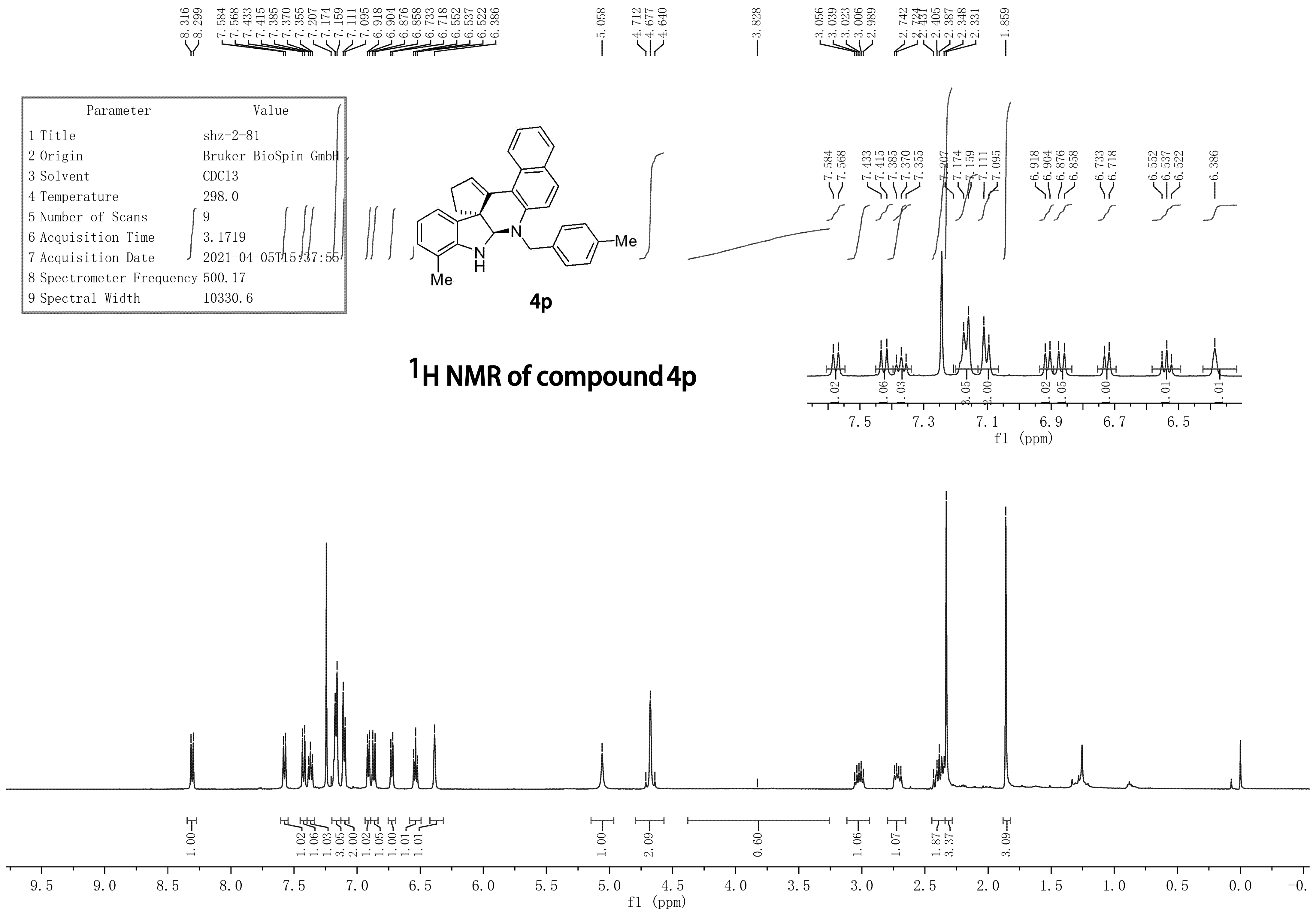

Parameter	Value
1 Title	SHZ-2-81-C
2 Origin	Bruker BioSpin GmbH
3 Solvent	CDCl3
4 Temperature	298.6
5 Number of Scans	396
6 Acquisition Time	1.1010
7 Acquisition Date	2021-04-05T15:41:06
8 Spectrometer Frequency	125.77
9 Spectral Width	29761.9

4p

^{13}C NMR of compound 4p

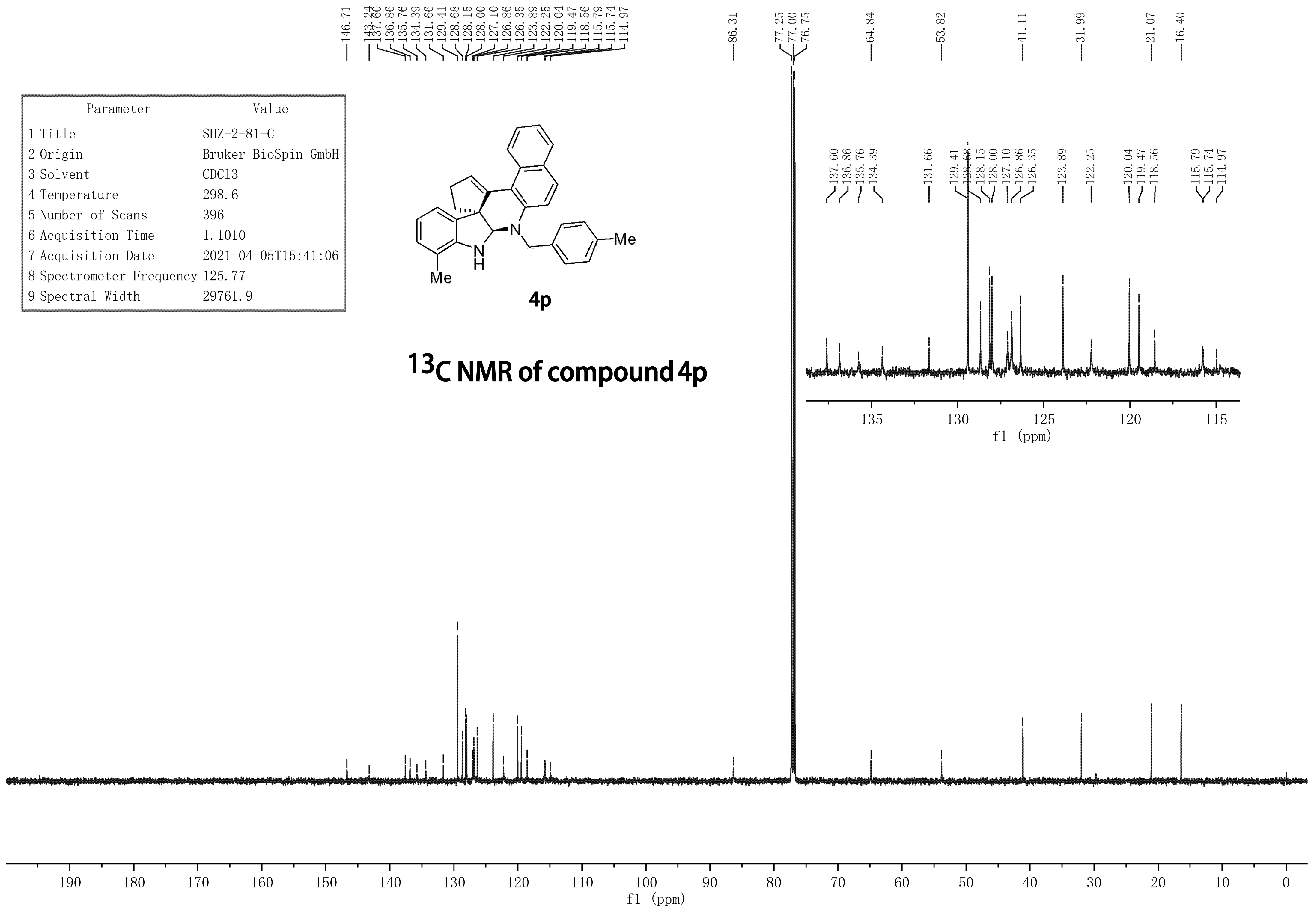

Parameter	Value
1 Title	shz-2-82-2
2 Origin	Bruker BioSpin GmbH
3 Solvent	CDCl3
4 Temperature	298.3
5 Number of Scans	8
6 Acquisition Time	3.1719
7 Acquisition Date	2021-04-05T16:07:07
8 Spectrometer Frequency	500.17
9 Spectral Width	10330.6

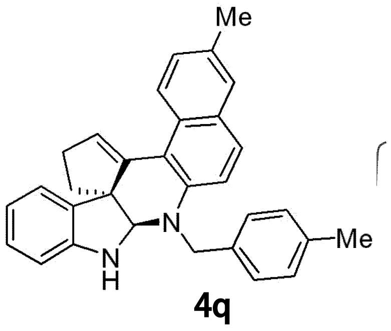

4q

^{1}H NMR of compound 4q

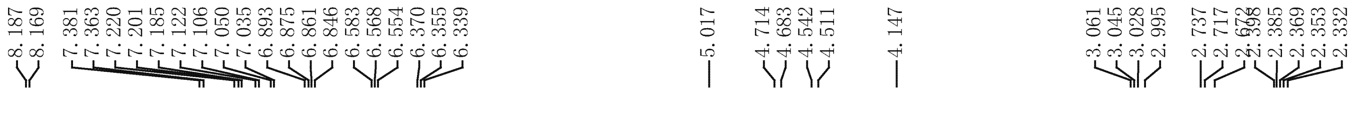

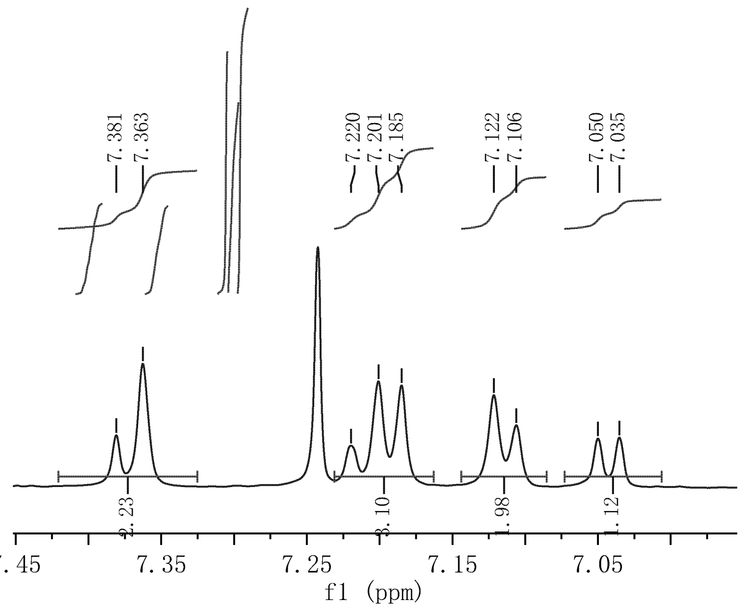

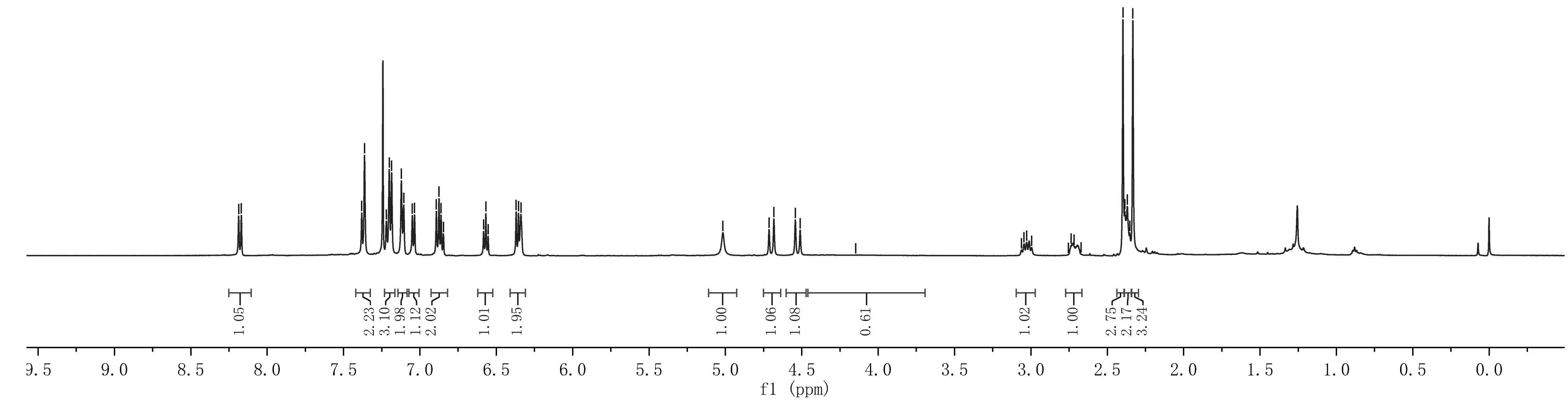

Parameter	Value
1 Title	shz-2-82-C
2 Origin	Bruker BioSpin GmbH
3 Solvent	CDCl3
4 Temperature	299.0
5 Number of Scans	93
6 Acquisition Time	1.1010
7 Acquisition Date	2021-04-02T19:50:20
8 Spectrometer Frequency	125.77
9 Spectral Width	29761.9

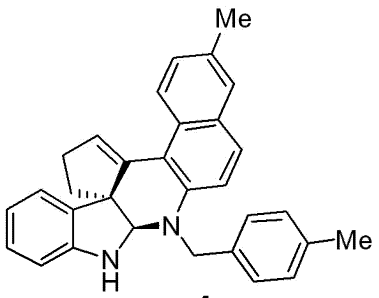

4q

^{13}C NMR of compound 4q

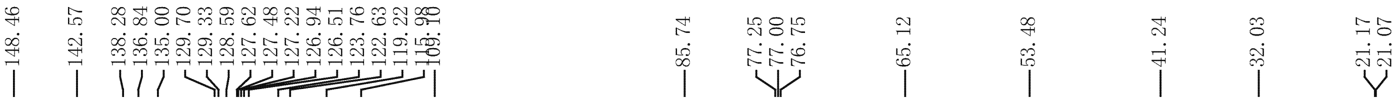

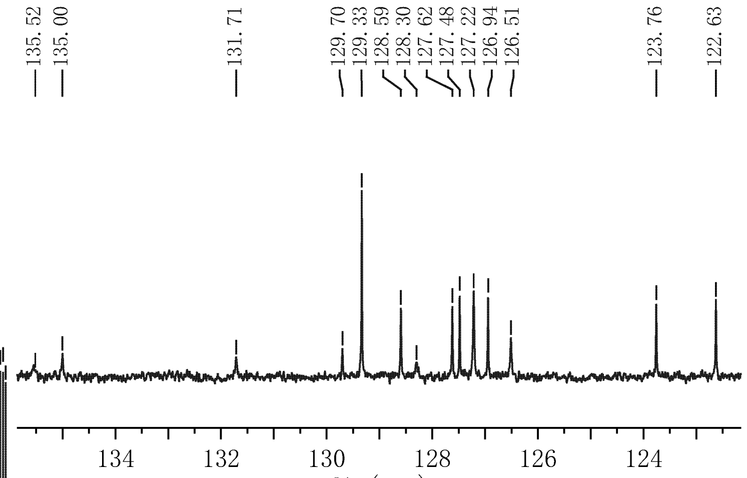

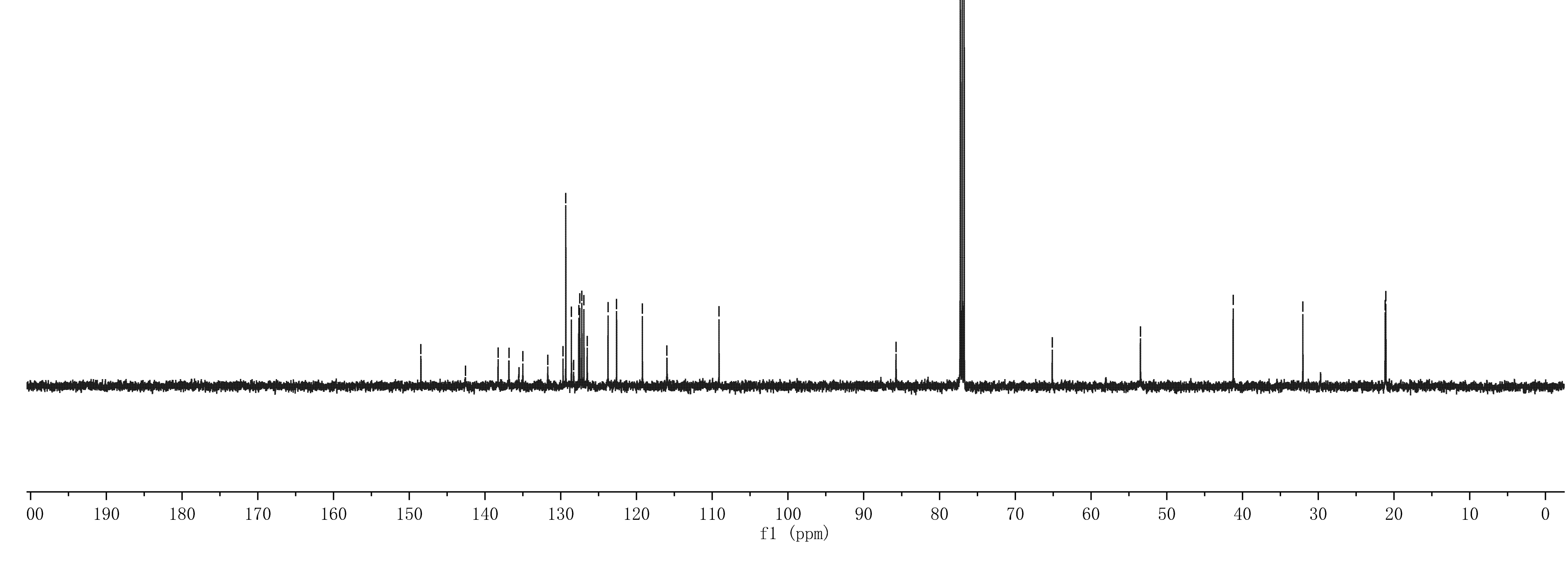

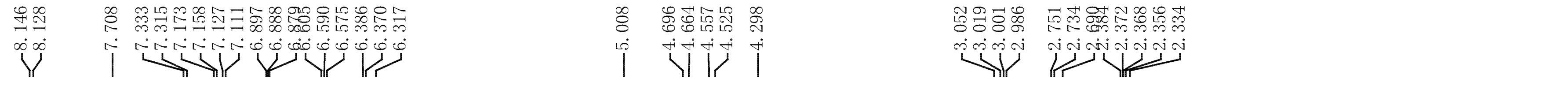

Parameter	Value
1 Title	shz-2-127
2 Origin	Bruker BioSpin GmbH
3 Solvent	CDCl3
4 Temperature	297.6
5 Number of Scans	11
6 Acquisition Time	3.1719
7 Acquisition Date	2021-04-29T15:50:15
8 Spectrometer Frequency	500.17
9 Spectral Width	10330.6

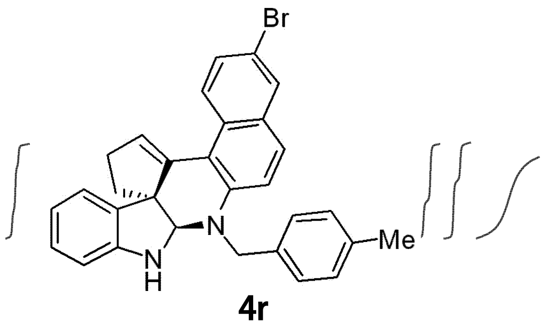

4r

^{1}H NMR of compound 4r

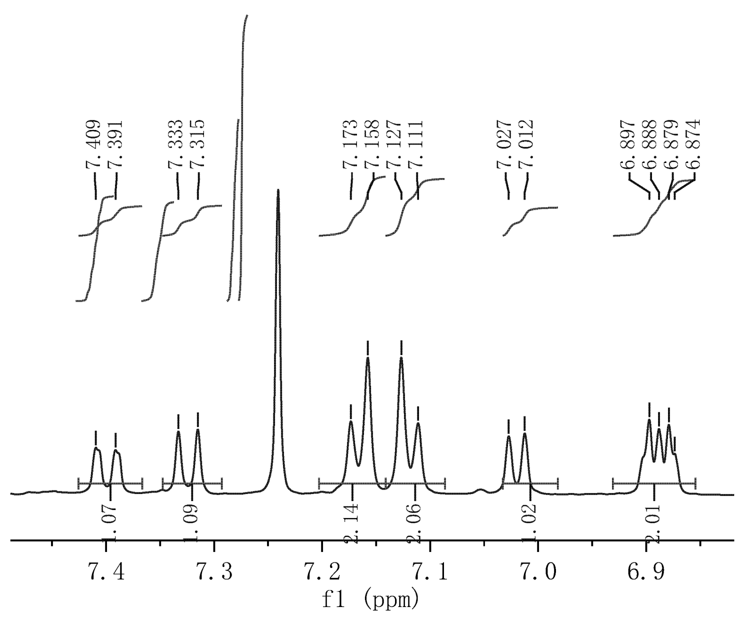

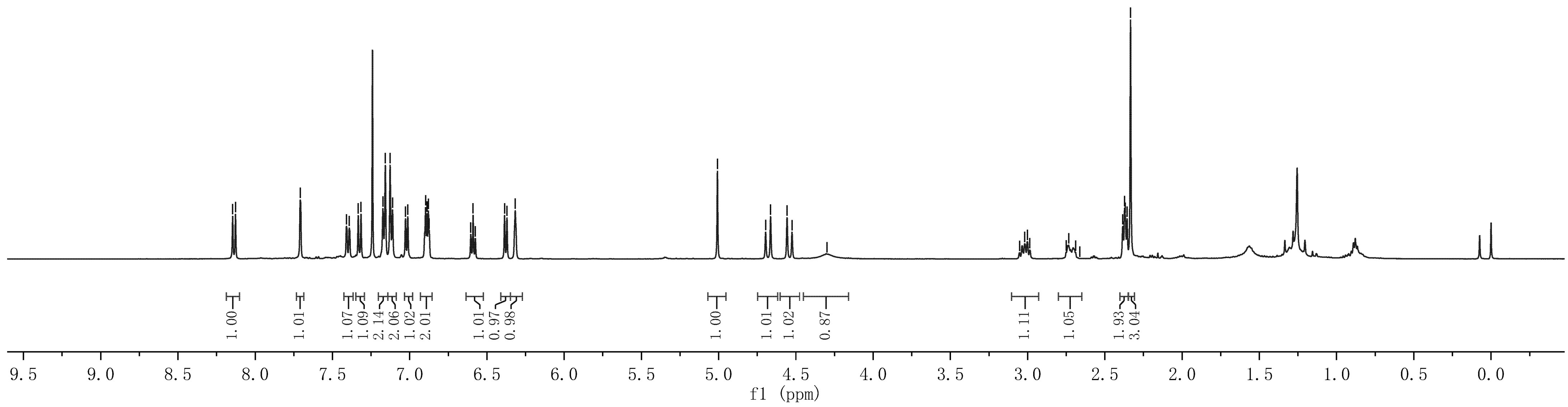

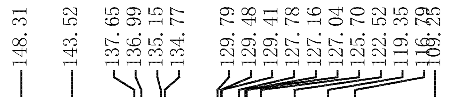

Parameter	Value
1 Title	shz-2-127-C
2 Origin	Bruker BioSpin GmbH
3 Solvent	CDCl3
4 Temperature	297.8
5 Number of Scans	46
6 Acquisition Time	1.1010
7 Acquisition Date	2021-04-29T15:52:52
8 Spectrometer Frequency	125.77
9 Spectral Width	29761.9

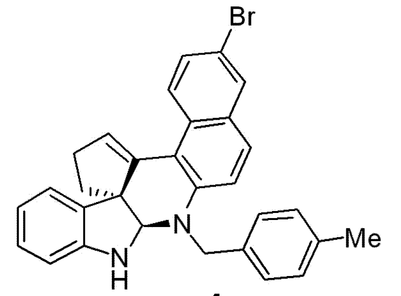

4r

^{13}C NMR of compound 4r

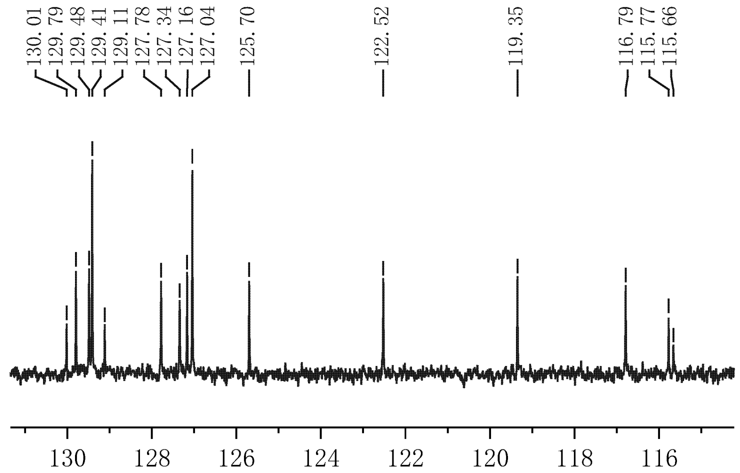

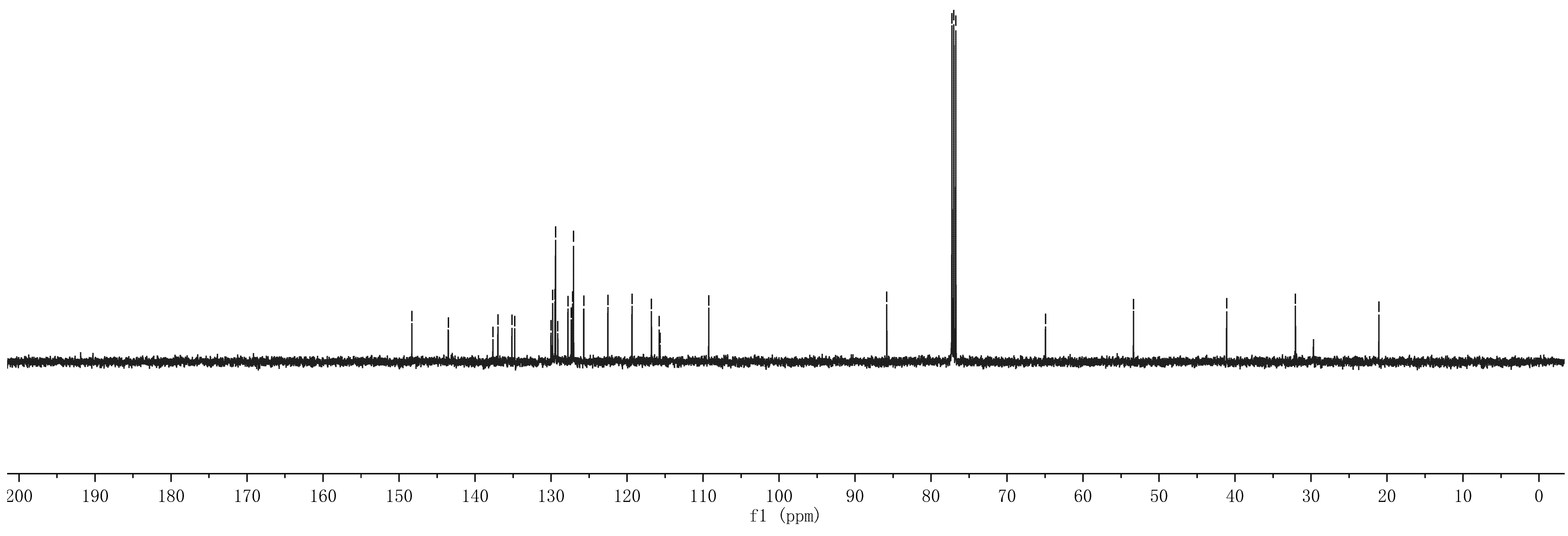

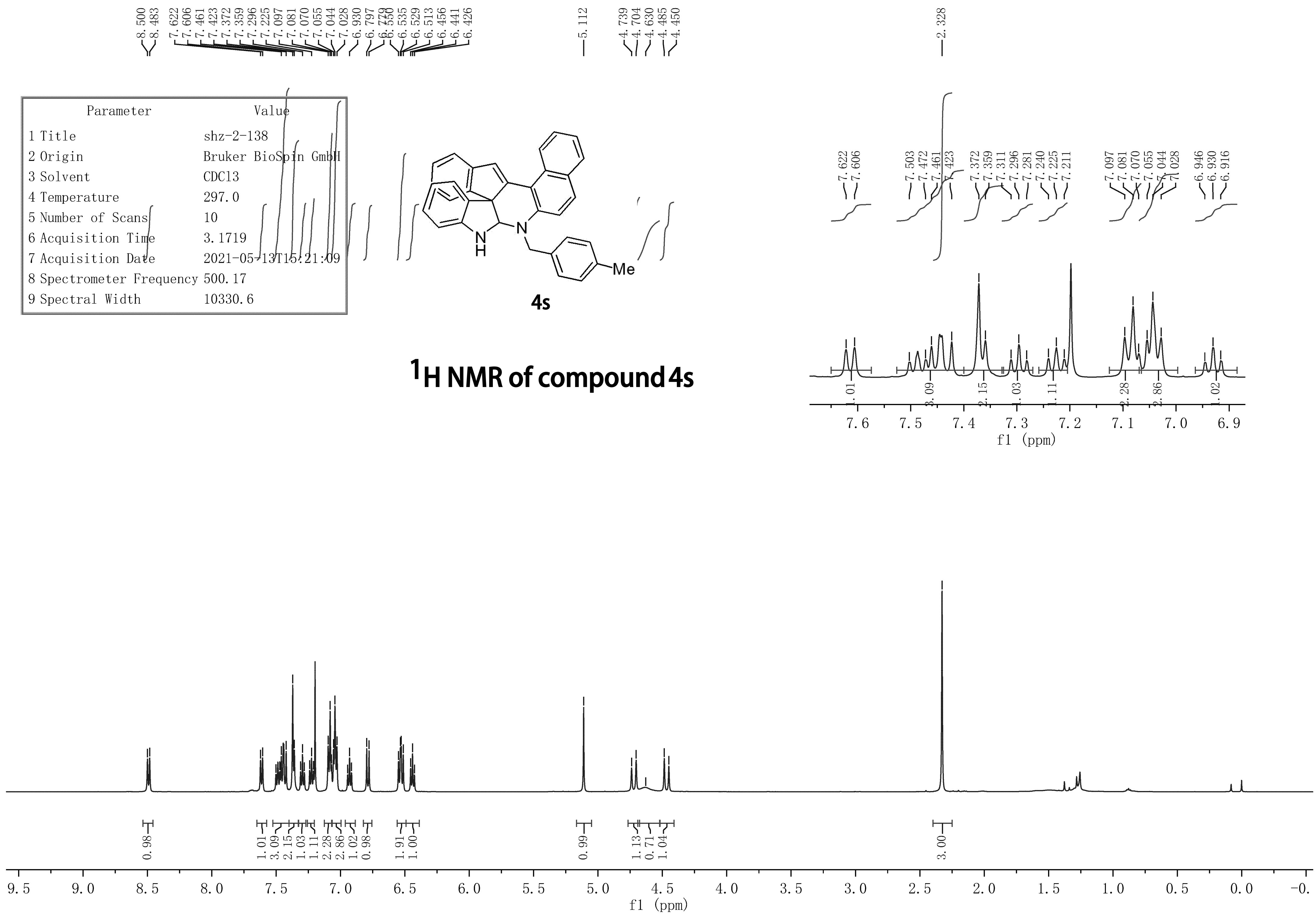

Parameter	Value
1 Title	shz-2-138
2 Origin	Bruker BioSpin GmbH
3 Solvent	CDCl3
4 Temperature	297.0
5 Number of Scans	10
6 Acquisition Time	3.1719
7 Acquisition Date	2021-05-13T15:21:09
8 Spectrometer Frequency	500.17
9 Spectral Width	10330.6

^{1}H NMR of compound 4s

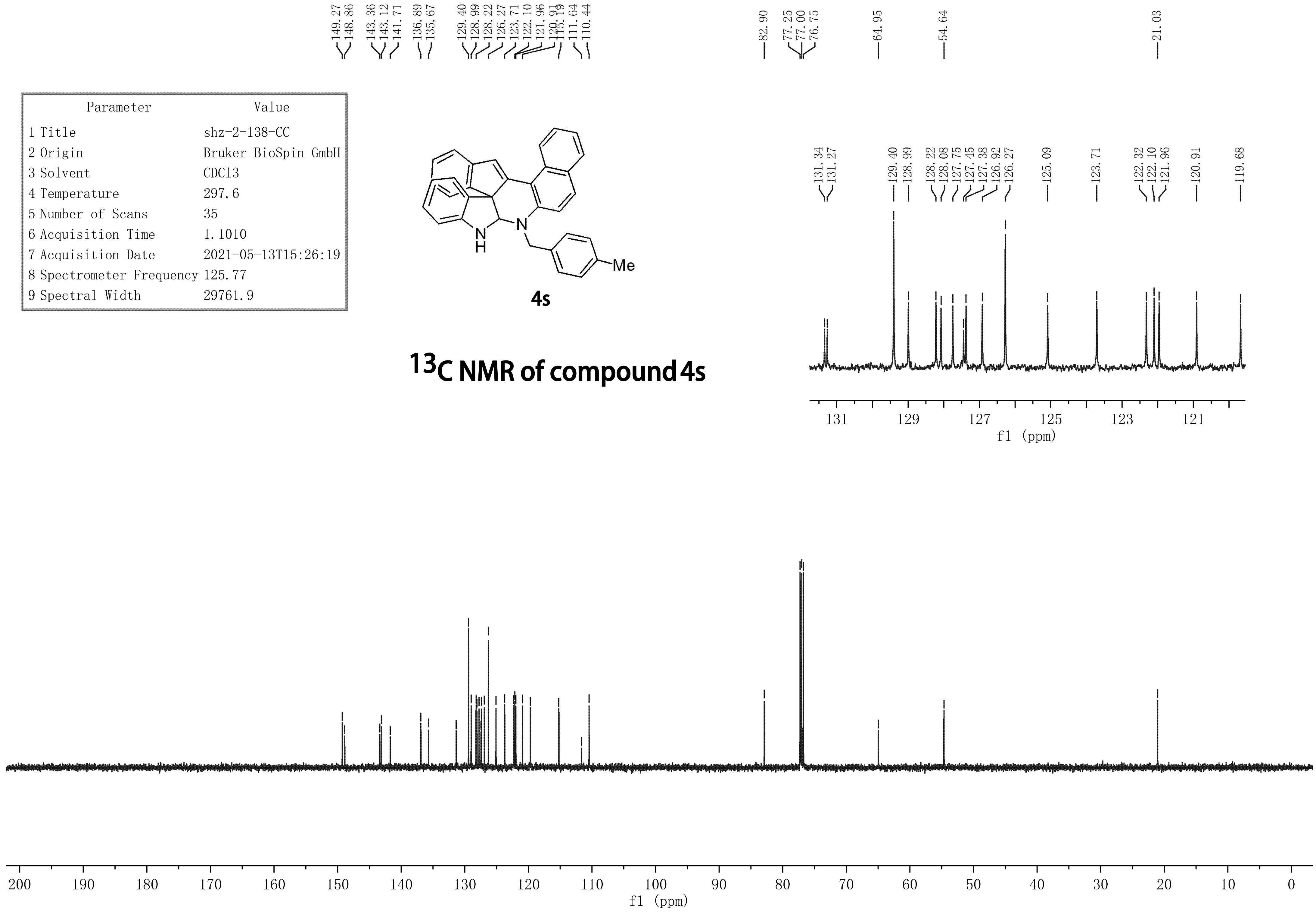

Parameter	Value
1 Title	shz-2-138-CC
2 Origin	Bruker BioSpin GmbH
3 Solvent	CDCl3
4 Temperature	297.6
5 Number of Scans	35
6 Acquisition Time	1.1010
7 Acquisition Date	2021-05-13T15:26:19
8 Spectrometer Frequency	125.77
9 Spectral Width	29761.9

^{13}C NMR of compound 4s

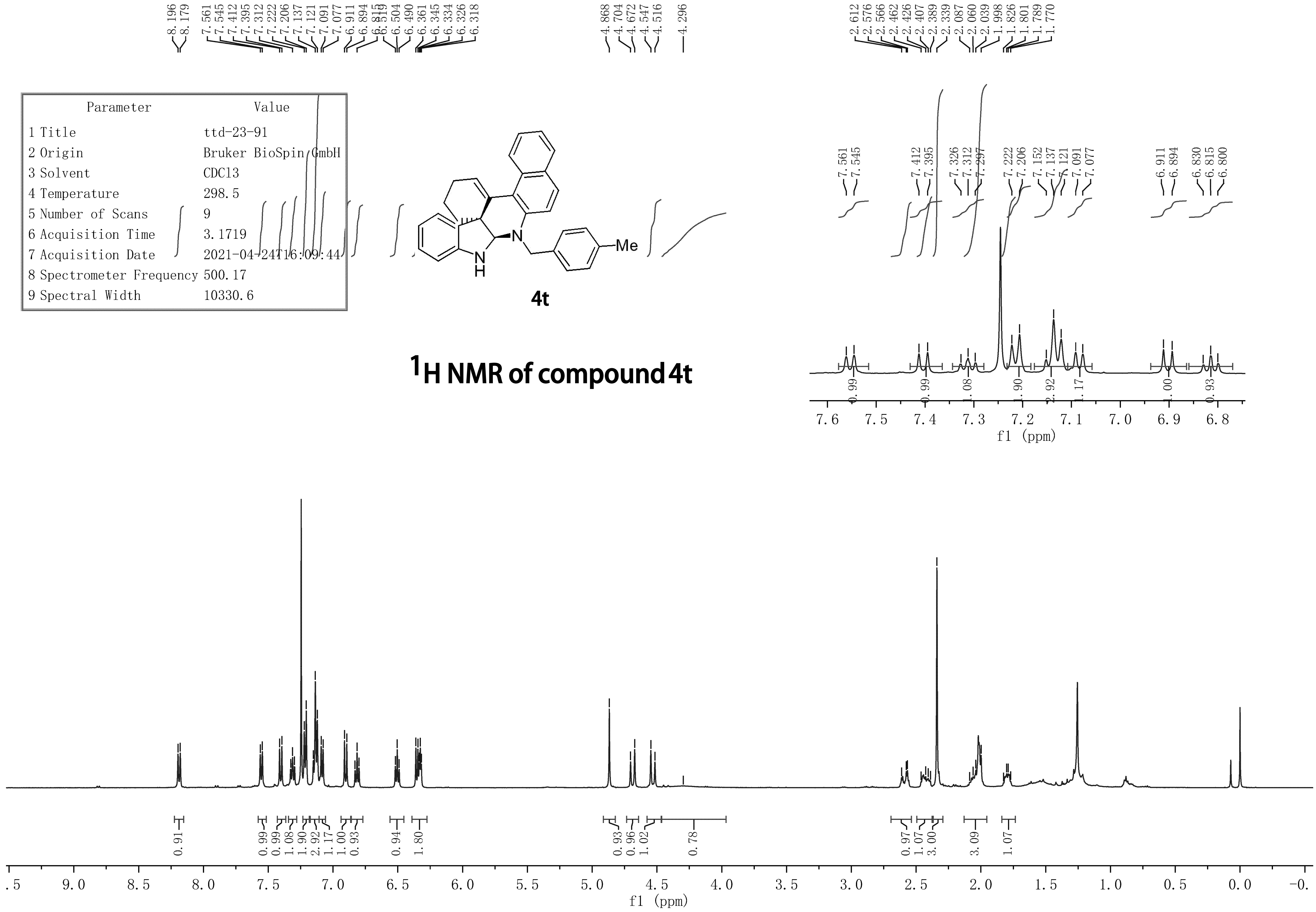

¹H NMR of compound 4t

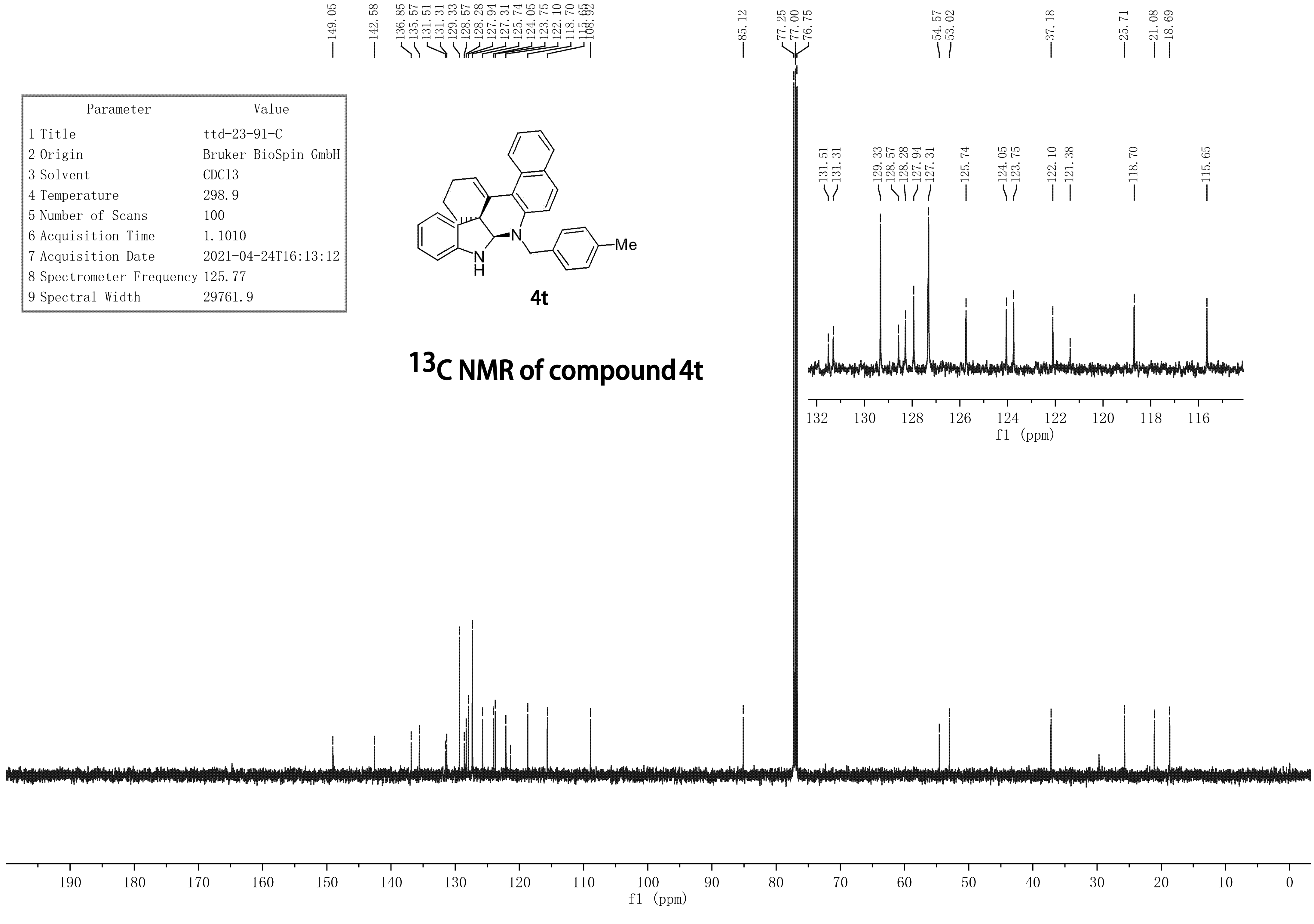

¹³C NMR of compound 4t

Parameter	Value
1 Title	ttd-23-91-C135
2 Origin	Bruker BioSpin GmbH
3 Solvent	CDCl3
4 Temperature	298.9
5 Number of Scans	26
6 Acquisition Time	1.1010
7 Acquisition Date	2021-04-24T16:18:41
8 Spectrometer Frequency	125.77
9 Spectral Width	29761.9

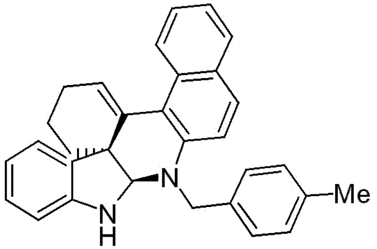

4t

^{13}C dept135 NMR of compound 4t

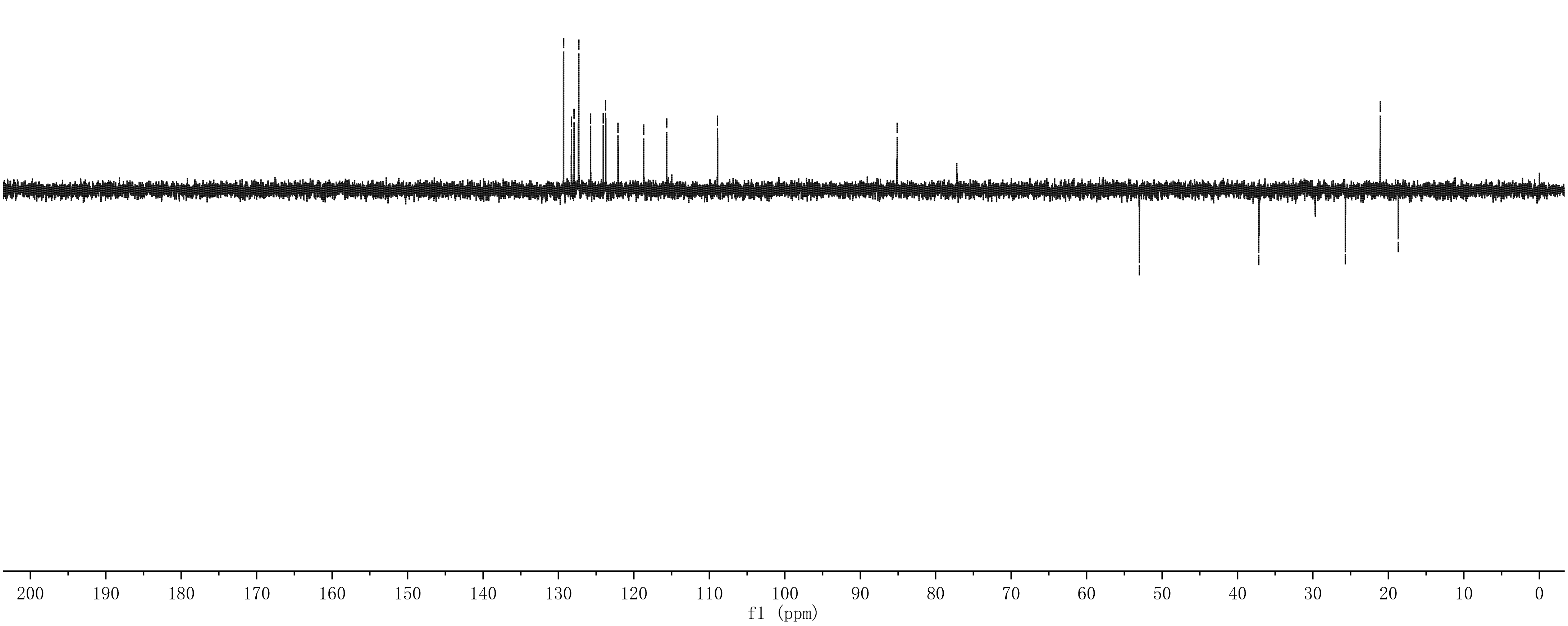

Parameter	Value
1 Title	ttd-22-123-600M
2 Origin	Bruker BioSpin GmbH
3 Solvent	CDCl3
4 Temperature	298.0
5 Number of Scans	7
6 Acquisition Time	2.7263
7 Acquisition Date	2020-12-26T16:37:11
8 Spectrometer Frequency	600.13
9 Spectral Width	12019.2

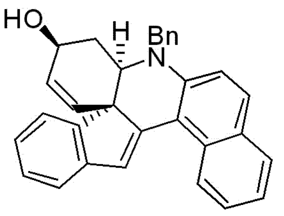

2aa

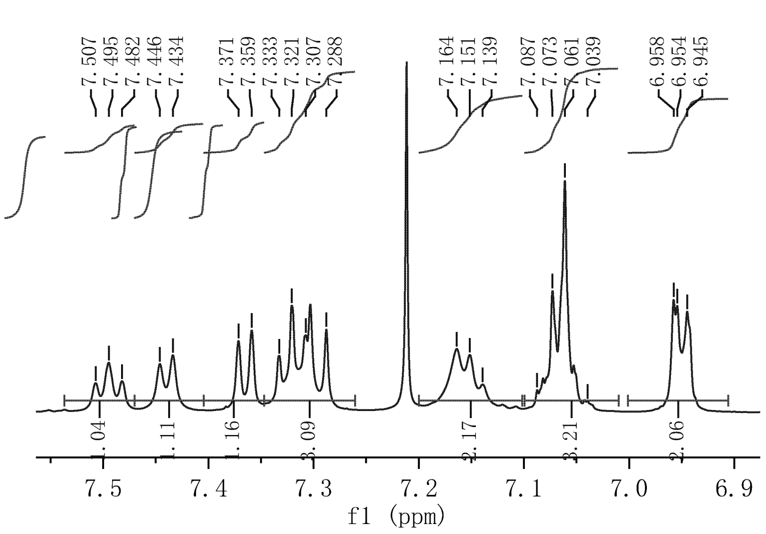

1H NMR of compound 2aa

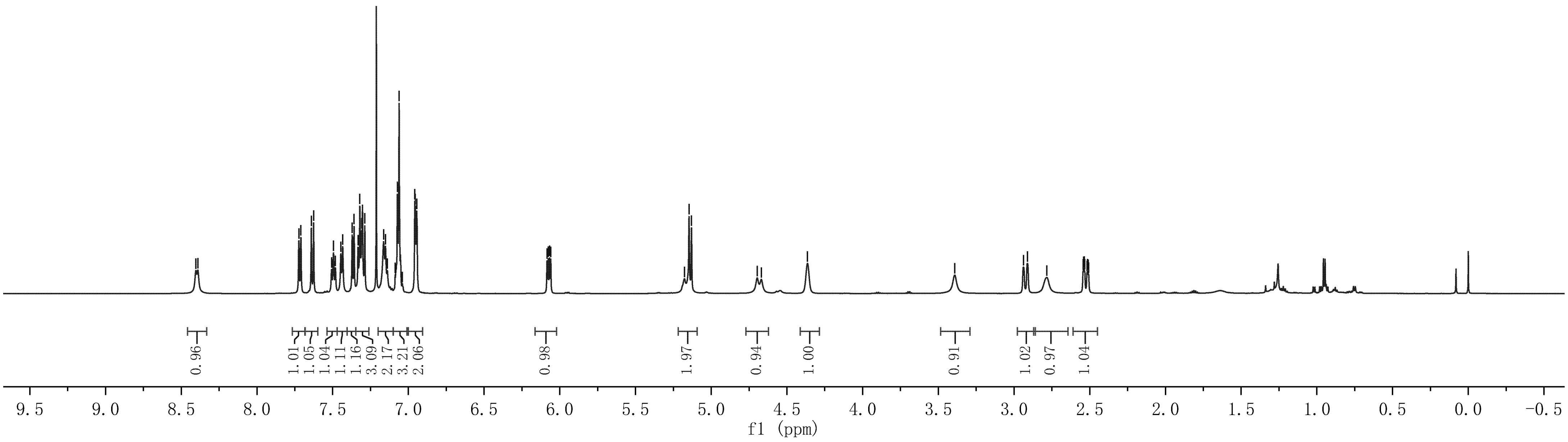

Parameter	Value
1 Title	ttd-22-123-600M
2 Origin	Bruker BioSpin GmbH
3 Solvent	CDCl3
4 Temperature	298.0
5 Number of Scans	14
6 Acquisition Time	0.9088
7 Acquisition Date	2020-12-27T11:36:02
8 Spectrometer Frequency	150.90
9 Spectral Width	36057.7

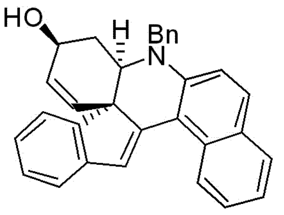

2aa

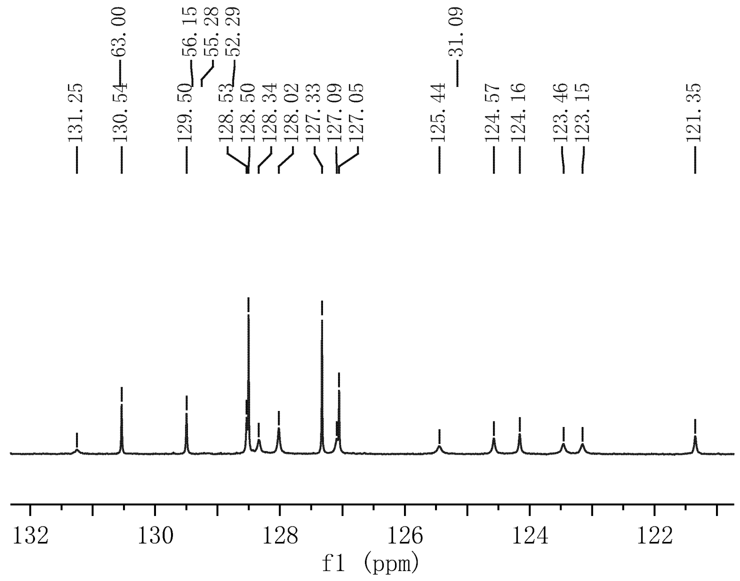

13C NMR of compound 2aa

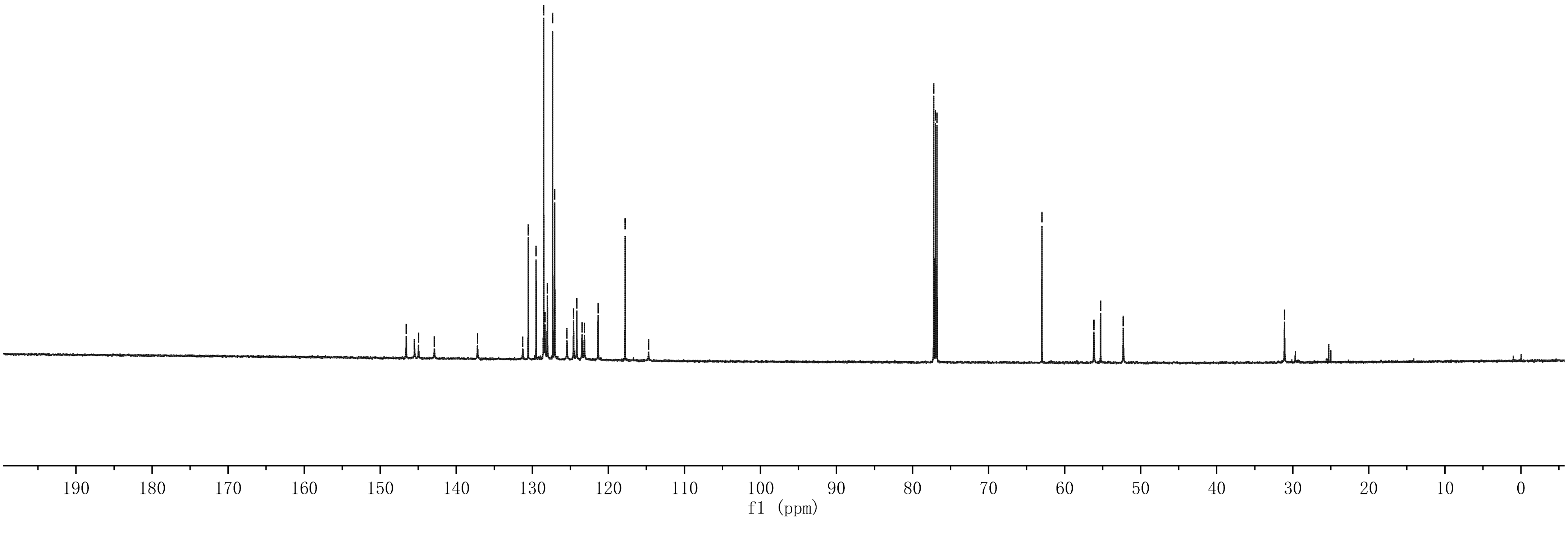

Parameter	Value
1 Title	ttd-22-123-C135
2 Origin	Bruker BioSpin GmbH
3 Solvent	CDCl3
4 Temperature	298.9
5 Number of Scans	31
6 Acquisition Time	1.1010
7 Acquisition Date	2020-12-26T20:33:32
8 Spectrometer Frequency	125.77
9 Spectral Width	29761.9

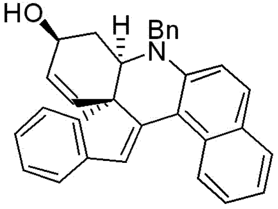

2aa

^{13}C dept135 NMR of compound 2aa

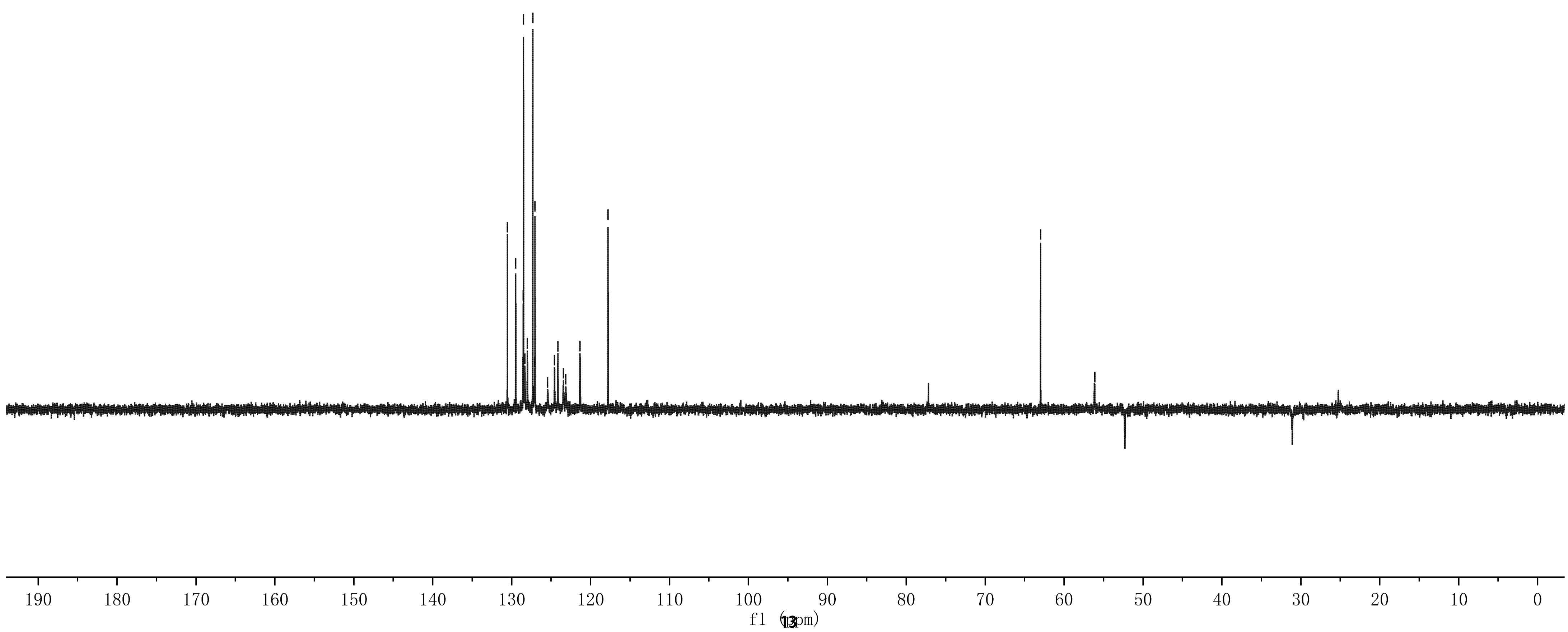

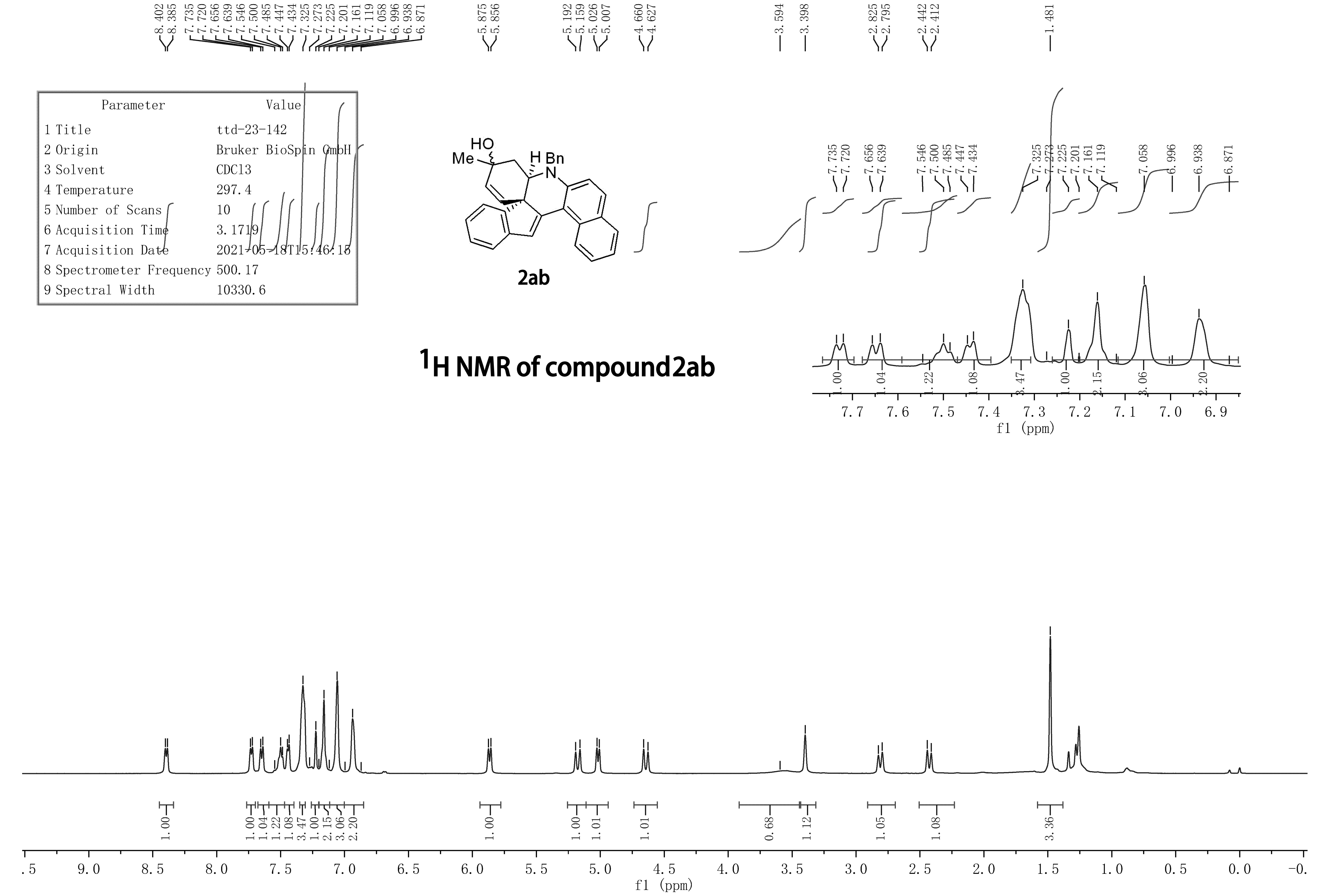

^{1}H NMR of compound2ab

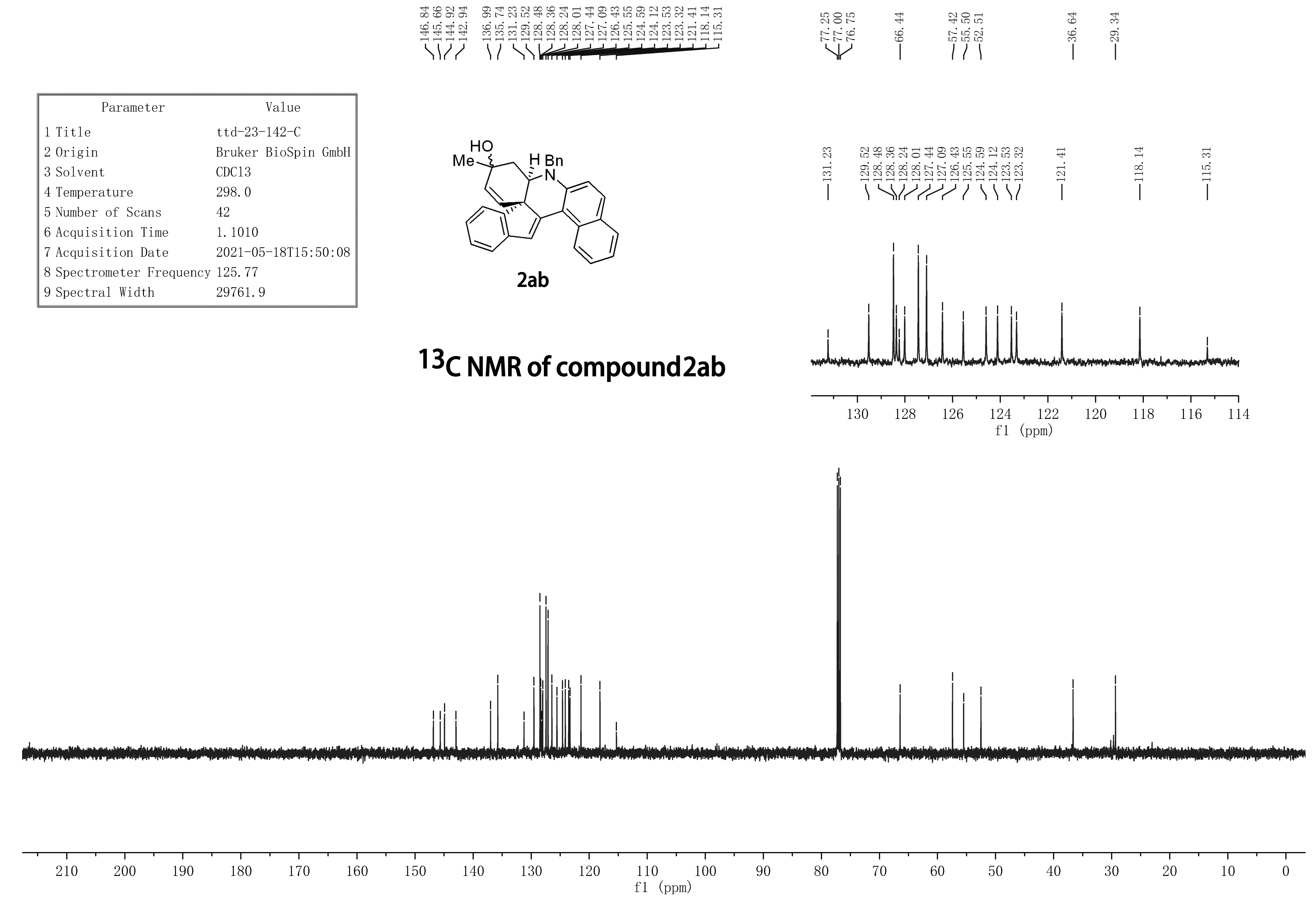

^{13}C NMR of compound2ab

Parameter	Value
1 Title	ttd-23-142-C135
2 Origin	Bruker BioSpin GmbH
3 Solvent	CDCl3
4 Temperature	297.8
5 Number of Scans	17
6 Acquisition Time	1.1010
7 Acquisition Date	2021-05-18T15:51:47
8 Spectrometer Frequency	125.77
9 Spectral Width	29761.9

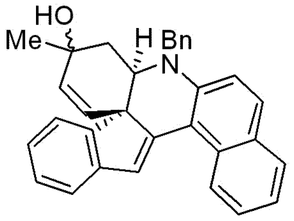

2ab

^{13}C dept135 NMR of compound 2ab

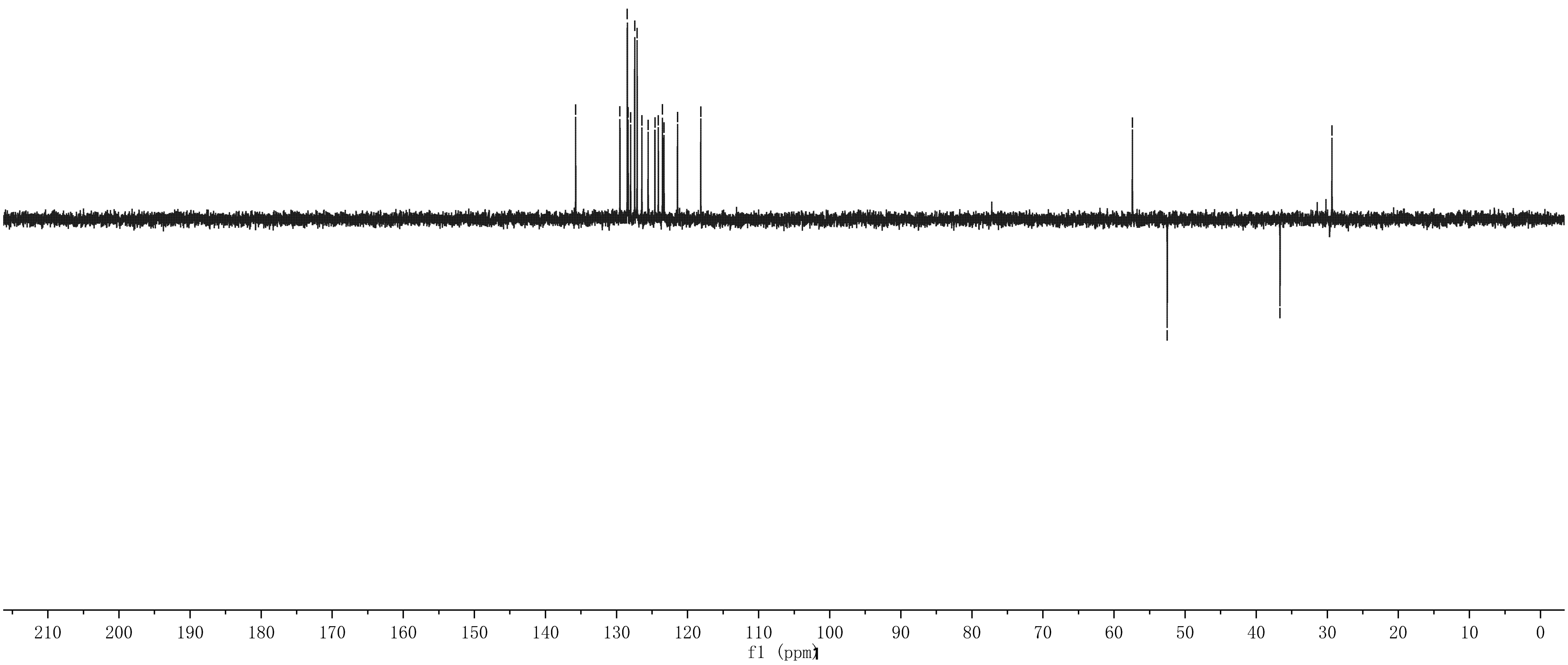

Parameter	Value
1 Title	ttd-22-119-600M
2 Origin	Bruker BioSpin GmbH
3 Solvent	CDCl3
4 Temperature	298.0
5 Number of Scans	8
6 Acquisition Time	2.7263
7 Acquisition Date	2020-12-26T16:21:08
8 Spectrometer Frequency	600.13
9 Spectral Width	12019.2

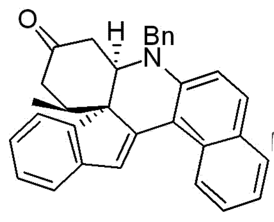

2ac

^{1}H NMR of compound 2ac

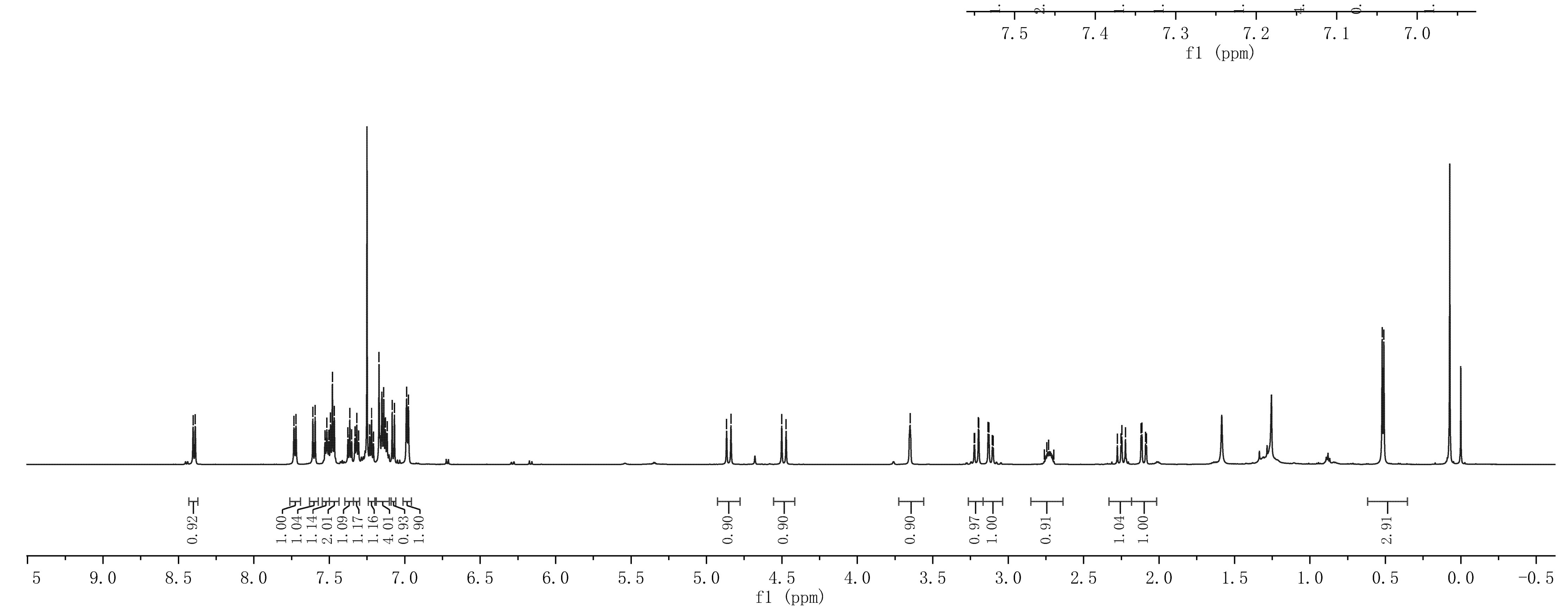

Parameter	Value
1 Title	ttd-22-119-C
2 Origin	Bruker BioSpin GmbH
3 Solvent	CDCl3
4 Temperature	298.9
5 Number of Scans	76
6 Acquisition Time	1.1010
7 Acquisition Date	2020-12-26T20:39:19
8 Spectrometer Frequency	125.77
9 Spectral Width	29761.9

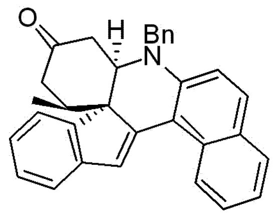

2ac

^{13}C NMR of compound 2ac

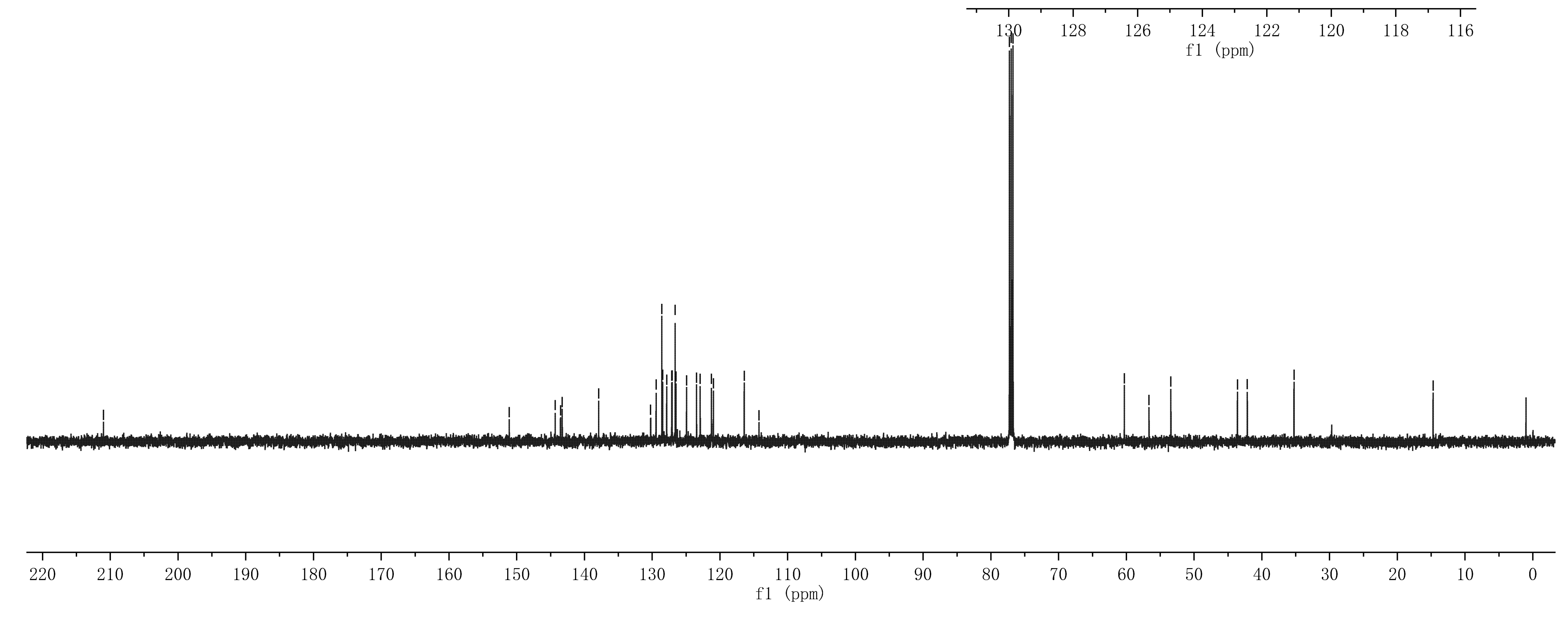

Parameter	Value
1 Title	ttd-22-119-C135
2 Origin	Bruker BioSpin GmbH
3 Solvent	CDCl3
4 Temperature	299.0
5 Number of Scans	33
6 Acquisition Time	1.1010
7 Acquisition Date	2020-12-25T16:12:47
8 Spectrometer Frequency	125.77
9 Spectral Width	29761.9

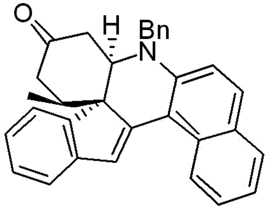

2ac

^{13}C dept135 NMR of compound 2ac

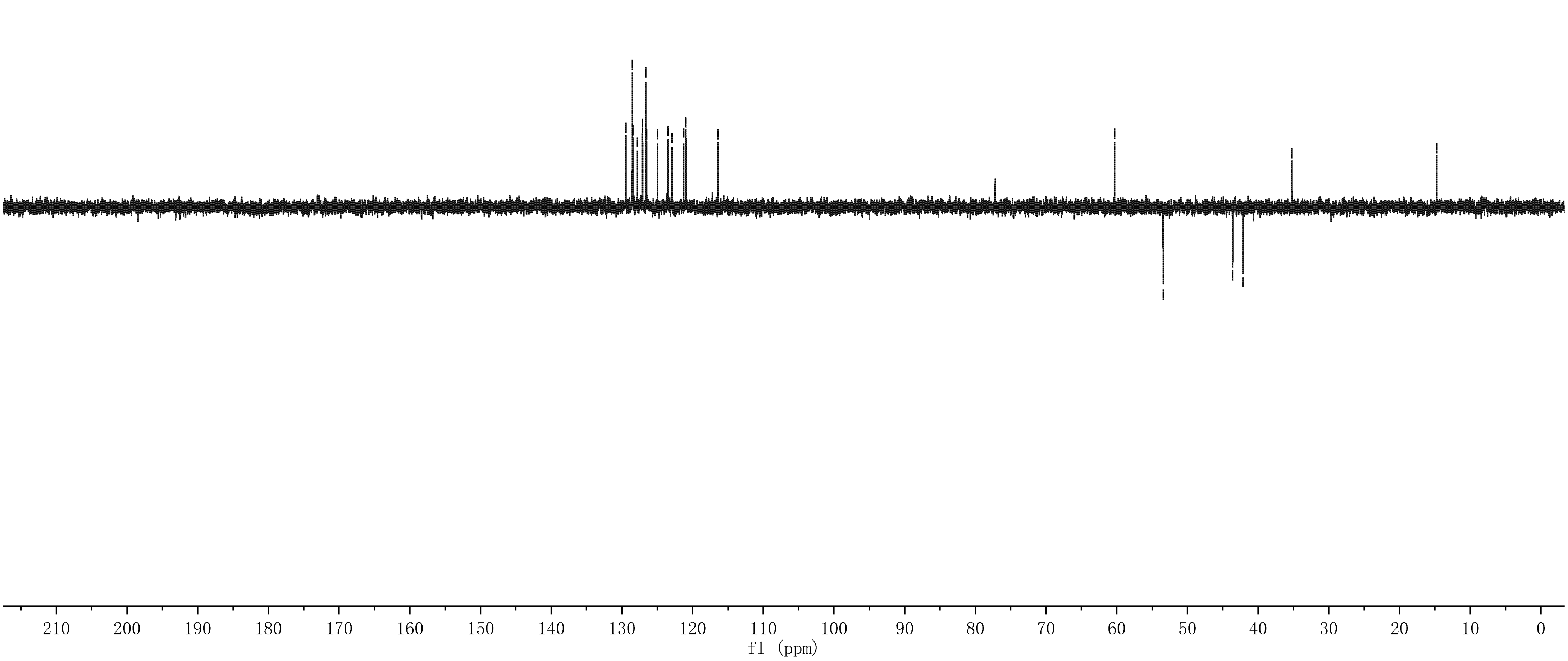

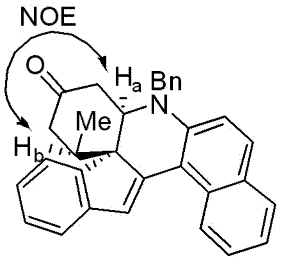

2ac

NOE NMR of compound 2ac

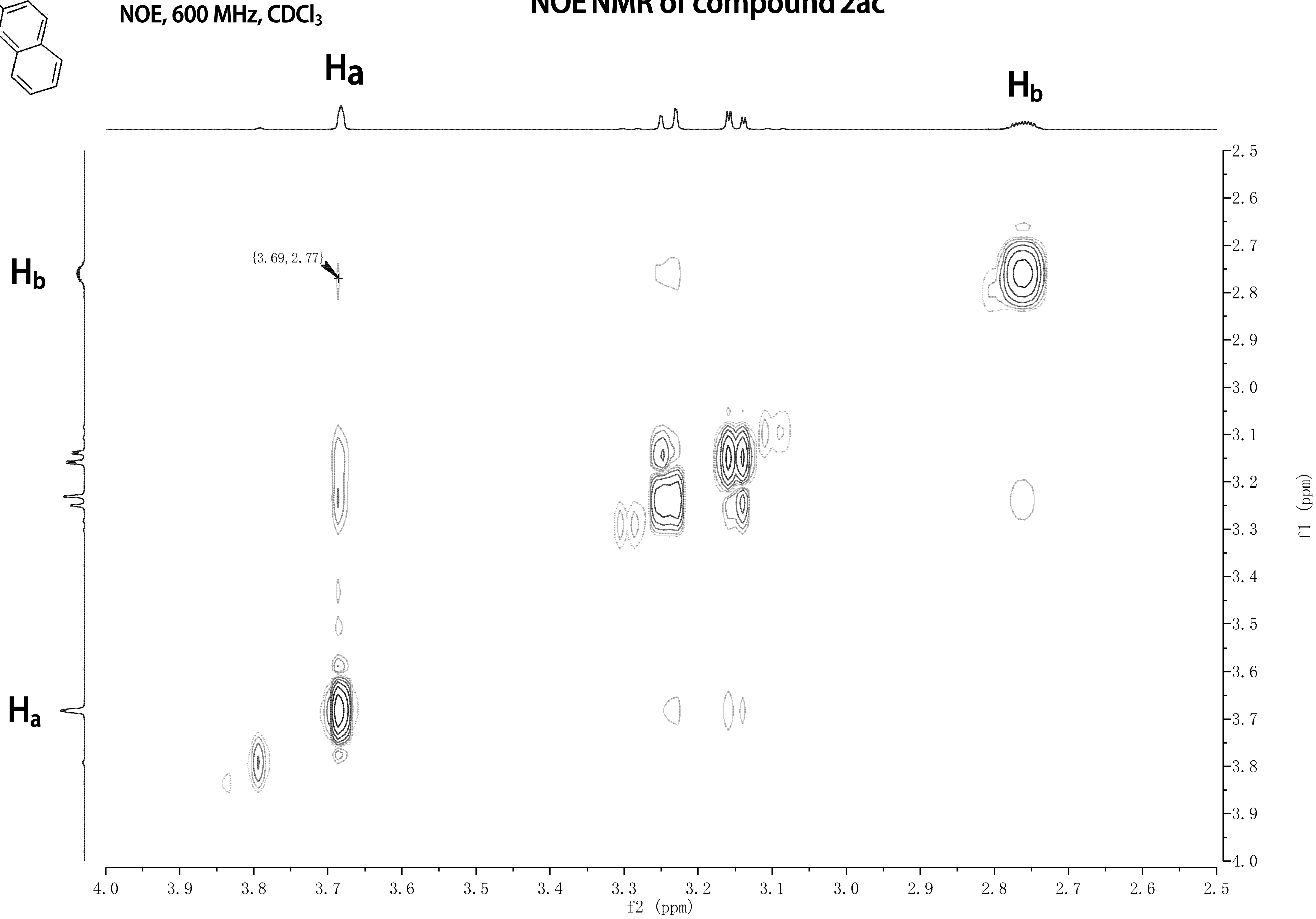

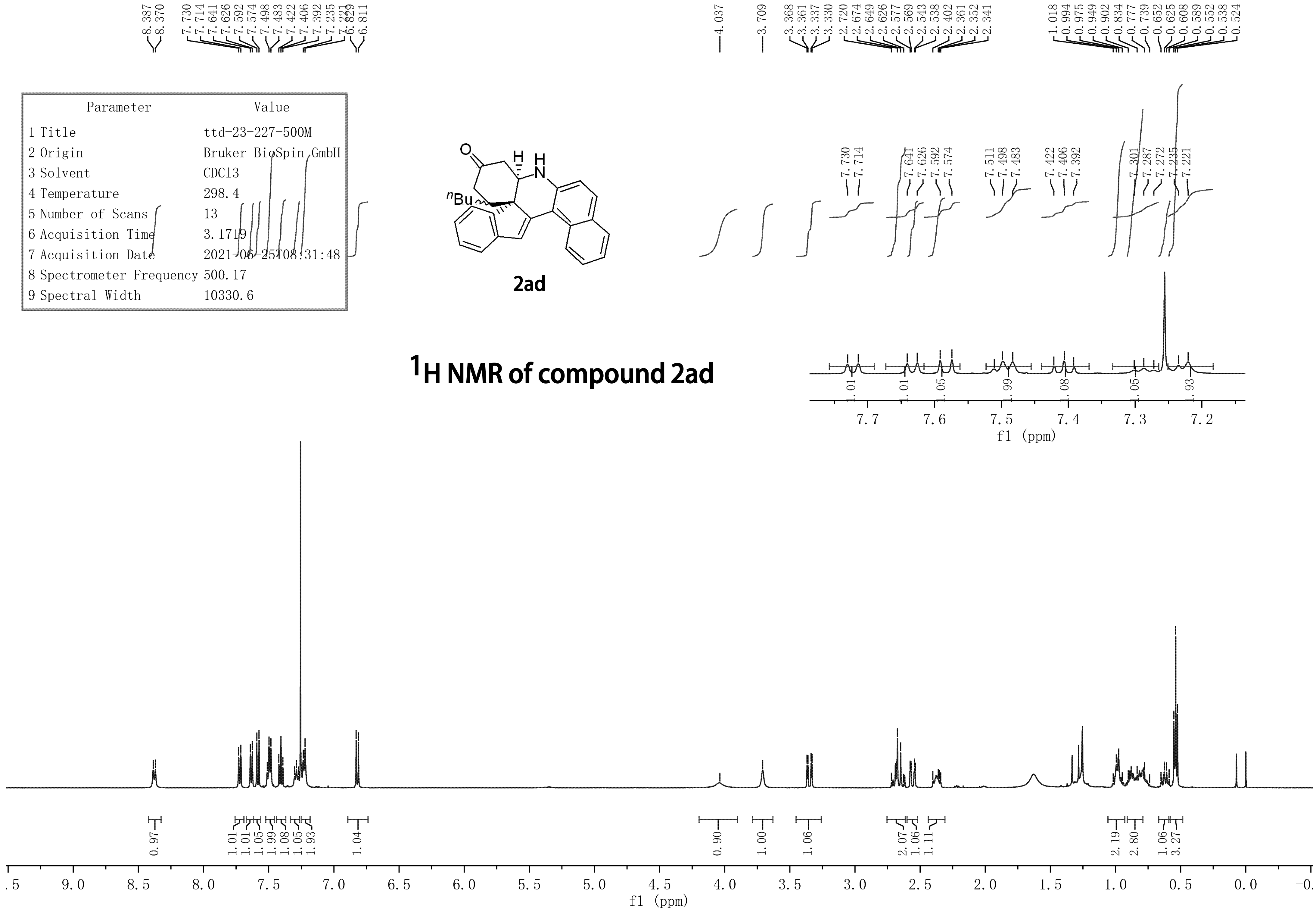

^{1}H NMR of compound 2ad

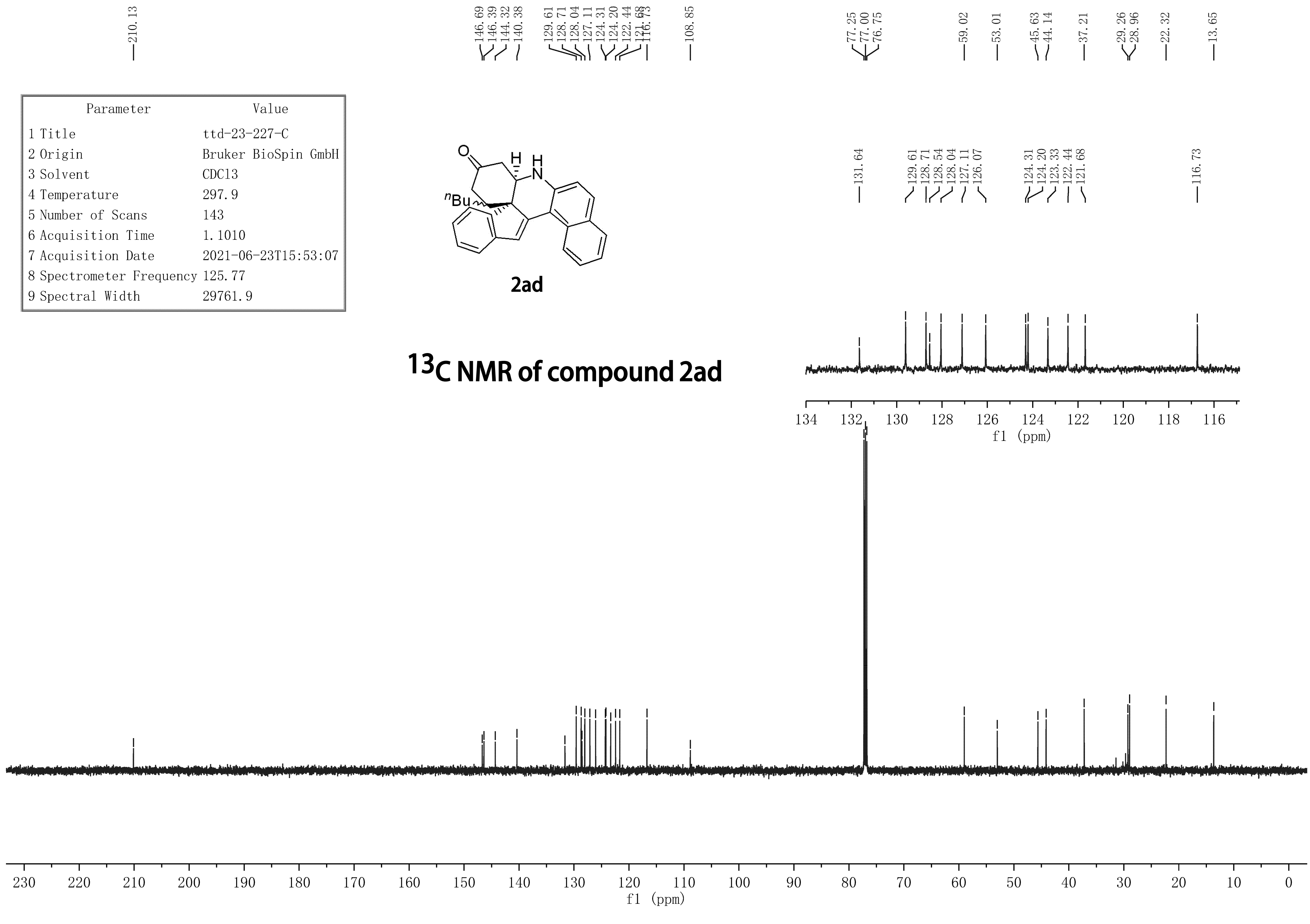

^{13}C NMR of compound 2ad

Parameter	Value
1 Title	ttd-23-227-C135
2 Origin	Bruker BioSpin GmbH
3 Solvent	CDCl3
4 Temperature	297.5
5 Number of Scans	42
6 Acquisition Time	1.1010
7 Acquisition Date	2021-06-23T15:50:12
8 Spectrometer Frequency	125.77
9 Spectral Width	29761.9

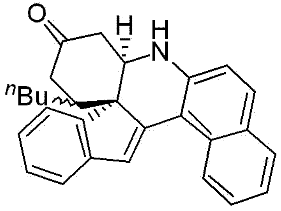

2ad

^{13}C dept135 NMR of compound 2ad

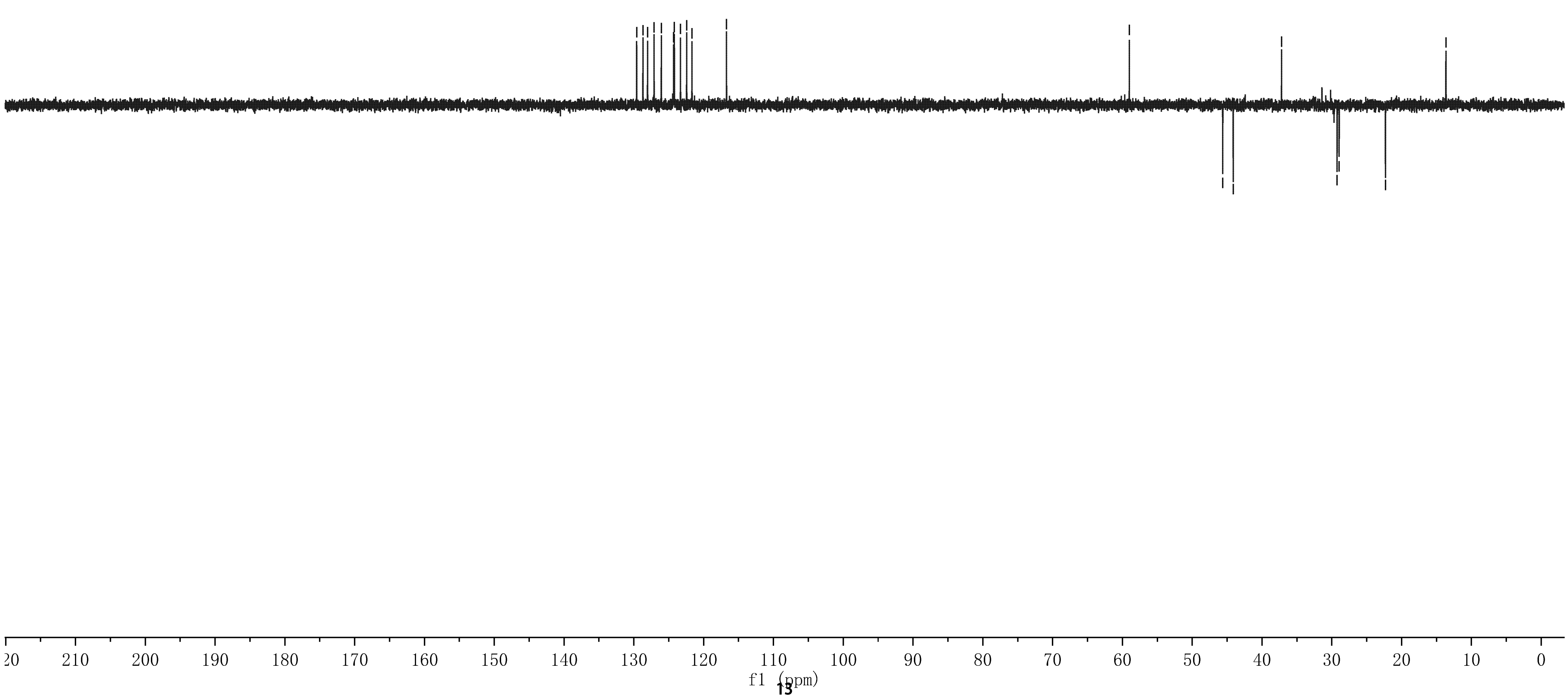

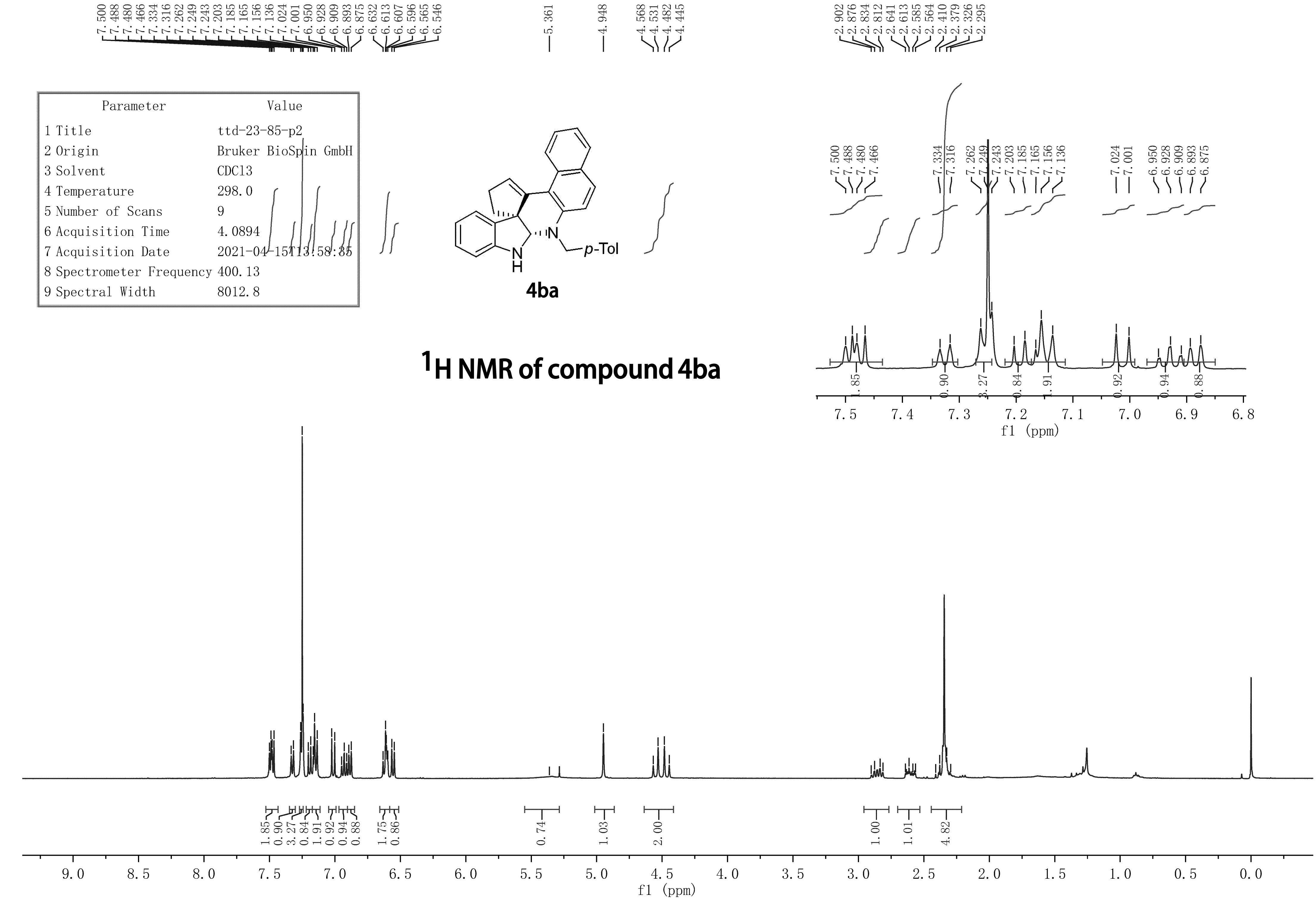

Parameter	Value
1 Title	ttd-23-85-p2
2 Origin	Bruker BioSpin GmbH
3 Solvent	CDCl3
4 Temperature	298.0
5 Number of Scans	9
6 Acquisition Time	4.0894
7 Acquisition Date	2021-04-15T13:58:35
8 Spectrometer Frequency	400.13
9 Spectral Width	8012.8

^{1}H NMR of compound 4ba

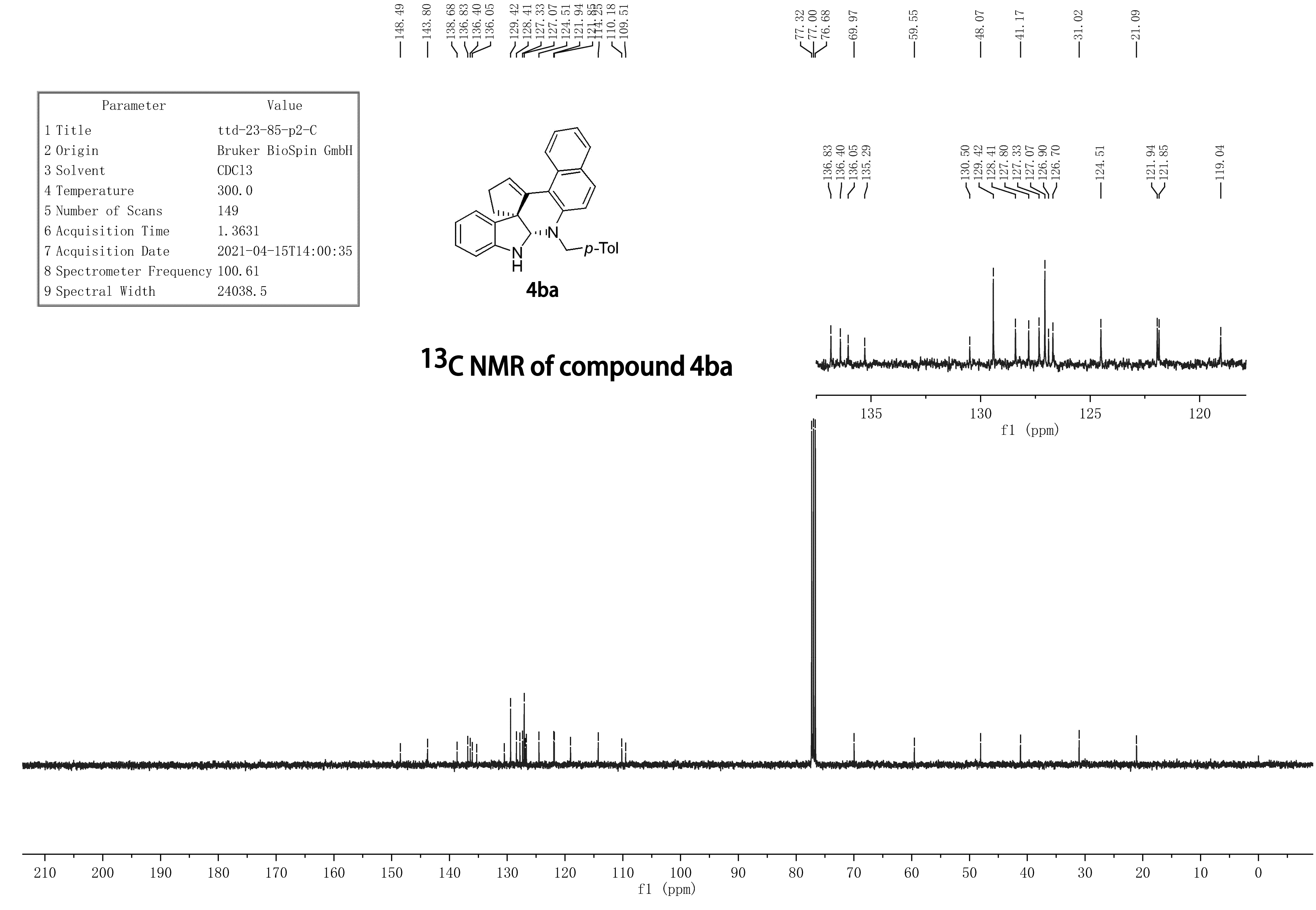

Parameter	Value
1 Title	ttd-23-85-p2-C
2 Origin	Bruker BioSpin GmbH
3 Solvent	CDCl3
4 Temperature	300.0
5 Number of Scans	149
6 Acquisition Time	1.3631
7 Acquisition Date	2021-04-15T14:00:35
8 Spectrometer Frequency	100.61
9 Spectral Width	24038.5

^{13}C NMR of compound 4ba

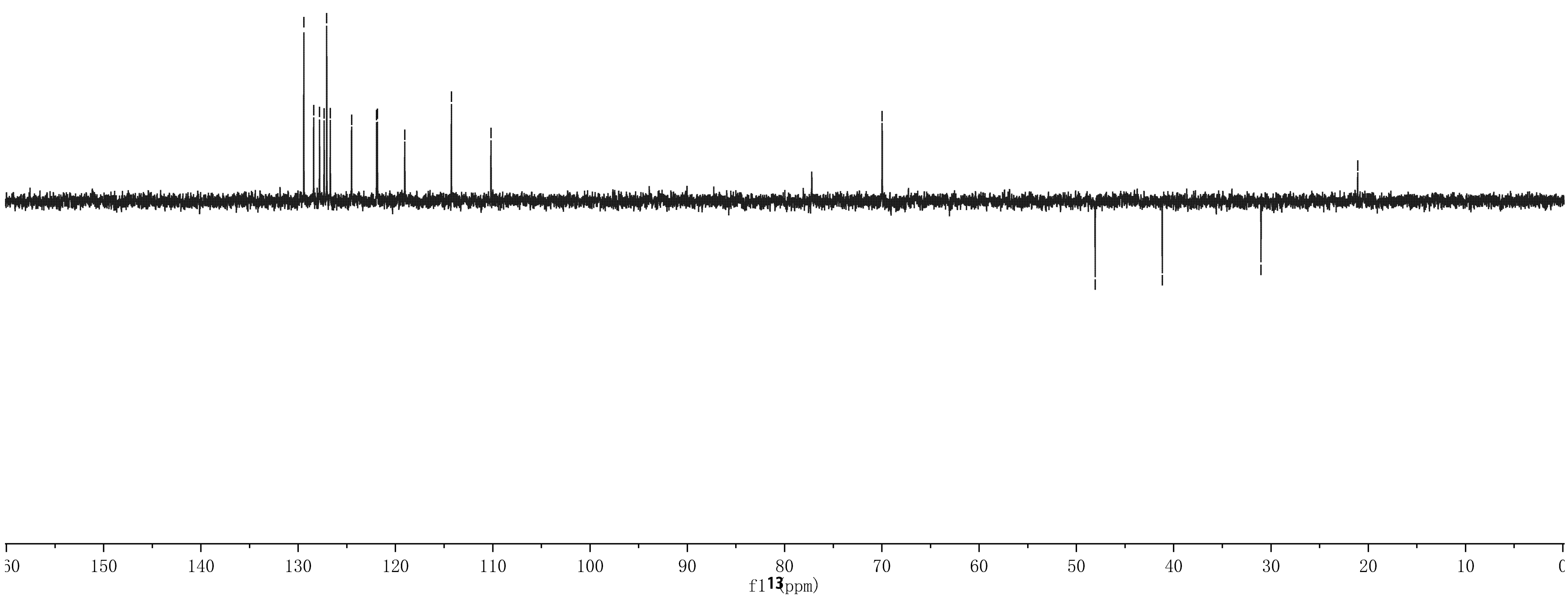

Parameter	Value
1 Title	ttd-23-85-p2-C135
2 Origin	Bruker BioSpin GmbH
3 Solvent	CDCl3
4 Temperature	300.0
5 Number of Scans	66
6 Acquisition Time	2.0316
7 Acquisition Date	2021-04-15T14:09:45
8 Spectrometer Frequency	100.61
9 Spectral Width	16129.0

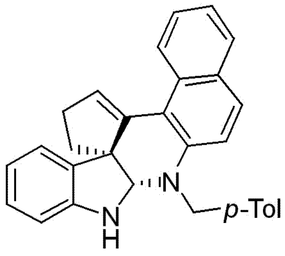

4ba

^{13}C dept135 NMR of compound 4ba

Parameter	Value
1 Title	ttd-23-183
2 Origin	Bruker BioSpin GmbH
3 Solvent	CDCl3
4 Temperature	297.6
5 Number of Scans	13
6 Acquisition Time	3.1719
7 Acquisition Date	2021-06-01T15:48:40
8 Spectrometer Frequency	500.17
9 Spectral Width	10330.6

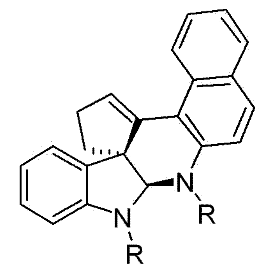

R = $COOCH_2CH_2Cl$

4bb

^{1}H NMR of compound 4bb

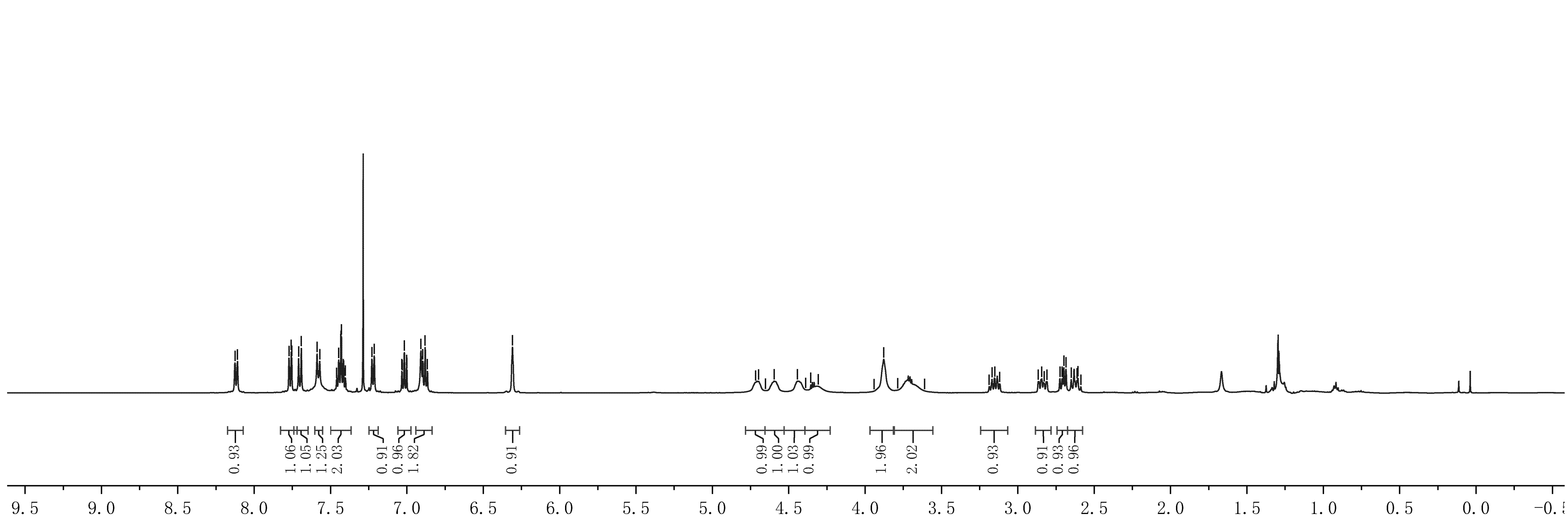

Parameter	Value
1 Title	ttd-23-183-C
2 Origin	Bruker BioSpin GmbH
3 Solvent	CDCl3
4 Temperature	298.0
5 Number of Scans	71
6 Acquisition Time	1.1010
7 Acquisition Date	2021-06-01T15:55:47
8 Spectrometer Frequency	125.77
9 Spectral Width	29761.9

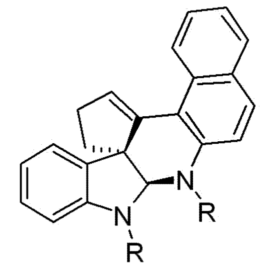

R = $COOCH_2CH_2Cl$

4bb

^{13}C NMR of compound 4bb

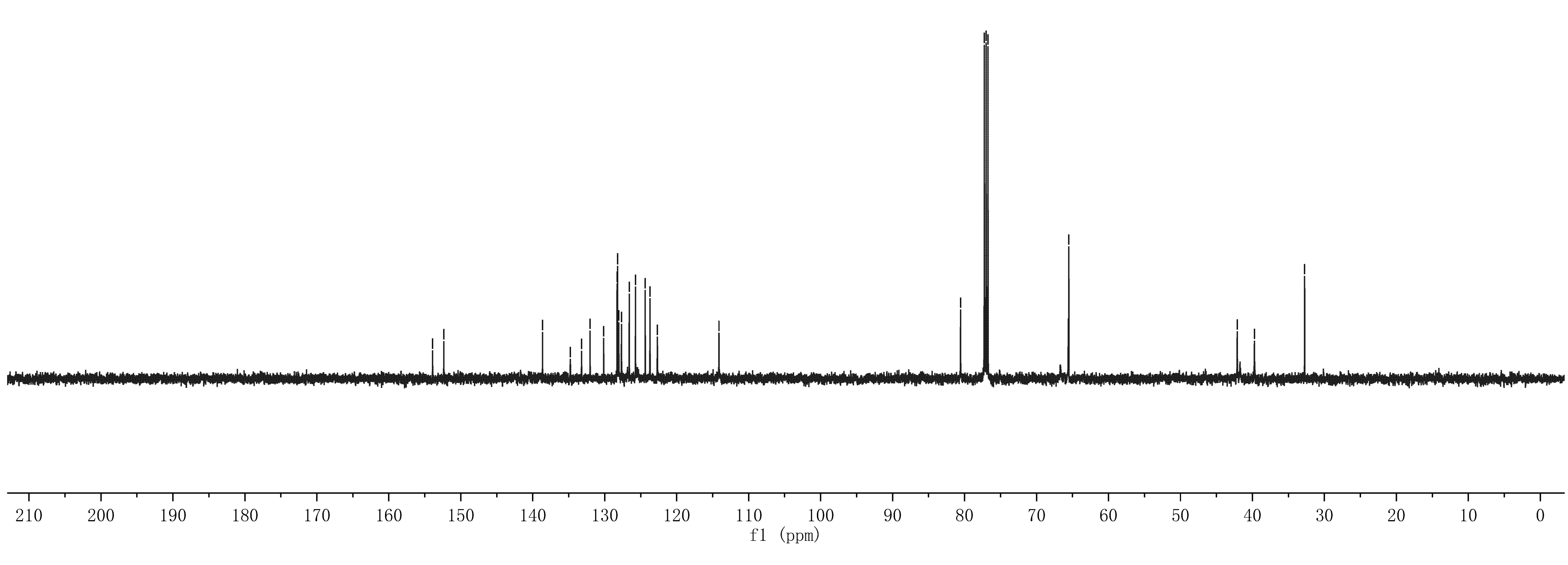

Parameter	Value
1 Title	ttd-23-183-C135
2 Origin	Bruker BioSpin GmbH
3 Solvent	CDCl3
4 Temperature	298.1
5 Number of Scans	21
6 Acquisition Time	1.1010
7 Acquisition Date	2021-06-01T16:00:21
8 Spectrometer Frequency	125.77
9 Spectral Width	29761.9

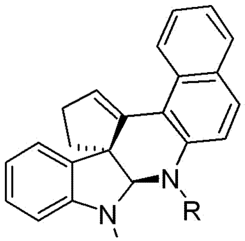

R = $COOCH_2CH_2Cl$

4bb

^{13}Cdept135 NMR of compound 4bb

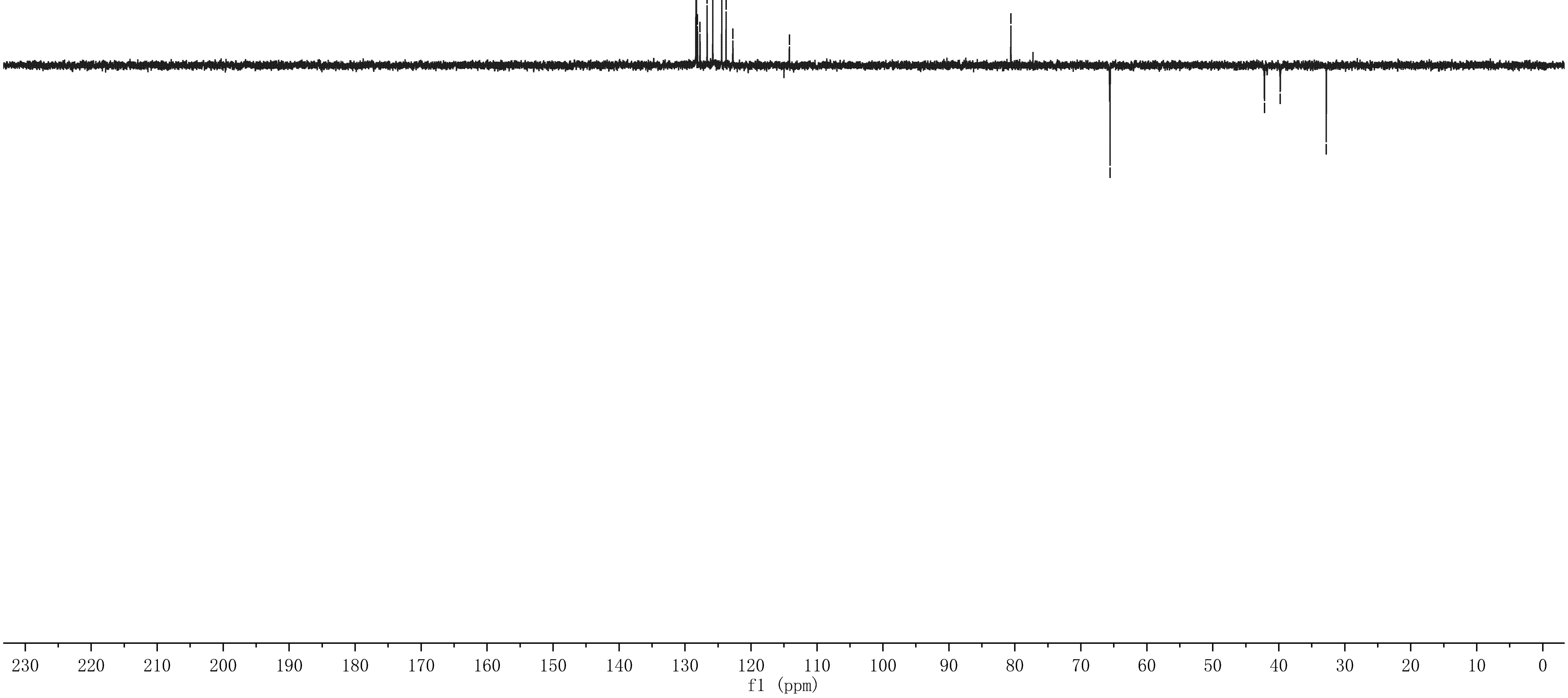

Supplement: Supplementary file 1 — Materials and Methods Supplementary Text Figs. S1 and S2 Tables S1 to S14 [file sciadv.adg4648_sm.pdf]
